# Diagnosis and treatment of Parkinson´s disease (guideline of the German Society for Neurology)

**DOI:** 10.1186/s42466-024-00325-4

**Published:** 2024-06-06

**Authors:** Günter Höglinger, German Parkinson’s Guidelines Committee, Mathias Bähr, Jos Becktepe, Daniela Berg, Kathrin Brockmann, Carsten Buhmann, Andrés Ceballos-Baumann, Joseph Claßen, Cornelius Deuschl, Günther Deuschl, Richard Dodel, Georg Ebersbach, Carsten Eggers, Thilo van Eimeren, Alessandra Fanciulli, Bruno Fimm, Ann-Kristin Folkerts, Madeleine Gausepohl, Alkomiet Hasan, Wiebke Hermann, Rüdiger Hilker-Roggendorf, Günter Höglinger, Matthias Höllerhage, Franziska Hopfner, Wolfgang Jost, Elke Kalbe, Jan Kassubek, Stephan Klebe, Christine Klein, Martin Klietz, Thomas Koeglsperger, Andrea Kühn, Paul Krack, Florian Krismer, Gregor Kuhlenbäumer, Johannes Levin, Inga Liepelt-Scarfone, Paul Lingor, Kai Loewenbrück, Matthias Löhle, Stefan Lorenzl, Sylvia Maaß, Walter Maetzler, Regina Menzel, Philipp T Meyer, Brit Mollenhauer, Manuela Neumann, Per Odin, Tiago Outeiro, Monika Pötter-Nerger, René Reese, Kathrin Reetz, Olaf Rieß, Viktoria Ruf, Anja Schneider, Christoph Schrader, Alfons Schnitzler, Klaus Seppi, Friederike Sixel-Döring, Alexander Storch, Lars Tönges, Claudia Trenkwalder, Uwe Walter, Tobias Wächter, Tobias Warnecke, Florian Wegner, Christian Winkler, Karsten Witt, Dirk Woitalla, Kirsten Zeuner, Claudia Trenkwalder

**Affiliations:** 1 Department of Neurology With Friedrich Baur Institute, LMU University Hospital, Ludwig-Maximilians-Universität (LMU) München, Marchioninistr. 15, Munich, 81377 Germany; 2 German Center for Neurodegenerative Diseases (DZNE), Munich, Germany; 3 Munich Cluster for Systems Neurology (SyNergy), Munich, Germany; 4 Fachklinik Paracelsus-Elena-Klinik, Kassel, Germany

**Keywords:** Parkinson’s disease, Diagnosis, Treatment, Care

## Abstract

**Introduction:**

The aim of this German national guideline is to optimize the clinical care of patients with Parkinson's disease (PD) in terms of diagnostics, drug and surgical treatment and care.

**Summary or definition of the topic:**

This guidance was prepared for the German Society of Neurology (DGN) in collaboration with the Austrian Society of Neurology (ÖGN) and the Swiss Neurological Society (SNG) for German-speaking countries. The guidelines for the diagnosis and treatment of PD have been revised by a national expert group and the guideline commission of the DGN at S2k level. The main objective of these guidelines is to optimize the clinical care of PD patients regarding diagnosis, including early detection, technical diagnostic examinations, and pharmacological as well as invasive treatment options.

**Recommendations:**

The updated PD diagnosis and treatment guidelines are emphasizing optimized clinical care. Key revisions include preferring the name "Parkinson's disease" over previous terms and adopting International Parkinson and Movement Disorder Society (MDS) diagnostic criteria. Recommendations cover genetic and imaging diagnostics, initial pharmacotherapy considering efficacy and patient factors, and tailored pharmacological combinations for complications. Guidelines extend to managing cognitive, affective, psychotic, and autonomic symptoms, along with non-oral therapies like pump therapy and deep brain stimulation. Special situations like akinetic crisis, driving ability, and care concepts are addressed, ensuring comprehensive management for PD patients at various stages and conditions.

**Conclusions:**

This guidance reflects the state of the art at the beginning of 2024.

**Supplementary Information:**

The online version contains supplementary material available at 10.1186/s42466-024-00325-4.

## Introduction

This article is an abbreviated and translated version of the guideline on Parkinson's disease (PD) [1] prepared for and approved by the German Society for Neurology (Deutsche Gesellschaft für Neurologie, DGN), covering both diagnostic and therapeutic options. A complete version of this guideline can be found on the website of the DGN (www.dgn.org) and the Arbeitsgemeinschaft wissenschaftlicher Medizinischer Gesellschaften (AWMF, https://register.awmf.org/de/leitlinien/detail/030-010, registry No. 030/010, last update: November 30th, 2023; valid until: October 24th, 2028; last access: March 30th, 2023). The level of the guideline is S2k. The guidelines target various professional groups including general practitioners, neurologists, neuroradiologists, neurosurgeons, neuropsychologists, psychologists, psychiatrists, human geneticists, nurses, physiotherapists, occupational therapists, speech therapists, social workers, service providers including health insurance companies and pension insurance providers, as well as patients and caregivers.

## Methodological approach

This project was managed by the coordinators Günter Höglinger and initially Alexander Storch, later Claudia Trenkwalder. Methodological expertise was provided by Richard Dodel, Ina Kopp (AWMF) and Monika Nothacker (AWMF). Katja Ziegler and Sonja van Eys supported the development as members of the DGN Guideline Committee. An open call by the DGN invited all members to volunteer as member of the German Parkinson’s Guidelines Committee. Experts were formally accepted following an independent assessment of their conflict of interest statements. Interdisciplinarity was established by including several medical societies and associations and a patient representative (see listing in the appendix). In order to translate the clinical problems into specific, searchable questions to be answered by scientific investigations, we used the PICO (*P*atient, *I*ntervention, *C*omparison, and *O*utcome) framework [2]. The questions were based on the identified key clinical areas covering all relevant topics on PD diagnosis, diagnostic tools, pharmacological and non-pharmacological treatments in different disease stages and conditions, and health care issues; in addition, PICO questions from published guidelines were included resulting in a total of 204 key questions. We conducted systematic reviews and review updates using standard Cochrane methodology. For searching, screening and selection of studies, data extraction and management, and data analysis we followed the guidance in the Cochrane Handbook [3]; the systematic literature search was carried out in the PubMed databases (https://pubmed.ncbi.nlm.nih.gov/; search period: from 2016 (last PD German Guideline), to 11/ 2021; language: German and English). The search terms required were structured as described in detail in the Guideline Report available online (https://register.awmf.org/de/leitlinien/detail/030-010). Each expert group developed standardized recommendations based upon a pre-defined template for all PICOs and a corresponding scientific background texts based on the available literature. The strength of recommendation followed the guidelines of the Oxford Centre for Evidence-based Medicine. *A strong recommendation was expressed as „should be recommended”, a less strong recommendation as “can be recommended”, and a less well established recommendation as “may be considered”*. All recommendations were initially voted on in a Delphi process; comments and proposed changes were reviewed and revised again. In a total of five structured and moderated consensus conferences (online) including the experts and the according to the principles of the NIH (National Institutes of Health), all recommendations were finally discussed by the consensus group and voted on in an AWMF-moderated process. The degree of consensus was classified as “strong consensus” in the case of > 95% consensus among the voting experts, as “consensus” in the case of > 75—95%, as “majority agreement” in the case of > 50 -75%, and as “no majority agreement” in the case of ≤ 50%. In this abbreviated guideline we prioritized recommendations with agreements of 90%—100%; *all recommendations which do not explicitly mention the consensus strength in this article, had reached a consensus of > 90%*. The final guideline was reviewed and approved by the DGN Guideline Committee. The quality of the guideline was assessed with the AGREE-II Instrument [4]. For further details regarding search strategy, study selection, data extraction, data synthesis, summarizing and interpreting results, see the methodological Appendix section of the German guideline (https://register.awmf.org/de/leitlinien/detail/030-010).

## What´s new?

The national guidelines for the diagnosis and treatment of PD, updated lastly in 2014, have now been revised during 2022–23 by a national expert Editorial Committee and the DGN Guideline Committee at S2k level. The main objective of these guidelines is to optimize the clinical care of PD patients covering all relevant topics on PD diagnosis, diagnostic tools, pharmacological and non-pharmacological treatments in different disease stages and conditions, and health care issues. Key updates in these guidelines include recommending the use of the term "Parkinson disease" instead of "idiopathic Parkinson syndrome" or "primary Parkinson syndrome" due to the identification of genetic variants and environmental factors often playing a relevant role in the etiology of the disease. It is also recommended to use the International Parkinson and Movement Disorder Society (MDS) diagnostic criteria for diagnosing PD. Further recommendations involve diagnostic tools and genetic investigations as well as cranial magnetic resonance and nuclear medicine imaging options in early diagnosis. Regarding pharmacological treatment, several medications are recommended for initial monotherapy. The selection for individual patients should consider efficacy, side effects, the patient’s age, comorbidities, and psychosocial profile. Pharmacological combination therapy should be considered if monotherapy is either not sufficient or cannot be increased to sufficient efficacy due to limiting side effects. Specific activating procedures for physical therapy are recommended. For patients with fluctuations and dyskinesias, various pharmacological options are available, with individual treatment tailored considering efficacy, side effects, and the patient’s preferences. Updated recommendations are given for the selection of non-oral therapies such as medication administration through pump therapy and deep brain stimulation (DBS). The guidelines also provide recommendations for the diagnosis and treatment of cognitive, affective, psychotic, and autonomic symptoms as well as sleep disorders, pain, and speech and swallowing disorders in PD patients. Additionally, recommendations are provided for management in special situations such as pregnancy and breastfeeding, akinetic crisis, medication or DBS withdrawal syndromes, and perioperative procedures. Driving ability and various care concepts such as daytime clinics, multidisciplinary in-patient therapies, and palliative care are also evaluated.

## Guidelines in detail

### Definition

The terms PD and "idiopathic Parkinson's syndrome" have historically been used interchangeably. However, in recent years, it has become clear that a significant number of cases of Parkinson's disease are caused by genetic variants or environmental factors and are therefore not "idiopathic" in nature. Hence, within the context of these guidelines, the broader term PD is recommended.

## Diagnosis

### Clinical diagnostic criteria

The MDS criteria from 2015 should be applied for diagnosing PD. Therapeutic response to levodopa therapy can improve diagnostic accuracy. However, diagnostic accuracy is significantly better with a longer course of the disease ≥ 5 years, making long-term clinical follow-up superior to diagnosis based on responsiveness to levodopa therapy at baseline. The severity of non-motor symptoms may be considered at the time of diagnosing PD for prognosis assessment.

### Imaging diagnostics

Cranial MRI (cMRI) should be performed early in the disease course for differential diagnosis in Parkinson syndromes. When assessing exclusion criteria for PD, cMRI scans with standardized sequences should be performed (T1- and T2-weighted (ideally high-resolution 3D) and may include iron-sensitive/susceptibility-weighted and diffusion-weighted sequences.

Transcranial brain parenchymal sonography (TCS) by a qualified examiner may be considered to support the differential diagnosis of PD versus atypical and secondary Parkinson syndromes. TCS should include assessment of substantia nigra, nucleus lentiformis, and 3rd ventricle. FDG-PET may be considered if there are sufficient clinical signs suggesting an atypical Parkinson syndrome and the findings have clinical implications (e.g., regarding diagnosis, prognosis, or therapy) (Consensus strength: 84%, consensus). FDG-PET may also be considered for the assessment of the risk of dementia in PD, again, only if the findings have clinical implications. Dopamine transporter SPECT (DAT-SPECT) can be considered performed early in the disease course to detect nigrostriatal deficit in clinically unclear Parkinson or tremor syndromes if the findings have clinical implications (Consensus strength: 82.8%, consensus). Cardiac MIBG scintigraphy or SPECT may be considered to differentiate PD from MSA if FDG-PET is not available.

### Genetic diagnostics

Diagnostic genetic testing should be offered upon patient request if either two first-degree relatives or one first-degree and one second-degree relative were diagnosed with PD or if disease manifestation occurs before age 50.

In PD patients with an onset age over 50, at least *LRRK2*, *SNCA*, and *VPS35* should be examined. Besides sequencing, the techniques used should also be able to detect deletions/duplications.

In PD patients with disease manifestation before age 50 who request genetic testing, *PRKN*, *PINK1*, *DJ1*, *LRRK2*, *SNCA*, and *VPS35* genes should be examined. If multiple family members are affected, testing should preferably start with the patient with the youngest age of onset and apply appropriate techniques (sequencing, deletions, duplications).

If suspicion of a genetic cause of PD persists despite a negative finding in the aforementioned tests, a neurologist specialized in neurogenetics or a geneticist should be consulted if the patient wishes to plan further clinical diagnostic procedures.

Investigations to assess polygenic risk should not be routinely performed in the context of clinical care. Investigations to detect genetic variants in the *GBA1* gene should also not be routinely performed. However, in cases of isolated PD or disease manifestation before age 50, or in Parkinson patients with rapid motor progression or fast deterioration of cognition, examination of the *GBA1* gene may be considered if the patient requests a genetic investigation after informed consent.

Diagnosis of hereditary PD does not allow for a reliable prediction of survival, quality of life, and cognitive impairment for individual patients and there is no specific approved pharmacological therapy for either monogenic PD or genetically complex PD to date. Deep brain stimulation is possible in monogenic PD, with the same inclusion and exclusion criteria as in genetically complex or sporadic PD.

## Pharmacotherapeutic options

The following paragraphs summarize essential information on the available classes of medications to treat PD:

- *Levodopa preparations:* Levodopa preparations can be used for PD therapy without prioritization among preparations based on the comprised decarboxylase inhibitor (Carbidopa or Benserazide). Retarded formulations with a decarboxylase inhibitor are not recommended for daytime therapy, but for night-time symptom management only. Rapidly soluble oral and inhalable Levodopa formulations can be used for acute symptom control, but inhalable Levodopa requires concurrent oral administration due to lacking decarboxylase inhibition in the former.
- *Dopamine agonists:* Ergoline dopamine agonists (Bromocriptine, Cabergoline, Pergolide) should not be used for PD therapy. Non-ergoline dopamine agonists (Pramipexole, Ropinirole, Piribedil, Rotigotine, with limitations Apomorphine) can be used for PD therapy, considering specific indications outlined below. Apomorphine is available for subcutaneous injection or infusion and sublingual use only, thus being restricted to specific indications. Pramipexole and Ropinirole in retarded formulations and Rotigotine as a transdermal patch allow once-daily dosing. Prioritization among dopamine agonists based on their specific efficacy profiles cannot be decisively derived from literature. Adjustment of Ropinirole dosage or switching to another dopamine agonist should be considered when co-administered with CYP1A2 inducers or inhibitors. In case of impaired liver function, Pramipexole should be prioritized as it is mainly metabolized by the kidneys. In case of impaired kidney function, prioritizing Ropinirole, Rotigotine, or Piribedil over Pramipexole is advisable.
- *COMT inhibitors: *Opicapone and Entacapone are largely equivalent in effect as COMT inhibitors and can be used for treating Levodopa-response fluctuations in PD, considering specific indications outlined below. Tolcapone should be used cautiously due to potential hepatotoxicity, primarily as a second-line treatment with rigorous clinical and laboratory safety monitoring.
- *MAO-B inhibitors:* No prioritization among various MAO-B inhibitors based on efficacy can be decisively derived from literature. MAO-B inhibitors Selegiline or Rasagiline can be used as monotherapy for early PD or in combination with Levodopa for treatment of PD with fluctuations. Safinamide, a dual-action MAO-B inhibitor, is not approved as monotherapy but can be used in combination with Levodopa for treating PD with fluctuations.
- *NMDA receptor antagonists:* Amantadine can be used for PD therapy, considering specific indications outlined below. Budipine is no longer recommended due to its side-effect profile.
- *Anticholinergics: *Anticholinergics should no longer be used as antiparkinsonian agents due to an unfavorable risk–benefit profile. Their use should be restricted to rare cases for tremor control.

The following paragraphs summarize pharmacological treatment recommendations for stages and conditions of PD.

### Initial monotherapy

The selection of various drug classes for initial monotherapy should consider their differing efficacy, side effects, patient age, comorbidities, and psychosocial profile. Motor fluctuations and dyskinesias are observed earlier in the disease course with initial monotherapy of Levodopa, particularly at high doses and pulsatile administration, compared to initial monotherapy with MAO-B inhibitors or dopamine agonists. Dopamine agonists or MAO-B inhibitors should therefore be considered preferentially over Levodopa for biologically younger patients. However, patients requiring Levodopa in initial monotherapy should of course receive it. Reasons for its initial use may include symptom severity, need for rapid therapeutic effects, multimorbidity, observed or expected side effects of other medication groups (e.g., impulse control disorders with agonists), and potentially better individual tolerability.

### Combination therapy

Pharmacological combination therapy should be considered following initial monotherapy when the efficacy of the initial monotherapy at an intermediate maintenance dose for dopa-sensitive target symptoms is inadequate or when the monotherapy's required dosage for symptom control cannot be achieved due to therapy-limiting side effects. Various combinations of Levodopa-preparations with dopamine-agonists and/or MAO-B inhibitors may be considered for combination therapy of PD without fluctuations.

### Therapy of fluctuations

Various medication options are available for treating fluctuations, including adjusting Levodopa intervals and dosages, additional administration of Levodopa preparations with modified galenic forms (soluble, inhaled, retarded), or adding dopamine agonists, MAO-B inhibitors, or COMT inhibitors. The prioritization of these options for PD patients with fluctuations should consider individual factors such as efficacy, side effect profile, and patient preference.

### Therapy of dyskinesias

Amantadine should be used to reduce dyskinesias in PD patients with Levodopa-induced motor complications, considering anticholinergic and hallucinogenic side effects. Safinamide may be considered for the treatment of moderate to severe dyskinesias, although the data on its effectiveness and dosage are not conclusive (Consensus strength: 85.7%, consensus).

### Therapy of tremor

For PD tremor refractory to standard Levodopa doses, increasing Levodopa daily dosage or high Levodopa single doses can be helpful in individual cases. However, the long-term increase in Levodopa dosage for severe tremor should be balanced against the increased risk of motor complications. Dopamine agonists should be used for PD disease symptoms in monotherapy and combination therapy, typically addressing symptoms of akinesia and rigidity, which often also improves Parkinson's tremor equivalently. Anticholinergics may only be considered in exceptional cases for PD patients with otherwise untreatable tremor due to their anticholinergic side effects. Betablockers may be considered for treating postural PD tremor (off-label). Initial treatment of PD tremor should follow the general therapeutic principles for PD as outlined above. The initial medication choice depends on clinical factors such as age, comorbidities, and severity of motor symptoms. When optimizing oral medication based on target symptoms of akinesia and rigidity failed to control tremor, special interventions such as deep brain stimulation (DBS) or pump therapies can be considered.

## Treatment of non-motor symptoms

### Pain

**Fig. 1:**
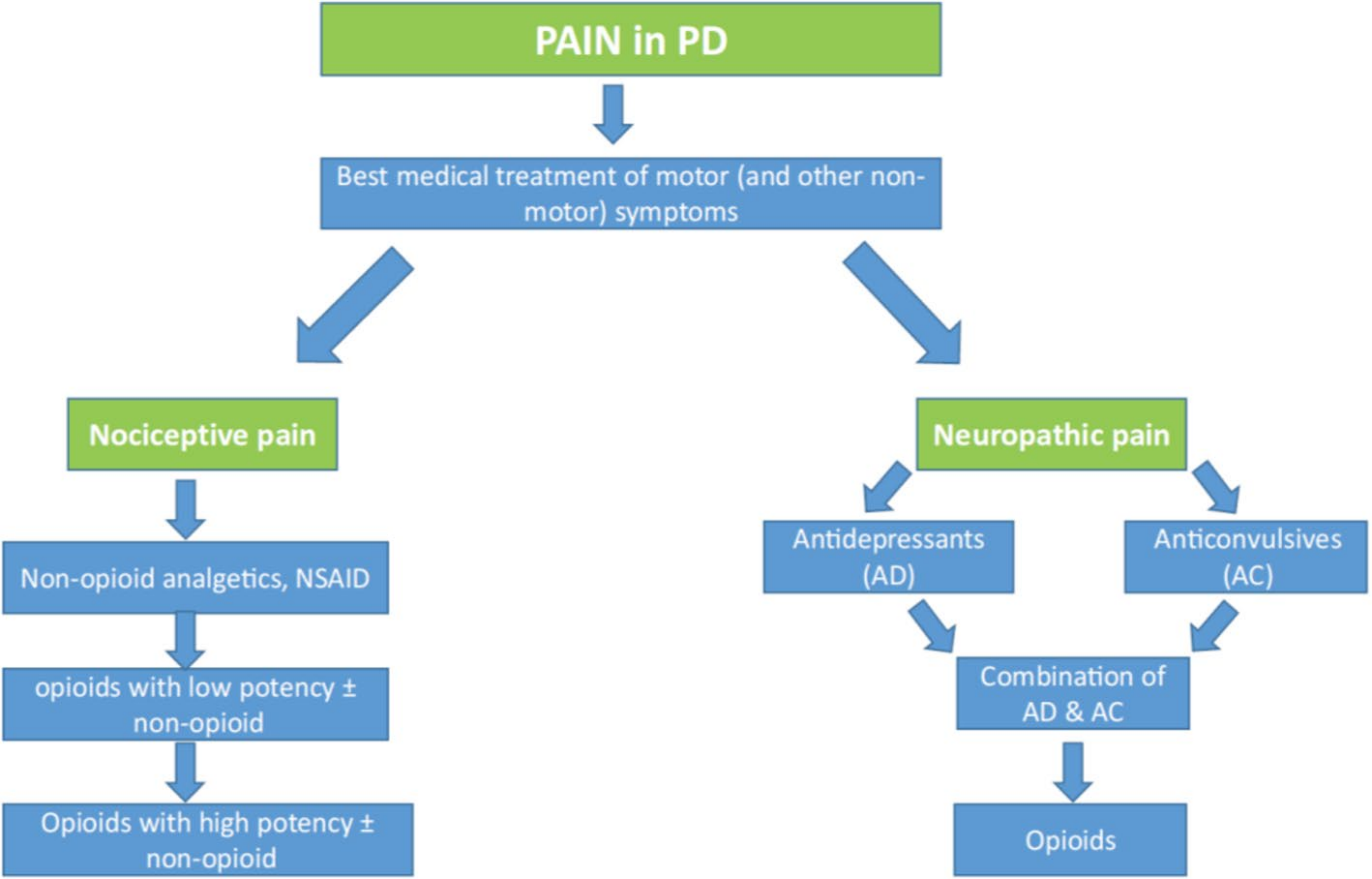
Treatment algorithm for pain in PD patients; nociceptive pain according to WHO three-step-pain treatment scheme (adapted with permission from [5])

The basis of pain therapy in PD is optimizing the PD medication. Nociceptive pain should be treated according to the 3-step WHO scheme. Neuropathic pain should be treated with anticonvulsants and/or antidepressants following the guideline for neuropathic pain treatment, with a preference for Gabapentin and/or Duloxetine (especially in comorbidity with depression). In cases of severe pain, treatment with extended-release Oxycodone/Naloxone may be considered. Figure 1 shows a proposed therapy algorithm for the treatment of pain in Parkinson's patients.

### Bladder dysfunction

Non-pharmacological treatment options for neurogenic bladder dysfunction in PD include bladder training, adjusting fluid intake throughout the day, and avoiding caffeine, alcohol, and carbonated drinks. Antimuscarinics such as Solifenacin, Trospium or Darifenacin can be considered for treatment of increased urinary frequency and urgency due to overactivity of the bladder detrusor in PD patients. A treatment with β3-receptor agonists (e.g., Mirabegron) for both urinary urgency and incontinence due to bladder detrusor overactivity may be considered for patients who have not adequately responded to antimuscarinics, cannot tolerate them, or have contraindications to them. Intravesical injection of Botulinum toxin A may be considered for treatment of detrusor overactivity in patients who have inadequately responded to oral therapy attempts provided that the patient's motor and cognitive performance level allows for subsequent intermittent self-catheterization, if needed. For non-pharmacological treatment of nocturia, patients should be advised to restrict fluid intake in the late afternoon and evening, avoid evening alcohol consumption, and aim for an upper body elevation of 10–20° while sleeping. Desmopressin therapy may be considered for treating nocturnal polyuria under close monitoring of blood pressure, serum electrolytes, and body weight. For treatment of nocturia due to reduced bladder capacity in Parkinson's patients, therapy with antimuscarinics such as Solifenacin, Trospium, or Darifenacin may be considered.

### Orthostatic hypotension

A Schellong test should be performed to diagnose *orthostatic hypotension* in PD patients. A tilt table examination can be considered if available, as this provides more comprehensive information. The following stepwise approach should be used for treating orthostatic hypotension in Parkinson's patients:

Eliminate or treat exacerbating and triggering factors such as infectious diseases, dehydration, and others, followed by reviewing concurrent medication (if antihypertensives are used, dose reduction or discontinuation should be aimed for) and implement non-pharmacological treatment measures. These are adequate fluid and salt intake, provided there are no internal contraindications to this (e.g. cardiac, hepatic, renal insufficiency), the avoidance of sumptuous meals or excessive alcohol consumption or the exposure to heat, elevating the upper body by 10–20° during sleep. Wearing an abdominal compression bandage during the day is more effective than compression stockings. In the prodromal phase of syncope isometric maneuvers to increase blood pressure (e.g. tensing the leg, buttock, abdominal and arm muscles) can be performed.

As pharmacological measures for the treatment of orthostatic hypotension in PD patients, a therapy with midodrine should be recommended, treatment with fludrocortisone can be recommended, treatment with droxidopa (L-Threo-DOPS) may be considered (off-label use, only available from international pharmacies).

For drug therapy of *nocturnal recumbent hypertension*, evening administration of clonidine, eplerenone, losartan, nebivolol, nitroglycerin (transdermal application) or sildenafil (in alphabetical order) may be considered.

### Constipation

General treatment recommendations from the AWMF guideline "Chronic Constipation" should be used for treating constipation in PD patients.

### Sleep disorders

Comorbid primary sleep disorders such as Restless Legs Syndrome (RLS) and sleep-related breathing disorders (SBAS) should be treated according to the guidelines applicable to these entities. If there are indications that motor or non-motor dopaminergic fluctuations are responsible for the sleep disorder, appropriate adjustments to dopaminergic pharmacotherapy should be made. Rapid Eye Movement Sleep Behavior Disorder (RBD) should be treated by creating a safe sleeping environment. Additionally, Clonazepam and/or Melatonin may be considered, considering possible side effects. In cases of insomnia or circadian rhythm disorders, underlying causes such as medication side effects and/or primary sleep disorders like SBAS should be ruled out. After excluding specifically treatable causes of sleep disorders, these should be treated with sleep hygiene measures, intensive physical training, and light therapy. Eszopiclone, Doxepin, Zolpidem, Trazodone, Melatonin, Venlafaxine (in comorbid depression), Nortriptyline, or Mirtazapine may be considered for insomnia despite weak evidence.

### Cognitive disorders

Cognitive disorders in PD patients were divided into PD with mild cognitive impairment (PD-MCI) and PD with dementia (PDD) according to international definition criteria.

- *PD-MCI*: Cognitive training should be offered to individuals with PD-MCI. Physical endurance training should be conducted in the aerobic range for 2–3 times a week, lasting 45 to 60 min each session. Pharmacological treatment with Rivastigmine, Donepezil, and Galantamine should not be used for PD-MCI (Consensus strength: 89.7%, consensus).
- *PDD*: Cognitive stimulation treatments should be offered to individuals with PDD. Rivastigmine should be used to treat PDD. Donepezil can be used for treating PDD (Off-Label Use). Galantamine should not be used for treating PDD.

### Affective disorders

Non-pharmacological interventions are recommended to treat affective disorders in PD patients: Cognitive behavioral therapy is recommended to treat depression, anxiety, and fear of disease progression. Physical interventions are recommended to treat depression, fatigue, and apathy.

Regarding pharmacotherapy, to treat *depressive disorders* in PD patients, optimal dopaminergic medication should be used and a therapy with Pramipexole should be conducted if therapy with a Dopamine agonist is possible. Rotigotine can be used as a second-line option. Severe depression in PD patients can be treated with Venlafaxine or Desipramine. Moderate depression in PD patients can be treated as follows:

- depression with lethargy: Venlafaxine, Citalopram, or Sertraline
- depression with agitation, anxiety, restlessness, or sleep disturbance: Mirtazapine (not if RBD is present) or Trazodone.
- depression with comorbid sleep disturbance, pain or drooling, in cognitively unimpaired patients: Amitriptyline.

*Anhedonia* in Parkinson's patients may be treated with optimal dopaminergic medication with Levodopa, and / or Rotigotine, Pramipexole or Piribedil.

*Apathy* in PD patients should be treated with optimal dopaminergic medication, and therapy with Pramipexole, Rotigotine, or Piribedil may be conducted if individualized dopamine agonist therapy is possible. Apathy in PD can be treated with Venlafaxine or Nortriptyline.

For treating *anxiety* disorders with affective fluctuations in PD patients, optimal dopaminergic medication should be used, and therapy with a non-ergot dopamine agonist may be conducted if individualized dopamine agonist therapy is possible. Constant anxiety disorders without affective fluctuations in PD patients should not be treated with adjusted dopaminergic therapy. Citalopram may be attempted for treating anxiety disorders in Parkinson's disease.

For treating *fatigue* in PD patients, optimal dopaminergic medication should be used, and therapy with Rotigotine may be conducted if individualized Dopamine agonist therapy is possible. Modafinil or Safinamide may be considered for treating fatigue in PD patients.

### Impulse control disorders

Patients should be made aware of the possibility of developing impulse control disorders (ICDs) before starting dopamine agonists. Gradual reduction of dopamine agonists is an effective treatment for ICDs. If adverse effects of dopamine agonists occur (e.g., dopamine agonist withdrawal syndrome), dopamine agonists should be reduced to the lowest tolerated dose. For patients with an indication for non-oral follow-up therapy, Levodopa-Carbidopa Intestinal Gel (LCIG) therapy may be considered for treating ICDs. Cognitive behavioral therapy may be used to treat ICDs. Bilateral STN-DBS is an effective therapy for treating ICDs in patients with an indication for non-oral follow-up therapy.

### Psychosis

PD patients with psychotic symptoms should be treated in the following steps:

If the symptomatology allows, the treatment should start with implementation of general non-pharmacological measures (e.g. stimulus shielding, reorienting measures, re-establishment of a circadian rhythm). Implementation of general therapeutic measures such as fluid supplementation for exsiccosis and treatment of an infection should be started, followed by reduction/adjustment of triggering medication in general (anticholinergic, antiglutamatergic, or sedative drugs) followed by PD medication, especially amantadine, MAO-B inhibitors, dopamine agonists and COMT inhibitors, or combination treatments. If these measures fail, specific anti-psychotic medication such as clozapine should be offered after appropriate risk–benefit assessment (risk of agranulocytosis, risk of myocarditis, risk of falls, anticholinergic side effects). Alternatively, quetiapine can be offered off-label in PD patients without cognitive impairment.

In case of cognitive impairment and failure of the general measures, a switch to an acetylcholinesterase inhibitor can be offered.

## Additional non-pharmacological treatments

### Dysarthria/Dysphagia

Patients with PD-related speech disorders should receive speech therapy. Patients with PD-related swallowing disorders should receive swallowing therapy.

### Activating procedures

PD patients with motor symptoms affecting daily life should have access to physiotherapy. The following recommendations can be made regarding the effectiveness, duration of treatment and intensity of physiotherapy for PD patients:

Patients with PD and impairment due to motor symptoms in everyday life should have access to physiotherapy treatment.

Physiotherapy should be geared towards the patient's motor deficits and use complex therapeutic approaches and be geared to the symptoms (possibly several, e.g. coordination and strength) and adapted to the patient's performance.

Physiotherapy should be carried out for at least 3 h/week. If this intensity cannot be maintained in the long term, e.g. for organizational reasons, and the patient is able to do so, part of the therapy can take the form of self-training.

Occupational therapy should be prescribed for PD patients experiencing limitations in daily activities, work participation, or upper limb dysfunctions, including impaired handwriting. PD patients should have access to artistic therapies.

## Invasive therapies

### Pump therapies

Four pump therapies (Continous subcutaneous Apomorphine infusion (CSAI), Levodopa-Carbidopa Intestinal Gel (LCIG), Levodopa/Entacapon/Carbidopa intestinalen Gel (LECIG) and Foslevodopa/Foscarbidopa-Infusion (CSFLI)) have been approved for the treatment of motor fluctuations that cannot be adequately treated conservatively.

*Continous subcutaneous Apomorphine infusion (CSAI)* should be used for the treatment of motor fluctuations to reduce off-phases, reduce dyskinesia and prolong the on-time. Continuous subcutaneous apomorphine infusion can alleviate non-motor symptoms, in particular sleep/fatigue and mood/apathy, attention/cognition as well as perception/hallucinations and attention/memory and other symptoms. These effects can be used as possible determinants in the selection of patients for apomorphine infusion therapy. Because of the complexity of the procedure and the frequent complications, Parkinson patients need to be closely monitored during apomorphine pump therapy. This treatment should only be started and continued by physicians who are experienced in this procedure.

*Levodopa‐carbidopa intestinal gel (LCIG)* is continuously delivered to the upper intestine by percutaneous endoscopic gastrostomy with jejunal tube extension (PEG‐J) using an external pump. This treatment should be used for orally insufficiently treated fluctuations. It can significantly increase on-time periods without dyskinesias and reduce off-time periods, particularly for motor fluctuations not adequately treated orally. LCIG can also improve non-motor symptoms such as sleep disturbances, apathy, gastrointestinal dysfunction, cardiovascular symptoms, attention/memory, urological symptoms and other symptoms in patients with orally uncontrollable motor fluctuations. These effects can be used as possible determinants in the selection of patients for LCIG treatment. Before commencing treatment, electrophysiological neuropathy screening should be performed, along with assessing the levels of vitamins B6, B12, and folic acid, as well as monitoring body weight. These parameters should be continually monitored during treatment, and if the values are low, appropriate substitutions or interventions should be implemented. LCIG treatment is quite safe, the most frequent complications are associated with the PEG-J. Because of the complexity of the procedure and the frequent complications, Parkinson patients should be closely monitored during LCIG pump therapy.

*Levodopa/Entacapon/Carbidopa intestinalen Gel (LECIG)* is a further PEG-J supported pump system to treat motor fluctuations in PD patients. Efficacy and side effects are similar to LCIG. Neuropathy monitoring applies as well. Special considerations should be given to additional side effects of entacapone such as diarrhoea. Because of the complexity of the procedure and the frequent complications, PD patients need to be closely monitored during LECIG pump therapy and this treatment should only be started and continued by physicians who are experienced in this procedure.

*Foslevodopa/Foscarbidopa-Infusion (CSFLI)* has been approved in Europe in 2023 for fluctuating PD patients. Foslevodopa and foscarbidopa are both prodrugs to improve absorption and tolerability of the substances, and are used for subcutaneous applications over 24 h. One randomized controlled trial is available for CSFLI treatment demonstrating its efficacy in treating motor fluctuations. Available data for long-term therapy or for clinical practice are limited due to the recent approval. Skin reactions seem to be the most frequent side effects. As this pump system has been licensed after the consensus conferences of this guideline process, no specific consensus can be reported for this therapy.

### Deep brain stimulation

*Subthalamic nucleus (STN) Deep Brain Stimulation (DBS)* should be offered to patients with PD with motor fluctuations with and without dyskinesia that cannot be adequately treated with oral medication and at least 33% improvement in PD motor symptoms using a standardized levodopa test, taking into account the contraindications such as cognitive decline and severe psychiatric symptoms or other severe comorbidities. STN-DBS should also be offered to patients with PD younger than 60 years and a disease duration of at least 4 years, with motor fluctuations with and without dyskinesia of less than 3 years duration, at least 50% improvement of motor PD symptoms by a standardized levodopa test. STN DBS should be preferred over *Globus Pallidus internus (GPi) DBS* for PD patients with motor fluctuations (with and without dyskinesias).

*Ventral intermediate nucleus (VIM) DBS* should not be used in treating PD patients with motor fluctuations (with and without dyskinesias).

*STN DBS* should also be offered to Parkinson's patients with severe, medication-resistant tremor, preferably performed bilaterally. Uni- or bilateral VIM DBS and GPi DBS are effective for treating medication-resistant PD tremor, especially when STN DBS is contraindicated. STN DBS and GPi DBS are equally effective for treating medication-resistant Parkinson's tremor, with the choice of target area based on individual symptom profiles. Uni- or bilateral VIM DBS may be considered for treating medication-resistant Parkinson's tremor if STN DBS or GPi DBS is contraindicated.

### Ablative procedures

*Pallidotomy* may be considered for advanced PD with difficult-to-control motor fluctuations when DBS or pump therapy is not suitable. Thalamotomy and subthalamotomy using radiofrequency ablation should not be performed for PD. *Radio-surgical procedures* like Gamma-Knife and Cyber-Knife are not recommended due to a lack of studies and potential high complication risks. *Magnetic resonance-guided focused ultrasound (MRgFUS)* for PD treatment is currently being evaluated in controlled studies. Unilateral MRgFUS for PD tremor has approval in Europe but should be performed within studies or registries. All ablative procedures recommended in this guideline should be performed unilaterally.

### Differential indication of invasive procedures

Invasive procedures should be considered when levodopa-dependent motor fluctuations persist despite optimizing oral/transdermal therapy. The decision for a specific procedure should consider not only motor symptom efficacy but also non-motor symptoms, patient characteristics, and individual preferences. This decision-making process should involve interdisciplinary consultation and patient engagement. PD patients should be informed about the possibility of invasive treatments when fluctuations first appear. Consideration for invasive procedures should be evaluated in PD patients meeting specific criteria, such as multiple (≥ 5) levodopa doses per day, significant off-time periods (≥ 2 h/day) or significant troublesome dyskinesia (≥ 1 h/ day). Comparisons for indications and efficacy of pump therapies and DBS are shown in Table 1.

**Table 1:** The influence of different invasive procedures on important multi-dimensional outome parameters and motor symptoms in PD

Item	Domain	CSAI	LCIG	LECIG	CSFLI	THS	MRgFUS (unilateral)
Quality of life (PDQ-39; PDQ-8)	QOL	-	+	?	?	+ +	+ +
Activities of daily living (ADL; UPDRS II)	ADL	+	+	?	+ +	+ +	+ +
Motor function in Off-state; MED-Off; (UPDRS III); Off-time	MS	+ +	+ +	+	+ +	+ +	+ +
Dyskinesias and fluctuations (UPDRS IV)	MS	+ +	+ +	+	+ +	+ +	+ +
Cardiovascular (incl. falls/orthostasis)	NMS^3^	±	+	?	?	-	?
Sleep/fatigue	NMS	+ +	±	?	?	+	?
Mood/cognition	NMS	+	±	?	?	+ +	?
Perceptual problems/ Hallucinations	NMS	+	±	?	?	+	?
Attention/ Memory	NMS	+	±	?	?	+	?
Gastrointestinal functions	NMS	+	±	?	?	+	?
Urogenital functions	NMS	+	±	?	?	+	?
Sexual functions	NMS	-	±	?	?	+	?
Other functions	NMS	+	±	?	?	+	?

*QOL*  Quality of life, *ADL* Activities of daily living, *MS* Motor symptoms, *NMS* Non-motor symptoms, *CSAI* Continuous subcutaneous apomorphine infusion, *LCIG* Levodopa/carbidopa intestinal gel, *LECIG* Levodopa/entacapone/carbidopa intestinal gel, *CSFLI* Continuous subcutaneous foscarbidopa/foslevodopa infusion, *THS* Deep brain stimulation, *MRgFUS * MR-guided focused ultrasound

- No efficacy or deterioration in open or controlled studies

? no studies or no positive expert consensus

+ improved according to expert opinion or open studies

+  + improved according to controlled studies

## Specific situations in PD

### Akinetic crisis and withdrawal syndromes

Adequate treatment for an *akinetic crisis* should be given early and sufficiently, ideally in an intermediate or intensive care unit, especially if complications develop. Risk factors that can cause an akinetic crisis, such as infections, should be treated immediately. Supportive therapy approaches such as fluid intake, thrombosis prophylaxis, fever-reducing measures and regular monitoring of vital functions should be implemented. Dopaminergic medication in the form of soluble levodopa via a nasogastric tube, subcutaneous or sublingual application of apomorphine or transdermal rotigotine should be ensured. Non-dopaminergic drugs such as intravenous amantadine sulphate should be considered or, in specific cases, benzodiazepines.

At present, no recommendation can be made on the specific treatment of *dopamine agonist withdrawal syndrome* due to a lack of evidence. Dopamine agonists should be discontinued slowly in order to be able to identify patients with dopamine agonist withdrawal syndromes at an early stage. In patients with severe, protracted dopamine agonist withdrawal syndrome, resumption of treatment with dopamine agonists should be considered.

Prevention of a *DBS-withdrawal syndrome* through close monitoring of the battery status every 3–6 months and identification of patients at particular risk of possible DBS-withdrawal syndrome should be carried out. Early resumption of effective DBS, possibly by early re-implantation, should be considered. If a pause in stimulation is unavoidable, transient bridging drug therapy approaches equivalent to those used in the akinetic crisis should be considered. Supportive therapeutic measures such as thrombosis prophylaxis and treatment of possible complications such as aspiration pneumonia can be considered.

### Pregnancy and lactation

There are no controlled trials for treatment of PD during pregnancy. There is no data, which show an increased rate for complications during pregnancy in women with PD. The following recommendations are based on case reports, case series and data from pharmacovigilance. Levodopa combined with Carbidopa should be considered if dopaminergic treatment is required during pregnancy. Medication with dopamine agonists or MAO-B inhibitors should be avoided during pregnancy due to insufficient data. Amantadine and the decarboxylase inhibitor Benserazide are contraindicated. Lactation should be avoided during a pharmacological treatment of PD due to insufficient data.

### Driving ability

A diagnosis of PD typically disqualifies holders of group 2 driving licenses (e.g., for trucks, buses, taxis) due to safety concerns. Group 1 driving licenses (e.g., for cars, motorcycles, agricultural vehicles) may be maintained for PD patients under individual assessment, especially with successful therapy or in mild cases. PD patients undergoing driving assessments should undergo motor evaluations (e.g., UPDRS III in the off-state), neuropsychological assessments, and possibly a driving behavior test. Lack of driving capability is presumed in cases of severe motor impairment, unpredictable motor fluctuations, or significant cognitive, attentional, psychomotor, or visual impairments. Patients should refrain from driving for the first 3 months after deep brain stimulation surgery.

### Care concepts

*Parkinson's day clinics* can provide complex diagnostic and therapeutic services for patients with unclear Parkinsonian syndromes or requiring intricate therapy adjustments and evaluations for invasive procedures. *Multidisciplinary inpatient therapy* (e.g., multimodal complex treatment) should be favored over standard inpatient therapy for PD patients. Integrated care models within *PD networks* and regular access to specialized PD nurses are recommended. *Palliative care* principles should ensure access to a multidisciplinary team consisting of various healthcare professionals to improve quality of life.

### Concluding remarks

This guidance aims to optimize the clinical care for treatment of PD patients with regard to diagnosis, medication and non-pharmacological treatment options. It reflects the state of the art at the beginning of 2024 and will be updated regularly. It may reflect circumstances which may apply specifically in Germany, Austria and Switzerland and may therefore not be applicable in specific aspects beyond these countries. Since this abbreviated version is of course containing very limited information, we recommend to consult the full, original version of this guideline in German language whenever possible.

### Supplementary Information

**Supplementary Material 1.**

## Data Availability

Not applicable.
