# Supplementary material for: Diagnosis and treatment of Parkinson´s disease (guideline of the German Society for Neurology)
Source: Neurol Res Pract. 2024 Jun 6;6:30. doi: 10.1186/s42466-024-00325-4 (PMC11157782; doi:10.1186/s42466-024-00325-4)
Supplement: Supplementary file 1 — Supplementary Material 1. [file 42466_2024_325_MOESM1_ESM.pdf]

AWMF-Registernummer: 030/010

Leitlinien für Diagnostik und Therapie in der Neurologie

publiziert bei: AWMF online
Portal der wissenschaftlichen Medizin

Parkinson-Krankheit

Entwicklungsstufe: S2k

Federführend: Prof. Dr. Günter Höglinger, München
Prof. Dr. Claudia Trenkwalder, Kassel/Göttingen

Herausgegeben von der Kommission Leitlinien der Deutschen Gesellschaft für Neurologie

unter Mitwirkung der beteiligten Fachgesellschaften und Organisationen

Deutsche Gesellschaft für Neurologie

Disclaimer: Keine Haftung für Fehler in Leitlinien der DGN e. V.

Die medizinisch-wissenschaftlichen Leitlinien der Deutschen Gesellschaft für Neurologie (DGN) e. V. sind systematisch entwickelte Hilfen für Ärzte und Ärztinnen zur Entscheidungsfindung in spezifischen Situationen. Sie beruhen auf aktuellen wissenschaftlichen Erkenntnissen und in der Praxis bewährten Verfahren und sorgen für mehr Sicherheit in der Medizin, sollen aber auch ökonomische Aspekte berücksichtigen. Die „Leitlinien" sind für Ärzte und Ärztinnen rechtlich nicht bindend; maßgeblich ist immer die medizinische Beurteilung des einzelnen Untersuchungs- bzw. Behandlungsfalls. Leitlinien haben daher weder – im Fall von Abweichungen – haftungsbegründende noch – im Fall ihrer Befolgung – haftungsbefreiende Wirkung.

Die Mitglieder jeder Leitliniengruppe, die Arbeitsgemeinschaft Wissenschaftlicher Medizinischer Fachgesellschaften e. V. und die in ihr organisierten Wissenschaftlichen Medizinischen Fachgesellschaften, wie die DGN, erfassen und publizieren die Leitlinien der Fachgesellschaften mit größtmöglicher Sorgfalt – dennoch können sie für die Richtigkeit des Inhalts keine rechtliche Verantwortung übernehmen. Insbesondere bei Dosierungsangaben für die Anwendung von Arzneimitteln oder bestimmten Wirkstoffen sind stets die Angaben der Hersteller in den Fachinformationen und den Beipackzetteln sowie das im einzelnen Behandlungsfall bestehende individuelle Nutzen-Risiko-Verhältnis der Patientin/des Patienten und ihrer/seiner Erkrankungen vom behandelnden Arzt oder der behandelnden Ärztin zu beachten! Die Haftungsbefreiung bezieht sich insbesondere auf Leitlinien, deren Geltungsdauer überschritten ist.

Version

AWMF-Versionsnr.: 8.1
Vollständig überarbeitet: 25. Oktober 2023
Gültig bis: 24. Oktober 2028
Kapitel: Extrapyramidalmotorische Störungen

Zitierhinweis

Höglinger G., Trenkwalder C. et al., Parkinson-Krankheit, S2k-Leitlinie, 2023, in: Deutsche Gesellschaft für Neurologie (Hrsg.), Leitlinien für Diagnostik und Therapie in der Neurologie. Online: www.dgn.org/leitlinien (abgerufen am TT.MM.JJJJ)

Korrespondenz

guenter.hoeglinger@med.uni-muenchen.de
claudia.trenkwalder@med.uni-goettingen.de

Im Internet

www.dgn.org
www.awmf.org

Herausgebende Fachgesellschaft

Deutsche Gesellschaft für Neurologie (DGN) e. V.
Reinhardtstraße 27 C, 10117 Berlin

Am Konsensusprozess beteiligte Fachgesellschaften, Berufsverbände und Organisationen

- Berufsverband deutscher Humangenetiker (BVDH) e. V.
  Priv.-Doz. Dr. med. Ulrich Finckh
- Berufsverband deutscher Neurologen (BDN) e. V.
  Prof. Dr. med. Martin Südmeyer
- Berufsverband Deutscher Psychologinnen und Psychologen (BDP) e. V.
  Prof. Dr. phil. Erich Kasten
- Deutsche Gesellschaft für Nuklearmedizin (DGN)
  Prof. Dr. Dr. med. Philipp T. Meyer
- Deutsche Gesellschaft für Geriatrie (DGG)
  Prof. Dr. med. Walter Maetzler
- Deutsche Gesellschaft für Humangenetik (GfH) e. V.
  Prof. Dr. med. Olaf Rieß
- Deutsche Gesellschaft für Psychiatrie, Psychotherapie und Nervenheilkunde (DGPPN) e. V.
  Prof. Dr. med. Frank Jessen
- Deutsche Vereinigung für Soziale Arbeit im Gesundheitswesen e. V. (DVSG)
  Regina Menzel, Stellvertretung: Alexander Thomas
- Deutscher Bundesverband für Logopädie (dbl) e. V.
  Madeleine Gausepohl
- Deutscher Verband Ergotherapie (DVE) e. V.
  Carsten Schulze, Stellvertretung: Isabel Illmann
- Deutscher Verband für Physiotherapie (ZVK) e. V.
  Marita Antony, Stellvertretung: Carl Christopher Büttner
- Gesellschaft für Neuropsychologie (GNP) e. V.
  Prof. Dr. phil. Bernd Leplow, Stellvertretung: Dr. phil. Hubert Ringendahl
- Österreichische Gesellschaft für Neurologie (ÖGN)
  Prof. Dr. med. Klaus Seppi
- Schweizerische Neurologische Gesellschaft (SNG)
  Dr. med. Ines Debove

- Deutscher Pflegerat (DPR) e. V.
  Annemarie Fajardo
- Berufsverband der Fachärzte für Psychosomatische Medizin und Psychotherapie Deutschlands (BPM) e. V.
  Dr. med. Norbert Hartkamp
- Bundesarbeitsgemeinschaft Künstlerische Therapien (BAG KT) e. V.
  Dr. phil. Stefan Mainka, Stellvertretung: Prof. Dr. phil. Sabine Koch
- Arzneimittelkommission der deutschen Ärzteschaft (AkdÄ)
  Priv.-Doz. Dr. med. Michael von Brevern
- Deutsche Gesellschaft für Neurochirurgie (DGNC) e. V.
  Prof. Dr. med. Jan Vesper
- Deutsche Gesellschaft für Klinische Neurophysiologie und Funktionelle Bildgebung (DGKN)
  Prof. Dr. med. Jens Volkmann
- Deutsche Gesellschaft für Parkinson und Bewegungsstörungen (DPG)
  Prof. Dr. med. Alexander Storch
- Arbeitsgemeinschaft Tiefe Hirnstimulation e. V. (THS AG)
  Priv.-Doz. Dr. med. René Reese, Priv.-Doz. Dr. med. Thomas Köglsperger
- Patientenvertretung
  Dr. phil. Christian Jung

Leitlinienkoordination

- Prof. Dr. med. Günter Höglinger, München
- Prof. Dr. med. Claudia Trenkwalder, Kassel/Göttingen

Leitliniensekretariate

- Prof. Dr. med. Richard Dodel, Essen (Methodiker, Leitlinienreport (siehe gesondertes Dokument))
- Editorial Office Leitlinien der DGN, Berlin

Redaktionskomitee

- Prof. Dr. med. Mathias Bähr, Göttingen
- Dr. med. Jos Becktepe, Kiel
- Prof. Dr. med. Daniela Berg, Kiel
- Priv.-Doz. Dr. med. Kathrin Brockmann, Tübingen
- Prof. Dr. med. Carsten Buhmann, Hamburg
- Prof. Dr. med. Andrés Ceballos-Baumann, München
- Prof. Dr. med. Joseph Claßen, Leipzig
- Priv.-Doz. Dr. med. Cornelius Deuschl, Essen
- Prof. Dr. med. Günther Deuschl, Kiel
- Prof. Dr. med. Georg Ebersbach, Beelitz-Heilstätten
- Prof. Dr. med. Carsten Eggers, Bottrop
- Prof. Dr. med. Thilo van Eimeren, Köln
- Ass. Prof. med. Dott.ssa mag. Dr. Alessandra Fanciulli, Innsbruck (A)
- Priv.-Doz. Dr.phil. Bruno Fimm, Aachen
- Dr. Ann-Kristin Folkerts, Köln
- Madeleine Gausepohl, Bad Segeberg
- Prof. Dr. med. Alkomiet Hasan, Augsburg
- Dr. med. Wiebke Hermann, Rostock
- Prof. Dr. med. Rüdiger Hilker-Roggendorf, Recklinghausen
- Prof. Dr. med. Günter Höglinger, München
- Priv.-Doz. Dr. med. Matthias Höllerhage, Hannover
- Prof. Dr. med. Franziska Hopfner, München
- Prof. Dr. med. Wolfgang Jost, Ortenau

- Prof. Dr. rer. nat. Elke Kalbe, Köln
- Prof. Dr. med. Jan Kassubek, Ulm
- Prof. Dr. med. Stephan Klebe, Essen
- Prof. Dr. med. Christine Klein, Lübeck
- Priv.-Doz. Dr. med. Martin Klietz, Hannover
- Priv.-Doz. Dr. med. Thomas Köglsperger, München
- Prof. Dr. med. Andrea Kühn, Berlin
- Prof. Dr. med. Paul Krack, Bern (CH)
- Priv.-Doz. Dr. med. Florian Krismer, PhD, Innsbruck (A)
- Prof. Dr. med. Dr. Gregor Kuhlenbäumer, Kiel
- Prof. Dr. med. Johannes Levin, München
- Dr. rer. nat. Inga Liepelt-Scarfone, Tübingen/Stuttgart
- Prof. Dr. med. Paul Lingor, München
- Dr. med. Kai Loewenbrück, Großschweidnitz
- Dr. med. Matthias Löhle, Rostock
- Prof. Dr. med. Stefan Lorenzl, Agatharied
- Dr. med. Sylvia Maaß, Murnau und München
- Prof. Dr. med. Walter Maetzler, Kiel
- Regina Menzel, Heidelberg
- Prof. Dr. med. Philipp T. Meyer, Freiburg i.B.
- Prof. Dr. med. Brit Mollenhauer, Kassel
- Prof. Dr. med. Manuela Neumann, Tübingen
- Prof. Dr. med. Per Odin, Lund
- Prof. Tiago Outeiro, PhD, Göttingen
- Priv.-Doz. Dr. med. Monika Pötter-Nerger, Hamburg
- Priv.-Doz. Dr. med. René Reese, Rostock
- Prof. Dr. med. Kathrin Reetz, Aachen
- Prof. Dr. med. Olaf Rieß, Tübingen
- Dr. med. Viktoria Ruf, München
- Prof. Dr. med. Anja Schneider, Bonn
- Priv.-Doz. Dr. med. Christoph Schrader, Hannover
- Prof. Dr. med. Alfons Schnitzler, Düsseldorf

- Prof. Dr. med. Klaus Seppi, Innsbruck/Kufstein (A)
- Priv.-Doz. Dr. med. Friederike Sixel-Döring, Kassel
- Prof. Dr. med. Alexander Storch, Rostock
- Prof. Dr. med. Lars Tönges, Bochum
- Prof. Dr. med. Claudia Trenkwalder, Kassel/Göttingen
- Prof. Dr. med. Thilo van Eimeren, Köln
- Prof. Dr. med. Uwe Walter, Rostock
- Prof. Dr. med. Tobias Wächter, Bad Gögging
- Prof. Dr. med. Tobias Warnecke, Osnabrück
- Prof. Dr. med. Florian Wegner, Hannover
- Prof. Dr. med. Christian Winkler PhD, Coppenbrügge
- Prof. Dr. med. Karsten Witt, Oldenburg
- Prof. Dr. med. Dirk Woitalla, Essen
- Prof. Dr. med. Kirsten Zeuner, Kiel

Hinweis

In dieser Leitlinie werden die häufig vorkommenden Personenbezeichnungen **Patientinnen/Patienten** mit **Pat.** abgekürzt. Dies hat nur redaktionelle Gründe, beinhaltet keine Wertung und meint grundsätzlich alle Geschlechter. Der Begriff **Parkinson-Krankheit** wird im weiteren Verlauf mit **PK** abgekürzt.

26.11.2023 – Bitte beachten Sie folgende Korrektur

- Tab. 6, „Dosierungsrichtlinien für die Therapie mit Dopaminagonisten“ auf S. 114 wurde durch die Dosierungsangabe für Rotigotin transdermal ergänzt. Diese fehlte in der Vorversion irrtümlich.

Was gibt es Neues?

- Empfehlung, zukünftig den Namen „Parkinson-Krankheit“ statt „idiopathisches Parkinson-Syndrom“ oder andere Begriffe zu verwenden
- Empfehlung, die MDS-Diagnosekriterien statt der UK-Brain-Bank-Kriterien bei der Diagnose Parkinson-Krankheit anzuwenden
- Neubewertung apparativer (v.a. bildgebender) und erstmals humangenetischer Zusatzuntersuchungen in der Diagnostik der Parkinson-Krankheit
- Empfehlungen zur Therapie motorischer, kognitiver, affektiver, psychotischer und dyautonomer Symptome sowie von Schlafstörungen, Schmerz, Dysarthrie und Dysphagie bei der Parkinson-Krankheit

- Empfehlungen zur Diagnose und Therapie von akinetischer Krise, Dopaminagonisten-Entzugssyndrom und THS-Entzugssyndrom sowie zur perioperativen Behandlung
- Empfehlungen zu Schwangerschaft und Stillzeit
- Aktualisierte Empfehlungen zu aktivierenden Verfahren
- Differenzialindikation nicht oraler Folgetherapien (invasiver Therapien) wie Pumpentherapien, Tiefer Hirnstimulation und ablativer Verfahren
- Aktualisierte Empfehlungen zur Fahreignung
- Bewertung von Versorgungskonzepten wie Tagesklinik, stationärer multidisziplinärer Komplextherapie, integrierten Parkinson-Versorgungsnetzwerken oder Palliative Care

Die wichtigsten Empfehlungen auf einen Blick

Definition

- Die Begriffe **„Parkinson-Krankheit“** und „idiopathisches Parkinson-Syndrom“ (IPS) wurden in der Vergangenheit häufig synonym verwendet. In den letzten Jahren wurde jedoch deutlich, dass eine nicht zu vernachlässigende Anzahl der Fälle mit Parkinson-Krankheit durch genetische Varianten verursacht werden und daher eben nicht „idiopathischer“ Natur ist. Daher empfehlen wir im Kontext dieser Leitlinie den allgemeineren Begriff Parkinson-Krankheit (PK).

Diagnose

- Zur Diagnosestellung einer Parkinson-Krankheit sollen die **MDS-Kriterien von 2015** angewendet werden. *Konsensstärke: 94,7%, Konsens.*
- Das therapeutische **Ansprechen auf eine Levodopa-Therapie** kann die Diagnosegenauigkeit verbessern. Allerdings ist die Diagnosegenauigkeit bei einem längeren Verlauf von ≥ 5 Jahren nochmals deutlich besser, sodass ein **langfristiges klinisches Follow-up** für die Diagnose der Parkinson-Krankheit einer Diagnose aufgrund einer Ansprechbarkeit auf eine Levodopa-Therapie überlegen ist. *Konsensstärke: 100%, starker Konsens.*
- Die **Ausprägung nicht motorischer Symptome** sollte bereits bei Diagnosestellung einer Parkinson-Krankheit zur Prognoseabschätzung herangezogen werden.
  Konsensstärke: 100%, starker Konsens.

Bildgebende Diagnostik

- Die **kraniale MRT (cMRT)** soll frühzeitig im Krankheitsverlauf zur Differenzialdiagnostik bei klinischem Parkinson-Syndrom erfolgen. Bei der Untersuchung der Ausschlusskriterien für eine Parkinson-Krankheit sollten cMRT-Aufnahmen mit standardisierten Sequenzen erfolgen (T1- und T2-gewichtet (idealerweise hochaufgelöst 3D) sowie fakultativ eisensensitiv/suszeptibilitäts-gewichtet und diffusionsgewichtet). *Konsensstärke 97%, starker Konsens.*
- Die **transkranielle Hirnparenchymsonographie (TCS)** durch einen qualifizierten Untersucher/eine qualifizierte Untersucherin kann zur Unterstützung der Differenzialdiagnose der Parkinson-Krankheit versus atypische und sekundäre Parkinson-Syndrome erwogen werden. Diese soll die

Beurteilung von Substantia nigra, Nucleus lentiformis sowie 3. Ventrikel einbeziehen. *Konsensstärke: 97,4%, starker Konsens*.

- Eine **FDG-PET** kann erwogen werden, wenn hinreichende klinische Hinweise auf ein atypisches Parkinson-Syndrom vorliegen und der Befund eine klinische Konsequenz hat (z.B. bzgl. Diagnose, Prognose, Therapie). *Konsensstärke: 84%, Konsens.*
- Eine **FDG-PET** kann erwogen werden, um das Risiko für das Auftreten einer Demenz bei der Parkinson-Krankheit zu bewerten, wenn der Befund eine klinische Konsequenz hat (z.B. bzgl. Diagnose, Prognose, Therapie*). Konsensstärke: 97%, starker Konsens.*
- Die **Dopamintransporter-SPECT (DAT-SPECT)** soll frühzeitig im Krankheitsverlauf zum Nachweis eines nigro-striatalen Defizits bei klinisch unklarem Parkinson- oder Tremor-Syndrom erfolgen, wenn der Befund eine klinische Konsequenz hat (z.B. bzgl. Diagnose, Prognose, Therapie). *Konsensstärke: 82,8%, Konsens.*
- Die **kardiale MIBG-Szintigraphie bzw. -SPECT** kann zur Abgrenzung einer Parkinson-Krankheit von einer MSA erwogen werden, sofern keine FDG-PET verfügbar ist. *Konsensstärke: 100%, starker Konsens.*

Genetische Diagnostik

- Eine diagnostische genetische Untersuchung soll bei Pat.-Wunsch angeboten werden, wenn entweder
  - zwei Verwandte ersten Grades oder ein Verwandter ersten und ein Verwandter zweiten Grades an einer PK erkrankt sind oder
  - bei einer Krankheitsmanifestation vor dem 50 Lebensjahr. *Konsensstärke: 96,4%, starker Konsens.*
- Bei Parkinson-Pat. mit einem Manifestationsalter über 50 Jahren, die eine genetische Untersuchung wünschen und die zwei Verwandte ersten Grades oder einen Verwandten ersten und einen Verwandten zweiten Grades mit einem Parkinson-Syndrom aufweisen, sollten mindestens *LRRK2*, *SNCA* und *VPS35* untersucht werden. Neben der Bestimmung der Sequenzabfolge müssen die angewandten Techniken auch Deletionen/Duplikationen nachweisen können. *Konsensstärke: 100%, starker Konsens.*
- Bei Parkinson-Pat. mit einer Krankheitsmanifestation vor dem 50. Lebensjahr, die eine genetische Untersuchung wünschen, sollten die Gene *PRKN*, *PINK1*, *DJ1*, *LRRK2*, *SNCA* und *VPS35* untersucht werden. Sind mehrere Familienmitglieder erkrankt, so sollte die Untersuchung präferenziell zuerst bei dem/der Pat. mit dem jüngsten Manifestationsalter durchgeführt werden. Neben der Bestimmung der Sequenzabfolge müssen die angewandten Techniken auch Gen-Deletionen/Duplikationen nachweisen können. *Konsensstärke: 100%, starker Konsens.*
- Sollte trotz eines negativen Befunds der unter den in den beiden vorgenannten Empfehlungen genannten Untersuchungen der Verdacht auf eine genetische Ursache der Parkinson-Krankheit fortbestehen, so sollte, wenn die/der Pat. dies wünscht, eine/ein neurogenetisch spezialisierte/r Neurologin/Neurologe oder eine/ein Humangenetikerin/Humangenetiker konsultiert werden,

um das weitere klinisch-diagnostische Vorgehen zu planen.
Konsensstärke: 100%, starker Konsens.

- Untersuchungen zur Erfassung des polygenen Risikos sollten nicht routinemäßig durchgeführt werden. *Konsensstärke: 96,2%, starker Konsens.*
- Untersuchungen zur Erfassung genetischer Varianten im *GBA1*-Gen sollten nicht routinemäßig durchgeführt werden. Bei einer isoliert erscheinenden PK sowie einer Krankheitsmanifestation vor dem 50. Lebensjahr oder bei Parkinson-Pat. mit raschem Verlauf (vor allem schnelle kognitive Verschlechterung nach klinischer Diagnosestellung) kann eine Untersuchung der *GBA1* erfolgen, wenn die/der Pat. dies nach sorgfältiger Aufklärung über Vor- und Nachteile wünscht. *Konsensstärke: 96,2%, starker Konsens.*
- Die Diagnose einer hereditären PK erlaubt keine sichere Vorhersage für die/den einzelne/n Pat. In Hinsicht auf Überlebensdauer, Lebensqualität und kognitive Einbußen. *Konsensstärke: 100%, starker Konsens.*
- Für die monogene Parkinson-Krankheit gelten die gleichen medikamentösen Therapieempfehlungen wie bei einer genetisch komplexen Parkinson-Krankheit. *Konsensstärke: 100%, starker Konsens.*
- Eine Tiefe Hirnstimulation ist bei monogener Parkinson-Krankheit möglich. Dabei gelten die gleichen Ein- und Ausschlusskriterien wie bei einer genetisch komplexen Parkinson-Krankheit. *Konsensstärke: 100%, starker Konsens.*

Therapie

- **Levodopa-Präparate:** *Konsensstärke: 92,9%, Konsens.*
  - Levodopa-Präparate können unter Beachtung der unten dargelegten Differenzialindikationen zur Therapie der Parkinson-Krankheit eingesetzt werden.
  - Eine Priorisierung der Präparate auf der Basis des Dopadecarboxylase-Inhibitors (Carbidopa oder Benserazid) kann aus der Literatur nicht abgeleitet werden.
  - Retardierte Darreichungsformen von Levodopa mit Decarboxylase-Hemmer sollen nicht zur Therapie von Pat. mit der Parkinson-Krankheit während der Wachzeit verwendet werden, sondern nur zur Behandlung der Parkinson-Symptome während der Nachtzeit.
  - Schnell lösliche orale und inhalative Levodopa-Formulierungen können zur Coupierung von Off-Situationen eingesetzt werden; inhalatives Levodopa kann aber nur in Kombination mit einem oralen Levodopa-Präparat eingesetzt werden, da es nicht mit einem Dopadecarboxylase-Hemmer kombiniert ist.
- **Dopaminagonisten:** *Konsensstärke: 96%, starker Konsens.*
  - Ergoline Dopaminagonisten (Bromocriptin, Cabergolin, Pergolid) sollen nicht mehr zur Therapie der Parkinson-Krankheit eingesetzt werden.
  - **Non-ergoline Dopaminagonisten (Pramipexol, Ropinirol, Piribedil, Rotigotin,** mit starken Einschränkungen **Apomorphin)** können unter Beachtung der unten dargelegten Differenzialindikationen zur Therapie der Parkinson-Krankheit eingesetzt werden.
  - Apomorphin steht zur s.c. Injektion oder Infusion bzw. als sublingual anwendbarer Film zur Verfügung und ist daher an spezifische Indikationen gebunden.

- Pramipexol und Ropinirol als Tabletten in Retard-Formulierungen und Rotigotin als transdermales Pflaster ermöglichen eine einmal tägliche Gabe.
- Eine Priorisierung der verschiedenen Dopaminagonisten untereinander hinsichtlich der Wirksamkeit kann nicht eindeutig aus der Literatur abgeleitet werden.
- Bei begleitender Medikation mit Medikamenten, die CYP1A2 induzieren oder hemmen, sollte eine Dosisanpassung von Ropinirol oder ein Wechsel auf einen anderen Dopaminagonisten erwogen werden.
- Bei eingeschränkter Leberfunktion sollte erwogen werden, Pramipexol, das zum größten Teil über die Niere verstoffwechselt wird, zu priorisieren.
- Bei eingeschränkter Nierenfunktion sollte erwogen werden, Ropinirol, Rotigotin oder Piribedil gegenüber Pramipexol zu priorisieren.

- **COMT-Hemmer:** *Konsensstärke: 100%, starker Konsens.*
  - **Opicapon** und **Entacapon** sind als COMT-Hemmer in der Wirkung weitgehend gleichwertig und können unter Beachtung der unten dargelegten Differenzialindikationen zur Behandlung von Fluktuationen bei der Parkinson-Krankheit eingesetzt werden.
  - **Tolcapon** sollte wegen der Hepatotoxizität nur als Mittel der zweiten Wahl und unter engmaschiger Sicherheitsmonitorierung (klinisch und laborchemisch) verwendet werden.

- **MAO-B-Hemmer:** *Konsensstärke: 100%, starker Konsens.*
  - Eine Priorisierung der verschiedenen MAO-B-Hemmer untereinander hinsichtlich der Wirksamkeit kann aus der Literatur nicht eindeutig abgeleitet werden.
  - Die MAO-B-Hemmer **Selegilin** oder **Rasagilin** können unter Beachtung der unten dargelegten Differenzialindikationen als Monotherapie der frühen Parkinson-Krankheit oder in Kombination mit Levodopa zur Therapie der Parkinson-Krankheit mit Wirkfluktuationen eingesetzt werden.
  - Der MAO-B-Hemmer **Safinamid** mit dualem Wirkmechanismus ist nicht als Monotherapeutikum zugelassen, kann aber in Kombination mit Levodopa unter Beachtung der unten dargelegten Differenzialindikationen zur Therapie der Parkinson-Krankheit mit Wirkfluktuationen eingesetzt werden.

- **NMDA-Rezeptor-Antagonisten:** *Konsensstärke: 95,2%, starker Konsens.*
  - **Amantadin** kann unter Beachtung der unten dargelegten Differenzialindikationen zur Therapie der Parkinson-Krankheit eingesetzt werden.
  - Budipin wird wegen seines Nebenwirkungsprofils nicht mehr empfohlen.

- **Anticholinergika:** *Konsensstärke: 96,4%, starker Konsens.*
  - Anticholinergika sollen aufgrund eines im Vergleich zu Therapie-Alternativen ungünstigen Nutzen-Risiko-Profils nicht als Antiparkinsonmittel eingesetzt werden.
  - Nur noch in absoluten Ausnahmefällen kann ein Einsatz bei Tremor erwogen werden.

- **Initiale Monotherapie:** *Konsensstärke: 100%, starker Konsens.*

- Bei der Auswahl der verschiedenen Substanzklassen in der initialen Monotherapie sollen die unterschiedlichen Effektstärken im Hinblick auf die Wirkung, die Nebenwirkungen, das Alter der Pat., Komorbiditäten und das psychosoziale Anforderungsprofil berücksichtigt werden.
- Motorische Fluktuationen und Dyskinesien sind nach initialer Monotherapie mit Levodopa v.a. bei hoher Dosierung und pulsatiler Anwendung im Krankheitsverlauf früher zu beobachten als unter initialer Monotherapie mit MAO-B-Hemmer oder Dopaminagonisten.
- Bei biologisch jüngeren Pat. sollte eine Bevorzugung von **Dopaminagonisten** oder **MAO-B-Hemmern** gegenüber Levodopa erwogen werden.
- Pat., die **Levodopa** in der initialen Monotherapie benötigen, sollen dieses auch erhalten.
- Gründe für den initialen Einsatz von Levodopa könnten z.B. sein
    - Schweregrad der Symptome
    - Notwendigkeit eines rasch einsetzenden therapeutischen Effekts
    - Multimorbidität
    - beobachtete oder zu erwartende Nebenwirkungen anderer Medikamentengruppen (z.B. Impulskontrollstörungen bei Agonisten)
    - ggf. bessere individuelle Verträglichkeit.

- **Kombinationstherapie:** Ausgehend von einer initialen Monotherapie, sollte eine pharmakologische Kombinationstherapie dann angeboten werden, wenn

  - die Wirksamkeit der initialen Monotherapie bei mittlerer Erhaltungsdosis auf die Dopa-sensitiven Zielsymptome unzureichend ist oder
  - die zur Symptomkontrolle notwendige Dosierung der Monotherapie aufgrund von therapie-limitierenden Nebenwirkungen nicht erreicht werden kann.
  - *Konsensstärke: 100%, starker Konsens.*

- **Fluktuationen:** *Konsensstärke: 100%, starker Konsens.*

  Zur Behandlung von Fluktuationen stehen verschiedene medikamentöse Therapieoptionen zur Verfügung:

  - Fraktionierung der Levodopa-Gaben und ggf. Dosisänderung
  - zusätzliche Gaben von Levodopa-Präparaten mit modifizierter Galenik (lösliches, inhalatives oder retardiertes Levodopa)
  - zusätzliche Gaben von Dopaminagonisten
  - zusätzliche Gabe von MAO-B-Hemmern oder
  - zusätzliche Gabe von COMT-Hemmern.
    Eine Priorisierung der einzelnen therapeutischen Optionen für Parkinson-Pat. mit Fluktuationen kann auf Grundlage von Studien nicht erfolgen. Die individuelle Reihenfolge der Therapieoptionen soll das Wirkspektrum, das Nebenwirkungsprofil und den Pat.wunsch berücksichtigen.

- **Dyskinesien:**

  - Amantadin sollte zur Reduktion von Dyskinesien bei Parkinson-Pat. mit Levodopa-induzierten motorischen Komplikationen unter Berücksichtigung anticholinerger und halluzinogener Nebenwirkungen eingesetzt werden. *Konsensstärke: 100%, starker Konsens.*

- Safinamid kann zur Behandlung mittelgradiger bis schwerer Dyskinesien erwogen werden. Die Datenlage zu Wirkung und Dosis von Safinamid auf Dyskinesien ist nicht eindeutig. *Konsensstärke: 85,7%, Konsens.*

- **Tremor:**
  - Gegen Parkinson-Tremor, der refraktär gegenüber Standarddosen von Levodopa ist, können im Einzelfall eine Erhöhung der Levodopa-Tagesdosis oder hohe Levodopa-Einzeldosen hilfreich sein. Eine dauerhafte Erhöhung der Levodopa-Dosis bei schwer behandelbarem Tremor sollte jedoch gegen ein erhöhtes Risiko motorischer Komplikationen abgewogen werden. *Konsensstärke: 100%, starker Konsens.*
  - Dopaminagonisten sollten zur Therapie der Symptome der Parkinson-Krankheit in Monotherapie und Kombinationstherapie verwendet werden. Es wird empfohlen, die Einstellung mit den Zielsymptomen Akinese und Rigor vorzunehmen, wobei sich meist äquivalent auch der Parkinson-Tremor bessert. *Konsensstärke: 100%, starker Konsens.*
  - Der Einsatz von Anticholinergika bei Parkinson-Pat. kann wegen der anticholinergen Nebenwirkungen nur noch in absoluten Ausnahmefällen bei anderweitig nicht behandelbarem Tremor erwogen werden. *Konsensstärke: 100%, starker Konsens.*
  - Betablocker können für die Therapie des posturalen Parkinson-Tremors erwogen werden (off-label). *Konsensstärke: 96,0%, starker Konsens.*
  - Die Erstbehandlung von Parkinson-Pat. mit Tremor richtet sich zunächst nach den allgemeinen Therapieprinzipien. Wenn diese Medikamente anhand der Zielsymptome Akinese und Rigor ausdosiert sind, kommen die besonderen hier genannten Interventionen (THS, Pumpentherapien) in Betracht. Die Wahl der initialen Medikation hängt von klinischen Faktoren wie Alter, Komorbiditäten und Schwere der motorischen Symptome ab. *Konsensstärke: 100%, starker Konsens.*

- **Schmerzen:** *Konsensstärke: 100%, starker Konsens.*
  - Basis der Therapie ist die Optimierung der Anti-Parkinson-Medikation.
  - Nozizeptive Schmerzen sollten gemäß dem 3-Stufen-WHO-Schema behandelt werden.
  - Neuropathische Schmerzen sollten mit Antikonvulsiva und/oder Antidepressiva gemäß der Leitlinie zur Behandlung neuropathischer Schmerzen behandelt werden, wobei bevorzugt Gabapentin und/oder Duloxetin (insbesondere bei Komorbidität mit Depression) eingesetzt werden sollten.
  - Bei ausgeprägten Schmerzen kann eine Behandlung mit retardiertem Oxycodon/Naloxon in Erwägung gezogen werden.

- **Blasenfunktionsstörungen:** *Konsensstärke: 100%, starker Konsens.*
  - Als nicht medikamentöse Therapiemaßnahmen bei der neurogenen Blasenstörung im Rahmen der Parkinson-Krankheit sollten das Blasentraining, die tageszeitliche Anpassung der Flüssigkeitsaufnahme und die Vermeidung von Koffein, Alkohol und kohlensäurehaltigen Getränken angewendet werden.

- Antimuskarinika (Solifenacin, Trospium, Darifenacin) sollten zur medikamentösen Behandlung des vermehrten Harndrangs und der Harndranginkontinenz aufgrund einer Überaktivität des Blasendetrusors bei Parkinson-Pat. erwogen werden.
- B3-Rezeptor-Agonisten (bspw. Mirabegron) können zur Behandlung des vermehrten Harndrangs und der Harndranginkontinenz aufgrund von Blasendetrusorüberaktivität bei jenen Parkinson-Pat. erwogen werden, die unzureichend auf Antimuskarinika angesprochen haben, die Antimuskarinika nicht vertragen haben oder bei denen Antimuskarinika kontraindiziert sind.
- Zur Behandlung der Detrusorüberaktivität kann bei Pat., die auf orale Therapieversuche unzureichend angesprochen haben, die intravesikale Injektion von Botulinumtoxin A erwogen werden, sofern das motorische und kognitive Leistungsniveau der Pat. einen nachfolgend nötigen, intermittierend sauberen Selbstkatheterismus erlaubt.
- Zur nicht pharmakologischen Behandlung der Nykturie sollte Pat. geraten werden, die Flüssigkeitsaufnahme am späten Nachmittag/Abend einzuschränken, abendlichen Alkoholkonsum zu vermeiden und eine Oberkörperhochlagerung um 10-20° während des Schlafens anzustreben.
- Zur Therapie der nächtlichen Polyurie kann unter engmaschigen Blutdruck-, Serumelektrolyt- und Körpergewichtskontrollen eine abendliche Therapie mit Desmopressin erwogen werden.
- Zur Therapie der Nykturie aufgrund verminderter Blasenkapazität sollte bei Parkinson-Pat. eine Therapie mit Antimuskarinika (Solifenacin, Trospium, Darifenacin) erwogen werden.

- **Orthostatische Hypotonie:** *Konsensstärke: 100%, starker Konsens.*

  - Zur Therapie der orthostatischen Hypotonie bei Pat. mit der Parkinson-Krankheit sollte folgendes Stufenschema angewendet werden:
    1. aggravierende/auslösende Faktoren beseitigen/behandeln (Infektionskrankheiten, Dehydratation etc.)
    2. Überprüfung der Begleitmedikation (falls Antihypertensiva angewandt werden, sollte eine Dosisreduktion/ein Absetzen angestrebt werden)
    3. nicht medikamentöse Therapiemaßnahmen anwenden (vgl. nachfolgende Empfehlungen)
    4. Beginn blutdrucksteigernder Medikamente.

- **Obstipation:** *Konsensstärke: 100%, starker Konsens.*

  - Zur Therapie einer Obstipation bei Parkinson-Pat. sollen die allgemeinen Therapieempfehlungen der AWMF-Leitlinie „Chronische Obstipation“ herangezogen werden.

- **Schlafstörungen:** *Konsensstärke: 95%, Konsens.*

  - Komorbide primäre Schlafstörungen wie RLS und SBAS sollen gemäß den für diese Entitäten gültigen Leitlinien therapiert werden.

- Ergeben sich Hinweise darauf, dass motorische oder nicht motorische dopaminerge Wirkfluktuationen für die Schlafstörung verantwortlich sind, soll eine entsprechende Anpassung der dopaminergen Pharmakotherapie erfolgen.
- Die RBD soll durch das Schaffen einer sicheren Schlafumgebung behandelt werden. Des Weiteren können Clonazepam und/oder Melatonin unter Beachtung möglicher Nebenwirkungen erwogen werden.
- Bei der Insomnie oder bei zirkadianen Rhythmusstörungen sollten zugrunde liegende Ursachen wie Medikationsnebenwirkungen und/oder primäre Schlafstörungen wie SBAS ausgeschlossen werden. Nach Ausschluss spezifisch behandelbarer Ursachen der Schlafstörungen sollten diese durch schlafhygienische Maßnahmen, intensives körperliches Training und Lichttherapie behandelt werden. Eszopiclon, Doxepin, Zolpidem, Trazodon, Melatonin, Venlafaxin (bei komorbider Depression), Nortriptylin oder Mirtazapin können bei insgesamt schwacher Evidenzlage bei der Insomnie erwogen werden.

▪ **Kognitive Störungen:**

- Rivastigmin, Donepezil und Galantamin sollen nicht zur Behandlung einer Parkinson-Krankheit mit Mild Cognitive Impairment (PD-MCI) eingesetzt werden.
  Konsensstärke: 89,7%, Konsens.
- Kognitives Training sollte bei Personen mit PD-MCI angeboten werden.
  Konsensstärke: 96,9%, starker Konsens.
- Ausdauertraining soll bei PD-MCI im aeroben Bereich durchgeführt werden und 2- bis 3-mal 45 bis 60 Minuten pro Woche durchgeführt werden.
  Konsensstärke: 96,8%, starker Konsens.
- Rivastigmin soll zur Behandlung der Parkinson-Krankheit mit Demenz (PKD) eingesetzt werden. *Konsensstärke: 96,6%, starker Konsens.*
- Donepezil kann zur Behandlung der PKD eingesetzt werden. Dabei handelt es sich um einen „Off-Label Use“. *Konsensstärke: 96,6%, starker Konsens.*
- Galantamin sollte nicht zur Behandlung einer PKD eingesetzt werden.
  Konsensstärke: 96,6%, starker Konsens.
- Kognitive Stimulation sollte bei Personen mit PKD angeboten werden.
  Konsensstärke: 96,8%, starker Konsens.

▪ **Affektive Störungen:**

- Zur Behandlung von depressiven Störungen bei Parkinson-Pat. soll die optimale dopaminerge Medikation eingesetzt werden und eine Therapie mit Pramipexol durchgeführt werden, falls eine Dopaminagonisten-Therapie individuell möglich ist. In zweiter Linie kann der Dopaminagonist Rotigotin eingesetzt werden.
  Konsensstärke: 100%, starker Konsens.
- Die Behandlung der schweren Depression bei Parkinson-Pat. kann erfolgen mit
  - Venlafaxin 75 bis 225 mg
  - Desipramin 25 bis 200 mg

  Konsensstärke: 100%, starker Konsens.

- Die Behandlung der mittelgradigen Depression bei Parkinson-Pat. kann folgendermaßen erfolgen:
- bei Antriebshemmung:
  - Venlafaxin 75 bis 150 mg
  - Citalopram 20 bis 40 mg oder
  - Sertralin 50 bis 100 mg
- bei Agitiertheit, Angst, Unruhe oder Schlafstörung:
  - Mirtazapin 15 bis 45 mg (nicht bei REM-Schlaf-Verhaltensstörung) oder
  - Trazodon 100 bis 200 mg
- bei Komorbidität von Schlafstörung, Schmerz, Speichelfluss und kognitiv unauffälligen Pat.:
  - Amitriptylin 10 bis 75 mg retard.

  Konsensstärke: 100%, starker Konsens.
- Zur Behandlung einer Anhedonie bei Parkinson-Pat. soll die optimale dopaminerge Medikation mit L-Dopa und/oder Rotigotin, Pramipexol oder Piribedil eingesetzt werden. *Konsensstärke: 100%, starker Konsens.*
- Zur Behandlung der Apathie bei Parkinson-Pat. soll die optimale dopaminerge Medikation eingesetzt werden und kann eine Therapie mit den Dopaminagonisten Pramipexol, Rotigotin oder Piribedil durchgeführt werden, falls eine Dopaminagonisten-Therapie individuell möglich ist. *Konsensstärke: 100%, starker Konsens.*
- Die Apathie bei der Parkinson-Krankheit kann mit folgender Medikation behandelt werden:
  - Venlafaxin 75 bis 225 mg retard oder
  - Nortriptylin 25 bis 150 mg.

  Konsensstärke: 96,2%, starker Konsens.
- Zur Behandlung von Angststörungen mit affektiven Fluktuationen bei Parkinson-Pat. soll die optimale dopaminerge Medikation eingesetzt werden, und eine Therapie mit einem Non-Ergot-Dopaminagonisten durchgeführt werden, falls eine Dopaminagonisten-Therapie individuell möglich ist. *Konsensstärke: 100%, starker Konsens.*
- Konstante Angststörungen ohne affektive Fluktuationen bei Parkinson-Pat. sollten nicht mittels einer angepassten dopaminergen Therapie behandelt werden. *Konsensstärke: 100%, starker Konsens.*
- Bei Angststörungen bei der Parkinson-Krankheit kann ein Therapieversuch mit Citalopram 20 bis 40 mg unternommen werden. *Konsensstärke: 95,0%, starker Konsens.*
- Zur Behandlung von Fatigue bei Parkinson-Pat. soll die optimale dopaminerge Medikation eingesetzt werden und kann eine Therapie mit Rotigotin durchgeführt werden, falls eine Dopaminagonisten-Therapie individuell möglich ist. *Konsensstärke: 100%, starker Konsens.*
- Zur Behandlung von Fatigue bei Parkinson-Pat. kann ein Therapieversuch mit Modafinil 100 bis 200 mg oder Safinamid 100 mg erwogen werden. *Konsensstärke: 96,2%, starker Konsens.*

- **Impulskontroll-Störungen (IKS):** *Konsensstärke: 100%, starker Konsens.*
  - Vor dem Einsatz von Dopaminagonisten sollte der Patient für das Auftreten einer IKS sensibilisiert werden.

- Das Absetzen des Dopaminagonisten ist eine effektive Behandlung einer IKS. Wir empfehlen eine schrittweise Reduktion der Dopaminagonisten.
- Im Falle von unerwünschten Wirkungen der Dopaminagonisten (z.B. Dopaminagonisten-Entzugssyndrom) sollten die Dopaminagonisten auf die niedrigste tolerierte Dosierung reduziert werden.
- Für Pat. mit einer Indikation für eine nicht orale Folgetherapie kann eine Levodopa-Carbidopa-Intestinal-Gel (LCIG)-Therapie zur Behandlung einer IKS erwogen werden.
- Eine kognitive Verhaltenstherapie kann zur Behandlung einer IKS eingesetzt werden.
- Die bilaterale STN-DBS ist eine effektive Therapie zur Behandlung einer IKS für Pat., die eine Indikation für eine nicht orale Folgetherapie haben.

- **Psychose:** *Konsensstärke: 100%, starker Konsens.*

  - Bei Parkinson-Pat. mit psychotischen Symptomen soll ein gestuftes Vorgehen erfolgen:
    1. Durchführung von allgemeinen nicht pharmakologischen Maßnahmen (u.a. Reizabschirmung, reorientierende Maßnahmen, Reetablierung eines zirkadianen Rhythmus)
    2. Durchführung allgemeiner therapeutischer Maßnahmen (u.a. Exiskose Behandlung, Behandlung eines Infekts)
    3. Reduktion/Anpassung auslösender Medikamente allgemein (anticholinerg, antiglutamaterg, sedierend) und Anti-Parkinson-Medikation (v.a. Amantadin, MAO-B-Hemmer, Dopaminagonisten und COMT-Hemmer, Kombinationsbehandlungen)
    4. Clozapin soll bei Versagen von 1–3 nach entsprechender Risiko-Nutzen-Abwägung (Agranulozytose-Risiko, Myokarditis-Risiko, Sturzgefahr, anticholinerge Nebenwirkungen) angeboten werden. Alternativ kann Quetiapin off label bei Parkinson-Pat. ohne kognitive Einschränkung angeboten werden.
    5. Bei kognitiver Störung und Versagen von 1–3 kann eine Umstellung auf einen Acetylcholinesterase-Hemmer angeboten werden.
  - Bei Versagen von 1–4 und ggfs. 5 kann eine Elektrokonvulsionsbehandlung angeboten werden.

- **Delir:** *Konsensstärke: 93.1%, Konsens.*

  - Beim Delir bei der Parkinson-Krankheit sollten Amantadin, Anticholinergika und Dopaminagonisten reduziert oder (ausschleichend) abgesetzt werden.

- **Dysarthrie/Dysphagie:**

  - Pat. mit Parkinson-bedingten Sprechstörungen sollen eine logopädische Sprechtherapie erhalten. *Konsensstärke: 97,0%, starker Konsens.*

- Pat. mit PK-bedingten Schluckstörungen sollen eine logopädische Schlucktherapie erhalten. *Konsensstärke: 94,1%, Konsens.*

- **Aktivierende Verfahren:** *Konsensstärke: 100%, starker Konsens*
  - Pat. mit der Parkinson-Krankheit und Beeinträchtigung durch motorische Symptome im Alltag sollen Zugang zu einer **physiotherapeutischen Behandlung** erhalten.
  - **Ergotherapie** sollte Menschen mit einer Parkinson-Krankheit verordnet werden, die unter Einschränkungen bei den Aktivitäten des täglichen Lebens, der beruflichen Teilhabe oder unter Funktionsstörungen der oberen Extremitäten, einschließlich einer Beeinträchtigung der Handschrift, leiden.
  - Bezüglich der Effektivität von **künstlerischen Therapien** bei Parkinson-Pat. Kann folgende Empfehlung gegeben werden: Parkinson-Pat. Sollten Zugang zu künstlerischen Therapien erhalten.

Invasive Therapien

- **Pumpentherapien:** *Konsensstärke: 100%, starker Konsens.*
  - Die Apomorphin-Pumpentherapie sollte für die Behandlung motorischer Fluktuationen zur Reduktion von Off-Phasen, zur Reduktion von Dyskinesien und zur Verlängerung der On-Zeit angewandt werden.
  - Die Levodopa-Carbidopa-Intestinal-Gel (LCIG)-Therapie kann die Dauer von On-Zeiten ohne störende Dyskinesien signifikant erhöhen und die Off-Zeiten signifikant reduzieren und sollte daher zur Behandlung oral nicht befriedigend zu behandelnder motorischer Fluktuationen angewandt werden.

- **Tiefe Hirnstimulation (THS):**
  - Die **STN-THS** soll unter Beachtung der Kontraindikationen Pat. mit einer Parkinson-Krankheit mit konservativ-medikamentös nicht ausreichend behandelbaren motorischen Fluktuationen mit und ohne Dyskinesien und mindestens 33%iger Verbesserung der motorischen Parkinson-Symptome durch einen standardisierten Levodopa-Test angeboten werden. *Konsensstärke: 96,2%, starker Konsens.*
  - Die **STN-THS** sollte ebenso Pat. mit einer Parkinson-Krankheit und mindestens 4-jähriger Krankheitsdauer, mit motorischen Fluktuationen mit und ohne Dyskinesien von weniger als 3 Jahren Dauer, mindestens 50%iger Besserung der motorischen Parkinson-Symptome durch einen standardisierten Levodopa-Test und jünger als 60 Jahren angeboten werden. *Konsensstärke: 96,2%, starker Konsens.*
  - Die **STN-THS** sollte der GPi-THS in den differenzialtherapeutischen Abwägungen bei der PK mit motorischen Fluktuationen mit und ohne Dyskinesien vorgezogen werden. *Konsensstärke: 100%, starker Konsens.*
  - Eine **VIM-THS** soll in der Behandlung der PK mit motorischen Fluktuationen mit und ohne Dyskinesien nicht eingesetzt werden. *Konsensstärke: 100%, starker Konsens.*

- Die **STN-THS** soll unter Beachtung der Kontraindikationen Pat. mit einer Parkinson-Krankheit mit schwerem, konservativ medikamentös nicht ausreichend behandelbarem Tremor angeboten werden und vorzugsweise bilateral erfolgen.
  Konsensstärke: 92,3%, Konsens.
- Die uni- oder bilaterale **VIM-THS** und die **GPi-THS** sind bei medikamentös nicht kontrollierbarem Parkinson-Tremor wirksam und können bei Kontraindikationen für eine **STN-THS** erwogen werden. *Konsensstärke: 92,3%, Konsens.*
- **STN-THS** und **GPi-THS** sind gleichwertig wirksam in der Behandlung des pharmakoresistenten Parkinson-Tremors. Die Wahl des Zielgebiets bei Pat. mit der Parkinson-Krankheit und medikamentös nicht kontrollierbarem Parkinson-Tremor soll daher unter Berücksichtigung des Weiteren individuellen Symptomprofils erfolgen. *Konsensstärke: 92,3%, Konsens.*
- Die uni- oder bilaterale **VIM-THS** ist bei medikamentös nicht kontrollierbarem Parkinson-Tremor wirksam und kann bei Kontraindikationen für eine STN-THS oder GPi-THS erwogen werden. *Konsensstärke: 92,3%, Konsens.*

- **Ablative Verfahren:** *Konsensstärke: 100%, starker Konsens.*

  - Die **Pallidotomie** kann bei fortgeschrittener Parkinson-Krankheit erwogen werden, wenn medikamentös schwer zu kontrollierende Wirkfluktuationen vorliegen und zugleich eine Therapie mittels THS oder Pumpentherapie nicht infrage kommt.
  - Die Thalamo- und auch Subthalamotomie mittels Radiofrequenzablation soll bei der Parkinson-Krankheit nicht durchgeführt werden.
  - Die Behandlung mittels radiochirurgischer Verfahren (Gamma-Knife, Cyber-Knife) ist mangels Studien und aufgrund des potenziell hohen Komplikationsrisikos nicht zu empfehlen.
  - Zum Einsatz des MRgFUS bei der Behandlung der Parkinson-Krankheit werden gegenwärtig die notwendigen Studien durchgeführt. Zur Behandlung des Parkinson-Tremors mit unilateraler MRgFUS gibt es in Europa bereits eine Zulassung für alle Kerngebiete (VIM, STN, GPi). Allerdings sollte diese Intervention aktuell im Rahmen von Studien oder Registern durchgeführt werden.
  - Alle ablativen Verfahren, die in dieser Leitlinie empfohlen werden, sollten nur unilateral eingesetzt werden.

- **Differenzialindikation invasiver Verfahren:**

  - Prinzipiell sollten invasive Verfahren insbesondere dann erwogen werden, wenn beeinträchtigende Levodopa-abhängige Wirkfluktuationen auftreten, die sich durch eine Optimierung der oralen/transdermalen Therapie nicht ausreichend verbessern lassen.
    Konsensstärke: 100%, starker Konsens.
  - Die Entscheidung für ein bestimmtes Verfahren soll neben der Wirksamkeit auf die motorische Symptomatik insbesondere auch nicht motorische Symptome und Pat.-Charakteristika sowie die individuelle Präferenz der Pat. mit einbeziehen, wobei die Faktoren im Einzelfall zu gewichten sind und in einer interdisziplinären Fallkonferenz und zusammen mit dem Pat. abgewogen werden sollen. *Konsensstärke: 100%, starker Konsens.*

- Pat. mit der PK sollten spätestens beim Auftreten erster Fluktuationen über die Möglichkeit invasiver Behandlungen informiert werden. *Konsensstärke: 95,2%, starker Konsens.*
- Bei Pat. mit einer Parkinson-Krankheit und mindestens einem der folgenden Kriterien soll die Indikation für ein invasives Verfahren geprüft werden:
    - ≥ 5 Einnahmezeitpunkte Levodopa/Tag (entsprechend Einnahmeintervallen von < 3 h)
    - ≥ 2 h Off-Symptome/Tag
    - ≥ 1 h störende Dyskinesien/Tag
      Konsensstärke: 95,2%, starker Konsens.

- **Fahreignung:** *Konsensstärke: 100%, starker Konsens*.
    - Mit der Diagnose einer Parkinson-Krankheit ist für Inhaber der Fahrerlaubnis für **Kraftfahrzeuge der Gruppe 2** (Lkw, Bus, Taxi, Personenbeförderung) die Fahreignung in der Regel nicht gegeben.
    - Die Fahrerlaubnis für **Kraftfahrzeuge der Gruppe 1** (Pkw, Krafträder, landwirtschaftliche Zugmaschinen) kann bei Parkinson-Pat. nach individueller Beurteilung bei erfolgreicher Therapie oder in leichten Fällen gegeben sein.
    - Bei der **Fahreignungsuntersuchung** von Parkinson-Pat. sollten außer der Erhebung motorischer Parameter (z.B. UPDRS III im Off) neuropsychologische Verfahren, die u.a. die Prüfung von visuell-räumlichen Aufmerksamkeitsleistungen, geteilter Aufmerksamkeit, exekutiven Funktionen und visuellen Wahrnehmungsleistungen erfassen, sowie ggf. eine Fahrverhaltensprobe durchgeführt werden.
    - Von einer **fehlenden Fahreignung** ist auszugehen bei schwerer motorischer Beeinträchtigung und/oder unvorhersagbaren ausgeprägten motorischen On/Off-Fluktuationen sowie höhergradigen Störungen der visuellen Funktion, Kognition, Aufmerksamkeit, Psychomotorik, Impulskontrolle oder Halluzinationen.
    - In den **ersten 3 Monaten postoperativ nach THS-Operation** sollen Pat. kein Kraftfahrzeug führen.

- **Versorgungskonzepte:** *Konsensstärke: 100%, starker Konsens.*
    - In einer **Parkinson-Tagesklinik** können Pat. mit unklaren Parkinson-Syndromen zur komplexen Diagnostik und Parkinson-Pat. für komplexe medikamentöse Therapieanpassungen, Voruntersuchungen, Beginn und Nachuntersuchungen von invasiven Verfahren wie Pen- oder Pumpentherapien oder der Tiefen Hirnstimulation aufgenommen werden, die ambulant nicht umgesetzt werden können.
    - Die **stationäre multidisziplinäre Therapie** (z.B. multimodale Komplexbehandlung) sollte gegenüber der stationären Standardtherapie der Parkinson-Krankheit bevorzugt eingesetzt werden.
    - Es kann empfohlen werden, dass Pat. im Sinne eines integrierten Versorgungsansatzes in **Parkinson-Netzwerken** behandelt werden.

- Es kann empfohlen werden, dass Personen mit PK regelmäßigen Zugang zu einer Betreuung durch eine **spezialisierte Parkinson-Nurse** haben.
- Als Grundsätze für **Palliative Care** (sowohl ambulant als auch stationär) können u.a. gelten: Pat. sollten Zugang zu einem multidisziplinären Team haben, das u.a. aus Physiotherapeut/Physiotherapeutin, Ergotherapeut/Ergotherapeutin, Logopäde/Loopädin, Psychologe/Psychologin, Sozialarbeiter/Sozialarbeiterin, Krankenpflegekraft und Arzt/Ärztin besteht. Der Einsatz eines multidisziplinären Teams kann zu einer Verbesserung der Lebensqualität führen.

Geltungsbereich und Zweck

Zielsetzung und Fragestellung

Ziel ist eine Optimierung der klinischen Versorgung von Pat. mit der Parkinson-Krankheit hinsichtlich Diagnostik, medikamentöser und operativer Behandlung.

Versorgungsbereich

Ambulant und stationär; Früherkennung, Diagnostik, Therapie; primärärztliche und spezialärztliche Versorgung

Patienten-/Patientinnenzielgruppe

Erwachsene Personen mit der Parkinson-Krankheit

Adressaten

Die Leitlinie richtet sich an Neurologen/Neurologinnen, Neurochirurgen/Neurochirurginnen, Neurophysiologen/Neurophysiologinnen, Neuropsychologen/Neuropsychologinnen, Psychologen/Psychologinnen, Psychiater/Psychiaterinnen, Humangenetiker/Humangenetikerinnen, Psycho-, Ergo- und Physiotherapeuten/innen, Pflegekräfte, Logopäden/Logopädinnen, Sozialarbeiter/Sozialarbeiterinnen, Patienten/Patientinnen und dient zur Information für Angehörige, Allgemeinärzte/Allgemeinärztinnen, Internisten/Internistinnen, Künstlerische Therapeuten/Therapeutinnen, Neuroradiologen/Neuroradiologinnen sowie für Leistungserbringer (Krankenkassen, Rentenversicherungsträger etc.).

Schlüsselwörter

Parkinson-Krankheit, Parkinson-Syndrom, Diagnose, Therapie, Versorgungskonzepte

Inhalt

1 Parkinson-Krankheit – Begriffsdefinitionen ... 24

2 Diagnose ... 31

2.1 Klinische Diagnosekriterien ... 31
2.2 Zerebrale MRT- und CT-Bildgebung ... 56
2.3 Hirnparenchymsonographie ... 61
2.4 Nuklearmedizinische Bildgebung ... 72
2.5 Molekulare Biomarker ... 79
2.6 Digitale Biomarker ... 81
2.7 Genetische Diagnostik ... 87

3 Therapie ... 105

3.1 Parkinson-Medikamente ... 105
3.2 Initiale Monotherapie ... 117
3.3 Frühe Kombinationstherapie ... 143
3.4 Fluktuationen ... 156
3.5 Dyskinesien ... 188
3.6 Parkinson-Tremor ... 193
3.7 Akinetische Krise/Entzugssyndrome ... 206
3.8 Spezielle Behandlungsfragen ... 224
3.9 Schmerz ... 241
3.10 Dysautonomie ... 249
3.11 Schlaf und Tagesmüdigkeit ... 277
3.12 Kognition ... 305
3.13 Affektive Störungen ... 361
3.14 Impulskontrollstörungen ... 400
3.15 Psychose ... 415
3.16 Delir ... 425
3.17 Psychosoziale Beratung ... 431
3.18 Dysarthrie und Dysphagie ... 434
3.19 Pumpentherapien ... 442
3.20 Tiefe Hirnstimulation ... 458
3.21 Ablative Therapien ... 482
3.22 Differenzialindikation invasiver Therapien ... 490
3.23 Aktivierende Therapien ... 509
3.24 Krankheitsmodifizierende Therapien ... 519
3.25 Fahrtauglichkeit ... 531
3.26 Parkinson-Tagesklinik ... 536
3.27 Stationäre multidisziplinäre Therapie ... 550
3.28 Versorgungsnetzwerke ... 553
3.29 Parkinson-Pflege ... 554
3.30 Palliativversorgung ... 557

Abkürzungsverzeichnis ... 565

1 Parkinson-Krankheit – Begriffsdefinitionen

Autoren/Autorinnen: Tiago Outeiro, Matthias Höllerhage, Christine Klein, Gregor Kuhlenbäumer, Viktoria Ruf, Manuela Neumann, Günter Höglinger

Die folgenden Definitionen sollen helfen, die Nosologie und die Krankheitsentitäten zu definieren, die in dieser Leitlinie behandelt werden.

Parkinson-Syndrom

Ein Parkinson-Syndrom ist klinisch definiert durch das Vorhandensein von Bradykinesie als Leitsymptom sowie von einem oder mehreren der Merkmale Rigor, Tremor und posturale Instabilität.

- **Bradykinese:** Bradykinese ist eine Verlangsamung der Einleitung und Ausführung spontaner und willkürlicher Bewegungen, die durch Fingerklopfen, Handbewegungen, Pronations-Supinationsbewegungen, Klopfen mit den Zehen und Klopfen mit den Füßen beurteilt werden können [1]. Bei der Parkinson-Krankheit (PK, Morbus Parkinson) ist die Bradykinese typischerweise mit einer Abnahme der Amplitude oder Geschwindigkeit bei fortgesetzten Bewegungen verbunden [1], während bei atypischen Parkinson-Syndromen die Abnahme fehlen kann [2].
- **Rigor:** Rigor ist ein geschwindigkeitsunabhängiger Widerstand, der bei passiven Bewegungen in den großen Gelenken beobachtet wird. Ein Zahnradphänomen kann vorhanden sein. Ein Zahnradphänomen ohne Widerstand wird nicht als Rigor angesehen [1].
- **Tremor:** Tremor ist eine unwillkürliche rhythmische Bewegung eines oder mehrerer Körperteile. Der Ruhetremor, definiert als ein 4–6-Hz-Tremor, der bei völlig entspannten Gliedmaßen auftritt und bei Bewegungen unterdrückt wird, ist charakteristisch, aber nicht zwingend erforderlich für die Diagnose eines Parkinson-Syndroms [1]. Bei atypischen Parkinson-Syndromen können auch andere Formen des Tremors auftreten.
- **Posturale Instabilität:** Posturale Instabilität (Haltungsinstabilität) ist eine Sturzneigung, die nicht durch eine primäre visuelle, vestibuläre, zerebelläre oder propriozeptive Dysfunktion verursacht wird [3]. Während sie bei atypischen Parkinson-Syndromen häufig und früh beobachtet wird, tritt die posturale Instabilität bei der PK erst spät auf und wird daher nach den aktuellen Diagnosekriterien nicht mehr als motorisches Kardinalsymptom der PK angesehen [1].

Symptomatisches (sekundäres) Parkinson-Syndrom

Ein Parkinson-Syndrom, das durch eine identifizierbare, nicht genetische Ursache verursacht wird, wird als symptomatisches oder sekundäres Parkinson-Syndrom bezeichnet. Die Hauptursachen für symptomatische Parkinson-Syndrome sind:

- **Hydrozephalus** (z. B. Normaldruckhydrozephalus)

- **Medikamente** (z. B. Antidopaminergika, Metoclopramid ...) (d. h. das medikamentös induzierte Parkinson-Syndrom)
- **metabolische Ursachen** (z. B. Morbus Wilson)
- **zerebrovaskuläre Läsionen** (z. B. strategische Infarkte oder chronische vaskuläre Enzephalopathie) (d. h. vaskuläres Parkinson-Syndrom)
- **neoplastische Läsionen** (z. B. mit der Mittellinie assoziierte Tumore)
- **enzephalitische/post-enzephalitische Läsionen** (z. B. Enzephalitis lethargica, HIV-Enzephalitis)
- **toxische Läsionen** (z. B. durch Kohlenmonoxidvergiftung, Schwefelkohlenstoffvergiftung, Cyanidvergiftung, Manganismus, Methanolvergiftung, Vergiftungen durch MPTP, MDMA etc.)
- **traumatisch bedingte Läsionen** (nach repetitiven Schädel-Hirn-Traumata) (d. h. chronisch-traumatische Enzephalopathie (CTE))

Hinweis: Die bei Normaldruckhydrozephalus oder chronischer vaskulärer Enzephalopathie beobachtete Gangstörung wird häufig als Parkinson-Syndrom der unteren Körperhälfte bezeichnet. Bei diesen Erkrankungen kann jedoch manchmal eine Gangapraxie auftreten, die durch eine Fehlfunktion des Frontalhirns verursacht werden. Diese sind daher eher als Differenzialdiagnose denn als Parkinson-Syndrom im engeren Sinne zu betrachten.

Hereditäres Parkinson-Syndrom

Hereditäre Parkinson-Syndrome werden durch seltene pathogene genetische Varianten („Mutationen") in einem Gen („monogen") verursacht. Pathogene Varianten in rezessiven Genen (z.B. *PRKN*) weisen dabei eine hohe Penetranz auf, d.h., fast alle Träger von Mutationen erkranken; bei Trägern von pathogenen Varianten in dominanten Genen ist die Penetranz zumeist (altersabhängig) reduziert (z. B. *LRRK2*), d.h., ein erheblicher Teil der Variantenträger bleibt zeitlebens gesund oder erkrankt spät. Gene wurden sowohl für klassische Parkinson-Syndrome, die klinisch der PK ähneln, als auch für atypische Parkinson-Syndrome oder Dystonien mit Parkinson-Syndrom identifiziert. Gegenstand dieser Leitlinie sind ausschließlich Gene, die mit einem klinisch klassischen Parkinson-Syndrom assoziiert sind, sodass in diesem Kontext von einer **hereditären PK** gesprochen werden kann. Hinsichtlich der Nomenklatur für erbliche Parkinson-Syndrome schlagen wir vor, den Empfehlungen der International Parkinson and Movement Disorder Society Task Force zu folgen (z. B. PARK-*SNCA*, ...; *www.mdsgene.org/g4d*).

Die sporadische PK ist genetisch komplex, d.h., Varianten in zahlreichen Genen tragen einzeln jeweils schwach zum Erkrankungsrisiko bei („polygen").

Eine Sonderstellung nehmen heterozygote Mutationen im Gen der Glukozerebrosidase (*GBA1*, PARK-*GBA1*) ein, da diese abhängig von der jeweiligen Variante das ganze Spektrum von schwachen bis hin zu starken Risikofaktoren abdecken [4]. Einige stark pathogene Varianten bedingen ein hohes Krankheitsrisiko, das dem schwach pathogener Varianten in Genen ähnelt, die zu den monogenen Ursachen eines Parkinson-Syndroms gezählt werden. In dieser Leitlinie wird *GBA1* zu den (starken) Risikofaktoren gezählt.

Nicht vererbbare Formen der PK, werden als „sporadische PK“ bezeichnet. Für sporadische Parkinson-Syndrome konnte mithilfe genomweiter Assoziationsstudien eine große Zahl gemeinsamer genetischer Varianten (SNPs, aus dem Englischen: single nucleotide polymorphisms; Einzelnukleotid-Polymorphismen) identifiziert werden, wobei jede einzelne Variante an sich nur geringfügig zum Risiko der Entwicklung eines Parkinson-Syndroms beiträgt. Die Vererbung einzelner Risikovarianten folgt nicht den Mendelschen Regeln. Polygene Risikoscores fassen das Risiko mehrerer Einzelnukleotid-Polymorphismen zusammen und können einen höheren Anteil des Risikos erklären. Die stetig anwachsende Menge und Verfügbarkeit molekularbiologischer Daten wird zu einem zunehmend besseren Verständnis der genetischen Komplexität von Parkinson-Syndromen führen und Möglichkeiten für zusätzliche Subklassifikationen und schließlich für personalisierte Therapien eröffnen.

Idiopathisches Parkinson-Syndrom

Die Begriffe „idiopathisches Parkinson-Syndrom“ (IPS) und „Parkinson-Krankheit (PK)“ wurden in der Vergangenheit häufig synonym verwendet. In den letzten Jahren wurde jedoch deutlich, dass einige Fälle der PK durch genetische Varianten verursacht werden (z. B. heterozygote *GBA1*-Mutationen, autosomal-dominante *LRRK2*-Mutationen; Einzelheiten siehe www.mdsgene.org/g4d). Daher ist für Fälle ohne identifizierbare Ursache in der internationalen Literatur aktuell der Begriff „idiopathische PK“ vorgesehen, während Fälle, die mit seltenen oder wenig häufigen pathogenen genetischen Varianten assoziiert sind, als „hereditäre (erbliche) Parkinson-Syndrome“ bezeichnet werden, auch wenn die Erblichkeit in der Familienanamnese nicht offensichtlich ist (z. B. aufgrund kleiner Familien, De-novo-Mutationen oder geringer Penetranz). Daher sollte der Begriff „Parkinson-Krankheit“ nicht synonym mit dem Begriff „idiopathisches Parkinson-Syndrom“ gebraucht werden. Mit zunehmendem Wissen und besserem Verständnis der pathophysiologischen Prozesse, die den Parkinson-Syndromen zugrunde liegen, wird der Begriff „idiopathisch“ womöglich künftig obsolet werden.

Parkinson-Krankheit

Die sporadische Parkinson-Krankheit (PK) ist eine Lewy-Körperchen-Krankheit, die zu einem Parkinson-Syndrom führt. Die hereditäre PK geht oft, aber nicht immer mit einer Lewy-Körperchen-Pathologie einher. Der Begriff PK kann jedoch sowohl bei Vorliegen eines klinisch manifesten Parkinson-Syndroms verwendet werden als auch im Rahmen entsprechender Symptomkonstellationen, die auf die künftige Entwicklung eines Parkinson-Syndroms hindeuten, z. B. bei prämotorischer oder prodromaler PK. In der überwiegenden Mehrzahl der Fälle handelt es sich um eine nicht vererbbare PK, die als „sporadische PK“ bezeichnet wird. In wenigen Fällen lassen sich jedoch Mutationen (z. B. in den Genen *SNCA*, *LRRK2*, *GBA1*) nachweisen, die dann als „erbliche Parkinson-Syndrome“ eingeordnet werden. Parkinson-Syndrome, die mit Mutationen in anderen Genen (z. B. *VPS35, DJ1, PARKIN, PINK1*) assoziiert sind, weisen in der Regel nicht die typischen histopathologischen Merkmale einer Lewy-Körperchen-Krankheit auf. Sie sollten daher nicht als PK bezeichnet werden, sondern als „erbliches Parkinson-Syndrom“ mit der von der Task Force der International Parkinson and Movement Disorder Society empfohlenen spezifischen Nomenklatur (z.B. PARK-*VPS35*).

Präklinische PK

Die präklinische PK ist definiert als Lewy-Körperchen-Krankheit in einem sehr frühen Stadium, die noch keine offensichtlichen motorischen oder nicht motorischen klinischen Anzeichen aufweist.

Prämotorische PK

Als prämotorische PK gilt eine Lewy-Körperchen-Krankheit in einem frühen Stadium, die zum Zeitpunkt der Untersuchung nur nicht motorische Symptome aufweist, z. B. REM-Schlaf-Verhaltensstörungen, Geruchsstörungen und/oder Depressionen.

Prodromale PK

Als prodromale PK wird eine Lewy-Körperchen-Krankheit im Frühstadium bezeichnet, die zum Zeitpunkt der Untersuchung nicht motorische Symptome und nur leichte motorische Symptome aufweist, jedoch noch nicht die Kriterien für die Diagnose eines klassischen Parkinson-Syndroms und damit der PK erfüllt.

PK mit Demenz

Die PK mit Demenz (PKD) ist definiert als eine Lewy-Körperchen-Krankheit im fortgeschrittenen Stadium, die mit einer neurokognitiven Störung einhergeht, die mehr als ein Jahr nach Manifestation der motorischen Symptome der PK auftritt.

Demenz mit Lewy-Körperchen

Die Demenz mit Lewy-Körperchen (DLK) ist definiert als eine Lewy-Körperchen-Krankheit, die sich primär als neurokognitive Störung darstellt, deren Ausbruch dem Auftreten der motorischen Symptome der PK vorausgeht oder innerhalb von weniger als einem Jahr folgt. Bei der neuropathologischen Untersuchung werden bei der DLK typischerweise höhere Stadien Alzheimer-assoziierter Veränderungen (Amyloid-Plaques und neurofibrilläre Tangles) beobachtet als bei einer reinen PKD [5].

Lewy-Körperchen-Demenz

Der Begriff „Lewy-Körperchen-Demenz“ bezieht sich auf alle Arten von Demenz, die mit der Lewy-Körperchen-Krankheit assoziiert sind (d. h. ein Oberbegriff für PKD und DLK).

Lewy-Körperchen-Krankheit

Die Lewy-Körperchen-Krankheit (LKK) ist ein Oberbegriff für klinisch heterogene neurodegenerative Erkrankungen (d. h. PK, einschließlich präklinischer, prodromaler und prämotorischer PK, PKD und DLK), die durch Alpha-Synuklein (aSyn)-immunreaktive Lewy-Körperchen und Lewy-Neuriten in bestimmten kortikalen und subkortikalen Hirnregionen definiert sind [6]. Den Goldstandard, um eine LKK eindeutig zu diagnostizieren, stellt gegenwärtig die (postmortale) neuropathologische Untersuchung des zentralen Nervensystems dar. Zwar sind für die verschiedenen klinischen Entitäten der LKK jeweils bestimmte Charakteristika (z. B. hinsichtlich regionaler Verteilung und Ausprägung von Neurodegeneration und Lewy-Körper-Pathologie) beschrieben, da die Übergänge zwischen den Entitäten jedoch fließend sind und mitunter beträchtliche Überlappungen bestehen, ist eine

Zuordnung zu einer dieser Entitäten im individuellen Fall nur in einem klinisch-pathologischen Kontext möglich [7].

In einigen Fällen kann die Diagnose von LKK genetisch gestellt werden, indem eine pathogene seltene oder seltene genetische Variante in *SNCA*, *LRRK2* oder *GBA1* nachgewiesen wird, die eindeutig mit der LKK-Pathologie assoziiert ist.

Atypisches Parkinson-Syndrom

Atypische Parkinson-Syndrome sind definiert als eine Gruppe von Parkinson-Syndromen, die durch eine andere neurodegenerative Krankheit als die Lewy-Körperchen-Krankheit verursacht werden (d. h. ein Oberbegriff für Krankheiten wie Multisystematrophie (MSA), kortikobasale Degeneration (CBD) und progressive supranukleäre Blickparese (PSP)).

Entwicklung von Biomarkern zur Unterstützung der Diagnose von Parkinson-Syndromen

Derzeit befinden sich verschiedenartige Biomarker in der Entwicklung, die eine frühzeitige, zuverlässige und präzise Diagnosestellung bei Parkinson-Syndromen am lebenden Pat. ermöglichen sollen. Hierbei stellen insbesondere PET-Tracer zur bildgebenden Darstellung von Alpha-Synuklein-Aggregaten im menschlichen Gehirn sowie sog. Seeding-Assays (z. B. der RT-QuIC-Assay), mithilfe derer pathologische aSyn-Aggregate in biologischen Flüssigkeiten wie Blut oder Liquor – möglicherweise auch spezifisch für bestimmte Krankheitsentitäten (z. B. LKK vs. Multisystematrophie) – nachgewiesen werden können, attraktive und vielversprechende Verfahren dar. Bevor solche Biomarker als diagnostischer Standard in der Routinediagnostik bei Parkinson-Syndromen eingesetzt werden können, sind jedoch noch weiterführende Forschungsarbeiten und Studien erforderlich.

Tab.1: Lexikon

Begriff	Definition	Referenz
Parkinson-Syndrom	Bradykinesie (d. h. Verlangsamung der spontanen und willkürlichen Bewegungen) plus eines oder mehrere der folgenden Merkmale:<br>▪ Rigor<br>▪ Tremor<br>▪ posturale Instabilität (Haltungsinstabilität), die nicht durch eine primäre visuelle, vestibuläre, zerebelläre oder propriozeptive Dysfunktion verursacht wird	[8], [1], [9]
Symptomatisches (sekundäres) Parkinson-Syndrom	Parkinson-Syndrom mit identifizierbarer Ursache (z. B. Normaldruckhydrozephalus, medikamenteninduziert, metabolisch, zerebrovaskulär, neoplastisch, enzephalitisch, toxisch, traumatisch usw.)	[10]

Hereditäres Parkinson-Syndrom	Parkinson-Syndrom, das durch seltene oder wenig häufige pathogene genetische Varianten („Mutationen“) verursacht wird; im Hinblick auf die Nomenklatur für erbliche Parkinson-Syndrome schlagen wir vor, den Empfehlungen der International Parkinson and Movement Disorder Society Task Force zu folgen (z. B. PARK-*SNCA*, PARK-*GBA1* usw.)	[10], [11], [12]
Idiopathisches Parkinson-Syndrom (IPS)	Parkinson-Syndrom mit unbekannter Ursache, d. h. nicht symptomatisch, nicht erblich (der Terminus ist daher im strengen Sinne nicht identisch mit der PK)	[10]
Parkinson-Krankheit (PK)	Lewy-Körperchen-Krankheit, die zu einem Parkinson-Syndrom führt (der Begriff kann bei Vorliegen eines klinisch manifesten Parkinson-Syndroms verwendet werden, aber auch bei Bedingungen, die auf die künftige Entwicklung eines Parkinson-Syndroms hindeuten, z. B. bei prämotorischer oder prodromaler PK.)	[8], [1], [9]
Präklinische PK	Lewy-Körperchen-Krankheit in einem sehr frühen Stadium, die noch keine offensichtlichen klinischen Symptome aufweist	[13]
Prämotorische PK	Lewy-Körperchen-Krankheit im Frühstadium, die nur nicht motorische Symptome zeigt, z. B. REM-Schlaf-Verhaltensstörung	[13]
Prodromale PK	Lewy-Körperchen-Krankheit im Frühstadium, die zwar mit nicht motorischen Symptomen und leichten motorischen Anzeichen einhergeht, jedoch noch nicht die Kriterien für die Diagnose eines klassischen Parkinson-Syndroms und somit der PK erfüllt	[14], [15]
PK mit Demenz (PKD)	Lewy-Körperchen-Krankheit im fortgeschrittenen Stadium, die mit einer neurokognitiven Störung einhergeht, die sich frühestens ein Jahr nach dem Auftreten der motorischen Symptome manifestiert	[16]
Lewy-Körperchen-Demenzen (LKD)	Neurokognitive Störung, die durch eine Lewy-Körper-Krankheit verursacht wird (d. h. ein Oberbegriff für PKD und DLK)	[16]
Lewy-Körperchen-Krankheit (LKK)	Krankheit, die durch das Vorhandensein von Alpha-Synuklein-immunreaktiven Lewy-Körpern und Lewy-Neuriten in bestimmten kortikalen und subkortikalen Hirnregionen	[6], [7]

	definiert ist (d. h. ein Oberbegriff für PK, PKD und DLK)	
Atypisches Parkinson-Syndrom	Parkinson-Syndrom, das durch eine andere neurodegenerative Krankheit als die Lewy-Körperchen-Krankheit verursacht wird (d. h. ein Oberbegriff für Krankheiten wie Multisystematrophie (MSA), kortikobasales Syndrom (CBD) und progressive supranukleäre Blickparese (PSP))	[10], [17]

Referenzen

1. Postuma RB, Berg D, Stern M, Poewe W, Olanow CW, Oertel W, et al. MDS clinical diagnostic criteria for Parkinson's disease. Mov Disord. 2015;30(12):1591-601
2. Ling H, Massey LA, Lees AJ, Brown P, Day BL. Hypokinesia without decrement distinguishes progressive supranuclear palsy from Parkinson's disease. Brain. 2012;135(Pt 4):1141-53
3. Gibb WR, Lees AJ. The relevance of the Lewy body to the pathogenesis of idiopathic Parkinson's disease. J Neurol Neurosurg Psychiatry. 1988;51(6):745-52
4. Sidransky E, Nalls MA, Aasly JO, Aharon-Peretz J, Annesi G, Barbosa ER, et al. Multicenter analysis of glucocerebrosidase mutations in Parkinson's disease. N Engl J Med. 2009;361(17):1651-61
5. Irwin DJ, Grossman M, Weintraub D, Hurtig HI, Duda JE, Xie SX, et al. Neuropathological and genetic correlates of survival and dementia onset in synucleinopathies: a retrospective analysis. Lancet Neurol. 2017;16(1):55-65
6. Attems J, Toledo JB, Walker L, Gelpi E, Gentleman S, Halliday G, et al. Neuropathological consensus criteria for the evaluation of Lewy pathology in post-mortem brains: a multi-centre study. Acta Neuropathol. 2021;141(2):159-72
7. Lippa CF, Duda JE, Grossman M, Hurtig HI, Aarsland D, Boeve BF, et al. DLB and PDD boundary issues: diagnosis, treatment, molecular pathology, and biomarkers. Neurology. 2007;68(11):812-9
8. Gibb WR, Lees AJ. The significance of the Lewy body in the diagnosis of idiopathic Parkinson's disease. Neuropathol Appl Neurobiol. 1989;15(1):27-44
9. Kalia LV, Lang AE. Parkinson's disease. Lancet. 2015;386(9996):896-912
10. Bloem BR, Okun MS, Klein C. Parkinson's disease. Lancet. 2021;397(10291):2284-303
11. Marras C, Lang A, van de Warrenburg BP, Sue CM, Tabrizi SJ, Bertram L, et al. Nomenclature of genetic movement disorders: Recommendations of the international Parkinson and movement disorder society task force. Mov Disord. 2016;31(4):436-57
12. Marras C, Lang A, van de Warrenburg BP, Sue CM, Tabrizi SJ, Bertram L, et al. Nomenclature of genetic movement disorders: Recommendations of the International Parkinson and Movement Disorder Society task force. Mov Disord. 2017;32(5):724-5
13. Postuma RB, Berg D. Advances in markers of prodromal Parkinson disease. Nat Rev Neurol. 2016;12(11):622-34
14. Berg D, Postuma RB, Adler CH, Bloem BR, Chan P, Dubois B, et al. MDS research criteria for prodromal Parkinson's disease. Mov Disord. 2015;30(12):1600-11
15. Heinzel S, Berg D, Gasser T, Chen H, Yao C, Postuma RB. Update of the MDS research criteria for prodromal Parkinson's disease. Mov Disord. 2019;34(10):1464-70
16. Walker Z, Possin KL, Boeve BF, Aarsland D. Lewy body dementias. Lancet. 2015;386(10004):1683-97
17. Jankovic J. Parkinsonism-plus syndromes. Mov Disord. 1989;4 Suppl 1:S95-119

2 Diagnose

2.1 Klinische Diagnosekriterien

Autoren/Autorinnen: Kirsten Zeuner, Wiebke Hermann, Daniela Berg

Fragestellung 1: Wie effektiv ist die Diagnose der PK durch einen klinischen Experten für Bewegungsstörungen unter Verwendung der UK-Parkinson's-Disease-Society-Brain Bank-Kriterien im Vergleich mit der Post-mortem-Diagnose?

Hintergrund

Die PK muss in der Versorgungsmedizin möglichst genau von sekundären Parkinson-Syndromen und Parkinson-Syndromen bei anderen neurodegenerativen Erkrankungen klinisch differenziert werden. Eine klinische Diagnose der PK sollte auf anerkannten Kriterien beruhen.

Evidenzgrundlage

Die Literatursuche in Pubmed erbrachte 70 Studien zu den Begriffen „Parkinson disease AND UK Brain Bank" und „Parkinson's disease diagnostic criteria AND post mortem" (27 Studien). Nach Screening der Abstracts und Inhalte wurden keine neuen Studien aus den Literaturreferenzen im Vergleich zu den Leitlinien von 2016 extrahiert.

Ergebnis

Die UK-Parkinson's-Disease-Society-Brain-Bank-Kriterien werden nach wie vor häufig im klinischen und wissenschaftlichen Alltag angewendet. Die UK Parkinson's Disease Society Brain Bank-Kriterien wurden neuropathologisch validiert und deshalb weltweit akzeptiert.

Drei von fünf Studien zur Validierung der klinisch gestellten Diagnose anhand der UK-Parkinson's-Disease-Society-Brain Bank-Kriterien bei Parkinson-Syndromen in verschiedenen Krankheitsstadien im Vergleich zu histopathologischen Post-mortem-Ergebnissen sind veröffentlicht worden.

Durch eine retrospektive Anwendung [1] der UK-Parkinson's-Disease-Society-Brain-Bank-Kriterien aus dem Jahr 1991 verbesserte sich in einer Studie mit 100 post mortem histopathologisch bestätigten Pat. die diagnostische Genauigkeit von 70% auf 82% bei den mit einer Parkinson-Erkrankung eingeordneten Fällen. Die Diagnosen in den 24 Pat. ohne Nachweis von Lewy-Körpern lauteten progressive supranukleäre Blicklähmung (n=6), Multisystematrophie (n=5), neuropathologische Befunde, vereinbar mit Alzheimer-Demenz (n=3) bzw. Alzheimer-typischen Veränderungen (n=3), vaskulär, lakunäre Infarkte (n=3), vaskuläre Atrophie ohne Lewy-Körper (n=2), postenzephalitisches Parkinson-Syndrom (n=1) und essenzieller Tremor ohne neuropathologische Auffälligkeiten (n=1).

Hingegen verbesserte die prospektive Anwendung der UK Parkinson's Disease Society Brain Bank-Kriterien mit 100 post mortem gesicherten Parkinson-Pat. [2] aus dem Jahr 2001 die diagnostische Genauigkeit auf 90% (Zunahme um 14%; 95%CI 3,8–24,2, p=0,008). Die 10 Fehldiagnosen waren

Multisystematrophie (n=6), progressive supranukleäre Blicklähmung (n=2), wahrscheinliches postenzephalitisches Parkinson-Syndrom (n=1) und vaskuläres Parkinson-Syndrom (n=1).

In der bisher letzten Arbeit der Gruppe zur Erstellung der UK-Parkinson's-Disease-Society-Brain-Bank-Kriterien aus dem Jahr 2002 [3], die die UK-Parkinson's-Disease-Society-Brain-Bank-Kriterien etabliert hatte, wurden 143 Pat. post mortem neuropathologisch untersucht, die zu Lebzeiten mit PK (n=73) und anderen Parkinson-Syndromen (n=70) diagnostiziert worden waren. Alle Pat. waren entsprechend der DGN-S3-Leitlinie zum idiopathischen Parkinson-Syndrom von 11 Neurologen untersucht worden, 92% der Pat. sogar von fünf Parkinson-Spezialisten. Die klinische Diagnose wurde bei 44 von 122 Pat. im weiteren Verlauf nach im Mittel 3,4 (0,5–12) Jahren revidiert. Die Sensitivität für die richtige klinische Diagnose lag unter Berücksichtigung der im Verlauf revidierten Diagnosen bei 91%, die Spezifität bei 98% und der positive prädiktive Voraussagewert bei 99% (72 von 73 Pat. waren korrekt als PK diagnostiziert worden).

Begründung der Empfehlung

Die UK-Parkinson's-Disease-Society-Brain-Bank-Kriterien sind die einzigen neuropathologisch validierten Kriterien zur Diagnosestellung einer PK. Da der klinische Verlauf bei diesen Kriterien berücksichtigt werden muss, führte ihre Anwendung dazu, dass häufig Diagnosen im Verlauf revidiert werden mussten, somit sind die Kriterien für inzidentelle Fälle unzureichend.

**Empfehlung**	**Modifiziert**<br>**Stand (2023)**
Es kann erwogen werden, die Diagnose einer PK anhand der UK-Parkinson's-Disease-Society-Brain-Bank-Kriterien zu stellen. Dabei muss jedoch berücksichtigt werden, dass die Kriterien aufgrund der für die Diagnosestellung notwendigen Einbeziehung des klinischen Verlaufs Einschränkungen der diagnostischen Sicherheit aufweisen und andere Kriterien (s.u.) als Alternative zur Verfügung stehen.	
Konsensstärke: 97,4%, starker Konsens	

Fragestellung 2: Wie effektiv ist die Diagnose der PK durch einen klinischen Experten für Bewegungsstörungen unter Verwendung der UK-Parkinson's-Disease-Society-Brain-Bank-Kriterien im Vergleich zu dem langfristigen klinischen Follow-up?

Hintergrund

Die diagnostischen Kriterien stützen sich bislang auf die UK-Parkinson's-Disease-Society-Brain-Bank-Kriterien. Sie sind die einzigen neuropathologisch validierten diagnostischen Kriterien für die Diagnose einer PK. Zu den diagnostischen Kriterien zählen eine Bradykinesie, ein Ruhetremor, eine asymmetrische Ausprägung der Symptomatik und eine Krankheitsprogression. Als supportive diagnostische Parameter werden ein Ansprechen auf Levodopa und eine Langzeitbeobachtung über mindestens fünf Jahre, motorische Fluktuationen und Dyskinesien gezählt.

Evidenzgrundlage

Für dieses Kapitel: In dieser Literaturrecherche fand sich eine longitudinale größere Kohortenstudie.

Ergebnis

In einer longitudinalen Studie aus Arizona wurden 232 Pat. eingeschlossen, von denen 131 Pat. eine mögliche oder wahrscheinliche PK hatten. Diese Pat. wurden jährlich evaluiert, dabei erfolgten eine klinische Untersuchung mittels UPDRS III und die Erfassung der Medikation. Pat., die ein gutes Ansprechen auf Levodopa zeigten, zusätzlich mindestens zwei klassische klinische Zeichen hatten (z.B. Tremor, Bradykinese) und keine symptomatischen Ursachen aufwiesen, wurden als Pat. mit wahrscheinlicher PK definiert. Nach ihrem Tod erfolgte eine neuropathologische Untersuchung. Hier zeigte sich, dass die Diagnose neuropathologisch bei 89 Pat. bestätigt und bei 42 Pat. nicht bestätigt werden konnte. Dabei spielte die Verlaufsbeobachtung eine besondere Rolle: Pat. mit klassischen Symptomen und einem Ansprechen auf die Levodopa-Therapie, aber einer Krankheitsdauer von < 5 Jahren zeigten einen positiven prädiktiven Wert von nur 53 %, während dieser Wert bei einem Verlauf von > 5 Jahren auf 88 % anstieg. Sobald motorische Fluktuation oder Dyskinesien auftraten, stieg der positive prädiktive Wert noch weiter auf 92 % bzw. 96 % an.

Begründung der Empfehlung

Die Studie von Adler et al. [4] ist eine Klasse-2-Evidenz-Studie. Nach dieser Studie ist die Beobachtung des Langzeitverlaufs, einer rein klinischen Diagnose nach den UK-Parkinson's-Disease-Society-Brain-Bank-Kriterien durch Experten/Expertinnen, selbst wenn eine positive Antwort auf dopaminerge Therapie mitberücksichtigt wird, überlegen. Je länger dieser Langzeitverlauf ist, desto genauer kann die Diagnose einer PK gestellt werden.

Empfehlung	Modifiziert<br>Stand (2023)
▪ Die Beobachtung des Krankheitsverlaufs über mehr als fünf Jahre verbessert die Wahrscheinlichkeit, eine richtige Diagnose zu stellen, von 53 % auf > 85 % und ist somit der Anwendung der UK-Parkinson's-Disease-Society-Brain-Bank-Kriterien allein überlegen, sodass Parkinson-Pat. immer über einen Langzeitverlauf reevaluiert werden sollten.<br>▪ Dabei sollte klinisch/anamnestisch v.a. das Auftreten von motorischen Fluktuationen und Dyskinesien berücksichtigt werden, da diese Symptome die Wahrscheinlichkeit, eine richtige Diagnose stellen zu können, nochmals deutlich verbessern, und somit im Langzeitverlauf ein wichtiges klinisches Merkmal darstellen.	
Konsensstärke: 100%, starker Konsens	

Fragestellung 3: Wie hoch sind Spezifizität und Sensitivität der MDS-Kriterien zur klinischen Diagnose einer Parkinson-Erkrankung im Vergleich zu den Kriterien der UK Parkinson's Disease Society Brain Bank?

Hintergrund

Der Verlauf der PK wird in drei Phasen unterteilt: In der präklinischen Phase hat der neurodegenerative Prozess bereits begonnen, jedoch liegen noch keine spezifischen Symptome vor. In der prodromalen Phase liegen bereits erste Symptome vor, die im Frühstadium der Parkinson-Erkrankung auftreten und somit Hinweise auf den klinischen Beginn einer PK sein können. Sie sind

jedoch unzureichend, um eine PK mit Sicherheit zu diagnostizieren. Die dritte, klinische Phase geht mit den typischen motorischen Symptomen der Parkinson-Erkrankung einher. Es existieren deshalb seit 2015 neue klinische Kriterien [5] der Movement Disorder Society, die in 2018 validiert wurden [6]. Ziel der neuen MDS-Kriterien zur Diagnose der PK und der Validierungsstudie war, die Genauigkeit der Diagnose zu verbessern.

Evidenzgrundlage

N=2 longitudinale größere Kohortenstudien, ein Review.

Ergebnis

Die Kriterien der MDS von 2015 [5] enthalten einige Besonderheiten. Zunächst wird zwischen negativen und positiven Merkmalen unterschieden. Die negativen Merkmale schließen sogenannte absolute Ausschlusskriterien und red flags ein. Zu den positiven Merkmalen zählen supportive Kriterien. Diese werden gewichtet, das heißt, wenn negative Kriterien in Form von red flags vorhanden sind, können diese durch supportive Merkmale ausgeglichen werden. Bei den absoluten Ausschlusskriterien ist dies nicht möglich. Es wurden einige Ausnahmen für die absoluten Ausschlusskriterien formuliert, um Sonderfälle zu berücksichtigen. Ferner ist die Demenz kein Ausschlusskriterium, da diese als MCI (mild cognitive impairment) schon früh im Rahmen der Parkinson-Erkrankung auftreten kann und im späteren Verlauf dann als Parkinson-Demenz klinisch evident wird. Auch das frühe Auftreten von kognitiven Einschränkungen, Fluktuationen v.a. der Vigilanz und Halluzinationen im Sinne einer Demenz vom Lewy-Körperchen-Typ (DLB) ist kein Ausschlusskriterium, da die DLB als Kontinuum im Spektrum der Parkinson-Manifestationen gewertet wird [7]. Der Zeitverlauf wird mit berücksichtigt und spielt eine besondere Rolle.

Die PK bleibt eine klinische Diagnose, aber zusätzliche apparative Diagnostik kann hinzugezogen werden und gilt als supportives Kriterium. Ferner wird erstmals auch ein nicht motorisches Symptom (Hyposmie) als supportives Kriterium mit gewertet.

Zu den klinischen Kriterien zählen Bradykinese, Rigor, Tremor. Als supportives Kriterium gilt der akute Levodopa-Test mit einem Ansprechen von > 30 % im UPDRS III. Es wird betont, dass das Ansprechen eindeutig sein muss, um als supportives Kriterium zu zählen. Weitere supportive Kriterien sind Levodopa-induzierte Dyskinesien, Ruhetremor einer Extremität, positiver Befund eines anderen apparativen diagnostischen Tests (das kann beispielsweise ein Riechtest sein, aber auch eine Metaiodobenzylguanidin-Szintigraphie (MIBG-Szintigraphie), die eine kardiale sympathische Denervierung nachweist).

Eine klinisch etablierte Diagnose (mit dem Ziel einer mindestens 90-%-Spezifität, bei etwas geringerer Sensitivität) kann nach den Kriterien dann gestellt werden, wenn mindestens zwei supportive Kriterien erfüllt sind, ohne dass red flags oder Ausschlusskriterien nachweisbar sind. Die Kriterien für eine klinisch wahrscheinliche Diagnose (Ziel mindestens 80-%-Spezifität bei mindestens 80-%-Sensitivität) sind dann erfüllt, wenn 1 red flag durch 1 supportives Kriterium bzw. 2 durch 2 ausgeglichen werden. Mehr als 2 red flags dürfen nicht vorliegen. Ebenso keine Ausschlusskriterien.

In einer Folgestudie von Postuma 2018 [6] erfolgte die Validierung dieser neuen MDS-Kriterien. Diese Studie wurde multizentrisch durchgeführt, wobei jeweils ein klinischer Experte/eine klinische Expertin für Bewegungsstörungen und ein unerfahrener Neurologe/eine unerfahrene Neurologin die klinische Einschätzung anhand der Kriterien vornahmen. Dabei wurden 434 Pat. mit einer PK und 192 mit Parkinson-Syndromen anderer Ätiologie rekrutiert und durch den Experten/die Expertin klassifiziert, während parallel durch den unerfahrenen Neurologen/die unerfahrene Neurologin anhand der neu entwickelten MDS-Kriterien eine Diagnoseeinordnung erfolgte. Die Diagnosegenauigkeit einer wahrscheinlichen PK lag insgesamt bei 92,6 %. Die Gesamtfehlerrate lag bei 7.4%. Spezifität und Sensitivität waren somit bei Anwendung der MDS-Kriterien höher als bei den bisher als Standard geltenden UK Brain Bank-Kriterien, hier betrug die Diagnosegenauigkeit 86,4%, d.h., es lag eine Fehlerrate von 13,6% vor, wobei sich die die Spezifität mit zunehmender Krankheitsdauer verbesserte.

Begründung der Empfehlung

Seit 2015 existieren die neuen, derzeit aktuellen Kriterien der Movement Disorder Society (MDS-Kriterien) zur Diagnose der PK. Als supportive Kriterien wurde der Levodopa-Test genannt ebenso wie die Levodopa-induzierten Dyskinesien, ein einseitiger Ruhetremor, ein nicht motorisches Symptom (Hyposmie) und weitere apparative Untersuchungen wie der DATScan (bei unauffälligem Befund ein Ausschlusskriterium) und die MIBG-Szintigraphie. Somit ist anhand dieser MDS-Kriterien die Diagnose einer PK klinisch mit hoher Spezifizität und Sensitivität zu stellen.

2018 erfolgte eine Validierung von Nichtexperten/Nichtexpertinnen gegen Experten/Expertinnen, die eine hohe Spezifität und Sensitivität ergab. Sensitivität und Spezifität der MDS-Kriterien waren höher als bei der Anwendung der histopathologisch validierten UK-Brain-Bank-Kriterien.

Dazu wurde in 2022 [8] die diagnostische Genauigkeit durch einen Neurologen/Geriater/eine Neurologin/eine Geriaterin anhand der derzeitigen MDS-Kriterien einerseits und in einer Subgruppe (n = 180 Pat., 67,4 %) durch Bewegungsstörungsspezialisten andererseits, beide gegenüber der neuropathologischen Diagnose bei 267 Parkinson-Pat. validiert. Basierend auf der neuropathologischen Diagnose, hatten 141 Pat. (52.8%) eine PK, während 126 Pat. (47.1%) einen Parkinsonismus aufwiesen. Innerhalb der ersten fünf Jahre nach Erkrankungsbeginn wurde unter Anwendung der MDS-Kriterien eine klinisch etablierte (n = 82, 58.2%) oder eine klinisch wahrscheinliche (n = 42, 29.7%) Diagnose einer PK gestellt und in 91.3% der nicht an Parkinson erkrankten Pat. als Nicht-Parkinson anhand der MDS-Kriterien identifiziert. Die gesamte diagnostische Genauigkeit lag für die klinisch wahrscheinliche PK bei 89.5%, mit einer Sensitivität von 87.9% und einer Spezifität von 91.3%, für die etablierte PK war sie mit einer Spezifität von 99,2% noch höher bei einer allerdings moderaten Sensitivität von 58,2%. Insgesamt fanden sich für das frühe Stadium die höchste Spezifität und Sensitivität unter Anwendung der MDS-Kriterien.

**Empfehlung**	**Neu**<br>**Stand (2023)**
▪ Zur Diagnosestellung einer PK sollen die MDS-Kriterien von 2015 angewendet werden.	

- Die Sensitivität und Spezifität der MDS-Kriterien sind denen der UK Brain Bank im klinischen Vergleich überlegen.
- Die Begleitung durch eine Expertin/einen Experten bei einem langen Krankheitsverlauf verbessert die Diagnosewahrscheinlichkeit, sodass Nachuntersuchungen in regelmäßigen Abständen erfolgen sollten.

Konsensstärke: 94,7%, Konsens

Wichtige Forschungsfragen:

Die MDS-Kriterien zur Diagnose der PK sollten neuropathologisch validiert werden.

Fragestellung 4: Wie effektiv ist der akute Levodopa-Test bzw. ein Apomorphin-Test im Vergleich mit dem langfristigen klinischen Follow-up für die Diagnose der Parkinson-Erkrankung?

Hintergrund

Viele Pat. mit einer PK zeigen eine klinische Besserung durch eine einzelne Dosis oralen Levodopas und/oder subkutanen Apomorphins.

Die Durchführung des Tests startet meistens mit Vorbehandlung mit Domperidon über >24 Stunden. Eine standardisierte Dosis Levodopa (150–250 mg) wird nach einigen Stunden (z.B. 12 Stunden) Medikamentenpause gegeben. Alternativ kann die Injektion von Apomorphin angewendet werden (z.B. in einer Dosierung mit schrittweiser Erhöhung, beginnend mit 1,5 mg, dann 3 mg und 4,5 mg). Als Messparameter für den Effekt kann zum Beispiel der Motorik-Teil der Unified Parkinson's Disease Rating Scale (UPDRS), d.h. der UPDRS III, benutzt werden (wird vor und etwa 1 Stunde nach Levodopa-Gabe bzw. 15–20 Minuten nach Apomorphininjektion durchgeführt).

Ein systematischer Review und eine diagnostische Studie untersuchten, wie effektiv Levodopa- und Apomorphin-Tests bezüglich der Diagnosestellung bei Personen mit Parkinson-Syndrom sind.

Evidenzgrundlage

Für diese Kapitel: In dieser Literaturrecherche fanden sich:

N=1 randomisierte, kontrollierte Studie

N=1 longitudinale größere Kohortenstudien

N=3 Falldarstellungen und Reviews.

Ergebnis

Eine erste Arbeit zur Diagnosesicherung einer PK erfolgte durch Merello et al. 2000 [9]. Hier erfolgte eine verblindete Auswertung des UPDRS. Die Sensitivität und Spezifität des akuten Levodopa-Provokationstests mit 250 mg Levodopa/Carbidopa lag für die PK bei 70.9% und 81.4%, und der positive prädiktive Wert bei 88.6%.

Ein Review [10] mit insgesamt 13 eingeschlossenen Studien stellte eine Sensitivität zwischen 75% und 86% sowie eine Spezifität zwischen 85% und 87% für die Diagnose einer PK mittels Apomorphin- bzw. Levodopa-Test fest. Dabei zeigte sich, dass der Apomorphin- und Levodopa-Test einer

chronischen Therapie mit Levodopa zur Diagnosesicherung einer PK nicht überlegen war. Bei De-novo-Pat. mit einer Parkinson-Erkrankung schnitt die chronische Levodopa-Behandlung sogar besser ab.

Eine weitere Arbeit [11] verfolgte 134 Pat. mit einer PK über 3 Jahre mit dem Ziel, den prädiktiven Wert eines Levodopa- und Apomorphin-Tests bezüglich Diagnose und dopaminergem Therapieeffekt festzustellen. Eingeschlossen wurden 83 Pat., bei denen aufgrund klinischer Verlaufsuntersuchungen unter Anwendung der UK-Brain-Bank-Kriterien und/oder Autopsie die Diagnose einer PK gestellt wurde, sowie 51 Kontroll-Pat. mit atypischen oder nicht klassifizierten Parkinson-Syndromen. Die Pat. erhielten oral Levodopa/Carbidopa (250 mg) bzw. subkutan Apomorphin (1,5, 3 und 4,5 mg). Der klinische Effekt wurde anhand des UPDRS III in wiederholten Untersuchungen ermittelt. Der Effekt wurde mit dem der chronischen Levodopa-Therapie und der klinischen Diagnose verglichen. Pat., die sich um mindestens 16 % im UPDRS III nach dem Levodopa- bzw. dem Apomorphin-Test verbesserten, hatten mit hoher Wahrscheinlichkeit eine PK. Die Sensitivität lag zwischen 70% und 77%, die Spezifität zwischen 63,9% und 71,1% für die Diagnose PK mittels Apomorphin- und Levodopa-Test, wobei höhere Dosierungen im Apomorphin-Test die Sensitivität und Spezifität erhöhten. Insgesamt waren Sensitivität und Spezifität im Levodopa-Test höher als im Apomorphin-Test. Zusätzlich wurden Pat., die über drei Jahre gut auf Levodopa ansprachen, mit denen verglichen, die keine Levodopa-Wirkung aufwiesen. Hier zeigte sich, dass die Vorhersage eines positiven chronischen Ansprechens auf die Therapie am besten war bei Pat., die sich im Levodopa-Test im UPDRS III um mindestens 14,5% verbesserten. Bei Verwendung dieses Cut-offs konnte somit das positive Therapieansprechen im Langzeitverlauf (chronische Responder) durch den Levodopa-Test mit einer Sensitivität von 69.4% und einer Spezifität von 79.4% vorhergesagt werden.

In einer weiteren Studie mit 210 an Parkinson erkrankten Pat., die nach zwei Jahren nachuntersucht wurden, wurde ein positiver Levodopa-Test mit einer in der Geruchstestung (Sniffin' Sticks) nachgewiesenen Hyposmie kombiniert. Hier verbesserte sich die Sensitivität der Diagnose einer PK auf 90 % und die Spezifität auf 74 % [12].

Als mögliche Nachteile des Levodopa- bzw. Apomorphin-Tests werden in der Literatur aufgeführt: Notwendigkeit einer Vorbehandlung mit Domperidon, Nebenwirkungsrisiko, geringe Standardisierung bezüglich Testmethodik und Auswertekriterien, Zeitaufwand und Kosten. Häufige Nebenwirkungen beim Apomorphin-Test sind Schläfrigkeit, Übelkeit, Erbrechen, Hypotension und Schwitzen. Levodopa hat weniger Nebenwirkungen als Apomorphin; Übelkeit und Erbrechen kommen jedoch ebenso wie eine orthostatische Hypotonie vor.

Begründung der Empfehlung

Die Evidenz zeigt, dass der Levodopa- bzw. der Apomorphin-Test die Diagnose einer PK mit relativ guter Sensitivität und Spezifität unterstützen. Allerdings ist deren Aussagekraft einer chronischen Levodopa-Therapie über drei Jahre, kombiniert mit klinischen Verlaufskontrollen, bezüglich der Differenzierung eines etablierten PK und atypischer Parkinson-Syndrome nicht überlegen. Bei De-novo-Pat. sind der Levodopa- und der Apomorphin-Test weniger aussagekräftig in der Differenzialdiagnostik im Vergleich zur chronischen Levodopa-Therapie mit entsprechenden

klinischen Verlaufsevaluationen. Ein negativer Levodopa-Test schließt ein Ansprechen auf länger dauernde Levodopa-Behandlung nicht aus. Deshalb wurde in den Konsensus-Empfehlungen der EFNS/MDS-ES [13] ein Levodopa- oder ein Apomorphin-Test bei De-novo-Pat.-nicht empfohlen. Dies entspricht den Parkinson-Leitlinien 2016, in denen die Empfehlung lautet, dass ein Levodopa- und ein Apomorphin-Test in der Differenzialdiagnostik nicht routinemäßig eingesetzt werden sollten.

Dies bedeutet aber nicht, dass die Tests in besonderen klinischen Situationen nicht wertvoll sein können, z.B.: 1. um den Effekt einer dopaminergen Therapie oder Dosiserhöhung abzuschätzen, 2. wenn es wichtig ist, schnell eine dopaminerge Antwort zu überprüfen, und schließlich 3. wenn es wichtig ist, quantitative und objektive Informationen über den dopaminergen Effekt zu bekommen (z.B. bei Evaluation des möglichen Ansprechens auf die Tiefe Hirnstimulation oder in der klinischen Forschung). Dazu passt, dass der Levodopa-Test in den aktuellen MDS-Kriterien von 2015 [5] als supportives Kriterium eingestuft wird, solange eine deutliche Verbesserung subjektiv oder von > 30 % in der MDS-UPDRS III zu verzeichnen ist.

Empfehlung	Modifiziert<br>Stand (2023)
▪ Routinemäßig sollten der akute Levodopa- und der Apomorphin-Test zur Diagnose der PK nicht eingesetzt werden.<br>▪ Der akute Levodopa-Test stellt aber ein supportives Kriterium der MDS-Parkinson-Kriterien dar, wenn die Verbesserung deutlich und im UPDRS III > 30 % beträgt und kann damit in der Frühdiagnose der PK erwogen werden.	
Konsensstärke: 100%, starker Konsens	

Fragestellung 5: Wie effektiv ist das therapeutische Ansprechen auf eine Levodopa-Therapie im Vergleich mit dem langfristigen klinischen Follow-up für die Diagnose der PK?

Hintergrund

Die Diagnose einer PK erfolgt mithilfe der klassischen klinischen Kriterien wie Bradykinesie, Ruhetremor und einer asymmetrischen Ausprägung der Symptomatik. Dazu zählen mehrere nicht motorische Symptome wie beispielsweise eine Hyposmie. Als zusätzliche diagnostische Parameter werden ein Ansprechen auf Levodopa und eine Langzeitbeobachtung und im Verlauf motorische Fluktuationen und Dyskinesien gezählt.

Evidenzgrundlage

Für diese Kapitel fand sich in dieser Literaturrecherche:

N=1 longitudinale größere Kohortenstudien

Ergebnis

In der Arbeit von Adler et al [4] wurden Pat. unterschieden mit der Diagnose:

(1) Wahrscheinliche PK (ProbPD): Diese hatten zwei von drei Kardinalzeichen, zu denen Ruhetremor, Bradykinese und Rigor zählten. Es gab keine Hinweise für eine symptomatische

Ursache, jedoch eine Verbesserung mit dopaminerger Medikation und einem kontinuierlichen, therapeutischen Ansprechen auf die dopaminerge Therapie

(2) Mögliche PK (PossPD): Hier lagen auch zwei der drei Kardinalzeichen vor, die Symptomatik bestand ≤ 5 Jahre und die dopaminerge Therapie wurde nicht getestet oder aber die Dosierung war nicht ausreichend.

Zur Gruppe 2 zählten 34 Pat., von denen 31 niemals mit Levodopa und drei Pat. in unzureichender Dosis behandelt wurden. Von diesen Pat. hatten nur 9 eine neuropathologisch bestätigte Diagnose einer Parkinson-Erkrankung, sodass dies einem positiven prädiktiven Wert von nur 26 % entsprach. Allerdings lag die Erkrankungsdauer hier nur bei 0,7 Jahren. Dazu schreiben die Autoren/Autorinnen, dass es aufgrund der kleinen Pat.-Zahl nicht möglich war, eine Verbesserung der klinischen Diagnosegenauigkeit aufgrund spezifischer klinischer Zeichen zu untersuchen.

Sobald die Ansprechbarkeit auf Levodopa nachgewiesen wurde, wechselten die Pat. von Gruppe 2 (PossPD) in die Gruppe 1 (ProbPD). In diesem Fall verbesserte ein positives Ansprechen auf eine Levodopa-Therapie die Diagnosegenauigkeit von 26 % auf 53 %.

Bei Pat. mit klassischen Symptomen und einem Ansprechen auf die Levodopa-Therapie, aber einer Krankheitsdauer von < 5 Jahren lag der positive prädiktive Wert der Diagnose einer Parkinson-Erkrankung bei 53 % und stieg bei einem Verlauf von > 5 Jahren auf 88 % an. Sobald motorische Fluktuation oder Dyskinesien auftraten, verbesserte sich der positive prädiktive Wert noch weiter auf 92 % bzw. 96 %.

Zusammenfassend kann der positive prädiktive Wert der Diagnosegenauigkeit einer PK durch die Langzeitbeobachtung des klinischen Verlaufs erheblich verbessert werden und ist dem Kriterium eines Ansprechens auf Levodopa überlegen.

Begründung der Empfehlung

Die Evidenz beruht auf einer Klasse-2-Studie von Adler, der bei 131 Pat. zeigen konnte, dass eine Langzeitbeobachtung von > 5 Jahren dem Nachweis eines positiven Ansprechens auf eine Levodopa-Therapie überlegen ist.

**Empfehlung**	**Modifiziert<br>Stand (2023)**
▪ Das therapeutische Ansprechen auf eine Levodopa-Therapie kann die Diagnosegenauigkeit verbessern. Allerdings ist die Diagnosegenauigkeit bei einem längeren Verlauf von ≥ 5 Jahren nochmals deutlich besser, sodass ein langfristiges klinisches Follow-up für die Diagnose der PK einer Diagnose aufgrund einer Ansprechbarkeit auf eine Levodopa-Therapie überlegen ist.<br>▪ Die Beobachtung des Krankheitsverlaufs sollte über mehr als fünf Jahre einschließlich regelmäßiger klinischer Verlaufskontrollen erfolgen, um einerseits die Diagnose einer PK zu bestätigen und andererseits entsprechende therapeutische Entscheidungen zu treffen.	
Konsensstärke: 100%, starker Konsens	

Fragestellung 6: Wie effektiv ist das therapeutische Ansprechen auf eine Dopaminagonisten-Therapie im Vergleich mit dem langfristigen klinischen Follow-up für die Diagnose der PK?

Hintergrund

Die Differenzialdiagnose der unterschiedlichen Parkinson-Syndrome bereitet oft gerade in den ersten Krankheitsjahren erhebliche Schwierigkeiten. Daraus resultiert die Suche nach klinisch eindeutigen Prädiktoren für die PK oder auch für atypische Parkinson-Syndrome. Die klinische Erfahrung zeigt, dass Pat. mit einer PK in den wesentlichen Kardinalsymptomen mit Ausnahme des Tremors eine erhebliche Verbesserung durch die Gabe von Levodopa oder Dopaminagonisten erfahren. Dagegen zeigen die meisten atypischen Parkinson-Syndrome im Verlauf der Erkrankung nur eine eingeschränkte oder gar keine Symptomverbesserung durch die Gabe von Levodopa oder eines Dopaminagonisten. Daraus resultiert die klinische Praxis des Levodopa-Tests: Eine klinische Verbesserung von > 30% in der UPDRS III wird als „positives Ansprechen“ und somit als Zeichen für eine PK gewertet, dagegen eine Verbesserung in der UPDRS von < 30% als „fehlendes Ansprechen“. Trotz der gängigen klinischen Praxis zeigen jedoch Pat. mit atypischen Parkinson-Syndromen, insbesondere in den ersten Jahren, oft noch eine positive Antwort auf dopaminerge Stimulation, wenn auch die fehlende Antwort einen schlechten Verlauf der Erkrankung prädizieren kann.

Evidenzgrundlage

Es wurden in einer formalen Literaturrecherche keine Studien gefunden, die die Effektivität der Dopaminagonisten-Gabe in der Prädiktion der korrekten Diagnose einer PK untersuchten.

Ergebnis

Somit bleibt zwar die klinische red flag eines fehlenden Ansprechens auf die Gabe von Levodopa oder eines Dopaminagonisten in der Prädiktion einer korrekten Diagnose eines Parkinson-Syndroms bestehen, die wissenschaftliche Evidenz dieser Erkenntnis ist aber durch fehlende systematische prospektive Langzeitstudien bislang nicht gegeben.

Begründung der Empfehlung

Aufgrund fehlender Evidenz kann keine evidenzbasierte Empfehlung bzgl. der Effektivität der mittleren Dopaminagonisten-Therapie (Tabelle 6 in Kapitel 3.1) für die Prädiktion einer korrekten Diagnose eines Parkinson-Syndroms gegeben werden. Nach klinischer Einschätzung ist die Effektivität von Dopaminagonisten bei atypischen Parkinson-Syndromen eher geringer als die von L-Dopa und insbesondere bei der MSA mit stärkeren Nebenwirkungen behaftet (z.B. ausgeprägtere Verstärkung einer orthostatischen Hypotension).

**Empfehlung**	**Neu**<br>**Stand (2023)**
Aufgrund fehlender Evidenz kann keine Empfehlung bzgl. der Effektivität der mittleren Dopaminagonisten-Therapie (siehe Kapitel „Anti-Parkinson-Medikamente“, Tab. 6) für die Prädiktion einer korrekten Diagnose einer PK gegeben werden.	
Konsensstärke: 97%, starker Konsens	

Fragestellung 7: Ist es sinnvoll, die MDS-Prodromalkriterien bei der Diagnose einer PK im Frühstadium einzubeziehen im Vergleich zum klinischen Verlauf über 3–5 Jahre?

Hintergrund

Den klassischen motorischen Symptomen Bradykinesie, Rigor und Ruhetremor, die die Diagnosestellung der Parkinson-Erkrankung ermöglichen, geht in der Regel eine Phase über Jahre bis Jahrzehnte voraus, in der sich der der Erkrankung zu Grunde liegende neurodegenerative Prozess im Nervensystem ausbreitet, aber in den betreffenden Hirnarealen noch nicht das Ausmaß erreicht, das zur Ausprägung dieser klassischen motorischen Symptome führt. Allerdings kann in dieser sogenannten prodromalen Phase der neurodegenerative Prozess zu anderen entweder nicht motorischen Symptomen oder motorischen Symptomen leichter Ausprägung führen, die typisch, aber nicht spezifisch für die zugrunde liegende Erkrankung sind.

Auf dem Boden dieser Tatsache hat die internationale Movement Disorders Society Kriterien erarbeitet, die mithilfe Bayesianischer Statistik berechnen, mit welcher Wahrscheinlichkeit sich ein Individuum bei Vorliegen bestimmter prodromaler Symptome und Risikofaktoren für die Parkinson-Erkrankung (z.B. Pestizidexposition, genetische Belastung) in der Prodromalphase der Erkrankung befindet. Kriterium für die Aufnahme von Prodromalmarkern und Risikofaktoren in die statistische Berechnung war, dass mindestens zwei longitudinale prospektive Studien vorlagen, anhand derer ein „Plausibilitätsquotient“ (likelihood ratio, LR) berechnet werden konnte. Diese LR fließt neben dem Alter der betreffenden Person (als sogenannte prior) in eine Wahrscheinlichkeitsformel ein, die berechnet, mit welcher Wahrscheinlichkeit sich eine Person bei Vorliegen oder Nichtvorliegen bestimmter Symptome in der Prodromalphase befindet. Die 2015 erstmals veröffentlichten Prodromalkriterien wurden 2019 aktualisiert.

Evidenzgrundlage

Für diese Kapitel fanden sich in dieser Literaturrecherche:

– die beiden Prodromalkriterien [14] und [15], die für jedes/n der aufgenommenen Prodromalsymptome bzw. Risikomarker mindestens 2 prospektive longitudinale Studien angeben

– eine longitudinale prospektive Studie in der Allgemeinbevölkerung, die die Entwicklung einer Parkinson-Erkrankung in Bezug auf die Ergebnisse von Untersuchungen gemäß der Prodromalkriterien über einen Zeitraum von 10 Jahre untersucht [16]

Eine Anwendung der Prodromalkriterien im Vergleich zu einem klinischen Verlauf über 3–5 Jahre findet sich in der Literatur nicht.

Ergebnis

Die auf prospektiven longitudinalen Studien basierenden prodromalen Kriterien für die Parkinson-Erkrankung fassen Risikofaktoren und Prodromalmarker hilfreich zusammen. Die Anwendung dieser Kriterien in der frühen Phase der Parkinson-Erkrankung kann die Diagnosestellung unterstützen. Dies gilt sowohl für einzelne Symptome – z.B. das Vorliegen einer REM-Schlaf-Verhaltensstörung (RBD) – wie auch für die Kombination von Symptomen. Die Anwendung der gesamten Prodromalkriterien bei

Individuen, in denen eine Diagnose noch nicht gestellt werden kann, kann bei Erreichen bestimmter Wahrscheinlichkeitswerte zur Diagnose „wahrscheinliches Vorliegen einer prodromalen Parkinson-Erkrankung" und zur Diagnose „mögliches Vorliegen einer Parkinson-Erkrankung" führen.

In einer prospektiven longitudinalen Studie in der Allgemeinbevölkerung fanden sich bei Individuen mit „wahrscheinlichem Vorliegen einer prodromalen Parkinson-Erkrankung" eine Spezifität für die Vorhersage der Parkinson-Erkrankung nach 3 Jahren von 98.8% und eine Sensitivität von 66.7% mit einem positiv prädiktiven Wert von 40.0%. Im Verlauf von 5 und 10 Jahren blieb die Spezifität stabil, die Sensitivität nahm ab, der prospektive prädiktive Wert nahm zu. Auch wenn kein direkter Vergleich der Prodromalkriterien mit einem klinischen Verlauf über 3–5 Jahre vorliegt, kann das Vorliegen eines „wahrscheinlichen Prodromalstadiums" die Diagnose Parkinson in den ersten Jahren, in denen die motorischen Symptome noch weniger ausgeprägt sind, unterstützen. Einschränkend ist hier zu erwähnen, dass das Vollbild der motorischen Symptome und ein Ansprechen auf dopaminerge Therapie auch unabhängig von den Prodromalkriterien bei klinischer Parkinson-Diagnose erheblich zur Diagnosesicherheit beitragen.

Begründung der Empfehlung

Die Evidenz beruht auf den Prodromalkriterien (erste und überarbeitete Version), denen jeweils für jeden Risikofaktor und jeden Prodromalmarker mindestens 2 prospektive, longitudinale Studien zugrunde liegen. In einer prospektiven Studie wurde ein moderater bis hoher prädiktiver Wert für die Entwicklung einer Parkinson-Erkrankung bei „wahrscheinlichem Vorliegen einer Prodromalphase" belegt. Da es sich bei der Parkinson-Erkrankung um einen kontinuierlichen degenerativen Prozess handelt, kann die Anwendung der Prodromalkriterien die Diagnosesicherheit in der Frühphase der Erkrankung stärken, auch wenn kein direkter Vergleich mit dem klinischen Verlauf nach 3–5 Jahren vorliegt.

Empfehlung	Neu<br>Stand (2023)
Die Anwendung der Prodromalkriterien in der frühen Phase der Parkinson-Erkrankung ist hilfreich und sollte im klinischen Alltag erwogen werden, damit mehr in den ersten Jahren Diagnosesicherheit entsteht. Selbstverständlich ist eine kontinuierliche Diagnoseüberprüfung insbesondere in der Frühphase der Erkrankung wichtig.	
Konsensstärke: 97,1%, starker Konsens	

Fragestellung 8: Welchen prädiktiven Wert hat eine validierte Geruchstestung zur Detektion einer Hyposmie bei Diagnosestellung im Vergleich zum klinischen Verlauf für die Diagnose und den Verlauf einer PK?

Hintergrund

Die Hyposmie ist ein nicht motorisches Symptom der Parkinson-Erkrankung, das bereits in der Prodromalphase auftritt. Liegt sie bereits bei Auftreten der ersten motorischen Symptome vor, wird die diagnostische Sicherheit erhöht. Riechstörungen werden oft erst auf Nachfrage berichtet und sind den Pat. häufig gar nicht bewusst. Objektive Messungen der Riechfunktion können mittels Bed-

side Tests wie dem University of Pennsylvania Smell identification Test (UPSIT) oder dem meistens in Deutschland verwendeten Sniffin' Sticks Test erfolgen [17]. Hierbei werden verschiedene Domänen des Riechens (Detektion, Identifikation, Diskrimination) unterschieden. Nach der „body vs. brain first"-Theorie könnte sich das Ausmaß der Neurodegeneration im Bulbus olfactorius und in den assoziierten Zentren und somit das Vorhandensein einer Hyposmie je nach Erkrankungsbeginn unterscheiden und durch eine fortgeschrittenere Neurodegeneration in den entsprechenden Zentren bedingt sein [18].

Evidenzgrundlage

Für diese Kapitel fanden sich in dieser Literaturrecherche:

die beiden Publikationen zu den Prodromalkriterien [14] [15], die für jedes/n der aufgenommen Prodromalsymptome bzw. Risikomarker mindestens 2 prospektive longitudinale Studien angeben. Eine Anwendung der Prodromalkriterien im Vergleich zu einem klinischen Verlauf über 3–5 Jahre findet sich in der Literatur nicht.

Zusätzlich erfolgte eine Literaturrecherche zu den Begriffen „Parkinson's disease AND olfactory impairment / hyposmia AND prediction" und „Parkinson's disease progression AND olfactory dysfunction". Hier fanden sich zwei Metaanalysen zu der epidemiologischen Verknüpfung von Parkinson-Erkrankung und Hyposmie ([19], [20]), zwei große epidemiologische Studien [21] [22], zwei Reviews ([23], [17]) sowie kleinere prospektive Kohortenstudien und crosssectionale Studien ([24], [25], [26], [27], [28]) und größere klinische prospektive Kohortenstudien ([29], [30], [31], [32]) sowie der PPMI-Kohorte ([33]). Zusätzlich konnten eine Studie zum prädiktiven Wert der Testung auf Hyposmie im Vergleich zum klinischen Verlauf mit dem Außenkriterium einer histopathologischen Diagnose sowie eine weitere Studie, die die diagnostische Sicherheit im Verlauf über allerdings nur zwei Jahre im Vergleich zur Testung auf Hyposmie und den akuten Levodopa-Test verglich, identifiziert werden [34], [12].

Ergebnis

Riechstörungen können erfragt oder objektiv mittels verschiedener Testverfahren gemessen werden. Riechstörungen werden subjektiv oft nicht wahrgenommen (bis zu 72% der Parkinson-Pat. [17]) oder erst auf Nachfrage berichtet, sodass sich die Durchführung standardisierter (Bedside)Tests bewährt hat. Hierfür sind in der Routine verschiedene einfach anzuwendende Tests (u.a. UPSIT (University of Pennsylvania Smell Identification Test), die abgeleitete Kurzversion B-SIT (Brief 12-item Smell Identification Test) sowie der Sniffin' Sticks Test) etabliert, dabei ist der Sniffin' Sticks Test der am meisten verwendete Test. Beim UPSIT sind einige der Gerüche nicht allgemein verbreitet und deshalb wurde der Tests auf verschiedene Kulturkreise angepasst. Der B-SIT wurde entworfen, um diese cross-kulturellen Differenzen zu überwinden. Beim Sniffin' Sticks Test werden verschiedene Teststifte mit Gerüchen präsentiert, die Auswertung erfolgt in 3 Modalitäten (Identifikation, Schwellenwert, Diskrimination). Dabei zeigte sich in einer Studie, dass der Subscore Identifikation des Sniffin' Sticks Tests gegenüber dem Gesamtscore (bestehend aus Schwellenwert, Identifikation und Diskrimination) nahezu gleichwertig abschnitt und somit eine Vereinfachung der Testverfahren möglich ist.

Aufwendigere Verfahren zur Untersuchung von Riechstörungen wie olfaktorisch evozierte Potenziale finden nur in spezialisierten Zentren Anwendung.

Riechstörungen sind auch in der Allgemeinbevölkerung häufig und betreffen ca. 60–80% der älteren Menschen ab 80 Jahren [23], sodass das isolierte Vorliegen einer Riechstörung zunächst als unspezifisch anzusehen ist, da auch Riechstörungen u.a. nach Viruserkrankungen, Nasenoperationen etc. auftreten können. Entsprechend haben Screening-Untersuchungen bei Pat. mit idiopathischer Hyposmie nur wenige Pat. identifizieren können, die im Verlauf eine Parkinson-Erkrankung entwickelten [24]. Hingegen wurde in größeren epidemiologischen Studien und Metaanalysen eine klare Assoziation zwischen Hyposmie und Parkinson-Erkrankung nachgewiesen [21], [19], wobei die Hyposmie den motorischen Symptomen in der Regel vorausgeht. Allerdings weisen auch im Verlauf der PK bis zu 44% der Pat. eine Normosmie auf [31]. In den Baseline-Daten der Parkinson's Progression Marker Initiative (PPMI)-Studie, einer multizentrischen Beobachtungsstudie mit frühen noch unbehandelten Pat. mit PK (n=416), ließ sich bei nur 34.9% eine Hyposmie nachweisen. Somit scheint nach aktueller Studienlage auch das Ausmaß der olfaktorischen Dysfunktion im Verlauf der Parkinson-Erkrankung progredient zu sein [20]. Die Wahrscheinlichkeit, dass Pat. aus dem Prodromalstadium einer klinisch isolierten REM-Schlafverhaltensstörung (iRBD) eine manifeste PK (sog. Konversion) entwickeln, ist bei Vorliegen einer Hyposmie im Vergleich zu Pat. mit einer Normosmie erhöht [25], [32]. Dies zeigte sich auch in den überarbeiteten MDS-Prodromalkriterien, die bei motorischen Symptomen und Hyposmie einen „Plausibilitätsquotienten" (likelihood ratio, LR) von 6.4 zur Entwicklung einer PK ausweisen [14], [15]. Zugleich ist bei Pat. mit einer Parkinson-Erkrankung das Vorliegen einer Hyposmie und einer REM-Schlaf-Verhaltensstörung mit einer schlechteren Prognose sowohl im Bezug auf motorische [33], [26] als auch auf kognitive Symptome assoziiert [27].

Basierend auf einem klinisch-genetischen Klassifikationsmodell – etabliert in der PPMI-Kohorte –, zeigten sich unter Berücksichtigung von Alter, Geschlecht, Riechfunktion (gemessen mittels UPSIT), genetischem Risiko sowie unter Berücksichtigung einer positiven Familienanamnese im Hinblick auf eine Parkinson-Erkrankung eine sehr hohe Sensitivität (0.834) und Spezifität (0.903) in der Diskrimination von Parkinson-Erkrankung gegen Kontrollen [22]. Bei Anwendung dieses Modells auf andere Studienkohorten (z.B. PARS) wurden hiermit sogar noch bessere AUC-Werte in der ROC-Analyse erreicht [22].

Bei der Differenzialdiagnose zeigte sich in einer kleinen Kohortenstudie beim Vergleich zwischen PK, atypischen Parkinson-Syndromen (MSA, PSP, DLB) und anderen Erkrankungen (u.a. essenzieller Tremor), dass eine mittels B-SIT diagnostizierte Hyposmie eine Abgrenzung der PK gegen Pat. ohne Parkinson-Syndrom (z.B. Pat. mit essenziellem Tremor) ermöglichte, jedoch keine Abgrenzung zwischen PK und atypischen Parkinson-Syndromen und auch keine Differenzierung der atypischen Parkinson-Syndrome gegen andere Erkrankungen (wie essenziellen Tremor [28]). Bei der Abgrenzung aller Parkinson-Syndrome (PK, atypische Parkinson-Syndrome, vaskuläres Parkinson-Syndrom) gegen andere Erkrankungen (wie essenziellen Tremor) ergab sich dabei eine Sensitivität von 80% bei einer Spezifität von 40%, einem positiven prädiktiven Wert von 80% und einem negativen prädiktiven Wert von 40% bei einer Gesamt-Diagnosesicherheit (accuracy) von 70%. Bei der Abgrenzung Parkinson-Erkrankung gegen andere Erkrankungen (z.B. essenzieller Tremor ohne atypische Parkinson-

Syndrome wie MSA) lag die Sensitivität bei 92%, die Spezifität bei 40%, der positive prädiktive Wert bei 71%, der negative prädiktive Wert bei 75% und die diagnostische Genauigkeit bei 72%.

In einer retrospektiven Analyse von Daten über einen Verlauf von zwei Jahren zeigte sich bei der differenzialdiagnostischen Abgrenzung einer PK gegen andere Parkinson-Syndrome bei Einbeziehung des Ansprechens im standardisierten Levodopa-Test allein oder einer Kombination aus Levodopa-Test und Hyposmie-Testung mittels Sniffin' Sticks eine Verbesserung der Diagnosesicherheit (accuracy) insbesondere durch Zunahme der Sensitivität (von 0.70 auf 0.90) bei Abnahme der Spezifität (0.90 auf 0.74), nahezu stabilem positivem prädiktivem Wert (0.97 auf 0.91) und deutlich höherem negativem prädiktivem Wert (von 0.52 auf 0.72) [12]. Allerdings war in dieser Studie die klinische Diagnose das Außenkriterium, sodass kein Vergleich zwischen der Testung der Hyposmie im Vergleich zum rein klinischen Verlauf erfolgte.

Hingegen wurde in einer Studie mit Daten der „Arizona Study of Aging and Neurodegenerative Disorders" (AZSAND, [34]) die Diagnose der PK histopathologisch gestellt und somit die klinische Diagnose nochmals validiert. In dieser Studie war die Diagnosesicherheit geringer bei Vorliegen eines Krankheitsverlaufs von weniger als 5 Jahren und/oder einem fehlenden Ansprechen auf die Medikation. Dabei war das Vorliegen einer „möglichen Parkinson-Erkrankung" sowie einer „wahrscheinlichen Parkinson-Erkrankung" vordefiniert worden. Dabei wurde die Diagnose einer PK lediglich bei 22% der Pat. mit möglicher PK histopathologisch verifiziert. Hierbei lagen zwar zwei von drei der Kardinalsymptome vor, aber der Krankheitsverlauf betrug weniger als fünf Jahre und meist erfolgte noch keine medikamentöse Therapie oder es war kein Ansprechen auf die Medikation zu verzeichnen. Hingegen erhöhte das Vorliegen einer nach vordefinierten Kriterien „wahrscheinlichen PK" (2/3 Kardinalsymptomen vorliegend, Ausschluss symptomatischer Ursachen und positives Ansprechen auf die Medikation) die Wahrscheinlichkeit einer histopathologischen Bestätigung der Diagnose auf 84,7%. Allerdings zeigte sich auch hier ein deutlicher Unterschied zwischen einem kürzeren und einem längeren Krankheitsverlauf (<5 Jahre positiver prädiktiver Wert (PPV) 70,6%, >5Jahre PPV 89,1%). Bei Einbeziehung eines Riechtests (UPSIT (Normwerte 34-40, Cut-off der Studie <22 Punkte) innerhalb der ersten beiden Jahre der Konsultationen zeigte sich eine deutliche Erhöhung des positiven prädiktiven Wertes bei den Pat. mit einer möglichen Parkinson-Erkrankung von 22% auf 83,3% und auf 89,7% bei den Pat. mit einer wahrscheinlichen PK.

Zusammenfassend erhöht somit insbesondere im Frühstadium der PK das Vorliegen einer Hyposmie den positiven prädiktiven Wert und die Diagnosesicherheit der PK [17], [28].

Begründung der Empfehlung

Die Evidenz beruht auf den Prodromalkriterien (erste und überarbeitete Version) und der Prävalenz des nicht motorischen Symptoms Hyposmie bei Parkinson. Insbesondere in der Frühphase der Erkrankung liegt eine Hyposmie nur bei einem Teil der Pat. vor, im Verlauf der Erkrankung kommt es bei der deutlichen Mehrheit der Pat. zu einer Hyposmie. Zusätzlich erhöht sich aber gerade in der Frühphase der Erkrankung die diagnostische Sicherheit, wenn eine Hyposmie detektierbar ist. Somit ist das Vorliegen einer Hyposmie bei Vorliegen in der Frühphase zur Diagnosestellung hilfreich, ein Nichtvorliegen schließt die Erkrankung jedoch nicht aus.

Empfehlung	Neu<br>Stand (2023)
Eine Geruchstestung, z.B. mittels des Sniffin' Sticks Tests, bei Diagnosestellung erhöht nach Ausschluss häufiger anderer Ursachen für eine Hyposmie den positiven prädiktiven Wert auf ≥80% und die Diagnosesicherheit einer PK und wird somit auch aufgrund der geringen Invasivität zur ergänzenden Diagnostik empfohlen. Allerdings schließt das Vorliegen einer Normosmie eine PK nicht aus.	
Konsensstärke: 97,3%, starker Konsens	

Fragestellung 9: Welchen prädiktiven Wert hat eine standardisierte polysomnographische Untersuchung zur Detektion einer isolierten REM-Schlaf-Verhaltensstörung (RBD) bei Diagnosestellung im Vergleich zum klinischen Verlauf für die Diagnose einer PK?

Hintergrund

Eine REM-Schlafverhaltensstörung (RBD) gehört zu den nicht motorischen prodromal auftretenden Symptomen der Parkinson-Erkrankung, die die diagnostische Sicherheit erhöhen können. Die REM-Schlaf-Verhaltensstörung ist durch lebhaftes, teilweise fremd- und eigengefährdendes Ausagieren von Träumen, teilweise auch mit Vokalisationen, und im Schlaflabor nachweisbare fehlende REM-Atonie gekennzeichnet. Die Diagnose einer REM-Schlaf-Verhaltensstörung kann anhand der Anamnese, Fremdanamnese und mittels Polysomnographie gestellt werden, dabei wird anhand der aktuellen Kriterien der ICSD-3 eine Bestätigung mittels Polysomnographie gefordert. RBD-Symptome können mittels Fragebögen erfasst und somit als mögliche RBD diagnostiziert werden, allerdings ist die geringe diagnostische Sicherheit von Fragebögen bekannt, da zum einen vielen Pat. die Symptome nicht bewusst und somit nur durch fremdanamnestische Angaben verifizierbar sind und sich zum anderen viele „mimics" ergeben wie z.B. andere Parasomnien, unwillkürliche Beinbewegungen (PLMS) und andere, die ebenso wie z.B. schwergradige schlafbezogene Atmungsstörung Symptome einer RBD hervorrufen bzw. imitieren können. Der Goldstandard der Diagnostik ist somit eine polysomnographische Untersuchung im Schlaflabor, in der zusätzlich zu den Verhaltensauffälligkeiten mit Boxen, Schlagen und Um-sich-Treten auch Vokalisationen erfasst werden und die Diagnose nach Erfüllen der Kriterien durch den zusätzlichen Nachweis einer unvollständigen oder fehlenden Atonie im REM-Schlaf und gleichzeitigem Ausschluss von „RBD-mimics" erst definitiv gestellt wird.

Evidenzgrundlage

Für diese Kapitel fanden sich in dieser Literaturrecherche:

die beiden Publikationen zu den Prodromalkriterien [14] [15] die für jedes/n der aufgenommenen Prodromalsymptome bzw. Risikomarker mindestens 2 prospektive longitudinale Studien angeben

eine Metaanalyse bezüglich der Prävalenz bei RBD [35] und die Auswertung von Baseline-Daten der Parkinson's Progression Marker Initiative (PPMI)-Studie [36]. Eine Anwendung der Prodromalkriterien im Vergleich zu einem klinischen Verlauf über 3–5 Jahre findet sich in der Literatur nicht. Bei der Literaturrecherche zu „Parkinson's disease AND REM-sleep behavior disorder"

fanden sich zusätzlich noch eine größere crosssectionale Studie zur Diagnosefindung der RBD bei Parkinson-Erkrankung [37] sowie 2 crosssectionale Studien ([26], [27]) und eine größere klinische prospektive Kohortenstudie der PPMI-Kohorte ([33]).

Ergebnis

Die Sensitivität und v.a. die Spezifität der Diagnose einer REM-Schlaf-Verhaltensstörung ist gering, wenn Fragebögen allein angewandt werden, Sensitivität und Spezifität sind am höchsten bei Kombination aus Anamnese, Fremdanamnese und Polysomnographie, allerdings können gelegentlich die klinischen und polysomnographischen Kriterien auch erst im Verlauf voll erfüllt sein [37], da sich die Symptomatik im Verlauf verstärken kann [29], [30].

Im Krankheitsverlauf scheinen Pat. mit einer klinisch isolierten REM-Schlaf-Verhaltensstörung (iRBD) bei Vorliegen einer Hyposmie einen rascheren Progress aufzuweisen [25], [32], sodass sie schneller die Kriterien für eine manifeste Parkinson-Erkrankung oder andere neurodegenerative Erkrankungen erfüllen (sog. Konversion), wenngleich ein nicht unerheblicher Teil von Pat. trotz der Diagnose einer Parkinson-Erkrankung weiterhin eine Normosmie aufweist (bis 44%) [31]. Zugleich ist bei Pat. mit einer Parkinson-Erkrankung das Vorliegen einer Hyposmie und einer REM-Schlaf-Verhaltensstörung mit einer schlechteren Prognose in Bezug auf eine Zunahme sowohl motorischer Einschränkungen [33], [26] als auch kognitiver Defizite assoziiert [27].

Der „Plausibilitätsquotient“ (likelihood ratio, LR) bei Vorliegen einer polysomnographisch nachgewiesen RBD beträgt wie in den ersten Prodromalkriterien 130. Eine RBD findet sich in einer Metaanalyse von 28 Studien bei 42.3% der Parkinson-Pat. Die hohe LR dieses Merkmals in der Prodromalphase ist auf die hohe Spezifität der RBD zurückzuführen. Da nur ein Teil der Parkinson-Pat. eine RBD im Verlauf der Erkrankung aufweist, ist ein hoher prädiktiver Wert nur bei Vorliegen dieses Symptoms zu verzeichnen.

Ein Vergleich des prädiktiven Werts einer iRBD im Vergleich zum klinischen Verlauf findet sich in der Literatur nicht.

Begründung der Empfehlung

Die Evidenz beruht auf den Prodromalkriterien (erste und überarbeitete Version) und der Prävalenz der RBD bei Parkinson.

Eine RBD findet sich einer Metaanalyse auch im Verlauf der Erkrankung bei weniger als der Hälfte der Parkinson-Pat. Das Prüfen des Vorliegens einer iRBD bei Diagnosestellung der PK ist hilfreich, da das Vorliegen einer iRBD die Diagnose einer alpha-Synukleinopathie (wie einer PK oder MSA) im Vergleich zu anderen Erkrankungen (wie z.B. essenzieller Tremor) wahrscheinlicher macht. Somit ist das Vorliegen einer RBD in der Frühphase der PK hilfreich, ein Nichtvorliegen schließt die Erkrankung jedoch nicht aus. Ein direkter Vergleich des prädiktiven Werts einer iRBD im Vergleich zum klinischen Verlauf ist in der Literatur nicht zu finden.

Empfehlung	Neu<br>Stand (2023)
▪ Das Prüfen des Vorliegens einer RBD bei Diagnosestellung der PK ist hilfreich.<br>▪ Die Diagnose einer wahrscheinlichen RBD sollte anhand von Anamnese und Fremdanamnese und nicht allein basierend auf Fragebögen gestellt werden. Zur definitiven Diagnosestellung einer RBD soll eine polysomnographische Untersuchung im Schlaflabor erfolgen.	
Konsensstärke: 100%, starker Konsens	

Fragestellung 10a: Wie ist die Wertigkeit einer validierten Geruchstestung zur Abgrenzung einer PK von atypischen Parkinson-Syndromen wie PSP oder MSA?

Hintergrund

Einschränkungen des Riechsinns (Hyp-/Anosmie) ist eines der häufigsten und im Krankheitsverlauf oftmals sehr früh auftretenden nicht motorischen Symptome bei der PK. Gleichzeitig ist es gerade am Anfang des Krankheitsverlaufs teilweise schwierig, atypische Parkinson-Syndrome von der PK abzugrenzen, sodass klinisch-diagnostische Marker für eine Unterscheidung zwischen den verschiedenen Krankheitsentitäten von großer Bedeutung sind.

Evidenzgrundlage

Die Literatursuche in Pubmed zu den Begriffen „Parkinson‘s disease AND olfactory impairment/hyposmia“ und „Parkinson syndromes respec. MSA resp. PSP AND hyposmia OR olfactory dysfunction OR smell test“ erbrachte 2 crosssectionale Studien ([38], [39]), 2 größere longitudinale Kohortenstudien bei der RBD ([40], [31]), 1 Metaanalyse ([41]), 1 Literatur-Vergleichsstudie ([42]) sowie 1 Review ([17]).

Ergebnis

Longitudinale Studien an kleinen Pat.-Gruppen von Pat. mit REM-Schlaf-Verhaltensstörung zeigen, dass Pat. ,die eine MSA entwickelten, im Gegensatz zu Pat., die eine PK entwickelten, meist eine Normosmie aufwiesen [31], [40]. Zudem wurde in einer kleinen crosssectionalen Studie bei Pat. mit PSP im Vergleich zu Pat. mit PK ein normaler Geruchssinn gefunden [38]. In weiteren kleineren cross-sectionalen Studien bei MSA und PSP-Pat. wurden keine Unterschiede zu Kontrollen oder allenfalls eine deutlich geringere Hyposmie als bei Pat. mit PK festgestellt [39].

Bei Verwendung des Subtests Identifikation des Sniffin’ Sticks Tests konnten ein positiver prädiktiver Wert von 85,7% und ein negativer prädiktiver Wert von 78,5% bei der Abgrenzung einer Parkinson-Erkrankung gegen Pat. mit einer MSA oder PSP gezeigt werden, dieser war vergleichbar zum Gesamttest (SDI Score), brauchte allerdings bedeutend kürzere Zeit für die Applikation. Bei Verwendung des besten Cut-offs mit hoher Spezifität war eine Unterscheidung zwischen Parkinson-Erkrankung und atypischen Parkinson-Syndromen mit einer guten Reliabilität möglich, wobei die Diskrimination zwischen Parkinson-Erkrankung und MSA besser war. In einer Metaanalyse mit mehr als 1.000 eingeschlossenen Pat. bestätigte sich, dass die Einschränkungen der Riechfunktion bei der

Parkinson-Erkrankung im Vergleich zur PSP deutlich stärker ausfallen, im Vergleich zu Pat. mit iRBD fanden sich keine signifikanten Unterschiede [41].

Begründung der Empfehlung

Zusammenfassend ist bei Vorliegen einer ausgeprägten Hyposmie eine Parkinson-Erkrankung im Vergleich zu einer MSA oder PSP wahrscheinlich, allerdings schließt eine Normosmie bei geringer Prävalenz auch eine Parkinson-Erkrankung nicht aus, genauso kann auch insbesondere bei der PSP und der MSA eine Hyposmie vorliegen [17]. Somit kann eine sehr ausgeprägte Riechstörung die Diagnose einer Parkinson-Erkrankung unterstützen, insbesondere in der Abgrenzung zur MSA, wenngleich Sensitivität und Spezifität nicht ausreichend sind, um eine sichere Abgrenzung zu erlauben.

**Empfehlung**	**Neu**<br>**Stand (2023)**
Eine Testung der Riechfunktion z.B. mittels Sniffin' Sticks Test, kann nach Ausschluss symptomatischer Ursachen einer Hyposmie als ergänzende Diagnostik erwogen werden. Bei Vorliegen einer ausgeprägten Hyposmie/Anosmie kann dies die Diagnose einer PK in der Differenzialdiagnose zu MSA und PSP unterstützen.	
Konsensstärke: 97,2%, starker Konsens	

Fragestellung 10b: Wie ist die Wertigkeit einer standardisierten polysomnographischen RBD-Diagnostik zur Abgrenzung einer PK von atypischen Parkinson-Syndromen wie PSP oder MSA?

Hintergrund

Die klinisch isolierte REM-Schlaf-Verhaltensstörung (iRBD) ist durch lebhaftes, teilweise fremd- und eigengefährdendes Ausagieren von Träumen, teilweise auch mit Vokalisationen, und im Schlaflabor nachweisbare fehlende REM-Atonie gekennzeichnet. Die RBD wurde erstmals 1986 beschrieben. RBD-Symptome können mittels Fragebögen erfasst werden (mögliche RBD), allerdings sind vielen Pat. die Symptome nicht bewusst, sodass sich häufig erst durch fremdanamnestische Angaben Hinweise auf eine RBD ergeben. Der Goldstandard der Diagnostik ist die polysomnographische Untersuchung im Schlaflabor, in der zusätzlich zu den Verhaltensauffälligkeiten mit Boxen, Schlagen und Um-sich-Treten auch Vokalisationen erfasst werden und die Diagnose nach Erfüllen der Kriterien durch den zusätzlichen Nachweis einer unvollständigen oder fehlenden Atonie im REM-Schlaf gestellt wird.

Evidenzgrundlage

Die Literatursuche in Pubmed zu den Begriffen „Parkinson's disease AND REM Sleep behavior disorder" und „Parkinson syndromes / MSA / PSP AND REM Sleep behavior disorder" erbrachte 2 Reviews ([43], [17]), 1 Metaanalyse ([44]) und einen systematischen Review ([45]), 1 longitudinale Kohortenstudie ([46]) sowie crossectionale monozentrische klinische Studien ([47], [48], [49], [43], [50], [51]).

Ergebnis

Pat. mit einer polysomnographisch gesicherten klinisch isolierten REM-Schlaf-Verhaltensstörung (iRBD) entwickeln im Verlauf mit sehr hoher Wahrscheinlichkeit (>90%) eine neurodegenerative Erkrankung, dabei liegen bei 98% der Pat. alpha-Synukleinopathien PK/DLB oder eine MSA vor [52], [42], [44], sodass die RBD als Prodromalstadium nicht nur der Parkinson-Erkrankung, sondern von alpha-Synukleinopathien allgemein anzusehen ist [44], wobei insgesamt bis zu 8% der Pat. dabei eine MSA entwickelten. Allerdings kommt die RBD auch bei atypischen Parkinson-Syndromen, z.B. der PSP, aber auch bei vaskulären Läsionen insbesondere im Hirnstamm sowie anderen u.a. neurodegenerativen Erkrankungen wie Alzheimer-Demenz vor, sodass die Diagnose einer RBD nicht das Vorliegen einer PSP oder anderer neurodegenerativer Erkrankungen ausschließt [45], [47], [48], [43]. Allerdings tritt eine RBD deutlich seltener bei anderen neurodegenerativen Erkrankungen als bei den alpha-Synukleinopathien auf und möglicherweise dann auch im Zusammenhang mit einer Ko-Pathologie aus Lewy-Körperchen und Tau [45]. Die Angaben der Häufigkeiten sind sehr unterschiedlich, was u.a. an der geringen Zahl an eingeschlossenen Pat. liegt. Bei der PSP wurde bei 20–50% der Pat. eine RBD gesehen [47], [48]. Bei der MSA [43] wurde in einer Studie bei 90% der Pat. polysomnographisch eine fehlende REM-Atonie nachgewiesen [51], in anderen kleineren Studien wurden jedoch teilweise auch nur Häufigkeiten um die 20% berichtet [49].

Die polysomnographischen Veränderungen bei Vorliegen einer RBD unterscheiden sich nicht grundsätzlich zwischen Pat. mit Parkinson-Erkrankung und MSA, können aber im Ausmaß je nach Kohorte variieren, wobei dies teilweise auf kleine Fallzahlen zurückzuführen ist [50], [46].

Begründung der Empfehlung

Die Evidenz beruht auf wenigen Studien mit guter Evidenzlage. Da insbesondere Korrelationen zu histopathologischen Untersuchungen bisher nicht in ausreichender Qualität vorhanden sind, muss offenbleiben, ob das Vorliegen einer RBD bei atypischen Parkinson-Syndromen und anderen neurodegenerativen Erkrankungen auf einer möglichen Ko-Pathologie von Alpha-Synuklein und anderen neurotoxischen Proteinen wie bspw. Tau beruht. Die Wahrscheinlichkeit, dass bei Nachweis einer RBD eine Alpha-Synukleinopathie vorliegt, ist höher als das Vorliegen einer anderen Pathologie, eine eindeutige Zuordnung ist mittels RBD jedoch nicht möglich.

**Empfehlung**	**Neu**<br>**Stand (2023)**
Eine Polysomnographie zur Diagnosesicherung einer RBD kann erwogen werden. Bei Nachweis einer RBD ist das Vorliegen einer alpha-Synukleinopathie (PK und MSA) wahrscheinlicher als das Vorliegen einer Tauopathie, jedoch schließt das Fehlen einer RBD eine Alpha-Synukleinopathie nicht aus und es kann nicht zwischen den verschiedenen Alpha-Synukleinopathien unterschieden werden.	
Konsensstärke: 100%, starker Konsens	

Fragestellung 11: Wie effektiv ist die Prognoseabschätzung bei der PK unter Berücksichtigung nicht motorischer Symptome (RBD, Hyposmie, Obstipation, Depression, Orthostase) im Vergleich zu rein motorischen Kriterien?

Hintergrund

Die Diagnose einer chronischen (neurodegenerativen) Erkrankung wie der PK ist für Pat. und deren Angehörige oft mit Verunsicherung und Zukunftsängsten verbunden, sodass sie sich bezüglich der Prognose und Therapieoptionen verlässliche Aussagen wünschen, die letztlich auch zu weitreichenden Lebensentscheidungen z.B. bezüglich Wohnformen und Therapielimitierung führen können. Darüber hinaus ist die Vorhersage des Verlaufs auch für die Behandler und Behandlerinnen von entscheidender Relevanz für die Wahl sowohl medikamentöser als auch unterstützender Therapien und zusätzlich von wissenschaftlichem Interesse bezüglich der Definition von Outcome- und prognostischen Parametern. Die PK ist eine heterogene Erkrankung mit verschiedenen Subtypen, deren Klassifizierung zunächst auf rein motorischen Subtypen basierte (u.a. akinetisch-rigider vs. Tremordominanztyp), in letzter Zeit jedoch auch zunehmend nicht motorische Symptome mit einbezieht.

Evidenzgrundlage

Die Abfrage von „parkinson's disease AND progression" ergab 3 Metaanalysen (von 2007 zur Progression der v.a. motorischen Symptome der PK, von 2023 zu Schlafstörungen, von 2021 zu Depressionen, [53], [54], [55]) sowie 1 retrospektive longitudinale Kohortenstudie ([56]), 2 crosssectionale Studien ([26], [27]) und 1 größere klinische prospektive Kohortenstudien der PPMI-Kohorte ([33]).

Ergebnis

In einer Metaanalyse von 2007 mit 27 eingeschlossenen Studien werden eine schlechtere Prognose und einer schnellere Zunahme motorischer Defizite assoziiert mit bereits bestehenden Einschränkungen der Aktivitäten des täglichen Lebens, kognitiven Einschränkungen und depressiven Symptomen [53]. Die Prävalenz der REM-Schlaf-Verhaltensstörung bei der PK wird in einer Metaanalyse von 2023 mit 46% angegeben. Das Vorliegen einer RBD war ebenfalls assoziiert mit einer schnelleren Progression motorischer Symptome und Fluktuationen, höherem Lebensalter, geringerem Bildungsstand, höheren Dosen dopaminerger Therapie (Levodopa-Äquivalenzdosis), längerer Erkrankungsdauer sowie ausgeprägteren autonomen und neuropsychiatrischen Symptomen wie kognitiven Auffälligkeiten und Halluzinationen [54]. In einer weiteren Metaanalyse zum Einfluss der Depression auf die Progression der PK wurden 129 Studien mit insgesamt 38.304 Teilnehmenden aus 28 Ländern eingeschlossen. Die Gesamtprävalenz einer Depression bei Parkinson-Pat. wird mit 38% angegeben. Pat. mit Depressionen zeigten dabei einen früheren Erkrankungsbeginn, niedrigeren Bildungsstand, schlechtere kognitive Leistungen und waren motorisch schwerer betroffen, dabei zeigte sich auch eine Assoziation zu Gangstörungen, insbesondere Freezing. Zudem war das Auftreten von Depressionen mit weiblichem Geschlecht und weiteren nicht motorischen Symptomen wie Angst und Apathie sowie Fatigue-Symptomen assoziiert.

In einer 2019 publizierten retrospektiven Kohortenstudie wurden zwischen 2009 und 2017 erhobene longitudinale Daten [56] von 111 Pat. mit autoptisch gesicherter PK anhand der Schwere und Ausprägung ihrer motorischen, kognitiven und autonomen und depressiven Symptome sowie Vorliegen oder Fehlen einer REM-Schlaf-Verhaltensstörung bei Diagnosestellung, basierend auf Cluster-Analysen der PPMI-Kohorte, in verschiedene Subtypen klassifiziert, die als vorherrschend mild-motorischer, intermediärer bzw. diffus maligner Subtyp bezeichnet wurden. Für die jeweiligen Subtypen wurde dann die Zeit bis zum Erreichen bestimmter Meilensteine der Erkrankung wie z.B. wiederholte Stürze, Rollstuhlgebundenheit, Demenz, Einweisung in eine Pflegeeinrichtung und Tod berechnet. Dabei erreichten Pat. mit dem diffus malignen Subtyp die Meilensteine signifikant früher als die anderen Subtypen, wobei das Alter als einziger relevanter weiterer Ko-Faktor identifiziert wurde. Dabei bestand der diffus maligne Subtyp überwiegend aus Männern in höherem Lebensalter mit geringerem Ansprechen auf Levodopa und wurde öfter fälschlicherweise als atypisches Parkinson-Syndrom klassifiziert.

Dabei erreichte dieser Subtyp alle Meilensteine früher als die anderen beiden Subtypen und hatte auch die kürzeste Überlebenszeit, allerdings waren alle Subtypen beim Tod im gleichen Maß von Einschränkungen betroffen.

Auch in anderen, teilweise kleineren Kohortenstudien wurde bei Parkinson-Pat. eine Assoziation zwischen dem Vorliegen einer REM-Schlaf-Verhaltensstörung und einer Hyposmie sowie einem schlechteren motorischen Outcome [33], [26] und kognitiven Defiziten gefunden [27].

Begründung der Empfehlung

Diese Daten zeigen, dass die Berücksichtigung nicht motorischer Symptome die Prognoseabschätzung bereits bei Diagnosestellung deutlich genauer möglich macht als die reine Berücksichtigung motorischer Einschränkungen.

**Empfehlung**	**Neu**<br>**Stand (2023)**
Die Ausprägung nicht motorischer Symptome sollte bereits bei Diagnosestellung einer PK zur Prognoseabschätzung herangezogen werden.	
Konsensstärke: 100%, starker Konsens	

Referenzen

1. Hughes AJ, Daniel SE, Kilford L, Lees AJ. Accuracy of clinical diagnosis of idiopathic Parkinson's disease: a clinico-pathological study of 100 cases. J Neurol Neurosurg Psychiatry. 1992;55(3):181-4
2. Hughes AJ, Daniel SE, Lees AJ. Improved accuracy of clinical diagnosis of Lewy body Parkinson's disease. Neurology. 2001;57(8):1497-9
3. Hughes AJ, Daniel SE, Ben-Shlomo Y, Lees AJ. The accuracy of diagnosis of parkinsonian syndromes in a specialist movement disorder service. Brain. 2002;125(Pt 4):861-70
4. Adler CH, Beach TG, Hentz JG, Shill HA, Caviness JN, Driver-Dunckley E, et al. Low clinical diagnostic accuracy of early vs advanced Parkinson disease: clinicopathologic study. Neurology. 2014;83(5):406-12

5. Postuma RB, Berg D, Stern M, Poewe W, Olanow CW, Oertel W, et al. MDS clinical diagnostic criteria for Parkinson's disease. Mov Disord. 2015;30(12):1591-601
6. Postuma RB, Poewe W, Litvan I, Lewis S, Lang AE, Halliday G, et al. Validation of the MDS clinical diagnostic criteria for Parkinson's disease. Mov Disord. 2018;33(10):1601-8
7. Berg D, Postuma RB, Bloem B, Chan P, Dubois B, Gasser T, et al. Time to redefine PD? Introductory statement of the MDS Task Force on the definition of Parkinson's disease. Mov Disord. 2014;29(4):454-62
8. Virameteekul S, Revesz T, Jaunmuktane Z, Warner TT, De Pablo-Fernández E. Clinical Diagnostic Accuracy of Parkinson's Disease: Where Do We Stand? Mov Disord. 2023;38(4):558-66
9. Merello M, Nouzeilles MI, Arce GP, Leiguarda R. Accuracy of acute levodopa challenge for clinical prediction of sustained long-term levodopa response as a major criterion for idiopathic Parkinson's disease diagnosis. Mov Disord. 2002;17(4):795-8
10. Clarke CE, Davies P. Systematic review of acute levodopa and apomorphine challenge tests in the diagnosis of idiopathic Parkinson's disease. J Neurol Neurosurg Psychiatry. 2000;69(5):590-4
11. Rossi P, Colosimo C, Moro E, Tonali P, Albanese A. Acute challenge with apomorphine and levodopa in Parkinsonism. Eur Neurol. 2000;43(2):95-101
12. Terroba Chambi C, Rossi M, Bril A, Vernetti PM, Cerquetti D, Cammarota A, Merello M. Diagnostic Value of Combined Acute Levodopa Challenge and Olfactory Testing to Predict Parkinson's Disease. Mov Disord Clin Pract. 2017;4(6):824-8
13. Berardelli A, Wenning GK, Antonini A, Berg D, Bloem BR, Bonifati V, et al. EFNS/MDS-ES/ENS [corrected] recommendations for the diagnosis of Parkinson's disease. Eur J Neurol. 2013;20(1):16-34
14. Berg D, Postuma RB, Adler CH, Bloem BR, Chan P, Dubois B, et al. MDS research criteria for prodromal Parkinson's disease. Mov Disord. 2015;30(12):1600-11
15. Heinzel S, Berg D, Gasser T, Chen H, Yao C, Postuma RB. Update of the MDS research criteria for prodromal Parkinson's disease. Mov Disord. 2019;34(10):1464-70
16. Mahlknecht P, Gasperi A, Djamshidian A, Kiechl S, Stockner H, Willeit P, et al. Performance of the Movement Disorders Society criteria for prodromal Parkinson's disease: A population-based 10-year study. Mov Disord. 2018;33(3):405-13
17. Marin C, Vilas D, Langdon C, Alobid I, López-Chacón M, Haehner A, et al. Olfactory Dysfunction in Neurodegenerative Diseases. Curr Allergy Asthma Rep. 2018;18(8):42
18. Borghammer P, Just MK, Horsager J, Skjærbæk C, Raunio A, Kok EH, et al. A postmortem study suggests a revision of the dual-hit hypothesis of Parkinson's disease. NPJ Parkinsons Dis. 2022;8(1):166
19. Sui X, Zhou C, Li J, Chen L, Yang X, Li F. Hyposmia as a Predictive Marker of Parkinson's Disease: A Systematic Review and Meta-Analysis. Biomed Res Int. 2019;2019:3753786
20. Ercoli T, Masala C, Cadeddu G, Mascia MM, Orofino G, Gigante AF, et al. Does Olfactory Dysfunction Correlate with Disease Progression in Parkinson's Disease? A Systematic Review of the Current Literature. Brain Sci. 2022;12(5)
21. Ross GW, Petrovitch H, Abbott RD, Tanner CM, Popper J, Masaki K, et al. Association of olfactory dysfunction with risk for future Parkinson's disease. Ann Neurol. 2008;63(2):167-73
22. Nalls MA, McLean CY, Rick J, Eberly S, Hutten SJ, Gwinn K, et al. Diagnosis of Parkinson's disease on the basis of clinical and genetic classification: a population-based modelling study. Lancet Neurol. 2015;14(10):1002-9
23. Attems J, Walker L, Jellinger KA. Olfaction and Aging: A Mini-Review. Gerontology. 2015;61(6):485-90
24. Marrero-González P, Iranzo A, Bedoya D, Serradell M, Niñerola-Baizán A, Perissinotti A, et al. Prodromal Parkinson disease in patients with idiopathic hyposmia. J Neurol. 2020;267(12):3673-82

25. Janzen A, Vadasz D, Booij J, Luster M, Librizzi D, Henrich MT, et al. Progressive Olfactory Impairment and Cardiac Sympathetic Denervation in REM Sleep Behavior Disorder. J Parkinsons Dis. 2022;12(6):1921-35
26. He R, Zhao Y, He Y, Zhou Y, Yang J, Zhou X, et al. Olfactory Dysfunction Predicts Disease Progression in Parkinson's Disease: A Longitudinal Study. Front Neurosci. 2020;14:569777
27. Kang SH, Lee HM, Seo WK, Kim JH, Koh SB. The combined effect of REM sleep behavior disorder and hyposmia on cognition and motor phenotype in Parkinson's disease. J Neurol Sci. 2016;368:374-8
28. Georgiopoulos C, Davidsson A, Engström M, Larsson EM, Zachrisson H, Dizdar N. The diagnostic value of dopamine transporter imaging and olfactory testing in patients with parkinsonian syndromes. J Neurol. 2015;262(9):2154-63
29. Sixel-Döring F, Trautmann E, Mollenhauer B, Trenkwalder C. Rapid eye movement sleep behavioral events: a new marker for neurodegeneration in early Parkinson disease? Sleep. 2014;37(3):431-8
30. Sixel-Döring F, Zimmermann J, Wegener A, Mollenhauer B, Trenkwalder C. The Evolution of REM Sleep Behavior Disorder in Early Parkinson Disease. Sleep. 2016;39(9):1737-42
31. Iranzo A, Marrero-González P, Serradell M, Gaig C, Santamaria J, Vilaseca I. Significance of hyposmia in isolated REM sleep behavior disorder. J Neurol. 2021;268(3):963-6
32. Mahlknecht P, Iranzo A, Högl B, Frauscher B, Müller C, Santamaría J, et al. Olfactory dysfunction predicts early transition to a Lewy body disease in idiopathic RBD. Neurology. 2015;84(7):654-8
33. Chen Y, Xue NJ, Fang Y, Jin CY, Li YL, Tian J, et al. Association of Concurrent Olfactory Dysfunction and Probable Rapid Eye Movement Sleep Behavior Disorder with Early Parkinson's Disease Progression. Mov Disord Clin Pract. 2022;9(7):909-19
34. Adler CH, Beach TG, Zhang N, Shill HA, Driver-Dunckley E, Mehta SH, et al. Clinical Diagnostic Accuracy of Early/Advanced Parkinson Disease: An Updated Clinicopathologic Study. Neurol Clin Pract. 2021;11(4):e414-e21
35. Zhang X, Sun X, Wang J, Tang L, Xie A. Prevalence of rapid eye movement sleep behavior disorder (RBD) in Parkinson's disease: a meta and meta-regression analysis. Neurol Sci. 2017;38(1):163-70
36. Paracha M, Herbst K, Kieburtz K, Venuto CS. Prevalence and Incidence of Nonmotor Symptoms in Individuals with and Without Parkinson's Disease. Mov Disord Clin Pract. 2022;9(7):961-6
37. Figorilli M, Marques AR, Meloni M, Zibetti M, Pereira B, Lambert C, et al. Diagnosing REM sleep behavior disorder in Parkinson's disease without a gold standard: a latent-class model study. Sleep. 2020;43(7)
38. Doty RL, Golbe LI, McKeown DA, Stern MB, Lehrach CM, Crawford D. Olfactory testing differentiates between progressive supranuclear palsy and idiopathic Parkinson's disease. Neurology. 1993;43(5):962-5
39. Robinson WP, van der Linden SM, Khan MA, Rentsch HU, Cats A, Russell A, Thomson G. HLA-Bw60 increases susceptibility to ankylosing spondylitis in HLA-B27+ patients. Arthritis Rheum. 1989;32(9):1135-41
40. Miyamoto T, Miyamoto M. Odor identification predicts the transition of patients with isolated RBD: A retrospective study. Ann Clin Transl Neurol. 2022;9(8):1177-85
41. Alonso CCG, Silva FG, Costa LOP, Freitas S. Smell tests to distinguish Parkinson's disease from other neurological disorders: a systematic review and meta-analysis. Expert Rev Neurother. 2021;21(3):365-79
42. Moscovich M, Heinzel S, Postuma RB, Reilmann R, Klockgether T, Jacobi H, et al. How specific are non-motor symptoms in the prodrome of Parkinson's disease compared to other movement disorders? Parkinsonism Relat Disord. 2020;81:213-8

43. Abbott SM, Videnovic A. Sleep Disorders in Atypical Parkinsonism. Mov Disord Clin Pract. 2014;1(2):89-96
44. Stankovic I, Fanciulli A, Kostic VS, Krismer F, Meissner WG, Palma JA, et al. Laboratory-Supported Multiple System Atrophy beyond Autonomic Function Testing and Imaging: A Systematic Review by the MoDiMSA Study Group. Mov Disord Clin Pract. 2021;8(3):322-40
45. Keir LHM, Breen DP. REM sleep behaviour disorder in patients without synucleinopathy. J Neurol Neurosurg Psychiatry. 2020;91(11):1239-40
46. Postuma RB, Pelletier A, Gagnon JF, Montplaisir J. Evolution of Prodromal Multiple System Atrophy from REM Sleep Behavior Disorder: A Descriptive Study. J Parkinsons Dis. 2022;12(3):983-91
47. Nomura T, Inoue Y, Takigawa H, Nakashima K. Comparison of REM sleep behaviour disorder variables between patients with progressive supranuclear palsy and those with Parkinson's disease. Parkinsonism Relat Disord. 2012;18(4):394-6
48. Arnulf I, Merino-Andreu M, Bloch F, Konofal E, Vidailhet M, Cochen V, et al. REM sleep behavior disorder and REM sleep without atonia in patients with progressive supranuclear palsy. Sleep. 2005;28(3):349-54
49. Nomura T, Inoue Y, Högl B, Uemura Y, Yasui K, Sasai T, et al. Comparison of the clinical features of rapid eye movement sleep behavior disorder in patients with Parkinson's disease and multiple system atrophy. Psychiatry Clin Neurosci. 2011;65(3):264-71
50. Iranzo A, Santamaría J, Rye DB, Valldeoriola F, Martí MJ, Muñoz E, et al. Characteristics of idiopathic REM sleep behavior disorder and that associated with MSA and PD. Neurology. 2005;65(2):247-52
51. Plazzi G, Corsini R, Provini F, Pierangeli G, Martinelli P, Montagna P, et al. REM sleep behavior disorders in multiple system atrophy. Neurology. 1997;48(4):1094-7
52. Iranzo A, Fernández-Arcos A, Tolosa E, Serradell M, Molinuevo JL, Valldeoriola F, et al. Neurodegenerative disorder risk in idiopathic REM sleep behavior disorder: study in 174 patients. PLoS One. 2014;9(2):e89741
53. Post B, Merkus MP, de Haan RJ, Speelman JD. Prognostic factors for the progression of Parkinson's disease: a systematic review. Mov Disord. 2007;22(13):1839-51; quiz 988
54. Maggi G, Vitale C, Cerciello F, Santangelo G. Sleep and wakefulness disturbances in Parkinson's disease: A meta-analysis on prevalence and clinical aspects of REM sleep behavior disorder, excessive daytime sleepiness and insomnia. Sleep Med Rev. 2023;68:101759
55. Cong S, Xiang C, Zhang S, Zhang T, Wang H, Cong S. Prevalence and clinical aspects of depression in Parkinson's disease: A systematic review and meta-analysis of 129 studies. Neurosci Biobehav Rev. 2022;141:104749
56. De Pablo-Fernández E, Lees AJ, Holton JL, Warner TT. Prognosis and Neuropathologic Correlation of Clinical Subtypes of Parkinson Disease. JAMA Neurol. 2019;76(4):470-9

2.2 Zerebrale MRT- und CT-Bildgebung

Autoren/Autorinnen: Jan Kassubek, Klaus Seppi, Cornelius Deuschl, Kathrin Reetz, Thilo van Eimeren

Fragestellung 12: Wie effektiv ist die cCT im Vergleich mit dem langfristigen klinischen Follow-up für die Differenzialdiagnose der PK gegenüber sekundären Parkinson-Syndromen?

Ergebnis

Die kraniale Computertomographie (cCT) grenzt die PK gegenüber sekundären Parkinson-Syndromen (insbesondere der Entität des vaskulären Parkinsonismus, die klinisch und apparativ nicht ausreichend definiert ist) [1] und anderen Diagnosen (z.B. MSA/PSP/CBD) nicht ausreichend effektiv ab.

Begründung

Es gibt gegenüber der alten Leitlinie keine neue Evidenz, die eine Wertigkeit in dieser Frage zeigen würde.

**Empfehlung**	**Neu**<br>**Stand (2023)**
Die cCT sollte nicht zur Differenzialdiagnose bei Parkinson-Syndromen eingesetzt werden. Die cCT sollte zum Ausschluss von symptomatischen Ursachen nur dann eingesetzt werden, wenn eine cMRT im individuellen Fall kontraindiziert ist.	
Konsensstärke: 91%, Konsens	

Fragestellung 13: Wie effektiv ist die Magnetresonanztomographie unter Berücksichtigung der verschiedenen Datenakquisitionstechniken (MR-Sequenzen) und der Nachbearbeitungsstrategien im Vergleich mit dem langfristigen klinischen Follow-up für die Diagnose der PK?

Ergebnis

Die kraniale MRT-Untersuchung (cMRT) bietet wertvolle diagnostische Hilfestellungen bei der Differenzialdiagnostik eines Parkinson-Syndroms, einschließlich des Ausschlusses symptomatischer Ursachen und der Abgrenzung von anderen neurodegenerativen Parkinson-Syndromen (bei hohem positivem prädiktivem Wert entsprechender MRT-Zeichen für andere neurodegenerative Parkinson-Syndrome). Die aktuellen Diagnosekriterien die PK beinhalten keine die Diagnose unterstützenden MRT-Zeichen, allerdings eröffnen sich in Zukunft mit der Entwicklung verschiedener Bildmodalitäten und Auswerteverfahren neue diagnostische und möglicherweise prognostische Anwendungen.

Bei der Untersuchung der Ausschlusskriterien für eine PK sollten im Rahmen der von den rezenten Diagnosekriterien [2] geforderten Miteinbeziehung von Untersuchungsbefunden, die für oder gegen das Vorliegen einer PK sprechen, cMRT-Aufnahmen mit standardisierten Sequenzen erfolgen. Nach einer Autorengruppe der International Parkinson Movement Disorder Society Neuroimaging Study Group sollten eine cMRT-Basisdiagnostik konventionelle MRT-Sequenzen wie T1- und T2-gewichtete Sequenzen (idealerweise hochaufgelöste 3D-Sequenzen) sowie fakultativ protonen-gewichtete

Sequenzen, eisensensitive (optimalerweise suszeptibilitätsgewichtete Sequenzen) und diffusionsgewichtete Sequenzen beinhalten [3].

T1- und T2-gewichtete Sequenzen zeigen bei der PK keine spezifischen Signalpathologien. Sie finden daher ihre Anwendung in der Differenzialdiagnose von symptomatischen Krankheitsursachen wie Mikro- bzw. Makroangiopathien, Hirntumoren, Hydrozephalus, demyelinisierenden Erkrankungen und anderen seltenen Entitäten wie z. B. Morbus Wilson, Erkrankungen aus dem Spektrum der NBIA-Erkrankungen (Neurodegeneration with Brain Iron Accumulation, Neurodegeneration mit Eisenspeicherung im Gehirn), striatopallidodentaler Kalzinose, dem Mangan-assoziierten Parkinson-Syndrom [3], [4].

Darüber hinaus kann die cMRT auch hilfreich für die Differenzialdiagnose von anderen neurodegenerativen Parkinson-Syndromen sein, indem sie frühzeitig spezifische Atrophiemuster oder Signalintensitätsveränderungen zeigt [5]. Es konnte in einer Metaanalyse gezeigt werden, dass insbesondere das Vorhandensein spezifischer Zeichen hierzu beiträgt, d.h., der positive prädiktive Wert ist hoch, der negative prädiktive Wert ist limitiert [6]. Das Vorliegen einer putaminalen Atrophie mit oder ohne putaminale Signalabschwächung in eisengewichteten Sequenzen, einer Atrophie des mittleren Kleinhirnstiels (MCP, middle cerebellar peduncle), einer pontozerebellären Atrophie, einer pontinen T2-gewichteten Veränderung in der Form einer in axialer Ausrichtung positionierten Kreuzformation (sogenanntes Semmelzeichen, engl. hot cross bun sign) sowie Diffusivitätserhöhung im Putamen oder mittleren Kleinhirnstiel bei Vorliegen eines Parkinson-Syndroms sind bei variabler Sensitivität sehr spezifisch für das Vorliegen einer MSA [7], [8] und in den rezenten MDS-Diagnosekriterien für MSA [9] als Marker für eine „klinisch etablierte MSA“ beinhaltet. Spezifische MRT-Abnormitäten wie eine Mittelhirnatrophie, die v.a. das Tegmentum betrifft (sogenanntes Kolibrizeichen, engl. humming bird sign), und eine Atrophie des oberen Kleinhirnstiels (SCP, superior cerebellar peduncle), werden mit der PSP in Verbindung gebracht. Die für eine PSP sprechenden planimetrischen Veränderungen beinhalten eine verminderte midsagittale Ratio zwischen Mittelhirn- und Ponsfläche, einen erhöhten MR-Parkinsonismus-Index (MRPI, berechnet sich aus dem Produkt der midsagittalen Ratio zwischen Pons- und Mittelhirnfläche sowie dem Quotienten aus dem MCP- und SCP- Durchmesser) oder MRPI2.0 (Produkt aus MRPI und Quotienten aus Breite des dritten Ventrikels und maximaler Abstand der Vorderhörner), welche in Metaanalysen und multizentrischen Studien eine hohe diagnostische Treffsicherheit der PSP gegenüber deren Differenzialdiagnosen inklusive PK aufweisen [10], [11], [12].

Im Folgenden wird der Stand der weiterentwickelten MRT-Sequenzen zusammengefasst, die zwar im Rahmen von Routine-cMRT-Protokollen akquiriert werden können, zumindest zum aktuellen Zeitpunkt aber nicht in der Routine für die cMRT-Bewertung bei PK eingesetzt werden, sodass dieser Absatz als **Perspektive** zu verstehen ist.

Die aktuellen Diagnosekriterien für die PK beinhalten keine die Diagnose unterstützenden MRT-Zeichen [2]. Allerdings ergaben in den letzten Jahren mit mindestens 3.0 Tesla Feldstärke durchgeführte Hochfeld-MRT-Studien wesentliche Befunde in der Substantia nigra von Betroffenen mit PK, deren diagnostische Validität in Metaanalysen bekräftigt wurde. Dazu zählen das Fehlen einer bei gesunden Personen typisch vorkommenden, dorsolateralen nigralen Hypointensität (am ehesten

dem Nigrosom 1 entsprechend, sogenanntes Schwalbenschwanz-Zeichen, engl. swallow tail sign) in eisengewichteten Sequenzen, hierbei bevorzugt in suszeptibilitätsgewichteter Bildgebung [13], [14], [15], [16], [17], und eine verminderte Ausdehnung oder ein abgeschwächtes nigrales Signal in der Neuromelanin-Bildgebung [18], [19]. Diese Befunde scheinen nach aktueller Studienlage typisch für Pat. mit der PK zu sein, kommen allerdings auch bei Pat. mit anderen neurodegenerativen Parkinson-Syndromen vor. Allerdings könnte die Auswertung extranigraler Regionen hilfreich sein [20], vor allem wenn diese Sequenzen als Teil eines multimodalen MRT-Protokolls eingesetzt werden. Unterschiedliche quantitative Marker wie quantitative eisengewichtete Sequenzen (z.B. R2*-Relaxationsrate; quantitatives Suszeptibilitäts-Mapping, QSM), wie spezielle diffusionsgewichtete Sequenzen (z.B. „free water-Imaging“; Diffusionstensorbildgebung, engl. diffusion tensor imaging, DTI einschließlich Neurite Orientation Dispersion and Density Imaging, NODDI [21], [22]; Diffusions-Kurtosis-Bildgebung, engl. diffusion kurtosis imaging, DKI), wie Protonen-MR-Spektroskopie (MRS) [23], wie Veränderungen diverser funktioneller Netzwerke in den Basalganglien, der supplementär-motorischen Area und anderen Netzwerken [24], [25], [26], wie multimodale Auswerteverfahren sowie computerbasierte MRT-Auswerteverfahren für die Diagnose der PK sind Gegenstand der aktuellen Forschung [3], [27], [28]. Für einzelne viel untersuchte Parameter wie den DTI-Parameter der regionalen fraktionalen Anisotropie in der Substantia nigra konnte aber bereits metaanalytisch gezeigt werden, dass sie keine diagnostische Wertigkeit bei PK aufweisen [29].

„Free-water-Imaging” und Neuromelanin-Bildgebung der posterioren Substantia nigra scheinen Potenzial als Progressionsmarker in frühen Stadien der PK zu haben [28]. Ob auch andere cMRT-Marker wie oben genannte quantitative Marker oder auch morphometrische oder volumetrische Marker (wie kortikale Dicke oder Gyrifikation) ein Potenzial als Progressions- oder Prognosemarker der PK haben, ist Gegenstand der aktuellen Forschung [28]. Die Klärung der Frage, ob die MRT-Methoden zur apparativen Phänotypisierung von spezifischen genetischen Parkinson-Formen beitragen können, benötigt noch eine deutlich größere Datenbasis (z.B. [30]).

**Empfehlung**	**Neu**<br>**Stand (2023)**
▪ Die kraniale MRT (cMRT) soll frühzeitig im Krankheitsverlauf zur Differenzialdiagnostik bei klinischem Parkinson-Syndrom erfolgen.<br>▪ Bei der Untersuchung der Ausschlusskriterien für eine PK sollten cMRT-Aufnahmen mit standardisierten Sequenzen erfolgen (T1- und T2-gewichtet (idealerweise hochaufgelöst 3D) sowie fakultativ eisensensitiv/suszeptibilitätsgewichtet und diffusionsgewichtet).<br>▪ Hierbei kann die cMRT nicht nur hilfreich für den Ausschluss eines symptomatischen Parkinson-Syndroms, sondern auch für die Differenzialdiagnose von anderen neurodegenerativen Parkinson-Syndromen sein, indem sie frühzeitig spezifische Atrophiemuster oder Signalintensitätsveränderungen zeigt.<br>▪ Die Dokumentation einer alternativen Ursache, die ein Parkinson-Syndrom auslösen kann und im Zusammenhang mit der Parkinson-Symptomatik steht bzw. welche in Zusammenschau aller Befunde eher das Parkinson-Syndrom erklärt als eine PK, zählt als absolutes Ausschlusskriterium für die Diagnose PK nach den neuen MDS-Diagnosekriterien.	

- *Anmerkung: Die verschiedenen Datenakquisitionstechniken und Nachbearbeitungsstrategien sind zwischen den einzelnen Zentren noch nicht vereinheitlicht; eine Homogenisierung derselben wäre wünschenswert, und Bestrebungen dahingehend gibt es bereits.*

Konsensstärke: 97%, starker Konsens

Referenzen

1. Kalra S, Grosset DG, Benamer HT. Differentiating vascular parkinsonism from idiopathic Parkinson's disease: a systematic review. Mov Disord. 2010;25(2):149-56
2. Postuma RB, Berg D, Stern M, Poewe W, Olanow CW, Oertel W, et al. MDS clinical diagnostic criteria for Parkinson's disease. Mov Disord. 2015;30(12):1591-601
3. Peralta C, Strafella AP, van Eimeren T, Ceravolo R, Seppi K, Kaasinen V, et al. Pragmatic Approach on Neuroimaging Techniques for the Differential Diagnosis of Parkinsonisms. Mov Disord Clin Pract. 2022;9(1):6-19
4. Heim B, Krismer F, De Marzi R, Seppi K. Magnetic resonance imaging for the diagnosis of Parkinson's disease. J Neural Transm (Vienna). 2017;124(8):915-64
5. Kassubek J. MRI-based neuroimaging: atypical parkinsonisms and other movement disorders. Curr Opin Neurol. 2018;31(4):425-30
6. Lee W. Conventional Magnetic Resonance Imaging in the Diagnosis of Parkinsonian Disorders: A Meta-Analysis. Mov Disord Clin Pract. 2021;8(2):217-23
7. Pellecchia MT, Stankovic I, Fanciulli A, Krismer F, Meissner WG, Palma JA, et al. Can Autonomic Testing and Imaging Contribute to the Early Diagnosis of Multiple System Atrophy? A Systematic Review and Recommendations by the Movement Disorder Society Multiple System Atrophy Study Group. Mov Disord Clin Pract. 2020;7(7):750-62
8. Lim SJ, Suh CH, Shim WH, Kim SJ. Diagnostic performance of T2* gradient echo, susceptibility-weighted imaging, and quantitative susceptibility mapping for patients with multiple system atrophy-parkinsonian type: a systematic review and meta-analysis. Eur Radiol. 2022;32(1):308-18
9. Wenning GK, Stankovic I, Vignatelli L, Fanciulli A, Calandra-Buonaura G, Seppi K, et al. The Movement Disorder Society Criteria for the Diagnosis of Multiple System Atrophy. Mov Disord. 2022;37(6):1131-48
10. Zhang K, Liang Z, Wang C, Zhang X, Yu B, Liu X. Diagnostic validity of magnetic resonance parkinsonism index in differentiating patients with progressive supranuclear palsy from patients with Parkinson's disease. Parkinsonism Relat Disord. 2019;66:176-81
11. Quattrone A, Bianco MG, Antonini A, Vaillancourt DE, Seppi K, Ceravolo R, et al. Development and Validation of Automated Magnetic Resonance Parkinsonism Index 2.0 to Distinguish Progressive Supranuclear Palsy-Parkinsonism From Parkinson's Disease. Mov Disord. 2022;37(6):1272-81
12. Nigro S, Antonini A, Vaillancourt DE, Seppi K, Ceravolo R, Strafella AP, et al. Automated MRI Classification in Progressive Supranuclear Palsy: A Large International Cohort Study. Mov Disord. 2020;35(6):976-83
13. Mahlknecht P, Krismer F, Poewe W, Seppi K. Meta-analysis of dorsolateral nigral hyperintensity on magnetic resonance imaging as a marker for Parkinson's disease. Mov Disord. 2017;32(4):619-23
14. Kim PH, Lee DH, Suh CH, Kim M, Shim WH, Kim SJ. Diagnostic performance of loss of nigral hyperintensity on susceptibility-weighted imaging in parkinsonism: an updated meta-analysis. Eur Radiol. 2021;31(8):6342-52
15. Cho SJ, Bae YJ, Kim JM, Kim HJ, Baik SH, Sunwoo L, et al. Iron-sensitive magnetic resonance imaging in Parkinson's disease: a systematic review and meta-analysis. J Neurol. 2021;268(12):4721-36

16. Chau MT, Todd G, Wilcox R, Agzarian M, Bezak E. Diagnostic accuracy of the appearance of Nigrosome-1 on magnetic resonance imaging in Parkinson's disease: A systematic review and meta-analysis. Parkinsonism Relat Disord. 2020;78:12-20
17. Pyatigorskaya N, Sanz-Morère CB, Gaurav R, Biondetti E, Valabregue R, Santin M, et al. Iron Imaging as a Diagnostic Tool for Parkinson's Disease: A Systematic Review and Meta-Analysis. Front Neurol. 2020;11:366
18. Cho SJ, Bae YJ, Kim JM, Kim D, Baik SH, Sunwoo L, et al. Diagnostic performance of neuromelanin-sensitive magnetic resonance imaging for patients with Parkinson's disease and factor analysis for its heterogeneity: a systematic review and meta-analysis. Eur Radiol. 2021;31(3):1268-80
19. Wang X, Zhang Y, Zhu C, Li G, Kang J, Chen F, Yang L. The diagnostic value of SNpc using NM-MRI in Parkinson's disease: meta-analysis. Neurol Sci. 2019;40(12):2479-89
20. Wang JY, Zhuang QQ, Zhu LB, Zhu H, Li T, Li R, et al. Meta-analysis of brain iron levels of Parkinson's disease patients determined by postmortem and MRI measurements. Sci Rep. 2016;6:36669
21. Atkinson-Clement C, Pinto S, Eusebio A, Coulon O. Diffusion tensor imaging in Parkinson's disease: Review and meta-analysis. Neuroimage Clin. 2017;16:98-110
22. Suo X, Lei D, Li W, Li L, Dai J, Wang S, et al. Altered white matter microarchitecture in Parkinson's disease: a voxel-based meta-analysis of diffusion tensor imaging studies. Front Med. 2021;15(1):125-38
23. Gu W, He C, Chen J, Li J. Proton Magnetic Resonance Spectroscopy for the Early Diagnosis of Parkinson Disease in the Substantia Nigra and Globus Pallidus: A Meta-Analysis With Trial Sequential Analysis. Front Neurol. 2022;13:838230
24. Tang S, Wang Y, Liu Y, Chau SW, Chan JW, Chu WC, et al. Large-scale network dysfunction in α-Synucleinopathy: A meta-analysis of resting-state functional connectivity. EBioMedicine. 2022;77:103915
25. Joshi D, Prasad S, Saini J, Ingalhalikar M. Role of Arterial Spin Labeling (ASL) Images in Parkinson's Disease (PD): A Systematic Review. Acad Radiol. 2023;30(8):1695-708
26. Shih YC, Tseng WI, Montaser-Kouhsari L. Recent advances in using diffusion tensor imaging to study white matter alterations in Parkinson's disease: A mini review. Front Aging Neurosci. 2022;14:1018017
27. Ravanfar P, Loi SM, Syeda WT, Van Rheenen TE, Bush AI, Desmond P, et al. Systematic Review: Quantitative Susceptibility Mapping (QSM) of Brain Iron Profile in Neurodegenerative Diseases. Front Neurosci. 2021;15:618435
28. Mitchell T, Lehéricy S, Chiu SY, Strafella AP, Stoessl AJ, Vaillancourt DE. Emerging Neuroimaging Biomarkers Across Disease Stage in Parkinson Disease: A Review. JAMA Neurol. 2021;78(10):1262-72
29. Hirata FCC, Sato JR, Vieira G, Lucato LT, Leite CC, Bor-Seng-Shu E, et al. Substantia nigra fractional anisotropy is not a diagnostic biomarker of Parkinson's disease: A diagnostic performance study and meta-analysis. Eur Radiol. 2017;27(6):2640-8
30. Filippi M, Balestrino R, Basaia S, Agosta F. Neuroimaging in Glucocerebrosidase-Associated Parkinsonism: A Systematic Review. Mov Disord. 2022;37(7):1375-93

2.3 Hirnparenchymsonographie

Autoren: Uwe Walter, Kai Loewenbrück

Fragestellung 14: Wie effektiv ist die Hirnparenchymsonographie in der Differenzialdiagnose der PK versus atypische und sekundäre Parkinson-Syndrome?

Hintergrund

Besonders in den frühen motorischen Krankheitsstadien ist die diagnostische Abgrenzung der PK von atypischen und sekundären Parkinson-Syndromen nicht immer anhand klinischer Kriterien möglich. Daher besteht Bedarf an zusatzdiagnostischen Verfahren zur Erhöhung der Diagnosesicherheit.

Evidenzgrundlage

In dieser Literaturrecherche fanden sich keine Studien mit histologischer Diagnosebestätigung, eine longitudinale größere Kohortenstudie und ein systematischer Review mit Metaanalyse, 9 Querschnittsstudien, 2 kleinere longitudinale Kohortenstudien und 2 Übersichtsarbeiten.

Ergebnis

Die transkranielle B-Bild-Sonographie (TCS) des Hirnparenchyms kann die diagnostische Abgrenzung der PK von atypischen und sekundären Parkinson-Syndromen in den ersten Jahren der Erkrankung unterstützen, da schon in dieser Phase die TCS-Befunde spezifisch sind [1]. In dieser Anwendung ist die Reliabilität der TCS von der Untersucherqualifikation abhängig [2], [3], daher soll die TCS durch einen gut ausgebildeten Untersucher/eine gut ausgebildete Untersucherin erfolgen. Wenn mittels TCS nur die Echogenität der Substantia nigra (SN) beurteilt wird, lässt sich die PK (i.d.R hyperechogene SN (SN+)) vor allem von der Multisystematrophie (MSA, i.d.R. normal echogene SN (SN-)) abgrenzen [4], [5]. Wenn auch andere atypische Parkinson-Syndrome in Betracht kommen (supranukleäre Blickparese, PSP, kortikobasale Degeneration, CBD), ist die alleinige TCS der SN weniger aussagekräftig, da v.a. bei der CBD häufig eine SN+ vorliegt [6], [5]. Eine Metaanalyse von 71 Studien bei über 5.000 Pat. ergab eine nur 75%ige Sensitivität und eine 70%ige Spezifität der SN-TCS in der Abgrenzung der PK von atypischen Parkinson-Syndromen [7]. Mehrere longitudinale und Querschnittsstudien haben hingegen gezeigt, dass die zusätzliche Beurteilung der Echogenität des Nucleus lentiformis (NL) sowie der Weite des 3. Ventrikels die diagnostische Validität erhöht [8], [9], [1], [10], [11], [12], [13] (Tab. 2). Die Befundkombination aus hyperechogenem NL (NL+) mit mindestens einem weiteren der 2 Befunde (SN- oder Dilatation des 3. Ventrikels > 10 mm) grenzt MSA und PSP am besten von der PK ab [9]. Zur Unterscheidung zwischen PK und Demenz mit Lewy-Körperchen (DLK) ist nur die TCS der SN richtungweisend, die zusätzliche Beurteilung der Basalganglien verbessert die Diskrimination nicht [14], [12]. Charakteristisch für die DLK ist eine bilateral-symmetrische SN+ (Asymmetrie-Index <1,15; ermittelt durch Division des größeren durch den kleineren der beidseitig erhobenen Messwerte). Allerdings ist die Spezifität dieses Befunds für die DLK limitiert (Tab. 2). Bei vaskulärem Parkinsonismus (VP) findet sich meist eine normale SN-Echogenität (SN-) [15], [16], [17]. Es wurde diskutiert, dass VP-Pat. mit SN+ möglicherweise doch eine, ggf. latente, PK haben [16], dies ist allerdings nicht systematisch untersucht worden. Die TCS

ermöglicht keine vollständige Diskrimination der Parkinson-Syndrome, sodass klinischer Verlauf und weitere zusatzdiagnostische Verfahren in die Diagnosefindung einbezogen werden müssen.

Tab. 2: Studien zur Hirnparenchymsonographie in der Differenzialdiagnose der PK versus atypische und sekundäre Parkinson-Syndrome

Studie	Vergleichsgruppen	Diagnostischer Goldstandard	TCS-Kriterium	Sensitivität, Spezifität PPV; LR+; LR-	Studienqualität, Kommentar
**PK versus atypische Parkinson-Syndrome (MSA, PSP, CBD)**					
[4]	PK (n=25) vs. MSA/PSP (n=23)	klin. Diagnose durch unabh. Experten	SN+ (moderat oder deutlich, uni- oder bilateral)	96%, 91%, 92%; 11,0; 0,04	niedrig (kleine Pat.-Zahl, auch mögliche PSP inkludiert)
[8]	MSA/PSP (n=40) vs PK (n=88)	klin. Diagnose durch unabh. Experten	NL+, kombiniert mit SN-	70%, 99%, 97%; 61,6; 0,30	moderat (retrospektives Design, auch mögliche PSP bzw. MSA inkludiert)
[9]	MSA/PSP (n=40) vs. PK (n=134)	klin. Diagnose durch unabh. Experten	SN-	72%, 98%, 91%; 32,4; 0,28	moderat (kein Follow-up, auch mögliche PSP bzw. MSA inkludiert)
	MSA/PSP (n=39) vs. PK (n=125)	dto.	NL+, kombiniert mit SN-	59%, 100%, 100%	dto.
	MSA/PSP (n=39) vs. PK (n=125)	dto.	NL+, kombiniert mit mindestens einem: SN- o. 3V+	82%, 98%, 94%; 51,3; 0,18	dto.
[5]	PK (n=43) vs. MSA/PSP/CBD (n=13)	klin. Diagnose im 12-Monats-Follow-up	SN+ (moderat oder deutlich, uni- oder bilateral)	91%, 85%, 95%; 5,89; 0,11	Moderat (kleine Anzahl von Pat. mit atyp. PS)
**[1]**	PK (n=353) vs. MSA/PSP (n=147) (gepoolte Daten von 5 Studien)	klin. Diagnose durch unabh. Experten	SN+ (moderat oder deutlich, uni- oder bilateral)	92%, 80%, 91%; 4,50; 0,10	Moderat (kein bzw. nur kurzes Follow-up, Grad der Verblindung uneinheitlich)
	MSA/PSP (n=79) vs. PK (n=213) (gepoolte Daten von 2 Studien)	dto.	NL+, kombiniert mit SN-	65%, 99%, 98%; 137,5; 0,36	dto.
**[10]**	MSA/PSP/CBD (n=17) vs. PK (n=13)	klin. Diagnose im 9-Monats-Follow-up	NL+	82%, 85%, 87%; 5,35; 0,21	niedrig, da kleine Pat.-Zahl, Grad der Verblindung unsicher

Studie	Vergleichsgruppen	Diagnostischer Goldstandard	TCS-Kriterium	Sensitivität, Spezifität PPV; LR+; LR-	Studienqualität, Kommentar
**[11]**	PK (n=22) vs. MSA (n=21)	klin. Diagnose durch unabh. Experten	SN+ (moderat oder deutlich, uni- oder bilateral)	86%, 76%, 79%; 3,63; 0,18	moderat (kein Follow-up, Grad der Verblindung unsicher)
	PK (n=22) vs. MSA (n=21)	dto.	NL-, kombiniert mit SN+ (deutlich, uni- oder bilateral)	68%, 95%, 94%; 14,3; 0,33	dto.
**[7]**	PK (n=4494) vs. MSA/PSP/CBD (n=594) (Metaanalyse von 71 Studien)	klin. Diagnose durch unabh. Experten	SN+	75% (95% KI: 60–86%), 70% (95% KI: 55–81%)	hoch (systemat. Review/ Metaanalyse)
**[12]**	MSA/PSP (n=92) vs. PK (n=121)	klin. Diagnose im 4-Jahres-Follow-up	NL+, kombiniert mit SN- oder milder SN+	88%, 97%, 96%; 35,5; 0,12	moderat (keine Definition von „milder“ SN+)
[13]	PK (n=156) vs. MSA/PSP/CBD (n=113)	klin. Diagnose im letzten Follow-up	NL-, kombiniert mit SN+ (deutlich, uni- oder bilateral)	75%, 83%, 86%; 4,46; 0,30	moderat (retrospekt. Design, keine Angabe zur Follow-up-Dauer)
	MSA/PSP (n=178) vs. PK (n=369) (gepoolte Daten von 3 Studien)	klin. Diagnose durch unabh. Experten	NL+, kombiniert mit SN-	52%, 99%, 96%; 48,2; 0,48	moderat (kein bzw. nur kurzes Follow-up, z.T. retrospekt. Design)
**PK versus Demenz mit Lewy-Körperchen (DLK)**					
[14]	PK (n=94) vs. DLK (n=12)	klin. Diagnose durch unabh. Experten	SN- oder asymmetrische SN+ (Ratio ≥1.15)	72%, 83%, 97%; 4,34; 0,33	niedrig (kleine Anzahl von DLK-Pat.)
**[12]**	PK (n=121) vs. DLK (n=20)	klin. Diagnose im 4-Jahres-Follow-up	SN- oder asymmetrische SN+	100%, 65%, 94%; 2,86; 0	niedrig (kleine Anzahl von DLK-Pat., „asymmetrische“ SN+ nicht defin.)
**PK versus vaskulärer Parkinsonismus (VP)**					
[15]	PK (n=80) vs. VP (n=30)	klin. Diagnose durch unabh. Experten	SN+	84%, 80%, 92%; 41,9; 0,20	moderat (kein Follow-up)
[16]	PK (n=78) vs. VP (n=12)	klin. Diagnose durch unabh. Experten	SN+	83%, 92%, 98%; 10,0; 0,18	niedrig (kleine Anzahl von VP-Pat.; Verblindung unsicher)

Studie	Vergleichsgruppen	Diagnostischer Goldstandard	TCS-Kriterium	Sensitivität, Spezifität PPV; LR+; LR-	Studienqualität, Kommentar
[17]	PK (n=372) vs. VP (n=48) [Derivationskohorte]	klin. Diagnose durch unabh. Experten	SN+	90%, 58%, 94%; 2,15; 0,18	moderat (retrospektives Design)
	PK (n=68) vs. VP (n=15) [Validationskohorte]	klin. Diagnose durch unabh. Experten, 12-Monats-Follow-up	SN+	94%, 67%, 93%; 2,82; 0,09	niedrig (kleine Anzahl von VP-Patienten)

KI = Konfidenzintervall, LR+ = positive Likelihood Ratio, LR- = negative Likelihood Ratio, NL+ = Hyperechogenität des Nucleus lentiformis (ein- oder beidseitig), NL- = normale Echogenität des Nucleus lentiformis (beidseitig), PPV = positiver prädiktiver Wert, SN+ = Hyperechogenität der Substantia nigra (ein- oder beidseitig), SN- = normale Echogenität der Substantia nigra (beidseitig)

Begründung der Empfehlung

Die Evidenz zur TCS der Substantia nigra (SN) zur Abgrenzung der PK von atypischen Parkinson-Syndromen ist bislang am größten und wurde in einem qualitativ guten systematischen Review mit Metaanalyse bewertet (75%ige Sensitivität, 70%ige Spezifität) [7]. Die Evidenz zur TCS mit kombinierter Beurteilung von SN, Nucleus lentiformis (NL) und z.T. 3. Ventrikel beruht auf einer größeren longitudinalen Studie sowie 7 Querschnitts-, Fallkontroll- bzw. kleineren longitudinalen Kohortenstudien mit variabler Qualität des Studiendesigns sowie 2 Übersichtsarbeiten mit Analyse gepoolter Daten mehrerer dieser Studien. Die Studienlage zur TCS bei DLK sowie bei VP ist begrenzt (insgesamt 5 Studien mit rel. Kleiner Fallzahl). In einem aktuellen Positionspapier der zuständigen Fachgesellschaften (Deutsche Gesellschaft für Ultraschall in der Medizin – DEGUM, Deutsche Gesellschaft für Klinische Neurophysiologie und Funktionelle Bildgebung – DGKN) wird aufgezeigt, wie der Status eines qualifizierten Untersuchers/einer qualifizierten Untersucherin für die „TCS in der Parkinson-Diagnostik“ im kurrikularen Ausbildungskonzept der DEGUM/DGKN erworben wird [18].

Empfehlung	Neu<br>Stand (2023)
▪ Die transkranielle Hirnparenchymsonographie (TCS) durch einen qualifizierten Untersucher/eine qualifizierte Untersucherin kann zur Unterstützung der Differenzialdiagnose der PK versus atypische und sekundäre Parkinson-Syndrome erwogen werden.<br>▪ Die TCS zur Differenzialdiagnose der PK versus atypische Parkinson-Syndrome soll die Beurteilung von Substantia nigra, Nucleus lentiformis sowie 3. Ventrikel einbeziehen.	
Konsensstärke: 97,4%, starker Konsens	

Fragestellung 15: Wie effektiv ist die Hirnparenchymsonographie in der Differenzialdiagnose der PK versus essenzieller Tremor?

Hintergrund

Die Unterscheidung zwischen essenziellem Tremor und beginnender tremordominanter PK ist nicht immer anhand der klinischen Untersuchung möglich. Daher besteht Bedarf an zusatzdiagnostischen Verfahren zur Erhöhung der Diagnosesicherheit.

Evidenzgrundlage

In dieser Literaturrecherche fanden sich keine Studien mit histologischer Diagnosebestätigung, ein qualitativ hochwertiger systematischer Review mit Metaanalyse der bis dato publizierten relevanten Studien zur alleinigen Hirnparenchymsonographie und 3 Querschnittsstudien zur Kombination von Hirnparenchymsonographie und olfaktorischer Testung.

Ergebnis

Ein systematischer Review mit Metaanalyse fand 18 geeignete Studien zur transkraniellen B-Bild-Sonographie (TCS) des Hirnparenchyms bei insgesamt 1.264 Parkinson-Pat. und 824 Pat. mit essenziellem Tremor [19]. Die Metaanalyse ergab eine 85%ige Sensitivität (95% KI, 79.4–88.6%) und 84%ige Spezifität (95% KI, 78.4–88.2%) der TCS in der Differenzialdiagnose der PK versus essenzieller Tremor. Zudem konnte in einer Subgruppenanalyse von 3 der 18 Studien gezeigt werden, dass die TCS eine ähnliche diagnostische Sensitivität und Spezifität wie der DATScan aufweist. Dabei ist die TCS der Substantia nigra (SN) ausreichend, da die TCS weiterer Hirnstrukturen in der Abgrenzung des essenziellen Tremors keinen Zusatznutzen erbringt [20]. Mittels Kombination von TCS und Screening auf Hyposmie (Sniffin' Sticks) wird eine höhere diagnostische Spezifität erreicht [17], [21], [22] (Tab. 3). Es gibt Hinweise darauf, dass ET-Pat. mit einer hyperechogenen SN (SN+) ein erhöhtes Risiko einer späteren PK haben [23]. Die TCS der SN erfordert einen gut ausgebildeten Untersucher/eine gut ausgebildete Untersucherin [2], [3].

Tab. 3: Studien zur Hirnparenchymsonographie in der Differenzialdiagnose der PK versus essenzieller Tremor

Studie	Vergleichsgruppen	Diagnostischer Goldstandard	TCS-Kriterium	Sensitivität, Spezifität PPV; LR+; LR-	Studienqualität, Kommentar
**PK (PK) versus essenzieller Tremor (ET): nur TCS**					
[19]	PK (n=1264) vs. ET (n=824) (Metaanalyse von 18 Studien)	klin. Diagnose durch unabh. Experten, teils DATScan	SN+ (mindestens einseitig)	85%, 84%, (86%); 4,11; 0,22	hoch (systematischer Review, große Pat.zahl)
**PK (PK) versus essenzieller Tremor (ET): Kombination von TCS und Riechtestung**					
[17]	PK (n=372) vs. ET (n=20) [Derivationskohorte]	klin. Diagnose durch unabh. Experten	SN+, kombiniert mit Hyposmie (Sniffin' Sticks)	66%, 100%, 100%	moderat (retrospektives Design)
	PK (n=68) vs. ET (n=15) [Validationskohorte]	klin. Diagnose durch unabh. Experten, 12-Monats-Follow-up	SN+, kombiniert mit Hyposmie (Sniffin' Sticks)	53%, 87%, 95%; 3,97; 0,54	niedrig (kleine Anzahl von ET-Pat.)

Studie	Vergleichsgruppen	Diagnostischer Goldstandard	TCS-Kriterium	Sensitivität, Spezifität PPV; LR+; LR-	Studienqualität, Kommentar
[21]	PK (n=37) vs. ET (n=26)	klin. Diagnose durch unabh. Experten	SN+, kombiniert mit Hyposmie (Sniffin' Sticks)	30%, 100%, 100%	moderat (rel. kleine Pat.zahl)
[22]	PK (n=30) vs. ET (n=21)	klin. Diagnose durch unabh. Experten	SN+, kombiniert mit Hyposmie (Sniffin' Sticks)	63%, 100%, 100%	niedrig (kleine Anzahl von ET-Patienten; Verblindung unsicher)

LR+ = positive Likelihood Ratio, LR- = negative Likelihood Ratio, PPV = positiver prädiktiver Wert, PSG = Polysomnographie, SN+ = Hyperechogenität der Substantia nigra (ein- oder beidseitig)

Begründung der Empfehlung

Die Evidenz zur TCS der Substantia nigra (SN) zur Abgrenzung der PK vom essenziellen Tremor basiert auf einem qualitativ guten systematischen Review mit Metaanalyse von 18 guten bis sehr guten Studien. Die diagnostische Wertigkeit der TCS ist mit einer 85%igen Sensitivität und einer 84%igen Spezifität ähnlich wie die des DATSCAN. Die Evidenz zur TCS in Kombination mit einem Screening-Test auf Hyposmie beruht auf 3 kleineren Querschnitts- bzw. Kohortenstudien. In einem aktuellen Positionspapier der zuständigen Fachgesellschaften (Deutsche Gesellschaft für Ultraschall in der Medizin – DEGUM, Deutsche Gesellschaft für Klinische Neurophysiologie und Funktionelle Bildgebung – DGKN) wird aufgezeigt, wie der Status eines qualifizierten Untersuchers für die „TCS in der Parkinson-Diagnostik" im kurrikularen Ausbildungskonzept der DEGUM/DGKN erworben wird [18].

Empfehlung	Neu<br>Stand (2023)
▪ Die TCS durch einen qualifizierten Untersucher/eine qualifizierte Untersucherin kann zur Unterstützung der Differenzialdiagnose der PK versus essenzieller Tremor erwogen werden.<br>▪ Die TCS zur Differenzialdiagnose der PK versus essenzieller Tremor kann zur Erhöhung der diagnostischen Sicherheit mit einem Screening-Test auf Hyposmie verbunden werden.	
Konsensstärke: 97,1%, starker Konsens	

Fragestellung 16: Wie effektiv ist die Hirnparenchymsonographie im Vergleich mit dem klinischen Follow-up für die Diagnose der PK bei Personen mit typischen Frühsymptomen*?

(*motorische Zeichen eines Parkinson-Syndroms, Hyposmie, Depression, REM-Schlaf-Verhaltensstörung)

Hintergrund

Die Diagnose einer inzidenten PK kann den frühzeitigen Beginn aufkommender Therapien zur Neuroprotektion/Neurorestauration ermöglichen. Bislang besteht keine Möglichkeit der Diagnosestellung durch ein einzelnes diagnostisches Verfahren. Daher besteht Bedarf an zusatzdiagnostischen Verfahren zur Erhöhung der Diagnosesicherheit, insbesondere bei Personen mit Frühsymptomen.

Evidenzgrundlage

In dieser Literaturrecherche fanden sich keine Studien mit histologischer Diagnosebestätigung, eine longitudinale Studie einer größeren deutschen Kohorte von Risikopersonen und eine gepoolte Datenanalyse von 5 deutschen longitudinalen Studien von z.T. großen Risikokohorten sowie 3 kleine longitudinale Beobachtungsstudien von Risikokohorten.

Ergebnis

Die transkranielle B-Bild-Sonographie (TCS) des Hirnparenchyms zeigt (je nach Cut-Off-Wert) bei 9–22% (im Mittel 13%) der gesunden erwachsenen Normalpopulation eine moderat bis deutlich hyperechogene Substantia nigra (SN+), wie sie bei 75–90% (im Mittel: 83%) der Parkinson-Pat. vorliegt [24]. Die TCS der SN erfordert einen gut ausgebildeten Untersucher/eine gut ausgebildete Untersucherin [2], [3]. Die SN+ ist bei über 50-jährigen Gesunden mit einem bis zu 20-fach erhöhten Risiko der Entwicklung einer PK innerhalb von 5 Jahren assoziiert, allerdings ist der positive prädiktive Wert mit ca. 6% gering [25]. Longitudinale Studien in Populationen mit einem epidemiologisch erhöhten Parkinson-Risiko (Vorhandensein milder motorischer Zeichen eines Parkinson-Syndroms, Hyposmie, Depression, REM-Schlaf-Verhaltensstörung) haben teilweise höhere positive prädiktive Werte der SN+ für eine inzidente PK gezeigt [26], [27], [28], [29] (Tab. 4). Eine kombinierte Analyse von 5 deutschen Follow-up-Studien in Risikokohorten legt nahe, dass die SN+ als Risikomarker einer PK bei unter 65-Jährigen und bei Frauen stärker zu gewichten ist [30]. Bei polysomnographisch bestätigter REM-Schlaf-Verhaltensstörung wurden vergleichsweise hohe positive prädiktive Werte (um 50%) der SN+ für die nachfolgende Entwicklung einer PK oder einer Demenz mit Lewy-Körperchen (DLK) gefunden [27], [29]. Der Vorhersagewert in Risikokohorten kann weiter gesteigert werden, wenn die TCS der SN mit einem Screening auf Hyposmie (z.B. mittels Sniffin' Sticks) verbunden wird [26], [29]. Heute wird zur Prädiktion der PK in Risikopopulationen die Prüfung/Bewertung einer ganzen Reihe von Risiko-markern (z.B. TCS der SN) und prodromalen markern im Verbund empfohlen, um die individuelle Vorhersagekraft zu verbessern [31], [32]. Es ist zu erwarten, dass künftig eine präzisere Vorhersage einer inzidenten PK durch die zusätzliche Einbeziehung laborchemischer und genetischer Befunde möglich wird [33].

Tab. 4: Studien zur Hirnparenchymsonographie im Vergleich mit dem klinischen Follow-up für die Diagnose der PK bei Personen mit typischen Frühsymptomen

Studie	Vergleichsgruppen	Diagnostischer Goldstandard	TCS-Kriterium	Sensitivität, Spezifität PPV; LR+; LR-	Studienqualität, Kommentar
[26]	46 Pat. mit aktueller schwerer Depression (Baseline)	klinische Diagnose PK gemäß MDS-Kriterien im 10-Jahres-Follow-up (n=3)	SN+	100%, 67%, 17%; 3,07; 0	moderat (niedrige Fallzahl von inzidentem PK)
	Dto.	dto.	SN+, kombiniert mit Hyposmie (Sniffin' Sticks) und milden motor. PS-Symptomen	100%, 98%, 75%; 46,0; 0	dto.
[27]	49 Pat. mit iRBD (bestätigt mit PSG)	‚linische Diagnose PK oder DLK gemäß MDS-Kriterien/ McKeith-Kriterien im 6-Jahres-Follow-up (n=20, davon 18 mit TCS)	SN+	44%, 68%, 44%; 1,38; 0,82	moderat (keine genaue Angabe des verwendeten Ultraschall-Systems)
[28]	650 Teilnehmende (davon 579 mit TCS), von diesen ca. 70% (Baseline) mit Hyposmie und/oder iRBD und/oder Depression	klinische Diagnose PK gemäß MDS-Kriterien im 6-Jahres-Follow-up (n=10, davon 9 mit TCS)	SN+	78%, 79%, 6%; 3,79; 0,28	hoch (prospektive Studie mit rel. großer Fallzahl)
[30]	2543 Teilnehmende (davon 2121 mit TCS), von diesen ca. 50% (Baseline) mit milden motorischen PS-Symptomen und/oder Hyposmie und/oder iRBD und/oder Depression	klinische Diagnose PK oder DLK gemäß MDS-Kriterien/ McKeith-Kriterien im 6-Jahres-Follow-up (n=34, davon 30 mit TCS)	SN+	70%, 78%, 4%; 3,17; 0,39	hoch (kombinierte Analyse von 5 prospektiven Follow-up-Studien an ausgewiesenen Zentren)
[29]	34 Pat. mit iRBD (bestätigt mit PSG)	klinische Diagnose PK oder DLK gemäß MDS-Kriterien / McKeith-Kriterien im Follow-up bis 11 Jahre (n=13)	SN+	61%, 71%, 57%; 2,15; 0,54	moderat (rel. kleine Kohorte)
	dto.	dto.	SN+, kombiniert mit Hyposmie (OSIT-J)	31%, 100%, 100%	dto.

iRBD = idiopathische REM-Schlaf-Verhaltensstörung, LR+ = positive Likelihood Ratio, LR- = negative Likelihood Ratio, MDS = International Parkinson and Movement Disorder Society, OSIT-J = japanische Version des Odor Stick Identification Test, PPV = positiver prädiktiver Wert, PSG = Polysomnographie, SN+ = Hyperechogenität der Substantia nigra (ein- oder beidseitig)

Begründung der Empfehlung

Die Evidenz zur Hirnparenchymsonographie im Vergleich mit dem klinischen Follow-up für die Diagnose der PK bei Personen mit typischen Frühsymptomen basiert auf einer Studie mit gepoolter Datenanalyse von 5 deutschen Follow-up-Studien in 3 großen und 2 kleinen Kohorten sowie – außerhalb dieser gepoolten Analyse – weiteren 2 kleinen longitudinalen Beobachtungsstudien in Risikokohorten mit etwa 10-jährigem Follow-up. In einem aktuellen Positionspapier der zuständigen Fachgesellschaften (Deutsche Gesellschaft für Ultraschall in der Medizin – DEGUM, Deutsche Gesellschaft für Klinische Neurophysiologie und Funktionelle Bildgebung – DGKN) wird aufgezeigt, wie der Status eines qualifizierten Untersuchers/einer qualifizierten Untersucherin für die „TCS in der Parkinson-Diagnostik“ im kurrikularen Ausbildungskonzept der DEGUM/DGKN erworben wird [18].

**Empfehlung**	**Neu**<br>**Stand (2023)**
▪ Die transkranielle Hirnparenchymsonographie (TCS) durch einen qualifizierten Untersucher/eine qualifizierte Untersucherin bei Personen mit typischen Frühsymptomen kann ein erhöhtes Risiko einer PK aufzeigen, allerdings ist der prädiktive Wert der alleinigen TCS im Individuum begrenzt.<br>▪ Bei Personen oberhalb des 50. Lebensjahrs mit polysomnographisch gesicherter isolierter REM-Schlaf-Verhaltensstörung sollte eine in der TCS nachgewiesene Hyperechogenität der Substania nigra in Kombination mit einer nachgewiesenen Hyposmie als Hinweis auf Vorliegen einer PK gewertet werden.	
Konsensstärke: 97%, starker Konsens	

Referenzen

1. Berg D, Godau J, Walter U. Transcranial sonography in movement disorders. Lancet Neurol. 2008;7(11):1044-55
2. Skoloudík D, Fadrná T, Bártová P, Langová K, Ressner P, Zapletalová O, et al. Reproducibility of sonographic measurement of the substantia nigra. Ultrasound Med Biol. 2007;33(9):1347-52
3. van de Loo S, Walter U, Behnke S, Hagenah J, Lorenz M, Sitzer M, et al. Reproducibility and diagnostic accuracy of substantia nigra sonography for the diagnosis of Parkinson's disease. J Neurol Neurosurg Psychiatry. 2010;81(10):1087-92
4. Walter U, Niehaus L, Probst T, Benecke R, Meyer BU, Dressler D. Brain parenchyma sonography discriminates Parkinson's disease and atypical parkinsonian syndromes. Neurology. 2003;60(1):74-7
5. Gaenslen A, Unmuth B, Godau J, Liepelt I, Di Santo A, Schweitzer KJ, et al. The specificity and sensitivity of transcranial ultrasound in the differential diagnosis of Parkinson's disease: a prospective blinded study. Lancet Neurol. 2008;7(5):417-24

6. Walter U, Dressler D, Wolters A, Probst T, Grossmann A, Benecke R. Sonographic discrimination of corticobasal degeneration vs progressive supranuclear palsy. Neurology. 2004;63(3):504-9
7. Shafieesabet A, Fereshtehnejad SM, Shafieesabet A, Delbari A, Baradaran HR, Postuma RB, Lökk J. Hyperechogenicity of substantia nigra for differential diagnosis of Parkinson's disease: A meta-analysis. Parkinsonism Relat Disord. 2017;42:1-11
8. Behnke S, Berg D, Naumann M, Becker G. Differentiation of Parkinson's disease and atypical parkinsonian syndromes by transcranial ultrasound. J Neurol Neurosurg Psychiatry. 2005;76(3):423-5
9. Walter U, Dressler D, Probst T, Wolters A, Abu-Mugheisib M, Wittstock M, Benecke R. Transcranial brain sonography findings in discriminating between parkinsonism and idiopathic Parkinson disease. Arch Neurol. 2007;64(11):1635-40
10. Hellwig S, Reinhard M, Amtage F, Guschlbauer B, Buchert R, Tüscher O, et al. Transcranial sonography and [18F]fluorodeoxyglucose positron emission tomography for the differential diagnosis of parkinsonism: a head-to-head comparison. Eur J Neurol. 2014;21(6):860-6
11. Li X, Xue S, Jia S, Zhou Z, Qiao Y, Hou C, et al. Transcranial sonography in idiopathic REM sleep behavior disorder and multiple system atrophy. Psychiatry Clin Neurosci. 2017;71(4):238-46
12. Monaco D, Berg D, Thomas A, Di Stefano V, Barbone F, Vitale M, et al. The predictive power of transcranial sonography in movement disorders: a longitudinal cohort study. Neurol Sci. 2018;39(11):1887-94
13. Alonso-Canovas A, Tembl Ferrairó JI, Martínez-Torres I, Lopez-Sendon Moreno JL, Parees-Moreno I, Monreal-Laguillo E, et al. Transcranial sonography in atypical parkinsonism: How reliable is it in real clinical practice? A multicentre comprehensive study. Parkinsonism Relat Disord. 2019;68:40-5
14. Walter U, Dressler D, Wolters A, Wittstock M, Greim B, Benecke R. Sonographic discrimination of dementia with Lewy bodies and Parkinson's disease with dementia. J Neurol. 2006;253(4):448-54
15. Tsai CF, Wu RM, Huang YW, Chen LL, Yip PK, Jeng JS. Transcranial color-coded sonography helps differentiation between idiopathic Parkinson's disease and vascular parkinsonism. J Neurol. 2007;254(4):501-7
16. Venegas-Francke P. Transcranial sonography in the discrimination of Parkinson's disease versus vascular parkinsonism. Int Rev Neurobiol. 2010;90:147-56
17. Busse K, Heilmann R, Kleinschmidt S, Abu-Mugheisib M, Höppner J, Wunderlich C, et al. Value of combined midbrain sonography, olfactory and motor function assessment in the differential diagnosis of early Parkinson's disease. J Neurol Neurosurg Psychiatry. 2012;83(4):441-7
18. Walter U, Berg D, Behnke S, Schminke U, Ertl M, Witte OW, Krogias C. Positionspapier der DEGUM, Sektion Neurologie, und der DGKN zur Untersucherqualifikation für die transkranielle B-Bild-Sonografie (TCS) des Gehirns in der Diagnostik der Parkinson-Krankheit. Klin Neurophysiol. 2023; DOI: 10.1055/a-2150-4468
19. Heim B, Peball M, Hammermeister J, Djamshidian A, Krismer F, Seppi K. Differentiating Parkinson's Disease from Essential Tremor Using Transcranial Sonography: A Systematic Review and Meta-Analysis. J Parkinsons Dis. 2022;12(4):1115-23
20. Alonso-Cánovas A, López-Sendón JL, Buisán J, deFelipe-Mimbrera A, Guillán M, García-Barragán N, et al. Sonography for diagnosis of Parkinson disease-from theory to practice: a study on 300 participants. J Ultrasound Med. 2014;33(12):2069-74
21. Chen W, Tan YY, Hu YY, Zhan WW, Wu L, Lou Y, et al. Combination of olfactory test and substantia nigra transcranial sonopraphy in the differential diagnosis of Parkinson's disease: a pilot study from China. Transl Neurodegener. 2012;1(1):25

22. López Hernández N, García Escrivá A, Shalabi Benavent M. Diagnostic value of combined assessment of olfaction and sustantia nigra hyperechogenicity for Parkinson's disease. Neurologia. 2015;30(8):496-501
23. Cardaioli G, Ripandelli F, Paolini Paoletti F, Nigro P, Simoni S, Brahimi E, et al. Substantia nigra hyperechogenicity in essential tremor and Parkinson's disease: a longitudinal study. Eur J Neurol. 2019;26(11):1370-6
24. Li DH, He YC, Liu J, Chen SD. Diagnostic Accuracy of Transcranial Sonography of the Substantia Nigra in Parkinson's disease: A Systematic Review and Meta-analysis. Sci Rep. 2016;6:20863
25. Berg D, Behnke S, Seppi K, Godau J, Lerche S, Mahlknecht P, et al. Enlarged hyperechogenic substantia nigra as a risk marker for Parkinson's disease. Mov Disord. 2013;28(2):216-9
26. Walter U, Heilmann R, Kaulitz L, Just T, Krause BJ, Benecke R, Höppner J. Prediction of Parkinson's disease subsequent to severe depression: a ten-year follow-up study. J Neural Transm (Vienna). 2015;122(6):789-97
27. Iranzo A, Stockner H, Serradell M, Seppi K, Valldeoriola F, Frauscher B, et al. Five-year follow-up of substantia nigra echogenicity in idiopathic REM sleep behavior disorder. Mov Disord. 2014;29(14):1774-80
28. Pilotto A, Heinzel S, Suenkel U, Lerche S, Brockmann K, Roeben B, et al. Application of the movement disorder society prodromal Parkinson's disease research criteria in 2 independent prospective cohorts. Mov Disord. 2017;32(7):1025-34
29. Miyamoto M, Miyamoto T. Relationship of substantia nigra hyperechogenicity to risk of Lewy body disease in idiopathic REM sleep behavior disorder patients: a longitudinal study. Sleep Med. 2020;68:31-4
30. Heinzel S, Kasten M, Behnke S, Vollstedt EJ, Klein C, Hagenah J, et al. Age- and sex-related heterogeneity in prodromal Parkinson's disease. Mov Disord. 2018;33(6):1025-7
31. Berg D, Postuma RB, Adler CH, Bloem BR, Chan P, Dubois B, et al. MDS research criteria for prodromal Parkinson's disease. Mov Disord. 2015;30(12):1600-11
32. Heinzel S, Berg D, Gasser T, Chen H, Yao C, Postuma RB. Update of the MDS research criteria for prodromal Parkinson's disease. Mov Disord. 2019;34(10):1464-70
33. Mahlknecht P, Marini K, Werkmann M, Poewe W, Seppi K. Prodromal Parkinson's disease: hype or hope for disease-modification trials? Transl Neurodegener. 2022;11(1):11

2.4 Nuklearmedizinische Bildgebung

Autoren/Autorinnen: Thilo van Eimeren, Rüdiger Hilker-Roggendorf, Jan Kassubek, Philipp T. Meyer

Fragestellung 17: Wie effektiv grenzt die FDG-PET im Vergleich mit dem langfristigen klinischen Follow-up die PK gegenüber anderen Diagnosen (MSA/PSP/CBD) ab?

Hintergrund

Gerade zu Beginn der Symptomatik stellt die Abgrenzung zwischen der PK und anderen neurodegenerativen Erkrankungen mit Parkinson-Syndrom (v.a. MSA, PSP und CBD) eine klinische Herausforderung dar. Mit der FDG-PET des Gehirns steht ein etabliertes Routineverfahren zur Verfügung, mit dem Auswirkungen der Erkrankung auf den Hirnmetabolismus erfasst und diagnostisch genutzt werden können. Anmerkung: Bei einem kortikobasalen Syndrom (CBS) liegt nicht immer eine CBD vor, allerdings praktisch nie eine Parkinson-Erkrankung. Im Sinne der Fragestellung wird hier daher auf eine Differenzierung zwischen CBS und CBD verzichtet.

Evidenzgrundlage

Der Bewertung lagen 3 rezente Metaanalysen zugrunde. Neuere Literatur, die in den Metaanalysen noch nicht erfasst wurde, wurde durchgesehen. Eine weitere Studie wurde als empfehlungsrelevant betrachtet, da sie eine Post-mortem-Validierung beinhaltete.

Ergebnis

Die FDG-PET grenzt mit hoher Sensitivität und Spezifität das PK gegenüber anderen Diagnosen (MSA/PSP/CBS) im Vergleich mit dem langfristigen klinischen Follow-up ab.

Begründung der Empfehlung

Rezente Metaanalysen zeigen durchgängig eine sehr hohe Sensitivität (84–91%) und Spezifität (91–96%) anderer Diagnosen (MSA/PSP/CBS) gegenüber der PK [1], [2], [3]. Die extrem hohe Spezifität weist dabei die besondere Wertigkeit der FDG-PET im Sinne eines Bestätigungstests aus. In einer Studie mit Post-mortem-Validierung durch eine histopathologische Diagnose zeigt die FDG-PET eine deutlich bessere Treffsicherheit im Vergleich zur klinischen Diagnose zum Zeitpunkt der Bildgebung [4].

Empfehlung	Modifiziert<br>Stand (2023)
Eine FDG-PET kann erwogen werden, wenn hinreichende klinische Hinweise auf ein atypisches Parkinson-Syndrom vorliegen und der Befund eine klinische Konsequenz hat (z.B. bzgl. Diagnose, Prognose, Therapie).	
Konsensstärke: 84%, Konsens	

Fragestellung 18: Wie effektiv ist die FDG-PET im Vergleich mit dem langfristigen klinischen Follow-up für die Prognose hinsichtlich des Auftretens einer Demenz bei der PK?

Hintergrund

Im gesamten Krankheitsverlauf der PK tritt eine Demenz in mehr als 80% der Fälle auf. Allerdings sind der Beginn und die Dynamik der demenziellen Entwicklung individuell äußerst variabel. Eine präzise Abschätzung der Prognose hinsichtlich eines frühen Auftretens einer Demenz kann wesentliche medizinische und soziale Vorteile haben. Die individuelle Wertigkeit der Prognose kann sehr unterschiedlich ausfallen.

Evidenzgrundlage

Der Bewertung lagen 5 longitudinale Studien zugrunde.

Ergebnis

Ein posteriorer kortikaler Hypometabolismus in der FDG-PET hat eine hohe prognostische Aussagekraft im Vergleich zum klinischen Follow-up hinsichtlich des Auftretens einer Demenz bei der PK.

Begründung der Empfehlung

Mehrere longitudinale Studien zeigen, dass das Muster eines posterioren kortikalen Hypometabolismus (typischerweise temporo-parietal, ggf. okzipital) mit kognitiver Verschlechterung korreliert und der Entwicklung einer Demenz um zum Teil mehrere Jahre vorausgeht [5], [6], [7], [8], [9]. Für die diesbezügliche prognostische Aussagekraft muss dabei nicht unbedingt bereits ein mildes kognitives Defizit (MCI) vorliegen. In einer prospektiven Studie bei 54 Pat. mit der PK ohne Demenz lag die Sensitivität bei 85% und die Spezifität bei 88% für das Auftreten einer Demenz innerhalb von 4 Jahren [8].

**Empfehlung**	**Neu**<br>**Stand (2023)**
Eine FDG-PET kann erwogen werden, um das Risiko für das Auftreten einer Demenz bei der PK zu bewerten, wenn der Befund eine klinische Konsequenz hat (z.B. bzgl. Diagnose, Prognose, Therapie).	
Konsensstärke: 97%, starker Konsens	

Fragestellung 19: Wie effektiv ist die präsynaptische Single-Photonen-Emissions-Computertomographie des Striatums (DAT-SPECT) im Vergleich mit dem langfristigen klinischen Follow-up für die Diagnose eines neurodegenerativen Parkinson-Syndroms?

Hintergrund

Die Treffsicherheit der klinischen Diagnose eines neurodegenerativen Parkinson-Syndroms ist nicht optimal. Es konnte wiederholt gezeigt werden, dass auch Experten/Expertinnen in ca. 20% der Fälle Fehldiagnosen stellen können. Ein objektiver Biomarker zur Diagnose eines neurodegenerativen Parkinson-Syndroms ist daher sehr hilfreich.

Evidenzgrundlage

Die Bewertung basiert auf 3 Metaanalysen. Neuere Literatur, die in den Metaanalysen noch nicht erfasst wurde, wurde durchgesehen. Zwei longitudinale Studien wurden als empfehlungsrelevant eingestuft, da sie sich den sogenannten SWEDD (subjects without evidence of dopaminergic deficit) widmeten.

Ergebnis

Die DAT-SPECT hat im Vergleich mit dem langfristigen klinischen Follow-up für die Diagnose eines neurodegenerativen Parkinson-Syndroms eine sehr hohe Sensitivität und Spezifität.

Begründung der Empfehlung

Die Evidenz zur Effektivität und Genauigkeit der DAT-SPECT hat sich im Vergleich zu den vorherigen Parkinson-Leitlinien nochmals erhöht. Die Durchführung wird in der DGN-Handlungsempfehlung (S1-Leitlinie) SPECT-Untersuchungen mit dem 123I-markierten Dopamintransporter-Liganden FP-CIT (DaTSCANTM) Stand: 5/2017 – AWMF-Registernummer: 031-037 aufgeführt. Es gibt mehrere neue Metaanalysen, die eine hohe bis sehr hohe Sensitivität und Spezifität ausweisen [10], [11], [12]. Die Sensitivität wurde von den meisten erfassten Studien mutmaßlich unterschätzt, da hier sehr wahrscheinlich auch sogenannte SWEDD (subjects without evidence of dopaminergic deficit) eingeschlossen waren, also Pat., bei denen klinisch eine PK diagnostiziert wurde, die aber kein Defizit in der DAT-SPECT aufwiesen. Der langfristige klinische Verlauf dieser Pat. hat in mehreren Studien gezeigt, dass diese Fälle weit überwiegend keine neurodegenerative PK aufweisen bzw. entwickeln [13], [14]. Dies hat dazu beigetragen, dass eine unauffällige DAT-SPECT als Ausschlusskriterium für die Diagnose einer PK gelten kann [15].

**Empfehlung**	**Modifiziert**<br>**Stand (2023)**
Die Dopamintransporter-SPECT (DAT-SPECT) soll frühzeitig im Krankheitsverlauf zum Nachweis eines nigro-striatalen Defizits bei klinisch unklarem Parkinson- oder Tremor-Syndrom erfolgen, wenn der Befund eine klinische Konsequenz hat (z.B. bzgl. Diagnose, Prognose, Therapie).	
Konsensstärke: 82,2%, Konsens	

Fragestellung 20: Wie effektiv grenzt die postsynaptische Single-Photonen-Emissions-Computertomographie des Striatums (IBZM-SPECT) im Vergleich mit dem langfristigen klinischen Follow-up die PK gegenüber anderen Diagnosen (MSA/PSP/CBD) ab?

Hintergrund

Gerade zu Beginn der Symptomatik stellt die Abgrenzung zwischen der PK und anderen neurodegenerativen Erkrankungen mit Parkinson-Syndrom (v.a. MSA, PSP und CBD) eine klinische Herausforderung dar. Die IBZM SPECT ermöglicht die Darstellung der postsynaptischen Dopaminrezeptoren, die bei der Parkinson-Erkrankung nicht reduziert sein sollten. Die vorherige Leitlinie kam zu einer negativen Bewertung („sollte nicht“) hinsichtlich der obigen Fragestellung.

Evidenzgrundlage

Seit der letzten Leitlinie wurden keine neuen Daten gefunden, die eine Wertigkeit in dieser Frage zeigen würden. Eine neue vergleichende Studie wurde als empfehlungsrelevant berücksichtigt.

Ergebnis

Die IBZM-SPECT grenzt die PK gegenüber anderen Diagnosen (MSA/PSP/CBD) nicht ausreichend effektiv ab.

Begründung der Empfehlung

Es gibt gegenüber der alten Leitlinie keine neue Evidenz, die eine Wertigkeit in dieser Frage zeigen würde. Zur differenzialdiagnostischen Abgrenzung der PK gegenüber anderen Diagnosen (MSA/PSP/CBD) ist laut einer prospektiven Vergleichsstudie die FDG-PET der IBZM-SPECT deutlich überlegen [16].

**Empfehlung**	**Geprüft<br>Stand (2023)**
Die IBZM-SPECT sollte nicht zur Differenzialdiagnose bei Parkinson-Syndromen eingesetzt werden.	
Konsensstärke: 96,9%, starker Konsens	

Fragestellung 21: Wie effektiv grenzt die kardiale MIBG-Szintigraphie bzw. Single-Photonen-Emissions-Computertomographie im Vergleich mit dem langfristigen klinischen Follow-up die PK gegenüber einer MSA ab?

Hintergrund

Gerade zu Beginn der Symptomatik stellt die Abgrenzung zwischen der PK und anderen neurodegenerativen Erkrankungen mit Parkinson-Syndrom (v.a. MSA, PSP, und CBD) eine klinische Herausforderung dar. Die kardiale MIBG-Szintigraphie bzw. -SPECT kann eine noradrenerge Denervierung anzeigen, die die meisten Pat. mit Parkinson-Erkrankung aufweisen. Pat. mit einer MSA zeigen dies in der Regel nicht.

Evidenzgrundlage

Es wurden 3 Metaanalysen bei der Empfehlung berücksichtigt. Zudem wurde eine retrospektive Vergleichsstudie zur FDG-PET als empfehlungsrelevant erachtet.

Ergebnis

Die kardiale MIBG-Szintigraphie bzw. -SPECT grenzt im Vergleich mit dem langfristigen klinischen Follow-up das PK gegenüber einer MSA mit guter Sensitivität und Spezifität ab.

Begründung der Empfehlung

Verschiedene Metaanalysen zeigen, dass die kardiale MIBG-Szintigraphie bzw. -SPECT im Vergleich mit dem langfristigen klinischen Follow-up die PK gegenüber einer MSA mit guter Sensitivität und

Spezifität abgrenzt [17], [18], [19]. Die Genauigkeit liegt dabei auf ähnlichem bzw. gering niedrigerem Niveau wie bei der FDG-PET (siehe Fragestellung 17).

Prospektive Vergleichsstudien zur FDG-PET fehlen. Eine retrospektive Vergleichsstudie kommt zu dem Ergebnis, dass die FDG-PET im Vergleich zur MIBG-Szintigraphie eine höhere, wenngleich statistisch nicht signifikant überlegene Trennschärfe (Fläche unter der ROC-Kurve: 82% vs. 69%) aufweist [20].

Jenseits des Vergleichs der diagnostischen Wertigkeit sind klinisch praktische Erwägungen zu berücksichtigen. Bei der MIBG-Szintigraphie bzw. -SPECT ist eine relativ hohe Störanfälligkeit für falsch positive (seltener falsch negative Befunde) durch medikamentöse Wechselwirkungen (z.B. Sympathomimetika, Kalziumantagonisten, trizyklische Antidepressiva, Opioide, Neuroleptika etc.) und ebenfalls im höheren Lebensalter auftretende Begleiterkrankungen (z.B. KHK, Diabetes, Polyneuropathie, Kardiomyopathien, Herzinsuffizienz) bei der Indikationsstellung und Durchführung zu berücksichtigen [21]. Zudem erlaubt die FDG PET, aber nicht die MIBG-Szintigraphie bzw. -SPECT, in vielen Fällen eine weitere Differenzierung unter den atypischen Parkinson-Syndromen. Unbedingt zu berücksichtigen sind Herzerkrankungen, Polyneuropathien und zahlreiche Medikamente, welche die MIBG-Bindung beeinflussen können [22].

**Empfehlung**	**Modifiziert<br>Stand (2023)**
Die kardiale MIBG-Szintigraphie bzw. -SPECT kann zur Abgrenzung einer PK von einer MSA erwogen werden, sofern keine FDG-PET verfügbar ist.	
Konsensstärke: 100%, starker Konsens	

Fragestellung 22: Wie effektiv grenzt die kardiale MIBG-Szintigraphie bzw. Single-Photonen-Emissions-Computertomographie im Vergleich mit dem langfristigen klinischen Follow-up die PK gegenüber einer 4R-Tauopathie (PSP und CBD) ab?

Hintergrund

Gerade zu Beginn der Symptomatik stellt die Abgrenzung zwischen der PK und anderen neurodegenerativen Erkrankungen mit Parkinson-Syndrom (v.a. MSA, PSP und CBD) eine klinische Herausforderung dar. Die kardiale MIBG-Szintigraphie bzw. -SPECT kann eine noradrenerge Denervierung anzeigen, die die meisten Pat. mit Parkinson-Erkrankung aufweisen. Pat. mit einer PSP und einer CBD zeigen dies in der Regel nicht.

Evidenzgrundlage

Es wurden 2 Metaanalysen bei der Empfehlung berücksichtigt.

Ergebnis

Die kardiale MIBG-Szintigraphie bzw. -SPECT grenzt im Vergleich mit dem langfristigen klinischen Follow-up die PK gegenüber einer PSP mit guter Sensitivität und Spezifität ab. Zu CBD ist die Datenlage zu gering, um eine Empfehlung zu stützen.

Begründung der Empfehlung

Metaanalysen legen eine vergleichbare Wertigkeit (im Vergleich zur MSA) bezüglich der Abgrenzung der PK gegenüber einer PSP nahe [18], [19].

Zu CBD bzw. dem kortikobasalen Syndrom ist die Datenlage zu gering.

Die praktischen Einschränkungen der MIBG-Szintigraphie bzw. -SPECT und die Vorteile der FDG-PET sind bereits bei den Fragestellungen 17 und 21 erörtert worden. Unbedingt zu berücksichtigen sind Herzerkrankungen, Polyneuropathien und zahlreiche Medikamente, welche die MIBG-Bindung beeinflussen können [22].

**Empfehlung**	**Neu**<br>**Stand (2023)**
▪ Die kardiale MIBG-Szintigraphie bzw. -SPECT kann zur Abgrenzung einer PK von einer PSP erwogen werden, sofern keine FDG-PET verfügbar ist.<br>▪ Die kardiale MIBG-Szintigraphie bzw. -SPECT kann aktuell zur Abgrenzung einer PK von einer CBD nicht empfohlen werden.	
Konsensstärke: 96,6%, starker Konsens	

Referenzen

1. Meyer PT, Frings L, Rücker G, Hellwig S. (18)F-FDG PET in Parkinsonism: Differential Diagnosis and Evaluation of Cognitive Impairment. J Nucl Med. 2017;58(12):1888-98
2. Gu SC, Ye Q, Yuan CX. Metabolic pattern analysis of (18)F-FDG PET as a marker for Parkinson's disease: a systematic review and meta-analysis. Rev Neurosci. 2019;30(7):743-56
3. Papathoma PE, Markaki I, Tang C, Lilja Lindström M, Savitcheva I, Eidelberg D, Svenningsson P. A replication study, systematic review and meta-analysis of automated image-based diagnosis in parkinsonism. Sci Rep. 2022;12(1):2763
4. Schindlbeck KA, Gupta DK, Tang CC, O'Shea SA, Poston KL, Choi YY, et al. Neuropathological correlation supports automated image-based differential diagnosis in parkinsonism. Eur J Nucl Med Mol Imaging. 2021;48(11):3522-9
5. Bohnen NI, Koeppe RA, Minoshima S, Giordani B, Albin RL, Frey KA, Kuhl DE. Cerebral glucose metabolic features of Parkinson disease and incident dementia: longitudinal study. J Nucl Med. 2011;52(6):848-55
6. Firbank MJ, Yarnall AJ, Lawson RA, Duncan GW, Khoo TK, Petrides GS, et al. Cerebral glucose metabolism and cognition in newly diagnosed Parkinson's disease: ICICLE-PD study. J Neurol Neurosurg Psychiatry. 2017;88(4):310-6
7. Baba T, Hosokai Y, Nishio Y, Kikuchi A, Hirayama K, Suzuki K, et al. Longitudinal study of cognitive and cerebral metabolic changes in Parkinson's disease. J Neurol Sci. 2017;372:288-93

8. Pilotto A, Premi E, Paola Caminiti S, Presotto L, Turrone R, Alberici A, et al. Single-subject SPM FDG-PET patterns predict risk of dementia progression in Parkinson disease. Neurology. 2018;90(12):e1029-e37
9. Booth S, Park KW, Lee CS, Ko JH. Predicting cognitive decline in Parkinson's disease using FDG-PET-based supervised learning. J Clin Invest. 2022;132(20)
10. Brigo F, Matinella A, Erro R, Tinazzi M. [^{123}I]FP-CIT SPECT (DaTSCAN) may be a useful tool to differentiate between Parkinson's disease and vascular or drug-induced parkinsonisms: a meta-analysis. Eur J Neurol. 2014;21(11):1369-e90
11. Buchert R, Buhmann C, Apostolova I, Meyer PT, Gallinat J. Nuclear Imaging in the Diagnosis of Clinically Uncertain Parkinsonian Syndromes. Dtsch Arztebl Int. 2019;116(44):747-54
12. Bega D, Kuo PH, Chalkidou A, Grzeda MT, Macmillan T, Brand C, et al. Clinical utility of DaTscan in patients with suspected Parkinsonian syndrome: a systematic review and meta-analysis. NPJ Parkinsons Dis. 2021;7(1):43
13. Batla A, Erro R, Stamelou M, Schneider SA, Schwingenschuh P, Ganos C, Bhatia KP. Patients with scans without evidence of dopaminergic deficit: a long-term follow-up study. Mov Disord. 2014;29(14):1820-5
14. Marek K, Seibyl J, Eberly S, Oakes D, Shoulson I, Lang AE, et al. Longitudinal follow-up of SWEDD subjects in the PRECEPT Study. Neurology. 2014;82(20):1791-7
15. Postuma RB, Berg D, Stern M, Poewe W, Olanow CW, Oertel W, et al. MDS clinical diagnostic criteria for Parkinson's disease. Mov Disord. 2015;30(12):1591-601
16. Hellwig S, Amtage F, Kreft A, Buchert R, Winz OH, Vach W, et al. [^{18}F]FDG-PET is superior to [^{123}I]IBZM-SPECT for the differential diagnosis of parkinsonism. Neurology. 2012;79(13):1314-22
17. Treglia G, Stefanelli A, Cason E, Cocciolillo F, Di Giuda D, Giordano A. Diagnostic performance of iodine-123-metaiodobenzylguanidine scintigraphy in differential diagnosis between Parkinson's disease and multiple-system atrophy: a systematic review and a meta-analysis. Clin Neurol Neurosurg. 2011;113(10):823-9
18. Treglia G, Cason E, Stefanelli A, Cocciolillo F, Di Giuda D, Fagioli G, Giordano A. MIBG scintigraphy in differential diagnosis of Parkinsonism: a meta-analysis. Clin Auton Res. 2012;22(1):43-55
19. Orimo S, Suzuki M, Inaba A, Mizusawa H. 123I-MIBG myocardial scintigraphy for differentiating Parkinson's disease from other neurodegenerative parkinsonism: a systematic review and meta-analysis. Parkinsonism Relat Disord. 2012;18(5):494-500
20. Brumberg J, Schröter N, Blazhenets G, Frings L, Volkmann J, Lapa C, et al. Differential diagnosis of parkinsonism: a head-to-head comparison of FDG PET and MIBG scintigraphy. NPJ Parkinsons Dis. 2020;6(1):39
21. Skowronek C, Zange L, Lipp A. Cardiac 123I-MIBG Scintigraphy in Neurodegenerative Parkinson Syndromes: Performance and Pitfalls in Clinical Practice. Front Neurol. 2019;10:152
22. Paolillo S, Rengo G, Pagano G, Pellegrino T, Savarese G, Femminella GD, et al. Impact of diabetes on cardiac sympathetic innervation in patients with heart failure: a 123I meta-iodobenzylguanidine (123I MIBG) scintigraphic study. Diabetes Care. 2013;36(8):2395-401

2.5 Molekulare Biomarker

Autoren/Autorinnen: Brit Mollenhauer, Kathrin Brockmann, Anja Schneider

Fragestellung 23: Wie effektiv ist die Untersuchung molekularer Biomarker (in CSF, Blut, Haut, Stuhl) im Vergleich mit langfristigen klinischen Follow-ups für die Diagnose einer PK?

Hintergrund

Nur wenige Biomarker in biologischen Flüssigkeiten haben sich in der Routine bei Parkinson bislang durchgesetzt.

Ergebnis

Inhaltliche Beschreibung der Studieninhalte mit Relevanz zur Fragestellung.

Der axonale Destruktionsmarker Neurofilament-Leichtketten (Neurofilament Light Chains, NfL) ist nicht erkrankungsspezifisch und findet sich im Liquor cerebrospinalis und im Blut erhöht bei verschiedenen neurologischen Erkrankungen [1]. Diese Ergebnisse konnten in verschiedenen Pat.-Kohorten repliziert werden [2]. Bei Pat. mit der PK sind die Werte im Kohortenmittel nur leicht höher als bei gesunden Kontrollen, es finden sich aber in einer Studie, die drei prospektive, unabhängige Pat.-Kohorten untersuchte, deutlich höhere Werte bei atypischen Parkinson-Syndromen, v.a. bei PSP und MSA [3]. Die Quantifizierung von NfL kann sowohl im Blut als auch im Liquor erfolgen und wird von einigen Laboren auch für die klinische Routine angeboten.

Die Alzheimer-Biomarker β-amyloid 1-42 und Gesamt-Tau-Protein im Liquor cerebrospinalis waren bei der PK in einer prospektiven Studie erniedrigt bzw. normal [4]. Ein niedriger β-amyloid 1-42 (niedriger Quotient von β-amyloid 1-42/1-40)-Wert im Liquor konnte in einer longitudinalen Kohortenstudie auf das Risiko für kognitive Defizite bei Parkinson hinweisen [5].

In den letzten Jahren haben sich zudem Methoden etabliert, die pathologisches fehlgefaltetes α-Synuklein (aSyn) im Liquor cerebrospinalis hoch sensitiv nachweisen. Diese „Seed Amplification Assays“ (SAA) funktionieren analog zu den Prionen-Assays. Durch die Hinzugabe von Protein-Monomer zur Pat.-Probe, in der sich das fehlgefaltete Protein (aSyn) befindet, wird eine Kaskade angestoßen, bei der das in der Probe befindliche pathologische Protein amplifiziert wird. Dieses Signal kann semiquantitativ über Fluoreszenz gemessen werden und zeigt sich in einer großen Metaanalyse über verschiedene Labore recht konsistent für Liquor cerebrospinalis mit Sensitivitäten und Spezifitäten über 90% [6]. Insbesondere konnten diese hohen Sensitivitäten und Spezifitäten in einer sehr großen internationalen multizentrischen Kohorte mit über 1.000 Probanden/Probandinnen repliziert werden [7]. Soweit bekannt ist, ist das Signal bis zu 10 Jahre vor der Parkinson-Erkrankung positiv, könnte sich also auch als früher Marker eignen, wie Daten von PK und Demenz mit Lewy Körperchen (DPB)-Konvertern einer prospektiven Kohorte von Probanden/Probandinnen mit REM-Schlafstörungen nahelegen [8]). Einige Studien zeigen ein unterschiedliches Signal für die PK versus MSA und viele Arbeitsgruppen arbeiten daran, derzeit das Verfahren in peripheren Flüssigkeiten bzw. Gewebe (wie z.B. Speichel, Hautbiopsie, olfaktorisches Epithel oder Blut) zu etablieren [9]. Seed amplification assays zeigen ein großes Potenzial, zukünftig als diagnostischer und spezifischer Biomarker für eine klare α-Synuklein-Pathologie im Rahmen einer

PK eingesetzt werden zu können. Aktuell wird dieses Verfahren in Deutschland aber noch nicht in der Routine angeboten.

**Empfehlung**	**Neu**<br>**Stand (2023)**
NfL (aus Liquor oder Serum) ist aufgrund fehlender Spezifität nicht als diagnostischer Biomarker einer PK geeignet, kann aber zur Abgrenzung von der PK zu atypischen Parkinson-Syndromen hilfreich sein.	
Konsensstärke: 100%, starker Konsens	

Referenzen

1. Gaetani L, Blennow K, Calabresi P, Di Filippo M, Parnetti L, Zetterberg H. Neurofilament light chain as a biomarker in neurological disorders. J Neurol Neurosurg Psychiatry. 2019;90(8):870-81
2. Mollenhauer B, Dakna M, Kruse N, Galasko D, Foroud T, Zetterberg H, et al. Validation of Serum Neurofilament Light Chain as a Biomarker of Parkinson's Disease Progression. Mov Disord. 2020;35(11):1999-2008
3. Hansson O, Janelidze S, Hall S, Magdalinou N, Lees AJ, Andreasson U, et al. Blood-based NfL: A biomarker for differential diagnosis of parkinsonian disorder. Neurology. 2017;88(10):930-7
4. Irwin DJ, Fedler J, Coffey CS, Caspell-Garcia C, Kang JH, Simuni T, et al. Evolution of Alzheimer's Disease Cerebrospinal Fluid Biomarkers in Early Parkinson's Disease. Ann Neurol. 2020;88(3):574-87
5. Siderowf A, Xie SX, Hurtig H, Weintraub D, Duda J, Chen-Plotkin A, et al. CSF amyloid {beta} 1-42 predicts cognitive decline in Parkinson disease. Neurology. 2010;75(12):1055-61
6. Yoo D, Bang JI, Ahn C, Nyaga VN, Kim YE, Kang MJ, Ahn TB. Diagnostic value of α-synuclein seeding amplification assays in α-synucleinopathies: A systematic review and meta-analysis. Parkinsonism Relat Disord. 2022;104:99-109
7. Siderowf A, Concha-Marambio L, Lafontant DE, Farris CM, Ma Y, Urenia PA, et al. Assessment of heterogeneity among participants in the Parkinson's Progression Markers Initiative cohort using α-synuclein seed amplification: a cross-sectional study. Lancet Neurol. 2023;22(5):407-17
8. Iranzo A, Fairfoul G, Ayudhaya ACN, Serradell M, Gelpi E, Vilaseca I, et al. Detection of α-synuclein in CSF by RT-QuIC in patients with isolated rapid-eye-movement sleep behaviour disorder: a longitudinal observational study. Lancet Neurol. 2021;20(3):203-12
9. Bellomo G, De Luca CMG, Paoletti FP, Gaetani L, Moda F, Parnetti L. α-Synuclein Seed Amplification Assays for Diagnosing Synucleinopathies: The Way Forward. Neurology. 2022;99(5):195-205

2.6 Digitale Biomarker

Digitale Biomarker zur Diagnose, Verlaufs- und Therapieevaluation bei der PK

Autoren: Walter Maetzler, Carsten Eggers

Grundsätzliche Aspekte

Der Begriff **digitaler Biomarker** wird hier als Charakteristikum oder als eine Reihe von Charakteristika, die von digitalen Gesundheitstechnologien erfasst werden und als Indikator für normale biologische Prozesse, pathogene Prozesse oder Reaktionen auf eine Exposition oder Intervention, einschließlich therapeutischer Interventionen, gemessen werden, definiert [1]. Die Autoren fokussieren auf tragbare digitale Gesundheitstechnologien und größere (N>200) longitudinale und Fall-(Kontroll-)Studien, die im häuslichen Umfeld oder unter „home-like“ Bedingungen durchgeführt wurden. In den letzten Jahren und Monaten wurden eine Reihe tragbarer digitaler Systeme von Zulassungsbehörden (EMA, FDA) bedingt als Optionen für die Evaluation der PK empfohlen. Diese Empfehlungen basieren auf dem Verständnis, dass die Geräte sicher einsetzbar sind und akzeptable Korrelationen zu klinisch basierten Messungen aufweisen [2]. Die Geräte müssen jedoch noch zeigen, ob und wie ihre Signale und Daten mit klinisch aussagekräftigen Ergebnissen auf Pat.-Ebene korrelieren („alltagsrelevante Daten“), ob diese Daten durch unabhängige Studien und Versuche bestätigt werden können und wie sich diese Messungen in klinisch relevante Ergebnisse (inklusive Diagnose, Verlaufsbeobachtung, Therapieevaluation und Prädiktion) bei der PK umsetzen lassen. Daher wird der Einsatz von den tragbaren digitalen Systemen bei der PK vom National Institute for Health and Care Excellence (NICE) in einer Stellungnahme von 01/2023 (www.nice.org.uk/guidance/dg51) nur zur Unterstützung der Behandlung empfohlen, wenn im Rahmen der Anwendung weitere Erkenntnisse gewonnen werden. Diese Erkenntnisse umfassen z.B., wie viele Ressourcen für die Anwendung der Geräte benötigt werden und ob die Messungen kosteneffektiv sind; wie häufig und wie lange gemessen werden muss, um relevante Behandlungsdaten zu generieren; und wie sich die Verwendung der Geräte auf die anwendenden Personen, auf deren Umfeld und deren Krankheit auswirkt (www.nice.org.uk/guidance/dg51). Das NICE hält auch fest, dass es derzeit keine belastbaren Belege dafür gibt, inwieweit sich die Anwendung von tragbaren digitalen Systemen auf die Lebensqualität von Pat. mit der PK und der sie betreuenden Personen auswirkt, wie die Geräte helfen sollen, Ressourcen im Gesundheitssystem zu sparen, bzw. welche Ressourcen gespart werden sollen. Die Autoren dieses Kapitels schließen sich dieser Einschätzung an.

Fragestellung 24: Wie effektiv ist die Untersuchung digitaler Biomarker im Vergleich mit dem langfristigen klinischen Follow-up für die Diagnose der PK?

Hintergrund

Die klinische Diagnose der PK stützt sich aktuell auf die klinische Untersuchung von motorischen und nicht motorischen Symptomen. Eine regelhafte Diagnoseunterstützung durch digitale Tools gibt es (noch) nicht. Durch die dynamische Entwicklung im Bereich der digitalen Gesundheitstechnologie ist ein zunehmendes Voranschreiten auch im diagnostischen Sektor zu erwarten.

Evidenzgrundlage

Aus einer Literaturrecherche in Pubmed mit den Suchbegriffen „Parkinson disease AND diagnosis AND digital biomarker“ wurden zwei Metaanalysen, drei größere Kohortenstudien und eine Fall-kontrollstudie identifiziert.

Ergebnis

In einer Metaanalyse [3] wurden Daten zur Geschwindigkeit von Anschlägen auf dem Keyboard herangezogen, um die motorischen Einschränkungen, u.a. der PK, zu untersuchen. Insgesamt 41 Studien fanden Eingang in die Analyse, hiervon waren 25 Studien zur PK. Es konnte gezeigt werden, dass die gepoolte Sensitivität und Spezifität für die Diagnosefindung der PK auf Basis der Keyboardanschläge bei 86% respektive 83% lag.

In einer weiteren Metaanalyse und systematischem Review wurde untersucht, wie mittels machine-learning-basierten Modellen von digitalen Daten u.a. in der Diagnosestellung diagnostische Genauigkeit erzielt werden kann [4]. Es konnten 49 verschiedene Studien in den Review eingeschlossen werden. Am häufigsten kamen Sensoren zur Anwendung, die am Körper der Pat. befestigt werden (Akzelerometer, Gyroskope, Magnetometer und andere Geschwindigkeitssensoren zur Erfassung von kinematischen Parametern), gefolgt von Smartphones oder Stand-alone-Videokameras. Die diagnostische Genauigkeit in Klassifikationsanalysen wurde insgesamt mit durchschnittlich 87% angegeben, wobei die höchste Genauigkeit für druckabhängige Signale vorlag (97%), gefolgt von Stimmaufzeichnungen.

In der DREAM-Studie (Parkinson’s Disease Digital Biomarker Challenge for digital diagnosis of PD Studie [5]) wurde anhand von Akzelerometerdaten aus Smartphones von 2.804 Teilnehmenden (656 Parkinson-Erkrankte (nach eigener Einschätzung) und 2.148 gesunde Kontrollen) eine einfache Gangaufgabe analysiert. Die diagnostische Genauigkeit zur Unterscheidung der beiden Gruppen wurde mit 0,87 (Area under the receiver-operating characteristic curve) angegeben.

In einer Fall-Kontrollstudie mit etwa 700 Pat. mit der PK und 7.000 Kontrollen wurde ein KI-basierter Algorithmus zur Evaluation des Atmens während des Schlafens entwickelt und angewendet [6]. Das KI-Modell konnte Pat. mit der PK von Kontrollen mit einer Genauigkeit (Area under the curve, AUC) von 0.85 bis 0.90 unterscheiden.

Die Nutzung von Sprachdaten wurde in einer großen Kohortenstudie mit Einschluss von 1.078 Parkinson-Pat. und 5.453 Kontrollen untersucht [7]. Herangezogen wurden 11.942 anhaltende Phonationen des Vokals „a“, die mittels Dysphonie-Analysen untersucht wurden. Hierbei fanden sich eine Sensitivität von 67,43% und eine Spezifität von 67,25% zur Unterscheidung von Pat. und Kontrollen.

Mittels automatisiert erhobener Daten von 6.614 Studienteilnehmenden mit Smartphones (iPhone) wurde in der m-Power-Studie die Reliabilität der Erfassung von Daten zu Stimme, Balance, Gang und Fingertapping untersucht [8]. Es wurden Daten von 807–1.674 gesunden Kontrollen (in Abhängigkeit von dem Task) bzw. 610–970 Parkinson-Pat. in die Analysen eingeschlossen. Für Gang, Balance und Stimme waren es geringe bis mittlere, für Tapping mittlere bis große Effektstärken zur Unterscheidung von Kontrollen und Parkinson-Pat. Es bestand eine niedrige bis gute Test-Retest-

Reliabilität für die meisten Features der Gang- und Balance-Tasks, eine gute bis exzellente Test-Retest-Reliabilität für Features der Stimme und des Tappings.

Begründung der Empfehlung

Die derzeit verfügbare Evidenz beruht auf zwei Metaanalysen und wenigen größeren longitudinalen bzw. Fall-Kontroll-basierten Beobachtungsstudien. Die Studien sind hinsichtlich der untersuchten Kohorten und verwendeten digitalen Gesundheitstechnologien, Algorithmen und Analyseansätze durchaus heterogen. Es herrscht bisher noch keine wissenschaftliche Eindeutigkeit hinsichtlich der zu verwendenden Tools. Das Potenzial von digitalen Biomarkern zur Diagnosestellung der PK ist zwar bereits mittels verschiedener Technologien umfassend untersucht, die Ergebnisse sind jedoch sehr variabel. Gute Ergebnisse hinsichtlich der diagnostischen Genauigkeit finden sich vor allem für kinematische Bewegungssensoren (vor allem für Druck), wobei keine eindeutige Empfehlung hinsichtlich der Platzierung oder der Art der erfassten Daten abgegeben werden kann.

Die Leitliniengruppe empfiehlt, dass digitale Gesundheitstechnologien in der Diagnosestellung supportiv eingesetzt werden können. Der Einsatz kann jedoch die klinische Diagnose der Erkrankung zum derzeitigen Stand der Technologie nicht ersetzen.

**Empfehlung**	**Neu**<br>**Stand (2023)**
Digitale Biomarker sollten zur Diagnosestellung der PK in der klinischen Routine aktuell nicht regelhaft eingesetzt werden.	
Konsensstärke: 100%, starker Konsens	

Fragestellung 25: Wie effektiv ist die Untersuchung digitaler Biomarker im Vergleich mit dem langfristigen klinischen Follow-up für die Verlaufsbeobachtung der PK?

Hintergrund

Verlaufsstudien bei der PK dienen zur Einschätzung der Krankheitsschwere und Symptomverteilung über die Zeit. Sie stellen eine wichtige Grundlage für das grundsätzliche Verständnis der Krankheitsprogression, und für die die Entwicklung von Therapieevaluationsparametern dar. Digitale Biomarker können komplementäre Daten zur Pat.-Einschätzung (z.B. Fragebögen, Diaries) und zur klinischen Evaluation (z.B. klinisch-neurologische Untersuchung, MDS-UPDRS) liefern, indem sie objektive und alltagsrelevante, über längere Zeiträume im häuslichen Umfeld erhobene Parameter liefern.

Evidenzgrundlage

In einer Literatursuche in Pubmed mit den Begriffen „Parkinson disease AND disease progression AND digital biomarker" wurden eine größere longitudinale Kohortenstudie und eine größere sowie eine große und mehrere kleinere Fall-Kontrollstudien identifiziert.

Ergebnis

In einer Longitudinalstudie an 388 Pat. im Frühstadium der PK, welche über durchschnittlich 390 Tage eine Smartwatch trugen und täglich unsupervidierte Assessments machten [9], zeigte sich eine mäßige bis starke Korrelation mit dem MDS-UPDRS Teil III für Ruhetremor (ρ = 0,70), Bradykinesie (ρ = -0,62) und Gang (ρ = -0,46). In einer Fall-Kontrollstudie mit etwa 700 Pat. mit der PK und 7.000 Kontrollen [6] wurde ein KI-basierter Algorithmus zur Evaluation des Atmens während des Schlafs entwickelt und angewendet. Das KI-Modell zeigte eine Korrelation von R=0.94 mit dem MDS-UPDRS-Gesamtscore. Kleinere Fallstudien weisen darauf hin, dass die digitale Analyse von unsupervidiertem Gang im häuslichen Bereich möglich und valide ist und Progression damit detektiert werden kann (z.B. [10]).

Begründung der Empfehlung

Die derzeit verfügbare Evidenz beruht auf wenigen größeren longitudinalen bzw. Fall-Kontroll-basierten Beobachtungsstudien sowie kleineren Fall-Kontrollstudien. Die Studien sind hinsichtlich der untersuchten Kohorten und verwendeten digitalen Gesundheitstechnologien, Algorithmen und Analyseansätze ausgesprochen heterogen. Das Potenzial von digitalen Biomarkern zur Verlaufsevaluation der PK ist daher von der Leitliniengruppe nicht adäquat abschätzbar und es wird keine Empfehlung ausgesprochen. Die Leitliniengruppe empfiehlt, digitale Gesundheitstechnologie in der klinischen Routine und in klinischen Studien explorativ zu verwenden, um die Entwicklung von digitalen Biomarkern zu unterstützen, und dabei darauf zu achten, dass alle Gruppen innerhalb der PK (inklusive Pat. mit kognitiven, nicht durch die PK bedingten Bewegungseinschränkungen, schweren durch die PK bedingten Einschränkungen) adäquat berücksichtigt sind.

Empfehlung	Neu<br>Stand (2023)
Digitale Biomarker sollten zur Verlaufsbeobachtung der PK in der klinischen Routine aktuell nicht regelhaft eingesetzt werden.	
Konsensstärke: 96,7%, starker Konsens	

Fragestellung 26: Wie effektiv ist die Untersuchung digitaler Biomarker im Vergleich mit dem langfristigen klinischen Follow-up für das Therapiemonitoring der PK?

Hintergrund

Digitale Biomarker können komplementäre Daten zu derzeit häufig eingesetzten Evaluationsinstrumenten, z.B. Pat.-Einschätzung (Fragebögen, Diaries) und klinischer Evaluation (klinisch-neurologische Untersuchung, MDS-UPDRS) liefern, indem sie objektive und alltagsrelevante, über längere Zeiträume im häuslichen Umfeld erhobene Parameter liefern.

Evidenzgrundlage

In einer Literatursuche in Pubmed mit den Begriffen „Parkinson disease AND treatment response AND digital biomarker“ wurden eine größere longitudinale Kohortenstudie und mehrere kleinere Fall-Kontrollstudien identifiziert.

Ergebnis

In einer Longitudinalstudie an N=388 Pat. im Frühstadium der PK, welche über durchschnittlich 390 Tage eine Smartwatch trugen und täglich unsupervidierte Assessments machten [9], zeigte sich eine leichte bis moderate Assoziation von Smartwatch-basierter Einschätzung von Tremor, Bradykinesie und Gang und der dopaminergen On- und Off-Einschätzung der Pat. (On vs. Off Cohen's d = 0,19–0,54). Gang unter dopaminergen On- und Off-Bedingungen und unsupervidierten Bedingungen wurde in kleineren Fallstudien untersucht, wobei die Unterscheidung gut bis sehr gut gelang [11], [10] und Gangvariabilität und Gangparameter während des Drehens besonders aussagekräftig scheinen [12].

Begründung der Empfehlung

Die derzeit verfügbare Evidenz beruht auf wenigen größeren longitudinalen bzw. Fall-Kontroll-basierten Beobachtungsstudien sowie kleineren Fall-Kontrollstudien. Die Studien sind hinsichtlich der untersuchten Kohorten und verwendeten digitalen Gesundheitstechnologien, Algorithmen und Analyseansätze ausgesprochen heterogen. Das Potenzial von digitalen Biomarkern zum Therapiemonitoring der PK ist daher von der Leitliniengruppe nicht adäquat abschätzbar, und es wird keine Empfehlung ausgesprochen. Die Leitliniengruppe empfiehlt, digitale Gesundheitstechnologie in der klinischen Routine und in klinischen Studien explorativ zu verwenden, um die Entwicklung von digitalen Biomarkern zu unterstützen, und dabei darauf zu achten, dass alle Gruppen innerhalb der PK (inklusive Pat. mit kognitiven, nicht durch die PK bedingten Bewegungseinschränkungen, schweren durch die PK bedingten Einschränkungen) adäquat berücksichtigt sind.

**Empfehlung**	**Neu**<br>**Stand (2023)**
Digitale Biomarker sollten für das Therapiemonitoring der PK in der klinischen Routine aktuell nicht regelhaft eingesetzt werden.	
Konsensstärke: 100%, starker Konsens	

Referenzen

1. Vasudevan S, Saha A, Tarver ME, Patel B. Digital biomarkers: Convergence of digital health technologies and biomarkers. NPJ Digit Med. 2022;5(1):36
2. Bloem BR, Post E, Hall DA. An Apple a Day to Keep the Parkinson's Disease Doctor Away? Ann Neurol. 2023;93(4):681-5
3. Alfalahi H, Khandoker AH, Chowdhury N, Iakovakis D, Dias SB, Chaudhuri KR, Hadjileontiadis LJ. Diagnostic accuracy of keystroke dynamics as digital biomarkers for fine motor decline in neuropsychiatric disorders: a systematic review and meta-analysis. Sci Rep. 2022;12(1):7690
4. Giannakopoulou KM, Roussaki I, Demestichas K. Internet of Things Technologies and Machine Learning Methods for Parkinson's Disease Diagnosis, Monitoring and Management: A Systematic Review. Sensors (Basel). 2022;22(5)
5. Zhang H, Deng K, Li H, Albin RL, Guan Y. Deep Learning Identifies Digital Biomarkers for Self-Reported Parkinson's Disease. Patterns (N Y). 2020;1(3)

6. Yang Y, Yuan Y, Zhang G, Wang H, Chen YC, Liu Y, et al. Artificial intelligence-enabled detection and assessment of Parkinson's disease using nocturnal breathing signals. Nat Med. 2022;28(10):2207-15
7. Arora S, Tsanas A. Assessing Parkinson's Disease at Scale Using Telephone-Recorded Speech: Insights from the Parkinson's Voice Initiative. Diagnostics (Basel). 2021;11(10)
8. Sahandi Far M, Eickhoff SB, Goni M, Dukart J. Exploring Test-Retest Reliability and Longitudinal Stability of Digital Biomarkers for Parkinson Disease in the m-Power Data Set: Cohort Study. J Med Internet Res. 2021;23(9):e26608
9. Burq M, Rainaldi E, Ho KC, Chen C, Bloem BR, Evers LJW, et al. Virtual exam for Parkinson's disease enables frequent and reliable remote measurements of motor function. NPJ Digit Med. 2022;5(1):65
10. Liu Y, Zhang G, Tarolli CG, Hristov R, Jensen-Roberts S, Waddell EM, et al. Monitoring gait at home with radio waves in Parkinson's disease: A marker of severity, progression, and medication response. Sci Transl Med. 2022;14(663):eadc9669
11. Evers LJ, Raykov YP, Krijthe JH, Silva de Lima AL, Badawy R, Claes K, et al. Real-Life Gait Performance as a Digital Biomarker for Motor Fluctuations: The Parkinson@Home Validation Study. J Med Internet Res. 2020;22(10):e19068
12. Shah VV, McNames J, Mancini M, Carlson-Kuhta P, Nutt JG, El-Gohary M, et al. Digital Biomarkers of Mobility in Parkinson's Disease During Daily Living. J Parkinsons Dis. 2020;10(3):1099-111

2.7 Genetische Diagnostik

Autoren/Autorinnen: Franziska Hopfner, Stephan Klebe, Christine Klein, Olaf Rieß, Gregor Kuhlenbäumer

Fragestellung 27a: Bei welchen Pat. mit PK besteht der begründete Verdacht auf eine monogene Ursache?

Fragestellung 27b: Bei welchen Pat. mit PK sollen eine genetische Beratung und eine genetische Untersuchung angeboten werden?

Hintergrund

Hereditäre, d.h. durch pathogene Varianten in einem einzelnen Gen verursachte Parkinson-Syndrome sind selten. Die Diagnose eines hereditären Parkinson-Syndroms kann durch genetische Diagnostik gesichert werden. Eine sichere Diagnose ist wichtig, um begründete Aussagen über die Prognose, die beste Therapie und über das Erkrankungsrisiko für Familienmitglieder zu treffen. Der begründete Verdacht einer monogenen Ursache ist vor allem vom Manifestationsalter und von der Familienanamnese abhängig. Die Nomenklatur der hereditären Parkinson-Syndrome besteht aus dem Terminus „PARK“, gefolgt von der Abkürzung des Gens, welches die pathogene Variante trägt, so wird z.B. eine hereditäre PK, die durch eine pathogene Variante im *LRRK2*-Gen versursacht wird, als PARK-*LRRK2* bezeichnet.

Evidenzgrundlage

Das Gesamtbild monogener Krankheiten setzt sich aus vielen Studien mit kleinen Stichproben von Familien und Einzelfällen zusammen. Da diese Studien unterschiedliche Schwerpunkte haben, sind sie sehr heterogen, was zu großen Schwankungen in der Schätzung der Häufigkeit der Symptome, des Verlaufs und weiterer Parameter führt. Hinzu kommt, dass mit der Zeit eine Publikationsverzerrung hin zu speziellen und extremen Phänotypen auftritt. Bereits bekannte Phänotypen lassen sich bei seit Langem als krankheitsursächlich nachgewiesenen pathogenen Varianten (in bekannten krankheitsassoziierten Genen) aufgrund des Fehlens eines Neuigkeitswerts nicht mehr publizieren. Eine standardisierte Einteilung von Studien in drei Evidenzklassen, die den meisten Abschnitten dieser Leitlinie zugrunde liegt, kann daher nicht sinnvoll angewendet werden. Die Datenbank MDSGene (Stand 01.05.2023, *https://www.mdsgene.org/*, [1]) hat zum Ziel, sämtliche Publikationen zu monogenen Formen von Bewegungsstörungen zu erfassen und statistisch auszuwerten. Kennzahlen bzgl. des Manifestationsalters und der initialen Symptome wurden daher aus der Datenbank MDSGene gewonnen. Es sind folgende Limitationen der Datenbank MDSGene zu beachten: Insgesamt sind die Fallzahlen pro Gen gering. Dies gilt vor allem für *DJ1*, *VPS35*, *SNCA* und *PINK1*, weniger für *LRRK2* und *PRKN*, was zu relativ großen Schwankungen der weiteren Angaben führen kann (Tabelle 5). Bezüglich der initialen Symptome ist zu beachten, dass die Angaben in Publikationen heterogen sind und daher bei bis zu 60% aller berichteten Pat. Angaben zu einzelnen Symptomen fehlen. Für das Manifestationsalter fehlt dagegen nur ein kleiner Teil der Daten (< 10% für jedes Gen mit Ausnahme von *DJ1* (19%)), sodass diese Zahlen relativ belastbar sind. Insgesamt gelten pathogene genetische Varianten in 7 Genen als gesicherte Ursache eines hereditären

Parkinson-Syndroms, von denen für 6 Daten in MDSGene vorhanden sind. Für die einzelnen im Folgenden besprochenen Gene sind folgende Publikationszahlen in MDSGene hinterlegt:

**Gen**	**Publikationen**
Leucine-rich repeat kinase 2 (*LRRK2*)	130
Synuclein alpha (*SNCA*)	51
VPS35 retromer complex component (*VPS35*)	9
Parkin RBR E3 ubiquitin protein ligase (*PRKN*)	174
PTEN induced kinase 1 (*PINK1*)	52
Parkinsonism-associated deglycase (*PARK7* auch bekannt als *DJ1*)	18

Pathogene genetische Varianten im Coiled-coil-Helix-Coiled-coil-Helix Domain containing 2 Gen (*CHCHD2*) wurden kürzlich als weitere sichere Ursache eines hereditären Parkinson-Syndroms durch die „Task Force for Nomenclature and Classification of Genetic Movement Disorders" der „International Parkinson and Movement Disorders Society (MDS)", www.movementdisorders.org*)* eingestuft. Zu *CHCHD2* wurden hier insgesamt 24 Publikationen ausgewertet, davon 2 Studien mit größeren Familien, 1 Einzelfallbericht und 21 Studien, in denen Serien von Parkinson-Pat., z.T. mit positiver Familienanamnese, auf Varianten in *CHCHD2* untersucht wurden. Darüber hinaus wurde das Gendiagnostikgesetz (www.gesetze-im-internet.de/gendg/index.html) verwendet, um die rechtlichen Rahmenbedingungen darzulegen.

Ergebnis

Pathogene genetische Varianten in insgesamt 7 Genen sind sichere Ursachen des hereditären Parkinson-Syndroms. Tabelle 5 zeigt den Median, die 90-Perzentile und die Altersspanne des Manifestationsalters sowie das prozentuale Auftreten der initialen Symptome für die sechs gut etablierten Gene. Das neu hinzugekommene Gen *CHCHD2* haben wir in einem separaten Absatz dargestellt. Altersverteilung und Verteilungen der initialen Symptome wurden anhand der Datenbank MDSGene [1] berechnet. Eine rezente weltweite, umfassende Erhebung von insgesamt 3888 Pat. mit pathogenen genetischen Varianten in den sechs genannten Genen kommt zu sehr ähnlichen Ergebnissen wie MDSGene [2]. Pathogene genetische Varianten in vier der ursächlichen Gene verursachen ein autosomal-dominantes hereditäres Parkinson-Syndrom (*LRRK2, SNCA, VPS35, CHCHD2*), drei weitere Formen des hereditären Parkinson-Syndroms sind durch biallelische pathogene genetische Varianten in den Genen *PRKN, PINK1* und *DJ1* verursacht. Unter biallelisch versteht man, dass beide Kopien des Gens eine pathogene genetische Variante tragen müssen, um die Krankheit zu verursachen. Gene, die eine atypische, oftmals juvenile hereditäre PK verursachen, wie z.B. *ATP13A2* (Kufor-Rakeb-Syndrom), *DNAJC6, FBXO7, SLC6A3* oder *SYNJ1*, sind nicht Gegenstand dieser Leitlinie. Die atypischen hereditären Parkinson-Syndrome sind sehr selten.

Autosomal-dominant vererbte Formen: Autosomal-dominante Krankheiten weisen im Allgemeinen folgende Charakteristika auf: Es finden sich mehrere Betroffene in aufeinanderfolgenden Generationen. Aufgrund reduzierter Penetranz können Generationen klinisch „übersprungen"

werden. Die Tabelle 5 zeigt, dass das Manifestationsalter der autosomal-dominanten hereditären Parkinson-Syndrome höher ist als das der autosomal-rezessiven Formen. Im Fall von PARK-*LRRK2* und PARK-*VPS35* ist es nahezu identisch mit dem mittleren Manifestationsalter der sporadischen PK, welches bei ungefähr 60 Jahren liegt [3], [4], [2]. Da diese Pat. unter einer klinisch nicht von der sporadischen PK unterscheidbaren Krankheit leiden, reproduktiv nicht benachteiligt sind und statistisch 50% aller Kinder eines betroffenen Elternteils Träger der pathogenen genetischen Variante sind, ist eine positive Familienanamnese der wichtigste Indikator eines autosomal-dominanten hereditären Parkinson-Syndroms. Eine Reihe von pathogenen genetischen Varianten in *LRRK2, SNCA* und *VPS35* zeigen eine reduzierte Penetranz, d.h., nicht alle Träger pathogener genetischer Varianten erkranken [5], [6], [7], [8]. Prominentestes Beispiel ist die bei Weitem häufigste *LRRK2*-Variante (Gly2019Ser oder G2019S), die in einigen Studien selbst im Alter von 80 Jahren nur eine Penetranz von ungefähr 40–50% erreicht [5], [6]. Aufgrund kleiner Familien, reduzierter altersabhängiger Penetranz und des Auftretens neuer („de-novo“) pathogener genetischer Varianten bei Pat. ist die positive Familienanamnese ein unsicherer Indikator und führt dazu, dass nicht alle Pat. mit autosomal-dominanter hereditärer PK diagnostiziert werden. Ein besseres Kriterium, wie z.B. ein besonderes klinisches Merkmal oder ein Biomarker, steht aber gegenwärtig nicht zur Verfügung.

Das *CHCHD2*-Gen wurde kürzlich von der Movement Disorder Society (MDS) in die Liste der Gene aufgenommen, in denen pathogene genetische Varianten eine autosomal-dominante hereditäre PK verursachen. Dies beruht vor allem auf einer japanischen Studie, in der die Kosegregation einer bislang nicht beschriebenen Variante im *CHCHD2*-Gen („Thr61Ile“) mit der PK in einer großen Familie gefunden wurde [9]. In drei weiteren kleineren Familien kosegregierten bekannte sehr seltene Varianten im *CHCHD2*-Gen mit der Krankheit [9] und in einer unabhängigen Publikation wurde Kosegregation der bereits erwähnten „Thr61Ile“-Variante mit der PK in einer mittelgroßen Familie gefunden [10]. Studien in großen Familien sind ein sehr sicherer Weg zur Identifikation von pathogenen genetischen Varianten in neuen krankheitsursächlichen Genen. In 22 weiteren Studien wurden Serien von einzelnen Pat. untersucht. Während die „Thr61Ile“-Variante als sicher pathogen eingestuft werden kann, ist dies für viele der in den Fallserien gefundenen Varianten noch nicht abschließend geklärt.

Autosomal-rezessiv vererbte Formen: Wichtigster Indikator eines autosomal-rezessiven (= biallelischen) hereditären Parkinson-Syndroms ist das jüngere Erkrankungsalter (Tabelle 5), das für alle drei Gene im Median bei ungefähr 30 Jahren liegt. Im Alter von 50 Jahren sind über 90% aller Pat. erkrankt. Bei einem autosomal-rezessiven Erbgang sind beide Elternteile Träger jeweils einer pathogenen genetischen Variante, jedoch klinisch nicht erkrankt. Selten kann auch ein Elternteil oder gar beide Elternteile Träger von neu aufgetretenen pathogenen genetischen Varianten sein. Statistisch wird nur jedes vierte Kind von heterozygoten Eltern biallelischer Träger pathogener genetischer Varianten sein und hat damit eine hohe Erkrankungswahrscheinlichkeit. Es ist daher in den meist kleinen europäischen Familien wenig wahrscheinlich, dass es in einer Generation mehrere Betroffene gibt, d.h., dass die Familienanamnese positiv ist. Aufgrund des Erbgangs sind auch Erkrankte in mehreren Generationen nicht zu erwarten. Kinder von Betroffenen sind alle heterozygote Träger einer pathogenen genetischen Variante. Insbesondere bei Verwandtenehen kann es daher zu einem erhöhten Wiederholungsrisiko kommen. Bei einer Stammbaumanalyse sollte

man jedoch auch beachten, dass es aufgrund der Häufigkeit der PK (auch als „sporadische" PK bezeichnet) in der Allgemeinbevölkerung auch zum Vorkommen mehrerer Betroffener in der gleichen oder in verschiedenen Generationen einer Familie kommen kann, d.h. Pat. mit einer hereditären PK und Pat. mit einer sporadischen PK (sog. Phänokopien). Es wird daher empfohlen, wenn möglich, den Pat. mit dem frühesten Erkrankungsalter als Indexpat. einer Familie genetisch zu untersuchen.

Beachtung des Gendiagnostikgesetzes: Gemäß Gendiagnostikgesetz (GenDG) §10 kann ein Arzt/eine Ärztin eine diagnostische genetische Untersuchung bei volljährigen, einwilligungsfähigen Pat. nach Aufklärung und Einwilligung gemäß GenDG im Rahmen seines/ihres Fachgebiets veranlassen. Bei einer behandelbaren Krankheit wie der hereditären PK soll laut GenDG §10 Abs. 1 auch eine genetische Beratung durch einen Arzt/eine Ärztin angeboten werden, der/die die Voraussetzungen nach §7 Abs. 1 und 3 GenDG erfüllt. Die genetische Beratung kann laut GenDG entweder durch einen Facharzt/eine Fachärztin für Humangenetik oder einen Neurologen/eine Neurologin mit der Qualifikation zur fachgebundenen humangenetischen Beratung (GenDG §7 Abs. 1) erfolgen. Obwohl nicht unmittelbar Gegenstand dieser Leitlinie, ist es wichtig, darauf hinzuweisen, dass eine prädiktive genetische Testung, d. h. eine genetische Untersuchung von potenziellen Trägern/Trägerinnen pathogener genetischer Varianten, die nicht erkrankt sind, nur durch Ärzte/Ärztinnen für Humangenetik oder durch Ärzte/Ärztinnen mit der Qualifikation zur fachgebundenen genetischen Beratung vorgenommen werden darf und eine genetische Beratung vor der Untersuchung und nach Vorliegen des Untersuchungsbefundes zwingend erfordert (GenDG, §7 Abs. 1).

Tab. 5: Angaben zu hereditären Parkinson-Syndromen

Form	PARK-*LRRK2*	PARK-*SNCA*	PARK-*VPS35*	PARK-*DJ1*	PARK-*PRKN*	PARK-*PINK1*
Gen	*LRRK2*	*SNCA*	*VPS35*	*DJ1* (PARK7)	*PRKN*	*PINK1*
Vererbungsmodus	autosomal-dominant mit reduzierter Penetranz*			autosomal-rezessiv mit hoher Penetranz		
Erkrankungsalter Median; 25./75. Perzentile (Bereich)	57; 47/65 (24 – 91) Jahre	46; 36/54 (19 – 77) Jahre	52; 45/61 (26 – 75) Jahre	27; 22/34 (14 – 40) Jahre	31; 23/38 (3 – 81) Jahre	32; 25/40 (9 – 67) Jahre
Anzahl Pat. in MDSGene (www.mdsgene.org)	723	146	68	35	1487	180

*mit Ausnahme der *SNCA*-Triplikation und pathogener Einzelnukleotidvarianten in *SNCA,* die vollständig penetrant sind

Begründung der Empfehlung

Für die sechs Gene in Tabelle 5 gibt es eine ausreichende Evidenz für die Kausalität und die Interpretierbarkeit der Varianten, um eine diagnostische genetische Untersuchung, d. h. eine Untersuchung von bereits an einer PK erkrankten Person zu rechtfertigen. Für *CHCHD2* trifft dies noch nicht zu, da viele potenziell pathogene genetische Varianten in diesem Gen noch nicht hinreichend interpretierbar sind.

Empfehlung	Modifiziert<br>Stand (2023)
1. Eine diagnostische genetische Untersuchung soll bei Pat.-Wunsch angeboten werden, wenn entweder<br>(I) zwei Verwandte ersten Grades oder ein Verwandter ersten und ein Verwandter zweiten Grades an einer PK erkrankt sind oder<br>(II) bei einer Krankheitsmanifestation vor dem 50. Lebensjahr.<br>2. Die Bestimmungen des Gendiagnostikgesetzes (GenDG) sind unbedingt einzuhalten. Für diagnostische genetische Untersuchungen sind dies insbesondere der Arztvorbehalt sowie die Aufklärung und Einwilligung gemäß GenDG. Eine genetische Beratung nach Vorliegen des Ergebnisses soll empfohlen werden.<br>3. Die Analyse von *CHCHD2* sollte bis zum Vorliegen größerer Datensätze aus verschiedenen ethnischen Gruppen zur Interpretation gefundener Varianten in spezialisierten neurologischen oder humangenetischen Zentren durchgeführt werden.	
Konsensstärke: 96,4%, starker Konsens	

Fragestellung 28: Welche phänotypischen und sonstigen Merkmale in den unter den in oben genannten Fragen definierten Pat.-Gruppen führen zur Empfehlung welcher Untersuchung?

Fragestellung 29: Welche Untersuchung hat die höchsten Erfolgsaussichten und den geringsten Anteil an falsch negativen/falsch positiven Ergebnissen? Welche Untersuchung ist wirtschaftlich?

Evidengrundlage

Die Evidenzgrundlage ist mit den PICO-Fragen 26 und 27 identisch.

Ergebnis

Die für eine genetische Untersuchung eines potenziell hereditären Parkinson-Syndroms infrage kommenden Pat.-Gruppen und Voraussetzungen gemäß Gendiagnostikgesetz sind in den Empfehlungen 1 und 2 der vorausgehenden Fragestellungen 27a und 27b bereits definiert.

Gruppe 1: Zwei Verwandte ersten Grades oder ein Verwandter ersten und ein Verwandter zweiten Grades sind an einer PK erkrankt und das Manifestationsalter liegt beiüber 50 Jahren. Eine autosomal-dominante hereditäre PK ist möglich; es sollten mindestens die Gene *LRRK2*, *SNCA* und *VPS35* untersucht werden.

Gruppe 2: Pat. mit PK mit einer Krankheitsmanifestation vor dem 50 Lebensjahr. Eine autosomal-rezessive oder autosomal-dominante hereditäre PK ist möglich. Daher müssen mindestens *PRKN*, *PINK1*, *DJ1*, *LRRK2*, *SNCA* und *VPS35* untersucht werden.

Da Varianten der Kopienzahl in *PRKN* und *SNCA* häufig sind, muss eine gezielte Analyse hierauf erfolgen. Seit einigen Jahren werden umfassende genetische Untersuchungen, d.h. Exomsequenzierungen, bei denen ca. 99% der kodierenden Bereiche (so gut wie aller Gene) erfasst werden, durchgeführt. In Pilotprojekten werden Genomsequenzierungen angewendet. Genomsequenzierungen ermöglichen z. B. auch den Nachweis von seltenen genetischen Varianten in

regulatorischen Sequenzen, von versteckten Spleißstellenmutationen in intronischen Bereichen oder von besonderen Strukturvarianten wie Inversionen. Daher stellt sich die Frage, ob zur genetischen Diagnostik eines Parkinson-Syndroms eine dieser Techniken genutzt werden soll. Die Abwägung kann nur individuell unter Einbeziehung der Pat. Erfolgen. Der Hauptvorteil einer exom-/genombasierten Diagnostik liegt darin, dass auch Gene erfasst werden, an die der/die die Untersuchung veranlassende Arzt/Ärztin nicht gedacht hat. Dies spielt v. a. dann eine Rolle, wenn die Pat. nicht unter einer reinen PK leiden, sondern weitere Symptome und Befunde aufweisen, die auf ein atypisches Parkinson-Syndrom oder eine andere mit einem Parkinson-Syndrom vergesellschaftete Krankheit hinweisen. Diesbezügliche Empfehlungen fallen nicht in den Bereich dieser Leitlinie. Bei der Wahl der Analyseart muss man die Pat. darüber aufklären, dass bei fast jeder genetischen Untersuchung, besonders aber bei einer umfassenden exom-/genombasierten Diagnostik auch kausale Varianten in Genen gefunden werden können, für die die Evidenz für eine eindeutige Aussage zur Kausalität (noch) nicht ausreicht bzw. die eine Bedeutung für die Entwicklung anderer Krankheiten haben. Diese Befunde können Pat. verunsichern und bedürfen sowohl einer besonders guten Aufklärung als auch einer Nachverfolgung, da sich die Evidenz für diese Befunde im Lauf der Zeit ändern kann.

Begründung der Empfehlung

Die Begründung der Empfehlung geht unmittelbar aus dem Abschnitt „Ergebnis“ hervor.

**Empfehlung**	**Neu**<br>**Stand (2023)**
1. Bei Parkinson-Pat. mit einem Manifestationsalter über 50 Jahren, die eine genetische Untersuchung wünschen und die zwei Verwandte ersten Grades oder einen Verwandten ersten- und einen Verwandten zweiten Grades mit einem Parkinson-Syndrom aufweisen, sollten mindestens *LRRK2*, *SNCA* und *VPS35* untersucht werden. Neben der Bestimmung der Sequenzabfolge müssen die angewandten Techniken auch Deletionen/Duplikationen nachweisen können.<br>2. Bei Parkinson-Pat. mit einer Krankheitsmanifestation vor dem 50. Lebensjahr, die eine genetische Untersuchung wünschen, sollten die Gene *PRKN*, *PINK1*, *DJ1*, *LRRK2*, *SNCA* und *VPS35* untersucht werden. Sind mehrere Familienmitglieder erkrankt, so sollte die Untersuchung präferenziell zuerst bei dem/der Pat. mit dem jüngsten Manifestationsalter durchgeführt werden. Neben der Bestimmung der Sequenzabfolge müssen die angewandten Techniken auch Gen-Deletionen/Duplikationen nachweisen können.<br>3. Sollte trotz eines negativen Befunds der unter Empfehlung 1 und 2 genannten Untersuchungen der Verdacht auf eine genetische Ursache der PK fortbestehen, so sollte, wenn die/der Pat. dies wünscht, eine/ein neurogenetisch spezialisierte/r Neurologin/Neurologe oder ein/e Humangenetikerin/Humangenetiker konsultiert werden, um das weitere klinisch-diagnostische Vorgehen zu planen.	
Konsensstärke: 100%, starker Konsens	

Fragestellung 30: Wie effektiv ist die genetische Untersuchung in den unter 1 definierten Gruppen für eine ätiologisch korrekte Diagnose?

Hintergrund

Im Gegensatz zu den meisten Fällen einer sporadischen – genetisch komplexen – PK gelingt bei den bekannten hereditären Parkinson-Syndromen meist eine klare ätiologische Zuordnung. Als Basis für eine sichere Zuordnung von pathogenen oder wahrscheinlich pathogenen genetischen Varianten werden auch in Deutschland die aktuellen Empfehlungen des American College of Medical Genetics and Genomics (ACMG) [11], [12] zugrunde gelegt. Dabei werden die wahrscheinlich pathogenen genetischen Varianten als „eine Veränderung, die aufgrund ihrer Eigenschaften sehr wahrscheinlich krankheitsverursachend ist“ und pathogene genetische Varianten als „eine Veränderung, die im Kontext der Indikation mit ausreichender Evidenz als klinisch relevant vorbeschrieben worden ist“ definiert.

Evidenzgrundlage

Die Einteilung in Evidenzklassen ist für diese PICO-Frage nur eingeschränkt anwendbar. Insbesondere randomisierte Studien sind für die meisten Fragestellungen nicht sinnvoll oder nicht möglich. In der Literaturrecherche fanden sich:

N=0 randomisierte, kontrollierte Studien

N=5 longitudinale größere Kohortenstudien

N=10 Falldarstellungen und Übersichtsartikel

Ergebnis

Die sehr schwankenden Angaben zur Häufigkeit der hereditären Parkinson-Syndrome sind bedingt durch den Aufbau der Studien, die zum Teil sehr heterogene Gruppen von Pat. und Kontrollen untersuchten. Die aktuell sieben etablierten monogenen Ursachen eines hereditären Parkinson-Syndroms (autosomal-dominant: *LRRK2*, *SNCA*, *VPS35*, CHCHD2; biallelisch/autosomal-rezessiv: *PRKN*, *PINK1*, *DJ1*) sind in unselektierten Gruppen von Pat. mit einer PK selten (siehe Tabelle 5). In gut charakterisierten Gruppen von Pat. mit jungem Erkrankungsbeginn (zumeist <50 Jahre) und/oder positiver Familienanamnese, sind diese, abhängig vom ethnischen Hintergrund, hingegen weit häufiger. Dies trifft vor allem für autosomal-dominante pathogene genetische Varianten in *LRRK2* als auch für biallelische (autosomal-rezessive) pathogene genetische Varianten in *PRKN* zu (https://www.mdsgene.org/, [13], [14]). Dabei liegt allerdings das mediane Erkrankungsalter bei Pat. mit PARK-*LRRK2* bei 57 Jahren und die 90-Perzentile bei 72 Jahren und kann somit nicht als Unterscheidungskriterium zur sporadischen PK dienen [3], [4]. In genetischen Untersuchungen mit großen Gruppen von Pat. mit PK liegt allerdings meist eine Verzerrung vor, da teilnehmende Zentren meist ein spezielles Interesse an hereditären Parkinson-Syndromen haben und häufig Pat. einschließen, bei denen eine monogene Ursache vermutet wird. Naturgemäß werden hier keine randomisierten Untersuchungen durchgeführt („Selektionsbias“). Für *LRRK2* konnte in Studien bei Pat. mit nordafrikanischer Herkunft und positiver Familienanamnese eine Frequenz von 41% pathogener *LRRK2*-Varianten gezeigt werden [13]. Dabei wurde ein Gründereffekt der häufigsten pathogenen *LRRK2*-Variante (Gly2019Ser oder G2019S) bestätigt. Es besteht ein Nord-Süd-Gradient dieser pathogenen *LRRK2*-Variante mit Frequenzen <5% in europäischen Ländern (https://www.mdsgene.org/, [15]). Bei Pat. mit einer positiven Familienanamnese für eine PK liegt

eine höhere Frequenz für pathogene genetische Varianten in *LRRK2* vor. Für die Bundesrepublik Deutschland kann man je nach Studie bei 1,3% der Pat. mit positiver Familienanamnese und 0,8% der sporadischen Pat. von pathogenen genetischen Varianten in *LRRK2* ausgehen [16], [17], [18], [19]. Die weltweite Frequenz von PARK-*LRRK2* liegt bei 1% der Parkinson-Syndrome und 4% der Pat. mit positiver Familienanamnese [20]. Im Vergleich zu *LRRK2* sind die beiden anderen autosomal-dominanten hereditären Parkinson-Syndrome (PARK-*SNCA*, PARK-*VPS35*) selten (siehe Tabelle 5). Für PARK-*SNCA* existieren lediglich Einzelfallberichte oder Beschreibungen von größeren Familien mit 1 bis maximal 12 Pat. bzw. für PARK-*VPS35* mit 2 – 17 Pat. (https://www.mdsgene.org/). Bei den biallelisch vererbten autosomal-rezessiven hereditären Parkinson-Syndromen stellen Pat. PARK-*PRKN* die größte Gruppe dar (https://www.mdsgene.org/). Allerdings scheint es auch bei den autosomal-rezessiven Formen ethnische und/oder regionale Unterschiede zu geben. In einer großen Studie mit 1587 Pat. (497 Pat. mit laut Stammbaumanalyse autosomal-rezessiven Erbgang; 1090 Pat. ohne positive Familienanamnese) wurden bei 12,5% biallelische pathogene genetische Varianten in *PRKN*, 1,9% in *PINK1* und 0,16% in *DJ1* gefunden [14]. Dabei waren vor allem Pat. mit nordafrikanischem Ursprung positiv für pathogene genetische Varianten in *PINK1*. Insgesamt ist bei diesen Frequenzen allerdings auch von einem Selektionsbias auszugehen. Dabei wird die Rate an positiv getesteten Pat. höher ausfallen, wenn Pat. mit frühem Erkrankungsbeginn untersucht werden [21], [22].

Begründung der Empfehlung

Die Effektivität der genetischen Diagnostik der hereditären Parkinson-Syndrome (PARK-*LRRK2*, PARK-*SNCA*, PARK-*VPS35*, PARK-*CHCHD2,* PARK-*PRKN*, PARK-*PINK1*, PARK-*DJ1*) ist aufgrund der aktuellen Studienlage nicht exakt anzugeben. Sie hängt maßgeblich von der untersuchten Pat.-Gruppe ab. Dabei spielen das Erkrankungsalter, die Familienanamnese, Konsanguinität der Eltern und die Herkunft bzw. der ethnische Hintergrund eine große Rolle. Als Grundlage zur Beurteilung der Pathogenität von genetischen Varianten dient die Einteilung des American College of Medical Genetics and Genomics (ACMG). Dabei werden benigne, wahrscheinlich pathogene und pathogene genetische Varianten in insgesamt 5 Klassen eingeteilt [11], [12]. Benigne Varianten ohne klinische Relevanz werden als Klasse 1, wahrscheinlich benigne Varianten als Klasse 2, Varianten unklarer Signifikanz als Klasse 3, wahrscheinlich pathogene genetische Varianten als Klasse 4 und pathogene genetische Varianten als Klasse 5 eingeordnet. Insbesondere die Varianten unklarer Signifikanz (Klasse 3) sollten in regelmäßigen Abständen hinsichtlich einer möglichen veränderten Klassifikation überprüft werden.

**Empfehlung**	**Neu**<br>**Stand (2023)**
1. Um eine hohe richtig positive Rate für hereditäre Parkinson-Syndrome zu erreichen, sollte in der genetischen Diagnostik eine Vorauswahl der zu untersuchenden Pat. anhand der in den PICO-Fragen 27a und 27b beschriebenen Kriterien erfolgen. Es ist klar, dass hierbei eine nicht bekannte Rate an hereditären Parkinson-Syndromen nicht erfasst wird. Dies trifft vor allem auf die autosomal-dominanten Formen mit spätem Beginn (PARK-*LRRK2*, PARK-*VPS35*) zu, die phänotypisch nicht von der sporadischen PK zu unterscheiden sind.<br>2. Für die Bewertung der Pathogenität von genetischen Varianten soll die Klassifikation des American College of Medical Genetics and Genomics (ACMG) verwendet werden. Dabei gelten	

wahrscheinlich pathogene (Klasse 4) oder pathogene genetische Varianten (Klasse 5) als krankheitsverursachend. Die Transmission (autosomal-dominant vs. biallelisch) soll in die Beurteilung mit einbezogen werden. Varianten unklarer Signifikanz (Klasse 3) sollten gegenwärtig ungefähr 4 Jahre nach Feststellung hinsichtlich der Neubewertung ihrer Klasse gemäß ACMG überprüft werden.
Konsensstärke: 100%, starker Konsens

Fragestellung 31: Wie effektiv ist die genetisch gesicherte Diagnose einer hereditären PK für die Einschätzung der Prognose der PK bzgl. der Parameter Überlebensdauer, Lebensqualität und kognitive Einbußen?

Hintergrund

Die PK und die Ausprägung der motorischen und der nicht motorischen Symptome haben Auswirkungen auf verschiedene Bereiche des täglichen Lebens. Es existieren wenig Studien, die Pat. mit hereditären Parkinson-Syndromen hinsichtlich der Parameter Überlebensdauer, Lebensqualität und kognitive Einbußen untersuchen und/oder mit Kontrollgruppen mit sporadischer PK verglichen haben. Dies wirft die Frage auf, ob eine zuverlässige Aussage bezüglich der in Frage 31 genannten Parameter für die hereditären Parkinson-Syndrome möglich ist.

Evidenzgrundlage

In der Literaturrecherche fanden sich:

N=0 randomisierte, kontrollierte Studien

N=3 longitudinale größere Kohortenstudien

N= 5 Falldarstellungen und Übersichtsartikel

Ergebnis

In einer prospektiven israelischen Studie zeigte sich eine verlängerte Überlebensrate von Pat. mit der pathologischen *G2019S*-Variante im *LRRK2*-Gen [23]. Pat. mit Triplikationen des *SNCA*-Gens zeigten im Vergleich zu Pat. mit Duplikation eine um 8 Jahre kürzere Überlebenszeit vom Beginn der motorischen Symptome bis zum Tod [24]. Belastbare Daten über eine unterschiedliche Lebenserwartung von Pat. mit PARK-*PRKN*, PARK-*PINK1*, PARK-*DJ1*, PARK-*CHCHD2* oder PARK-*VPS35* im Vergleich zur sporadischen PK existieren nicht. Hinsichtlich der Lebensqualität von Pat. mit hereditärer PK ist die Studienlage sehr schwach. Daher kann keine fundierte Aussage zur Lebensqualität gemacht werden. Der Krankheitsverlauf ist bei PARK-*LRRK2*-Pat. in einer prospektiven Studie über 7 Jahre mit 144 PARK-*LRRK2*-Pat. signifikant langsamer gewesen als bei Pat. ohne pathogene *LRKK2*-Variante, gemessen an der Unified Parkinson Disease Rating Scale [25]. Im Gegensatz dazu liegt eine schnellere Progredienz bei Pat. mit PARK-*SNCA* vor, die aber auch von der spezifischen pathogenen genetischen Variante abhängt. Pat. mit Triplikationen in *SNCA* und drei der vier bekannten pathologischen Varianten (A53T, H50Q, A30P) zeigen im Gegensatz zu Pat. mit Duplikationen wahrscheinlich eine schnellere Progredienz [24]. Keine suffizienten Daten liegen zur

Krankheitsprogredienz bei PARK-*VPS35,* PARK-*CHCHD2,* PARK-*PRKN,* PARK-*PINK1* und PARK-*DJ1* vor. Eine große Metaanalyse wertete die kognitiven und neuropsychiatrischen Symptome bei Pat. mit hereditärer PK aus [26]. Interessanterweise zeigten Pat. mit PARK-*PINK1* die größten kognitiven Beeinträchtigungen, gefolgt von Pat. mit PARK-*DJ1* und PARK-*SNCA*. In dieser Metaanalyse konnte bei 55% der Pat. mit PARK-*PRKN* mindestens eine psychiatrische Diagnose durch die Anwendung von validierten Skalen gestellt werden. Dabei wurde eine Depression bei 23.5%, eine Impulskontrollstörung bei 22.1%, eine Angststörung bei 18% und eine Psychose bei 7% der Pat. diagnostiziert. Eine Arbeit verglich 21 Pat. mit PARK-*PRKN* mit 23 Pat. mit einer sporadischen PK. Dabei konnten keine Unterschiede in der Häufigkeit von Depressionen gefunden werden [26]. Die Datenbank „MDSGene“ konnte bei 45% der Pat. mit einer PARK-*SNCA* eine kognitive Beeinträchtigung nachweisen. Dabei spielte die Art der genetischen Veränderungen von *SCNA* (Triplikationen vs. Duplikationen vs. Punktmutationen) keine Rolle (kognitive Beeinträchtigung bei 7 von 8 Fällen mit Triplikation, 27 von 39 Fällen mit Duplikation; 16 von 24 Fällen mit Punktmutationen) [27]. Allerdings gehen andere Studien von einer höheren Rate kognitiver Beeinträchtigungen bei Pat. mit *SNCA*-Triplikation im Vergleich zur *SNCA*-Duplikation aus [28]. Pat. mit PARK-*VPS35* and PARK-*LRRK2* zeigten die geringste Rate an kognitiven Beeinträchtigungen. Für Pat. mit PARK-*LRRK2* konnte in kleineren Arbeiten kein sicherer Einfluss auf die Kognition oder auf neuropsychiatrische Symptome gezeigt werden [26], [29], [30]. Bezüglich der Metaanalysen muss betont werden, dass die neuropsychologischen Untersuchungsmethoden und Untersuchungszeitpunkte zwischen den eingeschlossenen Studien sehr stark divergieren. Weitere nicht motorische Symptome, die für hereditäre Parkinson-Syndrome beschrieben sind, umfassen Depression, Halluzinationen, Schlafstörungen und autonome Symptome. Diese kommen wahrscheinlich am häufigsten bei Pat. mit PARK-*SNCA* und weniger häufig bei den anderen hereditären Parkinson-Syndromen vor [28].

Begründung der Empfehlung

Der Verlauf der hereditären Parkinson-Syndrome (PARK-*LRRK2*, PARK-*SNCA*, PARK-*VPS35*, PARK-*CHCHD2*, PARK-*PRKN*, PARK-*PINK1*, PARK-*DJ1*) ähnelt in vielen Fällen dem Verlauf einer sporadischen PK. Dabei fehlen allerdings für alle Formen prospektive longitudinale Vergleichsstudien. Eine Ausnahme stellen Pat. mit Triplikationen und drei der vier pathologischen Varianten (A53T, H50Q, A30P) im *SNCA*-Gen dar, die eine schnellere Progression und vermutlich einen höheren Anteil von kognitiven Defiziten haben, wobei hier ebenfalls keine prospektiven Studien existieren. Daher ist es bei einer hereditären PK auf Einzelfallebene nicht möglich, eine sichere Vorhersage über den Verlauf oder die Lebenserwartung zu machen.

**Empfehlung**	**Neu**<br>**Stand (2023)**
Die Diagnose einer hereditären PK erlaubt keine sichere Vorhersage für die einzelne Pat. in Hinsicht auf Überlebensdauer, Lebensqualität und kognitive Einbußen.	
Konsensstärke: 100%, starker Konsens	

Fragestellung 32: Wie effektiv ist die genetisch gesicherte Diagnose des hereditären Parkinson-Syndroms für die Therapieentscheidung?

Hintergrund

Genetische Untersuchungen können dazu beitragen, Erkrankungen zu diagnostizieren und Diagnosen abzusichern. Einige monogene Krankheiten (z.B. Mammakarzinom) können durch eine humangenetische Untersuchung bereits frühzeitig, effektiv und individuell behandelt werden. Für die sporadische PK und hereditäre Parkinson-Syndrome stehen bislang keine ursachenspezifischen Therapien zur Verfügung.

Evidenzgrundlage

Die Literatursuche in Pubmed erbrachte 144 Studien zu den Begriffen „Parkinson disease AND genetic AND treatment decision or therapy decision AND Genetic Parkinson disease treatment“. Nach Durchsicht der Abstracts wurden Arbeiten selektiert, welche in Bezug auf die PICO-Frage 32 relevante Informationen beinhalteten. Bei allen Arbeiten handelte es sich um Fallserien und/oder Übersichtsartikel.

Ergebnis

Rasch fortschreitende Analysemethoden bieten immer zuverlässigere und kosteneffizientere Ansätze, um hereditäre Parkinson-Syndrome zu diagnostizieren [31]. Eine umfassende genetische Untersuchung mit Pathogenitätsbewertung identifizierter genetischer Varianten liefert in vielen Fällen eine Antwort auf die Frage, ob eine hereditäre PK vorliegt (siehe Tabelle 5, Fragen 27a/27b). Das Ansprechen auf eine dopaminerge Therapie ist bei den meisten Pat. mit hereditärer PK als gut bis sehr gut [32], [33], [9] dokumentiert. Prospektive, randomisierte Studien mit einem Vergleich zwischen sporadischer PK und hereditärem Parkinson-Syndrom liegen allerdings nicht vor. Daher gelten für die hereditären Parkinson-Syndrome aktuell die gleichen medikamentösen und nicht medikamentösen Therapieempfehlungen wie für die sporadische PK. Spezifische Medikamente für hereditäre Parkinson-Syndrome gibt es bislang nicht. Einige Fallbeispiele berichten über eine erfolgreiche Tiefe Hirnstimulation bei Pat. mit hereditärer PK [34], [35], [36]. Dies gilt vor allem für Pat. mit PARK-*LRRK2* und PARK-*PRKN* [34], [37]. Fallserien berichten auch eine Verbesserung der motorischen Symptome bei Pat. mit *SNCA*-Duplikationen [38], [39], [40], [41], [39]. Vergleichsstudien über den Therapieerfolg einer Tiefen Hirnstimulation zwischen Pat. mit hereditärer PK und sporadischer PK gibt es nicht.

Begründung der Empfehlung

Derzeit stehen für die Behandlung der hereditären Parkinson-Syndrome noch keine individualisierten Behandlungsoptionen zur Verfügung. Es ist anzunehmen, dass in Zukunft spezifische Therapien für Träger bestimmter pathogener genetischer Varianten zur Verfügung stehen werden. Daher gelten für die hereditären Parkinson-Syndrome die allgemeinen medikamentösen Therapieempfehlungen. Eine Tiefe Hirnstimulation kann bei hereditären Parkinson-Syndromen erfolgen.

Empfehlung	Neu

	Stand (2023)
▪ Für die hereditäre PK gelten die gleichen medikamentösen Therapieempfehlungen wie bei einer sporadischen PK.<br>▪ Eine Tiefe Hirnstimulation ist bei monogener PK möglich. Dabei gelten die gleichen Ein- und Ausschlusskriterien wie bei einer sporadischen PK.	
Konsensstärke: 100%, starker Konsens	

Fragestellung 33: Gibt es eine Gruppe von Parkinson-Pat., bei denen komplex-genetische Faktoren zu berücksichtigen sind?

Hintergrund

Die sporadische PK wird durch multiple genetische Risikofaktoren in Kombination mit Umwelteinflüssen und weiteren Faktoren verursacht, die nur teilweise bekannt sind [42]. Häufige genetische Varianten können das Krankheitsrisiko allgemein, wenn auch nur in sehr geringem Ausmaß beeinflussen [42]. Wichtigste bekannte genetische Risikofaktoren der komplex genetischen PK sind Risikovarianten im Glucocerebrosidase-Gen (*GBA1*, Genlocus Chr 1: 155.23–155.24 Mb) [43].

Evidenzgrundlage

Die Literatursuche in Pubmed erbrachte 348 Studien zu den Begriffen „Parkinson disease AND genetic AND complex-genetic factors OR genetic diagnostics and Parkinson disease AND Parkinson disease OR *GBA1* OR GBA“. Nach Screening der Abstracts wurden Arbeiten selektiert, welche in Bezug auf die PICO-Frage 33 relevante Informationen beinhalteten. Bei allen Arbeiten handelte es sich um Fallserien und/oder Übersichtsartikel.

Ergebnis

Epidemiologische Studien zeigen, dass indirekte genetische Faktoren (u. a. erkrankte Verwandte ersten Grades, Familienanamnese von Tremor) das Risiko einer PK erhöhen [44], [45]. Polygene Risikoscores fassen die Summe häufiger, schwacher genetischer Risikovarianten zusammen [46]. Polygene Risikoscores spielen derzeit keine Rolle in der Diagnostik, für die Prognose oder für die Therapie der PK. Dies wird sich in Zukunft wahrscheinlich ändern [47], [48], [49]. Risikovarianten im *GBA1*-Gen sind bei 7–10% der Pat. vorhanden, die an der PK erkranken [43], [50]. Diese Risikovarianten können, wie alle genetischen Varianten heterozygot, homozygot oder compound-heterozygot vorliegen. Die Odds-Ratio (OR) dieser heterozygoten Risikovarianten liegt zwischen 2 und 10, was bei Krankheiten mit niedriger Prävalenz ungefähr einer Verdopplung bis Verzehnfachung des Erkrankungsrisikos entspricht. Einzelne Varianten lassen Aussagen über die Schwere der PK und den Verlauf der Erkrankung (früheres Erkrankungsalter, schwerere nicht motorische Symptome, frühere kognitive Beeinträchtigungen, raschere Progredienz der Erkrankung) zu [51], [52], [53], [54].

Die Wahrscheinlichkeit der Entwicklung einer PK bei Trägern/Trägerinnen von genetischen Varianten in *GBA1* hängt stark von der spezifischen Risikovariante (z. B. p.L444P: OR 10-15 und p.N370S: OR≤ 5) ab [55], [54]. Das Erkrankungsrisiko starker Risikovarianten in *GBA1* liegt nahe an dem Risiko

niedrigpenetranter pathogener genetischer Varianten in *LRRK2* [56], [54]. Hier ist die Grenze zwischen Risikovarianten und hereditärer PK mit reduzierter Penetranz unscharf. In dieser Leitlinie werden heterozygote pathogene Varianten im *GBA1*-Gen als Risikovarianten verstanden, d.h. nicht als Ursache des als monogen verstandenen „hereditären Parkinson-Syndroms“. Verschiedene biallelische, pathogene, genetische Varianten in *GBA1* verursachen die Gaucher-Krankheit. Schätzungen zeigen, dass die Gaucher-Krankheit mit einem um das 20–30-Fache erhöhten Risiko der Entwicklung einer PK einhergeht [57], [58]. Die Gaucher-Krankheit ist eine Multisystemkrankheit und daher nicht Gegenstand dieser Leitlinie. Einige Varianten, die für die Gaucher-Krankheit nicht pathogen sind, erhöhen nachweislich das Risiko, eine PK zu entwickeln [59], [60], [61]. Pathogene *GBA1*-Varianten können entsprechend ihrer Pathogenizität klassifiziert werden (PD GBA_schwer, PD GBA_mild, PD GBA_risiko) [54]. Retrospektive Studien legen nahe, dass Pat. mit Risikovarianten in *GBA1*-Gen bezüglich ihrer motorischen Fähigkeiten von der Tiefen Hirnstimulation profitieren [62], [63], [64]. Mehrere Fallserien berichteten aber über eine raschere Zunahme kognitiver Beeinträchtigung nach Tiefer Hirnstimulation bei Pat. mit Risikovarianten in *GBA1* als bei Pat. mit sporadischer PK oder anderen hereditären Parkinson-Syndromen. Eine entsprechende Vergleichsgruppe mit Risikovarianten in *GBA1*, aber ohne Tiefe Hirnstimulation wurde nicht eingeschlossen [65], [64], [62], [65]. Eine Folgestudie untersuchte 58 Pat. mit Risikovariante in *GBA1*-Gen und Tiefer Hirnstimulation sowie 82 Pat. mit Risikovariante im *GBA1*-Gen ohne Tiefe Hirnstimulation und fand eine raschere Zunahme der kognitiven Beeinträchtigung in der Gruppe mit Tiefer Hirnstimulation [66]. Die bisherigen Daten reichen für eine sichere Einschätzung einer beschleunigten kognitiven Beeinträchtigung nicht aus. Auch ist nicht klar, ob die Wahl des Zielgebiets (STN vs. GPi) eine Rolle spielt. Die Frage, ob starke Risikovarianten in *GBA1* mit einem höheren Risiko einer rascheren Progredienz der kognitiven Beeinträchtigung unter Tiefer Hirnstimulation einhergehen als Varianten mit niedrigem Risiko, kann gegenwärtig noch nicht zuverlässig beantwortet werden [67]. Aufgrund der vorhandenen Evidenz sollte bei bekannter *GBA1*-Risikovariante vor Tiefer Hirnstimulation eine umfassende Aufklärung über Chancen und Risiken unter besonderer Berücksichtigung des kognitiven Status im Einzelfall erfolgen. Pat. mit einer geplanten Tiefen Hirnstimulation kann die Untersuchung von *GBA1* angeboten werden. Gegenwärtig gibt es keine krankheitsmodifizierende Therapie für Träger von *GBA1*-Risikovarianten. Die medikamentöse Behandlung wird vom Vorliegen einer Variante nicht beeinflusst. Im menschlichen Genom existiert neben *GBA1* ein *GBAP1*-Pseudogen [68]. Die Sequenz des Pseudogens ist nahezu identisch mit der des *GBA1*-Gens. Hierdurch erlauben die gängigen „Short-Read-Next-Generation-Sequencing“-Techniken keine vollständig korrekte Sequenzierung des *GBA1*-Gens. Genaue Ergebnisse können mithilfe der konventionellen Sanger-Sequenzierung unter Verwendung *GBA1*-spezifischer Primer und mit „Long-Read-Next-Generation-Sequencing“-Techniken erzielt werden.

Begründung der Empfehlung

Umfassende genetische Untersuchungen (z. B. genomweite Genotypisierungs-Chips) zur Erfassung des polygenen Risikos spielen gegenwärtig keine Rolle für Diagnose, Prognose oder Therapie der PK. *GBA1*-Risikovarianten haben keinen Einfluss auf die Pharmakotherapie. Möglicherweise führen *GBA1*-Risikovarianten zu einer rascheren Progredienz kognitiver Einschränkungen bei Tiefer Hirnstimulation.

**Empfehlung**	**Neu**<br>**Stand (2023)**
1. Untersuchungen zur Erfassung des polygenen Risikos sollten nicht routinemäßig durchgeführt werden.<br>2. Untersuchungen zur Erfassung genetischer Varianten im *GBA1*-Gen sollten nicht routinemäßig durchgeführt werden. Bei einer isoliert erscheinenden PK sowie einer Krankheitsmanifestation vor dem 50. Lebensjahr oder bei Parkinson-Pat. mit raschem Verlauf (vor allem schnelle kognitive Verschlechterung nach klinischer Diagnosestellung) kann eine Untersuchung der *GBA1* erfolgen, wenn die/der Pat. dies nach sorgfältiger Aufklärung über Vor- und Nachteile wünscht.<br>3. Die Bestimmungen des Gendiagnostikgesetzes (GenDG) sind unbedingt einzuhalten. Für diagnostische genetische Untersuchungen sind dies insbesondere der Arztvorbehalt sowie die Aufklärung und Einwilligung gemäß GenDG. Eine genetische Beratung nach Vorliegen des Ergebnisses soll empfohlen werden.<br>4. Wird eine Therapie mit Tiefer Hirnstimulation erwogen, so kann eine Sequenzierung des *GBA1*-Gens bei Pat., die dies wünschen, durchgeführt werden. Sie erfordert vor der Untersuchung und wenn das Ergebnis vorliegt, eine umfassende Aufklärung über Chancen und Risiken der Tiefen Hirnstimulation bei Pat. mit *GBA1*-Risikovariante.	
Konsensstärke: 96,2%, starker Konsens	

Referenzen

1. Lill CM, Mashychev A, Hartmann C, Lohmann K, Marras C, Lang AE, et al. Launching the movement disorders society genetic mutation database (MDSGene). Mov Disord. 2016;31(5):607-9
2. Vollstedt EJ, Schaake S, Lohmann K, Padmanabhan S, Brice A, Lesage S, et al. Embracing Monogenic Parkinson's Disease: The MJFF Global Genetic PD Cohort. Mov Disord. 2023;38(2):286-303
3. Grover S, Kumar Sreelatha AA, Pihlstrom L, Domenighetti C, Schulte C, Sugier PE, et al. Genome-wide Association and Meta-analysis of Age at Onset in Parkinson Disease: Evidence From the COURAGE-PD Consortium. Neurology. 2022;99(7):e698-e710
4. Blauwendraat C, Heilbron K, Vallerga CL, Bandres-Ciga S, von Coelln R, Pihlstrøm L, et al. Parkinson's disease age at onset genome-wide association study: Defining heritability, genetic loci, and α-synuclein mechanisms. Mov Disord. 2019;34(6):866-75
5. Hentati F, Trinh J, Thompson C, Nosova E, Farrer MJ, Aasly JO. LRRK2 parkinsonism in Tunisia and Norway: a comparative analysis of disease penetrance. Neurology. 2014;83(6):568-9
6. Sierra M, González-Aramburu I, Sánchez-Juan P, Sánchez-Quintana C, Polo JM, Berciano J, et al. High frequency and reduced penetrance of LRRK2 G2019S mutation among Parkinson's disease patients in Cantabria (Spain). Mov Disord. 2011;26(13):2343-6

7. Sharma M, Ioannidis JP, Aasly JO, Annesi G, Brice A, Bertram L, et al. A multi-centre clinico-genetic analysis of the VPS35 gene in Parkinson disease indicates reduced penetrance for disease-associated variants. J Med Genet. 2012;49(11):721-6
8. Nishioka K, Ross OA, Ishii K, Kachergus JM, Ishiwata K, Kitagawa M, et al. Expanding the clinical phenotype of SNCA duplication carriers. Mov Disord. 2009;24(12):1811-9
9. Funayama M, Ohe K, Amo T, Furuya N, Yamaguchi J, Saiki S, et al. CHCHD2 mutations in autosomal dominant late-onset Parkinson's disease: a genome-wide linkage and sequencing study. Lancet Neurol. 2015;14(3):274-82
10. Shi CH, Mao CY, Zhang SY, Yang J, Song B, Wu P, et al. CHCHD2 gene mutations in familial and sporadic Parkinson's disease. Neurobiol Aging. 2016;38:217.e9-.e13
11. Richards S, Aziz N, Bale S, Bick D, Das S, Gastier-Foster J, et al. Standards and guidelines for the interpretation of sequence variants: a joint consensus recommendation of the American College of Medical Genetics and Genomics and the Association for Molecular Pathology. Genet Med. 2015;17(5):405-24
12. Rehder C, Bean LJH, Bick D, Chao E, Chung W, Das S, et al. Next-generation sequencing for constitutional variants in the clinical laboratory, 2021 revision: a technical standard of the American College of Medical Genetics and Genomics (ACMG). Genet Med. 2021;23(8):1399-415
13. Lesage S, Ibanez P, Lohmann E, Pollak P, Tison F, Tazir M, et al. G2019S LRRK2 mutation in French and North African families with Parkinson's disease. Ann Neurol. 2005;58(5):784-7
14. Lesage S, Lunati A, Houot M, Romdhan SB, Clot F, Tesson C, et al. Characterization of Recessive Parkinson Disease in a Large Multicenter Study. Ann Neurol. 2020;88(4):843-50
15. Correia Guedes L, Ferreira JJ, Rosa MM, Coelho M, Bonifati V, Sampaio C. Worldwide frequency of G2019S LRRK2 mutation in Parkinson's disease: a systematic review. Parkinsonism Relat Disord. 2010;16(4):237-42
16. Berg D, Schweitzer KJ, Leitner P, Zimprich A, Lichtner P, Belcredi P, et al. Type and frequency of mutations in the LRRK2 gene in familial and sporadic Parkinson's disease*. Brain. 2005;128(Pt 12):3000-11
17. Schlitter AM, Woitalla D, Mueller T, Epplen JT, Dekomien G. The LRRK2 gene in Parkinson's disease: mutation screening in patients from Germany. J Neurol Neurosurg Psychiatry. 2006;77(7):891-2
18. Hedrich K, Winkler S, Hagenah J, Kabakci K, Kasten M, Schwinger E, et al. Recurrent LRRK2 (Park8) mutations in early-onset Parkinson's disease. Mov Disord. 2006;21(9):1506-10
19. Möller JC, Rissling I, Mylius V, Höft C, Eggert KM, Oertel WH. The prevalence of the G2019S and R1441C/G/H mutations in LRRK2 in German patients with Parkinson's disease. Eur J Neurol. 2008;15(7):743-5
20. Healy DG, Falchi M, O'Sullivan SS, Bonifati V, Durr A, Bressman S, et al. Phenotype, genotype, and worldwide genetic penetrance of LRRK2-associated Parkinson's disease: a case-control study. Lancet Neurol. 2008;7(7):583-90
21. Alcalay RN, Caccappolo E, Mejia-Santana H, Tang MX, Rosado L, Ross BM, et al. Frequency of known mutations in early-onset Parkinson disease: implication for genetic counseling: the consortium on risk for early onset Parkinson disease study. Arch Neurol. 2010;67(9):1116-22
22. Sutherland RM, McCredie JA, Inch WR. Effect of splenectomy and radiotherapy on lymphocytes in Hodgkin's disease. Clin Oncol. 1975;1(4):275-84
23. Thaler A, Kozlovski T, Gurevich T, Bar-Shira A, Gana-Weisz M, Orr-Urtreger A, et al. Survival rates among Parkinson's disease patients who carry mutations in the LRRK2 and GBA genes. Mov Disord. 2018;33(10):1656-60
24. Kasten M, Klein C. The many faces of alpha-synuclein mutations. Mov Disord. 2013;28(6):697-701

25. Saunders-Pullman R, Mirelman A, Alcalay RN, Wang C, Ortega RA, Raymond D, et al. Progression in the LRRK2-Asssociated Parkinson Disease Population. JAMA Neurol. 2018;75(3):312-9
26. Piredda R, Desmarais P, Masellis M, Gasca-Salas C. Cognitive and psychiatric symptoms in genetically determined Parkinson's disease: a systematic review. Eur J Neurol. 2020;27(2):229-34
27. Trinh J, Zeldenrust FMJ, Huang J, Kasten M, Schaake S, Petkovic S, et al. Genotype-phenotype relations for the Parkinson's disease genes SNCA, LRRK2, VPS35: MDSGene systematic review. Mov Disord. 2018;33(12):1857-70
28. Kasten M, Marras C, Klein C. Nonmotor Signs in Genetic Forms of Parkinson's Disease. Int Rev Neurobiol. 2017;133:129-78
29. Alcalay RN, Mejia-Santana H, Mirelman A, Saunders-Pullman R, Raymond D, Palmese C, et al. Neuropsychological performance in LRRK2 G2019S carriers with Parkinson's disease. Parkinsonism Relat Disord. 2015;21(2):106-10
30. Gunzler SA, Riley DE, Chen SG, Tatsuoka CM, Johnson WM, Mieyal JJ, et al. Motor and non-motor features of Parkinson's disease in LRRK2 G2019S carriers versus matched controls. J Neurol Sci. 2018;388:203-7
31. Pillay NS, Ross OA, Christoffels A, Bardien S. Current Status of Next-Generation Sequencing Approaches for Candidate Gene Discovery in Familial Parkinson´s Disease. Front Genet. 2022;13:781816
32. van der Brug MP, Singleton A, Gasser T, Lewis PA. Parkinson's disease: From human genetics to clinical trials. Sci Transl Med. 2015;7(305):205ps20
33. Over L, Brüggemann N, Lohmann K. Therapies for Genetic Forms of Parkinson's Disease: Systematic Literature Review. J Neuromuscul Dis. 2021;8(3):341-56
34. Prendes Fernández P, Blázquez Estrada M, Sol Álvarez J, Álvarez Martínez V, Suárez San Martín E, García Fernández C, et al. Analysis of deep brain stimulation of the subthalamic nucleus (STN-DBS) in patients with monogenic PRKN and LRRK2 forms of Parkinson's disease. Parkinsonism Relat Disord. 2023;107:105282
35. Weill C, Gallant A, Baker Erdman H, Abu Snineh M, Linetsky E, Bergman H, et al. The Genetic Etiology of Parkinson's Disease Does Not Robustly Affect Subthalamic Physiology. Mov Disord. 2023;38(3):484-9
36. Weiss D, Landoulsi Z, May P, Sharma M, Schüpbach M, You H, et al. Genetic stratification of motor and QoL outcomes in Parkinson's disease in the EARLYSTIM study. Parkinsonism Relat Disord. 2022;103:169-74
37. Kuusimäki T, Korpela J, Pekkonen E, Martikainen MH, Antonini A, Kaasinen V. Deep brain stimulation for monogenic Parkinson's disease: a systematic review. J Neurol. 2020;267(4):883-97
38. Shimo Y, Natori S, Oyama G, Nakajima M, Ishii H, Arai H, Hattori N. Subthalamic deep brain stimulation for a Parkinson's disease patient with duplication of SNCA. Neuromodulation. 2014;17(1):102-3
39. Youn J, Oyama G, Hattori N, Shimo Y, Kuusimäki T, Kaasinen V, et al. Subthalamic deep brain stimulation in Parkinson's disease with SNCA mutations: Based on the follow-up to 10 years. Brain Behav. 2022;12(2):e2503
40. Antonini A, Pilleri M, Padoan A, Landi A, Ferla S, Biundo R, D'Avella D. Successful subthalamic stimulation in genetic Parkinson's disease caused by duplication of the α-synuclein gene. J Neurol. 2012;259(1):165-7
41. Perandones C, Aráoz Olivos N, Raina GB, Pellene LA, Giugni JC, Calvo DS, et al. Successful GPi stimulation in genetic Parkinson's disease caused by mosaicism of alpha-synuclein gene duplication: first description. J Neurol. 2015;262(1):222-3
42. Reid JB, Ross JJ. Mendel's genes: toward a full molecular characterization. Genetics. 2011;189(1):3-10

43. Sidransky E, Nalls MA, Aasly JO, Aharon-Peretz J, Annesi G, Barbosa ER, et al. Multicenter analysis of glucocerebrosidase mutations in Parkinson's disease. N Engl J Med. 2009;361(17):1651-61
44. Noyce AJ, Bestwick JP, Silveira-Moriyama L, Hawkes CH, Giovannoni G, Lees AJ, Schrag A. Meta-analysis of early nonmotor features and risk factors for Parkinson disease. Ann Neurol. 2012;72(6):893-901
45. Hopfner F, Höglinger GU, Kuhlenbäumer G, Pottegård A, Wod M, Christensen K, et al. β-adrenoreceptors and the risk of Parkinson's disease. Lancet Neurol. 2020;19(3):247-54
46. Dudbridge F. Power and predictive accuracy of polygenic risk scores. PLoS Genet. 2013;9(3):e1003348
47. Dehestani M, Liu H, Gasser T. Polygenic Risk Scores Contribute to Personalized Medicine of Parkinson's Disease. J Pers Med. 2021;11(10)
48. Liu H, Dehestani M, Blauwendraat C, Makarious MB, Leonard H, Kim JJ, et al. Polygenic Resilience Modulates the Penetrance of Parkinson Disease Genetic Risk Factors. Ann Neurol. 2022;92(2):270-8
49. Koch S, Schmidtke J, Krawczak M, Caliebe A. Clinical utility of polygenic risk scores: a critical 2023 appraisal. J Community Genet. 2023
50. Hruska KS, LaMarca ME, Scott CR, Sidransky E. Gaucher disease: mutation and polymorphism spectrum in the glucocerebrosidase gene (GBA). Hum Mutat. 2008;29(5):567-83
51. Cooper DN, Krawczak M, Polychronakos C, Tyler-Smith C, Kehrer-Sawatzki H. Where genotype is not predictive of phenotype: towards an understanding of the molecular basis of reduced penetrance in human inherited disease. Hum Genet. 2013;132(10):1077-130
52. Brockmann K, Berg D. The significance of GBA for Parkinson's disease. J Inherit Metab Dis. 2014;37(4):643-8
53. Pilotto A, Schulte C, Hauser AK, Biskup S, Munz M, Brockmann K, et al. GBA-associated parkinsonism and dementia: beyond α-synucleinopathies? Eur J Neurol. 2016;23(3):520-6
54. Höglinger G, Schulte C, Jost WH, Storch A, Woitalla D, Krüger R, et al. GBA-associated PD: chances and obstacles for targeted treatment strategies. J Neural Transm (Vienna). 2022;129(9):1219-33
55. Roos J, Müller S, Giese A, Appenzeller S, Ringelstein EB, Fiehler J, et al. Pontine autosomal dominant microangiopathy with leukoencephalopathy: Col4A1 gene variants in the original family and sporadic stroke. J Neurol. 2023;270(5):2631-9
56. Brockmann K, Srulijes K, Pflederer S, Hauser AK, Schulte C, Maetzler W, et al. GBA-associated Parkinson's disease: reduced survival and more rapid progression in a prospective longitudinal study. Mov Disord. 2015;30(3):407-11
57. Bultron G, Kacena K, Pearson D, Boxer M, Yang R, Sathe S, et al. The risk of Parkinson's disease in type 1 Gaucher disease. J Inherit Metab Dis. 2010;33(2):167-73
58. McNeill A, Duran R, Hughes DA, Mehta A, Schapira AH. A clinical and family history study of Parkinson's disease in heterozygous glucocerebrosidase mutation carriers. J Neurol Neurosurg Psychiatry. 2012;83(8):853-4
59. Straniero L, Asselta R, Bonvegna S, Rimoldi V, Melistaccio G, Soldà G, et al. The SPID-GBA study: Sex distribution, Penetrance, Incidence, and Dementia in GBA-PD. Neurol Genet. 2020;6(6):e523
60. Iwaki H, Blauwendraat C, Leonard HL, Liu G, Maple-Grødem J, Corvol JC, et al. Genetic risk of Parkinson disease and progression:: An analysis of 13 longitudinal cohorts. Neurol Genet. 2019;5(4):e348
61. Zhang Y, Shu L, Sun Q, Zhou X, Pan H, Guo J, Tang B. Integrated Genetic Analysis of Racial Differences of Common GBA Variants in Parkinson's Disease: A Meta-Analysis. Front Mol Neurosci. 2018;11:43

62. Lythe V, Athauda D, Foley J, Mencacci NE, Jahanshahi M, Cipolotti L, et al. GBA-Associated Parkinson's Disease: Progression in a Deep Brain Stimulation Cohort. J Parkinsons Dis. 2017;7(4):635-44
63. Weiss D, Brockmann K, Srulijes K, Meisner C, Klotz R, Reinbold S, et al. Long-term follow-up of subthalamic nucleus stimulation in glucocerebrosidase-associated Parkinson's disease. J Neurol. 2012;259(9):1970-2
64. Angeli A, Mencacci NE, Duran R, Aviles-Olmos I, Kefalopoulou Z, Candelario J, et al. Genotype and phenotype in Parkinson's disease: lessons in heterogeneity from deep brain stimulation. Mov Disord. 2013;28(10):1370-5
65. Mangone G, Bekadar S, Cormier-Dequaire F, Tahiri K, Welaratne A, Czernecki V, et al. Early cognitive decline after bilateral subthalamic deep brain stimulation in Parkinson's disease patients with GBA mutations. Parkinsonism Relat Disord. 2020;76:56-62
66. Duffin C. Researchers ask why diagnoses of cancer are too often delayed. Emerg Nurse. 2014;22(4):11
67. Pal G, Mangone G, Hill EJ, Ouyang B, Liu Y, Lythe V, et al. Parkinson Disease and Subthalamic Nucleus Deep Brain Stimulation: Cognitive Effects in GBA Mutation Carriers. Ann Neurol. 2022;91(3):424-35
68. Horowitz M, Wilder S, Horowitz Z, Reiner O, Gelbart T, Beutler E. The human glucocerebrosidase gene and pseudogene: structure and evolution. Genomics. 1989;4(1):87-96

3 Therapie

3.1 Parkinson-Medikamente

Anti-Parkinson-Medikamente und allgemeine Therapieprinzipien

Autoren/Autorinnen: Matthias Höllerhage, Günter Höglinger, Claudia Trenkwalder

Fragestellung 34: Welche Eigenschaften beeinflussen die Priorisierung der einzelnen Levodopa-Präparationen für individuelle Pat.?

Hintergrund

Zur Veränderung der Pharmakokinetik von Levodopa wurden sogenannte „modified-release"-Formulierungen entwickelt mit dem Ziel, einerseits gleichmäßigere Plasmaspiegel und andererseits eine schnellere Resorption zu erreichen. Levodopa wird in Deutschland in fester Kombination (4 : 1) entweder mit dem Dopadecarboxylase-Hemmer Carbidopa oder Benserazid vermarktet. Levodopa/Carbidopa und Levodopa/Benserazid sind in Standardform, in retardierter Form sowie in rasch löslicher Form erhältlich. Es stellt sich daher die Frage, ob es für die unterschiedlichen Formulierungen und die Wahl des Dopadecarboxylase-Inhibitors Priorisierungen im klinischen Einsatz gibt. Zudem gibt es seit Kurzem ein inhalatives Levodopa-Präparat [1], das zur Coupierung von Off-Phasen zugelassen ist.

Evidenzgrundlage

Es ergibt sich keine Evidenz dafür, dass „modified release"-Darreichungsformen von Levodopa mit Decarboxylasehemmer der „standard release"-Darreichungsform in der Behandlung früher Stadien der PK überlegen sind.

Ergebnis

Studien, die die in Deutschland verfügbaren Kombinationen aus Levodopa/Carbidopa und Levodopa/Benserazid in der Mengenrelation 4 : 1 vergleichen, sind nicht verfügbar, eine Präferenz für einen Dopadecarboxylase-Hemmer lässt sich aus der Literatur nicht ableiten.

In einer Studie aus dem Jahr 1999 wurde standard (immediate release) mit retardiertem (controlled-release) Levodopa/Carbidopa verglichen und innerhalb der 5 Jahre Beobachtungszeitraum kein Unterschied hinsichtlich Symptomkontrolle, Entwicklung von Fluktuationen oder Dyskinesien gefunden [2]. Zur Coupierung von Off-Phasen gibt es ein inhalatives Levodopa-Präparat (ohne Dopadecarboxylase-Inhibitor, das wegen der Umgehung des Mages-Darm-Trakts schnell in die Blutbahn aufgenommen wird und sich deswegen zur Coupierung von Off-Phasen bei Pat. eignet, die bereits Levodopa plus einen Dopadecarboxylase-Hemmer erhalten [1].

Begründung der Empfehlung

Wegen fehlender Vergleichsstudien kann keine Empfehlung hinsichtlich einer Präferenz des Dopa-decarboxylase-Inhibitors gegeben werden. Retardierte Präparate haben hinsichtlich der Symptomkontrolle, Motorfluktuationen oder Dyskinesien keinen Vorteil gegenüber Standard-

Release-Präparaten. Wegen der längeren Resorptionszeiten und der damit verbundenen längeren Zeiten von Nahrungskarenz und des höheren Risikos einer Interferenz von Nahrungsaufnahme mit der Levodopa-Resorption sollten retardierte Präparate nicht während der Wachzeit, sondern nur während der Nachtzeit gegeben werden. Schnell lösliche orale Levodopa-Präparate eignen sich zur besonders zur Coupierung von Off-Phasen. Das inhalative Levodopa-Präparat eignet sich zur Coupierung von Off-Phasen, allerdings wegen des fehlenden Dopadecarboxylase-Inhibitors nur bei Pat., die bereits Levodopa plus Dopadecarboxylase-Hemmer erhalten.

Empfehlung	Modifiziert<br>Stand (2023)
▪ Levodopa-Präparate können unter Beachtung der unten dargelegten Differenzialindikationen zur Therapie der PK eingesetzt werden.<br>▪ Eine Priorisierung der Präparate auf der Basis des Dopadecarboxylase-Inhibitors (Carbidopa oder Benserazid) kann aus der Literatur nicht abgeleitet werden.<br>▪ Retardierte Darreichungsformen von Levodopa mit Decarboxylase-Hemmer sollen nicht zur Therapie von Parkinson-Pat. während der Wachzeit verwendet werden, sondern nur zur Behandlung der Parkinson-Symptome während der Nachtzeit.<br>▪ Schnell lösliche orale und inhalative Levodopa-Formulierungen können zur Coupierung von Off-Situationen eingesetzt werden; inhalatives Levodopa kann aber nur **bei Pat. eingesetzt werden, die mit einem oralen Levodopa-Präparat behandelt werden,** da es nicht mit einem Dopadecarboxylase-Hemmer kombiniert ist.	
Konsensstärke: 92,9%, Konsens	

Fragestellung 35: Welche Eigenschaften beeinflussen die Priorisierung der einzelnen Dopaminagonisten für individuelle Pat.?

Hintergrund

Grundsätzlich sind non-ergoline Dopaminagonisten (Pramipexol, Ropinirol, Piribedil, Rotigotin, mit starken Einschränkungen Apomorphin) als Therapie der ersten Wahl zugelassen.

Die Dopaminagonisten Piribedil, Pramipexol und Ropinirol sind als Tabletten verfügbar, wobei von Pramipexol und Ropinirol neben der Standardformulierung auch Retard-Formulierungen verfügbar sind, die eine einmalige tägliche Gabe ermöglichen. Rotigotin ist als transdermales Pflaster verfügbar, das einmal täglich gewechselt wird. Apomorphin ist zur subkutanen Injektion oder Infusion bzw. als sublingual anwendbarer Film [3] verfügbar. Es stellt sich die Frage, ob es hinsichtlich der Wirksamkeit, des Nebenwirkungsprofils, Interaktionen oder Kontraindikationen Unterschiede zwischen den Präparaten gibt, die einen prioritären Einsatz bei bestimmten Pat.-Gruppen rechtfertigen würden.

Fibrotische Veränderungen retroperitoneal und im Bereich der Herzklappen sind für alle ergolinen Dopaminagonisten mit Ausnahme von Lisurid als Nebenwirkung belegt. Diese sind in der Regel

irreversibel. Der Einsatz ergoliner Dopaminagonisten wird daher grundsätzlich nicht mehr empfohlen.

Evidenzgrundlage

Es gibt keine rezenten kontrollierten Studien, in denen die Wirksamkeit bzw. Nebenwirkungen der einzelnen Dopaminagonisten miteinander verglichen wurden. In einer rezenten Metaanalyse wurden relative Effektivitätswerte u.a. von vier Dopaminagonisten berechnet, davon die drei nicht ergolinen Agonisten Pramipexol, Ropinirol und Rotigotin [4]. Es gibt eine frühere Doppel-Dummy-Studie, in der Pramipexol mit Rotigotin verglichen wurde [5]. Bezüglich möglicher Interaktionen wurden die Informationen zur Verstoffwechslung aus den Fachinformationen entnommen.

Ergebnis

In der Metaanalyse, in der u.a. die Dopaminagonisten Pramipexol, Ropinirol und Rotigotin untersucht wurden, ergaben sich Anhalte dafür, dass möglicherweise Ropinirol die höchste Effektivität der untersuchten Dopaminagonisten haben könnte (Effektivitätswert: Ropinirol: 2,171, (95%-KI: 1,888-2,489); Pramipexol (1,774; 1,607-1,958), Rotigotin (1,745; 1,514-2,009) [4]. In der Doppel-Dummy-Studie, die Pramipexol und Rotigotin verglichen hat, traten unter der Therapie mit Rotigotin häufiger Hautreaktionen auf, während sich keine Anhalte für eine unterschiedliche Wirksamkeit ergaben [5].

Piribedil wird in der Leber metabolisiert und großteils über den Urin ausgeschieden. Daten zu Pat. mit Leber- und/oder Niereninsuffizienz liegen nicht vor (Fachinformation).

Rotigotin wird über verschiedene CYP-Isoenzyme metabolisiert und dann zum großen Teil über die Niere ausgeschieden. Bei mittelschwerer Leberfunktionsstörung oder schwerer Nierenfunktionsstörung ist keine Dosisanpassung erforderlich. Bei akuter Verschlechterung der Nierenfunktion kann es zu einer unerwarteten Akkumulation von Rotigotin kommen (Fachinformation).

Pramipexol wird nur in geringem Maße metabolisiert und zu 90% über die Niere ausgeschieden. Bei Pat. mit eingeschränkter Nierenfunktion (Kreatinin-Clearance >30, < 50 mg/min) sollte eine Therapie mit 0,26 mg jeden 2. Tag begonnen werden. Für eine Kreatinin-Clearance < 30 mg/min liegen keine Daten vor (s. Fachinformation).

Ropinirol wird über das Cytochrom P450-Isoenzym CYP1A2 verstoffwechselt. Eine Dosisanpassung bei mäßiger Nierenfunktionsstörung (Kreatinin-Clearance >30 mg/min) ist nicht erforderlich. Für eine Kreatinin-Clearance < 30 mg/min liegen keine Daten vor. Bei Pat. mit terminaler Niereninsuffizienz und Hämodialyse ist die empfohlene Anfangsdosis der retardierten Formulierung 2 mg (s. Fachinformation).

Begründung der Empfehlung

Es existiert keine Evidenz, dass ergoline Dopaminagonisten Wirksamkeit zeigen, wenn andere zugelassene Substanzen oder Maßnahmen keine zufriedenstellende Einstellung ermöglichen. Ergoline Dopaminagonisten haben eine mit nicht ergolinen Dopaminagonisten vergleichbare Wirksamkeit, jedoch ein ungünstigeres Nebenwirkungsprofil. Ihr Einsatz wird deshalb nicht

empfohlen. Die Evidenzlage für einen Direktvergleich der vier Dopaminagonisten Piribedil, Pramipexol, Ropinirol, Rotigotin ist dünn. Beweise für eine unterschiedliche Wirksamkeit gibt es nicht. Lediglich eine Metaanalyse liefert Hinweise darauf, dass Ropinirol möglicherweise effektiver ist als Pramipexol und Rotigotin. Bezüglich der Nebenwirkungen treten unter Rotigotin häufiger dermale Nebenwirkungen auf. Bezüglich anderer Nebenwirkungen kann bei fehlenden Vergleichsstudien keine Aussage getroffen werden.

Hinsichtlich des Interaktionspotenzials muss bei Ropinirol beachtet werden, dass es über das Cytochrom P450-Isoenzym CYP1A2 verstoffwechselt wird.

Empfehlung	Neu<br>Stand (2023)
▪ Ergoline Dopaminagonisten (Bromocriptin, Cabergolin, Pergolid) sollen nicht mehr zur Therapie der PK eingesetzt werden.<br>▪ Non-ergoline Dopaminagonisten (Pramipexol, Ropinirol, Piribedil, Rotigotin, mit starken Einschränkungen Apomorphin) können unter Beachtung der unten dargelegten Differenzialindikationen zur Therapie der PK eingesetzt werden.<br>▪ Apomorphin steht zur s.c. Injektion oder Infusion bzw. als sublingual anwendbarer Film zur Verfügung und ist daher an spezifische Indikationen gebunden.<br>▪ Pramipexol und Ropinirol als Tabletten in Retard-Formulierungen und Rotigotin als transdermales Pflaster ermöglichen eine einmal tägliche Gabe.<br>▪ Eine Priorisierung der verschiedenen Dopaminagonisten untereinander hinsichtlich der Wirksamkeit kann nicht eindeutig aus der Literatur abgeleitet werden.<br>▪ Bei begleitender Medikation mit Medikamenten, die *CYP1A2* induzieren oder hemmen, sollte eine Dosisanpassung von Ropinirol oder ein Wechsel auf einen anderen Dopaminagonisten erwogen werden.<br>▪ Bei eingeschränkter Leberfunktion sollte erwogen werden, Pramipexol, das zum größten Teil über die Niere verstoffwechselt wird, zu priorisieren.<br>▪ Bei eingeschränkter Nierenfunktion sollte erwogen werden, Ropinirol, Rotigotin oder Piribedil gegenüber Pramipexol zu priorisieren.	
Konsensstärke: 96%, starker Konsens	

Fragestellung 36: Welche Eigenschaften beeinflussen die Priorisierung der einzelnen COMT-Hemmer (Entacapon, Tolcapon, Opicapon) für individuelle Pat.?

Hintergrund

Zur Therapie der PK stehen mit Entacapon, Tolcapon und Opicapon drei COMT-Hemmer zur Verfügung. Entacapon wird bis zu zehnmal täglich zu jeder Levodopa-Einnahme dazugegeben. Tolcapon wird bis zu dreimal täglich gegeben. Opicapon war zur Zeit der Erstellung der letzten Version der Leitlinien noch nicht verfügbar. Opicapon wird nur einmal täglich abends gegeben.

Evidenzgrundlage

Es existieren 2 Phase-III-Studien, in denen gezeigt wurde, dass Opicapon in der Dosis von 50 mg die Off-Zeit reduziert [6], [7]. Es gibt eine Metaanalyse, in der die Wirksamkeit der 3 COMT-Hemmer vergleichend analysiert wurde [8], und einen aktuellen Review-Artikel [9].

Ergebnis

In der ersten Phase-III-Studie wurden 600 Pat. in 5 Gruppen randomisiert (Placebo, Entacapon 200 mg, Opicapon 5 mg, Opicapon 25 mg, Opicapon 50 mg). In der Dosis von 50 mg führte Opicapon zu einer signifikanten Reduktion der Off-Zeit, während die Ergebnisse für 5 mg und 25 mg nicht signifikant waren. Im Vergleich zu Entacapon ergab sich ein nicht signifikanter Trend (p=0,051) dazu, dass Opicapon zu einer größeren Off-Zeit-Reduktion führte als Entacapon [7]. In der zweiten Studie wurden 427 Pat. in 3 Gruppen (Placebo, Opicapon 25 mg, Opicapon 50 mg) randomisiert. 50 mg Opicapon führten zu einer signifikanten Reduktion der Off-Zeit, während der Effekt für 25 mg nicht signifikant war [6].

In der rezenten Metaanalyse wurde gefunden, dass Entacapon, Opicapon und Tolcapon im Vergleich zu Placebo alle zu einer signifikanten Verlängerung der On-Zeit und zu mehr Dyskinesien führen. Tolcapon zeigte, verglichen mit Entacapon und Opicapon, die beste Effizienz hinsichtlich der Verlängerung der On-Zeit. Unter Opicapon war die Verlängerung der On-Zeit größer als unter Entacapon, wenngleich nicht statistisch signifikant. Die Nebenwirkungsrate war unter Tolcapon am höchsten. Auch Entacapon zeigte im Vergleich zu Placebo signifikant häufiger Nebenwirkungen, während bei Opicapon die Nebenwirkungsrate nicht signifikant höher war als bei Placebo [8].

Alle COMT-Hemmer sind wirksam hinsichtlich der Reduktion der Off-Zeit. Möglicherweise hat Opicapon gegenüber Entacapon eine etwas größere Wirksamkeit hinsichtlich der Reduktion der Off-Zeit. Während Entacapon zu jeder Einnahme von Levodopa ergänzt wird, wird Opicapon nur einmal am Tag gegeben mit Empfehlung, Opicapon mindestens eine Stunde vor oder nach Levodopa-Kombinationspräparaten einzunehmen (s. Fachinformation Opicapon). Das hat zur Folge, dass bei Therapie mit Opicapon ein zusätzlicher Medikamenteneinnahmezeitpunkt erforderlich ist. Eine direkte Vergleichsstudie der drei COMT-Hemmer ist nicht verfügbar.

Die häufigsten Nebenwirkungen von Entacapon sind Übelkeit, Bauchschmerzen, Diarrhoe, Urinverfärbung (harmlos). Die häufigsten Nebenwirkungen von Opicapon sind Übelkeit, Obstipation, Insomnie, trockener Mund und Hypertension. Die häufigsten Nebenwirkungen von Tolcapon sind Übelkeit, Diarrhoe, Halluzinationen, Leberwertanstieg, Urinverfärbung und Schwindel (dizzyness). Die Nebenwirkung Diarrhoe tritt bei Entacapon und Tolcapon häufiger auf als bei Opicapon [9].

Begründung der Empfehlung

Es gibt keine direkte Vergleichsstudie der COMT-Hemmer untereinander. Tolcapon ist möglicherweise etwas effizienter als Opicapon und Entacapon, aber potenziell lebertoxisch. Opicapon ist am besten verträglich und hat eine nicht signifikante Tendenz zu besserer Effizienz als Entacapon.

Empfehlung	Neu<br>Stand (2023)
▪ Opicapon und Entacapon sind als COMT-Hemmer in der Wirkung weitgehend gleichwertig und können unter Beachtung der unten dargelegten Differenzialindikationen zur Behandlung von Fluktuationen bei der PK eingesetzt werden.<br>▪ Tolcapon sollte wegen der Hepatotoxizität nur als Mittel der zweiten Wahl und unter engmaschiger Sicherheitsmonitorierung (klinisch und laborchemisch) verwendet werden.	
Konsensstärke: 100%, starker Konsens	

Fragestellung 37: Welche Eigenschaften beeinflussen die Priorisierung der einzelnen MAO-B-Hemmer (Selegelin, Rasagilin, Safinamid) für individuelle Pat.?

Hintergrund

Die MAO-B-Hemmer Rasagilin und Selegilin sind etabliert in der Monotherapie der PK. Die einmal indizierte Gabe kann im Krankheitsverlauf fortgeführt werden, wenn der MAO-B-Hemmer entweder eine relevante symptomatische Wirkung in dieser Phase der Erkrankung hat oder motorische Fluktuationen und/oder Dyskinesien reduziert. Safinamid hat einen dualen Wirkmechanismus, indem er bei beiden zugelassenen Dosierungen (50 mg und 100 mg) die MAO-B inhibiert und bei der höheren Dosierung zusätzlich glutamaterg wirkt. Safinamid ist für die Monotherapie nicht zugelassen, sondern nur für die Kombinationstherapie mit Levodopa bei Wirkfluktuationen.

Evidenzgrundlage

Bezüglich der Therapie der PK mit MAO-B-Hemmer existieren die meisten Daten für Rasagilin (siehe auch Kapitel über die initiale Monotherapie). Es existieren keine direkten Vergleichsstudien, in denen die MAO-B-Hemmer Rasagilin und Selegilin miteinander verglichen wurden. Safinamid ist, wie gesagt, nicht für die Monotherapie zugelassen.

Ergebnis

Eine Präferenz für einen MAO-B-Hemmer lässt sich aus der Literatur nicht eindeutig ableiten.

Begründung der Empfehlung

Weil sich aus der Literatur keine eindeutige Präferenz für den Einsatz eines MAO-B-Hemmers ableiten lässt, kann keine Priorisierung empfohlen werden. Bezüglich der Differenzialindikationen (Rasagilin, Selegilin in der Monotherapie und im fortgeschrittenen Stadium, Safinamid im fortgeschrittenen Stadium nur in der Kombinationstherapie) sei auf die entsprechenden Kapitel (3.2, 3.3, 3.4) verwiesen.

Empfehlung	Neu<br>Stand (2023)
▪ Eine Priorisierung der verschiedenen MAO-B-Hemmer untereinander hinsichtlich der Wirksamkeit kann aus der Literatur nicht eindeutig abgeleitet werden.<br>▪ Die MAO-B-Hemmer Selegilin oder Rasagilin können unter Beachtung der unten dargelegten Differenzialindikationen als Monotherapie der frühen PK oder in Kombination mit Levodopa zur Therapie der PK mit Wirkfluktuationen eingesetzt werden.<br>▪ Der MAO-B-Hemmer Safinamid mit dualem Wirkmechanismus ist nicht als Monotherapeutikum zugelassen, kann aber in Kombination mit Levodopa unter Beachtung der unten dargelegten Differenzialindikationen zur Therapie der PK mit Wirkfluktuationen eingesetzt werden.	
Konsensstärke: 100%, starker Konsens	

Fragestellung 38: Welche Eigenschaften beeinflussen die Priorisierung der einzelnen NMDA-Rezeptorantagonisten (Amantadin, Budipin) für individuelle Pat.?

Hintergrund

Theoretisch stehen die NMDA-Rezeptorantagonisten Amantadin und Budipin für die Therapie der PK zur Verfügung.

Evidenzgrundlage

Es existieren eine systematische Analyse aus dem Jahr 2003 [10] und ein rezenter Review [11] zu Amantadin in der Therapie der PK. Budipin ist wegen potenzieller schwerer kardialer Nebenwirkungen nicht mehr auf dem Markt.

Ergebnis

Amantadin hat einen positiven Effekt auf Levodopa-induzierte Dyskinesien (siehe Kapitel 3.5.). Für die Wirksamkeit und Sicherheit von Amantadin in der Frühphase der PK konnte keine ausreichende Evidenz gefunden werden [10], [11].

Begründung der Empfehlung

Amantadin ist der einzige NMDA-Rezeptor-Antagonist auf dem Markt. Es hat eine positive Wirkung auf Levodopa-induzierte Dyskinesien (siehe entsprechendes Kapitel) und keine nachgewiesene symptomatische Wirksamkeit in Frühstadien der PK.

Empfehlung	Neu<br>Stand (2023)
▪ Amantadin kann unter Beachtung der unten dargelegten Differenzialindikationen zur Therapie der PK eingesetzt werden.<br>▪ Budipin wird wegen seines Nebenwirkungsprofils nicht mehr empfohlen.	

Konsensstärke: 95,2%, starker Konsens

Fragestellung 39: Welche Eigenschaften beeinflussen die Priorisierung der einzelnen Anticholinergika (Biperiden, Bornaprin, Metixen, Trihexpyhenidyl) für individuelle Pat.?

Hintergrund

Anticholinergika (Biperiden, Bornapirin, Metixen, Trihexyphenidyl) sind als Medikamente zur Therapie der PK zugelassen.

Evidenzgrundlage

Weil seit der letzten Leitlinienerstellung keine randomisierten Studien, in denen Anticholinergika analysiert wurden, veröffentlicht wurden, hat sich die Evidenzlage seit der letzten Leitlinienerstellung nicht geändert.

Ergebnis

Weil seit der letzten Leitlinienerstellung keine randomisierten Studien, in denen Anticholinergika analysiert wurden, veröffentlicht wurden, hat sich die Evidenzlage seit der letzten Leitlinienerstellung nicht geändert.

Begründung der Empfehlung

Es existieren nur unzureichende Daten aus randomisierten Studien zu Wirksamkeit und Verträglichkeit von Anticholinergika bei der PK. Ihre Wirksamkeit ist aufgrund der vorliegenden Datenlage nicht hinreichend quantifizierbar, sie scheint aber insgesamt eher schwach ausgeprägt zu sein. Das trifft auch für die Indikation des Tremors zu. Bezüglich der Details sei auf das entsprechende Kapitel verwiesen.

Empfehlung	Modifiziert<br>Stand (2023)
▪ Anticholinergika sollen aufgrund eines im Vergleich zu Therapiealternativen ungünstigen Nutzen-Risiko-Profils nicht als Anti-Parkinson-Mittel eingesetzt werden.<br>▪ Nur noch in absoluten Ausnahmefällen kann ein Einsatz bei Tremor erwogen werden (siehe Kapitel 3.6).	
Konsensstärke: 96,4%, starker Konsens	

Fragestellung 40: Welche Dosierungsrichtlinien gelten für die einzelnen Parkinson-Medikamente (Dopaminagonisten, MAO-B-Hemmer, COMT-Hemmer, Amantadin, Anticholinergika)?

Hintergrund

Die verschiedenen Medikamente gegen die Symptome der PK sind in verschiedenen Dosierungen verfügbar. Daraus ergibt sich die Frage hinsichtlich der Eindosierungsschemata.

Evidenzgrundlage

In der vorhergehenden Version dieser Leitlinien gab es Tabellen mit Dosierungsrichtlinien, die sich für die bis dahin bekannten Medikamente nicht geändert haben. Weil diese grundsätzlich nicht mehr eingesetzt werden sollten, wurden die Eindosierungsrichtilinien für die ergolinen Dopaminagonisten entfernt.

Bezüglich der Dosierung von Rasagilin wurde seit der letzten Leitlinienerstellung eine Metaanalyse veröffentlicht [12]. Bezüglich der Dosierung von Safinamid wurden eine Phase I/III-Studie und eine Metaanalyse verwendet [13], [14].

Ergebnis

In einer Metaanalyse, in der Studien zu Rasagilin analysiert wurden, bei denen Rasagilin in einer Dosis von 1 mg oder 2 mg einmal täglich gegeben wurde, bestätigte sich, dass 1 mg und 2 mg Rasagilin zu einer Verbesserung des UPDRS Teil II und III führen, während nur die 1-mg-Dosierung zu einer Verbesserung des UPDRS Teil I führte. Darüber hinaus gibt es keinen Beweis für eine bessere Wirksamkeit auf die Motorik von 2 mg gegenüber 1 mg [12]. Zusammenfassend sollte Rasagilin nur mit einer Standarddosis von 1 mg gegeben werden.

Selegilin ist als Tabletten zu 5 mg bzw. 10 mg verfügbar. Die Halbwertszeit umfasst mehrere Tage und eine Dosis-Wirkungs-Beziehung ist aus den verfügbaren Studien nicht ableitbar.

In einer Metaanalyse wurden Studien zu Safinamid untersucht, bei denen Tagesdosierungen zwischen 50 und 200 mg angewendet wurden. Dabei ist die Datenlage für 200 mg sehr dünn. Insgesamt werden Dosierungen zwischen 50 und 100 mg als Add-on-Therapie zu Levodopa empfohlen, ein dosisabhängiger Wirksamkeitsunterschied konnte nicht gezeigt werden [13], [14]. Wichtigste Nebenwirkungen waren Dyskinesien und visuelle Halluzinationen.

Tab. 6: Dosierungsrichtlinien für die Therapie mit Dopaminagonisten.

Substanz	Beginn	Wöchentliche Steigerung	Erhaltungsdosis	Tages-Gesamtdosis
**Non-Ergot-Dopaminagonist**				
Piribedil	50 mg abends	50 mg alle 2 Wochen	2- bis 3-mal 50 mg, bis 100 - 50 - 100 mg	150–250 mg
Pramipexol Standard	3-mal 0,088 mg	2. Woche:<br>3-mal 0,18 mg<br>3. Woche:<br>3-mal 0,35 mg<br>Weiter wöchentl. um 3-mal 0,18 mg	3-mal 0,35–0,7 mg	1,05–3,3 mg
Pramipexol Retard	0,26 mg morgens	2. Woche:<br>1-mal 0,52 mg<br>3. Woche:<br>1-mal 1,05 mg<br>Weiter wöchentl. auf:<br>1-mal 2,1 mg<br>1-mal 3,15 mg	1-mal 1,05–2,1 mg	1,05–3,15 mg

Substanz	Beginn	Wöchentliche Steigerung	Erhaltungsdosis	Tages-Gesamtdosis
Ropinirol Standard	1 mg morgens	1 mg;<br>ab 6 mg: 1,5–3 mg	3-mal 3–8 mg	6–24 mg
Ropinirol Retard	2 mg morgens	2 mg	1-mal 6–24 mg	6–24 mg
Rotigotin trans-dermal	2 mg/24 h	2 mg/24 h	4–8 mg/24 h (Frühstadium)<br>8–16mg/24h (fortge-schrittenes Stadium)	6–16 mg/24 h

Tab. 7: Dosierung von MAO-B-Hemmern

Substanz	Beginn	Wöchentliche Steigerung	Erhaltungsdosis	Tages-Gesamtdosis
Rasagilin	1 mg	keine	1-mal 1 mg	1 mg
Selegilin	5 mg	5 mg	1-2-mal 5 mg/<br>1-mal 10 mg	5-10 mg
Safinamid	50 mg	Bei Off-Symptomen: 50–100 mg<br>Bei Dyskinesien: 100 mg	1-mal 50-100 mg	50-100 mg

Diese Eindosierungsempfehlungen gelten im ambulanten Bereich für behandelnde Personen, die nicht speziell auf Bewegungsstörungen spezialisiert sind. Im klinischen Setting oder in Spezialzentren für Bewegungsstörungen sind auch schnellere Eindosierungen möglich.

Fragestellung 41: Welches ist die Levodopa-Äquivalenzdosis der einzelnen Parkinson-Medikamente (Dopaminagonisten, MAO-B-Hemmer, COMT-Hemmer, Amantadin, Anticholinergika)?

Hintergrund

Die verfügbaren Medikamente gegen die Symptome der PK unterscheiden sich deutlich in ihrem eingesetzten Dosisbereich, sodass sich die Frage nach der Äquivalenzdosis stellt.

Evidenzgrundlage

Bezüglich der Äquivalenzdosis wurde in der letzten Version der Leitlinien eine Tabelle aus Tomlinson et al., 2010 verwendet [15]. Diese wurde in einer Arbeit von Schade et al. aus dem Jahr 2021 um neuere Medikamente (langwirksames Levodopa, Opicapon, Safinamid) ergänzt [16]. Darüber hinaus gibt es einen aktuellen systematischen Review, der Handlungsempfehlungen hinsichtlich der Äquivalenzdosen enthält [17]. Die Zusammenstellung der Tabellen wurde in diese Leitlinie übernommen.

Ergebnis

Tab. 8: Äquivalenzdosen (Referenzen 14–16)

Medikamentenklasse	Medikament	Einzeldosen (mg/100 mg L-Dopa)	Multiplikator
L-Dopa	L-Dopa (LD)	100	1
	Retardiertes L-Dopa	133	0,75
	Levodopa/Carbidopa-Intestinalgel	90	1,11<br>(Morgendosis und Erhaltungsdosis)
	Levodopa/Carbidopa/Entacapon-Intestinalgel	90<br>70	1,11 (Morgendosis)<br>1,46 (Erhaltungsdosis)
COMT-Hemmer*	Entacapon	LD x 0.33	LD x 0,33
	Tolcapon	LD x 0.5	LD x 0,5
	Opicapon	LD x 0.5	LD x 0,5
Dopaminagonisten<br>(Non-Ergot)	Pramipexol	1 mg Salz	100
	Ropinirol	5	20
	Rotigotin	3,3	30
	Piribedil	100	1
	Apomorphin (s.c. Infusion oder s.c. Injektion)	10	10
MAO-B-Hemmer	Selegilin 10 mg (oral)	10	10
	Selegilin 1,25 mg (sublingual)	1,25	80
	Rasaglin	1	100
	Safinamid	100 - 150	0,66 - 1
andere	Amantadin	100	1

In der Tabelle sind die Wirkstoffgruppen, die Wirkstoffe, die Äquivalenzdosis zu 100 mg Levodopa sowie der Einfachheit halber der Multiplikator, mit dem die Dosis des Wirkstoffs multipliziert werden muss, um die Äquivalenzdosis zu berechnen, genannt.

*Um die Äquivalenzdosis von COMT-Hemmern zu berechnen, wird die gesamte Levodopa-Dosis (inklusive retadierten Levodopas) mit dem entsprechenden Wert multipliziert und zu der Gesamtdosis von Levodopa addiert.

Referenzen

1. Paik J. Levodopa Inhalation Powder: A Review in Parkinson's Disease. Drugs. 2020;80(8):821-8
2. Koller WC, Hutton JT, Tolosa E, Capilldeo R. Immediate-release and controlled-release carbidopa/levodopa in PD: a 5-year randomized multicenter study. Carbidopa/Levodopa Study Group. Neurology. 1999;53(5):1012-9
3. Olanow CW, Factor SA, Espay AJ, Hauser RA, Shill HA, Isaacson S, et al. Apomorphine sublingual film for off episodes in Parkinson's disease: a randomised, double-blind, placebo-controlled phase 3 study. Lancet Neurol. 2020;19(2):135-44
4. Binde CD, Tvete IF, Gåsemyr JI, Natvig B, Klemp M. Comparative effectiveness of dopamine agonists and monoamine oxidase type-B inhibitors for Parkinson's disease: a multiple treatment comparison meta-analysis. Eur J Clin Pharmacol. 2020;76(12):1731-43
5. Poewe WH, Rascol O, Quinn N, Tolosa E, Oertel WH, Martignoni E, et al. Efficacy of pramipexole and transdermal rotigotine in advanced Parkinson's disease: a double-blind, double-dummy, randomised controlled trial. Lancet Neurol. 2007;6(6):513-20
6. Lees AJ, Ferreira J, Rascol O, Poewe W, Rocha JF, McCrory M, Soares-da-Silva P. Opicapone as Adjunct to Levodopa Therapy in Patients With Parkinson Disease and Motor Fluctuations: A Randomized Clinical Trial. JAMA Neurol. 2017;74(2):197-206
7. Ferreira JJ, Lees A, Rocha JF, Poewe W, Rascol O, Soares-da-Silva P. Opicapone as an adjunct to levodopa in patients with Parkinson's disease and end-of-dose motor fluctuations: a randomised, double-blind, controlled trial. Lancet Neurol. 2016;15(2):154-65
8. Song Z, Zhang J, Xue T, Yang Y, Wu D, Chen Z, et al. Different Catechol-O-Methyl Transferase Inhibitors in Parkinson's Disease: A Bayesian Network Meta-Analysis. Front Neurol. 2021;12:707723
9. Fabbri M, Ferreira JJ, Rascol O. COMT Inhibitors in the Management of Parkinson's Disease. CNS Drugs. 2022;36(3):261-82
10. Crosby N, Deane KH, Clarke CE. Amantadine in Parkinson's disease. Cochrane Database Syst Rev. 2003;2003(1):Cd003468
11. Rascol O, Fabbri M, Poewe W. Amantadine in the treatment of Parkinson's disease and other movement disorders. Lancet Neurol. 2021;20(12):1048-56
12. Chang Y, Wang LB, Li D, Lei K, Liu SY. Efficacy of rasagiline for the treatment of Parkinson's disease: an updated meta-analysis. Ann Med. 2017;49(5):421-34
13. Hattori N, Kogo Y, Koebis M, Ishida T, Suzuki I, Tsuboi Y, Nomoto M. The Effects of Safinamide Adjunct Therapy on Depression and Apathy in Patients With Parkinson's Disease: Post-hoc Analysis of a Japanese Phase 2/3 Study. Front Neurol. 2021;12:752632
14. Giossi R, Carrara F, Mazzari M, Lo Re F, Senatore M, Schicchi A, et al. Overall Efficacy and Safety of Safinamide in Parkinson's Disease: A Systematic Review and a Meta-analysis. Clin Drug Investig. 2021;41(4):321-39
15. Tomlinson CL, Stowe R, Patel S, Rick C, Gray R, Clarke CE. Systematic review of levodopa dose equivalency reporting in Parkinson's disease. Mov Disord. 2010;25(15):2649-53
16. Schade S, Mollenhauer B, Trenkwalder C. Levodopa Equivalent Dose Conversion Factors: An Updated Proposal Including Opicapone and Safinamide. Mov Disord Clin Pract. 2020;7(3):343-5
17. Jost ST, Kaldenbach MA, Antonini A, Martinez-Martin P, Timmermann L, Odin P, et al. Levodopa Dose Equivalency in Parkinson's Disease: Updated Systematic Review and Proposals. Mov Disord. 2023;38(7):1236-52

3.2 Initiale Monotherapie

Autoren/Autorinnen: Matthias Höllerhage, Günter Höglinger, Claudia Trenkwalder

Fragestellung 42: Wann ist die Indikation zur Pharmakotherapie der PK gegeben?

Hintergrund

Die Indikation für eine Pharmakotherapie der PK richtet sich in erster Linie nach den bei der betroffenen Person bestehenden funktionellen Einschränkungen bei Alltagstätigkeiten. Ziel der initialen Monotherapie im frühen Stadium der PK muss ein individueller Einsatz der für diese Indikation verfügbaren Substanzklassen (Dopaminagonisten, Levodopa, MAO-B-Hemmer) zur symptomatischen Kontrolle der Funktionsbeeinträchtigung sein. Dies hat unter einer sinnvollen Risiko-Nutzen-Abwägung zu gelten.

Evidenzgrundlage

Es gibt weiterhin keinen Beweis für die Existenz einer krankheitsmodifizierenden Therapie, sodass der Wunsch nach einer Krankheitsmodifikation gegenwärtig keine Indikation für eine Pharmakotherapie der PK darstellt. Bezüglich der potenziell krankheitsmodifizierenden Therapien sei auf das entsprechende Kapitel in dieser Leitlinie verwiesen. Es gibt zudem keine systematischen Studien, die untersucht haben, wann die Indikation für eine Pharmakotherapie der PK zu stellen ist.

Ergebnis

Der Zeitpunkt, wann die Indikation für eine Pharmakotherapie der PK zu stellen ist, ist nicht durch randomisierte kontrollierte Studien untersucht worden.

Begründung der Empfehlung

Die Empfehlung der letzten Parkinson-Leitlinie soll weiterhin Bestand haben.

Empfehlung	Geprüft<br>Stand (2023)
Die Pharmakotherapie der PK sollte rechtzeitig, altersgerecht und effizient beginnen. Je nach Alter, Erkrankungsdauer und sozialer Situation können folgende Therapieziele relevant werden:<br>▪ Therapie von motorischen und/oder nicht motorischen und/oder autonomen Störungen<br>▪ Verhaltens- und psychologische Symptome der Erkrankung<br>▪ Erhaltung der Selbstständigkeit in den Aktivitäten des täglichen Lebens<br>▪ Verhinderung/Verminderung von Pflegebedürftigkeit<br>▪ Erhaltung der Selbstständigkeit in Familie und Gesellschaft (soziale Kompetenz)<br>▪ Erhaltung der Berufsfähigkeit<br>▪ Erhalt/Steigerung der Lebensqualität<br>▪ Vermeidung von sekundären orthopädischen und internistischen Begleiterkrankungen<br>▪ Verhinderung/Behandlung von motorischen und nicht motorischen Komplikationen	

▪ Vermeidung von dopaminergen Nebenwirkungen
Konsensstärke: 100%, starker Konsens

Fragestellung 43: Wie effektiv und sicher ist Standard-release-Levodopa im Vergleich mit Placebo in der Monotherapie der PK im Frühstadium?

Hintergrund

Die Standardtherapie für die PK besteht seit mehr als 30 Jahren aus Levodopa. Levodopa ist die biochemische Vorstufe von Dopamin, welches bei der PK im Gehirn reduziert ist. Levodopa wird durch Dopadecarboxylasen zu Dopamin umgewandelt. Um den peripheren Metabolismus von Dopamin zu reduzieren, wird Levodopa mit peripherem Dopadecarboxylase-Hemmer (Benserazid oder Carbidopa) kombiniert. Dies erhöht den Übertritt von Levodopa durch die Blut-Hirn-Schranke.

Levodopa-Präparate sind ursächlich an der Entwicklung von motorischen Komplikationen beteiligt. So treten in Abhängigkeit des Levodopa-Plasmaspiegels Wirkungsschwankungen (Fluktuationen) auf. Das Abklingen der Levodopa-Wirkung wird als „wearing-off“ oder „end of dose“-Akinesie bezeichnet. Die Pat. durchleben während dieser Fluktuationen abwechselnd On- und Off-Phasen. Am Anfang sind die Fluktuationen in der Regel vorhersehbar, im weiteren Verlauf ist das Auftreten der Fluktuationen jedoch häufig unvorhersehbar. Diese motorischen Fluktuationen sind von Dyskinesien überlagert. Letztere umfassen unwillkürliche, choreatiforme Bewegungen zum Zeitpunkt der maximalen Wirksamkeit von Levodopa (sogenannte Peak-dose-Dyskinesien – diese sind in der Regel ohne begleitende Schmerzen) oder Dystonien während der Anflutungs- und/oder Abflutungsphase der Levodopa-Wirkung (sogenannte monophasische oder biphasische, oft schmerzhafte Dystonien). Um das Auftreten motorischer Komplikationen zu vermeiden bzw. hinauszuzögern, wird diskutiert, den Einsatz von Levodopa bei jüngeren Parkinson-Pat. möglichst aufzuschieben. Für die Entwicklung von Dyskinesien spielt jedoch die Höhe der täglichen Levodopa-Dosis und vor allem die Erkrankungsdauer eine entscheidende Rolle. Viele, wenn nicht alle Pat. werden im Verlauf Levodopa für eine effektive Symptomkontrolle benötigen (ca. 50–90% innerhalb von 4–6 Jahren [1], [2]. Langfristig, d.h. über mehr als fünf Jahre Krankheitsdauer, kann die Mehrzahl der Parkinson-Pat. i.d.R. nicht auf ein Levodopa-Präparat verzichten.

Evidenzgrundlage

Die wesentliche Grundlage für die Wirksamkeit von Levodopa plus Dopadecarboxylase-Hemmer lieferte bisher die ELLDOPA-Studie [3]. Seit der letzten Leitlinie wurde eine neue randomisierte doppelblinde Studie veröffentlicht, die in diese Leitlinie Einzug erhalten hat [4].

Eine Reihe von Studien haben nahegelegt, dass die Levodopa-Therapie wegen der Entwicklung von Motorfluktuationen möglicherweise verzögert werden sollte [2]. Im Jahr 2014 hat eine Studie systematisch untersucht, wie der Zusammenhang zwischen Krankheitsdauer, Levodopa-Dosis, Therapiedauer, Alter und der Entwicklung von Motorfluktuationen und Dyskinesien ist. Dabei wurden Pat. aus Ghana mit solchen aus Italien verglichen [1].

Es wurde eine Metaanalyse gefunden, in der 14 RCTs analysiert worden waren, hinsichtlich der Frage nach dem Auftreten von Dyskinesien unter einer Therapie mit Levodopa. Dabei wurden Levodopa-Monotherapien oder Kombinationstherapien aus Levodopa mit Entacapon, Selegilin, Amantadin und Anticholinergika verglichen mit Dopaminagonisten allein oder als Kombinationstherapie. Die mittlere Behandlungsdauer betrug 46,5 Monate (IQR: 37,5-57) [5].

Ergebnis

Die ELLDOPA-Studie [3] war randomisiert, doppelblind und placebokontrolliert in 33 Zentren in den USA und Kanada durchgeführt worden (fünf der Zentren führten zusätzlich 123I-Beta-CIT-SPECT durch). 361 unbehandelte Parkinson-Pat. wurden in vier Therapiegruppen randomisiert, die drei unterschiedliche Dosierungen von Levodopa/Carbidopa oder Placebo erhielten: 1) 150/37,5 mg täglich (insgesamt 92 Pat.), 2) 300/75 mg täglich (insgesamt 88 Pat.), 3) 600/150 mg täglich (insgesamt 91 Pat.), 4) Placebo (insgesamt 90 Pat.).

Levodopa reduzierte dosisabhängig die Verschlechterung der Symptome des PK. Es kam zu einer signifikanten Änderung der UPDRS-Punktwerte von Baseline zu Woche 42 (versus Placebo): Gesamtwerte ($p < 0,001$). Die Lebensqualität, die motorischen und psychischen Domänen verbesserten sich ebenfalls signifikant. Die UPDRS-Werte verschlechterten sich in den drei Levodopa-Gruppen während der zweiwöchigen Auswaschphase. Sie sanken jedoch nicht auf den Wert der Placebogruppe ab. Die Gruppe mit der höchsten Levodopa-Dosis zeigte die größte Verbesserung im UPDRS-Wert.

In Bezug auf die Entwicklung von Motorfluktuationen und Dyskinesien hat die Studie aus dem Jahr 2014, die den Zusammenhang zwischen Krankheitsdauer, Levodopa-Dosis, Therapiedauer, Alter und der Entwicklung von Motorfluktuationen und Dyskinesien untersucht hat und in die 91 Pat. aus Ghana eingeschlossen und 1 : 2 mit 182 italienischen Pat. gematcht wurden, gefunden, dass die Levodopa-Gesamtdosis sowie die Krankheitsdauer die Faktoren waren, die zur Entwicklung von Motorkomplikationen und Dyskinesien beigetragen haben, während die Krankheitsdauer zu Beginn der Levodopa-Therapie keinen Einfluss hatte [1]. In der Metaanalyse aus dem Jahr 2018 wurden 14 RCTs untersucht. Es wurde gefunden, dass eine Levodopa-Monotherapie mit einem größeren Risiko für die Entwicklung von Levodopa-induzierten Dyskinesien (LID) vergesellschaftet ist im Vergleich zu anderen Parkinson-Therapien (OR=2,82; 95%-KI: 1,73-4,60). In der Subgruppenanalyse wurde gefunden, dass eine Levodopa-Monotherapie mit einem größeren Risiko für LID verbunden ist im Vergleich zu einer Monotherapie mit Dopaminagonisten (OR: 3,45; 95%-KI: 2,28-5,21) oder einer Kombinationstherapie aus Levodopa und Dopaminagonist (OR: 5,97; 95%-KI: 2,26-15,76). Im Vergleich einer Levodopa-Monotherapie mit einer Kombinationstherapie mit MAO-B-Hemmern ergab sich kein signifikanter Unterschied (OR: 0,81; 0,59-1,01). Für Pat. mit einer Monotherapie mit Levodopa, verglichen mit Pat., die nie Levodopa als Add-on erhalten hatten, war die OR für die Entwicklung von LID bei 3,27 (95%-KI: 2,24-4,78), verglichen mit Pat., die irgendwann einmal Levodopa als Add-on erhalten hatten, immerhin noch bei 2,82 (95%-KI: 1,73–4,60). Interessanterweise hatte dabei weder die Levodopa-Äquivalenzdosis noch das Alter der Pat. einen signifikanten Einfluss auf die Entwicklung von LID [5]. Die Autoren/Autorinnen gaben an, dass weiterhin kein Beweis dafür existiert, dass LID zeit- oder dosisabhängig sind.

In der seit der letzten Leitlinienerstellung erfolgten randomisierten, doppelblinden und placebokontrollierten Studie von Verschuur et al. aus dem Jahr 2019 wurde untersucht, ob Levodopa plus Dopadecarboxylase-Hemmer (DDCI) auch eine krankheitsmodifizierende Wirkung hat. In der Studie wurden zwei Pat.-Gruppen randomisiert und doppelblind in einem Delayed-start-Design verglichen. Eine Gruppe wurde über 80 Wochen mit Levodopa/Carbidopa 100/25 mg dreimal täglich behandelt (early start) und eine weitere zunächst 40 Wochen mit Placebo und danach für weitere 40 Wochen ebenfalls mit Levodopa/Carbidopa 100/25 mg dreimal täglich (delayed start). Insgesamt wurde 445 Pat. eingeschlossen mit dem primären Endpunkt UPDRS-Punktwert zwischen der Baseline und nach 80 Wochen. Sekundäre Endpunkte waren die Veränderung des UPDRS-Punktwerts pro Woche zwischen Woche 4 und Woche 40 sowie Unterschiede in anderen Bewertungsskalen (u.a. von Kognition, Depressivität und Lebensumständen).

Es wurden Pat. mit einer frühen PK (Diagnose innerhalb der letzten 2 Jahre) und ohne symptomatische Therapie eingeschlossen. Es zeigte sich nach 80 Wochen kein Unterschied in den zwei Gruppen, jedoch eine schlechtere Lebensqualität in der Gruppe, die zunächst kein Levodopa/ Dopa-decarboxylase-Hemmer erhalten hatte [4].

In dieser war nach 40 Wochen der Punktwert im UPDRS in der Early-start-Gruppe um 3,1 ± 10,2 signifikant niedriger im Vergleich zur Baseline, während in der Delayed-start-Gruppe der Punktwert um 2,0 ± 12,3 höher war im Vergleich zur Baseline. Insgesamt ergab sich in der Behandlungsgruppe somit eine Verbesserung im UPDRS-Punktwert von 5,1 (95%-KI: 2.9–7,2) im Vergleich zur bis dahin unbehandelten Delayed-start-Gruppe. Nach 80 Wochen unterschied sich der Punktwert der beiden Gruppen im Vergleich untereinander nicht (27,0 ± 14,8 für die Early-start-Gruppe und 27,0 ± 14,3 für die Delayed-start-Gruppe).

Die unerwünschten Nebenwirkungen unterschieden sich in der Häufigkeit bis auf das häufigere Auftreten von Übelkeit in der ersten Phase in der Early-start-Gruppe (23,0% gegenüber 14,3%, p = 0,02) nicht voneinander. In der Delayed-Start-Gruppe wechselten von 223 Pat. 87 verfrüht in die zweite Studienphase zu den „Behandelten“ (47 vor Woche 22, 40 vor Woche 40). In der Early-start-Gruppe wechselten von 222 Pat. 24 verfrüht in die zweite Studienphase (5 vor Woche 22, 19 vor Woche 40) [4].

Begründung der Empfehlung

Dass Levodopa die effektivste Behandlung der PK darstellt, hatte bereits die ELLDOPA-Studie aus dem Jahr 2004 gezeigt. Kurzzeitige dopaminerge Nebenwirkungen sind selten und meist vorübergehend. Dennoch kann die Langzeittherapie mit Levodopa dosisabhängig motorische Komplikationen wie Fluktuationen und Dyskinesien verursachen.

Dass Levodopa ursächlich an der Entwicklung von Wirkfluktuationen und Dyskinesien beteiligt ist, war bereits allgemein anerkannt. Eine rezente Metaanalyse konnte bestätigen, dass Pat., die mit einer Monotherapie mit Levodopa therapiert wurden, ein höheres Risiko für die Entwicklung von LID haben [5]. Der genaue Zusammenhang ist weiterhin nicht verstanden, auch weil die Autorinnen/Autoren der Metaanalyse keine Beweise für einen Dosis- oder Therapiedauer-Effekt liefern konnten. Trotzdem sollte die niedrigste effektive Dosis verwendet werden.

Die Studie von Verschuur et al. aus dem Jahr 2019 konnte bestätigen, dass Levodopa einen guten symptomatischen Effekt hat, gemessen am Unterschied im UPDRS zwischen der Early-start- und der Delayed-start-Gruppe nach 40 Wochen. Ein Anhalt für eine verlaufsmodifizierende Wirkung von Levodopa ergab sich nicht. Pat., die im Delayed-start-Design später mit Levodopa behandelt wurden, hatten nach der 40-wöchigen Behandlungsphase den gleichen Punktwert im UPDRS wie Pat., die über 80 Wochen behandelt worden waren.

**Empfehlung**	**Modifiziert<br>Stand (2023)**
▪ Levodopa kann unter Beachtung der unten dargelegten Differenzialindikationen zur Monotherapie der frühen PK eingesetzt werden.<br>▪ Von Levodopa soll eine möglichst niedrige, aber ausreichend wirksame Dosis eingesetzt werden.<br>▪ Levodopa hat auf der Basis der vorliegenden Evidenz keinen negativen oder positiven Einfluss auf die Krankheitsprogression.	
Konsensstärke: 91%, Konsens	

Fragestellung 44: Wie effektiv und sicher sind Dopaminagonisten im Vergleich mit Placebo oder Levodopa in der Monotherapie der PK im Frühstadium?

Hintergrund

Zur Monotherapie der PK stehen auch Dopaminagonisten zur Verfügung. Sie binden v.a. an striatale D2-Rezeptoren. Es wird angenommen, dass diese, insbesondere in der retardierten oder transdermalen Formulierung, eine weniger pulsatile Stimulation der Rezeptoren induzieren als Levodopa [6]. Es stellt sich die Frage, wie effektiv und sicher Dopaminagonisten in der Therapie der PK sind und wie diese hinsichtlich Effektivität und Sicherheit im Vergleich zu Levodopa abschneiden.

Grundsätzlich sind non-ergoline Dopaminagonisten (Pramipexol, Ropinirol, Piribedil, Rotigotin, mit starken Einschränkungen Apomorphin) als Therapie der ersten Wahl zugelassen.

Aus regulatorischen Gründen durften ergoline Dopaminagonisten bisher nur als Mittel der zweiten Wahl eingesetzt werden. Fibrotische Veränderungen retroperitoneal und im Bereich der Herzklappen sind für alle ergolinen Dopaminagonisten mit Ausnahme von Lisurid belegt. Sie sind in der Regel irreversibel. Der Einsatz ergoliner Dopaminagonisten wird daher grundsätzlich nicht mehr empfohlen.

Eine klinisch effektive Dosis entzieht sich zunächst einer operationalen Definition; im Alltag empfiehlt sich eine objektiv erfasste Verbesserung der Kardinalsymptome der PK (beispielsweise gemessen mit der MDS-UPDRS, motorische Subskala) um mindestens 30% bis über 50%. Dies ist mit allen non-ergolinen Dopaminagonisten grundsätzlich zu erreichen. Allerdings ist die subjektive Verträglichkeit von Dopaminagonisten manchmal eingeschränkt, sodass ein Medikamentenwechsel erforderlich ist. Aufgrund der Metaanalysen ist jedoch zu erwarten, dass ein Wechsel auf eine andere Substanzklasse

(MAO-B-Hemmer oder beispielsweise Levodopa) oder auf einen anderen Dopaminagonisten sinnvoll ist, da unterschiedliche Dopaminagonisten ein unterschiedliches Nebenwirkungsprofil haben können.

Evidenzgrundlage

Die Wirksamkeit von Dopaminagonisten gegenüber Placebo ist in zahlreichen zentralen Zulassungsstudien für die einzelnen Substanzen (zuletzt vornehmlich non-ergoline Dopaminagonisten) dargelegt. Die Wirksamkeit wird ferner unterstützt durch drei Metaanalysen, unter anderem der Cochrane-Arbeitsgruppe [7], sowie durch die Handlungsempfehlung der Movement Disorder Society [8]. Eine Studie (PD-MED), in der nicht primär die Wirksamkeit im Vordergrund stand, hat sich mit der Frage auseinandergesetzt, welche der möglichen Ersttherapien (Levodopa, Dopaminagonisten, MAO-B-Hemmer) zu Beginn gewählt werden sollten. In dieser wurden 1.620 Pat. 1:1:1 open-label auf eine Therapie mit Levodopa, Dopaminagonist oder MAO-B-Hemmer randomisiert und anhand von durch die Pat. ausgefüllte Fragebögen die motorische Funktion und die Lebensqualität sowie Kognition und Therapieabbruch analysiert [9].

Aus pragmatischen Gründen werden hier sowohl die Studien vorgestellt, die Dopaminagonisten mit Placebo oder Levodopa verglichen haben, als auch Studien, die neben den o.g. Medikamenten(klassen) zusätzlich MAO-B-Hemmer untersucht haben. Studien, die ausschließlich MAO-B-Hemmer untersucht haben, werden unter der entsprechenden Schlüsselfrage vorgestellt.

Seit der Erstellung der letzten Leitlinie wurden zwei Metaanalysen publiziert, in denen Daten zu Monotherapien mit Dopaminagonisten, MAO-B-Hemmern und Levodopa verglichen wurden. Eine Metaanalyse aus dem Jahr 2016 hat publizierte Daten zu placebokontrollierten Studien, die Monotherapien mit Levodopa, Dopaminagonisten und MAO-B-Hemmern untersucht haben, verglichen und statistisch analysiert [10].

In einer weiteren Metaanalyse aus dem Jahr 2020 wurden Studien analysiert, in denen Daten zum Therapieansprechen auf die Dopaminagonisten Cabergoline, Pramipexol, Ropinirol und Rotigotin und die MAO-B-Hemmer Selegilin, Rasagilin und Safinamid veröffentlicht wurden, analysiert und mit Daten für Levodopa verglichen. Anhand eines statistischen Modells wurden für die verschiedenen Therapien relative Effektivitätswerte berechnet [11].

Zudem wurden seit der letzten Leitlinienerstellung weitere Studien und Metaanalysen veröffentlicht, in denen einzelne Dopaminagonisten untersucht wurden, die in den folgenden Tabellen zusammengefasst werden. Der Vollständigkeit halber werden in den Tabellen auch Studien aufgeführt, die bereits in die letzte Leitlinie Einzug erhalten haben.

Tab. 9: Zusammenfassung von Metaanalysen und systematischen Reviews zur Effektivität von Dopaminagonisten im Frühstadium der PK

Studien	Anzahl der Studien (n)	Dopaminagonist vs. Placebo oder Levodopa	Methodische Schwächen und Bemerkungen
(Chen et al. 2017) [12]	12	Rotigotin vs. Placebo	
(Giladi et al. 2016) [13]	6	Rotigotin vs. Placebo,<br>Rotigotin plus Levodopa vs. Placebo	Einschluss von Studien mit Early-stage-Pat. (kein Levodopa erlaubt) und Studien mit Advanced-stage-Pat. (Levodopa als Einschlusskriterium)
(Shen and Kong 2018) [14]	3	Pramixol unretardiert vs. retardiert vs. Placebo	geringe Anzahl Studien, nur 2 Studien mit Placebogruppe
(Márquez-Cruz et al. 2016) [10]	5	Levodopa vs. Placebo<br>Pramipexol vs. Placebo<br>Rasagilin vs. Placebo<br>Selegilin vs. Placebo	nur Pramipexol als DA-Agonist untersucht
(Binde et al. 2020) [11]	79	4 DA-Agonisten (Cabergolin, Pramipexol, Ropinirol, Rotigotin) vs.<br>3 MAO-B-Hemmer (Selegilin, Rasagilin, Safinamid (als Add-on)) vs.<br>Levodopa vs. Placebo	In der Studie wurden relative Effektstärken berechnet.
(Zhuo et al. 2017) [15]	24	Levodopa, verschiedene DA-Agonisten, verschiedene MAO-B-Hemmer	Untersuchung hinsichtlich Nebenwirkungen, keine Wirksamkeitsuntersuchung
(Kulisevsky and Pagonabarraga 2010) [16]*	40	Dopaminagonist vs. Placebo und untereinander	Qualität der Studien nicht gut bewertet
(Stowe et al. 2008) [7]*	21	Dopaminagonist vs. Placebo oder Levodopa	sehr gute Cochrane-Meta-analyse
(Fox et al. 2011) [8]*	68, von denen nicht alle zur Frage-stellung gehören	Dopaminagonist vs. Placebo oder Levodopa	Dieser systematische Review begründet die Handlungsempfehlungen der Movement Disorder Society
(Baker et al. 2009) [17]*	25	Dopaminagonist vs. Placebo oder Levodopa	Die Metaanalyse scheint den Effekt der Dopaminagonisten zu überschätzen.

*Studien, die bereits in die vorhergehenden Leitlinien Einzug erhalten hatten

Tab. 10: Zusammenfassung von randomisierten kontrollierten Studien (RCTs) zur Effektivität von Dopaminagonisten im Frühstadium der PK

Studien	Anzahl der Pat. (n)	Substanz vs. Placebo oder Levodopa	Follow-up-Dauer	Methodische Schwächen
(Zesiewicz et al. 2017) [18]	Placebo: n=40<br>Ropinirol: 2 mg: n=13, 4 mg: n=41, 8 mg: n=40, 12 mg, n=39, 24 mg: n=13	Ropinirol vs. Placebo	13–17 Wochen Auftitrierung, gefolgt von 4–7 Wochen Dosiserhaltung, Dosisreduktion 1 Woche, Washout 1–2 Wochen	viele Subgruppen, dadurch letztlich in einigen Gruppen kleine Pat.-Zahlen.<br>kurze Studiendauer
(Hauser et al. 2016b) [19]	Placebo: n=40, Rotigotin (≤ 6 mg): n=41 Rotigotin (≥ 8 mg): n=41	Rotigotine vs. Placebo	4–7 Auftitrierung, gefolgt von 12 Wochen Dosiserhaltung, Dosisreduktion bis zu 12 Tage, Washout 28 Tage	kurze Studiendauer
(Rascol et al. 2006b) [20] *	Placebo: n=204 Piribedil: n=197	Placebo vs. Piribedil	24 Monate	überraschend starker Effekt von Piripedil, viele Zentren in Indien, Mexiko und Argentinien
(Rascol et al. 2006a) [21] *	Ropinirol: n=179 Levodopa: n=89	Ropinirol vs. Levodopa	5 Jahre	Follow-up-Studie mit geringer „Completer"-Zahl
(Watts et al. 2007) [22] *	Rotigotin: n=181 Placebo: n=96	Rotigotin vs. Placebo	27 Wochen	(Last observation carried forward) LOCF statt ITT, nicht erklärte Geschlechtsunter-schiede
(Jankovic et al. 2007) [23] *	Rotigotin: n=181 Placebo: n=96	Rotigotin vs. Placebo	27 Wochen	Es handelt sich um eine Doppelpublikation
(Poewe et al. 2011) [24] *	Placebo: n=103 Pramipexol retard: n=223 Pramipexol standard: n=213	Pramipexol retard vs. Pramipexol standard vs. Placebo	33 Wochen inkl. Titrierung	
(Mizuno et al. 2013) [25] *	Rotigotin: n=82 Placebo: n=90	Rotigotin vs. Placebo	12 Wochen inkl. Titrierung	relativ hohe Drop-out-Rate
(Schapira et al. 2013) [26] *	Placebo: n=261 Pramipexol: n=274	Pramipexol vs.<br>Placebo	15 Monate, delayed start. Verum nach 9 (6) Monaten	Nur ein Teil der Pat. erhielt FP-CIT Bildgebung.

*Studien, die bereits in die vorhergehenden Leitlinien Einzug erhalten hatten

Seit der letzten Leitlinienerstellung wurden ein Review [27] und eine Metaanalyse [28] zu Impulskontrollstörungen unter Dopaminagonisten veröffentlicht.

Ergebnis

In einer Metaanalyse aus dem Jahr 2020, in der Monotherapien mit Dopaminagonisten (Cabergoline, Pramipexol, Ropinirol und Rotigotin), Monotherapien mit den MAO-B-Hemmern Selegilin, Rasagilin und Monotherapien mit Levodopa verglichen wurden, waren alle untersuchten Dopaminagonisten, MAO-B-Hemmer und Levodopa effektiv im Vergleich zu Placebo hinsichtlich einer motorischen Verbesserung (UPDRS). Für alle Substanzen wurde eine relative Effektivität berechnet. Die effektivste Therapie stellte dabei Ropinirol (Effektivitätswert: 2,171; 95%-KI: 1,888–2,489)dar, gefolgt von Levodopa (2,017; 1,733–2,336). Etwas weniger effektiv, aber untereinander ähnlich effektiv waren Pramipexol (1,774; 1,607–1,958), Rotigotin (1,745; 1,514–2,009), Selegilin (1,697; 1,491–1,924) und Rasagilin (1,657; 1,509–1,818). Am wenigsten effektiv war Cabergolin (1,402; 1,114–1,732). Bezüglich ernsthafter unerwünschter Ereignisse traten diese bei Pramipexol häufiger auf als bei Placebo. Bei allen anderen Medikamenten war dies nicht der Fall [11]. Dermale Nebenwirkungen wurden in dieser Studie nicht beschrieben. Eine Metaanalyse von Chen et al. aus dem Jahr 2017 [12], in der Studien mit Rotigotin vs. Placebo untersucht wurden, hat gefunden, dass unter Rotigotin mehr Nebenwirkungen auftreten als unter Placebo, wobei in dieser Analyse nicht nach einzelnen Nebenwirkungen aufgeschlüsselt wurde.

Ein systematischer Review aus dem Jahr 2016 hat publizierte Daten zu placebokontrollierten Studien, die Monotherapien mit Levodopa, Dopaminagonisten und MAO-B-Hemmern untersucht haben, verglichen und statistisch analysiert. Dabei wurden Studien mit Levodopa, Pramipexol, Rasagilin und Selegilin eingeschlossen. Insgesamt hatten die Pat., die mit Levodopa behandelt wurden, die größte Verbesserung im motorischen UPDRS gegenüber Placebo, gefolgt von Pramipexol und Selegilin. Bezüglich Übelkeit und Halluzinationen gab es in dieser Metaanalyse im indirekten Gruppenvergleich zwischen Behandlung mit Levodopa, Pramipexol, Rasagilin und Selegilin keine signifikanten Unterschiede. Dyskinesien traten häufiger bei mit Levodopa behandelten Pat. auf [10].

In einem rezenten Review wurden Impulskontrollstörungen unter Dopaminagonisten analysiert. Unter der Medikation mit Dopaminagonisten treten vermehrt Impulskontrollstörungen auf. Das Auftreten von Impulskontrollstörungen ist dabei in den ersten 2,5 Jahren der Therapie seltener als in den folgenden 2,5 Jahren [27]. Eine Metaanalyse hat gezeigt, dass junges Lebensalter, männliches Geschlecht, eine längere Erkrankungsdauer, die Dauer der Therapie mit Dopaminagonisten, eine höhere Dosierung sowie Rauchen mit einem höheren Risiko für die Entwicklung von Impulskontrollstörungen assoziiert sind [28].

Frühere Rotigotin-Studien hatten gezeigt, dass unter transdermaler Rotigotin-Therapie lokale Hautreaktionen zu den häufigsten Nebenwirkungen von Rotigotin gehören [29]. Dass Hautreaktionen spezifisch für Rotigotin sind, hatte auch eine Doppel-Dummy-Studie suggeriert, in die Probanden/Probandinnen in 3 Gruppen (Pramipexol, Rotigotin, Placebo) randomisiert wurden. In dieser Studie traten Hautreaktionen häufiger bei mit Rotigotin behandelten Probanden/Probandinnen auf, obwohl auch die beiden anderen Gruppen mit einem Dummy-Pflaster

behandelt wurden. Hinsichtlich der Effektivät war Rotigotin Pramipexol nicht unterlegen [30]. Anzumerken ist, dass diese Studie vor dem Marktstart von Pramipexol-Retard-Tabletten erfolgte.

Aus den Daten der Metaanalyse aus dem Jahr 2020 lässt sich schlussfolgern, dass Ropinirol möglicherweise effektiver ist als andere Dopaminagonisten. Unter Rotigotin sind lokale Hautreaktionen im Bereich der Applikationsstelle als Nebenwirkung zu berücksichtigen. Bezüglich des Vergleichs der Effektivität zwischen Ropinirol und Levodopa ist die Datenlage uneindeutig. Andere Dopaminagonisten scheinen weniger effektiv als Levodopa zu sein. Problematisch ist, dass aus den Metaanalysen ein Vergleich von Äquivalenzdosierungen nicht ableitbar ist.

Solange keine direkten Vergleichsstudien zwischen den heute gängigen Retard-Präparaten der einzelnen Dopaminagonisten zur Verfügung stehen, kann die Frage nach dem Effektivitätsvergleich der einzelnen Dopaminagonsten nicht abschließend beantwortet werden.

Bezüglich der selbst erlebten motorischen Funktion ergab sich in der PD-MED-Studie innerhalb von 7 Behandlungsjahren kein signifikanter Unterschied zwischen einem Therapiebeginn mit Levodopa oder Dopaminagonisten. Allerdings war die Quote von Therapieabbrüchen bei Dopaminagonisten höher als unter Levodopa [9].

Im Folgenden werden die Studienergebnisse, von Metaanalysen und randomisierten kontrollierten Studien, die einzelne Dopaminagonisten analysiert haben, in Tabellenform zusammengefasst. Der Vollständigkeit halber werden auch Studien aufgeführt, die bereits in die letzte Leitlinie Einzug erhalten haben. Diese wurden mit * markiert.

Tab. 11: Endpunkte von Metaanalysen und systematischen Reviews zu Dopaminagonisten im Frühstadium

Studien	Wirksamkeit gegen Placebo	Wirksamkeit gegen Levodopa	Sicherheit: motorische Fluktuationen	Verträglichkeit: Dyskinesien, Dystonien	Verträglichkeit: nicht motorische UAWs
(Chen et al. 2017) [12]	Rotigotin besser	nicht untersucht	nicht berichtet	in einer Studie häufiger unter Rotigotin	UAW häufiger unter Rotigotin
(Giladi et al. 2016) [13]	Rotigotin besser (in allen Stadien)	nicht untersucht	nicht untersucht	nicht untersucht	nicht untersucht
(Márquez-Cruz et al. 2016) [10]	Pramipexol besser	Levodopa besser	nicht berichtet	häufiger unter Levodopa	keine signifikanten Unterschiede
(Binde et al. 2020) [11]	Ropinirol, Pramipexol, Rotigotin, Cabergolin besser	Ropinirol besser, Pramipexol, Rotigotin, Cabergolin schlechter	nicht berichtet	häufiger unter Levodopa	bei Pramipexol häufiger, bei anderen Substanzen nicht signifikant häufiger
(Zhuo et al. 2017) [15]	nicht getestet	nicht getestet		häufiger unter Levodopa, verglichen mit Placebo, häufiger unter Agonisten,	Übelkeit, Müdigkeit, Benommenheit häufiger unter Agonisten

Studien	Wirksamkeit gegen Placebo	Wirksamkeit gegen Levodopa	Sicherheit: motorische Fluktuationen	Verträglichkeit: Dyskinesien, Dystonien	Verträglichkeit: nicht motorische UAWs
				verglichen mit Placebo	gegenüber Placebo Halluzinationen häufiger unter Ropinirol gegenüber Placebo
(Kulisevsky and Pagonabarraga 2010) [16]*	nicht getestet	nicht getestet		Ropinirol besser als Levodopa	Unterschiede der Agonisten
(Stowe et al. 2008) [7]*	Agonist besser	Levodopa besser	Agonist besser	Agonist besser	Levodopa besser
(Fox et al. 2011) [8]*, §	Piribedil, Pramipexol, Ropinirol, Rotigotin, Cabergolin, Pergolid besser				
(Baker et al. 2009) [17]*	Agonist besser	Levodopa besser	Agonist besser	Agonist besser	Levodopa besser

*Studien, die bereits in die vorhergehenden Leitlinien Einzug erhalten hatten

§ Das Review beinhaltet im Wesentlichen klinische Handlungsempfehlungen

Tab. 12: Endpunkte von randomisierten, kontrollierten Studien zu Dopaminagonisten im Frühstadium.

Studien	Wirksamkeit gegen Placebo (primärer Endpunkt)	Wirksamkeit gegen Levodopa (primärer Endpunkt)	Sicherheit: motorische Komplikationen	Verträglichkeit (primärer/sekundärer EP) Auftreten von Dyskinesien	Verträglichkeit nicht motorische UAWs
(Zesiewicz et al. 2017) [18]	Ropinirol besser, allerdings nur in einer Dosis-Gruppe signifikant	nicht untersucht	nicht berichtet	1 Patient mit Ropinirol, kein Patient mit Placebo	Übelkeit, Müdigkeit, Kopfschmerzen, Benommenheit, Hypertonus, Erbrechen
(Hauser et al. 2016a) [31]	Rotigotin besser	nicht untersucht	nicht berichtet	nicht häufiger als unter Placebo	nicht häufiger als Placebo
(Rascol et al. 2006b) [20]*	Piribedil besser		nicht signifikant		Übelkeit, Hypotension, Somnolenz

Studien	Wirksamkeit gegen Placebo (primärer Endpunkt)	Wirksamkeit gegen Levodopa (primärer Endpunkt)	Sicherheit: motorische Komplikationen	Verträglichkeit (primärer/sekundärer EP) Auftreten von Dyskinesien	Verträglichkeit nicht motorische UAWs
(Rascol et al. 2006a) [21]*			nicht berichtet	Ropinirol besser	nicht berichtet
(Watts et al. 2007) [22]*	Rotigotin besser				lokale Reaktion, Benommenheit, Übelkeit, Kopfschmerz
(Poewe et al. 2011) [24]*	Pramipexol extended release (ER) und Pramipexol immediate release (IR) besser				Somnolenz, Übelkeit, Verstopfung, Benommenheit, Mundtrockenheit
(Mizuno et al. 2013) [25]*	Rotigotin besser		2 Todesfälle unter Rotigotin, nach Einschätzung nicht therapieabhängig		nicht in Originalpublikation berichtet
(Schapira et al. 2013) [26]*	Nicht signifikant				Übelkeit, Ödeme, Müdigkeit, Somnolenz, Halluzinationen

*Studien, die bereits in die vorhergehenden Leitlinien Einzug erhalten hatten

Begründung der Empfehlung

Die Studien zeigen durchweg, dass Dopaminagonisten in der Therapie des Frühstadiums der PK im Vergleich zu Placebo effektiv sind. Bezüglich des Vergleichs mit Levodopa bleibt die Datenlage dünn. Diesbezüglich gibt es weiterhin nur eine RCT aus dem Jahr 2006, mit geringer Anzahl an Pat., die die Studie beendet haben, in der Levodopa mit einem Non-Ergot-Dopaminagonisten (Ropinirol) verglichen wurde und Ropinirol bezüglich des Auftretens von Dyskinesien besser abgeschnitten hat als Levodopa.

Eine rezente Metaanalyse aus dem Jahr 2016, die einen Vergleich verschiedener Monotherapien durchgeführt hat, hat ergeben, dass Pat., die mit Levodopa behandelt wurden, verglichen mit Pat., die mit Dopaminagonist oder MAO-B-Hemmer behandelt wurden, den besten symptomatischen Effekt hatten, wobei in dieser Metaanalyse keine Studie eingeschlossen wurde, in der Ropinirol untersucht wurde [10].

Die Metaanalyse von Binde et al. aus dem Jahr 2020 hat interessanterweise die größte Effektivität für Ropinirol gefunden, noch vor Levodopa [11], wobei der Vergleich anhand von berechneten

Effektivitätswerten erfolgte, ohne dass Studien existieren würden, die alle Medikamente miteinander verglichen hätten.

Häufigste Nebenwirkungen der Dopaminagonisten in den Studien sind Übelkeit, Müdigkeit, Benommenheit und für das als Pflaster applizierte Rotigotin lokale Hautreaktionen. Zudem treten unter Dopaminagonisten häufiger Impulskontrollstörungen auf als unter Placebo. Ödeme wurden als Nebenwirkungen in Studien mit Pramipexol genannt. Kardiale Valvulopathien sind ein Problem vorwiegend der Ergot-Dopaminagonisten, weshalb diese Wirkstoffe nicht mehr eingesetzt werden dürfen.

Dyskinesien treten bei einer Dauer der Erkrankung von mehreren Jahren unter oraler Levodopa-Gabe auf, wenn eine wirksame Levodopa-Dosis für die Erzielung eines antikinetischen Effekts erforderlich ist und diese die Dyskinesie-Schwelle überschreitet.

**Empfehlung**	**Modifiziert Stand (2023)**
▪ Non-ergoline Dopaminagonisten können unter Beachtung der unten dargelegten Differenzialindikationen zur initialen Monotherapie der frühen PK eingesetzt werden.<br>▪ Ein non-ergoliner Dopaminagonist soll bis zu einer klinisch effektiven, aber noch gut verträglichen Dosis titriert werden.<br>▪ Falls bei einem non-ergolinen Dopaminagonisten auftretende Nebenwirkungen eine suffiziente Therapie verhindern, sollte eine andere Substanzklasse (Levodopa oder MAO-B-Hemmer) oder ggf. ein anderer, non-ergoliner Dopaminagonist zur initialen Monotherapie eingesetzt werden.	
Konsensstärke: 100%, starker Konsens	

Fragestellung 45: Wie effektiv und sicher sind MAO-B-Hemmer im Vergleich mit Placebo oder Levodopa oder Dopaminagonisten in der Monotherapie der PK im Frühstadium?

Hintergrund

Selektive Monoaminooxidase-B-Hemmer (MAO-B-Hemmer) blockieren den Abbau von Dopamin, sodass dessen Spiegel im Striatum erhöht wird. Die MAO-B-Hemmer Rasagilin und Selegilin sind etabliert in der Monotherapie der PK, Safinamid ist für die Monotherapie nicht zugelassen. MAO-B-Hemmer führen in der zugelassenen Dosierung nicht zu einer unerwünschten Reaktion nach Konsum von Tyramin-reicher Nahrung (sogenannter Tyramin-Effekt oder Käse-Effekt) und sind somit sicherer im Vergleich zu non-selektiven Hemmern. Initial wurden MAO-B-Hemmer für die symptomatische Behandlung der PK im Spätstadium eingeführt. Später wurden sie zusätzlich für die Behandlung der PK auch im Frühstadium eingesetzt unter der Vorstellung, dass sie neben ihrem symptomatischen Effekt auch neuroprotektive Eigenschaften entfalten. Die Frage, die hier gestellt wird, ist, ob MAO-B-Hemmer effektiv in der symptomatischen Behandlung der PK im Frühstadium sind.

Evidenzgrundlage

Seit 2005 wurde keine Studie publiziert, die die Effektivität von MAO-B-Hemmern im Vergleich mit Levodopa in der Behandlung der PK im Frühstadium untersuchte. Nach 2005 wurden keine entsprechenden Studien publiziert, die einen Vergleich zwischen MAO-B-Hemmern und Dopaminagonisten untersucht haben.

Seit der letzten Leitlinienerstellung wurden mehrere Metaanalysen und systematische Reviews veröffentlicht, in denen MAO-B-Hemmer bei der PK analysiert wurden. Zusätzlich zu den Wirksamkeitsstudien wurde in der PD-MED-Studie untersucht, welche der möglichen Ersttherapien (Levodopa, Dopaminagonisten, MAO-B-Hemmer) zu Beginn gewählt werden sollte. Es wurden 1.620 Pat. 1:1:1 open-label auf eine Therapie mit Levodopa, Dopaminagonist oder MAO-B-Hemmer randomisiert und durch Pat. ausgefüllte Fragebögen wurden die motorische Funktion und die Lebensqualität sowie Kognition und Therapieabbruch analysiert [9].

Tab.13: Zusammenfassung von aktuellen Metaanalysen und systematischen Reviews MAO-B-Hemmern im Frühstadium

Studien	Anzahl der Studien (n)	MAO-B-Hemmer vs. Placebo oder Levodopa	Methodische Schwächen und Bemerkungen
(Binde et al. 2018) [32]	27	Rasagilin vs. Placebo und als Add-on zu Levodopa<br>Selegilin vs. Placebo und als Add-on zu Levodopa<br>Safinamid vs. Placebo und als Add-on zu Levodopa	Die Studie berechnet relative Effektstärken,<br>es wird nicht eindeutig klar, welche der Studien in der Studie unten erneut analysiert wurden.
(Binde et al. 2020) [11]	79	4 DA-Agonisten (Cabergolin, Pramipexol, Ropinirol, Rotigotin)<br>vs.<br>3 MAO-B-Hemmer (Selegilin, Rasagilin, Safinamid (als Add-on))<br>vs.<br>Levodopa vs. Placebo	Die Studie berechnet relative Effektstärken, es wird nicht eindeutig klar, welche der Studien aus der Studie oben erneut analysiert wurden.
(Chang et al. 2017)[33]	10	Rasagilin alleinn oder als Add-on vs. Placebo	
(Hauser et al. 2016a) [31]	2	Rasagilin vs. Placebo	Metaanalyse der TEMPO- und ADAGIO-Studie
(Márquez-Cruz et al. 2016) [10]	5	Levodopa vs. Placebo<br>Pramipexol vs. Placebo<br>Rasagilin vs. Placebo<br>Selegilin vs. Placebo	
(Zhuo et al. 2017) [15]	110	10 verschiedene Medikamente inkl. Selegilin und Rasagilin	

Seit der letzten Leitlinienerstellung wurde eine Pharmakokinetik-Studie (gesunde Probanden/Probandinnen) und zwei Wirksamkeitsstudien (bei Parkinson-Pat.) veröffentlicht, in denen MAO-B-Hemmer untersucht wurden. Diese sind in der folgenden Tabelle aufgeführt.

In der letzten Version der Leitlinien wurden vier randomisierte, kontrollierte Studien (RCT) (Biglan 2006 [34], Hauser 2009 [35], Olanow 2009 [36], Palhagen 2006 [37]) eingeschlossen. Eine RCT (Tolosa 2012) war aus der Analyse ausgeschlossen worden, da keine Parkinson-Pat. im Frühstadium untersucht wurden. Die vier Studien, die bereits in die letzte Version der Leitlinien Einzug erhalten haben, werden ebenfalls in der Tabelle aufgeführt.

Tab. 14 Zusammenfassung von randomisierten kontrollierten Studien (RCTs) zu MAO-B-Hemmern im Frühstadium

Studien	Anzahl der Pat. (n)	Substanz vs. Placebo oder Levodopa	Follow-up-Dauer	Methodische Schwächen
(Elgart et al. 2019) [38]	64 gesunde Probanden	nicht untersucht		reine Pharmakokinetik und Safety Studie, Single-Center-Studie, gesunde Probanden/Probandinnen<br>Vergleich von 32 japanischen Probanden/Probandinnen mit 32 kaukasischen Probanden/Probandinnen
(Hattori et al. 2019) [39]	Rasagilin: n=118<br>Placebo: n=126	Rasagilin	26 Wochen	Multicenter-Studie, die in Japan durchgeführt wurde
(Mizuno et al. 2017) [40]	Selegilin: n=146<br>Placebo: n=146	Selegilin	12 Wochen	Multicenter-Studie, die in Japan durchgeführt wurde
(Biglan et al. 2006) [34] *	Placebo: n=132<br>Rasagilin: n=124 (1 mg), n=124 (2 mg)	Placebo vs. Rasagilin, Rasagilin vs. Rasagilin	6 Monate Placebo vs. Rasagilin, danach 12 Monate<br>Rasagilin vs. Rasagilin	Überschätzung, keine Informationen über Confounding und Randomisierung, Beschreibung des Verblindungsprozesses nicht ausreichend, hohe Drop-out-Rate
(Hauser et al. 2009) [35] *	Placebo: n=138<br>Rasagilin (1 mg): n=128 (early start) und n=138 (late start)	Placebo vs. Rasagilin, Rasagilin vs. Rasagilin	6 Monate Placebo vs. Rasagilin, danach<br>6 Monate Rasagilin vs. Rasagilin	Überschätzung, schwierige Interpretation bei überlappenden Effekten in der Open-Label-Erweiterung, hohe Drop-out-Rate
(Pålhagen et al. 2006) [37] *	Placebo: n=76<br>Selegilin (10 mg): n=81<br>später in Kombination mit Levodopa	Placebo vs. Selegilin	Monotherapie mit Placebo bzw. Selegilin, später Kombinations-therapie mit Levodopa, bis zu 7 Jahren	Methodik nicht adäquat für Nachweis eines Effekts auf den Krankheitsverlauf

Studien	Anzahl der Pat. (n)	Substanz vs. Placebo oder Levodopa	Follow-up-Dauer	Methodische Schwächen
	(individuelle Dosis)			
(Olanow et al. 2009) [36] *	Rasagilin 1 mg early start: n=300<br>Rasagilin 1 mg delayed start: n=288<br>Rasagilin 2 mg early start: n=295<br>Rasagilin 2 mg delayed start: n=293	Placebo vs. Rasagilin,<br>Rasagilin vs. Rasagilin	36 Wochen Rasagilin vs Placebo, danach 72 Wochen Rasagilin early start vs. delayed start	Effekt nur für Rasagilin 1 mg, nicht aber für 2 mg, Unterschied kann nicht erklärt werden. Delayed-start-Design wird ebenfalls durch symptomatischen Effekt beeinflusst.

*Studien, die bereits in die vorhergehenden Leitlinien Einzug erhalten hatten

Ergebnis

In der PD-MED-Studie hatte sich kein Unterschied hinsichtlich der selbst eingeschätzten motorischen Funktion bei Pat., die mit Levodopa, Dopaminagonisten oder MAO-B-Hemmern behandelt wurden, gezeigt. In der Gruppe der mit MAO-B-Hemmer behandelten Pat. trat allerdings die größte Quote an Therapieabbrüchen innerhalb der sieben Studienjahre auf, was darauf schließen lässt, dass der langfristige symptomatische Effekt der MAO-B-Hemmer nicht ausreichend war [9].

Die Ergebnisse von Metaanalysen und RCTs, in denen MAO-B-Hemmer untersucht wurden, sind in den folgenden Tabellen zusammengefasst.

Tab. 15: Endpunkte von Meta-Analysen und systematische Reviews zu MAO-B-Hemmern im Frühstadium.

Studien	Wirksamkeit gegen Placebo	Wirksamkeit gegen Levodopa	Sicherheit: motorische Fluktuationen	Verträglichkeit: Dyskinesien, Dystonien	Verträglichkeit: nicht motorische UAWs
(Binde et al. 2018) [32]	Rasagilin, Selegilin, Safinamid besser	nicht getestet	nicht berichtet	nicht erhöht gegenüber Placebo	nicht erhöht gegenüber Placebo
(Binde et al. 2020) [11]	Rasagilin, Selegilin, Safinamid besser	Rasagilin schlechter<br>Selegilin schlechter	nicht berichtet	nicht berichtet	nicht erhöht gegenüber Placebo
(Chang et al. 2017) [33]	Rasagilin besser	nicht getestet	nicht berichtet	nicht berichtet	nicht berichtet
(Hauser et al. 2016a) [31]	Rasagilin besser	nicht getestet	nicht untersucht	nicht untersucht	nicht untersucht
(Márquez-Cruz et al. 2016) [10]	Rasagilin besser<br>Selegilin besser	Levodopa besser	nicht berichtet	nicht berichtet	seltener unter MAO-B-Hemmern, verglichen mit DA-

Studien	Wirksamkeit gegen Placebo	Wirksamkeit gegen Levodopa	Sicherheit: motorische Fluktuationen	Verträglichkeit: Dyskinesien, Dystonien	Verträglichkeit: nicht motorische UAWs
					Agonisten oder Levodopa
(Zhuo et al. 2017) [15]	Rasagilin besser<br>Selegilin besser	Levodopa besser	nicht berichtet	nicht berichtet	nur indirekt über die Absetzrate, bei der aber auch unzureichende Wirksamkeit eine Rolle spielt

Tab. 16: Endpunkte von randomisierten, kontrollierten Studien (RCTs) zu MAO-B-Hemmern im Frühstadium

Studien	Wirksamkeit gegen Placebo (primärer Endpunkt)	Wirksamkeit gegen Levodopa (primärer Endpunkt)	Sicherheit: Motorische Komplikationen	Verträglichkeit (Primärer/Sekundärer EP) Auftreten von Dyskinesien	Verträglichkeit Nicht-motorische UAWs
(Elgart et al. 2019) [38]	nicht untersucht	nicht untersucht	nicht untersucht	nicht untersucht	kein eindeutiger Unterschied zwischen Rasagilin und Placebo
(Hattori et al. 2019) [39]	Rasagilin besser	nicht untersucht	nicht berichtet	nicht untersucht	Kopfschmerzen und Ekzeme häufiger unter Rasagilin
(Mizuno et al. 2017) [40]	Selegilin besser	nicht untersucht	nicht berichtet	nicht untersucht	
(Biglan et al. 2006) [34] *	Rasagilin in beiden Konzentrationen besser in early start				
(Hauser et al. 2009) [35] *	Rasagilin besser in early start				
(Pålhagen et al. 2006) [37] *	Selegilin besser		kein Unterschied zwischen beiden Kombinationstherapien		
(Olanow et al. 2009) [36] *	Rasagilin besser in beiden Konzentrationen in early start				etwas mehr UAWs für Rasagilin 2 mg in der aktiven Phase

*Studien, die bereits in die vorhergehenden Leitlinien Einzug erhalten hatten

Aus diesen Studien wird weiterhin deutlich, dass MAO-B-Hemmer im Frühstadium der PK hinsichtlich der motorischen Zielsymptomatik effektiv sind. MAO-B-Hemmer sind weniger effektiv als Levodopa. In einer rezenten Metaanalyse waren Selegilin und Rasagilin ähnlich effektiv wie die Dopaminagonisten Pramipexol und Rotigotin und weniger effektiv als der Dopaminagonist Ropinirol bei den eingesetzten Dosierungen [11]. Im Jahr 2017 wurde eine Sekundäranalyse der NET-PD-LS1-Studie, einer Studie, in der Creatinmonohydrat untersucht wurde, durchgeführt. Bei der Sekundäranalyse wurde untersucht, ob die Behandlungszeit mit MAO-B-Hemmern in der Studienpopulation einen Einfluss auf die Rate der klinischen Verschlechterung hatte. Diese hat ergeben, dass Pat., die länger mit MAO-B-Hemmern behandelt wurden, eine geringere klinische Verschlechterung erfahren haben [41]. Wenngleich es allgemeiner Konsens ist, dass eine Verzögerung der Krankheitsprogression durch Einsatz von MAO-B-Hemmern nicht überzeugend dargelegt ist, geben diese Daten erneut Hinweise darauf, dass MAO-B-Hemmer zu einer Verzögerung des Krankheitsverlaufs führen könnten. Anzumerken ist, dass Vergleichsdaten über ähnlich lange Behandlungszeiträume für andere medikamentöse Therapien der PK nicht vorliegen. Zudem könnte die längere Dauer der Behandlung mit MAO-B-Hemmern einen Selektionsbias zu Pat. mit milderem Verlauf beinhalten.

Bezüglich der Effektivität im Vergleich von MAO-B-Hemmern und Dopaminagonisten hatte sich in der britischen Studie [42] ein nicht signifikanter Unterschied in der Webster-Skala zwischen den Therapiearmen Bromocriptin und Levodopa plus Selegilin in einem 9-Jahres-Follow-up gezeigt.

Bezüglich motorischer Komplikationen fand die Studie von Caraceni und Musicco [43] nach einem mittleren Follow-up von 34 Monaten keinen signifikanten Unterschied in den folgenden Kategorien:

- motorische Fluktuationen
- Zeitdauer bis zum Auftreten motorischer Fluktuationen
- Häufigkeit des Auftretens von Dyskinesien
- Unterschied in der Zeitdauer bis zum Auftreten von Dyskinesien zwischen Dopaminagonisten und MAO-B-Hemmer

In der britischen Studie [42] zeigten sich nicht signifikante Unterschiede bezüglich der Mortalität zwischen den Behandlungsarmen Levodopa plus Selegilin und Bromocriptin.

Auch in der anderen Studie [43] hatte sich kein signifikanter Unterschied bezüglich der Mortalität zwischen den mit Dopaminagonisten und den mit Selegilin behandelten Pat. ergeben.

Bezüglich Studienabbruch ergaben sich in einer [43] der Studien folgende Aussagen:

- Die meisten Studienabbrüche im Dopaminagonisten-Arm erfolgten aufgrund von Übelkeit/Erbrechen oder posturaler Hypotension oder der Kombination aus beiden (43/53 Pat.).
- Die meisten Studienabbrüche in der Selegilin-Gruppe erfolgten in den ersten sechs Monaten aufgrund mangelnder Wirksamkeit.
- Eine Kombinationsbehandlung wurde bei 40,7% der Pat. unter Therapie mit Dopaminagonisten und bei 63,9% der Pat. unter Selegilin begonnen.
- Der Beginn der Behandlung mit Levodopa konnte im Mittel um 30 Monate in der Dopaminagonisten-Gruppe hinausgezögert werden und um 15 Monate in der Selegilin-Gruppe.

Begründung der Empfehlung

Auch aktuelle Metaanalysen und RCTs konnten zeigen, dass MAO-B-Hemmer in der Therapie der PK im Frühstadium effektiv sind. Die beste Datenlage liegt für Rasagilin vor. Studien, in denen ein direkter Vergleich zwischen MAO-B-Hemmern, Dopaminagonisten und Levodopa klinisch untersucht wurde, existieren nicht. Anhand von Metaanalysen lässt sich ein Direktvergleich nur unzureichend abschätzen. Safinamid ist für die Monotherapie nicht zugelassen.

Die Frage nach einem möglichen neuroprotektiven bzw. krankheitsmodifizierenden Effekt von MAO-B-Hemmern ist weiterhin nicht abschließend geklärt. Zwar ist ein neuroprotektiver bzw. krankheitsmodifizierender Effekt von MAO-B-Hemmern nie zweifelsfrei gezeigt worden, die Sekundäranalyse einer Studie, die im Jahr 2017 veröffentlicht wurde, suggeriert aber, dass eine längere Behandlungsdauer mit MAO-B-Hemmern zu einer weniger starken klinischen Verschlechterung der PK geführt haben könnte [41].

**Empfehlung**	**Modifiziert<br>Stand (2023)**
Die MAO-B-Hemmer Selegilin oder Rasagilin können unter Beachtung der unten dargelegten Differenzialindikationen zur Monotherapie der frühen PK eingesetzt werden, wenn die zu erwartende vergleichsweise milde symptomatische Wirkung ausreichend erscheint.	
Konsensstärke: 100%, starker Konsens	

Fragestellung 46: Wie effektiv und sicher ist Amantadin im Vergleich mit Placebo oder Levodopa oder Dopaminagonisten in der Monotherapie der PK im Frühstadium?

Hintergrund

Die Wirksamkeit von Amantadin auf die motorischen Symptome der PK wurde in den 1960er-Jahren zufällig bei seinem Einsatz als antivirale Medikation bei grippekranken Parkinson-Pat. beschrieben (Schwab 1969). Der genaue Wirkmechanismus ist nicht lückenlos geklärt und möglicherweise multifaktoriell. Amantadin hat eine Wirkung auf die präsynaptische Dopaminfreisetzung, wirkt als Hemmer der Wiederaufnahme von Katecholaminen in die präsynaptische Nervenendigung, post-synaptisch moduliert Amantadin die Affinität von Dopaminrezeptoren und verfügt außerdem über anticholinerge Wirkungen sowie antagonistische Wirkungen an N-Methyl-D-Aspartat (NMDA)-Glutamat-Rezeptoren. Seit der Erstbeschreibung seiner symptomatischen Anti-Parkinson-Wirkung wird Amantadin als mögliche Alternative zu dopaminergen Pharmaka in der Behandlung früher Stadien der PK klinisch eingesetzt.

Evidenzgrundlage

Es gibt keine aktuelle Studie, in der Amantadin in der Monotherapie der PK untersucht wurde.

Ergebnis

Amantadin hat einen antidyskinetischen Effekt bei Levodopa-induzierten Dyskinesien. Daten, die einen Nutzen in der Monotherapie der PK nahelegen, existieren weiterhin nicht [44].

Begründung der Empfehlung

Eine qualitativ hochwertige, systematische Analyse [45] hatte gezeigt, dass es keine ausreichende Evidenz für Wirksamkeit und Sicherheit von Amantadin in der symptomatischen Behandlung früher Stadien der PK gibt. Diese Auffassung wurde in einem rezenten Review-Artikel bestätigt [44].

Empfehlung	Modifiziert<br>Stand (2023)
Amantadin soll nicht zur Monotherapie der frühen PK eingesetzt werden.	
Konsensstärke: 96,2%, starker Konsens	

Fragestellung 47: Wie effektiv und sicher sind Anticholinergika im Vergleich mit Placebo oder Levodopa oder Dopaminagonisten in der Monotherapie einer PK im Frühstadium?

Hintergrund

Die in der Behandlung der PK gebräuchlichen Anticholinergika wirken über die selektive Blockade muscarinerger striataler Rezeptoren und primär über den hierdurch modulierten Dopamin-Release in den Basalganglien. Beginnend mit dem Einsatz von Hyoscin/Scopolamin, stellen Anticholinergika die am längsten, auch heute noch verwendete Substanzgruppe dar. Studien zu Wirksamkeit und Verträglichkeit wurden zum großen Teil vor der Einführung dopaminerger Substanzen durchgeführt und entsprechen methodisch meist nicht aktuellen Anforderungen. Hieraus ergibt sich die Frage, welche Studienevidenz im Hinblick auf Wirksamkeit und Verträglichkeit von Anticholinergika bei der PK besteht und welche Empfehlungsstärke für ihren Einsatz gegeben werden kann.

Evidenzgrundlage

Seit der letzten Leitlinienerstellung wurden keine randomisierten kontrollierten Studien veröffentlicht, die die Wirkung von Anticholinergika in der Monotherapie der PK untersucht haben.

Ergebnis

Weil seit der letzten Leitlinienerstellung keine randomisierten Studien, in denen Anticholinergika analysiert wurden, veröffentlicht wurden, hat sich die Evidenzlage seit der letzten Leitlinienerstellung nicht geändert.

Begründung der Empfehlung

Es existieren nur unzureichende Daten aus randomisierten Studien zu Wirksamkeit und Verträglichkeit von Anticholinergika bei der PK. Ihre Wirksamkeit ist aufgrund der vorliegenden Datenlage nicht hinreichend quantifizierbar, sie scheint aber insgesamt eher schwach ausgeprägt.

Empfehlung	Modifiziert<br>Stand (2023)
▪ Anticholinergika sollen nicht zur Monotherapie der frühen PK eingesetzt werden.<br>▪ In absoluten Ausnahmefällen kann ein Einsatz von Anticholinergika bei anderweitig nicht behandelbarem Tremor erwogen werden (siehe Kapitel 3.6).	
Konsensstärke: 100%, starker Konsens	

Fragestellung 48: Wie beeinflussen Alter, Lebensumstände, Komorbiditäten, Komedikation und subjektive Präferenzen die Auswahl der für die Monotherapie der PK im Frühstadium zugelassenen Arzneimittelklassen (Levodopa, Dopaminagonisten, MAO-B-Hemmer, Amantadin, Anticholinergika)?

Hintergrund

Alter, Lebensumstände und Komorbiditäten können einen großen Einfluss auf die Therapiemöglichkeiten der PK haben. Betrachtet man die Effektivität verschiedener medikamentöser Therapien in der Behandlung der frühen PK, so spielen neben der unmittelbaren klinischen Wirksamkeit insbesondere potenzielle Unterschiede bezüglich Mortalität, dem Auftreten motorischer Fluktuationen und der Notwendigkeit einer zusätzlichen Therapie mit Levodopa im weiteren Krankheitsverlauf eine wichtige Rolle.

Für die medikamentöse Monotherapie der PK stehen zahlreiche Medikamente zur Verfügung. Hierzu gehören:

- Levodopa (in Kombination mit einem Decarboxylase-Hemmer)
- Non-Ergot-Dopaminagonisten
- MAO-B-Hemmer (Selegilin, Rasagilin).

Empfehlung	Modifiziert Stand (2023)
Bei der Auswahl der verschiedenen Substanzklassen in der initialen Monotherapie sollen die unterschiedlichen Effektstärken im Hinblick auf die Wirkung, die Nebenwirkungen, das Alter der Pat., Komorbiditäten und das psychosoziale Anforderungsprofil berücksichtigt werden.	
Konsensstärke: 100%, starker Konsens	

Fragestellung 49: Wie beeinflusst das Geschlecht die Auswahl der für die Monotherapie der PK im Frühstadium zugelassenen Arzneimittelklassen (Levodopa, Dopaminagonisten, MAO-B-Hemmer, Amantadin, Anticholinergika)?

Evidenzangaben

In einem rezenten Review wurde geschlechtsspezifische Unterschiede bei Bewegungsstörungen diskutiert. Zusammenfassend benötigen Männer, möglicherweise durch das höhere Körpergewicht, eine höhere Dosierung der dopaminergen Medikation, während Frauen häufiger Fluktuationen und Dyskinesien erleben. Daten zu Levodopa-Infusionstherapien, COMT-Hemmern oder MAO-B-Hemmern liegen nicht vor. Ebenso gibt es bislang keine Empfehlungen für eine geschlechtsspezifische Behandlung der PK [46].

Ergebnis

Es existieren keine Studien bzgl. geschlechtsspezifischer Therapie der PK.

Begründung der Empfehlung

Weil es keine Evidenz für eine geschlechtsspezifische Therapie für die PK gibt, sollte das Geschlecht keinen Einfluss auf die Wahl der frühen Monotherapie der PK haben.

**Empfehlung**	**Neu**<br>**Stand (2023)**
Das Geschlecht hat gegenwärtig für die Auswahl der Therapieform der PK keine Bedeutung.	
Konsensstärke: 100%, starker Konsens	

Fragestellung 50: Hat die initiale Auswahl der für die Monotherapie des PK im Frühstadium zugelassenen Arzneimittelklassen (Levodopa, Dopaminagonisten, MAO-B-Hemmer, Amantadin, Anticholinergika) einen Einfluss auf den längerfristigen Krankheitsverlauf (z.B. hinsichtlich Entwicklung von Fluktuationen oder Dyskinesien)?

Hintergrund

Die Therapie der PK wird lebenslang durchgeführt. Wegen der Therapiedauer stellt sich die Frage, ob der längerfristige Krankheitsverlauf und insbesondere die Entwicklung von Dyskinesien durch die Therapie beeinflusst werden können.

Evidenzgrundlage

Es gibt Studien, die das Risiko zur Entwicklung von Fluktuationen und Dyskinesien unter Therapie mit Levodopa mit anderen Therapieformen der PK verglichen haben. Diese werden unter Fragestellung 43 in diesem Kapitel vorgestellt. Bezüglich der Evidenzgrundlage zum Einfluss auf den Krankheitsverlauf sei auf das Kapitel zu krankheitsmodifizierenden Therapie verwiesen.

Ergebnis

Die Ergebnisse der Vergleichsstudien zwischen der Therapie mit Levodopa und anderen Therapieformen hinsichtlich der Entwicklung von Dyskinesien werden in diesem Kapitel unter Fragestellung 43 vorgestellt. Es gibt keine Therapie, die den Krankheitsverlauf beeinflusst. Die Details werden im Kapitel zur krankheitsmodifizierenden Therapie beschrieben.

Begründung der Empfehlung

Im Verlauf der PK treten unweigerlich Wirkfluktuationen und Dyskinesien auf. Der Zusammenhang zu Levodopa-Dosis, Krankheitsdauer oder Alter der Pat. wird dabei weiterhin kontrovers gesehen.

Amantadin und Anticholinergika sollten nicht zur Parkinson-Therapie im Frühstadium eingesetzt werden. Da mit Dopaminagonisten und MAO-B-Hemmern effektive Therapien für die PK im Frühstadium existieren, sollten diese bei jüngeren Pat. zunächst bevorzugt gegeben und gegenüber einer Monotherapie mit Levodopa bevorzugt werden, wenn das individuelle Nebenwirkungsprofil dies zulässt.

Empfehlung	Neu<br>Stand (2023)
▪ Keines der zugelassenen Anti-Parkinson-Medikamente hat nachgewiesene krankheitsmodifizierende Wirksamkeit (s. Kapitel 3.24).<br>▪ Motorische Fluktuationen und Dyskinesien sind nach initialer Monotherapie mit Levodopa v.a. bei hoher Dosierung und pulsatiler Anwendung im Krankheitsverlauf früher zu beobachten als unter initialer Monotherapie mit MAO-B-Hemmern oder Dopaminagonisten.<br>▪ Bei biologisch jüngeren Pat. sollte eine Bevorzugung von Dopaminagonisten oder MAO-B-Hemmern gegenüber Levodopa erwogen werden.<br>▪ Pat., die Levodopa in der initialen Monotherapie benötigen, sollen dieses auch erhalten.<br>▪ Gründe für den initialen Einsatz von Levodopa könnten z.B. sein<br>▪ Schweregrad der Symptome<br>▪ Notwendigkeit eines rasch einsetzenden therapeutischen Effekts<br>▪ Multimorbidität<br>▪ beobachtete oder zu erwartende Nebenwirkungen anderer Medikamentengruppen (z.B. Impulskontrollstörungen bei Agonisten)<br>▪ ggf. bessere individuelle Verträglichkeit.	
Konsensstärke: 100%, starker Konsens	

Referenzen

1. Cilia R, Akpalu A, Sarfo FS, Cham M, Amboni M, Cereda E, et al. The modern pre-levodopa era of Parkinson's disease: insights into motor complications from sub-Saharan Africa. Brain. 2014;137(Pt 10):2731-42
2. Stocchi F, Rascol O, Kieburtz K, Poewe W, Jankovic J, Tolosa E, et al. Initiating levodopa/carbidopa therapy with and without entacapone in early Parkinson disease: the STRIDE-PD study. Ann Neurol. 2010;68(1):18-27
3. Fahn S, Oakes D, Shoulson I, Kieburtz K, Rudolph A, Lang A, et al. Levodopa and the progression of Parkinson's disease. N Engl J Med. 2004;351(24):2498-508
4. Verschuur CVM, Suwijn SR, Boel JA, Post B, Bloem BR, van Hilten JJ, et al. Randomized Delayed-Start Trial of Levodopa in Parkinson's Disease. N Engl J Med. 2019;380(4):315-24

5. Giannakis A, Chondrogiorgi M, Tsironis C, Tatsioni A, Konitsiotis S. Levodopa-induced dyskinesia in Parkinson's disease: still no proof? A meta-analysis. J Neural Transm (Vienna). 2018;125(9):1341-9
6. Poewe W, Seppi K, Tanner CM, Halliday GM, Brundin P, Volkmann J, et al. Parkinson disease. Nat Rev Dis Primers. 2017;3:17013
7. Stowe RL, Ives NJ, Clarke C, van Hilten J, Ferreira J, Hawker RJ, et al. Dopamine agonist therapy in early Parkinson's disease. Cochrane Database Syst Rev. 2008(2):Cd006564
8. Fox SH, Katzenschlager R, Lim SY, Ravina B, Seppi K, Coelho M, et al. The Movement Disorder Society Evidence-Based Medicine Review Update: Treatments for the motor symptoms of Parkinson's disease. Mov Disord. 2011;26 Suppl 3:S2-41
9. Gray R, Ives N, Rick C, Patel S, Gray A, Jenkinson C, et al. Long-term effectiveness of dopamine agonists and monoamine oxidase B inhibitors compared with levodopa as initial treatment for Parkinson's disease (PD MED): a large, open-label, pragmatic randomised trial. Lancet. 2014;384(9949):1196-205
10. Márquez-Cruz M, Díaz-Martínez JP, Soto-Molina H, De Saráchaga AJ, Cervantes-Arriaga A, Llorens-Arenas R, Rodríguez-Violante M. A systematic review and mixed treatment comparison of monotherapy in early Parkinson's disease: implications for Latin America. Expert Rev Pharmacoecon Outcomes Res. 2016;16(1):97-102
11. Binde CD, Tvete IF, Gåsemyr JI, Natvig B, Klemp M. Comparative effectiveness of dopamine agonists and monoamine oxidase type-B inhibitors for Parkinson's disease: a multiple treatment comparison meta-analysis. Eur J Clin Pharmacol. 2020;76(12):1731-43
12. Chen F, Jin L, Nie Z. Safety and Efficacy of Rotigotine for Treating Parkinson's Disease: A Meta-Analysis of Randomised Controlled Trials. J Pharm Pharm Sci. 2017;20(0):285-94
13. Giladi N, Nicholas AP, Asgharnejad M, Dohin E, Woltering F, Bauer L, Poewe W. Efficacy of Rotigotine at Different Stages of Parkinson's Disease Symptom Severity and Disability: A Post Hoc Analysis According to Baseline Hoehn and Yahr Stage. J Parkinsons Dis. 2016;6(4):741-9
14. Shen Z, Kong D. Meta-analysis of the adverse events associated with extended-release versus standard immediate-release pramipexole in Parkinson disease. Medicine (Baltimore). 2018;97(34):e11316
15. Zhuo C, Zhu X, Jiang R, Ji F, Su Z, Xue R, Zhou Y. Comparison for Efficacy and Tolerability among Ten Drugs for Treatment of Parkinson's Disease: A Network Meta-Analysis. Sci Rep. 2017;8:45865
16. Kulisevsky J, Pagonabarraga J. Tolerability and safety of ropinirole versus other dopamine agonists and levodopa in the treatment of Parkinson's disease: meta-analysis of randomized controlled trials. Drug Saf. 2010;33(2):147-61
17. Baker WL, Silver D, White CM, Kluger J, Aberle J, Patel AA, Coleman CI. Dopamine agonists in the treatment of early Parkinson's disease: a meta-analysis. Parkinsonism Relat Disord. 2009;15(4):287-94
18. Zesiewicz TA, Chriscoe S, Jimenez T, Upward J, VanMeter S. A fixed-dose, dose-response study of ropinirole prolonged release in early stage Parkinson's disease. Neurodegener Dis Manag. 2017;7(1):49-59
19. Hauser RA, Slawek J, Barone P, Dohin E, Surmann E, Asgharnejad M, Bauer L. Evaluation of rotigotine transdermal patch for the treatment of apathy and motor symptoms in Parkinson's disease. BMC Neurol. 2016;16:90
20. Rascol O, Dubois B, Caldas AC, Senn S, Del Signore S, Lees A. Early piribedil monotherapy of Parkinson's disease: A planned seven-month report of the REGAIN study. Mov Disord. 2006;21(12):2110-5
21. Rascol O, Brooks DJ, Korczyn AD, De Deyn PP, Clarke CE, Lang AE, Abdalla M. Development of dyskinesias in a 5-year trial of ropinirole and L-dopa. Mov Disord. 2006;21(11):1844-50
22. Watts RL, Jankovic J, Waters C, Rajput A, Boroojerdi B, Rao J. Randomized, blind, controlled trial of transdermal rotigotine in early Parkinson disease. Neurology. 2007;68(4):272-6

23. Jankovic J, Watts RL, Martin W, Boroojerdi B. Transdermal rotigotine: double-blind, placebo-controlled trial in Parkinson disease. Arch Neurol. 2007;64(5):676-82
24. Poewe W, Rascol O, Barone P, Hauser RA, Mizuno Y, Haaksma M, et al. Extended-release pramipexole in early Parkinson disease: a 33-week randomized controlled trial. Neurology. 2011;77(8):759-66
25. Mizuno Y, Nomoto M, Kondo T, Hasegawa K, Murata M, Takeuchi M, et al. Transdermal rotigotine in early stage Parkinson's disease: a randomized, double-blind, placebo-controlled trial. Mov Disord. 2013;28(10):1447-50
26. Schapira AH, McDermott MP, Barone P, Comella CL, Albrecht S, Hsu HH, et al. Pramipexole in patients with early Parkinson's disease (PROUD): a randomised delayed-start trial. Lancet Neurol. 2013;12(8):747-55
27. Antonini A, Chaudhuri KR, Boroojerdi B, Asgharnejad M, Bauer L, Grieger F, Weintraub D. Impulse control disorder related behaviours during long-term rotigotine treatment: a post hoc analysis. Eur J Neurol. 2016;23(10):1556-65
28. Liu B, Luo W, Mo Y, Wei C, Tao R, Han M. Meta-analysis of related factors of impulse control disorders in patients with Parkinson's disease. Neurosci Lett. 2019;707:134313
29. Trenkwalder C, Kies B, Rudzinska M, Fine J, Nikl J, Honczarenko K, et al. Rotigotine effects on early morning motor function and sleep in Parkinson's disease: a double-blind, randomized, placebo-controlled study (RECOVER). Mov Disord. 2011;26(1):90-9
30. Poewe WH, Rascol O, Quinn N, Tolosa E, Oertel WH, Martignoni E, et al. Efficacy of pramipexole and transdermal rotigotine in advanced Parkinson's disease: a double-blind, double-dummy, randomised controlled trial. Lancet Neurol. 2007;6(6):513-20
31. Hauser RA, Abler V, Eyal E, Eliaz RE. Efficacy of rasagiline in early Parkinson's disease: a meta-analysis of data from the TEMPO and ADAGIO studies. Int J Neurosci. 2016;126(10):942-6
32. Binde CD, Tvete IF, Gåsemyr J, Natvig B, Klemp M. A multiple treatment comparison meta-analysis of monoamine oxidase type B inhibitors for Parkinson's disease. Br J Clin Pharmacol. 2018;84(9):1917-27
33. Chang Y, Wang LB, Li D, Lei K, Liu SY. Efficacy of rasagiline for the treatment of Parkinson's disease: an updated meta-analysis. Ann Med. 2017;49(5):421-34
34. Biglan KM, Schwid S, Eberly S, Blindauer K, Fahn S, Goren T, et al. Rasagiline improves quality of life in patients with early Parkinson's disease. Mov Disord. 2006;21(5):616-23
35. Hauser RA, Lew MF, Hurtig HI, Ondo WG, Wojcieszek J, Fitzer-Attas CJ. Long-term outcome of early versus delayed rasagiline treatment in early Parkinson's disease. Mov Disord. 2009;24(4):564-73
36. Olanow CW, Rascol O, Hauser R, Feigin PD, Jankovic J, Lang A, et al. A double-blind, delayed-start trial of rasagiline in Parkinson's disease. N Engl J Med. 2009;361(13):1268-78
37. Pålhagen S, Heinonen E, Hägglund J, Kaugesaar T, Mäki-Ikola O, Palm R. Selegiline slows the progression of the symptoms of Parkinson disease. Neurology. 2006;66(8):1200-6
38. Elgart A, Rabinovich-Guilatt L, Eyal E, Gross A, Spiegelstein O. Pharmacokinetics and safety of single and multiple doses of rasagiline in healthy Japanese and caucasian subjects. Basic Clin Pharmacol Toxicol. 2019;124(3):273-84
39. Hattori N, Takeda A, Takeda S, Nishimura A, Kitagawa T, Mochizuki H, et al. Rasagiline monotherapy in early Parkinson's disease: A phase 3, randomized study in Japan. Parkinsonism Relat Disord. 2019;60:146-52
40. Mizuno Y, Hattori N, Kondo T, Nomoto M, Origasa H, Takahashi R, et al. A Randomized Double-Blind Placebo-Controlled Phase III Trial of Selegiline Monotherapy for Early Parkinson Disease. Clin Neuropharmacol. 2017;40(5):201-7
41. Hauser RA, Li R, Pérez A, Ren X, Weintraub D, Elm J, et al. Longer Duration of MAO-B Inhibitor Exposure is Associated with Less Clinical Decline in Parkinson's Disease: An Analysis of NET-PD LS1. J Parkinsons Dis. 2017;7(1):117-27

42. Lees AJ, Katzenschlager R, Head J, Ben-Shlomo Y. Ten-year follow-up of three different initial treatments in de-novo PD: a randomized trial. Neurology. 2001;57(9):1687-94
43. Caraceni T, Musicco M. Levodopa or dopamine agonists, or deprenyl as initial treatment for Parkinson's disease. A randomized multicenter study. Parkinsonism Relat Disord. 2001;7(2):107-14
44. Rascol O, Fabbri M, Poewe W. Amantadine in the treatment of Parkinson's disease and other movement disorders. Lancet Neurol. 2021;20(12):1048-56
45. Crosby N, Deane KH, Clarke CE. Amantadine in Parkinson's disease. Cochrane Database Syst Rev. 2003;2003(1):Cd003468
46. Meoni S, Macerollo A, Moro E. Sex differences in movement disorders. Nat Rev Neurol. 2020;16(2):84-96

3.3 Frühe Kombinationstherapie

Autoren/Autorinnen: Matthias Höllerhage, Claudia Trenkwalder, Günter Höglinger

Fragestellung 51: Sollte bei unzureichender Wirksamkeit einer Monotherapie bei Parkinson-Pat., bei denen noch keine motorischen Komplikationen (Fluktuationen/Dyskinesien) aufgetreten sind, ein Wechsel der Monotherapie oder eine Kombinationstherapie erfolgen?

Hintergrund

In einigen Fällen kann durch die Initiierung einer Monotherapie keine befriedigende Symptomkontrolle erreicht werden, sodass sich in solchen Fällen die Frage stellt, ob zunächst eine alternative Monotherapie gewählt oder eine Kombinationstherapie begonnen werden sollte.

Evidenzgrundlage

Hinsichtlich der Frage nach einem Therapiewechsel von einer Monotherapie zu einer anderen Monotherapie liegen keine RCTs vor. Die besten Daten lassen sich aus den RCTs gewinnen, bei denen Levodopa als Rescue-Therapie erlaubt war. Der Zeitpunkt, wann die Indikation für eine Kombinationstherapie der PK zu stellen ist, ist nicht durch randomisierte kontrollierte Studien untersucht worden.

Ergebnis

Es gibt keine Studie, die systematisch den Wechsel von einer Monotherapie zu einer anderen analysiert hat. Deswegen existieren keine Daten, die belegen, dass es sinnvoll ist, bei unzureichender Wirksamkeit von einer Monotherapie zu einer anderen zu wechseln.

Begründung der Empfehlung

Weil keine ausreichenden Daten zum Wechsel von einer Monotherapie zur anderen vorliegen, empfehlen wir ein pragmatisches Vorgehen auf Basis von Expertenmeinungen.

Empfehlung	Neu<br>Stand (2023)

- Ausgehend von einer initialen Monotherapie, sollte eine pharmakologische Kombinationstherapie dann angeboten werden, wenn
  - die Wirksamkeit der initialen Monotherapie bei mittlerer Erhaltungsdosis (s. Kapitel 3.1, Tabellen 6 und 7) auf die Dopa-sensitiven Zielsymptome unzureichend ist
  - die zur Symptomkontrolle notwendige Dosierung der Monotherapie aufgrund von therapielimitierenden Nebenwirkungen nicht erreicht werden kann.
- Ausgehend von einer initialen Monotherapie mit einem MAO-B-Hemmer, sollte bei unzureichender Wirksamkeit eine Monotherapie oder Kombinationstherapie mit einem Dopaminagonisten oder Levodopa angeboten werden.
- Ausgehend von einer initialen Monotherapie mit Levodopa, sollte bei unzureichender Wirksamkeit eine Kombinationstherapie mit einem Dopaminagonisten angeboten werden.

▪ Ausgehend von einer initialen Monotherapie mit einem Dopaminagonisten, sollte bei unzureichender Wirksamkeit eine Kombinationstherapie mit Levodopa angeboten werden.
Konsensstärke: 100%, starker Konsens

Autoren/Autorinnen: Sylvia Maaß, Dr. Günter Höglinger

Fragestellung 52: Wie effektiv und sicher ist eine frühe Kombinationstherapie aus Dopaminagonisten und Levodopa bei Parkinson-Pat., bei denen noch keine motorischen Komplikationen (Fluktuationen/Dyskinesien) aufgetreten sind?

Hintergrund

In der DGN-Leitlinie von 2016 wurde die o.g. Fragestellung nicht explizit behandelt; hier galt als Empfehlung im Frühstadium der Erkrankung, dass MAO-B-Hemmer, Dopaminagonisten oder Levodopa in der symptomatischen Therapie der frühen PK verwendet werden sollten. Bei der Auswahl der verschiedenen Substanzklassen sollten die unterschiedlichen Effektstärken im Hinblick auf die Wirkung, die Nebenwirkungen, das Alter des Pat., Komorbiditäten und das psychosoziale Anforderungsprofil berücksichtigt werden. Auf eine Kombination der Behandlung mit Levodopa und einem Dopaminagonisten wurde unter den Gesichtspunkten der Effizienz und Sicherheit in der Leitlinie von 2016 nicht eingegangen.

Prinzipiell kann bei Pat. im frühen Stadium der Erkrankung eine zusätzliche Therapie mit einem Dopaminagonisten angezeigt sein, insbesondere, wenn nach einer initialen Aufdosierung von Levodopa (in Kombination mit einem Dopadecarboxylase-Hemmer) auf die üblicherweise angestrebten 300 mg Levodopa pro Tag bestimmte dopasensitive Symptome keine gute Responsivität auf Levodopa zeigen. Eine weitere Aufdosierung von Levodopa kann dann zwar erwogen und (ggfs. auch vorübergehend) durchgeführt werden, wobei in Anlehnung an die STRIDE-PD-Studie [1] zur Vermeidung von Levodopa-induzierten Komplikationen in der Frühphase der Erkrankung in diesem Fall einer Kombinationstherapie der Vorrang gegeben werden sollte, siehe hierzu auch Kapitel 3.2., Monotherapie, sowie die vorangehende Fragestellung in „Frühe Kombinationstherapie“.

Eine weitere Indikation zur frühen Kombinationstherapie sind fehlende Möglichkeiten der Ausdosierung einer Monotherapie bzw. die Notwendigkeit zur Dosisreduktion aufgrund von limitierenden Nebenwirkungen.

Evidenzgrundlage

Die Literatursuche in Pubmed erbrachte 23 Studien zu den Begriffen „Dopamine agonists AND levodopa AND early Parkinson’s disease“. Nach Screening der Abstracts und Inhalte wurden weitere Studien aus den Literaturreferenzen extrahiert und letztendlich 11 Studien selektiert, die in diese Leitlinie einbezogen wurden.

Insgesamt fanden sich dabei jedoch keine rezenten randomisierten kontrollierten Studien zum Thema in dieser Literaturrecherche. Die existierenden Veröffentlichungen bezogen sich dabei hauptsächlich auf Studien, die großteils bereits in den 90er-Jahren des vergangenen Jahrhunderts

durchgeführt worden waren, vorwiegend mit dem Ergot-Dopaminagonisten Bromocriptin, darunter eine prospektive randomisierte Studie [2] sowie eine multizentrische randomisierte prospektive Studie [3] (Evidenzgrad I).

Ebenfalls aus den 90er-Jahren datieren eine prospektive randomisierte Beobachtungsstudie, welche die Kombination von Levodopa mit dem Ergot-Dopaminagonist Lisurid vs. Levodopa mit Placebo untersuchte [4], sowie eine offene Beobachtungsstudie zu dem Non-Ergot-Dopaminagonisten Pirebidil aus dem Jahr 2004 [5]. Unter den zuletzt durchgeführten Studien fand sich eine Beobachtungsstudie zu Ropirinol transdermal in der Kombination mit Levodopa aus dem Jahr 2021 [6] (Evidenzgrad II).

Ferner fanden sich eine randomisierte doppelblinde Studie, die Bromocriptin, Levodopa und die Kombination beider untersuchte [7], sowie eine RCT, die Lisurid vs. Lisurid in Kombination mit Levodopa bei De-novo-Pat. untersuchte [8]. Der Non-Ergot-Dopaminagonisten Pramipexol in der Kombination mit Levodopa wurde in zwei RCTs [9] [10], zum einen bei Pat. im frühen Stadium, zum anderen bei Pat. mit therapierefraktärem Tremor im früheN bzw. mittlereN Stadium der Erkrankung untersucht. Zudem fandEN sich eine RCT zu Pirebidil in der Kombination mit Levodopa [11] sowie eine Studie, die Pirebidil in der Kombination mit Levodopa im Vergleich zu Bromocriptin in Kombination mit Levodopa untersuchte [12] (Evidenzgrad I).

Darüber hinaus fanden sich einige Übersichtsartikel [13], [14] (Evidenzgrad III).

Fazit

Es fanden sich kaum systematische, randomisierte, kontrollierte Studien zu den derzeit empfohlenen non-ergolinen Dopaminagonisten in der Kombination mit Levodopa und somit keine ausreichend validierte Evidenz zur Effizienz und Sicherheit der Kombinationstherapie. Derzeit beruhen die Empfehlungen zur Therapie überwiegend auf Kohorten- und Fallbeobachtungen und pathophysiologischen Überlegungen im Sinne einer Expertenmeinung.

Ergebnis

Die Wirksamkeit von Dopaminagonisten in der Monotherapie gegenüber Placebo ist in zahlreichen zentralen Zulassungsstudien (zuletzt vorwiegend nicht ergoline Dopaminagonisten) für die einzelnen Substanzen dargelegt [15], [16], [17], [18], [19]). Die Wirksamkeit wird ferner unterstützt durch drei Metaanalysen [20], [21], [22] sowie durch die Handlungsempfehlung der Movement Disorder Society [23]. Die Wirksamkeit einer frühen Kombination von Dopaminagonisten mit Levodopa und einem L-Dopa-sparenden Effekt wurde ebenfalls in Studien gezeigt [18], [24], [25].

Zur frühen Kombinationstherapie legen sämtliche in der o.g. Literaturrecherche aufgeführten Studien eine gute Wirksamkeit der Kombinationstherapie mit einem Dopaminagonisten und Levodopa nahe bzw. konnten diese nachweisen, wenngleich einschränkend erwähnt werden muss, dass die meisten Studien mit nicht mehr gebräuchlichen ergolinen Dopaminagonisten durchgeführt wurden (u. a. Bromocriptin, Lisurid).

Sicherheit und Verträglichkeit

Bereits in den vorangegangenen DGN-Leitlinien wurde eine geringere Verträglichkeit der Therapie mit einem Dopaminagonisten im Allgemeinen im Vergleich zu einer Therapie mit Levodopa beschrieben, wobei insbesondere Übelkeit, Müdigkeit, Blutdruckabfall, Impulskontrollstörungen, Mundtrockenheit und Benommenheit genannt werden. Nicht orale (transdermale) Applikationen zeigen außerdem eine erhöhte Rate von Hautreizungen. Zudem wurden bereits in der vorangegangenen DGN-Leitlinie aufgrund des Nebenwirkungsspektrums ergoliner Dopaminagonisten die Empfehlungen primär für non-ergoline Dopaminagonisten gegeben.

Die wenigen Studien, die die Kombination von derzeit in der Anwendung gebräuchlichen nicht ergolinen Dopaminagonisten mit Levodopa untersuchten (Pirebidil, Pramipexol, Ropirinol, Rotigotin), konnten ebenfalls eine gute Verträglichkeit, abgesehen von den typischen Nebenwirkungen der Dopaminagonisten, belegen [26], [11], [27], [6], [28].

Es lässt sich aus diesen Studien jedoch nicht ableiten, ob bezüglich des Nebenwirkungsspektrums vulnerable Pat.-Gruppen existieren, für die speziell bei einer frühen Kombinationstherapie von Dopaminagonisten mit Levodopa besondere Nebenwirkungsrisiken bestehen würden. Ob die Kombinationsbehandlung bei bestimmten Pat.-Gruppen mehr oder weniger unerwünschte Nebenwirkungen verursacht als eine Monotherapie, ist aus den vorliegenden Studien ebenfalls nicht ableitbar.

Da eine Durchführung breit angelegter Studien zur Beantwortung dieser Fragestellungen in absehbarer Zeit nicht realistisch ist, muss derzeit auf einen Expertenkonsens zurückgegriffen werden.

Insgesamt kann empfohlen werden, bei einer frühen Kombinationstherapie ebenso wie bei der Monotherapie mit einem nicht ergolinen Dopaminagonisten vorzugehen. Im Einzelnen heißt dies, vor der Kombinationsbehandlung eine Aufklärung der Pat. und der Angehörigen über mögliche Risiken vorzunehmen (insbesondere über Impulskontrollstörungen, psychotische Phänomene, erhöhte Tagesmüdigkeit, Übelkeit und arterielle Hypotonie) und die Aufklärung schriftlich zu dokumentieren. Zum praktischen Vorgehen siehe auch die entsprechenden Fragestellungen zur Therapie mit einem Dopaminagonisten in Kapitel 3, Pharmakotherapie motorischer Symptome, Monotherapie.

Begründung der Empfehlung

Auf der Basis der oben dargestellten Evidenzgrundlage wird zur Frage nach der Wirksamkeit und Sicherheit der Behandlung von Parkinson-Pat. mit einem Dopaminagonisten in Kombination mit Levodopa die u.g. Empfehlung gegeben. Die Evidenz beruht auf den o.g. Beobachtungsstudien, Fallserien sowie wenigen, zumeist älteren randomisierten kontrollierten Studien, die sich allerdings zumeist auf heute nicht mehr gebräuchliche ergoline Dopaminagonisten beziehen.

Empfehlung	Neu<br>Stand (2023)
▪ Parkinson-Pat. ohne motorische Komplikationen (Fluktuationen/Dyskinesien), die unter einer Monotherapie mit einem nicht ergolinen Dopaminagonisten in mittlerer Erhaltungsdosis eine unzureichende Wirkung auf die motorischen Zielsymptome oder limitierende Nebenwirkungen aufweisen, soll eine frühzeitige Kombinationstherapie mit Levodopa angeboten werden.<br>▪ Parkinson-Pat. ohne motorische Komplikationen (Fluktuationen/Dyskinesien), die unter einer Monotherapie mit Levodopa in mittlerer Erhaltungsdosis (ca. 300 mg, angepasst an Körpergewicht) eine unzureichende Wirkung auf die motorischen Zielsymptome oder limitierende Nebenwirkungen aufweisen, soll eine frühzeitige Kombinationstherapie mit einem nicht ergolinen Dopaminagonisten angeboten werden.<br>▪ Die Präparate sollen dabei im Hinblick auf die unterschiedlichen Effektstärken, die Nebenwirkungen, das Alter der Pat., deren Komorbiditäten und das psychosoziale Anforderungsprofil ausgewählt werden.<br>▪ Eine vorsichtige Eindosierung eines Dopaminagonisten zusätzlich zur Therapie mit Levodopa ist insbesondere bei Pat.-Gruppen mit erhöhtem Risikoprofil für potenzielle Nebenwirkungen zu empfehlen.	
Konsensstärke: 100%, starker Konsens	

Wichtige Forschungsfragen:

Randomisierte kontrollierte Studien zur Effizienz und Sicherheit einer frühen Kombinationstherapie mit Levodopa und einem (nicht ergolinen) Dopaminagonisten bei der PK fehlen derzeit weitestgehend.

Daher wären breit angelegte prospektive Studien wünschenswert, um unerwünschte Nebenwirkungen unter einer Kombinationstherapie auch im Langzeitverlauf zu dokumentieren, beispielsweise das Auftreten psychotischer Symptome bei geriatrischen Pat. oder Impulskontrollstörungen bei jüngeren Pat.

Kapitelautoren: Dr. Matthias Löhle, Dr. Sylvia Maaß, Prof. Dr. Günter Höglinger

Fragestellung 53: Wie effektiv und sicher ist Levodopa als Zusatztherapie zu einem MAO-B-Hemmer bei Parkinson-Pat., bei denen noch keine motorischen Komplikationen (Fluktuationen/Dyskinesien) aufgetreten sind?

Hintergrund

In Deutschland stehen gegenwärtig mit Selegilin, Rasagilin und Safinamid drei Substanzen mit MAO-B-inhibierender Wirkung zur Behandlung der PK zur Verfügung [29]. Während Selegilin und Rasagilin sowohl zur Monotherapie also auch zur Kombinationstherapie mit Levodopa zugelassen sind, kann das seit 2015 in Deutschland vermarktete Safinamid nur bei Pat. im mittleren bis Spätstadium mit motorischen Wirkfluktuationen in der Kombinationstherapie mit L-Dopa eingesetzt werden.

Prinzipiell kann bei Pat. im frühen Stadium der Erkrankung auch ohne motorische Wirkfluktuationen eine Kombinationstherapie mit MAO-B-Hemmern und Levodopa (in Kombination mit einem Dopa-

decarboxylase-Hemmer) angezeigt sein, z.B. wenn die initiale Monotherapie mit einem MAO-B-Hemmer keine ausreichende Kontrolle der Parkinson-Symptome leistet. Die vorliegende Frage beschäftigt sich mit der Bewertung der Effektivität und Sicherheit von MAO-B-Hemmern als Zusatztherapie zu L-Dopa bei Pat. ohne motorische Wirkfluktuationen, was in der DGN-Leitlinie von 2016 nicht explizit behandelt wurde. Da Safinamid in dieser Indikation aufgrund der oben genannten Zulassungseinschränkung bislang ausscheidet und diese Substanz zudem nicht nur MAO-B-inhibierende Wirkung hat, sondern auch spannungsabhängige Natrium- und Kalziumkanäle blockiert und zusätzlich über antiglutamaterge Effekte verfügt, fokussiert die Leitlinie auf Studiendaten zu den klassischen MAO-B-Hemmern Selegilin und Rasagilin.

Evidenzgrundlage

Die Literatursuche in Pubmed erbrachte 178 Einträge zu den Begriffen „MAO-B inhibitor(s) AND levodopa AND early Parkinson’s disease“. Nach Screening der Abstracts und Inhalte wurden weitere Studien aus den Literaturreferenzen extrahiert und letztendlich 20 Studien, 3 Übersichtsartikel und ein Artikel mit einer gepoolten Analyse selektiert, die in diese Leitlinie einbezogen wurden.

Insgesamt fanden sich dabei 8 doppelblinde, randomisierte ältere klinische Studien zur Kombinationstherapie zwischen MAO-B-Hemmern und Levodopa (Evidenzgrad I) sowie eine offene, prospektive Studie zur Kombinationstherapie zwischen Selegilin und Levodopa (Evidenzgrad II), wobei sich 7 dieser Studien auf Selegilin bezogen und zumeist aus den späten 80er- bzw. 90er-Jahren stammen. Zur besseren Einschätzung der Wirksamkeit und des Sicherheitsprofils der Substanzen bezogen wir daher auch 7 Studien zur Monotherapie mit MAO-B-Hemmern in der Frühphase der PK in diese Leitlinie ein. Zur Bewertung der Sicherheit in der Kombinationstherapie fanden zudem 5 doppelblinde, randomisierte Studien mit MAO-B-Hemmern bei Pat. mit motorischen Fluktuationen Berücksichtigung. Darüber hinaus wurden drei Übersichtsartikel und ein Artikel mit einer Analyse gepoolter Studiendaten verwendet.

Fazit

In den meist älteren Studien zu Selegilin wurden zumeist primär krankheitsmodifizierende Eigenschaften untersucht und/oder Einspareffekte auf die Levodopa-Dosis bewertet. Insgesamt sind die Effekte von Selegilin in der Kombinationstherapie mit Levodopa in mehreren RCTs aber belegt worden. Es fanden sich hingegen kaum systematische, randomisierte, kontrollierte Studien zu dem derzeit sicherlich am häufigsten verwendeten MAO-B-Hemmer Rasagilin in der Kombination mit Levodopa bei Pat. ohne motorische Wirkfluktuationen, während dessen Wirksamkeit und Sicherheit als Monotherapie in der Frühphase der PK gut belegt ist. Zu Safinamid fanden sich keine hinreichenden Daten, um dessen Einsatz in der Kombinationstherapie mit L-Dopa bei Pat. ohne motorische Wirkfluktuationen zu rechtfertigen. Insgesamt beruhen die u.g. Empfehlungen somit überwiegend auf Studiendaten aus älteren und qualitativ uneinheitlichen RCTs mit Selegilin, Schlussfolgerungen aus Studiendaten zu Rasagilin in der Anwendung als Monotherapie bzw. bei Pat. mit bereits bestehenden motorischen Fluktuationen und eigenen klinischen Erfahrungen im Sinne einer Expertenmeinung.

Ergebnis

Wirksamkeit

Die symptomatische Wirkung von MAO-B-Hemmern im frühen Stadium der PK ist in mehreren Studien gut belegt worden. Bereits in den späten 80er-Jahren konnte z.B. in der DATATOP-Studie gezeigt werden, dass es unter einer Behandlung mit Selegilin im Frühstadium der PK zur einer signifikanten Besserung der motorischen Symptome kommt [30]. In einer aktuelleren Studie bei Pat. mit früher, unbehandelter PK konnte unter Monotherapie mit Selegilin nach 12 Wochen eine Besserung im kombinierten UPDRS I+II+III um 6,26 Punkte (im Vergleich zu einer Besserung um 3,14 Punkte unter Placebo) nachgewiesen werden [31]. Die genaue Effektstärke einer frühen Kombinationstherapie von Selegilin mit Levodopa bei Pat. ohne motorische Wirkfluktuationen ist etwas schwieriger zu quantifizieren, da ältere Studien vorwiegend auf krankheitsmodifizierende Effekte von Selegilin fokussierten, hierzu Auswaschphasen am Ende der Selegilin-Behandlung nutzten und/oder den Zeitpunkt der Notwendigkeit einer Erhöhung der Levodopa-Dosis als klinischen Endpunkt verwendeten. So wurde z.B. In der SINDEPAR (Sinemet-Deprenyl-Parlodel)-Studie die Veränderung im UPDRS zwischen der initialen und der finalen Visite nach Washout aller Studiensubstanzen als primäres Outcome definiert [32]. In der Gruppe, die eine kombinierte Behandlung mit Selegilin und Levodopa erhielt, zeigte sich nach zwölfmonatiger Behandlung und zweimonatiger Auswaschphase noch eine signifikant geringere Symptomlast im UPDRS als unter Placebo und Levodopa, was von den Autorinnen/Autoren damals mit neuroprotektiven Effekten des Selegilins erklärt wurde, andererseits aber auch durch prolongierte symptomatische Effekte bedingt gewesen sein kann. In der SELEDO-Studie, einer randomisierten, placebokontrollierten, doppelblinden, multizentrischen Langzeitstudie, wurden mögliche Vorteile einer frühen Kombinationstherapie mit Selegilin und Levodopa über 5 Jahre hinweg untersucht und die Notwendigkeit der Erhöhung der Levodopa-Dosis als Endpunkt definiert [33]. In dieser Studie musste die Levodopa-Dosis bei Pat., die zeitgleich mit Selegilin behandelt wurden, signifikant später um mehr als 50% der Ausgangsdosis erhöht werden als bei Placebo-behandelten Pat. [33]. Während unter Selegilin-Behandlung die tägliche Levodopa-Dosis nach 5 Jahren bei den noch in der Studie verbliebenen Teilnehmenden im Mittel nur um 50 mg erhöht worden war, musste bei Studienteilnehmern unter Placebo im gleichen Zeitraum eine Erhöhung um 217 mg vorgenommen werden [33]. Eine ähnliche Studie der Norwegian-Danish Study Group kam zu vergleichbaren Ergebnissen [34]. Auch hier gab es einen signifikanten Unterschied in den zur Symptomkontrolle notwendigen täglichen Levodopa-Dosen zwischen mit Selegilin und mit Placebo behandelten Studienteilnehmenden nach 3 bzw. 4,5 Jahren Beobachtungsdauer, der Symptomprogress im UPDRS war zwischen den Gruppen hingegen statistisch nicht signifikant unterschiedlich. Zusammengenommen mit weiteren, älteren Studien mit ähnlichen Ergebnissen [35], [36], [37], kann insgesamt davon ausgegangen werden, dass der frühe Einsatz von Selegilin bei Levodopa-behandelten Pat. im zeitlichen Verlauf zu einem signifikanten Einspareffekt bei der Levodopa-Dosis führt, der mutmaßlich vordergründig über dessen symptomatische Wirkung zustande kommt, wobei zusätzliche neuroprotektive bzw. krankheitsmodifizierende Effekte nicht ausgeschlossen werden können.

Ähnliche Therapieeffekte lassen sich auch aus Studien mit dem MAO-B-Hemmer Rasagilin ableiten, der in der TEMPO-Studie bei Pat. mit früher PK als Monotherapie mit einer Tagesdosis von 1 mg/d nach 26 Behandlungswochen zu einer Besserung von 4,2 Punkten im Gesamt-UPDRS im Vergleich zu Placebo führte [38]. In der nachfolgenden ADAGIO-Studie wurde die symptomatische Wirksamkeit einer Monotherapie mit Rasagilin 1 mg/d im Frühstadium der PK ebenfalls gezeigt, hier führte die Substanz im Vergleich zu Placebo zu einer Besserung im Gesamt-UPDRS von 3,01 Punkten nach 36 Behandlungswochen [39]. Während mehrere Studien die Wirksamkeit einer Kombinationstherapie von Rasagilin und Levodopa bei Pat. mit motorischen Fluktuationen gut belegen [40], [41], [42], [43], [44], gibt es bislang keine genauen Daten aus doppelblinden, randomisierten klinischen Studien über die genaue Effektstärke einer Kombinationstherapie mit Rasagilin und Levodopa bei der frühen PK ohne Vorhandensein von motorischen Wirkfluktuationen. In der Literaturrecherche konnten wir lediglich eine doppelblinde, placebokontrollierte Studie finden, in der Rasagilin in Kombination mit Levodopa auch bei Pat. ohne motorische Wirkfluktuationen untersucht wurde, wobei auch in dieser Studie immerhin 32 (46%) der 70 Studienteilnehmenden bereits unter motorischen Wirkfluktuationen litten [45]. In dieser Studie zeigte sich bei Kombination von Levodopa mit Rasagilin 0,5 mg/d, 1 mg/d oder 2 mg/d zwar ein Trend zu einer Verbesserung im Gesamt-UPDRS, jedoch war dieser im Vergleich zu Placebo nicht statistisch signifikant, was vordergründig mit der geringen Stichprobengröße in dieser Studie erklärt wurde [45]. Auch wenn die Daten zur Kombinationstherapie von Levodopa mit Rasagilin bei Pat. ohne motorische Wirkfluktuationen für eine abschließende Bewertung der Effektstärke somit nicht ausreichend sind, gibt es nach Ansicht der Leitlinienautoren und -autorinnen angesichts der robusten symptomatischen Effekte einer Monotherapie mit Rasagilin in der Frühphase der PK und des im Vergleich zu Levodopa unterschiedlichen Wirkmechanismus wenig Zweifel, dass Rasagilin auch in der Kombination mit Levodopa effektiv motorische Symptome in der Frühphase der PK lindern kann.

Zum Einsatz von Safinamid bei Parkinson-Pat. ohne motorische Fluktuationen liegen gegenwärtig keine hinreichenden Studiendaten zur Effektstärke in der Monotherapie oder in der Kombinationstherapie mit L-Dopa vor.

Sicherheit und Verträglichkeit

Zahlreiche randomisierte, doppelblinde klinische Studien mit Selegilin und Rasagilin in der Frühphase der PK belegen eine gute Sicherheit und Verträglichkeit dieser beiden MAO-B-Hemmer. Aus gepoolten Daten mehrerer Langzeitstudien [46], [47], [48], [49] zur Monotherapie mit Selegilin bei Pat. mit früher PK lassen sich Insomnie, Schwindel, Übelkeit, Kopfschmerzen, Fatigue, trockener Mund und Angst als häufigste Nebenwirkungen von Selegilin identifizieren [50]. Nebenwirkungen scheinen unter einer Selegilin-Monotherapie jedoch insgesamt nicht signifikant häufiger als unter Placebobehandlung aufzutreten [50], was auch in einer neueren Studie reproduziert werden konnte [31]. Auch die frühe Kombinationstherapie von Selegilin und Levodopa zeichnet sich durch eine gute Verträglichkeit aus. So zeigen Studiendaten zur Kombinationstherapie beider Substanzen [51], [34], dass unter der Kombinationstherapie mit Selegilin und Levodopa nicht mehr Nebenwirkungen auftreten als unter einer Monotherapie mit Levodopa [50]. Als Nebenwirkungen wurden unter dieser Kombination am häufigsten Übelkeit, Schwindel, Fatigue, Obstipation und Insomnie beobachtet [50].

Im Vergleich zu Selegilin bietet Rasagilin aus pharmakologischer Sicht den Vorteil, dass bei dessen Metabolisierung keine Amphetamin-Derivate anfallen, was theoretisch dessen Verträglichkeit verbessern könnte. In der bereits zitierten TEMPO-Studie traten unter Monotherapie mit Rasagilin in der Tat nicht mehr Nebenwirkungen auf als unter Placebobehandlung [38]. Als häufigste Nebenwirkungen wurden in dieser Studie unter Monotherapie mit Rasagilin in der heute gebräuchlichen Tagesdosis von 1 mg/d Infektionen, Kopfschmerzen, unbeabsichtigte Verletzungen, Schwindel, Schmerzen, Übelkeit und Rückenschmerzen registriert. Auch in der nachfolgenden ADAGIO-Studie wurde die gute Verträglichkeit einer Monotherapie mit Rasagilin bestätigt, hier wurden als häufigste Nebenwirkungen Fatigue, Kopfschmerzen, Rückenschmerzen, Nasopharyngitis und Schwindel/Übelkeit berichtet, die aber unter Placebobehandlung in gleicher Häufigkeit dokumentiert wurden [39]. Leider liegen keine dezidierten Studien zur Verträglichkeit der Kombination von Rasagilin und Levodopa bei Parkinson-Pat. ohne motorische Wirkfluktuationen vor, sodass lediglich aus Erfahrungen mit dieser Kombination bei Pat. mit motorischen Fluktuationen berichtet werden können. In der PRESTO-Studie, in der Rasagilin 0,5 mg/d und 1 mg/d im Vergleich zu Placebo bei L-Dopa-behandelten Pat. mit mindestens 2,5 Stunden Off-Zeit pro Tag untersucht wurde, führte die Behandlung mit Rasagilin in der Dosis von 1 mg/d in Kombination mit L-Dopa signifikant häufiger zu Nebenwirkungen als die Behandlung mit Placebo und L-Dopa, was aber nicht häufiger zu einem Studienabbruch als unter Placebo führte [40]. Dagegen wurden in der LARGO-Studie, in der Rasagilin, Entacapon oder Placebo in Kombination mit Levodopa zur Reduktion der Off-Zeit eingesetzt wurden, unter der Kombinationstherapie mit Rasagilin und Levodopa keine häufigeren Nebenwirkungen im Vergleich zur Placebogruppe beobachtet [41]. Basierend auf allen verfügbaren Studien, kann insgesamt die Schlussfolgerung gezogen werden, dass Rasagilin sicher und gut verträglich ist [52]. Post-hoc-Analysen legen zudem nahe, dass Rasagilin von älteren (≥ 70 Jahre), nicht dementen Pat. in der Kombinationstherapie ähnlich gut vertragen wird wie im jüngeren Pat.-Alter, dass es im höheren Alter aber etwas häufiger zu Halluzinationen, Depression, Obstipation, Schmerzen, Schläfrigkeit und Gewichtsverlust kommt [53]. Zudem ist zu berücksichtigen, dass Pat. mit hohem Lebensalter, relevanten Nebenerkrankungen oder schwerer Behinderung zumeist von randomisierten, klinischen Studien ausgeschlossen werden und somit für diese Pat.-Gruppen keine verlässlichen Sicherheitsdaten zur Verfügung stehen. Aus Sicht der Leitlinienautoren und -autorinnen ist daher insbesondere bei älteren und komorbiden Pat. das Nebenwirkungspotenzial der MAO-B-Hemmer zu berücksichtigen und die Indikation für deren Einsatz individuell zu prüfen.

Zur Sicherheit und Verträglichkeit von Safinamid bei Parkinson-Pat. ohne motorische Wirkfluktuationen liegen weder für die Monotherapie noch für die Kombinationstherapie mit Levodopa belastbare Studiendaten vor.

Begründung der Empfehlung

Auf der Basis der oben dargestellten Evidenzgrundlage wird zur Frage nach der Wirksamkeit und Sicherheit der Behandlung von Parkinson-Pat. mit MAO-B-Hemmern in Kombination mit Levodopa die u.g. Empfehlung gegeben.

Empfehlung	Neu<br>Stand (2023)
1. Parkinson-Pat. ohne motorische Komplikationen (Fluktuationen/Dyskinesien), die unter einer Monotherapie mit den MAO-B-Hemmern Rasagilin oder Selegilin eine unzureichende Wirkung auf die motorischen Zielsymptome aufweisen, kann eine frühzeitige Kombinationstherapie mit Levodopa angeboten werden.<br>2. Bei Pat.-Gruppen mit erhöhtem Risikoprofil (hohes Lebensalter, hohe Komorbidität) und Notwendigkeit einer zusätzlichen Gabe von Levodopa zu einer vorbestehenden Behandlung mit einem MAO-B-Hemmer soll das Potenzial für Nebenwirkungen berücksichtigt und die Indikation für eine Fortführung des MAO-B-Hemmer individuell und kritisch geprüft werden.	
Konsensstärke: 100%, starker Konsens	

Wichtige Forschungsfragen:

Die Datenlage zur frühen Kombinationstherapie mit Rasagilin und Levodopa aus randomisierten, doppelblinden klinischen Studien bei Parkinson-Pat. ohne motorische Wirkfluktuationen ist unzureichend und beruht bislang weitestgehend auf einer Extrapolation von deren Effekten in der Monotherapie der frühen PK.

Randomisierte kontrollierte Studien zur Effizienz und Sicherheit einer frühen Kombinationstherapie mit Safinamid und Levodopa fehlen bislang vollständig und wären angesichts dessen unterschiedlicher Wirkansätze interessant und wünschenswert.

Referenzen

1. Stocchi F, Rascol O, Kieburtz K, Poewe W, Jankovic J, Tolosa E, et al. Initiating levodopa/carbidopa therapy with and without entacapone in early Parkinson disease: the STRIDE-PD study. Ann Neurol. 2010;68(1):18-27
2. Giménez-Roldán S, Tolosa E, Burguera JA, Chacón J, Liaño H, Forcadell F. Early combination of bromocriptine and levodopa in Parkinson's disease: a prospective randomized study of two parallel groups over a total follow-up period of 44 months including an initial 8-month double-blind stage. Clin Neuropharmacol. 1997;20(1):67-76
3. Przuntek H, Welzel D, Schwarzmann D, Letzel H, Kraus PH. Primary combination therapy of early Parkinson's disease. A long-term comparison between the combined regimen bromocriptine/levodopa and levodopa monotherapy--first interim report. Eur Neurol. 1992;32 Suppl 1:36-45
4. Vermersch P, Fondarai J, Petit H. [Randomized study during a year of early combination of L-dopa/lisuride in Parkinson disease]. Therapie. 1991;46(6):481-6
5. Suwantamee J, Nidhinandana S, Srisuwananukorn S, Laptikultham S, Pisarnpong A, Chankrachang S, Bundhukul A. Efficacy and safety of piribedil in early combination with L-dopa in the treatment of Parkinson's disease: a 6-month open study. J Med Assoc Thai. 2004;87(11):1293-300
6. Mochizuki H, Hattori N, Hasegawa K, Nomoto M, Uchida E, Terahara T, et al. Long-term study of ropinirole patch in Parkinson's disease patients with/without basal l-dopa. Parkinsonism Relat Disord. 2021;83:105-9

7. Weiner WJ, Factor SA, Sanchez-Ramos JR, Singer C, Sheldon C, Cornelius L, Ingenito A. Early combination therapy (bromocriptine and levodopa) does not prevent motor fluctuations in Parkinson's disease. Neurology. 1993;43(1):21-7
8. Rinne UK. [Combination therapy with lisuride and L-dopa in the early stages of Parkinson's disease decreases and delays the development of motor fluctuations. Long-term study over 10 years in comparison with L-dopa monotherapy]. Nervenarzt. 1999;70 Suppl 1:S19-25
9. Wong KS, Lu CS, Shan DE, Yang CC, Tsoi TH, Mok V. Efficacy, safety, and tolerability of pramipexole in untreated and levodopa-treated patients with Parkinson's disease. J Neurol Sci. 2003;216(1):81-7
10. Pogarell O, Gasser T, van Hilten JJ, Spieker S, Pollentier S, Meier D, Oertel WH. Pramipexole in patients with Parkinson's disease and marked drug resistant tremor: a randomised, double blind, placebo controlled multicentre study. J Neurol Neurosurg Psychiatry. 2002;72(6):713-20
11. Ziegler M, Castro-Caldas A, Del Signore S, Rascol O. Efficacy of piribedil as early combination to levodopa in patients with stable Parkinson's disease: a 6-month, randomized, placebo-controlled study. Mov Disord. 2003;18(4):418-25
12. Castro-Caldas A, Delwaide P, Jost W, Merello M, Williams A, Lamberti P, et al. The Parkinson-Control study: a 1-year randomized, double-blind trial comparing piribedil (150 mg/day) with bromocriptine (25 mg/day) in early combination with levodopa in Parkinson's disease. Mov Disord. 2006;21(4):500-9
13. Pringsheim T, Day GS, Smith DB, Rae-Grant A, Licking N, Armstrong MJ, et al. Dopaminergic Therapy for Motor Symptoms in Early Parkinson Disease Practice Guideline Summary: A Report of the AAN Guideline Subcommittee. Neurology. 2021;97(20):942-57
14. Sy MAC, Fernandez HH. Pharmacological Treatment of Early Motor Manifestations of Parkinson Disease (PD). Neurotherapeutics. 2020;17(4):1331-8
15. Adler CH, Sethi KD, Hauser RA, Davis TL, Hammerstad JP, Bertoni J, et al. Ropinirole for the treatment of early Parkinson's disease. The Ropinirole Study Group. Neurology. 1997;49(2):393-9
16. Hubble JP, Koller WC, Cutler NR, Sramek JJ, Friedman J, Goetz C, et al. Pramipexole in patients with early Parkinson's disease. Clin Neuropharmacol. 1995;18(4):338-47
17. Safety and efficacy of pramipexole in early Parkinson disease. A randomized dose-ranging study. Parkinson Study Group. Jama. 1997;278(2):125-30
18. Rinne UK, Bracco F, Chouza C, Dupont E, Gershanik O, Marti Masso JF, et al. Cabergoline in the treatment of early Parkinson's disease: results of the first year of treatment in a double-blind comparison of cabergoline and levodopa. The PKDS009 Collaborative Study Group. Neurology. 1997;48(2):363-8
19. A controlled trial of rotigotine monotherapy in early Parkinson's disease. Arch Neurol. 2003;60(12):1721-8
20. Stowe RL, Ives NJ, Clarke C, van Hilten J, Ferreira J, Hawker RJ, et al. Dopamine agonist therapy in early Parkinson's disease. Cochrane Database Syst Rev. 2008(2):Cd006564
21. Tan EK. Dopamine agonists and their role in Parkinson's disease treatment. Expert Rev Neurother. 2003;3(6):805-10
22. Inzelberg R, Carasso RL, Schechtman E, Nisipeanu P. A comparison of dopamine agonists and catechol-O-methyltransferase inhibitors in Parkinson's disease. Clin Neuropharmacol. 2000;23(5):262-6
23. Fox SH, Katzenschlager R, Lim SY, Barton B, de Bie RMA, Seppi K, et al. International Parkinson and movement disorder society evidence-based medicine review: Update on treatments for the motor symptoms of Parkinson's disease. Mov Disord. 2018;33(8):1248-66
24. Pramipexole vs levodopa as initial treatment for Parkinson disease: A randomized controlled trial. Parkinson Study Group. Jama. 2000;284(15):1931-8

25. Oertel W, Schulz JB. Current and experimental treatments of Parkinson disease: A guide for neuroscientists. J Neurochem. 2016;139 Suppl 1:325-37
26. Rascol O, Goetz C, Koller W, Poewe W, Sampaio C. Treatment interventions for Parkinson's disease: an evidence based assessment. Lancet. 2002;359(9317):1589-98
27. Raeder V, Boura I, Leta V, Jenner P, Reichmann H, Trenkwalder C, et al. Rotigotine Transdermal Patch for Motor and Non-motor Parkinson's Disease: A Review of 12 Years' Clinical Experience. CNS Drugs. 2021;35(2):215-31
28. Gray R, Ives N, Rick C, Patel S, Gray A, Jenkinson C, et al. Long-term effectiveness of dopamine agonists and monoamine oxidase B inhibitors compared with levodopa as initial treatment for Parkinson's disease (PD MED): a large, open-label, pragmatic randomised trial. Lancet. 2014;384(9949):1196-205
29. Jost WH. A critical appraisal of MAO-B inhibitors in the treatment of Parkinson's disease. J Neural Transm (Vienna). 2022;129(5-6):723-36
30. Effects of tocopherol and deprenyl on the progression of disability in early Parkinson's disease. N Engl J Med. 1993;328(3):176-83
31. Mizuno Y, Hattori N, Kondo T, Nomoto M, Origasa H, Takahashi R, et al. A Randomized Double-Blind Placebo-Controlled Phase III Trial of Selegiline Monotherapy for Early Parkinson Disease. Clin Neuropharmacol. 2017;40(5):201-7
32. Olanow CW, Hauser RA, Gauger L, Malapira T, Koller W, Hubble J, et al. The effect of deprenyl and levodopa on the progression of Parkinson's disease. Ann Neurol. 1995;38(5):771-7
33. Przuntek H, Conrad B, Dichgans J, Kraus PH, Krauseneck P, Pergande G, et al. SELEDO: a 5-year long-term trial on the effect of selegiline in early Parkinsonian patients treated with levodopa. Eur J Neurol. 1999;6(2):141-50
34. Larsen JP, Boas J. The effects of early selegiline therapy on long-term levodopa treatment and parkinsonian disability: an interim analysis of a Norwegian--Danish 5-year study. Norwegian-Danish Study Group. Mov Disord. 1997;12(2):175-82
35. Sivertsen B, Dupont E, Mikkelsen B, Mogensen P, Rasmussen C, Boesen F, Heinonen E. Selegiline and levodopa in early or moderately advanced Parkinson's disease: a double-blind controlled short- and long-term study. Acta Neurol Scand Suppl. 1989;126:147-52
36. Myllylä VV, Heinonen EH, Vuorinen JA, Kilkku OI, Sotaniemi KA. Early selegiline therapy reduces levodopa dose requirement in Parkinson's disease. Acta Neurol Scand. 1995;91(3):177-82
37. Lees AJ. Comparison of therapeutic effects and mortality data of levodopa and levodopa combined with selegiline in patients with early, mild Parkinson's disease. Parkinson's Disease Research Group of the United Kingdom. Bmj. 1995;311(7020):1602-7
38. A controlled trial of rasagiline in early Parkinson disease: the TEMPO Study. Arch Neurol. 2002;59(12):1937-43
39. Olanow CW, Rascol O, Hauser R, Feigin PD, Jankovic J, Lang A, et al. A double-blind, delayed-start trial of rasagiline in Parkinson's disease. N Engl J Med. 2009;361(13):1268-78
40. A randomized placebo-controlled trial of rasagiline in levodopa-treated patients with Parkinson disease and motor fluctuations: the PRESTO study. Arch Neurol. 2005;62(2):241-8
41. Rascol O, Brooks DJ, Melamed E, Oertel W, Poewe W, Stocchi F, Tolosa E. Rasagiline as an adjunct to levodopa in patients with Parkinson's disease and motor fluctuations (LARGO, Lasting effect in Adjunct therapy with Rasagiline Given Once daily, study): a randomised, double-blind, parallel-group trial. Lancet. 2005;365(9463):947-54
42. Hattori N, Takeda A, Takeda S, Nishimura A, Kato M, Mochizuki H, et al. Efficacy and safety of adjunctive rasagiline in Japanese Parkinson's disease patients with wearing-off phenomena: A phase 2/3, randomized, double-blind, placebo-controlled, multicenter study. Parkinsonism Relat Disord. 2018;53:21-7

43. Zhang Z, Shao M, Chen S, Liu C, Peng R, Li Y, et al. Adjunct rasagiline to treat Parkinson's disease with motor fluctuations: a randomized, double-blind study in China. Transl Neurodegener. 2018;7:14
44. Zhang L, Zhang Z, Chen Y, Qin X, Zhou H, Zhang C, et al. Efficacy and safety of rasagiline as an adjunct to levodopa treatment in Chinese patients with Parkinson's disease: a randomized, double-blind, parallel-controlled, multi-centre trial. Int J Neuropsychopharmacol. 2013;16(7):1529-37
45. Rabey JM, Sagi I, Huberman M, Melamed E, Korczyn A, Giladi N, et al. Rasagiline mesylate, a new MAO-B inhibitor for the treatment of Parkinson's disease: a double-blind study as adjunctive therapy to levodopa. Clin Neuropharmacol. 2000;23(6):324-30
46. Tetrud JW, Langston JW. The effect of deprenyl (selegiline) on the natural history of Parkinson's disease. Science. 1989;245(4917):519-22
47. Myllylä VV, Sotaniemi KA, Vuorinen JA, Heinonen EH. Selegiline as initial treatment in de novo parkinsonian patients. Neurology. 1992;42(2):339-43
48. Pålhagen S, Heinonen EH, Hägglund J, Kaugesaar T, Kontants H, Mäki-Ikola O, et al. Selegiline delays the onset of disability in de novo parkinsonian patients. Swedish Parkinson Study Group. Neurology. 1998;51(2):520-5
49. Allain H, Cougnard J, Neukirch HC. Selegiline in de novo parkinsonian patients: the French selegiline multicenter trial (FSMT). Acta Neurol Scand Suppl. 1991;136:73-8
50. Heinonen EH, Myllylä V. Safety of selegiline (deprenyl) in the treatment of Parkinson's disease. Drug Saf. 1998;19(1):11-22
51. Myllylä VV, Sotaniemi KA, Hakulinen P, Mäki-Ikola O, Heinonen EH. Selegiline as the primary treatment of Parkinson's disease--a long-term double-blind study. Acta Neurol Scand. 1997;95(4):211-8
52. Perez-Lloret S, Rascol O. Safety of rasagiline for the treatment of Parkinson's disease. Expert Opin Drug Saf. 2011;10(4):633-43
53. Tolosa E, Stern MB. Efficacy, safety and tolerability of rasagiline as adjunctive therapy in elderly patients with Parkinson's disease. Eur J Neurol. 2012;19(2):258-64

3.4 Fluktuationen

Autoren: Paul Lingor, Per Odin, Joseph Claßen

In den ersten Erkrankungsjahren kann bei den meisten Parkinson-Erkrankten mit einer mehrmals täglichen pulsatilen Gabe dopaminerger Medikamente eine gleichmäßige Einstellung motorischer und auch einiger nicht motorischer Symptome erreicht werden. Im weiteren Verlauf der Erkrankung nimmt die Anzahl der dopaminergen Neurone in der Substantia nigra weiter ab und ist nicht mehr ausreichend in der Lage, pulsatil aufgenommenes Levodopa kontinuierlich abzugeben [1]. Dadurch kommt es zu einem für Pat. spürbaren Wirkverlust vor der nächsten Medikamentengabe. Dabei wird zunächst implizit eine dreimal tägliche Einnahme unterstellt, das entspricht Einnahmeintervallen von ca. 4–5 Stunden. Umgekehrt wird die Einnahme der Medikation von einer spürbaren Besserung der Symptome begleitet, was v.a. motorische, aber auch einige nicht motorische Symptome betrifft. Dieser Wirkverlust – meist das erste Anzeichen beginnender Fluktuationen – wird auch als „wearing-off“ bezeichnet und betrifft ca. 40–50% aller Pat. nach etwa 5 Jahren Behandlungsdauer mit Levodopa [2]. Ein Screening für „wearing-off“ kann in der Praxis mit einem einfachen Fragebogen, dem WOQ-9, durchgeführt werden [3]. Im weiteren Verlauf kommen dann komplexere Wirkungsschwankungen (Fluktuationen) hinzu, die motorisch durch Überbeweglichkeit (Dyskinesien) oder Unterbeweglichkeit (Off-Phasen) gekennzeichnet sind, welche zunehmend schlechter mit den pulsatilen Einnahmezeitpunkten korrelieren. Auch nicht motorische Symptome können fluktuieren [4] und sind in individuelle Therapieentscheidungen einzubeziehen.

Fragestellung 54: Wie effektiv ist „retardiertes“ Levodopa im Vergleich mit Standard-Levodopa in der Behandlung von Parkinson-Pat. mit Fluktuationen?

Hintergrund

Bei retardierten Levodopa-Präparationen werden die maximalen Plasmakonzentrationen bei annähernd gleichen Plasmahalbwertszeiten im Vergleich zu Levodopa-Standardpräparaten später erreicht und im Plasma für etwas längere Zeit wirksame Levodopa-Spiegel aufgebaut, sodass die Wirkdauer von Levodopa verlängert werden kann. Levodopa/Carbidopa mit kontrollierter Freisetzung und Levodopa/Benserazid mit kontrollierter Freisetzung sind in Deutschland auf dem Markt erhältlich.

Evidenzgrundlage

Diese Empfehlungen basieren auf 2 Übersichtsartikeln zu Levodopa-Formulierungen [5], [6]. Die Übersichtsartikel zu den älteren Formen von L-Dopa mit verzögerter Freisetzung basieren auf 11 randomisierten/kontrollierten Studien sowie 1 Review zur IPX066 [7]. Die Übersichtsartikel zu IPX066 basieren auf 3 randomisierten Studien.

Ergebnis

Wirksamkeit

Bei retardierten Levodopa-Präparationen werden die maximalen Plasmakonzentrationen bei annähernd gleichen Plasmahalbwertszeiten im Vergleich zu Levodopa-Standardpräparaten später

erreicht und im Plasma für etwas längere Zeit wirksame Levodopa-Spiegel aufgebaut, sodass die Wirkdauer von Levodopa verlängert werden kann. Die Aufnahme von retardiertem Levodopa ist allerdings stärker von der Nahrungsaufnahme abhängig als die Aufnahme der Standardformulierung, da Letztere wegen seiner kürzeren Verweildauer im Gastrointestinaltrakt erlaubt, den Abstand zwischen Levodopa-Gabe und Nahrungsmittelaufnahme abzustimmen.

Die Evidenz lässt annehmen, dass retardiertes Levodopa motorische Fluktuationen zufriedenstellend reduzieren kann. In der klinischen Praxis findet sich allerdings teils auch eine Verlängerung der Off-Zeit nach direktem Wechsel von nicht retardiertem zu retardiertem Levodopa. Dieser Effekt ist möglicherweise auf die schlechtere intestinale Absorption von retardiertem Levodopa zurückzuführen. Aus diesem Grund wird retardiertes Levodopa im klinischen Alltag nur selten zur Optimierung motorischer Komplikationen tagsüber eingesetzt, sondern in der Regel nachts bei leerem Magen. Das Nebenwirkungsmuster ist zwischen den verschiedenen Zubereitungsformen ähnlich. Zusammenfassend mag eine Kombination aus retardiertem (nachts) und nicht retardiertem Levodopa (tagsüber) nützlich zur Therapie von motorischen Komplikationen sein.

Eine Reihe neuer Levodopa-Formulierungen mit längerer Wirkungsdauer sind/waren in der Entwicklung. IPX066 ist bereits in einigen Märkten erhältlich. Es handelt sich um eine oral verabreichte Levodopa/Carbidopa-haltige Formulierung mit modifizierter Wirkstofffreisetzung. Es wird schnell resorbiert, ähnlich wie herkömmliche Levodopa-Präparate mit sofortiger Wirkstofffreisetzung. Die IPX066-Kapsel setzt während ihrer Passage durch den Magen-Darm-Trakt kontinuierlich Levodopa frei. IPX066 sorgt über längere Zeiträume für konstantere therapeutische Levodopa-Plasmakonzentrationen. Darüber hinaus zeigte das IPX066-Studienprogramm eine überlegene Wirksamkeit von IPX066 gegenüber herkömmlichen oralen Levodopa/Carbidopa-Präparaten zur Behandlung von motorischen Komplikationen, insbesondere bei Off-Fluktuationen. IPX066 reduzierte auch die Häufigkeit der oralen Einnahme von Levodopa, wobei jedoch insgesamt höhere Dosierungen als bei herkömmlichen Präparaten erforderlich waren. IPX066 ist noch nicht auf dem deutschen Markt erhältlich und bekommt deswegen keine Empfehlung.

Begründung der Empfehlung

Evidenz deutet darauf hin, dass die auf dem europäischen Markt erhältlichen langwirksamen L-Dopa-Präparate die Off-Zeit bei fluktuierenden Pat. verkürzen könnten. Die klinische Erfahrung zeigt jedoch, dass die Probleme mit Fluktuationen oft am ehesten zunehmen, wenn diese Präparate tagsüber verwendet werden. Als Therapie für die Nacht ist die Behandlung jedoch gut etabliert.

**Empfehlung**	**Neu**<br>**Stand (2023)**
Retardierte Darreichungsformen von Levodopa mit Decarboxylasehemmer können zur Behandlung von Parkinson-Pat. mit Fluktuationen eingesetzt werden. Aufgrund der längeren Resorptionszeit sollten diese Präparate nicht während der Wachzeit, sondern nur zur Behandlung der Parkinson-Symptome während der Nachtzeit eingesetzt werden.	
Konsensstärke: 100%, starker Konsens	

Fragestellung 55: Wie effektiv ist schnell lösliches Levodopa im Vergleich mit Standard-Levodopa in der Behandlung von Parkinson-Pat. mit Fluktuationen?

Hintergrund

Die chronische Levodopa-Behandlung ist bei der Mehrheit der Parkinson-Pat. mit der Entwicklung potenziell behindernder motorischer Fluktuationen verbunden. In einer Pat.-Kohorte mit einer Off-Zeit von 5–6 h/d kann mithilfe herkömmlicher verschiedener Add-on-Therapien (Dopaminagonisten, COMT-Hemmer, MAO-B-Hemmer) unter Beachtung des Placebo-Effekts die Off-Zeit um ca. 50% reduziert werden [8], was nach wie vor eine therapeutisch unbefriedigende Situation darstellt. Herkömmlich formuliertes Levodopa verbessert die Beweglichkeit nach ungefähr 1 h, was für die Behandlung von Off-Zuständen zu spät ist. Orales lösliches Levodopa ist seit vielen Jahren verfügbar und entwickelt worden, um einen potenziell schnelleren Eintritt der Levodopa-Wirkung zu ermöglichen und damit Off-Zustände schneller aufzulösen. Orales lösliches Levodopa könnte zudem prinzipiell die potenziell schwerwiegenden Nachteile einer kontinuierlichen intraintestinalen Infusion von Levodopa vermeiden.

Evidenzgrundlage

1 Übersicht [9], 1 randomisierte kontrollierte Studie [10], 1 Open-label-Studie [11]

Ergebnis

Zu löslichen Formen von Levodopa konnten keine Studien identifiziert werden, die die Wirkung bei Fluktuationen untersuchen. Die Wirkungen einer löslichen Form Melevodopa, einem Prodrug von Levodopa, das zu einer im Vergleich mit herkömmlichen Formulierungen schnelleren duodenalen Aufnahme führt, wurden in einer randomisierten, doppelblinden, double-dummy, kontrollierten Parallelgruppen-Studie an 221 Pat. mit motorischen Fluktuationen, mit Levodopa in einer Standardformulierung verglichen. Es fanden sich keine Unterschiede hinsichtlich des primären Endpunkts der täglichen Off-Zeit. Es bleibt jedoch unklar, inwieweit die Ergebnisse auf die lösliche Formulierung von Levodopa, die unter dem Markennamen Madopar LT © vertrieben wird, übertragbar ist. In einer Studie [11] wurden die Pharmakokinetik, die Sicherheit, Verträglichkeit und Wirksamkeit von löslichem Levodopa bei 18 Pat. mit fortgeschrittener PK und motorischen Fluktuationen mit intermittierender Gabe von Levodopa in herkömmlicher Formulierung und Standarddosen verglichen. Für die Zwecke dieser Studie war eine „kontinuierliche“ Gabe von Levodopa definiert als schluckweise Verabreichung einer L-Dopa-Dispersion alle 5 bis 10 Minuten. Die Variabilität der L-Dopa-Konzentration im Plasma war bei kontinuierlicher gegenüber intermittierender oraler L-Dopa/Carbidopa-Behandlung geringer. Die mittlere Off-Zeit wurde durch die kontinuierliche orale L-Dopa-Therapie um 43% ($P < 0,001$) verkürzt. Es wurden keine Probleme mit der Sicherheit oder Verträglichkeit beobachtet. Die kontinuierliche orale Verabreichung von L-Dopa/Carbidopa war mit einer geringeren Plasmavariabilität und einer reduzierten Off-Zeit im Vergleich zur intermittierenden oralen L-Dopa/Carbidopa-Standardtherapie verbunden.

Begründung der Empfehlung

Es existieren keine kontrollierten Studien zur Wirksamkeit von löslichem Levodopa auf Fluktuationen in bedarfsweisem Einsatz.

**Empfehlung**	**Neu**<br>**Stand (2023)**
1. Lösliches Levodopa kann eingesetzt werden, um nach dem Aufwachen morgens einen gegenüber herkömmlichen Formulierungen schnelleren Wirkungseintritt auf die Beweglichkeit zu erzielen.<br>2. Lösliches Levodopa kann zum bedarfsweisen Einsatz bei tagsüber und unvorhergesehen auftretenden Off-Zuständen eingesetzt werden.	
Konsensstärke: 100%, starker Konsens	

Wichtige Forschungsfragen:

Welche Wirkung hat lösliches Levodopa auf das morgens nach dem Aufwachen bestehende Off im Vergleich zu Standardformulierungen von Levodopa?

Welche Wirkung hat lösliches Levodopa bei tagsüber und unvorhergesehen auftretenden Off-Zuständen im Vergleich zu On-demand-Therapien und im Vergleich zu Levodopa in Standardformulierungen?

Fragestellung 56: Wie effektiv ist inhalatives Levodopa im Vergleich mit Standard-Levodopa in der Behandlung von Pat. mit PK mit Fluktuationen?

Hintergrund

Inhalatives Levodopa-Pulver (Markenname: Inbrija®) ist eine neuartige Bedarfstherapie zur Behandlung von Off-Episoden für Pat., die Levodopa in Kombination mit Dopadecarboxylase-Hemmer einnehmen. Levodopa wird durch Inhalation unter Verwendung eines Inhalationsgeräts verabreicht, um die Plasmakonzentration von Levodopa schneller durch Umgehung der Magen-Darm-Passage zu erhöhen. Pro Off-Episode beträgt die Dosis 84 mg, für die die Pat. nacheinander 2 Kapseln mit einer Menge von je 42 mg in das selbst zu bedienende Inhalationsgerät einsetzen. Die tatsächlich inhalierte Dosis beträgt 66 mg Levodopa (2*33 mg). In pharmakokinetischen Studien (im vollständigen Off) stieg die maximale Plasmakonzentration nach einer 84-mg-Dosis inhalativen Levodopas durchschnittlich auf 0,6 µg/ml an. Da die typische Schwellenserumkonzentration für den On-Zustand bei etwa 1,0 µg/ml liegt, müssen die Pat. das inhalierbare Levodopa selbst so zeitig verabreichen, dass die zusätzliche inhalierte Dosis ausreicht, um die Gesamtkonzentration über diese Schwelle zu heben. Pat. müssen also in der Lage sein, den heraufziehenden Off-Zustand zu erkennen. Die durch inhalatives Levodopa erreichbare Zunahme der Serumkonzentration bedeutet, dass eine Anwendung inhalativen Levodopas zur Behandlung von Off-Zuständen nach dem Erwachen aus dem Schlaf und vor der Einnahme der ersten oralen Levodopa-Dosis nicht ausreicht, um einen On-Zustand zu erreichen. Das Konzentrationsmaximum (5–15 Minuten nach der Inhalation) war in einer

Vergleichsstudie mit inhalativem Levodopa nach Einnahme einer fett- und proteinreichen Mahlzeit schneller erreicht als mit Levodopa/Carbidopa in herkömmlicher Formulierung [12].

Evidenzgrundlage

1 Metaanalyse [13], 2 Übersichten [14], [15], 4 randomisierte doppelblinde kontrollierte Studien [16], [17], [18] [19]; 1 offene, randomisierte Studie [20] und 2 weitere Studien [21], [12].

Ergebnis

Eine Metaanalyse [13] analysierte die Ergebnisse von 4 doppelblinden randomisierten Studien und 1 offenen randomisierten Studie. Inhalatives Levodopa führte häufiger als Placebo zu einem On-Zustand, verbesserte die Beweglichkeit anhand des motorischen UPDRS stärker und führte aus Pat.-Sicht zu einer größeren globalen Verbesserung. Respiratorische Symptome (Husten, farbloser Auswurf, Rachenirritationen) und Schwindel waren die am häufigsten festgestellten Nebenwirkungen. Wegen methodischer Probleme wurde insgesamt eine nur mittlere Qualität der Evidenz in Bezug auf das Erreichen eines On-Zustands und wegen der Heterogenität der Studienergebnisse nur eine niedrige Qualität der Evidenz für eine motorische Besserung ermittelt. Weil die Studien nur Pat. mit Hoehn-&-Yahr-Stadien I–III einschlossen, konnten keine Aussagen zu Pat. in höheren Hoehn-&-Yahr-Stadien getroffen werden.

Die „Safety and Efficacy of Inhaled Levodopa for the Treatment of Parkinson’s Disease Motor Symptoms“ (SPAN)-Studie für das inhalative Levodopa-Pulver war eine randomisierte, doppelblinde, placebokontrollierte, multizentrische Phase-III-Studie. In diese Studie wurden PK-Pat. im Alter zwischen 30 und 85 Jahren eingeschlossen, die motorische Fluktuationen und mindestens 2 Stunden tägliche Off-Zeit erlebten und eine Verbesserung von 25% oder mehr im motorischen UPDRS-Score vom Off- zum On-Zustand nach der Einnahme einer oralen Levodopa/Dopadecarboxylase-Inhibitor-Kombination zeigten. Die Pat. inhalierten entweder Placebo oder Levodopa-Pulver in einer von zwei Dosierungen (60 mg und 84 mg). Primärer Endpunkt war die Veränderung des motorischen MDS-UPDRS-Werts von vor der Einnahme bis 30 Minuten nach der Einnahme in der 12. Woche während eines Off-Zustands in der Klinik, in der Verumgruppe (84 mg) im Vergleich zur Placebogruppe. Es wurden 351 Pat. randomisiert (60 mg, 115 Pat.; 84 mg 120 Pat., Placebo 116 Pat.), von denen 290 die Studie beendeten. Im Mittel nahm der UPDRS-Wert 30 Minuten nach der Einnahme in der Verumgruppe (84 mg) (-9,8 Punkte) signifikant stärker ab als nach Placebo (-5,9 Punkte).

Die Frage, ob inhalatives Levodopa auch wirksam ist, wenn nach dem Nachtschlaf noch kein On vorgelegen hat, wurde in einer randomisierten, doppelblinden Studie mit 2-fachem Crossover-Design untersucht [17]. 36 Pat. im Morgen-Off (die über Nacht keine Parkinson-Medikamente erhalten hatten) erhielten an zwei Tagen je eine Einzeldosis von inhalativem Levodopa (84 mg) oder Placebo unmittelbar nach ihrer ersten morgendlichen oralen Levodopa/Carbidopa-Dosis. Bei 36 Pat., die die Studie beendeten, lag die mediane Zeit bis zum On-Zustand bei 25,0 Minuten nach Levodopa/Carbidopa plus Levodopa-Pulver und bei 35,5 Minuten nach Levodopa/Carbidopa plus Placebo (p=0,26). Nach 30 Minuten hatten mehr Pat. nach Levodopa/Carbidopa plus Levodopa-Pulver (66,7%) ein On erreicht als Pat. mit Levodopa/Carbidopa plus Placebo (44,5%) (p=0,040).

Inhalierbares Levodopa-Pulver ist für die Behandlung von Off-Episoden unter Levodopa/Dopa-decarboxylase-Hemmer zugelassen. Frühmorgendliche Off-Episoden sind von der Zulassung nicht ausgenommen.

Begründung der Empfehlung

Die Empfehlung stützt sich auf 1 Metaanalyse und Sichtung von 4 randomisierten, doppelblinden, placebokontrollierten Studien und 1 offenen randomisierten Studie. Ein direkter Vergleich zu anderen On-demand-Therapien fehlt ebenso wie ein Vergleich zur Applikation von Levodopa in Standardformulierung. Real-world-Daten und Langzeitdaten zur Wirksamkeit und Sicherheit der Anwendung von inhalativem Levodopa fehlen.

Empfehlung	Neu<br>Stand (2023)
Inhalatives Levodopa kann bedarfsweise bei tagsüber und unvorhergesehen auftretenden Off-Zuständen eingesetzt werden.	
Konsensstärke: 100%, starker Konsens	

Wichtige Forschungsfragen:

Welche Wirkung hat inhalatives Levodopa auf das morgens nach dem Aufwachen bestehende Off im Vergleich zu löslichem Levodopa?

Welche Wirkung hat inhalatives Levodopa bei tagsüber und unvorhergesehen auftretenden Off-Zuständen im Vergleich zu anderen On-demand-Therapien und zu Levodopa in Standardformulierung?

Fragestellung 57: Wie effektiv ist die zusätzliche Gabe von Dopaminagonisten im Vergleich mit Placebo in der Behandlung von Parkinson-Pat. mit Fluktuationen?

Hintergrund

Zusätzliche Dopaminagonisten werden empfohlen als Behandlung der Wahl für fortgeschrittene Parkinson-Pat. mit motorischen Fluktuationen. Trotz der weit verbreiteten Verabreichung von Dopaminagonisten als Zusatztherapie auf Levodopa zur Behandlung von Pat. mit fortgeschrittener PK mit Fluktuationen fehlt der Vergleich bezüglich Effekt und Risiken der verschiedenen verfügbaren Dopaminagonisten.

Evidenzgrundlage

Diese Empfehlungen basieren auf 5 systematischen Übersichtsartikeln zur Dopaminagonistentherapie bei PK mit motorischen Fluktuationen ([22] (20 Studien), [23] (40 Studien), [8] (44 Studien), [24] (45 Studien), [25] (68 Studien)) sowie 1 systematischen Review zur Apomorphin-Infusion [26] (8 Studien)), 1 Konsensus-Publikation zur Apomorphin-Behandlung [27] sowie 1 Übersichtsartikel [28] und 1 RCT zum Apomorphin sublingual [29].

Ergebnis

Wirksamkeit

Alle heute in Deutschland verfügbaren Dopaminagonisten haben in Zulassungsstudien nachgewiesen, dass sie im fortgeschrittenen Stadium der PK zur Reduktion von motorischen Fluktuationen führen. Der Zusatz von Dopaminagonisten zur Levodopa-Behandlung bei fortgeschrittenen Parkinson-Pat. mit Motorfluktuationen führt zu: 1. verlängerter Zeit im On ohne störende Dyskinesien, 2. reduzierter Zeit im Off. 3. verbesserter motorischer Symptomatologie im On (laut UPDRS III) 4. verbessertem UPDRS II (ADL)-Score. Diese Wirkungen werden sowohl bei den retardierten (einmal täglich) als auch bei den nicht retardierten (3-mal täglich) Formen von Pramipexol und Ropinirol beobachtet. Es gibt Tendenzen in Richtung eines unterschiedlichen Wirkungsprofils zwischen den verschiedenen Dopaminagonisten, aber keine sicher signifikanten. Es wurde zum Beispiel kein signifikanter Unterschied in der Wirksamkeit zwischen den Dopaminagonisten bez. Off-Zeit- und Dyskinesie-Zeit-Reduktion beobachtet.

Bei Einleitung einer Therapie mit einem Dopaminagonisten ist zu beachten, dass es sich bei diesen grundsätzlich primär um non-ergoline Substanzen (Pramipexol, Ropinirol, Rotigotin, Piribedil und mit Einschränkungen Apomorphin) handeln muss, da dies in Fachinformationen beziehungsweise regulatorischen Anforderungen festgelegt ist. Die fibrotischen Veränderungen sind für alle ergolinen Dopaminagonisten mit Ausnahme von Lisurid (nicht mehr zugelassen) belegt. Sie sind in der Regel irreversibel. Da nicht ganz klar ist, ob diese fibrotischen Veränderungen einer Dosis-Wirkungs-Beziehung unterliegen, ist für die genannten Dopaminagonisten eine (relativ niedrige) Tageshöchst-dosis festgelegt worden. Es wird daher empfohlen, vor Beginn der Therapie eine Echokardiographie, einen Nierenfunktionstest, eine BSG-Bestimmung und eine Röntgen-Thoraxaufnahme durchzuführen, die Echokardiographie sollte alle 12 Monate wiederholt werden. Die Wiederholungsrate der übrigen genannten Untersuchungen obliegt der klinischen Einschätzung des/der Verordnenden. Es existiert keine Evidenz, dass ergoline Dopaminagonisten Wirksamkeit zeigen, wenn andere zugelassene Substanzen oder Maßnahmen keine zufriedenstellende Einstellung ermöglichen. Ergoline Dopaminagonisten haben eine mit nicht ergolinen Dopaminagonisten vergleichbare Wirksamkeit, jedoch ein ungünstigeres Nebenwirkungsprofil. Ihr Einsatz wird deshalb nicht empfohlen.

Der jeweilige Dopaminagonist soll entsprechend der Fachinformation gesteigert werden. Gleichzeitig ist darauf zu achten, dass Levodopa analog reduziert wird, um motorische und nicht motorische Nebenwirkungen der erhöhten dopaminergen Stimulation zu vermeiden.

Eine klinisch effektive Dosis entzieht sich zunächst einer operationalen Definition. Im Alltag empfiehlt sich, eine objektiv erfasste Verbesserung der Kardinalsymptome der PK (beispielsweise gemessen an der UPDRS) zu erheben. Dies ist mit allen non-ergolinen Dopaminagonisten grundsätzlich zu erreichen.

Störungen der Impulskontrolle können bei jeder dopaminergen Behandlung auftreten, aber das Risiko ist bei einer Behandlung mit Dopaminagonisten größer als bei einer Behandlung mit Levodopa. Störungen der Impulskontrolle können sich als unkontrolliertes Glücksspiel, Einkaufen, Essattacken, gesteigertes sexuelles Interesse und Agieren, gesteigerte Internetnutzung äußern und auch mit

Horten, Putzwahn, Hobbyismus in Verbindung gebracht werden. Das Risiko einer Impulskontrollstörung während der Behandlung mit Dopaminagonisten wurde in Prävalenzstudien auf 10–20 % geschätzt. Prospektive Studien haben gezeigt, dass fast 50 % der Pat., die mit Dopaminagonisten behandelt werden, irgendwann innerhalb von 5 Jahren eine Impulskontrollstörung entwickeln und dass die Inzidenz von Impulskontrollstörungen nach Absetzen von Dopaminagonisten abnimmt. Es ist sehr wichtig, Probleme mit der Impulskontrolle frühzeitig zu erkennen, denn eine unbehandelte Erkrankung kann maßgebliche Folgen für die Finanzen und das Zusammenleben haben. Man muss daher auch Angehörige über diese möglichen Nebenwirkungen informieren, da sie vom Pat. selten gemeldet werden. Das Risiko von Impulskontrollstörungen steigt mit höheren Dosen von Dopaminagonisten und einer Langzeitbehandlung. Das Risiko wurde auch mit jüngerem Alter, Beginn der PK in jungen Jahren, Alleinleben, männlichem Geschlecht, persönlicher oder familiärer Vorgeschichte von Spielsucht oder Alkoholismus, impulsiven Persönlichkeitsmerkmalen, aktuellem oder früherem Rauchen und Kaffeetrinken in Verbindung gebracht.

Es besteht ein Zusammenhang zwischen der Therapie mit Dopaminagonisten und der Tagesmüdigkeit. Bei manchen Pat. kann diese Müdigkeit ausgeprägt sein. Wenn die Dopaminagonisten mit anderen Parkinson-Medikamenten kombiniert werden, wird die Müdigkeit manchmal verstärkt. Zu Beginn einer Therapie mit Dopaminagonisten und bei Dosiserhöhungen sollten die Pat. daher über dieses Risiko aufgeklärt werden, einschließlich des erhöhten Risikos für unfreiwilliges Einschlafen während des Tages. Vor allem sollte man sich über die Gefahr plötzlicher Schlafattacken informieren. Wenn diese auftreten, sollten Pat. kein Kraftfahrzeug führen. Das Risiko für Müdigkeit ist möglicherweise etwas höher bei Pramipexol und Ropinirol, ist aber bei Behandlung mit allen Dopaminagonisten vorhanden. Pat., die ausgeprägte Somnolenz und/oder plötzliche Schlafattacken zeigen, sollten darüber informiert werden, dass sie auf Autofahren und andere Aktivitäten verzichten sollten, bei denen eine verminderte Aufmerksamkeit sie selbst oder andere gefährden könnte. Wenn diese Art von Müdigkeit auftritt, kann es sich lohnen, die Dosis zu verringern oder einen Dopaminagonisten mit einer anderen chemischen Struktur auszuprobieren.

Unter den Dopaminagonisten-Therapien nimmt die Apomorphin-Therapie eine Sonderstellung ein. Apomorphin-Injektionen nach Bedarf können verwendet werden, um Off-Perioden, die trotz oraler Therapie auftreten, schnell (innerhalb von 5–15 Minuten) zu beheben. Auch kontrollierte Studien zeigen eine deutliche Verkürzung der täglichen Zeit im Off. Eine andere Formulierung besteht aus sich schnell auflösenden Filmstreifen, die sublingual appliziert werden und verschiedene Mengen Apomorphin enthalten. Es hat sich in kontrollierten Studien gezeigt, dass auch dieses Medikament Off-Phasen beenden kann, wenn es als Bedarfsbehandlung eingesetzt wird (signifikante Reduktion im Vergleich zu Placebo im UPDRS III, Wirkeintritt ab 15 und Wirkdauer bis 90 Minuten)). Sowohl die subkutane als auch die sublinguale Behandlung können Übelkeit als Nebenwirkung hervorrufen, insbesondere zu Beginn der Therapie, und Domperidon kann als Prophylaxe eingesetzt werden. Die niedrigste wirksame Dosis ist individuell unterschiedlich und muss für jeden individuell titriert werden.

Praktische Ratschläge bei Dopaminagonistentherapie

- Die langwirksamen Formen von Pramipexol, Ropinirol und Rotigotin haben hauptsächlich Vorteile in Bezug auf das Arzneimittelmanagement und wahrscheinlich eine verbesserte Compliance.
- Vorsicht ist geboten, wenn Pat. älter sind und/oder an vaskulärer Dysautonomie leiden. Ebenso, wenn Pat. ein zuvor bekanntes Suchtproblem oder eine andere Störung der Impulskontrolle haben.
- Eine häufige Beobachtung ist, dass die Einfügung von Agonisten eine stimmungsaufhellende Wirkung hat, und Pramipexol war das erste Präparat, das in RCTs gezeigt hat, dass es eine Wirkung auf Depressionen bei der PK hat.
- Dopaminagonisten sollten langsam eingeführt werden, um Nebenwirkungen zu reduzieren. Die Übelkeit ist meist reversibel, während Blutdruckabfall und periphere Ödeme oft bestehen bleiben. Wenn Pat. die Einführung eines Agonisten nur schwer tolerieren, kann kurzzeitig Domperidon bis zu 10 mg 3 x täglich unter Berücksichtigung der kardialen Risiken angewendet werden.

Begründung der Empfehlung

Dopaminagonisten reduzieren die Zeit im Off pro Tag bei Pat. mit L-Dopa-Therapie und motorischen Fluktuationen. Ergoline Dopaminagonisten werden wegen des Fibroserisikos nicht mehr empfohlen. Apomorphin in Injektions- und Infusionsform oder sublingual reduziert die Zeit im Off pro Tag bei Pat. mit motorischen Schwankungen.

Empfehlung	Modifiziert<br>Stand (2023)
1. Dopaminagonisten können eingesetzt werden, um motorische Fluktuationen bei Pat. mit fortgeschrittener PK zu reduzieren.<br>2. Ergoline Dopaminagonisten (Bromocriptin, Cabergolin, Pergolid, Lisurid) sollen nicht mehr zur Therapie der PK eingesetzt werden.<br>3. Ein Dopaminagonist soll bis zu einer klinisch effektiven Dosis titriert werden. Sollten Nebenwirkungen dies verhindern, kann ein anderer Agonist oder eine andere Substanzklasse eingesetzt werden.<br>4. Intermittierende subkutane Apomorphininjektionen oder sublinguale Apomorphin-Gaben können zusätzlich zur oralen Therapie eingesetzt werden, um die tägliche Off-Dauer bei Pat. mit schweren motorischen Fluktuationen zu verkürzen.<br>5. Die kontinuierliche subkutane Apomorphin-Infusion kann eingesetzt werden, um Off-Dauer und Dyskinesien bei Pat. mit schweren motorischen Komplikationen zu bessern.	
Konsensstärke: 100%, starker Konsens	

Fragestellung 58: Wie effektiv ist die zusätzliche Gabe von Dopaminagonisten im Vergleich mit COMT-Hemmern in der Behandlung von Parkinson-Pat. mit Fluktuationen?

Hintergrund

Es existiert Evidenz aus kontrollierten Studien, dass bei Parkinson-Pat. mit Wirkfluktuationen die additive Gabe eines COMT-Hemmers (Entacapon, Tolcapon, Opicapon) oder eines Dopaminagonisten (Ropinirol, Pramipexol, Rotigotin, Apomorphin) die Off-Zeiten verringern und die Zeit im On-Zustand ohne störende Dyskinesien verbessent kann.

Evidenzgrundlage

1 Metaanalyse (Cochrane-Review) [8]; 1 Übersicht [9].

Ergebnis

Effektstärke von Nutzen und Risiken von Dopaminagonisten und COMT-Hemmern (sowie MAO-B-Hemmern), die als Zusatztherapie bei Parkinson-Pat. mit Fluktuationen eingesetzt werden, wurden 2010 in einer Cochrane-Metaanalyse in Form eines indirekten Vergleichs bewertet [8]. Daraus ergaben sich leichte Vorteile für die Gruppe der Dopaminagonisten. Für diesen Vergleich wurden Opicapon (und Safinamid sowie andere Substanzen) nicht berücksichtigt, weil sie zu diesem Zeitpunkt noch nicht zugelassen waren.

Die additive Gabe von COMT-Hemmern zu Levodopa mit entsprechenden Dosierungsintervallen kann zu geringeren Schwankungen der Levodopa-Plasmaspiegel führen.

Das klinische Nebenwirkungsprofil der COMT-Hemmer ist harmlos in Bezug auf die zu beobachtende gelbliche Urinverfärbung, während das Auftreten von Diarrhoe und/oder Anstieg der Leberenzyme zum Absetzen führt oder eine engmaschige Kontrolle notwendig macht. Der COMT-Hemmer Tolcapon ist trotz seiner zentralen Wirksamkeit dem nur peripher wirksamen COMT-Hemmer Entacapon auch aufgrund der Sicherheitsauflagen unterlegen und gilt als Mittel der zweiten Wahl.

Dopaminagonisten lösen vor allem in der Aufdosierungsphase Übelkeit und Schwindel aus. Kurz- oder langfristig kann es dosisabhängig zu Ödemen kommen. Vor diesem Hintergrund wird der additive Einsatz von COMT-Hemmern meist besser vertragen als die Gabe von Dopaminagonisten. Hinsichtlich der Vergleichbarkeit der Wirkung gibt es keine aussagekräftige Evidenz.

Begründung der Empfehlung

Es existieren keine zwischen dem additiven Einsatz von COMT-Hemmern und dem von Dopaminagonisten vergleichenden Studien bei Parkinson-Pat. mit Fluktuationen.

**Empfehlung**	**Geprüft**<br>**Stand (2023)**
Dopaminagonisten oder COMT-Hemmer können alternativ zur Behandlung von motorischen Komplikationen eingesetzt werden, sie unterscheiden sich durch ihr individuell unterschiedliches Nebenwirkungsprofil. Es gibt keine ausreichende Evidenz zur vergleichenden Beurteilung der Wirksamkeit der beiden Substanzklassen.	
Konsensstärke: 100%, starker Konsens	

Wichtige Forschungsfragen:

Direkter Vergleich der Wirkung von Dopaminagonisten und COMT-Hemmern zur Behandlung von Fluktuationen bei Parkinson-Pat.

Fragestellung 59: Wie effektiv ist die zusätzliche Gabe von Dopaminagonisten im Vergleich mit MAO-B-Hemmern in der Behandlung von Parkinson-Pat. mit Fluktuationen?

Hintergrund

Im fortgeschrittenen Stadium der PK wird meist eine Kombinationstherapie eingesetzt. Dies betrifft die Kombination von Levodopa mit Dopaminagonisten, COMT-Hemmern, MAO-B-Hemmern und weiteren Substanzen. Hier stellt sich die Frage, ob die zusätzliche Gabe von Dopaminagonisten oder von MAO-B-Hemmern effektiver ist.

Evidenzgrundlage

Für diese Kapitel: In dieser Literaturrecherche fand sich

- N=1 vergleichende Analyse [30].

Ergebnis

Direkte Vergleichsstudien zwischen Dopaminagonisten und MAO-B-Hemmern in fortgeschrittenen Stadien der PK wurden nicht gefunden.

Eine vergleichende Analyse [30], welche jedoch unterschiedliche Stadien der PK berücksichtigte und insgesamt 79 RCTs zu 4 Dopaminagonisten (Cabergolin, Pramipexol, Ropinirol, Rotigotin) und 3 MAO-B-Hemmern (Selegilin, Rasagilin, Safinamid) einschloss, kam zum Ergebnis, dass Dopaminagonisten allein und in Kombination mit Levodopa effektive Behandlungsmöglichkeiten darstellen. Das Gleiche galt auch für die MAO-B-Hemmer Rasagilin und Selegilin. In Kombination mit Levodopa zeigte Selegilin den besten Effekt, gefolgt von Pramipexol, Ropinirol, Rotigotin, Cabergolin, Rasagilin und Safinamid. Dopaminagonisten zeigten hier häufiger Nebenwirkungen.

Begründung der Empfehlung

Aufgrund dieser spärlichen Datenlage kann keine klare Empfehlung für eine der hier untersuchten Substanzgruppen gegeben werden. Selegilin und Pramipexol scheinen in einem gemischten Kollektiv von Parkinson-Erkrankten als zusätzliche Therapie zu Levodopa die besten Effekte gehabt zu haben. Cabergolin ist nicht mehr als First-line-Dopaminagonist zugelassen. Piribedil ist nicht untersucht worden. Da ein direkter Vergleich im Sinne der Fragestellung nicht möglich ist, wird auf die entsprechenden Empfehlungen zum Einsatz von Dopaminagonisten bzw. MAO-B-Hemmern verwiesen.

Empfehlung	Neu<br>Stand (2023)
Eine klare Empfehlung für den bevorzugten Einsatz einer der beiden Substanzklassen kann nicht abgegeben werden.	

Konsensstärke: 100%, starker Konsens

Wichtige Forschungsfragen:

Vergleichende Studien, die die Wirksamkeit von Dopaminagonisten mit derjenigen von MAO-B-Hemmern zusätzlich zu Levodopa bei fortgeschrittenen Parkinson-Erkrankten vergleichen, fehlen. Ob solche Studien aufgrund der Vielzahl der zugelassenen Substanzen und Therapieoptionen jemals durchgeführt werden, ist fraglich.

Fragestellung 60: Wie effektiv sind Apomorphin-Formulierungen im Vergleich mit Standard-Levodopa in der Behandlung von Parkinson-Pat. mit Fluktuationen?

Hintergrund

Nach zunächst erfolgreicher Therapie des Dopamin-Mangels kommt es beim Großteil der Parkinson-Pat. zur Entwicklung motorischer Komplikationen: Die Wirkdauer einzelner Medikamente wird merkbar kürzer und kann im weiteren Verlauf unvorhersehbar werden und zum Teil ganz ausbleiben. In diesen Off-Phasen treten die ursprünglichen Parkinson-Symptome hervor, aber auch Freezing, dystone Verkrampfungen und nicht motorische Symptome wie Schmerzen und emotionale, kognitive und autonome Veränderungen. Apomorphin, der stärkste Dopaminagonist, kann zur raschen und verlässlichen Beendigung von solchen Off-Zuständen eingesetzt werden, die trotz Anpassungen der oralen Therapie durch Fraktionierung der Levodopa-Gabe, andere Levodopa-Formulierungen, durch Zusatztherapie mit Dopaminagonisten, COMT-Hemmern oder MAO-B-Hemmern störend bleiben. Aufgrund geringer oraler Bioverfügbarkeit muss es subkutan bzw. sublingual verabreicht werden, führt dann aber nach 5-15 Minuten (subkutan) und ab 15 Minuten (sublingual) zur selben Besserung der Parkinson-Symptome wie Levodopa. Die Wirkdauer liegt bei 45 -120 Minuten, daher können beide Formulierungen zusätzlich zur laufenden oralen Medikation appliziert werden. Praktische Erfahrungen mit sublingualem Apomorphin sind aktuell noch limitiert.

Evidenzgrundlage

4 Übersichten ([8], [31], [27], [32]); 4 randomisierte Studien mit Vergleich gegen Placebo ([33], [34], [35], [36]); 1 randomisierte Studie mit Vergleich gegen lösliches Levodopa [37]

Ergebnis

Eine nach PRISMA-Kriterien durchgeführte Metaanalyse identifizierte 28 Studien über subkutanes Apomorphin zur Akutbehandlung von Off-Zuständen [32]. Subkutane Apomorphin-Injektionen führten zu einer Verbesserung der Motorik um mehr als 50 % im Vergleich zum Ausgangswert und eine deutliche Verringerung der täglichen Off-Zeiten. Die mittlere Latenz bis zum Wirkungseintritt reichte von 6 bis 24 min (MW 13). Die mittlere Dauer der Wirkung reichte von 28 bis 96 min (MW 56). Die am häufigsten berichteten Nebenwirkungen waren: Übelkeit (14 Studien, Häufigkeit zwischen 11 % und 100 %), gefolgt von Gähnen (10 Studien, 4 % bis 100 %), Schläfrigkeit (10 Studien, 8 % bis 40 %), lokalen Reaktionen an der Injektionsstelle (7 Studien, 13 % bis 100 %), Hypotonie (3 Studien, 2 % bis 31 %) und visuellen Halluzinationen (3 Studien, 4 % bis 8 %). Eine leichte Hypereosinophilie wurde in 4 Studien berichtet (20%–30%). Es gab keine Berichte über hämolytische

Anämie oder akute Psychosen. Schwere unerwünschte Wirkungen wie vorübergehende pektanginöse Beschwerden (15 %), Synkope (2 %), Erbrechen (2 %), Hypotonie (2 %) und Durchfall (2 %) wurden in vier Studien berichtet. In fünf Studien brachen 3 bis 18 % der Pat. die Therapie aufgrund von Übelkeit/Erbrechen (5–11 %), Hypotonie (5–6 %) oder Schwindel (3–5,5 %) ab.

Die einzige randomisierte Studie, die Apomorphin-Injektionen mit oraler Medikation verglich, war eine kleine (n=12) Einzeldosisstudie. Die Wirkung mit Apomorphin trat signifikant rascher ein als nach löslichem Levodopa (nach 8,1 versus 26,8 Minuten); sie erreichte dieselbe Stärke wie lösliches Levodopa [37]. Randomisierte Langzeitstudien im Vergleich zur Standardmedikation wurden nicht identifiziert.

Die Injektionen erfolgen mittels eines voreinstellbaren Pens in das subkutane Fettgewebe von Bauch, Oberschenkeln oder Oberarmen. Die Einzeldosen liegen meist zwischen 2 und 6 mg (im Durchschnitt 3–5 mg). Die Austestung der individuellen Dosis mittels Injektionen in steigender Dosierung jeweils im Off kann ambulant unter sorgfältiger Kontrolle erfolgen. Es werden bis zu 5 Injektionen/Tag verabreicht. Benötigen Pat. mehr als 5 Injektionen, wird allgemein der Wechsel zu einer kontinuierlichen subkutanen Apomorphin-Infusion empfohlen.

Da Apomorphin Übelkeit und Erbrechen auslösen kann, wird als Prämedikation (ab ein bis zwei Tage vor der ersten Injektion) der periphere Dopaminrezeptor-Blocker Domperidon eingesetzt. Aufgrund einer möglichen QT-Verlängerung muss vor Beginn der Domperidon-Therapie ein EKG durchgeführt werden; die Höchstdosis von Domperidon wurde in einer EMA-Stellungnahme auf 3 x 10 mg täglich begrenzt, bei möglichst kurzer Therapiedauer.

Weitere unerwünschte Wirkungen einer Apomorphin-Injektionstherapie können vermehrte oder neu auftretende Dyskinesien, Schwindel, Schläfrigkeit, Halluzinationen, Gähnen sowie Hämatome und Knötchenbildung an der Einstichstelle sein. Bei der Zulassungsstudie für das sublinguale Apomorphin traten als unerwünschte Nebenwirkungen unter anderem Schwindel, Übelkeit, Somnolenz und oropharyngeale Nebenwirkungen auf.

Aus der Gesamtheit der zur Verfügung stehenden offenen Studien ergeben sich keine Hinweise auf ein höheres Risiko für das Auftreten von Halluzinationen und kognitiver Verschlechterung als mit oralen Dopaminagonisten.

Begründung der Empfehlung

Die Empfehlung beruht auf einer Vielzahl von offenen Studien und klinischen Beobachtungen, aber nur einer einzigen kontrollierten Studie an einer kleinen Anzahl von Pat.

Empfehlung	Neu<br>Stand (2023)
1. Intermittierende subkutane Apomorphin-Injektionen oder Applikationen eines sublingualen Apomorphin-Films können zusätzlich zur oralen Therapie eingesetzt werden, um die tägliche Off-Dauer bei Pat. mit motorischen Fluktuationen zu verkürzen.	

2. Die subkutane Apomorphin-Injektion sollte nur von darin erfahrenen Ärzten/Ärztinnen initiiert werden und bedarf eines entsprechenden Monitorings.
Konsensstärke: 100%, starker Konsens

Wichtige Forschungsfragen

Direkter Vergleich der Wirkung von Apomorphin und anderen On-demand-Therapien bei Parkinson-Pat. mit Fluktuationen.

Fragestellung 61: Wie effektiv ist eine Verkürzung der Einnahmeintervalle von Levodopa oder nicht retardierten Agonisten in der Behandlung von Parkinson-Pat. mit Fluktuationen?

Hintergrund

Motorische Fluktuationen (MF) werden von Parkinson-Pat. (PD) als besonders störendes Krankheitsmerkmal angesehen. L-Dopa wird zunächst meist routinemäßig in Form von 3–4 Dosen täglich verabreicht. Welchen Effekt hat eine Anpassung der Behandlung mit L-Dopa (und Dopaminagonisten), beispielsweise durch eine Erhöhung der Anzahl der Dosen pro Tag und ggf. eine Verringerung der Größe der Einzeldosen, bei motorischen Schwankungen?

Evidenzgrundlage: Diese Empfehlungen basieren auf 2 Übersichtsartikeln [9], [38] sowie 5 klinischen Studien [39], [40], [41], [42], [43].

Ergebnis

Wirksamkeit/Levodopa

Die tägliche Gesamtdosis von oralem Levodopa kann individuell angepasst werden und dabei zwei Parameter berücksichtigen: die Größe jeder Dosis und die Häufigkeit der Dosiseinnahme. Im Frühstadium der PK werden jedoch häufig stereotype, seltene Standarddosen angewendet (3–4 Dosen pro Tag). Bei fortgeschrittener PK ist es schwierig, die Dosierung festzulegen, wenn das therapeutische Fenster eng ist und bei den Pat. On-Off-Schwankungen in der motorischen Leistungsfähigkeit auftreten. Die Strategie zur Optimierung der Therapie besteht dann häufig darin, die Anzahl der Dosiseinnahmen zu erhöhen und ggf. die Einzeldosen zu verringern. Kleine und häufige Dosen verringern die Plasmavariabilität und scheinen motorische Schwankungen im Vergleich zu höheren und weniger häufigen Dosen zu verringern. Diese Strategie ahmt die physiologische tonische dopaminerge Stimulation (das CDS („Continuous Dopaminergic Stimulation“)-Konzept) besser nach, als wenn Levodopa nur dreimal täglich verabreicht wird. Eine häufige Dosierung erfordert niedrigere, individuellere Dosen. Mit dem Fortschreiten der Krankheit steigt jedoch die Schwelle der Levodopa-Konzentration für die therapeutische Wirkung und es ist wichtig, dass jede Dosis groß genug ist, um einen angemessenen klinischen Effekt hervorzurufen [38].

Die Levodopa -Fragmentierung ist eine häufig verwendete Strategie [39]. Trotzdem gibt es in der Literatur nur sehr begrenzte Informationen über die Auswirkungen der Fragmentierung von L-Dopa. Ein offener Vergleich zwischen COMT-Hemmung (3 Tagesdosen) und Dosisfragmentierung (von 3 auf

4 Tagesdosen) bei 176 Pat. mit einer Krankheitsdauer von etwa 5 Jahren zeigte, dass beide Methoden gegen Off-Fluktuationen ähnlich wirksam waren [40].

Es gibt keine randomisierte klinische Studie (RCT), die die Wirkung von Dosis-Einnahmeanpassungen von Levodopa/Carbidopa auf die Off-Zeit-Reduktion im Vergleich zu Placebo untersucht haben [9]. Auch wenn dies nicht speziell für die Bewertung der Wirkung oraler Levodopa-Anpassungen konzipiert ist, kann man auf die Auswirkung auf die Off-Zeit-Reduktion schließen, indem wir uns die Daten von RCT-Studien ansehen, die gerätegestützte Therapien/chirurgische Therapien mit oraler Behandlung vergleichen. Die meisten Studien zeigten einen gewissen Nutzen bez. motorischer Fluktuationen bei sorgfältiger Anpassung der Dosis/des Einnahmeschemas. Betrachtet man die doppelblinde „Double-Dummy“-Studie, die Standard-Levodopa/Carbidopa mit Levodopa/Carbidopa-Darmgel (LCIG) vergleicht, wurde bei oraler Behandlung eine Reduktion von −2,14 h/Tag über 12 Wochen beobachtet [41].

Die „Hyperfragmentierung“ von Levodopa – die Einnahme einer großen Anzahl kleiner Dosen – wird seit Langem bei Einzelpat. mit dem Ziel eingesetzt, stabilere Plasmakonzentrationen und damit eine stabilere Wirkung zu erreichen. Der theoretische Nutzen einer häufigen Levodopa-Gabe wird durch praktische Probleme für die Pat. beeinträchtigt. Insbesondere in frühen Stadien hat die häufige Gabe von Levodopa keinen unmittelbaren Nutzen und die Compliance stellt ein Problem dar. LC-5, eine dispergierbare Mikrotablettenformulierung zur oralen Einnahme, die 5/1,25 mg Levodopa/Carbidopa enthält, ist seit 2014 in Schweden und seit 2016 in der Europäischen Union zugelassen. LC-5 ermöglicht die Fraktionierung von Levodopa in kleinen Dosen, zu Beginn der motorischen Fluktuationen eingenommen. Die Dosis wird aus dem Spender abgegeben, vorzugsweise in ein Glas Wasser, wo sich die Mikrotabletten sofort verteilen. Der Spender ist mit einem Alarm ausgestattet, um die Einhaltung der Behandlung zu erleichtern, und einer optionalen tagebuchähnlichen Funktion zur Selbstberichterstattung, die die Individualisierung und Feinabstimmung der Dosisgröße und des Intervalls ermöglicht. Sechs Dosen LC-5 pro Tag ergeben eine stabilere Levodopa-Plasmakonzentration im Vergleich zu drei Dosen Levodopa/Carbidopa/Entacapon [42]. Theoretisch zeigt auch die Wirkung einer L-Dopa-Infusionstherapie auf motorische Fluktuationen (bei LCIG/LECIG wird eine Levodopa-Dosis pro Minute verabreicht) die Wirkung einer Levodopa-Hyperfragmentierung [41], [71]. Neben der symptomatischen Wirkung der Fragmentierung von L-Dopa bei motorischen Schwankungen gibt es auch Hinweise darauf, dass eine frühe Fragmentierung der L-Dopa-Dosen im frühen Krankheitsverlauf das Risiko für die Entwicklung von Dyskinesien längerfristig senken könnte [44]. Umgekehrt gibt es starke Hinweise darauf, dass hohe individuelle L-Dopa-Dosen das Risiko für die Entwicklung von Fluktuationen und Dyskinesien erhöhen [43].

Wirksamkeit/Dopaminagonisten

Die weniger lang wirksamen peroralen Dopaminagonisten werden normalerweise zwei- bis dreimal täglich und die länger wirksamen (Rotigotinpflaster, Pramipexol ret., Ropinirol ret.) einmal täglich verabreicht. Es liegen keine veröffentlichten Studien zur Wirkung einer weiteren Fragmentierung der Dopaminagonisten-Dosen vor.

Begründung der Empfehlung

Bezüglich des Effekts einer Anpassung der oralen Levodopa- und Dopaminagonisten-Therapie mit häufigerer Dosierung liegen keine randomisierten kontrollierten Studien vor. Allerdings deuten indirekte Erkenntnisse aus klinischen Studien und klinischer Erfahrung darauf hin, dass zumindest eine Anpassung der L-Dopa-Medikation, meist mit mehr und niedrigeren Dosen, im Hinblick auf eine Verkürzung der Off-Zeit wirksam sein kann.

**Empfehlung**	**Neu**<br>**Stand (2023)**
Eine Anpassung der Levodopa-Dosen (häufigere und kürzere Dosierungsintervalle und ggf. kleinere Dosen) kann erfolgen, um Fluktuationen bei Parkinson-Pat. mit Fluktuationen zu reduzieren.	
Konsensstärke: 100%, starker Konsens	

Fragestellung 62: Wie effektiv ist die zusätzliche Gabe von MAO-B-Hemmern im Vergleich mit Placebo in der Behandlung von Parkinson-Pat. mit Fluktuationen?

Hintergrund

MAO-B-Hemmer sind in der Frühphase der PK als Monotherapie und Kombinationstherapie etabliert (Kapitel 3.1, 3.2 und 3.3). Wird die Gabe im fortgeschrittenen Krankheitsverlauf weitergeführt oder neu begonnen, setzt dies eine Wirksamkeit in dieser Krankheitsphase voraus. Aktuell sind Selegilin, Rasagilin und Safinamid als MAO-B-Hemmer zugelassen.

Evidenzgrundlage

In dieser Literaturrecherche fanden sich

- N=7 randomisierte, kontrollierte Studien [45], [46], [47], [48], [49], [50], [51]
- N=2 Metaanalysen, [52], [53]

Ergebnis

Die zusätzliche Gabe von Selegilin konnte bereits 1988 in einer RCT [45] moderate positive Effekte auf die Motorik zeigen. Über insgesamt 6 Wochen erhielten 50 Pat. Selegilin 5 mg zweimal täglich und 46 erhielten Placebo, zusätzlich zur vorbestehenden Medikation mit Levodopa. Selegilin-behandelte Pat. verbesserten sich von 1.29 auf 1.41 Punkte, Placebo-behandelte verschlechterten sich von 1.28 auf 1.11 Punkte. Die hier verwendeten motorischen Skalen gelten jedoch als überholt und mit den heutigen schwer vergleichbar.

In einer RCT [46] wurden 94 bzw. 46 Pat. mit oral löslichen Tabletten (ODT) mit Selegilin (finale Dosis 2.5 mg/Tag) bzw. Placebo behandelt. Alle Pat. hatten Fluktuationen mit mindestens 3 Stunden Off/Tag. Nach 12 Wochen zeigte sich in der Selegilin-Gruppe eine signifikante Besserung der Off-Zeit/Tag um 2.2 Stunden vs. 0.6 Stunden bei Placebo. Dyskinesiefreie Zeit im On war ebenfalls um 12% signifikant länger als unter Placebo (3%).

Ondo et al. [47] untersuchten in einer RCT Selegilin ODT in der Kombination mit Levodopa bei fortgeschrittener PK („wearing -off“ und mindestens 3 Stunden Off/Tag). Zielparameter waren „wearing-off“ und On-Off-Fluktuationen. 98 Pat. Wurden mit Selegilin und 50 Pat. Mit Placebo über 12 Wochen behandelt. Selegilin zeigte einen Trend, war jedoch nicht signifikant überlegen (11,6% vs. 9,8% Reduktion der Off-Zeit).

In der PRESTO-RCT [48] (1++) wurden Wirkung, Verträglichkeit und Sicherheit von Rasagilin bei 472 Pat. Mit Levodopa-Therapie und motorischen Fluktuationen (mindestens 2,5 Stunden Off/Tag) über 26 Wochen untersucht. Die Off-Zeit nahm um 1,85 Stunden (29%) vs. 0,91 Stunden (15%) unter Placebo ab. Auch die sekundären Zielparameter (z.B. Clinical global impression (CGI), UPDRS-ADL)) besserten sich.

Auch die LARGO-RCT [49] (1++) untersuchte die Wirkung von Rasagilin bei Levodopa-behandelten Pat. mit motorischen Fluktuationen. In der dreiarmigen Studie wurden 687 Pat. behandelt, davon 231 mit Rasagilin, 227 mit Entacapon und 229 mit Placebo. Primäres Zielkriterium war die tägliche Off-Zeit. Sowohl Entacapon (-1,2 h), als auch Rasagilin (-1,18 h) reduzierten die Off-Zeit und erhöhten die On-Zeit ohne behindernde Dyskinesien. Auch die sekundären Zielparameter besserten sich signifikant. Unterschiede bezüglich Nebenwirkungen fanden sich nicht. Die Autorinnen/Autoren kamen zu dem Schluss, dass beide Substanzen wirksam sind und sich keine signifikanten Differenzen ergeben.

Die 016-Studie [50] (1++) untersuchte 669 Pat. mit Fluktuationen, die 1:1:1 zu Safinamid in zwei Dosierungen oder Placebo randomisiert wurden. Nach 24 Wochen zeigte sich eine signifikante Zunahme der On-Zeit ohne beeinträchtigende Dyskinesien für Safinamid von 1.36 bzw. 1.37 Stunden (100 mg bzw. 50 mg/Tag) vs. 0.97 Stunden für Placebo. Es gab keine signifikanten Unterschiede in den Nebenwirkungen. In der Fortsetzungsstudie 018 [51], welche die gleiche Pat.-Population für weitere 18 Monate untersuchte, bestätigten sich die Effekte auf die Zunahme der On-Zeit. In einer Subgruppe von Pat. mit vorbestehenden mindestens moderaten Dyskinesien zeigte sich auch eine Verbesserung von Dyskinesien.

Eine rezente Metaanalyse aller drei MAO-B-Hemmer mit 27 Studien und insgesamt 7578 Pat. [52] identifizierte für die Kombinationstherapie mit Levodopa Selegilin als die effektivste Substanz, gefolgt von Rasagilin und Safinamid. Keine signifikanten Unterschiede hinsichtlich schwerer Nebenwirkungen konnten identifiziert werden.

In einer bayesianischen Netzwerk-Metaanalyse mit 31 RCTs und 7142 Pat. [53] zeigte sich eine signifikante Verbesserung unter Kombinationstherapie von Levodopa mit Selegilin oder Rasagilin oder Safinamid.

Begründung der Empfehlung

Es existieren qualitativ hochwertige RCTs zu Rasagilin und Safinamid für fortgeschrittene Pat. mit Fluktuationen. Diese zeigen für beide MAO-B-Hemmer positive Effekte auf die Reduktion der Off-Zeit und die Zunahme der On-Zeit ohne beeinträchtigende Dyskinesien. Auch die Metaanalysen bestätigen diese Aussagen. Daher kann hier eine klare Empfehlung für ihren Einsatz ausgesprochen werden.

RCTs zu Selegilin-Tabletten sind zumeist älteren Datums und aufgrund ihrer Methodik oft nicht direkt vergleichbar. Selegilin-Tabletten führen zu einem starken First-pass-Effekt, der Produktion von Amphetamin- und Amphetamin-Metaboliten und Nebenwirkungen. Für oral lösliche Selegilin-Tabletten (ODT), die in dieser Darreichungsform und Dosierung in Deutschland nicht verfügbar sind, zeigen zwei große RCTs nicht ganz konsistente Effekte. In Metaanalysen ist Selegilin jedoch vergleichbar mit Rasagilin und Safinamid.

In einigen Studien zeigt sich eine Reduktion von Dyskinesien, was jedoch am ehesten auf eine gleichzeitige Reduktion der Levodopa-Dosis zurückzuführen ist.

Direkte Vergleichsstudien zu Selegilin, Rasagilin und Safinamid wurden nicht gefunden. Die Wirksamkeit der drei Substanzen erscheint in Metaanalysen sehr vergleichbar.

**Empfehlung**	**Neu**<br>**Stand (2023)**
Wenn unter einer Levodopa-Therapie die motorischen Fluktuationen nicht ausreichend kontrollierbar sind, können MAO-B-Hemmer zur Verminderung der Off-Zeiten zusätzlich angeboten werden.	
Konsensstärke: 100%, starker Konsens	

Wichtige Forschungsfragen:

Randomisierte kontrollierte Head-to-head-Studien zur Wirksamkeit und Sicherheit verschiedener MAO-B-Hemmer fehlen bislang. Diese würden einen direkten Vergleich ermöglichen.

Fragestellung 63: Welche Unterschiede hinsichtlich Sicherheit und Verträglichkeit gibt es bei den zugelassenen MAO-B-Hemmern für die Behandlung von Parkinson-Pat. mit Fluktuationen?

Hintergrund

Derzeit sind 3 verschiedene MAO-B-Hemmer auf dem Markt, Selegilin, Rasagilin und Safinamid. Es gibt noch keine großen „Kopf-an-Kopf"-Studien, die diese vergleichen, aber basierend auf indirekten Vergleichen und klinischer Erfahrung, können einige Schlussfolgerungen über Ähnlichkeiten und Unterschiede gezogen werden. Selegilin und Rasagilin haben als Monotherapie signifikante Wirkungen gezeigt und alle 3 MAO-B-Hemmer haben eine Wirkung als Zusatz zur L-Dopa-Behandlung gezeigt.

Evidenzgrundlage

Diese Empfehlungen basieren auf 4 Metaanalysen ([53] (31 RCTs), [52] (27 RCTs), [30] (79 RCTs), [54] (21 RCTs)) 2 Reviews ([55], [56]) und 1 Switchstudie ([57]).

Sicherheit, Rasagilin vs. Selegilin

Früh in der Entwicklung zeigten sich deutliche Vorteile für Rasagilin gegenüber Selegilin (Müller & Reichmann 2012). An dieser Stelle sei an den Abbau von Selegilin zu Amphetamin und Metamphetamin erinnert (Müller und Reichmann 2012). Die Umstellung von Selegilin auf Rasagilin konnte meist ohne neue Nebenwirkungen durchgeführt werden [57].

In eine Metaanalyse von Jost et al. wurden 6 RCTs mit Rasagilin und 15 RCTs mit Selegilin eingeschlossen [54]. Das Risiko für Nebenwirkungen wie Schwindel, Halluzinationen, Durchfall und Synkopen war unter Rasagilin geringer als unter Selegilin (jeweils $p < 0{,}15$). Die Abbruchrate aufgrund von Nebenwirkungen war mit Rasagilin auf dem Niveau der Placebo-Behandlung, während die Rate bei Selegilin signifikant höher war als bei Placebo.

Sicherheit, Rasagilin vs. Selegilin vs. Safinamid

In der Metaanalyse von Yan et al. [53] gab es hinsichtlich der Sicherheit keinen statistischen Unterschied in der Häufigkeit von Nebenwirkungen zwischen Selegilin, Safinamid und Placebo. In einzelnen Studien war Rasagilin mit einer höheren Häufigkeit von Nebenwirkungen verbunden als Placebo. Hinsichtlich der Nebenwirkungen, die zum Abbruch der Therapie führten, wurden keine Unterschiede zwischen aktiven Medikamenten und Placebo oder zwischen den aktiven Medikamenten festgestellt.

Binde et al. [52] führten eine Metaanalyse von 27 randomisierten kontrollierten Studien durch. MAO-B-Hemmer wurden entweder als Monotherapie oder in Kombination mit Levodopa oder Dopaminagonisten untersucht. Zwischen den MAO-B-Hemmern gab es keine wesentlichen Unterschiede in der Inzidenz von SAE. Man fand, dass die MAO-Hemmer gut toleriert wurden. Bei der Anwendung eines der 3 MAO-B-Hemmer konnte man kein erhöhtes Risiko für SAE im Vergleich zu Placebo oder gemeinsamer Placebo- und Levodopa- oder Dopaminagonistenbehandlung feststellen. Dies bestätigte sich auch in einer weiteren Publikation [30] – einer Metaanalyse von 79 randomisierten kontrollierten Studien. MAO-B-Hemmer wurden als Monotherapie oder in Kombination mit Levodopa gegeben. Zwischen den MAO-B-Hemmern gab es keine wesentlichen Unterschiede in der Inzidenz von SAE.

Begründung der Empfehlung

Die MAO-B-Hemmer werden im Allgemeinen gut vertragen. Die Häufigkeit von Nebenwirkungen übersteigt in vielen Studien nur unwesentlich die von Placebo. Es liegen keine direkten Vergleichsstudien vor und daher ist es schwierig, eindeutige Aussagen zum unterschiedlichen Nebenwirkungsrisiko der Präparate zu treffen. Einige Analysen deuten auf ein geringeres Risiko von Nebenwirkungen bei Rasagilin im Vergleich zu Selegilin hin, andere auf ein geringeres Risiko von Nebenwirkungen bei Safinamid im Vergleich zu anderen. In diesem Zusammenhang ist zu beachten, dass Safinamid nur zur Kombinationstherapie mit Levodopa indiziert ist, während Selegilin und Rasagilin auch als Monotherapie eingesetzt werden können.

Empfehlung	Neu<br>Stand (2023)
Die MAO-B-Hemmer sind im Allgemeinen gut verträglich. Aufgrund fehlender Vergleichsstudien kann keine Empfehlung für oder gegen eine der Substanzen gegeben werden.	
Konsensstärke: 100%, starker Konsens	

Fragestellung 64: Wie effektiv ist die zusätzliche Gabe von COMT-Hemmern im Vergleich mit Placebo in der Behandlung von Parkinson-Pat. mit Wirkfluktuationen?

Hintergrund

Das Grundprinzip der pharmakologischen Wirkung der Catechol-O-Methyltransferase (COMT)-Hemmung ist die Kombination mit Levodopa, da COMT-Hemmer den Abbau von Levodopa peripher und zentral (nur für Tolcapon bewiesen) von Dopamin verlangsamen. So kann eine Kombination von oral gegebenem Levodopa mit einem Dopadecarboxylase-Hemmer (DDCH) und einem COMT-Hemmer das Ausmaß der Plasmaspiegelschwankungen von Levodopa im Vergleich zur Kombination aus Levodopa und einem DDCH verringern. Dies führt zu einem kontinuierlicheren Levodopa-Transport über die Blut-Hirn-Schranke und bei auch zentral wirksamen COMT-Hemmern, wie z.B. Tolcapon, auch zu gleichmäßigeren Dopaminspiegeln, wenn Levodopa und der COMT-Hemmer im ausreichenden zeitlichen Abstand oral eingenommen werden.

Klinisch kommt es dadurch zu einer geringeren Häufigkeit und Ausprägung von motorischen Komplikationen, insbesondere von Off-Phasen. Auf der anderen Seite können Dyskinesien auftreten, wenn die Zeitintervalle zwischen den Einnahmezeitpunkten unter pharmakokinetischen Gesichtspunkten nicht entsprechend weit auseinanderliegen. Dies war in der STRIDE-PD-Studie mit dem dabei durchgeführten fixen Einnahmeschema sowie auch in anderen Studien, die die Effizienz von COMT-Hemmern mit Placebo verglichen, nicht entsprechend berücksichtigt worden. Daher konnte zwar eine Verringerung von Off-Phänomenen verifiziert werden, andererseits aber eine Zunahme von Dyskinesien beobachtet werden.

Evidenzgrundlage

Diese Empfehlungen basieren auf 3 systematischen Übersichtsartikeln zur COMT-Hemmer-Therapie bei PK mit motorischen Fluktuationen ([58] (17 Studien), [59] (14 Studien), [60] (13 Studien)) sowie 1 Review zur Opicapon-Therapie [61].

Ergebnis

Wirksamkeit

Entacapon, Tolcapon und Opicapon sind Placebo überlegen, wenn nur berücksichtigt wird, ob COMT-Hemmer das Auftreten von Off-Phasen reduzieren. Insgesamt zeigt sich in bestehenden Studien die Tendenz, dass die Reduktion der Off-Zeit bei Tolcapon am ausgeprägtesten ist, gefolgt von Opicapon und dann Entacapon.

Die motorische Symptomatik im On-Zustand, gemessen mit UPDRS III, wird durch die Zugabe von COMT-Hemmern nicht beeinflusst.

Additive COMT-Hemmer können Dyskinesien verursachen. Dies hängt vom Studiendesign ab, bzw. davon, in welchem zeitlichem Abstand und in welcher Dosis Levodopa appliziert wurde. Prädisponierende Faktoren für die Entwicklung von Dyskinesien nach additiver Gabe von COMT-Hemmern sind hohe Levodopa-Dosis, junges Alter und, damit verbunden, oft geringeres Körpergewicht. Geringes Körpergewicht prädisponiert zu der mit Auftreten von Dyskinesien eng assoziierten Erhöhung der maximalen Levodopa-Konzentration nach oraler Levodopa-Einnahme. Bei der Einführung von COMT-Hemmern ist deswegen zu berücksichtigen, dass die maximale Levodopa-Konzentration ansteigt, was zu vermehrten Levodopa-bedingten Nebenwirkungen führen kann. Es kann daher erforderlich sein, die tägliche Levodopa-Gesamtdosis nach unten anzupassen. Dies kann erreicht werden, indem die Höhe der einzelnen Levodopa-Dosen verringert und/oder das Dosierungsintervall verlängert wird.

In Bezug auf die Sicherheit scheint Tolcapon das größte Risiko von Nebenwirkungen zu haben, während Opicapon das geringste Risiko hat. Tolcapon hat ein nicht unerhebliches Hepatotoxizitätsrisiko. In den ursprünglichen Studien wurden relativ wenige Anomalien der Transaminasen festgestellt und diese schienen reversibel nach Absetzen des Medikaments zu sein. Erst die Post-Marketing-Überwachung hat drei Fälle von tödlicher Lebertoxizität identifiziert, die zum Entzug der Produktzulassung von Tolcapon in einigen Märkten und Dosisbeschränkungen in anderen führte (s. Kapitel 3.1., Fragestellung 36 zu Sicherheit und Verträglichkeit von COMT-Hemmern).

Andere häufige Nebenwirkungen von COMT-Hemmern sind Durchfall (etwas häufiger bei Tolcapon als bei Entacapon und am seltensten bei Opicapon), aber zusätzlich zu Übelkeit, Müdigkeit, Halluzinationen und orthostatischer Hypotonie tritt auch Verstopfung auf. Durchfall tritt bei etwa 10 % der Pat. auf, die Entacapon einnehmen, und 1,3 % haben ausgeprägten Durchfall (www.drugs.com). Bei der Anwendung von Tolcapon wurde von bis zu 26 % der Anwendenden über Durchfall berichtet, was bei etwa 5–10 % der Pat. einen Abbruch der Behandlung erforderte.

Entacapon 200 mg kann zusammen mit jeder Levodopa-Dosis entweder separat oder als feste Kombinationspräparate Levodopa/Carbidopa/Entacapon eingenommen werden. Die Kombinationspräparate bieten einen praktischen Vorteil durch weniger Tabletten und werden entsprechend dem Levodopa-Gehalt dosiert. Die empfohlene Höchstdosis beträgt 2000 mg/Tag. Entacapon färbt Urin und Schweiß gelb.

Tolcapon wird dreimal täglich mit 100 mg dosiert. Es hat eine nicht unerhebliche Hepatotoxizität und ist daher ein Mittel der zweiten Wahl. Voraussetzung für die Anwendung von Tolcapon ist, dass Pat. auf Entacapon und Opicapon nicht angesprochen haben oder es nicht vertragen. Die Transaminasen müssen vor Beginn und in den ersten 12 Monaten alle 2 Wochen, dann 6 Monate lang alle 4 Wochen und dann alle 8 Wochen kontrolliert werden.

Opicapon wird mit 50 mg einmal täglich dosiert und kann am einfachsten nachts zusammen mit oder vor der letzten Levodopa-Dosis eingenommen werden. Die Produktmonographie rät davon ab,

Opicapon zusammen mit Levodopa einzunehmen, aber dafür gibt es keine gute Begründung (ggf. mögliche kompetitive Hemmung der Resorption).

Begründung der Empfehlung

Diese Empfehlungen basieren auf 3 systematischen Übersichtsartikeln zur COMT-Hemmer-Therapie bei der Parkinson-Erkrankung mit motorischen Fluktuationen sowie 1 Review zur Opicapon-Therapie. COMT-Hemmer reduzieren die Zeit im Off pro Tag bei Pat. mit Levodopa-Therapie und motorischen Fluktuationen.

Empfehlung	Modifiziert<br>Stand (2023)
1. Wenn unter einer Therapie mit Levodopa die motorischen Fluktuationen nicht ausreichend kontrollierbar sind, kann zusätzlich ein COMT-Hemmer angeboten werden.<br>2. Aufgrund der Sicherheitsaspekte sind Opicapon und Entacapon als Mittel der ersten Wahl, Tolcapone als Mittel der zweiten Wahl unter engmaschigem Sicherheitsmonitoring (klinisch und laborchemisch) einzusetzen.	
Konsensstärke: 100%, starker Konsens	

Fragestellung 65: Welche Unterschiede hinsichtlich Sicherheit und Verträglichkeit gibt es bei den zugelassenen COMT-Hemmern für die Behandlung von Parkinson-Pat. mit Fluktuationen?

Hintergrund

Zurzeit stehen drei COMT-Hemmer zur Behandlung von Fluktuationen zur Verfügung: Entacapon, Tolcapon und Opicapon. Im Gegensatz zu Entacapon und Opicapon ist Tolcapon lipophiler, es passiert die Blut-Hirn-Schranke und wirkt sowohl peripher als auch zentral. Aufgrund der Halbwertszeiten muss Entacapon zu jeder Levodopa-Gabe (bis zu zehnmal täglich), Tolcapon dreimal täglich und Opicapon einmal täglich eingenommen werden. Ein wichtiges Kriterium bei der Auswahl der Substanz jenseits der Wirksamkeit ist die Sicherheit und Verträglichkeit.

Evidenzgrundlage

Für diese Kapitel: In dieser Literaturrecherche fanden sich

- N=1 randomisierte, kontrollierte Studie [62]
- N=3 Reviews und N=1 bayesianische Netzwerkanalyse [63], [64], [65], [58]).

Ergebnis

In der RCT-Bi-Park-1-Studie [62] wurde bei insgesamt 600 Pat. mit Fluktuationen die Wirksamkeit, Sicherheit und Verträglichkeit von Opicapon mit Entacapon und Placebo verglichen. Diese Studie ist eine der wenigen, welche zwei COMT-Hemmer direkt miteinander vergleicht. Opicapon reduzierte signifikant die Zeit im Off im Vergleich zu Placebo und war Entacapon nicht unterlegen. Unerwünschte Nebenwirkungen traten in 50% (Placebo), 57% (Entacapon 200 mg) und 54% (Opicapon 50 mg) auf, ohne signifikanten Unterschied. Ein medikationsbedingter Studienabbruch

war selten (jeweils in 7%, 7% und 4%), ebenso schwere unerwünschte Nebenwirkungen (SAE, jeweils in 5%, 7% und 3%). Zum Therapieabbruch führten Diarrhoen (Entacapon) und Dyskinesien/Halluzinationen (Opicapon).

Ein systematisches Review [63] fasst 32 Studien mit insgesamt 4780 Pat. mit Tolcapon-Behandlung zusammen. Eine leichte Erhöhung von Leberfunktionsparametern fand sich in bis zu 27%. Drei Pat. mit tödlichem Leberversagen und ein Pat. mit schwerer reversibler Hepatopathie wurden berichtet, was zwischen 1998 und 2004 zu einer Pausierung der Marktzulassung von Tolcapon durch die EMA führte. Hiernach erfolgte die Wiederzulassung mit Auflagen (s.u.). Weitere drei Fälle schwerer Hepatotoxizität wurden seitdem in Studien berichtet. Allerdings fanden sich weitere 61 Fälle schwerer Leberfunktionsstörung in den Sicherheitsdatenbanken der EMA und der FDA.

In einem ausführlichen Review [64] werden die bis dahin veröffentlichten Daten zu Opicapon zusammengefasst. Hier gingen insgesamt Daten aus Studien an 1.651 Teilnehmenden ein. Gepoolte Sicherheitsdaten aus den Bi-Park-1- und Bi-Park-2-Studien (766 Parkinson-Pat.) zeigten Dyskinesien (20,4 %), Obstipation (6,4 %) und Schlafstörungen (3,4 %) als häufigste medikamenteninduzierte Nebenwirkung in den Opicapon-50-mg-Gruppen. Insbesondere fanden sich keine Hepatotoxizität, Diarrhoe oder Urinverfärbung in den Opicapon-Gruppen. Ein spezielles Sicherheitsmonitoring ist nicht erforderlich.

Ein systematisches Review [65], welches Daten zu Entacapon, Tolcapon und Opicapon aus 12 klinischen Studien und insgesamt 3.701 Pat. zusammengetragen hat, stellt in Bezug auf die Sicherheit fest, dass unter Einhaltung der Sicherheitsauflagen alle Leberfunktionsstörungen unter Tolcapon reversibel waren. Dyskinesien, Diarrhoen und Übelkeit waren die häufigsten unerwünschten Wirkungen bei Entacapon, bei Opicapon dominierten die Dyskinesien. Sowohl Entacapon als auch Tolcapon führen zu Urinverfärbung.

In einer bayesianischen Netzwerkanalyse [58] wurden insgesamt Daten aus 17 RCTs mit insgesamt 4124 Pat. analysiert. Die Autorinnen/Autoren fanden im Ranking der drei Substanzen das beste Sicherheitsprofil für Opicapon, gefolgt von Entacapon und Tolcapon.

Begründung der Empfehlung

Nur in einer RCT werden zwei COMT-Hemmer (Entacapon und Opicapon) direkt verglichen, jedoch erlauben die systematischen Reviews und Netzwerkanalysen einen guten indirekten Vergleich, da insgesamt umfangreiche Daten zur Sicherheit und Verträglichkeit von COMT-Hemmern vorliegen. Weniger Daten existieren zu Opicapon, da die Zulassung später als Entacapon oder Tolcapon erfolgte. Für Tolcapon stützen sich die Aussagen hauptsächlich auf Daten vor der Zulassungsbeschränkung durch die EMA sowie auf ein durch die Auflagen beschränktes Pat.-Kollektiv.

Insgesamt zeigt Opicapon das beste Sicherheitsprofil, welches v.a. durch Dyskinesien dominiert wird. Das Auftreten von Dyskinesien bestätigt die symptomatische dopaminerge Wirkung der Substanz. Daher kann bei Auftreten von Dyskinesien eine Reduktion der Levodopa-Dosis hilfreich sein.

Das Sicherheitsprofil von Entacapon wird durch Diarrhoen und Übelkeit gekennzeichnet. Da die Substanz zu jeder Levodopa-Dosis gegeben werden muss, ist in diesen Fällen ein Absetzen oft unvermeidlich.

Tolcapon zeigt aufgrund der zwar seltenen, aber schwerwiegenden Fälle der Hepatotoxizität das schlechteste Sicherheitsprofil und wurde von der EMA daher mit folgenden Auflagen belegt: a) Tolcapon soll nicht als erste Wahl bei der Behandlung von Fluktuationen genutzt werden, b) die Therapie mit Tolcapon soll bei mangelnder Wirksamkeit in den ersten drei Wochen abgesetzt werden, c) vor Therapie sollen Leberfunktionstests durchgeführt werden und die Therapie soll nicht begonnen werden, wenn diese auffällig sind, d) Leberwerte sollen unter Therapie alle zwei Wochen für 1 Jahr, alle 4 Wochen für weitere 6 Monate und danach alle 8 Wochen kontrolliert werden, e) bei einer Dosiserhöhung auf dreimal täglich 200 mg Tolcapon sollen die gleichen Maßnahmen wie in c) und d) erfolgen, f) bei Übersteigen des oberen Grenzwerts für die AST und ALT oder Symptomen der Leberfunktionsstörung muss die Therapie beendet werden. (https://www.ema.europa.eu/en/documents/product-information/tasmarepar-product-information_en.pdf)

Empfehlung	Neu<br>Stand (2023)
1. Opicapon und Entacapon sind als COMT-Hemmer in der Wirkung weitgehend gleichwertig und können zur Behandlung von Fluktuationen bei der PK eingesetzt werden.<br>2. Tolcapon sollte wegen der Hepatotoxizität nur als Mittel der zweiten Wahl und unter engmaschigem Sicherheitsmonitoring (klinisch und laborchemisch) verwendet werden.	
Konsensstärke: 100%, starker Konsens	

Fragestellung 66: Wie effektiv ist die zusätzliche Gabe von COMT-Hemmern im Vergleich mit MAO-B-Hemmern in der Behandlung von Parkinson-Pat. mit Fluktuationen?

Hintergrund

Im fortgeschrittenen Stadium der PK wird meist eine Kombinationstherapie eingesetzt. Dies betrifft die Kombination von Levodopa mit Dopaminagonisten, COMT-Hemmern, MAO-B-Hemmern und weiteren Substanzen. Hier stellt sich die Frage, ob die zusätzliche Gabe von COMT-Hemmern oder von MAO-B-Hemmern zum Levodopa effektiver ist.

Evidenzgrundlage

Für diese Kapitel: In dieser Literaturrecherche fanden sich

- N=2 randomisierte, kontrollierte Studien [66], [49]
- N=1 Metaanalyse [60].

Ergebnis

In einer RCT mit 16 Parkinson-Pat. [66] mit Fluktuationen wurde Levodopa entweder mit Selegilin (10 mg täglich) oder mit Entacapon (200 mg zu jeder Levodopa-Dosis) oder mit der Kombination beider

Substanzen ergänzt. Alle Pat. durchliefen randomisiert jeden der o.g. Behandlungsarme für jeweils 2 Wochen. Hier konnte eine Verbesserung der motorischen Funktionsparameter in jeder der drei Behandlungsgruppen beobachtet werden, ohne dass es eine signifikante Überlegenheit einer Gruppe gab. Dyskinesien waren ebenfalls in allen drei Behandlungsgruppen verstärkt, jedoch nur in der Kombinationsgruppe signifikant häufiger. Es fanden sich keine signifikanten Unterschiede im Nebenwirkungsprofil.

Die LARGO-RCT [49] untersuchte die Wirkung von Rasagilin bei Levodopa-behandelten Pat. mit motorischen Fluktuationen. In der dreiarmigen Studie wurden 687 Pat. behandelt, davon 231 mit Rasagilin, 227 mit Entacapon und 229 mit Placebo. Primäres Zielkriterium war die tägliche Off-Zeit. Sowohl Entacapon (-1,2 h), als auch Rasagilin (-1,18 h) reduzierten die Off-Zeit und erhöhten die On-Zeit ohne behindernde Dyskinesien. Auch die sekundären Zielparameter besserten sich signifikant. Unterschiede bezüglich Nebenwirkungen fanden sich nicht. Die Autoren/Autorinnen kamen zu dem Schluss, dass beide Substanzen wirksam sind und sich keine signifikanten Differenzen ergeben.

Eine Metaanalyse [60] fasste insgesamt 13 RCTs mit n=3775 Pat. mit fortgeschrittener PK zusammen, die unter motorischen Fluktuationen litten und zusätzlich zu einer bestehenden Therapie mit Levodopa entweder mit einem COMT-Hemmer (Tolcapon oder Entacapon) oder mit einem MAO-B-Hemmer (Selegilin oder Rasagilin) behandelt wurden. Für beide Gruppen fand sich eine Verbesserung sowohl im UPDRS-Gesamtscore als auch im UPDRS-ADL oder im UPDRS-Motor-Score allein. In beiden Gruppen fanden sich mehr Dyskinesien.

Einschränkend ist zu bemerken, dass in Lyytinen 2000 nur Selegilin als MAO-B-Hemmer und nur Entacapon als COMT-Hemmer untersucht wurde. Beide Substanzen werden heute in Deutschland aufgrund besser wirksamer und weniger nebenwirkungsträchtiger Alternativen seltener eingesetzt als zum Zeitpunkt der Publikation der Studie. In Rascol 2005 kommen nur Rasagilin oder Entacapon zum Einsatz. Eine Verallgemeinerung auf andere MAO-B-Hemmer (z.B. Safinamid) und andere COMT-Hemmer (z.B. Tolcapon oder Opicapon) ist nicht ohne Weiteres möglich. Zudem bemerken Talati et al. 2009, dass die gemessenen Effektstärken im UPDRS-ADL und im UPDRS-Motor für beide Substanzklassen nicht die Schwelle der klinischen Relevanz erreichen, wenngleich sie statistisch signifikant sind.

Begründung der Empfehlung

Da die Datengrundlage lediglich zwei RCTs berücksichtigt, die direkt die additive Gabe eines COMT-Hemmers mit der Gabe eines MAO-B-Hemmers verglichen, kann eine klare Empfehlung für die gesamte Substanzklasse nicht abgegeben werden. Auch fehlen Daten zu Safinamid und Opicapon, weswegen die Aussagen nur eingeschränkt verallgemeinert werden können. Es wird daher auf die entsprechenden Empfehlungen zum Einsatz von COMT-Hemmern bzw. MAO-B-Hemmern verwiesen.

**Empfehlung**	**Neu**<br>**Stand (2023)**
Eine klare Empfehlung für den bevorzugten Einsatz einer der beiden Substanzklassen kann nicht abgegeben werden	
Konsensstärke: 100%, starker Konsens	

Wichtige Forschungsfragen:

Neuere Metaanalysen sollten auch Safinamid und Opicapon berücksichtigen.

Fragestellung 67: Wie effektiv ist die zusätzliche Gabe des A2A-Rezeptor-Antagonisten Istradefyllin im Vergleich mit Placebo in der Behandlung von Parkinson-Pat. mit Fluktuationen?

Hintergrund

Die Blockade des Adenosin-A2A-Rezeptors auf den „medium spiny neurons" bewirkt eine Reduktion im überaktiven striatopallidalen Output. Istradefyllin, ein Koffein-Analogon und Adenosin-A2A-Rezeptor, ist in den USA und in Japan zur Behandlung der motorischen Fluktuationen zugelassen. In Jahre 2019 hat der Ausschuss für Humanarzneimittel CHMP der europäischen Arzneimittelbehörde EMA aufgrund von inkonsistenten Studienergebnissen von der Zulassungsempfehlung des Parkinson-Medikaments Istradefyllin abgesehen.

Evidenzgrundlage

Diese Empfehlungen basieren auf 1 gepoolten Analyse von 8 randomisierten/kontrollierten Studien zu Istradefyllin [67], 1 Metaanalyse über 9 Studien [68] und 1 narrativen, auf 8 solcher Studien basierenden Review [69].

Ergebnis

In der Metaanalyse [68] von 9 Studien verringerte Istradefyllin im Vergleich zu Placebo die Off-Zeit (-0,58 h [- 1,01 bis -0,16]; p = 0,007) und verringerte den On-Zustand mit Dyskinesie (0,69 [0,02 bis 1,37]; p = 0,043). Hinsichtlich der Verträglichkeit, UPDRS III, und unerwünschten Wirkungen gab es keinen signifikanten Unterschied zwischen Istradefyllin und Placebo. Eine gepoolte Analyse [67] bewertete Wirksamkeit und Sicherheit von Istradefyllin in einer Dosis von 20 und 40 mg/Tag bei Parkinson-Pat., die Levodopa mit Decarboxylase-Inhibitor erhielten und motorische Fluktuationen aufwiesen, in 8 randomisierten, placebokontrollierten, doppelblinden Phase-2b-/3-Studien. Bei 2.719 Pat. (Placebo, n = 992; 20 mg/Tag, n = 848; 40 mg/Tag, n = 879) wurden die Off-Stunden/Tag nach 12 Wochen gegenüber Placebo im Vergleich zum Ausgangwert bei Dosierungen von 20 mg/Tag (Least-Squares-Mean-Difference [LSMD] um [95% KI]) -0,38 h [-0,61, -0,15]) und bei 40 mg/Tag um -0,45 h [-0,68, -0,22]; p < 0,0001) signifikant reduziert; die On-Zeit ohne störende Dyskinesien (On-WoTD) nahm signifikant zu. Ähnliche Ergebnisse ergaben sich für die vier gesondert betrachteten Studien ohne signifikante Effekte auf den Endpunkt. Istradefyllin wurde im Allgemeinen gut vertragen; die durchschnittliche Studienabschlussrate bei mit Istradefyllin behandelten Pat. betrug in allen Studien 89,2%. Dyskinesien waren das häufigste unerwünschte Ereignis (Placebo, 9,6%; 20 mg/Tag, 16,1%; 40 mg/Tag, 17,7%). Im Review [69] wird die moderate, aber über mehrere Studien uneinheitliche Wirksamkeit von Istradefyllin auf Off-Zeiten und verminderte On-Zeiten mit Dyskinesien herausgestellt, mit einer in einzelnen Studien vergleichbaren Größenordnung wie die aus anderen Studien bekannten Effekte von Safinamid (100 mg/d) und Opicapon (50 mg/d). Höhere Dosen von Istradefyllin (40 mg) und eine niedrigere Levodopa-Dosis (Tagesdosis 400 mg) wurden als Prädiktoren für einen positiven Effekt auf die Endpunkte in den betrachteten Studien herausgehoben.

Begründung der Empfehlung

Istradefyllin reduzierte die Off-Zeit bei Pat., die mit Levodopa/Dopa-decarboxylase-Hemmer behandelt wurden und motorische Fluktuationen aufwiesen, bei guter Verträglichkeit. In 4 von 8 kontrollierten Studien wurde eine signifikante Reduktion von Off-Zeiten gefunden. Wegen der fehlenden Zulassung in Europa wird keine Empfehlung zum Einsatz von Istradefyllin bei Parkinson-Pat. gegeben.

Empfehlung	Neu<br>Stand (2023)
Wegen der fehlenden Zulassung in Europa wird keine Empfehlung zum Einsatz von Istradefyllin gegeben.	
Konsensstärke: 100%, starker Konsens	

Wichtige Forschungsfragen:

Ist die Wirkung von Istradefyllin von der Dosis der Levodopa-Therapie abhängig?

Fragestellung 68: Kann eine Priorisierung der verschiedenen Optionen zur Behandlung der Fluktuationen bei der PK empfohlen werden?

Hintergrund

Zur Behandlung von Fluktuationen stehen verschiedene medikamentöse Therapieoptionen zur Verfügung:

- Fraktionierung der Levodopa-Gaben und ggf. Dosisänderung,
- zusätzliche Gaben von Levodopa-Präparaten mit modifizierter Galenik (lösliches, inhalatives oder retardiertes Levodopa)
- zusätzliche Gaben von Dopaminagonisten
- zusätzliche Gaben von MAO-B-Hemmern oder
- zusätzliche Gaben von COMT-Hemmern.

Für jede dieser individuellen Optionen bestehen evidenzbasierte Empfehlungen. In der Praxis ist es jedoch erforderlich, diese Optionen für jede/n einzelne/n Pat. zu priorisieren.

Evidenzgrundlage

Es existieren keine randomisierten, kontrollierten Studien, die eine zeitliche Priorisierung der verschiedenen zur Verfügung stehenden Optionen zur Behandlung von Fluktuationen bei Parkinson-Kranken evaluieren. Zahlreiche Reviews thematisieren die o.g. Optionen und geben eine Expertenmeinung hinsichtlich ihres klinischen Einsatzes [9], [70]. Die Evidenz für die einzelnen o.g. Optionen wird in den jeweiligen Kapiteln des Kapitels 3.4 besprochen.

Ergebnis

Da es keine kontrollierten Studien zur Priorisierung der verschiedenen Therapieoptionen bei Pat. mit Parkinson-Erkrankung und Fluktuationen gibt und jede der o.g. Optionen in kontrollierten Studien gegen eine pulsatile Therapie mit Levodopa untersucht wurde, kann der Beginn der Therapie von Fluktuationen prinzipiell mit jeder der o.g. Optionen erfolgen. Bei der Wahl der Therapieoption sollen das Wirkspektrum, das Nebenwirkungsprofil sowie der Pat.-Wunsch berücksichtigt werden. Oft wird zunächst eine Fraktionierung der Levodopa-Dosis mit einer Verkürzung der Einnahmeintervalle (z.B. von 3-mal täglich auf 4- oder 5-mal täglich) erfolgen, da hier keine zusätzliche Medikamentengruppe eingeführt werden muss. Bei morgendlicher Akinesie ist die Hinzunahme von löslichem Levodopa morgens und/oder retardiertem Levodopa zur Nacht eine häufig gewählte Option. Kommt es hierunter weiterhin zu Fluktuationen, kann eine weitere o.g. Option hinzugenommen werden. Wird eine der o.g. Optionen aufgrund von Nebenwirkungen nicht vertragen, kann die Therapie auf eine andere Substanz der gleichen Substanzklasse oder auf eine Substanz einer anderen Substanzklasse geändert werden. Bei plötzlich einsetzenden Off-Phasen kann eine Bedarfstherapie mit subkutanem oder sublingualem Apomorphin, inhalativem oder löslichem Levodopa erfolgen.

Begründung der Empfehlung

Da es keine kontrollierten Studien gibt, die die Frage der Priorisierung beantworten, greift die Empfehlung auf die Evidenz zu den einzelnen Optionen zurück und verknüpft diese mit der Expertenmeinung.

**Empfehlung**	**Neu**<br>**Stand (2023)**
▪ Eine Priorisierung der einzelnen therapeutischen Optionen für Parkinson-Pat. mit Fluktuationen kann auf Grundlage von Studien nicht erfolgen.<br>▪ Die individuelle Reihenfolge der Therapieoptionen soll das Wirkspektrum, das Nebenwirkungsprofil und den Pat.-Wunsch berücksichtigen.	
Konsensstärke: 100%, starker Konsens	

Optional: Weitere RCTs wären erforderlich, um zu klären, welche Therapieoption bei Parkinson-Pat. mit Fluktuationen zu priorisieren ist.

Referenzen

1. Widnell K. Pathophysiology of motor fluctuations in Parkinson's disease. Mov Disord. 2005;20 Suppl 11:S17-22
2. Ahlskog JE, Muenter MD. Frequency of levodopa-related dyskinesias and motor fluctuations as estimated from the cumulative literature. Mov Disord. 2001;16(3):448-58
3. Antonini A, Martinez-Martin P, Chaudhuri RK, Merello M, Hauser R, Katzenschlager R, et al. Wearing-off scales in Parkinson's disease: critique and recommendations. Mov Disord. 2011;26(12):2169-75

4. van der Velden RMJ, Broen MPG, Kuijf ML, Leentjens AFG. Frequency of mood and anxiety fluctuations in Parkinson's disease patients with motor fluctuations: A systematic review. Mov Disord. 2018;33(10):1521-7
5. Livingston C, Monroe-Duprey L. A Review of Levodopa Formulations for the Treatment of Parkinson's Disease Available in the United States. J Pharm Pract. 2023:8971900221151194
6. Koller WC, Pahwa R. Treating motor fluctuations with controlled-release levodopa preparations. Neurology. 1994;44(7 Suppl 6):S23-8
7. Müller T, Möhr JD. Efficacy of carbidopa-levodopa extended-release capsules (IPX066) in the treatment of Parkinson Disease. Expert Opin Pharmacother. 2018;19(18):2063-71
8. Stowe R, Ives N, Clarke CE, Deane K, Wheatley K, Gray R, et al. Evaluation of the efficacy and safety of adjuvant treatment to levodopa therapy in Parkinson s disease patients with motor complications. Cochrane Database Syst Rev. 2010(7):Cd007166
9. Fabbri M, Barbosa R, Rascol O. Off-time Treatment Options for Parkinson's Disease. Neurol Ther. 2023;12(2):391-424
10. Stocchi F, Zappia M, Dall'Armi V, Kulisevsky J, Lamberti P, Obeso JA. Melevodopa/carbidopa effervescent formulation in the treatment of motor fluctuations in advanced Parkinson's disease. Mov Disord. 2010;25(12):1881-7
11. Warren Olanow C, Torti M, Kieburtz K, Leinonen M, Vacca L, Grassini P, et al. Continuous versus intermittent oral administration of levodopa in Parkinson's disease patients with motor fluctuations: A pharmacokinetics, safety, and efficacy study. Mov Disord. 2019;34(3):425-9
12. Safirstein BE, Ellenbogen A, Zhao P, Henney HR, 3rd, Kegler-Ebo DM, Oh C. Pharmacokinetics of Inhaled Levodopa Administered With Oral Carbidopa in the Fed State in Patients With Parkinson's Disease. Clin Ther. 2020;42(6):1034-46
13. Glenardi G, Handayani T, Barus J, Mangkuliguna G. Inhaled Levodopa (CVT-301) for the Treatment of Parkinson Disease: A Systematic Review and Meta-analysis of Randomized Controlled Trials. Neurol Clin Pract. 2022;12(2):139-48
14. Jost WH, Kulisevsky J, LeWitt PA. Inhaled levodopa for threatening impending OFF episodes in managing Parkinson's disease. J Neural Transm (Vienna). 2023;130(6):821-6
15. Martinez-Nunez AE, LeWitt PA. Drugs to the Rescue: Comparison of On-Demand Therapies for OFF Symptoms in Parkinson's Disease. J Parkinsons Dis. 2023;13(4):441-51
16. LeWitt PA, Hauser RA, Pahwa R, Isaacson SH, Fernandez HH, Lew M, et al. Safety and efficacy of CVT-301 (levodopa inhalation powder) on motor function during off periods in patients with Parkinson's disease: a randomised, double-blind, placebo-controlled phase 3 trial. Lancet Neurol. 2019;18(2):145-54
17. Hauser RA, Isaacson SH, Ellenbogen A, Safirstein BE, Truong DD, Komjathy SF, et al. Orally inhaled levodopa (CVT-301) for early morning OFF periods in Parkinson's disease. Parkinsonism Relat Disord. 2019;64:175-80
18. Lipp MM, Batycky R, Moore J, Leinonen M, Freed MI. Preclinical and clinical assessment of inhaled levodopa for OFF episodes in Parkinson's disease. Sci Transl Med. 2016;8(360):360ra136
19. LeWitt PA, Hauser RA, Grosset DG, Stocchi F, Saint-Hilaire MH, Ellenbogen A, et al. A randomized trial of inhaled levodopa (CVT-301) for motor fluctuations in Parkinson's disease. Mov Disord. 2016;31(9):1356-65
20. Grosset DG, Dhall R, Gurevich T, Kassubek J, Poewe WH, Rascol O, et al. Inhaled levodopa in Parkinson's disease patients with OFF periods: A randomized 12-month pulmonary safety study. Parkinsonism Relat Disord. 2020;71:4-10
21. Farbman ES, Waters CH, LeWitt PA, Rudzińska M, Klingler M, Lee A, et al. A 12-month, dose-level blinded safety and efficacy study of levodopa inhalation powder (CVT-301, Inbrija) in patients with Parkinson's disease. Parkinsonism Relat Disord. 2020;81:144-50

22. Ruan X, Lin F, Wu D, Chen L, Weng H, Yu J, et al. Comparative Efficacy and Safety of Dopamine Agonists in Advanced Parkinson's Disease With Motor Fluctuations: A Systematic Review and Network Meta-Analysis of Double-Blind Randomized Controlled Trials. Front Neurosci. 2021;15:728083
23. Kulisevsky J, Pagonabarraga J. Tolerability and safety of ropinirole versus other dopamine agonists and levodopa in the treatment of Parkinson's disease: meta-analysis of randomized controlled trials. Drug Saf. 2010;33(2):147-61
24. Stowe R, Ives N, Clarke CE, Handley K, Furmston A, Deane K, et al. Meta-analysis of the comparative efficacy and safety of adjuvant treatment to levodopa in later Parkinson's disease. Mov Disord. 2011;26(4):587-98
25. Fox SH, Katzenschlager R, Lim SY, Ravina B, Seppi K, Coelho M, et al. The Movement Disorder Society Evidence-Based Medicine Review Update: Treatments for the motor symptoms of Parkinson's disease. Mov Disord. 2011;26 Suppl 3:S2-41
26. Gaire S, Kafle S, Bastakoti S, Paudel A, Karki K. Continuous Subcutaneous Apomorphine Infusion in Advanced Parkinson's Disease: A Systematic Review. Cureus. 2021;13(9):e17949
27. Trenkwalder C, Chaudhuri KR, García Ruiz PJ, LeWitt P, Katzenschlager R, Sixel-Döring F, et al. Expert Consensus Group report on the use of apomorphine in the treatment of Parkinson's disease--Clinical practice recommendations. Parkinsonism Relat Disord. 2015;21(9):1023-30
28. Hislop J, Margolesky J, Shpiner DS. Sublingual apomorphine in treatment of Parkinson's disease: a review. Int J Neurosci. 2022:1-7
29. Olanow CW, Factor SA, Espay AJ, Hauser RA, Shill HA, Isaacson S, et al. Apomorphine sublingual film for off episodes in Parkinson's disease: a randomised, double-blind, placebo-controlled phase 3 study. Lancet Neurol. 2020;19(2):135-44
30. Binde CD, Tvete IF, Gåsemyr JI, Natvig B, Klemp M. Comparative effectiveness of dopamine agonists and monoamine oxidase type-B inhibitors for Parkinson's disease: a multiple treatment comparison meta-analysis. Eur J Clin Pharmacol. 2020;76(12):1731-43
31. Chen JJ, Obering C. A review of intermittent subcutaneous apomorphine injections for the rescue management of motor fluctuations associated with advanced Parkinson's disease. Clin Ther. 2005;27(11):1710-24
32. Castillo-Torres SA, Lees AJ, Merello M. Intermittent Apomorphine Use for off Period Rescue in Parkinson's Disease: A Pragmatic Review of over Three Decades of Clinical Experience. Mov Disord Clin Pract. 2023;10(2):190-208
33. Dewey RB, Jr., Hutton JT, LeWitt PA, Factor SA. A randomized, double-blind, placebo-controlled trial of subcutaneously injected apomorphine for parkinsonian off-state events. Arch Neurol. 2001;58(9):1385-92
34. Kidoguchi K, Ogawa M, Karam JD, McNeil JS, Fitch MS. Hemoglobin biosynthesis in individual bursts in culture: studies of human umbilical cord blood. Blood. 1979;53(3):519-22
35. Pahwa R, Koller WC, Trosch RM, Sherry JH. Subcutaneous apomorphine in patients with advanced Parkinson's disease: a dose-escalation study with randomized, double-blind, placebo-controlled crossover evaluation of a single dose. J Neurol Sci. 2007;258(1-2):137-43
36. van Laar T, Jansen EN, Essink AW, Neef C, Oosterloo S, Roos RA. A double-blind study of the efficacy of apomorphine and its assessment in 'off'-periods in Parkinson's disease. Clin Neurol Neurosurg. 1993;95(3):231-5
37. Merello M, Pikielny R, Cammarota A, Leiguarda R. Comparison of subcutaneous apomorphine versus dispersible madopar latency and effect duration in Parkinson's disease patients: a double-blind single-dose study. Clin Neuropharmacol. 1997;20(2):165-7
38. Nyholm D. Pharmacokinetic optimisation in the treatment of Parkinson's disease : an update. Clin Pharmacokinet. 2006;45(2):109-36
39. Nyholm D, Stepien V. Levodopa fractionation in Parkinson's disease. J Parkinsons Dis. 2014;4(1):89-96

40. Destée A, Rérat K, Bourdeix I. Is there a difference between levodopa/ dopa-decarboxylase inhibitor and entacapone and levodopa/dopa-decarboxylase inhibitor dose fractionation strategies in Parkinson's disease patients experiencing symptom re-emergence due to wearing-off? The Honeymoon Study. Eur Neurol. 2009;61(2):69-75
41. Olanow CW, Kieburtz K, Odin P, Espay AJ, Standaert DG, Fernandez HH, et al. Continuous intrajejunal infusion of levodopa-carbidopa intestinal gel for patients with advanced Parkinson's disease: a randomised, controlled, double-blind, double-dummy study. Lancet Neurol. 2014;13(2):141-9
42. Nyholm D, Ehrnebo M, Lewander T, Trolin CG, Bäckström T, Panagiotidis G, et al. Frequent administration of levodopa/carbidopa microtablets vs levodopa/carbidopa/entacapone in healthy volunteers. Acta Neurol Scand. 2013;127(2):124-32
43. Fahn S, Oakes D, Shoulson I, Kieburtz K, Rudolph A, Lang A, et al. Levodopa and the progression of Parkinson's disease. N Engl J Med. 2004;351(24):2498-508
44. Lin MM, Laureno R. Less Pulsatile Levodopa Therapy (6 Doses Daily) Is Associated with a Reduced Incidence of Dyskinesia. J Mov Disord. 2019;12(1):37-42
45. Golbe LI, Lieberman AN, Muenter MD, Ahlskog JE, Gopinathan G, Neophytides AN, et al. Deprenyl in the treatment of symptom fluctuations in advanced Parkinson's disease. Clin Neuropharmacol. 1988;11(1):45-55
46. Waters CH, Sethi KD, Hauser RA, Molho E, Bertoni JM. Zydis selegiline reduces off time in Parkinson's disease patients with motor fluctuations: a 3-month, randomized, placebo-controlled study. Mov Disord. 2004;19(4):426-32
47. Ondo WG, Sethi KD, Kricorian G. Selegiline orally disintegrating tablets in patients with Parkinson disease and "wearing off" symptoms. Clin Neuropharmacol. 2007;30(5):295-300
48. A randomized placebo-controlled trial of rasagiline in levodopa-treated patients with Parkinson disease and motor fluctuations: the PRESTO study. Arch Neurol. 2005;62(2):241-8
49. Rascol O, Brooks DJ, Melamed E, Oertel W, Poewe W, Stocchi F, Tolosa E. Rasagiline as an adjunct to levodopa in patients with Parkinson's disease and motor fluctuations (LARGO, Lasting effect in Adjunct therapy with Rasagiline Given Once daily, study): a randomised, double-blind, parallel-group trial. Lancet. 2005;365(9463):947-54
50. Borgohain R, Szasz J, Stanzione P, Meshram C, Bhatt M, Chirilineau D, et al. Randomized trial of safinamide add-on to levodopa in Parkinson's disease with motor fluctuations. Mov Disord. 2014;29(2):229-37
51. Borgohain R, Szasz J, Stanzione P, Meshram C, Bhatt MH, Chirilineau D, et al. Two-year, randomized, controlled study of safinamide as add-on to levodopa in mid to late Parkinson's disease. Mov Disord. 2014;29(10):1273-80
52. Binde CD, Tvete IF, Gåsemyr J, Natvig B, Klemp M. A multiple treatment comparison meta-analysis of monoamine oxidase type B inhibitors for Parkinson's disease. Br J Clin Pharmacol. 2018;84(9):1917-27
53. Yan R, Cai H, Cui Y, Su D, Cai G, Lin F, Feng T. Comparative efficacy and safety of monoamine oxidase type B inhibitors plus channel blockers and monoamine oxidase type B inhibitors as adjuvant therapy to levodopa in the treatment of Parkinson's disease: a network meta-analysis of randomized controlled trials. Eur J Neurol. 2023;30(4):1118-34
54. Jost WH, Friede M, Schnitker J. Indirect meta-analysis of randomised placebo-controlled clinical trials on rasagiline and selegiline in the symptomatic treatment of Parkinson's disease. Basal Ganglia. 2012;2(4):S17-S26
55. Jost WH. A critical appraisal of MAO-B inhibitors in the treatment of Parkinson's disease. J Neural Transm (Vienna). 2022;129(5-6):723-36
56. Müller WE, Reichmann H. Pharmakokinetik und Pharmakodynamik von Selegilin und Rasagilin. Psychopharmakotherapie. 2012(19):191–201
57. Müller T, Hoffmann JA, Dimpfel W, Oehlwein C. Switch from selegiline to rasagiline is beneficial in patients with Parkinson's disease. J Neural Transm (Vienna). 2013;120(5):761-5

58. Song Z, Zhang J, Xue T, Yang Y, Wu D, Chen Z, et al. Different Catechol-O-Methyl Transferase Inhibitors in Parkinson's Disease: A Bayesian Network Meta-Analysis. Front Neurol. 2021;12:707723
59. Deane KH, Spieker S, Clarke CE. Catechol-O-methyltransferase inhibitors for levodopa-induced complications in Parkinson's disease. Cochrane Database Syst Rev. 2004;2004(4):Cd004554
60. Talati R, Reinhart K, Baker W, White CM, Coleman CI. Pharmacologic treatment of advanced Parkinson's disease: a meta-analysis of COMT inhibitors and MAO-B inhibitors. Parkinsonism Relat Disord. 2009;15(7):500-5
61. Scott LJ. Opicapone: A Review in Parkinson's Disease. CNS Drugs. 2021;35(1):121-31
62. Ferreira JJ, Lees A, Rocha JF, Poewe W, Rascol O, Soares-da-Silva P. Opicapone as an adjunct to levodopa in patients with Parkinson's disease and end-of-dose motor fluctuations: a randomised, double-blind, controlled trial. Lancet Neurol. 2016;15(2):154-65
63. Artusi CA, Sarro L, Imbalzano G, Fabbri M, Lopiano L. Safety and efficacy of tolcapone in Parkinson's disease: systematic review. Eur J Clin Pharmacol. 2021;77(6):817-29
64. Fabbri M, Ferreira JJ, Lees A, Stocchi F, Poewe W, Tolosa E, Rascol O. Opicapone for the treatment of Parkinson's disease: A review of a new licensed medicine. Mov Disord. 2018;33(10):1528-39
65. Katsaiti I, Nixon J. Are There Benefits in Adding Catechol-O Methyltransferase Inhibitors in the Pharmacotherapy of Parkinson's Disease Patients? A Systematic Review. J Parkinsons Dis. 2018;8(2):217-31
66. Lyytinen J, Kaakkola S, Gordin A, Kultalahti E, Teräväinen H. Entacapone and selegiline with L-dopa in patients with Parkinson's disease: an interaction study. Parkinsonism Relat Disord. 2000;6(4):215-22
67. Hauser RA, Hattori N, Fernandez H, Isaacson SH, Mochizuki H, Rascol O, et al. Efficacy of Istradefylline, an Adenosine A2A Receptor Antagonist, as Adjunctive Therapy to Levodopa in Parkinson's Disease: A Pooled Analysis of 8 Phase 2b/3 Trials. J Parkinsons Dis. 2021;11(4):1663-75
68. Wang XL, Feng ST, Chen B, Hu D, Wang ZZ, Zhang Y. Efficacy and safety of istradefylline for Parkinson's disease: A systematic review and meta-analysis. Neurosci Lett. 2022;774:136515
69. Müller T. The role of istradefylline in the Parkinson's disease armamentarium. Expert Opin Pharmacother. 2023;24(7):863-71
70. Fox SH, Katzenschlager R, Lim SY, Barton B, de Bie RMA, Seppi K, et al. International Parkinson and movement disorder society evidence-based medicine review: Update on treatments for the motor symptoms of Parkinson's disease. Mov Disord. 2018;33(8):1248-66
71. Senek M, Nyholm D, Nielsen EI. Population pharmacokinetics of levodopa gel infusion in Parkinson's disease: effects of entacapone infusion and genetic polymorphism. Sci Rep. 2020;10(1):18057

3.5 Dyskinesien

Autoren: Dirk Woitalla, Georg Ebersbach, Claudia Trenkwalder

Fragestellung 69: Wie effektiv ist die zusätzliche Gabe von Amantadin im Vergleich mit Placebo in der Behandlung von Pat. mit fortgeschrittener PK mit Dyskinesien?

Hintergrund

Dyskinesien sind definiert als unwillkürliche auftretende Bewegungen, die bei Parkinson-Pat. nach der Behandlung mit Levodopa nach einigen Jahren Krankheitsdauer auftreten und zu einer signifikanten Einschränkung der Bewegung und auch Lebensqualität führen können. Das am häufigsten auftretende Dyskinesie-Muster besteht aus den sog. Peak-dose-Dyskinesien, die ungefähr 30 bis 90 min nach Einnahme einer oralen Levodopa-Dosis auftreten, meist stereotypisch mit choreatischen oder ballistischen Bewegungen auftreten und auch im Parkinson-Tiermodell nach wiederholten Levodopa-Gaben induziert werden können [1]. Levodopa-induzierte Dyskinesien sind charakteristisch für die Diagnose einer PK und bereits in den UK Brain Bank-Kriterien als Teil der diagnostischen Kriterien aufgeführt. Neben den häufigen Peak-dose-Dyskinesien können auch biphasische Dyskinesien auftreten, die beim Anfluten oder Abfluten einer Levodopa-Dosis bei einem intermediären Levodopa-Spiegel auftreten. Dyskinesien sind im Charakter choreatisch, können auch myoklonisch oder dyston sein und betreffen vor allem die oberen Extremitäten, aber auch den Kopf, Rumpf, Nacken und die unteren Extremitäten. Die biphasischen Dyskinesien sind häufiger dyston und gehen in einen Wearing-off-Zustand über. [2] Gelegentlich können auch die Atemmuskeln betroffen sein.

Als Ursache der Dyskinesien wird eine Kombination einer pulsatilen dopaminergen Stimulation der Dopaminrezeptoren über längere Zeit angenommen, verknüpft mit einer zunehmenden Degeneration, und der daraus folgenden Dysregulation von Genen und Proteinen in den nachgeschalteten neuronalen Netzwerken, die zu der Änderung des Aktivierungsmusters führt [3].

Evidenzgrundlage

Amantadin ist ein NMDA-Rezeptor-Antagonist mit geringer dopaminerger und anticholinerger Wirkung und wird überwiegend in der Therapie der Wirkungsfluktuationen und Dyskinesien eingesetzt. Es existiert eine konsistente Datenlage aus insgesamt sieben randomisierten, placebo-kontrollierten Therapiestudien an kleineren Kollektiven Levodopa-behandelter Parkinson-Pat. mit motorischen Komplikationen, die für eine kurzfristige antidyskinetische Wirksamkeit von Amantadin sprechen [4], [5], [6], [7], [8], [9], [10].

Eine Metaanalyse aller Studien unterstützt die Robustheit dieses Effekts [11]. Eine randomisierte, placebokontrollierte Studie spricht für die Persistenz der antidyskinetischen Wirkung von Amantadin im Langzeitverlauf [10].

Das Nebenwirkungsprofil umfasst psychiatrische Nebenwirkungen, Mundtrockenheit, Sehstörungen und QT-Zeit-Verlängerungen im EKG.

Ergebnis

Die Mehrzahl der mit Levodopa behandelten Parkinson-Pat. entwickeln unter langjähriger Dauertherapie mit Levodopa motorische Komplikationen in Form von Wirkungsfluktuationen und Dyskinesien. Der NMDA-Rezeptorantagonist Amantadin hat in mehreren randomisierten, placebokontrollierten Studien in den letzten Jahren konsistente Effekte auf die Reduktion von Dyskinesien gezeigt.

Empfehlung	Modifiziert<br>Stand (2023)
▪ Amantadin sollte zur Reduktion von Dyskinesien bei Parkinson-Pat. mit Levodopa-induzierten motorischen Komplikationen unter Berücksichtigung anticholinerger und halluzinogener Nebenwirkungen eingesetzt werden.<br>▪ Der Einsatz von Amantadin bedarf insbesondere bei geriatrischen Pat. eines umfassenden Monitorings (von psychiatrischen Nebenwirkungen wie Halluzinationen, Nierenretentionsparametern, Restharn- und EKG-Kontrollen wegen möglicher QT-Zeit-Verlängerung).	
Konsensstärke: 100%, starker Konsens	

Fragestellung 70: Wie effektiv ist die zusätzliche Gabe von MAO-B-Hemmern im Vergleich mit Placebo in der Behandlung von Pat. mit fortgeschrittener PK mit Dyskinesien?

Hintergrund

Dyskinesien sind definiert als unwillkürliche auftretende Bewegungen, die bei Parkinson-Pat. nach der Behandlung mit Levodopa auftreten (siehe oben).

Evidenzgrundlage

Der Effekt von Safinamid mit einem dualen Wirkmechanismus, einem partiellen NMDA-Rezeptor-Antagonisten und MAO-B-Hemmer, auf die Frequenz und Häufigkeit von Dyskinesien wurde in 2 Studien untersucht. In einer dreiarmigen prospektiven, randomisierten, placebokontrollierten Therapiestudie mit Safinamid über einen Zeitraum von 24 Wochen fand sich kein statistisch signifikanter Effekt von „troublesome dyskinesias“ unter 50 mg oder 100 mg Safinamid, jedoch auch keine Zunahme der Dyskinesien trotz signifikanter Zunahme der On-Zeit. Dies wird von den Autoren/Autorinnen keinem MAO-B-inhibitorischen, sondern einem NMDA-Rezeptor-antagonistischen Effekt zugschrieben [12]. Bei einer Post-hoc-Analyse, die den Effekt von 50 mg und 100 mg Safinamid bei einer Behandlungsdauer von 24 Monaten bei mit Levodopa behandelten Parkinson-Pat. mit motorischen Komplikationen untersuchte, konnte nur ein signifikanter Effekt der Dyskinesie-Reduktion bei 100 mg Safinamid beobachtet werden, wenn die Pat. bei Baseline mittelgradige bis schwere Dyskinesien aufwiesen, obwohl in der Gesamtheit der Pat. keine signifikanten Verbesserungen der Dyskinesien auftraten [13]. In der Gruppe der Parkinson-Pat. ohne Änderung der Levodopa-Dosis war eine Reduktion der Dyskinesien in einer 2-Jahres-Post-hoc-Analyse bei einer Dosis von 100 mg weiterhin signifikant [14].

Ergebnis

Die Datenlage zum Einfluss von Safinamid in einer Dosis von 50 mg oder 100 mg auf die Intensität und Frequenz von Dyskinesien ist nicht eindeutig. Sekundäre Effekte über die Einsparung des Levodopa sind nicht auszuschließen.

**Empfehlung**	**Modifiziert**<br>**Stand (2023)**
Safinamid kann zur Behandlung mittelgradiger bis schwerer Dyskinesien erwogen werden.<br>Die Datenlage zur Wirkung und Dosis von Safinamid auf Dyskinesien ist nicht eindeutig; Möglicherweise ist die partielle NMDA-Rezeptor-Wirkung für eine Reduktion von Dyskinesien verantwortlich, Effekte durch eine Einsparung des Levodopa können nicht ausgeschlossen werden.<br>Aufgrund mangelnder Evidenz kann keine Empfehlung zur Therapie von Dyskinesien mit den MAO-B-Hemmern Rasagilin oder Selegelin gegeben werden.	
Konsensstärke: 85,7%, Konsens	

Fragestellung 71: Wie effektiv ist die zusätzliche Gabe von COMT-Hemmern im Vergleich mit Placebo in der Behandlung von Pat. mit fortgeschrittener PK mit Dyskinesien?

Hintergrund

Dyskinesien sind definiert als unwillkürliche auftretende Bewegungen, die bei Parkinson-Pat. nach der Behandlung mit Levodopa auftreten (siehe oben).

Evidenzgrundlage

Es gibt keine ausreichende Evidenz zur Beurteilung der Wirksamkeit der Substanzklasse der COMT-Hemmer bei der Behandlung von Dyskinesien.

**Empfehlung**	**Neu**<br>**Stand (2023)**
Aufgrund mangelnder Evidenz kann keine Empfehlung zur Therapie von Dyskinesien mit COMT-Hemmern gegeben werden.	
Konsensstärke: 100%, starker Konsens	

Fragestellung 72: Wie effektiv ist die zusätzliche Gabe von Dopaminagonisten im Vergleich mit Placebo in der Behandlung von Pat. mit fortgeschrittener PK mit Dyskinesien?

Hintergrund

Dyskinesien sind definiert als unwillkürliche auftretende Bewegungen, die bei Parkinson-Pat. nach der Behandlung mit Levodopa auftreten.

Evidenzgrundlage

Es gibt keine ausreichende Evidenz zur Beurteilung der Wirksamkeit der Substanzklasse der Dopaminagonisten bei der Behandlung von Dyskinesien.

**Empfehlung**	**Neu**<br>**Stand (2023)**
Aufgrund mangelnder Evidenz kann keine Empfehlung zur Therapie von Dyskinesien mit Dopaminagonisten gegeben werden.	
Konsensstärke: 100%, starker Konsens	

Referenzen

1. Bariotto-Dos-Santos K, Ribeiro DL, Guimarães RP, Padovan-Neto FE. Rating L-DOPA-Induced Dyskinesias in the Unilaterally 6-OHDA-Lesioned Rat Model of Parkinson's Disease. J Vis Exp. 2021(176)
2. Jankovic J. Motor fluctuations and dyskinesias in Parkinson's disease: clinical manifestations. Mov Disord. 2005;20 Suppl 11:S11-6
3. Obeso JA, Rodriguez-Oroz MC, Rodriguez M, DeLong MR, Olanow CW. Pathophysiology of levodopa-induced dyskinesias in Parkinson's disease: problems with the current model. Ann Neurol. 2000;47(4 Suppl 1):S22-32; discussion S-4
4. da Silva-Júnior FP, Braga-Neto P, Sueli Monte F, de Bruin VM. Amantadine reduces the duration of levodopa-induced dyskinesia: a randomized, double-blind, placebo-controlled study. Parkinsonism Relat Disord. 2005;11(7):449-52
5. Del Dotto P, Pavese N, Gambaccini G, Bernardini S, Metman LV, Chase TN, Bonuccelli U. Intravenous amantadine improves levadopa-induced dyskinesias: an acute double-blind placebo-controlled study. Mov Disord. 2001;16(3):515-20
6. Luginger E, Wenning GK, Bösch S, Poewe W. Beneficial effects of amantadine on L-dopa-induced dyskinesias in Parkinson's disease. Mov Disord. 2000;15(5):873-8
7. Snow BJ, Macdonald L, McAuley D, Wallis W. The effect of amantadine on levodopa-induced dyskinesias in Parkinson's disease: a double-blind, placebo-controlled study. Clin Neuropharmacol. 2000;23(2):82-5
8. Thomas A, Iacono D, Luciano AL, Armellino K, Di Iorio A, Onofrj M. Duration of amantadine benefit on dyskinesia of severe Parkinson's disease. J Neurol Neurosurg Psychiatry. 2004;75(1):141-3
9. Verhagen Metman L, Del Dotto P, van den Munckhof P, Fang J, Mouradian MM, Chase TN. Amantadine as treatment for dyskinesias and motor fluctuations in Parkinson's disease. Neurology. 1998;50(5):1323-6
10. Wolf E, Seppi K, Katzenschlager R, Hochschorner G, Ransmayr G, Schwingenschuh P, et al. Long-term antidyskinetic efficacy of amantadine in Parkinson's disease. Mov Disord. 2010;25(10):1357-63
11. Elahi B, Phielipp N, Chen R. N-Methyl-D-Aspartate antagonists in levodopa induced dyskinesia: a meta-analysis. Can J Neurol Sci. 2012;39(4):465-72
12. Hattori N, Tsuboi Y, Yamamoto A, Sasagawa Y, Nomoto M. Efficacy and safety of safinamide as an add-on therapy to L-DOPA for patients with Parkinson's disease: A randomized, double-blind, placebo-controlled, phase II/III study. Parkinsonism Relat Disord. 2020;75:17-23

13. Borgohain R, Szasz J, Stanzione P, Meshram C, Bhatt MH, Chirilineau D, et al. Two-year, randomized, controlled study of safinamide as add-on to levodopa in mid to late Parkinson's disease. Mov Disord. 2014;29(10):1273-80
14. Cattaneo C, Ferla RL, Bonizzoni E, Sardina M. Long-Term Effects of Safinamide on Dyskinesia in Mid- to Late-Stage Parkinson's Disease: A Post-Hoc Analysis. J Parkinsons Dis. 2015;5(3):475-81

3.6 Parkinson-Tremor

Autoren/Autorinnen: Franziska Hopfner, Jos Becktepe, Günther Deuschl

Diagnose und Klassifikation des Parkinson-Tremors

Jede pathologische Form von Tremor bei Parkinson-Pat. wird als Parkinson-Tremor klassifiziert [1]. Der Ruhetremor stellt ein wichtiges Leitsymptom der PK dar und zeigt sich als häufigstes motorisches Zeichen der PK bei fast 90% der Pat. irgendwann im Verlauf der Krankheit [1]. Ruhetremor tritt meist zuerst an den oberen Extremitäten auf, seltener an den unteren Extremitäten, und breitet sich dann auf die andere Körperseite aus. Auch Lippen, Kinn und Gesicht können vom Ruhetremor betroffen sein. Eine Form des Ruhetremors, die spezifisch für die PK ist, ist der meist einseitig beginnende Pillendreher-Tremor, der durch repetitive Flexionsbewegungen von Daumen und Zeigefinger gekennzeichnet ist [2].

Viele Parkinson-Pat. zeigen zudem auch einen Aktionstremor in Form eines Halte- und Bewegungstremors oder in Form eines kurz nach Übergang von Ruhe in Aktion auftretenden Tremors in Ruhetremorfrequenz (Ruhetremordurchbruch, synonym: re-emergent Tremor) [3], [1].

In der 1998 veröffentlichen Tremor-Klassifikation der Movement Disorder Society (MDS) wurde der 4–6-Hz-Ruhetremor mit oder ohne Ruhetremordurchbruch als Typ-I-Parkinson-Tremor, ein isolierter höherfrequenter Aktionstremor als Typ-III-Parkinson-Tremor und eine Kombination beider Tremorformen als Typ II klassifiziert [3]. Der höherfrequente Aktionstremor scheint schlechter auf dopaminerge Therapie anzusprechen [4] als der klassische Typ-I-Parkinson-Tremor [5], während das Ansprechen des re-emergent Tremors auf Dopaminergika dem des Ruhetremor entspricht [6]. Klinische Studien unterscheiden meist nicht zwischen Aktionstremor in der Ruhetremorfrequenz und höherfrequentem Aktionstremor. In dem Consensus Statement zur Tremorklassifikation der Movement Disorder Society von 2018 wird der Parkinson-Tremor der Kategorie der Tremorformen mit prominenten zusätzlichen klinischen Zeichen (im Fall des Parkinson-Tremors der Parkinsonismus) zugeordnet [1].

Allgemeiner Behandlungshinweis für den Parkinson-Tremor

Die optimale Behandlung des Parkinson-Tremors kann nicht unabhängig von allgemeinen Therapierichtlinien bewertet werden, die für alle Symptome der PK gelten. Dem trägt auch diese Leitlinie durch die Einbettung spezifischer tremorbezogener Empfehlungen in die allgemeinen Empfehlungen der jeweiligen Phase der Erkrankung Rechnung. Großer Wert wurde darauf gelegt aufzuzeigen, wann eine Behandlungsmaßnahme nur zur Verbesserung des Symptoms „Tremor“ hinzugezogen werden kann. Hochspezifische Behandlungsstrategien zur Behandlung von Tremores im Rahmen verschiedener Tremorsyndrome finden sich in den „Tremor – Leitlinien für Diagnostik und Therapie in der Neurologie“ (AWMF-Registernummer: 030/011). Die vorliegenden Empfehlungen sind mit denen der Tremor-Leitlinie abgestimmt.

Fragestellung 73: Wie effektiv sind Dopaminergika (Levodopa/Dopaminagonisten) im Vergleich mit Placebo für die Behandlung des Tremors bei der PK?

Hintergrund

Levodopa gilt als das wirksamste orale Medikament zur Behandlung von motorischen Symptomen bei der PK [7]. Die dosisabhängige Wirksamkeit von Levodopa/Carbidopa bei der frühen PK wurde in der doppelblinden, randomisierten, placebokontrollierten ELLDOPA-Studie nachgewiesen [8]. Der Zweck dieser Studie war es jedoch, die Wirkung von Levodopa auf die Krankheitsprogression im Allgemeinen zu untersuchen [8]. Diese Studie hob jedoch auch das dosisabhängige Risiko motorischer Komplikationen durch Levodopa hervor. Nur wenige kontrollierte Studien haben sich speziell mit der Wirksamkeit von Levodopa bei Parkinson-Tremor befasst.

Evidenzgrundlage

In dieser Literaturrecherche fanden sich:

N=9 randomisierte, kontrollierte Studien

N=1 longitudinale größere Kohortenstudien

N=2 Falldarstellungen und Reviews.

Ergebnis

Koller et al. führten eine doppelblinde Kurzzeit-Vergleichsstudie (zwei Wochen Behandlungsdauer) zu den Wirkungen von Levodopa/Carbidopa, Trihexyphenidyl und Amantadin auf die Tremorreduktion bei der PK durch [9]. Die Reduktion der Tremoramplitude betrug 59 % bei Trihexyphenidyl, 55 % bei Levodopa und 23 % bei Amantadin. Nach der verblindeten Studienphase bevorzugten fünf von neun zuvor unbehandelten Pat. Trihexyphenidyl, vier bevorzugten Levodopa und keiner bevorzugte Amantadin.

Tedeschi et al. untersuchten in einer einfach verblindeten Crossover-Studie die akuten Wirkungen von 250 mg Levodopa (plus Carbidopa) im Vergleich zu Placebo auf Ruhe- und Haltetremor bei zehn Parkinson-Pat. Die Pat. zeigten ein gutes Ansprechen der Medikation auf Rigor und Akinese und boten keine Wirkfluktuationen.

Die Wirkung von 250 mg Levodopa (plus Carbidopa) wurde vier Stunden nach der letzten vorangegangenen Medikationseinnahme auf den akzelerometrisch gemessenen Fingertremor der stärker betroffenen Körperhälfte erhoben. In dieser Studie führte die Anwendung von Levodopa zu einer signifikanten Reduktion der Ruhe- und Haltetremoramplitude im Vergleich zu Placebo [10].

Hughes et al. untersuchten die Wirkungen von Levodopa und Apomorphin auf den Ruhetremor bei zwanzig Parkinson-Pat. mit Tremor-dominanter PK, bei zwölf Pat. mit Fluktuationen und schwerem Off-Tremor, bei fünf nicht fluktuierenden Pat. mit schwer behandelbarem Tremor und bei drei Pat. mit De-novo-Parkinson-Krankheit [11]. Die Pat. erhielten im Rahmen der Studie eine Einzeldosis von 250/25 mg Levodopa/Carbidopa mehr als 12 Stunden nach Pausieren der Parkinson-Medikation, gefolgt von 1,5 mg am nächsten Tag, dann 3 mg und schließlich 4,5 mg subkutanem Apomorphin. Die Beurteilung der Tremorschwere erfolgte anhand einer eigens entwickelten, nicht validierten 4-Punkte-Skala. 19/20 Pat. sprachen vergleichbar auf beide Therapien an, bei 10 Pat. klang der Tremor vorübergehend völlig ab. Im Durchschnitt kam es zu einer 50%igen Besserung des Ruhetremors [11].

Die Wirkung von L-Dopa versus Placebo auf die akzelerometrisch gemessene Amplitude von Parkinson-Ruhe- und -Haltetremor wurde von Hendersen et al. untersucht [12], [13]. Pat. mit Levodopa-responsiver PK (50% mit Wirkfluktuationen) erhielten > 12 Stunden nach Einnahme der letzten Parkinson-Medikation randomisiert und verblindet ansteigende Einzeldosen Levodopa/Benserazid (100/25, 200/50 bzw. 300/75 mg), Propranolol (20, 40 bzw. 80 mg) oder Placebo. Levodopa führte dosisabhängig zu einer signifikant stärkeren Reduktion der Ruhe- und Haltetremoramplitude als Placebo. Die höchste Levodopa-Dosis führte bei fünf/elf Pat. zu einer >50%igen und bei drei dieser Pat. zu einer >95%igen Abnahme der Amplitude des Ruhe- und Haltetremors.

Eine Studie untersuchte den Effekt von 200 mg Levodopa/Benserazid bei 76 Parkinson-Pat. im definierten Off (≥12 h nach ihrer letzten L-Dopa-Dosis) [14]. Bradykinese und Rigor mussten auf Levodopa ansprechen und die Pat. einen Ruhetremor an zumindest einer oberen Extremität zeigen. Die Levodopa-Einzeldosis führte im Mittel zu einer vergleichbaren Besserung von Bradykinese (43%), Rigor (45%) und Tremor (50%). Mittels Cluster-Analyse konnten die Autoren/Autorinnen zwischen drei Clustern mit gutem, intermediärem und schlechtem Ansprechen des Tremors auf Levodopa differenzieren. Ein, wenn auch geringerer, Unterschied der Antwort auf Levodopa in dieselbe Richtung fand sich für Bradykinese und Rigor. Pat. mit gutem Ansprechen der Medikation auf das Symptom Tremor hatten im Vergleich zu Pat., bei denen sich die Medikation nur insuffizient auf den Tremor auswirkte, eine deutlich stärkere motorische Behinderung im Off, einen ausgeprägteren Tremor im Off, eine längere Krankheitsdauer, eine höhere habituelle Levodopa-Äquivalenzdosis und eine höhere Frequenz von Dyskinesien, sodass die drei Cluster nur bedingt vergleichbar sind.

Insgesamt zeigt Levodopa eine stärkere Wirkung auf die motorische Parkinson-Symptomatik als der Einsatz von Dopaminagonisten [15]. In Einzelfällen scheint der Parkinson-Tremor jedoch stärker auf Dopaminagonisten als auf L-Dopa anzusprechen. Zur Wirkung von oralen Dopaminagonisten in der Behandlung des schwer behandelbaren Parkinson-Tremors liegen zwei randomisierte kontrollierte Studien vor.

Pogarell et al. behandelten 84 Parkinson-Pat. mit ausgeprägtem, therapierefraktärem Tremor (mediane Levodopa-Dosis allerdings nur 300 mg). Die Pat. wurden randomisiert mit Pramipexol (n=44) oder Placebo (n=40) behandelt [16]. Die Dauer der Aufdosierungsphase betrug bis zu sieben Wochen, die Dauer der Erhaltungsphase vier Wochen. Primärer Endpunkt der Studie war die absolute Änderung des Tremor-Scores, bestehend aus Items 16 (ADL), 20 und 21 (Ruhe- und Aktionstremor im Motor Score) der Unified-Parkinson's-Disease-Rating-Skala. Die mittlere Pramipexol-Dosis in der Erhaltungsphase lag bei 4,1 mg. Pramipexol führte zu einer signifikant stärkeren Abnahme des Tremor-Scores (-5,5 bzw. 48% gegenüber Baseline) als Placebo (-1,5 bzw. 14% gegenüber Baseline). Es konnte sowohl für Ruhe- als auch für Aktionstremor eine signifikante Tremorreduktion verzeichnet werden. Dabei war die Reduktion des Tremors in der Dosistitrationsphase besonders ausgeprägt, die Verbesserung des Tremors zeigte sich jedoch auch in der Erhaltungsphase stabil. Das Nebenwirkungsspektrum entsprach jenem von Non-Ergot-Dopaminagonisten in anderen Studien. Insgesamt ist anzumerken, dass die mediane Levodopa-Tagesdosis niedrig war, die Pat.-Zahl in dieser Studie relativ niedrig und die Therapiedauer kurz.

Navan et al. behandelten 30 Parkinson-Pat. mit zumindest mäßiggradigem Parkinson-Tremor randomisiert und doppelblind mit Pramipexol (n=10), dem Ergot-Dopaminagonisten Pergolid (n=10) oder Placebo (n=10) in einem Parallelgruppendesign [17]. Die Therapiedauer betrug inklusive einer Aufdosierungsphase von bis zu zehn Wochen insgesamt drei Monate. Die Maximaldosis betrug 4,5mg Pramipexol bzw. 4,5 mg Pergolid. Als primärer Outcome-Parameter diente die jeweilige Änderung eines von den Autoren/Autorinnen entwickelten Tremorscores von 0–10 jeweils für Ruhetremor, Haltetremor und das Zeichnen einer Archimedes-Spirale der jeweils stärker betroffenen oberen Extremität. Zu den sekundären Endpunkten zählten unter anderem der Punktwert auf der Unified-Parkinson's-Disease-Rating-Skala und eine akzelerometrische Tremor-Messung. Die Mehrheit der Pat. war bei Einschluss in diese Studie bereits mit Parkinson-Medikation vorbehandelt gewesen. Etwa die Hälfte der Pat. war mit Levodopa behandelt worden, wobei die mittleren Tagesdosen in den einzelnen Therapiearmen zwischen 383 und 550 mg lagen. Eine Vorbehandlung mit Dopaminagonisten war nicht erlaubt. Zwei Pat. in der Placebogruppe schieden aus der Studie aus. Vier Pat. in der Pergolid-Gruppe und kein Pat. in der Pramipexol-Gruppe schieden wegen Nebenwirkungen aus. Je ein/e zusätzliche/r Patient/in in der Pergolid- und Pramipexol-Gruppe erreichte aufgrund von Nebenwirkungen nicht die Zieldosis und wurde nicht in der Wirksamkeitsanalyse berücksichtigt. 8/9 Placebo-Pat. und 18/20 Pat. unter Dopaminagonisten erkannten zu Ende der Studie richtig, dass sie unter Placebo bzw. aktiver Studienmedikation standen. Sowohl Pramipexol als auch Pergolid führten zu einer signifikant stärkeren Abnahme des Tremorindex und des Punktwerts auf dem motorischen Teil der Unified-Parkinson's-Disease-Rating-Skala im Vergleich zu Placebo. Der Unterschied zwischen beiden Dopaminagonisten war nicht signifikant. Die Akzelerometrie zeigte eine signifikante Änderung lediglich des Haltetremors unter Pergolid. Während die Studie belegt, dass der Antitremor-Effekt von Dopaminagonisten wohl ein Klasseneffekt sein dürfte, stellen die geringe Pat.-Zahl, die hohe Dropout-Rate vor allem unter Pergolid sowie der potenzielle Entblindungseffekt wichtige Limitationen der Studie dar [17].

Der Effekt von Ropinirol wurde von Schrag et al. mittels einer retrospektiven Analyse von drei randomisierten, doppelblinden Multicenter-Studien, die insgesamt 844 unbehandelte Pat. einschlossen, untersucht [18]. Der Effekt einer Monotherapie mit Ropinirol, Levodopa, Bromocriptin bzw. Placebo auf Ruhe- und Aktionstremor, gemessen an Items 20 und 21 des Unified Parkinson's Disease Rating Score wurden verglichen. Ropinirol, Bromocriptin und Levodopa, aber nicht Placebo führten zu einer signifikanten Besserung des Ruhetremors. Kein Unterschied auf den Ruhetremor fand sich zwischen Ropinirol, Bromocriptin und Levodopa. Bei niedrigen Baseline-Aktionstremor-Scores führte Ropinirol in der placebokontrollierten Studie zu keiner signifikanten Abnahme des Aktionstremor-Scores. Die Verbesserung des Ruhetremors war unter Ropinirol signifikant besser als unter Placebo. Es gab keine signifikanten Unterschiede zwischen der Wirkung von Ropinirol und der von Levodopa (L-Dopa) oder Bromocriptin auf den Ruhetremor.

Begründung der Empfehlung

Insgesamt liegen zur Wirkung von Levodopa auf den Parkinson-Tremor Daten aus drei placebokontrollierten Single-Dose-Studien vor [11], [10], [12]. Diese zeigen im Einklang mit der klinischen Erfahrung einen guten Effekt von Levodopa auf den Parkinson-Tremor. Dies gilt sowohl für den Ruhe- als auch für den Aktionstremor. Aus den vorliegenden Daten lässt sich nicht ableiten, wie

hoch der Anteil der auf Levodopa schlecht ansprechenden Pat. mit Parkinson-Tremor ist. Auch liegen keine Studiendaten zum Effekt höherer Levodopa-Dosen auf den mit Standarddosen schlecht behandelbaren Parkinson-Tremor vor. Die klinische Erfahrung zeigt, dass der gegen Standarddosen refraktäre Parkinson-Tremor im Einzelfall gut auf eine Erhöhung der Levodopa-Tagesdosis oder auf hohe Levodopa-Einzeldosen (auch in löslicher Form) anspricht.

Orale Dopaminagonisten führen im Vergleich zu Placebo sowohl in Monotherapie als auch in Kombination mit Levodopa zu einer relevanten Besserung des Parkinson-Tremors. Die durchgeführten Studien zeigen, dass Dopaminagonisten wie auch Levodopa sowohl den Ruhe- als auch den Aktionstremor verbessern. Es ist anzunehmen, dass es sich bei der Wirkung von Dopaminagonisten auf den Parkinson-Tremor um einen Klasseneffekt von Dopaminagonisten handelt. Die vorliegenden Daten erlauben aber keinen sicheren Schluss über die Effektgröße von Dopaminagonisten auf den Parkinson-Tremor, insbesondere ob die Effektgröße von Dopaminagonisten gleich groß oder schwächer ist als jene von Levodopa. Es liegt eine schwache Evidenz dafür vor, dass Dopaminagonisten einen Zusatzeffekt bei mit Levodopa unzureichend behandeltem Parkinson-Tremor haben dürften. Ob dies auch bei Vorbehandlung mit Levodopa in höheren Dosierungen der Fall ist, ist nicht belegt.

Eine hohe Levodopa-Dosis wird insbesondere bei jüngeren Pat. mit schwer behandelbarem Parkinson-Tremor wegen des Risikos für die Auslösung motorischer Komplikationen vielfach vermieden. Eine Kombinationstherapie von Levodopa mit Dopaminagonisten kann bei individuell guter Verträglichkeit sinnvoll sein, um die Levodopa-Dosis in einem niedrigeren Bereich halten zu können. Zu bedenken ist aber, dass die Rate anderer, insbesondere neuropsychiatrischer Nebenwirkungen wie Impulskontrollstörungen unter oralen Dopaminagonisten deutlich höher ist als unter einer Behandlung mit Levodopa.

Welche Therapie – Levodopa und oder Dopaminagonist, in welcher Dosis und in welcher Kombination – gewählt wird, muss daher für jeden/jede Pat. individuell entschieden werden.

**Empfehlung**	**Neu**<br>**Stand (2023)**
▪ **Levodopa** wird zur Therapie der Symptome der PK in allen Stadien eingesetzt. Wird Levodopa in der Ersteinstellung mit den Zielsymptomen Akinese und Rigor eingesetzt, bessert sich meist äquivalent auch der Parkinson-Tremor. Gegen Parkinson-Tremor, der refraktär gegenüber Standarddosen von Levodopa ist, können im Einzelfall eine Erhöhung der Levodopa-Tagesdosis oder hohe Levodopa-Einzeldosen hilfreich sein. Eine dauerhafte Erhöhung der Levodopa-Dosis bei schwer behandelbarem Tremor sollte jedoch gegen ein erhöhtes Risiko motorischer Komplikationen abgewogen werden.<br>▪ **Dopaminagonisten** sollten zur Therapie der Symptome der PK in Monotherapie und Kombinationstherapie verwendet werden. Es wird empfohlen, die Einstellung mit den Zielsymptomen Akinese und Rigor vorzunehmen, wobei sich meist äquivalent auch der Parkinson-Tremor bessert.	
Konsensstärke: 100%, starker Konsens	

Fragestellung 74: Wie effektiv sind Anticholinergika im Vergleich mit Placebo für die Behandlung des Tremors bei der PK?

Hintergrund

Obgleich Anticholinergika gemeinhin als effektiv bei der Behandlung von Parkinson-Tremor gelten, gibt es keine klare Evidenz für eine spezifische Wirkung von Anticholinergika auf den Parkinson-Tremor [19]. Der Einsatz von Anticholinergika bei der PK ist aufgrund zunehmend aufkommender alternativer Behandlungsoptionen und des ausgeprägten Nebenwirkungsprofils auf junge, kognitiv nicht eingeschränkte Pat. mit anderweitig insuffizient behandelbarer Tremorsymptomatik sehr begrenzt. Bei älteren und multimorbiden Pat. sind Anticholinergika kontraindiziert [20]. Eine höhere kumulative Einnahme von Anticholinergika ist mit einem erhöhten Risiko für das Auftreten kognitiver Nebenwirkungen assoziiert [20], [21], [22].

Evidenzgrundlage

In dieser Literaturrecherche fanden sich:

N=2 randomisierte, kontrollierten Studien

N=1 Falldarstellungen und Reviews.

Ergebnis

In der oben bereits ausgeführten doppelblinden Kurzzeit-Vergleichsstudie (2 Wochen Behandlungsdauer) von Koller et al., in der die Wirkungen von Levodopa/Carbidopa, Trihexyphenidyl und Amantadin auf die Tremorreduktion bei der PK geprüft wurden, zeigte sich eine Reduktion der Tremoramplitude von 59 % durch eine Behandlung mit dem Anticholinergikum Trihexyphenidyl.

Eine Einzeldosisstudie, die die Wirkung einer fixen Dosis des Anticholinergikums Biperiden (5mg iv.) mit einer individuell wirksamen Apomorphin-Dosis auf Bradykinese, Rigor und Tremor untersuchte, ergab eine vergleichbare Besserung der akzelerometrisch gemessenen Amplitude von Ruhe-, Halte- und Bewegungstremor unter beiden Therapien. Dagegen war eine signifikante Besserung von Rigor und Bradykinese nur nach Apomorphin zu beobachten [23].

Ein Review der Cochrane Collaboration fand kein überzeugend stärkeres Ansprechen des Parkinson-Tremors auf Anticholinergika im Vergleich zu den anderen motorischen Kardinalsymptomen [24]. Neun Studien mit insgesamt 221 Pat. erfüllten die Einschlusskriterien dieses Cochrane Reviews. Alle eingeschlossenen Studien hatten ein doppelblindes Crossover-Design mit einer Studiendauer zwischen 5 und 20 Wochen. Die untersuchten Substanzen waren Benzhexol (mittlere Dosis 8 bis 20 mg/Tag), Orphenadrin, Benztropin, Bornaprin (8 bis 8,25 mg/Tag), Benapryzin (200 mg/Tag) und Methixin (45 mg/Tag). Die Outcome-Parameter variierten stark und die Studienmethodik genügte gegenwärtigen Kriterien in keinem Fall. Fünf Studien nutzten Tremor und andere Parkinson-Symptome als Outcome-Parameter. Diese Studien ergaben stark variable Resultate von einer signifikanten Besserung nur des Tremors bis zu einer Besserung anderer Symptome, aber nicht des Tremors. Abgesehen von einer Studie mit Methixin, zeigten alle Studien eine Besserung zumindest eines Outcome-Parameters unter Anticholinergika [24].

2020 untersuchten Sahoo et al. in einer offenen, nicht randomisierten Single-Dose-Studie den Effekt einer Einzeldosis von Levodopa/Carbidopa mit einer zusätzlichen Einzeldosis Trihexyphenidyl, einem zentral wirkenden Anticholinergikum [25]. Dazu wurden insgesamt 20 Parkinson-Pat., darunter Pat., welche mit Trihexyphenidyl vorbehandelt worden waren, und Pat., welche mit Levodopa vorbehandelt worden waren, ausgewählt. Die im Mittel 58 Jahre alten Pat. mit einer durchschnittlichen Krankheitsdauer von ca. fünf Jahren erhielten im definierten Off (≥12 Stunden nach der letzten Medikation) zunächst 4 mg Trihexyphenidyl und nach zwei Tagen, ebenfalls im definierten Off, 200 mg L-Dopa/Carbidopa. Die Veränderung des Unified Parkinson's Disease Rating Scores diente als primärer Zielparameter. L-Dopa führte zu einer ca. 60%igen, Trihexyphenidyl zu einer ca. 24%igen Besserung des UPDRS Motor Scores. Während das Ausmaß der Besserung der einzelnen motorischen Kardinalsymptome unter L-Dopa vergleichbar war, besserte sich unter Trihexyphenidyl vor allem der Tremor-Subscore (Unified Parkinson's Disease Motor Subscore Items 20 und 21). Auch die Besserung des Tremor-Subscores war unter Levodopa ausgeprägter als unter Trihexyphenidyl (ca. 67% vs. ca. 46%). Bei elf Pat. führte Levodopa zu einer stärkeren Abnahme des Tremors, nur bei 4/20 Pat. Trihexyphenidyl, wobei diese Pat. einen niedrigeren Tremor-Subscore im Off aufwiesen.

Begründung der Empfehlung

Die vorliegenden Studiendaten legen eine Wirksamkeit von Anticholinergika gegen motorische Kardinalsymptome der PK nahe. Einzelne Studien weisen auf eine stärkere Wirkung auf den Parkinson-Tremor als auf Rigor und Bradykinese hin. Die Effektgröße von Anticholinergika auf den Parkinson-Tremor dürfte schwächer als jene von Levodopa sein. Eine Zusatztherapie kann bei Pat. mit durch Dopaminergika nicht gut kontrolliertem Parkinson-Tremor erwogen werden. Der Einsatz von Anticholinergika bei der PK ist aufgrund des hohen Nebenwirkungsrisikos auf junge, kognitiv intakte Pat. limitiert und sollte nur in Ausnahmefällen therapeutische Anwendung finden.

**Empfehlung**	**Modifiziert<br>Stand (2023)**
Der Einsatz von **Anticholinergika** bei Parkinson-Pat. kann wegen der anticholinergen Nebenwirkungen nur noch in absoluten Ausnahmefällen bei anderweitig nicht behandelbarem Tremor erwogen werden.<br>Anticholinergika sollen bei geriatrischen und/oder kognitiv eingeschränkten Pat. nicht eingesetzt werden.	
Konsensstärke: 100%, starker Konsens	

Fragestellung 75: Wie effektiv und sicher sind Betablocker, Primidon oder Clozapin im Vergleich zu Placebo für die Behandlung des Tremors bei der PK?

Hintergrund

Betablocker und Primidon sind nachgewiesen wirksam in der Behandlung verschiedener Tremorsyndrome, insbesondere des essenziellen Tremors (siehe „Tremor – Leitlinien für Diagnostik und Therapie in der Neurologie" (AWMF-Registernummer: 030/011). Für eine Wirksamkeit in der

Behandlung des Parkinson-Tremors gibt es für Propranolol schwache Evidenz, für Primidon gibt es keine ausreichende Evidenz. Clozapin führt im Placebo-Vergleich zu einer signifikanten Besserung des Parkinson-Ruhe- und -Haltetremors, der Einsatz wird jedoch durch die teils gravierenden Nebenwirkungen limitiert.

Evidenzgrundlage

In dieser Literaturrecherche fanden sich N=7 randomisierte, kontrollierte Studien und N=2 Falldarstellungen und Reviews.

Betablocker

In mehreren kleineren Studien mit methodischen Limitationen wurde gezeigt, dass Betablocker wie Propranolol zu einer Besserung des Ruhe- und Aktionstremors bei der PK führen können [26], [27], [28], [12].

In einer randomisierten, verblindeten Crossover-Studie an 10 mit L-Dopa vorbehandelten Pat. wurde die Wirkung von retardiertem Propranolol (160 mg/Tag), Primidon (250 mg zur Nacht) und Clonazepam (4mg/Tag) auf den Parkinson-Tremor untersucht. Propranolol führte zu einer durchschnittlich 70%igen Reduktion der Ruhe- und 50%igen Reduktion der Haltetremor-Amplitude. Weder Clonazepam noch Primidon bewirkten eine relevante Reduktion der Tremor-Amplitude [28].

In einer Studie zur Wirkung von Einzeldosen L-Dopa (100/25, 200/50 bzw. 300/75mg) und Propranolol (20, 40 bzw. 80 mg) gegenüber Placebo auf die akzelerometrisch gemessene Ruhe- und Haltetremor-Amplitude bei 11 L-Dopa-responsiven Parkinson-Pat. führte Propranolol bei 3/11 Pat. zu einer >30%igen Besserung der Ruhe- und bei 4/11 Pat. zu einer >30%igen Besserung der Haltetremor-Amplitude (im Vergleich: L-Dopa führte in der höchsten Dosis bei 5/11 Pat. zu einer >50%igen und bei 3 dieser Pat. zu einer >95%igen Abnahme der Ruhe- und Haltetremor-Amplitude) [12].

In einem Cochrane Review von 2003 zur Therapie des Parkinson-Tremors mit Betablockern wurde keine hinreichende Evidenz für eine Wirkung gefunden. Die vier eingeschlossenen Studien (zwei mit Oxprenolol, zwei mit Propranolol) mit insgesamt 72 Pat. erfüllten zwar die Einschlusskriterien, wiesen jedoch gravierende methodische Mängel auf [29].

Begründung der Empfehlung

Die Evidenz für eine Wirkung von Propranolol auf den Parkinson-Tremor ist schwach, die klinische Erfahrung weist jedoch im Einzelfall auf eine Wirkung besonders bei ausgeprägtem Haltetremor hin. Aus den vorliegenden Studien zeigt sich ein Wirkeffekt sowohl auf die Ruhe- wie auch auf die Haltetremor-Amplitude durch nicht-kardioselektive Betablocker (i.e. Propranolol). Eine Gabe von Propranolol 20–80 mg/Tag zeigte bei Henderson, Yiannikas et al. eine gute Verträglichkeit und Reduktion sowohl der Ruhe- als auch der Haltetremor-Amplitude. Bei der Indikationsstellung müssen Kontraindikationen für Betablocker streng beachtet werden. Betablocker sollten unter Kenntnis der medikamentösen Wechselwirkungen und Anwendungsbeschränkungen angewendet werden und sollen nicht bei Pat. mit Asthma bronchiale, mit stark verlangsamter Herzfrequenz, mit akuter

Herzinsuffizienz oder mit einer bestimmten Störung der Erregungsleitung (AV-Block) verwendet werden.

**Empfehlung**
**Betablocker** können für die Therapie des posturalen Parkinson-Tremors erwogen werden (off-label).
Konsensstärke: 96%, starker Konsens

Primidon

Die Datenlage zu Primidon für die Behandlung des Parkinson-Tremors ist unzureichend. Aufgrund fehlender Evidenz kann daher keine Aussage hinsichtlich der Effektivität und Sicherheit von Primidon in der Behandlung des Parkinson-Tremors gegeben werden.

Begründung der Empfehlung

Die Datenlage zu Primidon für die Behandlung des Parkinson-Tremors ist unzureichend. Klinische Erfahrung zeigt keinerlei Hinweise für eine positive Wirkung.

**Empfehlung**
Auf den Einsatz von **Primidon** sollte in der Therapie des Parkinson-Tremors wegen unzureichender Datenlage verzichtet werden.
Konsensstärke: 93,3%, Konsens

Clozapin

Es liegen 2 randomisierte, kontrollierte Studien zur Wirkung von Clozapin auf den Parkinson-Tremor vor [30], [31]. Sie untersuchten in einem randomisierten, placebokontrollierten Setting den akuten Effekt von 12,5 mg Clozapin auf Ruhe- und Haltetremor (Abnahme des kumulativen Ruhe- und Haltetremor-Scores im UPDRS an der rechten oberen Extremität als Outcome-Parameter) bei 17 Parkinson-Pat. >12h nach Einnahme der letzten Parkinson-Medikation. Clozapin führte bis zu 5 Stunden nach Einnahme zu einer signifikant stärkeren Tremor-Reduktion, verglichen mit Placebo. 15/17 Pat. zeigten eine Besserung des Tremor-Scores > 50%. Die Besserung von Ruhe- und Haltetremor war vergleichbar.

Die 15 Pat. mit Ansprechen erhielten in einer anschließenden offenen Phase 25 bis 75 mg Clozapin täglich, aufgeteilt auf 2 Einzeldosen (mittlere Dosis 45 mg) über durchschnittlich 15,5 Monate. Clozapin führte in der offenen Phase weiterhin bei allen Pat. zu einer Besserung des Tremors. Bei keiner/keinem Pat. wurde über den Studienverlauf eine Toleranzentwicklung gegenüber dem Antitremor-Effekt von Clozapin beobachtet. Die sedierenden Nebenwirkungen besserten sich laut den Autoren/Autorinnen innerhalb einiger Wochen nach Therapiebeginn.

In einer weiteren randomisierten, kontrollierten, doppelblinden Crossover-Studie wurde die Wirkung von Clozapin mit der des Anticholinergikums Benztropin auf den Parkinson-Tremor verglichen [31]. Auf eine 6-wöchige Behandlungsphase mit Clozapin bzw. mit Benztropin folgten ein 2-wöchiges Washout und anschließend eine 6-wöchige Behandlung mit der jeweils anderen Substanz. Nach

anschließender Entblindung konnten sich die Pat. für eine offene Therapie mit Clozapin oder Benztropin entscheiden. Als primäre Outcome-Parameter dienten Item 16 der UPDRS und der Fahn Tolosa Marin Tremor Score. Nebenwirkungen wurden systematisch erfasst. Anfangsdosierungen und wöchentliche Dosissteigerungen betrugen 12,5 mg für Clozapin und 0,75 mg für Benztropin, die mögliche tägliche Maximaldosis von Clozapin lag bei 75 mg und von Benztropin bei 4,5 mg.

Die Tremor-Scores besserten sich unter beiden Medikamenten (mittlere Tagesdosis Clozapin von 39 mg und Benztropin 3,0 mg) vergleichbar um etwa ein Drittel. Ein geblindetes Videorating ergab die beste Tremorkontrolle bei 9/19 Pat. unter Clozapin, bei 9/19 Pat. unter Benztropin. Unter Benztropin wurden häufiger zumindest moderate Nebenwirkungen beobachtet. 4/19 Pat. entschieden sich im Verlauf für eine Open-label-Therapie mit Clozapin, 5/19 für Benztropin.

In einer größeren retrospektiven Studie von Trosch et al., die den Effekt von Clozapin beim M. Parkinson auf motorische Symptome einschließlich des Tremors sowie nicht motorische Symptome in einer multizentrischen Kohorte von 172 Pat. untersuchte, litten 106 der 172 Pat. auch unter einem Ruhetremor, bei 26 Pat. war der Tremor die Indikation für die Einstellung auf Clozapin. Clozapin führte (unter einer mittleren Erhaltungsdosis von 31mg/d) bei 42% der behandelten Pat. zu einer deutlichen Besserung, bei 16% zu einem Abklingen, bei 41% zu keiner Änderung des Tremors [32].

Zusätzlich wurde ein Effekt von Clozapin auf den Parkinson-Tremor in mehreren kleineren offenen Studien gezeigt [33], [34], [35], [36].

Begründung der Empfehlung

Clozapin führt im Placebo-Vergleich zu einer signifikanten Besserung des Parkinson-Ruhe- und -Haltetremors. In offenen Studien wurde auch eine Wirksamkeit auf den gegen andere Medikamente wie Anticholinergika refraktären Parkinson-Tremor gezeigt. In einer offenen Nachbeobachtung wurde keine Toleranzentwicklung über mehr als ein Jahr gefunden. Limitierend für den Einsatz von Clozapin ist das Risiko teils gravierender Nebenwirkungen [37]. Das dosisunabhängige Risiko für das Auftreten einer Agranulozytose liegt bei ca. 1% und ist am höchsten innerhalb der ersten 18 Therapiewochen. Weitere relevante Nebenwirkungen umfassen Sedierung, QT-Zeit-Verlängerung, vermehrten Speichelfluss, orthostatische Hypotension und Schwindel. Metabolische Nebenwirkungen (Gewichtszunahme, Hypercholesterinämie, Insulinresistenz, Diabetesentwicklung und vaskuläre Folgeerkrankungen), wie sie bei psychiatrischen Pat. häufiger auftreten, sind im Allgemeinen dosisabhängig und spielen daher für Parkinson-Pat. eine nachgeordnete Rolle, können im Einzelfall aber auch bei der PK therapielimitierend sein. Das Anfallsrisiko unter Clozapin ist ebenfalls dosisabhängig, das höchste Anfallsrisiko besteht bei rascher Aufdosierung und unter hohen Dosen.

Empfehlung
Der Einsatz von **Clozapin** zur Therapie des Parkinson-Tremors kann unter Berücksichtigung des Nebenwirkungsspektrums erwogen werden (off-label), wenn andere Medikamente nicht ausreichend wirksam oder kontraindiziert sind, eine chirurgische Parkinson-Therapie (noch) nicht gewünscht oder kontraindiziert ist und ein adäquates Nebenwirkungsmonitoring gewährleistet ist.
Konsensstärke: 96%, starker Konsens

Fragestellung 76: Unter welchen Umständen wird eine spezifische medikamentöse oder invasive Therapie notwendig?

Evidenzgrundlage

In dieser Literaturrecherche fanden sich:

N=3 randomisierte, kontrollierte Studien

N=2 Falldarstellungen und Reviews.

Nach Erstdiagnose sollte zunächst mit den Zielsymptomen wie Akinese und Rigor behandelt werden [9], [23], [18], [19], [38], in der Regel bessert sich darunter auch der Parkinson-Tremor entsprechend. Für die Auswahl der Medikamente zur Erstbehandlung der PK sei auf die entsprechenden Kapitel dieser Leitlinie verwiesen. In Fällen, in denen unter der Standardtherapie trotz Ansprechen der übrigen motorischen Symptome keine suffiziente Tremor-Suppression erreicht wird, kommen die aufgeführten, spezifisch auf den Tremor abzielenden Therapien wie Betablocker und in Ausnahmefällen Anticholinergika unter individueller Abwägung des Nebenwirkungsspektrums zum Einsatz.

Begründung der Empfehlung

Levodopa und Dopaminagonisten stellen die wirksamste medikamentöse Therapie der Symptome der PK und so auch des Parkinson-Tremors dar und werden in allen Stadien eingesetzt. Bei einer stadiengerechten medikamentösen Behandlung der PK mit den Zielsymptomen Akinese und Rigor eingesetzt, bessert sich meist äquivalent auch der Parkinson-Tremor. Zusätzlich stehen neben der medikamentösen Therapie auch effektive invasive Verfahren zur Behandlung des Parkinson-Tremors zur Verfügung (siehe Kapitel 3.22).

Empfehlung
Die Erstbehandlung von Parkinson-Pat. mit Tremor richtet sich zunächst nach den allgemeinen Therapieprinzipien. Wenn diese Medikamente anhand der Zielsymptome Akinese und Rigor ausdosiert sind, kommen die besonderen hier genannten Interventionen (THS, Pumpentherapien) in Betracht. Die Wahl der initialen Medikation hängt von klinischen Faktoren wie Alter, Komorbiditäten und Schwere der motorischen Symptome ab.
Konsensstärke: 100%, starker Konsens

Referenzen

1. Bhatia KP, Bain P, Bajaj N, Elble RJ, Hallett M, Louis ED, et al. Consensus Statement on the classification of tremors. from the task force on tremor of the International Parkinson and Movement Disorder Society. Mov Disord. 2018;33(1):75-87
2. Jankovic J. Parkinson's disease: clinical features and diagnosis. J Neurol Neurosurg Psychiatry. 2008;79(4):368-76
3. Deuschl G, Bain P, Brin M. Consensus statement of the Movement Disorder Society on Tremor. Ad Hoc Scientific Committee. Mov Disord. 1998;13 Suppl 3:2-23
4. Dirkx MF, Zach H, Bloem BR, Hallett M, Helmich RC. The nature of postural tremor in Parkinson disease. Neurology. 2018;90(13):e1095-e103

5. Raethjen J, Pohle S, Govindan RB, Morsnowski A, Wenzelburger R, Deuschl G. Parkinsonian action tremor: interference with object manipulation and lacking levodopa response. Exp Neurol. 2005;194(1):151-60
6. Belvisi D, Conte A, Cutrona C, Costanzo M, Ferrazzano G, Fabbrini G, Berardelli A. Re-emergent tremor in Parkinson's disease: the effect of dopaminergic treatment. Eur J Neurol. 2018;25(6):799-804
7. Management of Parkinson's disease: an evidence-based review. Mov Disord. 2002;17 Suppl 4:S1-166
8. Fahn S, Oakes D, Shoulson I, Kieburtz K, Rudolph A, Lang A, et al. Levodopa and the progression of Parkinson's disease. N Engl J Med. 2004;351(24):2498-508
9. Koller WC. Pharmacologic treatment of parkinsonian tremor. Arch Neurol. 1986;43(2):126-7
10. Tedeschi G, Sasso E, Marshall RW, Bonavita V. Tremor in Parkinson disease: acute response to oral levodopa. Ital J Neurol Sci. 1990;11(3):259-63
11. Hughes AJ, Lees AJ, Stern GM. Apomorphine in the diagnosis and treatment of parkinsonian tremor. Clin Neuropharmacol. 1990;13(4):312-7
12. Henderson JM, Yiannikas C, Morris JG, Einstein R, Jackson D, Byth K. Postural tremor of Parkinson's disease. Clin Neuropharmacol. 1994;17(3):277-85
13. Koller WC. Propranolol therapy for essential tremor of the head. Neurology. 1984;34(8):1077-9
14. Zach H, Dirkx MF, Roth D, Pasman JW, Bloem BR, Helmich RC. Dopamine-responsive and dopamine-resistant resting tremor in Parkinson disease. Neurology. 2020;95(11):e1461-e70
15. Stowe RL, Ives NJ, Clarke C, van Hilten J, Ferreira J, Hawker RJ, et al. Dopamine agonist therapy in early Parkinson's disease. Cochrane Database Syst Rev. 2008(2):Cd006564
16. Pogarell O, Gasser T, van Hilten JJ, Spieker S, Pollentier S, Meier D, Oertel WH. Pramipexole in patients with Parkinson's disease and marked drug resistant tremor: a randomised, double blind, placebo controlled multicentre study. J Neurol Neurosurg Psychiatry. 2002;72(6):713-20
17. Navan P, Findley LJ, Jeffs JA, Pearce RK, Bain PG. Randomized, double-blind, 3-month parallel study of the effects of pramipexole, pergolide, and placebo on Parkinsonian tremor. Mov Disord. 2003;18(11):1324-31
18. Schrag A, Keens J, Warner J. Ropinirole for the treatment of tremor in early Parkinson's disease. Eur J Neurol. 2002;9(3):253-7
19. Jankovic J, Tolosa E. Parkinson's disease and movement disorders: Lippincott Williams & Wilkins; 2007.
20. Ehrt U, Broich K, Larsen JP, Ballard C, Aarsland D. Use of drugs with anticholinergic effect and impact on cognition in Parkinson's disease: a cohort study. J Neurol Neurosurg Psychiatry. 2010;81(2):160-5
21. Taylor-Rowan M, Edwards S, Noel-Storr AH, McCleery J, Myint PK, Soiza R, et al. Anticholinergic burden (prognostic factor) for prediction of dementia or cognitive decline in older adults with no known cognitive syndrome. Cochrane Database Syst Rev. 2021;5(5):Cd013540
22. Woodford HJ, Stevenson JM. Anticholinergic drugs and dementia: time for transparency in the face of uncertainty. Cochrane Database Syst Rev. 2021;9(9):Ed000154
23. Schrag A, Schelosky L, Scholz U, Poewe W. Reduction of Parkinsonian signs in patients with Parkinson's disease by dopaminergic versus anticholinergic single-dose challenges. Mov Disord. 1999;14(2):252-5
24. Katzenschlager R, Sampaio C, Costa J, Lees A. Anticholinergics for symptomatic management of Parkinson's disease. Cochrane Database Syst Rev. 2003;2002(2):Cd003735
25. Sahoo LK, Holla VV, Batra D, Prasad S, Bhattacharya A, Kamble N, et al. Comparison of effectiveness of trihexyphenidyl and levodopa on motor symptoms in Parkinson's disease. J Neural Transm (Vienna). 2020;127(12):1599-606

26. Marsden CD, Parkes JD, Rees JE. Letter: Propranolol in Parkinson's disease. Lancet. 1974;2(7877):410
27. Foster NL, Newman RP, LeWitt PA, Gillespie MM, Larsen TA, Chase TN. Peripheral beta-adrenergic blockade treatment of parkinsonian tremor. Ann Neurol. 1984;16(4):505-8
28. Koller WC, Herbster G. Adjuvant therapy of parkinsonian tremor. Arch Neurol. 1987;44(9):921-3
29. Crosby NJ, Deane KH, Clarke CE. Beta-blocker therapy for tremor in Parkinson's disease. Cochrane Database Syst Rev. 2003;2003(1):Cd003361
30. Bonuccelli U, Ceravolo R, Salvetti S, D'Avino C, Del Dotto P, Rossi G, Murri L. Clozapine in Parkinson's disease tremor. Effects of acute and chronic administration. Neurology. 1997;49(6):1587-90
31. Friedman JH, Koller WC, Lannon MC, Busenbark K, Swanson-Hyland E, Smith D. Benztropine versus clozapine for the treatment of tremor in Parkinson's disease. Neurology. 1997;48(4):1077-81
32. Trosch RM, Friedman JH, Lannon MC, Pahwa R, Smith D, Seeberger LC, et al. Clozapine use in Parkinson's disease: a retrospective analysis of a large multicentered clinical experience. Mov Disord. 1998;13(3):377-82
33. Pakkenberg H, Pakkenberg B. Clozapine in the treatment of tremor. Acta Neurol Scand. 1986;73(3):295-7
34. Friedman JH, Lannon MC. Clozapine-responsive tremor in Parkinson's disease. Mov Disord. 1990;5(3):225-9
35. Jansen EN. Clozapine in the treatment of tremor in Parkinson's disease. Acta Neurol Scand. 1994;89(4):262-5
36. Low-dose clozapine for the treatment of drug-induced psychosis in Parkinson's disease. N Engl J Med. 1999;340(10):757-63
37. Yaw TK, Fox SH, Lang AE. Clozapine in Parkinsonian Rest Tremor: A Review of Outcomes, Adverse Reactions, and Possible Mechanisms of Action. Mov Disord Clin Pract. 2016;3(2):116-24
38. de Bie RMA, Clarke CE, Espay AJ, Fox SH, Lang AE. Initiation of pharmacological therapy in Parkinson's disease: when, why, and how. Lancet Neurol. 2020;19(5):452-61

3.7 Akinetische Krise/Entzugssyndrome

Autoren: Monika Pötter-Nerger, Matthias Löhle, Günter Höglinger

Fragestellung 77: Wie wird die Akinetische Krise bei der PK diagnostiziert?

Hintergrund

Die akinetische Krise ist klinisch definiert als eine akute, potenziell vital gefährdende Verschlechterung der Symptomatik bei Pat. mit einer PK, einhergehend mit einem variablen, transient doparesistenten Symptomkomplex mit ausgeprägter Akinese, Rigor, Dysphagie, vegetativer Begleitsymptomatik und Fieber [1], [2]. Die frühzeitige Diagnosestellung einer akinetischen Krise und die Abgrenzung von einer hypokinetischen Off-Fluktuation führen zu einer adäquaten Therapieadjustierung mit Vermeidung von Sekundärkomplikationen [3] und haben daher einen hohen Stellenwert. Somit stellt sich die Frage, wie eine akinetische Krise sicher und frühzeitig diagnostiziert werden kann.

Evidenzgrundlage

Die Literatursuche in Pubmed erbrachte 60 Studien zu den Begriffen „Parkinson disease AND akinetic crisis OR neuroleptic-like malignant syndrome“. Nach Screening der Abstracts und Inhalte wurden weitere Studien aus den Literaturreferenzen extrahiert und letztendlich 14 Studien selektiert, welche in diese Leitlinie einbezogen wurden. Es fanden sich keine randomisierten, kontrollierten Studien in dieser Literaturrecherche, aber zwei longitudinale größere Kohortenstudien sowie mehrere Falldarstellungen und Übersichtsartikel.

Ergebnis

Definition: Die akinetische Krise ist definiert als eine akute, potenziell vital gefährdende Verschlechterung der Symptomatik bei Pat. mit einer PK. Mit einer Inzidenz von 0.3% Parkinson-Pat./Jahr stellt sie ein relativ seltenes Ereignis dar [1], [2]. Engere Kriterien zur Definition der akinetischen Krise wurden als akute Verschlechterung der UPDRS >20 Punkte, begleitet von einer transienten Resistenz gegenüber einer dopaminergen Medikation über >3 Tage vorgeschlagen [4]. Die akute akinetische Krise wird häufig auch synonym als „Parkinson Hyperpyrexia Syndrom“, „malignes Syndrom“, „akute Akinesie“ oder „neuroleptic malignant-like syndrome“ bezeichnet, beruhend auf historisch beobachteten Ähnlichkeiten mit psychiatrischen Pat. unter hochdosierter, neuroleptischer Medikation, die Fieber und ein schweres Parkinson-Syndrom im Sinne eines „malignen, neuroleptischen Syndroms“ erleiden.

Risikofaktoren: Es fanden sich akinetische Krisen häufiger bei Pat. im fortgeschrittenen Erkrankungsstadium (Hoehn & Yahr>3), bei Vorliegen von Halluzinationen, Demenz und On-Off-Fluktuationen [2]. Des Weiteren scheinen genetisch bedingte Parkinson-Syndrome ein höheres Risiko für wiederholt auftretende, akinetische Krisen zu haben (2.12% Pat./Jahr), insbesondere Pat. mit genetischen Mutationen, die mit einer mitochondrialen Dysfunktion einhergehen wie *POLG*- oder *PINK1*-Mutationen [5]. Ein aktueller Übersichtsartikel auf Basis von 56 Ereignissen zwischen 1981 und 2022 beschreibt eine ausgeglichene Geschlechterverteilung (24 Frauen/56 Pat.) sowie eine variable Altersspanne (43–79 Jahre) und Erkrankungsdauer (1–25 Jahre) [3].

Auslösefaktoren: Als Auslösefaktoren einer akinetischen Krise gelten u.a. respiratorische oder urologische Infektionen, prämenstruelle Perioden, diabetische Entgleisungen mit Koma, Hyponatriämie [3], Traumata durch Stürze und Knochenbrüche, postoperative Komplikationen nach chirurgischen Eingriffen [1] oder akute Blutungsanämie [6], aber auch saisonal heißes Wetter [7] und Dehydrierung [2]. Als weitere Auslösefaktoren wurden Medikamenteneinnahmen von Antipyretika [8], Antiemetika oder Lithium [9] oder das abrupte Absetzen von Amantadin [10] diskutiert. Einen besonders häufigen Auslösefaktor für eine akinetische Krise stellen die Änderung oder das Auslassen der dopaminergen Medikation dar bzw. gastrointestinale Infektionen oder Ileus, welche die Resorption der dopaminergen Medikamente gefährden [1]. Auch die Einnahmen antidopaminerger Medikamente wie Risperidon, Amisulprid oder Antiemetika können akinetische Krisen auslösen [5].

Symptomatik: Die klinische Symptomatik trägt mit spezifischen Stigmata zur Diagnosestellung der akinetischen Krise bei. In einer japanischen multizentrischen Studie [2] wurden 72 Parkinson-Pat. und 21 Pat. mit atypischen Parkinson-Syndromen während einer akinetischen Krise beobachtet. Hohes Fieber war das am häufigsten mit motorischer Verschlechterung assoziierte Symptom, sodass die Autoren/Autorinnen das Vorliegen einer erhöhten Körperkerntemperatur als wichtigstes diagnostisches Kriterium einer akinetischen Krise erachteten. Weiterhin fanden sich in etwa der Hälfte der Pat. eine Bewusstseinsstörung, ein Verwirrtheitszustand, Appetitverlust, Dysphagie, allgemeines Krankheitsgefühl und autonome Symptome wie vermehrtes Schwitzen, Hypersalivation, Blutdruckschwankungen, selten zeigte sich ein Myoklonus [2]. In weiteren Fallbeschreibungen fanden sich ein verstärkter Tremor oder dystone Symptome [3].

Verlauf: Der klinische Verlauf kann zur Diagnosestellung einer akinetischen Krise beitragen. Die akinetische Krise ist gekennzeichnet durch eine akute Verschlechterung der motorischen Symptome, die in einer beobachteten Kohorte 20–31 Punkte auf der UPDRS-Skala betrug mit einer mittleren Verschlechterung im Hoehn-&-Yahr-Stadium von 2 auf 4–5 [1]. Die Parkinson-Symptomatik verschlechterte sich innerhalb von 2–3 Tagen, hielt im Schnitt 11 Tage an und zeigte eine Erholung nach 4–26 Tagen [1].

Labor: Im Labor fanden sich bei 80–100% der Pat. mit akinetischer Krise eine erhöhte Creatinkinase (CK) sowie erhöhtes Myoglobin im Serum (80%) und im Urin [2]. Weiterhin sind erhöhter Harnstoff, Leberwerte und LDH, erhöhte Leukozyten und CRP auch ohne Vorliegen einer simultanen Infektion [11] beschrieben.

Zusatzdiagnostik: Weitere apparative Zusatzdiagnostik sollte durchgeführt werden zum Ausschluss von Differenzialdiagnosen. Elektrophysiologisch fand sich im EEG in unter der Hälfte der Parkinson-Pat. eine Allgemeinveränderung [2]. Eine zerebrale Erregbarkeitssteigerung im Sinne eines non-konvulsiven Status als Ursache der Bewusstseinsstörung sollte als konkurrierende Ursache ausgeschlossen werden. Andere symptomatische Ursachen sollten laborchemisch ausgeschlossen werden wie z.B. eine thyreotoxische Krise, autoimmune Enzephalitis oder auch bildgebend wie z.B. ein bilateraler zerebraler Arteria-cerebri-anterior-Infarkt. Passend zu der klinischen Beobachtung zeigte sich in zwei kleineren Fallserien in seriellen DATScans während und nach der akinetischen Krise [12], [13] eine transiente, fast vollständig aufgehobene, striatale, präsynaptische Dopamintransporter-Bindung, die sich partiell nach der Krise wieder normalisierte. Dieser DATScan-

Befund wurde als so charakteristisch eingeordnet, dass von den Autoren/Autorinnen vorgeschlagen wurde, die Durchführung eines Dopamintransporter-SPECTs während der akinetischen Krise zur Diagnosestellung zu nutzen [13]. In der klinischen Praxis kann bei geeigneten technischen Voraussetzungen und Verfügbarkeit in der jeweiligen Klinik der Dopamintransporter-SPECT ergänzt werden.

Differenzialdiagnosen: Besonders relevant ist die diagnostische Abgrenzung einer akinetischen Krise von ausgeprägten hypokinetischen Wirkfluktuationen, die bei fortgeschrittenen Parkinson-Pat. häufig zu beobachten sind. Das wesentliche Unterscheidungsmerkmal ist die gute Responsivität der Akinese auf die wiederaufgenommene dopaminerge Medikation bei Wirkfluktuationen. Die hypokinetische Fluktuation kann effektiv durch Applikation einer adäquaten dopaminergen Medikation beendet werden, die akinetische Krise in der Akutphase nicht unmittelbar. Diese transiente, fehlende Dopa-Responsivität in der akinetischen Krise, die über 1–2 Wochen anhalten kann, wurde klinisch belegt durch Beobachtungen in Fallserien [14], [11] und einer italienischen Kohorte [1]. Es konnten Pat.-Gruppen identifiziert werden, in denen keine vorangegangene Änderung der Parkinson-Medikation vor der akinetischen Krise nachzuweisen war, mit konstanten L-Dopa-Plasmaspiegeln vor und während der akinetischen Episode [1]. Zusammenfassend ist die transiente Levodopa-Resistenz als ein Kernkriterium der Diagnosestellung einer akinetischen Krise zu sehen [1].

Begründung der Empfehlung

Auf der Basis der oben dargestellten Evidenzgrundlage wird zur Diagnosestellung einer akinetischen Krise bei Parkinson-Pat. die u.g. Empfehlung gegeben.

Empfehlung	Neu<br>Stand (2023)
Zur Diagnosestellung einer akinetischen Krise können folgende Kriterien herangezogen werden:<br>1. Vorliegen von Risikofaktoren als Auslöser, wie z.B. Operationen, Infektionen, saisonale Faktoren, Auslassen dopaminerger Medikamente oder Einnahme ungünstiger Medikamente<br>2. die klinische Symptomatik in Form einer akuten Verschlechterung der Symptomatik, bestehend aus Fieber und ausgeprägter Akinese, Rigor, Bewusstseinsstörung, Dysphagie, vegetativer Begleitsymptomatik, ggf. Myoklonus oder Dystonie<br>3. der klinische Verlauf mit einer (teils nur partiellen) Restitution nach 2–4 Wochen und hoher Letalität von 4–23%<br>4. laborchemisch erhöhte CK- und Myoglobin-Werte<br>5. Ausschluss konkurrierender Ursachen wie z.B. serotonerges Syndrom, intrakranielle Infektion, Intoxikationen z.B. mit Neuroleptika, Sepsis oder thyreotoxische Krise<br>6. das Vorliegen einer transienten Dopa-Resistenz der ausgeprägten Parkinson-Symptomatik<br>7. eine massiv reduzierte Belegung im Dopamintransporter-SPECT in der akuten Phase.	
Konsensstärke: 100%, starker Konsens	

Fragestellung 78: Wie wird die akinetische Krise bei der PK therapiert?

Hintergrund

Die Behandlung der Parkinson-Pat. während der akinetischen Krise stellt eine besondere Herausforderung dar [3]. Durch die ausgeprägte Dysphagie und gastrointestinale Motilitätsstörung ist die perorale Applikation der Medikamente eingeschränkt. Die transiente Levodopa-Resistenz begrenzt die Möglichkeiten der klassischen, dopaminergen Behandlungsansätze. Die Immobilität führt zu einer hohen Komplikationsrate mit intensivmedizinischem Behandlungsbedarf und hoher Letalität. Die adjustierte und frühzeitige Therapie der akinetischen Krise ist daher entscheidend für die Prognose.

Evidenzgrundlage

Nach der Literatursuche mit den Begriffen „Parkinson disease AND akinetic crisis OR neuroleptic-like malignant syndrome“ wurden nach Screening zu Angaben einer Therapie 23 Studien identifiziert, welche ausgewertet wurden. Da die akinetische Krise ein seltenes Ereignis ist, gibt es derzeit keine systematischen, randomisierten, kontrollierten Studien und somit keine validierte Evidenz zur optimalen Therapie in der akinetischen Krise. Derzeit beruhen die Empfehlungen zur Therapie auf Kohorten- und Fallbeobachtungen und pathophysiologischen Überlegungen im Sinne einer Expertenmeinung.

Ergebnis

Die Therapie basiert auf verschiedenen supportiven, dopaminergen und nicht dopaminergen Ansätzen und Behandlung von Komplikationen. Wichtig ist der frühe Beginn einer adäquaten Therapie, idealerweise unter intensivmedizinischen Bedingungen, um die Prognose zu verbessern.

Auslösefaktoren: Im ersten Schritt sollte eine Behandlung potenzieller Auslösefaktoren der akinetischen Krise erfolgen. Eine Pneumonie oder ein Harnwegsinfekt sollten antibiotisch therapiert werden, entgleiste Elektrolyte und Blutzuckerwerte sollten korrigiert werden, auslösende Medikamente wie Neuroleptika, Antiemetika sollten abgesetzt werden.

Supportive therapeutische Maßnahmen: Im zweiten Schritt sollten adäquate, supportive therapeutische Maßnahmen ergriffen werden. Vitalfunktionen sollten regelmäßig kontrolliert, fiebersenkende physikalische und ggf. medikamentöse Maßnahmen sollten angewendet werden. Eine Thromboseprophylaxe und ausreichende, intravenöse Flüssigkeitsgabe sollten gewährleistet sein.

Komplikationen: In der akinetischen Krise können Komplikationen auftreten, die die Prognose beeinträchtigen und daher verhindert bzw. frühzeitig therapiert werden müssen. Beschriebene Komplikationen sind Rhabdomyolysen [15], Aspirationspneumonien (19.2%), disseminierte intravasale Gerinnungsstörungen (8.1%) und akutes Nierenversagen (5.1%) [2]. Weitere Komplikationen können Thrombozytopenien, respiratorisches Versagen [16], sehr selten epileptische Anfälle, akutes Leberversagen, akute kardiale Dekompensation (<2%) [2] oder kardiale Arrhythmien darstellen. Des Weiteren ist eine „Critical Illness Neuromyopathy“ mit schlaffer Tetraparese und erneutem Anstieg von Muskelenzymen als Komplikation einer akinetischen Krise beschrieben

worden [17]. Bei Auftreten von Komplikationen sollten die bereits beschriebenen, supportiven Maßnahmen um intensivmedizinische Therapieansätze und Behandlung der Komplikationen wie eine invasive Beatmung, antiepileptische Therapien oder Dialyse ergänzt werden.

Dopaminerge Medikation: Als weitere wichtige Therapiesäule sollten die Wiederaufnahme und die Adjustierung der dopaminergen Medikation erfolgen. Bei ausgeprägter Dysphagie werden zur Sicherstellung einer effektiven Medikationszufuhr parenterale Applikationswege oder die Anwendung einer nasogastralen Sonde oder die Nutzung einer PEG-Sonde empfohlen [3]. Zu berücksichtigen ist eine transiente Levodopa-Resistenz, die zu einer adäquaten Dosisanpassung führen sollte. Verschiedene Substanzen werden hier verwendet.

- Das am häufigsten verwendete Medikament in der akinetischen Krise ist das Levodopa. Bei der häufig auftretenden Dysphagie sollte frühzeitig eine nasogastrale Sonde zur Levodopa-Applikation verwendet werden oder eine bereits gelegte PEG-Sonde genutzt werden. Zu berücksichtigen ist zum einen die Interferenz der dopaminergen Medikation mit der gastroenteralen Ernährung, denn eine parallele Applikation führt zu einem Wirkverlust des Levodopa [18]. Eine Möglichkeit ist die vorwiegende Applikation der Sondennahrung nachts und der dopaminergen Medikation tagsüber. Eine andere Möglichkeit ist das Ausstellen der Sondennahrung 1h vor und nach der Levodopa-Applikation. Darüber hinaus wird diskutiert, Sondenkost mit geringerem Proteingehalt mit einer Begrenzung des Proteingehalts auf 0.8 g/kg/Tag zu wählen [19]. Zum Zweiten sollte beachtet werden, dass nur bestimmte Levodopa-Präparate sondengängig sind. Lösliches Levodopa, aufgelöst in Wasser, kann sicher über die Sonde appliziert werden. Zum Dritten ist die Dosis des löslichen Levodopa zu berücksichtigen. Die Parkinson-Medikation des Pat. vor Beginn der akinetischen Krise sollte umgerechnet werden in die entsprechende Levodopa-Äquivalenzdosis (LEDD-Umrechnungstabelle siehe [20]) und eine weitere Dosiserhöhung unter Annahme der transienten Reduktion der Dopa-Sensitivität und Titration nach klinischen Symptomen durchgeführt werden. In speziellen, therapieresistenten Fällen kann ein nasogastrale Infusion mit Levodopa-Carbidopa-intestinalem Gel (LCIG) oder Levodopa/Entacapon/Carbidopa-intestinalem Gel (LECIG) in Erwägung gezogen werden [21], [67]. Diese Alternative bietet sich insbesondere an, wenn aufgrund einer anhaltenden, schweren Dysphagie bereits die Indikation für eine PEG-Anlage besteht oder wenn perspektivisch auch nach der akuten Krise bei Wirkfluktuationen eine LCIG-Therapie sinnvoll sein könnte. Hier sollte bei Legen der PEG ein ausreichend großer Zugang gewählt werden (20/9 Charrière).
- Ergänzend oder alternativ zu Levodopa können verschiedene Dopaminagonisten in der Therapie der akinetischen Krise eingesetzt werden. Apomorphin ist ein schnell wirksamer Dopaminagonist, der über intermittierende Applikationen in Form von subkutanen Pen-Injektionen oder sublingual eingelegten Apomorphin-Strips verabreicht werden kann oder kontinuierlich angewendet werden kann in Form einer Pumpenbehandlung. Fallbeschreibungen haben gute Effekte von Apomorphin-Applikationen nachweisen können [22], [23], entweder als Monotherapie oder als Kombinationstherapie mit Levodopa mit einer Restitution der Symptome innerhalb von 24 bis 48 h. In der Literatur verwendete Apomorphin-Dosen sind sehr variabel und liegen zwischen 0.7 und 8 mg/h in Form einer subkutanen kontinuierlichen Infusionsbehandlung und sollten symptomorientiert angepasst werden [22], [23]. Es ist jedoch zu berücksichtigen,

dass in der akuten Krise auch gegen Apomorphin eine transiente, fehlende Responsivität bestehen kann [22]. So wurde in einer Kohorte von 16 Parkinson-Pat. in einer akinetischen Krise beschrieben, dass trotz Apomorphin-Therapie in hohen Dosen (150–200 mg/Tag) zunächst über 2–10 Tage keine Besserung des UPDRS zu beobachten war und trotz Therapie 4 Pat. verstarben [24]. In einer anderen Kohorte wurde trotz Apomorphin eine Letalität von 15% festgestellt [25]. Apomorphin sollte wegen der häufigen Nebenwirkung Nausea mit Domperidon kombiniert werden, welches auch rektal appliziert werden kann [26]. Eine seltene Komplikation sind Hämolysen unter Apomorphin, hier muss die Therapie gestoppt werden.

- In einzelnen Fällen wurde der Einsatz einer transdermalen Applikation des Non-Ergot-Dopaminagonisten Rotigotin in der akinetischen Krise beschrieben [27], [28], [29]. Rotigotin wirkt über eine Stimulation von D3>D2>D1-Rezeptoren. Es wird als Pflaster über 24 h appliziert, welches täglich gewechselt wird. Es wurde in den beschriebenen Fällen mit einem 2mg/24h-Pflaster gestartet, welches rasch innerhalb von 4 Tagen auf 6mg/24h erhöht wurde [27], [28]. Bei guter Verträglichkeit zeigte sich in den beschriebenen Fällen eine gute Wirksamkeit mit einer Reduktion der Symptome um etwa 50% nach 2 Wochen und der Möglichkeit der Wiederaufnahme einer oralen Medikation.

Nicht-dopaminerge Therapie: Einen weiteren Therapieansatz kann die Anwendung nicht dopaminerger Substanzen darstellen.

- **Amantadin** hat insbesondere als intravenöse Applikation von Amantadin-Sulfat in der Behandlung der akinetischen Krise einen hohen Stellenwert [30], [31], [32], [33]. Der Wirkmechanismus ist vorwiegend antiglutamaterg über NMDA-Rezeptoren, anticholinerg und schwach dopaminerg [32]. Amantadin erscheint insbesondere aufgrund der NMDA-antagonistischen Wirkung vorteilhaft in der Levodopa-resistenten Phase der akinetischen Krise, da hier ein Ansprechen auf NMDA-Antagonisten unter der pathophysiologischen Annahme einer gesteigerten, exzitatorischen, glutamatergen Aktivität stets vorhanden sein kann [32]. Amantadin-Dosen von 200 mg bis 600 mg können angewendet werden. Nebenwirkungen wie Delir, Verwirrtheitszustände und Nierenversagen müssen berücksichtigt werden. Bei geriatrischen Pat. muss der Einsatz von Amantadin daher besonders kritisch abgewogen werden.
- Die intravenöse Applikation von **Dantrolen** wurde zur Muskelrelaxation bei ausgeprägtem Rigor in einigen Pat. während der akinetischen Krise eingesetzt. Dantrolen reduziert den Muskeltonus über die Blockade der Kalzium-Ausschüttung aus dem sarkoplasmatischen Retikulum [25]. Es wurden symptomorientierte Dosierungen von 1 mg/kg bis maximal 10 mg/kg verwendet. Als Nebenwirkungen sind Hautnekrosen bei Paravasaten beschrieben [25]. In deutschen Zentren ist der Einsatz dieses Medikaments jedoch unüblich.
- Alternativ kann der Einsatz von Benzodiazepinen wie **Diazepam** zur Reduktion des Muskeltonus erwogen werden.

Prognose: Die Prognose kann durch eine frühzeitige und adäquate Therapie der akinetischen Krise deutlich verbessert werden. In einer japanischen Kohorte konnte bei Parkinson-Pat. in 68.7% eine komplette Remission erzielt werden, jedoch zeigten sich in 31.3% der Pat. eine permanente, residuelle Verschlechterung und eine Letalität von 4% [2]. In anderen Kohorten wurde eine Letalität von 23% [5] oder 21.4% [3] beobachtet. Gründe für die erhöhte Mortalität waren hyperthermes

Koma, respiratorisches Versagen, Nierenversagen, Herzversagen, disseminierte, intravasale Gerinnung, septischer Schock in vor allem älteren, länger erkrankten Parkinson-Pat. [3]. Prognostisch ungünstige Faktoren für einen schlechten Verlauf nach der Krise werden in der Literatur unterschiedlich gewertet. In einer italienischen Kohorte war allein die Dauer der akinetischen Krise prädiktiv für den Verlauf. Je mehr Tage die akinetische Krise dauerte, desto höher war die Wahrscheinlichkeit eines letalen Verlaufs [1], [5]. In anderen Kohorten waren höheres Lebensalter und eine stärker ausgeprägte Akinese vor der akinetischen Krise [2], [34], eine stärkere motorische Beeinträchtigung, gemessen im Hoehn-&-Yahr-Stadium während der akinetischen Krise [2] und das Vorhandensein von Komplikationen wie akutem Nierenversagen oder DIC [2] prädiktiv für einen schlechteren Verlauf nach der akuten Krise.

Begründung der Empfehlung

Die Evidenz beruht auf Beobachtungsstudien sowie Fallberichten oder Fallserien ohne randomisierte, kontrollierte Studien. Therapeutische Ansätze können aus diesen vorliegenden Studien herangezogen und auf mittlerer Evidenzbasis empfohlen werden.

**Empfehlung**	**Neu**<br>**Stand (2023)**
1. Die adäquate Therapie solle frühzeitig und suffizient idealerweise auf einer Überwachungsstation oder Intensivstation erfolgen, insbesondere, wenn sich Komplikationen entwickeln.<br>2. Risikofaktoren, die eine akinetische Krise bedingen können, wie z.B. Infektionen sollen unverzüglich behandelt werden.<br>3. Supportive Therapieansätze wie Flüssigkeitszufuhr, Thromboseprophylaxe, fiebersenkende Maßnahmen und regelmäßige Kontrolle der Vitalfunktionen sollen erfolgen.<br>4. Eine dopaminerge Medikation in Form von löslichem Levodopa über eine nasogastrale Magensonde, subkutaner oder sublingualer Applikation von Apomorphin oder transdermalem Rotigotin soll sichergestellt werden.<br>5. Nicht dopaminerge Medikamente wie intravenöses Amantadinsulfat sollten in Erwägung gezogen werden oder in spezifischen Fällen Benzodiazepine.	
Konsensstärke: 93,3%, Konsens	

Wichtige Forschungsfragen: Randomisierte kontrollierte Studien zur Wirksamkeit und Sicherheit therapeutischer Optionen bei der akinetischen Krise von Parkinson-Pat. fehlen bislang vollständig.

Fragestellung 79: Wie wird das Dopaminagonisten-Entzugssyndrom bei der PK diagnostiziert?

Hintergrund

Das Dopaminagonisten-Entzugssyndrom (engl. dopamine agonist withdrawal syndrome, Abk. DAWS) wurde erstmals im Jahr 2010 von Rabinak & Nirenberg beschrieben [35]. Es handelt sich hierbei um

ein stereotypes Cluster aus psychiatrischen, autonomen und sensorischen Symptomen, die im zeitlichen Zusammenhang mit einer Reduktion oder dem Absetzen von Dopaminagonisten auftreten und denjenigen Symptomen ähneln, die auch beim Entzug von psychostimulativen Substanzen beobachtet werden [35]. Der Literatur nach kann davon ausgegangen werden, dass etwa 15–24% der Parkinson-Pat., bei denen die Dosis von Dopaminagonisten reduziert oder die Behandlung mit Dopaminagonisten beendet wird, ein DAWS erleiden [35], [36], [37], [38], [39]. Angesichts der Häufigkeit des DAWS und dessen weitreichender Konsequenzen [40], die im Einzelfall bis zu Suizidalität reichen können, ist es notwendig, das DAWS rechtzeitig zu erkennen und zu diagnostizieren, um Betroffene adäquat unterstützen zu können.

Evidenzgrundlage

Die Literaturrecherche in Pubmed mit dem Suchbegriff „dopamine agonist withdrawal syndrome“ AND „Parkinson's disease“ erbrachte insgesamt 28 Resultate. Hiervon wurden nach Durchsicht des Inhalts und unter Einbeziehung von Querverweisen 13 Literaturreferenzen einbezogen. Es fanden sich keine randomisierten, kontrollierten Studien und keine größeren longitudinalen Kohortenstudien bei der Literaturrecherche, die Evidenz beschränkt sich auf eine kleine prospektive Kohortenstudie, mehrere retrospektive Kohortenstudien, mehrere Falldarstellungen und Übersichtsartikel.

Ergebnis

Definition: Das DAWS wurde von den Erstbeschreibern definiert als schweres, stereotypes Cluster von physischen und psychologischen Symptomen, die mit dem Entzug von Dopaminagonisten in dosisabhängiger Beziehung korrelieren, klinisch signifikante Beeinträchtigungen oder soziale/berufliche Funktionsstörungen verursachen, nicht auf Levodopa und andere Parkinson-Medikamente ansprechen und nicht durch andere klinische Faktoren erklärt werden können [35]. Diese manchmal auch als „Rabinak-Nirenberg-Kriterien“ bezeichnete Definition wurde bislang nicht validiert, wurde aber von allen wesentlichen Kohortenstudien zum DAWS verwendet und hat sich in der Praxis bewährt [41], [36], [37], [38], [39].

Risikofaktoren: Bereits in der Erstbeschreibung des DAWS wurde das Vorhandensein von **Impulskontrollstörungen** als entscheidender Risikofaktor für dessen Auftreten identifiziert [35], was in späteren Studien bestätigt werden konnte [41], [36], [37], [38], [39]. Während zunächst noch davon ausgegangen wurde, dass das DAWS nahezu ausschließlich bei Pat. auftritt, bei denen Dopaminagonisten aufgrund von vorherigen Impulskontrollstörungen abgesetzt werden mussten [35], [37], konnte im Verlauf jedoch gezeigt werden, dass das DAWS auch beobachtet werden kann, wenn Dopaminagonisten aufgrund von anderen Nebenwirkungen, wie z.B. Halluzinationen, reduziert werden müssen [36], [38] und wenn diese im Zusammenhang mit einer Tiefen Hirnstimulation [42] oder einem Wechsel auf Levodopa/Carbidopa-Intestinalgel [43] abgesetzt werden. Als weitere Risikofaktoren für das DAWS wurden **höhere Tagesdosen von Dopaminagonisten** [35], [41], [36], [39] sowie **höhere kumulative Dosen von Dopaminagonisten** [35] berichtet. Zuletzt wurden neben Impulskontrollstörungen und einer höheren Tagesdosis von Dopaminagonisten zudem auch eine **vorherige Tiefe Hirnstimulation** als Risikofaktor für das DAWS identifiziert [39]. In der gleichen Studie wurde berichtet, dass sich das individuelle Risiko für ein DAWS mit Kenntnis von drei Risikofaktoren

(Vorhandensein von Impulskontrollstörungen, Tagesdosis des Dopaminagonisten ≥ 150 mg Levodopa-Äquivalent, vorherige Tiefe Hirnstimulation) abschätzen lässt. So entwickelten 92% der Pat. mit allen 3 Risikofaktoren ein DAWS nach Absetzen von Dopaminagonisten, während die Wahrscheinlichkeit für das Auftreten eines DAWS bei Abwesenheit aller drei Risikofaktoren nur 3% betrug [39].

Symptomatik: Im Rahmen des DAWS treten Symptome auf, die auch beim Entzug von anderen psychostimulativen Substanzen beobachtet werden können. Im Vordergrund stehen hierbei **psychiatrische Symptome** (Angst, Panikattacken, Dysphorie, Depression, Agitation, Schlafstörungen, Reizbarkeit, Fatigue, Suchtverlangen), **autonome Symptome** (orthostatische Hypotension, Schwitzen, Hitzewallungen, Übelkeit, Schwindel) sowie **generalisierte Schmerzen** [35], [42], [37], [40], [43]. Diese Symptome treten nicht isoliert, sondern als Kombination in Erscheinung, wobei bei den meisten Pat. mit DAWS mindestens 4 der o.g. Symptome beobachtet werden können [37]. In der bislang einzigen prospektiven Studie wurden Angst (91,7%), Schmerzen (50%), Schwitzen (41,7%) und Anhedonie (16,7%) als häufigste Symptome des DAWS beschrieben [38]. Ein wesentliches Kennzeichen der Symptome des DAWS ist hierbei, dass diese nicht auf Levodopa, Antidepressiva, Benzodiazepine oder kognitive Verhaltenstherapie ansprechen, sich aber bei Wiedereinführung von Dopaminagonisten dramatisch verbessern [35]. Nicht unerwähnt bleiben darf, dass in der Literatur auch mehrere Fallberichte über Suizide existieren, die in einem vermuteten oder möglichen kausalen Zusammenhang mit dem Auftreten eines DAWS standen [44], [45], [46], [47], [48]. Es ist daher aus Sicht der Autoren/Autorinnen besonders wichtig, im Falle des Auftretens eines DAWS aktiv nach Suizidalität zu fragen, um gefährdete Pat. rechtzeitig zu identifizieren.

Begründung der Empfehlung

Auf der Basis der oben dargestellten Evidenzgrundlage hat sich die Leitliniengruppe entschlossen, eine schwache Empfehlung zur Diagnosestellung eines Dopaminagonisten-Entzugssyndroms bei Parkinson-Pat. zu geben.

Empfehlung	Neu<br>Stand (2023)
Zur Diagnosestellung eines Dopaminagonisten-Entzugssyndroms bei Parkinson-Pat. können folgende Kriterien herangezogen werden:<br>1. Vorliegen eines schweren, stereotypen Clusters physischer und/oder psychologischer Symptome (vor allem Angst, Schmerzen, Schwitzen und Anhedonie)<br>2. Auftreten der Symptome korreliert mit dem Entzug von Dopaminagonisten in dosisabhängiger Weise.<br>3. Symptome verursachen klinisch signifikante Beeinträchtigungen oder soziale/berufliche Funktionsstörungen.<br>4. Symptome sprechen nicht auf Levodopa und andere Parkinsonmedikamente (außer Dopaminagonisten) an.<br>5. Symptome können nicht durch andere klinische Faktoren erklärt werden.	
Konsensstärke: 96,4%, starker Konsens	

Fragestellung 80: Wie wird das Dopaminagonisten-Entzugssyndrom bei der PK therapiert?

Hintergrund

Das Dopaminagonisten-Entzugssyndrom (engl. dopamine agonist withdrawal syndrome, Abk. DAWS) hat weitreichende Konsequenzen für betroffene Pat. und stellt eine Herausforderung für behandelnde Ärzte/Ärztinnen dar [40]. Bislang steht leider keine spezifische Therapie zur Verfügung. Umso wichtiger ist die Kenntnis über die begrenzten Interventionsmöglichkeiten und den Verlauf der DAWS, um Pat. hinreichend aufklären zu können und besonders schwer betroffene Pat. einer engmaschigen Begleitung zuzuführen.

Evidenzgrundlage

Die Literaturrecherche in Pubmed mit dem Suchbegriff „dopamine agonist withdrawal syndrome“ AND „Parkinson's disease“ erbrachte insgesamt 28 Resultate. Hiervon wurden nach Durchsicht des Inhalts 9 Literaturreferenzen einbezogen. Es fanden sich keine randomisierten, kontrollierten Studien und keine größeren longitudinalen Kohortenstudien bei der Literaturrecherche, die Evidenz beschränkt sich auf wenige Falldarstellungen und Übersichtsartikel.

Ergebnis

Therapie: Bislang steht keine spezifische Therapie zur Behandlung des DAWS zur Verfügung [40]. Levodopa und andere dopaminerge Medikamente (außer Dopaminagonisten) sind gegen Symptome des DAWS unwirksam [37], [40], [43], [49]. Auch wenn bislang keine kontrollierten Studien zur Therapie des DAWS vorliegen, lässt sich aus vorhandenen Falldarstellungen ableiten, dass Antidepressiva, Anxiolytika, Antiepileptika, Opiate und kognitive Verhaltenstherapie keinen therapeutischen Nutzen beim DAWS haben [35], [50]. Einzig der Wiederbeginn mit Dopaminagonisten führt zur Besserung der Entzugssymptomatik, was im Einzelfall bei Pat. mit schwerem DAWS oder Suizidalität infolge des DAWS nach Meinung der Leitlinienautoren auch erwogen werden sollte. Da es nach Wiedereinführung des Dopaminagonisten aber auch bei niedrigen Dosierungen häufig zum Wiederauftreten von Impulskontrollstörungen kommt, ist ein engmaschiges Monitoring bei diesen Pat. notwendig [40].

Angesichts des Fehlens einer spezifischen Therapie wird in mehreren Publikationen empfohlen, Parkinson-Pat. und deren Angehörige vor Beginn einer Behandlung mit Dopaminagonisten als auch vor dem Absetzen über das Risiko eines DAWS und dessen Symptome aufzuklären [40], [49], [38]. Auch wenn retrospektive Studien zeigen, dass das Risiko eines DAWS nicht von der Geschwindigkeit des Abdosierens des Dopaminagonisten abhängt [39], scheint es zudem ratsam, Dopaminagonisten langsam auszuschleichen, insbesondere dann, wenn zuvor höhere Dosierungen zum Einsatz kamen [43], [49], [51]. Für die einzelnen Dopaminagonisten wurden in einer kürzlichen Expertenmeinung folgende Raten zur Dosisreduktion als sinnvoll erachtet: Piribedil 50 mg/d jeden 3. Tag, Pramipexol (Salzform) 0,375 mg/d umtägig, Ropinirol 2 mg/d umtägig, Rotigotin 2 mg/d umtägig, Apomorphin s.c. Reduktion der Pumpenlaufrate um 0,5 mg/h umtägig [51]. Insbesondere bei Pat., die mehrere Risikofaktoren für ein DAWS aufweisen (siehe Fragestellung 79), kann die Reduktion des Dopaminagonisten auch noch langsamer vorgenommen werden.

Prognose: Die Dauer und Langzeitprognose des DAWS ist sehr variabel [40]. Es kann jedoch davon ausgegangen werden, dass sich das DAWS bei etwa der Hälfte der Pat. innerhalb von 6 Monaten spontan zurückbildet, während bei anderen Pat. langfristige Verläufe bis zu mehreren Jahren auftreten [35], [37]. In einigen Fällen ist es infolge der Schwere des DAWS unabdingbar, den Dopaminagonisten wieder anzusetzen, was zwar zu einer Besserung der Entzugssymptomatik führt, aber andererseits eine Rückkehr und Chronifizierung von Impulskontrollstörungen mit sich bringen kann [35], [37].

Begründung der Empfehlung

Die Evidenz beruht auf retrospektiven Beobachtungsstudien sowie Fallberichten. Eine ausreichende Evidenz für eine Empfehlung zur spezifischen Behandlung des DAWS liegt nicht vor, sodass sich die Leitliniengruppe entschlossen hat, keine Empfehlung zur spezifischen Therapie des DAWS zu geben.

**Empfehlung**	**Neu**<br>**Stand (2023)**
1. Gegenwärtig kann aufgrund mangelnder Evidenz keine Empfehlung zur spezifischen Therapie des Dopaminagonisten-Entzugssyndroms gegeben werden.<br>2. Das Absetzen von Dopaminagonisten sollte langsam erfolgen, um Pat. mit Dopaminagonisten-Entzugssyndromen frühzeitig identifizieren zu können.<br>3. Bei Pat. mit schwerem, protrahiertem Dopaminagonisten-Entzugssyndrom sollte eine Wiederaufnahme der Behandlung mit Dopaminagonisten erwogen werden.	
Konsensstärke: 100%, starker Konsens	

Wichtige Forschungsfragen:

Randomisierte kontrollierte Studien zur Wirksamkeit und Sicherheit therapeutischer Optionen beim DAWS fehlen bislang vollständig.

Fragestellung 81: Wie wird das Tiefe-Hirnstimulations-Entzugssyndrom (THS-ES) bei der PK diagnostiziert?

Hintergrund

In der neueren Zeit ist der Begriff des „Tiefe Hirnstimulations-Entzugssyndroms“ (THS-ES) in der Literatur verwendet worden oder auch „DBS Withdrawal Syndrome“ [52]. Es handelt sich um ein seltenes Ereignis bei Parkinson-Pat. mit langjährig implantiertem THS-System, bei denen eine akute, transient Levodopa-resistente Verschlechterung der Motorik mit vegetativen Begleitsymptomen, Bewusstseinsstörungen und laborchemischem Anstieg der CK im Rahmen eines unerwarteten Ausfalls des THS-Systems auftritt. Die klinischen Merkmale des THS-ES ähneln denen einer akinetischen Krise, es wird in der Literatur jedoch das THS-ES als eigene Entität diskutiert aufgrund unterschiedlicher pathophysiologischer Mechanismen und Therapieansätze [53].

Evidenzgrundlage

Die Literatursuche in Medline via Pubmed erbrachte 76 Studien zu den Begriffen „Parkinson disease AND DBS withdrawal“. Nach Screening der Inhalte wurden 14 Literaturreferenzen einbezogen. Es fanden sich keine randomisierten, kontrollierten Studien in dieser Literaturrecherche, sondern Falldarstellungen und Übersichtsartikel.

Ergebnis

Definition: Das Tiefe-Hirnstimulations-Entzugssyndrom (THS-ES) ist definiert durch einen potenziell vital gefährdenden Zustand mit akuter Zunahme der Akinese, weiterer Kardinalsymptome der Parkinson-Erkrankung, autonomer Instabilität, Fieber und CK-Erhöhung, welche innerhalb von Stunden bis Tagen nach Schrittmacherausfall bei Pat. mit langjähriger THS auftreten [52]. Weitere Charakteristika sind ein fehlendes Ansprechen des THS-ES auf eine alleinige, dopaminerge Medikationsanpassung, die Notwendigkeit eines intensivmedizinischen Aufenthalts und die Restitution nach einer möglichst frühen Wiederaufnahme einer effektiven THS [54], [55].

Risikofaktoren: Pat.-bezogene Risikofaktoren für ein THS-ES sind ein junges Erkrankungsalter bei Erstmanifestation der Erkrankung, eine bilaterale Implantation im Nucleus subthalamicus (STN), eine längere Implantationsdauer > 5 Jahre, ein fortgeschrittenes Erkrankungsstadium > 15 Jahre, ein höheres Lebensalter und ein exzellentes Ansprechen auf die THS mit postoperativ niedrig dosierter, dopaminerger Therapie [56], [57], [58]. Einschränkend muss berücksichtigt werden, dass bei den wenigen beschriebenen Fällen diese ersten Beobachtungen in weiteren prospektiven Untersuchungen größerer Kohorten überprüft werden müssen.

Auslösefaktoren: Den Auslösefaktor des THS-ES stellt der abrupte, längerfristige Ausfall des THS-Systems dar, der durch unterschiedliche Faktoren bedingt sein kann. Bei den wiederaufladbaren Schrittmachersystemen sind regelmäßige Aufladeprozeduren durch den Pat. über 15–25min/Tag oder 90min/Woche obligatorisch [59], welche bei Pat. im höheren Lebensalter mit kognitiven Einschränkungen manchmal unbeabsichtigt ausgelassen oder ineffizient durchgeführt werden, sodass ein Leerlaufen des Akkus mit Ausschalten der THS erfolgen kann [60]. Batteriebetriebene THS-Systeme bergen aufgrund der Notwendigkeit der wiederholten operativ-chirurgischen Wechsel ein erhöhtes Infektionsrisiko von bis zu 5–15% [59], [61], welche mit der Notwendigkeit einer Explantation einzelner Bestandteile oder sogar des gesamten THS-Systems und einer Stimulationspause von 6 Wochen bis 3 Monaten [62], [63] einhergehen. Strukturelle Versorgungsaspekte wie weite Anfahrtswege für den Pat. in THS-Zentren, ländliche medizinische Unterversorgung oder finanzielle Aspekte einer ungeklärten Kostenübernahme oder durch fehlende regelmäßige Batteriekontrolle können einen verzögerten operativen Stimulatorwechsel für ein THS-ES bedingen. Eine besondere Herausforderung in der THS-Versorgung stellte die COVID-Pandemie dar. Das plötzliche „Leerlaufen“ der Batterien bei verschobenen oder ausgefallenen ambulanten Kontrollterminen sowie Streichung „elektiver“ Operationen führte zu Versorgungsengpässen mit dem Risiko von THS-ES [56], [64].

Verlauf: In einer monozentrischen, retrospektiven Studie wurden aus 434 THS-Pat. der klinische Verlauf von 15 THS-Pat. (3.5%) mit transienter Explantation von Bestandteilen des THS-Systems aufgrund einer Infektion und Stimulationspause über 2–3 Monate untersucht. 12 Pat. konnten trotz

schwerer motorischer Fluktuationen und motorischer Verschlechterung die Wartezeit bis zur Reimplantation zu Hause verbringen. Bei 3 Pat. trat jedoch ein THS-ES auf mit der Notwendigkeit eines intensivmedizinischen Aufenthalts und mit einem letalen Ausgang bei 2 Pat. [54].

Differenzialdiagnosen: Abzugrenzen vom THS-ES sind perioperative akinetische Krisen während der Neuimplantation einer THS, die auf die zu schnelle Reduktion der dopaminergen Medikamente zurückzuführen sind und nicht auf einen Entzug einer chronischen Stimulation [65], [66].

Begründung der Empfehlung

Auf der Basis der oben dargestellten Evidenzgrundlage können zur Diagnosestellung einer THS-ES bei Parkinson-Pat. folgende Empfehlung herangezogen werden.

Empfehlung	Neu<br>Stand (2023)
Zur Diagnosestellung einer THS-ES bei Parkinson-Pat. können folgende Kriterien herangezogen werden:<br>1. das Vorliegen von Pat.-bezogenen oder von technischen, THS-System-assoziierten Risikofaktoren<br>2. die klinische Symptomatologie in Form einer akuten Verschlechterung der Parkinson-Symptomatik, begleitet von Fieber, Vigilanzstörung, vegetativer Begleitsymptomatik sowie CK-Erhöhung<br>3. der Ausfall eines langjährig implantierten, zuvor symptomatisch gut wirksamen THS-Systems<br>4. der klinische Verlauf mit Vorliegen einer transienten Levodopa-Resistenz der ausgeprägten Symptomatik und Restitution nach Wiederaufnahme einer effektiven THS.	
Konsensstärke: 100%, starker Konsens	

Fragestellung 82: Wie wird das Tiefe-Hirnstimulations-Entzugssyndrom bei der PK therapiert?

Hintergrund

Die Behandlung der Parkinson-Pat. während des Tiefe-Hirnstimulations-Entzugssyndroms (THS-ES) stellt bei unumgänglichen Stimulationspausen wie z.B. bei infektbedingter Explantation von THS-Bestandteilen eine besondere Herausforderung dar.

Evidenzgrundlage

Nach der Literatursuche in Medline via Pubmed mit den Begriffen „Parkinson disease AND DBS withdrawal“ wurden nach Screening zu Angaben einer Therapie 8 Studien identifiziert, welche ausgewertet wurden. Da das THS-ES ein seltenes Ereignis ist, gibt es derzeit keine systematischen, randomisierten, kontrollierten Studien und somit keine validierte Evidenz zu der optimalen Therapie.

Ergebnis

Die wesentliche Therapie der THS-ES bei Parkinson-Pat. liegt in der raschen Wiederaufnahme einer effektiven THS und ggf. frühen Reimplantation eines Impulsgebers oder weiterer THS-Bestandteile.

Die transiente Behandlung während einer unumgänglichen Stimulationspause z.B. im Rahmen einer Infektion einer THS-Systemkomponente ist angelehnt an die Therapierichtlinien einer akinetischen Krise (Fragestellung 2).

Auslösefaktoren: Die beste Therapie ist die Prävention des THS-ES durch engmaschige Kontrollen des Batteriestatus alle 3–6 Monate mithilfe des Pat.-Programmiergeräts durch die Pat. und ambulante ärztliche Kontrollen. Bei Pat. mit Risikofaktoren für ein THS-ES muss mit einem ausreichenden Sicherheitsabstand vor dem Erreichen des Austauschindikators (elective replacement indicator, ERI) der Schrittmacherwechsel mit hoher Priorität erfolgen [64].

Frühzeitige Wiederaufnahme einer effektiven THS: Bei Pat. mit Risikofaktoren für das Entstehen eines THS-ES (langjährige, bilaterale Implantation im Nucleus subthalamicus (STN) > 5 Jahre, fortgeschrittenes Erkrankungsstadium > 15 Jahre, höheres Lebensalter und exzellentes Ansprechen auf die THS) [56], [57], [58] sollte unverzüglich eine Reimplantation bzw. Wiederaufnahme der Stimulation erfolgen. Bei prädisponierten Parkinson-Pat. wurde beschrieben, dass trotz deutlicher Erhöhung der dopaminergen Dosis und der klassischen Therapieansätze, wie sie in der akinetischen Krise angewendet werden, keine Symptombesserung zu erzielen war, bis eine effektive THS wieder begonnen werden konnte [54], [58]. Nach chirurgischem Standard wird bei THS-System-Infektionen empfohlen, erst 6 Wochen bis 3 Monate nach Systemexplantation eine Reimplantation durchzuführen [62], [63], um ein Übergreifen der Infektion auf das neue Schrittmachersystem nicht zu riskieren. In einer Fallserie von 5 STN-THS-Pat. wurde jedoch aufgrund eines bestehenden THS-ES eine „frühe“ Reimplantation des Schrittmachers auf der kontralateralen Seite in der infraklavikulären Loge unter fortlaufender antibiotischer Abdeckung nach im Mittel 23 Tagen durchgeführt, sobald der lokale Befund und der allgemeine Status des/der Pat. eine Operation erlaubten [58]. Alle 5 Pat. überlebten ohne weitere schwerwiegende Komplikationen und erlangten ihre Unabhängigkeit in den Aktivitäten des täglichen Lebens zurück. In einer Nachbeobachtung über 1 Jahr zeigte keine/keiner der Pat. eine Reinfektion des neuen THS-Systems [58]. Das Vorliegen eines THS-ES rechtfertigt somit außerhalb der neurochirurgischen Standards eine vorzeitige Reimplantation eines IPG <6 Wochen.

Medikamentöse Therapie: Ist eine Stimulationspause unumgänglich, sind transiente, medikamentöse, überbrückende Therapieansätze äquivalent zu den Ansätzen in der akinetischen Krise (Fragestellung 78) anwendbar. In der Literatur zum THS-ES wurden lösliche Präparationen von hoch dosiertem Levodopa (bis zu 5x Ausgangsdosis), intravenös appliziertes Amantadin-Sulfat sowie Pramipexol und Motilium über eine Magensonde verabreicht [53]. Des Weiteren wurden subkutane Apomorphin-Pumpen oder LCIG-Pumpen verwendet [58]. Zu berücksichtigen ist, dass in einer Pat.-Kohorte mit THS-ES beschrieben wurde, dass trotz Erhöhung der dopaminergen Medikation auf >3g/Tag die motorische Beweglichkeit sich weiter verschlechterte bis auf ein Hoehn-&-Yahr-Stadium 5 [58].

Supportive therapeutische Maßnahmen: Wie bei der akinetischen Krise sollten adäquate supportive therapeutische Maßnahmen ergriffen werden. Vitalfunktionen sollten regelmäßig kontrolliert, fiebersenkende physikalische und ggf. medikamentöse Maßnahmen sollten angewendet werden. Eine Thromboseprophylaxe und ausreichende intravenöse Flüssigkeitsgabe sollten gewährleistet sein. Darüber hinaus sollte ein Screening auf mögliche Komplikationen wie z.B.

Aspirationspneumonien oder akutes Nierenversagen erfolgen, um diese frühestmöglich zu behandeln.

Begründung der Empfehlung

Die Evidenz beruht auf wenigen Fallberichten oder Fallserien ohne randomisierte, kontrollierte Studien. Therapeutische Ansätze können aus diesen vorliegenden Studien erwogen werden.

Empfehlung	Neu<br>Stand (2023)
1. Eine Prävention des THS-ES durch engmaschige Kontrollen des Batteriestatus alle 3–6 Monate und eine Identifikation der Pat. Mit besonderem Risiko für ein mögliches THS-ES sollten durchgeführt werden.<br>2. Eine frühzeitige Wiederaufnahme einer effektiven THS, ggf. durch frühzeitige Reimplantation, kann erwogen werden.<br>3. Bei unumgänglicher Stimulationspause können transiente medikamentöse überbrückende Therapieansätze äquivalent zu den Ansätzen in der akinetischen Krise (Fragestellung 78) wie die hoch dosierte Gabe von L-Dopa, Amantadin i.v. oder die Anwendung einer s.c. Apomorphinpumpe oder LCIG-Pumpe erwogen werden.<br>4. Supportive therapeutische Maßnahmen wie Thromboseprophylaxe und Behandlung von möglichen Komplikationen wie z.B. Aspirationspneumonie können erwogen werden.	
Konsensstärke: 100%, starker Konsens	

Wichtige Forschungsfragen: Randomisierte kontrollierte Studien zur Wirksamkeit und Sicherheit therapeutischer Optionen bei dem THS-ES in Parkinson-Pat. Fehlen bislang vollständig.

Referenzen

1. Onofrj M, Thomas A. Acute akinesia in Parkinson disease. Neurology. 2005;64(7):1162-9
2. Takubo H, Harada T, Hashimoto T, Inaba Y, Kanazawa I, Kuno S, et al. A collaborative study on the malignant syndrome in Parkinson's disease and related disorders. Parkinsonism Relat Disord. 2003;9 Suppl 1:S31-41
3. Wang JY, Huang JF, Zhu SG, Huang SS, Liu RP, Hu BL, et al. Parkinsonism-Hyperpyrexia Syndrome and Dyskinesia-Hyperpyrexia Syndrome in Parkinson's Disease: Two Cases and Literature Review. J Parkinsons Dis. 2022;12(6):1727-35
4. Thomas A, Onofrj M. Akinetic crisis, acute akinesia, neuroleptic malignant-like syndrome, Parkinsonism-hyperpyrexia syndrome, and malignant syndrome are the same entity and are often independent of treatment withdrawal. Mov Disord. 2005;20(12):1671; author reply -2
5. Bonanni L, Onofrj M, Valente EM, Manzoli L, De Angelis MV, Capasso M, Thomas A. Recurrent and fatal akinetic crisis in genetic-mitochondrial parkinsonisms. Eur J Neurol. 2014;21(9):1242-6
6. Tackenberg B, Möller JC, Eggert K, Sommer N, Oertel WH. Akinetic crisis caused by rectus sheath hematoma and acute anemia due to subcutaneous administration of apomorphine. Mov Disord. 2006;21(1):126-7
7. Douglas A, Morris J. It was not just a heatwave! Neuroleptic malignant-like syndrome in a patient with Parkinson's disease. Age Ageing. 2006;35(6):640-1

8. JiYi X, Hantao S, Lijiao Z, Bin Q. Case report: cold medicines cause neuroleptic malignant-like syndrome in a patient with Parkinson's disease. Eur J Clin Pharmacol. 2022;78(7):1203-4
9. Koehler PJ, Mirandolle JF. Neuroleptic malignant-like syndrome and lithium. Lancet. 1988;2(8626-8627):1499-500
10. Dos Santos DT, Imthon AK, Strelow MZ, Pille A, Schumacher-Schuh AF. Parkinsonism-hyperpyrexia Syndrome After Amantadine Withdrawal: Case Report and Review of the Literature. Neurologist. 2021;26(4):149-52
11. Kuno S, Mizuta E, Yamasaki S. Neuroleptic malignant syndrome in parkinsonian patients: risk factors. Eur Neurol. 1997;38 Suppl 2:56-9
12. Kaasinen V, Joutsa J, Noponen T, Päivärinta M. Akinetic crisis in Parkinson's disease is associated with a severe loss of striatal dopamine transporter function: a report of two cases. Case Rep Neurol. 2014;6(3):275-80
13. Martino G, Capasso M, Nasuti M, Bonanni L, Onofrj M, Thomas A. Dopamine transporter single-photon emission computerized tomography supports diagnosis of akinetic crisis of parkinsonism and of neuroleptic malignant syndrome. Medicine (Baltimore). 2015;94(13):e649
14. Pfeiffer RF, Sucha EL. "On-off"-induced lethal hyperthermia. Mov Disord. 1989;4(4):338-41
15. Jayaram L, Chancellor AM. Rhabdomyolysis and akinetic hyperthermic crisis complicating Parkinson's disease. Aust N Z J Med. 1997;27(2):194-5
16. Sahu H, Manjunath MB, Ray A, Vikram NK. Neuroleptic malignant-like syndrome causing thrombocytopaenia: a rare association. BMJ Case Rep. 2018;11(1)
17. Capasso M, De Angelis MV, Di Muzio A, Anzellotti F, Bonanni L, Thomas A, Onofrj M. Critical Illness Neuromyopathy Complicating Akinetic Crisis in Parkinsonism: Report of 3 Cases. Medicine (Baltimore). 2015;94(28):e1118
18. Gordon PH, Frucht SJ. Neuroleptic malignant syndrome in advanced Parkinson's disease. Mov Disord. 2001;16(5):960-2
19. Bonnici A, Ruiner CE, St-Laurent L, Hornstein D. An interaction between levodopa and enteral nutrition resulting in neuroleptic malignant-like syndrome and prolonged ICU stay. Ann Pharmacother. 2010;44(9):1504-7
20. Tomlinson CL, Stowe R, Patel S, Rick C, Gray R, Clarke CE. Systematic review of levodopa dose equivalency reporting in Parkinson's disease. Mov Disord. 2010;25(15):2649-53
21. Newman EJ, Grosset DG, Kennedy PG. The parkinsonism-hyperpyrexia syndrome. Neurocrit Care. 2009;10(1):136-40
22. Bonuccelli U, Piccini P, Corsini GU, Muratorio A. Apomorphine in malignant syndrome due to levodopa withdrawal. Ital J Neurol Sci. 1992;13(2):169-70
23. Auffret M, Drapier S, Vérin M. New tricks for an old dog: A repurposing approach of apomorphine. Eur J Pharmacol. 2019;843:66-79
24. Thomas A, Iacono D, Luciano AL, Armellino K, Onofrj M. Acute akinesia or akinetic crisis in Parkinson's disease. Neurol Sci. 2003;24(3):219-20
25. Kipps CM, Fung VS, Grattan-Smith P, de Moore GM, Morris JG. Movement disorder emergencies. Mov Disord. 2005;20(3):322-34
26. Gálvez-Jiménez N, Lang AE. Perioperative problems in Parkinson's disease and their management: apomorphine with rectal domperidone. Can J Neurol Sci. 1996;23(3):198-203
27. Dafotakis M, Sparing R, Juzek A, Block F, Kosinski CM. Transdermal dopaminergic stimulation with rotigotine in Parkinsonian akinetic crisis. J Clin Neurosci. 2009;16(2):335-7
28. Fiore S, Persichino L, Anticoli S, De Pandis MF. A neuroleptic malignant-like syndrome (NMLS) in a patient with Parkinson's disease resolved with rotigotine: a case report. Acta Biomed. 2014;85(3):281-4
29. Onofrj M, Bonanni L, Cossu G, Manca D, Stocchi F, Thomas A. Emergencies in parkinsonism: akinetic crisis, life-threatening dyskinesias, and polyneuropathy during L-Dopa gel treatment. Parkinsonism Relat Disord. 2009;15 Suppl 3:S233-6

30. Greulich W, Fenger E. Amantadine in Parkinson's disease: pro and contra. J Neural Transm Suppl. 1995;46:415-21
31. Danielczyk W. Twenty-five years of amantadine therapy in Parkinson's disease. J Neural Transm Suppl. 1995;46:399-405
32. Kornhuber J, Weller M, Riederer P. Glutamate receptor antagonists for neuroleptic malignant syndrome and akinetic hyperthermic parkinsonian crisis. J Neural Transm Park Dis Dement Sect. 1993;6(1):63-72
33. Lange KW, Kornhuber J, Riederer P. Dopamine/glutamate interactions in Parkinson's disease. Neurosci Biobehav Rev. 1997;21(4):393-400
34. Zheng KS, Dorfman BJ, Christos PJ, Khadem NR, Henchcliffe C, Piboolnurak P, Nirenberg MJ. Clinical characteristics of exacerbations in Parkinson disease. Neurologist. 2012;18(3):120-4
35. Rabinak CA, Nirenberg MJ. Dopamine agonist withdrawal syndrome in Parkinson disease. Arch Neurol. 2010;67(1):58-63
36. Cunnington AL, White L, Hood K. Identification of possible risk factors for the development of dopamine agonist withdrawal syndrome in Parkinson's disease. Parkinsonism Relat Disord. 2012;18(9):1051-2
37. Pondal M, Marras C, Miyasaki J, Moro E, Armstrong MJ, Strafella AP, et al. Clinical features of dopamine agonist withdrawal syndrome in a movement disorders clinic. J Neurol Neurosurg Psychiatry. 2013;84(2):130-5
38. Chaudhuri KR, Todorova A, Nirenberg MJ, Parry M, Martin A, Martinez-Martin P, et al. A Pilot Prospective, Multicenter Observational Study of Dopamine Agonist Withdrawal Syndrome in Parkinson's Disease. Mov Disord Clin Pract. 2015;2(2):170-4
39. Garcia X, Mohammad ME, Patel S, Yu XX, Fernandez HH. Dopamine agonist withdrawal syndrome associated factors: A retrospective chart review. Clin Park Relat Disord. 2022;7:100153
40. Nirenberg MJ. Dopamine agonist withdrawal syndrome: implications for patient care. Drugs Aging. 2013;30(8):587-92
41. Limotai N, Oyama G, Go C, Bernal O, Ong T, Moum SJ, et al. Addiction-like manifestations and Parkinson's disease: a large single center 9-year experience. Int J Neurosci. 2012;122(3):145-53
42. Thobois S, Ardouin C, Lhommée E, Klinger H, Lagrange C, Xie J, et al. Non-motor dopamine withdrawal syndrome after surgery for Parkinson's disease: predictors and underlying mesolimbic denervation. Brain. 2010;133(Pt 4):1111-27
43. Solla P, Fasano A, Cannas A, Mulas CS, Marrosu MG, Lang AE, Marrosu F. Dopamine agonist withdrawal syndrome (DAWS) symptoms in Parkinson's disease patients treated with levodopa-carbidopa intestinal gel infusion. Parkinsonism Relat Disord. 2015;21(8):968-71
44. Driver-Dunckley E, Samanta J, Stacy M. Pathological gambling associated with dopamine agonist therapy in Parkinson's disease. Neurology. 2003;61(3):422-3
45. Santos-García D, Macías M, Llaneza M, Aneiros A. Suicide following duodenal levodopa infusion for Parkinson's disease. Mov Disord. 2009;24(13):2029-30
46. Flament M, Loas G, Godefroy O, Krystkowiak P. Suicide without depression after withdrawal of a dopamine agonist in a patient with Parkinson's disease. J Neuropsychiatry Clin Neurosci. 2011;23(4):E32
47. Zorko N, Kojovic M, Flisar D, Pirtosek Z, Kramberger MG. Suicide in Parkinson's Disease Patients Treated With Levodopa-Carbidopa Intestinal Gel. Mov Disord. 2015;30(10):1434-5
48. Kwan C, Kolivakis T, Huot P. Dopamine Agonist Withdrawal Syndrome and Suicidality in Parkinson's Disease. Can J Neurol Sci. 2023;50(5):779-80
49. Yu XX, Fernandez HH. Dopamine agonist withdrawal syndrome: A comprehensive review. J Neurol Sci. 2017;374:53-5
50. Dorfman BJ, Nirenberg MJ. Dopamine agonist withdrawal syndrome in a patient with restless legs syndrome. Parkinsonism Relat Disord. 2013;19(2):269-70

51. Koschel J, Ray Chaudhuri K, Tönges L, Thiel M, Raeder V, Jost WH. Implications of dopaminergic medication withdrawal in Parkinson's disease. J Neural Transm (Vienna). 2022;129(9):1169-78
52. Grimaldi S, Eusebio A, Carron R, Regis JM, Velly L, Azulay JP, Witjas T. Deep Brain Stimulation-Withdrawal Syndrome in Parkinson's Disease: Risk Factors and Pathophysiological Hypotheses of a Life-Threatening Emergency. Neuromodulation. 2023;26(2):424-34
53. Rajan R, Krishnan S, Kesavapisharady KK, Kishore A. Malignant Subthalamic Nucleus-Deep Brain Stimulation Withdrawal Syndrome in Parkinson's Disease. Mov Disord Clin Pract. 2016;3(3):288-91
54. Reuter S, Deuschl G, Falk D, Mehdorn M, Witt K. Uncoupling of dopaminergic and subthalamic stimulation: Life-threatening DBS withdrawal syndrome. Mov Disord. 2015;30(10):1407-13
55. Hariz MI, Johansson F. Hardware failure in parkinsonian patients with chronic subthalamic nucleus stimulation is a medical emergency. Mov Disord. 2001;16(1):166-8
56. Holla VV, Neeraja K, Surisetti BK, Prasad S, Kamble N, Srinivas D, et al. Deep Brain Stimulation Battery Exhaustion during the COVID-19 Pandemic: Crisis within a Crisis. J Mov Disord. 2020;13(3):218-22
57. Azar J, Elinav H, Safadi R, Soliman M. Malignant deep brain stimulator withdrawal syndrome. BMJ Case Rep. 2019;12(5)
58. Reuter S, Deuschl G, Berg D, Helmers A, Falk D, Witt K. Life-threatening DBS withdrawal syndrome in Parkinson's disease can be treated with early reimplantation. Parkinsonism Relat Disord. 2018;56:88-92
59. Sarica C, Iorio-Morin C, Aguirre-Padilla DH, Najjar A, Paff M, Fomenko A, et al. Implantable Pulse Generators for Deep Brain Stimulation: Challenges, Complications, and Strategies for Practicality and Longevity. Front Hum Neurosci. 2021;15:708481
60. Mitchell KT, Volz M, Lee A, San Luciano M, Wang S, Starr PA, et al. Patient Experience with Rechargeable Implantable Pulse Generator Deep Brain Stimulation for Movement Disorders. Stereotact Funct Neurosurg. 2019;97(2):113-9
61. Voges J, Waerzeggers Y, Maarouf M, Lehrke R, Koulousakis A, Lenartz D, Sturm V. Deep-brain stimulation: long-term analysis of complications caused by hardware and surgery--experiences from a single centre. J Neurol Neurosurg Psychiatry. 2006;77(7):868-72
62. Piacentino M, Pilleri M, Bartolomei L. Hardware-related infections after deep brain stimulation surgery: review of incidence, severity and management in 212 single-center procedures in the first year after implantation. Acta Neurochir (Wien). 2011;153(12):2337-41
63. Morishita T, Foote KD, Burdick AP, Katayama Y, Yamamoto T, Frucht SJ, Okun MS. Identification and management of deep brain stimulation intra- and postoperative urgencies and emergencies. Parkinsonism Relat Disord. 2010;16(3):153-62
64. Miocinovic S, Ostrem JL, Okun MS, Bullinger KL, Riva-Posse P, Gross RE, Buetefisch CM. Recommendations for Deep Brain Stimulation Device Management During a Pandemic. J Parkinsons Dis. 2020;10(3):903-10
65. Kim JH, Kwon TH, Koh SB, Park JY. Parkinsonism-hyperpyrexia syndrome after deep brain stimulation surgery: case report. Neurosurgery. 2010;66(5):E1029
66. Akçakaya MO, Akçakaya NH, Kasımcan M, Kırış T. Life-threatening parkinsonism-hyperpyrexia syndrome following bilateral deep brain stimulation of the subthalamic nucleus. Neurol Neurochir Pol. 2018;52(2):289-92
67. Senek M, Nyholm D, Nielsen EI. Population pharmacokinetics of levodopa gel infusion in Parkinson's disease: effects of entacapone infusion and genetic polymorphism. Sci Rep. 2020;10(1):18057

3.8 Spezielle Behandlungsfragen

3.8.1 Perioperatives Management

Autoren:/Autorinnen Sylvia Maaß, Monika Pötter-Nerger, Joseph Claßen

Fragestellung 83: Wie muss die Pharmakotherapie bei der PK perioperativ sicher und wirksam umgestellt werden?

Hintergrund

In der Literatur besteht Konsens darüber, dass das perioperative Risiko bei Parkinson-Pat. Insgesamt erhöht ist [1], [2], [3]. Als Gründe werden beispielsweise Dysphagie, verschlechterte Lungenbelüftung durch Beeinträchtigung der Atemhilfsmuskulatur [4], ein höheres Risiko für Stürze und Harnwegsinfekte [5] angegeben. Umso wichtiger ist eine möglichst kontinuierliche Gabe der dopaminergen Medikation im perioperativen Setting. Durch fehlerhafte Gabe oder abruptes Absetzen steigt das Morbiditätsrisiko und die Entstehung akinetischer Krisen wird begünstigt [6]. Auch steigt das Risiko der raschen Entwicklung von zum Teil irreversiblen Kontrakturen durch diskontinuierliche Gabe der dopaminergen Medikation und Immobilisation im Zusammenhang mit Aufenthalten auf der Intensivstation, wobei insbesondere Pat. In den späteren Stadien der Erkrankung gefährdet sind [7], [8].

Eine perioperative Umstellung der dopaminergen Medikation wird dann notwendig, wenn die übliche dopaminerge Medikation für mehr als einige wenige Stunden der OP-Dauer unterbrochen werden muss, z. B. im Rahmen abdomineller operativer Eingriffe, die postoperativ einen längeren „Nüchtern-Status“ der Pat. Erfordern [1], bei peri-/postoperativen Störungen der Resorption oder bei erforderlicher Ernährung via nasogastraler Sonde im Nachgang einer OP, z. B. bei längerfristiger Beatmungspflichtigkeit/Nachbeatmung [3]

Evidenzgrundlage

Nach der Literatursuche mit den Begriffen: „Parkinson disease AND pharmacological therapy OR pharmacological AND treatment OR drug therapy OR drug treatment AND perioperative“ wurden nach Screening der Abstracts und Inhalte 5 Studien identifiziert, die in diese Leitlinie einbezogen wurden. Es fanden sich dabei insgesamt keine randomisierten, kontrollierten Studien in dieser Literaturrecherche zum Thema.

Es fanden sich drei größere Open-label-Kohortenstudien, zudem zwei kleinere, prospektive Open-label-Studien. Darüber hinaus fanden sich mehrere Übersichtsartikel und mehrere Falldarstellungen.

Letztendlich wurden in Ermangelung randomisierter, kontrollierter Studien die oben genannten Studien und Übersichtsartikel selektiert und in diese Leitlinie einbezogen.

Da derzeit keine systematischen, randomisierten, kontrollierten Studien und somit keine validierte Evidenz zur optimalen perioperativen Umstellung der Pharmakotherapie bei der PK existiert, beruhen die Empfehlungen zur Therapie auf den verfügbaren Kohorten- und Fallbeobachtungen und pathophysiologischen Überlegungen im Sinne einer Expertenmeinung.

Ergebnis

Empfehlungen zum perioperativen Management der Pharmakotherapie bei der PK

Prinzipiell ist eine rasche Weiterführung der gewohnten Pharmakotherapie bei Parkinson-Pat. Postoperativ anzustreben. Falls dies nicht möglich ist, beispielsweise bei länger erforderlichem „Nüchtern-Status“, bei längerer postoperativer Beatmungspflichtigkeit oder bei postoperativem Auftreten von Schluck- bzw. Resorptionsstörungen, sollte eine rasche Umstellung der Pharmakotherapie auf orale oder parenterale Alternativen erfolgen.

Wichtig ist der frühe Beginn einer adäquaten oralen bzw. parenteralen Therapieumstellung, um die Prognose zu verbessern, Komplikationen zu vermeiden und der Entwicklung einer akinetischen Krise entgegenzusteuern.

Supportive Maßnahmen: Selbstverständlich müssen immer alle nicht medikamentösen, supportiven Maßnahmen ergriffen werden, um eine rasche (Wieder-)Oralisierung der zuvor eingenommenen Parkinson-Medikation zu ermöglichen. Dazu gehören allgemeine Maßnahmen wie Rehydrierung, Ausgleich von Elektrolytentgleisungen, Kontrolle der Vitalfunktionen, insbesondere Blutdruck, Behandlung von Infekten und zeitnahe Mobilisierung.

Typische antidopaminerg wirksame Antipsychotika und antidopaminerg wirksame Antiemetika sollen nicht verwendet werden.

Perioperative Möglichkeiten der Umstellung der Pharmakotherapie bei der PK

1. Applikation von Levodopa:

   Falls eine Applikation von Medikamenten via nasogastraler Sonde/PEG möglich ist, sollte die Tagesdosis von Levodopa umgestellt werden auf lösliches Levodopa (Madopar® LT). Dies kann aufgelöst in Wasser sicher über die Sonde verabreicht werden.

   Die Dosis des löslichen Levodopa ist zu berücksichtigen, dabei muss die präoperative Parkinsonmedikation umgerechnet werden in die entsprechende Levodopa-Äquivalenzdosis (LEDD Umrechnungstabelle siehe [9].

   Alternativ kann eine Gabe von Levodopa-Carbidopa Intestinalem Gel (LCIG) oder Levodopa/Entacapon/Carbidopa intestinalem Gel (LECIG) über eine PEG-J-Sonde (JET-PEG) erwogen werden [10]. Dies erscheint jedoch aufgrund der schwieriger zu platzierenden Sonde und der etwas aufwendigeren Pflege sowie der Pumpenbedienung im perioperativen Setting insbesondere auf nicht neurologischen Abteilungen weniger geeignet.

   Auf Interferenzen von Levodopa mit der Sondennahrung und konsekutivem Wirkungsverlust muss geachtet werden, da Sondennahrung auf Intensivstationen typischerweise kontinuierlich verabreicht wird. Eine zeitliche Protokollvorgabe der Applikation von Levodopa und Sondennahrung sollte erstellt werden.

   Da Resorptionsstörungen im postoperativen Verlauf häufig sind, kann es hilfreich sein, die Ernährung auf hochkalorische, eiweißarme Nahrung (2kcal/ml) [11] umzustellen und diese im Einzelfall nachts zu verabreichen, damit das Levodopa untertags besser resorbiert werden kann.

Auch wenn hierdurch ein unphysiologischer Ernährungsrhythmus erzielt wird, kann der Patient u. U. mehr von der regelmäßigen Gabe der Levodopa-Einzeldosen profitieren.

Alternativ kann die Sondennahrung 1 Stunde vor und nach Levodopa-Gabe gestoppt werden, was bei multiplen Einnahmezeitpunkten jedoch in der Praxis oft schwer umsetzbar ist.

Eine weitere Alternative insbesondere bei Ileus-Symptomatik, Resorptionsproblemen oder Interaktion von Nahrung und Levodopa ist die Gabe von rektal zu applizierendem Levodopa in Form von Suppositorien [6], die derzeit in Deutschland jedoch nicht erhältlich sind. Es kann im Einzelfall auf eine rektale Gabe von schnell löslichem Levodopa/Benserazid zurückgegriffen werden. Insbesondere bei einer Ileus-Symptomatik kann dies hilfreich sein.

2. Parenterale Verabreichung von Dopaminagonisten:

Applikation von Rotigotin transdermal:

Eine Umstellung auf transdermal zu applizierendes Rotigotin ist bei einfacher Anwendung mittlerweile relativ geläufig im Bereich der intensivmedizinischen Behandlung von Parkinson-Pat. Es handelt sich bei Rotigotin um einen Non-Ergot-Dopaminagonisten, der über eine Stimulation von D3>D2>D1-Rezeptoren wirkt. Es wird als Pflaster über 24h appliziert, das täglich gewechselt wird.

In einer kleinen offenen prospektiven multizentrischen Studie wurde Rotigotin bei 14 Parkinson-Pat. Und geplantem operativem Eingriff in Vollnarkose angewendet. Die Dosierung wurde individuell nach Entscheidung des behandelnden Neurologen/der behandelnden Neurologin festgesetzt und die Behandlung am Abend vor der OP begonnen und nach dem OP-Tag weitergeführt. Hier wurde eine gute Wirksamkeit beschrieben bei insgesamt guter Verträglichkeit [12].

Zur allgemeinen Umstellung von Dopaminagonisten auf transdermales Rotigotin außerhalb des perioperativen Settings existieren drei weitere Studien:

in einer prospektiven, open-label multizentrischen Studie einer koreanischen Kohorte wurden 116 Pat. Eingeschlossen, die eine Therapie (Monotherapie oder Kombination mit Levodopa) mit dem Dopaminagonisten Ropirinol erhielten.

Die Medikation wurde über Nacht auf Rotigotin transdermal umgestellt in einer Umstellungsrate von Ropirinol zu Rotigotin von 1,5 : 1. Die Verträglichkeit war gut bei fehlendem Wirkungsverlust [13].

Ähnliche Ergebnisse zeigte eine US-amerikanische Kohorte in einer multizentrischen Open-label-Studie, die ebenfalls über Nacht eine Umstellung der Dopaminagonisten Ropirinol, Pramipexol und Cabergolin untersuchte. Das Verhältnis der Umstellung lag bei Ropirinol auf Rotigotin bei 1 : 1, bei Pramipexol auf Rotigotin bei 1 : 4 [14].

Eine weitere asiatische multizentrische Open-label-Studie mit 87 Pat. Zeigte ebenfalls gute Verträglichkeit und Wirksamkeit bei der Umstellung von Pramipexol und Ropirinol auf Rotigotin

Pflaster, wobei hier Ropirinol auf Rotigotin im Verhältnis 1–1,5: 1 und Pramipexol zu Rotigotin im Verhältnis 1:4 verabreicht wurden [15].

Insgesamt kann empfohlen werden, bei erstmaliger Einführung eines Dopaminagonisten die intiale Dosierung niedrig zu beginnen, z. B. mit 2–4mg/24h, das innerhalb von 4 Tagen auf 6mg/24h erhöht werden kann [16] in Abhängigkeit vom Auftreten von Nebenwirkungen. Um Nebenwirkungen in Form von Halluzinationen und deliranten Syndromen gerade bei älteren Pat. Zu vermeiden, sollte hier vorsichtig aufdosiert werden. Weitere Nebenwirkungen der Therapie sind wie bei allen Dopaminagonisten Übelkeit, Erbrechen und orthostatische Probleme, weswegen hier auch eine Vorbehandlung mit Domperidon nötig sein kann, insbesondere bei Pat., die bislang nicht mit einem Dopaminagonisten behandelt wurden.

Cave: Das Rotigotin-Plaster muss wegen der Aluminiumschicht vor MRT-Untersuchungen und Kardioversionen abgenommen werden.

Applikation von Apormophin s.c.

Eine alternative Möglichkeit ist die Anwendung einer kontinuierlichen subkutanen Apomorphin-Infusion mittels Pumpe. Die Dosierung von Apomorphin ist interindividuell unterschiedlich, anzustreben sind Tagesdosierungen von 20–40 mg/Tag.

Alternativ kann eine Gabe von 3–5mg Apomorphin s.c. direkt postoperativ, ebenfalls unter Domperidon-Schutz, erwogen werden [6], [17]. Dem steht entgegen, dass bei der Applikation von Apomorphin als stärkstem zugelassenem Dopaminagonisten mit Übelkeit und Erbrechen zu rechnen ist. Es liegen keine Daten vor, ob dies durch präoperative Medikation mit Domperidon (3 x 20 mg/Tag, 3 Tage) wirksam verhindert werden kann. Domperidon sollte mit der niedrigsten wirksamen Dosis über einen kürzestmöglichen Zeitraum eingenommen werden; die Tageshöchstdosis von Domperidon ist auf 3 x 10 mg begrenzt. Domperidon kann auch rektal verabreicht werden [18]. Zu beachten ist in jedem Fall eine mögliche QTc-Zeitverlängerung unter der Gabe von Domperidon. Eine seltene Komplikation unter Apomorphin ist eine hämolytische Anämie; hier muss die Therapie abgebrochen werden.

Verabreichung von Amantadin i.v.

Amantadin ist als intravenös applizierbares Präparat gerade bei erforderlicher parenteraler Gabe von Parkinson-Medikamenten seit vielen Jahren im Einsatz.

Der Wirkmechanismus ist vorwiegend antiglutamaterg über NMDA-Rezeptoren, anticholinerg und schwach dopaminerg [19].

Es existiert eine kleine Fallserie, wobei Parkinson-Pat. Perioperativ zweimal täglich 200 mg Amantadin infundiert bekamen, während alle anderen Parkinson-Medikamente gestoppt wurden. Die mittlere Infusionsdauer betrug 3 Tage. Die Autoren/Autorinnen berichteten über eine gute Verträglichkeit und Effizienz [20].

Amantadin kann laut Fachinformation 1–3-mal täglich in einer Dosierung von 200 mg i.v. verabreicht werden. Zu beachten ist die Gefahr der Akkumulation bei Niereninsuffizienz.

Nebenwirkungen wie Delir, Halluzinationen und nächtliche Albträume müssen berücksichtigt werden; ggfs. Muss eine abendliche/nächtliche Verabreichung vermieden werden. Zu beachten ist auch, dass der NMDA-Antagonist Amantadin Herzrhythmusstörungen hervorrufen kann (Long-QT-Syndrom), weswegen ein präoperatives EKG mit der Frage nach einer QTc-Zeit-Verlängerung zwingend durchzuführen ist.

Zusammenfassung

Es existieren mehrere Möglichkeiten, die Pharmakotherapie bei der PK perioperativ oral und parenteral umzustellen. Wichtig ist die frühe Umstellung der Parkinson-Medikation, um Komplikationen zu vermeiden und die Prognose zu verbessern. Bei möglicher oraler Gabe sollte löslichem Levodopa der Vorzug gegeben werden. Bei nicht oraler Gabe steht der Dopaminagonist Rotigotin als einfach zu handhabende transdermale Applikation zur Verfügung. Alternativ kann auf Apomorphin s.c. unter Domperidon-Schutz zurückgegriffen werden. Zudem steht die intravenöse Gabe von Amantadin zur Verfügung.

Begründung der Empfehlung:

Die Evidenz beruht auf Beobachtungsstudien sowie Fallberichten oder Fallserien ohne randomisierte, kontrollierte Studien. Therapeutische Ansätze können aus diesen vorliegenden Studien herangezogen und auf mittlerer Evidenzbasis empfohlen werden.

Empfehlung	Neu<br>Stand (2023)
Zur sicheren und wirksamen Umstellung der Pharmakotherapie bei Parkinson-Pat. Können folgende Präparate herangezogen werden:<br>1. **Levodopa:** bei möglicher oraler Gabe der Medikation über Sonde (via MNS/PEG): Errechnung der Levodopa-Äquivalenzdosis (LEDD) und Gabe von löslichem Levodopa über die Sonde<br>2. **Rotigotin transdermales Pflaster:**<br>Bei Pat., die bislang keinen Dopaminagonisten erhielten, kann eine Dosis von 2–4mg/24h Rotigotin als Anfangsdosis empfohlen werden mit schrittweiser Erhöhung über mehrere Tage in Abhängigkeit von der Verträglichkeit.<br>Bei Pat., die Pramipexol erhielten, kann eine Umstellung auf Rotigotin im Verhältnis 1 : 4 empfohlen werden.<br>Ropirinol kann auf Rotigotin in einem Verhältnis von 1–1,5 : 1 umgestellt werden.<br>CAVE: Das Pflaster muss vor MRT-Untersuchungen und Kardioversionen abgenommen werden (Aluminiumschicht).<br>3. **Kontinuierliche subkutane Apomorphin-Infusion** mittels Pumpe: Tagesdosierungen zwischen 20 und 40 mg/Tag können unter Domperidon-Schutz angestrebt werden.<br>4. **Amantadin**: intravenöse Gabe von 200 mg Amantadin bis dreimal täglich möglich. Cave: Niereninsuffizienz, Halluzinationen sowie delirante Syndrome.<br>5. **Allgemeine Empfehlungen:**<br>Sobald wie möglich sollte eine Rückkehr zur ursprünglichen Parkinson-Medikation angestrebt werden.	

Antidopaminerge Antiemetika und (typische, antidopaminerg wirksame) Antipsychotika sollen nicht eingesetzt werden.<br><br>Supportive Maßnahmen wie frühe Mobilisierung, Rehydrierung, logopädische Evaluierung sollten zeitgleich durchgeführt werden, um eine rasche Rückkehr zur oralen Pharmakotherapie zu ermöglichen.
Konsensstärke: 95,2%, starker Konsens

Wichtige Forschungsfragen: Randomisierte kontrollierte Studien zur wirksamen und sicheren Umstellung der Pharmakotherapie bei der PK fehlen bislang vollständig.

3.8.2 Untersuchungsabstände

Autoren/Autorinnen: Joseph Claßen, Monika Pötter-Nerger, Sylvia Maaß

Fragestellung 84: Was ist ein angemessener zeitlicher Abstand für Follow-up-Untersuchungen zur Therapieanpassung nach der Erstdiagnose einer PK?

Hintergrund

Nach der Erstdiagnose einer PK folgt die Einleitung einer medikamentösen Therapie. Dafür stehen als Primärtherapie Levodopa und Dopaminagonisten und MAO-B-Hemmer zur Verfügung, die bedarfs- und indikationsgerecht eingesetzt werden sollen. Allen Therapien gemeinsam ist die einschleichende Dosierung, damit bei auftretenden Nebenwirkungen mit einer Verzögerung der Aufdosierungsgeschwindigkeit, Verminderung der Dosierung oder Substanzwechsel reagiert werden kann. Gleichzeitig gilt, dass die Wirksamkeit einer Therapie mit Levodopa oder einem Dopaminagonisten nicht zuverlässig aus der unmittelbaren Antwort auf die Medikamenteneinnahme abgeleitet werden kann. Vielmehr wird sich auch im Falle einer schwachen oder ausbleibenden unmittelbaren Antwort auf die Medikamenteneinnahme eine positive Beeinflussung der Motorik bei gleichbleibender Medikamentendosis oft erst im Verlauf von mehreren Wochen allmählich aufbauen. Nach Erreichung einer stabilen Therapieresponse wird ein klinisches Therapiemonitoring durchgeführt, um spät auftretende Nebenwirkungen (z.B. Impulskontrollstörungen bei Dopaminagonisten) und das Nachlassen der Wirksamkeit durch Krankheitsprogression zu erkennen und durch Anpassung der Therapie auszugleichen. Der Krankheitsverlauf selbst ist heterogen und hängt von vielen Faktoren ab. Klinische Verlaufsuntersuchungen sind schließlich auch sinnvoll, um die Diagnose einer PK im Verlauf zu überprüfen und ggf. zu revidieren, wenn die therapeutische Wirksamkeit dopaminerger Medikation frühzeitig abnimmt oder verloren geht.

Evidenzgrundlage

Es wurden weder für die Initiierung einer Therapie noch für die frühe Phase nach Etablierung eines stabilen Therapieregimes systematische Untersuchungen zum zeitlichen Abstand von Verlaufsuntersuchungen nach Erstdiagnose einer PK identifiziert. Es wurden Untersuchungsintervalle in ausgewählten multizentrischen, großen Studien zu pharmakologischen Interventionen in der Frühphase der Parkinson-Krankheit betrachtet.

Die ELLDOPA-Studie [21] war eine randomisierte, doppelblinde, placebokontrollierte Studie, in der 361 Pat. Im Frühstadium einer PK über einen Zeitraum von 40 Wochen Carbidopa-Levodopa in einer Tagesdosis von 37,5 und 150 mg, 75 und 300 mg bzw. 150 und 600 mg oder ein entsprechendes Placebo erhielten und anschließend die Behandlung für 2 Wochen unterbrechen mussten. Der primäre Endpunkt war die Veränderung der Werte auf der Unified Parkinson's Disease Rating Scale (UPDRS) zwischen Studienbeginn und 42 Wochen.

Die ADAGIO-Studie [22] war eine placebokontrollierte, doppelblinde, multizentrische Studie mit verzögertem Beginn, in der 1176 Pat. Mit unbehandelter PK im Frühstadium randomisiert entweder 72 Wochen lang 1 mg oder 2 mg Rasagilin pro Tag (Gruppen mit frühem Beginn) oder 36 Wochen lang Placebo erhielten, gefolgt von 1 mg oder 2 mg Rasagilin pro Tag für 36 Wochen (Gruppen mit verzögertem Beginn). Endpunkt war die Notwendigkeit der Aufnahme einer zusätzlichen antiparkinsonschen Therapie. Die LEAP-Studie [23] war eine multizentrische, doppelblinde, placebokontrollierte Studie mit verzögertem Beginn, in der 445 Parkinson-Pat. Im Frühstadium randomisiert entweder 80 Wochen lang Levodopa/Carbidopa (3-mal 100/25mg pro Tag) (Gruppe mit frühem Beginn) oder 40 Wochen lang Placebo erhielten, gefolgt von 40 Wochen lang Levodopa in Kombination mit Carbidopa (Gruppe mit verzögertem Beginn). Der primäre Endpunkt war der Unterschied zwischen den Gruppen in Bezug auf die mittlere Veränderung der Gesamtpunktzahl auf der UPDRS zwischen den Gruppen. Zu den sekundären Endpunkten gehörte die Progression der Symptome (UPDRS-Score) zwischen den Wochen 4 und 40.

Ergebnis

Zum optimalen zeitlichen Abstand von Verlaufsuntersuchungen existieren keine systematischen Untersuchungen. Zwischen den Visitenintervallen der 3 ausgewählten Studien zeigte sich eine beachtliche Heterogenität, die im Einzelnen nicht gesondert begründet wurde. Visiten in der ELLDOPA-Studie [21] wurden am Ende der Wochen 3, 9, 24 und 40 durchgeführt. In der ADAGIO-Studie [22] wurden 12-wöchige Untersuchungsintervalle gewählt. In der LEAP-Studie [23] wurde nach Beginn der medikamentösen Therapie die erste Visite nach 4 Wochen, die zweite Visite nach 22 Wochen durchgeführt.

Begründung der Empfehlung

Die Empfehlung begründet sich ausschließlich auf Expertenmeinung.

Empfehlung	Neu<br>Stand (2023)
1. Verlaufsuntersuchungen nach Erstdiagnose einer PK sollten in geeigneten Abständen durchgeführt werden, um (i) das frühe Therapieansprechen zu verifizieren, (ii) die Therapieverträglichkeit zu eruieren, (iii) spät einsetzendes Therapieansprechen zu verifizieren, sowie (iv) einen frühzeitigen Wirkungsverlust oder die ausbleibende Wirkung dopaminerger Therapien festzustellen.<br>2. Die Abstände sollten angepasst an das klinische Bild, die Art der Ersttherapie, ggf. vorhandene Komorbiditäten und andere Faktoren wie die soziale und berufliche Situation der Pat. Individuell gewählt werden.	

3. Häufig gewählte Intervalle sind anfangs 4–6 Wochen und 3 Monate. Danach wird eine Nachuntersuchung in mindestens jährlichen Abständen für sinnvoll erachtet. Es erscheint sinnvoll, kleinere Intervalle bei schlechtem Therapieansprechen, schlechter Therapieverträglichkeit und rascher Krankheitsprogression zu wählen, um die Diagnose einer PK zu überprüfen.

Konsensstärke: 100%, starker Konsens

3.8.3 Freezing of Gait

Autoren: Monika Pötter-Nerger, Joseph Classen, Günter Höglinger, Sylvia Maaß

Fragestellung 85: Wie effektiv sind dopaminerge und nicht dopaminerge Substanzen im Vergleich zu Placebo für die Behandlung des Freezing of Gait bei der PK?

Hintergrund

Freezing of Gait (FoG) stellt eine paroxysmale, meist Sekunden anhaltende Unfähigkeit von Pat. Dar, eine Gangbewegung zu initiieren oder fortzuführen. Subjektiv wird FoG von Pat. Empfunden, als ob die „Füße am Boden kleben“ oder „eingefroren“ sind, während der Oberkörper weiterhin einer Propulsionstrajektorie folgt, resultierend in einem erhöhten Risiko von Stürzen (Reviews [24], [25], [26]). Die Inzidenz von FoG ist abhängig von der Erkrankungsdauer mit etwa 50% nach 5 Jahren [27]. Die Behandlung des FoG ist komplex und erfolgt multidisziplinär mit Pharmakotherapie, Physio-, Ergotherapie und Verhaltenstherapie. Eine nach klassischen Indikationskriterien implantierte Tiefe Hirnstimulation kann ebenfalls in der Therapie des FoG angewendet werden. In diesem Abschnitt ist der Fokus auf die pharmakologischen, dopaminergen und nicht dopaminergen Therapieoptionen des FoG gerichtet. In pragmatischen Leitfäden wird weitgehend eine Differenzierung des FoG-Subtyps vorgenommen („Off-Fog“, „Off-On-FoG“, „On-Fog“). Im Wesentlichen wurden aus pathophysiologisch basierten Hypothesen in pragmatischen Leitfäden im Expertenkonsens dopaminerge Substanzen für den Subtyp „Off-FoG“ und nicht dopaminerge Substanzen für den Subtyp „On-FoG“ angewendet. Die Erbringung der Evidenz steht für dieses Vorgehen jedoch noch aus, da in den Studien nicht dopaminerger Substanzen keine klare Trennung der FoG-Subtypen erfolgte und Pat. Sowohl mit Off-Fog als auch mit On-FoG eingeschlossen wurden.

Evidenzgrundlage

Die Literatursuche erbrachte zu den Begriffen „Parkinson disease AND antiparkinson agents AND Placebo IN Humans“ 16 Literaturreferenzen. Nach Screening der Inhalte und Ergänzung von Studien nach Expertenmeinung wurden letztendlich 29 Studien (17 randomisierte, kontrollierte Studien, 9 prospektive Kohortenstudien, 3 Fallserien) selektiert.

Ergebnis

1. Dopaminerge Substanzen

   Für Levodopa konnte der positive Effekt auf FoG nach kleineren Kohortenstudien [28] auch in der randomisierten, doppelblinden, placebokontrollierten ELLDOPA-Studie anhand des UPDRS-FoG-Items nachgewiesen werden [21]. Es wurden 361 Pat. In einem frühen Erkrankungsstadium (< 2

Jahre) über 42 Wochen beobachtet, die Levodopa in verschiedenen Dosierungen (150 mg, 300 mg, 600 mg) oder Placebo erhalten hatten. In der Placebogruppe zeigten 14,4% der Pat. FoG. In den Behandlungsgruppen mit Levodopa war dosisabhängig nur in 5,5-9,8% der Pat. FoG nachweisbar. Darüber hinaus war der Symptombeginn des FoG in den mit Levodopa behandelten Behandlungsarmen später [29].

Levodopa scheint in der Behandlung von FoG wirkungsvoller zu sein als Dopaminagonisten. In der multizentrischen, randomisierten, doppelblinden CALM-PD Studie wurde Pramipexol (3x 0,5 mg) mit Placebo vs. Levodopa (3x100/25mg) mit Placebo in 301 Pat.im frühen Stadium der PK (< 7 Jahre) verglichen. Es zeigte sich in einem Beobachtungszeitraum von 4 Jahren, dass Levodopa zwar mit einem höheren Risiko für die Entwicklung von Fluktuationen gegenüber Pramipexol einhergeht, aber mit einem signifikant geringeren Risiko für die Entwicklung von FoG (25.3% vs. 37.1%) [30]. Dieser Befund konnte auch für andere Dopaminagonisten wie Ropinirol beobachtet werden. So zeigte sich in einer randomisierten, doppelblinden Studie über 5 Jahre in 268 Pat., dass in der Ropinirol-Gruppe (mittlere Dosierung 16mg/Tag) signifikant häufiger FoG als in der mit Levodopa (753mg/Tag) behandelten Gruppe auftrat (32% vs. 25%), allerdings mit dem Vorteil einer reduzierten Inzidenz von Dyskinesien nach 5 Jahren unter Ropinirol (20% vs. 45%) [31].

Der Monoaminoxidase-B-Hemmer Selegilin (10 mg) wurde in der multizentrischen, randomisierten, doppelblinden, placebokontrollierten DATATOP-Studie in 800 Levodopa-naiven Pati. Im frühen Stadium der PK (Hoehn-&-Yahr-Stadium 1–2) in 4 Behandlungsarmen untersucht (1. Selegilin 2. Tocopherol 3.Kombination von Selegilin mit Tocopherol 4. Placebo) [32]. Zu Beginn wiesen 7.1% Pat. FoG auf, nach 2 Jahren 20.7%. Selegilin führte im Vergleich zu Placebo zu einer 53% Reduktion des Risikos, FoG in dem Bebachtungszeitraum von 2 Jahren zu entwickeln, mit einem Wiederanstieg in der „Washout-Phase“, sodass von einem vorwiegend symptomatischen Effekt ausgegangen wird. Es erfolgte 5 Jahre später eine erneute randomisierte Untersuchung der DATATOP-Kohorte von nun 368 Pat. Im mittleren Erkrankungsstadium (Hoehn & Yahr 2) unter dopaminerger Therapie. Selegilin (10 mg) in Kombination mit Levodopa führte in einem Beobachtungszeitraum von 2 Jahren zu einem signifikant reduzierten Risiko, FoG zu entwickeln (15.5%), gegenüber der Placebogruppe (28.9%), einhergehend mit einem reduzierten Risiko von Wearing-off-Phasen [33]. Da in dieser Studie keine Washout-Phase erfolgte, kann nicht sicher unterschieden werden, ob der Effekt von Selegilin auf FoG symptomatisch oder verlaufsmodifizierend ist. In der multizentrischen, randomisierten, doppelblinden, placebokontrollierten LARGO-Studie wurde über einen Beobachtungszeitraum von 18 Wochen der irreversible MAO-B-Hemmer Rasagilin (1mg 1x/Tag) verglichen mit dem COMT-Hemmer-Entacapon (200 mg 4–5x/Tag) oder Placebo bei 687 mit Levodopa behandelten Pat. in einem fortgeschritteneren Stadium (Hoehn-&-Yahr-Stadium 2.1 im MED On, 8–9 Jahre Erkrankungsdauer, bestehende Wirkfluktuationen) untersucht [34]. Für beide Medikamente zeigte sich eine gegenüber Placebo signifikante Off-Zeit-Reduktion, jedoch nur für Rasagilin ergab sich eine signifikante Reduktion im FoG-Item des UPDRS gegenüber Placebo.

2. Nicht dopaminerge Substanzen:

Nicotin-Bitartrat als oral verfügbarer, direkter zentral wirksamer, cholinerger Nikotin-Rezeptoragonist zeigte in einer multizentrischen, randomisierten, doppelblinden Studie [35] bei 65 Pat. mit PK bei guter Verträglichkeit eine signifikante Reduktion von FoG (40% der Pat. in Verum- vs. 15% in Placebogruppe) und Stürzen (47% vs. 11%) nach 10 Wochen gegenüber der Placebogruppe. In der randomisierten, doppelblinden, Placebo-kontrollierten Phase-2-RESPOnD-Studie [36] zeigte der indirekte Acetylcholinesterase Hemmer Rivastigmin in 130 Pat. mit PK über 32 Wochen eine signifikante 20–30%ige Reduktion der Schrittzeitvariabilität während verschiedener Gangstrecken sowie eine Reduktion der Sturzhäufigkeit/Monat um 45%. Allerdings verblieben die subjektiven Wahrnehmungen des FoG unverändert [36]. Zu berücksichtigen ist das Nebenwirkungsprofil cholinerger Medikamente (Appetitlosigkeit, Schwindel, Übelkeit, Erbrechen, Durchfall, Tremor).

Die Studienlage für Methylphenidat als ein noradrenerg-dopaminerg wirkendes psychomotorisches Stimulans ist heterogen. Es zeigten sich in drei ersten offenen Studien mit Verabreichung von 10–80 mg in jeweils 5–21 Pat. positive Effekte auf FoG [37], [38], [39]. In zwei randomisierten, doppelblinden, placebokontrollierten Studien in 17 bzw. 24 Pat. mit PK und moderatem FoG zeigte sich in einem Bebachtungszeitraum von 3 Monaten für Methylphenidat (80 mg/Tag) kein Unterschied zu Placebo bezüglich Gangqualität, FoGQ oder UPDRS, Schreitlänge während kognitiver Dual-Task-Aufgaben und Reaktionszeiten [40], [41]. Die größte, multizentrische, randomisierte, parallele, doppelblinde, placebokontrollierte Studie über 3 Monate an 13 Zentren in Frankreich in 65 Parkinson-Pat. mit Methylphenidat (1mg/kg/Tag) hingegen erbrachte positive Effekte. Eine Studie zu Atomoxetin als Noradrenalin-Wiederaufnahmehemmer wurde vorzeitig abgebrochen, sodass keine verwertbaren Daten bestehen [42]. Zu berücksichtigen bei dem Einsatz noradrenerger Substanzen sind Kontraindikationen wie die gleichzeitige Einnahme von Monoaminooxidase-Hemmern, Glaukom, Hyperthyreose, schwerer Bluthochdruck, vorbestehende zerebrovaskuläre Erkrankungen und das noradrenerge Nebenwirkungsprofil (z.B. Schlaflosigkeit, Nervosität, Kopfschmerzen).

Amantadin, mit NMDA antagonistischer, anticholinerger und indirekt dopaminerger Wirkung wurde in mehreren Studien zu FoG untersucht. Nach inkohärenten Beobachtungen aus großen Kohortenstudien von negativen [43] oder positiven Effekten [27], [44] des Amantadin auf FoG in Pat. mit PK wurden systematische Untersuchungen zu Kurz- und Langzeiteffekten durchgeführt. Zwei randomisierte, placebokontrollierte Studien in 10 bzw. 42 Pat. mit der PK zeigten, dass die intravenöse Applikation von Amantadin (400 mg/Tag) über 2 bzw. 5 Tage keine direkten, dem Placebo überlegenen Effekte auf den Freezing of Gait Questionnaire (FoGQ, [45]), UPDRS oder 4x10m Gehstrecke aufwies [46], [47]. In einer Analyse von zwei Phase-3-, multizentrischen, randomisierten, placebokontrollierten Ease-LID- and EASE-LID-3-Studien [48] zeigte sich, dass 100 Pat. mit der PK und Wirkschwankungen von der oralen, abendlichen Einnahme der retardierten Form von Amantadin (274mg) über 12 Wochen bezüglich der MDS-UPDRS Teil II mehr profitierten als 96 Pat. in der Placebogruppe. Amantadin scheint somit in einer oralen Applikationsform über längere Zeiträume effektiv in der Behandlung des FoG zu sein. Zu berücksichtigen ist das Nebenwirkungsprofil von Amantadin mit Schlafstörungen, Unruhe,

Harnverhalt, Schwindel, neuropsychiatrischen Komplikationen und kardialer QT-Zeit-Verlängerung, welches insbesondere bei älteren Pat. berücksichtigt werden muss.

Koffein als nicht selektiver kompetitiver Adenosin-A1- und Adenosin-A2-Rezeptorblocker wurde in einer kleinen, prospektiven, placebokontrollierten Studie in 16 Pat. mit der PK und FoG mit 100 mg/Tag verblindet über 6 Wochen und anschließend über 18 Monate offen nachuntersucht. Koffein verbesserte spezifisch „akinetisches Freezing" mit im Langzeitverlauf wohl auftretenden Habituationseffekten [49].

Istradefyllin als ein A2A-Rezeptor-Antagonist zeigte in einer kleinen, prospektiven Studie in 14 Pat. mit PK und FoG nach 1 Monat eine signifikante Verbesserung im FoG Q (Baseline 12.14, 1 Monat, 9.79 Punkte) und in einigen, allerdings nicht allen Pat. eine Verbesserung der Gangleistung im Timed Up-and-Go Test [50]. Zu berücksichtigen sind der Off-label-Status in der Parkinson-Therapie und das Nebenwirkungsprofil von Adenosinrezeptor-Antagonisten (Schwindel, Verstopfung, Übelkeit, Halluzination und Schlaflosigkeit).

L-threo-3,4-dihydroxyphenylserin (L-Dops) wurde in Einzelfallbeschreibungen, Fallserien [51], [52] und kleinen, prospektiven, randomisierten Studien in Dosierungen von 300–900 mg/Tag mit heterogenen Ergebnissen [53], [54] untersucht, insbesondere unter einer Kombination mit COMT-Hemmer zeigte sich jedoch eine Verbesserung von FoG innerhalb von 4 Wochen auf einer visuellen Analog-Skala [54]. Mögliche Nebenwirkungen des L-Dops sind Kopfschmerzen, Schwindel, Nausea, Hypertension, Fatigue.

Begründung der Empfehlung

Für Levodopa und die MAO-Hemmer Selegilin und Rasagilin belegen Level-I-Studien die Effektivität der Substanz gegenüber Placebo in der Behandlung von FoG. Die Dopaminagonisten Pramipexol und Ropinirol sind in der Behandlung des FoG dem Levodopa in Level-I-Studien unterlegen, zu berücksichtigen ist hier jedoch das erhöhte Risiko von Wirkfluktuationen unter Levodopa. Alle dopaminergen Substanzen sind zugelassen in der Parkinson-Therapie.

Die Studienlage zu der Anwendung einer cholinergen Medikation in Form von Nikotin (off-label) oder Rivastigmin (on-label für Parkinson-Demenz) zur Behandlung des FoG ist in Level-1-Studien nicht eindeutig, für beide Substanzen ergeben sich aber Hinweise auf eine generell verbesserte Gangqualität. Für Methylphenidat (off-label) liegen drei Level-I-Studien mit jedoch heterogenen Ergebnissen vor, in der größten multizentrischen Studie waren jedoch positive Effekte auf FoG nachweisbar. Für Amantadin (on-label) existiert eine heterogene Studienlage, in retardierter Formulierung zeigten sich in zwei Phase-3-Studien positive Effekte auf FoG. Adenosin-Rezeptorblocker wie Koffein (off-label) oder Istradefyllin (off-label) wurden in kleinen, prospektiven Studien untersucht mit positivem Effekt auf FoG. Da Istradefyllin nicht in Deutschland zugelassen ist, wird von einer Empfehlung abgesehen. Die Anwendung von L-threo-3,4-dihydroxyphenylserin (L-Dops) (off-label) wurde in Fallserien und einer kleinen prospektiven Studie mit heterogenen Ergebnissen untersucht, bei fehlender Zulassung in Deutschland wird von einer Empfehlung hier abgesehen.

Empfehlung	Neu<br>Stand (2023)
▪ Levodopa soll als symptomatische Therapie des FoG angewendet werden.<br>▪ Es wird empfohlen, Levodopa in der Behandlung des FoG den Dopaminagonisten Pramipexol und Ropinirol unter Abwägung des erhöhten Risikos von Dyskinesien vorzuziehen.<br>▪ MAO-B-Hemmer wie Selegilin und Rasagilin werden in der frühen PK zur symptomatischen Behandlung des FoG empfohlen.<br>▪ Die Anwendung cholinerg stimulierender Substanzen wie Nikotin (off-label) oder Rivastigmin (Cave: on-label nur für Demenz) zur Behandlung des FoG können in Erwägung gezogen werden.<br>▪ Der Einsatz der noradrenergen Substanz Methylphenidat (off-label) kann zur Behandlung des FoG in Erwägung gezogen werden.<br>▪ Die kontinuierliche Anwendung von Amantadin in Standardpräparation peroral kann zur Behandlung des FoG in Erwägung gezogen werden, obwohl die Studiendaten für die retardierte Form vorliegen.<br>▪ Adenosinrezeptor-Antagonisten wie Koffein (off-label) können zur Behandlung des FoG in Erwägung gezogen werden.	
Konsensstärke: 91,7%, Konsens	

Wichtige Forschungsfragen:

Die heterogene, fluktuierende und methodisch schwierige Erfassung der FoG-Ausprägung erschwert die quantitative Evaluation von medikamentösen Effekten. In den größeren, multizentrischen Studien wurden meist nur die UPDRS-Items 13 und 14 aus dem UPDRS II gewählt, welche ein eher grobes Maß darstellen. Derzeit werden neue untersucherbasierte FoG-Skalen und Pat.-fokussierte Fragebögen zu FoG und Lebensqualität durch das Internationale FoG Consortium erstellt, um standardisiert das Symptom besser zu erfassen.

3.8.4 Schwangerschaft und Stillzeit

Autoren: Matthias Höllerhage, Günter Höglinger, Claudia Trenkwalder

Fragestellung 86: Wie muss die Pharmakotherapie bei der PK während der Schwangerschaft sicher und wirksam erfolgen?

Evidenzangaben

Es wurde keine randomisierte kontrollierte Studie gefunden, in der die Behandlung der PK während der Schwangerschaft untersucht wurde. In der Literaturrecherche wurden 9 Review-Artikel gefunden, die im Wesentlichen auf Fallberichten bzw. Fallserien aufbauten, sowie eine Pharmakovigilanz-Studie zu dopaminerger Medikation während der Schwangerschaft beim Restless-Legs-Syndrom.

Ergebnis

In der Pharmakovigilanz-Studie aus dem Jahr 2013, in der 59 Schwangerschaften unter Therapie mit Levodopa, Pramipexol, Ropinirol oder Rotigotin eingeschlossen wurden, ergab sich kein Anhalt für ein erhöhtes Fehlbildungsrisiko unter diesen Therapien [55]. In einer Fallserie aus der Türkei wurden bei 4 von 15 Schwangerschaften unter einer Medikation für die PK Komplikationen berichtet. Dabei handelte es sich um Therapien mit Rasagilin plus Pramipexol, Pramipexol als Monotherapie, Piribidil plus Levodopa/Benserazid und Rasagilin plus Levodopa/Benserazid [56]. Es sei darauf verwiesen, dass laut den individuellen Fachinformationen Levodopa/Benserazid in der Schwangerschaft kontraindiziert ist.

Die vorhandenen Daten zu Schwangerschaft bei Pat. mit PK wurden in einem rezenten Review-Artikel zusammengefasst. Demnach gibt es keine Daten, die für eine höhere Komplikationsrate der Schwangerschaft bei Pat. mit einer PK sprechen. Bezüglich der Medikation lagen die meisten Daten für Levodopa vor und es wurden keine erhöhten Missbildungsraten unter Levodopa berichtet [57].

Laut Embrytox ist bei Notwendigkeit einer dopaminergen Medikation Levodopa, am ehesten in Kombination mit Carbidopa akzeptabel. Zu Dopaminagonisten oder MAO-B-Hemmern findet sich dort keine Informationen. Amantadin ist teratogen bei Tier und Mensch.

Begründung der Empfehlung

Die Datenlage zur Therapie der PK in der Schwangerschaft ist begrenzt. Weil noch die meisten Daten für Levodopa vorliegen und Präparate, die den Dopadecarboxylase-Hemmer Benserazid beinhalten, in der Schwangerschaft kontraindiziert sind, sollte bei Notwendigkeit eine Therapie mit Levodopa in Kombination mit Carbidopa erfolgen.

Empfehlung	Neu<br>Stand (2023)
▪ Bei Notwendigkeit einer dopaminergen Medikation in der Schwangerschaft sollte Levodopa in Kombination mit Carbidopa erwogen werden.<br>▪ Aufgrund der unzureichenden Datenlage sollte eine Medikation mit Dopaminagonisten oder MAO-B-Hemmern in der Schwangerschaft vermieden werden.<br>▪ Amantadin und der Decarboxylase-Hemmer Benserazid sind in der Schwangerschaft kontraindiziert.	
Konsensstärke: 92,3%, Konsens	

Stillzeit: Wie muss die Pharmakotherapie bei der PK während der Stillzeit sicher und wirksam erfolgen?

Evidenzangaben

Es existieren keine kontrollierten Studien oder Fallberichte zur medikamentösen Therapie der PK in der Stillzeit.

Ergebnis

Die Datenlage zur medikamentösen Therapie der PK während der Stillzeit lässt keine Empfehlungen zur medikamentösen Therapie während der Stillzeit zu.

Begründung der Empfehlung

Aus pragmatischen Gründen sollte wegen der unzureichenden Datenlage empfohlen werden, dass während der Stillzeit keine medikamentöse Therapie der PK erfolgt oder auf Stillen verzichtet wird.

**Empfehlung**	**Neu**<br>**Stand (2023)**
Aufgrund der unzureichenden Datenlage sollte Stillen unter einer medikamentösen Behandlung der Parkinson-Krankheit vermieden werden.	
Konsensstärke: 100%, starker Konsens	

Referenzen

1. Katus L, Shtilbans A. Perioperative management of patients with Parkinson's disease. Am J Med. 2014;127(4):275-80
2. Pepper PV, Goldstein MK. Postoperative complications in Parkinson's disease. J Am Geriatr Soc. 1999;47(8):967-72
3. Wüllner U, Standop J, Kaut O, Coenen V, Kalenka A, Wappler F. [Parkinson's disease. Perioperative management and anesthesia]. Anaesthesist. 2012;61(2):97-105
4. Mehanna R, Jankovic J. Respiratory disorders associated with dystonia. Mov Disord. 2012;27(14):1816-9
5. Mueller MC, Jüptner U, Wuellner U, Wirz S, Türler A, Hirner A, Standop J. Parkinson's disease influences the perioperative risk profile in surgery. Langenbecks Arch Surg. 2009;394(3):511-5
6. Reichmann H. [Perioperative Management of PD Patients]. Fortschr Neurol Psychiatr. 2016;84 Suppl 1:S14-7
7. Hu MT, Bland J, Clough C, Ellis CM, Chaudhuri KR. Limb contractures in levodopa-responsive parkinsonism: a clinical and investigational study of seven new cases. J Neurol. 1999;246(8):671-6
8. Sixel-Döring F, Rausch-Hertel M, Trenkwalder C. Severe Contractions in Parkinson's Disease Caused by Immobilization and Lack of Dopaminergic Treatment. The Internet Journal of Neurology. 2007;10(1)
9. Jost ST, Kaldenbach MA, Antonini A, Martinez-Martin P, Timmermann L, Odin P, et al. Levodopa Dose Equivalency in Parkinson's Disease: Updated Systematic Review and Proposals. Mov Disord. 2023;38(7):1236-52
10. Olanow CW, Kieburtz K, Odin P, Espay AJ, Standaert DG, Fernandez HH, et al. Continuous intrajejunal infusion of levodopa-carbidopa intestinal gel for patients with advanced Parkinson's disease: a randomised, controlled, double-blind, double-dummy study. Lancet Neurol. 2014;13(2):141-9
11. Bonnici A, Ruiner CE, St-Laurent L, Hornstein D. An interaction between levodopa and enteral nutrition resulting in neuroleptic malignant-like syndrome and prolonged ICU stay. Ann Pharmacother. 2010;44(9):1504-7

12. Wüllner U, Kassubek J, Odin P, Schwarz M, Naumann M, Häck HJ, et al. Transdermal rotigotine for the perioperative management of Parkinson's disease. J Neural Transm (Vienna). 2010;117(7):855-9
13. Kim HJ, Jeon BS, Lee WY, Lee MC, Kim JW, Kim JM, et al. Overnight switch from ropinirole to transdermal rotigotine patch in patients with Parkinson disease. BMC Neurol. 2011;11:100
14. LeWitt PA, Boroojerdi B, MacMahon D, Patton J, Jankovic J. Overnight switch from oral dopaminergic agonists to transdermal rotigotine patch in subjects with Parkinson disease. Clin Neuropharmacol. 2007;30(5):256-65
15. Chung SJ, Kim JM, Kim JW, Jeon BS, Singh P, Thierfelder S, et al. Switch from oral pramipexole or ropinirole to rotigotine transdermal system in advanced Parkinson's disease: an open-label study. Expert Opin Pharmacother. 2015;16(7):961-70
16. Dafotakis M, Sparing R, Juzek A, Block F, Kosinski CM. Transdermal dopaminergic stimulation with rotigotine in Parkinsonian akinetic crisis. J Clin Neurosci. 2009;16(2):335-7
17. Chen JJ, Obering C. A review of intermittent subcutaneous apomorphine injections for the rescue management of motor fluctuations associated with advanced Parkinson's disease. Clin Ther. 2005;27(11):1710-24
18. Gálvez-Jiménez N, Lang AE. Perioperative problems in Parkinson's disease and their management: apomorphine with rectal domperidone. Can J Neurol Sci. 1996;23(3):198-203
19. Kornhuber J, Weller M, Riederer P. Glutamate receptor antagonists for neuroleptic malignant syndrome and akinetic hyperthermic parkinsonian crisis. J Neural Transm Park Dis Dement Sect. 1993;6(1):63-72
20. Kim YE, Kim HJ, Yun JY, Jeon BS. Intravenous amantadine is safe and effective for the perioperative management of patients with Parkinson's disease. J Neurol. 2011;258(12):2274-5
21. Fahn S, Oakes D, Shoulson I, Kieburtz K, Rudolph A, Lang A, et al. Levodopa and the progression of Parkinson's disease. N Engl J Med. 2004;351(24):2498-508
22. Rascol O, Fitzer-Attas CJ, Hauser R, Jankovic J, Lang A, Langston JW, et al. A double-blind, delayed-start trial of rasagiline in Parkinson's disease (the ADAGIO study): prespecified and post-hoc analyses of the need for additional therapies, changes in UPDRS scores, and non-motor outcomes. Lancet Neurol. 2011;10(5):415-23
23. Verschuur CVM, Suwijn SR, Boel JA, Post B, Bloem BR, van Hilten JJ, et al. Randomized Delayed-Start Trial of Levodopa in Parkinson's Disease. N Engl J Med. 2019;380(4):315-24
24. Gilat M, Lígia Silva de Lima A, Bloem BR, Shine JM, Nonnekes J, Lewis SJG. Freezing of gait: Promising avenues for future treatment. Parkinsonism Relat Disord. 2018;52:7-16
25. Nonnekes J, Snijders AH, Nutt JG, Deuschl G, Giladi N, Bloem BR. Freezing of gait: a practical approach to management. Lancet Neurol. 2015;14(7):768-78
26. Gao C, Liu J, Tan Y, Chen S. Freezing of gait in Parkinson's disease: pathophysiology, risk factors and treatments. Transl Neurodegener. 2020;9:12
27. Giladi N, Treves TA, Simon ES, Shabtai H, Orlov Y, Kandinov B, et al. Freezing of gait in patients with advanced Parkinson's disease. J Neural Transm (Vienna). 2001;108(1):53-61
28. Schaafsma JD, Balash Y, Gurevich T, Bartels AL, Hausdorff JM, Giladi N. Characterization of freezing of gait subtypes and the response of each to levodopa in Parkinson's disease. Eur J Neurol. 2003;10(4):391-8
29. Fahn S. Does levodopa slow or hasten the rate of progression of Parkinson's disease? J Neurol. 2005;252 Suppl 4:Iv37-iv42
30. Holloway RG, Shoulson I, Fahn S, Kieburtz K, Lang A, Marek K, et al. Pramipexole vs levodopa as initial treatment for Parkinson disease: a 4-year randomized controlled trial. Arch Neurol. 2004;61(7):1044-53
31. Rascol O, Brooks DJ, Korczyn AD, De Deyn PP, Clarke CE, Lang AE. A five-year study of the incidence of dyskinesia in patients with early Parkinson's disease who were treated with ropinirole or levodopa. N Engl J Med. 2000;342(20):1484-91

32. Giladi N, McDermott MP, Fahn S, Przedborski S, Jankovic J, Stern M, Tanner C. Freezing of gait in PD: prospective assessment in the DATATOP cohort. Neurology. 2001;56(12):1712-21
33. Shoulson I, Oakes D, Fahn S, Lang A, Langston JW, LeWitt P, et al. Impact of sustained deprenyl (selegiline) in levodopa-treated Parkinson's disease: a randomized placebo-controlled extension of the deprenyl and tocopherol antioxidative therapy of parkinsonism trial. Ann Neurol. 2002;51(5):604-12
34. Rascol O, Brooks DJ, Melamed E, Oertel W, Poewe W, Stocchi F, Tolosa E. Rasagiline as an adjunct to levodopa in patients with Parkinson's disease and motor fluctuations (LARGO, Lasting effect in Adjunct therapy with Rasagiline Given Once daily, study): a randomised, double-blind, parallel-group trial. Lancet. 2005;365(9463):947-54
35. Lieberman A, Lockhart TE, Olson MC, Smith Hussain VA, Frames CW, Sadreddin A, et al. Nicotine Bitartrate Reduces Falls and Freezing of Gait in Parkinson Disease: A Reanalysis. Front Neurol. 2019;10:424
36. Henderson EJ, Lord SR, Brodie MA, Gaunt DM, Lawrence AD, Close JC, et al. Rivastigmine for gait stability in patients with Parkinson's disease (ReSPonD): a randomised, double-blind, placebo-controlled, phase 2 trial. Lancet Neurol. 2016;15(3):249-58
37. Auriel E, Hausdorff JM, Herman T, Simon ES, Giladi N. Effects of methylphenidate on cognitive function and gait in patients with Parkinson's disease: a pilot study. Clin Neuropharmacol. 2006;29(1):15-7
38. Pollak L, Dobronevsky Y, Prohorov T, Bahunker S, Rabey JM. Low dose methylphenidate improves freezing in advanced Parkinson's disease during off-state. J Neural Transm Suppl. 2007(72):145-8
39. Devos D, Krystkowiak P, Clement F, Dujardin K, Cottencin O, Waucquier N, et al. Improvement of gait by chronic, high doses of methylphenidate in patients with advanced Parkinson's disease. J Neurol Neurosurg Psychiatry. 2007;78(5):470-5
40. Espay AJ, Dwivedi AK, Payne M, Gaines L, Vaughan JE, Maddux BN, et al. Methylphenidate for gait impairment in Parkinson disease: a randomized clinical trial. Neurology. 2011;76(14):1256-62
41. Delval A, Moreau C, Bleuse S, Guehl D, Bestaven E, Guillaud E, et al. Gait and attentional performance in freezers under methylphenidate. Gait Posture. 2015;41(2):384-8
42. Jankovic J. Atomoxetine for freezing of gait in Parkinson disease. J Neurol Sci. 2009;284(1-2):177-8
43. Macht M, Kaussner Y, Möller JC, Stiasny-Kolster K, Eggert KM, Krüger HP, Ellgring H. Predictors of freezing in Parkinson's disease: a survey of 6,620 patients. Mov Disord. 2007;22(7):953-6
44. Malkani R, Zadikoff C, Melen O, Videnovic A, Borushko E, Simuni T. Amantadine for freezing of gait in patients with Parkinson disease. Clin Neuropharmacol. 2012;35(6):266-8
45. Giladi N, Tal J, Azulay T, Rascol O, Brooks DJ, Melamed E, et al. Validation of the freezing of gait questionnaire in patients with Parkinson's disease. Mov Disord. 2009;24(5):655-61
46. Kim YE, Yun JY, Yang HJ, Kim HJ, Gu N, Yoon SH, et al. Intravenous amantadine for freezing of gait resistant to dopaminergic therapy: a randomized, double-blind, placebo-controlled, cross-over clinical trial. PLoS One. 2012;7(11):e48890
47. Lee JY, Oh S, Kim JM, Kim JS, Oh E, Kim HT, et al. Intravenous amantadine on freezing of gait in Parkinson's disease: a randomized controlled trial. J Neurol. 2013;260(12):3030-8
48. Hauser RA, Mehta SH, Kremens D, Chernick D, Formella AE. Effects of Gocovri (Amantadine) Extended-Release Capsules on Motor Aspects of Experiences of Daily Living in People with Parkinson's Disease and Dyskinesia. Neurol Ther. 2021;10(2):739-51
49. Kitagawa M, Houzen H, Tashiro K. Effects of caffeine on the freezing of gait in Parkinson's disease. Mov Disord. 2007;22(5):710-2

50. Matsuura K, Kajikawa H, Tabei KI, Satoh M, Kida H, Nakamura N, Tomimoto H. The effectiveness of istradefylline for the treatment of gait deficits and sleepiness in patients with Parkinson's disease. Neurosci Lett. 2018;662:158-61
51. Ogawa N, Kuroda H, Yamamoto M, Nukina I, Ota Z. Improvement in freezing phenomenon of Parkinson's disease after DL-threo-3, 4-dihydroxyphenylserine. Acta Med Okayama. 1984;38(3):301-4
52. Teelken AW, van den Berg GA, Muskiet FA, Staal-Schreinemachers AL, Wolthers BG, Lakke JP. Catecholamine metabolism during additional administration of DL-threo-3,4-dihydroxyphenylserine to patients with Parkinson's disease. J Neural Transm Park Dis Dement Sect. 1989;1(3):177-88
53. Tohgi H, Abe T, Takahashi S. The effects of L-threo-3,4-dihydroxyphenylserine on the total norepinephrine and dopamine concentrations in the cerebrospinal fluid and freezing gait in parkinsonian patients. J Neural Transm Park Dis Dement Sect. 1993;5(1):27-34
54. Fukada K, Endo T, Yokoe M, Hamasaki T, Hazama T, Sakoda S. L-threo-3,4-dihydroxyphenylserine (L-DOPS) co-administered with entacapone improves freezing of gait in Parkinson's disease. Med Hypotheses. 2013;80(2):209-12
55. Dostal M, Weber-Schoendorfer C, Sobesky J, Schaefer C. Pregnancy outcome following use of levodopa, pramipexole, ropinirole, and rotigotine for restless legs syndrome during pregnancy: a case series. Eur J Neurol. 2013;20(9):1241-6
56. Tüfekçioğlu Z, Hanağası H, Yalçın Çakmaklı G, Elibol B, Esmeli Tokuçoğlu F, Kaya ZE, et al. Use of anti-Parkinson medication during pregnancy: a case series. J Neurol. 2018;265(8):1922-9
57. Young C, Phillips R, Ebenezer L, Zutt R, Peall KJ. Management of Parkinson's Disease During Pregnancy: Literature Review and Multidisciplinary Input. Mov Disord Clin Pract. 2020;7(4):419-30

3.9 Schmerz

Autoren: Christian Winkler, Carsten Buhmann

Fragestellung 87: Wie unterscheiden sich Schmerzen bei Parkinson-Pat. im Vergleich zu Menschen ohne PK in Bezug auf Häufigkeit, Lebensqualität und Risikofaktoren?

Hintergrund zum Thema Schmerz bei Parkinson

Schmerzen gehören zu den häufigen nicht motorischen Beschwerden bei Vorliegen einer PK. Die Ätiologie und der Phänotyp von Schmerzen bei Parkinson-Pat. sind komplex, oft multifaktoriell und werden – selbst in Fällen mit scheinbar nicht mit Parkinson zusammenhängenden Schmerzen, z. B. aufgrund einer Arthrose der Wirbelsäule oder der Gelenke – durch motorische oder nicht motorische Symptome verstärkt [1]. Entsprechend ist eine exakte ätiologische Zuordnung der Schmerzen oft schwierig oder nicht möglich.

Evidenzgrundlage

Epidemiologische Daten zu Schmerzen in unterschiedlichen Stadien der PK liegen vor. Die Bewertung dieser Daten ist eingeschränkt, weil kaum Fragebögen vorliegen bzw. verwendet werden, die für Schmerzen bei der PK validiert sind. Ebenso gibt es keine einheitliche Einschätzung unter Expertinnen/Experten über die Einteilung der Schmerz-Subtypen bei der PK. Zur Therapie von Schmerzen bei der PK liegen vereinzelt Ergebnisse in kontrollierten Studien vor, wobei der Schmerz zumeist nicht der primäre Endpunkt der Studien war. Vergleichende Untersuchungen zur Wertigkeit unterschiedlicher Therapeutika zur Reduktion von Schmerzen bei der PK liegen nicht vor, da die Studien zumeist Placebo bzw. einen „standard of care“ als Kontrolle verwendeten. Die Bewertung der Fragen erfolgte daher überwiegend anhand von Übersichtsartikeln/Metaanalysen.

Fragestellung 88: Wie unterscheiden sich Schmerzen bei Parkinson-Pat. im Vergleich zu Menschen ohne Parkinson in Bezug auf Häufigkeit, Lebensqualität und Risikofaktoren?

Ergebnis

Schmerzen bei der PK sind häufig [2], überwiegend chronisch [3] und beeinflussen die Lebensqualität gemäß zahlreichen Studien negativ [4], [5], [6]. Die Prävalenz von Schmerzen wird je nach untersuchten Pat.-Populationen, verwendeten Studiendesigns und Adressierung unterschiedlicher Schmerztypen mit 68–95 % der Parkinson-Pat. angegeben [1]. Schmerzen können bei einem Teil der Pat. bereits frühzeitig vor oder während der Diagnose einer PK vorliegen [7]. Im Verlauf der Erkrankung berichtet dann der Großteil der Pat. über das Vorliegen von Schmerzen [8], [3], [9], [10]. Die Schmerzen sind damit vermutlich häufiger als in der allgemeinen Population, wo in verschiedenen Studien die Prävalenz von gelegentlichen oder regelmäßigen Schmerzen von Menschen über 65 Jahren mit 50–80% angegeben wird. Weibliches Geschlecht, Dyskinesien, Haltungsstörungen, motorische Komplikationen und Depression wurden als Prädiktoren für Schmerzen bei Parkinson gefunden (Übersicht in [11]).

Begründung der Empfehlung

Die Empfehlung erfolgte anhand von unkontrollierten Studien, Übersichtsartikeln und Metaanalysen.

Empfehlung	Neu<br>Stand (2023)
Das Vorliegen von Schmerzen bei der PK soll erfragt werden, weil sie sehr häufig vorhanden sind und die Lebensqualität negativ beeinflussen.	
Konsensstärke: 100%, starker Konsens	

Fragestellung 89: Welche Schmerz-Subtypen können bei der PK auftreten?

Ergebnis

Eine allgemeingültige und unzweifelhafte Einteilung von Schmerzen bei der PK in spezielle Unterkategorien existiert nicht.

Lange Zeit wurde die Klassifikation der Schmerzen bei Parkinson nach Ford am häufigsten verwendet, welche unterscheidet zwischen muskuloskelettalen, radikulären/neuropathischen, dystoniebedingten und akathischen sowie zentralen Schmerzen [12], [13]. Wasner und Deuschl schlugen eine vierstufige Taxonomie vor, um die Klassifizierung von Schmerzen bei Parkinson zu verbessern. Diese Taxonomie ordnet nozizeptive, neuropathische und sonstige Schmerzen verschiedenen Kategorien zu und führt eine weitere Charakterisierung in Unterkategorien durch [14]. Ein kürzlich vorgeschlagenes Schmerzklassifizierungssystem für Parkinson (PD-PCS) soll eine Unterscheidung von Parkinson-abhängigen und Parkinson-unabhängigen Schmerzen anhand von vier Fragen ermöglichen, bevor eine weitere Unterteilung anhand der Kriterien der International Association for the Study of Pain (IASP) erfolgt. In den IASP-Kriterien wurden den bekannten Kategorien der neuropathischen und nozizeptiven Schmerzen mit dem noziplastischen Schmerz ein neu beschriebener Mechanismus hinzugefügt [15]. 2019 wurde die Bezeichnung noziplastischer Schmerz erstmals für zentrale Schmerzen und das Restless-Legs-Syndrom bei der PK verwendet [16], jedoch bislang nicht in Studien zu Schmerz bei Parkinson verwendet. Das PD-PCS-System ist jedoch unter Berücksichtigung noziplastischer Schmerzen bei der PK validiert [10].

Nach der PD-PCS-Klassifizierung gelten als Parkinson-assoziiert Schmerzen, die früh mit den motorischen Symptomen auftreten, auf dopaminerge Medikation ansprechen oder durch die PK verstärkt werden, einschließlich von Off-Phasen assoziierter Schmerzen. Diese Schmerzen können nozizeptiven, noziplastischen oder neuropathischen Charakter aufweisen [17], [18]. Diese werden abgegrenzt von Schmerzen, die unabhängig von der PK auftreten. Demnach könnten Schmerzen, die bei Zunahme einer Rigidität ebenfalls zunehmen oder die durch Parkinson-Medikamente verbessert werden, als Parkinson-bedingt oder Parkinson-abhängig bezeichnet werden. Berücksichtigt werden sollten in jedem Fall Schmerzen, die abhängig von motorischen Fluktuationen auftreten und häufig mit einer Dystonie assoziiert sind, sowie vom Pat. als innen liegend oder in den Eingeweiden lokalisierte viszerale Schmerzen. Auch unangenehme und zum Teil schmerzhafte Empfindungen in den Extremitäten wie bei Ödemen oder in Verbindung mit einer Bewegungsunruhe und nächtlicher Häufung wie bei Restless-Legs-Syndrom sollten erfragt werden. Von der PK weniger abhängige Schmerzen wären demnach die ebenfalls häufig auftretenden degenerativen Schmerzen in Gelenken

und der Wirbelsäule, wobei auch diese gelegentlich durch Parkinson-Medikamente verbessert werden können.

Übereinstimmung herrscht darüber, dass muskuloskelettale Schmerzen der häufigste Schmerztyp bei der Parkinson-Krankheit sind. Diese nozizeptiven Schmerzen werden in epidemiologischen Untersuchungen bei 40–80% der Pat. berichtet [19], [8], [9], [20]. Neuropathische Schmerzen werden in einer Größenordnung zwischen 10 und 30% beschrieben [9], [10]. Mylius et al. ordnen die Häufigkeit der Parkinson-assoziierten noziplastischen Schmerzen mit 22% zwischen den nozizeptiven (55%) und den neuropathischen (16%) Schmerzen ein [17], [18].

Begründung der Empfehlung

Die Empfehlung erfolgte anhand des neuen PD-PCS-Klassifizierungssystems für Schmerzen bei Parkinson [10]

Empfehlung	Neu<br>Stand (2023)
▪ Bei der PK soll zwischen Parkinson-assoziierten und Nicht-Parkinson-assoziierten Schmerzen unterschieden werden.<br>▪ Bei den Parkinson-assoziierten Schmerzen sollte zwischen nozizeptiven, noziplastischen und neuropathischen Schmerzen unterschieden werden.	
Konsensstärke: 100%, starker Konsens	

Fragestellung 90: Welche Skalen/Fragebögen sind geeignet, Schmerzen bei Parkinson-Pat. zu erfassen?

Ergebnis

Die Erfassung von Schmerzen bei der PK ist bedeutsam, weil sie häufig auftreten und die Lebensqualität von Pat. stark beeinflussen. Viele Pat. sehen ihre Schmerzen nicht im Zusammenhang mit ihrer PK und berichten diese deshalb oft nicht, was eine Ursache dafür sein könnte, dass Neurologen im Gegensatz zu Orthopäden oder Allgemeinmedizinern in der Behandlung des Schmerzes eine untergeordnete Rolle im Praxisalltag spielen [3]. Deshalb sollte gezielt nach Schmerz gefragt werden, wobei folgende Subtypen berücksichtigt werden sollten: muskuloskelettale Schmerzen, neuropathische Schmerzen, fluktuationsabhängige und dystone Schmerzen, viszerale Schmerzen (oft als tief innen liegend bezeichnet) und Extremitätenschmerzen bei Ödemen oder Restless-Legs-Syndrom.

Idealerweise sollten die Erfassung und die Quantifizierung mittels Skalen oder Fragebögen optimiert werden.

In den meisten Parkinson-Skalen finden Schmerzen nur wenig Berücksichtigung. In der Unified Parkinson Disease Rating Scale (UPDRS) wird unter Item 17 nach „Sensorischen Beschwerden infolge von Parkinsonismus“ gefragt und unter Item 34, inwieweit Dyskinesien schmerzhaft sind. Auch im

neueren UPDRS der Movement Disorder Society (MDS-UPRDS) wird wenig nach Schmerzen gefragt (Item 1.9: Schmerz und andere Empfindungen; Item 4.6: Schmerzhafte Dystonie im Off-Zustand).

In der Non-Motor Symptom Scale (NMSS) wird lediglich unter Item 27 nach Schmerzen gefragt, die nicht durch andere Erkrankungen erklärt werden können. In der neueren MDS Non-Motor Rating Scale (MDS-NMS) werden Schmerzen ausführlicher berücksichtigt mit einer Unterteilung in Schmerzen in Muskeln/Gelenken/Rücken versus tiefliegenden oder dumpfen Körperschmerzen versus Dystonie-bedingte Schmerzen versus Schmerzen durch andere Ursachen wie nächtlicher Schmerz oder Gesichtsschmerzen. Ergänzend kann in der MDS-NMS dokumentiert werden, inwieweit und in welcher Ausprägung sich Schmerzen durch einen Wechsel vom On ins Off verändern.

Derzeit stehen zwei Fragebögen zur Verfügung, die für eine Verwendung bei Parkinson-Pat. validiert sind: die King’s Parkinson’s disease Pain Scale (KPPS) [19], [21] und das Parkinson’s disease pain classification system (PD-PCS) [21] [18]. In der KPPS werden Schmerzen zunächst in 7 Kategorien unterteilt (muskuloskelettal, tief im Körper sitzende Schmerzen, fluktuationsabhängige Schmerzen, nächtliche Schmerzen, Gesichtsschmerzen, Schmerzen durch Ödeme, radikuläre Schmerzen), bevor eine weitere Charakterisierung anhand von 14 Unterkategorien erfolgt. Im PD-PCS wird zunächst ein Zusammenhang von Schmerzen mit der PK erfragt, bevor eine weitere Unterteilung der Schmerzen erfolgt. Ein Zusammenhang der Schmerzen mit der PK wird dabei entsprechend des PD-PCS angenommen, wenn zumindest einer der nachfolgenden Aspekte bejaht wird: Schmerz in Zusammenhang mit Beginn der PK, Schmerzverstärkung bei Zunahme von Rigor/Bradykinesie/Tremor, Schmerzen in Zusammenhang mit motorischen Fluktuationen, Schmerzreduktion nach Einnahme von Parkinson-Medikamenten.

Begründung der Empfehlung:

Die Empfehlung basiert auf o.g. Daten.

**Empfehlung: Welche Skalen/Fragebögen sind geeignet, Schmerzen bei Parkinson-Pat. zu erfassen?**	**Neu**<br>**Stand (2023)**
Zur Schmerzerfassung bei Parkinson-Pat. sollten folgende Skalen/Fragebögen verwendet werden:<br>1. Schmerzerfassung und Schmerzkategorisierung: Parkison’s disease pain classification system (PD-PCS)<br>2. Schmerzerfassung und Quantifizierung: King’s Parkinon’s disease Pain Scale (KPPS)	
Konsensstärke: 100%, starker Konsens	

Fragestellung 91: Wie werden Schmerzen bei der PK therapiert?

Ergebnis

In jedem Fall sollte in der Praxis versucht werden, die Schmerzen gemäß ihrer Ätiologie zu klassifizieren (mechanism-based classification approach), um sie dann entsprechend gezielt therapieren zu können.

Die Optimierung der dopaminergen oder anderen Anti-Parkinson-Therapie ist essenziell, um die Parkinson-assoziierten Schmerzen zu verbessern. Dies schließt auch die Therapie der schmerzhaften Dystonien und des zu den noziplastischen Schmerzen bei Parkinson gehörenden Restless-Legs-Syndroms mit ein.

Unabhängig davon, ob Parkinson-assoziiert oder nicht, sollte die Therapie der nozizeptiven Schmerzen nach dem WHO-3-Stufen-Plan zur Schmerztherapie erfolgen. Die Therapie der neuropathischen Schmerzen richtet sich nach den Leitlinien zur Therapie des neuropathischen Schmerzes bei anderen Erkrankungen.

Abbildung 1 zeigt einen Vorschlag für einen Therapie-Algorithmus zur Behandlung von Schmerzen bei Parkinson-Pat. [1].

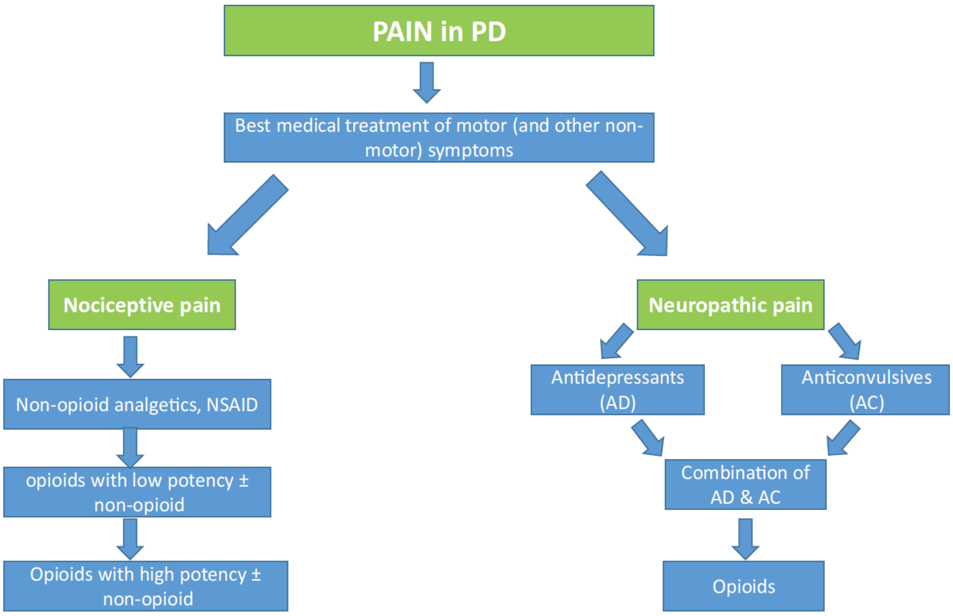

Figure 1. Propoal of a treatment algorithm for pain in PD patients. Nociceptive pain according to WHO three-step pain treatment scheme.

Für die Therapie von Schmerzen bei der PK gibt es keine einheitliche Vorgehensweise, da die Schmerzen bei Pat. qualitativ und quantitativ sehr unterschiedlich sein können und weil es keine vergleichenden kontrollierten Studien gibt. In kontrollierten und unkontrollierten Studien zu einzelnen Wirkstoffen waren Schmerzen zumeist nicht der primäre Endpunkt, oft erfolgte keine Analyse nach unterschiedlichen Schmerzsubtypen, und als Kontrolle diente zumeist ein Placebo bzw. ein „Standard of care". Für einige Wirkstoffe konnten in Studien schmerzreduzierende Effekte nachgewiesen werden (Pramipexol, Rotigotin, Safinamid; zusammengefasst in [22], [1]). Auch weiterführende Therapien wie kontinuierliche Pumpentherapien mit Apomorphin oder Levodopa bzw. die Tiefe Hirnstimulation im Nucleus subthalamicus konnten zu einer Reduktion von speziellen Schmerzsubtypen führen (zusammengefasst in [1]). Es ist davon auszugehen, dass ein Teil der Schmerzreduktion bei den klassischen Anti-Parkinson-Medikamenten zurückzuführen ist auf eine verbesserte dopaminerge Stimulation bzw. eine Reduktion von motorischen Fluktuationen, sodass auch durch weitere Wirkstoffe wie Levodopa und COMT-Hemmer eine schmerzreduzierende Behandlung versucht werden kann.

Tab. 17 gibt einen Überblick über die wenigen größeren RCTs zu Anti-Parkinson-Medikamenten und Opioden zur Therapie von Schmerzen bei Parkinson-Pat. [1].

Table 1
Shows larger randomized controlled trials (RCT) of antiparkinsonian drugs and opioids assessing the effect on pain in PD patients

Treatment	Study design and applied scales for pain	Patients & Methods	Results Means with (SD), (range) or (CI)	References
Pramipexole	RCT, VAS-P	138 vs. 148 controls	–3,3 (–8.9 to 0.3) vs. –2.4 (–8.9 to 2.6) Group comparison –1.3 (–3.3 to 0.8) *p* = 0.19	Barone et al. 2010 [96]
Safinamide	RCT (*post hoc*), PDQ-39	440 vs. 438 controls	reduction of the number of concomitant pain treatments of 23.6% [5% confidence interval (CI): 41.1%, 1.0%; *p* = 0.0421] PDQ-39: item 37 (–0.26 vs. –0.07; *p* = 0.0009), item 38 (0,19 vs. 0.1; *p* = 0.1585), item 39 (–0.18 vs. –0.03; *p* = 0.0060)	Cattaneo et. al. 2017 [63]
Entacapone	RCT, PDQ-39	281 vs. 274 controls	(0.04) vs. 0.1 (0.04); *p* = 0.9. SMD = 1.81, *p* < 0.0001	Olanow et al. [64]
Oxycodone	RCT, KPPS	88 vs. 106 controls	5.0 (95% CI 4.5 to 5.5) versus 5.6 (5.1 to 6.0). Difference –0.6, 95% CI –1.3 to 0.0; *p* = 0.058).	Trenkwalder et al. [78]
Pardoprunox	RCT (*post-hoc*), VAS-P	140 vs. 133 controls	During OFF time –2.2 (2.7) vs. –1.0 (2.7), no p-values given During ON time –2.3 (2.8) vs. –0.5 (3.0), no *p*-values given	Rascol et al. [65]
Rotigotine	RCT, Likert pain scale	178 vs. 88 controls	–0.9 (SD 2.2) vs. –0.1 (SD 2.3), *p* = 0.004 Difference –0.77 [–1.28 to –0.25]	Trenkwalder et al. [72]
	RCT (*post-hoc*), Likert pain scale	178 vs. 88 controls	'any pain': –0.88 [95% CI: –1.56, –0.19], *p* = 0.013) 'moderate-to-severe' pain: (–1.38 [–2.44, –0.31], *p* = 0.012 UPDRS III or PDSS-2 responders showed greater improvement in pain than non-responders	Kassubek et al. [73]

Tab. 18 listet Studien niedrigerer Evidenz bezüglich der Behandlungseffekte von Parkinson-Medikamenten auf Schmerz bei Parkinson-Pat. (Klasse C III oder IV)

- **Rotigotin**
  - RECOVER; post-hoc Analyse [1]
    - Prädiktoren für Therapieansprechen: höherer Schmerzlevel, Verbesserung in UPDRS III und PDSS-2 durch Rotigotin Behandlung
  - NEUPAD; nicht-interventionelle multizentrische Beobachtungsstudie über 4 Wochen [2]
    - nicht-signifikante Reduktion der "affective dimension" von Schmerz (wahrscheinlich in Abhängigkeit von der motorischen Verbesserung)
- **Safinamid**
  - post-hoc-Analyse der Studien 016 und SETTLE (placebo-kontrolliert) über 24 Wochen (Safinamid N=498) [3]
    - Verbesserung in 2 von 3 Domänen der physischen Einschränkungen (musculoskelettaler und neuropathischer Schmerz) im PDQ-39
    - Reduzierte Häufigkeit von begleitenden Schmerztherapien
  - post-hoc-Analyse der Studie 018 (placebo-kontrolliert) über 2 Jahre (Safinamid N=180) [4]
    - Signifikante Verbesserung der PDQ-39 items 37 (painful cramps or spasm) und 39 (unpleasantly hot or cold)
    - Significant reduzierte Anzahl an begleitender Schmerztherapie vs. Baseline und vs. Plazebo
  - open-label prospektive Single Center Studie über 12 Wochen (N=12) [5]
    - Signifikante Verbesserung in King's Pain Scale for Parkinson's Disease (KPPS) und Brief Pain Inventory (BPI) Intensity and Interference
  - open-label prospektive Multicenter Studie über 6 Monate (SAFINOMOTOR; Phase IV); N=50 [6]
    - Verbesserung der Lebensqualität mit Signifikanz für PDQ-39SI-8 (Schmerz und Unwohlsein)
    - Signifikante Verbesserung auf der King´s Parkinson Pain scale (KPPS); -43,6% (p < 0,0001)
- **Levodopa-Carbidopa Intestinal Gel -** GLORIA; internationale postmarketing-Studie [7]
  - weniger schmerzhafte Dyskinesien (nach 12 Mo. Behandlung) und Muskelkrämpfe (nach 6 Mo.)

1 Kassubek et al., BMC Neurol 2014
2 Timmermann et al., Postgrad Med 2017
3 Cattaneo et al., J Parkinsons Dis 2017
4 Cattaneo et al., Adv Ther 2018
5 Geroin et al., J Neural Transm 2020
6 Garcia et al., Brain Sci. 2021
7 Antonini et al., Relat Disord 2015

Tab. 19 listet Studien niedriger Evidenz zu Effekten anderer Medikamente auf Schmerzen bei Parkinson-Pat. auf

- Nicht-opioide Analgetika
  - Keine Studien
- Duloxetin (Antidepressiva)
  - Open-label; N=20, 6 Wochen; Reduktion von zentralem und peripherem neuropathischen Schmerz [1]
- Gabapentin (Antikonvulsiva)
  - Keine Studien, aber möglicherweise bevorzugt anzuwenden bei neuropathischen Schmerz und Parkinson, da
  - 2 RCTs zeigen einen geringen positiven Effekt auf motorische Symptome bei Parkinson [2,3]
- BoNT
  - 2 RCT´s; Incobotulinum toxin A (N=45, schmerzhafte dystone Plantarflexion der Zehen) und Onabotulinumtoxin A (N=12; Gliederschmerzen) [4]
  - In beiden Studien: Schmerz Reduktion in BoNT Gruppe vs. baseline, aber nicht vs. Kontrollgruppe mit Placebo Injektion [5]
- Cannabinoide
  - 2 offene unkontrollierte Beobachtungsstudien (n=22 and n=20) [6,7]
    - Schmerzreduktion gemessen mit verschiedenen Scores 30min nach Rauchen von 0,5 – 1,0 g. Marihuana
  - 1 Placebo- kontrollierte Studie (Nabilon)
  - 2 Fragegebogen-gestützte nationale Studien in der Parkinson Community (verschiedene Cannabinoide)

1 Djaldetti et al., Clin Neuropharmacol. 2007
2 Olson et al., Am J Med 1997
3 Van Blercom et al. Clin. Neuropharmacol 2004
4 Rieu et al., Parkinsonism Relat Disord 2018
5 Bruno et al., Can J Neurol Sci 2018
6 Lotan et al., Clin Neuropharmacol 2014
7 Shohet et al., Eur J Pain 2017

Begründung der Empfehlung

Auf der Basis der oben dargestellten Evidenzgrundlage werden zum Aspekt Schmerzen bei der PK folgende Empfehlungen gegeben.

Empfehlung	Neu<br>Stand (2023)
▪ Ausgeprägte chronische Schmerzen bei Parkinson sollten analog anderer Schmerzerkrankungen multimodal interdisziplinär behandelt werden.<br>▪ Die Therapie sollte gemäß der Ätiologie der Schmerzen erfolgen.<br>▪ Basis der Therapie ist die Optimierung der Anti-Parkinson-Medikation, wobei zur Behandlung Dopamin-abhängiger Schmerzen Levodopa, Dopaminagonisten, COMT-Hemmer oder Safinamid eingesetzt werden können.<br>▪ Nozizeptive Schmerzen sollten gemäß des 3-Stufen-WHO-Schemas behandelt werden.<br>▪ Neuropathische Schmerzen sollten mit Antikonvulsiva und/oder Antidepressiva gemäß der Leitlinie zur Behandlung neuropathischer Schmerzen behandelt werden, wobei bevorzugt Gabapentin und/oder Duloxetin (insbesondere bei Komorbidität mit Depression) eingesetzt werden sollten.<br>▪ Bei ausgeprägten Schmerzen kann eine Behandlung mit retardiertem Oxycodon/Naloxon in Erwägung gezogen werden.	
Konsensstärke: 100%, starker Konsens	

Referenzen

1. Buhmann C, Kassubek J, Jost WH. Management of Pain in Parkinson's Disease. J Parkinsons Dis. 2020;10(s1):S37-s48
2. Schapira AHV, Chaudhuri KR, Jenner P. Non-motor features of Parkinson disease. Nat Rev Neurosci. 2017;18(7):435-50
3. Buhmann C, Wrobel N, Grashorn W, Fruendt O, Wesemann K, Diedrich S, Bingel U. Pain in Parkinson disease: a cross-sectional survey of its prevalence, specifics, and therapy. J Neurol. 2017;264(4):758-69
4. Silverdale MA, Kobylecki C, Kass-Iliyya L, Martinez-Martin P, Lawton M, Cotterill S, et al. A detailed clinical study of pain in 1957 participants with early/moderate Parkinson's disease. Parkinsonism Relat Disord. 2018;56:27-32
5. Lubomski M, Davis RL, Sue CM. Health-Related Quality of Life for Parkinson's Disease Patients and Their Caregivers. J Mov Disord. 2021;14(1):42-52
6. Martinez-Martin P, Deuschl G. Effect of medical and surgical interventions on health-related quality of life in Parkinson's disease. Mov Disord. 2007;22(6):757-65
7. Lin CH, Wu RM, Chang HY, Chiang YT, Lin HH. Preceding pain symptoms and Parkinson's disease: a nationwide population-based cohort study. Eur J Neurol. 2013;20(10):1398-404
8. Valkovic P, Minar M, Singliarova H, Harsany J, Hanakova M, Martinkova J, Benetin J. Pain in Parkinson's Disease: A Cross-Sectional Study of Its Prevalence, Types, and Relationship to Depression and Quality of Life. PLoS One. 2015;10(8):e0136541
9. Martinez-Martin P, Manuel Rojo-Abuin J, Rizos A, Rodriguez-Blazquez C, Trenkwalder C, Perkins L, et al. Distribution and impact on quality of life of the pain modalities assessed by the King's Parkinson's disease pain scale. NPJ Parkinsons Dis. 2017;3:8
10. Mylius V, Perez Lloret S, Cury RG, Teixeira MJ, Barbosa VR, Barbosa ER, et al. The Parkinson disease pain classification system: results from an international mechanism-based classification approach. Pain. 2021;162(4):1201-10
11. Buhmann C, Ip CW, Oehlwein C, Tönges L, Wolz M, Reichmann H, Kassubek J. [Parkinson Disease and Pain - diagnostic and therapeutic approaches to a challenging non-motor symptom]. Fortschr Neurol Psychiatr. 2018;86(S 01):S48-s58
12. Ford B. Pain in Parkinson's disease. Clin Neurosci. 1998;5(2):63-72
13. Ford B. Pain in Parkinson's disease. Mov Disord. 2010;25 Suppl 1:S98-103
14. Wasner G, Deuschl G. Pains in Parkinson disease--many syndromes under one umbrella. Nat Rev Neurol. 2012;8(5):284-94
15. IASP (2021) *https://www.iasp-pain.org/resources/terminology/-nociplastic-pain*. Zugegriffen: Juni 2023.
16. Marques A, Attal N, Bouhassira D, Moisset X, Cantagrel N, Rascol O, et al. How to diagnose parkinsonian central pain? Parkinsonism Relat Disord. 2019;64:50-3
17. Mylius V, Möller JC, Bohlhalter S, Ciampi de Andrade D, Perez Lloret S. Diagnosis and Management of Pain in Parkinson's Disease: A New Approach. Drugs Aging. 2021;38:559-77
18. Mylius V, Perez Lloret S, Brook CS, Krüger MT, Hägele-Link S, Gonzenbach R, et al. [The new Parkinson's disease pain classification system (PD-PCS)]. Nervenarzt. 2022;93(10):1019-27
19. Chaudhuri KR, Rizos A, Trenkwalder C, Rascol O, Pal S, Martino D, et al. King's Parkinson's disease pain scale, the first scale for pain in PD: An international validation. Mov Disord. 2015;30(12):1623-31
20. Rodriguez-Blazquez C, Schrag A, Rizos A, Chaudhuri KR, Martinez-Martin P, Weintraub D. Prevalence of Non-Motor Symptoms and Non-Motor Fluctuations in Parkinson's Disease Using the MDS-NMS. Mov Disord Clin Pract. 2021;8(2):231-9
21. Jost WH, Rizos A, Odin P, Löhle M, Storch A. [King's Parkinson's disease pain scale : Intercultural adaptation in the German language]. Nervenarzt. 2018;89(2):178-83
22. Karnik V, Farcy N, Zamorano C, Bruno V. Current Status of Pain Management in Parkinson's Disease. Can J Neurol Sci. 2020;47(3):336-43

3.10 Dysautonomie

Autoren/Autorinnen: Florian Krismer, Alessandra Fanciulli, Dirk Woitalla, Wolfgang Jost, Claudia Trenkwalder

Fragestellung 92: Welche Blasenfunktionsstörungen können bei der PK auftreten?

Hintergrund

Beschwerden des unteren Harntrakts sind ein häufiges nicht motorisches Zeichen der PK und lassen sich anhand der Harnblasenfunktion in eine Füll- oder Speicherphase sowie eine Entleerungs- bzw. Miktionsphase untergliedern [1], [2], [3]. Als neurogene Blasenfunktionsstörungen werden alle Beeinträchtigungen der Blasenfunktion bezeichnet, die ein neurologisches Korrelat aufweisen. Gerade bei älteren Pat. müssen nicht neurogene von neurogenen Blasenstörungen abgegrenzt werden. Abhängig von der zugrunde liegenden Störung kommt es zum Auftreten unterschiedlicher klinischer Symptome. Bei einer Detrusorüberaktivät finden sich eine Pollakisurie, eine Nykturie, ein häufig bis ständig vorhandenes und äußerst unangenehmes Harndranggefühl („Urge“-Symptomatik) und eine Dranginkontinenz. Die Detrusor-Sphinkter-Dyssynergie führt häufig zu einem unterbrochenen Harnstrahl und Startschwierigkeiten und kann in einer Restharnbildung enden. Der hypokontraktile Detrusor resultiert in einem schwachen Harnstrahl und dem Restharngefühl. Ein hypoaktiver Sphinkter führt unmittelbar bei Anstieg des abdominellen Drucks zu einem unwillkürlichen Harnverlust. Eine Kenntnis über mögliche Blasenfunktionsstörungen im Rahmen der PK sind für eine adäquate Pat.-Versorgung unerlässlich.

Evidenzgrundlage

Die Diagnose der neurogenen Blasenstörung ist in den Leitlinien der DGN [2] und der EAU [4] dargelegt. Zudem wird das Themengebiet in systematischen Übersichtsartikeln [5], epidemiologischen Studien [6] und Expert/Expertinnen-Stellungnahmen behandelt [3], [7].

Ergebnis

Bei der PK finden sich zumeist Beschwerden der Füll- bzw. Speicherphase, die in Zusammenhang mit einer Detrusorüberaktivität stehen [2]. Als Symptome finden sich daher eine Nykturie, ein häufiges bis ständig vorhandenes und äußerst unangenehmes Harndranggefühl („Urge“-Symptomatik), und eine Harndranginkontinenz [6], [8]. Die Häufigkeit und Ausprägung der Beschwerden ist abhängig vom Alter und dem Krankheitsstadium [6], [8]. Eine rezente Metaanalyse schätzt die gepoolte Prävalenz von Beschwerden des unteren Harntrakts bei Parkinson-Pat. auf 61% (95% KI 53–69; I2 = 99%; 27 Studien; n = 5,179), wobei in höheren Hoehn-&-Yahr-Stadien die Beschwerden häufiger auftreten [5]. Die Prävalenz der Harninkontinenz bei Parkinson-Pat. betrug in dieser Metaanalyse 30% (95% CI 22-39; 21 Studien). Etwas seltener wurde das Vorliegen einer Harnretention berichtet (27% (17-37; 14 Studien; n = 1,991)).

In der weiterführenden Diagnostik gilt es, im engen Austausch mit urologischen und gynäkologischen Kolleginnen/Kollegen nicht neurogene Blasenstörungen von neurogenen Blasenstörungen abzugrenzen (bspw. Prostata-Hyperplasie beim Mann oder Beckenbodenprolaps bei Frauen). Neben

einer urologischen/gynäkologischen Konsiliaruntersuchung ist zudem eine Harnuntersuchung zum Ausschluss reversibler/behandelbarer Ursachen (Harnwegsinfekt) und als Screening-Untersuchung für raumfordernde Erkrankungen, Proteinurie bzw. Diabetes mellitus unerlässlich. Das Führen eines Blasentagebuchs ermöglicht die Bestimmung der Miktionsfrequenz, der Harnmenge und von Episoden mit Harndrang(-inkontinenz) [9] und erleichtert dadurch die Interpretation erweiterter diagnostischer Maßnahmen. Letztere können eine Restharnmessung mittels Blasenultraschall oder Einmalkatheterismus, die Uroflowmetrie zur Bestimmung der Harnflussrate, des Miktionsvolumen und der -zeit und die Urodynamik sein. Letztere gilt als der Goldstandard in der Abklärung und Zuordnung der neurogenen Blasenstörung.

**Empfehlung**	**Neu**<br>**Stand (2023)**
▪ Bei Pat. mit der PK finden sich zumeist Beschwerden, die der Blasenfüllphase bzw. -speicherphase zugeordnet werden können und sich als Nykturie, und/oder ein häufiges bis ständig vorhandenes und äußerst unangenehmes Harndranggefühl („Urgency“) und/oder eine Harndranginkontinenz präsentieren können.	
Konsensstärke: 100%, starker Konsens	

Fragestellung 93: Wie werden Blasenfunktionsstörungen bei der PK therapiert?

Hintergrund

Neurogene Blasenfunktionsstörungen finden sich (in Abhängigkeit vom Krankheitsstadium) bei etwa 60% aller Pat. mit einer PK [5]. Die Beschwerden betreffen zumeist die Blasenfüll- bzw. -speicherphase, wodurch es zum Auftreten einer Pollakisurie, einer Nykturie, zu einem häufig bis ständig vorhandenen und äußerst unangenehmen Harndranggefühl (Urge-Symptomatik) und einer Dranginkontinenz kommen kann. Die Beschwerden beeinträchtigen die Lebensqualität der Pat. und bedürfen häufig (nach eingehender Abklärung) einer medikamentösen Therapie.

Evidenzgrundlage

Die Behandlung der neurogenen Blasenstörungen bei Parkinson-Pat. basieren auf Expertenmeinungen und sind in den Leitlinien der einschlägigen Fachgesellschaften und systematischen Übersichtsartikeln/Metaanalysen dargestellt [10], [2], [4], [3].

Ergebnis

Die vordergründigen Therapieziele der neurogenen Blasenfunktionsstörung bei Pat. mit der PK sind die Verbesserung der Lebensqualität und die Verhinderung von Sekundärschädigung der oberen Harnwege durch Verminderung der Episoden mit Inkontinenz und Verbesserung der Miktionskontrolle [3], [11]. Nicht medikamentöse Therapiemaßnahmen wie Blasentraining und Verhaltensmaßnahmen (die tageszeitliche Anpassung der Trinkmenge, Vermeidung von Koffein, Alkohol und kohlensäurehaltigen Getränken) können Beschwerden im Zuge der neurogenen Blasenstörung lindern [12], [13], [14]. Zur Vermeidung einer sozialen Stigmatisierung durch die Blaseninkontinenz ist die Anwendung von Kontinenzhilfen hilfreich (z.B. Kondomurinal, Einlagen) [2].

Antimuskarinika, darunter Oxybutinin, Tolterodin, Solifenacin, Darifenacin und Trospium, sind die Medikamente der ersten Wahl zur Behandlung des Harndrangs und der Harndranginkontinenz aufgrund von Blasendetrusorüberaktivität. Sowohl Solifenacin [15] als auch Fesoterodin [16] und Oxybutinin [17] wurden in randomisierten klinischen Studien untersucht, an denen auch Pat. mit der PK teilnahmen. Eine von der International Parkinson's Disease and Movement Disorders Society (MDS) beauftragte Task Force bewertete jedoch nur Solifenacin als möglicherweise nützliches Antimuskarinikum für die Behandlung der Symptome einer überaktiven Blase bei Parkinson-Pat. [10]. Mögliche Nebenwirkungen von Antimuskarinika sind: erhöhte Restharnmengen, Xerostomie, Obstipation und Akkomodationsstörungen [18]. Delir, Verwirrtheit und eine Verschlechterung einer vorbestehenden kognitiven Einschränkung sind weitere Nebenwirkungen, die auf die zentrale anticholinerge Wirkung zurückzuführen sind und die Anwendung von Anticholinergika bei Pat. mit Parkinson Demenz einschränken. Trospium, welches die Blut-Hirn-Schranke nicht durchdringt, und Darifenacin, welches selektiv den peripheren M3-Muskarinrezeptor-Subtyp stimuliert, rufen weniger häufig kognitive Nebenwirkungen hervor und können gegebenenfalls bevorzugt werden [19], [20]. Das Restharnvolumen sollte während der Titrationsphase und nach Dosisänderungen von Antimuskarinika kontrolliert werden, da dieses aufgrund einer medikamentös induzierten Blasendetrusorschwäche ansteigen kann [21].

Mirabegron, ein B3-Rezeptoragonist, wurde in randomisierten kontrollierten Studien bei Pat. mit einer Detrusorüberaktivität und neurologischen Erkrankungen einschließlich der PK untersucht [22], [23]. Eine Metaanalyse dieser Studien fand heraus, dass eine Therapie mit Mirabegron zu einer signifikanten Linderung der Beschwerden einer überaktiven Blase führt und ein akzeptables Nebenwirkungsprofil aufweist [24]. Mirabegron war – in einem indirekten Vergleich mit anti-muskarinerg wirkenden Medikamenten – seltenerer mit Auftreten von Harnretention, Verstopfung, kognitiver Beeinträchtigung und einem trockenen Mund vergesellschaftet. Angesichts des günstigen Sicherheitsprofils von Beta-3-Rezeptor-Agonisten können diese als Alternative für Pat. in Betracht gezogen werden, bei denen Antimuskarinika kontraindiziert sind oder nicht vertragen wurden [25], [26], [14]. Beta-3-Rezeptoragonisten können auch Pat. mit suboptimalem Ansprechen auf Antimuskarinika angeboten werden [27].

In refraktären Fällen können auch invasivere Optionen in Betracht gezogen werden. Die intravesikale Injektion von Botulinumtoxin A kann Inkontinenz-Episoden aufgrund von Detrusorüberaktivität verringern [28]. Die Behandlung reduziert die Detrusorkontraktilität, was die willentliche Entleerung beeinträchtigt und zu einem Harnverhalt führt. Daher ist dieses Verfahren nur bei Pat. indiziert, die in der Lage sind, einen sauberen intermittierenden Selbstkatheterismus durchzuführen, und nicht beispielsweise bei Pat. mit schweren kognitiven oder motorischen Störungen [29], [30].

Neuere Erkenntnisse deuten darauf hin, dass die perkutane [31] und transkutane [32], [33] Stimulation des Nervus tibialis posterior die Symptome der überaktiven Blase und die urodynamischen Befunde bei Parkinson-Pat. verbessern kann.

Bei Pat., die über eine Nykturie berichten, soll anhand der Aufzeichnungen des Blasentagebuchs unterschieden werden, ob eine erhöhte nächtliche Polyurie (d.h. mehr als 1/3 des 24h-Gesamtharnvolumens wird über Nacht ausgeschieden – z.B. bei Drucknatriurese bei Pat. mit

nächtlicher Hypertonie), eine allgemein erhöhte Harnausscheidung (>40 ml/kg Körpergewicht – z.B. bei unkontrolliertem Diabetes) beziehungsweise eine verminderte Blasenkapazität (z.B. bei überaktiver Blase) den Nykturie-Episoden zugrunde liegt. Eine Kombination von mehr als einer Ursache ist häufig. Im Rahmen der Erstabklärung sollten außerdem eine Reihe anderer Faktoren berücksichtigt werden, die zu einer Nykturie beitragen können. Dazu gehören: nächtliche (Liegend-)Hypertonie, die sich bei ca. 50% der Parkinson-Pat. mit orthostatischer Hypotonie findet, abendliche Einnahme von Diuretika, Schlafapnoe, psychogene Polydipsie und unzureichende Sekretion des antidiuretischen Hormons (entweder idiopathisch, medikamentös bedingt oder aufgrund hypophysärer Läsionen).

Zur nicht pharmakologischen Behandlung der Nykturie sollte den Pat. geraten werden, die Flüssigkeitsaufnahme am späten Nachmittag/Abend einzuschränken, abendlichen Alkoholkonsum zu vermeiden und eine Oberkörperhochlagerung um 10–20° während des Schlafens anzustreben [34]. Eine pharmakologische Behandlung mit Desmopressin zur Bettzeit kann bei Pat. mit nächtlicher Polyurie unter engmaschiger Blutdruck-, Serumelektrolyten- und Körpergewichtkontrolle angedacht werden [35]. Die abendliche Verabreichung von Antimuskarinika kann bei Pat. mit Nykturie aufgrund einer verminderten Blasenkapazität in Betracht gezogen werden.

**Empfehlung**	**Neu**<br>**Stand (2023)**
Als nicht medikamentöse Therapiemaßnahmen bei der neurogenen Blasenstörung im Rahmen der PK sollten das Blasentraining, die tageszeitliche Anpassung der Flüssigkeitsaufnahme und die Vermeidung von Koffein, Alkohol und kohlensäurehaltigen Getränken angewendet werden.	
Konsensstärke: 100%, starker Konsens	

**Empfehlung**	**Neu**<br>**Stand (2023)**
Zur Vermeidung einer sozialen Stigmatisierung durch die Blaseninkontinenz sollte die Anwendung von Kontinenzhilfen empfohlen werden.	
Konsensstärke: 100%, starker Konsens	

**Empfehlung**	**Neu**<br>**Stand (2023)**
Antimuskarinika (Solifenacin, Trospium, Darifenacin) sollten zur medikamentösen Behandlung des vermehrten Harndrangs und der Harndranginkontinenz aufgrund einer Überaktivität des Blasendetrusors bei Parkinson-Pat. erwogen werden.	
Konsensstärke: 100%, starker Konsens	

**Empfehlung**	**Neu**<br>**Stand (2023)**
B3-Rezeptor-Agonisten (bspw. Mirabegron) können zur Behandlung des vermehrten Harndrangs und der Harndranginkontinenz aufgrund von Blasendetrusorüberaktivität bei jenen Parkinson-Pat. erwogen werden, die unzureichend auf Antimuskarinika angesprochen haben, die Antimuskarinika nicht vertragen haben oder bei denen Antimuskarinika kontraindiziert sind.	
Konsensstärke: 100%, starker Konsens	

**Empfehlung**	**Neu**<br>**Stand (2023)**
Zur Behandlung der Detrusorüberaktivität kann bei Pat., die auf orale Therapieversuche unzureichend angesprochen haben, die intravesikale Injektion von Botulinumtoxin A erwogen werden, sofern das motorische und kognitive Leistungsniveau der Pat. einen nachfolgend nötigen, sauberen intermittierenden Selbstkatheterismus erlaubt.	
Konsensstärke: 100%, starker Konsens	

**Empfehlung**	**Neu**<br>**Stand (2023)**
Zur nicht pharmakologischen Behandlung der Nykturie sollte Pat. geraten werden, die Flüssigkeitsaufnahme am späten Nachmittag/Abend einzuschränken, abendlichen Alkoholkonsum zu vermeiden und eine Oberkörperhochlagerung um 10–20° während des Schlafens anzustreben.	
Konsensstärke: 100%, starker Konsens	

**Empfehlung**	**Neu**<br>**Stand (2023)**
Zur Therapie der nächtlichen Polyurie kann unter engmaschiger Blutdruck-, Serumelektrolyt- und Körpergewichtskontrollen eine abendliche Therapie mit Desmopressin erwogen werden.	
Konsensstärke: 100%, starker Konsens	

**Empfehlung**	**Neu**<br>**Stand (2023)**
Zur Therapie der Nykturie aufgrund verminderter Blasenkapazität sollte bei Parkinson-Pat. eine Therapie mit Antimuskarinika (Solifenacin, Trospium, Darifenacin) erwogen werden.	
Konsensstärke: 100%, starker Konsens	

Fragestellung 94: Wie wird erektile Dysfunktion bei der PK diagnostiziert?

Hintergrund

Die erektile Dysfunktion ist definiert als die Unfähigkeit, eine penile Erektion, die für einen befriedigenden Geschlechtsverkehr ausreicht, zu erreichen und aufrechtzuerhalten (NIH Consensus Conference, 1993). Eine Erektionsstörung hat einen großen Einfluss auf die Lebensqualität und das Wohlbefinden von Pat. Die Häufigkeit einer erektilen Dysfunktion nimmt exponentiell in höherem Alter zu [36]. Eine Abnahme der Libido, eine erektile Dysfunktion und Einschränkungen in der Ejakulation finden sich bei bis zu 80% der männlichen Parkinson-Pat. [8].

Evidenzgrundlage

Die Diagnose der erektilen Dysfunktion ist in der einschlägigen Leitlinie der DGN dargelegt [37].

Ergebnis

Die Diagnostik der erektilen Dysfunktion liegt heute primär in der Hand der Urologinnen/Urologen. Eine ausführliche Anamneseerhebung ist zwingend erforderlich, um modifizierbare Ursachen und Einflussfaktoren zu detektieren. Insbesondere ist eine ausführliche Medikamentenanamnese erforderlich, um eine medikamentös bedingte erektile Dysfunktion, z.B. aufgrund einer Behandlung mit SSRI oder β-Blocker, zu erkennen. Auch sollten sowohl ein neurologischer als auch ein urologischer Status erhoben werden und Anzeichen einer peripher arteriellen Verschlusskrankheit beachtet werden. Eine Laboruntersuchung sollte kardiovaskuläre Risikofaktoren (Nüchternglukose, HbA1c, Lipidstatus) und häufige Ursachen einer Polyneuropathie (Nieren- und Lebefunktionsparameter, Serumproteinelektrophorese, Vitamin B1, B6, B12 und Folsäurespiegel) umfassen und bei Pat. mit Gynäkomastie eine Hormonbestimmung (Testosteron) beinhalten. Bei Verdacht auf eine arterielle Minderversorgung kann, in Einzelfällen, ein Provokationstest (+/- Gefäßsonographie) mit intrakavernöser Applikation von Alprostadil erforderlich sein. Ergänzende elektrophysiologische Untersuchungen (bspw. N. pudendus SSEP) können indirekte Hinweise auf eine neurogene Genese liefern [37], [4].

**Empfehlung**	**Neu**<br>**Stand (2023)**
Die Diagnosestellungen bei Pat. mit Verdacht auf erektile Dysfunktion soll in interdisziplinärer Zusammenarbeit mit Kolleginnen/Kollegen der Urologie erfolgen. Die Basisdiagnostik umfasst eine ausführliche Anamnese, eine klinisch-neurologische und klinisch-urologische Untersuchung sowie eine Labordiagnostik. Gegebenenfalls kann eine Zusatzdiagnostik erforderlich werden; aus neurologischer Sicht spielen primär elektrophysiologische Untersuchungen eine Rolle.	
Konsensstärke: 100%, starker Konsens	

Fragestellung 95: Wie wird erektile Dysfunktion bei der PK therapiert?

Hintergrund

Eine Erektionsstörung kann Lebensqualität und Wohlbefinden von Pat. deutlich vermindern. Die Häufigkeit der erektilen Dysfunktion nimmt exponentiell mit dem Alter zu [36]. Eine Abnahme der Libido, eine erektile Dysfunktion und Ejakulationseinschränkungen finden sich bei bis zu 80% der männlichen Parkinson-Pat. [8] und stellen somit ein signifikantes Behandlungsproblem dar.

Evidenzgrundlage

Die Behandlung der erektilen Dysfunktion bei Parkinson-Pat. basiert auf Expertenmeinungen und ist in den Leitlinien der einschlägigen Fachgesellschaften und systematischen Übersichtsartikeln dargelegt [10], [37], [4].

Ergebnis

In systematischen Übersichtsartikeln wurde die Wirksamkeit der oralen Phosphodiesterase 5 (PDE-5)-Hemmer (Sildenafil, Tadalafil, Vardenafil) eingehend untersucht [38], [10]. Sildenafil wurde in 4 hochqualitativen Studien bei 75 männlichen Pat. untersucht [39], [40], [41], [42] und die Wirksamkeit auf die erektile Dysfunktion bei Parkinson-Pat. hinreichend belegt. Mit Hinblick auf die Arzneimittelsicherheit gilt es, mögliche nachteilige kardiovaskuläre Folgen und eine Aggravation einer orthostatischen Hypotonie zu berücksichtigen. Ein systematischer Review, der einen indirekten Wirksamkeitsvergleich der Wirksamkeit zwischen den unterschiedlichen PDE-Hemmern (Sildenafil, Tadalafil, Vardenafil) anstrebte, legt nahe, dass alle PDE-5-Hemmer gleichermaßen wirksam sind [43]. PDE-5-Hemmer mit einer langen Halbwertszeit (Tadalafil) sollten bei Pat. mit nachgewiesener orthostatischer Hypertonie allerdings mit Vorsicht angewendet werden, da die Gefahr des Auftretens einer lang anhaltenden und häufig behandlungsbedürftigen arteriellen Hypotonie besteht. Eine intraurethrale oder intrakavernöse Applikation von vasodilatorisch wirksamen Prostaglandinen kann in Betracht gezogen werden, wenngleich es bislang keine Untersuchungen bei Parkinson-Pat. gibt und ein erhöhtes Risiko für das Auftreten eines Priapismus und von Blutungen besteht [44], [4]. Fallserien und eine multizentrische doppelblinde Studie lassen einen positiven Therapieeffekt von Apomorphin (sublingual oder subkutan appliziert) auf die erektile Dysfunktion vermuten [45], [46]. Mechanische Erektionshilfen wurden in Einzelfällen angewandt; entsprechende Hilfsmittel stellen nur für einen kleinen Teil der Pat. eine akzeptable therapeutische Alternative dar [4].

**Empfehlung**	**Neu**<br>**Stand (2023)**
Zur medikamentösen Therapie der erektilen Dysfunktion bei Parkinson-Pat. mit einem geringen kardiovaskulären Risiko während des Geschlechtsverkehrs sollte eine Therapie mit PDE-5-Hemmer (Sildenafil) erwogen werden. Eine mögliche Demaskierung/Aggravation einer orthostatischen Hypotonie ist in Betracht zu ziehen.	
Konsensstärke: 100%, starker Konsens	

Fragestellung 96: Wie wird orthostatische Hypotonie bei der PK diagnostiziert?

Hintergrund

Die neurogene orthostatische Hypotonie ist ein häufiges nicht motorisches Zeichen der PK mit einer geschätzten Prävalenz von 30.1% (95% KI: 22.9–38.4%, [47]). Die orthostatische Hypotonie kann in jedem Krankheitsstadium auftreten, wenngleich die Häufigkeit mit fortgeschrittenem Alter und längerer Krankheitsdauer zunimmt [48]. Meist werden seitens der Pat. unspezifische Symptome und Beschwerden, wie das Auftreten eines lageabhängigen Schwindels und das Gefühl einer Benommenheit, berichtet. Bei einem Teil der Pat. kommt es zum Auftreten von transienten Bewusstseinsverlusten (Synkopen). Die neurogene orthostatische Hypotonie ist somit eine häufige Sturzursache und ein beträchtlicher Risikofaktor für das Eintreten von Komorbiditäten. Pathophysiologisch wird bei der orthostatischen Hypotonie, bedingt durch die PK, eine Denervation peripherer sympathetischer Fasern angenommen. Internistische Begleiterkrankungen (unter anderem Infektionskrankheiten, Dehydratation und kardiale Erkrankungen) können eine orthostatische Hypotonie bei Parkinson-Pat. demaskieren oder exazerbieren. Zudem können häufig eingesetzte Parkinson-Medikamente Symptome einer orthostatischen Hypotonie aggravieren.

Evidenzgrundlage

Die Definition der orthostatischen Hypotonie erfolgte auf Basis eines Expertenkonsensus [49], [50]. Handlungsempfehlungen zur Diagnostik bei Synkopen wurden durch eine Expertenkommission der European Society of Cardiology (ESC) erstellt [50] und werden in der einschlägigen Leitlinie der DGN zusammengefasst [51].

Ergebnis

Die orthostatische Hypotonie ist als Abfall des systolischen Blutdrucks um mindestens 20 mmHg oder des diastolischen Blutdrucks um mindestens 10 mmHg innerhalb von drei Minuten nach dem Aufstehen oder während einer Kipptisch-Untersuchung (mit einer Lageänderung um zumindest 60° aus der Liegendposition) definiert. Alternativ lässt sich eine orthostatische Hypotonie durch einen Abfall des systolischen Blutdrucks unter 90 mmHg im Stehen (unabhängig von der Differenz des Blutdruckabfalls) diagnostizieren [49], [50]. Im Expertenkonsensus werden zwei Methoden zur Feststellung der orthostatischen Hypotonie zugelassen und sollten für die Diagnose der orthostatischen Hypotonie bei Parkinson-Pat. erwogen werden: der aktive Stehversuch („Schellong-Test“, Messung in der Liegendposition und minütlich im Stehen für zumindest 3 Minuten) und die Kipptisch-Untersuchung (mit Aufrichtung um zumindest 60°). Die für die PK charakteristische neurogene Genese lässt sich aufgrund einer Pulsstarre bzw. durch Bestimmung der neurogenen orthostatischen Hypotonie-Ratio identifizieren [50], [52], [53].

Empfehlung	Neu<br>Stand (2023)
Zur Diagnose einer orthostatischen Hypotonie bei Pat. mit der PK soll ein Schellong-Test durchgeführt werden. Eine Kipptisch-Untersuchung kann bei Verfügbarkeit erwogen werden, da diese eine umfassendere Aussagekraft aufweist.	
Konsensstärke: 100%, starker Konsens	

Fragestellung 97: Wie wird orthostatische Hypotonie bei der PK therapiert?

Hintergrund

Die orthostatische Hypotonie ist als Abfall des systolischen Blutdrucks um mindestens 20 mmHg oder des diastolischen Blutdrucks um mindestens 10 mmHg im Stehen innerhalb von drei Minuten nach dem Aufstehen im Vergleich zu den Ruhewerten im Liegen definiert. Mit einem Abfall des systolischen Blutdrucks auf unter 90 mmHg im Stehen (unabhängig von der Differenz des Blutdruckabfalls) gelten die Kriterien der orthostatischen Hypotonie ebenfalls als erfüllt [49], [50]. Die für die PK charakteristische neurogene Genese lässt sich mit der Pulsstarre bzw. der nOH-Ratio identifizieren [50], [54], [52], [53]. Die orthostatische Hypotonie stellt ein häufiges Begleitsymptom der PK mit einer Prävalenz von etwa 30% dar [47] und kann durch häufig eingesetzte Parkinson-Medikamente (bspw. Levodopa) oder internistische Komorbiditäten (Infekte, kardiale Erkrankungen, etc.) demaskiert, aggraviert oder exazerbiert werden. Durch die orthostatische Hypotonie kann es zum Auftreten transienter Bewusstseinsverluste (Synkopen) kommen, die mitunter schwerwiegende Folgeerkrankungen nach sich ziehen. Die Therapie der orthostatischen Hypotonie stellt eine besondere Herausforderung dar.

Evidenzgrundlage

Die Behandlung der symptomatischen orthostatischen Hypotonie basiert auf Expertenmeinungen und ist in den Leitlinien der einschlägigen Fachgesellschaften, in systematischen Übersichtsartikeln und hochrangig publizierten Expertenmeinungen ausgeführt [50], [55], [56], [10], [51].

Ergebnis

Die Expertenempfehlungen basieren auf Studien, die in der überwiegenden Mehrheit ein gemischtes Pat.-Kollektiv untersuchten (Pat. mit orthostatischer Hypotonie unterschiedlichster Genese) und häufig nur eine geringe Fallzahl umfassten. Auch entspricht das Studiendesign zumeist nicht den qualitativen Anforderungen an eine Zulassungsstudie, wodurch die Empfehlungen überwiegend auf der klinischen Erfahrung der Expertinnen/Experten basiert.

In der Therapie der orthostatischen Hypotonie bei der PK ist es empfehlenswert, ein Stufenschema anzuwenden [50], [55], [56]. Zunächst sollten exazerbierende Faktoren für eine orthostatische Hypotonie (Infektionskrankheiten, kardiale Begleiterkrankungen, Dehydratation, Kombination mehrerer blutdrucksenkenden Substanzen in der Prämedikation) ausgeschlossen bzw. behandelt werden.

In einem weiteren Schritt können nicht pharmakologische Therapiemaßnahmen erwogen werden. Das rasche Trinken von kaltem Wasser führte in zumindest sechs Studien zu einer signifikanten Steigerung des Blutdrucks, die etwa 30 Minuten anhielt [57], [58], [59], [60]. Ein länger anhaltender Effekt (>45 Minuten) ist anhand der vorliegenden Studienlage nicht zu erwarten [61], [62]. Eine übermäßige Salzzufuhr hatte keinen additiven Effekt in drei Studien, die dies untersuchten [63], [64], [65], und kann somit bei ausreichender Hydratation nicht als Zusatzmaßnahme empfohlen werden. Nach dem Verzehr üppiger Mahlzeiten kommt es zu einem signifikanten Blutdruckabfall [66], wodurch es ratsam ist, mehrere kleinere Mahlzeiten über den Tag verteilt zu sich zu nehmen. Das Schlafen mit erhöhter Kopfposition in Kombination mit einer medikamentös induzierten Volumenexpansion durch Fludrocortison wurde in Open-label-Studien untersucht und in beiden Studien konnten ein Blutdruckanstieg sowie eine verbesserte Toleranz auf orthostatische Belastungen gezeigt werden [67], [68]. Physikalische Gegenmaßnahmen (in die Hocke gehen oder Kreuzen der Beine sowie die Anspannung der Bein-, Gesäß-, Bauch- und Armmuskeln) in der Prodromalphase einer Synkope vermögen es, den Blutdruck zu stabilisieren [69], [70], [71], [72]. Das Tragen einer abdominellen Kompressionsbinde führte in zwei randomisierten Cross-over-Studien und zwei unabhängigen Fallserien zu einer Anhebung des Blutdrucks und einer Linderung der OH-Beschwerden [73], [74], [72], [69]. Das Tragen von Kompressionsstrümpfen war in zwei kleinen Studien [75], [76] anderen (nicht medikamentösen) Therapiemaßnahme unterlegen, weshalb auf einen Therapieversuch mit Kompressionsstrümpfen verzichtet werden kann.

Medikamentös wurde Midodrin in mehreren randomisierten Doppelblind-Studien mit unterschiedlichem Studiendesign und unterschiedlichen Komparatoren untersucht und es konnte die Wirksamkeit von Midodrin etabliert werden [77], [78], [79], [80], [81], [82]. Sicherheitsbedenken gibt es in Hinblick auf den therapieinduzierten Liegendhypertonus und die Harnretention. Parästhesien, generalisierter oder vorwiegend die Kopfhaut betreffender Pruritus, Piloerektion und Schüttelfrost sind relativ häufige und teils unangenehme unerwünschte Arzneimittelwirkungen von Midodrin.

Fludrocortison kann als weitere Therapieoption erwogen werden, wenngleich die Evidenz hierfür spärlich ist und die Empfehlung auf Grundlage der klinischen Erfahrung erfolgt. Zwei randomisierte, doppelt verblindete Crossover-Studien mit kleinen Fallzahlen können als belastbare Evidenz angeführt werden [83], [80], wobei in einer der beiden Studien etwa die Hälfte der Pat. als Therapieversager klassifiziert wurde und somit häufige Reevaluationen des Therapieeffekts vonnöten sind [80]. In Hinblick auf die Arzneimittelsicherheit ist auch bei Fludrocortison ein therapieinduzierter Liegendhypertonus zu berücksichtigen. Des Weiteren können unter Therapie mit Fludrocortison Elektrolytstörungen auftreten.

Für eine Therapie mit Droxidopa gibt es Evidenz aus sechs randomisierten, kontrollierten Studien bei Pat. mit der PK, der Multisystematrophie und dem reinen autonomen Versagen. Vier dieser Studien [84], [85], [86], [87] konnten einen positiven Therapieeffekt einer Behandlung mit Droxidopa belegen, bei zwei Studien wurde der primäre Endpunkt verfehlt [88], [89]. Einige weitere, in ihrer Fallzahl deutlich kleinere, klinische Studien und Fallserien unterstützen die Beobachtung eines positiven Therapieeffekts von Droxidopa [90], [91], [92], [93], [94], [95], [96], [97]. Auch gibt es Ergebnisse aus einer Langzeitbeobachtung, die ein akzeptables Langzeitnebenwirkungsprofil nahelegen [98]. Aufgrund dieser Studienlage erfolgte in den USA eine Zulassung durch die Food and

Drug Administration; in Europa ist Droxidopa für die Behandlung der orthostathischen Hypotonie durch die EMA nicht formal zugelassen. Vergleichbar mit den anderen pharmakologischen Interventionen ist mit einem Liegendhypertonus bei mit Droxidopa behandelten Pat. zu rechnen. Weitere kleinere Fallserien zu Therapien mit Atomoxetin, Yohimbin, Pyridostigmin wurden veröffentlicht [55]. Die klinische Anwendung letzterer Medikamente ist häufig jedoch auf spezialisierte Dysautonomie-Zentren beschränkt.

**Empfehlung**	**Neu**<br>**Stand (2023)**
Zur Therapie der orthostatischen Hypotonie bei Pat. mit der PK sollte folgendes Stufenschema angewendet werden:<br>1. aggravierende/auslösende Faktoren beseitigen/behandeln (Infektionskrankheiten, Dehydratation etc.)<br>2. Überprüfung der Begleitmedikation (falls Antihypertensiva angewandt werden, sollte eine Dosisreduktion/ein Absetzen angestrebt werden)<br>3. nicht medikamentöse Therapiemaßnahmen anwenden (vgl. nachfolgende Empfehlungen)<br>4. Beginn blutdrucksteigernder Medikamente (vgl. nachfolgende Empfehlungen).	
Konsensstärke: 100%, starker Konsens	

**Empfehlung**	**Neu**<br>**Stand (2023)**
Zur Behandlung der orthostatischen Hypotonie bei Pat. mit der PK sollten folgende nicht medikamentöse Therapiemaßnahmen empfohlen werden:<br>1. eine ausreichende Flüssigkeits- und Salzzufuhr, sofern keine internistischen Kontraindikationen hierfür bestehen (bspw. Herz-, Leber-, Niereninsuffizienz)<br>2. die Vermeidung von üppigen Mahlzeiten oder übermäßigem Alkoholkonsum<br>3. die Vermeidung von Hitze-Exposition<br>4. die Oberkörperhochlagerung um 10–20° während des Schlafens<br>5. das Tragen einer abdominellen Kompressionsbinde untertags ist wirksamer als Kompressionsstrümpfe<br>6. isometrische blutdrucksteigernde Manöver in der Prodromalphase einer Synkope (bspw. Anspannung der Bein-, Gesäß-, Bauch- und Armmuskeln)	
Konsensstärke: 95%, Konsens	

Empfehlung	Neu<br>Stand (2023)
Zur Behandlung der orthostatischen Hypotonie bei Pat. mit der PK sollte als pharmakologische Maßnahme eine Therapie mit Midodrin erwogen werden.	
Konsensstärke: 100%, starker Konsens	

Empfehlung	Neu<br>Stand (2023)
Zur Behandlung der orthostatischen Hypotonie bei Pat. mit der PK kann als pharmakologische Maßnahme eine Therapie mit Fludrocortison erwogen werden.	
Konsensstärke: 100%, starker Konsens	

Empfehlung	Neu<br>Stand (2023)
Zur Behandlung der orthostatischen Hypotonie bei Pat. mit der PK kann als pharmakologische Maßnahme eine Therapie mit Droxidopa (L-Threo-DOPS) erwogen werden (off label use, nur über internationale Apotheke zu beziehen).	
Konsensstärke: 95%, Konsens	

Fragestellung 98: Wie wird nächtliche Hypertonie beim bei der PK diagnostiziert?

Hintergrund

Neben der orthostatischen Hypotonie stellt die nächtliche (oder Liegend-)Hypertonie häufig ein großes therapeutisches Problem dar. Es handelt sich um eine paradoxe Hypertonie im Liegen, die sich in etwa der Hälfte der Pat. mit neurogener OH entwickelt, schwerwiegend sein kann und im Schlaf mehrere Stunden andauert (nächtliche Hypertonie). Eine solche Hypertonie unterscheidet sich von der essenziellen arteriellen Hypertonie, da die meisten Pat. mit Liegendhypertonie im Sitzen normale oder erniedrigte Blutdruckwerte aufweisen und im Stehen starke Blutdruckabfälle erleiden. Blutdrucksteigernde Medikamente, die zur Behandlung der OH eingesetzt werden, können eine Liegendhypertonie demaskieren oder aggravieren, wenngleich sich die Liegendhypertonie auch unabhängig von einer Behandlung mit blutdrucksteigernden Medikamenten entwickeln kann [99]. Die zugrunde liegende Pathophysiologie ist bislang nicht vollends verstanden. Eine Störung der afferenten, zentralen und efferenten Bahnen des arteriellen Baroreflex-Bogens, eine Beeinträchtigung der Renin-Angiotensin-Aldosteron-Systems und eine Überempfindlichkeit der vaskulären Adrenozeptoren werden als mögliche Ursachen diskutiert. Mögliche Komplikationen einer Liegendhypertonie können chronische Endorganschädigungen, hypertensive Krisen und damit assoziierte Erkrankungen sowie vermehrte Druck-Natriurese und damit verbundene Zunahme der OH-Beschwerden umfassen.

Evidenzgrundlage

Empfehlungen der einschlägigen Fachgesellschaften (American Autonomic Society, European Federation of Autonomic Societies und European Society of Hypertension) zur Diagnostik der Liegendhypertonie bei Pat. mit neurogener orthostatischer Hypotonie wurden in einem Konsensuspapier dargelegt [99]. Im Übrigen finden die Empfehlungen zur Diagnostik und Therapie der Hypertonie aus der nationalen VersorgungsLeitlinie „Hypertonie" Anwendung [54].

Ergebnis

Bei Pat. mit neu diagnostizierter neurogener orthostatischer Hypotonie kann zum Zeitpunkt der Diagnose und in regelmäßigen Abständen im Krankheitsverlauf eine Abklärung auf eine Liegend- und nächtliche Hypertonie erwogen werden. Insbesondere sind entsprechende Abklärungen zielführend, sobald eine Behandlung mit blutdrucksteigernden Medikamenten begonnen bzw. ihre Dosierung erhöht wird oder wenn Pat. mit neurogenem kardiovaskulärem autonomem Versagen über mehrere Episoden von Nykturie klagen oder Knöchelödeme entwickeln.

Durch die Liegendphase im Rahmen des Schellong-Tests gewinnt man bereits erste Erkenntnisse, was das Vorliegen eines Liegendhypertonus (Definition s. unten) anbelangt. Zusätzlich ist das Führen eines Blutdrucktagebuchs (Messung zu unterschiedlichen Tageszeiten; zumindest dreimal täglich (früh, mittags und vor dem Schlafengehen) in Liege-, Sitz- und Stehposition) durch die Pat. in Erwägung zu ziehen, um Erkenntnisse über zirkadiane und tätigkeitsabhängige Blutdruckschwankungen zu erlangen. In einem zweiten Schritt kann eine ambulante 24-Stunden-Langzeit-Blutdruckmessung in Erwägung gezogen werden, um das Vorhandensein einer nächtlichen Hypertonie festzustellen und absolute Blutdruckwerte im Tagesverlauf zu dokumentieren. Um eine akkurate Interpretation der 24h-Blutdruck-Messung zu gewährleisten, sollten die Pat. angewiesen werden, am Untersuchungstag ein Aktivitätstagebuch zu erstellen, in dem die Zeiten der Medikamenteneinnahme (insbesondere Antihypotensiva und Antihypertensiva), der Mahlzeiten, der körperlichen Aktivitäten und des eventuellen Aufstehens nachtsüber, z.B. zur Nutzung des Badezimmers, angegeben werden. Die Diagnose Liegendhypertonie definiert sich durch einen systolischen Blutdruck ≥ 140 mmHg und/oder diastolischen BD ≥ 90 mmHg, gemessen nach mindestens 5 Minuten Ruhe in Rückenlage bei Pat. mit vorbekannter neurogener orthostatischer Hypotonie [99].

Man unterscheidet zwischen folgenden Schweregraden:

- Milde Liegendhypertonie: systolische BD-Werte von 140–159 mmHg oder diastolisch von 90–99 mmHg;
- Moderate Liegendhypertonie: systolische BD-Werte von 160–179 mmHg oder diastolisch von 10–109 mmHg;
- Schwere Liegendhypertonie: wenn systolischer BD ≥180 mmHg oder diastolischer BP ≥110 mmHg.

Zudem findet sich häufig bei Pat. mit kardiovaskulärem autonomem Versagen eine nächtliche Hypertonie, die mit dem Verlust des physiologischen Blutdruckabfalls (> 10%) im Schlaf einhergeht. Es werden zwei nächtliche Blutdruckprofile unterschieden:

- reduziertes Absinken: gekennzeichnet durch einen mittleren nächtlichen Blutdruckabfall <10%, bezogen auf die mittleren Blutdruckwerte untertags.
- fehlendes Absinken ODER Steigen: wenn der mittlere Blutdruck während der Nacht nicht sinkt oder sogar steigt.

**Empfehlung**	**Neu**<br>**Stand (2023)**
Bei Pat. mit der PK und einer neurogenen orthostatischen Hypotonie sollten zur Diagnose der Liegend- oder nächtlichen Hypertonie ein Schellong-Test mit zumindest 5-minütiger Liegephase, das Führen eines Blutdrucktagebuchs und eine ambulante 24-Stunden-Langzeit-Blutdruckmessung angestrebt werden.	
Konsensstärke: 95%, Konsens	

Fragestellung 99: Wie wird nächtliche Hypertonie bei der PK therapiert?

Hintergrund

Die paradoxe Hypertonie im Liegen, die sich in etwa der Hälfte der Pat. mit neurogener orthostatischer Hypotonie findet, kann chronische Endorganschädigungen, hypertensive Krisen (und damit assoziierte Erkrankungen) sowie eine vermehrte nächtliche Druck-Natriurese mit Zunahme der Nykturie-Episoden und der OH-Beschwerden am Folgetag bedingen. Hierdurch kommt der Behandlung der nächtlichen und Liegendhypertonie ein besonderer Stellenwert zu.

Evidenzgrundlage

Empfehlungen für die Therapie der nächtlichen Hypertonie bei der PK basieren überwiegend auf Expertenmeinungen und einzelnen klinischen Studien mit kleinen Fallzahlen. Die Stellungnahmen zur Behandlung der Liegendhypertonie bei Pat. mit neurogener orthostatischer Hypotonie der einschlägigen Fachgesellschaften (American Autonomic Society, European Federation of Autonomic Societies und European Society of Hypertension) finden hier Anwendung [100]. Die Diagnostik und Therapie der Hypertonie ist überdies in der nationalen VersorgungsLeitlinie „Hypertonie“ ausgeführt [54].

Ergebnis

Für nicht medikamentöse Therapiemaßnahmen und Handlungsempfehlungen gibt es keinen direkten Effektivitätsnachweis. Die Expertenempfehlungen [100] basieren auf indirekten Schlussfolgerungen aus Studien mit anderen Zielsetzungen. Auch Medikamentenempfehlungen basieren überwiegend auf pharmakokinetischen und -dynamischen Überlegungen und dem indirekten Effektivitätsnachweis. Für die Therapie der nächtlichen bzw. Liegendhypertonie bei Pat. mit der PK empfehlen Expertinnen/Exerten die Anwendung eines Stufenschemas [100]. An erster Stelle des Stufenschemas finden sich nicht medikamentöse Präventions- und Verhaltensmaßnahmen (Vermeidung der Liegendposition untertags, Pat.-Edukation mit Hinblick auf die Einnahmezeitpunkte

etwaiger blutdrucksteigernder Medikamente (z. B. keine Einnahme nach 16:00 Uhr nachmittags), Verminderung der Flüssigkeitsaufnahme in den Abendstunden, Verzehr einer kleinen Mahlzeit vor dem Zubettgehen, Oberkörperhochlagerung um 10–20° während des Schlafens). Bei gleichzeitigem Vorliegen einer behandlungsbedürftigen orthostatischen Hypotonie ist eine Vermeidung von langwirksamen (bspw. Fludrocortison) zugunsten kurzwirksamer (bspw. Midodrin, Ephedrin, Droxidopa) Präparate in Betracht zu ziehen.

Für transdermal appliziertes Nitroglycerin konnte ein signifikanter Abfall des Liegendblutdrucks in einer offenen Beobachtungsstudie gezeigt werden [34]. Eine randomisierte, placebokontrollierte und doppelblinde Studie in 11 Pat. konnte nachweisen, dass die Einnahme von Losartan beim Zubettgehen einen markanten Blutdruckabfall zur Folge hat [101]. Ähnlich verhält es sich mit Eplerenon, für das in einer doppelblinden Crossover-Studie eine substanzielle nächtliche Blutdruckreduktion nachgewiesen werden konnte [102]. In einer doppelblinden und randomisierten Vergleichsstudie waren Sildenafil und Nebivolol mit einem signifikanten Blutdruckabfall assoziiert. Im Rahmen dieser Studie war Metoprolol ein aktiver Komparator, der keinen Einfluss auf die Liegendhypertonie hatte [103]. Auch für Clonidin konnte eine Reduktion der nächtlichen Blutdruckwerte in einer randomisierten Studie bei 23 Pat. gezeigt werden [104].

**Empfehlung**	**Neu**<br>**Stand (2023)**
Zur Therapie der (nächtlichen) Liegendhypertonie sollte bei Pat. mit der PK folgendes Stufenschema angewendet werden:<br>1. nicht medikamentöse Präventions- und Verhaltensmaßnahmen (vgl. untenstehende Empfehlung)<br>2. Umstellung von langwirksamen auf kurzwirksame blutdrucksteigernde Medikamente<br>3. pharmakologische Blutdrucksenkung (vgl. untenstehende Empfehlung).	
Konsensstärke: 95%, Konsens	

**Empfehlung**	**Neu**<br>**Stand (2023)**
Zur Prävention und Therapie der (nächtlichen) Liegendhypertonie sollten die folgenden nicht medikamentösen Präventions- und Verhaltensmaßnahmen bei Pat. mit der PK angestrebt werden:<br>1. die Vermeidung der Liegendposition untertags<br>2. die Pat.-Edukation mit Hinblick auf die Einnahmezeitpunkte etwaiger blutdrucksteigernder Medikamente (keine Einnahme nach 16:00 Uhr)<br>3. die Verminderung der Flüssigkeitsaufnahme in den Abendstunden (nach 18:00 Uhr)<br>4. der Verzehr einer kleinen Mahlzeit vor dem Zubettgehen<br>5. die Oberkörperhochlagerung um 10–20° während des Schlafens.	
Konsensstärke: 95%, Konsens	

**Empfehlung**	**Neu**<br>**Stand (2023)**
Zur medikamentösen Therapie der nächtlichen Liegendhypertonie kann eine abendliche Gabe von Clonidin, Eplerenon, Losartan, Nebivolol, Nitroglycerin (transdermale Applikation) oder Sildenafil (alphabetische Reihung) erwogen werden.	
Konsensstärke: 95%, Konsens	

Autoren/Autorinnen: Claudia Trenkwalder, Dirk Woitalla

Fragestellung 100: Wie wird Obstipation bei der PK diagnostiziert?

Evidenzgrundlage

In der Literaturrecherche fanden sich Reviews, eine Metaanalyse und die 2022 konsentierte AWMF-Leitlinie zur chronischen Obstipation, die in weiten Teilen auf die Obstipation bei Parkinson-Pat. zutrifft und auch therapeutisch anwendbar ist. Diese Leitlinie wurde hier als Grundlage verwendet und in einigen Bereichen durch die bei der PK typischen Merkmale und Pathophysiologie ergänzt und modifiziert.

Die Pathogenese der Obstipation bei Parkinson ist primär durch die Erkrankung bedingt und wird durch mehrere Faktoren verstärkt: Hierbei spielt die reduzierte körperliche Aktivität eine wichtige Rolle, ebenso der verminderte Muskeltonus der abdominellen Muskulatur und eine Ernährung mit wenig faserreicher Kost und zu geringer Flüssigkeitsaufnahme. Dies führt mit der reduzierten Motilität des Gastrointestinaltrakts bei der PK zu einer verlängerten Passagezeit und kann sogar ein Megakolon hervorrufen [105]. Hinzu kommen eventuell auch noch die Parkinson-Medikamente, die eine Obstipation verstärken können, insbesondere alle anticholinerg wirksamen Substanzen. [106], [107]. In der AWMF-Leitlinie „Chronische Obstipation“ werden in Tabelle 4 zahlreiche Medikamente genannt, die eine Obstipation verursachen oder verstärken können. Für die Behandlung bei der PK sind neben den Anticholinergika die folgenden Medikamente für eine Obstipation relevant: Opoide, trizyklische Antidepressiva, kalziumhaltige Antazida, Antihypertensiva, Spasmolytika, Sympathomimetika, Diuretika.

Eine weitere, seltene Ursache der Obstipation bei der PK besteht aus dem Anismus, einer fehlenden Entspannung oder unwillkürlichen Kontraktionen des Schließmuskels während der Defäkation. Dies ist ein sehr seltenes Symptom und wird auch als „Outlet-Constipation“ bezeichnet [105].

Ergebnis

Bei Parkinson-Pat. und einer Obstipation werden die folgenden Symptome berichtet: Es treten Probleme bei der Stuhlentleerung auf, es besteht ein harter Stuhl, es können mehrere Tage ohne Stuhlentleerung vergehen, und es besteht ein Völlegefühl, teilweise mit abdominellen Schmerzen. Diese Beschwerden können bereits als Zeichen einer prodromalen PK auftreten. Zahlreiche Kohortenstudien und eine Metaanalyse bestätigen inzwischen, dass Obstipation zu den sehr frühen

nicht motorischen Symptomen zählt und in Verbindung mit Hyposmie, geringen motorischen Auffälligkeiten und einer REM-Schlaf-Verhaltensstörung die Konversion von einer prämotorischen Phase in eine manifeste klinische PK sehr wahrscheinlich macht [108]. Eine der größten neuropathologischen Studien zu diesem Thema zeigt, dass bei der PK eine Lewy-Körper-Pathologie im gastrointestinalen System nachgewiesen werden konnte, mit einem rostro-caudalen Gradienten, beginnend im oberen Gastrointestinaltrakt, und ausgeprägten Veränderungen weiter caudal, im Vagus-Nerv und dem Truncus sympathicus mit einer Häufigkeit von bis zu 80% [109].

Bei der klinischen Diagnose PK besteht bei 24–63% der Betroffenen eine Obstipation, abhängig von den Diagnosekriterien, die für eine Obstipation angewandt wurden [110]. Im Verlauf sind es bis 90% [105]. Die Obstipation wird dabei als eines der für die Lebensqualität relevantesten Symptome in der Frühphase der PK bezeichnet und sei häufiger, als sie subjektiv beklagt wird [105].

Begründung der Empfehlung

In der Leitlinie der AWMF zum Thema „Chronische Obstipation“ [111] werden die Probleme der Definition der Obstipation im klinischen Alltag erläutert, die auch bei Betroffenen mit der PK in fast identischer Weise auftreten: Probert et al. haben schon 1994 auf die Diskrepanz zwischen subjektiv berichteter Obstipation und Konsensus-Definitionen hingewiesen. Diese Diskrepanz besteht bis heute und führt zu großer Variabilität hinsichtlich der berichteten Prävalenz. Eine wesentliche Schwierigkeit besteht unter anderem darin, dass eine rein objektive Definition der Obstipation z. B. alleinig basierend auf der Stuhlfrequenz, dem gesamten Beschwerdekomplex der Pat. mit chronischer Obstipation nicht gerecht wird und durch derartige Definitionen ein großer Teil der Pat. nicht erfasst wird. So gibt es viele Pat., die z. B. sehr mühevoll und nur unter großer Anstrengung und mit starkem Pressen Stuhlgang haben können, dies aber durchaus auch täglich. Zudem hat sich gezeigt, dass z. B. Messungen der gastrointestinalen Transitzeit eher mit dem Parameter der Stuhlkonsistenz als mit dem der Stuhlfrequenz korrelieren. International haben sich aufgrund dieser Schwierigkeiten daher Definitionen etabliert, die eine Kombination aus subjektiven (wie z. B. starkes Pressen oder unvollständige Entleerung) und objektiven Parametern (z. B. Stuhlfrequenz, Stuhlkonsistenz) enthält. Den derzeitigen internationalen Standard stellt in dem Zusammenhang die Rom-IV-Klassifikation dar [112], auf deren Basis diese hier vorliegende Definition gewählt wurde [111].

Weiterhin empfiehlt die AWMF-Leitlinie eine genaue Erfassung der Symptome bei erstmaliger Diagnosestellung: „Die Basisdiagnostik der chronischen Obstipation soll eine genaue Anamnese mit Analyse des Stuhlverhaltens, der Medikamenteneinnahme, der Begleitsymptome und -erkrankungen sowie möglicher verursachender Erkrankungen, eine körperliche Untersuchung inklusive Anusinspektion und rektal-digitaler Untersuchung mit Prüfung des Sphinkterruhetonus, des Kneifdrucks und des Defäkationsversuchs beinhalten.“ [111]

Es werden keine weitergehenden Untersuchungen empfohlen, außer es bestehen starke Beschwerden und/oder Schmerzen: „Weitergehende Untersuchungen sollen bei starken Beschwerden bzw. hohem Leidensdruck oder Warnsymptomen unmittelbar (= ohne vorherige probatorische Therapie), sonst bei mangelndem Ansprechen der Beschwerden auf die probatorische Therapie erfolgen. Sie dienen im ersten Schritt (a) dem Ausschluss organischer Ursachen und im

zweiten (b) der Klärung der Pathophysiologie mittels Funktionsuntersuchungen." Von einer Stuhluntersuchung auf Bakterien wird grundsätzlich abgeraten. [111]. Welche weiterführenden Untersuchungen durchgeführt werden können, wird in der Leitlinie aufgeführt (S. 1539 und Tab. 5 der Leitlinie „Chronische Obstipation" [111]).

**Empfehlung**	**Neu**<br>**Stand (2023)**
Zur Diagnosestellung einer Obstipation bei Parkinson-Pat. sollten die folgenden Kriterien der AWMF-LL „Chronische Obstipation" herangezogen werden.<br>Eine chronische Obstipation liegt vor, wenn folgende drei Kriterien aktuell seit mindestens 3 Monaten bestehen, wobei der initiale Beginn der Symptomatik wenigstens 6 Monate vor Diagnosestellung liegen sollte:<br>1. ≥ 2 der folgenden Symptome sollten vorhanden sein:<br>▪ klumpiger oder harter Stuhl (Bristol Stool Form Scale 1–2) bei > 25 % der Stuhlentleerungen<br>▪ starkes Pressen bei > 25 % der Stuhlentleerungen<br>▪ subjektiv unvollständige Entleerung bei > 25 % der Stuhlentleerungen<br>▪ subjektive Obstruktion bei > 25 % der Stuhlentleerungen<br>▪ manuelle Manöver zur Erleichterung der Defäkation bei >25 % der Stuhlentleerungen (digitale Manipulation, Beckenbodenunterstützung)<br>▪ < 3 spontane Stuhlgänge pro Woche.<br>2. Weiche Stühle kommen ohne die Einnahme von Laxantien nur selten vor.<br>3. Die Kriterien für ein Reizdarmsyndrom sind nicht erfüllt.	
Konsensstärke: 100%, starker Konsens	

Fragestellung 101: Wie wird Obstipation bei der PK therapiert?

Evidenzgrundlage

In der Literaturrecherche fanden sich drei RCTs zur Therapie der Obstipation bei der PK und die 2022 konsentierte AWMF-Leitlinie zur chronischen Obstipation [111].

Die allgemeine Behandlung der Obstipation beginnt mit einer faserreichen Diät [113], ausreichender Flüssigkeitszufuhr, Bewegung und Physiotherapie. Trotzdem benötigen viele Parkinson-Pat. eine zusätzliche medikamentöse Behandlung. Die effizienteste Behandlung zur Verbesserung der verlängerten Passagezeit bei der PK ist die Therapie mit Macrogol. Medikamentöse Alternative ist das Prucaloprid, das bei Parkinson-Pat. wegen der teilweise sehr ausgeprägten Obstipation ebenfalls zur Anwendung kommt. Dyssynergetische Defäkation kann eine dopaminerge Therapie, in Einzelfällen auch mit Apomorphin-Injektionen bedingen, die „Outlet-Constipation" kann mit Botulinumtoxin behandelt werden [114].

Ergebnis

Die nicht medikamentöse Behandlung umfasst die faserreiche Diät und die tägliche Trinkmenge von 1.5–2 Litern. Eine darüberhinausgehende Flüssigkeitszufuhr hat keinen weiteren Effekt auf die Obstipation (LL „Chron. Obstipation", [111]). Ebenso ist eine dem Alltag entsprechende Bewegung anzustreben. „Ein therapeutischer Effekt einer über das normale altersentsprechende Maß hinausgehenden körperlichen Aktivität auf die Obstipation sollte nicht in Aussicht gestellt werden" (Empfehlung LL chron. Obstipation, [111]). Es besteht jedoch ein Zusammenhang zwischen Obstipation und körperlicher Inaktivität.

Eine faserreiche Diät kann bei Beginn zu abdominellen Störungen bis zu Schmerzen führen, die jedoch bei längerer Verabreichung adaptieren. Bei älteren Pat. wird deshalb das folgende Vorgehen in der LL „Chron. Obstipation" empfohlen: „Bei der Basistherapie obstipierter geriatrischer Pat. sollte die individuelle Toleranz natürlicher Ballaststoffe berücksichtigt und auf eine ausreichende Flüssigkeitsaufnahme geachtet werden. Die Gabe von löslichen Ballaststoffen kann bei nicht tolerierten natürlichen Ballaststoffen bei geriatrischen Pat. versucht werden." Es ist hier insbesondere zu beachten, dass Ballaststoffe möglicherweise bei Parkinson-Pat. mit Nebenwirkungen verbunden sind, da die Parkinson-typische Obstipation überwiegend mit einer längeren Transitzeit verbunden ist. „Pat. mit Slow-transit-Obstipation oder Defäkationsstörung sprechen auf Ballaststoffe schlechter an als solche ohne nachweisbare Störung und diese Pat. weisen auch häufiger Nebenwirkungen unter Ballaststofftherapie auf." (Leitlinie „Chron. Obstipation", Empfehlung, [111]).

Eine Kolonmassage wird bei Parkinson-Pat. empfohlen, einige kleinere Studien zeigten hier eine Verbesserung, die Abdominalmassage war genauso wirksam wie eine Lebensstilberatung [115]. In einer weiteren Studie wird eine höhere Effektivität einer Abdominalmassage nach dem Tensegrity-Prinzip, welches auf dem Ausgleich der Spannung von Muskeln, Faszien und Bändern beruht, im Vergleich zur klassischen Abdominalmassage aufgezeigt [116].

In der Leitlinie „Chronische Obstipation" wird die Therapie mit Macrogol als medikamentöse Therapie der ersten Wahl empfohlen. „Die Wirksamkeit und Sicherheit von Macrogol (Polyethylenglycol 3350 bzw. 4000) bei chronischer Obstipation konnte in zahlreichen Studien nachgewiesen werden. Eine Metaanalyse [117] kommt zu dem Schluss, dass PEG in Stuhlfrequenz, Stuhlform, Erleichterung von abdominellen Schmerzen und der Notwendigkeit der Einnahme von Laxantien der Lactulose überlegen ist (bessere Wirksamkeit bei weniger Nebenwirkungen)." (LL „Chronische Obstipation", [111]). Die Anwendung sollte mit ausreichend Flüssigkeit erfolgen und die Dosierung individuell angepasst werden. Pat. sollten darauf hingewiesen werden, dass es sich um ein kaum resorbierbares Arzneimittel handelt.

Weitere Substanzen, die als Mittel der ersten Wahl bei chronischer Obstipation verabreicht werden sollten, sind die Kombination von Bisacodyl und Natriumpicosulfat, die beide auch kaum resorbiert werden.

„Bislang gibt es keine Studie, die PEG und Bisacodyl/Natriumpicosulfat direkt vergleicht. Gemäß einer Network-Metaanalyse scheinen Bisacodyl/Natriumpicosulfat effektiver als PEG zu sein [118]. Hierfür spricht auch, dass diese Substanzen zumeist als Rescue-Medikation in Obstipationsstudien verwendet werden." (LL Chronische Obstipation, [111])

Für die Anwendung von Probiotika stehen auch zwei RCTs mit Parkinson-Pat. zur Verfügung. In der ersten RCT wurden 120 Parkinson-Pat. randomisiert und erhielten entweder fermentierte Milch, die multiple probiotische Bakterienstämme enthielt, kombiniert mit probiotischen Fasern, oder Placebo für vier Wochen. In der Behandlungsgruppe gab es eine signifikante Zunahme der Stuhlentleerungen pro Woche, gemessen an einem Stuhltagebuch. Weiterhin verbesserten sich die Häufigkeit von Stuhlentleerungen, die Konsistenz der Stühle und die zusätzliche Einnahme von Laxantien in der Behandlungsgruppe war geringer im Vergleich zur Placebogruppe [119]. In einer zweiten, monozentrischen doppelblinden RCT wurden von 280 Parkinson-Pat. 72 mit entweder multiplen Bakterienstämmen in Probiotika-Kapseln oder mit Placebo-Kapseln über 4 Wochen behandelt. Der Endpunkt waren neben Stuhltagebüchern und Änderungen der Lebensqualität die Zahl der spontanen Stuhlentleerungen pro Woche während der letzten 2 Wochen der Behandlung. Unter Probiotika zeigte sich eine signifikante Zunahme der Stuhlentleerungen pro Woche gegenüber Placebo neben der verbesserten Lebensqualität. 65% der Studienteilnehmerinnen und -teilnehmer waren mit der Probiotika-Behandlung zufrieden, ein Patient beendete die Studie vorzeitig wegen einer nicht schweren Nebenwirkung [120].

Derzeit werden auch Effekte von Probiotika auf motorische und nicht motorische Symptome wie Kognition, Depression und Psychose diskutiert. Daten aus Langzeitstudien liegen jedoch nicht vor. Dies stellt bei Parkinson-Pat. auch eine besondere Herausforderung dar, da eine Konstanz aller Faktoren einschließlich der Medikation erfolgen muss [121].

In der LL „Chronische Obstipation“ findet sich dazu die folgende Empfehlung, die allgemein gilt: „Probiotika, Präbiotika und Synbiotika können bei funktioneller chronischer Obstipation versucht werden.“

Bei schwerer Obstipation stehen rektale Entleerungshilfen zur Verfügung, hierzu werden in der Leitlinie „Chron. Obstipation“ Bisacodyl-Zäpfchen oder CO_2-freisetzende Zäpfchen empfohlen.

Evidenzgrundlage

Neben der LL „Chronische Obstipation“ mit allgemeinen Empfehlungen zur Therapie der chronischen Obstipation liegen RCTs mit gering unterschiedlicher Methodik zur Therapie mit Probiotika und abdomineller Massage speziell bei der PK vor.

**Empfehlung**	**Neu**<br>**Stand (2023)**
Zur Therapie einer Obstipation bei Parkinson-Pat. sollen die allgemeinen Therapieempfehlungen der AWMF-Leitlinie „Chronische Obstipation“ herangezogen werden: Diese empfehlen allgemeine Maßnahmen (Auswahl der wichtigsten Empfehlungen, weitere Detail-Empfehlungen in der Original-Leitlinie).<br>▪ Auf eine tägliche Trinkmenge von 1,5–2,0 Litern sollte geachtet werden.<br>▪ Körperliche Inaktivität sollte vermieden werden.<br>▪ Eine regelmäßige Unterdrückung des Stuhldrangs sollte vermieden werden.<br>▪ Bei der Basistherapie obstipierter geriatrischer Pat. sollte die individuelle Toleranz natürlicher Ballaststoffe berücksichtigt und auf eine ausreichende Flüssigkeitsaufnahme geachtet werden.	

- Die Gabe von löslichen Ballaststoffen kann bei nicht tolerierten natürlichen Ballaststoffen bei geriatrischen Pat. versucht werden.
- Beim Auftreten von unangenehmen Begleitsymptomen sollte die Zufuhr von Ballaststoffen probatorisch reduziert und andere Maßnahmen zur Obstipationstherapie zunächst bevorzugt werden.
- Macrogole sollten als Arzneimittel der ersten Wahl angewendet werden. Eine Begrenzung des Einnahmezeitraums ist unbegründet.
- Natriumpicosulfat/Bisacodyl und Prucaloprid sollten als Arzneimittel der zweiten Wahl gegeben werden bei Pat., bei denen die bisherige konventionelle Therapie (Lebensstil, Ballaststoffe, konventionelle medikamentöse Therapien, konventionelle „Laxantien“) nicht ausreichend effektiv oder schlecht verträglich war
- Diese Empfehlungen gelten auch für geriatrische Pat.
- Weitere Empfehlungen zur medikamentösen Arzneimitteltherapie enthalten die Substanzklasse der Antrachinone, Linaclotid, Plecanatid, Zucker und Zuckeralkohole (Lactulose, Lactitol, Sorbit und Lactose).
- Nicht medikamentöse Therapieempfehlungen umfassen Biofeedback und Beckenbodentraining sowie Akupunkturverfahren (Akupunktur, Akupressur, Moxibustion, Elektro-Akupunktur), die bei chronischer Obstipation eingesetzt werden können.
- Spezifische Empfehlungen für Parkinson-Pat. mit chronischer Obstipation nach Evidenzgrundlage:
- Probiotika, Präbiotika und Synbiotika können bei Parkinson-Pat. bei chronischer Obstipation erwogen werden ohne stammspezifische Empfehlungen.
- Abdominelle Massagen des Kolons können bei Parkinson-Pat. bei chronischer Obstipation erwogen werden.

Konsensstärke: 100%, starker Konsens

Referenzen

1. Abrams P, Cardozo L, Fall M, Griffiths D, Rosier P, Ulmsten U, et al. The standardisation of terminology of lower urinary tract function: report from the Standardisation Sub-committee of the International Continence Society. Neurourol Urodyn. 2002;21(2):167-78
2. Haensch C. Diagnostik und Therapie von neurogenen Blasenstörungen, S1-Leitlinie, 2020. Deutsche Gesellschaft für Neurologie (Hrsg), Leitlinien für Diagnostik und Therapie in der Neurologie Online: wwwdgnorg/leitlinien (abgerufen am 21072023).
3. Panicker JN, Fowler CJ, Kessler TM. Lower urinary tract dysfunction in the neurological patient: clinical assessment and management. Lancet Neurol. 2015;14(7):720-32
4. Blok B. EAU Guidelines on Neuro-Urology. *https://uroweb.org/guidelines/neuro-urology* (abgerufen am 21.07.2023).
5. Li FF, Cui YS, Yan R, Cao SS, Feng T. Prevalence of lower urinary tract symptoms, urinary incontinence and retention in Parkinson's disease: A systematic review and meta-analysis. Front Aging Neurosci. 2022;14:977572
6. Barone P, Antonini A, Colosimo C, Marconi R, Morgante L, Avarello TP, et al. The PRIAMO study: A multicenter assessment of nonmotor symptoms and their impact on quality of life in Parkinson's disease. Mov Disord. 2009;24(11):1641-9
7. Sakakibara R, Tateno F, Yamamoto T, Uchiyama T, Yamanishi T. Urological dysfunction in synucleinopathies: epidemiology, pathophysiology and management. Clin Auton Res. 2018;28(1):83-101

8. Sakakibara R, Kishi M, Ogawa E, Tateno F, Uchiyama T, Yamamoto T, Yamanishi T. Bladder, bowel, and sexual dysfunction in Parkinson's disease. Parkinsons Dis. 2011;2011:924605
9. Konstantinidis C, Kratiras Z, Samarinas M, Skriapas K. Optimal bladder diary duration for patients with suprapontine neurogenic lower urinary tract dysfunction. Int Braz J Urol. 2016;42(4):766-72
10. Seppi K, Ray Chaudhuri K, Coelho M, Fox SH, Katzenschlager R, Perez Lloret S, et al. Update on treatments for nonmotor symptoms of Parkinson's disease-an evidence-based medicine review. Mov Disord. 2019;34(2):180-98
11. Sakakibara R, Panicker J, Finazzi-Agro E, Iacovelli V, Bruschini H. A guideline for the management of bladder dysfunction in Parkinson's disease and other gait disorders. Neurourol Urodyn. 2016;35(5):551-63
12. Wallace SA, Roe B, Williams K, Palmer M. Bladder training for urinary incontinence in adults. Cochrane Database Syst Rev. 2004;2004(1):Cd001308
13. Corcos J, Gajewski J, Heritz D, Patrick A, Reid I, Schick E, Stothers L. Canadian Urological Association guidelines on urinary incontinence. Can J Urol. 2006;13(3):3127-38
14. Takahashi S, Takei M, Asakura H, Gotoh M, Ishizuka O, Kato K, et al. Clinical Guidelines for Female Lower Urinary Tract Symptoms (second edition). Int J Urol. 2021;28(5):474-92
15. Zesiewicz TA, Evatt M, Vaughan CP, Jahan I, Singer C, Ordorica R, et al. Randomized, controlled pilot trial of solifenacin succinate for overactive bladder in Parkinson's disease. Parkinsonism Relat Disord. 2015;21(5):514-20
16. Yonguc T, Sefik E, Inci I, Kusbeci OY, Celik S, Aydın ME, Polat S. Randomized, controlled trial of fesoterodine fumarate for overactive bladder in Parkinson's disease. World J Urol. 2020;38(8):2013-9
17. Bennett N, O'Leary M, Patel AS, Xavier M, Erickson JR, Chancellor MB. Can higher doses of oxybutynin improve efficacy in neurogenic bladder? J Urol. 2004;171(2 Pt 1):749-51
18. Winge K, Fowler CJ. Bladder dysfunction in Parkinsonism: mechanisms, prevalence, symptoms, and management. Mov Disord. 2006;21(6):737-45
19. Todorova A, Vonderheid-Guth B, Dimpfel W. Effects of tolterodine, trospium chloride, and oxybutynin on the central nervous system. J Clin Pharmacol. 2001;41(6):636-44
20. Kay GG, Ebinger U. Preserving cognitive function for patients with overactive bladder: evidence for a differential effect with darifenacin. Int J Clin Pract. 2008;62(11):1792-800
21. Rai BP, Cody JD, Alhasso A, Stewart L. Anticholinergic drugs versus non-drug active therapies for non-neurogenic overactive bladder syndrome in adults. Cochrane Database Syst Rev. 2012;12(12):Cd003193
22. Cho SY, Jeong SJ, Lee S, Kim J, Lee SH, Choo MS, Oh SJ. Mirabegron for treatment of overactive bladder symptoms in patients with Parkinson's disease: A double-blind, randomized placebo-controlled trial (Parkinson's Disease Overactive bladder Mirabegron, PaDoMi Study). Neurourol Urodyn. 2021;40(1):286-94
23. Moussa M, Chakra MA, Dabboucy B, Fares Y, Dellis A, Papatsoris A. The safety and effectiveness of mirabegron in Parkinson's disease patients with overactive bladder: a randomized controlled trial. Scand J Urol. 2022;56(1):66-72
24. Cheng B, Huang S, Huang Q, Zhou Z, Bao Y. The efficacy and safety of medication for treating overactive bladder in patients with Parkinson's disease: a meta-analysis and systematic review of randomized double-blind placebo-controlled trials. Int Urogynecol J. 2023
25. Geoffrion R. No. 353-Treatments for Overactive Bladder: Focus on Pharmacotherapy - An Addendum. J Obstet Gynaecol Can. 2017;39(12):1221-9
26. Kelleher C, Hakimi Z, Zur R, Siddiqui E, Maman K, Aballéa S, et al. Efficacy and Tolerability of Mirabegron Compared with Antimuscarinic Monotherapy or Combination Therapies for Overactive Bladder: A Systematic Review and Network Meta-analysis. Eur Urol. 2018;74(3):324-33

27. El Helou E, Labaki C, Chebel R, El Helou J, Abi Tayeh G, Jalkh G, Nemr E. The use of mirabegron in neurogenic bladder: a systematic review. World J Urol. 2020;38(10):2435-42
28. Giannantoni A, Rossi A, Mearini E, Del Zingaro M, Porena M, Berardelli A. Botulinum toxin A for overactive bladder and detrusor muscle overactivity in patients with Parkinson's disease and multiple system atrophy. J Urol. 2009;182(4):1453-7
29. Schurch B, de Sèze M, Denys P, Chartier-Kastler E, Haab F, Everaert K, et al. Botulinum toxin type a is a safe and effective treatment for neurogenic urinary incontinence: results of a single treatment, randomized, placebo controlled 6-month study. J Urol. 2005;174(1):196-200
30. Cruz F, Herschorn S, Aliotta P, Brin M, Thompson C, Lam W, et al. Efficacy and safety of onabotulinumtoxinA in patients with urinary incontinence due to neurogenic detrusor overactivity: a randomised, double-blind, placebo-controlled trial. Eur Urol. 2011;60(4):742-50
31. Kabay SC, Kabay S, Yucel M, Ozden H. Acute urodynamic effects of percutaneous posterior tibial nerve stimulation on neurogenic detrusor overactivity in patients with Parkinson's disease. Neurourol Urodyn. 2009;28(1):62-7
32. Amarenco G, Ismael SS, Even-Schneider A, Raibaut P, Demaille-Wlodyka S, Parratte B, Kerdraon J. Urodynamic effect of acute transcutaneous posterior tibial nerve stimulation in overactive bladder. J Urol. 2003;169(6):2210-5
33. Perissinotto MC, D'Ancona CA, Lucio A, Campos RM, Abreu A. Transcutaneous tibial nerve stimulation in the treatment of lower urinary tract symptoms and its impact on health-related quality of life in patients with Parkinson disease: a randomized controlled trial. J Wound Ostomy Continence Nurs. 2015;42(1):94-9
34. Shannon J, Jordan J, Costa F, Robertson RM, Biaggioni I. The hypertension of autonomic failure and its treatment. Hypertension. 1997;30(5):1062-7
35. Sakakibara R, Matsuda S, Uchiyama T, Yoshiyama M, Yamanishi T, Hattori T. The effect of intranasal desmopressin on nocturnal waking in urination in multiple system atrophy patients with nocturnal polyuria. Clin Auton Res. 2003;13(2):106-8
36. Braun M, Wassmer G, Klotz T, Reifenrath B, Mathers M, Engelmann U. Epidemiology of erectile dysfunction: results of the 'Cologne Male Survey'. Int J Impot Res. 2000;12(6):305-11
37. Haensch C. Diagnostik und Therapie der erektilen Dysfunktion, S1-Leitlinie, 2018; in: Deutsche Gesellschaft für Neurologie (Hrsg.), Leitlinien für Diagnostik und Therapie in der Neurologie. Online: www.dgn.org/leitlinien (abgerufen am 21.07.2023).
38. Lombardi G, Nelli F, Celso M, Mencarini M, Del Popolo G. Treating erectile dysfunction and central neurological diseases with oral phosphodiesterase type 5 inhibitors. Review of the literature. J Sex Med. 2012;9(4):970-85
39. Zesiewicz TA, Helal M, Hauser RA. Sildenafil citrate (Viagra) for the treatment of erectile dysfunction in men with Parkinson's disease. Mov Disord. 2000;15(2):305-8
40. Hussain IF, Brady CM, Swinn MJ, Mathias CJ, Fowler CJ. Treatment of erectile dysfunction with sildenafil citrate (Viagra) in parkinsonism due to Parkinson's disease or multiple system atrophy with observations on orthostatic hypotension. J Neurol Neurosurg Psychiatry. 2001;71(3):371-4
41. Raffaele R, Vecchio I, Giammusso B, Morgia G, Brunetto MB, Rampello L. Efficacy and safety of fixed-dose oral sildenafil in the treatment of sexual dysfunction in depressed patients with idiopathic Parkinson's disease. Eur Urol. 2002;41(4):382-6
42. Bernard BA, Metman LV, Levine L, Ouyang B, Leurgans S, Goetz CG. Sildenafil in the Treatment of Erectile Dysfunction in Parkinson's Disease. Mov Disord Clin Pract. 2017;4(3):412-5
43. Moore RA, Derry S, McQuay HJ. Indirect comparison of interventions using published randomised trials: systematic review of PDE-5 inhibitors for erectile dysfunction. BMC Urol. 2005;5:18

44. Mostile G, Jankovic J. Treatment of dysautonomia associated with Parkinson's disease. Parkinsonism Relat Disord. 2009;15 Suppl 3:S224-32
45. Dula E, Keating W, Siami PF, Edmonds A, O'Neil J, Buttler S. Efficacy and safety of fixed-dose and dose-optimization regimens of sublingual apomorphine versus placebo in men with erectile dysfunction. The Apomorphine Study Group. Urology. 2000;56(1):130-5
46. O'Sullivan JD, Hughes AJ. Apomorphine-induced penile erections in Parkinson's disease. Mov Disord. 1998;13(3):536-9
47. Velseboer DC, de Haan RJ, Wieling W, Goldstein DS, de Bie RM. Prevalence of orthostatic hypotension in Parkinson's disease: a systematic review and meta-analysis. Parkinsonism Relat Disord. 2011;17(10):724-9
48. Palma JA, Gomez-Esteban JC, Norcliffe-Kaufmann L, Martinez J, Tijero B, Berganzo K, Kaufmann H. Orthostatic hypotension in Parkinson disease: how much you fall or how low you go? Mov Disord. 2015;30(5):639-45
49. Freeman R, Wieling W, Axelrod FB, Benditt DG, Benarroch E, Biaggioni I, et al. Consensus statement on the definition of orthostatic hypotension, neurally mediated syncope and the postural tachycardia syndrome. Clin Auton Res. 2011;21(2):69-72
50. Brignole M, Moya A, de Lange FJ, Deharo JC, Elliott PM, Fanciulli A, et al. 2018 ESC Guidelines for the diagnosis and management of syncope. Eur Heart J. 2018;39(21):1883-948
51. Diehl R. Synkopen, S1-Leitlinie, 2020, in: Deutsche Gesellschaft für Neurologie (Hrsg.), Leitlinien für Diagnostik und Therapie in der Neurologie. Online: *www.dgn.org/leitlinien* (abgerufen am 08.06.2023).
52. Norcliffe-Kaufmann L, Palma JA, Kaufmann H. A validated test for neurogenic orthostatic hypotension at the bedside. Ann Neurol. 2018;84(6):959-60
53. Fanciulli A, Kerer K, Leys F, Seppi K, Kaufmann H, Norcliffe-Kaufmann L, Wenning GK. Validation of the Neurogenic Orthostatic Hypotension Ratio with Active Standing. Ann Neurol. 2020;88(3):643-5
54. AWMF-Register-Nr. nvl-009; Bundesärztekammer (BÄK), Kassenärztliche Bundesvereinigung (KBV), Arbeitsgemeinschaft der Wissenschaftlichen Medizinischen Fachgesellschaften (AWMF). Nationale VersorgungsLeitlinie Hypertonie – Konsultationsfassung, Version 1.0. 2022 [zitiert: 2023-06-08]. *www.leitlinien.de/hypertonie*.
55. Eschlböck S, Wenning G, Fanciulli A. Evidence-based treatment of neurogenic orthostatic hypotension and related symptoms. J Neural Transm (Vienna). 2017;124(12):1567-605
56. Wieling W, Kaufmann H, Claydon VE, van Wijnen VK, Harms MPM, Juraschek SP, Thijs RD. Diagnosis and treatment of orthostatic hypotension. Lancet Neurol. 2022;21(8):735-46
57. Jordan J, Shannon JR, Grogan E, Biaggioni I, Robertson D. A potent pressor response elicited by drinking water. Lancet. 1999;353(9154):723
58. Jordan J, Shannon JR, Black BK, Ali Y, Farley M, Costa F, et al. The pressor response to water drinking in humans : a sympathetic reflex? Circulation. 2000;101(5):504-9
59. Shannon JR, Diedrich A, Biaggioni I, Tank J, Robertson RM, Robertson D, Jordan J. Water drinking as a treatment for orthostatic syndromes. Am J Med. 2002;112(5):355-60
60. Young TM, Mathias CJ. The effects of water ingestion on orthostatic hypotension in two groups of chronic autonomic failure: multiple system atrophy and pure autonomic failure. J Neurol Neurosurg Psychiatry. 2004;75(12):1737-41
61. Mathias CJ, Holly E, Armstrong E, Shareef M, Bannister R. The influence of food on postural hypotension in three groups with chronic autonomic failure--clinical and therapeutic implications. J Neurol Neurosurg Psychiatry. 1991;54(8):726-30
62. Senard JM, Brefel C, Carel C, Tran MA, Montastruc JL. Water drinking and the heart. Lancet. 1999;353(9168):1971-2
63. Lipp A, Tank J, Franke G, Arnold G, Luft FC, Jordan J. Osmosensitive mechanisms contribute to the water drinking-induced pressor response in humans. Neurology. 2005;65(6):905-7

64. Raj SR, Biaggioni I, Black BK, Rali A, Jordan J, Taneja I, et al. Sodium paradoxically reduces the gastropressor response in patients with orthostatic hypotension. Hypertension. 2006;48(2):329-34
65. Z'Graggen WJ, Hess CW, Humm AM. Acute fluid ingestion in the treatment of orthostatic intolerance - important implications for daily practice. Eur J Neurol. 2010;17(11):1370-6
66. Puvi-Rajasingham S, Mathias CJ. Effect of meal size on post-prandial blood pressure and on postural hypotension in primary autonomic failure. Clin Auton Res. 1996;6(2):111-4
67. Ten Harkel AD, Van Lieshout JJ, Wieling W. Treatment of orthostatic hypotension with sleeping in the head-up tilt position, alone and in combination with fludrocortisone. J Intern Med. 1992;232(2):139-45
68. van Lieshout JJ, ten Harkel AD, Wieling W. Fludrocortisone and sleeping in the head-up position limit the postural decrease in cardiac output in autonomic failure. Clin Auton Res. 2000;10(1):35-42
69. Tutaj M, Marthol H, Berlin D, Brown CM, Axelrod FB, Hilz MJ. Effect of physical countermaneuvers on orthostatic hypotension in familial dysautonomia. J Neurol. 2006;253(1):65-72
70. Ten Harkel AD, van Lieshout JJ, Wieling W. Effects of leg muscle pumping and tensing on orthostatic arterial pressure: a study in normal subjects and patients with autonomic failure. Clin Sci (Lond). 1994;87(5):553-8
71. van Lieshout JJ, ten Harkel AD, Wieling W. Physical manoeuvres for combating orthostatic dizziness in autonomic failure. Lancet. 1992;339(8798):897-8
72. Smit AA, Wieling W, Fujimura J, Denq JC, Opfer-Gehrking TL, Akarriou M, et al. Use of lower abdominal compression to combat orthostatic hypotension in patients with autonomic dysfunction. Clin Auton Res. 2004;14(3):167-75
73. Fanciulli A, Goebel G, Metzler B, Sprenger F, Poewe W, Wenning GK, Seppi K. Elastic Abdominal Binders Attenuate Orthostatic Hypotension in Parkinson's Disease. Mov Disord Clin Pract. 2016;3(2):156-60
74. Figueroa JJ, Singer W, Sandroni P, Sletten DM, Gehrking TL, Gehrking JA, et al. Effects of patient-controlled abdominal compression on standing systolic blood pressure in adults with orthostatic hypotension. Arch Phys Med Rehabil. 2015;96(3):505-10
75. Paschen S, Hansen C, Welzel J, Albrecht J, Atrsaei A, Aminian K, et al. Effect of Lower Limb vs. Abdominal Compression on Mobility in Orthostatic Hypotension: A Single-Blinded, Randomized, Controlled, Cross-Over Pilot Study in Parkinson's Disease. J Parkinsons Dis. 2022;12(8):2531-41
76. Newton JL, Frith J. The efficacy of nonpharmacologic intervention for orthostatic hypotension associated with aging. Neurology. 2018;91(7):e652-e6
77. Wright RA, Kaufmann HC, Perera R, Opfer-Gehrking TL, McElligott MA, Sheng KN, Low PA. A double-blind, dose-response study of midodrine in neurogenic orthostatic hypotension. Neurology. 1998;51(1):120-4
78. Jankovic J, Gilden JL, Hiner BC, Kaufmann H, Brown DC, Coghlan CH, et al. Neurogenic orthostatic hypotension: a double-blind, placebo-controlled study with midodrine. Am J Med. 1993;95(1):38-48
79. Low PA, Gilden JL, Freeman R, Sheng KN, McElligott MA. Efficacy of midodrine vs placebo in neurogenic orthostatic hypotension. A randomized, double-blind multicenter study. Midodrine Study Group. Jama. 1997;277(13):1046-51
80. Kaufmann H, Brannan T, Krakoff L, Yahr MD, Mandeli J. Treatment of orthostatic hypotension due to autonomic failure with a peripheral alpha-adrenergic agonist (midodrine). Neurology. 1988;38(6):951-6
81. Fouad-Tarazi FM, Okabe M, Goren H. Alpha sympathomimetic treatment of autonomic insufficiency with orthostatic hypotension. Am J Med. 1995;99(6):604-10

82. Ramirez CE, Okamoto LE, Arnold AC, Gamboa A, Diedrich A, Choi L, et al. Efficacy of atomoxetine versus midodrine for the treatment of orthostatic hypotension in autonomic failure. Hypertension. 2014;64(6):1235-40
83. Schoffer KL, Henderson RD, O'Maley K, O'Sullivan JD. Nonpharmacological treatment, fludrocortisone, and domperidone for orthostatic hypotension in Parkinson's disease. Mov Disord. 2007;22(11):1543-9
84. Freeman R, Landsberg L, Young J. The treatment of neurogenic orthostatic hypotension with 3,4-DL-threo-dihydroxyphenylserine: a randomized, placebo-controlled, crossover trial. Neurology. 1999;53(9):2151-7
85. Kaufmann H, Saadia D, Voustianiouk A, Goldstein DS, Holmes C, Yahr MD, et al. Norepinephrine precursor therapy in neurogenic orthostatic hypotension. Circulation. 2003;108(6):724-8
86. Kaufmann H, Freeman R, Biaggioni I, Low P, Pedder S, Hewitt LA, et al. Droxidopa for neurogenic orthostatic hypotension: a randomized, placebo-controlled, phase 3 trial. Neurology. 2014;83(4):328-35
87. Hauser RA, Isaacson S, Lisk JP, Hewitt LA, Rowse G. Droxidopa for the short-term treatment of symptomatic neurogenic orthostatic hypotension in Parkinson's disease (nOH306B). Mov Disord. 2015;30(5):646-54
88. Hauser RA, Hewitt LA, Isaacson S. Droxidopa in patients with neurogenic orthostatic hypotension associated with Parkinson's disease (NOH306A). J Parkinsons Dis. 2014;4(1):57-65
89. Biaggioni I, Freeman R, Mathias CJ, Low P, Hewitt LA, Kaufmann H. Randomized withdrawal study of patients with symptomatic neurogenic orthostatic hypotension responsive to droxidopa. Hypertension. 2015;65(1):101-7
90. Carvalho MJ, van den Meiracker AH, Boomsma F, Man in 't Veld AJ, Freitas J, Costa O, de Freitas AF. Improved orthostatic tolerance in familial amyloidotic polyneuropathy with unnatural noradrenaline precursor L-threo-3,4-dihydroxyphenylserine. J Auton Nerv Syst. 1997;62(1-2):63-71
91. Mathias CJ, Senard JM, Braune S, Watson L, Aragishi A, Keeling JE, Taylor MD. L-threo-dihydroxyphenylserine (L-threo-DOPS; droxidopa) in the management of neurogenic orthostatic hypotension: a multi-national, multi-center, dose-ranging study in multiple system atrophy and pure autonomic failure. Clin Auton Res. 2001;11(4):235-42
92. Kaufmann H, Oribe E, Yahr MD. Differential effect of L-threo-3,4-dihydroxyphenylserine in pure autonomic failure and multiple system atrophy with autonomic failure. J Neural Transm Park Dis Dement Sect. 1991;3(2):143-8
93. Matsubara S, Sawa Y, Yokoji H, Takamori M. Shy-Drager syndrome. Effect of fludrocortisone and L-threo-3,4-dihydroxyphenylserine on the blood pressure and regional cerebral blood flow. J Neurol Neurosurg Psychiatry. 1990;53(11):994-7
94. Sakoda S, Suzuki T, Higa S, Ueji M, Kishimoto S, Matsumoto M, Yoneda S. Treatment of orthostatic hypotension in Shy-Drager syndrome with DL-threo-3,4-dihydroxyphenylserine: a case report. Eur Neurol. 1985;24(5):330-4
95. Kachi T, Iwase S, Mano T, Saito M, Kunimoto M, Sobue I. Effect of L-threo-3,4-dihydroxyphenylserine on muscle sympathetic nerve activities in Shy-Drager syndrome. Neurology. 1988;38(7):1091-4
96. Man in 't Veld AJ, Boomsma F, van den Meiracker AH, Schalekamp MA. Effect of unnatural noradrenaline precursor on sympathetic control and orthostatic hypotension in dopamine-beta-hydroxylase deficiency. Lancet. 1987;2(8569):1172-5
97. Biaggioni I, Robertson D. Endogenous restoration of noradrenaline by precursor therapy in dopamine-beta-hydroxylase deficiency. Lancet. 1987;2(8569):1170-2

98. Isaacson S, Vernino S, Ziemann A, Rowse GJ, Kalu U, White WB. Long-term safety of droxidopa in patients with symptomatic neurogenic orthostatic hypotension. J Am Soc Hypertens. 2016;10(10):755-62
99. Fanciulli A, Jordan J, Biaggioni I, Calandra-Buonaura G, Cheshire WP, Cortelli P, et al. Consensus statement on the definition of neurogenic supine hypertension in cardiovascular autonomic failure by the American Autonomic Society (AAS) and the European Federation of Autonomic Societies (EFAS) : Endorsed by the European Academy of Neurology (EAN) and the European Society of Hypertension (ESH). Clin Auton Res. 2018;28(4):355-62
100. Jordan J, Fanciulli A, Tank J, Calandra-Buonaura G, Cheshire WP, Cortelli P, et al. Management of supine hypertension in patients with neurogenic orthostatic hypotension: scientific statement of the American Autonomic Society, European Federation of Autonomic Societies, and the European Society of Hypertension. J Hypertens. 2019;37(8):1541-6
101. Arnold AC, Okamoto LE, Gamboa A, Shibao C, Raj SR, Robertson D, Biaggioni I. Angiotensin II, independent of plasma renin activity, contributes to the hypertension of autonomic failure. Hypertension. 2013;61(3):701-6
102. Arnold AC, Okamoto LE, Gamboa A, Black BK, Raj SR, Elijovich F, et al. Mineralocorticoid Receptor Activation Contributes to the Supine Hypertension of Autonomic Failure. Hypertension. 2016;67(2):424-9
103. Okamoto LE, Gamboa A, Shibao CA, Arnold AC, Choi L, Black BK, et al. Nebivolol, but not metoprolol, lowers blood pressure in nitric oxide-sensitive human hypertension. Hypertension. 2014;64(6):1241-7
104. Shibao C, Gamboa A, Abraham R, Raj SR, Diedrich A, Black B, et al. Clonidine for the treatment of supine hypertension and pressure natriuresis in autonomic failure. Hypertension. 2006;47(3):522-6
105. Warnecke T, Schäfer KH, Claus I, Del Tredici K, Jost WH. Gastrointestinal involvement in Parkinson's disease: pathophysiology, diagnosis, and management. NPJ Parkinsons Dis. 2022;8(1):31
106. Jost WH. Gastrointestinal motility problems in patients with Parkinson's disease. Effects of antiparkinsonian treatment and guidelines for management. Drugs Aging. 1997;10(4):249-58
107. Kenna JE, Bakeberg MC, Gorecki AM, Chin Yen Tay A, Winter S, Mastaglia FL, Anderton RS. Characterization of Gastrointestinal Symptom Type and Severity in Parkinson's Disease: A Case-Control Study in an Australian Cohort. Mov Disord Clin Pract. 2021;8(2):245-53
108. Yao L, Liang W, Chen J, Wang Q, Huang X. Constipation in Parkinson's Disease: A Systematic Review and Meta-Analysis. Eur Neurol. 2023;86(1):34-44
109. Beach TG, Adler CH, Sue LI, Vedders L, Lue L, White Iii CL, et al. Multi-organ distribution of phosphorylated alpha-synuclein histopathology in subjects with Lewy body disorders. Acta Neuropathol. 2010;119(6):689-702
110. Stocchi F, Torti M. Constipation in Parkinson's Disease. Int Rev Neurobiol. 2017;134:811-26
111. Andresen V, Becker G, Frieling T, Goebel-Stengel M, Gundling F, Herold A, et al. Aktualisierte S2k-Leitlinie chronische Obstipation der Deutschen Gesellschaft für Gastroenterologie, Verdauungs- und Stoffwechselkrankheiten (DGVS) und der Deutschen Gesellschaft für Neurogastroenterologie & Motilität (DGNM) Z Gastroenterol. 2022;60(10):1528-72
112. Mearin F, Lacy BE, Chang L, Chey WD, Lembo AJ, Simren M, Spiller R. Bowel Disorders. Gastroenterology. 2016
113. Bae SH. Diets for constipation. Pediatr Gastroenterol Hepatol Nutr. 2014;17(4):203-8
114. Farid M, El Monem HA, Omar W, El Nakeeb A, Fikry A, Youssef T, et al. Comparative study between biofeedback retraining and botulinum neurotoxin in the treatment of anismus patients. Int J Colorectal Dis. 2009;24(1):115-20

115. McClurg D, Hagen S, Jamieson K, Dickinson L, Paul L, Cunnington A. Abdominal massage for the alleviation of symptoms of constipation in people with Parkinson's: a randomised controlled pilot study. Age Ageing. 2016;45(2):299-303
116. Kassolik K, Andrzejewski W, Wilk I, Brzozowski M, Voyce K, Jaworska-Krawiecka E, et al. The effectiveness of massage based on the tensegrity principle compared with classical abdominal massage performed on patients with constipation. Arch Gerontol Geriatr. 2015;61(2):202-11
117. Lee-Robichaud H, Thomas K, Morgan J, Nelson RL. Lactulose versus Polyethylene Glycol for Chronic Constipation. Cochrane Database Syst Rev. 2010(7):Cd007570
118. Luthra P, Camilleri M, Burr NE, Quigley EMM, Black CJ, Ford AC. Efficacy of drugs in chronic idiopathic constipation: a systematic review and network meta-analysis. Lancet Gastroenterol Hepatol. 2019;4(11):831-44
119. Barichella M, Pacchetti C, Bolliri C, Cassani E, Iorio L, Pusani C, et al. Probiotics and prebiotic fiber for constipation associated with Parkinson disease: An RCT. Neurology. 2016;87(12):1274-80
120. Tan AH, Lim SY, Chong KK, MAA AM, Hor JW, Lim JL, et al. Probiotics for Constipation in Parkinson Disease: A Randomized Placebo-Controlled Study. Neurology. 2021;96(5):e772-e82
121. Tan AH, Hor JW, Chong CW, Lim SY. Probiotics for Parkinson's disease: Current evidence and future directions. JGH Open. 2021;5(4):414-9

3.11 Schlaf und Tagesmüdigkeit

Autoren/Autorinnen: Friederike Sixel-Döring, Wiebke Herrmann

Fragestellung 102: Welche Symptome von Schlafstörungen können bei der PK auftreten?

Hintergrund

Schlafstörungen sowie eine erhöhte Tagesschläfrigkeit gehören zu den häufigsten nicht motorischen Symptomen der PK, können 60 bis 90% der Pat. betreffen und haben einen nachhaltig negativen Einfluss auf die Lebensqualität [1], [2]. Zudem stellen die Schlafstörungen der Pat. eine erhebliche Belastung für die Angehörigen bzw. Pflegepersonen dar [3]. Eine Behandlung der Schlafstörungen kann zu mehr Wohlbefinden und damit zur verbesserten Versorgungsqualität von Pat. mit PK beitragen. Kenntnis der Symptome sowie der zugrunde liegenden Ätiologie/Pathophysiologie sind erforderlich, um zu zielführenden Behandlungsansätzen zu kommen.

Evidenzgrundlage

Es wurden seit 2015 publizierte systematische Reviews/Metaanalysen berücksichtigt, die den aktuellen Erkenntnisstand zusammenfassen. Weitere Literatur wurde aus den Referenzen extrahiert.

Ergebnis

Pat. mit PK können folgende Symptome von Schlafstörungen aufweisen: Ein- und Durchschlafstörungen mit Erleben einer (subjektiv) verkürzten Schlafdauer (Insomnie), Schmerzen sowie unwillkürliche Bewegungen und Unruhe in den Gliedmaßen (Restless-Legs-Syndrom/limb motor restlessness), nächtliche Zunahme oder Wiederauftreten motorischer Symptome wie Hypokinese/Akinesie und Tremor, Nykturie, Verhaltensstörungen (Parasomnien), am häufigsten die REM-Schlaf-Verhaltensstörung mit Albträumen, Vokalisationen und motorischem Ausagieren, seltene andere (NREM-)Parasomnien, Ängste [4], [5], nicht erholsamer Schlaf mit Tagesschläfrigkeit und/oder
-müdigkeit sowie Atemaussetzer im Schlaf [6]. Pat. mit PK berichten dabei in Bezug auf insomnische Beschwerden v.a. über Durchschlafstörungen und Früherwachen [7], [8], weniger jedoch über Einschlafstörungen.

Diese Symptome von Schlafstörungen lassen sich einer multifaktoriellen Ätiologie zuordnen, die komorbide Schlafstörungen, motorische und nicht motorische Parkinson-Symptome während der Nacht, die Koexistenz und Einflussnahme neuropsychiatrischer Störungen wie Angst, Depression und Psychose, autonome Störungen, Nebenwirkungen der Parkinson-Medikation sowie die Auswirkungen der Neurodegeneration in zentralen schlafregulierenden Zentren umfasst [4], [8], [9], [10], [5], [7]. Ein bidirektionaler Zusammenhang zwischen Schlafstörungen und der Krankheitsprogression wird vermutet [7]. Als komorbide, sogenannte primäre Schlafstörungen werden Schlafstörungen bezeichnet, die als eigenständige Diagnosen in der ICSD-3 (International Classification of Sleep Disorders) geführt werden. Dazu zählen u.a. das Restless-Legs-Syndrom, schlafbezogene Atmungsstörungen und Parasomnien. Alle diese Schlafstörungen können bei Pat. mit PK auftreten, bei einigen lässt sich ein direkter Zusammenhang zur PK bzw. anderen Alpha-Synukleinopathien nachweisen, insbesondere bei der REM-Schlaf-Verhaltensstörung.

- **Restless-Legs-Syndrom (RLS):** Es gibt zweifellos eine klinische Überschneidung zwischen nächtlichem Bewegungsdrang bei PK als RLS-Mimic, z.B. im Rahmen des Wearing-off, und RLS, sodass eine diagnostische Differenzierung schwierig sein kann [4]. Es existieren verschiedene Studien, in denen die Prävalenz des RLS bei der PK untersucht wurde und dabei unterschiedliche Ergebnisse gefunden wurden [11], [12]. Eine Metaanalyse kam dabei jedoch zu dem Ergebnis einer bei der PK erhöhten Gesamtprävalenz [13]. Allerdings unterscheidet sich die Pathophysiologie beider Erkrankungen, sodass aktuell auch aufgrund longitudinaler Daten davon ausgegangen wird, dass das RLS bei der PK eine Komorbidität ist und bei De-novo-Pat. zunächst mit derselben Prävalenz wie bei Gesunden vorliegt, die Prävalenz allerdings mit zunehmender Krankheitsdauer und höheren Dosierungen dopaminerger Therapie ansteigt. Die Frage, ob bei Vorliegen eines primären RLS ein erhöhtes Risiko besteht, im Verlauf an einer PK zu erkranken, kann derzeit nicht abschließend beantwortet werden [14], eine prospektive, fragebogengestützte Studie zeigte zumindest eine erhöhte Inzidenz der PK bei schwer betroffenen RLS-Pat. [15].
- **Schlafbezogene Atmungsstörungen (SBAS):** Unter schlafbezogenen Atmungsstörungen werden verschiedene respiratorische Ereignisse wie obstruktive und zentrale Apnoen mit resultierenden wiederholten Entsättigungen und/oder Aufwachreaktionen (Arousal) und konsekutiven Symptomen wie fragmentiertem Nachtschlaf und Tagesschläfrigkeit subsumiert. SBAS treten bei Pat. mit PK auf, wobei verschiedene Studien zu unterschiedlichen Ergebnissen bezüglich der genauen Prävalenz und des Schweregrads der SBAS bei PK kamen [5]. Da das Auftreten von Tagesschläfrigkeit bei der PK nicht allein von dem Ausmaß der schlafbezogenen Atmungsstörung abhängig zu sein scheint und möglicherweise auch das Risiko für schwergradige SBAS bei PK nicht erhöht ist, werden eine individuelle Diagnostik und Therapie empfohlen [5]. Hierbei ist zu berücksichtigen, dass SBAS generell mit einem erhöhten Risiko für kardiovaskuläre Folgeerkrankungen einhergeht und deshalb im Fall einer schwergradigen SBAS auch bei Pat. mit PK therapiert werden sollte. Eine rezente Metaanalyse zeigte darüber hinaus auch, dass das Vorliegen einer obstruktiven Schlafapnoe (OSA) bei Parkinson-Pat. mit schwerer ausgeprägten kognitiven und motorischen Symptomen assoziiert ist [16]. Auch eine vermehrte Nykturie kann, wie auch bei SBAS-Pat. ohne PK, Ausdruck einer SBAS sein.
- **Motorische Symptome:** Nachts können gehäuft Parkinson-Symptome auftreten bzw. wieder auftreten, z.B. im Sinne eines Wearing-off.

  Dies umfasst sowohl die Unfähigkeit, sich selbst im Bett zu lagern, selbstständig aufzustehen, die Wiederkehr von Ruhetremor, Auftreten schmerzhafter dystoner Verkrampfungen im „motorischen Off“ vor allem zum Morgen hin sowie den aktigraphischen Nachweis einer im Laufe der Nacht zunehmenden Hypokinesie sowie anteilig auch eine vermehrte Nykturie. Diese Symptome signalisieren dabei v.a. einen nächtlichen dopaminergen Mangel [17], [18].
- **Neuropsychiatrische Symptome:** Angststörungen, Depression, halluzinatorisches Erleben, Psychose, abnorme nächtliche Aktivität im Rahmen von Impulskontrollstörungen verschlechtern die subjektive und objektive Schlafqualität [19], [20], [21], [5].
- **Autonome und andere nicht motorische Symptome:** Nicht motorische Symptome wie Nykturie, Obstipation, Reflux, Orthostase, Schmerzen und vermehrtes Schwitzen können zu

Schlafstörungen, insbesondere Schlaffragmentierung, führen oder vorbestehende Schlafstörungen weiter verschlechtern, insbesondere in fortgeschritteneren Krankheitsstadien [22].

- **Nebenwirkungen der Pharmakotherapie:** Dopaminerge Medikation, insbesondere Dopaminagonisten sowie Amantadin, können die Schlaffragmentation erhöhen und den Tiefschlafanteil mindern [23], [24]. Darüber hinaus werden Tagesschläfrigkeit und ungewolltes Einschlafen mit der Einnahme dopaminerger Medikation, v.a. Dopaminagonisten, in Zusammenhang gebracht [25]. Gleichzeitig können Therapeutika der PK jedoch auch die Schlafqualität, u.a. über eine Zunahme der nächtlichen Beweglichkeit, verbessern [26], [27], sodass die Nettoeffekte der medikamentösen Therapien differenziert betrachtet werden müssen. Hierbei ist zu berücksichtigen, dass unterschiedliche Dopaminagonisten je nach Dosis, Rezeptorprofil und Wirkdauer unterschiedliche Wirkungen auf den Schlaf haben können [26], [5].
- **Neurodegenerative Veränderungen in schlafregulierenden Zentren:** Die REM-Schlaf-Verhaltensstörung (RBD) gilt als prodromales Merkmal einer Alpha-Synuklein-assoziierten Neurodegeneration sowie als Progressionsmarker bei manifester PK [28], [29]. Somit erkranken Pat. mit einer initial noch klinisch isolierten RBD (iRBD) im Verlauf mit hoher Wahrscheinlichkeit an einer Alpha-Synukleinopathie, d.h. an einer PK, DLB oder MSA. Hingegen kann eine RBD auch komorbide bei anderen neurodegenerativen Erkrankungen wie der Alzheimer-Krankheit oder z.B. Tau-assoziierten atypischen Parkinson-Syndromen auftreten, hierbei ist aber auch in Einzelfällen eine histopathologische Ko-Pathologie mit Alpha-Synuklein nachgewiesen worden. Seltener tritt die RBD zudem auch im Zusammenhang mit anderen Schlafstörungen (wie beispielsweise der Narkolepsie Typ 1) auf. Die RBD kann dabei der PK um Jahre bis Jahrzehnte vorausgehen.
- Bei der Parkinson-Erkrankung werden zudem sowohl Veränderungen in der Mikrostruktur als auch eine progrediente Dekonstruktion der Makrostruktur des Schlafes mit zunehmendem Tiefschlafverlust beschrieben [10], [30], [9], [7], sodass die restaurative Funktion des Schlafs zur Gewährleistung der metabolischen Homöostase im Gehirn gestört wird. Zudem wird eine Beeinträchtigung des glymphatischen Systems – des zerebralen Pendants zum lymphatischen System, bestehend aus gefäßbegleitenden Kanälchen und Astrozyten – durch die Störung der Schlafarchitektur mit vermindertem Tiefschlafanteil vermutet, welche wiederum durch eine Einschränkung nächtlicher regenerativer Mechanismen, u.a. einer verminderten Clearance neurotoxischer Proteine wie Amyloid-ß und a-Synuklein, zu einer weiteren Beeinträchtigung schlafregulierender Zentren und möglicherweise auch einer aggravierten Neurodegeneration beiträgt, sodass ein bidirektionaler Zusammenhang zwischen Schlafstörungen und Neurodegeneration vermutet wird [7], [31], [32].
- Zusätzlich wurden bei Parkinson-Pat. Störungen der zirkadianen Rhythmik mit Veränderungen der Expression bestimmter Uhrengene („Clock genes“) und konsekutiv gestörter zirkadianer Rhythmik der ADH-Sekretion, der Nierentätigkeit, der Blutdruckregulation, der rhythmischen Transmitter- und Hormonsekretion, u.a. von Cortisol und Melatonin, sowie ein fortschreitender Verlust orexinerger Neurone im präfrontalen Kortex und Hypothalamus mit resultierenden, signifikant erniedrigten Melatonin- und Orexin-Spiegeln nachgewiesen [10], [33], [34], [35]. Auch

hierbei wird eine bidirektionale Beziehung zwischen der gestörten zirkadianen Rhythmik und den neurodegenerativen Prozessen angenommen [35].

Begründung der Empfehlung

Die Literatur zeigt, dass Schlafstörungen bei der PK häufig sind und mit Einschränkungen der Aktivitäten des täglichen Lebens, des Wohlbefindens in der Nacht sowie der Lebensqualität einhergehen. Deshalb ist eine ausführliche diagnostische und auch therapeutische Würdigung von Schlafstörungen so wie auch anderen nicht motorischen Symptomen bei der PK notwendig.

**Empfehlung**	**Neu**<br>**Stand (2023)**
Schlafstörungen bei der PK treten bei den Betroffenen häufig und mit vielfältiger Symptomatik auf und sollten bei den diagnostischen und therapeutischen Entscheidungen berücksichtigt werden. Hierzu gehören: Insomnie (Ein- und Durchschlafstörungen), Schmerzen, Restless-Legs-Syndrom, Akinese, Tremor und Rigor, Nykturie, Parasomnien, Atmungsstörungen, Albträume, REM-Schlafstörungen.	
Konsensstärke: 100%, starker Konsens	

Fragestellung 103: Welche Screeninginstrumente existieren für Nachtschlafstörungen bei der PK?

Hintergrund

Die klinische Bedeutung von Schlafstörungen für Parkinson-Pat. und ihr Umfeld steht oft im Kontrast zur fehlenden spontanen Beschwerdeschilderung in der ärztlichen Konsultation. Neben der gezielten Schlafanamnese sowie der Fremdanamnese mittels Befragung von Bettpartnern/Betttpartnerinnen sowie Pflegepersonen ermöglicht die systematisierte Abfrage mit validierten Fragebögen hier einen ersten Überblick, erste ätiologische Rückschlüsse sowie eine Beurteilung des Schweregrads. Zusätzlich können Schlaftagebücher wie Morgen- und Abendprotokolle zur Anwendung kommen.

Evidenzgrundlage

Es wurden die Empfehlungen eines von der Movement Disorder Society beauftragten Expertengremiums übernommen und die Validierungsstudien der jeweiligen Screening-Fragebögen berücksichtigt.

Ergebnis

Folgende Skalen für das Screening auf Schlafprobleme sowie die Bewertung des Schweregrads der Schlafstörung bei der PK werden empfohlen: die Parkinson’s Disease Sleep Scale (PDSS-2), der Pittsburgh Sleep Quality Index (PSQI) und Scales for Outcomes in Parkinson’s Disease - Sleep Scale (SCOPA-S) [36].

- PDSS-2: Basierend auf der ursprünglichen PDSS als visueller Analogskala [37], wurde inzwischen eine numerische Skala als PDSS-2 validiert [38]. Mit 15 Fragen werden neben der subjektiven Schlafqualität und Erholsamkeit des Schlafes auch PK-spezifische nächtliche Symptome wie

Akinese, Nykturie, Albträume, Bewegungsunruhe, nächtliche Schmerzen sowie nächtliche Atmungsprobleme in der letzten Woche erfragt. Bei der PDSS-2 wurde eine Punktzahl von > 18 als relevante Schlafbeeinträchtigung eingeschätzt. Ab diesem Wert ist eine weitere Abklärung mittels vPSG wünschenswert [39].

- PSQI: Dieser 19 Punkte umfassende Fragebogen erfasst die Schlafqualität, Schlafgewohnheiten und schlafbezogenen Symptome der letzten 4 Wochen. Ein Schwellenwert von > 5 Punkten wurde als Abgrenzung von normalem zu auffälligem Schlaf validiert [40].
- SCOPA-S: Dieser mehrere Subskalen umfassende Fragebogen erfasst ebenfalls PK-spezifisch nächtliche Schlafstörungen und Tagesschläfrigkeit in den letzten 4 Wochen, ohne allerdings potenzielle Ursachen abzufragen [41], [42].

Begründung der Empfehlung

Die Empfehlungen zum Einsatz der verschiedenen Skalen beruhen auf Expertenmeinungen. Eine validierte Vergleichsstudie zwischen den verschiedenen Skalen existiert nicht. Da jedoch bekanntermaßen objektive (z.B. auf der Polysomnographie im Schlaflabor basierende) und subjektive Schlafqualität oft diskrepant sind, ist eine externe Validierung bei fehlendem Goldstandard schwierig bzw. nicht möglich. Die Übereinstimmung zwischen den verschiedenen Fragebögen wurde bei der PK nicht systematisch untersucht, insgesamt ist die Übereinstimmung in Studien, in denen mehrere Fragebögen verwendet wurden, jedoch gering, was auch an den unterschiedlich konzipierten Fragen sowie erfassten Zeiträumen festgemacht werden kann.

Zusammenfassend lässt sich sagen, dass der PSQI nicht speziell für die Erfassung von Parkinson-assoziierten Schlafstörungen validiert wurde, sodass er eher als Screeninginstrument für das Vorhandensein einer generellen Schlafbeeinträchtigung eingesetzt werden kann. Die SCOPA-S ist ebenso wie die PDSS-2 für die Erfassung von Schlafstörungen bei der PK validiert und erfasst zusätzlich Tagesschläfrigkeit. Die PDSS hingegen erlaubt, ein Profil potenzieller Ursachen für „schlechten Schlaf" zu erstellen. Therapiestudien zum Schlaf bei der PK verwenden meist die PDSS-2 in ihrer numerischen Version, die einfache Vergleiche und Verlaufskontrollen ermöglicht [42].

**Empfehlung**	**Neu**<br>**Stand (2023)**
Als Screeninginstrument für Schlafstörungen bei der PK soll die PDSS-2 eingesetzt werden, da die numerische Skala einfache Vergleiche erlaubt und die Auswertung der abgefragten Beschwerden und Symptome Hinweise auf potenzielle Ursachen der Schlafstörungen gibt.	
Konsensstärke: 100%, starker Konsens	

Fragestellung 104: Wie werden Nachtschlafstörungen bei der PK objektiv diagnostiziert?

Hintergrund

Die o.g. Fragebögen als Screeninginstrumente erlauben – außer bei eindeutigem Wiederauftreten motorischer Parkinson-Symptome in der Nacht – keine ätiologische Zuordnung von

Nachtschlafstörungen zur Ursache, zumal der Betroffene seine Schlafstörung nur aus der subjektiven Wahrnehmung schildern kann. Eine Fremdanamnese sowie Schlaftagebücher können zur weiteren Diagnostik hilfreich sein. Eine objektive Beurteilung erfordert jedoch den Einsatz von apparativer Diagnostik, die den Ablauf des Nachtschlafs mit der Aufzeichnung motorischer Phänomene und Verhaltensweisen koordiniert beurteilen lässt.

Evidenzgrundlage

Die Empfehlungen zur apparativen Diagnostik gemäß der S2K-Leitlinie „Insomnie bei neurologischen Erkrankungen“ [43] sowie Hinweise zur Diagnostik aus dem „Handbuch Schlafmedizin“ [44] sowie die International Classification of Sleep Disorders 3rd Edition (ICSD-3) wurden berücksichtigt. Des Weiteren wurde Pubmed durchsucht nach validierter Weiterentwicklung der verschiedenen Methoden.

Ergebnis

Die Aktigraphie kann eingesetzt werden, um Bett- und Schlafenszeiten über den gesamten Tag zu erfassen [43]. Sie ermöglicht mittels Akzelerometrie das Abschätzen von Aktivitäts- und Ruhephasen auch über längere Zeiträume in der häuslichen Umgebung, was eine Kalkulation der Gesamtschlafzeit, der Schlafeffizienz, der Einschlaflatenz und der Schlaffragmentation zulässt, wobei die Genauigkeit mit zunehmender Krankheitsschwere abnimmt [45]. Neue Studiendaten weisen auf die Möglichkeit der Detektion einer REM-Schlaf-Verhaltensstörung mittels Aktigraphie hin [46]. Die Aktigraphie ermöglicht zudem ein Screening auf das Vorliegen zirkadianer Rhythmusstörungen.

Die kardiorespiratorische Polygraphie erlaubt die Integration von Daten zu Atmung, Herztätigkeit, Oxigenierung sowie zur Körperposition und wird in erster Linie zum Screening auf schlafbezogene Atmungsstörungen eingesetzt [44]. In Weiterentwicklung befindlich sind Polygraphiegeräte, die mittels EEG-Kanälen zu einer fast vollständigen Polysomnographie aufgerüstet werden und somit eine Zwischenstellung zwischen der ambulanten Polygraphie und der Video-Polysomnographie im Schlaflabor einnehmen können.

Die videogestützte kardiorespiratorische Polysomnographie (vPSG) im Schlaflabor ist der Goldstandard der schlafmedizinischen Diagnostik. Die vPSG ermöglicht über die zusätzliche Ableitung des EEG und Aufzeichnung des Muskeltonus die Bestimmung der Schlafstadien und damit eine Beurteilung der Schlafeffizienz, Schlafstruktur sowie mittels der synchronisierten Videoaufzeichnung die Analyse und Zuordnung des Verhaltens im Schlaf/in der Nacht. Für die sichere Diagnose einer RBD ist eine vPSG erforderlich (ICSD-3). Des Weiteren ermöglicht die vPSG die Abklärung nächtlicher Störungen, deren Ursache sich nicht aus einer sorgfältigen Anamnese/Fremdanamnese bzw. den o.g. Screeninginstrumenten erschließt. In einer multizentrischen Studie wurde eine Punktzahl von > 18 auf der PDSS-2 als Grenzwert validiert, ab dem eine Schlafstörung klinisch relevant bei der PK ist und eine vPSG zur Abklärung der Schlafstörung empfohlen wird [39].

Begründung der Empfehlung

Objektive Verfahren wie die Aktigraphie, die kardiorespiratorische Polygraphie und die Polysomnographie sollten zur Diagnostik von Schlafstörungen bei der PK eingesetzt werden,

insbesondere dann, wenn die Anamnese, Fremdanamnese sowie Screeninginstrumente keine eindeutige Zuordnung der Schlafstörung zulassen oder wenn behandlungsbedürftige Schlafstörungen wie beispielsweise schwergradige Insomnien, Tagesschläfrigkeit, z.B. mit möglicher Beeinträchtigung der Fahrtauglichkeit, oder therapierelevante schlafbezogene Atmungsstörungen vorliegen oder vermutet werden, deren Behandlung dringend indiziert ist.

Empfehlung	Neu<br>Stand (2023)
▪ Bei Vorliegen relevanter therapiebedürftiger Schlafstörungen, insbesondere, wenn erste therapeutische Maßnahmen wie die Optimierung der Medikation nicht zu einer Besserung geführt haben, sollten objektive Messverfahren wie die Aktigraphie, die kardiorespiratorische Polygraphie oder die Video-Polysomnographie erwogen werden.<br>▪ Die Aktigraphie kann erwogen werden zur Abschätzung der Gesamtschlafzeit, der Schlafeffizienz sowie der Wachzeit bei Parkinson-Pat. Hiermit kann auch der Schlafrhythmus über einen längeren Zeitraum beurteilt werden. Ihre Genauigkeit lässt jedoch insbesondere bei Pat. in fortgeschritteneren Krankheitsstadien nach.<br>▪ Die vPSG ist der Goldstandard der objektivierbaren schlafmedizinischen Diagnostik. Sie soll eingesetzt werden zur Diagnosesicherung einer RBD. Bei dem Verdacht auf das Vorliegen einer schwergradigen schlafbezogenen Atmungsstörung sollten eine Polygraphie sowie ggf. eine Polysomnographie durchgeführt werden.	
Konsensstärke: 100%, starker Konsens	

Fragestellung 105: Wie werden Nachtschlafstörungen bei der PK differenziell therapiert?

Hintergrund

Die Vielfalt der klinischen Manifestationen und zugrunde liegenden Pathomechanismen der Schlafstörungen bei der PK implizieren, dass eine Therapie individuell gemäß der im Vordergrund stehenden Symptome und der spezifischen schlafbezogenen Diagnose konzipiert werden muss. Es gibt allerdings nur wenige randomisierte kontrollierte Studien zu Schlafstörungen bei der PK.

Evidenzgrundlage

Es wurden eine narrative Übersichtsarbeit aus 2022, eine systematische Übersichtsarbeit aus 2017 über Publikationen zwischen 2005 und 2015 zum Thema sowie eine weitere Übersichtsarbeit aus 2021 ausgewertet. Auswahlkriterium war die Berücksichtigung einer ätiologischen Spezifikation in den zitierten Therapieansätzen. Des Weiteren wurden die aktualisierten Empfehlungen der Movement Disorder Society berücksichtigt. Weitere spezifische Therapieansätze/Therapiestudien wurden den Referenzen entnommen. Bei entsprechender Indikation werden Querverweise auf andere gültige Leitlinien benannt.

Ergebnis

Orientiert an der ätiologischen Zuordnung/schlafmedizinischen Diagnose, ergeben sich folgende Therapieempfehlungen:

- **Komorbide, primäre Schlafstörungen:**

  RLS: Es gibt keine randomisierten kontrollierten Studien zur Therapie des RLS, welches konkomitant zu einer PK auftritt. Gemäß der S2k-LL RLS [47] ist zunächst der Eisenstoffwechsel zu kontrollieren; Ferritin und Transferrinsättigung sind auf hochnormale Werte anzuheben und RLS-aggravierende Medikamente wie Antidepressiva sollten – wenn möglich – reduziert bzw. abgesetzt werden. Falls diese Maßnahmen nicht ausreichen, kann neben einer Optimierung der nächtlichen dopaminergen Therapie gemäß dem individuellen Risikoprofil vor allem auch der Einsatz von Alpha-2-delta-Liganden oder Opioiden erwogen werden [14], [4].

  Schlafbezogene Atmungsstörungen: Es besteht Einigkeit in allen hierzu gefundenen Veröffentlichungen [14], [4], [48], dass diese gemäß den gültigen Leitlinien (teilaktualisierte S3-LL der DGSM [49]) zu behandeln sind. Hierbei werden bei begleitender Adipositas eine Gewichtsreduktion sowie als Goldstandard eine nächtliche Überdrucktherapie (PAP-Therapie, überwiegend als CPAP, ggf. als BiPAP) empfohlen. Eine randomisierte kontrollierte Studie konnte zeigen, dass eine CPAP-Therapie bei PK-assoziierter SBAS den Apnoe-Hypopnoe-Index reduzieren und die Schlaflatenz verkürzen konnte [50]. Allerdings ist die Adhärenz an diese Therapien oft gering und belastet teilweise in der Anwendung sowohl Pat. als auch Angehörige, sodass hier auch eine Nutzen-Aufwand-Auswirkungen-Abwägung unter Berücksichtigung der Lebensqualität der Pat. und der Angehörigen erfolgen sollte.

- **Wiederauftreten von Parkinson-Symptomen in der Nacht als „wearing-off“:**

  Zeigen sich motorische Symptome der PK wie nächtliche Akinese, Off-assoziierte Schmerzen, andere motorische oder nicht motorische Fluktuationen oder auch Dyskinesien, sollte die dopaminerge Therapie modifiziert werden [14], [48].

  Deshalb empfiehlt sich ein differenzielles Vorgehen: Bei Hinweisen auf nächtliches „wearing-of“ und Fluktuationen ist eine Optimierung der dopaminergen Therapie, z.B. mittels Etablierung oder Optimierung langwirksamer Dopaminagonisten oder retardierter Levodopa-Präparate oder ggf. sogar Pumpentherapien, und anschließende Reevaluation der Schlafqualität zu empfehlen. Bei erhöhter Tagesschläfrigkeit sollte der Versuch einer Reduktion der dopaminergen Therapie erfolgen, ggf. eine Umstellung auf einen anderen Dopaminagonisten bzw. auf Levodopa. Zusätzlich sollte eine Abklärung zum Ausschluss einer schlafbezogenen Atmungsstörung erfolgen und entsprechende Therapien eingeleitet werden. Bei anhaltenden Schlafstörungen sollte zum Ausschluss einer behandlungsbedürftigen, primären Schlafstörung ggf. eine objektive Abklärung, z.B. mittels vPSG, erwogen werden.

- **Neurodegenerative Veränderungen in schlafregulierenden Zentren:**

  RBD: Eine RBD soll diagnostisch gesichert und von anderen (motorischen) nächtlichen Verhaltensstörungen, nächtlicher Unruhe, Verwirrtheit, Halluzinationen abgegrenzt werden. Nach wie vor gibt es keine Behandlungsempfehlung mit hohem Evidenzniveau aus klinischen Studien zur Behandlung der RBD. Nach Expertenmeinung wird das Schaffen einer sicheren Schlafumgebung empfohlen, der Einsatz von Clonazepam oder Melatonin oder Rivastigmin kann versucht werden, wobei beim Einsatz von Clonazepam mögliche Überhangeffekte und unerwünschte Wirkungen auf Alertheit und Gleichgewicht insbesondere bei älteren Pat. zu

beachten sind [51]. Eine in 2022 publizierte Analyse aller bisherigen klinischen und wissenschaftlichen Evidenz zur pharmakologischen Behandlung der RBD weist die meisten positiven Ergebnisse für Clonazepam und Melatonin nach, die als Mittel der ersten Wahl gelten, wenngleich einschränkend darauf hingewiesen wird, dass Zweifel an der Eignung der bisherigen Messmethoden zur präzisen und objektiven Erfassung der RBD-Schwere bestehen [52], sodass der Evidenzgrad entsprechend abgeschwächt wird. Weitere Therapieansätze, wie beispielsweise der Einsatz von Gamma-Hydroxybuttersäure (GHB, Xyrem), sind bisher weder ausreichend validiert noch sind sie aufgrund des Nebenwirkungsprofils generell anzuraten, zumal eine Verordnung nur über BtM-Zulassung möglich ist [53].

Störungen der zirkadianen Rhythmik: Grundsätzlich besteht eine pathophysiologische Rationale für die Applikation von Melatonin zur Behandlung einer PK-assoziierten Insomnie auf dem Boden einer zirkadianen Rhythmusstörung. Eine multizentrische randomisierte, doppelblinde, placebokontrollierte Studie an insgesamt nur 34 Pat. zeigte die Sicherheit und Effektivität von 2 mg Melatonin mit verzögerter Freisetzung, allerdings gemessen mit dem Nicht-Parkinson-spezifischen PSQI, der Non-motor Symptom Scale NMSS sowie dem PDQ39 [54]. In einer doppelblinden, placebokontrollierten randomisierten Pilotstudie hingegen konnte kein positiver Effekt auf die subjektive Schlafqualität oder die BMAL1-Spiegel als Substrat durch Melatonin nachgewiesen werden [55]. Das MDS-EBM Committee stuft den Einsatz von Melatonin bei insgesamt schwacher Evidenz in Anbetracht des günstigen Nebenwirkungsprofils als möglicherweise nützlich und damit zu rechtfertigen ein [56].

Neben allgemeinen Hinweisen zur Schlafhygiene [14] wurden auch weitere nicht pharmakologische Maßnahmen untersucht, die am ehesten der zirkadianen Rhythmisierung zuzuordnen sind: Eine randomisierte klinische Studie zeigte einen positiven Effekt auf die Schlafqualität und körperliche Aktivität tagsüber durch Anwendung von hellem Licht [57]. In einer offenen, retrospektiven longitudinalen Studie wurde deren anhaltender positiver Effekt nachgewiesen [58]. Hochintensives körperliches Training besserte im Vergleich zu schlafhygienischen Maßnahmen in einer randomisierten, kontrollierten Studie die Schlafqualität sowie die polysomnographisch gemessene Schlafeffizienz von Parkinson-Pat. signifikant [54]. Eine Metaanalyse aller Studien zu körperlichem Training bestätigte die Effektivität für eine mittlere bis maximale Trainingsintensität [59].

Störungen der Makro- und Mikrostruktur des Schlafs mit Tiefschlafverlust: Diese manifestieren sich als anderweitig nicht erklärbare Ein- und Durchschlafstörungen sowie nicht erholsamer Schlaf. Hierbei steht im Vordergrund der Ausschluss anderer Ursachen der Schlafstörungen, u.a. RLS oder schlafbezogener Atmungsstörungen sowie unzureichender Medikation/Nebenwirkungen der Therapie. Nach Ausschluss kausal behandelbarer Ursachen der Insomnie können auch medikamentöse Therapieoptionen erwogen werden.

Es gibt derzeit keine schlafrestaurativen pharmakologischen Therapieansätze. Zur symptomatischen Therapie sei auf das Kapitel 8.2 „Insomnie bei neurodegenerativen Bewegungsstörungen“ der Leitlinie „Insomnie bei neurologischen Erkrankungen“ [43] verwiesen, wo bei allerdings unzureichender Evidenz der Einsatz von Eszopiclon, Doxepin, Zolpidem, Trazodon, Ramelteon, Melatonin sowie des Antipsychotikums Pimavanserin und der

Antidepressiva Venlafaxin und Nortriptylin (bei komorbider Depression) zur Verbesserung der subjektiven Schlafqualität als möglich erklärt wird. Basierend auf einer randomisierten kontrollierten Studie an nicht PK-betroffenen insomnischen Pat. [60], wird in der klinischen Praxis auch das tetrazyklische Antidepressivum Mirtazapin in einer Dosis von 7,5 bis 15 mg eingesetzt. Kürzlich wurde ein positiver Effekt von Zonisamid auf die subjektive Schlafqualität von Parkinson-Pat. vom Tremor-Dominanztyp in einer offenen Studie gezeigt, sodass auch diese Substanz in dieser Pat.-Subgruppe erwogen werden kann [61].

Begründung der Empfehlung

Komorbide primäre Schlafstörungen wie RLS und SBAS sollen gemäß der für diese Entitäten gültigen Leitlinien therapiert werden. Ergeben sich Hinweise, dass motorische oder nicht motorische dopaminerge Wirkfluktuationen für die Schlafstörung verantwortlich sind, soll eine entsprechende Anpassung der dopaminergen Therapie erfolgen. Auch wenn das Evidenzniveau nicht hoch ist, soll die RBD durch das Schaffen einer sicheren Schlafumgebung behandelt werden. Des Weiteren können Clonazepam und/oder Melatonin unter Beachtung möglicher unerwünschter Effekte eingesetzt werden. Die höhere Empfehlungsstärke für die nicht pharmakologische Intervention der sicheren Schlafumgebung ergibt sich aus deren fehlenden Nebenwirkungen. Bei Schlafstörungen, die nicht den vorgenannten Entitäten zuzuordnen und somit eher Folgen der krankheitsimmanenten Schlafstruktur- und zirkadianen Rhythmusstörung sind, vordringlich insomnische Beschwerden, sollten behandelbare Ursachen wie beispielsweise medikationsbedingte Schlafstörungen und insbesondere primär behandelbare Schlafstörungen wie RLS und SBAS ausgeschlossen werden. Nach Ausschluss spezifisch behandelbarer Ursachen der Schlafstörungen sollten diese durch schlafhygienische Maßnahmen, intensives körperliches Training und Lichttherapie behandelt werden. Eszopiclon, Doxepin, Zolpidem, Trazodon, Melatonin, Venlafaxin, Nortriptylin, Mirtazapin können bei insgesamt schwacher Evidenzlage bei der Insomnie erwogen werden. Die stärkere Empfehlung für die nicht pharmakologischen Interventionen ergibt sich wiederum aus deren fehlenden Nebenwirkungen.

Empfehlung	Neu<br>Stand (2023)
▪ Komorbide primäre Schlafstörungen wie RLS und SBAS sollen gemäß den für diese Entitäten gültigen Leitlinien therapiert werden.<br>▪ Ergeben sich Hinweise darauf, dass motorische oder nicht motorische dopaminerge Wirkfluktuationen für die Schlafstörung verantwortlich sind, soll eine entsprechende Anpassung der dopaminergen Pharmakotherapie erfolgen.<br>▪ Die RBD soll durch das Schaffen einer sicheren Schlafumgebung behandelt werden. Des Weiteren können Clonazepam und/oder Melatonin unter Beachtung möglicher Nebenwirkungen erwogen werden.<br>▪ Bei der Insomnie oder zirkadianen Rhythmusstörungen sollten zugrunde liegende Ursachen wie Medikationsnebenwirkungen und/oder primäre Schlafstörungen wie SBAS ausgeschlossen werden. Nach Ausschluss spezifisch behandelbarer Ursachen der Schlafstörungen sollten diese durch schlafhygienische Maßnahmen, intensives körperliches Training und Lichttherapie behandelt werden. Eszopiclon, Doxepin, Zolpidem, Trazodon, Melatonin, Venlafaxin (bei	

komorbider Depression), Nortriptylin oder Mirtazapin können bei insgesamt schwacher Evidenzlage bei der Insomnie erwogen werden.
Konsensstärke: 95%, Konsens

Fragestellung 106: Wie effektiv sind retardierte Levodopa-Präparate oder Dopaminagonisten für die Behandlung von nächtlichen motorischen Symptomen bei der PK?

Hintergrund

Die Klagen von Parkinson-Pat. über nächtliches Wiederauftreten von motorischen Parkinson-Symptomen mit erschwertem Drehen im Bett, Problemen beim selbstständigen Lagern, Aufstehen aus dem Bett zum Toilettengang bis hin zu Off-dystonen Krämpfen oder Schwierigkeiten beim Einschlafen durch Wiederkehr des Ruhetremors legen die Verabreichung langwirksamer Dopaminergika vor der Nachtruhe nahe.

Evidenzgrundlage

Für diese Frage wurden die aktualisierten Empfehlungen des MDS-EBM Committee sowie eine danach publizierte Übersichtsarbeit analysiert, des Weiteren wurde Pubmed nach Studien durchsucht, die aufgrund ihres Publikationsdatums noch keine Berücksichtigung in den vorgenannten Übersichten gefunden haben.

Ergebnis

Das MDS-EBM Committee [56] bewertet in der Rubrik der Dopaminergika einzig den Einsatz von Rotigotin als wahrscheinlich effektiv. Für Levodopa-/Carbidopa-Präparate mit verzögerter Freisetzung sowie für den Dopaminagonisten Piribedil wird die Evidenz als unzureichend eingestuft. Retardiertes Levodopa/Carbidopa und Piribedil können ohne spezielles Monitoring mit akzeptablem Risiko eingesetzt werden. Eine Verbesserung von mittels PDSS erfassten Schlafstörungen durch den Einsatz eines Levodopa-/Carbidopa-/Entacapon-Präparats zur Nacht konnte in einer offenen Anwendungsbeobachtung gezeigt werden [62]. Eine randomisierte, plazebokontrollierte Studie zum Schlaf bei Parkinson-Pat. mit Rotigotin konnte den Nachweis einer signifikanten Verbesserung auf der PDSS-2 führen [26]. Eine weitere doppelblinde, placebokontrollierte Studie wies mittels Polysomnographie eine signifikante Zunahme der Schlafeffizienz, eine Reduktion von „wake after sleep onset“ (WASO; ein Maß für die Schlaffragmentierung) sowie eine Verkürzung der Schlaflatenz unter Rotigotin nach [63]. Eine Metaanalyse von randomisierten, kontrollierten Studien zu Rotigotin, in die Daten von insgesamt 1.187 Pat. einflossen [26], zeigte eine signifikante Verbesserung der Schlafstörungen bei PK unter Rotigotin im Vergleich zu Placebo. Im Vergleich von Rotigotin zu Pramipexol erwiesen sich beide Dopaminagonisten als gleichermaßen effektiv, im Vergleich zu Ropinirol zeigte sich Rotigotin als non-inferior. Eine weitere, retrospektive Analyse aus der klinischen Studie zu Pramipexol (NCT00466167) verglich den Effekt von retardiertem Pramipexol gegen Pramipexol mit sofortiger Freisetzung auf Schlafstörungen, die mit der PDSS erfasst wurden: Beide Präparationen führten zu Verbesserungen nächtlicher Störungen, mit numerisch stärkerer, jedoch statistisch nicht signifikanter Verbesserung unter der retardierten Galenik [64].

Mit Apomorphin wurde ein weiterer Dopaminagonist getestet, der als ausschließlich subkutan zu applizierendes Präparat zu den Eskalationstherapien für Parkinson-Pat. in fortgeschrittenen Krankheitsphasen mit schweren Fluktuationen gehört: Eine kleine Studie an 17 Pat. mit Schlafstörungen, die tagsüber die subkutane Apomorphin-Infusionstherapie benutzten, zeigte eine signifikante subjektive Verbesserung des Nachtschlafs sowie der Tagesschläfrigkeit, als die Apomorphin-Infusion auf die Nacht ausgedehnt wurde [65]. Eine multizentrische, randomisierte, kontrollierte doppelblinde Crossover-Studie untersuchte die Wirkung einer ausschließlich nächtlichen subkutanen Apomorphin-Infusion bei fluktuierenden Parkinson-Pat. mit mittelgradigen bis schweren Schlafstörungen (NCT02940912) und wies eine signifikante Verbesserung der Schlafqualität auf der PDSS unter Apomorphin im Vergleich zu Placebo nach [66].

Auch die intestinale Levodopa-Instillationstherapie über eine PEG-J als weitere Eskalationstherapie für Parkinson-Pat. mit schweren Fluktuationen wurde bezüglich ihrer Wirkung auf Schlafstörungen getestet: Eine erste prospektive polysomnographisch gestützte Pilotstudie an 7 Parkinson-Pat. zeigte einen statistisch nicht signifikanten Trend zur Verbesserung der subjektiven Schlafqualität nach 6-monatiger Anwendung der Levodopa-Instillation, die Schlafarchitektur wurde hingegen nicht beeinflusst [67]. Eine multinationale prospektive Anwendungsbeobachtung (NCT02611713) von Levodopa-/Carbidopa-Intestinalgel ergab eine signifikante Verbesserung der subjektiven Schlafqualität sowie der Tagesschläfrigkeit [68].

Zu berücksichtigen ist beim Einsatz speziell der Dopaminagonisten jedoch ihre unerwünschte Eigenschaft, Tagesschläfrigkeit und plötzliche Schlafattacken auslösen zu können, wobei es sich offenbar um einen dosisabhängigen Effekt handelt: Höhere Dosierungen induzieren vermehrt Wachphasen während des Nachtschlafs, erhöhen die Schlaffragmentierung und mindern den Tiefschlafanteil, während niedrige Dosierungen die Schlafqualität verbessern und Wachphasen während des Nachtschlafs mindern [48].

Ob der in einer Übersichtsarbeit [69] beschriebene positive Effekt der Tiefen Hirnstimulation auf polysomnographisch erfasste objektive Schlafparameter wie Schlaflatenz und Schlaffragmentierung eine direkte Auswirkung der Stimulation oder vielmehr eine Folge der damit verbundenen Reduktion der dopaminergen Medikation ist, kann derzeit nicht beantwortet werden.

Begründung der Empfehlung

Zu Rotigotin liegen inzwischen mehrere randomisierte kontrollierte Studien vor, die eine Verbesserung der Schlafstörungen bei PK nachweisen, sodass gemäß den EBM-Kriterien eine starke Empfehlung für den Einsatz des Rotigotin-Pflasters gegeben werden kann. Die Evidenzlage für den Einsatz von anderen Dopaminagonisten wie Pramipexol oder Ropinirol ist mangels entsprechender RCTs schwächer, ausgehend vom Wirkprinzip insbesondere in der verzögerten Freisetzungsform jedoch auch empfehlenswert.

Für Pat. in weit fortgeschrittenen Krankheitsphasen, die zur Symptomkontrolle mit einer Pumpentherapie versorgt werden, kann bei entsprechender Einschränkung der Nachtschlafqualität die Ausdehnung der Pumpenlaufzeit auf die Nacht erwogen werden.

Bei der Bewertung der Evidenzlage ist zu berücksichtigen, dass es nur für die Pharmazeutika Evidenz geben kann, die in entsprechenden randomisierten kontrollierten Studien untersucht wurden. Viele Substanzen, die ihren berechtigten Platz in der Pharmakotherapie der PK haben, wurden bislang nicht mit dem primären Endpunkt der Besserung der Schlafstörungen getestet. So erklärt sich z.B. die schlechte Evidenzlage für retardierte Levodopa-Präparate. Die klinische Erfahrung zeigt jedoch klar den Nutzen dieser Präparate, wenn Parkinson-Pat. nachts durch Wiederauftreten von motorischen oder nicht motorischen Symptomen gestört werden. Insbesondere bei Kontraindikationen für den Einsatz von Dopaminagonisten sollten retardierte Levodopa-Präparate erwogen werden.

**Empfehlung**	**Neu**<br>**Stand (2023)**
▪ Bei Schlafstörungen sollte bei Hinweisen auf einen nächtlichen dopaminergen Mangel (wie beispielsweise „wearing-off“) der Einsatz langwirksamer Dopaminagonisten, wie beispielsweise Rotigotin, angeboten werden.<br>▪ Alternativ kann der Einsatz von retardierten Levodopa-Präparaten angeboten werden.<br>▪ Für Pat. mit Wirkfluktuationen und Dyskinesien, die zur Symptomkontrolle mit einer Pumpentherapie versorgt werden, kann bei entsprechender Einschränkung der Nachtschlafqualität die Ausdehnung der Pumpenlaufzeit auf die Nacht angeboten werden.	
Konsensstärke: 100%, starker Konsens	

Fragestellung 107: Welche Screeninginstrumente existieren für exzessive Tagesschläfrigkeit bei der PK?

Hintergrund

Erhöhte Tagesschläfrigkeit und plötzliches ungewolltes Einschlafen – sog. Schlafattacken – zeigen eine erhöhte Prävalenz bei Parkinson-Pat. im Vergleich zur Normalbevölkerung und bedeuten ein erhebliches Gesundheitsrisiko, tragen zur Krankheitslast bei, schränken Tagesaktivitäten und Teilhabe ein und mindern die Lebensqualität [70]. Zudem zeigte sich in einer großen epidemiologischen Studie, dass Tagesschläfrigkeit ein frühes Symptom der PK darstellt [71]. Dabei scheint es jedoch auch so zu sein, dass Tagesschläfrigkeit sich in fortgeschritteneren Krankheitsstadien aggraviert und auch verstärkt auftritt [72]. Dabei konnte eine prospektive, cross-sektionale Analyse an 550 älteren Pat. mit verschiedenen Erkrankungen in Pflegeheimen eine Assoziation zwischen Tagesschläfrigkeit und PK nachweisen, wobei sich die Tagesschläfrigkeit als negativer Prädiktor für Mortalität darstellte [73].

Evidenzgrundlage

Als Grundlage dienten die rezenteste Übersichtsarbeit zum Thema Tagesschläfrigkeit bei PK aus der Pubmed von 2022 [74], sowie die Validierungsstudien der Epworth Sleepiness Scale (ESS)

Ergebnis

Es ist zwischen Tagesschläfrigkeit und Müdigkeit/Fatigue zu unterscheiden, wobei es zwischen den beiden Symptomen Überschneidungen gibt und Pat. sich in beiden Fällen als müde,

konzentrationsgemindert und erschöpft beschreiben können. Jedoch schlafen die der an Fatigue/Müdigkeit leidenden Pat. in Ruhephasen nicht (ungewollt) ein. Die an Tagesschläfrigkeit leidenden Pat. hingegen werden kurze Schlafintrusionen unter Umständen nicht bewusst als solche wahrnehmen.

Insofern kommt der Fremdanamnese durch das soziale Umfeld besondere Bedeutung zu [74]. Wie schon in der Fragestellung zu den Screeninginstrumenten erwähnt, sind Fragebögen zur Erfassung der Tagesschläfrigkeit bei der PK validiert. Die SCOPA-S erfasst dabei auch Tagesschläfrigkeit in den letzten 4 Wochen. Validiert zur Erfassung und Graduierung von Tagesschläfrigkeit wurde jedoch insbesondere die ESS [75]. Meist wird ein Cut-off von > 10 Punkten als Grenzwert für exzessive Tagesschläfrigkeit angewendet. Ihre Anwendbarkeit bei Parkinson-Pat. konnte nachgewiesen werden [76]. Allerdings muss bei der Anwendung der Fragebögen berücksichtigt werden, dass ein erheblicher Unterschied zwischen subjektiver und objektiver Tagesschläfrigkeit bestehen kann [77]. Zudem zeigte sich in einer Studie an PK-Pat. nach Behandlung mit Rotigotin zwar eine Besserung des Nachtschlafs, jedoch ohne Auswirkungen auf die objektive und subjektive Tagesschläfrigkeit [78], sodass bei der Tagesschläfrigkeit wahrscheinlich weitere Mechanismen, wie beispielsweise Einwirkungen unterschiedlicher Transmittersysteme wie des serotonergen/orexinergen Systems oder anderer Faktoren eine Rolle spielen, die bisher nur unzureichend verstanden sind.

Begründung der Empfehlung

Empfehlung	Neu<br>Stand (2023)
▪ Neben Anamnese und Fremdanamnese ist die Epworth Sleepiness Scale (ESS) als Screeninginstrument für Tagesschläfrigkeit bei der PK validiert und sollte angewendet werden.	
Konsensstärke: 100%, starker Konsens	

Fragestellung 108: Wie wird exzessive Tagesschläfrigkeit bei der PK objektiv diagnostiziert?

Hintergrund

Tagesschläfrigkeit bedeutet nach der ICDC-3-Definition eine Unfähigkeit, während des Tages wach und aufmerksam zu bleiben aufgrund eines imperativen und nicht unterdrückbaren Bedürfnisses zu schlafen, sowie unvermeidbare Perioden von Schläfrigkeit und ungewolltem Einschlafen. Dabei ist bei Gesunden, aber noch viel stärker bei Pat. mit Schlafstörungen und auch bei Pat. mit PK eine Diskrepanz zwischen dem subjektiven Erleben und objektiv gemessenen Schlafparametern bekannt, die dazu führen kann, dass Schlaf nicht als solcher wahrgenommen wird, umgekehrt kann auch Tagesschläfrigkeit unter Umständen nicht registriert werden. Eine solche Diskrepanz zwischen objektiven Messungen und subjektivem Erleben kommt insbesondere bei der chronischen Insomnie vor, wobei hier objektiv messbare Schlafepisoden nicht als solche erkannt werden und deshalb die Schlafdauer als subjektiv zu kurz eingeschätzt wird. Diese Form der Diskrepanz zwischen objektiven und subjektiven Messungen wird hierbei als Schlafdiskrepanz bzw. Schlaf-Wach-Zustandsdiskrepanz bezeichnet [79]. Aufgrund der abweichenden Beurteilungen von Schlaf und dessen Auswirkungen ist eine objektive Diagnostik von Tagesschläfrigkeit, insbesondere bei der Beurteilung von

Fahrtauglichkeit, aber auch zur Abschätzung von Therapieeffekten zumindest bei bestimmten Fragestellungen sinnvoll. Parkinson-Pat. berichten dabei über eine erhöhte Tagesschläfrigkeit und ungewollten Einschlafattacken auch beim Fahren [80].

Evidenzgrundlage

Nach der Literaturrecherche zu „Parkinson's disease" and „excessive daytime sleepiness" wurden Metaanalysen (Evidenzklasse 1) sowie crosssektionale Kohortenstudien berücksichtigt.

Ergebnis

Als objektive Verfahren zur Messung der Tagesschläfrigkeit stehen verschiedene, allerdings in der Durchführung recht aufwendige Tests zur Verfügung. Als Goldstandard gelten dabei der Multiple Schlaflatenztest (MSLT) sowie der Multiple Wachbleibetest (MWT), die in der Durchführung jedoch an ein Schlaflabor gebunden sind. Dabei ist der MWT insbesondere dafür gedacht, die Fähigkeit bzw. Unfähigkeit, wach zu bleiben, zu messen [81], während der MSLT die Latenz misst, die die Pat. benötigen, um in ruhiger, abgedunkelter Umgebung einzuschlafen [82], [83]. Somit misst der MSLT die Fähigkeit, in monotonen Situationen einzuschlafen, während der MWT die Fähigkeit misst, trotz einer monotonen Situation wach zu bleiben. Da diese Konzepte sehr unterschiedlich sind, ist die Übereinstimmung zwischen beiden Tests gering. Bezüglich der Messung von Veränderungen der Tagesschläfrigkeit zeigte der MSLT eine Veränderung unter verschiedenen Therapien an [83].

Bei den Fragebögen misst die Epworth Sleepiness Scale (ESS) die subjektive Tagesschläfrigkeit, allerdings zeigen sich sehr widersprüchliche Ergebnisse zur Übereinstimmung der subjektiven und objektiven Tagesschläfrigkeit bei OSA-Pat. Während eine chinesische Studie in einer cross-sektionalen Kohorte an 100 Pat. mit obstruktiver Schlafapnoe (OSA) eine moderate Korrelation zwischen ESS und MSLT zeigte und die ROC-Analyse für den ESS zur Detektion von Tagesschläfrigkeit eine AUC von 0,8 erbrachte ([84], bester Cut-off > 12 Punkte), war in anderen Studien a**n** OSA-Pat. eine nur geringe Korrelation zwischen ESS- und MSLT-Tests nachweisbar, d.h., subjektive und objektive Schläfrigkeit unterschieden sich deutlich [85], [86]. In einer crosssektionalen Kohortenstudie kamen die Autoren bei der Untersuchung von 290 OSA-Pat. Zu dem Ergebnis, dass der MSLT die objektive Tagesschläfrigkeit besser darstellte als der ESS ([87], Evidenzklasse 1). Hingegen zeigte sich in einer anderen Studie, die sowohl den MSLT als auch den MWT sowie zwei verschiedenen Fragebögen zur Tagesschläfrigkeit benutzte (ESS sowie Karolinska Sleepiness Scale (KSS)) eine in Abhängigkeit vom Zeitpunkt der Testung variierende Übereinstimmung der Fragebögen mit den objektiven Messmethoden des MSLT und MWT [77].

Bezüglich der Fahrtauglichkeit zeigte sich allerdings eine gute Übereinstimmung zwischen MWT und Fahrsimulationstest bei Pat. mit OSA [88]. Allerdings gibt es nicht genug Evidenz, dass der MWT wirklich eine gute Assoziation zu Unfällen in der realen Umwelt hat, sodass keine generelle Evaluation der Fahrtauglichkeit mittels MWT empfohlen werden kann [89]. Bei der Fahrtauglichkeitstestung in OSA-Pat. zeigte sich **keine** Assoziation zwischen dem ESS, dem MSLT und dem Fahrsimulatortest in einer kleinen crosssektionalen Studie. Allerdings waren die Leistungen im TAP-Test und Psychomotor Vigilance Test Reaction Time (PVT RT) mit dem Fahrsimulatortest assoziiert [90].

Bezüglich der Fahrtauglichkeit zeigte sich bei PK in Fahrsimulationstests eine höhere Wahrscheinlichkeit für Unfälle als bei Kontrollen [91]. Auch der MSLT ist zur Detektion von Tagesschläfrigkeit nicht generell validiert und indiziert, um Tagesschläfrigkeit bei anderen Erkrankungen als der Narkolepsie zu messen, wobei er bei der PK zumindest bereits in verschiedenen Studien evaluiert wurde. Hingegen ist der MWT bei der PK nicht ausreichend validiert. Leider zeigen verschiedene Studien, dass weder MSLT noch MWT geeignet sind, gut zwischen gesunden Probanden/Probandinnen und Pat. mit Schlafstörungen zu unterschieden, was auch an einer großen Standardabweichung und an Boden- sowie Deckeneffekten liegt.

Allerdings zeigen Studien, dass bei der PK auch die subjektive Wahrnehmung von Tagesschläfrigkeit deutlich von der objektiv gemessenen Wahrscheinlichkeit einzuschlafen abweichen kann [77]. Insbesondere vor dem Beginn der Tagschlaftests zeigten ein Viertel der Pat. keinerlei subjektive Wahrnehmung ihrer anschließend in den Tagschlaftests nachweisbaren Tagesschläfrigkeit, sodass sich aus dieser Studie folgern lässt, dass bei der Frage nach Fahrtauglichkeit oder einer z.B. unfallrelevanten Tagesschläfrigkeit bei der Arbeitstätigkeit eine rein subjektive Evaluation nicht erfolgen sollte, da damit keine ausreichende Sicherheit gegeben ist. Allerdings zeigte sich in verschiedenen Studien, dass die Nachtschlafqualität nicht ausreichend mit subjektiven und objektiven Messungen der Tagesschläfrigkeit korreliert [92], [78], sodass sich hier die Frage stellt, welche weiteren Mechanismen für die **Tagesschläfrigkeit** verantwortlich sind. In anderen Studien zeigte sich zudem eine deutlich häufigere subjektive Tagesschläfrigkeit als anhand des MSLT nachweisbar, sodass sich auch hier für die Zukunft die Frage nach anderen Einflussfaktoren und besseren Messmethoden zur Detektion und Beschreibung von Tagesschläfrigkeit stellt. Eine kürzlich publizierte Studie zeigte einen Zusammenhang zwischen monoaminerger (dopaminerger/serotonerger) Innervation des Thalamus und subjektiver Tagesschläfrigkeit [93]. Allerdings zeigte sich in weiteren Studien bei Vorliegen einer subjektiven Tagesschläfrigkeit keine Assoziation zu den bisher bekannten Risikofaktoren wie beispielsweise dopaminergen Therapien oder Krankheitsschwere [94]. Letztlich sollten Methoden zur Detektion von **Tagesschläfrigkeit** und weiteren Untersuchung der zugrunde liegenden Mechanismen weiterentwickelt werden.

Begründung der Empfehlung

Aktuell existiert kein allgemeingültiger Standard zur objektiven Diagnostik von Tagesschläfrigkeit bei der PK. Deshalb sollten eine individuelle Ursachensuche sowie Risikoabwägung erfolgen und entsprechend den mutmaßlichen Ursachen sowie dem Ausmaß der Tagesschläfrigkeit eine weitere Diagnostik und Therapie eingeleitet werden.

**Empfehlung**	**Neu**<br>**Stand (2023)**
Bei der Diagnostik von **Tagesschläfrigkeit** sollten eine umfassende Eigenanamnese, Fremdanamnese, Fragebögen wie der ESS sowie Evaluation möglicher Gründe für Tagesschläfrigkeit ggf. einschließlich Messung der Nachtschlafqualität mittels vPSG und ggf. MSLT oder weitere neuropsychologische Testverfahren wie PVT und/oder Fahrsimulationstests erwogen werden.	
Konsensstärke: 92,6%, Konsens	

Fragestellung 109: Wie wird exzessive Tagesschläfrigkeit bei der PK therapiert?

Hintergrund

Exzessive Tagesschläfrigkeit ist ein häufiges Symptom bei der PK, welches subjektiv von 40 bis 90% der Pat. im Laufe der Erkrankung angegeben wird und das relevante Auswirkungen auf die Aktivitäten des täglichen Lebens sowie die Lebensqualität der Pat. und der Angehörigen hat [95], [96].

Evidenzgrundlage

Eingegebene Suchbegriffe umfassen „Parkinson’s disease“, „treatment sleep disorders“, „treatment excessive daytime sleepiness“.

Ergebnis

Die Therapie der **Tagesschläfrigkeit** sollte, angepasst an die möglichen Ursachen, differenziert erfolgen:

Pat. mit **Tagesschläfrigkeit** sollten zunächst im Hinblick auf nächtliche motorische Komplikationen wie Fluktuationen und „wearing-off“ befragt werden. Bei Hinweisen auf solche nächtlichen motorischen Komplikationen sollten entsprechende Veränderungen der Medikation erwogen werden. Diese beinhalten, wie bereits erwähnt, insbesondere die Umstellung auf langwirksame bzw. Retard-Formulierungen von Dopaminagonisten bzw. Levodopa. Dabei sollte jedoch auch die Gesamtdosis der Medikation bedacht und ggf. eine Adjustierung der Dosis z.B. mit Dosisreduktion oder Umverteilungen der Medikation erwogen werden, da auch ungünstiges Timing zu Schlafstörungen und **Tagesschläfrigkeit** führen kann [97]. Auch kann eine Umstellung der Therapie auf beispielsweise Amantadin erwogen werden [6]. Falls auch sonst motorische Komplikationen im Vordergrund stehen, können auch invasive Therapieoptionen wie Pumpentherapien oder THS erwogen werden, da auch hier eine Verbesserung der nächtlichen Beweglichkeit und somit des Schlafs erreicht werden kann. Eine kürzlich erschienene Metaanalyse zeigte bei der Auswertung von Daten von >1200 Pat. über eine Laufzeit von 24 Monaten in einem offenen Studiendesign eine Verbesserung des Schlafs anhand subjektiver Fragebögen-Scores [98].

Wenn die **Tagesschläfrigkeit** auch nach Optimierung der dopaminergen Therapie persistieren sollte und Hinweise auf behandlungsbedürftige Schlafstörungen wie beispielsweise eine relevante Insomnie vorliegen oder sich Hinweise auf therapierelevante schlafbezogene Atmungsstörungen ergeben, sollte eine weitere Diagnostik, u.a. mittels Polygraphie oder vPSG, erwogen werden. Anschließend sollte entsprechend der o.g. Ansätze die Insomnie bzw. die sonstigen primären Schlafstörungen behandelt werden (s.o.).

Begleitend kann unabhängig vom Vorliegen einer primären Schlafstörung oder einer auf die medikamentöse Therapie der PK zurückführbare **Tagesschläfrigkeit** eine nicht pharmakologische Therapie erfolgen. Diese umfasst zunächst eine Optimierung der Schlafhygiene mit regelmäßigen Schlafzeiten und Naps. Zudem sollten Strategien zur Erhöhung sozialer Aktivitäten und körperlicher Bewegung eingesetzt werden. Dabei kann auch ein Versuch mit Lichttherapie, insbesondere zur Verlagerung der Schlafphasen nach hinten bei verfrühten Bettzeiten und zur Steigerung der

Aktivitäten, zum Einsatz kommen [57]. Bezüglich des Einsatzes von Sporttherapie/Bewegung wurde in einer kürzlich erschienenen Metaanalyse insbesondere ein signifikant positiver Einfluss von Krafttraining auf Schlaf und Kognition nachgewiesen [99]. In einer anderen Metaanalyse zeigten sich ebenfalls positive Auswirkungen von Sport v.a. auf subjektive Schlafqualität, dabei wurden jedoch insbesondere bei starker sportlicher Anstrengung, weniger bei milder bis moderater Intensität Effekte auf den Schlaf beobachtet [59].

Nach Ausschluss sonstiger behandlungsbedürftiger Ursachen von Tagesschläfrigkeit und insbesondere zur Ergänzung der nicht pharmakologischen Therapien können pharmakologische Ansätze erwogen werden, wobei die Evidenz für medikamentöse Therapieansätze begrenzt ist.

Koffein in Höchstdosierungen bis 400 mg/d zeigten geringe Effekte auf die ESS (1,7 Punkte nach 6 Monaten), was als eher nicht klinisch relevant eingeordnet werden kann [100]. Modafinil ist für die Therapie von Tagesschläfrigkeit bei der Narkolepsie zugelassen und wurde in 3 Studien bei der PK untersucht, wobei sich hier unterschiedliche Resultate zeigten. Hingegen konnte in einer Metaanalayse eine Reduktion der subjektiven **Tagesschläfrigkeit** anhand des ESS nachgewiesen werden, wobei objektive Messungen keine Effekte zeigten [101]. Für andere Substanzen wie beispielsweise das von der FDA zugelassene Istradefyllin sind keine ausreichenden Daten vorhanden [102]. Auch für Methylphenidat [103] zeigten sich zwar in einer offenen Studien Effekte, aber aufgrund der nicht ausreichenden Evidenz kann keine Empfehlung ausgesprochen werden. Bei der Gammahydroxybuttersäure zeigte sich in einer randomisierten doppelblinden IIa-Studie ein Effekt der Medikation mit Nachweis einer Verbesserung auch objektiver PSG-basierter Messwerte. Allerdings traten unter GHB neue Fälle von SBAS und Parasomnien auf und in anderen Populationen kam es zu deutlichen Nebenwirkungen bis hin zu Todesfällen bei unerkannter SBAS, sodass hier weitere Studien notwendig sind und keine Empfehlung ausgesprochen werden kann [103]. Der selektive Noradrenalin-Wiederaufnahme-Hemmer Axonometin wird beim ADHS eingesetzt und zeigte in einer kleinen doppelblinden, randomisierten Studie bei der PK eine Verbesserung von Tagesschläfrigkeit und Kognition [104]. Allerdings werden auch hier weitere Studienergebnisse benötigt, um die Effekte zu bestätigen.

Die MDS empfiehlt zudem ein Fahrverbot bis zur Besserung der Symptome [97]. Gleichzeitig sollten Komorbiditäten und Begleitmedikationen anderer Erkrankungen, wie beispielsweise sedierende Antidepressiva oder Hypnotika, kontrolliert und ggf. reduziert oder abgesetzt werden.

Begründung der Empfehlung

Große randomisierte Studien zur Therapie der Tagesschläfrigkeit fehlen bisher. Basierend auf den verfügbaren kleineren Fall-Kontroll-Studien und den wenigen kleinen randomisierten Studien, können medikamentöse Therapien der **Tagesschläfrigkeit** nur begrenzt empfohlen werden. Nicht medikamentöse Therapieoptionen können und sollten angewandt werden, da die Nutzen-Risiko-Abwägung günstig ist.

Empfehlung	Modifiziert<br>Stand (2023)
▪ Zur Therapie der Tagesschläfrigkeit bei der PK sollte zunächst eine ursächliche Diagnostik erfolgen, um die **Tagesschläfrigkeit** differenziert therapieren zu können.<br>▪ Bei Hinweisen auf durch motorische oder nicht motorische Komplikationen der PK bedingte TSS sollte zunächst eine Optimierung der Therapie der PK erfolgen.<br>▪ Nicht medikamentöse Therapieverfahren der **Tagesschläfrigkeit** sollen angewandt werden, diese beinhalten z.B. die Einhaltung von Schlafhygiene, Bewegung sowie Lichttherapie.<br>▪ Zugrunde liegende, für eine **Tagesschläfrigkeit** ursächliche Schlafstörungen sollten diagnostiziert und behandelt werden, erst nach Ausschluss einer relevanten primären Schlafstörung sowie einer durch die PK direkt bedingten **Tagesschläfrigkeit** sollten medikamentöse Therapien der **Tagesschläfrigkeit** wie beispielsweise Modafinil erwogen werden.<br>▪ Die Evidenz für pharmakologische Therapien der Tagesschläfrigkeit ist generell unzureichend, medikamentöse Therapien wie beispielsweise Modafinil oder Gammahydroxybuttersäure sollten nur auf individueller Basis nach Ausschluss von Kontraindikationen im Einzelfall erwogen werden.	
Konsensstärke: 100%, starker Konsens	

Fragestellung 110: Wie sind die Auswirkungen von Nachtschlafstörungen auf Pat. und Angehörige?

Hintergrund

Schlafstörungen bei der PK beeinträchtigen die Tagesbefindlichkeit und wirken sich auf das soziale Umfeld aus.

Evidenzgrundlage

Suchbegriffe in Pubmed umfassten „Parkinsons's disease", „quality of life" und „sleep disturbances". Eingeschlossen wurden Kohortenstudien sowie Metaanalysen.

Ergebnis

Schlafstörungen beeinträchtigen nach neueren Daten sowohl die Pat. als auch die Angehörigen bzw. die Pflegepersonen [105]. Dabei zeigte sich in einer crosssektionalen Kohortenstudie, dass die Pat. insbesondere unter insomnischen Beschwerden litten, welche signifikant zur empfundenen Krankheitslast beitrugen [106] In kleineren crosssektionalen Studien zeigte sich ein klarer Zusammenhang zwischen Schlafstörungen und Lebensqualität bei Parkinson-Pat. [2], [107], [108]. In einer crosssektionalen Fragebogenstudie an Pat. und Angehörigen zeigten sich Assoziationen zwischen Schlafstörungen und depressiver Verstimmung bei Pat. und Partner/Partnerinnen. Zusätzlich beklagten die Angehörigen einen gestörten Nachtschlaf durch mehrfaches Erwachen aufgrund von Bedürfnissen oder Problemen des an Parkinson erkrankten Partners/der an Parkinson erkrankten Partnerin [109], [110]. In einer weiteren crosssektionalen Studie zu Schlafstörungen bei PK mit auch polysomnographischen Untersuchungen zeigte sich ein Zusammenhang zwischen Lebensqualität und Insomnie, aber nicht mit PSG-bestätigter obstruktiver Schlafapnoe [111].

Zusätzlich zur Lebensqualität lassen sich auch Auswirkungen von Schlafstörungen auf andere Lebensbereiche der Pat. nachweisen. So fanden sich in kleineren crosssektionalen Kohortenstudien bei polysomnographisch bestätigter bereits sehr leichtgradiger SBAS kognitive Beeinträchtigungen [112], die ebenfalls in Metaanalysen bestätigt werden konnten [113]. Auch fand sich in verschiedenen Studien ein direkter Zusammenhang mit Stimmungsschwankungen, v.a. depressiven Symptomen [109]. Zusätzlich wurden jedoch auch Assoziationen zwischen Schlafstörungen und Verschlechterung der Motorik beschrieben [114], [115], wohingegen eine Verbesserung der nächtlichen Motorik auch mit besserem Schlaf einherging [116].

Begründung der Empfehlung

Störungen des Nachtschlafs haben eine hohe Relevanz für Parkinson-Pat. und ihre Angehörigen.

Empfehlung	Neu<br>Stand (2023)
▪ Schlafstörungen haben ausgeprägte negative Implikationen für Pat. und deren Angehörige, welche sich u.a. negativ auf Motorik, Lebensqualität und Stimmung der Pat. sowie auf Stimmung und Nachtschlafqualität der Angehörigen auswirken, sodass Schlafstörungen in der klinischen Routinediagnostik und Therapie berücksichtigt werden sollen.<br>▪ Therapieentscheidungen sollten dabei nicht nur die Belange der Pat., sondern auch die Auswirkungen auf die Angehörigen berücksichtigen.	
Konsensstärke: 96,8%, starker Konsens	

Fragestellung 111: Was sind die Auswirkungen von Tagesschläfrigkeit auf Pat. und Angehörige?

Hintergrund

Erhöhte Tagesschläfrigkeit und ungewolltes Einschlafen am Tag schränken die Fähigkeit zur Teilhabe signifikant ein.

Evidenzgrundlage

Suchbegriffe in pub med umfassten „Parkinsons's disease“, „quality of life“ und „excessive daytime sleepiness“. Eingeschlossen wurden Kohortenstudien und Übersichtsarbeiten.

Ergebnis

Erhöhte Tageschläfrigkeit und ungewolltes Einschlafen schränkent die Fahrtauglichkeit und damit die eigenständige Mobilität der Betroffenen signifikant ein. Auch darüber hinaus führt imperatives Einschlafen in Alltagssituationen zu einer Gefährdung und impliziert einen signifikant negativen Einfluss auf die Lebensqualität [117], [70], [4]. In einer crosssektionalen Kohortenstudie wurde insbesondere der negative Einfluss der Tagesschläfrigkeit auf die Lebensqualität untersucht und ein Zusammenhang festgestellt [95]. Eine longitudinale Kohortenstudie konnte zeigen, dass Tagesschläfrigkeit mit der Krankheitsdauer zunimmt [117]. Wenngleich hierzu keine expliziten Untersuchungen gefunden wurden, idoch absolut nachvollziehbar, dass das ungewollte Einschlafen bzw. die reduzierte Attenz und Teilhabefähigkeit der Betroffenen, die mit erhöhter Tagesschläfrigkeit

einhergehen, Kommunikation und Aktivitäten mit dem sozialen Umfeld wesentlich einschränken und somit bidirektionalen Einfluss nehmen.

Begründung der Empfehlung

Erhöhte Tagesschläfrigkeit und ungewolltes Einschlafen führen zu vielfältigen Einschränkungen im Alltagsleben der Betroffenen wie auch ihrer Angehörigen.

**Empfehlung**	**Neu**<br>**Stand (2023)**
▪ Pat. mit erhöhter Tageschläfrigkeit sind nicht fahrtauglich und darüber hinaus in vielen Aspekten der Kommunikation und sozialen Teilhabe eingeschränkt.<br>▪ Diese Einschränkungen haben Auswirkungen auf das soziale Umfeld.	
Konsensstärke: 92,6%, Konsens	

Fragestellung 112: Wie ist die Prognose von Parkinson-Pat. mit Schlafstörungen im Vergleich zu Pat. ohne Schlafstörungen? Welche Schlafstörungen beeinflussen die Prognose von Parkinson-Pat. im Besonderen?

Hintergrund

Aufgrund der restaurativen Funktion des Schlafs implizieren Schlafstörungen eine Beschleunigung des der PK zugrunde liegenden neurodegenerativen Prozesses.

Evidenzgrundlage

Berücksichtigt wurden Kohortenstudien und Übersichtsarbeiten.

Ergebnis

Abgesehen von den eingangs erwähnten pathophysiologischen Zusammenhängen zwischen Schlaf und Neurodegeneration, gibt es auch klinische Hinweise, dass Schlafstörungen mit einer verstärkten Neurodegeneration bzw. rascheren Progression assoziiert sind, sodass Schlafstörungen auch einen prognostisch negativen Einfluss auf den Verlauf der PK zu haben scheinen [118], [7], [31], [32]. Insbesondere die REM-Schlaf-Verhaltensstörung (RBD) ist mit einem ungünstigeren Krankheitsverlauf assoziiert: Parkinson-Pat. mit einer RBD waren eher von einem rascher fortschreitenden Verlauf mit früherem Auftreten von motorischen Komplikationen und kognitiven Störungen betroffen als Pat. ohne RBD, sodass die RBD als Progressionsmarker aufgefasst werden kann [119], [120].

Auch das Vorliegen einer schlafbezogenen Atmungsstörung (SBAS) ist in der Allgemeinbevölkerung mit einem erhöhten Risiko metabolischer Komplikationen und kardiovaskulärer Ereignisse vergesellschaftet. Bei der PK zeigen sich bei Pat. mit SBAS kognitive Einschränkungen, v.a. eine Beeinträchtigung der Exekutivfunktionen und des Arbeitsgedächtnisses. Hingegen sind negative Auswirkungen auf die Motorik bisher noch umstritten. Allerdings zeigte eine kürzlich erschienene Metaanalyse einen Zusammenhang zwischen obstruktiver Schlafapnoe sowie kognitiven und motorischen Einschränkungen [16]. Eine rezente Metaanalyse von 19 Studien bestätigte einen

Zusammenhang zwischen OSA und schwergradigeren kognitiven und motorischen Beeinträchtigungen bei PK. Dabei zeigte sich auch, dass das Vorliegen einer RBD sowie Veränderungen der EEG-Mikrostruktur wie Abnahme des Deltaanteils, aber auch der Schlafspindeln, mit rascheren kognitiven Einbußen assoziiert waren [121].

Begründung der Empfehlung

Klinische Studien und Erkenntnisse der pathophysiologischen Zusammenhänge zeigen den negativen Einfluss von Schlafstörungen auf den Verlauf der PK. Dabei ist insbesondere das Vorliegen einer REM-Schlaf-Verhaltensstörung mit einer schlechteren Prognose und einem rascheren Krankheitsfortschreiten assoziiert.

Empfehlung	Neu<br>Stand (2023)
▪ Schlafstörungen sind mit einer rascheren Krankheitsprogression assoziiert.<br>▪ PK mit RBD im Frühstadium signalisiert eine Erkrankung mit progredienten kognitiven und autonomen Störungen sowie motorischen Komplikationen.	
Konsensstärke: 96,2%, starker Konsens	

Referenzen

1. Barone P, Antonini A, Colosimo C, Marconi R, Morgante L, Avarello TP, et al. The PRIAMO study: A multicenter assessment of nonmotor symptoms and their impact on quality of life in Parkinson's disease. Mov Disord. 2009;24(11):1641-9
2. Avidan A, Hays RD, Diaz N, Bordelon Y, Thompson AW, Vassar SD, Vickrey BG. Associations of sleep disturbance symptoms with health-related quality of life in Parkinson's disease. J Neuropsychiatry Clin Neurosci. 2013;25(4):319-26
3. Bartolomei L, Pastore A, Meligrana L, Sanson E, Bonetto N, Minicuci GM, et al. Relevance of sleep quality on caregiver burden in Parkinson's disease. Neurol Sci. 2018;39(5):835-9
4. Chahine LM, Amara AW, Videnovic A. A systematic review of the literature on disorders of sleep and wakefulness in Parkinson's disease from 2005 to 2015. Sleep Med Rev. 2017;35:33-50
5. Stefani A, Högl B. Sleep in Parkinson's disease. Neuropsychopharmacology. 2020;45(1):121-8
6. Shen Y, Huang JY, Li J, Liu CF. Excessive Daytime Sleepiness in Parkinson's Disease: Clinical Implications and Management. Chin Med J (Engl). 2018;131(8):974-81
7. Zahed H, Zuzuarregui JRP, Gilron R, Denison T, Starr PA, Little S. The Neurophysiology of Sleep in Parkinson's Disease. Mov Disord. 2021;36(7):1526-42
8. Videnovic A. Management of sleep disorders in Parkinson's disease and multiple system atrophy. Mov Disord. 2017;32(5):659-68
9. Zhang Y, Ren R, Sanford LD, Yang L, Zhou J, Tan L, et al. Sleep in Parkinson's disease: A systematic review and meta-analysis of polysomnographic findings. Sleep Med Rev. 2020;51:101281
10. Mantovani S, Smith SS, Gordon R, O'Sullivan JD. An overview of sleep and circadian dysfunction in Parkinson's disease. J Sleep Res. 2018;27(3):e12673
11. Gjerstad MD, Tysnes OB, Larsen JP. Increased risk of leg motor restlessness but not RLS in early Parkinson disease. Neurology. 2011;77(22):1941-6

12. Bhalsing K, Suresh K, Muthane UB, Pal PK. Prevalence and profile of Restless Legs Syndrome in Parkinson's disease and other neurodegenerative disorders: a case-control study. Parkinsonism Relat Disord. 2013;19(4):426-30
13. Alonso-Navarro H, García-Martín E, Agúndez JAG, Jiménez-Jiménez FJ. Association between restless legs syndrome and other movement disorders. Neurology. 2019;92(20):948-64
14. Schütz L, Sixel-Döring F, Hermann W. Management of Sleep Disturbances in Parkinson's Disease. J Parkinsons Dis. 2022;12(7):2029-58
15. Szatmari S, Jr., Bereczki D, Fornadi K, Kalantar-Zadeh K, Kovesdy CP, Molnar MZ. Association of Restless Legs Syndrome With Incident Parkinson's Disease. Sleep. 2017;40(2)
16. Elfil M, Bahbah EI, Attia MM, Eldokmak M, Koo BB. Impact of Obstructive Sleep Apnea on Cognitive and Motor Functions in Parkinson's Disease. Mov Disord. 2021;36(3):570-80
17. Lees AJ, Blackburn NA, Campbell VL. The nighttime problems of Parkinson's disease. Clin Neuropharmacol. 1988;11(6):512-9
18. Sringean J, Anan C, Thanawattano C, Bhidayasiri R. Time for a strategy in night-time dopaminergic therapy? An objective sensor-based analysis of nocturnal hypokinesia and sleeping positions in Parkinson's disease. J Neurol Sci. 2017;373:244-8
19. Zhu K, van Hilten JJ, Marinus J. The course of insomnia in Parkinson's disease. Parkinsonism Relat Disord. 2016;33:51-7
20. Rutten S, Vriend C, van der Werf YD, Berendse HW, Weintraub D, van den Heuvel OA. The bidirectional longitudinal relationship between insomnia, depression and anxiety in patients with early-stage, medication-naïve Parkinson's disease. Parkinsonism Relat Disord. 2017;39:31-6
21. Lee AH, Weintraub D. Psychosis in Parkinson's disease without dementia: common and comorbid with other non-motor symptoms. Mov Disord. 2012;27(7):858-63
22. Bjørnarå KA, Dietrichs E, Toft M. Clinical features associated with sleep disturbances in Parkinson's disease. Clin Neurol Neurosurg. 2014;124:37-43
23. Brunner H, Wetter TC, Hogl B, Yassouridis A, Trenkwalder C, Friess E. Microstructure of the non-rapid eye movement sleep electroencephalogram in patients with newly diagnosed Parkinson's disease: effects of dopaminergic treatment. Mov Disord. 2002;17(5):928-33
24. Tholfsen LK, Larsen JP, Schulz J, Tysnes OB, Gjerstad MD. Changes in insomnia subtypes in early Parkinson disease. Neurology. 2017;88(4):352-8
25. Frucht SJ, Greene PE, Fahn S. Sleep episodes in Parkinson's disease: a wake-up call. Mov Disord. 2000;15(4):601-3
26. Trenkwalder C, Kies B, Rudzinska M, Fine J, Nikl J, Honczarenko K, et al. Rotigotine effects on early morning motor function and sleep in Parkinson's disease: a double-blind, randomized, placebo-controlled study (RECOVER). Mov Disord. 2011;26(1):90-9
27. Pahwa R, Stacy MA, Factor SA, Lyons KE, Stocchi F, Hersh BP, et al. Ropinirole 24-hour prolonged release: randomized, controlled study in advanced Parkinson disease. Neurology. 2007;68(14):1108-15
28. Berg D, Postuma RB, Adler CH, Bloem BR, Chan P, Dubois B, et al. MDS research criteria for prodromal Parkinson's disease. Mov Disord. 2015;30(12):1600-11
29. Pagano G, De Micco R, Yousaf T, Wilson H, Chandra A, Politis M. REM behavior disorder predicts motor progression and cognitive decline in Parkinson disease. Neurology. 2018;91(10):e894-e905
30. Priano L, Bigoni M, Albani G, Sellitti L, Giacomotti E, Picconi R, et al. Sleep microstructure in Parkinson's disease: cycling alternating pattern (CAP) as a sensitive marker of early NREM sleep instability. Sleep Med. 2019;61:57-62
31. Scott-Massey A, Boag MK, Magnier A, Bispo D, Khoo TK, Pountney DL. Glymphatic System Dysfunction and Sleep Disturbance May Contribute to the Pathogenesis and Progression of Parkinson's Disease. Int J Mol Sci. 2022;23(21)

32. Bohnen NI, Hu MTM. Sleep Disturbance as Potential Risk and Progression Factor for Parkinson's Disease. J Parkinsons Dis. 2019;9(3):603-14
33. Keir LHM, Breen DP. New awakenings: current understanding of sleep dysfunction and its treatment in Parkinson's disease. J Neurol. 2020;267(1):288-94
34. Videnovic A, Noble C, Reid KJ, Peng J, Turek FW, Marconi A, et al. Circadian melatonin rhythm and excessive daytime sleepiness in Parkinson disease. JAMA Neurol. 2014;71(4):463-9
35. Hood S, Amir S. Neurodegeneration and the Circadian Clock. Front Aging Neurosci. 2017;9:170
36. Högl B, Arnulf I, Comella C, Ferreira J, Iranzo A, Tilley B, et al. Scales to assess sleep impairment in Parkinson's disease: critique and recommendations. Mov Disord. 2010;25(16):2704-16
37. Chaudhuri KR, Pal S, DiMarco A, Whately-Smith C, Bridgman K, Mathew R, et al. The Parkinson's disease sleep scale: a new instrument for assessing sleep and nocturnal disability in Parkinson's disease. J Neurol Neurosurg Psychiatry. 2002;73(6):629-35
38. Trenkwalder C, Kohnen R, Högl B, Metta V, Sixel-Döring F, Frauscher B, et al. Parkinson's disease sleep scale--validation of the revised version PDSS-2. Mov Disord. 2011;26(4):644-52
39. Muntean ML, Benes H, Sixel-Döring F, Chaudhuri KR, Suzuki K, Hirata K, et al. Clinically relevant cut-off values for the Parkinson's Disease Sleep Scale-2 (PDSS-2): a validation study. Sleep Med. 2016;24:87-92
40. Buysse DJ, Reynolds CF, 3rd, Monk TH, Berman SR, Kupfer DJ. The Pittsburgh Sleep Quality Index: a new instrument for psychiatric practice and research. Psychiatry Res. 1989;28(2):193-213
41. Martínez-Martín P, Cubo-Delgado E, Aguilar-Barberà M, Bergareche A, Escalante S, Rojo A, et al. [A pilot study on a specific measure for sleep disorders in Parkinson's disease: SCOPA-Sleep]. Rev Neurol. 2006;43(10):577-83
42. Martinez-Martin P, Visser M, Rodriguez-Blazquez C, Marinus J, Chaudhuri KR, van Hilten JJ. SCOPA-sleep and PDSS: two scales for assessment of sleep disorder in Parkinson's disease. Mov Disord. 2008;23(12):1681-8
43. DGN. (Hrsg.) S2-Leitlinie Insomnie bei neurologischen Erkrankungen, Version 5.1, 02.03.2020, verfügbar unter: *https://register.awmf.org/de/leitlinien/detail/030-045*.
44. Pollmächer T, Wetter C, Bassetti CLA, Högl B, Randerath WH. Handbuch Schlafmedizin: Urban&Fischer Verlag; 2020.
45. Maglione JE, Liu L, Neikrug AB, Poon T, Natarajan L, Calderon J, et al. Actigraphy for the assessment of sleep measures in Parkinson's disease. Sleep. 2013;36(8):1209-17
46. Raschellà F, Scafa S, Puiatti A, Martin Moraud E, Ratti PL. Actigraphy Enables Home Screening of Rapid Eye Movement Behavior Disorder in Parkinson's Disease. Ann Neurol. 2023;93(2):317-29
47. DGN (Hrsg.) S2k-Leitlinie Restless-Legs-Syndrom (RLS), Version 5.2, 25.06.2022, verfügbar unter: *https://register.awmf.org/de/leitlinien/detail/030-081*.
48. Taximaimaiti R, Luo X, Wang XP. Pharmacological and Non-pharmacological Treatments of Sleep Disorders in Parkinson's Disease. Curr Neuropharmacol. 2021;19(12):2233-49
49. DGSM (Hrsg.) S3-Leitlinie Nicht erholsamer Schlaf/Schlafstörungen - Schlafbezogene Atmungsstörungen, Version 3.1, 23.12.2016, verfügbar unter: *https://register.awmf.org/de/leitlinien/detail/063-001*.
50. Neikrug AB, Liu L, Avanzino JA, Maglione JE, Natarajan L, Bradley L, et al. Continuous positive airway pressure improves sleep and daytime sleepiness in patients with Parkinson disease and sleep apnea. Sleep. 2014;37(1):177-85
51. Videnovic A, Ju YS, Arnulf I, Cochen-De Cock V, Högl B, Kunz D, et al. Clinical trials in REM sleep behavioural disorder: challenges and opportunities. J Neurol Neurosurg Psychiatry. 2020;91(7):740-9

52. Gilat M, Marshall NS, Testelmans D, Buyse B, Lewis SJG. A critical review of the pharmacological treatment of REM sleep behavior disorder in adults: time for more and larger randomized placebo-controlled trials. J Neurol. 2022;269(1):125-48
53. During EH, Hernandez B, Miglis MG, Sum-Ping O, Hekmat A, Cahuas A, et al. Sodium oxybate in treatment-resistant rapid-eye-movement sleep behavior disorder. Sleep. 2023;46(8)
54. Ahn JH, Kim M, Park S, Jang W, Park J, Oh E, et al. Prolonged-release melatonin in Parkinson's disease patients with a poor sleep quality: A randomized trial. Parkinsonism Relat Disord. 2020;75:50-4
55. Delgado-Lara DL, González-Enríquez GV, Torres-Mendoza BM, González-Usigli H, Cárdenas-Bedoya J, Macías-Islas MA, et al. Effect of melatonin administration on the PER1 and BMAL1 clock genes in patients with Parkinson's disease. Biomed Pharmacother. 2020;129:110485
56. Erratum. Mov Disord. 2019;34(5):765
57. Videnovic A, Klerman EB, Wang W, Marconi A, Kuhta T, Zee PC. Timed Light Therapy for Sleep and Daytime Sleepiness Associated With Parkinson Disease: A Randomized Clinical Trial. JAMA Neurol. 2017;74(4):411-8
58. Martino JK, Freelance CB, Willis GL. The effect of light exposure on insomnia and nocturnal movement in Parkinson's disease: an open label, retrospective, longitudinal study. Sleep Med. 2018;44:24-31
59. Cristini J, Weiss M, De Las Heras B, Medina-Rincón A, Dagher A, Postuma RB, et al. The effects of exercise on sleep quality in persons with Parkinson's disease: A systematic review with meta-analysis. Sleep Med Rev. 2021;55:101384
60. Karsten J, Hagenauw LA, Kamphuis J, Lancel M. Low doses of mirtazapine or quetiapine for transient insomnia: A randomised, double-blind, cross-over, placebo-controlled trial. J Psychopharmacol. 2017;31(3):327-37
61. Shulman R, Nakhla M, Daneman D. The Ongoing Transmutation of Type 1 Diabetes: Disparities in Care and Outcomes. Can J Diabetes. 2021;45(5):381-2
62. Park KW, Jo S, Lee SH, Hwang YS, Lee D, Ryu HS, Chung SJ. Therapeutic Effect of Levodopa/Carbidopa/Entacapone on Sleep Disturbance in Patients with Parkinson's Disease. J Mov Disord. 2020;13(3):205-12
63. Pierantozzi M, Placidi F, Liguori C, Albanese M, Imbriani P, Marciani MG, et al. Rotigotine may improve sleep architecture in Parkinson's disease: a double-blind, randomized, placebo-controlled polysomnographic study. Sleep Med. 2016;21:140-4
64. Xiang W, Sun YQ, Teoh HC. Comparison of nocturnal symptoms in advanced Parkinson's disease patients with sleep disturbances: pramipexole sustained release versus immediate release formulations. Drug Des Devel Ther. 2018;12:2017-24
65. Fernández-Pajarín G, Sesar Á, Ares B, Castro A. Evaluating the Efficacy of Nocturnal Continuous Subcutaneous Apomorphine Infusion in Sleep Disorders in Advanced Parkinson's Disease: The APO-NIGHT Study. J Parkinsons Dis. 2016;6(4):787-92
66. De Cock VC, Dodet P, Leu-Semenescu S, Aerts C, Castelnovo G, Abril B, et al. Safety and efficacy of subcutaneous night-time only apomorphine infusion to treat insomnia in patients with Parkinson's disease (APOMORPHEE): a multicentre, randomised, controlled, double-blind crossover study. Lancet Neurol. 2022;21(5):428-37
67. De Fabregues O, Ferré A, Romero O, Quintana M, Álvarez-Sabin J. Sleep Quality and Levodopa Intestinal Gel Infusion in Parkinson's Disease: A Pilot Study. Parkinsons Dis. 2018;2018:8691495
68. Standaert DG, Aldred J, Anca-Herschkovitsch M, Bourgeois P, Cubo E, Davis TL, et al. DUOGLOBE: One-Year Outcomes in a Real-World Study of Levodopa Carbidopa Intestinal Gel for Parkinson's Disease. Mov Disord Clin Pract. 2021;8(7):1061-74
69. Zuzuárregui JRP, Ostrem JL. The Impact of Deep Brain Stimulation on Sleep in Parkinson's Disease: An update. J Parkinsons Dis. 2020;10(2):393-404

70. Sobreira-Neto MA, Pena-Pereira MA, Sobreira EST, Chagas MHN, Fernandes RMF, Tumas V, Eckeli AL. High Frequency of Sleep Disorders in Parkinson's Disease and Its Relationship with Quality of Life. Eur Neurol. 2017;78(5-6):330-7
71. Gao J, Huang X, Park Y, Hollenbeck A, Blair A, Schatzkin A, Chen H. Daytime napping, nighttime sleeping, and Parkinson disease. Am J Epidemiol. 2011;173(9):1032-8
72. Simuni T, Caspell-Garcia C, Coffey C, Chahine LM, Lasch S, Oertel WH, et al. Correlates of excessive daytime sleepiness in de novo Parkinson's disease: A case control study. Mov Disord. 2015;30(10):1371-81
73. Piovezan RD, Jadczak AD, Tucker G, Visvanathan R. Daytime Sleepiness Predicts Mortality in Nursing Home Residents: Findings from the Frailty in Residential Aged Care Sector Over Time (FIRST) Study. J Am Med Dir Assoc. 2023
74. Liu H, Li J, Wang X, Huang J, Wang T, Lin Z, Xiong N. Excessive Daytime Sleepiness in Parkinson's Disease. Nat Sci Sleep. 2022;14:1589-609
75. Johns MW. A new method for measuring daytime sleepiness: the Epworth sleepiness scale. Sleep. 1991;14(6):540-5
76. Hagell P, Broman JE. Measurement properties and hierarchical item structure of the Epworth Sleepiness Scale in Parkinson's disease. J Sleep Res. 2007;16(1):102-9
77. Bargiotas P, Lachenmayer ML, Schreier DR, Mathis J, Bassetti CL. Sleepiness and sleepiness perception in patients with Parkinson's disease: a clinical and electrophysiological study. Sleep. 2019;42(4)
78. Liguori C, Mercuri NB, Albanese M, Olivola E, Stefani A, Pierantozzi M. Daytime sleepiness may be an independent symptom unrelated to sleep quality in Parkinson's disease. J Neurol. 2019;266(3):636-41
79. Bensen-Boakes DB, Lovato N, Meaklim H, Bei B, Scott H. "Sleep-wake state discrepancy": toward a common understanding and standardized nomenclature. Sleep. 2022;45(10)
80. Ghorayeb I, Loundou A, Auquier P, Dauvilliers Y, Bioulac B, Tison F. A nationwide survey of excessive daytime sleepiness in Parkinson's disease in France. Mov Disord. 2007;22(11):1567-72
81. Mitler MM, Gujavarty KS, Browman CP. Maintenance of wakefulness test: a polysomnographic technique for evaluation treatment efficacy in patients with excessive somnolence. Electroencephalogr Clin Neurophysiol. 1982;53(6):658-61
82. Carskadon MA, Dement WC, Mitler MM, Roth T, Westbrook PR, Keenan S. Guidelines for the multiple sleep latency test (MSLT): a standard measure of sleepiness. Sleep. 1986;9(4):519-24
83. Arand D, Bonnet M, Hurwitz T, Mitler M, Rosa R, Sangal RB. The clinical use of the MSLT and MWT. Sleep. 2005;28(1):123-44
84. Cai SJ, Chen R, Zhang YL, Xiong KP, Lian YX, Li J, et al. Correlation of Epworth Sleepiness Scale with multiple sleep latency test and its diagnostic accuracy in assessing excessive daytime sleepiness in patients with obstructive sleep apnea hypopnea syndrome. Chin Med J (Engl). 2013;126(17):3245-50
85. Benbadis SR, Mascha E, Perry MC, Wolgamuth BR, Smolley LA, Dinner DS. Association between the Epworth sleepiness scale and the multiple sleep latency test in a clinical population. Ann Intern Med. 1999;130(4 Pt 1):289-92
86. Ziemba AW, Kozlowski S, Nazar K, Brzezińska Z, Kaciuba-Uścilko H. The hyperglycemic effect of adrenaline infused after exhausting, prolonged physical exercise in dogs. Experientia. 1975;31(2):193-4
87. Fong SY, Ho CK, Wing YK. Comparing MSLT and ESS in the measurement of excessive daytime sleepiness in obstructive sleep apnoea syndrome. J Psychosom Res. 2005;58(1):55-60
88. Pizza F, Contardi S, Mondini S, Trentin L, Cirignotta F. Daytime sleepiness and driving performance in patients with obstructive sleep apnea: comparison of the MSLT, the MWT, and a simulated driving task. Sleep. 2009;32(3):382-91

89. Littner MR, Kushida C, Wise M, Davila DG, Morgenthaler T, Lee-Chiong T, et al. Practice parameters for clinical use of the multiple sleep latency test and the maintenance of wakefulness test. Sleep. 2005;28(1):113-21
90. Huang Y, Hennig S, Fietze I, Penzel T, Veauthier C. The Psychomotor Vigilance Test Compared to a Divided Attention Steering Simulation in Patients with Moderate or Severe Obstructive Sleep Apnea. Nat Sci Sleep. 2020;12:509-24
91. Frucht S, Rogers JD, Greene PE, Gordon MF, Fahn S. Falling asleep at the wheel: motor vehicle mishaps in persons taking pramipexole and ropinirole. Neurology. 1999;52(9):1908-10
92. Stauder M, Klerman EB, Wang W, Erickson H, Videnovic A. Relationships of self-reported and objective measures of sleep, sleepiness, and sleep quality in Parkinson's disease. Parkinsonism Relat Disord. 2022;101:57-61
93. Yoo SW, Oh YS, Ryu DW, Lee KS, Lyoo CH, Kim JS. Low thalamic monoamine transporter availability is related to excessive daytime sleepiness in early Parkinson's disease. Neurol Sci. 2020;41(5):1081-7
94. Cochen De Cock V, Bayard S, Jaussent I, Charif M, Grini M, Langenier MC, et al. Daytime sleepiness in Parkinson's disease: a reappraisal. PLoS One. 2014;9(9):e107278
95. Yoo SW, Kim JS, Oh YS, Ryu DW, Lee KS. Excessive daytime sleepiness and its impact on quality of life in de novo Parkinson's disease. Neurol Sci. 2019;40(6):1151-6
96. Zhang L, Chen Y, Liang X, Wang L, Wang J, Tang Y, Zhu X. Prediction of Quality of Life in Patients With Parkinson's Disease With and Without Excessive Daytime Sleepiness: A Longitudinal Study. Front Aging Neurosci. 2022;14:846563
97. Ferreira JJ, Katzenschlager R, Bloem BR, Bonuccelli U, Burn D, Deuschl G, et al. Summary of the recommendations of the EFNS/MDS-ES review on therapeutic management of Parkinson's disease. Eur J Neurol. 2013;20(1):5-15
98. Chaudhuri KR, Antonini A, Pahwa R, Odin P, Titova N, Thakkar S, et al. Effects of Levodopa-Carbidopa Intestinal Gel on Dyskinesia and Non-Motor Symptoms Including Sleep: Results from a Meta-Analysis with 24-Month Follow-Up. J Parkinsons Dis. 2022;12(7):2071-83
99. Yang Y, Wang G, Zhang S, Wang H, Zhou W, Ren F, et al. Efficacy and evaluation of therapeutic exercises on adults with Parkinson's disease: a systematic review and network meta-analysis. BMC Geriatr. 2022;22(1):813
100. Postuma RB, Anang J, Pelletier A, Joseph L, Moscovich M, Grimes D, et al. Caffeine as symptomatic treatment for Parkinson disease (Café-PD): A randomized trial. Neurology. 2017;89(17):1795-803
101. Rodrigues TM, Castro Caldas A, Ferreira JJ. Pharmacological interventions for daytime sleepiness and sleep disorders in Parkinson's disease: Systematic review and meta-analysis. Parkinsonism Relat Disord. 2016;27:25-34
102. Suzuki K, Miyamoto M, Miyamoto T, Uchiyama T, Watanabe Y, Suzuki S, et al. Istradefylline improves daytime sleepiness in patients with Parkinson's disease: An open-label, 3-month study. J Neurol Sci. 2017;380:230-3
103. Devos D, Krystkowiak P, Clement F, Dujardin K, Cottencin O, Waucquier N, et al. Improvement of gait by chronic, high doses of methylphenidate in patients with advanced Parkinson's disease. J Neurol Neurosurg Psychiatry. 2007;78(5):470-5
104. Weintraub D, Mavandadi S, Mamikonyan E, Siderowf AD, Duda JE, Hurtig HI, et al. Atomoxetine for depression and other neuropsychiatric symptoms in Parkinson disease. Neurology. 2010;75(5):448-55
105. Lauretani F, Testa C, Salvi M, Zucchini I, Giallauria F, Maggio M. Clinical Evaluation of Sleep Disorders in Parkinson's Disease. Brain Sci. 2023;13(4)
106. Karlsen KH, Larsen JP, Tandberg E, Maeland JG. Influence of clinical and demographic variables on quality of life in patients with Parkinson's disease. J Neurol Neurosurg Psychiatry. 1999;66(4):431-5

107. Scaravilli T, Gasparoli E, Rinaldi F, Polesello G, Bracco F. Health-related quality of life and sleep disorders in Parkinson's disease. Neurol Sci. 2003;24(3):209-10
108. Yu RL, Tan CH, Wu RM. The impact of nocturnal disturbances on daily quality of life in patients with Parkinson's disease. Neuropsychiatr Dis Treat. 2015;11:2005-12
109. Smith MC, Ellgring H, Oertel WH. Sleep disturbances in Parkinson's disease patients and spouses. J Am Geriatr Soc. 1997;45(2):194-9
110. Wade R, Pachana NA, Dissanayaka N. Factors Related to Sleep Disturbances for Informal Carers of Individuals With PD and Dyadic Relationship: A Rural Perspective. J Geriatr Psychiatry Neurol. 2021;34(5):389-96
111. Shafazand S, Wallace DM, Arheart KL, Vargas S, Luca CC, Moore H, et al. Insomnia, Sleep Quality, and Quality of Life in Mild to Moderate Parkinson's Disease. Ann Am Thorac Soc. 2017;14(3):412-9
112. Hermann W, Schmitz-Peiffer H, Kasper E, Fauser M, Franke C, Wienecke M, et al. Sleep Disturbances and Sleep Disordered Breathing Impair Cognitive Performance in Parkinson's Disease. Front Neurosci. 2020;14:689
113. Pushpanathan ME, Loftus AM, Thomas MG, Gasson N, Bucks RS. The relationship between sleep and cognition in Parkinson's disease: A meta-analysis. Sleep Med Rev. 2016;26:21-32
114. Schreiner SJ, Imbach LL, Werth E, Poryazova R, Baumann-Vogel H, Valko PO, et al. Slow-wave sleep and motor progression in Parkinson disease. Ann Neurol. 2019;85(5):765-70
115. O'Dowd S, Galna B, Morris R, Lawson RA, McDonald C, Yarnall AJ, et al. Poor Sleep Quality and Progression of Gait Impairment in an Incident Parkinson's Disease Cohort. J Parkinsons Dis. 2017;7(3):465-70
116. Ray Chaudhuri K, Martinez-Martin P, Antonini A, Brown RG, Friedman JH, Onofrj M, et al. Rotigotine and specific non-motor symptoms of Parkinson's disease: post hoc analysis of RECOVER. Parkinsonism Relat Disord. 2013;19(7):660-5
117. Amara AW, Chahine LM, Caspell-Garcia C, Long JD, Coffey C, Högl B, et al. Longitudinal assessment of excessive daytime sleepiness in early Parkinson's disease. J Neurol Neurosurg Psychiatry. 2017;88(8):653-62
118. Sundaram S, Hughes RL, Peterson E, Müller-Oehring EM, Brontë-Stewart HM, Poston KL, et al. Establishing a framework for neuropathological correlates and glymphatic system functioning in Parkinson's disease. Neurosci Biobehav Rev. 2019;103:305-15
119. Postuma RB, Bertrand JA, Montplaisir J, Desjardins C, Vendette M, Rios Romenets S, et al. Rapid eye movement sleep behavior disorder and risk of dementia in Parkinson's disease: a prospective study. Mov Disord. 2012;27(6):720-6
120. Nomura T, Inoue Y, Kagimura T, Nakashima K. Clinical significance of REM sleep behavior disorder in Parkinson's disease. Sleep Med. 2013;14(2):131-5
121. Dijkstra F, de Volder I, Viaene M, Cras P, Crosiers D. Polysomnographic Predictors of Sleep, Motor, and Cognitive Dysfunction Progression in Parkinson's Disease. Curr Neurol Neurosci Rep. 2022;22(10):657-74

3.12 Kognition

Autoren/Autorinnen: Inga Liepelt-Scarfone, Elke Kalbe, Ann-Kristin Folkerts, Karsten Witt

Allgemeiner Hintergrund

Kognitive Störungen kommen in leichter Ausprägung im Mittel bei ca. 25–28% aller Personen mit PK vor [1], [2]. Bei bis zu einem Drittel (19–36%) der Personen mit PK ist eine leichte kognitive Störung (engl. Parkinson's Disease with mild cognitive impairment; PD-MCI) bereits zum Zeitpunkt der klinischen Diagnose nachweisbar [3]. Das Auftreten einer PD-MCI erhöht das Risiko zur Entwicklung einer PK mit Demenz (PKD) [4], [5]. Im Durchschnitt beträgt die Prävalenz der PKD circa 24–31% [6], [7]. Nach einer Krankheitsdauer von mehr als 12 bzw. 15 Jahren haben über die Hälfte aller Personen mit PK eine Demenz entwickelt [8], [9]. Eine frühe und valide Diagnose der PKD als Voraussetzung für Therapiemaßnahmen ist essenziell.

Neben dem Nachweis der kognitiven Leistungsminderung durch geeignete Testverfahren sind ein schleichender Beginn und progredienter Verlauf der kognitiven Störung im Rahmen des (langjährigen) Krankheitsverlaufs für die Diagnose einer PD-MCI bzw. PKD ausschlaggebend.

Kognitive Einschränkungen können exekutive Funktionen, Gedächtnis, Aufmerksamkeit und Arbeitsgedächtnis, visuokognitive Leistungen und die Sprache betreffen [2]. Bei PD-MCI führen kognitive Einschränkungen nicht zu dem Verlust der Selbstständigkeit in den Aktivitäten des täglichen Lebens (ADL), wobei leichte Einschränkungen bei komplexen Aufgaben auftreten können [2]. Bei PKD ist der Schweregrad der Einschränkungen in diesen kognitiven Domänen mit dem Verlust der Selbstständigkeit in den ADL assoziiert [10]. Darüber hinaus stützt das Auftreten von Verhaltensauffälligkeiten (z.B. Halluzinationen, Apathie) die Diagnose einer PKD [10].

Fragestellung 113: Welche Screeninginstrumente (Level-I-Diagnostik) sind geeignet für die Erfassung einer leichten kognitiven Störung bei PD-MCI im Vergleich zu Personen mit PK ohne kognitive Störungen?

Hintergrund

Im Durchschnitt entwickeln 31% aller Personen mit PD-MCI innerhalb von sieben Jahren eine Demenz [11]. Eine valide und reliable Testdiagnostik einer PD-MCI ermöglicht die Eingrenzung einer Risikogruppe für PKD und hat somit einen hohen Stellenwert bei der Früherkennung der Demenz [12].

Die Leitlinien zum diagnostischen Vorgehen einer Expertengruppe der Movement Disorder Society (MDS) unterscheiden zwischen dem Nachweis der kognitiven Leistungsminderung in einem für PK validierten Screeningverfahren (Level I) sowie in einer ausführlichen neuropsychologischen Testung (Level II) [2]. Für die Level-I-Diagnose ist die Anwendung reliabler und valider deutschsprachiger Screeningverfahren mit einer hohen diagnostischen Güte notwendig, um Einschränkungen in wesentlichen Leistungsbereichen zu detektieren.

Evidenzgrundlage

Die Literatursuche in Pubmed erbrachte 1.020 Studien, eine doppelte Nennung wurde ausgeschlossen. Für die Literaturrecherche wurden folgende Schlagworte verwendet: (Parkinson disease OR Parkinson* OR Parkinson disorders OR Movement Disorders NOT [Wolff parkinson white syndrome OR wolff* AND parkinson*] OR [wpw AND syndrome] OR wpw) AND (Screening OR Screening Instrument OR Screening Measure OR Screening tool OR Questionnaire OR Montreal Cognitive Assessment OR Addenbrooke's Cognitive Examination-Revised OR Parkinson Neuropsychometric Dementia Assessment OR Mini-Mental State Examination OR DemTect) AND (PD-MCI OR mild cognitive impairment OR mild cognitive impairment OR cognitive dysfunction OR MCI OR PD-NC OR PDNC OR PD-CN OR PDCN OR normal cognition OR (normal cognitive AND status OR function) AND (Meta-Analysis OR Systematic Review OR Reviews OR [original studies AND German)].

Nach dem Screening der Abstracts und der Inhalte wurde eine Literaturreferenz als relevant für die Leitlinie bewertet. Zusätzlich wurden 12 Studien aus anderen Quellen selektiert, welche ebenfalls in diese Leitlinie einbezogen wurden.

Es fanden sich:

- 1 longitudinale größere Kohortenstudie
- 8 Originalarbeiten zur Ermittlung der diagnostischen Güte des betreffenden Testverfahrens für eine PD-MCI, diagnostiziert nach MDS-Level II, internationale statistische Klassifikation der Krankheiten und verwandter Gesundheitsprobleme (ICD)-10, Diagnostic and Statistical Manual of Mental Disorders (DSM)-IV oder DSM-5-Kriterien (Goldstandard)
- 2 Übersichtsartikel mit Testempfehlungen von Expertengruppen

Darüber hinaus wurden 7 Artikel, welche über relevante Aspekte der Verfahren informieren, einbezogen.

Ergebnis

Aufgrund adäquater Testgütekriterien (Reliabilität, Validität und Sensitivität für Veränderung) und Berücksichtigung von Aufgaben, welche alle relevanten kognitiven Domänen testen, wurden von der Expertengruppe des MDS das Montreal Cognitive Assessment (MoCA), die Mattis-Demenz-Skala (MDRS) [2], und die Parkinson's Disease-Cognitive Rating Scale (PD-CRS) [13] für die Beurteilung des globalen kognitiven Leistungsniveaus bei PK empfohlen. Für die PD-CRS ist aktuell keine deutschsprachige Version erhältlich. Zur Beurteilung einer PD-MCI [2] ist ein MoCA-Cut-off-Wert unterhalb von 26 Punkten empfohlen (Sensitivität 90% und Spezifität von 75%) [14], jedoch werden alternative Testwerte zur Klassifikation von PD-MCI berichtet [13]. Die Klassifikation von PD-MCI, basierend auf dem MoCA, erhöht das Risiko einer PKD (0,5 Jahre bis 9 Jahre) [4]. Die diagnostische Güte der deutschen Version (Cut-off 25/26 Punkte, Sensitivität: 55.5% bis 70%; Spezifität: 65.4% bis 83.3%) des MoCA für die Level-II-Diagnose einer PD-MCI ist belegt [15], [16]. Für die deutsche Version des MoCA sind Normwerte verfügbar (Webkalkulator: http://www.mocatest.ch), welche für den Einfluss von Geschlecht, Alter und Bildung adjustieren [17]. Erste Hinweise deuten auf die

Überlegenheit einer PK-spezifischen Gewichtung der MoCA-Itemwerte für die Klassifikation von PD-MCI in deutschsprachigen Kohorten von Personen mit PK hin [15], [16]. Diese Befunde werden aktuell nicht durch Daten in internationalen Kohorten gestützt.

Ein Testwert der MDRS unterhalb von 140 Punkten (maximal 144 erreichbare Punkte; Sensitivität 77%, Spezifität 65%) kann zwischen Personen mit PK mit und ohne PD-MCI (Level-II-Diagnostik) in internationalen Kohorten differenzieren [18]. Die diagnostische Güte der deutschen Version der MDRS für PD-MCI ist aktuell noch ungeklärt. Nationale Studien belegen, dass die MDRS die kognitive Leistung von Personen mit PK und einer Kontrollgruppe von Personen ohne neurodegenerative Erkrankung gut differenzieren kann (Cohen's d: 0,99 ≤ d ≤ 1,01) [12]. Die Sensitivität der deutschen Version des MDRS zum Nachweis von kognitiven Änderungen durch die Tiefe Hirnstimulation ist im Vergleich zum MoCA geringer [19].

Zudem belegt eine internationale Studie die diagnostische Güte der Scales for Outcomes in Parkinson's disease – cognition (SCOPA-COG) für die Level-II-Klassifikation von PD-MCI bei einem Testwert unterhalb von 30 Punkten mit einer Sensitivität von 76% und einer Spezifität von 74% [14]. Im direkten Vergleich mit dem MoCA ist die diagnostische Güte des SCOPA-COG jedoch geringer [14]. Von der Expertengruppe der MDS wurde die Empfehlung mit Vorbehalt ausgesprochen [13]. Kritiken an der SCOPA-COG beziehen sich auf den noch fehlenden Nachweis spezifischer Reliabilitätswerte (z.B. Übereinstimmung von Beurteilenden), der Sensitivität für Veränderung bei PK sowie auf die starke Gewichtung von Aufgaben zum Gedächtnis [13].

Die diagnostische Güte des Mini-Mental-Status-Test (MMST) für PD-MCI ist in verschiedenen Studien ebenfalls belegt, jedoch ist der MMST hinsichtlich der Trennschärfe für PD-MCI dem MoCA unterlegen [14], [20], [13]. Eine validierte Version des MMST ist in der deutschen Adaptation des Consortium to Establish a Registry for Alzheimer's Disease (CERAD-Plus) enthalten [21]. Einflüsse des Bildungsgrads auf die Testleistung im MMST sind belegt [22]. Die Normierung des CERAD-Plus adjustiert für den Effekt des Geschlechts, des Alters und des Bildungsgrads (https://www.memoryclinic.ch/de/main-navigation/neuropsychologen/cerad-plus/). Weitere Kritik am MMST bezieht sich auf das Fehlen von exekutiven Aufgaben sowie eine mangelnde Veränderungssensitivität [13].

Die Addenbrooke's Cognitive Examination (ACE) inkludiert den MMST. Die Durchführungszeit für die 24 Aufgaben beträgt ca. 15 Minuten. Der diagnostische Trennwert in internationalen Studien der ACE für PD-MCI nach DSM-5 variiert in den verschiedenen Versionen der ACE in Abhängigkeit von dem Bildungsgrad [23]. Für die ACE-I (Version I der ACE) beträgt die Sensitivität zwischen 85% und 86% und die Spezifität zwischen 57% und 63% für die verschiedenen Cut-off-Werte (0–8 Bildungsjahre <84 Punkte, 9–12 Bildungsjahre <87 Punkte, >12 Bildungsjahre <91 Punkte) [23]. Für die ACE-III (Version III der ACE) beträgt die Sensitivität zwischen 77% und 93% und die Spezifität zwischen 64% und 79% für die verschiedenen Cut-off-Werte (0–8 Bildungsjahre <84 Punkte, 9–12 Bildungsjahre <86 Punkte, >12 Bildungsjahre <89 Punkte) [23]. Sensitivitätswerte für die Kurzversion der ACE (Mini-ACE) liegen zwischen 72% und 85%; die Spezifität liegt bei 51% bis 80% (0–8 Bildungsjahre <23 Punkte, 9-12 Bildungsjahre <25 Punkte, >12 Bildungsjahre <26 Punkte) [23]. Für die italienische Version der revidierten Version der ACE (ACE-R) wurde ein diagnostischer Testwert unterhalb 78 Punkten mit

einer Sensitivität von 59% und einer Spezifität von 81% für die Level-II-Diagnose einer PD-MCI ermittelt [24]. Die deutsche Version der ACE-I und ACE-R wurde bei Personen ohne PD-MCI und Demenz (z.B. Alzheimer-Erkrankung, frontotemporale Demenz, vaskuläre Demenz) validiert [25], [26]. Die diagnostische Güte der deutschen Version der ACE für eine MCI bei PK ist aktuell noch ungeklärt. Der Nachweis der Reliabilität und der Sensitivität für Veränderung bei PK steht noch aus.

Die Frontal Assessment Battery (FAB) ist ein Screeningverfahren zur Ermittlung des Schweregrads exekutiver Dysfunktion [27]. Eine Studie belegt bei Anwendung eines Cut-offs unterhalb von 17 Punkten eine hohe Sensitivität des Verfahrens (84%) für PD-MCI bei geringer Spezifität (45%) [28]. Eine deutsche Validierung der Skala (FAB-D) liegt vor [29].

Für das Parkinson Neuropsychometric Dementia Assessment (PANDA) liegen bisher keine Cut-off-Werte für den Vergleich von Personen mit PK mit und ohne PD-MCI vor. Angaben zum Cut-off (<17 Punkte, Sensitivität=77%, Spezifität=91%) beziehen sich auf eine Kohorte von Personen mit PD-MCI und PKD im Vergleich zu einer gesunden Kontrollgruppe [30]. Nach Einschätzung der Autorinnen/Autoren ist das PANDA vor allem zur Früherkennung (leichter) kognitiver Störungen geeignet. Aktuell sind spezifische Aspekte der Reliabilität sowie der Nachweis der Sensitivität für Veränderungen noch nicht nachgewiesen [13]. Es wurde weiterhin angemerkt, dass das PANDA keinen spezifischen Subtest zur Erfassung der Aufmerksamkeit enthält [13]. Allerdings fließt die Aufmerksamkeit als kognitive Basisfunktion in die Subtests ein.

Begründung der Empfehlung

Auf Basis der oben dargestellten Evidenzgrundlage werden Screeningverfahren zur Prüfung des globalen kognitiven Leistungsniveaus wie folgt empfohlen:

- Empfehlung: Nachweis der diagnostischen Trennschärfe für eine PD-MCI in internationalen Kohorten und für die deutsche Version der Skala, die Testgüte des Verfahrens bei PK ist hinreichend belegt (Reliabilität, Validität und Sensitivität für Veränderung) und das Verfahren detektiert alle wesentlichen mit einer PD-MCI assoziierten kognitiven Leistungsbereiche.
- Empfehlung mit Vorbehalt: Nachweis der diagnostischen Trennschärfe für eine PD-MCI in internationalen Kohorten oder für die deutsche Version der Skala, Nachweise darüber, dass kognitive Störungen bei PK in nationalen Kohorten mit dem Verfahren detektiert werden und die Testgüte des Verfahrens bei PK eingeschränkt belegt ist (Fehlen des Nachweises spezifischer Testgütekriterien bei PK) oder das Verfahren nicht alle wesentlichen mit einer PD-MCI assoziierten kognitiven Leistungsbereiche detektiert.
- Mögliche Testanwendung: Nachweis der diagnostischen Trennschärfe für eine PD-MCI in internationalen Kohorten oder für die deutsche Version der Skala, die Testgüte des Verfahrens bei PK ist nicht belegt, aber die Testgüte der deutschen Version des Verfahrens für andere Erkrankungen ist belegt.

Empfehlung	Neu<br>Stand (2023)
Für die Level-I-Diagnose einer PD-MCI sollten kognitive Screeninginstrumente verwendet werden. Hierbei sollten, wo immer verfügbar, Korrekturen für Alter, Bildung und ggf. Geschlecht zur Anwendung kommen.<br>Geeignet sind besonders (sollte-Empfehlungsgrad):<br>▪ MoCA mit einem Cut-off unter 26 Punkten<br>▪ MDRS mit einem Cut-off unter 140 Punkten<br>Weiterhin können folgende Instrumente verwendet werden (kann-Empfehlungsgrad):<br>▪ SCOPA-COG mit einem Cut-off unter 30 Punkten<br>▪ ACE/ ACE-R; Cut-off-Werte sind bildungsabhängig<br>▪ PANDA mit einem Cut-off unter 17 Punkten	
Konsensstärke: 100%, starker Konsens	

Fragestellung 114: Welche Screeninginstrumente (Level-I-Diagnostik) sind geeignet für die Erfassung kognitiver Defizite bei Personen mit PKD im Vergleich zu Personen mit PK ohne Demenz?

Hintergrund

Die Leitlinien zum diagnostischen Vorgehen einer Expertengruppe der MDS unterscheidet zwischen dem Nachweis der kognitiven Leistungsminderung in einem für die PK validierten Screeningverfahren (Level I) sowie einer ausführlichen neuropsychologischen Testung (Level II) [31]. Für die Level-I-Diagnose ist die Anwendung reliabler und valider deutschsprachiger Screeningverfahren mit einer hohen diagnostischen Güte notwendig, welche Einschränkungen in wesentlichen Leistungsbereichen detektieren.

Evidenzgrundlage

Die Literatursuche in Pubmed erbrachte 1.067 Studien, eine doppelte Nennung wurde ausgeschlossen. Für die Literaturrecherche wurden folgende Schlagworte verwendet: (Parkinson disease OR Parkinson* OR Parkinson disorders OR Movement Disorders NOT [Wolff parkinson white syndrome OR wolff* AND parkinson*] OR [wpw AND syndrome] OR wpw) AND (Screening OR Screening Instrument OR Screening Measure OR Screening tool OR Questionnaire OR Montreal Cognitive Assessment OR Addenbrooke’s Cognitive Examination-Revised OR Parkinson Neuropsychometric Dementia Assessment OR Mini-Mental State Examination OR DemTect) AND (PDD OR Parkinson disease with dementia OR Parkinson dementia OR cognitive dysfunction OR PD-MCI OR MCI OR PD-NC OR PDNC OR PD-CN OR PDCN OR non-demented OR Parkinson's disease without dementia OR [normal cognitive AND status] OR function OR controls OR control group OR PDnD) AND (Meta-Analysis OR Systematic Review OR Reviews OR [original studies AND German]).

Nach Screening der Abstracts und der Inhalte wurde 1 Literaturreferenz als relevant für die Leitlinie bewertet. Zusätzlich wurden 20 Studien aus anderen Quellen selektiert, welche ebenfalls in diese Leitlinie einbezogen wurden.

Es fanden sich:

- 13 Originalarbeiten zur Ermittlung der diagnostischen Güte für eine PKD, diagnostiziert nach MDS-Level II, ICD-10, DSM-IV oder DSM-5-Kriterien
- 2 Übersichtsartikel mit Testempfehlungen von Expertengruppen.

Darüber hinaus wurden 6 Artikel, welche über relevante Aspekte der Verfahren informieren, einbezogen.

Ergebnis

Aufgrund adäquater Testgütekriterien (Reliabilität, Validität und Sensitivität für Veränderung) und Berücksichtigung von Aufgaben, welche alle relevanten kognitiven Domänen testeten, wurden von der Expertengruppe der MDS das MoCA, die MDRS und die PD-CRS [13] für die Beurteilung des globalen kognitiven Leistungsniveaus bei der PK empfohlen. Für die PD-CRS ist aktuell keine deutschsprachige Version erhältlich.

Basierend auf den Ergebnissen verschiedener internationaler Studien [14], [32], [20], empfiehlt die Expertengruppe der MDS einen Cut-off-Wert unterhalb von 21 Punkten (Sensitivität 81% und Spezifität von 95%) im MoCA für die Diagnose einer PKD nach Level II oder DSM-5-Kriterien [13]. Die Testdauer des MoCA beträgt ca. 10 Minuten und ist somit kürzer als die Testdauer der MDRS (20–30 Minuten) oder der SCOPA-COG (15 Minuten) [13].

Eine internationale Studie belegt die Trennschärfe der MDRS mit einem Punktwert unterhalb von 123 (Sensitivität 93%, Spezifität 91%) für PKD, diagnostiziert nach DSM IV-TR [33]. Die diagnostische Güte der deutschen Version des MDRS für eine PKD ist aktuell noch ungeklärt, wobei die Skala in nationalen Studien kognitive Einschränkungen bei der PK detektieren kann [12].

Zusätzlich wurde für die Scales for Outcomes in Parkinson's disease – cognition (SCOPA-COG) in internationalen Studien für einen Wert unterhalb von 23 Punkten die höchste diagnostische Genauigkeit für die Level-II-Klassifikation einer PKD ermittelt (Sensitivität 80–85%, Spezifität 87–96%) [14], [34]. Im direkten Vergleich mit dem MoCA ist die Trennschärfe der SCOPA-COG für PKD vergleichbar hoch [14]. Von der Expertengruppe der MDS wurde die Empfehlung zur Verwendung der SCOPA-COG mit Vorbehalt ausgesprochen [13]. Kritiken an der SCOPA-COG beziehen sich auf den noch fehlenden Nachweis spezifischer Reliabilitätswerte (z.B. Übereinstimmung von Beurteilenden), der Sensitivität für Veränderung bei der PK sowie der starken Gewichtung von Aufgaben zum Gedächtnis [13].

Die Anwendung des MMST zur Level-I-Diagnose einer PKD ist von Experten/Expertinnen der MDS empfohlen [31]. In nationalen Kohorten wurden für den MMST starke Effekte für die Diskrimination zwischen Personen mit PKD und nicht dementen Parkinson-Pat. nachgewiesen [12]. Eine validierte Version des MMST ist in der deutschen Adaptation des CERAD-Plus enthalten [21]. Von einer Expertengruppe wurde ein Wert unterhalb von 26 Punkten für die Klassifikation einer PKD

empfohlen [31]. Internationale Studien belegen eine hohe Spezifität dieses Cut-offs (100%), jedoch ist die Sensitivität des MMST (47%) einer Diagnosestellung, die auf einer ausführlichen neuropsychologischen Testung (Level II) basiert, unterlegen [35]. Im Vergleich zur Level-II-Diagnose einer PKD hat ein Testwert unterhalb von 25 Punkten eine hohe Trennschärfe (Sensitivität 71% und Spezifität 90%) [14], jedoch wurden auch höhere Werte (Cut-off < 29 Punkte) zur Klassifikation einer PKD (Sensitivität 78%, Spezifität 63%) berichtet [20]. Einflüsse des Bildungsgrade auf die Testleistung im MMST sind belegt [22]. Die Normierung des CERAD-Plus adjustiert für den Effekt des Geschlechts, des Alters und des Bildungsgrads (https://www.memoryclinic.ch/de/main-navigation/neuropsychologen/cerad-plus/). Weitere Kritik am MMST bezieht sich auf das Fehlen von exekutiven Aufgaben sowie die mangelnde Sensitivität des MMST für Veränderung [13].

Für das PANDA wurde in nationalen Kohorten ein starker Effekt zur Diskrimination zwischen PKD und nicht dementen Personen mit PK nachgewiesen [12]. Für einen Testwert unterhalb von 15 Punkten wurde eine gute Trennschärfe für PKD nach DSM-IV-Kriterien für die deutsche Version ermittelt (Sensitivität 73%, Spezifität 71%) [36] und in internationalen Studien bestätigt (Sensitivität 72%-97%, Spezifität 83%-100%) [37], [38]. Aufgrund der hohen Sensitivität des PANDA ist seine Anwendung besonders in den Frühphasen einer PKD empfehlenswert und beträgt zwischen 10 und 15 Minuten [30], [13]. Aktuell sind spezifische Aspekte der Reliabilität sowie die Sensitivität für Veränderungen noch nicht nachgewiesen [13]. Es wurde weiterhin angemerkt, dass das PANDA keinen spezifischen Subtest zur Erfassung der Aufmerksamkeit enthält [13]. Allerdings fließt die Aufmerksamkeit als kognitive Basisfunktion in die Subtests ein.

Die Durchführungszeit des Cambridge Cognitive Assessment – Revised (CAMCOG-R) beträgt ca. 25 bis 30 Minuten [13]. Bei einem Wert unterhalb von 81 Punkten wurde eine Sensitivität von 95% und eine Spezifität von 94% für die DSM-IV-Diagnose einer PKD nachgewiesen [39]. Eine Validierung der Befunde in internationalen und nationalen Kohorten steht noch aus. Weitere Kritik am CAMCOG-R bezieht sich auf die motorischen Fähigkeiten, welche für einige Aufgaben erforderlich sind, die Kulturabhängigkeit einzelner Aufgaben (z.B. Wiedererkennung bekannter Persönlichkeiten) sowie das Fehlen von spezifischen Reliabilitätskennwerten und der Sensitivität für Veränderung. Einflüsse von Alter und Schweregrad der PK auf die Testleistung sind beschrieben [40]. Die deutsche CAMCOG-R-Version ist für die Anwendung bei Personen ohne PK validiert [41].

Die ACE inkludiert den MMST, die Durchführungszeit für die 24 Aufgaben beträgt ca. 15 Minuten. Der diagnostische Trennwert in internationalen Studien für eine PKD nach DSM-5 variiert für die verschiedenen Versionen der ACE in Abhängigkeit vom Bildungsgrad [23]. Für die ACE-I beträgt die Sensitivität zwischen 82% und 90% und die Spezifität zwischen 74% und 93% für die verschiedenen Cut-off-Werte (0–8 Bildungsjahre <75 Punkte, 9–12 Bildungsjahre <77 Punkte, >12 Bildungsjahre <81 Punkte) [23]. Für die ACE-III beträgt die Sensitivität zwischen 81% und 89% und die Spezifität zwischen 74% und 93% für die verschiedenen Cut-off-Werte (0–8 Bildungsjahre <71 Punkte, 9–12 Bildungsjahre <78 Punkte, >12 Bildungsjahre <79 Punkte) [23]. Sensitivitätswerte für die Kurzversion der ACE (Mini-ACE) betragen zwischen 72% und 91% und die Spezifität beträgt zwischen 75% bis 92% (0–8 Bildungsjahre <18 Punkte, 9–12 Bildungsjahre <21 Punkte, >12 Bildungsjahre <22 Punkte) [23].

Die deutsche Version der ACE-I und ACE-R wurde bei Personen ohne PD-MCI und Demenz (z.B. Alzheimer-Erkrankung, frontotemporale Demenz, vaskuläre Demenz) validiert [25], [26]. Die diagnostische Güte der deutschen Version der ACE für PD-MCI ist aktuell noch ungeklärt. Der Nachweis der Reliabilität und der Sensitivität für Veränderung der Skala bei PK steht noch aus.

Begründung der Empfehlung

Auf der Basis der oben dargestellten Evidenzgrundlage werden Screeningverfahren zur Prüfung der globalen kognitiven Leistungsfähigkeit wie folgt empfohlen:

- Empfehlung: Nachweis der diagnostischen Trennschärfe für eine PKD in internationalen Kohorten und für die deutsche Version der Skala, die Testgüte des Verfahrens bei PK ist hinreichend belegt (Reliabilität, Validität und Sensitivität für Veränderung) und das Verfahren detektiert alle wesentlichen mit einer PKD assoziierten kognitiven Leistungsbereiche.
- Empfehlung mit Vorbehalt: Nachweis der diagnostischen Trennschärfe für eine PKD in internationalen Kohorten oder für die deutsche Version der Skala, Nachweise darüber, dass kognitive Störungen bei PK in nationalen Kohorten mit dem Verfahren detektiert werden und die Testgüte des Verfahrens bei PK eingeschränkt belegt ist (Fehlen des Nachweises spezifischer Testgütekriterien bei PK) oder das Verfahren nicht alle wesentlichen mit einer PKD assoziierten kognitiven Leistungsbereiche detektiert.
- Mögliche Testanwendung: Nachweis der diagnostischen Trennschärfe für eine PKD in internationalen Kohorten oder für die deutsche Version der Skala, die Testgüte des Verfahrens bei PK ist nicht belegt, aber die Testgüte der deutschen Version des Verfahrens für andere Erkrankungen ist belegt.

<table>
<tr><th>Empfehlung</th><th>Neu
Stand (2023)</th></tr>
<tr><td colspan="2">Für die Level-I-Diagnose einer PKD sollten kognitive Screeninginstrumente verwendet werden. Hierbei sollten, wo immer verfügbar, Korrekturen für Alter, Bildung und ggf. Geschlecht zur Anwendung kommen.
Geeignet sind besonders (sollte-Empfehlungsgrad):
▪ MoCA mit einem Cut-off unter 21 Punkten
▪ MDRS mit einem Cut-off unter 123 Punkten
▪ MMST mit einem Cut-off unter 26 Punkten
▪ PANDA mit einem Cut-off unter 15 Punkten
Weiterhin können folgende Instrumente verwendet werden (kann-Empfehlungsgrad):
▪ SCOPA-COG mit einem Cut-off unter 23 Punkten
▪ ACE/ ACE-R; Cut-off-Werte sind bildungsabhängig.</td></tr>
<tr><td colspan="2">Konsensstärke: 100%, starker Konsens</td></tr>
</table>

Fragestellung 115: Welche neuropsychologischen Testverfahren/Testbatterien (Level-II-Diagnostik) sind geeignet für die Erfassung kognitiver Defizite einer PD-MCI im Vergleich zu Personen mit PK ohne kognitive Störungen?

Hintergrund

Eine valide und reliable Testdiagnostik einer PD-MCI ermöglicht die Eingrenzung einer Risikogruppe für PKD und hat somit einen hohen Stellenwert bei der Früherkennung der Demenz [12]. Die Symptomausprägung von kognitiven Störungen ist sehr heterogen und individuell verschieden [42]. In frühen Phasen der PK sind oftmals exekutive Funktionen beeinträchtigt [43], [44], wie beispielsweise die Planung und die Überwachung mentaler Prozesse sowie das Umsetzen angemessener Verhaltensweisen. Aufmerksamkeitsstörungen und Gedächtnisprobleme sind ebenfalls frühe Anzeichen einer kognitiven Leistungsabnahme bei Personen mit PK [3], [45]. Normierte und standardisierte neuropsychologische Testverfahren in verschiedenen Sprachen können kognitive Einschränkungen bei Personen mit PK detektieren [46]. Für eine Level-II-Testung müssen zwei Tests für jede der fünf kognitiven Domänen (exekutive Funktionen, Aufmerksamkeit und Arbeitsgedächtnis, Gedächtnis, Sprache, visuokognitive Leistungen) angewendet werden. Der Cut-off zur Beurteilung kognitiver Störungen sollte nach MDS-Expertenkriterien zwischen 1 und 2 Standardabweichungen unterhalb des Mittelwerts einer Referenzgruppe liegen [2]. Die Anwendung von zwei neuropsychologischen Tests pro kognitive Domäne hat eine gute diagnostische Trennschärfe für die Level-II-Diagnose einer PD-MCI (Sensitivität von 81,3% und Spezifität von 85,7%) [47]. Generell bietet die Level-I-Diagnose einer PD-MCI eine geringere diagnostische Sicherheit als die Level-II-Kriterien [48], [35], [4]. Da viele kognitive Funktionstests sprachbasiert sind, werden oftmals länderspezifische Normen zur Beurteilung der Testleistung benötigt. Empfehlungen von internationalen Expertengruppen sind daher nicht unmittelbar auf die Situation in Deutschland übertragbar.

Evidenzgrundlage

Die Literatursuche in Pubmed erbrachte 1.160 Studien, eine doppelte Nennung wurde ausgeschlossen. Für die Literaturrecherche wurden folgende Schlagworte verwendet: (Parkinson disease OR Parkinson* OR Parkinson disorders OR Movement Disorders NOT [Wolff parkinson white syndrome OR wolff* AND Parkinson]* OR [wpw AND syndrome] OR wpw) AND (test batter* OR Cognitive test* OR neuropsychological test*) OR MDS task force criteria OR CERAD OR Consortium to Establish a Registry for Alzheimer's Disease OR Trail Making Test part B OR Phonemic Fluency Test OR memory OR executive OR visu* OR attention* OR language OR verbal OR fluen*) AND (PD-MCI OR mild cognitive impairment OR mild cognitive impairment OR cognitive dysfunction OR MCI OR PD-NC OR PDNC OR PD-CN OR PDCN OR normal cognition OR [normal cognitive AND status OR function]) AND (Meta-Analysis OR Systematic Review OR Reviews OR [original studies AND German]).

Nach Screening der Abstracts und Inhalte wurden 4 Literaturreferenzen als relevant für die Leitlinie bewertet. Zusätzlich wurden 5 Studien aus anderen Quellen selektiert, welche ebenfalls in diese Leitlinie einbezogen wurden.

Es fanden sich:

- 1 Metaanalyse
- 5 Originalarbeiten
- 2 Übersichtsartikel mit Testempfehlungen von Expertengruppen.

Ergebnis

Ein liberaler Cut-off (-1 Standardabweichung unterhalb des Mittelwerts der Referenzpopulation) zur Beurteilung kognitiver Einschränkung ermöglicht die frühe Detektion einer PD-MCI mit dem Risiko einer falsch positiven Klassifikation. Ein konservativer Cut-off eignet sich hingegen, um Personen mit einem Risiko für die zeitnahe Entwicklung einer PKD zu identifizieren [49], [50]. Als optimaler diagnostischer Wert wird ein Cut-off-Wert unterhalb der 1,5-fachen Standardabweichung des Mittelwerts der Referenzpopulation in mindestens zwei Tests einer Domäne (domänenspezifische PD-MCI) oder verschiedenen Domänen vorgeschlagen [49].

Neuropsychologische Tests, welche in einer großen multizentrischen Analyse mit hoher Trennschärfe zwischen Personen einer Kontrollgruppe (KG) und Personen mit PK unterschieden, inkludierten neuropsychologische Verfahren zur Testung der kognitiven Leistung in allen fünf kognitiven Domänen: Sprache (Wechsler-Intelligenztest: Gemeinsamkeiten finden), Aufmerksamkeit und Arbeitsgedächtnis (Wechsler-Intelligenztest: Zahlen-Symbol-Test, Trail Making Test (TMT) A, Stroop-Test: Farbwörter benennen und Farbwörter lesen, Zahlenspanne vorwärts, Zahlenspanne Gesamtwert (vorwärts und rückwärts), exekutive Funktionen (Wortflüssigkeit [Controlled Oral Word Association Test], Stroop-Test-Interferenz-Bedingung, TMT B, modifizierter Wisconsin Card Sorting Test – nicht perseverative Fehler), Gedächtnis (Wechsler-Gedächtnistest: Logisches Gedächtnis I und II) und visuell-konstruktive Leistungen (Benton Test Linien-Orientierung) [46]. Mit Ausnahme der Subtests zum logischen Gedächtnis wurde in einem unabhängigen Review bestätigt, dass die oben genannten neuropsychologischen Tests zwischen Kontrollpersonen und Personen mit PD-MCI differenzieren. Zusätzlich wurden folgende Verfahren und Paradigmen identifiziert: Gedächtnis, Wortliste lernen und abrufen, Wortliste abrufen, Wortliste wiedererkennen, Figuren abzeichnen, Rey-Osterrieth Complex Figure Test (ROCFT) [51]).

Neuropsychologische Tests, welche in nationalen Studien zwischen Personen ohne neurodegenerative Erkrankungen und Personen mit PK differenzieren, sind in den Empfehlungen von Mitgliedern einer nationalen Expertengruppe zusammengefasst [12]). Die höchsten Effektstärken zur Diskrimination zwischen Personen mit und ohne PK wurden für Tests zur Prüfung der Exekutivfunktionen (z.B. Fünf-Punkte-Test, Turm von London; Wisconsin Card Sorting Test – Anzahl Kategorie; TMT B/A), der Aufmerksamkeit (z.B. Digit Ordering Test, TMT A) und der visuokognitiven Fähigkeiten (z.B. ROCFT, Leistungsprüfsystem – Erkennen des Wesentlichen in einer Figur; Testbatterie für visuelle Objekt- und Raumwahrnehmung (VOSP) – Würfelanzahl analysieren) ermittelt. Im Bereich Gedächtnis erzielten der Benton Visual Retention Test und die Subaufgabe zum logischen Gedächtnis aus dem Wechslerl-Gedächtnistest I und II mittlere Effektstärken.

Die Ergebnisse einer Metaanalyse demonstrieren, dass Tests verschiedener kognitiver Domänen, die zwischen der Leistung von Personen mit und ohne PD-MCI mit hoher Effektstärke (Hedges $g > 0,8$) differenzieren, vor allem in den Bereichen Aufmerksamkeit und Arbeitsgedächtnis, exekutive

Funktionen, Sprache, visuell-räumliche Aufgaben und verbale/visuelle Lernleistung unmittelbare sowie deren verzögerte Wiedergabe Unterschiede zeigen [52].

Ergebnisse der LANDSCAPE-Studie belegen, dass alle Subtests des CERAD-Plus zwischen der kognitiven Leistung von Personen mit und ohne PD-MCI unterscheiden [53]. Im CERAD-Plus sind eine Reihe der zuvor genannten Aufgaben enthalten, wie beispielsweise der modifizierte Boston Naming Test (BNT), semantische und phonematische Wortflüssigkeitsaufgaben, Wortliste lernen, Wortliste abrufen, Wortliste wiedererkennen, Figuren abzeichnen, Figuren abrufen sowie der TMT.

Begründung der Empfehlung

Auf Basis der oben dargestellten Evidenz werden in Anlehnung an die Empfehlungen der Mitglieder der Arbeitsgruppe „Neuropsychologie bei Parkinson“ der Deutschen Gesellschaft für Parkinson und Bewegungsstörungen e.V. (DPG) [12] die in Tabelle 20 dargestellten standardisierten neuropsychologischen Testverfahren empfohlen. Für jede kognitive Domäne sind Tests für verschiedene kognitive Funktionen berücksichtigt. Grundlage der Empfehlung war das Vorliegen einer deutschen Testversion. Vorrangig wurden Verfahren berücksichtigt, welche in internationalen und/oder nationalen Studien zwischen PK-Personen mit und ohne PD-MCI differenzieren. Die Testempfehlung der Sprachtests ist aktuell nur für den BNT (CERAD-Plus) empirisch gesichert.

Die in Tabelle 20 gelisteten neuropsychologischen Testverfahren können für die Level-II-Diagnose einer PD-MCI herangezogen werden (Expertenmeinung).

Tab. 20: Testverfahren für die standardisierte neuropsychologische Diagnostik einer PD-MCI (adaptiert nach [12])

Domäne/Funktion	Testverfahren	Untertest
**Aufmerksamkeit**		
Aufmerksamkeitsspanne	Wechsler-Gedächtnistest	Zahlen- und visuelle Merkspanne vorwärts
Informationsverarbeitungs-geschwindigkeit	CERAD-Plus	Trail Making Test A
	Wechsler Intelligenztest für Erwachsene<br>Symbol Digit Modalities Test#	Zahlen-Symbol-Test
Selektive/fokussierte Aufmerksamkeit	Farbe-Wort-Interferenztest	Farbwörter lesen<br>Farben benennen
Arbeitsgedächtnis	Digit Ordering Test	
	Wechsler-Gedächtnistest:	Zahlenspanne und visuelle Merkspanne rückwärts
	Wechsler-Intelligenztest für Erwachsene	Buchstaben-Zahlen-Folge
	CERAD-Plus	Trail Making Test B-A
**Exekutivfunktionen – basale kognitive Regulationsprozesse**		
Initiieren/<br>Divergentes Denken	CERAD-Plus	Semantische Wortflüssigkeit Tiere<br>Phonematische Flüssigkeit S-Wörter

Domäne/Funktion	Testverfahren	Untertest
	Regensburger Wortflüssigkeitstest	
	Controlled Oral Word Association Test/FAS Test	
	Fünf-Punkte-Test	
Aufgabenwechsel (set-shifting)	CERAD-Plus	Trail Making Test B/A
**Exekutivfunktionen – komplexe kognitive Regulationsprozesse**		
Problemlösen und Planen	Wisconsin Card Sorting Test*	
	Behavioral Assessment of Dysexecutive Syndrome	
Abstraktionsvermögen/Logisches Denken	Leistungsprüfsystem	Logisches Denken
	Wechsler-Intelligenztest für Erwachsene	Gemeinsamkeiten finden
**Gedächtnis**		
Verbales Kurzzeitgedächtnis	Wechsler-Gedächtnistest	Logisches Gedächtnis I
	CERAD-Plus	Wortliste lernen, abrufen, wiederkennen
Verbales Langzeitgedächtnis	Wechsler-Gedächtnistest	Logisches Gedächtnis II
Verbales / visuelles assoziatives Gedächtnis	Wechsler-Gedächtnistest	verbale Paarkennung I
Visuelles Gedächtnis	CERAD-Plus	Figuren abrufen
	Rey-Osterrieth Complex Figure Test*	Figur abrufen<br>Figuren wiedererkennen
**Sprache**		
Auditives Sprachverständnis	ADAS-Cog	Anweisungen befolgen
Benennen	CERAD-Plus	Modifizierter Boston Naming Test
**Visuo-kognitive Leistungen**		
Visuoperzeption und -konstruktion	Rey-Osterrieth Complex Figure Test*	
	CERAD-Plus	Figuren abzeichnen
	Wechsler-Intelligenztest für Erwachsene	Bilder ergänzen
Visuell-räumliche Perzeption	VOSP	Würfelanzahl analysieren
	Leistungsprüfsystem	räumliches Vorstellungsvermögen<br>Erkennen des Wesentlichen in einer Figur

Legende: *Deutsche Testversion enthält amerikanische Normdaten; #. Kein deutsches Handbuch verfügbar, niederländische und englische Normdaten kommerziell erhältlich; ADAS-Cog. Alzheimer Disease

Assessment Scale Abschnitt zur Kognition; CERAD-Plus. Consortium to Establish a Registry for Alzheimer's Disease; VOSP – Testbatterie für visuelle Objekt- und Raumwahrnehmung

Empfehlung	Neu<br>Stand (2023)
Für die Level-II-Diagnose einer PD-MCI sind folgende Aspekte zu berücksichtigen:<br>▪ Für eine Level-II-Testung sollen zwei Tests für jede der fünf kognitiven Domänen (exekutive Funktionen, Aufmerksamkeit und Arbeitsgedächtnis, Gedächtnis, Sprache, visuokognitive Leistungen) angewendet werden.<br>▪ Für die Diagnose domänenspezifischer kognitiver Störungen sollen mindestens zwei Tests verschiedener Unterfunktionen einer kognitiven Domäne herangezogen werden.<br>▪ Als Cut-off zur Beurteilung der Testleistung soll ein Wert im Bereich von einer bis zwei Standardabweichungen unterhalb des Mittelwerts der Normstichprobe angewendet werden.	
Konsensstärke: 100%, starker Konsens	

Fragestellung 116: Welche neuropsychologischen Testverfahren/Testbatterien (Level-II-Diagnostik) sind geeignet für die Erfassung kognitiver Defizite bei Personen mit PKD im Vergleich zu Personen mit PK ohne Demenz?

Hintergrund

Eine Level-II-Testung sollte primär durchgeführt werden, wenn Ergebnisse der Level-I-Testung nicht eindeutig eine PKD bestätigen, jedoch der Verdacht einer Demenz weiterhin besteht. Darüber hinaus kann eine Level-II-Testung dazu beitragen, zwischen dem Vorliegen einer depressiven Störung und einer PKD zu differenzieren [31]. Im Vergleich mit einer neuropsychologischen Routinetestung ist die diagnostische Genauigkeit der Level-II-Testung für PKD exzellent (Sensitivität 100%, Spezifität 92,7%) und der Trennschärfe einer Level-I-Testung überlegen [48]. Somit eignet sich die Level-II-Testung insbesondere für eine frühe Diagnosestellung einer PKD. Verschlechterungen in Testverfahren zur Aufmerksamkeit [54] und zu den visuell-räumlichen Leistungen sind mit der späteren Konversion zur PKD assoziiert [55], [56]. Da viele kognitive Funktionstests sprachbasiert sind, werden oftmals länderspezifische Normen zur Beurteilung der Testleistung benötigt. Empfehlungen von internationalen Expertengruppen sind daher nicht unmittelbar auf die Situation in Deutschland übertragbar.

Evidenzgrundlage

Die Literatursuche in PubMed erbrachte 3.078 Studien, eine doppelte Nennung wurde ausgeschlossen. Für die Literaturrecherche wurden folgende Schlagworte verwendet: (Parkinson disease OR Parkinson* OR Parkinson disoders OR Movement Disorders NOT [Wolff parkinson white syndrome OR wolff* AND parkinson*] OR [wpw AND syndrome] OR wpw) AND (test batter* OR Cognitive test* OR neuropsychological test*) OR MDS task force criteria OR CERAD OR Consortium to Establish a Registry for Alzheimer's Disease OR Trail Making Test part B OR Phonemic Fluency Test OR memory OR executive OR visu* OR attention* OR language OR verbal OR fluen*) AND (PDD OR

Parkinson disease with dementia OR Parkinson dementia OR cognitive dysfunction OR PD-MCI OR MCI OR PD-NC OR PDNC OR PD-CN OR PDCN OR non-demented OR Parkinson's disease without dementia OR [normal cognitive AND status] OR function OR controls OR control group OR PDnD) AND (Meta-Analysis OR Systematic Review OR Reviews OR [original studies AND German]).

Nach Screening der Abstracts und Inhalte wurden 3 Literaturreferenzen als relevant für die Leitlinie bewertet. Zusätzlich wurden 4 Studien aus anderen Quellen selektiert, welche ebenfalls in diese Leitlinie einbezogen wurden.

Es fanden sich:

- 1 Metaanalyse
- 5 Originalarbeiten
- 1 Übersichtsartikel mit Testempfehlungen einer Expertengruppe.

Ergebnis

Bezogen auf den Schweregrad der Einschränkungen, weisen Personen mit PKD Einschränkungen unterhalb einer Standardabweichung im Vergleich zu Normwerten einer Referenzgruppe [50] auf. Neuropsychologische Tests, welche in nationalen Studien kognitive Einschränkungen bei PK im Vergleich zu Personen ohne neurodegenerative Erkrankungen differenzieren, sind in den Empfehlungen von Mitgliedern einer nationalen Expertengruppe zusammengefasst [12]. Die höchsten Effektstärken zur Diskrimination zwischen Personen mit und ohne PK wurden für Tests zur Prüfung der Exekutivfunktionen (z.B. Fünf-Punkte-Test; Turm von London; Wisconsin Card Sorting Test – Anzahl Kategorie, TMT B/A), der Aufmerksamkeit (z.B. Digit Ordering Test, TMT A) und der visuell-räumlichen Fähigkeiten (z.B. ROCFT, Leistungsprüfsystem – Erkennen des Wesentlichen in einer Figur, VOSP – Würfelanzahl analysieren) ermittelt. Im Bereich Gedächtnis erzielte der Benton Visual Retention Test eine hohe Effektstärke und der Subtest Logisches Gedächtnis aus dem Wechsler Gedächtnistest erzielte für Part I hohe und für den Part II mittlere Effektstärke.

Internationale Studien belegen, dass eine Reihe von Testverfahren zwischen Personen mit und ohne PKD differenzieren. Nachfolgend genannt sind Aufgaben, für die eine deutsche Version des Tests oder Äquivalente vorliegen: Wortflüssigkeit (semantisch, phonematisch), Wortliste unmittelbarer und freier Abruf (Hopkins Verbal Learning Test), konstruktive Praxis (ROCFT, Benton Test Linien-Orientierung, VOSP – unvollständige Buchstaben, Uhrentest), Sprache (BNT, Wechsler-Gedächtnistest: Gemeinsamkeiten finden) [57], [58]. Im Vergleich zu Personen mit PD-MCI und Demenz anderer neurodegenerativer Erkrankungen, wie beispielsweise der Alzheimer Erkrankung, differenzieren Aufgaben zur Wortflüssigkeit (Controlled Oral Word Association Test) am stärksten zwischen Personen mit PK und Demenz vs. PD-MCI [59].

Eine Metaanalyse belegt, dass Personen mit PKD im Vergleich zu Personen mit PD-MCI Einschränkungen in allen Bereichen der visuell-kognitiven Leistungen aufweisen [60]. Die Tests Corsi Block Tapping Test, Benton Test Linien-Orientierung (30 Items Langversion), ROCFT abzeichnen, Figuren abzeichnen aus dem CERAD-Plus sowie dem ROCFT Verzögerte Wiedergabe können am besten zwischen Personen mit PKD und mit PD-MCI unterscheiden.

Ergebnisse der LANDSCAPE-Studie belegen, dass alle Subtests des CERAD-Plus zwischen der kognitiven Leistung von Personen mit und ohne Demenz bei PK differenzieren [53]. Im CERAD-Plus sind eine Reihe der zuvor genannten Aufgaben enthalten, wie beispielsweise der modifizierte BNT, semantische und phonematische Wortflüssigkeitsaufgaben, Wortliste lernen, Wortliste abrufen, Wortliste wiedererkennen, Figuren abzeichnen und Figuren abrufen sowie der TMT. Internationale Studien belegen die moderate (semantische Wortflüssigkeit, Wortliste wiedererkennen) bis hohe diagnostische Trennschärfe der CERAD-Plus-Subtests für PKD, mit der besten Differenzierbarkeit zwischen dementen und nicht dementen Personen mit PK für den Untertest Wortliste lernen [61].

Begründung der Empfehlung

Auf Basis der oben dargestellten Evidenz werden in Anlehnung an die Empfehlungen der Mitglieder der Arbeitsgruppe „Neuropsychologie bei Parkinson“ der DPG [12] die in Tabelle 21 dargestellten standardisierten neuropsychologischen Testverfahren empfohlen. Für jede kognitive Domäne sind Tests für verschiedene kognitive Funktionen berücksichtigt. Grundlage der Empfehlung war das Vorliegen einer deutschen Testversion. Vorrangig wurden Verfahren berücksichtigt, welche in internationalen und/oder nationalen Studien zwischen Personen mit und ohne PKD differenzieren. Darüber hinaus wurde auf die Praktikabilität der Anwendung bei Personen mit PKD geachtet (mittlerer Schwierigkeitsgrad und zeitökonomische Durchführung). Die Testempfehlung der Sprachtests ist aktuell nur für den BNT (CERAD-Plus) empirisch gesichert. Empfehlungen zur Anwendung der Testbatterie zur Aufmerksamkeitsprüfung für den Untertest Go/Nogo sind Expertenmeinungen und empirisch nicht gesichert.

Die in Tabelle 21 gelisteten neuropsychologischen Testverfahren können für die Level-II-Diagnose einer PKD herangezogen werden (Expertenmeinung).

Tab. 21: Testverfahren für die standardisierte neuropsychologische Diagnostik einer PKD (adaptiert von [12])

Domäne/Funktion	Testverfahren	Untertest
**Aufmerksamkeit**		
Aufmerksamkeitsspanne	Wechsler-Gedächtnistest	Zahlen- und visuelle Merkspanne vorwärts
Informationsverarbeitungs-geschwindigkeit	CERAD-Plus	Trail Making Test A
	Wechsler-Intelligenztest für Erwachsene	Zahlen-Symbol-Test
	Zahlen-Verbindungs-Test	
Selektive/fokussierte Aufmerksamkeit	Nürnberger Alters-Inventar	Farb-Wort-Test
	Testbatterie zur Aufmerksamkeitsprüfung	Go/Nogo
Arbeitsgedächtnis	Digit Ordering Test	
	Wechsler-Gedächtnistest:	Zahlenspanne rückwärts<br>visuelle Merkspanne rückwärts

Domäne/Funktion	Testverfahren	Untertest
	Wechsler-Intelligenztest für Erwachsene	Buchstaben-Zahlen-Folge
	CERAD-Plus	Trail Making Test B-A
**Exekutivfunktionen – basale kognitive Regulationsprozesse**		
Initiieren<br>Divergentes Denken	CERAD-Plus	Semantische Wortflüssigkeit Tiere<br>Phonematische Flüssigkeit S-Wörter
	Regensburger Wortflüssigkeitstest	
	Controlled Oral Word Association Test/FAS Test	
Wechseln	CERAD-Plus	Trail Making Test B/A
**Exekutivfunktionen – komplexe kognitive Regulationsprozesse**		
Problemlösen und Planen	Wisconsin Card Sorting Test*	
	Behavioral Assessment of Dysexecutive Syndrome	
Allgemeines Abstraktionsvermögen / Logisches Denken	Wechsler-Intelligenztest für Erwachsene	Gemeinsamkeiten finden
**Gedächtnis**		
Verbales Kurzzeitgedächtnis:	Wechsler-Gedächtnistest	Logisches Gedächtnis I
	CERAD-Plus	Wortliste lernen, abrufen, wiederkennen
Verbales Langzeitgedächtnis	Wechsler-Gedächtnistest	Logisches Gedächtnis II
Verbales / visuelles assoziatives Gedächtnis	Wechsler-Gedächtnistest	Verbale Paarkennung I
Visuelles Gedächtnis	CERAD-Plus	Figuren abrufen
	Rey-Osterrieth Complex Figure Test*	Figur abrufen<br>Figuren wiedererkennen
**Sprache**		
Auditives Sprachverständnis	ADAS-Cog	Anweisungen befolgen
Benennen	CERAD-Plus	modifizierter Boston Naming Test
**Visuokognitive Leistungen**		
Visuoperzeption und konstruktion	Rey-Osterrieth Complex Figure Test*	
	CERAD-Plus	Figuren abzeichnen
	Wechsler Intelligenztest für Erwachsene	Bilder ergänzen
Visuell-räumliche Perzeption	VOSP	Würfelanzahl analysieren

Domäne/Funktion	Testverfahren	Untertest
		Unvollständige Buchstaben
	Leistungsprüfsystem	Räumliches Vorstellungsvermögen<br>Erkennen des Wesentlichen in einer Figur

Legende: *Deutsche Testversion enthält amerikanische Normdaten; ADAS-Cog. Alzheimer Disease Assessment Scale Abschnitt zur Kognition; CERAD-Plus. Consortium to Establish a Registry for Alzheimer's Disease; VOSP – Testbatterie für visuelle Objekt- und Raumwahrnehmung

Empfehlung	Neu<br>Stand (2023)
Für die Level-II-Diagnose einer PKD sind folgende Aspekte zu berücksichtigen:<br>▪ Zur Diagnosesicherung einer PKD durch eine Level-II-Testung sollen zwei Tests für jede der fünf kognitiven Domänen (exekutive Funktionen, Aufmerksamkeit und Arbeitsgedächtnis, Gedächtnis, Sprache, visuokognitive Leistungen) angewendet werden.<br>▪ Als Cut-off zur Beurteilung der Testleistung soll ein Wert im Bereich von einer bis zwei Standardabweichungen unterhalb des Mittelwerts der Normstichprobe angewendet werden.	
Konsensstärke: 96,6%, starker Konsens	

Fragestellung 117: Welche Skalen/Verfahren sind geeignet für die Erfassung kognitiv assoziierter Aktivitäten des täglichen Lebens bei Personen mit PKD im Vergleich zu Personen mit PK ohne Demenz?

Hintergrund

Das Kernkriterium einer PKD zur Differenzierung einer PD-MCI ist die offensichtliche Minderung der Alltagskompetenz, hervorgerufen durch den kognitiven Leistungsabbau [10]. Unter Alltagskompetenz, den ADL, versteht man, dass ein erwachsener Mensch alltägliche Aufgaben in seinem Kulturkreis selbstständig und unabhängig in einer eigenverantwortlichen Weise erfüllen kann. Komplexe Aufgaben des Alltags, die instrumentellen Aktivitäten des täglichen Lebens (IADL), umfassen beispielsweise Finanzen regeln, Einkaufen oder soziale Aktivitäten, bei denen oftmals eine Interaktion mit der Umwelt erforderlich ist. Die Basis-ADL (BADL) dienen in erster Linie der Aufrechterhaltung des körperlichen Wohlbefindens, beispielsweise essen, sich waschen und sich anziehen. Einschränkungen in den IADL gehen denen in den BADL voraus [62]. Leichte Einschränkungen in den IADL sind bereits bei 30% bis 50% der Personen mit PD-MCI zu beobachten [63], [64]. Personen mit PK tendieren dazu, ihre Alltagskompetenz zu überschätzen [65]. Darüber hinaus können Einschränkungen der Motorik [66], der Stimmung [67] und andere Verhaltensauffälligkeiten [68] die Alltagskompetenz negativ beeinflussen. Die Trennung von kognitiven und anderen Einflussgrößen auf die Alltagskompetenz stellt somit eine besondere Herausforderung dar.

Evidenzgrundlage

Die Literatursuche in PubMed erbrachte 82 Studien. Für die Literaturrecherche wurden folgende Schlagworte verwendet: (Parkinson disease OR Parkinson* OR Parkinson disorders OR Movement Disorders NOT [Wolff parkinson white syndrome OR wolff* AND parkinson*] OR [wpw AND syndrome] OR wpw) AND (daily life activities OR everyday life OR everyday function OR activities of daily living OR activities in daily life OR activities of daily living OR Katz Index OR Lawton Scale OR Cognitive Disability Index OR Naturalistic Action Test OR Bayer ADL OR Instrumental ADL OR IADL OR basic ADL OR BADL OR Functional Activities Questionnaire OR cognitive ADL* OR Erlangen Test Activities of Daily Living OR cognitive activity OR cognitive activities) AND ((PDD OR Parkinson disease with dementia OR Parkinson dementia OR cognitive dysfunction OR PD-MCI OR MCI OR PD-NC OR PDNC OR PD-CN OR PDCN OR non-demented OR Parkinson's disease without dementia OR [normal cognitive AND status] OR function OR controls OR control group OR PDnD) AND (humans NOT animals AND [english OR german]).

Nach Screening der Abstracts und Inhalte wurden 6 Literaturreferenzen als relevant für die Leitlinie bewertet. Zusätzlich wurden 7 Studien aus anderen Quellen selektiert, welche ebenfalls in diese Leitlinie einbezogen wurden.

Es fanden sich:

- 12 Originalarbeiten, 1 Übersichtsartikel mit Testempfehlungen einer Expertengruppe

Darüber hinaus wurden 3 Artikel, welche über relevante Aspekte der Verfahren informieren, einbezogen.

Ergebnis

Der Instrumental Activities of Daily Living (IADL)-Fragebogen von Lawton & Brody [69] wird als Selbst- und Fremdbeurteilungsverfahren angewandt [70] und umfasst 8 verschiedene Funktionsbereiche (z.B. einkaufen, Verkehrsmittel benutzen), welche auf einer dreistufigen Likert-Skala eingeschätzt werden. Aufgrund der guten Testgüte des Verfahrens empfiehlt eine Expertengruppe der MDS die Anwendung der Skala zur Beurteilung von Einschränkungen durch die PK-Erkrankung in der klinischen Routine [71]. In einer nationalen Kohorte wurde die Testleistung von 85 Personen mit PK ohne Demenz und 21 Personen mit PKD verglichen [70]. Für die Selbstangaben der Personen mit PK ist die Sensitivität der Skala bei einem Cut-off von <18 Punkten gering (57%), aber die Spezifität hoch (89%). Für die Fremdeinschätzung durch eine Person mit regelmäßigem Kontakt zu der eingeschätzten Person wurden eine Sensitivität von 71% und eine Spezifität von 82% (Cut-off <19 Punkte) für die PKD-Diagnose nach DSM-IV-Kriterien ermittelt. Die diagnostische Trennschärfe für PKD wurde bisher in internationalen Studien nicht untersucht. Gruppenvergleiche belegen jedoch, dass die fremdbeurteilte Skala die Testleistung zwischen Personen mit und ohne Demenz (auch PD-MCI vs. PKD) differenzieren kann [72], [73].

Ein weiteres Maß zur Beurteilung der ADL ist die Schwab-und-England-Skala. Diese kann von Fach- und Pflegekräften oder von Personen mit PK selbst eingeschätzt werden [71]. In 10er-Schritten wird die ADL-Leistung von 0 (bettlägerig) bis 100 (völlig unabhängig) beurteilt. Aufgrund der guten Testgüte des Verfahrens bei PK wird die Skala zur klinischen Beurteilung von Einschränkungen durch

die PK-Erkrankung empfohlen [71]. In einer nationalen Kohorte wurde bei einem Cut-off unterhalb von 75% eine Sensitivität von 71% und eine Spezifität von 77% für PKD ermittelt [70]. Die Testleistung von Personen mit PKD kann im Gruppenmittel nicht zwischen der Testleitung von PD-MCI differenzieren, wohl jedoch zwischen Personen mit PK ohne kognitive Einschränkung [74].

Der Abschnitt II zur Beurteilung der ADL-Leistung der Unified Parkinson's Disease Rating Scale (UPDRS-II) misst sowohl den funktionellen Status als auch Symptome der Parkinson-Erkrankung (10-[71]. Die diagnostische Trennschärfe für PKD in einer nationalen Kohorte ergab eine Sensitivität von 67% und eine Spezifität von 78% (Cut-off >15 Punkte) des Verfahrens [70]. Einschätzungen auf der Schwab-und-England-Skala differenzieren Gruppen mit und ohne Demenz [75]. Jedoch kann im Gruppenmittel die Leistung von Personen mit und ohne PD-MCI nicht unterschieden werden [74]. Für die MDS-UPDRS-II liegen bisher keine Informationen zur Trennschärfe einer PKD vor.

Von einer Expertengruppe der MDS wurde der Pill-Questionnaire zur Beurteilung der ADL-Leistung vorgeschlagen [31]. Aufgaben der Personen mit PK ist es, die Einnahme ihrer Parkinson-Medikation zu berichten. Somit ist der Pill-Questionnaire in erster Linie eine Gedächtnisaufgabe und verlangt keine Demonstration von direkt beobachtbaren ADL-Funktionen oder eine Transferleistung. Internationale und nationale Studien belegen eine gute Trennschärfe des Pill-Questionnaire für PKD (Sensitivität 89% bis 91%; Spezifität 89% bis 97%) [76], [70]. Jedoch wird explizit darauf hingewiesen, dass der Pill-Questionnaire eine hohe Falsch-positiv-Rate aufweist und das Vorliegen einer PKD bei positivem Testergebnis weiter abgeklärt werden muss [76]. Darüber hinaus kann die Testausführung möglicherweise durch verschiedene Faktoren (z.B. Vorliegen einer Apathie, Depression, Bildungsniveau) negativ beeinflusst sein. Die Reliabilität des Verfahrens und die Sensitivität für Veränderung sind für PK bisher nicht belegt.

Für die folgenden Verfahren liegen Kennwerte für die diagnostische Trennschärfe einer PKD in einer nationalen Kohorte vor: Nurses' Observation Scale for Geriatric Patients (NOSGER) ADL Skala (Cut-off >7 Punkte, Sensitivität 67%, Spezifität 86%), NOSGER IADL Skala (Cut-Off >11 Punkte, Sensitivität 62%, Spezifität 76%), die Nürnberger-Alters-Inventar-Beobachtungsskala (NAB-ADL, Cut-Off >22 Punkte, Sensitivität 81%, Spezifität 80%) und Nürnberger-Alters-Inventar-Aktivitätenskala (NAA-ADL Cut-Off >34, Sensitivität 76%, Spezifität 85%) [70]. Diese Verfahren wurden für die Anwendung im deutschen Sprachraum entwickelt, jedoch nicht spezifisch, um Einschränkungen bei PK zu detektieren. Die Testgüte der NOSGER-Skala wurde in einer Gruppe von nicht neurodegenerativ erkrankten Personen und Personen mit Demenz belegt [77], [78]. Für die Interpretation des NAB-ADL und NAA-ADL liegen Normwerte aus repräsentativ quotierten Altersgruppen (55 bis 95 Jahre) vor [79]. Eine Replikation der Befunde in weiteren Kohorten steht noch aus.

Die diagnostische Trennschärfe der Parkinson's Disease Cognitive Functional Rating Scale (PD-CRFT) ist sowohl für den Vergleich von PKD und nicht dementen Personen mit PK gegeben (Cut-off >6 Punkte, Sensitivität 83%, Spezifität 83%) [80]. Studien belegen die Reliabilität und Validität des Verfahrens [80], [81], [82]. Die diagnostische Trennschärfe für PKD wurde für die deutsche Version der Skala bisher nicht überprüft.

Begründung der Empfehlung

Auf der Basis der oben dargestellten Evidenzgrundlage werden ADL-Verfahren wie folgt empfohlen:

- Empfehlung: Nachweis der diagnostischen Trennschärfe für eine PKD in internationalen Kohorten und für die deutsche Version der Skala, Verwendung der deutschen Version, die Testgüte des Verfahrens bei PK ist hinreichend belegt (Reliabilität, Validität und Sensitivität für Veränderung).
- Empfehlung mit Vorbehalt: Nachweis der diagnostischen Trennschärfe für eine PKD in internationalen Kohorten oder für die deutsche Version der Skala, die Testgüte des Verfahrens bei PK ist eingeschränkt belegt (Fehlen des Nachweises spezifischer Testgütekriterien bei PK).
- Mögliche Testanwendung: Nachweis der diagnostischen Trennschärfe für eine PKD in internationalen Kohorten oder für die deutsche Version der Skala, die Testgüte des Verfahrens bei PK ist nicht belegt, aber die Testgüte der deutschen Version des Verfahrens für andere Erkrankungen ist belegt.

**Empfehlung**	**Neu**<br>**Stand (2023)**
▪ Als orientierende Screeningverfahren für Einschränkungen in den ADL sollte der IADL-Fragebogen zur Fremdeinschätzung von Lawton & Brody mit einem Cut-off unter 19 Punkten, der UPDRS-II mit einem Cut-off über 15 Punkten, der PD-CRFT mit einem Cut-off oberhalb von 6 Punkten, die Schwab-und-England-Skala mit einem Cut-off unterhalb von 75% sowie der Pill-Questionnaire angewendet werden.<br>▪ Folgende ADL-Verfahren können als orientierende Verfahren zur Beurteilung von ADL-Einschränkungen verwendet werden: NAB-ADL mit einem Cut-off über 22 Punkten, NAA-ADL mit einem Cut-off oberhalb von 34 Punkten, NOSGER-ADL mit einem Cut-off über 7 Punkten und NOSGER-IADL mit einem Cut-off oberhalb von 11 Punkten.<br>▪ Bei einem positiven Testergebnis soll durch weitere Maßnahmen (z.B. Anamnesegespräch) überprüft werden, ob die ADL-Einschränkung primäre Konsequenz der kognitiven Leistungsminderung ist (Expertenmeinung).	
Konsensstärke: 100%, starker Konsens	

Fragestellung 118: Wie effektiv sind kognitives Training, physische Interventionen und spezifische Ernährungsweisen im Vergleich zur Vergleichsintervention zur Prävention der Diagnose PD-MCI?

Hintergrund

Aus dem Nicht-Parkinson-Demenzbereich wird der Anteil der potenziell modifizierbaren Demenzfälle auf 40% geschätzt, wobei hierbei insbesondere lebensstilbezogene Faktoren und ihr Einfluss auf das Demenzrisiko über die gesamte Lebensspanne in den Blick genommen werden ([83]. So können z.B. regelmäßige körperliche Aktivität sowie eine ausgewogene Ernährung und ihr Effekt auf kardiovaskuläre Risikofaktoren, **Erholung,** der Verzicht auf das Rauchen und auf einen schädlichen Alkoholkonsum die Gesundheit von Personen stärken und Krankheiten vermeiden [84]. Als mögliche Vorstufe einer Demenz kommt der PD-MCI bei der PK eine besondere Bedeutung in der Prävention der PKD zu. Der bereits in diesem Kapitel dargelegten Häufigkeit von PD-MCI stehen fehlende

pharmakologische Therapieoptionen gegenüber [85]. Vor diesem Hintergrund, aber auch unabhängig von pharmakologischen Optionen gewinnen nicht pharmakologische Interventionsansätze zur Behandlung und Prävention einer PD-MCI zunehmend an Bedeutung [85]. In Kohorten ohne PD verringert physisches Training mit einer Frequenz von zweimal pro Woche das Risiko von kognitiven Störungen, vor allem wenn gemeinsam mit anderen Personen trainiert wird [86]. Physische Interventionen und Nahrungsergänzungsmittel können dem inflammatorischen Prozess bei neurodegenerativen Erkrankungen entgegenwirken [87]. Kognitive und physische Interventionen verbessern die Leistungsfähigkeit bei Personen mit PK [88], [89], [90]. Der Effekt von spezifischen Ernährungsweisen auf die Kognition bei Personen mit PK wird aktuell diskutiert [91].

Evidenzgrundlage

Die Literatursuche in Pubmed erbrachte 66 Studien. Für die Literaturrecherche wurden folgende Schlagworte verwendet: (Parkinson disease OR Parkinson* OR Parkinson disorders OR Movement Disorders NOT [Wolff parkinson white syndrome OR wolff* AND parkinson*] OR [wpw AND syndrome] OR wpw) AND (cognitive training OR cognitive interventions) OR [global cognitive function AND training] OR [executive function AND training] OR [processing speed AND training] OR [memory AND training] OR [attention AND training] OR [mood AND training] OR [emotion AND training] OR [motivation AND training] OR [brain cortex AND training] OR [orientation AND training] OR physical training OR physical interventions OR [gait AND training] OR [balance AND training] OR [mobility AND training] OR specific nutrition OR diet OR comparable OR similar OR analogous) AND (intervention OR controlled intervention OR control intervention OR wait list OR waiting list) AND (PD-MCI OR mild cognitive impairment OR mild cognitive impairment) OR cognitive dysfunction OR [prevention AND diagnosis]) AND (humans NOT animals) AND (2016:2021) AND [english OR german]). Nach Screening der Abstracts und Inhalte wurde keine Interventionsstudie mit Endpunkt des Zeitpunkts der Diagnose PD-MCI, auch nicht aus anderen Quellen, identifiziert. In die Leitlinie eingeschlossen wurde 1 Langzeitbeobachtungsstudie, welche den Effekt von physischer Aktivität auf die Diagnose von PD-MCI untersucht, identifiziert durch eine weitere unsystematische Suche in Pubmed.

Ergebnis

Selbstangaben von 307 Personen der Parkinson’s Progression Markers Initiative (PPMI)-Studie über ein vermindertes Aktivitätsniveau, erhoben mit dem Physical Activity Scale for the Elderly (PASE), waren mit einem erhöhten Risiko einer PD-MCI und PKD nach zwei Jahren assoziiert [92]. Aktuell liegen keine Studien zur Prävention von PD-MCI durch kognitive und physische Interventionen sowie spezifische Ernährungsweisen vor.

Begründung der Empfehlung

Es besteht keine Evidenz für den präventiven Effekt von kognitiven und physischen Interventionen sowie spezifischen Ernährungsweisen auf PD-MCI.

Empfehlung	Neu<br>Stand (2023)
Die empirische Datenlage ist nicht ausreichend, um die Wirksamkeit von kognitiven und physischen Interventionen sowie speziellen Ernährungsweisen zur Prävention von PD-MCI zu beurteilen (Expertenmeinung).	
Konsensstärke: 96,9%, starker Konsens	

Fragestellung 119: Wie effektiv sind jeweils kognitives Training, physische Intervention und spezifische Ernährungsweisen im Vergleich zur Vergleichsintervention zur Prävention der PKD?

Hintergrund

Die Diagnose einer PKD verringert die Lebensqualität Betroffener und von deren Angehöriger und erhöht die Wahrscheinlichkeit für eine Übersiedlung in eine Pflegeeinrichtung und Mortalität [93]. Somit kommen der Prävention der PKD und der Stabilisierung der kognitiven Leistung im Prodromalstadium der Demenz eine wesentliche Bedeutung zu. Physische Aktivität verringert das Risiko einer Demenz nach drei Jahren in älteren Personen (>65 Jahre) [94], [95]. Bei Personen mit PK ist ein vermehrtes sedentäres Verhalten mit einer schlechteren kognitiven Leistung und ADL-Einschränkungen assoziiert [96], [97]. Kognitive und physische Interventionen modulieren die kognitive Leistung auch im Prodromalstadium der Demenz, d.h. bei Personen mit PD-MCI [88], [90], [98]. Personen mit PK und kognitiven Störungen haben höhere Homocysteinspiegel, verringerte Folsäure- und Vitamin-B12-Werte im Vergleich zu Personen mit PK ohne kognitive Störungen [99].

Evidenzgrundlage

Die Literatursuche in Pubmed erbrachte drei Studien. Für die Literaturrecherche wurden folgende Schlagworte verwendet: (Parkinson disease OR Parkinson* OR Parkinson disorders OR Movement Disorders NOT [Wolff parkinson white syndrome OR wolff* AND parkinson*] OR [wpw AND syndrome] OR wpw) AND (cognitive training OR cognitive interventions) OR [global cognitive function AND training] OR [executive function AND training] OR [processing speed AND training] OR [memory AND training] OR [attention AND training] OR [mood AND training] OR [emotion AND training] OR [motivation AND training] OR [brain cortex AND training] OR [orientation AND training] OR physical training OR physical interventions OR [gait AND training] OR [balance AND training] OR [mobility AND training] OR specific nutrition OR diet OR comparable OR similar OR analogous) AND (intervention OR controlled intervention OR control intervention OR wait list OR waiting list) AND ([prevention AND diagnosis] OR PDD OR Parkinson disease with dementia OR Parkinson disease dementia OR Parkinson dementia) AND (humans NOT animals) AND (2016:2021) AND [english OR german]). Nach Screening der Abstracts und Inhalte wurde keine Interventionsstudie mit Endpunkt des Zeitpunkts der Diagnose PKD, auch nicht aus anderen Quellen, identifiziert. In die Leitlinie eingeschlossen wurde eine Langzeitstudie, welche den Effekt von physischer Aktivität auf die Diagnose von PKD untersucht, identifiziert durch eine weitere unsystematische Suche in Pubmed.

Ergebnis

Selbstangaben von 307 Personen der PPMI-Studie über ein vermindertes Aktivitätsniveau, erhoben mit dem PASE, waren mit einem erhöhten Risiko einer PD-MCI und PKD nach zwei Jahren assoziiert [92].

Begründung der Empfehlung

Es besteht keine Evidenz für den präventiven Effekt von kognitiven und physischen Interventionen sowie spezifischen Ernährungsweisen auf PKD.

Empfehlung	Neu<br>Stand (2023)
Die empirische Datenlage ist nicht ausreichend, um die Wirksamkeit von kognitiven und physischen Interventionen sowie speziellen Ernährungsweisen zur Prävention von PKD zu beurteilen (Expertenmeinung).	
Konsensstärke: 100%, starker Konsens	

Wichtige wissenschaftliche Fragestellungen

Folgende Fragestellungen sind zu diesem Thema bislang unbeantwortet und haben große Auswirkung auf die hier getroffenen Empfehlungen:

- Können kognitive Interventionen bei Personen mit PK und PD-MCI zur Vorbeugung der PKD beitragen?
- Kann physisches Training bei Personen mit PK und PD-MCI zur Vorbeugung der PKD beitragen?
- Wie lange sollen kognitive bzw. körperliche Interventionsmaßnahmen durchgeführt werden?
- Besteht für kognitives und physisches Training ein Zusammenhang zwischen Dosis und Wirkung? Welche Trainingsdosis zeigt die größten Effekte?

Fragestellung 120: Wie effektiv ist Memantin im Vergleich mit Placebo in der Behandlung von kognitiven Defiziten bei PD-MCI?

Hintergrund

Kognitive Störungen kommen in leichter Ausprägung im Mittel bei ca. 25–28% aller Personen mit PK vor [1], [2]. Bei bis zu einem Drittel (19–36%) der Personen ist eine PD-MCI bereits zum Zeitpunkt der klinischen Diagnose nachweisbar [3]. Das Auftreten einer PD-MCI erhöht das Risiko zur Entwicklung einer PKD [4], [5]. Im Durchschnitt entwickeln 31% aller Personen mit PD-MCI innerhalb von sieben Jahren eine Demenz [11]. Memantin hemmt die (pathologische) Aktivität von NMDA-Rezeptoren. Memantin wird zur Therapie von kognitiven Störungen bei Personen mit Alzheimer-Erkrankung eingesetzt [100]. Somit stellt sich die Frage, ob Memantin die Kognition bei PD-MCI verbessern kann.

Evidenzgrundlage

Die Literatursuche in PubMed erbrachte drei Studien. Für die Literaturrecherche wurden folgende Schlagworte verwendet: (Parkinson disease OR Parkinson* OR Parkinson disorders OR Movement Disorders NOT [Wolff parkinson white syndrome OR wolff* AND parkinson*] OR [wpw AND syndrome] OR wpw) AND (Memantine OR memantine) AND (placebo) AND cognitive deficit OR treatment OR therapy OR cognitive dysfunction OR PD-MCI OR mild cognitive impairment OR mild cognitive impairment) AND (humans NOT animals) AND (2016:2021) AND (english OR german).

Nach Screening der Abstracts und Inhalte wurde keine Literaturreferenz als relevant für die Leitlinie bewertet. Zusätzlich wurde eine Studie durch eine unsystematische Suche in Pubmed selektiert, welche in diese Leitlinie einbezogen wurde.

Es fand sich:

- 1 randomisierte kontrollierte Studie (RCT).

Ergebnis

Es wurde eine RCT mit Crossover-Design mit jeweils sechswöchiger Behandlung (Memantin vs. Placebo) zur Verbesserung kognitiver Symptome bei PD-MCI durchgeführt [101]. In die Studie eingeschlossen wurden 12 Personen mit PD-MCI, diagnostiziert nach Level-II-Kriterien der Expertengruppe der MDS, von denen 10 Personen (8 Männer, 2 Frauen) die Studie beendet haben. Die Memantin-Dosis betrug 5 mg/Tag pro Woche, mit wöchentlicher Dosiserhöhung bis zu einer Zieldosis von 20 mg/Tag. Die Personen wurden für drei Wochen mit der maximalen Dosis behandelt. Nach einer sechswöchigen behandlungsfreien Phase wurde die Medikation (Memantin vs. Placebo) gewechselt, Personen, welche im ersten Durchgang Memantin bekommen hatten, erhielten nun Placebo und vice versa. Während der maximalen Behandlungsphase wurden eine neuropsychologische Testung und eine Magnetresonanztomographie durchgeführt. Die Zeit bis zur Beendigung des TMT Part A war unter Gabe von Memantin verlängert im Vergleich zu Placebo. Die Anzahl korrekter Antworten in der 2-back-Aufgabe zur Evaluation des Arbeitsgedächtnisses war gegenüber der Placebogruppe ebenfalls verschlechtert. In weiteren Testverfahren (MMST, MoCA, Paced Auditory Serial Addition Test, TMT B) konnte kein Gruppenunterschied detektiert werden. Im MRT fand sich für die Behandlung mit Memantin vs. Placebo eine Aktivitätsminderung im rechten Gyrus lingualis und linken Gyrus frontalis superior für die Durchführung der 2-back-Aufgabe versus der 0-back-Aufgabe.

Begründung der Empfehlung

Auf der Basis der oben dargestellten Evidenzgrundlage erfolgt die nachfolgende Empfehlung. Es gibt eine negative RCT-Studie, die allerdings zu wenig Pat. einschloss und nur eine sehr kurze Behandlungsphase inkludierte. Insgesamt besteht für die Anwendung von Memantin aktuell keine ausreichende Evidenz.

Empfehlung	Neu<br>Stand (2023)
Memantin soll nicht für die Therapie einer PD-MCI angewendet werden.	
Konsensstärke: 96,7%, starker Konsens	

Fragestellung 121: Wie effektiv ist Memantin im Vergleich mit Placebo in der Behandlung von kognitiven Defiziten bei PKD?

Hintergrund

Im Durchschnitt beträgt die Prävalenz der PKD circa 24–31% [6], [7]. Nach einer Krankheitsdauer von mehr als 12 bzw. 15 Jahren haben über die Hälfte aller Personen mit PK eine Demenz entwickelt [8], [9]. Der Schweregrad der Einschränkungen ist mit dem Verlust der Selbstständigkeit in den ADL assoziiert [10]. Darüber hinaus stützt das Auftreten von Verhaltensauffälligkeiten (z.B. Halluzinationen, Apathie) die Diagnose einer PKD [10]. Memantin hemmt die (pathologische) Aktivität von NMDA-Rezeptoren.

Evidenzgrundlage

Die Literatursuche in PubMed erbrachte drei Studien. Für die Literaturrecherche wurden folgende Schlagworte verwendet: (Parkinson disease OR Parkinson* OR Parkinson disorders OR Movement Disorders NOT [Wolff parkinson white syndrome OR [wolff* AND parkinson*] OR [wpw AND syndrome OR wpw]) AND (Memantine OR memantine) AND (placebo) AND (cognitive deficit OR treatment OR therapy OR cognitive dysfunction OR PDD OR Parkinson disease with dementia OR Parkinson disease dementia OR Parkinson dementia) AND (humans NOT animals) AND (2016:2021) AND (english OR german).

Nach Screening der Abstracts und Inhalte wurde 1 Literaturreferenz als relevant für die Leitlinie bewertet. Zusätzlich wurde eine Studie durch eine unsystematische Suche in Pubmed selektiert, welche in diese Leitlinie einbezogen wurde.

Es fand sich:

- 2 Metaanalysen.

Ergebnis

Es wurden zwei systematische Reviews mit Metaanalysen zur Behandlung kognitiver Symptome bei PKD durchgeführt. In einem Cochrane Review mit Metaanalyse wurden drei Studien mit insgesamt 243 randomisierten Personen mit PKD oder Demenz mit Lewy-Körperchen (DLK) für die Beurteilung des Behandlungseffekts mit Memantin nach 24 Wochen herangezogen [100]. In die Studien wurden überwiegend Personen mit mildem Schweregrad der Demenzerkrankung (MMST >19 Punkte) einbezogen. In zwei der im Review berücksichtigten Studien wurde neben Memantin zeitgleich mit Cholinesterasehemmer therapiert. Die Therapie mit Memantin führte zu einer geringen, aber signifikanten Verbesserung im globalen klinischen Eindruck (-0,35; 95% Konfidenzintervall (KI) -0,60

bis -0,09), den kognitiven Funktionen (-1,90, 95% KI -3,73 bis -0,07) und einer leichten Minderung von Verhaltensauffälligkeiten und der Stimmung (-2,18; 95% KI -5.57 bis 1.21), wobei die Evidenz von den Autoren/Autorinnen als gering vertrauenswürdig beurteilt wurde. Verbesserungen in den ADL (-0,27; 95% KI -0,65 bis 0,11) konnten nicht nachgewiesen werden.

In einer weiteren Metaanalyse wurden sechs Studien zur Beurteilung der Effektivität von Memantin auf die Verbesserung der Leistung in verschiedenen kognitiven Domänen einbezogen [102]. Gemittelt über alle Domänen, wurde eine mittlere Effektstärke von d = 0,25 (N = 57, 95% KI 0,14 bis 0,36) in der gemischten Population mit PKD und DLK ermittelt. Die ermittelten Effektstärken waren höher in Studien, welche ein globales kognitives Leistungsmaß (z.B. MMST, MDRS) inkludiert hatten. In Personen mit PKD wurden ebenfalls geringe Effekte für die Behandlung kognitiver Einschränkungen mit Memantin (d = 0,30; 95% KI = 0,09 bis 0,50) ermittelt. Ein Einfluss der Behandlungsdauer auf die Effekte der kognitiven Leistungsfähigkeit konnte nicht gezeigt werden.

Begründung der Empfehlung

Auf der Basis der oben dargestellten Evidenzgrundlage erfolgt nachfolgende Empfehlung. Insgesamt besteht für die Anwendung von Memantin keine ausreichende Evidenz. Limitationen der Studie beziehen sich auf lediglich geringe Effekte, welche durch die Medikation mit Memantin erzielt werden. Darüber hinaus beziehen sich die Berichte der Effekte überwiegend auf eine gemischte Kohorte von Personen mit PKD und DLK.

**Empfehlung**	**Neu**<br>**Stand (2023)**
Memantin soll nicht für die Therapie einer PKD angewandt werden.	
Konsensstärke: 96,6%, starker Konsens	

Fragestellung 122: Wie effektiv sind Acetylcholinesterase-Hemmer (AChE) im Vergleich mit Placebo in der Behandlung von kognitiven Defiziten bei PD-MCI?

Hintergrund

Änderungen in der cholinergen Aktivität sind bereits bei nicht dementen Personen mit PK nachweisbar [103], [104]. Einschränkungen in der Aufmerksamkeit, im Gedächtnis und in den exekutiven Funktionen sind mit der cholinergen Aktivität bei Personen mit PK assoziiert [105]. AChE (Rivastigmin, Donepezil, Galantamin) hemmen die Isoformen der Acetylcholinesterase und damit den Abbau von Acetylcholin. Dies verlängert die postsynaptische Rezeptorstimulation. Rivastigmin hemmt zusätzlich die Butyrylcholinesterase, die ebenfalls Acetylcholin inaktiviert.

Evidenzgrundlage

Die Literatursuche in Pubmed erbrachte 7 Studien. Für die Literaturrecherche wurden folgende Schlagworte verwendet: (Parkinson disease OR Parkinson* OR Parkinson disorders OR Movement Disorders NOT [Wolff parkinson white syndrome OR wolff* AND parkinson*] OR [wpw AND syndrome] OR wpw) AND (acetylcholinesterase inhibitor OR acetylcholinesterase inhibitor OR AChEI

OR AChE inhibitor) AND (placebo) AND (cognitive deficit OR treatment OR therapy OR cognitive dysfunction OR PD-MCI OR mild cognitive impairment OR mild cognitive impairment) AND (humans NOT animals) AND (2016:2021) AND (english OR german).

Nach Screening der Abstracts und Inhalte wurde 2 Literaturreferenzen als relevant für die Leitlinie bewertet. Zusätzlich wurde zwei Studien aus anderen Quellen selektiert, welche in diese Leitlinie einbezogen wurden.

Es fanden sich:

- 3 RCT
- 1 nicht randomisierte Open-label Studie.

Ergebnis

Rivastigmin

Es wurden eine RCT zur Therapie von Personen mit PD-MCI mit Rivastigmin [106] und eine nicht randomisierte Open-label-Studie [107] zum Effektivitätsnachweis von Donepezil durchgeführt.

Die Effektivität von Rivastigmin als transdermales Pflaster zur Verbesserung der globalen kognitiven Leistung bei Personen mit PD-MCI wurde in einem RCT mit Crossover-Design durchgeführt [106]. Randomisiert wurden 28 Personen mit PD-MCI auf die Gabe von Rivastigmin (n = 14) für 10 Wochen (max. Dosis 9,5/24 Stunden mg) versus Placebo (n = 14, Phase 1). Nach einer Behandlungspause von 4 Wochen erhielten Personen, welche im ersten Durchgang Rivastigmin bekommen hatten, nun das Placebo und vice versa. Für den primären Endpunkt ergab sich lediglich ein Trend für eine Verbesserung im klinischen Eindruck. Bezogen auf die sekundären Outcomes, ergab sich keine statistisch bedeutsame Verbesserung in den kognitiven Skalen, im Verhalten und im Schweregrad psychiatrischer Begleiterkrankungen, der Lebensqualität oder in der Alltagskompetenz.

Donepezil

In einer Open-label-Studie wurden 80 Personen mit PD-MCI auf die Gabe von Donepezil (5 oder 10 mg/Tag) oder Placebo für 48 Wochen im Verhältnis 1 : 1 randomisiert [107]. Es zeigte sich keine Änderung im MMST, dem MoCA, im klinischen globalen Eindruck, in der Clinical-Dementia-Rating Scale und der Motorik. Eine Netzwerkanalyse im Beta-2-Band in einer Elektroenzephalographie zeigt eine signifikante Erhöhung der Aktivierung unter Behandlung von Donepezil vs. Placebo nach 48 Wochen, bezogen auf den rechten parahippocampalen Kortex, den rechten entorhinalen Kortex und den rechten temporalen Pol.

In einer gemischten Kohorte von nicht dementen Personen ohne Differenzierung von PD-MCI wurden zwei doppelblinde RCTs zur Beurteilung der Effektivität von Donepezil durchgeführt [108], [109].

In der Studie von Sawada et al. (2018) wurden 145 nicht demente Personen (MMST-Wert > 24) randomisiert, 72 Personen wurden mit Donepezil (5 mg/Tag) und 73 Personen mit Placebo behandelt [108]. Veränderungen im MMST, in der FAB und im Wechsler-Gedächtnistest wurden als sekundäre

Endpunkte definiert. Primärer Endpunkt war die Prävention einer Psychose, wobei das Auftreten einer Psychose unter Gabe von Donepezil und Placebo vergleichbar hoch war. Nach 96 Wochen zeigte sich unter Donepezil eine stabile Leistung im MMST im Vergleich zur Placebogruppe, welche sich im MMST verschlechterte.

Die Leistung in der Behandlungsgruppe war im Abschnitt zum auditorischen Gedächtnistest im Wechsler-Gedächtnistest im Vergleich zur Placebogruppe ebenfalls verbessert. Keine Effekte ergaben sich für die Subaufgaben zum visuellen Gedächtnis und zur Aufmerksamkeit aus dem Wechsler-Gedächtnistest sowie der FAB [108]. Im Vergleich zur Placebogruppe traten in der Gruppe mit Donepezil weniger häufig auditorische Halluzinationen und Nasopharyngitis (kombinierte Entzündungen der Nase und des Rachens) auf.

In einer weiteren Studie von Sawada et al. (2020), welche nach Einschätzung der Autoren/Autorinnen eine Folgeuntersuchung mit einer Teilkohorte darstellt, wurde die Effektivität von Donepezil (n=48) versus Placebo (n= 50) nach 96 Wochen beurteilt [109]. In einer Anschlussphase wurden beide Gruppen für weitere 24 Wochen mit 5 mg/Tag Donepezil behandelt. Die Veränderung im MMST und in der FAB unterschied sich nicht zwischen den 22 Personen mit Gabe von Donepezil für 120 Wochen und den 21 Personen, welche nach 96 Wochen von der Behandlung mit Placebo auf Donepezil umgestellt wurden. Somit wurde das primäre Studienziel nicht erreicht. Die Leistung im Wechsler-Gedächtnistest – ein sekundärer Endpunkt – war verbessert in der Gruppe mit längerer Gabe von Donepezil, ebenso der Schweregrad der Parkinson-Symptome, erfasst mit dem UPDRS Part III (kein Studienendpunkt). Als häufigste Nebenwirkung traten Halluzinationen und Wahnvorstellungen auf, wobei die Auftretensrate höher war in der Gruppe, welche von Beginn an mit Donpezil für 120 Wochen behandelt wurde, im Vergleich zu Personen, welche von Placebo auf Donepezil umgestellt wurden.

Galantamin

Für Galantamin wurden keine Studien zur Behandlung von Personen mit PD-MCI identifiziert.

Begründung der Empfehlung

Auf der Basis der oben dargestellten Evidenzgrundlage erfolgt die nachfolgende Empfehlung. Insgesamt besteht für die Anwendung von Donepezil, Rivastigmin und Galantamin aktuell keine ausreichende Evidenz. Die Verbesserung der Kognition stellt in Studien zur Evaluation der Wirksamkeit von Donepezil lediglich den sekundären Endpunkt dar. Die Gabe von Donepezil führte nicht zu einer Verbesserung im globalen kognitiven Leistungsniveau, lediglich in kognitiven Teilfunktionen.

**Empfehlung**	**Neu**<br>**Stand (2023)**
Rivastigmin, Donepezil und Galantamin sollen nicht zur Behandlung einer PD-MCI eingesetzt werden.	
Konsensstärke: 89,7%, Konsens	

Fragestellung 123: Wie effektiv sind Acetylcholinesterase-Hemmer im Vergleich mit Placebo in der Behandlung von kognitiven Defiziten bei PKD?

Hintergrund

Eine verminderte cholinerge Aktivität ist bei Pat. mit PKD nachgewiesen [110], [111]. Acetylcholinesterase-Hemmer (Rivastigmin, Donepezil, Galantamin) hemmen die Isoformen der Acetylcholinesterase und damit den Abbau von Acetylcholin. Dies verlängert die postsynaptische Rezeptorstimulation. Rivastigmin hemmt zusätzlich die Butyrylcholinesterase, die ebenfalls Acetylcholin inaktiviert.

Evidenzgrundlage

Die Literatursuche in Pubmed erbrachte 11 Studien. Für die Literaturrecherche wurden folgende Schlagworte verwendet: (Parkinson disease OR Parkinson* OR Parkinson disorders OR Movement Disorders NOT [Wolff parkinson white syndrome OR wolff* AND parkinson*] OR [wpw AND syndrome] OR wpw) AND (acetylcholinesterase inhibitor OR acetylcholinesterase inhibitor OR AChEI OR AChE inhibitor) AND (placebo) AND (cognitive deficit OR treatment OR therapy OR cognitive dysfunction OR PDD OR Parkinson's disease dementia OR Parkinson dementia) AND (humans NOT animals) AND (2016:2021) AND (english OR german). Nach Screening der Abstracts und Inhalte wurde keine Literaturreferenz als relevant für die Leitlinie bewertet. Zusätzlich wurden 4 Studien aus anderen Quellen selektiert, welche in diese Leitlinie einbezogen wurden.

Es fanden sich:

- 3 Metaanalysen.

Ergebnis

In einem Cochrane Review (2012) wurden in sechs RCT-Studien die Effekte verschiedener Acetylcholinesterase-Hemmer bei DLK, PKD und PD-MCI metaanalytisch betrachtet. In vier Studien wurden Personen mit PKD eingeschlossen, in einer Studie Personen sowohl mit PKD als auch mit DLK rekrutiert und eine Studie analysierte Personen mit DLK [112]. Es zeigten sich insgesamt positive Effekte für die Gabe von Acetylcholinesterase-Hemmern hinsichtlich der globalen Beeinträchtigung (ADCS-CGIC) und bei gepoolter Analyse des MMST- und der Alzheimer Disease Assessment Scale (Abschnitt zur Kognition, ADAS-Cog)-Daten bei PKD und DLK. Bei getrennter Analyse für Rivastigmin und Donepezil bestanden diese Effekte fort. Der Einfluss der Acetylcholinesterase-Hemmer auf neuropsychiatrische Symptome wurde in den verschiedenen Studien mit unterschiedlichen Skalen gemessen, war insgesamt weniger robust als die Effekte auf die Kognition und nur für Rivastigmin, nicht aber für Donepezil signifikant. Nebenwirkungen führten bei Rivastigmin – häufiger als bei Donepezil-Medikation – zum Abbruch der Therapie. Allerdings wurde in den Studien nur die Tabletten- und nicht die (nebenwirkungsärmere) Pflasterapplikation von Rivastigmin verwendet. Unter der Medikation wurde eine Verstärkung der Parkinson-Symptome und insbesondere des Tremors dokumentiert. Dieses führte aber nicht zu einer Beeinträchtigung der UPDRS-Werte Part III (Motorik) oder des Gesamtwerts der UPDRS.

Eine weitere Metaanalyse zur Effektivitätsanalyse von Acetylcholinesterase-Hemmern bei PKD inkludierte eine RCT zur Effektivitätsbeurteilung von Rivastigmin versus Placebo, drei RCTs zum Vergleich der Gabe von Donepezil versus Placebo, jedoch keine RCT zu Galantamin [113]. Die gepoolte Analyse zeigte, dass die Gabe von Acetylcholinesterase-Hemmern der Abnahme im globalen kognitiven Leistungsniveau (MMST), im Gedächtnis und der Sprache (ADAS-cog) entgegenwirken kann. Die kognitiven Testdaten wurden durch eine gebesserte klinische Symptomatik (Clinician's Global Impression of Change) in der Donepezil-versus-Placebo-Gruppe gestützt. Ebenfalls zeigten sich Verbesserungen für den Schweregrad der psychiatrischen Begleitsymptomatik, gemessen mit dem Neuropsychiatrischen Inventar (NPI). Jedoch waren die Tremor-Rate und die Anzahl unerwünschter Nebenwirkungen unter Behandlung mit Acetylcholinesterase-Hemmern im Vergleich zur Gabe von Placebo erhöht, das Risiko für Stürze und der Schweregrad der Parkinson-Symptome hingegen nicht. Separate Analysen für Rivastigmin und Donepezil bestätigen den positiven Therapieeffekt beider Wirkstoffe auf die kognitiven Funktionen und den klinischen Eindruck ohne Auswirkungen auf die Sturzereignisse. Eine Besserung der psychiatrischen Begleitsymptome, also eine Verringerung der NPI-Werte, konnte unter der Gabe von Donepezil nicht belegt werden, jedoch auch keine gesteigerte Rate unerwünschter Nebenwirkungen und eine Verstärkung von Tremor. Effektivitätsbeurteilungen von Acetylcholinesterase-Hemmern bei Personen mit DLK und PK bestätigten ebenfalls, dass Rivastigmin und Donepezil die kognitive Leistung verbessern können, die Behandlung mit Galantamin versus Placebo aber nicht [114]. Die Studien wiesen aufgrund der breit angelegten Einschlusskriterien eine große Heterogenität auf, mit Auswirkungen des Studiendesigns (doppelblinde versus unverblindete RCTs oder placebokontrolliert versus nicht placebokontrolliert), Art des AChE (Rivastigmin, Donepezil und Galantamin) und Stichprobengröße auf die Interpretation der Studienergebnisse. Die Therapie mit Rivastigmin ist für die Therapie von Verhaltensauffällig-keiten wirksamer als die Gabe von Donepezil oder Galantamin, wobei auch hier die Heterogenität der Ergebnisse verschiedener Studien sehr groß war. Die Gabe von Donepezil verbesserte die Alltagsaktivität versus Placebo, eine metaanalytische Beurteilung von Rivastigmin zur Verbesserung der Alltagskompetenz konnte aufgrund des Vorliegens von nur einer Studie nicht erfolgen.

Begründung der Empfehlung

Auf der Basis der oben dargestellten Evidenzgrundlage erfolgt die nachfolgende Empfehlung. Die Empfehlung zur Behandlung kognitiver Symptome bei PKD mit Donepezil ist aufgrund der fehlenden Zulassung durch die Europäische Arzneimittel-Agentur eingeschränkt. Für die Behandlung der PKD mit Galantamin besteht keine ausreichende Datenlage.

**Empfehlung**	**Modifiziert**<br>**Stand (2023)**
▪ Rivastigmin soll zur Behandlung der PKD eingesetzt werden.<br>▪ Donepezil kann zur Behandlung der PKD eingesetzt werden. Dabei handelt es sich um einen Off-label Use.<br>▪ Galantamin sollte nicht zur Behandlung einer PKD eingesetzt werden.	
Konsensstärke: 96,6%, starker Konsens	

Fragestellung 124: Wie effektiv sind Nahrungsergänzungsmittel und pflanzliche Therapeutika im Vergleich mit Placebo in der Behandlung von kognitiven Defiziten bei PD-MCI?

Hintergrund

Etwa 50% aller Parkinson-Pat. nehmen Nahrungsergänzungsmittel ein [115]. Diese betreffen zu 71% eine Vitamin-D-Substitution, 44% Vitamin B12, 38% Vitamin C und 38% Fischöl (Omega-3-Fettsäuren) [115]. Viele Pat. wünschen sich, durch den Einsatz von Nahrungsergänzungsmitteln und pflanzlichen Therapeutika, Maßnahmen an der Hand zu haben, welche im Gegensatz zu der symptomatischen Therapie einen präventiven oder erkrankungsmodifizierenden Effekt bringen.

Evidenzgrundlage

Die Literatursuche in Pubmed erzielte 370 Treffer. Für die Literaturrecherche wurden folgende Schlagworte verwendet: (Parkinson) AND (Nahrungsergänzungsmittel OR nutraceutical OR dietary supplements OR medical food OR medicinal herbs OR synbiotics OR probiotics OR plant extracts) AND (executive function OR memory OR cognition OR dementia OR attention OR concentration OR language) AND (not animal). Plural und Umstellungen im Fachterminus wurden berücksichtigt. 8 Studien wurden als relevant für die Fragestellung identifiziert. Die Mehrheit der Treffer beschäftigt sich mit dem potenziellen Nutzen von Nahrungsergänzungsmitteln, es existieren keine verblindeten, placebokontrollierten Studien. Ausgeschlossen wurden Studien, welche in ihren Prüfmedikamenten von Inhaltsstoffen tierischen oder menschlichen Ursprungs berichteten.

Ergebnis

Es gibt eine große Anzahl von Übersichtsartikeln und Expertenmeinungen, welche den potenziellen Nutzen von Nahrungsergänzungsmitteln und pflanzlichen Therapeutika auf kognitive Störungen bei PK beschreiben [116]. Die klinische Prüfung ist hier sehr viel seltener.

Vitamin D

Einige Studien berichten von einer geringeren Vitamin-D (25-hydroxyvitamin D)-Konzentration bei Personen mit PK [117]. Die verringerte Vitamin-D-Konzentration war in einer Studie mit der Schwere der Erkrankung, aber auch mit dem Schweregrad kognitiver Defiziten verbunden [118]. Personen mit PK und einem höheren Vitamin-D-Spiegel zeigten hingegen verbesserte Leistungen in der verbalen Flüssigkeit und bessere Gedächtnisleistungen [119]. Kontrollierte Studien über den Einfluss einer Vitamin-D-Substitution zur Therapie kognitiver Defizite wurden nicht gefunden.

Vitamin B6

Ähnlich verhält es sich mit der Vitamin-B6-Konzentration, bei der eine inverse Korrelation zum Auftreten der PK besteht, jedoch keine Substitutionstherapiestudien existieren [120].

Spezifische Ernährungsansätze

Eine Studie berichtet einen milden positiven Effekt auf kognitive Leistungen einer ketogenen Diät im Vergleich zu einer fettarmen Diät [121]. Eine weitere Studie an 80 Personen mit PK randomisierte die Hälfte in eine mediterrane Diät, die andere Hälfte erhielt eine landesübliche (indische) Kost. Nach 10

Wochen zeigte sich eine leicht verbesserte kognitive Leistung (im Bereich der exekutiven Funktionen, Aufmerksamkeit und Sprache) in der Gruppe mit mediterraner Diät [122].

Kreatin und Coenzym Q10

Eine chinesische Studie randomisierte 75 Personen mit PK in eine Therapiegruppe mit 38 Pat., die eine Behandlung mit Kreatin und Coenzym Q10 über 18 Monate erhielten. Sowohl motorische als auch kognitive Parameter wurden unkorrigiert für multiple Tests ausgewertet und es zeigte sich eine statistisch signifikant geringere Leistungsabnahme im MoCA-Test nach 12 und 18 Monaten [123].

Koffein

Widersprüchlich ist die Datenlage zum Kaffeekonsum mit positiven und auch negativen Effekten für kognitive Variablen wie den MoCA-Test und den MMST [124], [125].

Begründung der Empfehlung

Auf der Basis der oben dargestellten Evidenzgrundlage erfolgt die nachfolgende Empfehlung. Insgesamt besteht für die Anwendung von Nahrungsergänzungsmitteln oder pflanzlichen Präparaten zur Therapie des PD-MCI keine ausreichende Evidenz.

**Empfehlung**	**Neu**<br>**Stand (2023)**
Nahrungsergänzungsmittel und pflanzliche Therapeutika sollen zur Behandlung von kognitiven Defiziten bei PD-MCI nicht eingesetzt werden.	
Konsensstärke: 96,6%, starker Konsens	

Fragestellung 125: Wie effektiv sind Nahrungsergänzungsmittel und pflanzliche Therapeutika im Vergleich mit Placebo in der Behandlung von kognitiven Defiziten bei PKD?

Hintergrund

Die Effizienz der Pharmakotherapie zur Behandlung einer PKD ist begrenzt. Die Suche nach alternativen Präparaten wie Nahrungsergänzungsmitteln und pflanzlichen Therapeutika ist groß. Der Markt für Nahrungsergänzungsmittel hat in Deutschland ein Volumen von 1,2 Milliarden Euro [126].

Evidenzgrundlage

Die Literatursuche in Pubmed erzielte 370 Treffer. Für die Literaturrecherche wurden folgende Schlagworte verwendet: (Parkinson) AND (Nahrungsergänzungsmittel OR nutraceutical OR dietary supplements OR medical food OR, medicinal herbs OR, synbiotics OR, probiotics OR plant extracts) AND (executive function OR memory OR cognition OR dementia OR attention OR concentration OR language) AND (not animal). Plural und Umstellungen im Fachterminus wurden berücksichtigt. Nach Screening wurde anhand des Titels und des Abstracts und im relevanten Fall auch nach Volltextsichtung bewertet. Eine Studie wurde als relevant identifiziert. Die Mehrheit der Treffer beschäftigt sich mit dem potenziellen Nutzen von Nahrungsergänzungsmitteln, es existiert eine

verblindete, aber nicht placebokontrollierte Studie. Es wurden Studien ausgeschlossen, welche in Ihren Prüfmedikamenten von Inhaltsstoffen tierischen oder menschlichen Ursprungs berichteten.

Ergebnis

Eine chinesische Studie zeigte eine leichte Verbesserung im MMST und in der Dementia Rating Scale (Sum of Boxes) nach einer 6-monatigen Behandlung mit Donepezil einmal täglich in Kombination mit dl-3n-butylphthalidine, Oxiracetam und EGb761 dreimal täglich versus die alleinige Gabe von Donepezil. Die Studie war nicht placebokontrolliert und so ergab sich in der Experimentalgruppe (EG) eine zusätzliche Medikation (dreimal am Tag) mit dem Studienpräparat [127].

Insgesamt fand sich eine große Anzahl von Übersichtsartikeln und Expertenmeinungen, welche den potenziellen Nutzen von Nahrungsergänzungsmitteln und pflanzlichen Therapeutika auf kognitive Störungen bei PK beschreiben [116].

Begründung der Empfehlung

Die hier dargestellte Zusammenfassung stellt fest, dass Nahrungsergänzungsmittel oder pflanzliche Präparate zur Therapie des PKD nicht ausreichend geprüft sind, es gibt keine RCTs.

**Empfehlung**	**Neu**<br>**Stand (2023)**
Nahrungsergänzungsmittel und pflanzliche Therapeutika sollen zur Behandlung von kognitiven Defiziten bei der PKD nicht eingesetzt werden.	
Konsensstärke: 96,6%, starker Konsens	

Fragestellung 126: Wie effektiv sind kognitive Interventionen im Vergleich mit aktiven und passiven Kontrollgruppen in der Behandlung von kognitiven Defiziten bei PD-MCI?

Hintergrund

Angesichts fehlender Pharmakotherapie für Personen mit leichter kognitiver Störung bei PK gelten nicht pharmakologische Interventionen und v.a. kognitive Interventionen als vielversprechende Therapieansätze. Es lassen sich verschiedene Formen kognitiver Interventionen unterscheiden: kognitives Training, kognitive Rehabilitation und kognitive Stimulation. Für PD-MCI erscheinen vom Schwierigkeitsgrad bzw. von der Zielsetzung her kognitives Training und die kognitive Rehabilitation besonders geeignet. Kognitive Stimulation ist dagegen eher bei weiter fortgeschrittenen kognitiven Beeinträchtigungen bzw. Demenz indiziert (vgl. Fragestellung 127).

Kognitives Training beinhaltet den Einsatz standardisierter Papier-und-Bleistift- oder digitaler Aufgaben (z.B. mit Laptop, Tablet oder Smartphone), die direkt auf das Training bestimmter kognitiver Funktionen (z.B. exekutive Funktionen, Aufmerksamkeit, Gedächtnis) abzielen [128]. Häufig ist eine Anpassung des Schwierigkeitsgrads der Übungen an das individuelle kognitive Niveau möglich. Manche Programme enthalten auch psychoedukative Anteile. Kognitives Training wird in

der Praxis als Einzel- oder Gruppentherapie angeboten. Ein häusliches Eigentraining ist ebenfalls möglich.

Kognitive Rehabilitation stellt einen individualisierten Ansatz dar, der in der Praxis als Einzeltherapie angeboten wird. Gemeinsam mit den Personen mit PD-MCI und ihren Angehörigen werden individuelle alltagsbezogene Ziele für die kognitive Rehabilitation definiert. Psychoedukation ist häufig Bestandteil der kognitiven Rehabilitation [128].

Evidenzgrundlage

Die initiale Literatursuche in Pubmed erbrachte 17 Studien. Für die Literaturrecherche wurden folgende Schlagworte verwendet: (Parkinson disease OR Parkinson* OR Parkinson disorders OR Movement Disorders NOT [Wolff parkinson white syndrome OR [wolff* AND parkinson*] OR [wpw AND syndrome OR wpw]) AND (cognitive training OR cognitive interventions OR cognitive stimulation OR reminiscence therapy OR reality orientation OR Reality Therapy) AND (comparable OR similar OR analogous) AND (intervention OR controlled intervention OR control intervention OR wait list OR waiting list OR controls OR control group) AND (cognitive deficits OR treatment OR therapy OR cognitive dysfunction OR PD-MCI OR mild cognitive impairment OR mild cognitive impairment) AND (humans NOT animals) AND (2016:2021) AND (english OR german). Da den Autoren/Autorinnen weitere Studien bekannt waren, erfolgte eine ergänzende Literaturrecherche in Pubmed mit den folgenden Suchbegriffen: „Parkinson“ AND „mild cognitive impairment“ AND „cognitive training“. Zusätzlich wurden die Studien, die in systematische Reviews und Metaanalysen aus dem Bereich „Parkinson“ und „kognitive Interventionen“ inkludiert worden waren [129], [90], [130], [85], [131], berücksichtigt.

Es fanden sich:

- 13 randomisierte, kontrollierte Studien.

Studien, die „nicht demente“ Personen mit PK eingeschlossen hatten, zu denen sowohl Personen mit und ohne PD-MCI zählen können, wurden ausgeschlossen, sofern die Ergebnisse der Personen mit PD-MCI nicht separat ausgewiesen wurden. Ebenso wurden Studien ausgeschlossen, die „kognitiv beeinträchtigte“ Personen nur als Gesamtgruppe analysiert und damit potenziell sowohl Personen mit PD-MCI als auch mit Demenz inkludiert hatten.

Ergebnis

Insgesamt wurden in den letzten ca. 10 Jahren einige Studien zum Thema veröffentlicht, sodass zunehmend Evidenz verfügbar ist. Allerdings müssen einige Limitationen bei der Interpretation bzw. bei den Schlussfolgerungen aus den Studien berücksichtigt werden. Hierzu gehören (i) kleine Stichprobengrößen, (ii) unterschiedliche Operationalisierungen von PD-MCI (z.B. mit oder ohne Verwendung der MDS-Kriterien für die Diagnose einer PD-MCI [132]; unterschiedliche Testbatterien als Basis), (iii) eine fehlende Berücksichtigung des Krankheitsschweregrads bei der Analyse der Trainingseffekte sowie (iv) unterschiedliche und teilweise auch nicht eindeutig definierte Endpunkte. (v) Zudem sind die verwendeten Trainings heterogen (d.h. digitale sowie Papier-und-Bleistift-

Trainings, Einzel- und Gruppentrainings, unterschiedliche Längen und Intensitäten des Trainings). Die berücksichtigten RCTs werden im Folgenden beschrieben.

Studien mit „klassischen" digitalen Trainings

In einer RCT mit 17 Personen mit PD-MCI in der EG und 14 Personen mit PD-MCI in der KG wurde computerisiertes kognitives Training mit dem „CoRe"-Tool mit einer unspezifischen Scheinintervention verglichen. Die Interventionen wurden jeweils dreimal wöchentlich à 45 Minuten über vier Wochen angeboten [133]. Es wurde eine neuropsychologische Testbatterie zur Baseline vor Intervention, nach dem vierwöchigen Training und nach 6 Monaten durchgeführt. Nach 4 Wochen Intervention zeigte sich die EG gegenüber der KG hinsichtlich verschiedener Parameter, u.a. in der globalen Kognition, dem Gedächtnis und in exekutiven Funktionen, überlegen. Diese Überlegenheit der EG zeigte sich auch nach 6 Monaten in einzelnen Parametern dieser Domänen.

In der RCT von Bernini et al. [134] wurde digitales kognitives Training mit dem „CoRe"-Tool (EG1, n=21) mit einem kognitiven Papier-und-Bleistift-Training (EG2, n=14) sowie einer weiteren aktiven KG mit unstrukturierten Aktivitäten verglichen (KG, n=18). Die Trainings wurden viermal wöchentlich à 45 Minuten für drei Wochen durchgeführt. EG1 zeigte sich beiden anderen Gruppen gegenüber im Bereich der globalen Kognition überlegen. Beide EGs zeigten sich in Effekten auf die Aufmerksamkeit der KG überlegen.

DeLuca et al. [135] verglichen in einer RCT bei Personen mit PD-MCI digitales kognitives Training mit der „ERICA"-Plattform (EG, n=30) mit analogem kognitivem Training im Einzelsetting (n=30, KG). Die Intervention wurde dreimal wöchentlich für je 60 Minuten über 8 Wochen durchgeführt. Beide Gruppen zeigten signifikante Verbesserungen kognitiver Funktionen; im Vergleich zeigte sich eine Überlegenheit des digitalen Trainings in Parametern der Aufmerksamkeit, der Orientierung sowie visuokognitiven Leistungen.

In der RCT von Giguère-Rancourt et al. [136] wurde ein digital gestütztes metakognitives Training, d.h. ein für Personen mit PD-MCI angepasstes „Goal Management Training" (EG, n=6), mit einer Achtsamkeitsintervention (KG, n=6) verglichen; beide Interventionen inkludierten 5 wöchentliche Sitzungen à 60–90 Minuten. Die Personen mit PD-MCI wurden zur Baseline und nach der Intervention sowie 4 und 12 Wochen nach der Intervention untersucht. Primäre Endpunkte waren exekutive Skalen. Für die EG und die KG zeigten sich kurzfristige Verbesserungen sowohl in der Selbst- als auch in der Fremdeinschätzung exekutiver Alltagsleistungen sowie in der Fehlerrate bei der Durchführung der „Zoo Map"-Aufgabe aus der Behavioral-Assessment-of-Dysexecutive-Syndrome-Testbatterie; allerdings gab es keinen Unterschied zwischen den beiden Gruppen.

In der RCT von Lawrence et al. [130] wurden sechs Gruppen verglichen, wovon drei für die hiesige Fragestellung relevant sind: ein digitales, auf das individuelle kognitive Profil der Personen mit PD-MCI zugeschnittenes kognitives Training mit der Plattform Smartbrain Pro (EG1, n=7), ein digitales, jedoch vorstrukturiertes kognitives Training mit der gleichen Plattform (EG2, n=7) und eine passive KG (n=7). Für EG1 wurde eine signifikante Verbesserung im Arbeitsgedächtnis berichtet; für EG2 zeigte sich ein Benefit in den Gedächtnisleistungen; Interaktionseffekte können auf Basis der berichteten Ergebnisse nicht identifiziert werden.

París et al. [137] verglichen in einer RCT digitales kognitives Training, kombiniert mit Papier-und-Bleistift-Übungen (EG, n=18), mit Sprechtherapie (KG, n=15). Die Trainings fanden dreimal wöchentlich mit 45-minütigen Sitzungen über 4 Wochen statt. Die Interventionen wurden um ein supervidiertes häusliches Training einmal wöchentlich ergänzt. Es wurde eine umfangreiche neuropsychologische Testbatterie unmittelbar vor sowie nach Interventionsende durchgeführt. Die EG zeigte sich im Vergleich zur KG in mehreren kognitiven Domänen (d.h. exekutive Funktionen, Aufmerksamkeit, Verarbeitungsgeschwindigkeit, Gedächtnis und Visuokognition) überlegen.

In der RCT von Vlagsma et al. [138] wurde ein analoges Exekutivtraining („ReSET: Strategic Executive Treatment“) (EG1, n=28) mit einem digitalen Aufmerksamkeitstraining mit dem Programm „Cogniplus“ (EG2, n=21) verglichen. Beide Interventionen beinhalteten insgesamt 14 einstündige Sitzungen, die ein- bis zweimal wöchentlich angeboten wurden. Personen mit PD-MCI wurden ausführlich zur Baseline vor der Intervention (T0), nach der Intervention (T1) sowie drei bis fünf Monate nach Interventionsende (T2) neuropsychologisch untersucht. In beiden Gruppen waren Verbesserungen sowohl in einer Selbsteinschätzungsskala zu exekutiven Alltagsleistungen als hinsichtlich selbst definierter individueller Therapieziele von T1 nach T2 festzustellen; die Effekte waren jedoch signifikant stärker in EG1. Beide Endpunkte verbesserten sich auch zum Follow-up nach drei bis fünf Monaten (T2); allerdings lagen hierbei keine Unterschiede zwischen den Gruppen vor. Effekte in kognitiven Tests wurden von T0 bis T1 nicht beobachtet; von T0 bis T2 gab es einen signifikanten Interaktionseffekt in einem Exekutivtest zugunsten der EG1.

Studien mit digitalen Trainings mit Gamification-Ansätzen bzw. virtueller Realität

In einer RCT von Maggio et al. [139] wurde ein kognitives Training mit virtueller Realität („BTS Nirvana-System“; EG1, n=10) mit klassischem kognitivem Training im Einzelsetting (EG2, n=10) verglichen. Die Trainings fanden dreimal wöchentlich für je 60 Minuten über acht Wochen statt. Vor und nach dem Training wurde eine umfangreiche neuropsychologische Testbatterie durchgeführt. Es zeigte sich eine Überlegenheit der EG1 im Vergleich zur EG2 in der globalen Kognition sowie den Domänen exekutive Funktionen sowie Aufmerksamkeit.

Die RCT von van de Weijer et al. [140] untersuchte Effekte eines häuslichen kognitiven Trainings mit Gamification-Ansatz („AquaSnap™“) (n=21), das dreimal wöchentlich für 30 Minuten über 12 Wochen angeboten wurde, im Vergleich zu einer passiven KG (n=20). Eine neuropsychologische Testbatterie wurde zur Baseline vor Intervention (T0), nach dem Training (T1) und 12 Wochen nach Interventionsende (T2) durchgeführt. Von T0 nach T2 ergab sich ein Effekt zugunsten der EG in der globalen Kognition.

Studien mit ausschließlich analogen Trainings

Sousa et al. [141] verglichen Effekte eines zusätzlichen kognitiven Papier-und-Bleistift-Trainings (EG, n=24) im Vergleich zu einem allgemeinen Rehabilitationsprogramm ohne zusätzliches kognitives Training (KG, n=15). Das kognitive Training wurde zweimal wöchentlich für 120 Minuten über 4 Wochen durchgeführt. Es zeigte sich eine Überlegenheit der EG im Vergleich zur KG von der Baseline- zur Post-Testung in den Domänen globale Kognition, exekutive Funktionen sowie Visuokognition.

Angelucci et al. [142] verglichen in ihrer RCT kognitives Training mit Fokus auf Set-Shifting (d.h. exekutiven)-Fähigkeiten (EG, n=7) mit einem Kontrolltraining, in dem einfache kognitive Übungen zur Daueraufmerksamkeit und Sprache durchgeführt wurden (KG, n=8). Beide Trainings wurden dreimal pro Woche für je 45 Minuten über vier Wochen durchgeführt. Die Studie zeigte einen Benefit des Set-Shifting-Trainings auf exekutive Leistungen.

In einer RCT verglichen Costa et al. [143] ein Set-Shifting-Training (EG, n=9) mit einer aktiven KG, die Sprach- und Atemübungen erhielt (n=8). Beide Interventionen wurden dreimal wöchentlich für jeweils 45 Minuten über vier Wochen angeboten. Es erfolgte eine neuropsychologische Testbatterie zur Baseline und nach Interventionsende. Es zeigte sich ein Interaktionseffekt zugunsten der EG im prospektiven Gedächtnis sowie in einer Set-Shifting-Aufgabe als exekutivem Maß.

In ihrer RCT verglichen Kalbe et al. [144], [145] Effekte eines kognitiven Gruppentrainings (EG, n=33) mit denen einer aktiven KG (N=31), die ein physisches Trainingsprogramms leichter Intensität, von dem keine kognitiven Effekte zu erwarten waren, erhielt. Die Interventionen wurden zweimal wöchentlich für je 90 Minuten über 6 Wochen durchgeführt. Vor und nach der Intervention (T0 bzw. T1) und nach einem 6- sowie 12-Monats-Follow-up wurde eine neuropsychologische Testbatterie durchgeführt. Es zeigten sich eine Überlegenheit der EG gegenüber der KG von T0 zu T1 hinsichtlich der Wortflüssigkeit als exekutivem Maß, eine Überlegenheit der KG im Arbeitsgedächtnis sowie eine Überlegenheit der EG in den Gedächtnisleistungen im 6-Monats-Follow-up.

Begründung der Empfehlung

Auf der Basis der oben dargestellten Evidenzgrundlage erfolgt nachfolgende Empfehlung.
Trotz der Heterogenität der Studien (u.a. Art, Intensität und Dauer der Intervention, Art der KG, Endpunkte) und methodisch kritischer Aspekte (u.a. kleine Stichprobengrößen, Fehlen definierter primärer Endpunkte) zeigen sich über die Studien hinweg positive Effekte kognitiver Trainings auf kognitive Leistungen. Dies deckt sich auch mit den Ergebnissen der systematischen Überblicksarbeiten und Metaanalysen zu kognitiven Interventionen bei PK [129], [90], [146], die allerdings auch Studien mit Personen ohne kognitive Störungen bzw. gemischten Kohorten (mit und ohne PD-MCI) inkludiert hatten.

Auch wenn durch die unterschiedlichen Trainingsdauern und -intensitäten in den Studien keine diesbezüglichen klaren Empfehlungen möglich sind, wurden zumeist mehrwöchige Interventionen mit mehreren Trainingseinheiten pro Woche evaluiert. Längerfristiges und regelmäßiges Training ist daher indiziert.

Es gibt Hinweise darauf, dass digitales kognitives Training besonders zielführend sein könnte. Jedoch zeigen auch Papier-und-Bleistift-Trainings und Trainings sowohl in der Gruppen- als auch in der Einzeltherapie positive Effekte. Auch wenn gute, wissenschaftlich basierte Eigentrainings (v.a. digital) verfügbar sind, sollte die Therapie, wenn möglich, supervidiert sein. Grundsätzlich sollte das Training den Präferenzen bzw. Möglichkeiten der Personen mit und ohne PD-MCI entsprechen, um eine hohe Therapieadhärenz zu erreichen. Multidomänentrainings (in denen mehrere kognitive Domänen adressiert werden, z.B. Gedächtnis, exekutive Funktionen, Aufmerksamkeit) erscheinen angesichts der Tatsache, dass bei PD-MCI in der Mehrzahl mehrere kognitive Domänen betroffen sind,

zielführend. Jedoch zeigen auch Studien, die einzelne Domänen trainieren (v.a. exekutive Funktionen), positive Effekte und können daher ebenfalls empfohlen werden. Über Transfereffekte auf nicht trainierte kognitive und nicht kognitive Domänen können mangels Evidenz keine Aussagen getroffen werden.

Empfehlung	Neu<br>Stand (2023)
Kognitives Training sollte bei Personen mit PD-MCI angeboten werden.	
Konsensstärke: 96,6%, starker Konsens	

Wichtige Forschungsfragen

Trotz der über alle Studien hinweg gezeigten positiven Effekte auf unterschiedliche kognitive Leistungen braucht es weitere Forschung. Die folgenden Forschungsfragen erscheinen hierbei besonders wichtig:

- Wie effektiv ist kognitives Training für Personen mit PD-MCI mit unterschiedlichen motorischen Subtypen sowie motorischen Schweregraden?
- Welche kognitive Trainingsart (digital versus Papier und Bleistift; Einzel- versus Gruppentherapie; supervidiertes versus nicht supervidiertes Training; Einsatz von „Booster-Trainings“; Multidomänen- versus Singledomänen-Trainings) zeigt sich überlegen?
- Welche Trainingsintensität geht mit den größtmöglichen Effekten auf die kognitive Leistungsfähigkeit einher?
- Welche Effekte auf nicht kognitive Parameter (z.B. motorische Symptomatik, neuropsychiatrische Symptome, Lebensqualität) sind durch ein kognitives Training bei Personen mit PD-MCI zu erwarten?
- Welche Personen mit PD-MCI mit welchen Charakteristika (u.a. soziodemographisch: Alter, Bildung, Geschlecht; kognitiv: kognitives Gesamtniveau zu Beginn des Trainings, welche Domänen sind wie stark betroffen, fluide Intelligenz; krankheitsspezifisch: Krankheitsdauer- und schwere, dopaminerge Medikation, motorischer Subtyp; psychologisch: Ausmaß der Selbstwirksamkeit; biologisch: Genetik, Hippocampusvolumen) profitieren von welchem Training?
- Können kognitive Interventionen bei Personen mit PK ohne kognitive Störungen zur Vorbeugung der PD-MCI beitragen?

Fragestellung 127: Wie effektiv sind kognitive Interventionen im Vergleich mit aktiven und passiven Kontrollgruppen in der Behandlung von kognitiven Defiziten bei PKD?

Hintergrund

Zur Behandlung kognitiver Störungen bei Personen mit PKD steht Rivastigmin als Pharmakotherapie zur Verfügung (vgl. Fragestellung 123), die Effekte sind allerdings limitiert. In der Therapie kognitiver Störungen bei Demenzen unterschiedlicher Ätiologie stellen kognitive Interventionen eine wichtige Säule in der nicht pharmakologischen Therapie dar. Hierzu gehören kognitive Stimulation, kognitives Training, kognitive Rehabilitation sowie Reminiszenztherapie.

Unter *kognitiver Stimulation* sind Interventionen mit verschiedenen Aktivitäten zu fassen, die darauf abzielen, die kognitiven und sozialen Funktionen indirekt zu aktivieren [131]. Es geht um die globale Stimulation der Fähigkeiten, nicht um ein Training spezifischer kognitiver Funktionen. Meist im Gruppensetting durchgeführt, kommen kognitiv stimulierende Übungen, Spiele und Gesprächsrunden zum Einsatz. Es werden Elemente verschiedener etablierter Techniken und Methoden angewendet: Reminiszenztherapie, Realitätsorientierung, kognitives Training, Musiktherapie, Alltagstraining, Bewegungs- und Entspannungsübungen sowie multisensorische Stimulation.

Kognitives Training beinhaltet den Einsatz standardisierter Papier-und-Bleistift- oder digitaler Aufgaben (z.B. mit Laptop, Tablet oder Smartphone), die direkt auf das Training bestimmter kognitiver Funktionen (z.B. exekutive Funktionen, Aufmerksamkeit, Gedächtnis) abzielen [128]. Häufig ist eine Anpassung des Schwierigkeitsgrads der Übungen an das individuelle kognitive Niveau möglich. Manche Programme enthalten auch psychoedukative Anteile. Kognitives Training wird in der Praxis als Einzel- oder Gruppentherapie angeboten. Ein häusliches Eigentraining ist ebenfalls möglich.

Kognitive Rehabilitation stellt einen individualisierten Ansatz dar, der in der Praxis als Einzeltherapie angeboten wird. Gemeinsam mit Personen mit PKD und ihren Angehörigen werden individuelle alltagsbezogene Ziele für die kognitive Rehabilitation definiert. Psychoedukation ist häufig Bestandteil der kognitiven Rehabilitation [128].

Reminiszenztherapie, auch Biographiearbeit genannt, kann im Einzel- oder Gruppensetting angeboten werden und hat das Ziel, autobiographische Erinnerungen zu stimulieren [147]. Sie stellt damit auch eine Art Gedächtnistraining dar. Es werden eigene Erlebnisse und Erfahrungen thematisiert. Bei Personen mit Demenz sind im Sinne eines ressourcenorientierten Ansatzes oft länger zurückliegende Ereignisse Gegenstand der Therapie, z.B. aus der Jugend und dem jüngeren Erwachsenenalter, die besser abrufbar sind als weniger lang zurückliegende. Hierbei kommen Materialien mit Erinnerungswert zum Einsatz, z.B. Fotos, Gegenstände, Musik oder Zeitungsartikel.

Es sollte berücksichtigt werden, dass kognitive Interventionen nicht immer eindeutig den oben genannten Ansätzen zuzuordnen sind. Insbesondere die Grenzen zwischen kognitivem Training und kognitiver Stimulation sind fließend. Für Personen mit Demenz erscheint mit zunehmendem Demenzgrad kognitives Training weniger zielführend und die kognitive Stimulation als eher indirekter Ansatz zur Förderung der globalen geistigen Leistungsfähigkeit angezeigt.

Evidenzgrundlage

Die initiale Literatursuche in Pubmed erbrachte 31 Studien. Für die Literaturrecherche wurden folgende Schlagworte verwendet: (Parkinson disease OR Parkinson* OR Parkinson disorders OR Movement Disorders NOT [Wolff parkinson white syndrome OR [wolff* AND parkinson*] OR [wpw AND syndrome] OR wpw) AND (cognitive training OR cognitive interventions OR cognitive stimulation OR reminiscence therapy OR reality orientation OR Reality Therapy) AND (comparable OR similar OR analogous) AND (intervention OR controlled intervention OR control intervention OR wait list OR waiting list OR controls OR control group) AND (cognitive deficits OR treatment OR therapy OR cognitive dysfunction OR PDD OR Parkinson disease with dementia OR Parkinson disease dementia OR Parkinson dementia) AND (humans NOT animals) AND (2016:2021) AND (english OR german). Nach Screening der Abstracts und Inhalte wurde keine Literaturreferenz als relevant für die Leitlinie bewertet. Da den Autorinnen/Autoren weitere Studien bekannt waren, erfolgte eine ergänzende Literaturrecherche in PubMed mit den folgenden Suchbegriffen: „Parkinson’s disease dementia“ AND „cognitive stimulation“; „Parkinson’s disease dementia“ AND „cognitive training“; „Parkinson’s disease dementia“ AND „cognitive rehabilitation“; „Parkinson’s disease dementia“ AND „cognitive therapy“; „Parkinson’s disease dementia“ AND „cognitive intervention“; „Parkinson’s disease dementia“ AND „reminiscence“; „Parkinson’s disease dementia“ AND „biography work“. Zusätzlich wurden die Studien, die in systematische Reviews und Metaanalysen aus dem Bereich „Demenz“ und „kognitive Interventionen“ inkludiert worden waren [131], [147], [128], berücksichtigt. In den genannten Reviews konnten keine weiteren Studien zu PKD identifiziert werden. Es wurden ausschließlich Studien berücksichtigt, in denen Personen mit PKD als eigene Studiengruppe analysiert wurden. Entsprechend wurden die verfügbaren Studien, die Mischpopulationen (z.B. PKD und DLK) untersucht und berichtet hatten, exkludiert [148], [149].

Zusätzlich wurde 5 Artikel aus den vorgenannten zusätzlichen Quellen selektiert, welche in diese Leitlinie einbezogen wurden.

Es fanden sich:

- 1 randomisierte, kontrollierte Studie
- 3 Reviews
- 1 S3-Leitlinie zur Diagnostik und Therapie der Demenzen (DGPPN & DGN, 2016).

Ergebnis

Kognitive Stimulation

In den S3-Leitlinien zur Diagnostik und Therapie der Demenzen [150] wird kognitive Stimulation mit dem Empfehlungsgrad B („sollte angeboten werden“) empfohlen. Allerdings wird hierbei nicht zwischen Demenzen unterschiedlicher Ätiologie unterschieden, sodass die spezifische Wirkung bei PDD-Pat. unklar ist. In dem 2023 publizierten Cochrane Review zur Evaluation kognitiver Stimulation bei Demenzen [131] wurden 37 RCTs mit insgesamt 2.766 Personen mit Demenz inkludiert. Die Autorinnen/Autoren kamen zu dem Schluss, dass kognitive Stimulation kleine, kurzfristige positive Effekte auf die Kognition, die Kommunikation, die soziale Interaktion sowie die Lebensqualität und

psychische sowie Verhaltenssymptome bei leichter bis mittelgradiger Demenz hat. Für Personen mit PKD wurde eine Crossover-Pilot-RCT durchgeführt, die die Effekte eines kognitiven Stimulationsprogramms „NEUROvitalis Sinnreich“ (EG, n=12; zweimal wöchentlich à 60 Minuten für acht Wochen) im Vergleich zu einer passiven KG (n=6) untersucht hat [151]. Es zeigte sich trotz der sehr kleinen Stichprobengrößen ein statistischer Trend für eine Überlegenheit der EG gegenüber der KG in der globalen Kognition und in psychischen und Verhaltenssymptomen.

Kognitives Training und kognitive Rehabilitation

Ein Cochrane Review zum Thema kognitives Training bei leichter bis mittelgradiger Demenz unterschiedlicher Ätiologie von 2019 [128] berichtet, dass es Hinweise auf leichte bis moderate positive Effekte auf die globale Kognition und die verbale Flüssigkeit als exekutives bzw. Sprachmaß geben könnte. Bis dato liegen keine Studien zu Effekten kognitiver Trainings bei Personen mit PKD vor. Kognitives Training und kognitive Rehabilitation werden in der S3-Leitlinie [150] mangels positiver Evidenz nicht empfohlen.

Reminiszenztherapie

In der S3-Leitlinie „Demenzen“ [150] wird auch Reminiszenztherapie mit einem Empfehlungsgrad B für Demenzen unterschiedlicher Ätiologie empfohlen. Der 2018 publizierte Cochrane Review [147] mit insgesamt 22 RCTs mit 1.972 Personen mit Demenz unterschiedlicher Ätiologie zeigt positive Effekte auf Kognition, Lebensqualität, Kommunikationsverhalten sowie Stimmung. Allerdings liegt für Personen mit PKD keine spezifische Evidenz vor.

Begründung der Empfehlung

Die Empfehlung zur kognitiven Stimulation beruht vorwiegend auf den Empfehlungen der S3-Leitlinie „Demenzen“ [150] sowie dem Cochrane Review zur kognitiven Stimulation bei Demenzen unterschiedlicher Ätiologie [131]. Die Empfehlung zur Reminiszenztherapie beruht ausschließlich auf den Empfehlungen der S3-Leitlinie „Demenzen“ [150] sowie dem Cochrane Review zur Reminiszenztherapie bei Demenzen unterschiedlicher Ätiologie [147].

**Empfehlung**	**Neu**<br>**Stand (2023)**
▪ Kognitive Stimulation sollte bei Personen mit PKD angeboten werden.<br>▪ Reminiszenztherapie kann bei Personen mit PKD angeboten werden.	
Konsensstärke: 96,8%, starker Konsens	

Wichtige Forschungsfragen

Im Bereich kognitiver Interventionen bei Personen mit PKD besteht dringender Forschungsbedarf. Die folgenden Forschungsfragen erscheinen besonders wichtig:

- Wie effektiv sind kognitive Stimulation, kognitive Rehabilitation und Reminiszenztherapie für Personen mit PKD mit unterschiedlicher Demenzausprägung?

- Ist kognitives Training bei Personen mit PKD mit leichter Demenzausprägung effektiv?
- Welche Effekte auf nicht kognitive Parameter (z.B. motorische Symptomatik, neuropsychiatrische Symptome, Lebensqualität) sind durch die kognitiven Interventionen zu erwarten?
- Welche Parameter (z.B. Alter, kognitives Baseline-Niveau, Krankheitsdauer und -schwere, weitere neuropsychiatrische Symptome) haben einen Einfluss auf die Effekte kognitiver Interventionen bei Personen mit PKD?
- Welche Effekte sind von Kombinationstherapien (z.B. Acetylcholinesterase-Hemmer mit kognitiver Stimulation) zu erwarten?

Fragestellung 128: Wie effektiv sind physische Interventionen (Physiotherapie und z.B. Nordic Walking, aerobes Training, Tanzen etc.) im Vergleich mit einer Vergleichsintervention in der Behandlung von kognitiven Defiziten beim Vorliegen von PD-MCI?

Hintergrund

Ausdauertraining und Sport senken das Risiko für eine kognitive Störung [152] bzw. eine Demenz im Alter [153] bei gesunden Personen. Auch bei Menschen mit PK wurde in zahlreichen Studien nicht nur der Effekt unterschiedlicher physischer Interventionen auf die Motorik, sondern auch auf die Kognition untersucht.

Evidenzgrundlage

Die initiale Literatursuche in Pubmed erbrachte 208 Studien. Für die Literaturrecherche wurden folgende Schlagworte verwendet: (Parkinson) AND (physical exercise OR physical modalities OR physiotherapy OR physical therapy OR exercise movement OR exercise therapy OR physical exercise OR aerobic exercise OR resistance training OR strength training OR weight-lifting OR weight-bearing) AND (cognition OR cognitive function OR executive function OR problem solving OR perception, memory OR attention OR concentration OR learning). Plural und Umstellungen der Fachtermini wurden berücksichtigt. Nach Screening von Titel und des Abstracts wurden 23 Studien als relevant für die Leitlinie bewertet. Studien mit dementen Studienteilnehmenden wurden ausgeschlossen. Nicht berichtet werden die Ergebnisse zum Exergaming, da hier physisches Training mit kognitivem Training kombiniert wird und somit spezifische Effekte auf die kognitive Leistungsfähigkeit nicht ermittelbar sind. Nur eine Studie fokussierte auf Personen ausschließlich mit PD-MCI. Zwei RCTs, ein systematischer Review und eine nicht randomisierte Studie zur Therapie der PD-MC wurden für die Empfehlung herangezogen, Letztere, weil die Studie kognitive Variablen als primären Endpunkt definierte. Zahlreiche Studien definieren kognitive Variablen als sekundäre Endpunkte oder berichten explorativ über mehrere Variablen, die oft keine Korrektur für multiples Testen in den statistischen Analysen beinhalten, was die Aussagekraft der Studien schmälert.

Ergebnisse

In den folgenden Studien wurden als primärer Endpunkt die Auswirkungen physischer Aktivität auf kognitive Variablen untersucht. Die einzige Studie, die PD-MCI als Einschlusskriterium verwendet, randomisiert (1:1) 50 Personen mit PK in eine aktive EG (Ausdauertraining 6-mal 60 Minuten pro

Woche für 4 Wochen) und eine passive KG. Die aktive EG zeigte dabei eine Besserung der globalen Kognition, Aufmerksamkeit und des Arbeitsgedächtnisses nach 4 Wochen gegenüber der passiven KG, wobei die Verbesserung der globalen Kognition noch über 6 Monate lang nachweisbar war [154]. In einer kleinen Studie führten 9 Personen mit PK ohne kognitive Defizite ein Laufbandtraining 3 x 45 Minuten/Woche über 3 Wochen durch und 8 Personen mit PK wurden in eine passive KG randomisiert. Es zeigte sich ein signifikant positiver Effekt des Trainings auf die FAB im Verlauf [155]. Eine weitere RCT schloss 76 Personen mit PK ein und konnte zeigen, dass ein Ausdauertraining der Physiotherapie (Gehen, Stretching, 60 Minuten, beide Trainings dreimal pro Woche über 12 Wochen) im Verlauf hinsichtlich einer Verbesserung exekutiver Funktionen überlegen ist [156]. In einer nicht randomisierten Studie nahmen 10 Personen mit PK an einem Ausdauertraining teil (eine Stunde, dreimal die Woche über 6 Monate), während 10 Personen mit PK als passive KG dienten. Es zeigte sich gegenüber der KG im Verlauf eine signifikante Verbesserung exekutiver Funktionen. Zusammenfassend zeigte zudem ein systematischer Review über RCTs einen Vorteil physischer Aktivität gegenüber passiven KG in den Bereichen globale Kognition, Denkgeschwindigkeit, Aufmerksamkeit und mentale Flexibilität [157].

Die folgenden Studien definierten kognitive Parameter als sekundären Endpunkt oder es handelt sich um explorative Studien mit multiplen Zielparametern.

Zweimal pro Woche eine Stunde Yoga für 12 Wochen führte in einer kleinen Studie (10 Personen mit PK in der EG und 10 in der passiven KG) zu keinen positiven kognitiven Effekten [158]. In einer dreiarmigen Studie wurden 8 Personen mit PK zum Ausdauersport, 8 einem Gruppensportprogramm und 8 einer passiven KG zugeordnet. Die Intervention umfasste eine Stunde Training zweimal pro Woche über 6 Monate und zeigte einen positiven Verlauf des Ausdauertrainings auf Teilbereiche der exekutiven Funktionen im Vergleich zu beiden Komparatoren [159]. Auch die chinesische Bewegungstherapie Wuqinxi zeigte in einer randomisierten Studie (1 : 1) bei insgesamt 30 Personen mit PK nach dem Bewegungstraining (90 Minuten, zweimal die Woche über 12 Wochen) bessere Leistungen in der FAB und im Stroop-Test im Vergleich zu einer passiven KG (33859712). Eine Studie unter Einschluss von 107 Personen mit PK, die auf drei Gruppen ((i) Ausdauer- und Mobilitätstraining, (ii) ein multimodales Training oder (iii) kognitives Training) randomisiert wurden, zeigten in der ersten Gruppe und der dritten Gruppe kognitiv stabile Verläufe im Bereich der exekutiven Funktionen, Aufmerksamkeit und des Arbeitsgedächtnisses nach 4 Monaten, während die zweite Gruppe Verschlechterungen zeigte [160]. In einer Studie unter Einschluss von 57 Personen mit PK, die 1 : 1 auf ein Ausdauertraining oder ein Stretching-Training randomisiert wurden (beide Gruppen trainierten über 45 Minuten, dreimal pro Woche über 6 Monate), fand sich eine im Verlauf gebesserte kognitive Kontrolle (Antisakkaden-Paradigma) in der Ausdauertrainingsgruppe [161]. In einer kleinen Studie zeigte Tangotanzen (18 Tänzer mit zweimal eine Stunde Tango pro Woche über 12 Wochen) keine Besserung in den primären Outcomes (MoCA und globaler klinischer Eindruck) gegenüber 15 Personen, die einer passiven KG zugeordnet waren [162]. Eine Studie zum Folkloretanz hingegen zeigte bessere MoCA-Werte nach der Intervention im Vergleich zu einer passiven KG [163]. Eine weitere Studie randomisierte 39 Personen mit PK (1:1:1) auf die Gruppen Krafttraining, Physiotherapie und passive KG. Die Interventionen erfolgten zweimal pro Woche über 60 Minuten für insgesamt 12 Wochen. Krafttraining führte im Verlauf zu einer Verbesserung im MoCA im

Vergleich zur passiven KG [164]. Von 99 Personen mit PK wurden 50 in eine EG mit einem 10-wöchigen Gleichgewichtstraining randomisiert, 49 wurden in eine KG randomisiert. In einer Subanalyse waren die kognitiven Aspekte einer motorisch-kognitiven Dual-Task-Aufgabe in der Trainingsgruppe im Verlauf besser als in der KG [165]. Eine weitere Studie schloss 44 Personen mit PK ein. Die globale Kognition zeigte sich im Verlauf nach einer intensiven Physiotherapie (viermal pro Woche über 4 Wochen) gegenüber einer regulären Physiotherapie (zweimal pro Woche über 16 Wochen) gebessert, während es keine Unterschiede zur ebenfalls hochfrequent durchgeführten Lee Silverman Voice Treatment (LSVT) BIG-Therapie gab [166]. Ein Agility Boot Camp Training zeigte eine Verbesserung in einer motorisch-kognitiven Dual-task-Aufgabe und in den exekutiven Leistungen im Verlauf gegenüber einer passiven KG. Einschränkend muss eingeräumt werden, dass hier 46 Personen mit PK mit einem Freezing beim Gehen selektiert wurden [167].

Dieser Bericht exkludiert Studien, die ungeeignet waren, weil diese zu wenige Angaben zu der Methodik berichteten [168], [169], [170], [171], [172], [173] oder vorläufige Daten von Machbarkeitsstudien oder Pilotstudien berichteten [174], [175], [176].

Begründung der Empfehlung

Die Evidenz der Empfehlung beruht auf zwei RCTs, einem systematischen Review und einer nicht randomisierten Studie. Da nur eine RCT die in der Fragestellung betrachtete PD-MCI untersuchte, besteht ein Evidenzgrad von 3. Die Dauer, die Art und die Häufigkeit des Ausdauertrainings sind nicht untersucht. Daher handelt es sich um eine Expertenempfehlung.

**Empfehlung**	**Neu**<br>**Stand (2023)**
Ausdauertraining soll bei PD-MCI im aeroben Bereich 2- bis 3-mal 45 bis 60 Minuten pro Woche durchgeführt werden.	
Konsensstärke: 96,8%, starker Konsens	

Wichtige wissenschaftliche Fragestellungen

Folgende Fragestellungen sind zu diesem Thema bislang unbeantwortet und haben große Auswirkung auf die hier getroffenen Empfehlungen:

- Was ist der Nutzen des physischen Trainings auf die Kognition, bezogen auf die Verrichtung der ADL der Personen mit PK?
- Bei PK kommt es zu unterschiedlichen Subformen des PD-MCI. Es existieren PD-MCI mit einem Störungsprofil in einer kognitiven Domaine (z.B. amnestisch oder dysexekutiv) und multiple Domänen in bestimmten Kombinationen [177]. Sollen diese Unterformen alle nach einem Trainingsschema behandelt werden („one size fits it all“) oder können für die Subformen spezifische Empfehlungen geben werden?
- Die Dosis des körperlichen Trainings lag in den Studien zwischen zwei- und sechsmal pro Woche. Besteht eine Dosis-Wirkungs-Kurve? Welche Trainingsdosis zeigt die größten Effekte?

- Wie lange sollte die körperliche Trainingsbehandlung durchgeführt werden? Wie lange wirkt diese, bezogen auf die Verbesserung kognitiver Leistungen?

Fragestellung 129: Wie effektiv sind physische Interventionen (Physiotherapie und z.B. Nordic Walking, aerobes Training, Tanzen etc.) im Vergleich mit einer Vergleichsintervention in der Behandlung von kognitiven Defiziten bei PKD

Hintergrund

Gesunde Erwachsene profitieren von einem Ausdauertraining hinsichtlich kognitiver Fertigkeiten. Hierbei kommt es zu einer Verbesserung der Aufmerksamkeit und der psychomotorischen Geschwindigkeit, der exekutiven Funktionen und des Gedächtnisses [178]. Diese kognitiven Domänen sind auch bei PK betroffen und ein Progress dieser Störungen kann in bis zu 80% der Fälle zu einer Demenz führen [8]. Personen mit einer Alzheimer-Erkrankung profitieren dosisabhängig von Ausdauersport und bessern hierbei insbesondere die exekutiven Leistungen [179]. Zahlreiche Studien belegen, dass ein physisches Training die kognitiven Fertigkeiten bei nicht dementen Personen mit PK verbessert [157]. Zudem ist eine höheres „Lebenszeit-Trainingspensum" mit einem verbesserten globalen kognitiven Status (höherer MMST-Wert) bei Personen mit einer PK verbunden [180]. Dieses legt die Vermutung nahe, dass auch Personen mit PKD von einem physischen Training hinsichtlich kognitiver Fertigkeiten profitieren könnten.

Evidenzgrundlage

Die initiale Literatursuche in Pubmed erbrachte 208 Studien. Für die Literaturrecherche wurden folgende Schlagworte verwendet: (Parkinson) AND (physical exercise OR physical modalities OR physiotherapy OR physical therapy OR exercise movement OR exercise therapy OR physical exercise OR aerobic exercise OR resistance training OR strength training OR weight-lifting OR weight-bearing) AND (cognition OR cognitive function OR executive function OR problem solving OR perception OR memory OR attention OR concentration OR learning OR dementia). Plural und Umstellungen der Fachtermini wurden berücksichtigt. Nach Screening von Titeln und Abstracts wurden 2 Studien als relevant für die Leitlinie bewertet. Einschlusskriterien war das Vorhandensein einer PKD, operationalisiert nach internationalen Kriterien.

Ergebnisse

In der PRIDE Study absolvierten 3 Personen mit PKD ein multimodales Training mit Balance, Ausdauer und Gehtraining, jeweils 60 Minuten, dreimal pro Woche über einen Zeitraum von 8 Wochen. Die Autoren/Autorinnen inkludierten weitere 5 Personen mit DLK, welche gemeinsam als Gruppe im Verlauf des Trainings eine leichte Verbesserung kognitiver Fertigkeiten zeigten. Ein Fallbericht untersuchte eine Person mit PKD [181] und in einer weiteren Studie waren zwei Personen mit PKD eingeschlossen [182].

Begründung der Empfehlung

Die dargelegten Arbeiten zeigen, dass die Thematik bislang nicht hinreichend untersucht wurde. Es lässt sich aus den Daten keine konklusive Empfehlung ableiten.

Empfehlung	Neu<br>Stand (2023)
Die empirische Datenlage ist nicht ausreichend, um die Wirksamkeit von physischen Interventionen bei PKD zu beurteilen (Expertenmeinung).	
Konsensstärke: 96,7%, starker Konsens	

Referenzen

1. Aarsland D, Bronnick K, Williams-Gray C, Weintraub D, Marder K, Kulisevsky J, et al. Mild cognitive impairment in Parkinson disease: a multicenter pooled analysis. Neurology. 2010;75(12):1062-9
2. Litvan I, Aarsland D, Adler CH, Goldman JG, Kulisevsky J, Mollenhauer B, et al. MDS Task Force on mild cognitive impairment in Parkinson's disease: critical review of PD-MCI. Mov Disord. 2011;26(10):1814-24
3. Yarnall AJ, Breen DP, Duncan GW, Khoo TK, Coleman SY, Firbank MJ, et al. Characterizing mild cognitive impairment in incident Parkinson disease: the ICICLE-PD study. Neurology. 2014;82(4):308-16
4. Boel JA, de Bie RMA, Schmand BA, Dalrymple-Alford JC, Marras C, Adler CH, et al. Level I PD-MCI Using Global Cognitive Tests and the Risk for Parkinson's Disease Dementia. Mov Disord Clin Pract. 2022;9(4):479-83
5. Hoogland J, Boel JA, de Bie RMA, Schmand BA, Geskus RB, Dalrymple-Alford JC, et al. Risk of Parkinson's disease dementia related to level I MDS PD-MCI. Mov Disord. 2019;34(3):430-5
6. Aarsland D, Zaccai J, Brayne C. A systematic review of prevalence studies of dementia in Parkinson's disease. Mov Disord. 2005;20(10):1255-63
7. Emre M. Dementia associated with Parkinson's disease. Lancet Neurol. 2003;2(4):229-37
8. Hely MA, Reid WG, Adena MA, Halliday GM, Morris JG. The Sydney multicenter study of Parkinson's disease: the inevitability of dementia at 20 years. Mov Disord. 2008;23(6):837-44
9. Aarsland D, Kurz MW. The epidemiology of dementia associated with Parkinson disease. J Neurol Sci. 2010;289(1-2):18-22
10. Emre M, Aarsland D, Brown R, Burn DJ, Duyckaerts C, Mizuno Y, et al. Clinical diagnostic criteria for dementia associated with Parkinson's disease. Mov Disord. 2007;22(12):1689-707; quiz 837
11. Saredakis D, Collins-Praino LE, Gutteridge DS, Stephan BCM, Keage HAD. Conversion to MCI and dementia in Parkinson's disease: a systematic review and meta-analysis. Parkinsonism Relat Disord. 2019;65:20-31
12. Liepelt-Scarfone I, Gräber S, Kalbe E, Riedel O, Ringendahl H, Schmidt N, et al. [Guidelines for the Neuropsychological Assessment of Patients with Parkinson's Disease]. Fortschr Neurol Psychiatr. 2021;89(7-08):363-73
13. Skorvanek M, Goldman JG, Jahanshahi M, Marras C, Rektorova I, Schmand B, et al. Global scales for cognitive screening in Parkinson's disease: Critique and recommendations. Mov Disord. 2018;33(2):208-18
14. Dalrymple-Alford JC, MacAskill MR, Nakas CT, Livingston L, Graham C, Crucian GP, et al. The MoCA: well-suited screen for cognitive impairment in Parkinson disease. Neurology. 2010;75(19):1717-25
15. Fengler S, Kessler J, Timmermann L, Zapf A, Elben S, Wojtecki L, et al. Screening for Cognitive Impairment in Parkinson's Disease: Improving the Diagnostic Utility of the MoCA through Subtest Weighting. PLoS One. 2016;11(7):e0159318

16. Sulzer P, Becker S, Maetzler W, Kalbe E, van Nueten L, Timmers M, et al. Validation of a novel Montreal Cognitive Assessment scoring algorithm in non-demented Parkinson's disease patients. J Neurol. 2018;265(9):1976-84
17. Thomann AE, Goettel N, Monsch RJ, Berres M, Jahn T, Steiner LA, Monsch AU. The Montreal Cognitive Assessment: Normative Data from a German-Speaking Cohort and Comparison with International Normative Samples. J Alzheimers Dis. 2018;64(2):643-55
18. Pirogovsky E, Schiehser DM, Litvan I, Obtera KM, Burke MM, Lessig SL, et al. The utility of the Mattis Dementia Rating Scale in Parkinson's disease mild cognitive impairment. Parkinsonism Relat Disord. 2014;20(6):627-31
19. Gülke E, Alsalem M, Kirsten M, Vettorazzi E, Choe CU, Hidding U, et al. Comparison of Montreal cognitive assessment and Mattis dementia rating scale in the preoperative evaluation of subthalamic stimulation in Parkinson's disease. PLoS One. 2022;17(4):e0265314
20. Hoops S, Nazem S, Siderowf AD, Duda JE, Xie SX, Stern MB, Weintraub D. Validity of the MoCA and MMSE in the detection of MCI and dementia in Parkinson disease. Neurology. 2009;73(21):1738-45
21. Monsch AU. [Neuropsychological examination in evaluating dementia]. Praxis (Bern 1994). 1997;86(35):1340-2
22. Uhlmann RF, Larson EB. Effect of education on the mini-mental state examination as a screening test for dementia. J Am Geriatr Soc. 1991;39(9):876-80
23. Lucza T, Ascherman Z, Kovács M, Makkos A, Harmat M, Juhász A, et al. Comparing Sensitivity and Specificity of Addenbrooke's Cognitive Examination-I, III and Mini-Addenbrooke's Cognitive Examination in Parkinson's Disease. Behav Neurol. 2018;2018:5932028
24. Federico A, Maier A, Vianello G, Mapelli D, Trentin M, Zanette G, et al. Screening for Mild Cognitive Impairment in Parkinson's Disease: Comparison of the Italian Versions of Three Neuropsychological Tests. Parkinsons Dis. 2015;2015:681976
25. Alexopoulos P, Greim B, Nadler K, Martens U, Krecklow B, Domes G, et al. Validation of the Addenbrooke's cognitive examination for detecting early Alzheimer's disease and mild vascular dementia in a German population. Dement Geriatr Cogn Disord. 2006;22(5-6):385-91
26. Alexopoulos P, Ebert A, Richter-Schmidinger T, Schöll E, Natale B, Aguilar CA, et al. Validation of the German revised Addenbrooke's cognitive examination for detecting mild cognitive impairment, mild dementia in alzheimer's disease and frontotemporal lobar degeneration. Dement Geriatr Cogn Disord. 2010;29(5):448-56
27. Dubois B, Slachevsky A, Litvan I, Pillon B. The FAB: a Frontal Assessment Battery at bedside. Neurology. 2000;55(11):1621-6
28. Mazancova AF, Růžička E, Jech R, Bezdicek O. Test the Best: Classification Accuracies of Four Cognitive Rating Scales for Parkinson's Disease Mild Cognitive Impairment. Arch Clin Neuropsychol. 2020
29. Benke T, Karner E, Delazer M. FAB-D: German version of the Frontal Assessment Battery. J Neurol. 2013;260(8):2066-72
30. Kalbe E, Calabrese P, Kohn N, Hilker R, Riedel O, Wittchen HU, et al. Screening for cognitive deficits in Parkinson's disease with the Parkinson neuropsychometric dementia assessment (PANDA) instrument. Parkinsonism Relat Disord. 2008;14(2):93-101
31. Dubois B, Burn D, Goetz C, Aarsland D, Brown RG, Broe GA, et al. Diagnostic procedures for Parkinson's disease dementia: recommendations from the movement disorder society task force. Mov Disord. 2007;22(16):2314-24
32. Lucza T, Karádi K, Kállai J, Weintraut R, Janszky J, Makkos A, et al. Screening Mild and Major Neurocognitive Disorders in Parkinson's Disease. Behav Neurol. 2015;2015:983606
33. Llebaria G, Pagonabarraga J, Kulisevsky J, García-Sánchez C, Pascual-Sedano B, Gironell A, Martínez-Corral M. Cut-off score of the Mattis Dementia Rating Scale for screening dementia in Parkinson's disease. Mov Disord. 2008;23(11):1546-50

34. Verbaan D, Jeukens-Visser M, Van Laar T, van Rooden SM, Van Zwet EW, Marinus J, van Hilten JJ. SCOPA-cognition cutoff value for detection of Parkinson's disease dementia. Mov Disord. 2011;26(10):1881-6
35. Barton B, Grabli D, Bernard B, Czernecki V, Goldman JG, Stebbins G, et al. Clinical validation of Movement Disorder Society-recommended diagnostic criteria for Parkinson's disease with dementia. Mov Disord. 2012;27(2):248-53
36. Riedel O, Klotsche J, Spottke A, Deuschl G, Förstl H, Henn F, et al. Cognitive impairment in 873 patients with idiopathic Parkinson's disease. Results from the German Study on Epidemiology of Parkinson's Disease with Dementia (GEPAD). J Neurol. 2008;255(2):255-64
37. Gasser AI, Calabrese P, Kalbe E, Kessler J, Rossier P. Cognitive screening in Parkinson's disease: Comparison of the Parkinson Neuropsychometric Dementia Assessment (PANDA) with 3 other short scales. Rev Neurol (Paris). 2016;172(2):138-45
38. Pignatti R, Bertella L, Scarpina F, Mauro A, Portolani E, Calabrese P. Italian version of the Parkinson Neuropsychometric Dementia Assessment (PANDA): a useful instrument to detect cognitive impairments in Parkinson's Disease. J Parkinsons Dis. 2014;4(2):151-60
39. Hobson P, Meara J. The detection of dementia and cognitive impairment in a community population of elderly people with Parkinson's disease by use of the CAMCOG neuropsychological test. Age Ageing. 1999;28(1):39-43
40. Athey RJ, Porter RW, Walker RW. Cognitive assessment of a representative community population with Parkinson's disease (PD) using the Cambridge Cognitive Assessment-Revised (CAMCOG-R). Age Ageing. 2005;34(3):268-73
41. Nübling G, Loosli SV, Wlasich E, Prix C, Schönecker S, Freudelsperger L, et al. [A German version of the Cambridge examination for mental disorders of older people with Down's syndrome and others with intellectual disabilities : A diagnostic procedure for detecting dementia in people with Down's syndrome]. Z Gerontol Geriatr. 2020;53(6):546-51
42. Kehagia AA, Barker RA, Robbins TW. Neuropsychological and clinical heterogeneity of cognitive impairment and dementia in patients with Parkinson's disease. Lancet Neurol. 2010;9(12):1200-13
43. Dirnberger G, Jahanshahi M. Executive dysfunction in Parkinson's disease: a review. J Neuropsychol. 2013;7(2):193-224
44. Fengler S, Liepelt-Scarfone I, Brockmann K, Schäffer E, Berg D, Kalbe E. Cognitive changes in prodromal Parkinson's disease: A review. Mov Disord. 2017;32(12):1655-66
45. Muslimovic D, Post B, Speelman JD, Schmand B. Cognitive profile of patients with newly diagnosed Parkinson disease. Neurology. 2005;65(8):1239-45
46. Hoogland J, van Wanrooij LL, Boel JA, Goldman JG, Stebbins GT, Dalrymple-Alford JC, et al. Detecting Mild Cognitive Deficits in Parkinson's Disease: Comparison of Neuropsychological Tests. Mov Disord. 2018;33(11):1750-9
47. Goldman JG, Holden S, Ouyang B, Bernard B, Goetz CG, Stebbins GT. Diagnosing PD-MCI by MDS Task Force criteria: how many and which neuropsychological tests? Mov Disord. 2015;30(3):402-6
48. Barton BR, Bernard B, Czernecki V, Goldman JG, Stebbins G, Dubois B, Goetz CG. Comparison of the Movement Disorder Society Parkinson's disease dementia criteria with neuropsychological testing. Mov Disord. 2014;29(10):1252-7
49. Liepelt-Scarfone I, Graeber S, Feseker A, Baysal G, Godau J, Gaenslen A, et al. Influence of different cut-off values on the diagnosis of mild cognitive impairment in Parkinson's disease. Parkinsons Dis. 2011;2011:540843
50. Dalrymple-Alford JC, Livingston L, MacAskill MR, Graham C, Melzer TR, Porter RJ, et al. Characterizing mild cognitive impairment in Parkinson's disease. Mov Disord. 2011;26(4):629-36
51. Ciafone J, Little B, Thomas AJ, Gallagher P. The Neuropsychological Profile of Mild Cognitive Impairment in Lewy Body Dementias. J Int Neuropsychol Soc. 2020;26(2):210-25

52. Wallace ER, Segerstrom SC, van Horne CG, Schmitt FA, Koehl LM. Meta-Analysis of Cognition in Parkinson's Disease Mild Cognitive Impairment and Dementia Progression. Neuropsychol Rev. 2022;32(1):149-60
53. Lillig R, Ophey A, Schulz JB, Reetz K, Wojtala J, Storch A, et al. A new CERAD total score with equally weighted z-scores and additional executive and non-amnestic "CERAD-Plus" tests enhances cognitive diagnosis in patients with Parkinson's disease: Evidence from the LANDSCAPE study. Parkinsonism Relat Disord. 2021;90:90-7
54. Lawson RA, Williams-Gray CH, Camacho M, Duncan GW, Khoo TK, Breen DP, et al. Which Neuropsychological Tests? Predicting Cognitive Decline and Dementia in Parkinson's Disease in the ICICLE-PD Cohort. J Parkinsons Dis. 2021;11(3):1297-308
55. Mahieux F, Fénelon G, Flahault A, Manifacier MJ, Michelet D, Boller F. Neuropsychological prediction of dementia in Parkinson's disease. J Neurol Neurosurg Psychiatry. 1998;64(2):178-83
56. Williams-Gray CH, Foltynie T, Brayne CE, Robbins TW, Barker RA. Evolution of cognitive dysfunction in an incident Parkinson's disease cohort. Brain. 2007;130(Pt 7):1787-98
57. Sanyal J, Banerjee TK, Rao VR. Dementia and cognitive impairment in patients with Parkinson's disease from India: a 7-year prospective study. Am J Alzheimers Dis Other Demen. 2014;29(7):630-6
58. Biundo R, Weis L, Facchini S, Formento-Dojot P, Vallelunga A, Pilleri M, Antonini A. Cognitive profiling of Parkinson disease patients with mild cognitive impairment and dementia. Parkinsonism Relat Disord. 2014;20(4):394-9
59. Lim KB, Kim J, Lee HJ, Yoo J, Kim HS, Kim C, Lee H. COWAT Performance of Persons with Alzheimer Dementia, Vascular Dementia, and Parkinson Disease Dementia According to Stage of Cognitive Impairment. Pm r. 2019;11(7):737-44
60. Liebermann-Jordanidis H, Roheger M, Boosfeld L, Franklin J, Kalbe E. Which Test Is the Best to Assess Visuo-Cognitive Impairment in Patients with Parkinson's Disease with Mild Cognitive Impairment and Dementia? A Systematic Review and Meta-Analysis. J Parkinsons Dis. 2022;12(6):1749-82
61. Camargo CHF, Bronzini A, Tolentino ES, Medyk C, Schultz-Pereira GL. Can the CERAD neuropsychological battery be used to assess cognitive impairment in Parkinson's disease? Arq Neuropsiquiatr. 2018;76(3):145-9
62. Rosenthal E, Brennan L, Xie S, Hurtig H, Milber J, Weintraub D, et al. Association between cognition and function in patients with Parkinson disease with and without dementia. Mov Disord. 2010;25(9):1170-6
63. Glonnegger H, Beyle A, Cerff B, Gräber S, Csoti I, Berg D, Liepelt-Scarfone I. The Multiple Object Test as a Performance Based Tool to Assess Cognitive Driven Activity of Daily Living Function in Parkinson's Disease. J Alzheimers Dis. 2016;53(4):1475-84
64. Martin RC, Triebel KL, Kennedy RE, Nicholas AP, Watts RL, Stover NP, et al. Impaired financial abilities in Parkinson's disease patients with mild cognitive impairment and dementia. Parkinsonism Relat Disord. 2013;19(11):986-90
65. Shulman LM, Pretzer-Aboff I, Anderson KE, Stevenson R, Vaughan CG, Gruber-Baldini AL, et al. Subjective report versus objective measurement of activities of daily living in Parkinson's disease. Mov Disord. 2006;21(6):794-9
66. Brod M, Mendelsohn GA, Roberts B. Patients' experiences of Parkinson's disease. J Gerontol B Psychol Sci Soc Sci. 1998;53(4):P213-22
67. Kempen GI, Steverink N, Ormel J, Deeg DJ. The assessment of ADL among frail elderly in an interview survey: self-report versus performance-based tests and determinants of discrepancies. J Gerontol B Psychol Sci Soc Sci. 1996;51(5):P254-60
68. Laatu S, Karrasch M, Martikainen K, Marttila R. Apathy is associated with activities of daily living ability in Parkinson's disease. Dement Geriatr Cogn Disord. 2013;35(5-6):249-55

69. Lawton MP, Brody EM. Assessment of older people: self-maintaining and instrumental activities of daily living. Gerontologist. 1969;9(3):179-86
70. Christ JB, Fruhmann Berger M, Riedl E, Prakash D, Csoti I, Molt W, et al. How precise are activities of daily living scales for the diagnosis of Parkinson's disease dementia? A pilot study. Parkinsonism Relat Disord. 2013;19(3):371-4
71. Shulman LM, Armstrong M, Ellis T, Gruber-Baldini A, Horak F, Nieuwboer A, et al. Disability Rating Scales in Parkinson's Disease: Critique and Recommendations. Mov Disord. 2016;31(10):1455-65
72. Dujardin K, Dubois B, Tison F, Durif F, Bourdeix I, Péré JJ, Duhamel A. Parkinson's disease dementia can be easily detected in routine clinical practice. Mov Disord. 2010;25(16):2769-76
73. Giovannetti T, Britnell P, Brennan L, Siderowf A, Grossman M, Libon DJ, et al. Everyday action impairment in Parkinson's disease dementia. J Int Neuropsychol Soc. 2012;18(5):787-98
74. Leroi I, McDonald K, Pantula H, Harbishettar V. Cognitive impairment in Parkinson disease: impact on quality of life, disability, and caregiver burden. J Geriatr Psychiatry Neurol. 2012;25(4):208-14
75. Stella F, Banzato CEM, Quagliato E, Viana MA, Christofoletti G. Dementia and functional decline in patients with Parkinson's disease. Dement Neuropsychol. 2008;2(2):96-101
76. Martinez-Martin P. Dementia in Parkinson's disease: usefulness of the pill questionnaire. Mov Disord. 2013;28(13):1832-7
77. Wahle M, Häller S, Spiegel R. Validation of the NOSGER (Nurses' Observation Scale for Geriatric Patients): reliability and validity of a caregiver rating instrument. Int Psychogeriatr. 1996;8(4):525-47
78. Spiegel R, Brunner C, Ermini-Fünfschilling D, Monsch A, Notter M, Puxty J, Tremmel L. A new behavioral assessment scale for geriatric out- and in-patients: the NOSGER (Nurses' Observation Scale for Geriatric Patients). J Am Geriatr Soc. 1991;39(4):339-47
79. Oswald WD, Fleischmann UM. Das Nürnberger-Alters-Inventar (NAI): Hofgrefe, Göttingen, Germany; 1999.
80. Kulisevsky J, Fernández de Bobadilla R, Pagonabarraga J, Martínez-Horta S, Campolongo A, García-Sánchez C, et al. Measuring functional impact of cognitive impairment: validation of the Parkinson's disease cognitive functional rating scale. Parkinsonism Relat Disord. 2013;19(9):812-7
81. Ruzafa-Valiente E, Fernández-Bobadilla R, García-Sánchez C, Pagonabarraga J, Martínez-Horta S, Kulisevsky J. Parkinson's Disease--Cognitive Functional Rating Scale across different conditions and degrees of cognitive impairment. J Neurol Sci. 2016;361:66-71
82. Rosenblum S, Meyer S, Richardson A, Hassin-Baer S. Early identification of subjective cognitive functional decline among patients with Parkinson's disease: a longitudinal pilot study. Sci Rep. 2022;12(1):22242
83. Livingston G, Huntley J, Sommerlad A, Ames D, Ballard C, Banerjee S, et al. Dementia prevention, intervention, and care: 2020 report of the Lancet Commission. Lancet. 2020;396(10248):413-46
84. *https://www.bundesgesundheitsministerium.de/krankenversicherung-praevention.html*.
85. Kalbe E, Folkerts AK. [Cognitive Training in Parkinson's Disease - A New Therapy Option?]. Fortschr Neurol Psychiatr. 2016;84 Suppl 1:S24-35
86. Nagata K, Tsunoda K, Fujii Y, Jindo T, Okura T. Impact of exercising alone and exercising with others on the risk of cognitive impairment among older Japanese adults. Arch Gerontol Geriatr. 2023;107:104908
87. Bacanoiu MV, Danoiu M, Rusu L, Marin MI. New Directions to Approach Oxidative Stress Related to Physical Activity and Nutraceuticals in Normal Aging and Neurodegenerative Aging. Antioxidants (Basel). 2023;12(5)

88. Pupíková M, Rektorová I. Non-pharmacological management of cognitive impairment in Parkinson's disease. J Neural Transm (Vienna). 2020;127(5):799-820
89. Giustiniani A, Maistrello L, Danesin L, Rigon E, Burgio F. Effects of cognitive rehabilitation in Parkinson disease: a meta-analysis. Neurol Sci. 2022;43(4):2323-37
90. Gavelin HM, Domellöf ME, Leung I, Neely AS, Launder NH, Nategh L, et al. Computerized cognitive training in Parkinson's disease: A systematic review and meta-analysis. Ageing Res Rev. 2022;80:101671
91. Kurowska A, Ziemichód W, Herbet M, Piątkowska-Chmiel I. The Role of Diet as a Modulator of the Inflammatory Process in the Neurological Diseases. Nutrients. 2023;15(6)
92. Jones JD, Baxter F, Timblin H, Rivas R, Hill CH. Physical inactivity is associated with Parkinson's disease mild cognitive impairment and dementia. Mental Health and Physical Activity. 2022;23
93. Bjornestad A, Pedersen KF, Tysnes OB, Alves G. Clinical milestones in Parkinson's disease: A 7-year population-based incident cohort study. Parkinsonism Relat Disord. 2017;42:28-33
94. Llamas-Velasco S, Contador I, Villarejo-Galende A, Lora-Pablos D, Bermejo-Pareja F. Physical Activity as Protective Factor against Dementia: A Prospective Population-Based Study (NEDICES). J Int Neuropsychol Soc. 2015;21(10):861-7
95. Yoon M, Yang PS, Jin MN, Yu HT, Kim TH, Jang E, et al. Association of Physical Activity Level With Risk of Dementia in a Nationwide Cohort in Korea. JAMA Netw Open. 2021;4(12):e2138526
96. Troutman SBW, Erickson KI, Grove G, Weinstein AM. Sedentary Time is Associated with Worse Attention in Parkinson's Disease: A Pilot Study. J Mov Disord. 2020;13(2):146-9
97. Bode M, Sulzer P, Schulte C, Becker S, Brockmann K, Elben S, et al. Multidomain cognitive training increases physical activity in people with Parkinson's disease with mild cognitive impairment. Parkinsonism Relat Disord. 2023;113:105330
98. Petrelli A, Kaesberg S, Barbe MT, Timmermann L, Fink GR, Kessler J, Kalbe E. Effects of cognitive training in Parkinson's disease: a randomized controlled trial. Parkinsonism Relat Disord. 2014;20(11):1196-202
99. Xie Y, Feng H, Peng S, Xiao J, Zhang J. Association of plasma homocysteine, vitamin B12 and folate levels with cognitive function in Parkinson's disease: A meta-analysis. Neurosci Lett. 2017;636:190-5
100. McShane R, Westby MJ, Roberts E, Minakaran N, Schneider L, Farrimond LE, et al. Memantine for dementia. Cochrane Database Syst Rev. 2019;3(3):Cd003154
101. Kawashima S, Matsukawa N. Memantine for the patients with mild cognitive impairment in Parkinson's disease: a pharmacological fMRI study. BMC Neurol. 2022;22(1):175
102. Brennan L, Pantelyat A, Duda JE, Morley JF, Weintraub D, Wilkinson JR, Moberg PJ. Memantine and Cognition in Parkinson's Disease Dementia/Dementia With Lewy Bodies: A Meta-Analysis. Mov Disord Clin Pract. 2016;3(2):161-7
103. van der Zee S, Kanel P, Gerritsen MJJ, Boertien JM, Slomp AC, Müller M, et al. Altered Cholinergic Innervation in De Novo Parkinson's Disease with and Without Cognitive Impairment. Mov Disord. 2022;37(4):713-23
104. Gargouri F, Gallea C, Mongin M, Pyatigorskaya N, Valabregue R, Ewenczyk C, et al. Multimodal magnetic resonance imaging investigation of basal forebrain damage and cognitive deficits in Parkinson's disease. Mov Disord. 2019;34(4):516-25
105. van der Zee S, Müller M, Kanel P, van Laar T, Bohnen NI. Cholinergic Denervation Patterns Across Cognitive Domains in Parkinson's Disease. Mov Disord. 2021;36(3):642-50
106. Mamikonyan E, Xie SX, Melvin E, Weintraub D. Rivastigmine for mild cognitive impairment in Parkinson disease: a placebo-controlled study. Mov Disord. 2015;30(7):912-8
107. Baik K, Kim SM, Jung JH, Lee YH, Chung SJ, Yoo HS, et al. Donepezil for mild cognitive impairment in Parkinson's disease. Sci Rep. 2021;11(1):4734

108. Sawada H, Oeda T, Kohsaka M, Umemura A, Tomita S, Park K, et al. Early use of donepezil against psychosis and cognitive decline in Parkinson's disease: a randomised controlled trial for 2 years. J Neurol Neurosurg Psychiatry. 2018;89(12):1332-40
109. Sawada H, Oeda T, Kohsaka M, Tomita S, Umemura A, Park K, et al. Early-start vs delayed-start donepezil against cognitive decline in Parkinson disease: a randomized clinical trial. Expert Opin Pharmacother. 2021;22(3):363-71
110. Hilker R, Thomas AV, Klein JC, Weisenbach S, Kalbe E, Burghaus L, et al. Dementia in Parkinson disease: functional imaging of cholinergic and dopaminergic pathways. Neurology. 2005;65(11):1716-22
111. Tiraboschi P, Hansen LA, Alford M, Sabbagh MN, Schoos B, Masliah E, et al. Cholinergic dysfunction in diseases with Lewy bodies. Neurology. 2000;54(2):407-11
112. Rolinski M, Fox C, Maidment I, McShane R. Cholinesterase inhibitors for dementia with Lewy bodies, Parkinson's disease dementia and cognitive impairment in Parkinson's disease. Cochrane Database Syst Rev. 2012;2012(3):Cd006504
113. Pagano G, Rengo G, Pasqualetti G, Femminella GD, Monzani F, Ferrara N, Tagliati M. Cholinesterase inhibitors for Parkinson's disease: a systematic review and meta-analysis. J Neurol Neurosurg Psychiatry. 2015;86(7):767-73
114. Matsunaga S, Kishi T, Yasue I, Iwata N. Cholinesterase Inhibitors for Lewy Body Disorders: A Meta-Analysis. Int J Neuropsychopharmacol. 2015;19(2)
115. Mischley LK, Farahnik J, Mantay L, Punzi J, Szampruch K, Ferguson T, Fox DJ. Parkinson Symptom Severity and Use of Nutraceuticals. Nutrients. 2023;15(4)
116. Rabiei Z, Solati K, Amini-Khoei H. Phytotherapy in treatment of Parkinson's disease: a review. Pharm Biol. 2019;57(1):355-62
117. Wolf JA, Jr., Grimm EA. Activated lymphocyte cytotoxicity: concepts and confusions. Ann Inst Pasteur Immunol. 1988;139(4):435-8
118. Barichella M, Cereda E, Iorio L, Pinelli G, Ferri V, Cassani E, et al. Clinical correlates of serum 25-hydroxyvitamin D in Parkinson's disease. Nutr Neurosci. 2022;25(6):1128-36
119. Peterson AL, Murchison C, Zabetian C, Leverenz JB, Watson GS, Montine T, et al. Memory, mood, and vitamin D in persons with Parkinson's disease. J Parkinsons Dis. 2013;3(4):547-55
120. de Lau LM, Koudstaal PJ, Witteman JC, Hofman A, Breteler MM. Dietary folate, vitamin B12, and vitamin B6 and the risk of Parkinson disease. Neurology. 2006;67(2):315-8
121. Phillips MCL, Murtagh DKJ, Gilbertson LJ, Asztely FJS, Lynch CDP. Low-fat versus ketogenic diet in Parkinson's disease: A pilot randomized controlled trial. Mov Disord. 2018;33(8):1306-14
122. Paknahad Z, Sheklabadi E, Derakhshan Y, Bagherniya M, Chitsaz A. The effect of the Mediterranean diet on cognitive function in patients with Parkinson's disease: A randomized clinical controlled trial. Complement Ther Med. 2020;50:102366
123. Li Z, Wang P, Yu Z, Cong Y, Sun H, Zhang J, et al. The effect of creatine and coenzyme q10 combination therapy on mild cognitive impairment in Parkinson's disease. Eur Neurol. 2015;73(3-4):205-11
124. Cho BH, Choi SM, Kim JT, Kim BC. Association of coffee consumption and non-motor symptoms in drug-naïve, early-stage Parkinson's disease. Parkinsonism Relat Disord. 2018;50:42-7
125. Cho BH, Choi SM, Kim BC. Gender-dependent effect of coffee consumption on tremor severity in de novo Parkinson's disease. BMC Neurol. 2019;19(1):194
126. *https://www.destatis.de/DE/Presse/Pressemitteilungen/Zahl-der-Woche/2022/PD22_44_p002.html*.
127. Zhang C, Zang Y, Song Q, Li H, Hu L, Zhao W, et al. The efficacy of a "cocktail therapy" on Parkinson's disease with dementia. Neuropsychiatr Dis Treat. 2019;15:1639-47
128. Bahar-Fuchs A, Martyr A, Goh AM, Sabates J, Clare L. Cognitive training for people with mild to moderate dementia. Cochrane Database Syst Rev. 2019;3(3):Cd013069

129. Leung IH, Walton CC, Hallock H, Lewis SJ, Valenzuela M, Lampit A. Cognitive training in Parkinson disease: A systematic review and meta-analysis. Neurology. 2015;85(21):1843-51
130. Lawrence BJ, Gasson N, Johnson AR, Booth L, Loftus AM. Cognitive Training and Transcranial Direct Current Stimulation for Mild Cognitive Impairment in Parkinson's Disease: A Randomized Controlled Trial. Parkinsons Dis. 2018;2018:4318475
131. Woods B, Aguirre E, Spector AE, Orrell M. Cognitive stimulation to improve cognitive functioning in people with dementia. Cochrane Database Syst Rev. 2012(2):Cd005562
132. Litvan I, Goldman JG, Tröster AI, Schmand BA, Weintraub D, Petersen RC, et al. Diagnostic criteria for mild cognitive impairment in Parkinson's disease: Movement Disorder Society Task Force guidelines. Mov Disord. 2012;27(3):349-56
133. Alloni A, Quaglini S, Panzarasa S, Sinforiani E, Bernini S. Evaluation of an ontology-based system for computerized cognitive rehabilitation. Int J Med Inform. 2018;115:64-72
134. Bernini S, Panzarasa S, Barbieri M, Sinforiani E, Quaglini S, Tassorelli C, Bottiroli S. A double-blind randomized controlled trial of the efficacy of cognitive training delivered using two different methods in mild cognitive impairment in Parkinson's disease: preliminary report of benefits associated with the use of a computerized tool. Aging Clin Exp Res. 2021;33(6):1567-75
135. De Luca R, Latella D, Maggio MG, Di Lorenzo G, Maresca G, Sciarrone F, et al. Computer assisted cognitive rehabilitation improves visuospatial and executive functions in Parkinson's disease: Preliminary results. NeuroRehabilitation. 2019;45(2):285-90
136. Giguère-Rancourt A, Plourde M, Racine E, Couture M, Langlois M, Dupré N, Simard M. Goal management training and psychoeducation / mindfulness for treatment of executive dysfunction in Parkinson's disease: A feasibility pilot trial. PLoS One. 2022;17(2):e0263108
137. París AP, Saleta HG, de la Cruz Crespo Maraver M, Silvestre E, Freixa MG, Torrellas CP, et al. Blind randomized controlled study of the efficacy of cognitive training in Parkinson's disease. Mov Disord. 2011;26(7):1251-8
138. Vlagsma TT, Duits AA, Dijkstra HT, van Laar T, Spikman JM. Effectiveness of ReSET; a strategic executive treatment for executive dysfunctioning in patients with Parkinson's disease. Neuropsychol Rehabil. 2020;30(1):67-84
139. Maggio MG, De Cola MC, Latella D, Maresca G, Finocchiaro C, La Rosa G, et al. What About the Role of Virtual Reality in Parkinson Disease's Cognitive Rehabilitation? Preliminary Findings From a Randomized Clinical Trial. J Geriatr Psychiatry Neurol. 2018;31(6):312-8
140. van de Weijer SCF, Duits AA, Bloem BR, de Vries NM, Kessels RPC, Köhler S, et al. Feasibility of a Cognitive Training Game in Parkinson's Disease: The Randomized Parkin'Play Study. Eur Neurol. 2020;83(4):426-32
141. Sousa NMF, Neri A, Brandi IV, Brucki SMD. Impact of cognitive intervention on cognitive symptoms and quality of life in idiopathic Parkinson's disease: a randomized and controlled study. Dement Neuropsychol. 2021;15(1):51-9
142. Angelucci F, Peppe A, Carlesimo GA, Serafini F, Zabberoni S, Barban F, et al. A pilot study on the effect of cognitive training on BDNF serum levels in individuals with Parkinson's disease. Front Hum Neurosci. 2015;9:130
143. Costa A, Peppe A, Serafini F, Zabberoni S, Barban F, Caltagirone C, Carlesimo GA. Prospective memory performance of patients with Parkinson's disease depends on shifting aptitude: evidence from cognitive rehabilitation. J Int Neuropsychol Soc. 2014;20(7):717-26
144. Kalbe E, Folkerts AK, Ophey A, Eggers C, Elben S, Dimenshteyn K, et al. Enhancement of Executive Functions but Not Memory by Multidomain Group Cognitive Training in Patients with Parkinson's Disease and Mild Cognitive Impairment: A Multicenter Randomized Controlled Trial. Parkinsons Dis. 2020;2020:4068706
145. Schmidt N, Tödt I, Berg D, Schlenstedt C, Folkerts AK, Ophey A, et al. Memory enhancement by multidomain group cognitive training in patients with Parkinson's disease and mild

cognitive impairment: long-term effects of a multicenter randomized controlled trial. J Neurol. 2021;268(12):4655-66
146. Lawrence BJ, Gasson N, Bucks RS, Troeung L, Loftus AM. Cognitive Training and Noninvasive Brain Stimulation for Cognition in Parkinson's Disease: A Meta-analysis. Neurorehabil Neural Repair. 2017;31(7):597-608
147. Woods B, O'Philbin L, Farrell EM, Spector AE, Orrell M. Reminiscence therapy for dementia. Cochrane Database Syst Rev. 2018;3(3):Cd001120
148. Watermeyer TJ, Hindle JV, Roberts J, Lawrence CL, Martyr A, Lloyd-Williams H, et al. Goal Setting for Cognitive Rehabilitation in Mild to Moderate Parkinson's Disease Dementia and Dementia with Lewy Bodies. Parkinsons Dis. 2016;2016:8285041
149. Leroi I, Vatter S, Carter LA, Smith SJ, Orgeta V, Poliakoff E, et al. Parkinson's-adapted cognitive stimulation therapy: a pilot randomized controlled clinical trial. Ther Adv Neurol Disord. 2019;12:1756286419852217
150. DGN, DGPPN. (Hrsg.) S3-Leitlinie Demenzen, verfügbar unter: *https://register.awmf.org/assets/guidelines/038-013l_S3-Demenzen-2016-07.pdf*.
151. Folkerts AK, Dorn ME, Roheger M, Maassen M, Koerts J, Tucha O, et al. Cognitive Stimulation for Individuals with Parkinson's Disease Dementia Living in Long-Term Care: Preliminary Data from a Randomized Crossover Pilot Study. Parkinsons Dis. 2018;2018:8104673
152. Sofi F, Valecchi D, Bacci D, Abbate R, Gensini GF, Casini A, Macchi C. Physical activity and risk of cognitive decline: a meta-analysis of prospective studies. J Intern Med. 2011;269(1):107-17
153. Hamer M, Chida Y. Physical activity and risk of neurodegenerative disease: a systematic review of prospective evidence. Psychol Med. 2009;39(1):3-11
154. Avenali M, Picascia M, Tassorelli C, Sinforiani E, Bernini S. Evaluation of the efficacy of physical therapy on cognitive decline at 6-month follow-up in Parkinson disease patients with mild cognitive impairment: a randomized controlled trial. Aging Clin Exp Res. 2021;33(12):3275-84
155. Picelli A, Varalta V, Melotti C, Zatezalo V, Fonte C, Amato S, et al. Effects of treadmill training on cognitive and motor features of patients with mild to moderate Parkinson's disease: a pilot, single-blind, randomized controlled trial. Funct Neurol. 2016;31(1):25-31
156. Silveira CRA, Roy EA, Intzandt BN, Almeida QJ. Aerobic exercise is more effective than goal-based exercise for the treatment of cognition in Parkinson's disease. Brain Cogn. 2018;122:1-8
157. da Silva FC, Iop RDR, de Oliveira LC, Boll AM, de Alvarenga JGS, Gutierres Filho PJB, et al. Effects of physical exercise programs on cognitive function in Parkinson's disease patients: A systematic review of randomized controlled trials of the last 10 years. PLoS One. 2018;13(2):e0193113
158. Cheung C, Bhimani R, Wyman JF, Konczak J, Zhang L, Mishra U, et al. Effects of yoga on oxidative stress, motor function, and non-motor symptoms in Parkinson's disease: a pilot randomized controlled trial. Pilot Feasibility Stud. 2018;4:162
159. de Oliveira RT, Felippe LA, Bucken Gobbi LT, Barbieri FA, Christofoletti G. Benefits of Exercise on the Executive Functions in People with Parkinson Disease: A Controlled Clinical Trial. Am J Phys Med Rehabil. 2017;96(5):301-6
160. Gobbi LTB, Pelicioni PHS, Lahr J, Lirani-Silva E, Teixeira-Arroyo C, Santos P. Effect of different types of exercises on psychological and cognitive features in people with Parkinson's disease: A randomized controlled trial. Ann Phys Rehabil Med. 2021;64(1):101407
161. Johansson ME, Cameron IGM, Van der Kolk NM, de Vries NM, Klimars E, Toni I, et al. Aerobic Exercise Alters Brain Function and Structure in Parkinson's Disease: A Randomized Controlled Trial. Ann Neurol. 2022;91(2):203-16

162. Rios Romenets S, Anang J, Fereshtehnejad SM, Pelletier A, Postuma R. Tango for treatment of motor and non-motor manifestations in Parkinson's disease: a randomized control study. Complement Ther Med. 2015;23(2):175-84
163. Solla P, Cugusi L, Bertoli M, Cereatti A, Della Croce U, Pani D, et al. Sardinian Folk Dance for Individuals with Parkinson's Disease: A Randomized Controlled Pilot Trial. J Altern Complement Med. 2019;25(3):305-16
164. Silva-Batista C, Corcos DM, Kanegusuku H, Piemonte MEP, Gobbi LTB, de Lima-Pardini AC, et al. Balance and fear of falling in subjects with Parkinson's disease is improved after exercises with motor complexity. Gait Posture. 2018;61:90-7
165. Conradsson D, Löfgren N, Nero H, Hagströmer M, Ståhle A, Lökk J, Franzén E. The Effects of Highly Challenging Balance Training in Elderly With Parkinson's Disease: A Randomized Controlled Trial. Neurorehabil Neural Repair. 2015;29(9):827-36
166. Schaible F, Maier F, Buchwitz TM, Schwartz F, Hoock M, Schönau E, et al. Effects of Lee Silverman Voice Treatment BIG and conventional physiotherapy on non-motor and motor symptoms in Parkinson's disease: a randomized controlled study comparing three exercise models. Ther Adv Neurol Disord. 2021;14:1756286420986744
167. King LA, Mancini M, Smulders K, Harker G, Lapidus JA, Ramsey K, et al. Cognitively Challenging Agility Boot Camp Program for Freezing of Gait in Parkinson Disease. Neurorehabil Neural Repair. 2020;34(5):417-27
168. Harvey M, Weston KL, Gray WK, O'Callaghan A, Oates LL, Davidson R, Walker RW. High-intensity interval training in people with Parkinson's disease: a randomized, controlled feasibility trial. Clin Rehabil. 2019;33(3):428-38
169. Youm C, Kim Y, Noh B, Lee M, Kim J, Cheon SM. Impact of Trunk Resistance and Stretching Exercise on Fall-Related Factors in Patients with Parkinson's Disease: A Randomized Controlled Pilot Study. Sensors (Basel). 2020;20(15)
170. Albrecht F, Pereira JB, Mijalkov M, Freidle M, Johansson H, Ekman U, et al. Effects of a Highly Challenging Balance Training Program on Motor Function and Brain Structure in Parkinson's Disease. J Parkinsons Dis. 2021;11(4):2057-71
171. Yang Y, Chen L, Yao J, Wang N, Liu D, Wang Y, et al. Early implementation of intended exercise improves quality of life in Parkinson's disease patients. Neurol Sci. 2022;43(3):1761-7
172. Michels K, Dubaz O, Hornthal E, Bega D. "Dance Therapy" as a psychotherapeutic movement intervention in Parkinson's disease. Complement Ther Med. 2018;40:248-52
173. Hasegawa N, Shah VV, Harker G, Carlson-Kuhta P, Nutt JG, Lapidus JA, et al. Responsiveness of Objective vs. Clinical Balance Domain Outcomes for Exercise Intervention in Parkinson's Disease. Front Neurol. 2020;11:940
174. Johansson H, Freidle M, Ekman U, Schalling E, Leavy B, Svenningsson P, et al. Feasibility Aspects of Exploring Exercise-Induced Neuroplasticity in Parkinson's Disease: A Pilot Randomized Controlled Trial. Parkinsons Dis. 2020;2020:2410863
175. Pohl P, Dizdar N, Hallert E. The Ronnie Gardiner Rhythm and Music Method - a feasibility study in Parkinson's disease. Disabil Rehabil. 2013;35(26):2197-204
176. King LA, Peterson DS, Mancini M, Carlson-Kuhta P, Fling BW, Smulders K, et al. Do cognitive measures and brain circuitry predict outcomes of exercise in Parkinson Disease: a randomized clinical trial. BMC Neurol. 2015;15:218
177. Kalbe E, Rehberg SP, Heber I, Kronenbuerger M, Schulz JB, Storch A, et al. Subtypes of mild cognitive impairment in patients with Parkinson's disease: evidence from the LANDSCAPE study. J Neurol Neurosurg Psychiatry. 2016;87(10):1099-105
178. Smith PJ, Blumenthal JA, Hoffman BM, Cooper H, Strauman TA, Welsh-Bohmer K, et al. Aerobic exercise and neurocognitive performance: a meta-analytic review of randomized controlled trials. Psychosom Med. 2010;72(3):239-52

179. Zeng Y, Wang J, Cai X, Zhang X, Zhang J, Peng M, et al. Effects of physical activity interventions on executive function in older adults with dementia: A meta-analysis of randomized controlled trials. Geriatr Nurs. 2023;51:369-77
180. Paul KC, Chuang YH, Shih IF, Keener A, Bordelon Y, Bronstein JM, Ritz B. The association between lifestyle factors and Parkinson's disease progression and mortality. Mov Disord. 2019;34(1):58-66
181. Tabak R, Aquije G, Fisher BE. Aerobic exercise to improve executive function in Parkinson disease: a case series. J Neurol Phys Ther. 2013;37(2):58-64
182. Telenius EW, Engedal K, Bergland A. Effect of a high-intensity exercise program on physical function and mental health in nursing home residents with dementia: an assessor blinded randomized controlled trial. PLoS One. 2015;10(5):e0126102

3.13 Affektive Störungen

Autoren/Autorinnen: Elke Kalbe, Karsten Witt, Carsten Buhmann, Ann-Kristin Folkerts

Hintergrund

Affektive Störungen umfassen u.a. depressive Störungen, Apathie und Angststörungen. Auch Fatigue als neuropsychiatrisches Symptom wird an dieser Stelle unter den affektiven Störungen abgehandelt. Affektive Störungen stellen bei der PK eine für Pat. und ihre Angehörigen in hohem Maße beeinträchtigende und häufige Problematik dar. Beispielsweise ermittelte eine Metaanalyse mit 30 Studien unter Berücksichtigung von N=7.142 Parkinson-Pat. mit einer Erkrankungsdauer von über 3 Jahren eine Prävalenz depressiver Störungen von 47,2%, einer Apathie von 45,5% und der Angststörungen von 42,9% [1].

Depressive Störungen

Depressive Störungen sind laut Entwurfsfassung des ICD-11 (Code 6A20.Z) [2] durch eine depressive Stimmung (z.B. traurig, reizbar, leer) oder Freudlosigkeit (d.h. Anhedonie) gekennzeichnet, die von anderen kognitiven, verhaltensbezogenen oder neurovegetativen Symptomen begleitet wird und die Funktionsfähigkeit der Person erheblich beeinträchtigen. Unterschieden werden hierbei einzelne depressive Episoden, rezidivierende depressive Störungen, dysthyme Störungen und gemischte depressive Störungen und Angststörungen.

Studien indizieren, dass es eine enge negative Korrelation der Lebensqualität mit dem Ausmaß depressiver Symptome gibt, während der Zusammenhang zu motorischen Einschränkungen nicht oder weniger gegeben ist [3], [4].

In einer Metaanalyse zur Prävalenz von depressiven Störungen bei Parkinson-Pat. ermittelten Cong et al. (2022) [5] auf Grundlage von 129 Studien mit insgesamt N=38.304 Parkinson-Pat. eine Prävalenz von 38%. Depressive Störungen waren mit einer längeren Erkrankungsdauer, der Erkrankungsschwere, verschiedenen motorischen Symptomen (v.a. Bradykinese) und nicht motorischen Symptomen (Apathie, Angststörungen, Fatigue) assoziiert.

Anhedonie

Anhedonie, d.h. die Unfähigkeit, Freude zu empfinden, kann ein Symptom sowohl für depressive als auch für apathische Syndrome sein [6]. Die Prävalenz wird zwischen 10 und 40% beschrieben [7].

Affektive Fluktuationen

40 bis 50% der Parkinson-Betroffenen berichten über motorische Fluktuationen. Viel weniger ist über die nicht motorischen Fluktuationen bekannt, welche sich häufig in der Domäne von Emotion und Affekt abspielen. In einem systematischen Review folgern die Autorinnen und Autoren, dass im Durchschnitt bei 35% der Parkinson-Pat. Angstgefühle, bei ebenfalls 35% Symptome einer Depression bestehen und auch Panikattacken nicht selten sind [8]. Die Zuordnung hypo- und hyperdopaminerger Symptome ist in der nicht für den deutschen Sprachraum validierten Skala „Ardouin Scale of Behavior in Parkinson's Disease" vorgeschlagen, welche u.a. nächtliche Hyperaktivität, Tagesschläfrigkeit, die Impulskontrollstörungen und das Punding dem

hyperdopaminergen Verhaltensmuster und u.a. Depression, Anhedonie, Apathie und Fatigue einem hypodopaminergen Verhaltensmuster zuordnet [9].

Apathie

Apathie ist definiert als Mangel an Motivation, der durch ein vermindertes zielorientiertes Denken und Handeln sowie einen reduzierten emotionalen Ausdruck gekennzeichnet ist. Es handelt sich demnach um eine Antriebsproblematik, die sich negativ auf die Therapieadhärenz der Parkinson-Pat. auswirken kann und häufig eine große Belastung für die Angehörigen darstellt [10].

Eine Metaanalyse aus dem Jahr 2015 identifizierte eine Prävalenz von 39,8% für das Vorliegen einer apathischen Symptomatik bei Parkinson-Betroffenen, wobei das Auftreten einer Apathie u.a. mit höherem Alter, kognitiven Beeinträchtigungen, dem Vorliegen von depressiven Störungen sowie einer fortgeschritteneren Parkinson-Symptomatik assoziiert ist [10].

Angststörungen

Laut ICD-11 [2] werden Angststörungen (Code 6B00-6B0Z) definiert als psychische Störungsbilder, die sich durch übermäßige Furcht und Angst und damit verbundene Verhaltensstörungen auszeichnen, wobei die Symptome so schwerwiegend sind, dass sie zu erheblichem Leid oder erheblichen Beeinträchtigungen in persönlichen, familiären, sozialen, schulischen, beruflichen oder anderen wichtigen Funktionsbereichen führen. Unter die Angststörungen lassen sich unterschiedliche Diagnosen subsumieren, z.B. generalisierte Angststörungen, Panikstörungen, Agoraphobien, spezifische Phobien oder soziale Angststörungen.

Für einen systematischen Review mit Metaanalyse aus dem Jahr 2016 konnten Broen et al. [11] 45 Studien mit insgesamt N=2399 Parkinson-Pat. identifizieren, in denen Ängste mit validierten Instrumenten erhoben worden waren. Sie berichten eine Punktprävalenz von 31% für Angststörungen allgemein, wobei die generalisierte Angststörung mit 14,1% die häufigste Form der Angststörungen darstellt. Weiterhin liegen die Punktprävalenzen für soziale Angststörungen bei 13,8%, für nicht näher bezeichnete Ängste bei 13,3%, für spezifische Phobien bei 13,0% und für Panikstörungen bei 6,8%.

Im Zuge chronisch progredienter Erkrankungen ist zudem die sogenannte Progredienzangst beschrieben. Hierbei handelt sich um eine „Realangst“, die aus der Erfahrung einer schweren, potenziell lebensbedrohlichen oder zur Behinderung führenden Erkrankung und ihrer Diagnostik und Therapie entsteht [12]. Differenzialdiagnostisch ist die Progredienzangst klar von den Angststörungen abzugrenzen. Berg et al. (2011) [12] verglichen unterschiedliche chronische Erkrankungen hinsichtlich ihres Ausmaßes an Progredienzängsten, wobei Parkinson-Pat. die zweithöchsten Werte zeigten. In einer Querschnittsstudie von Folkerts et al. (2022) [13] mit N=120 Parkinson-Pat. fanden sich moderate Progredienzangst-Ausprägungen in 63,0% der Befragten; 17,6% zeigten dysfunktionale Ausmaße, die mitunter behandlungsbedürftig sind.

Fatigue

Fatigue im Zusammenhang mit PK wird definiert als ein (nahezu) tägliches Gefühl „eines deutlich verminderten Energieniveaus oder einer erhöhten Wahrnehmung von Anstrengung, die in keinem

Verhältnis zu den versuchten Aktivitäten oder dem allgemeinen Aktivitätsniveau steht" [14]. Man unterscheidet zwischen der physischen Fatigue (d.h. ein Gefühl der physischen Erschöpfung bzw. einem Mangel an physischer Energie) und der mentalen Fatigue (d.h. die kognitiven Auswirkungen, die während und nach längeren Perioden anhaltender geistiger Anstrengung auftreten) [15].

Die Prävalenz von Fatigue bei Parkinson-Pat. wird sehr unterschiedlich berichtet und liegt zwischen 33 und 70%. Fatigue tritt häufiger in späteren Parkinson-Stadien auf [16]; sie kann sich jedoch auch in prämotorischen Parkinson-Stadien manifestieren [17]. Fatigue beeinträchtigt die Aktivitäten des täglichen Lebens und schränkt die soziale und berufliche Teilhabe sowie Lebensqualität der Parkinson-Pat. erheblich ein [18].

Fragestellung 130: Welche anamnestischen Fragen sind geeignet, bei Parkinson-Pat. affektive Störungen (depressive Störungen, Anhedonie, affektive Fluktuationen, Apathie, Angststörungen, Progredienzangst, Fatigue) zu erfassen?

Hintergrund

Trotz der Häufigkeit der affektiven Störungen sind diese neuropsychiatrischen Symptome unterdiagnostiziert und daher unbehandelt, was wahrscheinlich einen erheblichen negativen Einfluss auf die Lebensqualität und die Gesamtprognose hat [11], [10], [19], [20], [21]. So übersahen z.B. Neurologinnen und Neurologen bei einer Erhebung der Diagnosegüte an der Universität Miami, Florida, USA im ambulanten Kontakt jede zweite Depression bei Parkinson-Pat. [21]. Weitere Studien zeigen, dass nur ein Viertel der Parkinson-Pat. mit depressiven Störungen antidepressiv behandelt werden [22], [23]. Vor diesem Hintergrund ist der Einsatz aussagekräftiger Anamnesefragen und validierter Skalen für die möglichst frühzeitige Erfassung von affektiven Störungen bei PK-Pat. außerordentlich wichtig.

Die Diagnose affektiver Störungen bei Parkinson-Pat. ist schwierig, insbesondere, weil Anzeichen und Symptome affektiver Störungen sich mit den motorischen und anderen nicht motorischen Symptomen überschneiden können. Zudem sind beispielsweise die diagnostischen Kriterien depressiver Störungen, die bei Personen ohne PK aufgestellt wurden, möglicherweise nicht ohne Weiteres auf Parkinson-Pat. anwendbar, da das Spektrum der depressiven Symptome nicht vollständig gleich ist [24]. Depressive Symptome bei Parkinson-Betroffenen umfassen oft Dysphorie, Pessimismus, Angst sowie somatische Symptome und seltener Schuldgefühle, Versagensgefühle, Selbstvorwürfe oder vollzogene Suizide [25], [26], [27]. Andere fanden bei Parkinson-Pat. häufig ein Gefühl der Leere und Hoffnungslosigkeit, verminderte Reaktionsfähigkeit auf emotionale Reize oder Verlust der Fähigkeit, Freude zu genießen und zu empfinden (Anhedonie) [28]. Die klinische Diagnose einer Depression bei PK bezieht sich meistens auf das Vorhandensein solcher Symptome und nicht auf morgendliche Dysphorie, Gewichtsverlust oder zu viel oder zu wenig Schlaf [24]. Eine weitere Schwierigkeit und somit Herausforderung für die Diagnostik depressiver Störungen bei Parkinson-Pat. ergibt sich häufig aus einer begleitenden Demenz. Zum einen können mögliche Symptome einer depressiven Störung auch Anzeichen für eine Demenz sein (z.B. Anhedonie, Reizbarkeit) [24], zum anderen können auch kognitive Störungen depressionsbedingt sein. Bei fortgeschrittenen PK ist eine Überlappung einer Depression und einer Demenz häufig und stellt differenzialdiagnostisch eine Herausforderung dar.

Apathie kann bei Parkinson-Erkrankten isoliert, als Ausdruck einer Depression, als Wirkfluktuation oder auch im Kontext mit motorischen und kognitiven Symptomen auftreten. Insbesondere die klinische Abgrenzung der Apathie von den affektiven Symptomen einer Depression und von exekutiven Dysfunktionen, z.B. beim Planen und Organisieren, ist herausfordernd [29]. Bei der Differenzierung zwischen Depression und Apathie stoßen auch standardisierte Fragebögen an ihre Grenzen. Hier kann es hilfreich sein, die Pat. dahingehend zu befragen, ob die Stimmung durch externe Reize aufgehellt werden kann, was eher für eine Apathie spricht.

Fatigue bei Parkinson-Pat. geht zwar häufig im Sinne einer Komorbidität mit Tagesschläfrigkeit, Depression und Apathie einher, kann und sollte aber als eigenständiges Phänomen durch u.a. eine sorgfältige Anamnese von diesen getrennt werden [30].

Evidenzgrundlage

Die Literatursuche in PubMed erzielte 4 Treffer mit dem Suchstring ((((((Parkinson disease [MeSH]) OR (Parkinson* [tiab])) OR (Parkinsonian disorders [MeSH])) OR (Movement disorders [MeSH])) NOT ((((Wolff parkinson white syndrome [MeSH]) OR (wolff[tiab] AND parkinson*[tiab])) OR (wpw[tiab] AND syndrome[tiab])) OR (wpw [tiab]))) AND (((((((anamnesis[tiab]) OR (anamnesis[MeSH])) OR ((anamnes*[tiab] OR anamnesis[MeSH]) AND question[tiab])) OR (medical record[tiab] OR medical record[MeSH])) OR (medical history[tiab])) AND ((((standard scale[tiab] OR scale*[tiab]) OR (rating scale[tiab] OR rating scales[tiab])) OR (rating scale[MeSH])) OR (standardized rating scales[tiab]))) AND (((((((((Affective disorders[tiab] OR Affective disorder[tiab]) OR (Anhedonia[tiab] OR Anhedonia[MeSH])) OR (Depression[tiab] OR Depression[MeSH])) OR (affective fluctuations)) OR (Apathy[tiab] OR Apathy[MeSH])) OR (anxiety disorder[tiab] OR Anxiety disorder[MeSH])) OR (fear of progression)) OR (fear of recurrence)) OR (Fatigue[tiab] OR Fatigue[MeSH])))) AND ((((humans[mesh:noexp]) NOT (animals[mesh:noexp])) AND (2016:2021[pdat])) AND ((english[Filter] OR german[Filter]))). Inkludiert wurden nur Studien im Zeitraum von 2016 bis 2021 in englischer und deutscher Sprache. Nach Screening der Abstracts und Inhalte wurde keine Publikation als relevant identifiziert. Nach Erweiterung der Recherche um Publikationen, die den Expertinnen und Experten bekannt waren, sowie Literaturreferenzen aus diesen Publikationen wurden letztendlich 4 Publikationen selektiert, welche in diese Leitlinie einbezogen wurden. Es fanden sich keine kontrollierten Studien zu der genannten Fragestellung. Da bisher Evidenz fehlt, welche Fragen im Anamnesegespräch besonders geeignet sind, um in der klinischen Praxis affektive Störungen initial zu erfassen, erfolgte die Auswahl der Fragen auf Basis der Sichtung der bei PK üblicherweise eingesetzten Skalen (vgl. Fragestellunge 131) und der klinischen Erfahrung der Autorinnen/Autoren.

Ergebnis und Begründung für die Empfehlung

Das anamnestische Gespräch zur Diagnostik affektiver Störungen spielt in der Praxisroutine eine wesentliche Rolle. Zum einen bestehen limitierte zeitliche und personelle Ressourcen für den Einsatz (halb-)strukturierter klinischer Interviews oder standardisierter Skalen als Selbst- oder Fremdevaluation, mit denen affektive Störungen erfasst oder ihr Schwergrad evaluiert werden kann (vgl. Fragestellung 131). Zum anderen sollten Skalen per se nicht als alleiniges Instrument zur Diagnostik affektiver Störungen eingesetzt werden. Zu beachten ist nämlich, dass z.B. die Diagnose einer Depression nicht allein auf der Grundlage eines Scores gestellt werden kann, da hohe Depressionsscores bei somatischen Symptomen trotz fehlender Kernsymptome einer Depression (z.B. Traurigkeit, Interessensverlust, Anhedonie) sowie niedrige Scores trotz schwerer depressiver

Symptome auftreten können, wenn somatische oder vegetative Probleme fehlen. Alle Depressionsskalen enthalten Items zu Symptomen, die z.B. mit Depression, aber auch mit Apathie, Parkinsonismus und kognitiver Beeinträchtigung assoziiert sein können. Dies trifft u.a. insbesondere auf die Hamilton Depression Rating Scale (HAM-D) und in geringerem Maßeauf die Montgomery-Asberg Depression Rating Scale (MADRS) und das Beck-Depressions-Inventar (BDI) zu [31]. Ferner erfassen Skalen in der Regel das subjektive Befinden in den vorausgegangenen 1 oder 2 Wochen. Zudem berücksichtigen sie bei motorisch fluktuierenden Pat. nicht den objektiven klinischen Zustand. So können Pat. ihren eigenen Zustand im Off anders wahrnehmen als im On [32]. Zusätzlich können Off-Perioden auch mit fluktuierenden nicht motorischen Symptomen, Depressionen, Angstzuständen und Wahnvorstellungen einhergehen [24], [32], [33].

Für die Anamnese stellt sich die Frage, welche Fragen geeignet sind, um sensitiv und spezifisch affektive Störungen zu erfassen. Aufgrund der o.g. Evidenzlage und Erwägungen schlagen die Autorinnen/Autoren dieses Kapitels als Expertenkonsensusmeinung nachfolgende Fragen zur Erfassung affektiver Störungen vor. Die Anzahl und die Auswahl der Fragen wurden so gewählt, dass die Anwendung im klinischen Alltag sinnvoll und praktikabel erscheint. Bei der Anamneseerhebung sollten die Pat. in einem klinischen On-Status sein, der ggf. mit der Unified Parkinson's Disease Rating Scale Teil 3 (motorische Untersuchung; UPDRS-III) dokumentiert werden sollte. Bei einem erkennbaren klinischen Off-Status sollte erst eine Medikamentengabe erfolgen oder das Ergebnis als „vorbehaltlich" dokumentiert und im Verlauf im On-Status überprüft werden. Eine Fremdanamnese kann zur ergänzenden Diagnostik wertvolle Hinweise liefern. Dies trifft insbesondere bei Vorliegen kognitiver Störungen der Parkinson-Pat. zu.

Empfehlung

Die folgenden anamnestischen Fragen werden zur Erfassung affektiver Störungen (depressive Störungen, Anhedonie, affektive Fluktuationen, Apathie, Angststörungen, Fatigue) in der klinischen Routine empfohlen. Sofern die Anamnese Hinweise auf eine affektive Symptomatik ergibt, sollten zur weiteren Diagnostik standardisierte (halb-)strukturierte Interviews und/oder Skalen zum Einsatz kommen (vgl. Fragestellung 131). Es empfiehlt sich, für die unten genannten anamnestischen Fragen zu dokumentieren, ob subjektiv ein On-Zustand besteht; idealerweise sollte im Vorfeld des Anamnesegesprächs ein Best-on-Zustand hergestellt werden.

**Empfehlung: anamnestische Fragen zu affektiven Störungen**	**Modifiziert**<br>**Stand (2023)**
1. Zur Erfassung von <u>affektiven Störungen</u> bei Parkinson-Pat. sollen die beiden Fragen aus dem Zwei-Fragen-Test gestellt werden:<br>▪ Fühlten Sie sich im letzten Monat häufig niedergeschlagen, traurig, bedrückt oder hoffnungslos?<br>▪ Hatten Sie im letzten Monat deutlich weniger Lust und Freude an Dingen, die Sie sonst gerne tun?<br>2. Zusätzlich sollten folgende Fragen gestellt werden:<br>▪ Haben Sie jemals an einer depressiven Störung gelitten und sind Sie jemals antidepressiv	

therapiert worden? [34]

- Schlafen Sie gut ein bzw. durch?
- Wie ist Ihr Appetit?
- Sind sie oft innerlich unruhig?
- Können Sie sich weiterhin an Dingen erfreuen?
- Haben Sie Schwierigkeiten, sich zu konzentrieren oder aufmerksam zu blieben?
- Ändert sich Ihre Stimmung, wenn Ihre Medikamentenwirkung abnimmt oder ausbleibt?

3. Zur Erfassung von <u>Apathie und affektiven Fluktuationen</u> bei Parkinson-Pat. sollen folgende Fragen gestellt werden:
   - Haben Sie weiterhin Interesse an Tätigkeiten, Dingen oder Menschen?
   - Schlafen Sie tagsüber viel?
   - Unternehmen Sie Dinge mit Freunden oder Familie?
   - Ändern sich Ihr Antrieb und Ihre Motivation, wenn Ihre Medikamentenwirkung abnimmt oder ausbleibt?
4. Zur Erfassung von <u>Angststörungen und affektiven Fluktuationen</u> bei Parkinson-Pat. sollen folgende Fragen gestellt werden:
   - Sind sie öfters ängstlich?
   - Sind Sie öfters innerlich unruhig oder angespannt?
   - Neigen Sie zu Panikattacken?
   - Haben Sie Angst, unter Menschen zu gehen?
   - Treten Angstgefühle auf, wenn Ihre Medikamentenwirkung abnimmt oder ausbleibt?
5. Zur Erfassung von <u>Progredienzangst</u> bei Parkinson-Pat. sollen folgende Fragen gestellt werden:
   - Haben Sie Angst vor Ihrem weiteren Krankheitsverlauf?
   - Sehen Sie pessimistisch, mutlos oder hoffnungslos in die Zukunft, wenn Sie an den weiteren Verlauf Ihrer PK denken?
6. Zur Erfassung von <u>Fatigue und affektiven Fluktuationen</u> bei Parkinson-Pat. sollen folgende Fragen gestellt werden:
   - Fühlen Sie sich häufig müde oder erschöpft trotz ausreichend Schlaf?
   - Fehlt Ihnen häufiger die Kraft, Dinge zu tun?
   - Schlafen Sie tagsüber viel?
   - Haben Sie Schwierigkeiten, sich zu konzentrieren oder aufmerksam zu bleiben?
   - Fühlen Sie sich erschöpft oder abgeschlagen, wenn Ihre Medikamentenwirkung abnimmt oder ausbleibt?

Konsensstärke: 93,4%, Konsens

Fragestellung 131: Welche Skalen/Fragebögen sind geeignet, affektive Störungen (depressive Störungen, Anhedonie, affektive Fluktuationen, Apathie, Angststörungen, Progredienzangst, Fatigue) bei Parkinson-Pat. mit affektiven Störungen im Vergleich zu Parkinson-Pat. ohne affektive Störungen zu erfassen?

Welche Skalen/Fragebögen sind geeignet, bei Parkinson-Pat. eine depressive Störung leichter versus mittelgradige versus schwere Ausprägung zu differenzieren?

Anmerkung

Aufgrund der inhaltlichen Nähe der o.g. Fragen werden diese in einem gemeinsamen Unterkapitel abgehandelt.

Evidenzgrundlage

Als Grundlage der Empfehlungen für den Einsatz von standardisierten Verfahren zur Erfassung affektiver Störungen bei Parkinson-Pat. wurden die Empfehlungen der International Parkinson and Movement Disorder Society (MDS) berücksichtigt [35]. Zusätzlich wurden relevante Übersichtsarbeiten über den folgenden Suchstring ermittelt: *„Parkinson's disease", „neuropsychiatric", „review"* in Kombination mit *„questionnaire"* bzw. *„scale"* bzw. *„assessment"*. Hierbei wurden 5 bzw. 41 bzw. 81 Treffer erzielt, wobei drei relevante Übersichtsarbeiten zu neuropsychiatrischen Störungen bei PK identifiziert werden konnten, die auch Übersichten zu psychometrischen Skalen inkludieren [36], [37], [38]. In den Arbeiten von Mueller et al. (2018) [38] sowie Weintraub et al. (2022) [36] werden neuropsychiatrische Symptome bei Parkinson-Pat. überblicksartig dargestellt und Möglichkeiten der Diagnostik und Therapie erörtert. Martinez-Martin et al. (2016) [37] legten eine systematische Überblicksarbeit zur diagnostischen Akkuratheit von Screenings zur Erfassung neuropsychiatrischer Symptome bei PK-Pat. vor. Darüber hinaus fanden sich auch schon in den Leitlinien zur Diagnostik und Behandlung des Parkinson-Syndroms (2016) Hinweise zur Diagnostik unterschiedlicher affektiver Störungen bei Parkinson-Pat.; auch diese wurden hier herangezogen [39].

Da Fatigue in den genannten Überblicksarbeiten zu neuropsychiatrischen und affektiven Störungen bei Parkinson-Pat. weitgehend unberücksichtigt blieb, erfolgte eine separate Literatursuche mit folgendem Suchstring: *„Parkinson´s disease", „fatigue", „review"* in Kombination mit *„assessment", „questionnaire"* bzw. *„scale"*. Es gab hierbei 49 bzw. 15 bzw. 35 Treffer. Insgesamt wurden 4 Arbeiten als relevant erachtet [40], [30], [41], [42].

Ergebnis und Begründung für die Empfehlung

Allgemeine Hinweise zur Verwendung standardisierter Skalen in der Diagnostik affektiver Störungen bei Parkinson-Pat.

Wie bereits bei Fragestellung 130 erörtert, sollten für die Interpretation standardisierter Skalen in der Diagnostik affektiver Störungen stets auch Informationen aus Selbst- und ggf. Fremdanamnese berücksichtigt werden. Zusätzlich ist bei der Interpretation der Skalenergebnisse zu beachten, ob die Diagnostik im On- oder Off-Zustand erfolgte und ob weitere Symptome, wie z.B. kognitive Störungen, einen Einfluss auf die Diagnostik haben könnten.

Allgemeine Skalen zur Erfassung nicht motorischer und neuropsychiatrischer Symptome

Allgemeine Skalen bzw. Subskalen zur Erfassung nicht motorischer Symptome bei Parkinson-Pat. (Tab. 22) enthalten in der Regel einzelne oder mehrere Items zur Erfassung unterschiedlicher affektiver Symptome. Hierzu gehören folgende krankheitsspezifische Instrumente, die von der MDS empfohlen werden und auch in deutschen Versionen verfügbar sind: Movement Disorder Society Unified Parkinson's Disease Rating Scale, Teil 1 (UPDRS-I), Movement Disorder Society Non-Motor Rating Scale (MDS-NMS), Non-Motor Symptom Scale (NMSS), Non-Motor Symptom Questionnaire (NMSQ). Ein weiteres weit verbreitetes und auch in deutscher Version verfügbares krankheitsunspezifisches Instrument ist das Neuropsychiatrische Inventar (NPI). Die Beurteilung der Symptomatik fußt bei all diesen Skalen jedoch auf einzelnen oder wenigen Items, die nur teilweise als Subskala ausgewertet werden, teilweise aber auch ohne gesonderte Wertung in einen Gesamtwert nicht motorischer Symptome eingehen. Somit kann die Erfassung affektiver Störungen mit diesen Skalen eher als Einstiegsdiagnostik angesehen werden, um bei Bedarf weitere Skalen zur spezifischen Diagnostik anzuwenden.

Tab. 22: Allgemeine Skalen zur Erfassung nicht motorischer Symptome bei PK bzw. neuropsychiatrischer Symptome mit Items zu affektiven Störungen

Skala	Erfasst depressive Störungen	Erfasst Apathie	Erfasst Angst-störungen	Erfasst Fatigue	Empfohlen von
Movement Disorder Society Unified Parkinson's Disease Rating Scale, Teil 1 (UPDRS-I)	X	X	X	X	[36]<br>[38]<br>[37]
Movement Disorder Society Non-Motor Rating Scale (MDS-NMS)	X	X	X	(X)<br>keine eigene Subskala	[36]
Non-Motor Symptom Scale (NMSS)	(X)<br>keine eigene Subskala			(X)<br>keine eigene Subskala	[36]<br>[38]
Non-Motor Symptom Questionnaire (NMSQ)	(X)<br>keine eigene Subskala		(X)<br>keine eigene Subskala	(X)<br>keine eigene Subskala	[36]
Neuropsychiatrisches Inventar (NPI)	X	X	X		[36]<br>[38]<br>[37]

Anmerkung: Es wurden folgende relevante Übersichtsarbeiten zugrunde gelegt: [36], [38], [37].

(Halb-)strukturierte klinische Interviews

Den Goldstandard zur Diagnosestellung affektiver Störungen bei Parkinson-Pat. stellt laut MDS-Empfehlung die Durchführung eines (halb-)strukturierten Interviews auf Basis der DSM-Kriterien (vgl. Strukturiertes Klinisches Interview für DSM-5®-Störungen, SCID-5) dar (wie z.B. für die Diagnostik depressiver Störungen definiert [31]). Eine Umsetzung dieses Standards ist in klinischen Settings selten gegeben.

Spezifische Skalen zur Erfassung affektiver Symptome

Für die verschiedenen affektiven Symptome stehen eine Vielzahl von Skalen zur Verfügung. Hierbei handelt es sich vorrangig um Selbsteinschätzungsfragebögen. Ferner stehen Fremdeinschätzungsskalen (z.B. Hamilton Rating Scale for Depression (HAM-D); Montgomery-Asberg Depression Rating Scale (MADRS)) zur Verfügung, die in der klinischen Routine insbesondere dann wertvolle Informationen liefern, wenn keine validen Aussagen durch die Pat. aufgrund fortgeschrittener kognitiver Störungen möglich sind. Selbst- und Fremdeinschätzungsskalen haben bei der Detektion von depressiven Störungen bei PK einen in etwa vergleichbaren Nutzen [31], [43].

Die MDS empfiehlt die folgenden Skalen [44], die an Parkinson-Pat. validiert worden sind. Es sind nur Instrumente gelistet, für die Validierungsstudien für die deutsche Version vorliegen:

Depressive Störungen

- Beck Depressions-Inventar II (BDI-II)
- Cornell Scale for Depression in Dementia (CSDD)
- Geriatrische Depressionsskala (GDS-15)
- Hamilton Rating Scale for Depression (HAM-D)
- Hospital Anxiety and Depression Scale (HADS)
- Montgomery-Asberg Depression Rating Scale (MADRS)

In der Praxis hat die GDS-15 als Screeninginstrument einen Vorteil bezüglich der Kürze, günstiger psychometrischer Eigenschaften und des fehlenden Urheberrechtsschutzes.

Anhedonie

- Snaith-Hamilton Pleasure Scale (SHAPS; [6])

Affektive Fluktuationen

- keine Empfehlung der MDS

Apathie

- keine deutschsprachigen Versionen der empfohlenen Skalen verfügbar

Angststörungen

- Keine Empfehlungen der MDS. Zwar enthält die von der MDS zur Erfassung depressiver Störungen empfohlene Hospital Anxiety and Depression Scale (HADS) eine Subskala zu Angststörungen, sie wird aber auf Basis der Datenlage lediglich als mögliche Skala vorgeschlagen, erhält aber nicht den Status „empfohlen“ [45]. Dasselbe gilt für das Beck-Angst-Inventar (BAI) und die Hamilton-Angst-Skala (HAMA).

Fatigue

- Fatigue Severity Scale (FSS)
- Multidimensional Fatigue Inventory (MFI)

Darüber hinaus geben neuere Übersichtsarbeiten zusätzliche Empfehlungen zu Skalen zur Erfassung affektiver Störungen [36], [37], [38]. Auch hier sind nur Instrumente gelistet, für die Validierungsstudien für die deutsche Version vorliegen:

Depressive Störungen

- Center for Epidemiologic Studies Depression Rating Scale – Revised (CESD-R)
- Inventory for Depressive Symptoms (IDS)
- Zwei-Fragen-Test
- Patient Health Questionnaire (PHQ-9)
- Unified Parkinson’s Disease Rating Scale (UPDRS), Teil 1, Fragen zu depressiven Störungen
- WHO-5 Well-being Index (WHO-5)

Apathie

- Apathy Evaluation Scale (AES)

Angststörungen

- keine Empfehlungen für Instrumente, die in einer validierten deutschen Version vorliegen. Die beiden in einer Übersichtsarbeit aus dem Jahr 2016 [37] genannten Skalen Parkinson’s Anxiety Scale (PAS) und Geriatric Anxiety Inventory (GAI) wurden in die deutsche Sprache übersetzt, in dieser Form jedoch bislang nicht validiert.

Fatigue

- Functional Assessment of Chronic Illness Therapy – Fatigue (FACIT-F)
- Fatigue Impact Scale for Daily Use (D-FIS)

Die oben angegebenen Quellen enthalten keine Empfehlungen zur Erfassung der Progredienzangst. Progredienzangst kann über die Kurzform des Progredienzangst-Fragebogens (PA-F) erhoben werden [46]. Allerdings liegen bis dato keine diagnostischen Kriterien für eine dysfunktionale Progredienz vor, sodass die Differenzierung zwischen funktionaler und dysfunktionaler Progredienzangst und ihre Abbildung über Skalen bislang nicht möglich sind und dem klinischen Urteil obliegen.

Empfehlungen für ein regelmäßiges Screeningpaket

Weintraub et al. (2022) [36] empfehlen, regelmäßig, d.h. alle 6 bis 12 Monate, ein „Screeningpaket" bei Parkinson-Pat. durchzuführen, um mögliche affektive Störungen zu monitoren. Hier wird empfohlen, das Fragebogenpaket den Parkinson-Pat. im Vorfeld der neurologischen Routineuntersuchung postalisch zuzustellen. Die Autorinnen/Autoren schlagen folgende Skalen vor, wobei die letztgenannte Skala nicht als validierte deutsche Version vorliegt: Movement Disorder Society Unified Parkinson's Disease Rating Scale, Teil 1 (UPDRS-I), Geriatrische Depressionsskala, 15-Item-Version (GDS-15) und Parkinson's Anxiety Scale (PAS).

Differenzierung leichter versus mittelgradige versus schwere depressive Störungen

Den 2016 zuletzt aktualisierten S3-Leitlinien zur Diagnostik und Behandlung des Parkinson-Syndroms [39] folgend, können als Maß zur Differenzierung der Schwere depressiver Störungen die Skalen Beck-Depressions-Inventar II (BDI-II), Hamilton Rating Scale for Depression (HAM-D) und Montgomery-Asberg Depression Rating Scale (MADRS) empfohlen werden [31]; alle genannten Skalen liegen in einer deutschen validierten Version vor. Allerdings ist die Trennschärfe zwischen depressiven und motorischen Symptomen bei der Hamilton Rating Scale for Depression (HAM-D) gering [24]. Die Cornell Scale for Depression in Dementia (CSDD) könnte zur Beurteilung des Schweregrads einer depressiven Störung bei Parkinson-Pat. mit komorbider Demenz besonders nützlich sein. Für PK werden angepasste Cut-off-Scores vorgeschlagen [31], [43].

**Empfehlung: Skalen/Fragebögen zur Erfassung affektiver Störungen**	**Neu**<br>**Stand (2023)**

1. Zur Einstiegsdiagnostik affektiver Störungen sollten allgemeine Skalen bzw. Subskalen zur Erfassung nicht motorischer Symptome (hier spezifische Items bzw. Subskalen zu affektiven Störungen) bei Parkinson-Pat. angewendet werden:
   - Movement Disorder Society Unified Parkinson's Disease Rating Scale, Teil 1 (MDS-UPDRS-I)
   - Movement Disorder Society Non-Motor Rating Scale (MDS-NMS)
   - Non-Motor Symptom Scale (NMSS)
   - Non-Motor Symptom Questionnaire (NMSQ)
2. Zur Einstiegsdiagnostik affektiver Störungen können die entsprechenden Items aus dem folgenden Instrument verwendet werden:
   - Neuropsychiatrisches Inventar (NPI)
3. Zur spezifischen Diagnostik affektiver Störungen sollen spezifische und für Parkinson-Pat. validierte Skalen verwendet werden. Folgende Skalen eignen sich hierfür, werden im klinischen Alltag häufig verwendet und werden von den Autorinnen/Autoren empfohlen:
   - Depressive Störungen
     - Beck-Depressions-Inventar II (BDI-II)
     - Geriatrische Depressionsskala (GDS-15)
     - Zwei-Fragen-Test

- Anhedonie
  - Snaith-Hamilton Pleasure Scale (SHAPS)
- Apathie
  - Apathy Evaluation Scale (AES)
- Angststörungen
  - Hospital Anxiety and Depression Scale (HADS)[1]
- Fatigue
  - Fatigue Severity Scale (FSS)

4. Zur Erfassung von Progredienzangst kann folgender Fragebogen eingesetzt werden:
   - Kurzform des Progredienzangst-Fragebogens (PA-F)[2]
5. Bei vorliegenden depressiven Störungen kann zur Differenzierung des Schweregrads folgende Skala eingesetzt werden:
   - Beck-Depressions-Inventar II (BDI-II)

[1]Laut MDS besitzt diese Skala nur begrenzte Validität bei Parkinson-Pat.; valide deutschsprachige Alternativen fehlen bislang.

[2]Bislang wenig eingesetzt, aber aufgrund der Relevanz des Themas von den Autorinnen/Autoren empfohlen

Konsensstärke: 100%, starker Konsens

Fragestellung 132: Wie effektiv ist eine dopaminerge Therapie im Vergleich mit Placebo oder einem pharmakologischen Komparator (dopaminerg oder nicht dopaminerg) in der Behandlung von affektiven Störungen (depressive Störungen, Anhedonie, affektive Fluktuationen, Apathie, Angststörungen, Progredienzangst, Fatigue) bei Parkinson-Pat.?

Hintergrund

Die Optimierung der dopaminergen Ersatztherapie ist ein wesentlicher Faktor zur Behandlung nicht motorischer fluktuierender, aber auch nicht fluktuierender affektiver Symptome bei Parkinson-Pat. [24]. Dies gilt sowohl für depressive Störungen als auch für die Apathie, wobei Letztere einzeln oder in Kombination mit depressiven Störungen und Angstzuständen auftreten kann und pathophysiologisch am ehesten in erster Linie auf eine mesolimbische dopaminerge Denervierung zurückzuführen ist [47].

Evidenzgrundlage

Die Literatursuche in Pubmed brachte 1201 Treffer, doppelte Nennungen wurden ausgeschlossen. Für die Literaturrecherche wurde folgender Suchstring verwendet: (((((Parkinson disease [MeSH]) OR (Parkinson* [tiab])) OR (Parkinsonian disorders [MeSH])) OR (Movement disorders [MeSH])) NOT ((((Wolff parkinson white syndrome [MeSH]) OR (wolff[tiab] AND parkinson*[tiab])) OR (wpw[tiab] AND syndrome[tiab])) OR (wpw [tiab]))) AND ((((dopaminergic therapy[tiab] OR dopaminergic therapy[MeSH]) OR (dopaminergic treatment[tiab] OR dopaminergic treatment[MeSH]) OR

"Dopamine Agents/pharmacology"[MeSH]) AND ((((compar*[tiab]) OR (Placebo[tiab] OR Placebo[MeSH])) OR (active control[tiab])) OR (non-dopaminergic[tiab]))) AND ((((((((Affective disorder*[tiab] OR Affective disorder[MeSH]) OR (Anhedonia[tiab] OR Anhedonia[MeSH])) OR (Depression[tiab] OR Depression[MeSH])) OR (affective fluctuations)) OR (Apathy[tiab] OR Apathy[MeSH])) OR (anxiety disorder[tiab] OR Anxiety disorder[MeSH])) OR (fear of progression)) OR (fear of recurrence)) OR (Fatigue[tiab] OR Fatigue[MeSH]))) AND ((((humans[mesh:noexp]) NOT (animals[mesh:noexp])) AND (2016:2021[pdat])) AND ((english[Filter] OR german[Filter]))). Die so gefundenen Treffer wurden anhand des Titels und des Abstracts und im relevanten Fall auch im Volltext gelesen. Zur Behandlung einer Depression mittels Dopaminagonisten (DA) liegen 4 randomisierte, placebokontrollierte Studien vor. Die weiteren affektiven Syndrome wurden in Beobachtungsstudien als sekundäre Endpunkte untersucht oder auch in Metaanalysen zusammengefasst (39 Studien, Evidenzgrad 2 und 3).

Ergebnis

Depression

Die einmalige Gabe von Levodopa hat einen kurzfristigen antidepressiven Effekt [48]. Longitudinale Studien geben nur indirekte Hinweise auf einen antidepressiven Effekt von L-Dopa, z.B. wenn der Abbau von L-Dopa gehemmt wird und damit die Verfügbarkeit von L-Dopa steigt [49]. In der bislang größten randomisierten kontrollierten Studie (RCT) wurden N=287 Parkinson-Pat. 1 : 1 in die Therapie mit Pramipexol oder einem Placebo randomisiert. Nach 12 Wochen hatten sich die depressiven Symptome, operationalisiert mittels Beck-Depressions-Inventar (BDI), in der Pramipexol-Gruppe gegenüber der Placebo-Gruppe signifikant verbessert; dieses unter einem akzeptablen Sicherheitsprofil [50]. In drei Metaanalysen zeigte sich ein antidepressiver Effekt von Pramipexol bei PK [51], [52], [53]. Pramipexol war in einer Studie dem Ergot-Dopaminagonisten Pergolid bei der Behandlung einer Depression überlegen [54]. In einer RCT mit N=44 Parkinson-Pat. führten die Medikamente Pramipexol und Citalopram nach 8 Wochen zu einer vergleichbaren Reduktion im BDI [55]. Pramipexol und Sertalin reduzierten in einer nicht randomisierten Studie den Summenwert der Hamilton Depression Rating Skala über einen 14-wöchigen Studienverlauf im vergleichbaren Ausmaß. Pramipexol führte jedoch im Vergleich zu Sertalin häufiger zu einer Remission der Depression (60,6% vs. 27,3%) [56]. Diese Studienlage belegt eine antidepressive Wirkung von Pramipexol.

In einer RCT konnte eine antidepressive Wirkung für Ropinirol als sekundärer Studienendpunkt gezeigt werden [57]. Eine RCT mit dem Wirkstoff Rotigotin bei N=380 Parkinson-Pat. (1:1 randomisiert zu Placebo) war negativ [58]. Dies zeigte sich auch in einer weiteren RCT mit dem sekundären Endpunkt „Depression“ [59]. Subanalysen einer Beobachtungsstudie zeigten einen positiven Effekt von Rotigotin auf Symptome einer Depression [60], und zwei Metaanalysen unter Einschluss von 8 bzw. 10 RCTs zeigten ebenfalls einen positiven Effekt von Rotigotin auf Symptome einer Depression bei PK [61], [62]. Insgesamt ist die Studienlage daher für Ropinirol und Piribedil seitens der Behandlung einer Depression bei PK ungenügend.

Anhedonie

RCTs mit dem primären Endpunkt Anhedonie konnten in der o.g. Literaturrecherche nicht ausfindig gemacht werden. Anhedonie zählt zu den „hypodopaminergen“ Symptomen der PK [9]. Eine einmalige Gabe von L-Dopa führt bei PK-Pat. mit einem fortgeschrittenen Stadium zu einer Verbesserung der Anhedonie [48]. Des Weiteren berichten Beobachtungsstudien und Sekundäranalysen positive Effekte von Rotigotin, Pramipexol und Piribedil auf die Symptome einer Anhedonie [60], [63], [64], [65]. Zusammenfassend existieren Hinweise für positive Effekte von Non-Ergot-Dopaminagonisten auf Symptome einer Anhedonie, dieses aus Beobachtungsstudien oder Subanalysen.

Affektive Fluktuationen

RCTs mit dem primären Endpunkt affektive Fluktuationen konnten durch die Literaturrecherche nicht identifiziert werden. Zu diesem Thema gibt es Expertenempfehlungen, welche den Therapien der motorischen Fluktuationen entliehen sind und darauf abzielen, eine stabile Plasmakonzentration von L-Dopa und Non-Ergot-Dopaminagonisten zu realisieren [66].

Apathie

Eine Apathie bessert sich unter einer L-Dopa-Therapie [67]. N=22 Parkinson-Pat. wurden auf Pramipexol, Ropinirol, oder L-Dopa eingestellt. Es zeigte sich ein besserer Effekt von Pramipexol auf Symptome einer Apathie im Vergleich zu den beiden anderen Komparatoren [68]. In einer Meta-analyse und in einigen Fallberichten sowie Beobachtungsstudien zeigte sich ein positiver Effekt von Pramipexol und Rotigotin auf die Symptome einer Apathie [60], [63], [64], [65]. Wie eine RCT mit Placebo als Komparator zeigte, kann Piribedil erfolgreich gegen die Symptome einer Apathie nach einer Tiefen Hirnstimulation (STN-THS) eingesetzt werden [65]. Eine RCT zur Therapie der Apathie bei De-novo-Parkinson-Pat. mit Ropinirol war negativ [59].

Angststörungen

Angstsymptome im Rahmen von affektiven Fluktuationen wurden erfolgreich mit L-Dopa und Non-Ergot-Dopaminagonisten therapiert. Dieses zeigte sich in Fallberichten, Beobachtungsstudien und in zwei Metaanalysen [69], [70], [71]. Studien, die Angst im Sinne eines nicht fluktuierenden Symptoms als primären Endpunkt definieren, fehlen.

Fatigue

Im Rahmen der ELLDOPA-Studie erhielten N=361 De-novo-Parkinson-Pat. über 40 Wochen Placebo oder L-Dopa in unterschiedlicher Dosierung. Es zeigte sich für die L-Dopa-Gruppen ein deutlich geringerer Progress der Fatigue im Verlauf der Studie [72]. Dieser Befund passt zu der Beobachtung, dass L-Dopa Fatigue reduziert [73]. Fatigue ist somit ein hypodopaminerges Syndrom. Zwei Studien (davon eine RCT) berichten von einer Verbesserung des Fatigue-Ausmaßes unter Rotigotin [60], [74]. Zwei Metaanalysen unter Einschluss von 10 bzw. 8 Studien und insgesamt über N=1800 bzw. N=1675 Parkinson-Pat. berichten einen positiven Behandlungsaspekt von Rotigotin auf die Fatigue-Symptomatik [61], [62]. Pramipexol ist hinsichtlich der Wirkung auf die Fatigue-Symptomatik weniger gut untersucht. Studien geben Hinweise sowohl auf Verbesserungen als auch auf Verschlechterungen [75], [76].

Begründung der Empfehlung

Die hier dargestellte Zusammenfassung stellt fest, dass die Effektivität von L-Dopa und DA seitens der Symptomvielfalt affektiver Störungen sehr unterschiedlich untersucht ist. RCTs liegen zur Depression vor, Metaanalysen liegen zu den Themenfeldern Apathie und Fatigue vor, während Anhedonie und Angst nur im Rahmen von Beobachtungsstudien betrachtet wurden.

**Empfehlung**	**Neu**<br><br>**Stand (2023)**
Depressive Störungen<br><br>Zur Behandlung von depressiven Störungen bei Parkinson-Pat. soll die optimale dopaminerge Medikation eingesetzt werden und eine Therapie mit Pramipexol durchgeführt werden, falls eine Dopaminagonisten-Therapie individuell möglich ist. In zweiter Linie kann der Dopaminagonist Rotigotin eingesetzt werden.<br><br>*Anhedonie*<br><br>Zur Behandlung einer Anhedonie bei Parkinson-Pat. soll die optimale dopaminerge Medikation mit Levodoa und/oder Rotigotin, Pramipexol oder Piribedil eingesetzt werden.<br><br>*Affektive Fluktuationen*<br><br>Zur Behandlung von affektiven Fluktuationen bei Parkinson-Pat. soll die optimale dopaminerge Medikation und können Nicht-Ergot-Dopaminagonisten eingesetzt werden, ohne dass eine Überlegenheit eines Dopaminagonisten angegeben werden kann.<br><br>*Apathie*<br><br>Zur Behandlung der Apathie bei Parkinson-Pat. soll die optimale dopaminerge Medikation eingesetzt werden. Zusätzlich kann eine Therapie mit den Dopaminagonisten Pramipexol, Rotigotin oder Piribedil durchgeführt werden, falls eine Dopaminagonisten-Therapie individuell möglich ist.<br><br>*Angststörungen*<br><br>Zur Behandlung von Angststörungen mit affektiven Fluktuationen bei Parkinson-Pat. soll die optimale dopaminerge Medikation eingesetzt werden und eine Therapie mit einem Nicht-Ergot-Dopaminagonisten durchgeführt werden, falls eine Dopaminagonisten-Therapie individuell möglich ist.<br><br>Konstante Angststörungen ohne affektive Fluktuationen bei Parkinson-Pat. sollten nicht mittels einer angepassten dopaminergen Therapie behandelt werden.<br><br>*Fatigue*<br><br>Zur Behandlung von Fatigue bei Parkinson-Pat. soll die optimale dopaminerge Medikation eingesetzt werden. Zusätzlich kann eine Therapie mit Rotigotin durchgeführt werden, falls eine Dopaminagonisten-Therapie individuell möglich ist.	
Konsensstärke: 100%, starker Konsens	

Fragestellung 133: Wie effektiv ist die nicht dopaminerge Pharmakotherapie im Vergleich mit Placebo oder einem pharmakologischen Komparator (dopaminerg oder nicht dopaminerg) bzw. einer Kombinationstherapie in der Behandlung von affektiven Störungen (depressive Störungen, Anhedonie, affektive Fluktuationen, Apathie, Angststörungen, Progredienzangst, Fatigue) bei Parkinson-Pat.?

Hintergrund

Neben dem dopaminergen sind bei affektiven Störungen bei PK auch andere Neurotransmittersysteme betroffen. Hierbei spielt die serotonerge Degeneration bereits bei De-novo-Parkinson-Pat. eine prominente Rolle [77], [78]. Ferner wird diskutiert, dass das glutamaterge System u.a. auch eine Rolle bei der Modulation von Emotionen spielt und möglicherweise anti-glutamaterge Substanzen Depression oder Angstzustände bei PK verbessern könnten [79].

In der Nationalen VersorgungsLeitlinie für unipolare Depression [80] wird darauf hingewiesen, dass insbesondere Pat. mit schwerer Depression („Major Depression“) von einer antidepressiven Therapie profitieren, während dieser Effekt bei Personen mit leichter Depression („Minor Depression“) oder dysthymer Störung nicht so deutlich ist. Es ist unklar, ob diese für Pat. mit schwerer Depression ohne PK getroffene Einstufung auch auf Parkinson-Pat. angewendet werden kann. Darüber hinaus erfüllen die meisten Parkinson-Pat. mit Anzeichen einer Depression nicht die Kriterien für eine schwere Depression. Des Weiteren ist die klinische Datenlage zur Behandlung von affektiven Störungen bei Parkinson-Erkrankten mit nicht dopaminergen Substanzen sehr begrenzt [24].

Evidenzgrundlage

Die Literatursuche in Pubmed erbrachte 30 Studien mit dem folgenden Suchstring: ((((((Parkinson disease [MeSH]) OR (Parkinson* [tiab])) OR (Parkinsonian disorders [MeSH])) OR (Movement disorders [MeSH])) NOT ((((Wolff parkinson white syndrome [MeSH]) OR (wolff[tiab] AND parkinson*[tiab])) OR (wpw[tiab] AND syndrome[tiab])) OR (wpw [tiab])))) AND ((((((((((((((((non-dopaminergic therapy[tiab]) OR (non-dopaminergic treatment[tiab])) OR (non-Dopamine[tiab] AND (therapy[tiab] OR treatment[tiab])))) OR (adenosine*[tiab])) OR (Glutamatergic[tiab])) OR (adrenergic[tiab])) OR (noradrenergic[tiab])) OR (cholinergic [tiab])) OR (serotonergic[tiab])) OR (GLP-1 agonists[tiab])) OR (calcium channel blockers[tiab])) OR (iron chelators[tiab])) OR (anti-inflammatories[tiab])) OR (neurotrophic factor*[tiab])) OR (gene therap*[tiab])) AND ((((compar*[tiab]) OR (Placebo[tiab] OR Placebo[MeSH])) OR (active control[tiab])) OR (non-dopaminergic[tiab]) OR (combination therapy[tiab] OR combination therapy[MeSH]))) AND ((((((((Affective disorder*[tiab] OR Affective disorder[MeSH]) OR (Anhedonia[tiab] OR Anhedonia[MeSH])) OR (Depression[tiab] OR Depression[MeSH])) OR (affective fluctuations)) OR (Apathy[tiab] OR Apathy[MeSH])) OR (anxiety disorder[tiab] OR Anxiety disorder[MeSH])) OR (fear of progression)) OR (fear of recurrence)) OR (Fatigue[tiab] OR Fatigue[MeSH]))))) AND ((((humans[mesh:noexp]) NOT (animals[mesh:noexp])) AND (2016:2021[pdat])) AND ((english[Filter] OR german[Filter])). Inkludiert wurden nur Studien aus dem Zeitraum 2016 bis 2021, die in englischer und deutscher Sprache veröffentlicht wurden. Nach Screening der Titel und Abstracts sowie Volltexte wurden final 6 Publikationen als relevant identifiziert. Nach Erweiterung der Recherche, der Berücksichtigung der 2016 zuletzt aktualisierten S3-Leitlinie PK [39] (Kapitel 2.5.12. und 2.5.19), der

Einbeziehung der Expertinnen/Experten bekannten Publikationen und der Extraktion von Publikationen aus diesen Literaturreferenzen wurden letztendlich 17 Publikationen selektiert, welche in diese Leitlinie einbezogen wurden. Es fanden sich darunter 10 prospektive randomisierte, kontrollierte Studien (RCTs) zu der genannten Fragestellung, wobei sich zwei Literaturstellen [81], [82] auf dieselbe Studienpopulation beziehen, eine Metaanalyse [83], ein systematisches Review ohne Metaanalyse [84], eine Post-hoc-Analyse einer RCT mit Placebo-Komparator [85], zwei prospektive einarmige offene Studien [86], [87], ein Expertenkonsensus ([24] sowie die 2016 zuletzt aktualisierte S3-Leitlinie PK [39].

Ergebnis

Depressive Störungen/Angststörungen

Die 2016 zuletzt aktualisierte S3-Leitlinie PK [39] empfiehlt trizyklische Antidepressiva (TCA), Antidepressiva neuerer Generation wie selektive Serotonin-Reuptake-Hemmer (SSRIs) und Venlafaxin sowie Omega-3-Fettsäuren zur Behandlung der Depression bei PK .

Wie bereits in der S3-Leitlinie PK [39] beschrieben, zeigten die wesentlichen Ergebnisse der 2010 publizierten Metaanalyse [83] keinen Unterschied in der Behandlung der Depression bei PK zwischen selektiven Serotonin-Wiederaufnahmehemmern (SSRI) und sowohl Placebo als auch trizyklischen Antidepressiva (TZA). Eine systematische Übersichtsarbeit [84] ergab, dass für die Behandlung depressiver Störungen bei Parkinson-Pat. die TZA Nortriptylin (zwei RCTs vorliegend; [81], [88]) und Desipramin (1 RCT vorliegend; [89]) wahrscheinlich wirksam sind.

In der Studie von Menza et al. [81] war Nortriptylin, nicht aber Paroxetin gegenüber Placebo in einem 8-wöchigen RCT effektiv in der Verbesserung der depressiven Symptomatik bei Parkinson-Betroffenen und sekundärer Endpunkte wie Schlaf, Angst und soziale Funktion. In der 16-wöchigen, verblindeten Extensionsphase war das Auftreten eines Rezidivs signifikant häufiger unter Placebo als unter Nortriptylin und Paroxetin [82]. In der RCT von Devos et al. [89] trat die antidepressive Wirkung von Desipramin nach 14 Tagen früher ein als die von Citalopram nach 30 Tagen, wobei allerdings leichte unerwünschte Ereignisse (insbesondere Mundtrockenheit und Obstipation) in der Desipramin-Gruppe doppelt so häufig auftraten wie bei Citalopram oder Placebo. Zudem konnte in einer weiteren RCT ohne Placebo-Arm gezeigt werden, dass niedrig dosiertes Amitriptylin und Sertralin einen ähnlichen guten antidepressiven Effekt aufwiesen [90].

Ein neue RCT über 4 Wochen, die nicht in den o.g. Metaanalysen berücksichtigt wurde, zeigte an letztlich 23 Parkinson-Pat., dass die Behandlung mit 50 mg 5-Hydroxytryptophan (5-HTP), welches ein Zwischenmetabolit von L-Tryptophan bei der Produktion von Serotonin ist, signifikante Verbesserungen der depressiven Symptomatik (operationalisiert mittels der Hamilton Depression Rating Scale [HAM-D]) im Vergleich zu Placeboerbrachte. Keine signifikante Effektivität zeigte sich in Bezug auf das Beck-Depressions-Inventar II (BDI-II). Ebenso zeigte sich keine Wirkung von 5-HTP auf die durch die Apathy Scale (AS) beurteilte Apathie-Symptomatik [91].

Interessanterweise zeigte eine 12-wöchige RCT mit Omega-3-Fettsäuren gegenüber Placebo eine signifikante Reduktion der Depressionswerte [92].

Depressive Störungen/Apathie

Safinamid ist eine primär über die Monoaminoxidase-B (MAO-B)-Hemmung dopaminerg wirksame Substanz. Sie hat aber auch einen zweiten, weniger prominenten Wirkmechanismus in Form der Hemmung der abnormalen Glutamat-Freisetzung.

Die Effektivität von Safinamid auf nicht motorische Parkinson-Symptome, einschließlich affektiver Störungen, wurde im Rahmen einer Post-hoc-Analyse der Daten aus zwei RCT-Zulassungsstudien untersucht. Hier zeigte sich in der Safinamid-Gruppe gegenüber der Placebogruppe ein signifikanter Einfluss auf die Lebensqualität (PDQ-39-Subskala „Emotionales Wohlbefinden“, N=418 bzw. N=428 Pat.) und die depressive Symptomatik (GRID-HAMD, N=353 bzw. N=404 Pat.) nach 6 und 24 Monaten [85]. Eine prospektive einarmige offene (nicht kontrollierte) Studie zur Wirksamkeit von Safinamid auf nicht motorische Symptome zeigte über einen Beobachtungszeitraum von 6 Monaten an letztlich N=44 Parkinson-Pat. eine signifikante Verbesserung der Stimmung und Apathie-Symptomatik bei insgesamt guter Verträglichkeit der Substanz [86]. Ob dieser Effekt im Wesentlichen durch die nicht dopaminerge Modulation der glutamatergen Hyperaktivität bedingt ist oder der primär dopaminergen reversiblen MAO-B-Hemmung zuzuschreiben ist, bleibt offen.

Anhedonie

Zur Behandlung der Anhedonie existieren keine RCTs.

Fatigue

Bezüglich des Fatigue-Syndroms bei PK zeigte die Behandlung mit Methylphenidat 30 mg pro Tag in einer RCT mit N=36 Pat. über 6 Wochen eine signifikante Reduktion in den beiden primären Endpunkten Fatigue Severity Scale (FSS) und Multidimensionales Fatigue Inventory (MFI) [93]. In der S3-Leitlinie [39] wird jedoch bemängelt, dass eine Gruppenanalyse (Verum vs. Placebo) nicht durchgeführt wurde und statistisch keine Korrektur für multiples Testen hinsichtlich multipler Endpunkte berücksichtigt wurde, sodass keine Empfehlung zur Behandlung des Fatigue-Syndroms mit Methylphenidat ausgesprochen wurde. Neuere Studien zu der Fragestellung liegen nicht vor.

Der Effekt von Modafinil zur Behandlung der Fatigue-Symptomatik bei PK wurde in zwei RCTs untersucht, die auch in ein systematisches Review inkludiert wurden [84]. Da beide Studien nur kleine Fallzahlen einschlossen (N=19 bzw. N=13) und keine primären Zielparameter definierten [94], [95], wurde in der S3-Leitlinie [39] Modafinil nicht zur Behandlung des Fatigue-Syndroms bei PK empfohlen. Neuere Studien zu der Fragestellung liegen nicht vor.

Die Behandlung von 39 Parkinson-Pat. mit Fatigue und Wirkfluktuationen mit Safinamid als Add-on-Therapie über 24 Wochen zeigte bei Studienende im Vergleich zu Studienbeginn eine Verbesserung der Fatigue gemäß den validierten Versionen der Fatigue Severity Scale (FSS) und Parkinson Fatigue Scale-16 (PFS-16). 46,2 % (FSS) bzw. 41 % (PFS-16) der Pat. waren bei Studienende Fatigue-frei [87].

Begründung der Empfehlung

Zusammenfassend lässt sich feststellen, dass die Effektivität der nicht dopaminergen Pharmakotherapie seitens der Symptomvielfalt affektiver Störungen sehr unterschiedlich untersucht

ist. RCTs unterschiedlicher Güte liegen zur Depression und zur Fatigue vor, eine Post-hoc-Analyse und eine offene nicht kontrollierte Studie liegen zur Apathie vor, eine offene nicht kontrollierte Studie zur Fatigue. Angst wurde als sekundärer Endpunkt in RCTs untersucht, während Anhedonie nicht adressiert wurde.

Aufgrund der o.g. Evidenzlage schlagen die Autorinnen und Autoren dieses Kapitels als Expertenkonsensusmeinung vor, affektive Fluktuationen ähnlich motorischer Fluktuationen durch eine Optimierung der dopaminergen Therapie zu behandeln. Eine Optimierung der dopaminergen Therapie bildet auch die Basis der nicht fluktuierenden affektiven Störungen wie Anhedonie, Depression, Apathie, Angststörung oder Fatigue. Möglicherweise bietet eine Optimierung der dopaminergen Therapie mit dem auch antiglutamaterg wirksamen Safinamid einen zusätzlichen Nutzen in der Behandlung von Depression, Apathie und Fatigue.

Antidepressiva mit anticholinergen Eigenschaften gehen insbesondere bei älteren und kognitiv eingeschränkten Parkinson-Pat. mit einem erhöhten Risiko für eine Verschlechterung der kognitiven Funktionen, eine Auslösung von Halluzinationen oder Psychosen, eine Verstärkung der Obstipation und einem erhöhten kardiovaskulären Risiko einher und sollten nur mit großer Vorsicht angewendet werden [24].

Von den TZA erscheint Desipramin aufgrund der Studienlage, der relativ selektiven Noradrenalin-Wiederaufnahmehemmung (NRI) mit der unter den TZA geringsten anticholinergen und antihistaminischen Wirkung am ehesten geeignet für den Einsatz bei PK. Allerdings ist das Produkt in der EU nicht als Fertigarzneimittel im Handel, kann aber als Fertigarzneimittel Norpramin über die internationale Apotheke aus den USA in den Stärken 10, 25, 50, 100 oder 150 mg bezogen werden. Nortriptylin als TZA mit der besten Datenlage bei PK wirkt hauptsächlich als Serotonin- und Noradrenalin-Wiederaufnahmehemmer (SNRI), hat im Vergleich zu anderen TZA die höchste Affinität zum Dopamintransporter (DAT), jedoch trotzdem nur eine schwache Dopamin-Wiederaufnahmehemmung (DRIs) und wirkt aufgrund seiner zusätzlichen Antagonisierung von H1- und 5-HT2A-Rezeptoren auch sedierend bzw. schlafverbessernd. Allerdings zeigt Nortriptylin wie die meisten anderen TZA (und im Gegensatz zu Desipramin) auch eine hohe Affinität zur Antagonisierung muskarinischer Acetylcholinrezeptoren und wirkt dadurch stark anticholinerg. Deshalb und wegen der potenziellen Kardiotoxizität aufgrund der TZA-typischen Natrium- bzw. Kalziumkanal blockenden Wirkung ist der Einsatz von Nortriptylin für Parkinson-Pat. problematisch.

Obwohl es theoretische Argumente für den Einsatz von Noradrenalin-/Serotonin-Wiederaufnahmehemmern gibt, sind die wenigen kontrollierten Studien nicht zuletzt aufgrund der geringen Fallzahl der untersuchten Pat. nur unter Vorbehalt zu bewerten. Trotz vermutlich im Vergleich zu TZA etwas geringerer Wirkstärke erscheinen SSRI/NRI möglicherweise für Parkinson-Betroffene aufgrund des vergleichsweise geringeren Nebenwirkungsprofils vorteilhafter.

Für die Effektivität des SSRI Paroxetin ist die Datenlage kontrovers, der SSRI Citalopram und der SNRI Venlafaxin retard wurden als wirksam gefunden. 5-Hydroxytryptophan (5-HTP) könnte einen klinischen Nutzen in der Behandlung depressiver Symptome bei PK haben. Insgesamt sind größere Studien mit einer längeren Behandlungsdauer jedoch erforderlich, bevor eine Empfehlung abgegeben werden kann. Die Kombination eines SSRI mit einem irreversiblen MAO-B-Hemmer wie

Selegilin wird aufgrund des Risikos der Auslösung eines serotonergen Syndroms nicht empfohlen. Wie bei der Behandlung affektiver Störungen bei anderen Erkrankungen empfohlen, sollte eine medikamentöse Therapie auch bei Parkinson-Erkrankten mit einer Psychotherapie kombiniert werden. Theoretisch erscheint der Einsatz von Trazodon als dual wirksamer serotonerger Wirkstoff mit präsynaptischer Hemmung der Serotonin-Wiederaufnahme und postsynaptischer Blockade der 5HT2A-Rezeptoren, starker Affinität zu zentralen alpha-1-Rezeptoren, schwacher antagonistischer Affinität zu H1-Rezeptoren und fehlender anticholinerger Wirkung ebenfalls interessant. Allerdings weist die vorhandene Studie aufgrund der geringen Fallzahl (N=22) trotz längster Studiendauer (5 Monate), des fehlenden Einschlusskriteriums „Vorliegen einer depressiven Störung" und des Fehlens eines wahren Placeboarms methodische Mängel auf.

Empfehlung

In dieser Empfehlung werden nicht nur die Ergebnisse der RCTs bewertet, sondern es wird auch das Wirksamkeitsprofil der Antidepressiva berücksichtigt, welches psychiatrischen Studien entliehen und im klinischen Kontext bezogen auf die PK von den Autorinnen/Autoren bewertet wurde.

**Empfehlung: nicht dopaminerge Therapie zur Behandlung der Depression**	**Neu**<br>**Stand (2023)**
Die Behandlung der schweren Depression bei Parkinson-Pat. kann erfolgen mit:<br>▪ Venlafaxin 75 bis 225 mg,<br>▪ Desipramin 25 bis 200 mg<br>Die Behandlung der mittelgradigen Depression bei Parkinson-Pat. kann folgendermaßen erfolgen:<br><u>bei Antriebshemmung</u><br>▪ Venlafaxin 75 bis 150 mg<br>▪ Citalopram 20 bis 40 mg, oder<br>▪ Sertralin 50 bis 100 mg<br><u>bei Agitiertheit, Angst, Unruhe oder Schlafstörung</u><br>▪ Mirtazapin 15 bis 45 mg (nicht bei REM-Schlaf-Verhaltensstörung) oder<br>▪ Trazodon 100 bis 200 mg<br><u>bei Komorbidität von Schlafstörung, Schmerz, Speichelfluss und kognitiv unauffälligen Pat.</u><br>▪ Amitriptylin 10 bis 75 mg retard.	
Konsensstärke: 100%, starker Konsens	

**Empfehlung: nicht dopaminerge Therapie zur Behandlung der Anhedonie**	**Neu**<br>**Stand (2023)**
Es gibt keine geprüfte nicht dopaminerge Medikation für die Anhedonie als isoliertes Symptom. Eine Empfehlung kann nicht ausgesprochen werden.	
Konsensstärke: 100%, starker Konsens	

**Empfehlung: nicht dopaminerge Therapie zur Behandlung der Apathie**	**Neu**<br>**Stand (2023)**
Die Apathie bei der PK kann mit folgender Medikation behandelt werden:<br>▪ Venlafaxin 75 bis 225 mg retard oder<br>▪ Nortriptylin 25 bis 150 mg.	
Konsensstärke: 96,2%, starker Konsens	

**Empfehlung: nicht dopaminerge Therapie zur Behandlung der Fatigue**	**Neu**<br>**Stand (2023)**
▪ Es gibt keine ausreichend geprüfte nicht dopaminerge Medikation gegen Fatigue als isoliertes Symptom.<br>▪ Es kann ein Therapieversuch mit Modafinil 100 bis 200 mg oder Safinamid 100 mg erwogen werden.	
Konsensstärke: 96,2%, starker Konsens	

**Empfehlung: nicht dopaminerge Therapie zur Behandlung der Angst/Panik**	**Neu**<br>**Stand (2023)**
▪ Es gibt keine ausreichend geprüfte nicht dopaminerge Medikation gegen Angst/Panik als isoliertes Symptom.<br>▪ Es kann ein Therapieversucht mit Citalopram 20 bis 40 mg unternommen werden.	
Konsensstärke: 95%, Konsens	

Fragestellung 134: Wie effektiv ist eine nicht pharmakologische Therapie (Psychotherapie, Ergotherapie, physische Interventionen) im Vergleich mit Warteliste oder einem aktiven Komparator (pharmakologisch oder nicht pharmakologisch) bzw. einer Kombinationstherapie in der Behandlung von affektiven Störungen (depressive Störungen, Apathie, Angststörungen, Progredienzangst, Fatigue) bei Parkinson-Pat.?

Aufbau des Kapitels und Hintergrund

Dieses Unterkapitel hat die Wirksamkeit nicht pharmakologischer Therapien in der Behandlung von affektiven Störungen bei Parkinson-Pat. zum Gegenstand. Da sich die Literatursuchen und die Evidenz für die verschiedenen affektiven Störungen unterscheiden, erfolgt die Darstellung störungsspezifisch.

Die Auswahl nicht pharmakologischer Therapieoptionen, die in diesem Leitlinienkapitel diskutiert werden, richtet sich nach Ansätzen, die aktuell ärztlich verordnet werden bzw. die von den Krankenkassen übernommen bzw. teilweise erstattet werden. Zu beachten ist hierbei allerdings, dass spezifische Angebote (z.B. Tanzen) hierfür als Rehasport zertifiziert sein müssen. Entsprechende Angebote sind bislang nur punktuell verfügbar.

Fragestellung 135: Depression: Wie effektiv sind psycho- und ergotherapeutische Ansätze sowie physische Interventionen im Vergleich mit aktiven und passiven Kontrollgruppen in der Behandlung von depressiven Störungen bei Parkinson-Pat.?

Evidenzgrundlage

- Psychotherapie: N=3 Metaanalysen
- Ergotherapie: N=0 Metaanalysen, 1 randomisierte kontrollierte Studie
- Physische Interventionen: N=8 Metaanalysen

Eine Literatursuche in Pubmed mit den Suchbegriffen *„Parkinson's disease" AND „depression" AND „systematic review"* ergab 176 Treffer, wobei schließlich 11 systematische Reviews mit Meta-analysen aus dem Zeitraum 2015 bis 2022 sowie eine RCT aus dem Jahr 2014 als Evidenzgrundlage berücksichtigt werden konnten. Zusätzlich fand Nationale VersorgungsLeitlinie für unipolare Depression [80] Eingang in die Beurteilung der Evidenzgrundlage und die Erarbeitung von Therapieempfehlungen.

Ergebnis

Psychotherapie

Unter den psychotherapeutischen Interventionen lassen sich verschiedene Schulen subsumieren. Eine gängige Einteilung ist die Unterscheidung der (kognitiven) Verhaltenstherapie (KVT), der psychodynamischen Therapien sowie der systemischen Therapie. Die Literatursuche identifizierte zwei Metaanalysen, die Effekte der KVT analysierten [96], [97] sowie eine Metaanalyse, die sowohl Effekte der KVT als auch psychodynamische Ansätze berücksichtigte [98].

In der Metaanalyse von Bomasang-Layno et al. [99] wurden Effekte pharmakologischer und nicht pharmakologischer Interventionen aus insgesamt 20 RCTs auf die Depression analysiert. Hierbei konnten auch 5 RCTs zu Effekten von Psychotherapie inkludiert werden (4 KVT, 1 Psychodrama). Die Effekte der Psychotherapieinterventionen im Vergleich zu Kontrollgruppen waren signifikant und groß; im Vergleich dazu zeigten Antidepressiva (10 RCTs) einen mittleren Effekt.

In der Metaanalyse von Xie et al. [98] wurden 10 Studien mit N=724 Parkinson-Pat. eingeschlossen, die KVT oder psychodynamische Therapie erhielten. Die Psychotherapie-Interventionen waren den Kontrollgruppen im Effekt auf die Depression signifikant überlegen. Subgruppenanalysen ergaben, dass Behandlungen von über 6 Wochen effektiver waren als kürzere Behandlungszeiträume.

Zhang et al. [96] inkludierten sieben RCTs mit insgesamt N=191 Parkinson-Pat. und fanden eine Überlegenheit der KVT gegenüber aktiven bzw. passiven Kontrollgruppen auf Depression mit einem signifikanten mittleren Effekt.

Luo et al. [97] konnten in ihrer Metaanalyse 13 Studien mit insgesamt N=507 Pat. inkludieren, die KVT erhalten hatten; insgesamt zeigte sich ein großer signifikanter Effekt auf die depressive Symptomatik im Vergleich zu Kontrollgruppen. Weiterhin wurde ermittelt, dass längere Interventionen von 8 oder mehr Wochen einen größeren Effekt erbrachten als kürzere Interventionsdauern. Während der Vergleich von online und offline durchgeführten Interventionen keinen Unterschied zeigte, waren individuell durchgeführte Interventionen signifikant effektiver als Gruppenangebote.

Laut der Nationalen VersorgungsLeitlinie für unipolare Depression (2022) [80] werden abhängig davon, wie viele Symptome auftreten, wie schwer sie ausgeprägt sind und wie stark sie die Pat. einschränken, unterschiedliche Interventionen empfohlen. Bei einer ersten leichten depressiven Episode sollen zunächst niedrigschwellige Interventionen wie beratende Gespräche oder Online-Programme zur Anwendung kommen. Bei mittelschweren Depressionen oder bei rezidivierenden leichten Episoden können eine Psychotherapie oder eine medikamentöse Behandlung in Erwägung gezogen werden. Bei schweren Depressionen sollen beide genannten Ansätze kombiniert angeboten werden.

Schließlich zeigten Wang et al. [100] in einer rezenten Netzwerk-Metaanalyse zu Effekten nicht pharmakologischer Interventionen auf Depression bei Personen mit PK, in 62 Studien mit N=3050 Pat., dass KVT nach Tanzen und LSVT-BIG den stärksten Effekt hat.

Ergotherapie

Aufgrund der nur wenigen Studien zu Ergotherapie bei Parkinson-Pat., die den Endpunkt Depression berücksichtigt haben, liegen keine Metaanalysen vor. Sturkenboom et al. [101] konnten in ihrer RCT mit N=191 Parkinson-Pat. keinen Effekt von häuslicher Ergotherapie im Vergleich zu einer passiven Kontrollgruppe auf die depressive Symptomatik bei Parkinson-Pat. finden.

Physische Interventionen

In einer Metaanalyse von Tian et al. [102] konnten 14 RCTs mit insgesamt N=516 Parkinson-Pat. und unterschiedlichen physischen Interventionen (z.B. Krafttraining, Laufbandtraining, Yoga, Tanzen)

eingeschlossen werden. Physische Interventionen erwiesen sich mit einem signifikanten moderaten Effekt den Kontrollgruppen gegenüber in der Wirkung auf Depression überlegen. Yang et al. [103] konnten 34 Studien mit 2007 Parkinson-Pat. inkludieren, die Depression als Endpunkt hatten; die Metaanalyse zeigte im Vergleich zu Kontrollgruppen, dass die depressive Symptomatik signifikant verbessert werden konnte; Tanzen schnitt hierbei besonders gut ab.

Gollan et al. [104] inkludierten drei RCTs mit insgesamt 237 Parkinson-Pat. in ihre Metaanalyse zu Krafttraining im Vergleich zu physisch aktiven Kontrollgruppen. Es zeigte sich kein signifikanter Effekt auf die depressive Symptomatik; allerdings zeigte eine Subgruppenanalyse eine signifikante Überlegenheit von Körper-Geist-Training im Vergleich zu Krafttraining.

Es liegen zwei Metaanalysen zu spezifischen Effekten von Tanzen bei PK vor, die Depression als Endpunkt analysierten und zu vergleichbaren Ergebnissen kamen: Wang et al. [105], die 6 RCTs mit N=165 Parkinson-Pat. untersuchten, und Zhang 2019 [106] mit 5 Studien und N=136. Bei beiden Metaanalysen ergaben sich keine signifikanten Effekte. Allerdings exkludierten beide Studien bei einer Sensitivitätsanalyse dieselbe RCT und fanden dann einen signifikanten positiven Effekt auf den Endpunkt Depression. Bemerkenswerterweise zeigten Wang et al. [100] in einer rezenten Netzwerk-Metaanalyse zu Effekten nicht pharmakologischer Interventionen auf Depression bei Personen mit PK, in 62 Studien mit N=3050 Pat, dass Tanzen den stärksten Effekt hat.

Drei Metaanalysen zur Untersuchung der spezifischen Effekte von Körper-Geist-Intervention (z.B. Yoga, Qigong) wurden identifiziert. Während Wang et al. 2021 [107] bei fünf inkludierten RCTs mit N=123 Pat. keinen signifikanten positiven Effekt der Interventionen auf Depression fanden, zeigten Song et al. 2017 [108] mit bereits 15 RCTs und N=755 Pat. und dann Jin et al. 2020 [109] mit 22 Studien und N=1199 Parkinson-Pat. signifikante Verbesserungen der Depression im Vergleich zu den Kontrollgruppen.

Metaanalysen zum Vergleich verschiedener Interventionen

Wang et al. [100] führten eine Netzwerk-Metaanalyse zur Fragestellung durch, welche nicht pharmakologischen Interventionen einen positiven Effekt auf depressive Symptome bei Parkinson-Pat. haben. Sie inkludierten 62 Studien mit N=3050 Pat. Die meisten Interventionen zeigten keine signifikanten Effekte, jedoch schnitt Tanzen am besten ab, gefolgt von LSVT-BIG-Therapie und KVT. Der Mangel an Effekten der nicht pharmakologischen Interventionen, die sich in anderen Studien als effektiv herausgestellt haben, wurde u.a. mit der konfundierenden antidepressiven Pharmakotherapie begründet.

Begründung der Empfehlung

Die vier berücksichtigten Metaanalysen zu psychotherapeutischen Interventionen zur Behandlung von Depression bei Parkinson-Pat. demonstrieren eine überzeugende Evidenz für die Effekte von KVT. Auch laut der Nationalen VersorgungsLeitlinie für unipolare Depression (2022) [80] soll Psychotherapie angeboten werden (allerdings kommen hiernach alle Richtlinienverfahren infrage) – bei schweren Depressionen stets in Kombination mit einer medikamentösen Behandlung, bei mittelschweren Depressionen oder bei rezidivierenden leichten Episoden als Alternative zu

medikamentöser Behandlung. Aufgrund der vorwiegend für die KVT vorliegende Evidenz bei Parkinson-Pat. wird die Empfehlung hier nur für dieses Verfahren formuliert.

Für die Ergotherapie besteht aktuell keine Evidenz zu Behandlungseffekten auf Depression bei Parkinson-Pat., sodass keine Empfehlungen ausgesprochen werden.

Die zwei berücksichtigten übergreifenden Metaanalysen zu physischen Interventionen sowie mehrere Metaanalysen zu spezifischen physischen Interventionen zur Behandlung von Depression bei Parkinson-Pat. zeigen eine überzeugende Evidenz für die Wirksamkeit. Die Nationale VersorgungsLeitlinie für unipolare Depression (2022) [80] empfiehlt, Pat. mit einer depressiven Störung zu sportlichen Aktivitäten zu motivieren, idealerweise innerhalb einer Gruppe; auch sollen sie zur Teilnahme an einem strukturierten und supervidierten körperlichen Training motiviert und bei der Umsetzung unterstützt werden; allerdings soll hierfür keine Kontraindikation für körperliche Belastungen bestehen. Bei PK sollte das Sturzrisiko bei der Wahl und Intensität der körperlichen Aktivität besonders berücksichtigt werden.

**Empfehlung: Psychotherapie bei depressiven Störungen**	**Neu**<br><br>**Stand (2023)**
Kognitive Verhaltenstherapie soll Parkinson-Pat. zur Behandlung depressiver Störungen angeboten werden.	
Konsensstärke: 100%, starker Konsens	

**Empfehlung: physische Interventionen bei depressiven Störungen**	**Neu**<br><br>**Stand (2023)**
Physische Interventionen sollen Parkinson-Pat. mit depressiven Störungen angeboten werden.	
Konsensstärke: 100%, starker Konsens	

Wichtige Forschungsfragen

Zur Behandlung depressiver Störungen bei Parkinson-Pat. ergeben sich folgende wichtige Forschungsfragen:

- Wie stellen sich die Effekte hinsichtlich unterschiedlicher Angebotsformate (z.B. Einzel- vs. Gruppentherapie, analog vs. digital) der KVT bei Parkinson-Pat. dar?
- Welche Effekte weiterer psychotherapeutischer Ansätze (z.B. achtsamkeitsbasierte Ansätze, psychodynamische Therapie, systemische Therapie) sind in der Behandlung von Depression bei Parkinson-Pat. zu erwarten?
- Welche physischen Interventionstypen zeigen die größten Effekte in der Behandlung von Depression bei Parkinson-Pat.?

Fragestellung 136: Apathie: Wie effektiv sind psycho- und ergotherapeutische Ansätze sowie physische Interventionen im Vergleich mit aktiven und passiven Kontrollgruppen in der Behandlung von Apathie bei Parkinson-Pat.?

Evidenzgrundlage

- Psychotherapie: N=0 Metaanalysen
- Ergotherapie: N=0 Metaanalysen
- *Physische Interventionen: N=2 Metanalysen; N=3 randomisierte kontrollierte Studie*

Folgende Literatursuche wurde in PubMed durchgeführt: „Parkinson's disease" AND „apathy" AND „systematic review". Die Literatursuche in PubMed lieferte insgesamt 32 Publikationen, innerhalb derer N=2 Metaanalysen im Bereich der physischen Interventionen identifiziert werden konnten. Zusätzlich wurden spezifische Literatursuchen hinsichtlich randomisierter kontrollierter Studien mit folgenden Suchstrings durchgeführt: „Parkinson's disease" „apathy" „psychotherapy" (16 Treffer); „Parkinson's disease" „apathy" „occupational therapy" (N=10 Treffer); „Parkinson's disease" AND „apathy" AND „physical" (N=54 Treffer), wobei lediglich für die physischen Interventionen N=3 randomisierte kontrollierte Studien identifiziert werden konnten.

Ergebnis

Psychotherapie

Unter den psychotherapeutischen Interventionen lassen sich verschiedene Schulen subsumieren. Eine gängige Einteilung ist die Unterscheidung der (kognitiven) Verhaltenstherapie (KVT), der psychodynamischen Therapien sowie der systemischen Therapie. Die initiale Literaturrecherche ermittelte keine Metaanalysen im Bereich Psychotherapie. Eine zusätzliche Suche konnte keine randomisierten kontrollierten Studien zu psychotherapeutischen Interventionen zur Behandlung der Apathie bei Parkinson-Pat. ermitteln.

Ergotherapie

Bis dato liegen keine Metaanalysen sowie randomisierten kontrollierten Studien vor, die den Effekt von Ergotherapie zur Behandlung von apathischen Symptomen bei Parkinson-Pat. untersucht haben.

Physische Interventionen

Insgesamt wird Apathie nur sehr selten als Endpunkt im Rahmen von physischen Interventionsstudien definiert. Eine Metaanalyse aus dem Jahr 2022 [105] mit lediglich N=2 randomisierten kontrollierten Studien (insgesamt N=62 Parkinson-Pat.), die Apathie als Endpunkt definiert hatten, ergab keinen signifikanten Effekt von Tanztherapie im Vergleich zu aktiven Kontrollgruppen auf Apathie-Werte. Eine Metaanalyse von 2019, die dieselben Studien berücksichtigte, kam zu demselben Ergebnis [106]. Allerdings zeigte eine randomisierte kontrollierte Studie mit N=20 Parkinson-Pat. aus dem Jahr 2015 einen positiven Effekt von Nordic Walking im

Vergleich zu einer passiven Kontrollgruppe auf die Apathie-Symptomatik [110]. Eine randomisierte kontrollierte Studie, die die Wirksamkeit eines Aerobic-Trainings im Vergleich zu einem Stretching-Programm bei N=35 Parkinson-Pat. untersucht hat, konnte keine Überlegenheit eines der Trainingsprogramme ermitteln [111]. Der Vergleich einer physischen Intervention im Gruppenformat und eines Einzeltrainings ohne Supervision bei N=30 Parkinson-Pat. konnte keine Überlegenheit einer Trainingsart in Bezug auf die Apathie-Symptomatik zeigen [112].

Begründung der Empfehlung

Apathie ist bis dato kaum als Endpunkt in nicht pharmakologischen Interventionsstudien definiert worden. Aufgrund der fehlenden Evidenz kann keine Empfehlung für die psycho- und ergotherapeutische Behandlung von Apathie bei Parkinson-Pat. ausgesprochen werden. Eine randomisierte kontrollierte Studie liefert Hinweise auf einen möglichen Effekt von physischen Interventionen zur Behandlung von apathischen Symptomen bei Parkinson-Pat.

**Empfehlung: Physische Interventionen bei Apathie**	**Neu**<br><br>**Stand (2023)**
Physische Interventionen können für die Behandlung einer Apathie-Symptomatik bei Parkinson-Pat. angeboten werden.	
Konsensstärke: 100%, starker Konsens	

Wichtige Forschungsfragen

Zur nicht pharmakologischen Behandlung von Apathie bei Parkinson-Pat. ergeben sich folgende wichtige Forschungsfragen:

- Haben psycho- und ergotherapeutische Ansätze einen positiven Einfluss auf die Apathie-Symptomatik bei Parkinson-Pat.?
- Welche weiteren Arten der physischen Interventionen haben einen positiven Effekt auf die Apathie-Symptomatik bei Parkinson-Pat.?
- Welche Faktoren (z.B. Alter, Geschlecht, Krankheitsdauer, -schwere) beeinflussen den Effekt physischer Interventionen auf die Apathie-Symptomatik bei Parkinson-Pat.?

Fragestellung 137: Angststörungen, Progredienzangst: Wie effektiv sind psycho- und ergotherapeutische Ansätze sowie physische Interventionen im Vergleich mit aktiven und passiven Kontrollgruppen in der Behandlung von Angststörungen bei Parkinson-Pat.?

Evidenzgrundlage

- Psychotherapie: N=2 Metaanalysen
- Ergotherapie: N=0 Metaanalysen
- Physische Interventionen: N=2 Metaanalysen

Eine Literatursuche in PubMed mit den Suchbegriffen „Parkinson's disease“ AND „anxiety“ AND „systematic review“ ergab 88 Treffer, wobei schließlich vier systematische Reviews mit Metaanalysen [97], [96], [103], [113] aus dem Zeitraum 2013 bis 2023 als Evidenzgrundlage berücksichtigt werden konnten. Zusätzlich fand die 2021 überarbeitete S3-Leitlinie [114] für die Behandlung von Angststörungen Eingang in die Beurteilung der Evidenzgrundlage und die Erarbeitung von Therapieempfehlungen.

Ergebnis

Psychotherapie

Unter den psychotherapeutischen Interventionen lassen sich verschiedene therapeutische Schulen subsumieren. Eine gängige Einteilung ist die Unterscheidung der (kognitiven) Verhaltenstherapie (KVT), der psychodynamischen Therapien sowie der systemischen Therapie. Die Literatursuche identifizierte zwei Metaanalysen [97], [96] aus den Jahren 2020 und 2021, die lediglich den Ansatz der KVT berücksichtigten.

Zhang et al. [96] inkludierten insgesamt fünf RCTs und eine nicht randomisierte kontrollierte Studie mit insgesamt N=163 Parkinson-Pat. in ihre Metaanalyse. Es zeigte sich ein signifikanter moderater Effekt von KVT, die in unterschiedlichen Formaten angeboten wurde (Einzel- vs. Gruppentherapie; analoge vs. telefonbasierte Therapie) im Vergleich zu aktiven und passiven Kontrollgruppen.

In der 2021 veröffentlichen Metaanalyse von Luo et al. [97] wurden schließlich 10 Studien mit insgesamt N=383 Parkinson-Pat. in den Vergleich von KVT und aktiven bzw. passiven Kontrollgruppen eingeschlossen. Hier zeigte sich ein signifikanter großer Effekt zugunsten der KVT. Subgruppenanalysen konnten ferner demonstrieren, dass Interventionen über einen Zeitraum von mindestens 10 Wochen die größten Effekte auf die Reduktion von Ängsten zeigten. Es gab keine Unterschiede zwischen den Effekten bei analogen und digitalen Angebotsformaten. Allerdings zeigte sich die Einzeltherapie der Gruppentherapie überlegen.

Eine ergänzende Suche in Pubmed mit den Suchbegriffen „Psychodynamic“ bzw. „Psychoanalysis“ bzw. „Systemic therapy“ AND „Parkinson's disease“ AND „anxiety“ ergab keine Suchtreffer. Über Effekte psychodynamischer und systemischer Therapieansätze spezifisch für Parkinson-Pat. lassen sich daher keine Aussagen treffen.

Die Autorinnen/Autoren der S3-Leitlinie zur Behandlung von Angststörungen [114] sprechen den höchsten Empfehlungsgrad für die KVT zur Behandlung von generalisierter Angststörung, Panikstörung/Agoraphobie, spezifischen Phobien und sozialer Angststörung aus, wohingegen psychodynamische Therapieansätze erst angeboten werden sollten, wenn die KVT keine Erfolge erzielt hat; die systemische Therapie bietet sich bei der Behandlung von sozialen Angststörungen ebenfalls an, wenn die KVT sowie die psychodynamische Therapie ausgeschöpft sind.

Zur Behandlung von Progredienzangst bei Parkinson-Pat. konnten keine Metaanalysen und RCTs identifiziert werden.

Ergotherapie

Die Literatursuche erzielte keine Treffer für eine Metaanalyse zu den Effekten von Ergotherapie auf Angststörungen bei Parkinson-Pat. Auch findet Ergotherapie keine Berücksichtigung in der S3-Leitlinie zur Behandlung von Angststörungen [114]. Daraufhin erfolgte eine Recherche in Pubmed auf RCT-Ebene mit den Suchbegriffen „Occupational therapy“ AND „Parkinson’s disease“ AND „anxiety“, durch die ebenfalls keine Evidenz detektiert werden konnte.

Physische Interventionen

Physische Interventionen umfassen eine große Bandbreite an unterschiedlichen Bewegungsansätzen (z.B. Physiotherapie, Ausdauer- und Krafttraining, Tai-Chi, Yoga und Tanzen). Eine groß angelegte Netzwerk-Metaanalyse aus dem Jahr 2022 [103] berücksichtigte insgesamt 250 Studien mit N=13.011 Parkinson-Pat. zu physischen Interventionen, wobei lediglich 13 Studien mit insgesamt N=757 Menschen mit Parkinson eine Angstsymptomatik als Endpunkt berücksichtigt hatten. Die Netzwerk-Metaanalyse ergab lediglich für Yoga eine signifikante Überlegenheit gegenüber aktiven und passiven Kontrollgruppen. Allerdings wurde in diesem Vergleich nur eine Yoga-Studie berücksichtigt.

Eine 2023 publizierte Metaanalyse [113] fokussierte sich spezifisch auf die Effekte physischer Interventionen und ihre Effekte auf eine Angstsymptomatik bei Parkinson-Pat. Die Autorinnen und Autoren inkludierten fünf RCTs mit insgesamt N=328 Parkinson-Pat. und schlussfolgerten, dass die Evidenzlage unzureichend sei und sich keine Empfehlungen ableiten ließen.

In der S3-Leitlinie zur Behandlung von Angststörungen [114] wird Sport lediglich für Panikstörungen/Agoraphobien empfohlen. Die Basis hierfür ist ein Expertenkonsens, der in der Empfehlung resultierte, dass Sport als ergänzende Therapie angeboten werden kann.

Begründung der Empfehlung

Die zwei berücksichtigten Metaanalysen, die psychotherapeutische Interventionen zur Behandlung einer Angstsymptomatik bei Parkinson-Pat. untersuchten, bieten eine überzeugende Evidenz für die Effekte von KVT, die durch die Empfehlungen der S3-Leitlinie zur Behandlung von Angststörungen [114] bestätigt wird. Da es Überlappungen zwischen Angststörungen und Progredienzängsten gibt, wurde zudem der Einsatz der KVT bei ausgeprägten Progredienzängsten unter den Autoren dieses Kapitels diskutiert. Hierbei wurden auch RCTs bei anderen Zielgruppen (z.B. Pat. mit Krebserkrankungen) berücksichtigt, die positive Effekte einer KVT auf Progredienzängste zeigten (z.B. [115]). Auf dieser Grundlage wurde die Empfehlung formuliert.

Für die Ergotherapie besteht aktuell keine Evidenz, sodass keine Empfehlungen ausgesprochen werden können.

Für physische Interventionen zur Behandlung von Angststörungen bei Parkinson-Pat. besteht insgesamt großer Forschungsbedarf, da eine Angstsymptomatik bisher kaum als Endpunkt definiert worden ist. Vor diesem Hintergrund wurde keine Empfehlung ausgesprochen.

Empfehlung: Psychotherapie bei Angststörungen	Neu<br><br>Stand (2023)
Kognitive Verhaltenstherapie soll Parkinson-Pat. zur Behandlung von Angststörungen angeboten werden.	
Konsensstärke: 100%, starker Konsens	

Empfehlung: Psychotherapie bei Progredienzangst	Neu<br><br>Stand (2023)
Kognitive Verhaltenstherapie kann Parkinson-Pat. mit ausgeprägten Progredienzängsten angeboten werden.	
Konsensstärke: 100%, starker Konsens	

Wichtige Forschungsfragen

Zur Behandlung von Angststörungen sowie Progredienzangst bei Parkinson-Pat. ergeben sich folgende wichtige Forschungsfragen:

- Welche Parkinson-Pat. mit welchen Charakteristika (z.B. soziodemographische, krankheitsbezogene oder (neuro-)psychologische Faktoren) profitieren am meisten von KVT zur Behandlung von Angststörungen?
- Welche Effekte weiterer psychotherapeutischer Ansätze (z.B. achtsamkeitsbasierte Ansätze, psychodynamische Therapie, systemische Therapie) sind in der Behandlung von Angststörungen bei Parkinson-Pat. zu erwarten?
- Welchen Effekt hat die KVT auf den Umgang mit (dysfunktionalen) Progredienzängsten bei Parkinson-Pat.?

Fragestellung 138: Fatigue: Wie effektiv sind psycho- und ergotherapeutische Ansätze und physische Interventionen im Vergleich mit aktiven und passiven Kontrollgruppen in der Behandlung von Fatigue bei Parkinson-Pat.?

Evidenzgrundlage

- Psychotherapie: N=2 Metaanalysen; N=1 aktuelle randomisierte, kontrollierte Studie, die bisher nicht in den Metaanalysen abgedeckt worden ist
- Ergotherapie: N=0 Metaanalysen; N=1 randomisierte, kontrollierte Studie
- Physische Interventionen: N=5 Metaanalysen

Eine Literatursuche in PubMed mit den Suchbegriffen „Parkinson's disease" AND „fatigue" AND „systematic review" ergab 40 Treffer, wobei schließlich fünf systematische Reviews mit Metaanalysen [97], [116], [117] [107], [105] als Evidenzgrundlage eingeschlossen werden konnten. Zusätzlich fand eine rezente Metaanalyse [118] Eingang in die Erarbeitung der Therapieempfehlungen.

Ergebnis

Psychotherapie

Unter den psychotherapeutischen Interventionen lassen sich verschiedene Schulen subsumieren. Eine gängige Einteilung ist die Unterscheidung der (kognitiven) Verhaltenstherapie (KVT), der psychodynamischen Therapien sowie der systemischen Therapie. Luo et al. [97] inkludierten in ihrer 2021 erschienen Metaanalyse zwei Studien zu KVT im Vergleich zu aktiven Kontrollgruppen mit N=32 Parkinson-Pat. und konnten keinen signifikanten Effekt auf die Fatigue-Symptomatik ermitteln. Jiang et al. [116] inkludierten lediglich eine RCT mit N=12 Parkinson-Pat. in ihre Metaanalyse, die eine Kombination aus KVT mit Lichttherapie im Vergleich zu einer Placebogruppe untersuchte, wobei sich keine positiven Effekte auf die Fatigue-Symptomatik ergaben. Eine rezente RCT von Bogosian et al. [119] aus dem Jahr 2022 konnte ebenfalls keine signifikanten Effekte einer achtsamkeitsbasierten Intervention im Vergleich zu einer passiven Kontrollgruppe auf die Fatigue-Symptomatik von Parkinson-Pat. demonstrieren.

Ergotherapie

Aufgrund fehlender Studien zu Ergotherapie, die den Endpunkt Fatigue berücksichtigt haben, konnte in keinem der systematischen Reviews eine Metaanalyse gerechnet werden. Sturkenboom et al. [101] konnten zudem in ihrer RCT mit N=191 Parkinson-Pat. keinen Effekt von häuslicher Ergotherapie im Vergleich zu einer passiven Kontrollgruppe auf die Fatigue-Symptomatik bei Parkinson-Pat. finden.

Physische Interventionen

In das Cochrane Review von Elbers et al. [117] konnten 2015 lediglich zwei RCTs mit N=57 Parkinson-Pat. zu physischen Interventionen im Vergleich zu passiven Kontrollgruppen inkludiert werden, wobei kein positiver Effekt auf die Fatigue-Symptomatik ermittelt werden konnte. Zwei Metaanalysen zu spezifischen physischen Interventionsansätzen (d.h. Körper-Geist-Interventionen und Tanztherapie) konnten jeweils nur N=3 ([107]; N=74 Parkinson-Pat.) bzw. N=2 ([105]; N=58 Parkinson-Pat.) RCTs einschließen, die keine Überlegenheit der physischen Interventionen gegenüber den aktiven und passiven Kontrollgruppen zeigen konnten. Eine Metaanalyse [116] aus 2023 mit N=4 RCTs und insgesamt N=100 Parkinson-Pat. zu verschiedenen physischen Interventionsansätzen zeigte einen Trend für eine Überlegenheit gegenüber aktiven und passiven Kontrollgruppen. Eine rezente Metaanalyse (Folkerts, im Druck) konnte auf 30 RCTs zu unterschiedlichen medikamentösen und nicht medikamentösen Therapieansätzen zurückgreifen. Acht Studien mit N=324 Parkinson-Pat. konnten für eine Metaanalyse zu physischen Interventionen im Vergleich mit aktiven und passiven Kontrollgruppen berücksichtigt werden. Es zeigte sich ein signifikanter kleiner Effekt im Vergleich zu aktiven und passiven Kontrollgruppen auf die Fatigue-Symptomatik bei Parkinson-Pat.

Begründung der Empfehlung

Bis dato gibt es kaum Studien zu psycho- und ergotherapeutischen Ansätzen, in denen Fatigue als Endpunkt definiert worden ist. Die bisherige Evidenz gibt zudem keine Hinweise, dass diese Therapien effektiv zur Behandlung von Fatigue bei Parkinson-Pat. eingesetzt werden sollten.

Die Evidenz zu physischen Interventionen zur Behandlung der Fatigue-Symptomatik ist in den letzten Jahren stetig gewachsen. Die rezente und damit größte Metaanalyse von Folkerts et al. (2023) stellt damit die Grundlage für die Empfehlung von physischen Interventionen bei bestehender Fatigue dar.

**Empfehlung: Physische Interventionen bei Fatigue**	**Neu**<br>**Stand (2023)**
Physische Interventionen sollen für die Behandlung einer Fatigue-Symptomatik bei Parkinson-Pat. angeboten werden.	
Konsensstärke: 100%, starker Konsens	

Wichtige Forschungsfragen

Zur nicht pharmakologischen Behandlung von Fatigue bei Parkinson-Pat. ergeben sich folgende wichtige Forschungsfragen:

- Welche Arten der physischen Interventionen zeigen die größten Effekte auf die Fatigue-Symptomatik bei Parkinson-Pat.?
- Welche Interventionen wirken auf welche Unterformen der Fatigue (d.h. physische vs. mentale Fatigue) bei Parkinson-Pat.?
- Welche Faktoren (z.B. Alter, Geschlecht, Krankheitsdauer, -schwere) beeinflussen den Effekt physischer Interventionen auf die Fatigue-Symptomatik bei Parkinson-Pat.?
- Welche Komponenten sollte ein Programm zum Management von Fatigue bei Parkinson-Pat. enthalten?

Referenzen

1. Macías-García P, Rashid-López R, Cruz-Gómez Á J, Lozano-Soto E, Sanmartino F, Espinosa-Rosso R, González-Rosa JJ. Neuropsychiatric Symptoms in Clinically Defined Parkinson's Disease: An Updated Review of Literature. Behav Neurol. 2022;2022:1213393
2. *https://www.bfarm.de/DE/Kodiersysteme/Klassifikationen/ICD/ICD-11/uebersetzung/_node.html;jsessionid=272C7E204316C0BDAB6ABCAC99C2E713.internet271*; Abruf am 13.06.2023.
3. Reiff J, Schmidt N, Riebe B, Breternitz R, Aldenhoff J, Deuschl G, Witt K. Subthreshold depression in Parkinson's disease. Mov Disord. 2011;26(9):1741-4
4. Qin Z, Zhang L, Sun F, Liu H, Fang X, Chan P. Depressive symptoms impacting on health-related quality of life in early Parkinson's disease: results from Chinese L-dopa exposed cohort. Clin Neurol Neurosurg. 2009;111(9):733-7

5. Cong S, Xiang C, Zhang S, Zhang T, Wang H, Cong S. Prevalence and clinical aspects of depression in Parkinson's disease: A systematic review and meta-analysis of 129 studies. Neurosci Biobehav Rev. 2022;141:104749
6. Leentjens AF, Dujardin K, Marsh L, Martinez-Martin P, Richard IH, Starkstein SE, et al. Apathy and anhedonia rating scales in Parkinson's disease: critique and recommendations. Mov Disord. 2008;23(14):2004-14
7. Nagayama H, Maeda T, Uchiyama T, Hashimoto M, Nomoto N, Kano O, et al. Anhedonia and its correlation with clinical aspects in Parkinson's disease. J Neurol Sci. 2017;372:403-7
8. van der Velden RMJ, Broen MPG, Kuijf ML, Leentjens AFG. Frequency of mood and anxiety fluctuations in Parkinson's disease patients with motor fluctuations: A systematic review. Mov Disord. 2018;33(10):1521-7
9. Ardouin C, Chéreau I, Llorca PM, Lhommée E, Durif F, Pollak P, Krack P. [Assessment of hyper- and hypodopaminergic behaviors in Parkinson's disease]. Rev Neurol (Paris). 2009;165(11):845-56
10. den Brok MG, van Dalen JW, van Gool WA, Moll van Charante EP, de Bie RM, Richard E. Apathy in Parkinson's disease: A systematic review and meta-analysis. Mov Disord. 2015;30(6):759-69
11. Broen MP, Narayen NE, Kuijf ML, Dissanayaka NN, Leentjens AF. Prevalence of anxiety in Parkinson's disease: A systematic review and meta-analysis. Mov Disord. 2016;31(8):1125-33
12. Berg P, Book K, Dinkel A, Henrich G, Marten-Mittag B, Mertens D, et al. [Fear of progression in chronic diseases]. Psychother Psychosom Med Psychol. 2011;61(1):32-7
13. Folkerts AK, Haarmann L, Nielsen J, Saliger J, Eschweiler M, Karbe H, et al. Fear of Progression is Determined by Anxiety and Self-Efficacy but not Disease-Specific Parameters in Patients with Parkinson's Disease: Preliminary Data from a Multicenter Cross-Sectional Study. J Parkinsons Dis. 2022;12(8):2543-53
14. Kluger BM, Herlofson K, Chou KL, Lou JS, Goetz CG, Lang AE, et al. Parkinson's disease-related fatigue: A case definition and recommendations for clinical research. Mov Disord. 2016;31(5):625-31
15. Lou JS. Physical and mental fatigue in Parkinson's disease: epidemiology, pathophysiology and treatment. Drugs Aging. 2009;26(3):195-208
16. Metta V, Logishetty K, Martinez-Martin P, Gage HM, Schartau PE, Kaluarachchi TK, et al. The possible clinical predictors of fatigue in Parkinson's disease: a study of 135 patients as part of international nonmotor scale validation project. Parkinsons Dis. 2011;2011:125271
17. Friedman JH, Friedman H. Fatigue in Parkinson's disease: a nine-year follow-up. Mov Disord. 2001;16(6):1120-2
18. Herlofson K, Kluger BM. Fatigue in Parkinson's disease. J Neurol Sci. 2017;374:38-41
19. Schrag A. Quality of life and depression in Parkinson's disease. J Neurol Sci. 2006;248(1-2):151-7
20. Burn DJ. Beyond the iron mask: towards better recognition and treatment of depression associated with Parkinson's disease. Mov Disord. 2002;17(3):445-54
21. Shulman LM, Taback RL, Rabinstein AA, Weiner WJ. Non-recognition of depression and other non-motor symptoms in Parkinson's disease. Parkinsonism Relat Disord. 2002;8(3):193-7
22. Richard IH, Kurlan R. A survey of antidepressant drug use in Parkinson's disease. Parkinson Study Group. Neurology. 1997;49(4):1168-70
23. Weintraub D, Moberg PJ, Duda JE, Katz IR, Stern MB. Recognition and treatment of depression in Parkinson's disease. J Geriatr Psychiatry Neurol. 2003;16(3):178-83
24. Schwarz J, Odin P, Buhmann C, Csoti I, Jost W, Wüllner U, Storch A. Depression in Parkinson's disease. J Neurol. 2011;258(Suppl 2):S336-8
25. Brown RG, MacCarthy B, Gotham AM, Der GJ, Marsden CD. Depression and disability in Parkinson's disease: a follow-up of 132 cases. Psychol Med. 1988;18(1):49-55

26. Storch A, Ebersbach G, Fuchs G, Jost WH, Odin P, Reifschneider G, Bauer M. [Depression in Parkinson's disease. Part 1: epidemiology, signs and symptoms, pathophysiology and diagnosis]. Fortschr Neurol Psychiatr. 2008;76(12):715-24
27. Kummer A, Cardoso F, Teixeira AL. Suicidal ideation in Parkinson's disease. CNS Spectr. 2009;14(8):431-6
28. Lemke MR. Depressive symptoms in Parkinson's disease. Eur J Neurol. 2008;15 Suppl 1:21-5
29. Pagonabarraga J, Kulisevsky J. Apathy in Parkinson's Disease. Int Rev Neurobiol. 2017;133:657-78
30. Kluger BM. Fatigue in Parkinson's Disease. Int Rev Neurobiol. 2017;133:743-68
31. Schrag A, Barone P, Brown RG, Leentjens AF, McDonald WM, Starkstein S, et al. Depression rating scales in Parkinson's disease: critique and recommendations. Mov Disord. 2007;22(8):1077-92
32. Nissenbaum H, Quinn NP, Brown RG, Toone B, Gotham AM, Marsden CD. Mood swings associated with the 'on-off' phenomenon in Parkinson's disease. Psychol Med. 1987;17(4):899-904
33. Raudino F. Non motor off in Parkinson's disease. Acta Neurol Scand. 2001;104(5):312-5
34. Kasten M, Kertelge L, Tadic V, Brüggemann N, Schmidt A, van der Vegt J, et al. Depression and quality of life in monogenic compared to idiopathic, early-onset Parkinson's disease. Mov Disord. 2012;27(6):754-9
35. *https://www.movementdisorders.org/MDS/Education/Rating-Scales/MDS-Recommended-Rating-Scales.htm*; Abruf am 21.06.2023.
36. Weintraub D, Aarsland D, Chaudhuri KR, Dobkin RD, Leentjens AF, Rodriguez-Violante M, Schrag A. The neuropsychiatry of Parkinson's disease: advances and challenges. Lancet Neurol. 2022;21(1):89-102
37. Martinez-Martin P, Leentjens AF, de Pedro-Cuesta J, Chaudhuri KR, Schrag AE, Weintraub D. Accuracy of screening instruments for detection of neuropsychiatric syndromes in Parkinson's disease. Mov Disord. 2016;31(3):270-9
38. Mueller C, Rajkumar AP, Wan YM, Velayudhan L, Ffytche D, Chaudhuri KR, Aarsland D. Assessment and Management of Neuropsychiatric Symptoms in Parkinson's Disease. CNS Drugs. 2018;32(7):621-35
39. DGN. S3-Leitlinie Parkinson-Syndrom, idiopathisch. Online: *https://register.awmf.org/assets/guidelines/030-010k_S3_Parkinson_Syndrome_Idiopathisch_2016-06-abgelaufen.pdf*; (abgerufen am 21.06.2023).
40. Elbers RG, Rietberg MB, van Wegen EE, Verhoef J, Kramer SF, Terwee CB, Kwakkel G. Self-report fatigue questionnaires in multiple sclerosis, Parkinson's disease and stroke: a systematic review of measurement properties. Qual Life Res. 2012;21(6):925-44
41. Falup-Pecurariu C. Fatigue assessment of Parkinson's disease patient in clinic: specific versus holistic. J Neural Transm (Vienna). 2013;120(4):577-81
42. Bruno AE, Sethares KA. Fatigue in Parkinson disease: an integrative review. J Neurosci Nurs. 2015;47(3):146-53
43. Williams JR, Hirsch ES, Anderson K, Bush AL, Goldstein SR, Grill S, et al. A comparison of nine scales to detect depression in Parkinson disease: which scale to use? Neurology. 2012;78(13):998-1006
44. *https://www.movementdisorders.org/MDS/Education/Rating-Scales/MDS-Recommended-Rating-Scales.htm*; Abruf am 12.06.2023.
45. Leentjens AF, Dujardin K, Marsh L, Martinez-Martin P, Richard IH, Starkstein SE, et al. Anxiety rating scales in Parkinson's disease: critique and recommendations. Mov Disord. 2008;23(14):2015-25

46. Mehnert A, Herschbach P, Berg P, Henrich G, Koch U. [Fear of progression in breast cancer patients--validation of the short form of the Fear of Progression Questionnaire (FoP-Q-SF)]. Z Psychosom Med Psychother. 2006;52(3):274-88
47. Pagonabarraga J, Kulisevsky J, Strafella AP, Krack P. Apathy in Parkinson's disease: clinical features, neural substrates, diagnosis, and treatment. Lancet Neurol. 2015;14(5):518-31
48. Witt K, Daniels C, Herzog J, Lorenz D, Volkmann J, Reiff J, et al. Differential effects of L-dopa and subthalamic stimulation on depressive symptoms and hedonic tone in Parkinson's disease. J Neuropsychiatry Clin Neurosci. 2006;18(3):397-401
49. Fung VS, Herawati L, Wan Y. Quality of life in early Parkinson's disease treated with levodopa/carbidopa/entacapone. Mov Disord. 2009;24(1):25-31
50. Barone P, Poewe W, Albrecht S, Debieuvre C, Massey D, Rascol O, et al. Pramipexole for the treatment of depressive symptoms in patients with Parkinson's disease: a randomised, double-blind, placebo-controlled trial. Lancet Neurol. 2010;9(6):573-80
51. Ji N, Meng P, Xu B, Zhou X. Efficacy and safety of pramipexole in Parkinson's disease with anxiety or depression: a meta-analysis of randomized clinical trials. Am J Transl Res. 2022;14(3):1757-64
52. Jiang DQ, Jiang LL, Wang Y, Li MX. The role of pramipexole in the treatment of patients with depression and Parkinson's disease: A meta-analysis of randomized controlled trials. Asian J Psychiatr. 2021;61:102691
53. Leentjens AF, Koester J, Fruh B, Shephard DT, Barone P, Houben JJ. The effect of pramipexole on mood and motivational symptoms in Parkinson's disease: a meta-analysis of placebo-controlled studies. Clin Ther. 2009;31(1):89-98
54. Rektorová I, Rektor I, Bares M, Dostál V, Ehler E, Fanfrdlová Z, et al. Pramipexole and pergolide in the treatment of depression in Parkinson's disease: a national multicentre prospective randomized study. Eur J Neurol. 2003;10(4):399-406
55. Ziaei E, Emami Ardestani P, Chitsaz A. Comparison of pramipexole and citalopram in the treatment of depression in Parkinson's disease: A randomized parallel-group trial. J Res Med Sci. 2022;27:55
56. Barone P, Scarzella L, Marconi R, Antonini A, Morgante L, Bracco F, et al. Pramipexole versus sertraline in the treatment of depression in Parkinson's disease: a national multicenter parallel-group randomized study. J Neurol. 2006;253(5):601-7
57. Pahwa R, Stacy MA, Factor SA, Lyons KE, Stocchi F, Hersh BP, et al. Ropinirole 24-hour prolonged release: randomized, controlled study in advanced Parkinson disease. Neurology. 2007;68(14):1108-15
58. Chung SJ, Asgharnejad M, Bauer L, Ramirez F, Jeon B. Evaluation of rotigotine transdermal patch for the treatment of depressive symptoms in patients with Parkinson's disease. Expert Opin Pharmacother. 2016;17(11):1453-61
59. Castrioto A, Thobois S, Anheim M, Quesada JL, Lhommée E, Klinger H, et al. A randomized controlled double-blind study of rotigotine on neuropsychiatric symptoms in de novo PD. NPJ Parkinsons Dis. 2020;6(1):41
60. Ray Chaudhuri K, Martinez-Martin P, Antonini A, Brown RG, Friedman JH, Onofrj M, et al. Rotigotine and specific non-motor symptoms of Parkinson's disease: post hoc analysis of RECOVER. Parkinsonism Relat Disord. 2013;19(7):660-5
61. Yan J, Ma H, Liu A, Huang J, Wu J, Yang J. Efficacy and Safety of Rotigotine Transdermal Patch on Neuropsychiatric Symptoms of Parkinson's Disease: An Updated Meta-Analysis and Systematic Review. Front Neurol. 2021;12:722892
62. Wang HT, Wang L, He Y, Yu G. Rotigotine transdermal patch for the treatment of neuropsychiatric symptoms in Parkinson's disease: A meta-analysis of randomized placebo-controlled trials. J Neurol Sci. 2018;393:31-8

63. Lemke MR, Brecht HM, Koester J, Reichmann H. Effects of the dopamine agonist pramipexole on depression, anhedonia and motor functioning in Parkinson's disease. J Neurol Sci. 2006;248(1-2):266-70
64. Lemke MR, Brecht HM, Koester J, Kraus PH, Reichmann H. Anhedonia, depression, and motor functioning in Parkinson's disease during treatment with pramipexole. J Neuropsychiatry Clin Neurosci. 2005;17(2):214-20
65. Thobois S, Lhommée E, Klinger H, Ardouin C, Schmitt E, Bichon A, et al. Parkinsonian apathy responds to dopaminergic stimulation of D2/D3 receptors with piribedil. Brain. 2013;136(Pt 5):1568-77
66. Fabbri M, Barbosa R, Rascol O. Off-time Treatment Options for Parkinson's Disease. Neurol Ther. 2023;12(2):391-424
67. Czernecki V, Pillon B, Houeto JL, Pochon JB, Levy R, Dubois B. Motivation, reward, and Parkinson's disease: influence of dopatherapy. Neuropsychologia. 2002;40(13):2257-67
68. Pérez-Pérez J, Pagonabarraga J, Martínez-Horta S, Fernández-Bobadilla R, Sierra S, Pascual-Sedano B, et al. Head-to-Head Comparison of the Neuropsychiatric Effect of Dopamine Agonists in Parkinson's Disease: A Prospective, Cross-Sectional Study in Non-demented Patients. Drugs Aging. 2015;32(5):401-7
69. Sawada H, Umemura A, Kohsaka M, Tomita S, Park K, Oeda T, Yamamoto K. Pharmacological interventions for anxiety in Parkinson's disease sufferers. Expert Opin Pharmacother. 2018;19(10):1071-6
70. Prediger RD, Matheus FC, Schwarzbold ML, Lima MM, Vital MA. Anxiety in Parkinson's disease: a critical review of experimental and clinical studies. Neuropharmacology. 2012;62(1):115-24
71. Troeung L, Egan SJ, Gasson N. A meta-analysis of randomised placebo-controlled treatment trials for depression and anxiety in Parkinson's disease. PLoS One. 2013;8(11):e79510
72. Schifitto G, Friedman JH, Oakes D, Shulman L, Comella CL, Marek K, Fahn S. Fatigue in levodopa-naive subjects with Parkinson disease. Neurology. 2008;71(7):481-5
73. Lou JS, Kearns G, Benice T, Oken B, Sexton G, Nutt J. Levodopa improves physical fatigue in Parkinson's disease: a double-blind, placebo-controlled, crossover study. Mov Disord. 2003;18(10):1108-14
74. Hauser RA, Slawek J, Barone P, Dohin E, Surmann E, Asgharnejad M, Bauer L. Evaluation of rotigotine transdermal patch for the treatment of apathy and motor symptoms in Parkinson's disease. BMC Neurol. 2016;16:90
75. Shannon KM, Bennett JP, Jr., Friedman JH. Efficacy of pramipexole, a novel dopamine agonist, as monotherapy in mild to moderate Parkinson's disease. The Pramipexole Study Group. Neurology. 1997;49(3):724-8
76. Hauser RA, Schapira AH, Rascol O, Barone P, Mizuno Y, Salin L, et al. Randomized, double-blind, multicenter evaluation of pramipexole extended release once daily in early Parkinson's disease. Mov Disord. 2010;25(15):2542-9
77. Maillet A, Météreau E, Tremblay L, Favre E, Klinger H, Lhommée E, et al. Serotonergic and Dopaminergic Lesions Underlying Parkinsonian Neuropsychiatric Signs. Mov Disord. 2021;36(12):2888-900
78. Maillet A, Krack P, Lhommée E, Météreau E, Klinger H, Favre E, et al. The prominent role of serotonergic degeneration in apathy, anxiety and depression in de novo Parkinson's disease. Brain. 2016;139(Pt 9):2486-502
79. Pagonabarraga J, Tinazzi M, Caccia C, Jost WH. The role of glutamatergic neurotransmission in the motor and non-motor symptoms in Parkinson's disease: Clinical cases and a review of the literature. J Clin Neurosci. 2021;90:178-83
80. Nationale Versorgungs-Leitlinie für Unipolare Depression. NVL-Programm 2022. Online: *https://www.leitlinien.de/themen/depression/version-3* (abgerufen am 13.06.2023).

81. Menza M, Dobkin RD, Marin H, Mark MH, Gara M, Buyske S, et al. A controlled trial of antidepressants in patients with Parkinson disease and depression. Neurology. 2009;72(10):886-92
82. Menza M, Dobkin RD, Marin H, Mark MH, Gara M, Buyske S, et al. The impact of treatment of depression on quality of life, disability and relapse in patients with Parkinson's disease. Mov Disord. 2009;24(9):1325-32
83. Skapinakis P, Bakola E, Salanti G, Lewis G, Kyritsis AP, Mavreas V. Efficacy and acceptability of selective serotonin reuptake inhibitors for the treatment of depression in Parkinson's disease: a systematic review and meta-analysis of randomized controlled trials. BMC Neurol. 2010;10:49
84. Seppi K, Weintraub D, Coelho M, Perez-Lloret S, Fox SH, Katzenschlager R, et al. The Movement Disorder Society Evidence-Based Medicine Review Update: Treatments for the non-motor symptoms of Parkinson's disease. Mov Disord. 2011;26 Suppl 3(0 3):S42-80
85. Cattaneo C, Müller T, Bonizzoni E, Lazzeri G, Kottakis I, Keywood C. Long-Term Effects of Safinamide on Mood Fluctuations in Parkinson's Disease. J Parkinsons Dis. 2017;7(4):629-34
86. Santos García D, Labandeira Guerra C, Yáñez Baña R, Cimas Hernando MI, Cabo López I, Paz Gonález JM, et al. Safinamide Improves Non-Motor Symptoms Burden in Parkinson's Disease: An Open-Label Prospective Study. Brain Sci. 2021;11(3)
87. Pauletti C, Locuratolo N, Mannarelli D, Maffucci A, Petritis A, Menini E, Fattapposta F. Fatigue in fluctuating Parkinson's disease patients: possible impact of safinamide. J Neural Transm (Vienna). 2023;130(7):915-23
88. Andersen J, Aabro E, Gulmann N, Hjelmsted A, Pedersen HE. Anti-depressive treatment in Parkinson's disease. A controlled trial of the effect of nortriptyline in patients with Parkinson's disease treated with L-DOPA. Acta Neurol Scand. 1980;62(4):210-9
89. Devos D, Dujardin K, Poirot I, Moreau C, Cottencin O, Thomas P, et al. Comparison of desipramine and citalopram treatments for depression in Parkinson's disease: a double-blind, randomized, placebo-controlled study. Mov Disord. 2008;23(6):850-7
90. Antonini A, Tesei S, Zecchinelli A, Barone P, De Gaspari D, Canesi M, et al. Randomized study of sertraline and low-dose amitriptyline in patients with Parkinson's disease and depression: effect on quality of life. Mov Disord. 2006;21(8):1119-22
91. Meloni M, Puligheddu M, Carta M, Cannas A, Figorilli M, Defazio G. Efficacy and safety of 5-hydroxytryptophan on depression and apathy in Parkinson's disease: a preliminary finding. Eur J Neurol. 2020;27(5):779-86
92. da Silva TM, Munhoz RP, Alvarez C, Naliwaiko K, Kiss A, Andreatini R, Ferraz AC. Depression in Parkinson's disease: a double-blind, randomized, placebo-controlled pilot study of omega-3 fatty-acid supplementation. J Affect Disord. 2008;111(2-3):351-9
93. Mendonça DA, Menezes K, Jog MS. Methylphenidate improves fatigue scores in Parkinson disease: a randomized controlled trial. Mov Disord. 2007;22(14):2070-6
94. Lou JS, Dimitrova DM, Park BS, Johnson SC, Eaton R, Arnold G, Nutt JG. Using modafinil to treat fatigue in Parkinson disease: a double-blind, placebo-controlled pilot study. Clin Neuropharmacol. 2009;32(6):305-10
95. Tyne HL, Taylor J, Baker GA, Steiger MJ. Modafinil for Parkinson's disease fatigue. J Neurol. 2010;257(3):452-6
96. Zhang Q, Yang X, Song H, Jin Y. Cognitive behavioral therapy for depression and anxiety of Parkinson's disease: A systematic review and meta-analysis. Complement Ther Clin Pract. 2020;39:101111
97. Luo F, Ye M, Lv T, Hu B, Chen J, Yan J, et al. Efficacy of Cognitive Behavioral Therapy on Mood Disorders, Sleep, Fatigue, and Quality of Life in Parkinson's Disease: A Systematic Review and Meta-Analysis. Front Psychiatry. 2021;12:793804

98. Xie CL, Wang XD, Chen J, Lin HZ, Chen YH, Pan JL, Wang WW. A systematic review and meta-analysis of cognitive behavioral and psychodynamic therapy for depression in Parkinson's disease patients. Neurol Sci. 2015;36(6):833-43
99. Bomasang-Layno E, Fadlon I, Murray AN, Himelhoch S. Antidepressive treatments for Parkinson's disease: A systematic review and meta-analysis. Parkinsonism Relat Disord. 2015;21(8):833-42; discussion
100. Wang Y, Sun X, Li F, Li Q, Jin Y. Efficacy of non-pharmacological interventions for depression in individuals with Parkinson's disease: A systematic review and network meta-analysis. Front Aging Neurosci. 2022;14:1050715
101. Sturkenboom IH, Graff MJ, Hendriks JC, Veenhuizen Y, Munneke M, Bloem BR, Nijhuis-van der Sanden MW. Efficacy of occupational therapy for patients with Parkinson's disease: a randomised controlled trial. Lancet Neurol. 2014;13(6):557-66
102. Tian J, Kang Y, Liu P, Yu H. Effect of Physical Activity on Depression in Patients with Parkinson's Disease: A Systematic Review and Meta-Analysis. Int J Environ Res Public Health. 2022;19(11)
103. Yang Y, Wang G, Zhang S, Wang H, Zhou W, Ren F, et al. Efficacy and evaluation of therapeutic exercises on adults with Parkinson's disease: a systematic review and network meta-analysis. BMC Geriatr. 2022;22(1):813
104. Gollan R, Ernst M, Lieker E, Caro-Valenzuela J, Monsef I, Dresen A, et al. Effects of Resistance Training on Motor- and Non-Motor Symptoms in Patients with Parkinson's Disease: A Systematic Review and Meta-Analysis. J Parkinsons Dis. 2022;12(6):1783-806
105. Wang LL, Sun CJ, Wang Y, Zhan TT, Yuan J, Niu CY, et al. Effects of dance therapy on non-motor symptoms in patients with Parkinson's disease: a systematic review and meta-analysis. Aging Clin Exp Res. 2022;34(6):1201-8
106. Zhang Q, Hu J, Wei L, Jia Y, Jin Y. Effects of dance therapy on cognitive and mood symptoms in people with Parkinson's disease: A systematic review and meta-analysis. Complement Ther Clin Pract. 2019;36:12-7
107. Wang K, Li K, Zhang P, Ge S, Wen X, Wu Z, et al. Mind-Body Exercises for Non-motor Symptoms of Patients With Parkinson's Disease: A Systematic Review and Meta-Analysis. Front Aging Neurosci. 2021;13:770920
108. Song R, Grabowska W, Park M, Osypiuk K, Vergara-Diaz GP, Bonato P, et al. The impact of Tai Chi and Qigong mind-body exercises on motor and non-motor function and quality of life in Parkinson's disease: A systematic review and meta-analysis. Parkinsonism Relat Disord. 2017;41:3-13
109. Jin X, Wang L, Liu S, Zhu L, Loprinzi PD, Fan X. The Impact of Mind-body Exercises on Motor Function, Depressive Symptoms, and Quality of Life in Parkinson's Disease: A Systematic Review and Meta-analysis. Int J Environ Res Public Health. 2019;17(1)
110. Cugusi L, Solla P, Serpe R, Carzedda T, Piras L, Oggianu M, et al. Effects of a Nordic Walking program on motor and non-motor symptoms, functional performance and body composition in patients with Parkinson's disease. NeuroRehabilitation. 2015;37(2):245-54
111. Sacheli MA, Neva JL, Lakhani B, Murray DK, Vafai N, Shahinfard E, et al. Exercise increases caudate dopamine release and ventral striatal activation in Parkinson's disease. Mov Disord. 2019;34(12):1891-900
112. Sajatovic M, Ridgel AL, Walter EM, Tatsuoka CM, Colón-Zimmermann K, Ramsey RK, et al. A randomized trial of individual versus group-format exercise and self-management in individuals with Parkinson's disease and comorbid depression. Patient Prefer Adherence. 2017;11:965-73
113. Abuoaf R, AlKaabi R, Mohamed Saleh A, Zerough U, Hartley T, van Niekerk SM, et al. The effect of physical exercise on anxiety in people with parkinson's disease: A systematic review of randomized control trials. NeuroRehabilitation. 2023;52(3):387-402

114. (DGPM) DGfPMuÄPeV. S3-Leitlinie Behandlung von Angststörungen, Version 2.0, 06.04.2021. Online: *https://register.awmf.org/assets/guidelines/051-028k_S3_Behandlung-von-Angststoerungen_2021-06.pdf* (abgerufen am 13.06.2023).
115. Herschbach P, Book K, Dinkel A, Berg P, Waadt S, Duran G, et al. Evaluation of two group therapies to reduce fear of progression in cancer patients. Support Care Cancer. 2010;18(4):471-9
116. Jiang C, Luo Y, Qu Y, Wang C, Li Z, Zhou J, Xu Z. Pharmacological and Behavioral Interventions for Fatigue in Parkinson's Disease: A Meta-Analysis of Randomized Controlled Trials. J Geriatr Psychiatry Neurol. 2023:8919887231163291
117. Elbers RG, Verhoef J, van Wegen EE, Berendse HW, Kwakkel G. Interventions for fatigue in Parkinson's disease. Cochrane Database Syst Rev. 2015;2015(10):Cd010925
118. Folkerts AK, Nielsen J, Gollan R, Lansu A, Solfronk D, Monsef I, et al. Physical Exercise as a Potential Treatment for Fatigue in Parkinson's Disease? A Systematic Review and Meta-Analysis of Pharmacological and Non-Pharmacological Interventions. J Parkinsons Dis. 2023;13(5):659-79
119. Bogosian A, Hurt CS, Hindle JV, McCracken LM, Vasconcelos ESDA, Axell S, et al. Acceptability and Feasibility of a Mindfulness Intervention Delivered via Videoconferencing for People With Parkinson's. J Geriatr Psychiatry Neurol. 2022;35(1):155-67

3.14 Impulskontrollstörungen

Autoren: Karsten Witt, Thilo van Eimeren

Fragestellung 139: Welche Testverfahren sind geeignet für die Erfassung von Impulskontroll-störungen bei Pat. mit PK im Vergleich zu Parkinson-Pat. ohne Impulskontrollstörung?

Hintergrund

Eine Metaanalyse unter Einschluss von 30 Studien unter Berücksichtigung von 7.142 Parkinson-Pat. mit einer Erkrankungsdauer von über 3 Jahren berichtet von einer Prävalenz depressiver Störungen von 47,2%, einer Apathie bei 45,5%, einer Angststörung bei 42,9%, psychotischen Störungen bei 19,4% und einer Impulskontrollstörung (IKS) bei 18,5% [1]. Damit sind die IKS die fünfthäufigste neurobehaviorale Störung bei PK. In der bislang größten Studie zur Erhebung von IKS-Symptomen bei über 3.000 Parkinson-Pat. zeigte sich eine IKS bei 14% der Pat. Ein Drittel von ihnen litten unter mehreren Symptomen einer IKS [2]. Eine multizentrische französische Studie belegte eine 5-Jahres-Inzidenz von 46% für eine IKS bei Parkinson-Pat. [3]. IKS bestehen hauptsächlich aus vier Verhaltensstörungen:

- Spielsucht ist definiert als ein anhaltendes oder wiederholtes Spielverhalten, welches dem Pat. durch Geldverluste schadet, dessen Aufgabe mit Dysphorie und Ungehaltenheit beantwortet wird und mit einem nicht kontrollierbaren Spielverhalten einhergeht [4].
- Das pathologische Kaufverhalten ist ein anhaltendes oder wiederholtes Kaufverhalten, welches unangemessen zum Einkommen des Käufers ist oder dass für den Einkauf keine Verwendung besteht. Oftmals kommt es zu finanziellen Verlusten und Schulden [5].
- Hypersexualität ist definiert durch ein abnormes Interesse oder eine übermäßige Beschäftigung mit sexuellen Inhalten, Gedanken, Pornographie und kann mit inadäquatem selbst-stimulierendem Verhalten oder der Inanspruchnahme sexueller Dienstleistungen einhergehen. Selten ist ein exhibitionistisches Verhalten, eine Paraphilie oder eine Zoophilie assoziiert [6].
- Das Binge Eating ist definiert als eine unangemessen hohe Nahrungsaufnahme über einen kurzen Zeitraum, was zu einer Gewichtszunahme führt und mit einer Schädigung des Körpermetabolismus einhergehen kann [7].

Von den IKS können weitere Verhaltensauffälligkeiten abgegrenzt werden, wie z.B. das Dopaminerge Dysregulations Syndrom (DDS) und das Punding [8]. Beim DDS kommt es zu einem unangemessenen Gebrauch von dopaminergen Medikamenten, zumeist schnell wirkenden Medikamente wie lösliches Levodopa, jenseits der Dosis, die für eine Kontrolle motorischer Symptome ausreichend wäre [9]. Das Punding beschreibt ein repetitives, unproduktives und zumeist stereotypes Verhalten [8]. Auch wenn die hier genannten Themenfelder die Majorität der IKS wiedergeben, so können im Einzelfall sehr individuelle pathologische Handlungsmuster mit den Merkmalen einer IKS evident sein, welche u.a. Tattooing, Kleptomanie, rücksichtsloses Autofahren und viele weitere Bereiche betreffen können. Das sehr breite klinische Spektrum an Verhaltensauffälligkeiten bei PK erfordert eine genaue Anamnese, dieses auch jenseits des Einsatzes eines standardisierten Testverfahrens. Da die Verhaltensmuster einer IKS nicht zwingend mit einem persönlichen Leidensdruck für den Pat.

einhergehen und sogar Emotionen der Befriedigung beim Ausführen einer IKS bestehen [3], ist eine Fremdanamnese als Ergänzung zu empfehlen. Da eine IKS zu jedem Zeitpunkt der Erkrankung und auch mit einer deutlichen Latenz mit dem Beginn einer Therapie mit Dopaminagonisten (DA) beginnen kann [10], ist eine anhaltende Aufmerksamkeit zur Detektion einer IKS bei PK geboten.

Da eine IKS mit einem selbst- oder fremdschädigendem Verhalten einhergeht und die Lebensqualität der Pat. erheblich reduziert [11], ist eine adäquate Diagnostik angezeigt.

Evidenzgrundlage

Die Literatursuche in Pubmed erbrachte 386 Studien zu dem Suchterminus „Parkinson disease AND impulse control AND screening OR questionnaire“. Nach Screening der Abstracts und der geeigneten Volltexte wurden keine weiteren Studien aus den Literaturreferenzen extrahiert. Es wurde dem Umstand Rechnung getragen, dass Testinstrumente vorgeschlagen werden, welche in deutscher Sprache validiert sind. Im deutschsprachigen Raum zeigte sich ein validiertes Testverfahren, welches an einer größeren Kohorte untersucht wurde (Evidenzgrad 2).

Ergebnis

Testverfahren: In der Zeit vor 2009 wurden Skalen verwendet, die primär zur Erfassung psychiatrischer Krankheitsbilder entwickelt wurden und somit nicht für die IKS bei PK validiert wurden. Beispiele sind hierfür die Barratt Impulsivity Skala und das Tridimensionale Persönlichkeitsinventar.

Im angloamerikanischen wurde der „Parkinson’s disease dopamine dysregulation syndrome-patient and caregiver (DDS-PC) inventory“ an einer kleinen Fallzahl validiert [12]. Die „Parkinson’s Impulse-Control Scale for the Severity Rating of Impulse Control Behaviors in Parkinson’s Disease“ (PICS) [13] und die Ardouin-Skala [14] sind sensitive Testinstrumente, welche für den angloamerikanischen bzw. den französischen Sprachraum validiert sind [15]. Der „Questionnaire for Impulsive-Compulsive Disorders in Parkinson’s Disease“ (QUIP) liegt auch als validierte Übersetzung in die deutsche Sprache vor. Die Skala enthält 3 Abschnitte. Im ersten Abschnitt werden die IKS (Spielsucht, Kaufsucht, Hypersexualität und Essstörungen), im Abschnitt 2 andere kompulsive Verhaltensmuster (Punding, Hobbyism und ritualisiertes Gehen) und im 3. Abschnitt der Medikamentenübergebrauch abgefragt. Eine Befragung mittels QUIP erfolgt für das Auftreten von IKS-Symptomen sowohl während des gesamten Krankheitsverlaufs (QUIP-anytime) als auch zum Zeitpunkt der Befragung (QUIP-current). Der Fragebogen wird durch die QUIP Rating Scale (QUIP-RS) abgeschlossen, welche die Schwere der Symptomausprägung beschreibt. Gemessen an dem Standard einer ausführlichen psychiatrischen Symptomerhebung, zeigte die amerikanische Originalversion des QUIP eine sehr gute Sensitivität zur Detektion neuropsychiatrischer Störungen im IKS-Spektrum [16]. Auch in der deutschen Übersetzung zeigte sich der QUIP als sensitiver Test mit einer Sensitivität für eine IKS von 68–91% im QUIP-current und 68–91% im QUIP-anytime [17]. Die Autoren/Autorinnen der internationalen, bizentrischen Studie schlossen aus dieser Validierung, dass der QUIP ein geeignetes Screeningverfahren zur Detektion von Symptomen einer IKS im deutschsprachigen Raum darstellt. Einem positiven Test sollte eine ausführliche psychiatrische Exploration folgen, um das individuelle Ausmaß und die

Schwere dieser Symptome, bezogen auf die individuelle Lebenssituation, bewerten zu können und um ein geeignetes Therapiekonzept entwickeln zu können.

In einem von der Movement Disorder Society in Auftrag gegebenen Übersicht zur Bewertung der Skalen zur Erfassung von Symptomen einer IKS wird der QUIP als einziges Screeninginstrument empfohlen [18].

Risikofaktoren: Die Kenntnis über Risikofaktoren einer IKS bei PK erleichtert die Erstellung eines personalisierten Risikoprofils, welches seitens der Therapieaufklärung, der Auftretenswahrscheinlichkeit für eine IKS und der Screeningfrequenz nach Symptomen einer IKS hilfreich sein kann. Die Medikation mit einem DA ist hierbei mit dem höchsten Risiko für eine IKS vergesellschaftet und wird in der folgenden Fragestellung ausführlich dargelegt. Weitere Risikofaktoren sind genetisch bedingt, betreffen unter anderem den Polymorphismus im dopaminergen System (DRD1, DRD2, DRD3, DRD4, COMT, DAT) [19] und sind der routinemäßigen klinischen Testung nicht zugänglich bzw. bei der Vielfalt der Polymorphismen nicht sinnvoll. Weitere Risikofaktoren betreffen eine Alkohol- oder eine Spielsucht in der Familien- oder in der Eigenanamnese des Pat., jüngeres Lebensalter, männliches Geschlecht, Depression, Angststörungen, ein frühes Erkrankungsalter [2] und das Vorliegen einer Alexithymie [20].

Begründung der Empfehlung

Der hier dargestellte diagnostische Test mit dem Ziel, eine IKS qualitativ und quantitativ zu erfassen, beruht auf einer Validierung anhand einer Kohortenstudie, die weitere Empfehlung entspricht einer Expertenmeinung.

**Empfehlung**	**Neu**<br><br>**Stand (2023)**
▪ Für die Erfassung von Impulskontrollstörungen sollte der QUIP-Test verwendet werden.<br>▪ Der QUIP-Test als Screeningtest sollte durch eine Anamnese und eine Fremdanamnese ergänzt werden.	
Konsensstärke: 96,7%, starker Konsens	

Fragestellung 140: Wie effektiv ist die Änderung der dopaminergen medikamentösen Parkinson-Therapie in der Behandlung von Impulskontrollstörungen bei PK im Gegensatz zu keiner Veränderung der medikamentösen Therapie?

Hintergrund

Dopaminagonisten (DA) erhöhen das Risiko für eine IKS um den Faktor 2–3,5 und sind damit der größte Risikofaktor für eine IKS. Die Pathophysiologie der IKS ist unvollständig bekannt. Das mesolimbische dopaminerge System, welches im Belohnungskontext erwartete und erhaltene Belohnungen gegeneinander abwägt [21] (reward prediction error), sowie die Top-down-Kontrolle der für eine Belohnungsinterpretation wichtigen funktionellen Verbindungen

zum anterioren cingulären Kortex (Inhibitionskontrolle) [22], [23] und orbitofrontalen Kortex (Belohnungsbewertung und Aktivierung belohnungsbezogener Erinnerungen [24], [25]) sind bei der Entstehung einer IKS beteiligt [26]. Durch die PK-bedingte Denervierung mesolimbischer Neurone und eine unphysiologische, medikamentöse postsynaptische Stimulation [27] wird eine Sensitivierung von D2/D3-Rezeptoren ausgelöst, welchen eine Schlüsselrolle bei der Entstehung einer IKS zukommt [28] und durch das D2/D3-affine Profil der DA verstärkt wird. Obwohl in diesem Modell die DA als IKS-auslösend in dem Vordergrund stehen, wurden IKS auch unter höheren Dosen von Levodopa [2], Amantadin [29], Levodopa/Carbidopa Intestinales Gel [30] und MAO-B-Hemmern [31] beschrieben, was über ihre direkt oder indirekt dopaminerge Wirkung erklärt werden kann.

IKS wurden bei allen Non-Ergot-DA beschrieben [32], [31], [33]. Die einzelnen DA sind mit einem unterschiedlichen Risiko für die Entwicklung einer IKS verbunden, wobei das Risiko für Pramipexol und Ropinerol höher ist als für den DA Rotgotin [32]. Diese Unterschiede könnten in der arzneispezifischen Affinität für die D2/D3-Rezeptoren, ihrer Wirkdauer, ihrer Applikationsform (oral vs. transdermal), zusammengenommen in Unterschieden der Pharmakokinetik oder Pharmakodynamik begründet sein. Da der DA Piribedil in vielen Ländern nicht erhältlich ist, kann auch ein international unterschiedliches Verordnungsverhalten dieses Bild mit verzerren (und z.B. für Piribedil seltener berichtet werden).

Diese Informationen führen zur ersten Teilfrage dieser Leitlinie mit der Frage, ob der Wechsel zwischen den DA beim Auftreten einer IKS empfohlen werden kann. Neben dem Absetzen der DA, was oft mit einer für den Pat. nicht akzeptablen Verschlechterung motorischer Funktionen einhergeht, ist der Wechsel zu einer kontinuierlichen Levodopa/Carbidopa intestinalem Gel-Infusion eine denkbare Möglichkeit, motorische Funktionen aufrechtzuerhalten und DA zu meiden.

Evidenzgrundlage

Eine Literatursuche in Pubmed mit den Suchterminus „Parkinson AND impulse control AND management OR treatment“ ergab 532 Treffer, die nach Titel, Abstract und im geeigneten Fall im Volltext hinsichtlich der drei Unterfragestellungen bewertet wurden.

Die Frage nach der Effektivität der Änderung der dopaminergen Medikation zur Behandlung einer IKS impliziert mehrere mögliche Änderungen der dopaminergen Medikation, die hier separat recherchiert wurden.

1. Der Wechsel zwischen den DA, dieses mit der Rationale, dass eine unterschiedliche D2/D3-Rezeptoraffinität Auswirkungen auf das klinische Bild hat.

   Hier wurden eine retrospektive Studie und Fallserien gefunden. Randomisierte Studien zu diesem Thema liegen nicht vor.
2. Änderung der dopaminergen Medikation durch eine Dosisreduktion der DA bis hin zum Absetzen. Dieses führt oft zu einem Beginn oder einer Dosisanpassung von Levodopa.

   Hierzu liegen Beobachtungsstudien vor, keine randomisierten klinischen Studien.

3. Die Änderung der dopaminergen Medikation kann den Wechsel der DA-Therapie zu einer Therapie mit Levodopa/Carbidopa intestinalem Gel (LCIG)- oder Levodopa/Entacapon/Carbidopa intestinalem Gel (LECIG) beinhalten.

Für die Unterfrage 3 wurde eine erweiterte Suche notwendig, um die geeignete Literatur zu finden. Die Literaturrecherche erfolgte mit dem Suchterminus „Parkinson AND impulse control AND intestinal OR jejunal OR gel“. Unter den 17 Treffern fanden sich wenige Fallserien und eine Beobachtungsstudie.

Ergebnis

Wechsel des DA zur Behandlung einer IKS:

In einer Post-hoc-Analyse von Phase-4-Studien zum Wirkstoff Rotigotin wurden die Daten von insgesamt 786 Parkinson-Pat. in einem Zeitraum von bis zu 6 Jahren ausgewertet. Bei 9% der Pat. wurde eine IKS als unerwünschte Wirkung berichtet, 97% dieser IKS wurden als milde bis moderat eingestuft und führten bei 9,9% dieser Pat. zum Absetzen von Rotigotin [10]. In einer prospektiven Beobachtungsstudie unter Einschluss von 233 Parkinson-Pat., die einen DA einnahmen, waren bei 39% der Pat. die Kriterien einer IKS erfüllt. Pramipexol und Ropinerol führten häufiger zu einer IKS (42%) als Rotigotin (19%, $p < 0.01$) [31]. Eine retrospektive Analyse von 82 Pat., die auf eine subkutane Apomorphintherapie wechselten, schloss 7 Pat. mit einer IKS ein. Zwar besserte sich oder verschwand die IKS bei diesen Pat., 6 Pat. erkrankten jedoch neu an einer IKS [34]. In einer prospektiven, multizentrischen europäischen Studie wurden 425 Parkinson-Pat., die auf einen DA eingestellt waren, hinsichtlich des Auftretens einer IKS beobachtet [35]. Die Prävalenz für eine IKS lag für Rotigotin bei 4,9%, für Pramipexol retardiert bei 6,6% und für Pramipexol unretadiert bei 19% [35]. Studien, die im Rahmen einer IKS auf einen DA mit geringerem IKS-Risiko wechseln, fanden sich in der Literaturrecherche nicht. Aus diesen Daten lässt sich schließen, dass die DA unterschiedlich hohe Risiken für eine IKS besitzen, ein Wechsel zu einem anderen DA zur Behandlung einer IKS lässt sich hieraus nicht ableiten.

Absetzen der DA zur Behandlung einer IKS:

In einer frühen Pat.-Serie zu diesem Thema zeigte sich, dass 80% der Pat. mit einer IKS nach Dosisreduktion die IKS verloren, nach dem Absetzen der DA waren alle beobachteten Pat. frei von einer IKS [36]. Eine Nachbeobachtung von 30 Parkinson-Pat. mit einer IKS unter einem DA, deren DA-Therapie beendet wurde, zeigte einen Rückgang der IKS bei 50% im ersten Jahr und bei weiteren 20% im zweiten Jahr nach dem Absetzen des DA [3]. Auch die deutliche Dosisreduktion war effektiv. Das Absetzen des DA ist somit eine effektive Behandlung einer IKS, die unter einem DA aufgetreten ist. Wenn das Absetzen nicht gelingt, z.B. im Rahmen eines Dopamin-Agonisten Entzugssyndroms (DAWS), sollte schrittweise auf die niedrigste tolerable Dosierung reduziert werden.

Wechsel der DA zu einer kontinuierlichen Levodopa/Carbidopa intestinalen Gel (LCIG)-Infusion oder einre Levodopa/Entacapon/Carbidopa intestinalen Gel (LECIG)-Infusion:

Eine Fallserie beschreibt 8 Parkinson-Pat., die unter einem DA eine IKS entwickelten und auf eine LCIG-Therapie umgestellt wurden. Nach einer Therapiedauer von 3 Jahren litten noch 2 der 8 Parkinson-Pat. unter einer IKS [37]. In einer internationalen prospektiven Beobachtungsstudie

wurden 62 Parkinson-Pat. über einen Zeitraum von 6 Monaten untersucht. Der QUIP-RS-Summenwert verminderte sich um 64,4% im Verlauf von 6 Monaten [38]. In einer weiteren Fallserie wurden 6 Parkinson-Pat. mit einer IKS auf eine LCIG Therapie umgestellt. Nach 24 Wochen waren bei allen Pat. die IKS-Symptome „nahezu vollständig" verschwunden, so die Autoren/Autorinnen [39]. In allen Fallserien zu diesem Thema wurden Pat. nicht wegen, sondern mit ihrer Komorbidität einer IKS auf eine LCIG-Therapie umgestellt. Die klinischen und demographischen Daten dieser Fallserien zeigen, dass motorische Fluktuationen vorhanden waren, die Pat. somit eine Indikation für eine nicht orale Folgetherapie hatten. Zu der Arznei LECIG fanden sich keine Studien zu diesem Thema. Somit zeigen die Fallserien, dass bei Parkinson-Pat., die eine Indikation für eine nicht orale Folgetherapie haben, eine LCIG-Therapie effektiv eine IKS behandeln kann.

Begründung der Empfehlung

Die Evidenz beruht auf Beobachtungsstudien sowie Fallberichten oder Fallserien ohne randomisierte, kontrollierte Studien. Therapeutische Ansätze können aus diesen vorliegenden Studien herangezogen und auf mittlerer Evidenzbasis empfohlen werden.

**Empfehlung**	**Neu**<br><br>**Stand (2023)**
▪ Vor dem Einsatz von Dopaminagonisten sollten Pat. für das Auftreten einer IKS sensibilisiert werden.<br>▪ Das Absetzen des Dopaminagonisten ist eine effektive Behandlung einer IKS. Wir empfehlen eine schrittweise Reduktion der DA.<br>▪ Im Fall von unerwünschten Wirkungen der Dopaminagonisten-Reduktion (z.B. Dopaminagonisten-Entzugssyndrom) sollten die DA auf die niedrigste, tolerierte Dosierung reduziert werden.<br>▪ Für Pat. mit einer Indikation für eine nicht orale Folgetherapie kann eine Levodopa-Carbidopa Intestinale Gel (LCIG)-Therapie zur Behandlung einer IKS erwogen werden.	
Konsensstärke: 100%, starker Konsens	

Fragestellung 141: Wie effektiv sind nicht dopaminerge Pharmakotherapieansätze in der Behandlung von Impulskontrollstörungen bei PK im Gegensatz zu keinen nicht dopaminergen Pharmakotherapieansätzen bzw. Placebo?

Hintergrund

Obwohl das Absetzen der DA-Therapie eine effektive Behandlungsmethode für eine IKS bei PK darstellt, kann eine starke Dosisreduktion oder das Absetzen des DA zu einer intolerablen Verschlechterung des motorischen Status führen, nicht motorische Symptome verschlechtern und/oder auch ein DA-Entzugssyndrom auslösen. Diese Folgesymptome sind für die Pat. oft nicht tolerabel. In diesen Fällen kann die DA-Therapie nicht abgesetzt werden oder nicht ausreichend reduziert werden. Ein Jahr nach DA-Beendigung können noch bis zu 50% der Parkinson-Pat. eine manifeste IKS zeigen und nach 2 Jahren sind es noch 30% [3]. Aus diesem Grund wurde nach

Pharmakotherapien gesucht, welche nicht auf der Basis eines dopaminergen Mechanismus die IKS behandelt. Hierbei fokussierte sich die Prüfung u.a. auf Substanzen, die erfolgreich zur Suchtbekämpfung genutzt werden.

Evidenzgrundlage

Die Literatursuche in Pubmed inkludierte den Suchterminus „Parkinson AND impulse control AND management OR treatment“ und erbrachte 517 Treffer, die systematisch in Hinblick auf nicht dopaminerge Therapieoptionen durchsucht wurden. Es fanden sich 2 placebokontrollierte Studien und zahlreiche Fallserien und Fallberichte.

Ergebnis

Naltrexon, ein Opioidrezeptor-Antagonist, wurde in einer randomisierte placebokontrollierten Studie an 50 Parkinson-Pat. mit IKS untersucht. In einer täglichen Dosis zwischen 50 und 100 mg verfehlte Naltrexon den primären Endpunkt (Clinical Global Impression-Severity), der sekundäre Endpunkt (Reduktion der Schwere von IKS-Symptomen im QUIP-RS) wurde jedoch erreicht [40].

In einer doppelblinden Crossover-Studie wurde die Effektivität von Amantadin in einer Dosierung von 200 mg/d auf die Spielsucht bei entsprechend erkrankten Parkinson-Pat. untersucht. In dieser 17 Wochen langen Studie wurde die Dosierung der DA konstant gehalten. Die Spielsucht verschwand unter Amantadin oder wurde erheblich reduziert. Der Therapieeffekt setzte schon nach 4 Tagen ein [41]. Es gibt Fallberichte, welche eine Entwicklung einer IKS unter Amantadin beschreiben [42], [43], dieses fiel auch in einer epidemiologischen Studie zu diesem Thema auf [2].

15 Parkinson-Pat., die auch unter einer Dosisreduktion von DA und Levodopa keine Besserung ihrer IKS zeigten, wurden in einer offenen Behandlungsstudie mit dem Antikonvulsivum Zonisamid in einer Dosierung von bis zu 200 mg/d behandelt und zeigten eine klinische Besserung in der Clinical Global Impression Scale und der Barratt Impulsiveness Scale [44].

In einer Fallserie von 4 Pat. führte der Einsatz von Clozapin (12,5–37,5mg/d) trotz Fortführung der DA zu einer kompletten Remission der IKS [45]. Weitere positive Fallberichte zu Clozapin existieren [46], [47]. Clozapin birgt das Risiko einer dosisunabhängigen Agranulozytose. 3 Pat. wurden erfolgreich mit Valproinsäure therapiert [48].

Einzelberichte beschreiben den Einsatz von Risperidon und Quetiapin in niedriger Dosierung [47].

Alle hier genannten Therapien erfolgen off-label, es gibt keine zugelassenen Medikamente zur Behandlung einer IKS bei PK.

Begründung der Empfehlung

Auf der Basis der oben dargestellten Evidenzgrundlage findet sich nur eine placebokontrollierte doppelt verblindete Studie bei Pat. mit einer Spielsucht.

Empfehlung	Neu<br>Stand (2023)
Nicht dopaminerge Therapieansätze sollten aufgrund von unzureichender Evidenz nicht eingesetzt werden.	
Konsensstärke: 96,7%, starker Konsens	

Fragestellung 142: Wie effektiv sind verhaltenstherapeutische Ansätze in der Behandlung von Impulskontrollstörungen bei PK im Gegensatz zu keiner verhaltenstherapeutischen Therapie?

Hintergrund

Viele Pat. haben den verständlichen Wunsch nach einer nicht pharmakologischen Therapie ihrer Krankheitssymptome. Zudem zeigt sich – wie oben dargelegt – dass selbst das Absetzen der DA nicht sofort und in einem Teil der Pat. nicht vollständig die IKS-Symptome zur Remission bringt. Das Absetzen der DA birgt zudem das Risiko für eine Verschlechterung des motorischen Status und einer höheren Symptomlast nicht motorischer Symptome bis hin zum DA-Entzugssyndrom. Daher sind nicht pharmakologische Therapieansätze allein oder in Kombination mit einem besten medikamentösen Management willkommen.

Evidenzgrundlage

Die Literatursuche in Pubmed erbrachte nur 11 Treffer für den Suchterminus „Parkinson AND impulse control AND cognitive-behavioral OR psychological therapy OR psychological intervention OR CBT“. Nach Screening der Abstracts und Inhalte wurden weitere Studien aus den Literaturreferenzen extrahiert und letztendlich 19 Artikel selektiert, welche in diese Leitlinie einbezogen wurden. Es fanden sich eine randomisierte, kontrollierte Studie mit einer Fallzahl von 27 Parkinson-Pat. in dieser Literaturrecherche sowie mehrere Falldarstellungen und Übersichtsartikel.

Ergebnis

Eine Studie prüfte die Effektivität einer kognitiven Verhaltenstherapie bei Parkinson-Pat. mit einer IKS. Die Psychotherapie wurde direkt auf die Bedürfnisse der Pat. und ihrer Angehörigen zugeschnitten und umfasste 12 Therapiestunden, in welchen eine ausführliche Psychoedukation über Störung des Affekts und der Kognition, die IKS und ihre Auswirkungen auf das soziale Funktionieren erfolgte. Es wurden Techniken zur Vermeidung und zur Bewältigung der IKS-Symptome vermittelt. Die kognitive Verhaltenstherapie beinhaltete das A-B-C-Modell von Albert Ellis (Activation experiences – Beliefs – Consequences). Die relativ kleine Studie randomisierte 10 Parkinson-Pat. mit IKS in den Therapiearm einer kognitiven Verhaltenstherapie und beließ 17 Parkinson-Pat. mit einer IKS auf der Warteliste als Kontrollgruppe. Die kognitive Verhaltenstherapie führte zu einer signifikanten Besserung der IKS-Symptome und auch der Ergebnisse im Neuropsychiatrischen Inventar (NPI) besserten sich. Die Studie zeigte die Machbarkeit und auch die Effektivität einer kognitiven Verhaltenstherapie bei der Behandlung einer IKS bei PK [49].

Begründung der Empfehlung

Auf der Basis der oben dargestellten Evidenzgrundlage wird zum Einsatz einer kognitiven Verhaltenstherapie zur Behandlung einer IKS bei Parkinson-Pat. die u.g. Empfehlung gegeben.

**Empfehlung**	**Neu**<br>**Stand (2023)**
Eine kognitive Verhaltenstherapie kann zur Behandlung einer IKS eingesetzt werden.	
Konsensstärke: 100%, starker Konsens	

Fragestellung 143: Wie effektiv ist die STN-DBS bei der Behandlung von IKS bei PK im Vergleich zur Behandlung ohne STN-DBS bei PK mit IKS?

Hintergrund

Die frühe Krankheitsmanifestation und ein jüngeres Lebensalter sind Risikofaktoren für die Entwicklung einer IKS bei PK. Eine IKS tritt zumeist nicht sofort nach dem Beginn einer DA-Therapie auf, es können Jahre vergehen [10]. Pat. mit einer IKS zeigen nicht selten Peak-dose-Dyskinesien. Diese Eigenschaften finden sich auch bei Parkinson-Pat., die für eine nicht orale Folgetherapie geeignet sind und hier insbesondere für eine STN-DBS.

Nach einer STN-DBS kann in der Regel die dopaminerge Medikation um ca. 50% reduziert werden und die motorischen Fertigkeiten bleiben gleich oder werden besser [50]. Diese Medikamentenreduktion ist hinsichtlich der Behandlung einer IKS wünschenswert. Hypodopaminerge Verhaltensmuster sind durch ein amotivationales Verhalten gekennzeichnet, welches sich durch eine geringe intrinsische Stimulation auszeichnet. Klinisch ist die Apathie das Hauptmerkmal dieses hypodopaminergen Zustandsbilds. Ein hyperdopaminerges Verhaltensmuster ist durch eine hohe Impulsivität, Ideenreichtum, eine Zunahme innerer Handlungsimpulse gekennzeichnet und begünstigt eine IKS [51]. Zwischen diesen beiden Polen kann es bei Pat. bereits im mittleren Erkrankungsstadium zu vermehrten nicht motorischen Fluktuationen kommen, welche durch das Auf und Ab der dopaminergen Stimulation vor und nach Medikamenteneinnahme auftreten. Nach einer STN-DBS nehmen diese nicht motorischen Fluktuationen ab, das dopaminerge Stimulationsniveau bleibt konstanter [52]. Mit der mit der STN-DBS verbundenen Reduktion der dopaminergen Medikation geht eine Desensitivierung dopaminerger Rezeptoren einher. So führt z.B. eine weit überschwellige Levodopa-Dosis vor der STN-DBS zu psychostimulierender Reaktion, welche nach einem Jahr einer STN-DBS ausbleibt [53]. Dieser desensitivierende Effekt ist vermutlich ein Kernelement der positiven Wirkung der STN-DBS auf die IKS [54]. Die STN-DBS kann jedoch auch die Impulsivität erhöhen [55] und im geeigneten Fall eine IKS auslösen, dieses ist klinisch jedoch selten relevant.

Evidenzgrundlage

Es erfolgte eine Literaturrecherche in Pubmed mit dem Suchterminus „Parkinson disease AND impulse control AND STN OR DBS“, so wurden 92 Treffer identifiziert. Einzelfallbeschreibungen,

Fallserien, retrospektive Betrachtungen und eine prospektive Studie sowie zahlreiche Übersichtsarbeiten und Expertenmeinungen zeigten sich bei der Durchschau der Treffer.

Ergebnis

Einige Studien [56], [57], [58], [59] zu diesem Thema berichten ihre Daten unvollständig und entziehen sich daher einer systematischen Bewertung [60]. In einigen weiteren Beobachtungsstudien [61], [62] werden Parkinson-Pat. mit einer IKS oder einem DDS einer unilateralen oder bilateralen Therapie mit den Zielpunkten STN oder GPI zugeführt. Diese zeigen eine Reduktion der IKS nach bilateraler STN-DBS, es werden aber postoperativ unerwartet hohe Fallzahlen von neuen IKS berichtet, z.T. bei Pat. mit einer postoperativen täglichen Levodopa-Äquivalenzdosis von 2250 mg und einer Suchterkrankung in der Vorgeschichte [61]. Viele Studien aus diesem Feld sind retrospektiv, nicht explizit mit der Fragestellung, wie sich eine DBS auf eine IKS auswirkt, durchgeführt worden. Diese Studien berichten aus einem Spektrum von „impulse control and related disorders“, ohne dieses scharf zu definieren [63]. Diese Studien können keinen klaren Beitrag bei der Bewertung der Fragestellung erbringen.

Ein klareres Bild vermitteln randomisierte Studien, wie eine Subanalyse aus der Earlystim-Studie, die eine Abnahme von nicht motorischen Fluktuationen und eine Abnahme hyperdopaminerger Verhaltensmuster nach der STN-DBS berichtet [54]. Eine weitere retrospektive Studie unter Einschluss von 69 Parkinson-Pat. zeigte eine signifikante Reduktion des hyperdopaminergen Verhaltens, gemessen an der Ardouin Skala, es zeigten sich aber gehäuft Fälle einer postoperativen Apathie [64]. In einer Fallserie mit 7 Parkinson-Pat. mit einer Spielsucht waren diese nach 18 Monaten nach einer STN-DBS in kompletter Remission [65]. Weitere Fallserien mit insgesamt 5 weiteren Parkinson-Pat. berichten ebenfalls einen positiven Verlauf der IKS nach einer STN-DBS [58], [66], [67]. In einer aktuellen prospektiven Studie wurden 217 Parkinson-Pat. mittels STN-DBS therapiert. 23 Parkinson-Pat. litten unter einer IKS, die ein Jahr nach der OP bei 22 Parkinson-Pat. nicht mehr nachweisbar war. 8 Pat. entwickelten jedoch neu eine IKS. Während die Remission der IKS mit der Medikamentenreduktion assoziiert war, war das Neuauftreten einer IKS mit dem Ausmaß der Apathie präoperativ assoziiert [68].

Die STN-DBS kann über eine Reduktion der dopaminergen Medikation zu einer partiellen oder einer vollständigen Remission einer IKS bei PK führen [58], [66], [67], [64], [65]. Das Zusammenspiel zwischen Medikamentenreduktion und stimuliationsbedingter Zunahme einer Impulsivität ist jedoch komplex und nicht für jeden Pat. mit einer IKS prädizierbar. Die Reduktion hoher Medikamentendosen postoperativ kann zu einer Apathie führen [64], [69], was bei dem postoperativen Management mitberücksichtigt werden muss. Die bisherigen Studien und Fallberichte zu diesem Thema berichteten über Pat., die auch ohne IKS eine Indikation für eine STN-DBS hatten. Es gibt also keine Studie, die jenseits der etablierten Indikation für eine STN-DBS die Auswirkungen der STN-DBS auf eine IKS bei PK untersucht. Dieses limitiert die Aussagekraft der Studien auf das Spektrum der Pat., die eine Indikation für eine nicht orale Folgetherapie haben.

Begründung der Empfehlung

Die Evidenz beruht auf Beobachtungsstudien sowie Fallberichten oder Fallserien ohne randomisierte, kontrollierte Studien. Therapeutische Ansätze können aus diesen vorliegenden Studien herangezogen und auf mittlerer Evidenzbasis empfohlen werden.

Empfehlung	Neu<br>Stand (2023)
Die bilaterale STN-DBS ist eine effektive Therapie zur Behandlung einer IKS für Pat., die eine Indikation für eine nicht orale Folgetherapie haben.	
Konsensstärke: 100%, starker Konsens	

Referenzen

1. Macías-García P, Rashid-López R, Cruz-Gómez Á J, Lozano-Soto E, Sanmartino F, Espinosa-Rosso R, González-Rosa JJ. Neuropsychiatric Symptoms in Clinically Defined Parkinson's Disease: An Updated Review of Literature. Behav Neurol. 2022;2022:1213393
2. Weintraub D, Koester J, Potenza MN, Siderowf AD, Stacy M, Voon V, et al. Impulse control disorders in Parkinson disease: a cross-sectional study of 3090 patients. Arch Neurol. 2010;67(5):589-95
3. Corvol JC, Artaud F, Cormier-Dequaire F, Rascol O, Durif F, Derkinderen P, et al. Longitudinal analysis of impulse control disorders in Parkinson disease. Neurology. 2018;91(3):e189-e201
4. Nautiyal KM, Okuda M, Hen R, Blanco C. Gambling disorder: an integrative review of animal and human studies. Ann N Y Acad Sci. 2017;1394(1):106-27
5. Dittmar H. Compulsive buying--a growing concern? An examination of gender, age, and endorsement of materialistic values as predictors. Br J Psychol. 2005;96(Pt 4):467-91
6. Lim SY, Evans AH, Miyasaki JM. Impulse control and related disorders in Parkinson's disease: review. Ann N Y Acad Sci. 2008;1142:85-107
7. Nirenberg MJ, Waters C. Compulsive eating and weight gain related to dopamine agonist use. Mov Disord. 2006;21(4):524-9
8. Weintraub D, Aarsland D, Biundo R, Dobkin R, Goldman J, Lewis S. Management of psychiatric and cognitive complications in Parkinson's disease. Bmj. 2022;379:e068718
9. Giovannoni G, O'Sullivan JD, Turner K, Manson AJ, Lees AJ. Hedonistic homeostatic dysregulation in patients with Parkinson's disease on dopamine replacement therapies. J Neurol Neurosurg Psychiatry. 2000;68(4):423-8
10. Antonini A, Chaudhuri KR, Boroojerdi B, Asgharnejad M, Bauer L, Grieger F, Weintraub D. Impulse control disorder related behaviours during long-term rotigotine treatment: a post hoc analysis. Eur J Neurol. 2016;23(10):1556-65
11. Alvarado-Bolaños A, Cervantes-Arriaga A, Rodríguez-Violante M, Llorens-Arenas R, Calderón-Fajardo H, Millán-Cepeda R, et al. Impact of Neuropsychiatric Symptoms on the Quality of Life of Subjects with Parkinson's Disease. J Parkinsons Dis. 2015;5(3):541-8
12. Cabrini S, Baratti M, Bonfà F, Cabri G, Uber E, Avanzi M. Preliminary evaluation of the DDS-PC inventory: a new tool to assess impulsive-compulsive behaviours associated to dopamine replacement therapy in Parkinson's disease. Neurol Sci. 2009;30(4):307-13
13. Okai D, Askey-Jones S, Mack J, Martin A, Chaudhuri KR, Samuel M, et al. Parkinson's Impulse-Control Scale for the Severity Rating of Impulse-Control Behaviors in Parkinson's Disease: A Semistructured Clinical Assessment Tool. Mov Disord Clin Pract. 2016;3(5):494-9

14. Ardouin C, Chéreau I, Llorca PM, Lhommée E, Durif F, Pollak P, Krack P. [Assessment of hyper- and hypodopaminergic behaviors in Parkinson's disease]. Rev Neurol (Paris). 2009;165(11):845-56
15. Rieu I, Martinez-Martin P, Pereira B, De Chazeron I, Verhagen Metman L, Jahanshahi M, et al. International validation of a behavioral scale in Parkinson's disease without dementia. Mov Disord. 2015;30(5):705-13
16. Weintraub D, Hoops S, Shea JA, Lyons KE, Pahwa R, Driver-Dunckley ED, et al. Validation of the questionnaire for impulsive-compulsive disorders in Parkinson's disease. Mov Disord. 2009;24(10):1461-7
17. Probst CC, Winter LM, Möller B, Weber H, Weintraub D, Witt K, et al. Validation of the questionnaire for impulsive-compulsive disorders in Parkinson's disease (QUIP) and the QUIP-rating scale in a German speaking sample. J Neurol. 2014;261(5):936-42
18. Evans AH, Okai D, Weintraub D, Lim SY, O'Sullivan SS, Voon V, et al. Scales to assess impulsive and compulsive behaviors in Parkinson's disease: Critique and recommendations. Mov Disord. 2019;34(6):791-8
19. Hall A, Weaver SR, Compton LJ, Byblow WD, Jenkinson N, MacDonald HJ. Dopamine genetic risk score predicts impulse control behaviors in Parkinson's disease. Clin Park Relat Disord. 2021;5:100113
20. Goerlich-Dobre KS, Probst C, Winter L, Witt K, Deuschl G, Möller B, van Eimeren T. Alexithymia-an independent risk factor for impulsive-compulsive disorders in Parkinson's disease. Mov Disord. 2014;29(2):214-20
21. Clark CA, Dagher A. The role of dopamine in risk taking: a specific look at Parkinson's disease and gambling. Front Behav Neurosci. 2014;8:196
22. Paulus MP, Hozack N, Frank L, Brown GG. Error rate and outcome predictability affect neural activation in prefrontal cortex and anterior cingulate during decision-making. Neuroimage. 2002;15(4):836-46
23. Tessitore A, Santangelo G, De Micco R, Vitale C, Giordano A, Raimo S, et al. Cortical thickness changes in patients with Parkinson's disease and impulse control disorders. Parkinsonism Relat Disord. 2016;24:119-25
24. O'Doherty J, Kringelbach ML, Rolls ET, Hornak J, Andrews C. Abstract reward and punishment representations in the human orbitofrontal cortex. Nat Neurosci. 2001;4(1):95-102
25. Volkow ND, Fowler JS, Wang GJ. The addicted human brain viewed in the light of imaging studies: brain circuits and treatment strategies. Neuropharmacology. 2004;47 Suppl 1:3-13
26. Probst CC, van Eimeren T. The functional anatomy of impulse control disorders. Curr Neurol Neurosci Rep. 2013;13(10):386
27. Cools R, Lewis SJ, Clark L, Barker RA, Robbins TW. L-DOPA disrupts activity in the nucleus accumbens during reversal learning in Parkinson's disease. Neuropsychopharmacology. 2007;32(1):180-9
28. Drew DS, Muhammed K, Baig F, Kelly M, Saleh Y, Sarangmat N, et al. Dopamine and reward hypersensitivity in Parkinson's disease with impulse control disorder. Brain. 2020;143(8):2502-18
29. Weintraub D, Sohr M, Potenza MN, Siderowf AD, Stacy M, Voon V, et al. Amantadine use associated with impulse control disorders in Parkinson disease in cross-sectional study. Ann Neurol. 2010;68(6):963-8
30. Contreras Chicote A, Velilla Alonso G, Mas Serrano M, Grandas Perez F. New-onset impulse control disorders after treatment with levodopa-carbidopa intestinal gel in Parkinson's disease. Neurologia (Engl Ed). 2023;38(3):220-1
31. Garcia-Ruiz PJ, Martinez Castrillo JC, Alonso-Canovas A, Herranz Barcenas A, Vela L, Sanchez Alonso P, et al. Impulse control disorder in patients with Parkinson's disease under dopamine agonist therapy: a multicentre study. J Neurol Neurosurg Psychiatry. 2014;85(8):840-4

32. Gungabissoon U, Kirichek O, El Baou C, Galwey N. Comparison of long-term use of prolonged-release ropinirole and immediate-release dopamine agonists in an observational study in patients with Parkinson's disease. Pharmacoepidemiol Drug Saf. 2020;29(5):591-8
33. Micheli FE, Giugni JC, Espinosa ME, Calvo DS, Raina GB. Piribedil and pathological gambling in six parkinsonian patients. Arq Neuropsiquiatr. 2015;73(2):115-8
34. García Ruiz PJ, Sesar Ignacio A, Ares Pensado B, Castro García A, Alonso Frech F, Alvarez López M, et al. Efficacy of long-term continuous subcutaneous apomorphine infusion in advanced Parkinson's disease with motor fluctuations: a multicenter study. Mov Disord. 2008;23(8):1130-6
35. Rizos A, Sauerbier A, Antonini A, Weintraub D, Martinez-Martin P, Kessel B, et al. A European multicentre survey of impulse control behaviours in Parkinson's disease patients treated with short- and long-acting dopamine agonists. Eur J Neurol. 2016;23(8):1255-61
36. Mamikonyan E, Siderowf AD, Duda JE, Potenza MN, Horn S, Stern MB, Weintraub D. Long-term follow-up of impulse control disorders in Parkinson's disease. Mov Disord. 2008;23(1):75-80
37. Todorova A, Samuel M, Brown RG, Chaudhuri KR. Infusion Therapies and Development of Impulse Control Disorders in Advanced Parkinson Disease: Clinical Experience After 3 Years' Follow-up. Clin Neuropharmacol. 2015;38(4):132-4
38. Catalan MJ, Molina-Arjona JA, Mir P, Cubo E, Arbelo JM, Martinez-Martin P. Improvement of impulse control disorders associated with levodopa-carbidopa intestinal gel treatment in advanced Parkinson's disease. J Neurol. 2018;265(6):1279-87
39. Catalán MJ, de Pablo-Fernández E, Villanueva C, Fernández-Diez S, Lapeña-Montero T, García-Ramos R, López-Valdés E. Levodopa infusion improves impulsivity and dopamine dysregulation syndrome in Parkinson's disease. Mov Disord. 2013;28(14):2007-10
40. Papay K, Xie SX, Stern M, Hurtig H, Siderowf A, Duda JE, et al. Naltrexone for impulse control disorders in Parkinson disease: a placebo-controlled study. Neurology. 2014;83(9):826-33
41. Thomas A, Bonanni L, Gambi F, Di Iorio A, Onofrj M. Pathological gambling in Parkinson disease is reduced by amantadine. Ann Neurol. 2010;68(3):400-4
42. Walsh RA, Lang AE. Multiple impulse control disorders developing in Parkinson's disease after initiation of amantadine. Mov Disord. 2012;27(2):326
43. Beal E. Parkinson disease: Amantadine administration is associated with impulse control disorders in PD. Nat Rev Neurol. 2011;7(2):62
44. Bermejo PE, Ruiz-Huete C, Anciones B. Zonisamide in managing impulse control disorders in Parkinson's disease. J Neurol. 2010;257(10):1682-5
45. Liang J, Groves M, Shanker VL. Clozapine Treatment for Impulse Control Disorders in Parkinson's Disease Patients: A Case Series. Mov Disord Clin Pract. 2015;2(3):283-5
46. Bonfils NA, Benyamina A, Aubin HJ, Luquiens A. Clozapine use for refractory impulse control disorders in Parkinson's disease: a case report. Psychopharmacology (Berl). 2015;232(19):3677-9
47. Raja M, Bentivoglio AR. Impulsive and compulsive behaviors during dopamine replacement treatment in Parkinson's Disease and other disorders. Curr Drug Saf. 2012;7(1):63-75
48. Hicks CW, Pandya MM, Itin I, Fernandez HH. Valproate for the treatment of medication-induced impulse-control disorders in three patients with Parkinson's disease. Parkinsonism Relat Disord. 2011;17(5):379-81
49. Okai D, Askey-Jones S, Samuel M, O'Sullivan SS, Chaudhuri KR, Martin A, et al. Trial of CBT for impulse control behaviors affecting Parkinson patients and their caregivers. Neurology. 2013;80(9):792-9
50. Alexoudi A, Shalash A, Knudsen K, Witt K, Mehdorn M, Volkmann J, Deuschl G. The medical treatment of patients with Parkinson's disease receiving subthalamic neurostimulation. Parkinsonism Relat Disord. 2015;21(6):555-60; discussion

51. Volkmann J, Daniels C, Witt K. Neuropsychiatric effects of subthalamic neurostimulation in Parkinson disease. Nat Rev Neurol. 2010;6(9):487-98
52. Lhommée E, Klinger H, Thobois S, Schmitt E, Ardouin C, Bichon A, et al. Subthalamic stimulation in Parkinson's disease: restoring the balance of motivated behaviours. Brain. 2012;135(Pt 5):1463-77
53. Castrioto A, Kistner A, Klinger H, Lhommée E, Schmitt E, Fraix V, et al. Psychostimulant effect of levodopa: reversing sensitisation is possible. J Neurol Neurosurg Psychiatry. 2013;84(1):18-22
54. Lhommée E, Wojtecki L, Czernecki V, Witt K, Maier F, Tonder L, et al. Behavioural outcomes of subthalamic stimulation and medical therapy versus medical therapy alone for Parkinson's disease with early motor complications (EARLYSTIM trial): secondary analysis of an open-label randomised trial. Lancet Neurol. 2018;17(3):223-31
55. Ballanger B, van Eimeren T, Moro E, Lozano AM, Hamani C, Boulinguez P, et al. Stimulation of the subthalamic nucleus and impulsivity: release your horses. Ann Neurol. 2009;66(6):817-24
56. Temel Y, Kessels A, Tan S, Topdag A, Boon P, Visser-Vandewalle V. Behavioural changes after bilateral subthalamic stimulation in advanced Parkinson disease: a systematic review. Parkinsonism Relat Disord. 2006;12(5):265-72
57. Lu C, Bharmal A, Suchowersky O. Gambling and Parkinson disease. Arch Neurol. 2006;63(2):298
58. Witjas T, Baunez C, Henry JM, Delfini M, Regis J, Cherif AA, et al. Addiction in Parkinson's disease: impact of subthalamic nucleus deep brain stimulation. Mov Disord. 2005;20(8):1052-5
59. Houeto JL, Mesnage V, Mallet L, Pillon B, Gargiulo M, du Moncel ST, et al. Behavioural disorders, Parkinson's disease and subthalamic stimulation. J Neurol Neurosurg Psychiatry. 2002;72(6):701-7
60. Broen M, Duits A, Visser-Vandewalle V, Temel Y, Winogrodzka A. Impulse control and related disorders in Parkinson's disease patients treated with bilateral subthalamic nucleus stimulation: a review. Parkinsonism Relat Disord. 2011;17(6):413-7
61. Lim SY, O'Sullivan SS, Kotschet K, Gallagher DA, Lacey C, Lawrence AD, et al. Dopamine dysregulation syndrome, impulse control disorders and punding after deep brain stimulation surgery for Parkinson's disease. J Clin Neurosci. 2009;16(9):1148-52
62. Moum SJ, Price CC, Limotai N, Oyama G, Ward H, Jacobson C, et al. Effects of STN and GPi deep brain stimulation on impulse control disorders and dopamine dysregulation syndrome. PLoS One. 2012;7(1):e29768
63. Okun MS, Weintraub D. Should impulse control disorders and dopamine dysregulation syndrome be indications for deep brain stimulation and intestinal levodopa? Mov Disord. 2013;28(14):1915-9
64. Abbes M, Lhommée E, Thobois S, Klinger H, Schmitt E, Bichon A, et al. Subthalamic stimulation and neuropsychiatric symptoms in Parkinson's disease: results from a long-term follow-up cohort study. J Neurol Neurosurg Psychiatry. 2018;89(8):836-43
65. Ardouin C, Voon V, Worbe Y, Abouazar N, Czernecki V, Hosseini H, et al. Pathological gambling in Parkinson's disease improves on chronic subthalamic nucleus stimulation. Mov Disord. 2006;21(11):1941-6
66. Bandini F, Primavera A, Pizzorno M, Cocito L. Using STN DBS and medication reduction as a strategy to treat pathological gambling in Parkinson's disease. Parkinsonism Relat Disord. 2007;13(6):369-71
67. Knobel D, Aybek S, Pollo C, Vingerhoets FJ, Berney A. Rapid resolution of dopamine dysregulation syndrome (DDS) after subthalamic DBS for Parkinson disease (PD): a case report. Cogn Behav Neurol. 2008;21(3):187-9

68. Santin MDN, Voulleminot P, Vrillon A, Hainque E, Béreau M, Lagha-Boukbiza O, et al. Impact of Subthalamic Deep Brain Stimulation on Impulse Control Disorders in Parkinson's Disease: A Prospective Study. Mov Disord. 2021;36(3):750-7
69. Thobois S, Ardouin C, Lhommée E, Klinger H, Lagrange C, Xie J, et al. Non-motor dopamine withdrawal syndrome after surgery for Parkinson's disease: predictors and underlying mesolimbic denervation. Brain. 2010;133(Pt 4):1111-27

3.15 Psychose

Autoren: Johannes Levin, Alkomiet Hasan

Fragestellung 144: Welche psychotischen Störungen können bei der PK auftreten (z.B. Albträume, Verkennungen, Pseudohalluzinationen, Halluzinationen)?

Hintergrund

Die Art der psychotischen Symptome bei der PK kann unterschiedlich sein.

Evidenzgrundlage

Es wurden keine kontrollierten Studien identifiziert, in denen das Spektrum der psychotischen Störungen bei der PK untersucht wurde. Vier Übersichtsarbeiten und zwei Fallstudien wurden bei der Literaturrecherche als relevant bewertet [1], [2], [3], [4], [5], [6], [7].

Ergebnis

Die Prodromalphase umfasst in den meisten Fällen lebhafte Träume und Albträume. Auf diese Phase können die Entwicklung von Illusionen (z. B. Verkennung von Objekten als Personen) und ein falsches Gefühl der Anwesenheit von Objekten oder Lebewesen folgen. Danach entwickeln die Pat. typischerweise Pseudohalluzinationen mit erhaltener Einsicht. So kann ein Patient/eine Patientin beispielsweise ein Haustier der Familie sehen, das Jahre zuvor gestorben ist, oder Käfer, die an den Wänden krabbeln. Der Inhalt dieser vorwiegend visuellen Pseudohalluzinationen ist in der Regel wiederkehrend. Schließlich können die Pat. Halluzinationen ohne Einsicht und/oder Wahnvorstellungen entwickeln.

Empfehlung mit Begründung der Empfehlung

Die Evidenz beruht auf mehreren Übersichtsarbeiten und Fallstudien. Das Spektrum der psychotischen Störungen umfasst lebhafte Träume, Albträume, illusionäre Verkennungen, Pseudohalluzinationen bis hin zu Halluzinationen mit Verlust der Einsicht und Entwicklung von Wahnvorstellungen, z.B. Eifersuchtswahn. Halluzinationen sind in der Regel visuell. Auf dieses Spektrum sollte bei der Anamnese und Untersuchung besonders geachtet werden, da diese oft nicht spontan berichtet werden.

Empfehlung	Neu<br><br>Stand (2023)
Psychotische Symptome und Symptome der Prodromalphase wie lebhafte Träume und Albträume, Verkennung von Objekten als Personen, ein falsches Gefühl der Anwesenheit von Objekten oder Lebewesen, Pseudohalluzinationen mit erhaltener Einsicht (häufig z.B. initial Insekten oder Haustiere), Halluzinationen und/oder Wahnvorstellungen sollen bei Menschen mit PK im Verlauf der Erkrankung regelmäßig abgefragt werden. Angehörige sollen mit einbezogen werden.	
Konsensstärke: 100%, starker Konsens	

Fragestellung 145: Welche Risikofaktoren existieren für psychotische Störungen bei der PK?

Hintergrund

Wissen um Risikofaktoren psychotischer Störungen bei der PK sollte bei Prophylaxe und Therapie Berücksichtigung finden.

Evidenzgrundlage

Es wurden keine kontrollierten Studien identifiziert, in denen Risikofaktoren der psychotischen Störungen bei der PK prospektiv untersucht wurden. Sechs Übersichtsarbeiten und drei Fallstudien wurden bei der Literaturrecherche als relevant bewertet [1], [2], [3], [5], [8], [9], [6], [7], [10].

Ergebnis

Leichte kognitive Beeinträchtigungen oder eine demenzielle Entwicklung, Depressionen, hohes Lebensalter, Dauer und Schweregrad der PK sowie erhebliche psychiatrische (v.a. aktive oder frühere Suchterkrankung) oder medizinische Komorbiditäten (z.B. ausgeprägte Mikroangiopathie) sind Risikofaktoren für die Entwicklung einer Psychose bei PK. Weitere wichtige und modifizierbare Risikofaktoren sind sensorische Deprivation aufgrund verminderter Sehkraft oder verminderten Hörvermögens. Zusätzlich gibt es bedeutende pharmakologische Risiken wie komplexes Medikamentenregime (Differenzialdiagnose zum Delir). Visuelle Halluzinationen treten am häufigsten bei schlechten Lichtverhältnissen (z.B. schlecht beleuchtete Stationen, fehlende Kontraste in der Raumgestaltung) auf.

Empfehlung mit Begründung der Empfehlung

Die Evidenz beruht auf mehreren Übersichtsarbeiten und zwei Fallstudien. Das Spektrum der Risikofaktoren ist breit. Auf dieses Spektrum sollte bei der Anamnese und Untersuchung besonders geachtet werden, da sich hier Behandlungsansätze ergeben.

Empfehlung	Neu<br><br>Stand (2023)
Risikofaktoren für die Entwicklung psychotischer Symptome wie kognitive Störungen, Depressionen, hohes Lebensalter, Dauer und Schweregrad der PK, psychiatrische oder medizinische Komorbiditäten, sensorische Deprivation (Sehen oder Hören) oder Polypharmazie sollen im Rahmen der Anamneseerhebung erhoben und eingeordnet werden.	
Konsensstärke: 100%, starker Konsens	

Fragestellung 146: Welche diagnostischen Instrumente existieren für psychotische Störungen bei der PK?

Hintergrund

Es gibt kein validiertes diagnostisches Instrument zur Diagnose von psychotischen Störungen bei der PK.

Evidenzgrundlage

Es wurde eine kontrollierte Studie identifiziert, in der ein diagnostisches Instrument zur Diagnose von psychotischen Störungen bei PK prospektiv untersucht wurde [11]. Sechs Übersichtsarbeiten wurden bei der Literaturrecherche als relevant bewertet [1], [2], [3], [5], [8], [9].

Ergebnis

Im Jahr 2022 entwickelten Greger und Kollegen ein Instrument zur gezielten Erfassung von Symptomen der psychotischen Störungen bei PK [11]. Im Jahr 2007 entwickelte eine Arbeitsgruppe des NINDS-NIMH eine Reihe von Diagnosekriterien für psychotische Störungen bei PK. Sie beruhen auf der Diagnose einer PK auf der Grundlage der Kriterien der UK Brain Bank für PK, mindestens einem charakteristischen Symptom der Psychose, einschließlich Illusionen, falscher Präsenzwahrnehmung, Halluzinationen oder Wahnvorstellungen, und Auftreten der Symptome nach der Diagnose der PK [12]. Es gibt diverse weitere Skalen wie zum Beispiel das Neuropsychiatric Inventory (NPI) für Pat. im fortgeschrittenen Stadium, das Schedule for Assessment of Positive Symptoms (SAPS), die Positive and Negative Syndrome Scale (PANSS) oder die Brief Psychiatric Rating Scale (BPRS). Im Jahr 2008 untersuchte die Movement Disorder Society mehrere Skalen zur Diagnose von psychotischen Störungen bei PK und kam zu dem Schluss, dass keine einzige Skala geeignet ist. Sie empfahl, je nach Pat. eine Kombination aus verschiedenen Skalen zu verwenden [13].

Empfehlung mit Begründung der Empfehlung

Die Evidenz beruht auf einer prospektiven Studie mehreren Übersichtsarbeiten. Es gibt diverse diagnostische Instrumente (s.o.). Die Autoren dieses Leitlinienkapitels sind der Meinung, dass ein Instrument zur Diagnose von psychotischen Störungen insbesondere bei diagnostischer Unsicherheit bei PK eingesetzt werden sollte, und schließen sich der Empfehlung der Movement Disorder Society an, dass die Auswahl des Instruments fallbasiert erfolgen sollte. Einschränkend sollte beachtet werden, dass der Umgang mit den Skalen trainiert werden muss.

**Empfehlung**	**Neu**<br>**Stand (2023)**
Die Diagnosekriterien der NINDS-NIMH sollten in die Diagnostik von psychotischen Symptomen bei Menschen mit einer PK in Ergänzung zur klinischen Evaluation verwendet werden.	
Konsensstärke: 100%, starker Konsens	

Fragestellung 147: Wie werden psychotische Symptome bei der PK diagnostiziert?

Hintergrund

Es gibt keine Standardmethode zur Diagnose von psychotischen Symptomen bei der PK.

Evidenzgrundlage

Es wurden keine kontrollierten Studien identifiziert, in denen ein diagnostisches Instrument zur Diagnose von psychotischen Störungen bei PK prospektiv untersucht wurde. Sechs Übersichtsarbeiten wurden bei der Literaturrecherche als relevant bewertet [1], [2], [3], [5], [8], [9].

Ergebnis

Bevor eine durch Parkinson-Medikamente provozierte Psychose als Ursache eines psychotischen Syndroms diagnostiziert wird, müssen andere Ursachen ausgeschlossen werden. Zu den wichtigen Differenzialdiagnosen, die in Betracht gezogen werden müssen, gehören unter anderem andere neurodegenerative Erkrankungen, Psychosen im Zusammenhang mit den zahlreichen Ursachen von Delirien (siehe Kapitel Delir), schwere depressive Störungen, Nebenwirkungen dopaminerger Medikamente (v.a. Dopaminagonisten, COMT- Hemmer), Nebenwirkungen anderer Medikamente (z.B. Opiate, anticholinerge Medikamente), Hyponatriämien, Substanzmissbrauch oder Substanzintoxikation. Es gibt keine Standardmethode zur Diagnose von psychotischen Störungen bei PK. Verschiedene Skalen können die Diagnosestellung erleichtern (siehe Fragestellung lfd. Nr. 145).

Empfehlung mit Begründung der Empfehlung

Die Evidenz beruht auf mehreren Übersichtsarbeiten. Es gibt diverse diagnostische Instrumente (s.o., Fragestellung lfd. Nr. 146). Es sollte ein Ausschluss von Differenzialdiagnosen stattfinden und der Empfehlung in Fragestellung lfd. Nr. 146 sollte gefolgt werden.

**Empfehlung**	**Neu**<br><br>**Stand (2023)**
Psychotische Symptome bei Menschen mit einer PK sollten primär klinisch unter Würdigung der Risikofaktoren und anamnestischer Angaben diagnostiziert werden. Verschiedene Skalen (siehe Hintergrundtext) können bei diagnostischer Unsicherheit angewendet werden.	
Konsensstärke: 96,8%, starker Konsens	

Fragestellung 148: Führen präventive Maßnahmen bei Menschen mit einer PK zu einer Reduktion des Risikos des Auftretens psychotischen Erlebens?

Hintergrund

Aufgrund von Umweltfaktoren, die das Risiko einer Psychose bei der PK erhöhen, stellt sich die Frage, ob präventive Maßnahmen das Risiko einer Manifestation senken können.

Evidenzgrundlage

Es wurden keine kontrollierten Studien identifiziert, in denen präventive Maßnahmen bei Menschen mit einer PK mit dem Ziel einer Reduktion des Risikos des Auftretens psychotischen Erlebens bei PK prospektiv untersucht wurden. Fünf Übersichtsarbeiten wurden bei der Literaturrecherche als relevant bewertet [1], [2], [3], [5], [14].

Ergebnis

Bei der Behandlung aller Parkinson-Pat. sollten allgemeine Maßnahmen zur Reduktion des Psychoserisikos angewendet werden. Zu diesen Maßnahmen gehören die Einhaltung des zirkadianen Rhythmus, Optimierung normaler Sinneseindrücke (z.B. Verwendung von Hör- und Sehhilfen, ausreichendes Licht) oder die Aufrechterhaltung eines familiären Umfelds. Auch spezifische Komorbiditäten wie Infektionen, Dehydrierung etc. sollten behandelt werden. Auf nicht unbedingt notwendige Medikamente, insbesondere auf anticholinerge, antiglutamaterge oder sedierende Medikamente, sollte verzichtet werden. Eine Polypharmazie sollte im Sinne des Deprescribings reduziert werden.

Empfehlung mit Begründung der Empfehlung

Die Evidenz beruht auf mehreren Übersichtsarbeiten. Den unter „Ergebnis" gelisteten Empfehlungen zu a) allgemeinen Maßnahmen, b) Komorbiditäten, c) allgemeinen Medikamenten sollte gefolgt werden.

**Empfehlung**	**Neu**<br><br>**Stand (2023)**
Präventive Maßnahmen wie Einhaltung des zirkadianen Rhythmus, Optimierung normaler Sinneseindrücke, Aufrechterhaltung eines familiären Umfelds, Behandlung spezifischer Komorbiditäten (Infektionen, Dehydrierung etc.) und Verzicht auf nicht unbedingt notwendige Medikamente (insbesondere anticholinerge, antiglutamaterge oder sedierende Medikamente) sollten bei Pat. mit Parkinson angewendet werden.	
Konsensstärke: 100%, starker Konsens	

Fragestellung 149: Führt eine Veränderung der Parkinson-Therapie bei Menschen mit einer PK und Psychose zu einer Reduktion des psychotischen Erlebens?

Hintergrund

Da Anti-Parkinson-Medikamente bei Menschen mit einer PK eine Psychose auslösen können, ist es naheliegend, dass eine Veränderung der Parkinson-Therapie zu einer Reduktion des psychotischen Erlebens führen kann.

Evidenzgrundlage

Es wurden keine kontrollierten Studien identifiziert, in denen eine Veränderung der Parkinson-Therapie bei Menschen mit einer PK und psychotischem Erleben in Bezug auf die Effektivität der Reduktion von psychotischem Erleben bei PK prospektiv untersucht wurden. Fünf Übersichtsarbeiten wurden bei der Literaturrecherche als relevant bewertet [1], [2], [3], [5], [14].

Ergebnis

Die Anti-Parkinson-Medikamente können so weit reduziert werden, wie es im Hinblick auf die Bewegungsstörung toleriert wird. Das Regime sollte auf eine vereinfachte Strategie umgestellt

werden, bei der zunächst Anticholinergika vermieden und dann Amantadin, MAO-B-Hemmer oder Dopaminagonisten in dieser Reihenfolge reduziert werden. Dann kann es außerdem hilfreich sein, COMT-Hemmer und Retardpräparate der dopaminergen Medikamente zu vermeiden.

Empfehlung mit Begründung der Empfehlung

Die Evidenz beruht auf mehreren Übersichtsarbeiten. Den unter „Ergebnis" gelisteten Empfehlungen zur Veränderung der Parkinson-Therapie bei Menschen mit einer PK und Psychose sollte gefolgt werden.

**Empfehlung**	**Neu**<br><br>**Stand (2023)**
Im Falle von psychotischen Symptomen bei Menschen mit einer PK soll die Parkinson-Medikation angepasst werden:<br>1. Reduktion der Dosierung<br>2. Vereinfachung der Kombinationen<br>3. Die Reduktion/das Absetzen sollte in folgender Reihenfolge erfolgen: Anticholinergika > Amantadin > MAO-B-Hemmer > Dopaminagonisten > COMT-Hemmer.	
Konsensstärke: 100%, starker Konsens	

Fragestellung 150: Führt eine Behandlung mit Acetylcholinesterase-Hemmern bei Menschen mit einer PK und Psychose zu einer Reduktion des psychotischen Erlebens?

Hintergrund

Acetylcholinesterase-Hemmer können die Symptome kognitiver Defizite lindern. Da kognitive Defizite ein Risikofaktor für die Entwicklung einer Psychose bei PK sind, ist es plausibel, dass eine Behandlung mit Acetylcholinesterase-Hemmern zu einer Reduktion des psychotischen Erlebens führen könnte.

Evidenzgrundlage

Es wurden keine abgeschlossenen kontrollierten Studien identifiziert, in denen die Behandlung mit Acetylcholinesterase-Hemmern bei Menschen mit einer PK und psychotischem Erleben in Bezug auf die Effektivität der Reduktion von psychotischem Erleben bei PK prospektiv untersucht wurde. Eine vorzeitig beendete Studie liegt vor [15]. Fünf Übersichtsarbeiten wurden bei der Literaturrecherche als relevant bewertet [1], [2], [3], [5], [14].

Ergebnis

Die begrenzten Daten einer abgebrochenen Studie zur Untersuchung, ob die Behandlung mit einem Acetylcholinesterase-Hemmer bei Parkinson-Pat. mit leichten visuellen Halluzinationen den Progress zu einer Psychose verzögert, sprechen für eine abwartende Haltung anstelle einer frühen Behandlung mit Rivastigmin. In diversen Übersichtsarbeiten werden Acetylcholinesterase-Hemmer bei ausgeprägten visuellen Halluzinationen empfohlen.

Empfehlung mit Begründung der Empfehlung

Die Evidenz beruht auf einer abgebrochenen, nicht konklusiven Studie (unklarer Evidenzgrad) und mehreren Übersichtsarbeiten. Als Ergebnis kann bei Menschen mit einer PK und Psychose bei Versagen anderer Maßnahmen ein Therapieversuch mit Acetylcholinesterase-Hemmern erfolgen. Es handelt sich hierbei um eine Off-label-Anwendung. Besonders zu würdigen sind Interaktionen auf der CYP-Ebene sowie kardiale und gastrointestinale Nebenwirkungen.

Empfehlung	Modifiziert<br>Stand (2023)
Acetylcholinesterase-Hemmer können bei Menschen mit PK und psychotischen Symptomen als Therapieoption im Fall des Versagens anderer nicht pharmakologischer und pharmakologischer Maßnahmen zur Behandlung angeboten werden.	
Konsensstärke: 100%, starker Konsens	

Fragestellung 151: Führt eine Behandlung mit antipsychotischen Substanzen bei Menschen mit einer PK und Psychose zu einer Reduktion des psychotischen Erlebens?

Hintergrund

Da antipsychotische Substanzen auch bei Psychosen anderen Ursprungs zu einer Reduktion des psychotischen Erlebens führen, ist es naheliegend, dass sie auch bei Behandlung von Menschen mit einer PK und Psychose zu einer Reduktion des psychotischen Erlebens führen. Allerdings ist der Wirkmechanismus der meisten antipsychotischen Substanzen v.a. durch eine D2R-Blockade begründet, sodass eine Anwendung bei der PK nicht möglich ist.

Evidenzgrundlage

Die Literatursuche hat verschiedene Evidenzquellen (RCTs, Systematic Reviews, Meta-Analysen) identifiziert. Nach Ende der Recherche wurde jedoch eine umfassende Metaanalyse veröffentlicht [16]. Diese Metaanalyse wurde als Evidenzgrundlage für die Empfehlung verwendet. Die Metaanalyse ist methodisch hochwertig und hat einen Evidenzgrad von 1. Diese Netzwerk-Metaanalyse inkludierte 19 Studien mit 1.242 Teilnehmenden mit einer Psychose bei PK und untersuchte Pimavanserin, Quetiapin, Olanzapin, Clozapin, Ziprasidon, und Risperidon. In dieser Metaanalyse zeigten nur Clozapin und Pimavanserin eine ausreichende Suppression der psychotischen Symptome ohne Verschlechterung der Motorik. Quetiapin verschlechterte die Kognition.

Ergebnis

Schwerwiegende psychotische Symptome, die im Zusammenhang mit einer PK stehen, erfordern eine spezifische antipsychotische Pharmakotherapie, wenn bei der Prüfung von Differenzialdiagnosen keine bessere Erklärung gefunden wurde, wenn eine Anpassung der Anti-Parkinson-Medikamente nicht zu einer Besserung des psychotischen Erlebens führte und wenn eine nicht pharmakologische Behandlung die vorhandenen Symptome nicht beseitigen oder kontrollieren

konnte. Bezüglich der für die Behandlung von psychotischen Störungen zugelassenen Präparate haben nur Clozapin, Quetiapin und Olanzapin keinen relevanten Einfluss auf die Motorik. Olanzapin soll aufgrund der anticholinergen Eigenschaften nicht bei der PK eingesetzt werden. Basierend auf der hochwertigen Metaanalyse, soll Clozapin zur Behandlung der Psychose bei PK angeboten werden. Clozapin hat seine Wirksamkeit bei der Behandlung von Psychose bei PK in Dosierungen von 6,25 bis 50 mg pro Tag gezeigt. Clozapin ist jedoch aufgrund des Risikos einer Agranulozytose und Myokarditis und der daraus resultierenden Notwendigkeit einer regelmäßigen Überwachung sowie anderer Nebenwirkungen wie u.a. Sedierung, Speichelfluss (Aspirationsrisiko, erhöhtes Pneumonierisiko), Obstipation und Sturzgefahr [17] nicht immer die Behandlung der ersten Wahl. Stattdessen wird in der klinischen Praxis häufig Quetiapin eingesetzt, da keine Überwachung erforderlich ist und es ein geringeres Risiko für extrapyramidale Nebenwirkungen birgt. Allerdings hat sich in der Metaanalyse keine Wirksamkeit von Quetiapin in einer Dosierung von 25 bis 150 mg pro Tag bei der Behandlung von Psychose bei PK gezeigt. Auch zeigten sich dyskognitive Effekte.

Das Dilemma der Abwägung von Wirksamkeit und Sicherheit bei der Behandlung von Psychose bei PK mit Clozapin oder Quetiapin könnte sich nun mit der Zulassung von Pimavanserin durch die Food and Drug Administration (FDA), einem neuartigen selektiven inversen 5-HT2A-Agonisten, der sich bei der Behandlung von Psychose bei PK als wirksam erwiesen hat, ändern [16]. Durch den fehlenden dopaminergen Antagonismus zeigte sich in der Metaanalyse keine Verschlechterung der motorischen oder kognitiven Symptome bei gleichzeitiger Wirksamkeit auf die psychotischen Symptome [16]. Wie alle antipsychotisch wirksamen Substanzen birgt es das Risiko einer QTc-Verlängerung. Die FDA kam jedoch 2018 zu dem Schluss, dass der Nutzen von Pimavanserin seine potenziellen Risiken überwiegt [18]. Pimavanserin ist aktuell nicht in Europa zugelassen. Limitierend für den Einsatz sind weiterhin die hohen Tagestherapiekosten.

Es ist eine Metaanalyse, basierend auf 14 Studien, verfügbar (1 RCT, 9 prospektive Beobachtungsstudien, 4 retrospektive Studien), die zeigte, dass die Elektrokonvulsionsbehandlung bei Menschen mit PK sowohl psychotische als auch andere nicht motorische und motorische Symptome verbessert [19].

Benzodiazepine mit kurzer Halbwertszeit wären aus klinisch-pragmatischen Überlegungen möglich, aber hier liegen keine entsprechenden randomisierten Studien vor und insbesondere die erhöhte Sturzgefahr und eine mögliche paradoxe Reaktion schränken den Einsatz ein (Expertenmeinung).

**Empfehlung**	**Modifiziert**<br>**Stand (2023)**
**Clozapin** soll bei Menschen mit PK und psychotischen Symptomen bei Versagen anderer Strategien (Anpassung Anti-Parkinson-Medikation; nicht pharmakologische Maßnahmen) für die Behandlung angeboten werden, wobei über die mögliche Nebenwirkung der Agranulozytose und der Myokarditis aufgeklärt werden muss und entsprechende Kontrollen erfolgen müssen.	
Konsensstärke: 100%, starker Konsens	

Empfehlung	Modifiziert<br>Stand (2023)
**Quetiapin** kann zur Behandlung der Psychose bei Parkinson-Pat. ohne vorbestehende kognitive Einschränkung genutzt werden. Über die geringe Evidenzlage zur Wirksamkeit soll eine Aufklärung erfolgen.	
Konsensstärke: 92,9%, Konsens	

Empfehlung	Geprüft<br>Stand (2023)
Olanzapin und andere in dieser Leitlinie nicht explizit empfohlene Antipsychotika sollen bei Menschen mit PK und psychotischen Symptomen nicht angeboten werden.	
Konsensstärke: 100%, starker Konsens	

Empfehlung	Neu<br>Stand (2023)
Bei Parkinson-Pat. mit psychotischen Symptomen soll ein gestuftes Vorgehen erfolgen:<br>1. Durchführung von allgemeinen nicht pharmakologischen Maßnahmen (u.a. Reizabschirmung, reorientierende Maßnahmen, Reetablierung eines zirkadianen Rhythmus)<br>2. Durchführung allgemeiner therapeutischer Maßnahmen (u.a. Exsikkose-Behandlung, Behandlung eines Infekts etc.)<br>3. Reduktion/Anpassung auslösender Medikamente allgemein (anticholinerg, antiglutamaterg, sedierend) und von Anti-Parkinson-Medikation (v.a. Amantadin, MAO-B-Hemmer, Dopaminagonisten und COMT-Hemmer, Kombinationsbehandlungen)<br>4. Clozapin soll bei Versagen von 1.–3. nach entsprechender Risiko-Nutzen-Abwägung (Agranulozytose-Risiko, Myokarditis-Risiko, Sturzgefahr, anticholinerge Nebenwirkungen) angeboten werden. Alternativ kann Quetiapin off-label bei Menschen mit PK ohne kognitive Einschränkung angeboten werden.<br>5. Bei kognitiver Störung und Versagen von 1.–3. kann eine Umstellung auf einen Acetylcholinesterase-Hemmer angeboten werden.<br>6. Bei Versagen von 1.–4. und ggfs. 5. kann eine Elektrokonvulsionsbehandlung angeboten werden.	
Konsensstärke: 100%, starker Konsens	

Referenzen

1. Levin J, Hasan A, Höglinger GU. Psychosis in Parkinson's disease: identification, prevention and treatment. J Neural Transm (Vienna). 2016;123(1):45-50
2. Haussmann R, Bauer M, Donix M. [Evidence-based treatment of psychosis associated with Parkinson's disease]. Nervenarzt. 2016;87(5):543-51
3. Samudra N, Patel N, Womack KB, Khemani P, Chitnis S. Psychosis in Parkinson Disease: A Review of Etiology, Phenomenology, and Management. Drugs Aging. 2016;33(12):855-63
4. Ffytche DH, Creese B, Politis M, Chaudhuri KR, Weintraub D, Ballard C, Aarsland D. The psychosis spectrum in Parkinson disease. Nat Rev Neurol. 2017;13(2):81-95
5. Weiss D, Höglinger G, Klostermann F, Weise D, Zeuner KE, Reichmann H. [Hallucinations in Patients with Idiopathic Parkinson's Disease]. Fortschr Neurol Psychiatr. 2022;90(10):456-64
6. Naasan G, Shdo SM, Rodriguez EM, Spina S, Grinberg L, Lopez L, et al. Psychosis in neurodegenerative disease: differential patterns of hallucination and delusion symptoms. Brain. 2021;144(3):999-1012
7. Warren N, O'Gorman C, Hume Z, Kisely S, Siskind D. Delusions in Parkinson's Disease: A Systematic Review of Published Cases. Neuropsychol Rev. 2018;28(3):310-6
8. Zhong M, Gu R, Zhu S, Bai Y, Wu Z, Jiang X, et al. Prevalence and Risk Factors for Minor Hallucinations in Patients with Parkinson's Disease. Behav Neurol. 2021;2021:3469706
9. Omoto S, Murakami H, Shiraishi T, Bono K, Umehara T, Iguchi Y. Risk factors for minor hallucinations in Parkinson's disease. Acta Neurol Scand. 2021;143(5):538-44
10. Cravanas B, Jr., Frei K. The effects of Cannabis on hallucinations in Parkinson's disease patients. J Neurol Sci. 2020;419:117206
11. Greger J, Aladeen T, Rainka M, Kale A, Capote H. Evaluating rates of reporting symptoms of Parkinson's disease psychosis: provider versus targeted questionnaire. Int J Neurosci. 2022;132(5):459-65
12. Ravina B, Marder K, Fernandez HH, Friedman JH, McDonald W, Murphy D, et al. Diagnostic criteria for psychosis in Parkinson's disease: report of an NINDS, NIMH work group. Mov Disord. 2007;22(8):1061-8
13. Fernandez HH, Aarsland D, Fénelon G, Friedman JH, Marsh L, Tröster AI, et al. Scales to assess psychosis in Parkinson's disease: Critique and recommendations. Mov Disord. 2008;23(4):484-500
14. Powell A, Matar E, Lewis SJG. Treating hallucinations in Parkinson's disease. Expert Rev Neurother. 2022;22(6):455-68
15. van Mierlo TJM, Foncke EMJ, Post B, Schmand BA, Bloem BR, van Harten B, et al. Rivastigmine for minor visual hallucinations in Parkinson's disease: A randomized controlled trial with 24 months follow-up. Brain Behav. 2021;11(8):e2257
16. Yunusa I, Rashid N, Seyedin R, Paratane D, Rajagopalan K. Comparative Efficacy, Safety, and Acceptability of Pimavanserin and Other Atypical Antipsychotics for Parkinson's Disease Psychosis: Systematic Review and Network Meta-Analysis. J Geriatr Psychiatry Neurol. 2023;36(5):417-32
17. Wagner E, Siafis S, Fernando P, Falkai P, Honer WG, Röh A, et al. Efficacy and safety of clozapine in psychotic disorders-a systematic quantitative meta-review. Transl Psychiatry. 2021;11(1):487
18. Hawkins T, Berman BD. Pimavanserin: A novel therapeutic option for Parkinson disease psychosis. Neurol Clin Pract. 2017;7(2):157-62
19. Takamiya A, Seki M, Kudo S, Yoshizaki T, Nakahara J, Mimura M, Kishimoto T. Electroconvulsive Therapy for Parkinson's Disease: A Systematic Review and Meta-Analysis. Mov Disord. 2021;36(1):50-8

3.16 Delir

Autoren: Georg Ebersbach, Johannes Levin, Alkomiet Hasan

Die PK ist ein unabhängiger Risikofaktor für die Entwicklung eines Delirs. Die Entwicklung eines Delirs im Rahmen der PK ist eine relevante Komplikation, die mit einem hohen Risiko für lang anhaltende Verschlechterungen von Motorik, der vegetativen Funktionen, höheren Hirnfunktionen und Psychopathologie einhergeht. Auch erhöht das Delir die Mortalität der Erkrankung. Die wenigen hierzu vorliegenden Studien erlauben keine Aussage, ob das Delir im Rahmen einer PK durch einen spezifischen Phänotyp gekennzeichnet ist. Während eines Delirs können eine Zunahme der motorischen Symptome und ein reduziertes Ansprechen auf dopaminerge Medikation auftreten.

Die klinische Diagnose eines Delirs bei PK erfordert die Abgrenzung von PK-immanenten Symptomen und der deliranten Symptomatik. Dies umfasst zum einen motorische Symptome wie die Hypokinese und zum anderen die verschiedenen nicht motorischen Symptome, die in den unterschiedlichen Stadien der PK bestehen können. Neuropsychiatrische Symptome wie Halluzinationen und Wahnstörungen, aber auch Apathie, Bewegungsunruhe, Insomnie und schlafassoziierte Verhaltensstörungen stellen Überschneidungen mit den möglichen Symptomen eines Delirs dar. Verhaltensstörungen und Fluktuationen der Vigilanz treten in besonderem Maß bei der Demenz mit Lewy-Körperchen (DLB) auf. Zudem können Nebenwirkungen der Anti-Parkinson-Therapie zu Symptomüberschneidungen führen. Diese umfassen psychomotorische Unruhezustände, optische Halluzinationen, vegetative Symptome, Agitiertheit und Verwirrtheit.

Bisher liegen nur unzureichende Kenntnisse zu Prävalenz, Inzidenz, Verlauf und Prognose des Delirs bei PK vor. Ebenso fehlen klinische Studien, aus denen sich Empfehlungen zum evidenzbasierten Management des Delirs bei PK ableiten lassen.

Fragestellung 152: Welche Risikofaktoren existieren für ein Delir bei der PK?

Evidenzgrundlage

Es wurden keine kontrollierten Studien identifiziert, in denen spezifische Risikofaktoren für ein Delir bei PK untersucht wurden. Allgemeine Risikofaktoren für die Entwicklung eines Delirs [1] wurden in zwei Übersichtsarbeiten als auch für das Delir bei PK relevant bewertet [2], [3].

**Empfehlung**	**Neu**<br><br>**Stand (2023)**
Folgende Faktoren können für das Auftreten eines Delirs prädisponieren: höheres Lebensalter (meist >65 Jahre), vorbestehender Substanzgebrauch, Polypharmazie, Hörminderung, internistische Komorbiditäten und kognitive Störungen. Als Auslöser können Infekte, metabolische Störungen, psychische Stressoren, Medikamentennebenwirkungen und Schmerzen in Betracht kommen. Längere Behandlungen im Krankenhaus, insbesondere auf Intensivstationen, größere operative Eingriffe und liegende Zugänge wie Infusionsschläuche und Katheter, können das Risiko eines Delirs bei stationär behandelten Pat. erhöhen.	
Konsensstärke: 100%, starker Konsens	

Fragestellung 153: Welche diagnostischen Instrumente existieren für ein Delir bei PK?

Evidenzgrundlage

Es wurden keine Studien identifiziert, in denen diagnostische Instrumente für ein Delir bei PK untersucht wurden. Laut drei Übersichtsarbeiten ist die Verwendung von Skalen möglich, die nicht spezifisch für die Diagnose des Delirs bei PK entwickelt wurden [2], [3], [4].

Empfehlung	Neu<br>Stand (2023)
Zum Delir-Screening können die Nurse Delirium Screening Scale (Nu-Desc) [5] und die Delirium Observation Scale (DOS-S) [6] verwendet werden. Um die Diagnose eines Delirs zu stellen und zur Verlaufsbeurteilung kann die Confusion Assessment Method (CAM) [1] eingesetzt werden.	
Konsensstärke: 100%, starker Konsens	

Fragestellung 154: Wie wird ein Delir bei PK diagnostiziert?

Evidenzgrundlage

Es wurden keine kontrollierten Studien identifiziert, in denen spezifische Diagnosekriterien für ein Delir bei PK untersucht wurden. Es wurden drei Übersichtsarbeiten identifiziert, die sich mit der Diagnose des Delirs bei PK auseinandersetzen [2], [3], [4].

Ergebnis

Grundsätzlich gelten für die Diagnose eines Delirs die im ICD-10/11 bzw. DSM-V genannten Kriterien. Danach wird das Delir als akute und transiente Aufmerksamkeits- und Wahrnehmungsstörung mit begleitender Störung des Gedächtnisses, der Orientierung, der Sprache und der Auffassung beschrieben. Im ICD-11 und DSM-V wird das Delir zusätzlich durch den fluktuierenden Verlauf charakterisiert. Störungen des Schlaf-Wach-Rhythmus, Halluzinationen und Wahnvorstellungen sowie psychomotorische Unruhe sind weitere Symptome, die mit einem Delir assoziiert sein können. Das Delir präsentiert sich in drei klinischen Prägnanztypen: (I) der hyperaktiven, der (II) hypoaktiven Form und als (III) gemischtes Delir, das durch wechselndes Auftreten von hyper- und hypoaktiven Zuständen gekennzeichnet ist. Die gemischte Verlaufsform ist dabei am häufigsten.

Die Diagnose des Delirs bei PK wird durch Überlappung von Symptomen des Delirs mit solchen, die der PK zuzuordnen sind, erschwert (z.B. Apathie, Vigilanzfluktuationen, medikamentös induzierte Halluzinationen). Bildgebung, EEG und Laboruntersuchungen erfolgen zur Differenzialdiagnose und Identifikation auslösender Faktoren.

Empfehlung	Neu<br>Stand (2023)
Die Diagnose eines Delirs sollte bei der PK auf der Grundlage der im ICD-10/11 bzw. DSM-V aufgeführten Kriterien gestellt werden. Hierbei sollten mögliche Überlappungen mit anderen psychopathologischen Komplikationen der PK beachtet werden. Die Confusion Assessment Method (CAM, [1]) kann zur Diagnose eines Delirs bei PK eingesetzt werden. Differenzialdiagnosen und auslösende Faktoren für ein Delir sollten durch paraklinische Diagnostik identifiziert werden.	
Konsensstärke: 100%, starker Konsens	

Fragestellung 155: Führen präventive Maßnahmen bei PK zu einer Reduktion des Risikos des Auftretens eines Delirs?

Evidenzgrundlage

Es wurden keine kontrollierten Studien identifiziert, in denen präventive Maßnahmen zur Vermeidung eines Delirs bei PK untersucht wurden. Es wurden zwei Übersichtsarbeiten identifiziert, die sich mit der Prävention des Delirs bei PK auseinandersetzen [2], [3].

Ergebnis

Die Prävention umfasst die Einhaltung des Tag-Nacht-Rhythmus, die Benutzung von Hilfsmitteln, die frühzeitige Mobilisierung, die ausreichende Nahrungs- und Flüssigkeitszufuhr, eine ruhige Umgebung sowie, soweit möglich, die Vermeidung von Kathetern und Zugängen [7], [8]. Eine ausreichende Flüssigkeitszufuhr sowie die Behandlung von Schmerzen und Infektionen ist sowohl zur Vermeidung als auch zur Therapie eines Delirs relevant. Eine Schulung des Krankenhauspersonals zum Delir ist wünschenswert. Die Einbeziehung der Angehörigen spielt bei der Delir-Prävention eine entscheidende Rolle [1]. Sofern die Möglichkeit besteht, kann die Mitaufnahme einer Begleitperson das Risiko eines Hospitalisationsdelirs bei besonders gefährdeten Pat., z.B. solchen mit demenziellen oder psychotischen Symptomen, vermindern [2], [3].

Empfehlung	Neu<br>Stand (2023)
Zur Prävention eines Delirs bei PK können bei Delir-gefährdeten Personen folgende Maßnahmen eingesetzt werden:<br>1. frühzeitige Mobilisierung<br>2. Vermeidung von Kathetern und Zugängen<br>3. Einhaltung des Tag-Nacht-Rhythmus<br>4. sensorische Hilfsmittel (Hörgeräte, Sehhilfen)<br>5. Nahrungs- und Flüssigkeitszufuhr<br>6. ruhige Umgebung<br>7. Umgebung mit guter Beleuchtung und guten Farbkontrasten<br>8. Behandlung von Schmerzen und Infektionen<br>9. Anwesenheit/Mitaufnahme von Bezugspersonen	

10.	reorientierende Maßnahmen (z.B. bei jedem Kontakt Vorstellung mit Namen, Uhr im Sichtbereich)
Konsensstärke: 96,9%, starker Konsens	

Fragestellung 156: Führt eine Veränderung der Parkinson-Therapie bei PK zu einer Reduktion eines Delirs?

Evidenzgrundlage

Es wurden keine kontrollierten Studien identifiziert, in denen die Auswirkungen einer Veränderung der Parkinson-Therapie auf ein Delir bei PK untersucht wurden. Es wurden drei Übersichtsarbeiten identifiziert, die sich mit den Auswirkungen der Parkinson-Therapie auf das Delir bei PK auseinandersetzen [2], [3], [9].

Ergebnis

Beim Delir sollten Medikamente mit potenziell höherer delirogener Potenz abgesetzt werden [10]. Hierzu zählen Dopaminagonisten, Anticholinergika und Amantadin. Häufig resultiert dies in einer Levodopa-Monotherapie [2]. Da nach einem abrupten Absetzen dieser Substanzen Entzugssymptome (DWAS: dopoamine agonist withdrawal syndrome; MDES: malignes dopaminerges Entzugssyndrom) auftreten können, ist ein engmaschiges Monitoring erforderlich.

Empfehlung	Neu<br><br>Stand (2023)
Beim Delir bei PK sollten Amantadin, Anticholinergika und Dopaminagonisten reduziert oder (ausschleichend) abgesetzt werden.	
Konsensstärke: 93,1%, Konsens	

Fragestellung 157: Führen andere pharmakologische Therapien zu einer Reduktion des Delirs bei PK?

Evidenzgrundlage

Es wurden keine kontrollierten Studien identifiziert, in denen die Auswirkungen anderer pharmakologischer Therapien auf ein Delir bei PK untersucht wurden. Es wurden drei Übersichtsarbeiten identifiziert, die sich mit der pharmakologischen Therapie des Delirs bei PK auseinandersetzen [2], [3], [9].

Ergebnis

Medikamentöse Therapieoptionen umfassen die Behandlung von potenziell ein Delir verursachenden Faktoren, wie akuten Infektionen, Schmerzen oder metabolischen Störungen. Eine antipsychotische Behandlung des Delirs mit Quetiapin und Clozapin ist bisher beim PK noch unzureichend untersucht, während andere Antipsychotika aufgrund ihrer dopaminantagonistischen Wirkung zur Behandlung des Delirs beim PK kontraindiziert sind. Clozapin kann aufgrund seiner anticholinergen Eigenschaften

ein Delir verstärken. Quetiapin wird in anderer Indikation (z.B. Delir bei Demenz) eingesetzt, aber entsprechende Studien fehlen, ebenso zum Einsatz von Benzodiazepinen bei starker Angst und Unruhe. Die Gabe von Benzodiazepinen erhöht die Sturz- und Aspirationsgefahr und muss daher mit äußerster Zurückhaltung erfolgen. Auch sind paradoxe Effekte möglich.

**Empfehlung**	**Neu**<br>**Stand (2023)**
Bei Delir bei der PK sollten auslösende Faktoren (Schmerzen, Infektionen, metabolische Störungen) medikamentös behandelt werden. Quetiapin kann unter Kontrolle von Kognition, Kreislauf und Blasenentleerung zur Behandlung des Delirs eingesetzt werden, wobei beachtet werden muss, dass dies für die Indikation Delir im Allgemeinen untersucht worden ist [11]. Der Einsatz von Clozapin und Benzodiazepinen kann im Einzelfall unter besonderer Beachtung möglicher Nebenwirkungen, v.a. Sturzgefahr, erwogen werden. Generell kann aufgrund der anticholinergen Eigenschaften auch eine Verschlechterung des Delirs bei PK unter den hier genannten Antipsychotika auftreten.	
Konsensstärke: 100%, starker Konsens	

Fragestellung 158: Führen nicht pharmakologische Therapien bei PK zu einer Reduktion des Delirs?

Evidenzgrundlage

Es wurden keine kontrollierten Studien identifiziert, in denen nicht pharmakologische Maßnahmen zur Reduktion eines Delirs bei PK untersucht wurden. Es wurden drei Übersichtsarbeiten identifiziert, die sich mit nicht pharmakologische Maßnahmen zur Reduktion eines Delirs bei PK auseinandersetzen [2], [3], [9].

Ergebnis

Nicht medikamentöse Maßnahmen spielen sowohl zur Prophylaxe bei delirgefährdeten Pat. als auch bei einem manifesten Delir eine entscheidende Rolle (s. Antwort auf Frage 155). Eine Einbeziehung der Angehörigen unterstützt die Kommunikation und (Re-)Orientierung der Betroffenen [8]. Wenn möglich, ist eine stationäre Mitaufnahme des Lebenspartners/der Lebenspartnerin oder einer engen Bezugsperson hilfreich [3]. Angepasste Stationsabläufe mit direkter Bezugspflege und milieutherapeutische Maßnahmen zur Orientierung am Tag und in der Nacht wirken sich positiv auf Pat. mit Delir aus. Eine ausreichende Nahrungs- und Flüssigkeitszufuhr und die Versorgung mit Sehhilfen und Hörgeräten sind weitere wichtige Pfeiler der nicht medikamentösen Therapie [1].

Empfehlung	Neu<br>Stand (2023)
Zur nicht medikamentösen Therapie eines Delirs bei PK können folgende Maßnahmen eingesetzt werden:<br>1. frühzeitige Mobilisierung<br>2. Vermeidung von Kathetern und Zugängen<br>3. Einhaltung des Tag-Nacht-Rhythmus<br>4. sensorische Hilfsmittel (Hörgeräte, Sehhilfen)<br>5. Nahrungs- und Flüssigkeitszufuhr<br>6. ruhige Umgebung<br>7. Umgebung mit guter Beleuchtung und guten Farbkontrasten<br>8. Behandlung von Schmerzen und Infektionen<br>9. Anwesenheit Mitaufnahme von Bezugspersonen<br>10. Reorientierende Maßnahmen (z.B. bei jedem Kontakt Vorstellung mit Namen, Uhr im Sichtbereich)	
Konsensstärke: 97%, starker Konsens	

Referenzen

1. Inouye SK, Westendorp RG, Saczynski JS. Delirium in elderly people. Lancet. 2014;383(9920):911-22
2. Ebersbach G, Ip CW, Klebe S, Koschel J, Lorenzl S, Schrader C, et al. Management of delirium in Parkinson's disease. J Neural Transm (Vienna). 2019;126(7):905-12
3. Franke C, Ebersbach G. [Delirium in idiopathic Parkinson's disease]. Nervenarzt. 2020;91(2):107-13
4. Lawson RA, McDonald C, Burn DJ. Defining delirium in idiopathic Parkinson's disease: A systematic review. Parkinsonism Relat Disord. 2019;64:29-39
5. Gaudreau JD, Gagnon P, Harel F, Tremblay A, Roy MA. Fast, systematic, and continuous delirium assessment in hospitalized patients: the nursing delirium screening scale. J Pain Symptom Manage. 2005;29(4):368-75
6. Schuurmans MJ, Shortridge-Baggett LM, Duursma SA. The Delirium Observation Screening Scale: a screening instrument for delirium. Res Theory Nurs Pract. 2003;17(1):31-50
7. Inouye SK. Prevention of delirium in hospitalized older patients: risk factors and targeted intervention strategies. Ann Med. 2000;32(4):257-63
8. Young J, Murthy L, Westby M, Akunne A, O'Mahony R. Diagnosis, prevention, and management of delirium: summary of NICE guidance. Bmj. 2010;341:c3704
9. Vardy ER, Teodorczuk A, Yarnall AJ. Review of delirium in patients with Parkinson's disease. J Neurol. 2015;262(11):2401-10
10. Rudolph JL, Salow MJ, Angelini MC, McGlinchey RE. The anticholinergic risk scale and anticholinergic adverse effects in older persons. Arch Intern Med. 2008;168(5):508-13
11. Rivière J, van der Mast RC, Vandenberghe J, Van Den Eede F. Efficacy and Tolerability of Atypical Antipsychotics in the Treatment of Delirium: A Systematic Review of the Literature. Psychosomatics. 2019;60(1):18-26

3.17 Psychosoziale Beratung

Autorin: Regina Menzel

Fragestellung 159: Welche Bedeutung hat psychosoziale Beratung zur Bewältigung persönlicher, sozialer, beruflicher und wirtschaftlicher Belastungen?

Hintergrund

Die PK hat für Betroffene und deren Angehörige einschneidende Auswirkungen auf die Lebenssituation. Eine Studie mit Parkinson-Pat. zeigte, dass aus Sicht der Betroffenen insbesondere Informationen über den Umgang mit der Erkrankung, Veränderungen in Beziehungen und soziale Auswirkungen sowie Informationen über gesetzliche, finanzielle und bürokratische Auswirkungen des Lebens mit der PK fehlen [1].

Evidenzgrundlage

Expertenmeinung auf der Basis der zitierten Literatur.

Ergebnis

Belastungen im Zusammenhang mit einer PK können bei den Betroffenen, aber auch bei deren Angehörigen in folgenden Bereichen auftreten [2], [3], [4]:

- Anpassung an die veränderte Lebenssituation mit der Diagnose PK (Probleme bei der Krankheitsverarbeitung, Zukunftsängste, Probleme durch veränderte Rollen, Probleme bei der Entwicklung von Perspektiven, Suchtverhalten)
- Veränderungen in sozialen Beziehungen (Partnerschaftskonflikte, Konflikte im Umgang mit gegenseitigen Erwartungen, familiäre Belastungen, Überlastung der Angehörigen)
- finanzielle Belastungen (durch Zuzahlung zu medikamentöser und therapeutischer Behandlung, Fahrtkosten, finanzielle Einbußen durch Lohnersatzleistungen oder den Bezug von Erwerbsminderungsrenten)
- Funktions- und Leistungseinschränkungen (körperliche und psychische Einschränkungen, Pflegebedürftigkeit)
- Alltagsbewältigung und Wohnumfeld (Probleme bei der Bewältigung des Alltags und der Selbstversorgung, Gefährdung der Weiterführung des Haushalts, problematische Wohnsituation)
- Teilhabe am Arbeitsleben (Probleme am Arbeitsplatz, eingeschränkte berufliche Leistungsfähigkeit, Verlust der Erwerbsfähigkeit und des Arbeitsplatzes).

Soziale Arbeit ist ein fester Bestandteil des multidisziplinären Behandlungsteams. Personen mit PK und ihre Angehörigen werden bei persönlichen, sozialen, beruflichen und wirtschaftlichen Belastungen im Zusammenhang mit der Erkrankung beraten, begleitet und unterstützt.

Psychosoziale und sozialrechtliche Beratung beinhaltet u.a. Information zu Leistungsansprüchen, Hilfestellung bei der Antragstellung (z.B. Schwerbehindertenausweis), Einleitung von medizinischen und beruflichen Rehabilitationsmaßnahmen, Unterstützung bei der Organisation häuslicher und

stationärer Pflege, Information über Beratungsstellen und Selbsthilfegruppen sowie über Vorsorgevollmacht und rechtliche Betreuung [5].

Gegenseitige Unterstützungs- und Beratungsleistungen werden auch durch Selbsthilfeverbände erbracht.

Es wurden keine Studien zur Wirksamkeit spezifischer psychosozialer Beratungsangebote bei Parkinson-Pat. gefunden. Die Bedeutung psychosozialer Beratung wurde aber in internationalen Leitlinien [6] und anderen Leitlinien für Pat.-Gruppen mit ähnlichem sozialem und psychischem Belastungsspektrum festgestellt (siehe z.B. [7], [8], [9], [10]). Den besonderen Bedarf an psychosozialer Unterstützung bei neurologischen Erkrankungen beschreiben auch [11]. Pat. und Angehörige erleben die Unterstützung durch soziale Arbeit bei der Bewältigung der mit der Erkrankung zusammenhängenden Probleme als hilfreich [12].

Begründung der Empfehlung

Wenn im ärztlichen Gespräch soziale, berufliche und wirtschaftliche Belastungen durch die PK deutlich werden, soll auf zusätzliche psychosoziale und sozialrechtliche Beratungsmöglichkeiten hingewiesen werden. Psychosoziale Beratung für Parkinson-Pat. wird in Akutkrankenhäusern, Fachkliniken, Rehabilitationseinrichtungen, ambulanten Beratungsstellen, Pflegeeinrichtungen, Behörden und anderen Institutionen insbesondere durch Sozialarbeiter/Sozialarbeiterinnen, Sozialpädagogen/Sozialpädagoginnen und Psychologen/Psychologinnen jeweils mit eigenen Schwerpunkten geleistet.

Empfehlung	Modifiziert<br>Stand (2023)
Pat. mit PK und deren Angehörige sollten in allen Phasen der Erkrankung Zugang zu psychosozialer und sozialrechtlicher Beratung erhalten.	
Konsensstärke: 100%, starker Konsens	

Referenzen

1. Kleiner-Fisman G, Gryfe P, Naglie G. A Patient-Based Needs Assessment for Living Well with Parkinson Disease: Implementation via Nominal Group Technique. Parkinsons Dis. 2013;2013:974964
2. Ansen H. Soziale (Alten-)Arbeit im Krankenhaus. In: Aner K, Karl U, editors. Handbuch Soziale Arbeit und Alter. VS Verlag für Sozialwissenschaften; 2010. p. 139-47.
3. Menzel R. Case-Management im Krankenhaus — eine Aufgabe der Klinischen Sozialarbeit. In: Brinkmann V, editor. Case Management, Organisationsentwicklung und Change Management in Gesundheits- und Sozialunternehmen. Wiesbaden: Betriebswirtschaftlicher Verlag Dr. Th. Gabler; 2010. p. 259,76.
4. Möller B, Reiff J. Auswirkungen des Morbus Parkinson auf die Lebensqualität von Pat. und Angehörigen. In: Nebel A, Deuschl G, editors. Forum Logopädie: Dysarthrie und Dysphagie bei Morbus Parkinson: Thieme; 2017. p. 39-41.

5. Deutsche Vereinigung für Soziale Arbeit im Gesundheitswesen: Produkt- und Leistungsbeschreibung der Sozialen Arbeit im Gesundheitswesen. 2019, 4. Auflage.
6. NICE Guideline: Parkinson´s disease in adults 2017. p. 23.
7. DEGAM. S3 Leitlinie Schlaganfall, Version 3.3 2020 [157]. Available from: *https://register.awmf.org/de/leitlinien/detail/053-011*.
8. NICE Guideline Social work with adults experiencing complex needs [NG216]. 2022. p. 29, 66.
9. NICE Guideline: Multiple Sclerosis in adults: management aktualisiert 202313-09-2023
10. DKG. S3 Leitlinie Psychoonkologische Diagnostik, Beratung und Behandlung von erwachsenen Krebspatient*innen 2023
11. Wallesch CW, Schlote A. Psychosoziale Folgen neurologischer Erkrankungen. Neurologische Rehabilitation. 2007;13:61-5
12. Layer H, Mühlum A. Krankenhaussozialarbeit aus Sicht der Pat.. Evaluationsstudie im Auftrag der DVSK. In: Gödecker-Geenen N, Nau H, Weis I, editors. Der Patient im Krankenhaus und sein Bedarf an psychosozialer Beratung Eine empirische Bestandsaufnahme. Münster: LIT Verlag; 2005.

3.18 Dysarthrie und Dysphagie

Autoren/Autorinnen: Tobias Warnecke, Madeleine Gausepohl

Fragestellungen 160: Welche klinischen Manifestationen der Dysarthrie können bei der PK auftreten? Wie wird die Dysarthrie bei der PK diagnostiziert? Wie wird die Dysarthrie bei der PK behandelt?

Hintergrund

Etwa 70–80% aller Pat. mit der PK entwickeln im Krankheitsverlauf eine Sprechstörung (Dysarthrie). Mit Fortschreiten der PK nimmt deren Intensität zu [1], [2]. Nach einer kleinen retrospektiven Studie mit post mortem histopathologisch bestätigter Diagnose tritt eine Dysarthrie bei PK durchschnittlich nach sieben Krankheitsjahren auf [3]. Die hypokinetisch-rigide Dysarthrie von Parkinson-Pat. ist klinisch charakterisiert durch eine monotone Stimmlage mit reduzierter Lautstärke und vermindertem Tonumfang, Schwierigkeiten bei der Sprechinitiierung bis hin zu Sprechblockaden, verminderter Sprechatmung, wechselndem Sprechtempo, unpräziser Konsonantenbildung und einem behauchten oder rauen Stimmklang [4], [5].

Die Diagnostik der Parkinson-bedingten Dysarthrie sowie der Evaluation des Störungsmusters erfolgt üblicherweise durch eine detaillierte logopädische Untersuchung des Sprechens. Vor der ersten Untersuchung kann der Einsatz von spezifischen Fragebögen sinnvoll sein, wie z. B. dem Voice Handicap Index (VHI), der funktionelle, physische und emotionale Aspekte erfasst [6]. Bei der klinischen Dysarthrie-Untersuchung von Parkinson-Pat. kommt der Beurteilung des spontanen und nicht stimulierten Sprechens sowie der Beurteilung der Stimulierbarkeit der verschiedenen Sprachmerkmale mithilfe maximaler Leistungstests besondere Bedeutung zu. Die Spontansprache von Parkinson-Pat. kann anhand der Atmung, Phonation, Artikulation, nasalen Resonanz und Prosodie bewertet werden. Bei der Interpretation sollte bekannt sein, ob sich die Pat. während der Observation in einer On-Phase oder in einer Off-Phase befinden. Außerdem sollten kognitive Kommunikationsstörungen berücksichtigt werden [7]. Zur Diagnostik der Parkinson-bedingten Dysarthrie können standardisierte und evaluierte Testverfahren, wie z.B. die Frenchay-Dysarthrie-Untersuchung, sowie audiovisuelle Aufzeichnungen, z.B. Videoaufnahme der Spontansprache oder dB-Messgerät, herangezogen werden [8].

Medikamentöse Therapie und Tiefe Hirnstimulation spielen in der Therapie Parkinson-bedingter Sprechstörungen eine untergeordnete Rolle. Die logopädische Therapie wird allgemein als das effektivste Verfahren zur Behandlung der Unverständlichkeit des Sprechens bei Parkinson-Pat. angesehen [9]. Verschiedene logopädische Behandlungsprogramme, die in Einzel- oder Gruppentherapie angewendet werden können, konzentrieren sich auf spezielle Komponenten der Dysarthrie wie Atemübungen oder Stimmtraining [10].

Das Lee Silverman Voice Treatment (LSVT® LOUD) ist ein intensiviertes Behandlungsprogramm, das spezifisch für Parkinson-Pat. entwickelt wurde. Es fokussiert sich auf die amplitudenbasierte Steigerung der Sprechlautstärke mit unmittelbarer Übertragung in die tägliche Kommunikation. Die hohe Intensität des Programms (viermal wöchentlich über einen Zeitraum von vier Wochen je eine Stunde) führt bei Parkinson-Pat. dazu, dass sie ihre zu leise Stimme besser wahrnehmen, bestärkt sie

darin, dass ein lauteres Sprechen normal ist, und gewöhnt sie daran, die neue lautere Stimme im Alltag einzusetzen [11]. Ein weiteres und vergleichbares intensiviertes Behandlungsprogram für Parkinson-Pat. ist das sog. Pitch Limiting Voice Treatment (PLVT), mit dem möglicherweise sogar ein noch natürlicherer Stimmklang durch Reduktion der Tonhöhe erzielt werden kann [12]. Mittlerweile gibt es erste Daten, die zeigen, dass intensivierte Behandlungsprogramme wie LSVT® LOUD zukünftig auch über ein Tablet digital angeboten werden können [13].

In der Literatur kommen außerdem technischen Kommunikationshilfen, wie Pacing Board, Stimmverstärker, verzögertes auditives Feedback oder rechnergestützte, tragbare visuelle und akustische Feedbackgeräte sowie nicht invasive Hirnstimulationsverfahren wie die repetitive transkranielle Magnetstimulation (rTMS) zur Anwendung [10], [14].

Evidenzgrundlage und Ergebnisse

Ein Cochrane Review zum Vergleich verschiedener logopädischer Therapiemethoden zur Behandlung von Sprechstörungen bei der PK kommt zu dem Schluss, dass aufgrund zu geringer Pat.-Zahlen keine Aussage über die Effektivität eines logopädischen Verfahrens im Vergleich zu einem anderen logopädischen Verfahren getroffen werden kann. Ein größerer Teil der vorliegenden positiven Daten zur Wirksamkeit von logopädischer Sprechtherapie bei der PK betrifft aber die LSVT® LOUD-Therapie. Allerdings handelt es sich um kleine Pat.-Zahlen mit einem maximalen Nachbeobachtungszeitraum von 12 Wochen [10].

Ein weiterer Cochrane Review kommt zu dem Ergebnis, dass es nur eine unzureichende Evidenz für den Vergleich der Effektivität von logopädischer Therapie mit medikamentöser Therapie bei der PK gibt [1].

Eine randomisierte kontrollierte Studie zur Effektivität der repetitiven transkraniellen Magnetstimulation (rTMS) auf die Stimmfunktion von Parkinson-Pat. ergab, dass die 15-Hz-rTMS des dorsolateralen präfrontalen Kortex im Vergleich zur Scheinbehandlung zwar die Stimmung und die subjektive Einschätzung der Sprechfunktion, nicht aber objektive Parameter wie Grundfrequenz und Stimmintensität verbesserte. Eine positive Evidenz für die Wirksamkeit der rTMS liegt damit bislang nicht vor [14].

Eine Metaanalyse randomisierter kontrollierter Studien zur Effektivität von logopädischer Sprechtherapie auf die hypokinetische Dysarthrie von Parkinson-Pat. konnte sieben Studien für die quantitative Analyse berücksichtigen. Hier zeigten sich signifikant positive Effekte der logopädischen Sprechtherapie auf die Verständlichkeit, den Schalldruckpegel sowie die Halbton-Standardabweichung [9].

Begründung der Empfehlungen

Zusammenfassend gibt es für die Effektivität verschiedener Verfahren der logopädischen Sprechtherapie in der Behandlung der PK-bedingten Sprechstörungen positive Wirksamkeitshinweise aus einem systematischen Review und einer Metaanalyse. Der größte Teil der positiven Daten liegt für intensivierte Behandlungsprogramme und insbesondere die LSVT® LOUD-Therapie vor. Die positiven Hinweise für die Wirksamkeit anderer Therapiestrategien, wie Maßnahmen zur

Optimierung der Verständlichkeit des Sprechens und zur Reduzierung des zu hohen Sprechtempos, stammen aus einzelnen methodisch guten, aber nicht randomisierten klinischen Studien. Insgesamt unterstützt auch die klinische Erfahrung der Expertengruppe allgemein den Einsatz logopädischer Sprechtherapie bei Parkinson-Pat. mit Sprechstörungen.

**Empfehlung**	**Modifiziert**<br><br>**Stand (2023)**
▪ Parkinson-Pat. mit Sprechstörungen sollten eine detaillierte logopädische Untersuchung des Sprechens erhalten, um eine gezielte und individuell an das Störungsmuster angepasste Behandlung initiieren zu können.<br>▪ Pat. mit Parkinson-bedingten Sprechstörungen sollen eine logopädische Sprechtherapie erhalten.<br>▪ Die logopädische Therapie sollte eine Verbesserung von Stimmlautstärke und Tonumfang zum Ziel haben, hierzu sollten intensivierte Therapieprogramme wie LSVT® LOUD zur Anwendung kommen.<br>▪ Behandlungsstrategien zur Optimierung der Verständlichkeit des Sprechens und zur Reduzierung des zu hohen Sprechtempos können eingesetzt werden.<br>▪ Logopädische Therapie kann die Aufrechterhaltung eines adäquaten Maßes an Kommunikationsfähigkeit während des gesamten Krankheitsverlaufs sicherstellen, hierzu können auch technische Hilfsmittel eingesetzt werden.	
Konsensstärke: 97%, starker Konsens	

Fragestellungen 161: Welche klinischen Manifestationen der Dysphagie können bei der PK auftreten? Wie wird die Dysphagie bei der PK diagnostiziert? Wie wird die Dysphagie bei der PK behandelt?

Hintergrund

Mehr als 80% aller Parkinson-Pat. entwickeln im Krankheitsverlauf eine Schluckstörung (Dysphagie) [15]. Diese Schluckstörungen sind mit zahlreichen und z. T. schwerwiegenden klinischen Komplikationen assoziiert, insbesondere Beeinträchtigung der Lebensqualität, unzureichender Medikamentenwirkung, Malnutrition und Aspirationspneumonien. Die Dysphagie kann bei der PK alle Phasen des Schluckakts, also die orale, die pharyngeale und die ösophageale Phase betreffen und durch sehr unterschiedliche Störungsmuster charakterisiert sein [16]. Klinisch relevante pharyngeale Dysphagien können bereits früh im Krankheitsverlauf auftreten [17] und mit motorischen Komplikationen, wie z.B. dem Delayed-on-Phänomen (verzögerter Wirkeintritt einer Levodopa-Dosis), assoziiert sein [18], [19]. Dual-Task-Situationen können zu einer Verschlechterung von Schlucksicherheit oder Schluckeffektivität führen [20]. Außerdem kann oropharyngeales Freezing auftreten, das phänomenologisch durch Akinese, Festination oder Trembling in place während des Schluckens gekennzeichnet ist [21].

Als diagnostische Screening-Verfahren stehen Fragebögen, wie insbesondere der Fragebogen zur Beurteilung von Dysphagien bei Parkinson-Pat. (Swallowing Disturbance Questionnaire; SDQ) oder der Münchener Dysphagie-Test – Parkinson's Disease (MDT-PD), zur Verfügung [22], [23]. Für den MDT-PD existieren allerdings bislang widersprüchliche Studienergebnisse [24]. Als klinisches Screening-Instrument kann ein modifizierter Wassertest eingesetzt werden, der das maximale Schluckvolumen misst. Neben der ausführlichen klinischen Schluckuntersuchung können zur Schweregradbestimmung und detaillierten Störungsmusteranalyse außerdem apparative Verfahren, wie insbesondere die flexible endoskopische Evaluation des Schluckakts (FEES), ggf. mit endoskopischem Levodopa-Test, und die Videofluoroskopie des Schluckens (VFSS), eingesetzt werden. Außerdem kann die High-Resolution-Manometrie (HRM) ösophageale Motilitätsstörungen detektieren. Häufige therapierelevante Befunde sind hierbei in der oralen Phase repetitive Pumpbewegungen der Zunge, orale Residuen, Leaking (= vorschneller und unkontrollierter Bolusübertritt in den Pharynx) sowie fragmentiertes Abschlucken des Bolus, in der pharyngealen Phase Aspirationen (auch ohne Auslösung eines Hustenreflexes), pharyngeale Residuen sowie eine verringerte Schluckfrequenz und in der ösophagealen Phase eine hypo- bis amotile Ösophaguspassage sowie diffuse Ösophagusspasmen. FEES und VFSS können außerdem zur Therapiekontrolle eingesetzt werden [16], [25].

Neben einer Optimierung der dopaminergen Medikation wird die logopädische Schlucktherapie als effektives Verfahren zur Behandlung der Dysphagie angesehen. Hierzu steht potenziell eine Vielzahl verschiedener Methoden mit unterschiedlichen Effekten auf die Schluckfunktion zur Verfügung.

Evidenzgrundlage und Ergebnisse

In einem älteren systematischen Review, der Studien bis zum Mai 2008 berücksichtigt hat, wurde bereits geschlussfolgert, dass sich allgemein positive Effekte der logopädischen Schlucktherapie bei Parkinson-Pat. ableiten lassen [26]. Ein systematischer Review, der bis einschließlich 2013 publizierte Studien berücksichtigt, findet positive Wirksamkeitsnachweise für das EMST-Training und die video-assistierte logopädische Schlucktherapie (VAST) [27]. In einem aktuelleren systematischen Review zur rehabilitativen Therapie der Parkinson-bedingten Dysphagie, der Publikationen von 2008 bis 2018 einschloss, wurden 11 Studien mit insgesamt 402 Pat. eingehender analysiert. Darunter befanden sich vier randomisierte kontrollierte Studien. Verschiedene Techniken der logopädischen Schlucktherapie wurden als wirksam eingestuft mit Effekten auf Koordination, die Geschwindigkeit und das Volumen des Schluckens sowie auf die Lebensqualität und soziale Beziehungen [28]. In einem Review zu gastrointestinalen Symptomen bei der PK werden Levodopa, Apomorphin und Rotigotin als potenziell wirksame medikamentöse Therapieoptionen bei einzelnen Parkinson-Pat. beschrieben. Als nicht medikamentöse Therapieverfahren, die potenziell effektiv sind, werden angedickte Flüssigkeiten und das Kinn-zur-Brust-Manöver, LSVT®-LOUD, Vermeidung von Dual-Task-Situationen, EMST150-Training und die videoassistierte logopädische Schlucktherapie (VAST) identifiziert [24]. Zu vergleichbaren Ergebnissen kommt ein systematischer Review inkl. Konsensusempfehlungen einer internationalen Expertengruppe. Es werden die Optimierung der dopaminergen Medikation und verschiedene Verfahren der logopädischen Schlucktherapie, wie konventionelle logopädische Schlucktherapie, angedickte Flüssigkeiten, Haltungsänderungen und

EMST-Training als effektiv beurteilt. Hier wird allerdings von LSVT®-LOUD als primärer Behandlungsoption für die Parkinson-bedingte Dysphagie abgeraten [29].

Eine randomisierte kontrollierte Studie verglich bei dysphagischen Parkinson-Pat. ein vierwöchiges Krafttraining der exspiratorischen Muskulatur mit einem speziellen Ausatemtrainer (EMST150) und ein Scheintraining mit dem gleichen Gerät. Das Training der exspiratorischen Muskulatur führte zu einer signifikanten, leichtgradigen Reduktion des Aspirationsgrads sowie einer verbesserten Larynxelevation während des Schluckens. Hier wäre für den klinischen Alltag bedeutsam, ob sich durch dieses Training auch die Lebensqualität der Parkinson-Pat. bessert oder die Pneumonierate reduziert wird. Es sind nur Pat. mit leicht- bis mittelgradig gestörter Schluckfunktion eingeschlossen worden, sodass sich die Ergebnisse nicht auf alle Parkinson-Pat. übertragen lassen [30]. In einer zweiten randomisierten kontrollierten Studie zur EMST150-Therapie kam es unter dem gleichen Trainingsprotokoll nach vier Wochen zu einer klinisch relevanten Verbesserung der Schluckeffektivität (signifikante Reduktion pharyngealer Residuen), die auch noch nach drei Monaten vorhanden war. Auch in diese Studie sind nur Pat. mit leicht- bis mittelgradig gestörter Schluckfunktion eingeschlossen worden [31].

Eine randomisierte kontrollierte Studie verglich eine video-assistierte logopädische Schlucktherapie (VAST) mit einer konventionellen logopädischen Schlucktherapie. In beiden Gruppen fand sich unmittelbar nach Therapieende eine Reduktion von Nahrungsresiduen im Pharynx, diese fiel in der VAST-Gruppe signifikant größer aus. Auch in diese Studie wurden nur Pat. mit leicht- bis mittelgradig gestörter Schluckfunktion eingeschlossen, sodass sich die Ergebnisse ebenfalls nicht auf alle Parkinson-Pat. übertragen lassen. Zudem gab es in dieser Studie keine Placebo-Kontrollgruppe [32].

Ein systematischer Review zum Effekt der Tiefen Hirnstimulation auf die Schluckfunktion von Parkinson-Pat., der Publikationen bis 2012 einschloss, kam zu dem Ergebnis, dass in keiner der eingeschlossenen Studien eine klinisch relevante Verbesserung oder Verschlechterung des Schluckens unter beidseitiger Tiefer Hirnstimulation des Nucleus subthalamicus nachgewiesen werden konnte. Subklinische positive Effekte fanden sich aber insbesondere für die pharyngeale Phase des Schluckaktes. Studien zum Vergleich der Wirkung einer Stimulation des Nucleus subthalamicus mit einer Stimulation des Globus pallidus internus auf die Schluckfunktion von Parkinson-Pat. gab es nicht. Randomisierte kontrollierte Studien waren nicht verfügbar [33]. Auch ein neuer, narrativer Review, der Publikationen bis zum Januar 2020 berücksichtigen konnte, kommt zu dem Ergebnis, dass weiterhin aussagekräftige randomisierte kontrollierte Studien zu diesem Thema fehlen [34].

Begründung der Empfehlungen

Zusammenfassend gibt es für Effektivität verschiedener Verfahren der logopädischen Schlucktherapie in der Behandlung der Parkinson-bedingten Dysphagie konsistent positive Wirksamkeitshinweise aus mehreren systematischen Reviews. Für das EMST-Training liegen mittlerweile positive Wirksamkeitsnachweise aus zwei randomisierten kontrollierten Studien vor. Die positiven Hinweise für die Wirksamkeit bestimmter medikamentöser Therapiestrategien, wie insbesondere für Levodopa sowie Apomorphin, stammen aus einigen methodisch guten, aber nicht randomisierten klinischen Studien. Die Erfahrungen der Expertengruppe sowie anderer

Autoren/Autorinnen unterstützen aber zusätzlich den Einsatz von logopädischer Schlucktherapie sowie die Optimierung der dopaminergen Medikation bzw. im Fall einer Tiefen Hirnstimulation ggf. eine Optimierung der Stimulationsparameter als wesentliche Maßnahmen in der Behandlung von Dysphagien bei der PK. Die in der logopädischen Therapie einzusetzenden Methoden sind dabei vom individuellen Störungsmuster des jeweiligen Pat. abhängig. Die Wirksamkeit der dopaminergen Medikation muss im Einzelfall geprüft werden.

**Empfehlung**	**Modifiziert**<br>**Stand (2023)**
▪ Zur frühzeitigen Diagnostik Parkinson-bedingter Dysphagien können standardisierte Fragebögen und regelmäßige klinische Schluckuntersuchungen eingesetzt werden, welche die Effektivität und die Sicherheit des Schluckens beurteilen.<br>▪ Zur Schweregradbestimmung inklusive des zuverlässigen Nachweises stiller Aspirationen sowie zur detaillierten Störungsmusteranalyse der Parkinson-bedingten oropharyngealen Dysphagie können die flexible endoskopische Evaluation des Schluckakts (FEES) oder die Videofluoroskopie des Schluckens (VFSS) eingesetzt werden.<br>▪ Zum Nachweis einer Levodopa-Responsivität der Parkinson-bedingten pharyngealen Dysphagie kann die FEES mit spezifischem Protokoll (FEES-Levodopa-Test) genutzt werden.<br>▪ Außerdem kann die High-Resolution-Manometrie (HRM) ösophageale Motilitätsstörungen detektieren.<br>▪ Pat. mit Parkinson-bedingten Schluckstörungen sollen eine logopädische Schlucktherapie erhalten.<br>▪ Bei der logopädischen Schlucktherapie ist auf eine Störungsmuster-spezifische Auswahl der Therapieverfahren zu achten. Die Effektivität der jeweiligen Verfahren kann durch spezielle Diagnostik zu Therapiebeginn und im Verlauf mittels FEES oder VFSS beurteilt werden. Die FEES kann auch zur videoassistierten logopädischen Schlucktherapie (VAST) genutzt werden.<br>▪ Parkinson-Pat. mit leicht- und mittelgradiger hypokinetischer Dysphagie sollten ein vierwöchiges EMST-Training erhalten. Hierdurch können die Schluckeffektivität und die Schlucksicherheit verbessert werden.<br>▪ Bei Parkinson-Pat. mit hypokinetischer Dysphagie kann auch eine Optimierung der dopaminergen Medikation eine Verbesserung des Schluckens bewirken.	
Konsensstärke: 94,1%, Konsens	

Referenzen

1. Herd CP, Tomlinson CL, Deane KH, Brady MC, Smith CH, Sackley CM, Clarke CE. Speech and language therapy versus placebo or no intervention for speech problems in Parkinson's disease. Cochrane Database Syst Rev. 2012;2012(8):Cd002812
2. Skodda S, Rinsche H, Schlegel U. Progression of dysprosody in Parkinson's disease over time--a longitudinal study. Mov Disord. 2009;24(5):716-22

3. Müller J, Wenning GK, Verny M, McKee A, Chaudhuri KR, Jellinger K, et al. Progression of dysarthria and dysphagia in postmortem-confirmed parkinsonian disorders. Arch Neurol. 2001;58(2):259-64
4. Logemann JA, Fisher HB, Boshes B, Blonsky ER. Frequency and cooccurrence of vocal tract dysfunctions in the speech of a large sample of Parkinson patients. J Speech Hear Disord. 1978;43(1):47-57
5. Stewart C, Winfield L, Hunt A, Bressman SB, Fahn S, Blitzer A, Brin MF. Speech dysfunction in early Parkinson's disease. Mov Disord. 1995;10(5):562-5
6. Nawka T, Wiesmann U, Gonnermann U. [Validation of the German version of the Voice Handicap Index]. Hno. 2003;51(11):921-30
7. Dashtipour K, Tafreshi A, Lee J, Crawley B. Speech disorders in Parkinson's disease: pathophysiology, medical management and surgical approAcetylcholinesterase-Hemmer. Neurodegener Dis Manag. 2018;8(5):337-48
8. Rusz J, Tykalova T, Ramig LO, Tripoliti E. Guidelines for Speech Recording and Acoustic Analyses in Dysarthrias of Movement Disorders. Mov Disord. 2021;36(4):803-14
9. Muñoz-Vigueras N, Prados-Román E, Valenza MC, Granados-Santiago M, Cabrera-Martos I, Rodríguez-Torres J, Torres-Sánchez I. Speech and language therapy treatment on hypokinetic dysarthria in Parkinson disease: Systematic review and meta-analysis. Clin Rehabil. 2021;35(5):639-55
10. Herd CP, Tomlinson CL, Deane KH, Brady MC, Smith CH, Sackley CM, Clarke CE. Comparison of speech and language therapy techniques for speech problems in Parkinson's disease. Cochrane Database Syst Rev. 2012;2012(8):Cd002814
11. Ramig LO, Fox C, Sapir S. Parkinson's disease: speech and voice disorders and their treatment with the Lee Silverman Voice Treatment. Semin Speech Lang. 2004;25(2):169-80
12. de Swart BJ, Willemse SC, Maassen BA, Horstink MW. Improvement of voicing in patients with Parkinson's disease by speech therapy. Neurology. 2003;60(3):498-500
13. Griffin M, Bentley J, Shanks J, Wood C. The effectiveness of Lee Silverman Voice Treatment therapy issued interactively through an iPad device: A non-inferiority study. J Telemed Telecare. 2018;24(3):209-15
14. Dias AE, Barbosa ER, Coracini K, Maia F, Marcolin MA, Fregni F. Effects of repetitive transcranial magnetic stimulation on voice and speech in Parkinson's disease. Acta Neurol Scand. 2006;113(2):92-9
15. Kalf JG, de Swart BJ, Bloem BR, Munneke M. Prevalence of oropharyngeal dysphagia in Parkinson's disease: a meta-analysis. Parkinsonism Relat Disord. 2012;18(4):311-5
16. Suttrup I, Warnecke T. Dysphagia in Parkinson's Disease. Dysphagia. 2016;31(1):24-32
17. Patel B, Legacy J, Hegland KW, Okun MS, Herndon NE. A comprehensive review of the diagnosis and treatment of Parkinson's disease dysphagia and aspiration. Expert Rev Gastroenterol Hepatol. 2020;14(6):411-24
18. Fukae J, Fujioka S, Umemoto G, Arahata H, Yanamoto S, Mishima T, Tsuboi Y. Impact of Residual Drug in the Pharynx on the Delayed-On Phenomenon in Parkinson's Disease Patients. Mov Disord Clin Pract. 2020;7(3):273-8
19. Labeit B, Berkovich E, Claus I, Roderigo M, Schwake AL, Izgelov D, et al. Dysphagia for medication in Parkinson's disease. NPJ Parkinsons Dis. 2022;8(1):156
20. Labeit B, Claus I, Muhle P, Regner L, Suntrup-Krueger S, Dziewas R, Warnecke T. Effect of cognitive and motor dual-task on oropharyngeal swallowing in Parkinson's disease. Eur J Neurol. 2021;28(3):754-62
21. Labeit B, Claus I, Muhle P, Lapa S, Suntrup-Krueger S, Dziewas R, et al. Oropharyngeal freezing and its relation to dysphagia - An analogy to freezing of gait. Parkinsonism Relat Disord. 2020;75:1-6

22. Manor Y, Giladi N, Cohen A, Fliss DM, Cohen JT. Validation of a swallowing disturbance questionnaire for detecting dysphagia in patients with Parkinson's disease. Mov Disord. 2007;22(13):1917-21
23. Simons JA, Fietzek UM, Waldmann A, Warnecke T, Schuster T, Ceballos-Baumann AO. Development and validation of a new screening questionnaire for dysphagia in early stages of Parkinson's disease. Parkinsonism Relat Disord. 2014;20(9):992-8
24. Warnecke T, Schäfer KH, Claus I, Del Tredici K, Jost WH. Gastrointestinal involvement in Parkinson's disease: pathophysiology, diagnosis, and management. NPJ Parkinsons Dis. 2022;8(1):31
25. Warnecke T, Suttrup I, Schröder JB, Osada N, Oelenberg S, Hamacher C, et al. Levodopa responsiveness of dysphagia in advanced Parkinson's disease and reliability testing of the FEES-Levodopa-test. Parkinsonism Relat Disord. 2016;28:100-6
26. Baijens LW, Speyer R. Effects of therapy for dysphagia in Parkinson's disease: systematic review. Dysphagia. 2009;24(1):91-102
27. van Hooren MR, Baijens LW, Voskuilen S, Oosterloo M, Kremer B. Treatment effects for dysphagia in Parkinson's disease: a systematic review. Parkinsonism Relat Disord. 2014;20(8):800-7
28. López-Liria R, Parra-Egeda J, Vega-Ramírez FA, Aguilar-Parra JM, Trigueros-Ramos R, Morales-Gázquez MJ, Rocamora-Pérez P. Treatment of Dysphagia in Parkinson's Disease: A Systematic Review. Int J Environ Res Public Health. 2020;17(11)
29. Schindler A, Pizzorni N, Cereda E, Cosentino G, Avenali M, Montomoli C, et al. Consensus on the treatment of dysphagia in Parkinson's disease. J Neurol Sci. 2021;430:120008
30. Troche MS, Okun MS, Rosenbek JC, Musson N, Fernandez HH, Rodriguez R, et al. Aspiration and swallowing in Parkinson disease and rehabilitation with EMST: a randomized trial. Neurology. 2010;75(21):1912-9
31. Claus I, Muhle P, Czechowski J, Ahring S, Labeit B, Suntrup-Krueger S, et al. Expiratory Muscle Strength Training for Therapy of Pharyngeal Dysphagia in Parkinson's Disease. Mov Disord. 2021;36(8):1815-24
32. Manor Y, Mootanah R, Freud D, Giladi N, Cohen JT. Video-assisted swallowing therapy for patients with Parkinson's disease. Parkinsonism Relat Disord. 2013;19(2):207-11
33. Troche MS, Brandimore AE, Foote KD, Okun MS. Swallowing and deep brain stimulation in Parkinson's disease: a systematic review. Parkinsonism Relat Disord. 2013;19(9):783-8
34. Chang MC, Park JS, Lee BJ, Park D. The Effect of Deep Brain Stimulation on Swallowing Function in Parkinson's Disease: A Narrative Review. Dysphagia. 2021;36(5):786-99

3.19 Pumpentherapien

Autoren: Christoph Schrader, Lars Tönges

Fragestellung 162: Wie effektiv und sicher ist die Apomorphin-Pumpentherapie im Vergleich mit der oralen/transdermalen Standardtherapie bei der Behandlung von motorischen Fluktuationen und Dyskinesien der PK?

Hintergrund

Apomorphin ist ein gemischter D1- und D2-Agonist und von allen Dopaminagonisten der potenteste. Die orale Bioverfügbarkeit beträgt nur 4 %, weshalb es subkutan appliziert werden muss, um seinen vollen Effekt zu entfalten.

Der motorische Effekt nach einer Einzeldosis von subkutanem Apomorphin ist vergleichbar mit dem von Levodopa, setzt aber mit einer Latenz von 4–12 Minuten deutlich rascher ein, und die mittlere Wirkdauer beträgt 45–60 Minuten. Anders als Levodopa benötigt es keinen aktiven Transportmechanismus, um das Zentralnervensystem zu erreichen. Die Absorption variiert mit Hauttemperatur und Blutfluss, und die Resorption ist am besten, wenn sie im Subkutangewebe der Bauchwand erfolgt [1].

Zur Glättung motorischer Fluktuationen kann eine Apomorphin-Lösung kontinuierlich über eine kleine Pumpe subkutan appliziert werden. Die Pumpe wird außerhalb des Körpers an einem Gürtel oder Halsband getragen. Üblicherweise wird Apomorphin über einen Subkutankatheter über 12–16 (in Einzelfällen auch bis zu 24) Stunden in das Unterhautfettgewebe des Abdomens (oder auch der Oberschenkel) infundiert.

Die Einstellung erfolgt in der Regel stationär in einem spezialisierten Zentrum. Vor Beginn sollten mittels EKG eine verlängerte QTc-Zeit, Bradyarrhythmien, Tachykardien oder Vorhofflimmern sowie eine vorbestehende hämolytische Anämie ausgeschlossen werden. Am Tag vor Beginn der Behandlung sollten Pat. mit 3x 10 mg Domperidon vorbehandelt werden, das nach Erreichen einer stabilen Flussrate zügig wieder beendet werden kann. Die kontinuierliche Infusion wird für gewöhnlich mit einer Flussrate von 0,5–1,0 mg Apomorphin/h am ersten Tag begonnen und die tägliche stündliche Flussrate in Abhängigkeit von Wirksamkeit und Verträglichkeit graduell gesteigert. Übliche Dosen liegen zwischen 5 und 7 mg/h, in Einzelfällen höher.

Während der kontinuierlichen Subkutaninfusion von Apomorphin oder bereits zuvor sollten andere Dopaminagonisten, MAO-B- und COMT-Hemmer, Amantadin und schließlich Levodopa schrittweise reduziert werden, normalerweise innerhalb der ersten 7 Tage.

Vor allem bei bestehenden Dyskinesien ist es das Ziel, die orale Medikation so weit wie möglich zu reduzieren und, wenn möglich, tagsüber ganz abzusetzen. Diese Umstellung kann mehrere Wochen in Anspruch nehmen und ambulant vorbereitet werden.

Zur Behandlung von Schlafstörungen und schlafassoziierter motorischer Symptome kann eine nächtliche Infusionstherapie in ausgewählten Fällen hilfreich sein [2].

Apomorphin über ein Pumpensystem ist zugelassen „zur Behandlung von motorischen Fluktuationen (‚On-Off‘-Phänomen) bei Parkinson-Pat., die durch oral angewendete Anti-Parkinson-Mittel nicht hinreichend eingestellt werden können“.

Evidenzgrundlage

Obwohl Apomorphin seit über 30 Jahren eingesetzt wird, fanden sich in dieser Literaturrecherche lediglich eine randomisiert kontrollierte Studie und 8 longitudinale Kohortenstudien, mit einer Ausnahme allerdings mit n < 50 (n = 11–43).

Ergebnis

Wirksamkeit:

Die einzige randomisierte kontrollierte Untersuchung, die TOLEDO-Studie, untersuchte die Wirksamkeit und Sicherheit von kontinuierlicher Apomorphin-Infusion gegenüber Placebo an 106 Pat. über einen Zeitraum von 12 Wochen [3]. Primärer Endpunkt war die absolute Reduktion von täglicher Off-Zeit. Die Begleitmedikation wurde reduziert, wenn während des Krankenhausaufenthalts in den ersten 10 Tagen nach Beginn der Behandlungsphase dopaminerge unerwünschte Wirkungen (z. B. Dyskinesien) auftraten. Bis zu 300 mg Levodopa oral bei Bedarf waren erlaubt.

Die Apomorphin-Infusion (im Mittel 4,68 [±1,50] mg/h) reduzierte die Off-Zeit um 37% im Vergleich zu Baseline und signifikant gegen Placebo um 1,89 [3,2–0,6] h/d (bzw. 28% mehr als Placebo).

Von den sekundären Endpunkten war Apomorphin dem Placebo in folgenden Kategorien signifikant überlegen: Anzahl von Pat. mit > 2 h Off-Reduktion/d: -33,4 %; Patient Global Impression of Change: -1,20; On-Zeit ohne behindernde Dyskinesien: -1,97 h/d; Reduktion der Levodopa-Äquivalenztagesdosis -328,5 mg. Apomorphin war nicht überlegen in Bezug auf Reduktion der oralen Levodopa-Dosis/d, der UPDRS III im On sowie in der Lebensqualität.

In den anderen unkontrollierten, teilweise multizentrischen und nur zum Teil prospektiven, auf maximal 2 Jahre ausgelegten Beobachtungsstudien fanden sich – zumindest bei den Pat., die bei der Therapie blieben – Reduktionen der täglichen Off-Zeit in einer Größenordnung von 40 bis 80 % gegenüber dem Ausgangswert. Über Dyskinesien wurde nur in einem Teil der Studien berichtet. Viele Pat. berichteten insgesamt über eine Besserung und es gab Hinweise auf einen Zusammenhang zwischen der Reduktion der oralen Medikation und dem Ausmaß der Besserung von Dyskinesien [4], [5], [6], [7], [8], [9].

Sicherheit:

In puncto Arzneimittelsicherheit fanden sich in der TOLEDO-Studie in der Apomorphin-Gruppe signifikant mehr unerwünschte Ereignisse als in der Placebogruppe. Zu den häufigsten unerwünschten Ereignissen zählten Knötchenbildung unter der Haut (44 %), Übelkeit und Somnolenz (je 22 %), Erythem an der Infusionsstelle (17 %), Dyskinesien (15 %), Kopfschmerz (13 %) und Insomnie (11 %). Bei 11 % führten unerwünschte Ereignisse zum Studienabbruch innerhalb der 12 Wochen Beobachtungszeit, bei 48 % führten sie zur Änderung der Dosis [3].

In der offenen 52-wöchigen Phase hatte die Knötchenbildung zugenommen (54%), wohingegen die übrigen UAW in ihrer Häufigkeit unverändert geblieben waren. Die Abbruchrate wegen UAW lag bei 16,7% [5].

In den älteren unkontrollierten Kohortenstudien wurden UAW nur teilweise beschrieben. Am häufigsten waren Knötchenbildungen (8–100%), Nausea (7–27%), Psychose (8–40%), Hypomanie/Impulskontrollstörungen (3–9%), Somnolenz (5–67%), symptomatische Orthostasereaktion (16–25%) und hämolytische Anämie (1,2–1,5%).

In der Open-label-Extensionsstudie der TOLEDO-Studie über weitere 52 Wochen, in die 84 von den ursprünglich 106 Pat. eintraten und die von 59 Pat. beendet wurde, war die Off-Zeit um 53% gegenüber Baseline (-3,66 h/d) reduziert [5].

Begründung der Empfehlung

Die Evidenz beruht auf einer randomisierten kontrollierten Studie und 8 offenen Beobachtungsstudien.

**Empfehlung**	**Neu**<br><br>**Stand (2023)**
▪ Die Apomorphin-Pumpentherapie sollte für die Behandlung motorischer Fluktuationen zur Reduktion von Off-Phasen,Reduktion von Dyskinesien und Verlängerung der On-Zeit angewandt werden.<br>▪ Wegen der komplexen Handhabung dieses Verfahrens und der Häufigkeit von Komplikationen ist eine engmaschige Pat.-Begleitung empfohlen und es sollte nur von in dieser Therapieform erfahrenen Ärzten/Ärztinnen begonnen und begleitet werden.	
Konsensstärke: 100%, starker Konsens	

Fragestellung 163: Wie effektiv und sicher ist die Apomorphin-Pumpentherapie im Vergleich mit der oralen/transdermalen Standardtherapie bei der Behandlung der nicht motorischen Parkinson-Symptome und von deren Fluktuationen (Schlaf, Schmerz, vegetative Symptome (übermäßiges Schwitzen, Dysurie, gastrointestinale Symptome, orthostatische Hypotonie), neuropsychiatrische Symptome (Apathie, Fatigue, Depression, Angst, Impulskontrollstörung, Punding, Dopamindysregulationssyndrom))?

Hintergrund

Die Häufigkeit und Schwere nicht motorischer Symptome nimmt im Verlauf des idiopathischen Parkinson-Syndroms zu und beeinträchtigt zunehmend die Lebensqualität [10]. Apomorphin ist der stärkste und schnellstwirksame Dopaminagonist mit jedoch kurzer Halbwertszeit. Insbesondere bei Pat. mit orthostatischer Hypotension, Impulskontrollstörungen und Psychosen in der Vorgeschichte können Dopaminagonisten erhöht komplikationsträchtig sein. Auf der anderen Seite können Dopaminagonisten insbesondere in Bezug auf affektive Störungen auch hilfreich sein. Hier soll

diskutiert werden, inwiefern eine Apomorphin-Pumpentherapie nicht motorische Symptome im Rahmen der fortgeschrittenen PK beeinflusst.

Evidenzgrundlage

Zu dieser Fragestellung finden sich keine randomisierten, kontrollierten Studien und keine größeren Kohortenstudien. Es finden sich 3 longitudinale größere Beobachtungsstudien, die die Effekte von subkutaner Apomorphin-Infusion auf der Non-Motor-Symptom-Scale (NMSS) im Vergleich zu Levodopa/Carbidopa-Intestinalgel (LCIG) bzw. Deep Brain Stimulation (DBS) untersuchen, und eine Übersichtsarbeit.

Ergebnis

Obwohl Apomorphin deutlich länger als DBS und LCIG verfügbar ist – auf dem europäischen Markt ist es seit den frühen 1990ern verfügbar –, ist die Datenlage insbesondere zu nicht motorischen Symptomen limitiert. Die NMSS-Daten aus der TOLEDO-Studie [3] sind noch nicht publiziert. Einige offene Beobachtungs- und fallbasierte Studien mit insgesamt 93 Pat. zeigten, dass subkutane Apomorphin-Infusion günstige Effekte sowohl auf den NMSS-Gesamtscore als auch auf bestimmte nicht motorische Subdomänen haben kann [6], [7], [11]. Kurz zusammengefasst, minderte sich der NMSS-Gesamtscore in allen Domänen über einen Zeitraum von 6 bis 12 Monaten Behandlungsdauer mit besonderer Betonung der Domänen Schlaf/Müdigkeit und Stimmung/Apathie, Aufmerksamkeit/Kognition sowie Wahrnehmung/Halluzinationen und Aufmerksamkeit/Gedächtnis und sonstige Symptome. Die geringsten Effekte fanden sich in den Domänen Kkardiovaskulär und Sexualfunktion. Bemerkenswert ist, dass LCIG in den EuroInf-Studien in nahezu allen Domänen relativ größere Effekte zeigte [7], [11].

Obwohl Apomorphin als starker D1- und D2-Rezeptoragonist prinzipiell ein größeres halluzinogenes Risiko als Levodopa birgt, wurden unter Apomorphin-Infusion günstige Effekte in Bezug auf die Rückbildung milder visueller Halluzinationen beschrieben. Als mögliche Gründe hierfür wurden die Reduktion der Begleitmedikation inklusive der Polypharmazie und das vergleichsweise junge Pat.-Alter sowie die kurze Krankheitsdauer (62,2 Jahre bzw. 13.5 Jahre) angenommen. Ferner wurde auch eine mögliche günstige psychotrope Wirkung von Apomorphin – basierend auf einer gewissen Strukturähnlichkeit mit Piperidin – postuliert [12].

Begründung der Empfehlung

Die Empfehlung beruht auf 3 offenen Beobachtungstudien mit insgesamt 93 Pat., die einen Behandlungszeitraum vom maximal 12 Monaten abbilden.

Empfehlung	Neu<br><br>Stand (2023)
Die kontinuierliche subkutane Apomorphin-Infusion kann im NMSS gemessene nicht motorische Symptome, insbesondere Schlaf/Müdigkeit und Stimmung/Apathie, Aufmerksamkeit/Kognition sowie Wahrnehmung/Halluzinationen und Aufmerksamkeit /Gedächtnis und sonstige Symptome lindern. Diese Effekte können bei der Auswahl der Pat. für eine Apomorphin-Infusionstherapie als mögliche Determinanten herangezogen werden.	
Konsensstärke: 95,3%, starker Konsens	

Fragestellung 164: Wie nachhaltig ist die klinische Symptomkontrolle durch die Apomorphin-Pumpentherapie im Vergleich mit der oralen/transdermalen Standardtherapie in der Behandlung der PK mit motorischen Fluktuationen mit und ohne Dyskinesien?

Hintergrund

Eine invasive Therapieform bedeutet für den Pat. eine große Umstellung von der vormaligen oralen Medikation. Sie sollte nach Möglichkeit nachhaltig sein. Im Folgenden soll die langzeitige Wirksamkeit bzw. Sicherheit evaluiert werden.

Evidenzgrundlage

In dieser Literaturrecherche zu dieser Frage fanden sich 1 randomisierte, kontrollierte Studie mit einem Beobachtungszeitraum von 18 Monaten sowie 8 longitudinale Kohortenstudien.

Ergebnis

In der Open-label-Phase der TOLEDO-Studie, die einschließlich der verblindeten Phase einen Beobachtungszeitraum von 18 Monaten umfasste, lag die mittlere Off-Zeit-Reduktion bei -3,66 Stunden (-45 %); störende Dyskinesien waren moderat reduziert, die orale Levodopa-Dosis konnte um etwa 1/4, die Levodopa-Äquivalenzdosis um etwa 1/3 reduziert werden. 30% haben die Studie nicht zu Ende geführt, 17% wegen unerwünschter Ereignisse (Hautreaktionen, Fatigue, Autoimmunhämolyse, Delir, Demenz, Aufmerksamkeitsdefizit, Lymphom, Übelkeit, Panikattacken und Somnolenz) [13].

Größere Langzeitstudien fehlen. Die prospektiven Open-labelStudien umfassten meist nur geringe Pat.zahlen (< 50 Pat.) mit einem Beobachtungszeiträumen von mindestens 1,5 Jahren (im Mittel 40 Monate) [14], [5], [7], [15], [11], [12]. Sie beschreiben einen stabilen motorischen Effekt mit Off-Zeit-Reduktion von 25-50% gegenüber dem Ausgangswert, aber Abbruchraten von 20 bis 83%.

Eine offene Studie mit 114 Pat., die über mindestens 6 Monate mit subkutanen Apomorphin-Infusionen behandelt worden sind, beschäftigte sich mit den Gründen für die Beendigung der Behandlung [16]. Die mittlere Behandlungsdauer bis zum Abbruch betrug 2,42 ± 2,23 Jahre (0,5–9,2). Mit 38% war die Wiederkehr schwerer Dyskinesien der Grund für die Beendigung, ca. 16 % (überwiegend ältere Pat.) stoppten wegen kognitiver Schwierigkeiten, ca. 14% wegen

Hautreaktionen, ca. 12% wegen Haltungsinstabilität, je 11% wegen Halluzinationen bzw. Depression/Ängstlichkeit.

Die längste prospektive Untersuchung über einen Zeitraum von 5 Jahren ist mit 12 Pat. (Alter bei Studienbeginn 58 Jahre, Krankheitsdauer 9 Jahre) klein: Nur 2 von 12 Pat. (17 %) waren nach 5 Jahren immer noch mit Apomorphin behandelt. Die mittlere Behandlungsdauer war 30 Monate. 3 waren in den Jahren 2–5 verstorben (nicht im Verhältnis mit der Behandlung stehend), 5 hatten wegen Wiederkehr schwerer Fluktuationen (schwere Dyskinesien und Off-Phasen) in den Jahren 3–5 abgebrochen und wurden nachfolgend mit DBS bzw. LCIG behandelt, 2 brachen im zweiten Jahr wegen Hautknötchen ab, 1 war lost to follow-up. Bei denjenigen, die bei der Behandlung blieben, blieben die Off-Phasen unter Kontrolle, die Dauer und Behinderung durch Dyskinesien hatten sich nicht signifikant verbessert [17].

Begründung der Empfehlung

Die Evidenz beruht auf 1 kontrollierten, randomisierten Studie mit Open-label-Erweiterung, die einen Zeitraum von 18 Monaten abbildet, und 8 Beobachtungsstudien mit einer mittleren Beobachtungsdauer von 40 (18–60) Monaten. Kontrollierte Langzeitdaten sind nicht verfügbar. Die Wirksamkeit einer Apomorphin-Pumpentherapie ist belegt für einen Zeitraum von 18 Monaten. Mit zunehmender Behandlungsdauer steigt das Risiko eines Therapieabbruchs (im Mittel nach 2,5 Jahren) [16], [17].

**Empfehlung**	**Neu**<br><br>**Stand (2023)**
▪ Die Wirksamkeit einer Apomorphin-Pumpentherapie zur Behandlung motorischer Fluktuationen ist belegt für einen Zeitraum von 18 Monaten, in Einzelfällen von bis zu 5 Jahren.<br>▪ Die mittlere Behandlungsdauer bis zum Abbruch der kontinuierlichen subkutanen Apomorphin-Infusion aus unterschiedlichen Gründen liegt im Mittel bei 2,5 Jahren.	
Konsensstärke: 95%, Konsens	

Fragestellung 165: Wie effektiv und sicher ist die Levodopa-Carbidopa-Intestinalgel-Pumpentherapie im Vergleich mit der oralen/transdermalen Standardtherapie bei der Behandlung von motorischen Fluktuationen und Dyskinesien der PK.

Hintergrund

Levodopa/Carbidopa-Intestinalgel (LCIG) ist eine hochkonzentrierte Formulierung von Levodopa/Carbidopa (20/5 mg/ml), die über ein Pumpensystem direkt in das Jejunum infundiert wird. Durch eine kontinuierliche Infusion werden konstante Plasmaspiegel erreicht, was eine gleichmäßige Stimulation striataler Dopaminrezeptoren ermöglichen soll.

Das System besteht aus einer tragbaren Pumpe, einer angeschlossenen Kassette mit 100 ml LCIG (= 2000/500 mg Levodopa/Carbidopa) und einer perkutan endoskopischen Gastrostomie mit jejunaler

Extension (PEG-J). Die Pumpe ermöglicht die Voreinstellung einer Morgendosis und einer kontinuierlichen Flussrate sowie die Applikation von Boli.

Die Einstellung auf eine LCIG-Behandlung erfolgt in der Regel stationär. Der klassische Behandlungsbeginn sieht eine Nasojejunalphase vor, in der vor der endoskopischen Anlage einer PEG-J die Wirksamkeit über eine Nasojejunalsonde geprüft wird und ferner den Pat. die Möglichkeit eingeräumt wird, den Effekt einer kontinuierlichen dopaminergen Stimulation zu erleben. In Einzelfällen kann bei klarer Indikation und entsprechendem Wunsch der Pat. auf diese Phase verzichtet werden.

Die Umstellung von oraler/transdermaler Therapie auf LCIG erfolgt nach einem definierten Schema: Die Morgendosis entspricht i.d.R. der Menge morgendlichen oralen Levodopas, die kontinuierliche Flussrate/h entspricht der Levodopa-Äquivalenztagesdosis abzüglich der Morgendosis geteilt durch die Laufzeit/Tag in Stunden. Extradosen liegen bei Beginn der Behandlung i.d.R. zwischen 0,5 und 2 ml (= 10–40 mg Levodopa). Die mittlere Titrationsdauer liegt bei 5–10 Tagen, das Absetzen langwirksamer Dopaminagonisten vor Beginn der Behandlung verkürzt die Titrationsdauer auf die Hälfte.

Die Zulassung sieht eine Infusionsdauer von 16 h/d vor; bis zu 20% der Pat. haben eine 24-h-Infusion (außerhalb der Zulassung) zur Kontrolle nächtlicher Akinesie.

Angestrebt wird tagsüber eine Monotherapie. Bei nächtlichen Symptomen, die mit retardiertem Levodopa zur Nacht nicht hinreichend zu kontrollieren sind, kann eine Kombination mit einem langwirksamen (idealerweise transdermalen) Dopaminagonisten und/oder COMT-Hemmer erwogen werden.

Zugelassen ist LCIG in der EU für Pat. „mit fortgeschrittener, auf Levodopa-reaktiver PK mit schweren motorischen Fluktuationen und Hyper- oder Dyskinesien, wenn verfügbare Kombinationen von Anti-Parkinson-Mitteln nicht zu zufriedenstellenden Ergebnissen geführt haben“.

Es gibt eine Weiterentwicklung mit einer Levodopa/Carbidopa/Entacapon-Gel-Formulierung, die über eine kleinere und handlichere Pumpe ebenfalls über eine PEG-J intestinal infundiert wird [18]. Eine Kartusche beinhaltet 940 mg Levodopa, 235 mg Carbidopa und 940 mg Entacapon. Der COMT-Hemmer-Anteil lässt ca. 35% des Levodopa-Bedarfs einsparen [19]. Damit gehen aber auch für Entacapon bekannte, mögliche Nebenwirkungen einher (u.a. harmlose Verfärbung des Urins, aber auch unstillbare Diarrhoen in ca. 10% [20]).

Das Präparat Foslevodopa ist eine subkutane Formulierung von Levodopa/Carbidopa im Verhältnis 20 : 1, für die aktuell eine Zulassung für eine 24-Stunden-Therapie beantragt ist. Präklinische Studien haben mit LCIG vergleichbare stabile Plasmaspiegel nachgewiesen [21]. Die Ergebnisse der Zulassungsstudie über 12 Wochen zeigten eine mit LCIG vergleichbare Wirksamkeit in Bezug auf motorische Fluktuationen; die häufigsten UAW standen in Zusammenhang mit der subkutanen Applikationsweise und bestanden in subkutanen Hautknötchen und Erythemen [22].

Evidenzgrundlage

Für diese Kapitel: In dieser Literaturrecherche fanden sich:

- N = 2 randomisierte, kontrollierte Studien,
- N= 18 longitudinale größere Kohortenstudien.

Ergebnis

Wirksamkeit:

Zwei randomisierte kontrollierte Studien, die den motorischen Effekt von LCIG beschreiben, wurden identifiziert [23], [24].

Eine schwedische multizentrische randomisierte kontrollierte Studie zeigte bei 21 Pat. über einen Zeitraum von 3 Wochen in einem Crossover-Design eine signifikante Reduktion von schweren Off-Phasen mit LCIG im Vergleich zur oralen Therapie. Es gab keinen Unterschied in dem Auftreten von Dyskinesien. UPDRS Part II und Part IV waren in der LCIG-Gruppe signifikant besser, wohingegen Part III im On unverändert war [23].

Die qualitativ beste Studie war eine doppelblinde Doppel-Dummy-randomisierte kontrollierte Studie über 12 Wochen mit 37 Pat. in der LCIG-Gruppe und 34 Pat. mit einer oralen Therapie. Die Off-Phasen in der LCIG-Gruppe waren von 6,3 auf 2,3 h/d um 64 %, reduziert und um -1,91 h/Tag gegenüber oraler Therapie besser. Die On-Zeit ohne störende Dyskinesien war signifikant besser als in der oralen Gruppe. UPDRS Teil II war signifikant besser in der LCIG-Gruppe als in der oral behandelten, der UPDRS III war im motorischen On unverändert [24].

Mehrere offene Studien bestätigen die Wirksamkeit hinsichtlich der Reduktion von On-Zeit ohne störende Dyskinesien [7], [25], [26], [27], [28], [29], [30], [31], [32], [33], [34], [35], [36], [37], [38], [39]. Die Verbesserung und Stabilität bleiben bei mehreren Studien über mindestens 12 Monate erhalten. Die meisten offenen Studien hatten keine Kontrollgruppe, viele waren retrospektiv oder haben Pat. nicht in einer konsekutiven Art und Weise rekrutiert.

Sicherheit:

In puncto (Arzneimittel-)Sicherheit fanden sich in der Placebo-kontrollierten Doppel-Dummy-Studie bei 95 % in der LCIG-Gruppe und 100 % in der Vergleichsgruppe unerwünschte Ereignisse; die Mehrheit trat innerhalb der ersten Wochen ein und fast alle (89%) waren assoziiert mit der Anlage der PEG-J (Bauchschmerzen, Übelkeit, Verstopfung, Flatulenz, Erythem an der Inzisionsstelle). 3 Pat. haben die Studie innerhalb der 12 Wochen Beobachtungszeit wegen unerwünschter Wirkungen abgebrochen: einer in der LCIG-Gruppe (Psychose) und zwei in der Vergleichsgruppe (Peritonitis/Pneumonie und Wundsekretion) [24].

Das offene GLORIA-Register, in dem 208 Pat. auf stabilem Therapieregime über 24 Monate verfolgt wurden, hat mittelfristige unerwünschte Ereignisse dokumentiert: 69% hatten mindestens ein unerwünschtes Ereignis, 45% mindestens ein schwerwiegendes Ereignis, 10% beendeten wegen eines schwerwiegenden Ereignisses die Behandlung. Zu den schwerwiegenden, zum Behandlungsabbruch führenden Ereignissen zählten PEG-J-assoziierte Probleme (3%), neuropsychiatrische Komplikationen (1%) und Polyneuropathien (0,5%); die übrigen waren

krankheitsimmanent (Aspiration, Pneumonie, Krankheitsprogress) oder auf andere Erkrankungen zurückzuführen (ICB, Gallengangskarzinom). PEG-J-Komplikationen wie Dislokationen, PEG-J-Okklusionen, Pumpenfehler, Stomainfektionen kamen bei 21% vor, neuropsychiatrische Komplikationen (Delir, Halluzinationen, Depression) bei 10%, Gewichtsverlust bei 6 % und Polyneuropathien bei 11,5% [40].

Die Ursache von neu aufgetretenen Polyneuropathien unter LCIG, deren Inzidenz auch in anderen Serien um mehr als 10% liegt, ist nicht abschließend geklärt. Mehrere Studien haben einen Abfall von Vitamin B6, B12 und Folat unter LCIG beobachtet, die Substrate im Levodopa-Metabolismus sind [41], [42], [43], [44]. Insbesondere Vitamin B6 fällt bei hohen Dosen Levodopa/Carbidopa rasch ab [44].

Begründung der Empfehlung

Die Evidenz beruht auf einer einzigen kontrollierten Studie mit einer Beobachtungsdauer von 3 Monaten, daher ist die Evidenz für Nachhaltigkeit und Komplikationen nur limitiert. Eine längerfristige offene Studie über 2 Jahre zeigte anhaltende Veränderungen in Off- und On-Zeit in einem multinationalen prospektiven Register. Unerwünschte Ereignisse sind in der kontrollierten Studie und in den offenen Studien berichtet worden und vorwiegend PEG-J-assoziiert.

**Empfehlung**	**Neu**<br><br>**Stand (2023)**
▪ LCIG kann die Dauer von On-Zeiten ohne störende Dyskinesien signifikant erhöhen und die Off-Zeiten signifikant reduzieren und sollte daher zur Behandlung oral nicht befriedigend zu behandelnder motorischer Fluktuationen angewandt werden.<br>▪ Die Behandlung ist verhältnismäßig sicher, die häufigsten Komplikationen sind PEG-J-assoziiert.<br>▪ Vor Behandlungsbeginn sollten ein elektrophysiologisches Neuropathie-Screening und die Bestimmung von Vitaminen B6, B12 und Folsäure sowie des Körpergewichts erfolgen und unter Behandlung monitoriert bzw. bei erniedrigten Werten substituiert werden.<br>▪ Wegen der komplexen Handhabung dieses Verfahrens und der Häufigkeit von Komplikationen ist eine engmaschige Pat.-Begleitung empfohlen und es sollte nur von in dieser Therapieform erfahrenen Ärzten/Ärztinnen begonnen und begleitet werden.	
Konsensstärke: 100%, starker Konsens	

Fragestellung 166: Wie effektiv ist die Levodopa-Carbidopa Intestinalgel-Pumpentherapie im Vergleich mit der oralen/transdermalen Standardtherapie bei der Behandlung der nicht-motorischen Parkinson-Symptome und deren Fluktuationen (Schlaf, Schmerz, vegetative Symptome (übermäßiges Schwitzen, Dysurie, gastrointestinale Symptome, orthostatische Hypotonie), neuropsychiatrische Symptome (Apathie, Fatigue, Depression, Angst, Impulskontrollstörung, Punding, Dopamindysregulationssyndrom)) der PK?

Hintergrund

Die Häufigkeit und Schwere nicht motorischer Symptome nimmt im Verlauf der PK zu und beeinträchtigt zunehmend die Lebensqualität [10]. Hier soll diskutiert werden, inwiefern eine LCIG-Therapie nicht motorische Symptome im Rahmen der PK beeinflusst.

Evidenzgrundlage

Für diese Kapitel: In dieser Literaturrecherche fanden sich:

- N=0 randomisierte, kontrollierte Studien
- N=12 longitudinale größere Kohortenstudien
- N=2 Übersichtsarbeiten.

Ergebnis

Bis 2015 ließen sich 8 offene Studien identifizieren, die bestätigten, dass LCIG den Gesamtscore des NMSS über einen Behandlungszeitraum von 6 bis 25 Monaten reduziert, mit spezifischen positiven Effekten auf Schlaf und autonome Dysfunktion und hier besonders auf gastrointestinale Symptome [7], [27], [29], [31], [34], [37], [38], [45]. Neuere Übersichtsarbeiten haben den generell positiven Effekt von LCIG auf nicht motorische Symptome bestätigt [46], [47]. Studien, die in diese Übersichtsarbeiten eingeschlossen wurden, waren neben anderen das GLORIA-Register, das nach 24 Monaten Behandlungsdauer günstige Effekte von LCIG auf Schlafstörungen, Apathie und gastrointestinale Dysfunktion im NMSS nachgewiesen hat [25], und die Interimsanalyse der DUOGLOBE-Studie, die eine generelle Verbesserung des NMSS-Gesamtscores nach 6 Monaten beschrieb [48]. Weitere offene Studien mit einem 6-Monate-Beobachtungszeitraum fanden Verbesserungen im Gesamt-NMSS-Score mit besonderen Effekten in den Domänen kardiovaskuläre Symptome, Aufmerksamkeit/Gedächtnis, urologische Symptome und sonstige Symptome [7], [34]. Nach Daten des GLORIA-Registers kann der Ausgangswert des NMSS die nicht motorische Antwort auf eine LCIG-Behandlung nach 2 Jahren Behandlungsdauer vorhersagen [49].

Bemerkenswert ist, dass LCIG in den EuroInf-Studien im Vergleich mit einer nicht randomisierten Kohorte mit Apomorphin-Infusionen in nahezu allen Domänen größere Effekt zeigte [7], [11].

Begründung der Empfehlung

LCIG hat in 12 offenen longitudinalen Studien nachgewiesenermaßen einen Effekt auf nicht motorische Symptome als sekundären Zielpunkt. Im Label hingegen sind nicht motorische Symptome aber nicht.

**Empfehlung**	**Neu**<br><br>**Stand (2023)**
LCIG kann bei Pat. mit oral nicht zu kontrollierenden motorischen Fluktuationen auch nicht motorische Symptome wie Schlafstörungen, Apathie, gastrointestinale Dysfunktion, kardiovaskuläre Symptome, Aufmerksamkeit/Gedächtnis, urologische Symptome und sonstige Symptome verbessern. Diese Effekte können bei der Auswahl der Pat. für eine LCIG-Behandlung	

als mögliche Determinanten herangezogen werden.
Konsensstärke: 100%, starker Konsens

Fragestellung 167: Wie nachhaltig ist die klinische Symptomkontrolle durch die Levodopa-Carbidopa-Intestinalgel-Pumpentherapie im Vergleich mit der oralen/transdermalen Standard-therapie in der Behandlung der PK mit motorischen Fluktuationen mit und ohne Dyskinesien?

Hintergrund

Eine invasive Therapieform bedeutet für den Pat. eine große Umstellung von der bisherigen oralen Medikation. Sie sollte nach Möglichkeit nachhaltig sein. Im Folgenden soll die langzeitige Wirksamkeit bzw. Sicherheit beleuchtet werden.

Evidenzgrundlage

Für diese Kapitel: In dieser Literaturrecherche fanden sich:

- N=0 randomisierte, kontrollierte Studien
- N=15 longitudinale größere Kohortenstudien.

Ergebnis

Randomisierte kontrollierte Langzeitstudien existieren nicht. Offene Studien mit kumulativ > 600 Pat. über mindestens 12–24 Monate berichten einen stabilen Effekt in Bezug auf Reduktion von Off-Zeit und On-Zeit mit behindernden Dyskinesien ohne deutliche Zunahme von motorischen Fluktuationen über diesen Zeitraum [25], [27], [28], [29], [32], [33], [36], [37], [40], [48], [50].

Die häufigsten Gründe für das Beenden der Behandlung sind rezidivierende PEG-J-Dislokationen [39], [51], die primär bei deliranten oder dementen Pat. auftreten [52]. Etwa 35–50 % der Therapieabbrüche finden im ersten Jahr statt. In einer italienischen Studie von 905 Pat. mit einer mittleren Behandlungsdauer von 6 Jahren fand sich eine Gesamtabbruchrate von 25,7 %, von denen 9,5 % innerhalb des ersten Jahres abbrachen. Die Gründe für den Abbruch im ersten Jahr waren überwiegend fehlende Effektivität auf axiale Symptome (Gangstörung und Stürze) [53]; als weitere Faktoren für den frühen Abbruch wurden sozialmedizinische Faktoren (alleinstehendes Leben) identifiziert [52]. Die häufigsten Gründe für spätes Therapieabbrechen waren PEG-J-assoziiert (Stomainfektionen/Sondendislokationen), Schwierigkeiten in der Bedienung des Systems durch alte Pat./Angehörige und kognitiver Abbau im Rahmen der PK [53].

Begründung der Empfehlung

Die Evidenz beruht auf 15 offenen Langzeitstudien mit einer großen Pat.-Zahl, die einen Zeitraum von mind. 12–24 Monaten abbilden und in denen Behandlungsdauern von bis zu 10 Jahren dokumentiert sind, in denen die Effektivität hinsichtlich der Kontrolle motorischer Fluktuationen beschrieben wird. Kontrollierte Langzeitdaten sind nicht verfügbar.

Empfehlung	Neu<br><br>Stand (2023)
▪ LCIG ist in Bezug auf langfristige Reduktion von motorischen Fluktuationen wirksam und sollte als dauerhafte Behandlung angewandt werden.<br>▪ Die mittlere Behandlungsdauer in großen Langzeitstudien liegt bei 6 Jahren.<br>▪ Vor Behandlungsbeginn soll eine ausführliche Aufklärung hinsichtlich der zu erwartenden Therapieeffekte insbesondere in Bezug auf Nicht-Dopa-responsible Symptome wie Haltungsinstabilität, Stürze und On-Freezing erfolgen.	
Konsensstärke: 94,5%, Konsens	

Referenzen

1. Pessoa RR, Moro A, Munhoz RP, Teive HAG, Lees AJ. Apomorphine in the treatment of Parkinson's disease: a review. Arq Neuropsiquiatr. 2018;76(12):840-8
2. De Cock VC, Dodet P, Leu-Semenescu S, Aerts C, Castelnovo G, Abril B, et al. Safety and efficacy of subcutaneous night-time only apomorphine infusion to treat insomnia in patients with Parkinson's disease (APOMORPHEE): a multicentre, randomised, controlled, double-blind crossover study. Lancet Neurol. 2022;21(5):428-37
3. Katzenschlager R, Poewe W, Rascol O, Trenkwalder C, Deuschl G, Chaudhuri KR, et al. Apomorphine subcutaneous infusion in patients with Parkinson's disease with persistent motor fluctuations (TOLEDO): a multicentre, double-blind, randomised, placebo-controlled trial. Lancet Neurol. 2018;17(9):749-59
4. De Gaspari D, Siri C, Landi A, Cilia R, Bonetti A, Natuzzi F, et al. Clinical and neuropsychological follow up at 12 months in patients with complicated Parkinson's disease treated with subcutaneous apomorphine infusion or deep brain stimulation of the subthalamic nucleus. J Neurol Neurosurg Psychiatry. 2006;77(4):450-3
5. Kanovský P, Kubová D, Bareš M, Hortová H, Streitová H, Rektor I, Znojil V. Levodopa-induced dyskinesias and continuous subcutaneous infusions of apomorphine: results of a two-year, prospective follow-up. Mov Disord. 2002;17(1):188-91
6. Martinez-Martin P, Reddy P, Antonini A, Henriksen T, Katzenschlager R, Odin P, et al. Chronic subcutaneous infusion therapy with apomorphine in advanced Parkinson's disease compared to conventional therapy: a real life study of non motor effect. J Parkinsons Dis. 2011;1(2):197-203
7. Martinez-Martin P, Reddy P, Katzenschlager R, Antonini A, Todorova A, Odin P, et al. EuroInf: a multicenter comparative observational study of apomorphine and levodopa infusion in Parkinson's disease. Mov Disord. 2015;30(4):510-6
8. Pietz K, Hagell P, Odin P. Subcutaneous apomorphine in late stage Parkinson's disease: a long term follow up. J Neurol Neurosurg Psychiatry. 1998;65(5):709-16
9. Stibe CM, Lees AJ, Kempster PA, Stern GM. Subcutaneous apomorphine in parkinsonian on-off oscillations. Lancet. 1988;1(8582):403-6
10. Poewe W, Seppi K, Tanner CM, Halliday GM, Brundin P, Volkmann J, et al. Parkinson disease. Nat Rev Dis Primers. 2017;3:17013
11. Dafsari HS, Martinez-Martin P, Rizos A, Trost M, Dos Santos Ghilardi MG, Reddy P, et al. EuroInf 2: Subthalamic stimulation, apomorphine, and levodopa infusion in Parkinson's disease. Mov Disord. 2019;34(3):353-65
12. Todorova A, Ray Chaudhuri K. Subcutaneous apomorphine and non-motor symptoms in Parkinson's disease. Parkinsonism Relat Disord. 2013;19(12):1073-8
13. Katzenschlager R, Poewe W, Rascol O, Trenkwalder C, Deuschl G, Chaudhuri KR, et al. Long-term safety and efficacy of apomorphine infusion in Parkinson's disease patients with persistent motor fluctuations: Results of the open-label phase of the TOLEDO study. Parkinsonism Relat Disord. 2021;83:79-85
14. Di Rosa AE, Epifanio A, Antonini A, Stocchi F, Martino G, Di Blasi L, et al. Continuous apomorphine infusion and neuropsychiatric disorders: a controlled study in patients with advanced Parkinson's disease. Neurol Sci. 2003;24(3):174-5
15. Katzenschlager R, Hughes A, Evans A, Manson AJ, Hoffman M, Swinn L, et al. Continuous subcutaneous apomorphine therapy improves dyskinesias in Parkinson's disease: a prospective study using single-dose challenges. Mov Disord. 2005;20(2):151-7
16. Olivola E, Fasano A, Varanese S, Lena F, Santilli M, Femiano C, et al. Continuous subcutaneous apomorphine infusion in Parkinson's disease: causes of discontinuation and subsequent treatment strategies. Neurol Sci. 2019;40(9):1917-23

17. Antonini A, Isaias IU, Rodolfi G, Landi A, Natuzzi F, Siri C, Pezzoli G. A 5-year prospective assessment of advanced Parkinson disease patients treated with subcutaneous apomorphine infusion or deep brain stimulation. J Neurol. 2011;258(4):579-85
18. Öthman M, Widman E, Nygren I, Nyholm D. Initial Experience of the Levodopa-Entacapone-Carbidopa Intestinal Gel in Clinical Practice. J Pers Med. 2021;11(4)
19. Senek M, Nyholm D, Nielsen EI. Population pharmacokinetics of levodopa gel infusion in Parkinson's disease: effects of entacapone infusion and genetic polymorphism. Sci Rep. 2020;10(1):18057
20. Nyholm D, Jost WH. Levodopa-entacapone-carbidopa intestinal gel infusion in advanced Parkinson's disease: real-world experience and practical guidance. Ther Adv Neurol Disord. 2022;15:17562864221108018
21. Rosebraugh M, Stodtmann S, Liu W, Facheris MF. Foslevodopa/foscarbidopa subcutaneous infusion maintains equivalent levodopa exposure to levodopa-carbidopa intestinal gel delivered to the jejunum. Parkinsonism Relat Disord. 2022;97:68-72
22. Soileau MJ, Aldred J, Budur K, Fisseha N, Fung VS, Jeong A, et al. Safety and efficacy of continuous subcutaneous foslevodopa-foscarbidopa in patients with advanced Parkinson's disease: a randomised, double-blind, active-controlled, phase 3 trial. Lancet Neurol. 2022;21(12):1099-109
23. Nyholm D, Nilsson Remahl AI, Dizdar N, Constantinescu R, Holmberg B, Jansson R, et al. Duodenal levodopa infusion monotherapy vs oral polypharmacy in advanced Parkinson disease. Neurology. 2005;64(2):216-23
24. Olanow CW, Kieburtz K, Odin P, Espay AJ, Standaert DG, Fernandez HH, et al. Continuous intrajejunal infusion of levodopa-carbidopa intestinal gel for patients with advanced Parkinson's disease: a randomised, controlled, double-blind, double-dummy study. Lancet Neurol. 2014;13(2):141-9
25. Antonini A, Poewe W, Chaudhuri KR, Jech R, Pickut B, Pirtošek Z, et al. Levodopa-carbidopa intestinal gel in advanced Parkinson's: Final results of the GLORIA registry. Parkinsonism Relat Disord. 2017;45:13-20
26. Antonini A, Marano P, Gusmaroli G, Modugno N, Pacchetti C, Sensi M, et al. Long-term effectiveness of levodopa-carbidopa intestinal gel on motor and non-motor symptoms in advanced Parkinson's disease: results of the Italian GLORIA patient population. Neurol Sci. 2020;41(10):2929-37
27. Bohlega S, Abou Al-Shaar H, Alkhairallah T, Al-Ajlan F, Hasan N, Alkahtani K. Levodopa-Carbidopa Intestinal Gel Infusion Therapy in Advanced Parkinson's Disease: Single Middle Eastern Center Experience. Eur Neurol. 2015;74(5-6):227-36
28. Buongiorno M, Antonelli F, Cámara A, Puente V, de Fabregues-Nebot O, Hernandez-Vara J, et al. Long-term response to continuous duodenal infusion of levodopa/carbidopa gel in patients with advanced Parkinson disease: The Barcelona registry. Parkinsonism Relat Disord. 2015;21(8):871-6
29. Cáceres-Redondo MT, Carrillo F, Lama MJ, Huertas-Fernández I, Vargas-González L, Carballo M, Mir P. Long-term levodopa/carbidopa intestinal gel in advanced Parkinson's disease. J Neurol. 2014;261(3):561-9
30. Eggert K, Schrader C, Hahn M, Stamelou M, Rüssmann A, Dengler R, et al. Continuous jejunal levodopa infusion in patients with advanced parkinson disease: practical aspects and outcome of motor and non-motor complications. Clin Neuropharmacol. 2008;31(3):151-66
31. Fasano A, Ricciardi L, Lena F, Bentivoglio AR, Modugno N. Intrajejunal levodopa infusion in advanced Parkinson's disease: long-term effects on motor and non-motor symptoms and impact on patient's and caregiver's quality of life. Eur Rev Med Pharmacol Sci. 2012;16(1):79-89

32. Fernandez HH, Standaert DG, Hauser RA, Lang AE, Fung VS, Klostermann F, et al. Levodopa-carbidopa intestinal gel in advanced Parkinson's disease: final 12-month, open-label results. Mov Disord. 2015;30(4):500-9
33. Foltynie T, Magee C, James C, Webster GJ, Lees AJ, Limousin P. Impact of Duodopa on Quality of Life in Advanced Parkinson's Disease: A UK Case Series. Parkinsons Dis. 2013;2013:362908
34. Honig H, Antonini A, Martinez-Martin P, Forgacs I, Faye GC, Fox T, et al. Intrajejunal levodopa infusion in Parkinson's disease: a pilot multicenter study of effects on nonmotor symptoms and quality of life. Mov Disord. 2009;24(10):1468-74
35. Merola A, Zibetti M, Angrisano S, Rizzi L, Lanotte M, Lopiano L. Comparison of subthalamic nucleus deep brain stimulation and Duodopa in the treatment of advanced Parkinson's disease. Mov Disord. 2011;26(4):664-70
36. Pålhagen SE, Dizdar N, Hauge T, Holmberg B, Jansson R, Linder J, et al. Interim analysis of long-term intraduodenal levodopa infusion in advanced Parkinson disease. Acta Neurol Scand. 2012;126(6):e29-33
37. Reddy P, Martinez-Martin P, Rizos A, Martin A, Faye GC, Forgacs I, et al. Intrajejunal levodopa versus conventional therapy in Parkinson disease: motor and nonmotor effects. Clin Neuropharmacol. 2012;35(5):205-7
38. Sensi M, Preda F, Trevisani L, Contini E, Gragnaniello D, Capone JG, et al. Emerging issues on selection criteria of levodopa carbidopa infusion therapy: considerations on outcome of 28 consecutive patients. J Neural Transm (Vienna). 2014;121(6):633-42
39. Zibetti M, Merola A, Artusi CA, Rizzi L, Angrisano S, Reggio D, et al. Levodopa/carbidopa intestinal gel infusion in advanced Parkinson's disease: a 7-year experience. Eur J Neurol. 2014;21(2):312-8
40. Poewe W, Bergmann L, Kukreja P, Robieson WZ, Antonini A. Levodopa-Carbidopa Intestinal Gel Monotherapy: GLORIA Registry Demographics, Efficacy, and Safety. J Parkinsons Dis. 2019;9(3):531-41
41. Pauls KAM, Toppila J, Koivu M, Eerola-Rautio J, Udd M, Pekkonen E. Polyneuropathy monitoring in Parkinson's disease patients treated with levodopa/carbidopa intestinal gel. Brain Behav. 2021;11(12):e2408
42. Taher J, Naranian T, Poon YY, Merola A, Mestre T, Suchowersky O, et al. Vitamins and Infusion of Levodopa-Carbidopa Intestinal Gel. Can J Neurol Sci. 2022;49(1):19-28
43. Müller T, van Laar T, Cornblath DR, Odin P, Klostermann F, Grandas FJ, et al. Peripheral neuropathy in Parkinson's disease: levodopa exposure and implications for duodenal delivery. Parkinsonism Relat Disord. 2013;19(5):501-7 ; discussion
44. Loens S, Chorbadzhieva E, Kleimann A, Dressler D, Schrader C. Effects of levodopa/carbidopa intestinal gel versus oral levodopa/carbidopa on B vitamin levels and neuropathy. Brain Behav. 2017;7(5):e00698
45. Antonini A, Yegin A, Preda C, Bergmann L, Poewe W. Global long-term study on motor and non-motor symptoms and safety of levodopa-carbidopa intestinal gel in routine care of advanced Parkinson's disease patients; 12-month interim outcomes. Parkinsonism Relat Disord. 2015;21(3):231-5
46. Antonini A, Odin P, Pahwa R, Aldred J, Alobaidi A, Jalundhwala YJ, et al. The Long-Term Impact of Levodopa/Carbidopa Intestinal Gel on 'Off'-time in Patients with Advanced Parkinson's Disease: A Systematic Review. Adv Ther. 2021;38(6):2854-90
47. Prakash N, Simuni T. Infusion Therapies for Parkinson's Disease. Curr Neurol Neurosci Rep. 2020;20(9):44
48. Aldred J, Anca-Herschkovitsch M, Antonini A, Bajenaru O, Bergmann L, Bourgeois P, et al. Application of the '5-2-1' screening criteria in advanced Parkinson's disease: interim analysis of DUOGLOBE. Neurodegener Dis Manag. 2020;10(5):309-23
49. Ray Chaudhuri K, Antonini A, Robieson WZ, Sanchez-Soliño O, Bergmann L, Poewe W. Burden of non-motor symptoms in Parkinson's disease patients predicts improvement in quality of

life during treatment with levodopa-carbidopa intestinal gel. Eur J Neurol. 2019;26(4):581-e43

50. Slevin JT, Fernandez HH, Zadikoff C, Hall C, Eaton S, Dubow J, et al. Long-term safety and maintenance of efficacy of levodopa-carbidopa intestinal gel: an open-label extension of the double-blind pivotal study in advanced Parkinson's disease patients. J Parkinsons Dis. 2015;5(1):165-74
51. Nyholm D, Klangemo K, Johansson A. Levodopa/carbidopa intestinal gel infusion long-term therapy in advanced Parkinson's disease. Eur J Neurol. 2012;19(8):1079-85
52. Viljaharju V, Mertsalmi T, Pauls KAM, Koivu M, Eerola-Rautio J, Udd M, Pekkonen E. Single-Center Study of 103 Consecutive Parkinson's Disease Patients with Levodopa-Carbidopa Intestinal Gel. Mov Disord Clin Pract. 2022;9(1):60-8
53. Sensi M, Cossu G, Mancini F, Pilleri M, Zibetti M, Modugno N, et al. Which patients discontinue? Issues on Levodopa/carbidopa intestinal gel treatment: Italian multicentre survey of 905 patients with long-term follow-up. Parkinsonism Relat Disord. 2017;38:90-2

3.20 Tiefe Hirnstimulation

Autoren/Autorinnen: René Reese, Paul Krack, Andrea Kühn, Alfons Schnitzler, Alexander Storch

Die Tiefe Hirnstimulation (THS) ist ein klinisch etabliertes und von den gesetzlichen Krankenkassen zugelassenes invasiv-chirurgisches Verfahren zur symptomatischen Behandlung von Pat. mit der PK, die an konservativ-medikamentös-therapierefraktären motorischen Fluktuationen, Dyskinesien und/oder einem Tremor leiden. Es sind drei Zielpunkte zur Implantation von Elektroden zur THS definiert: Nucleus subthalamicus (STN), Globus pallidus internus (GPi), Ncl. ventro-intermedius thalami / posteriore subthalamische Area (VIM). Deren Auswahl ist individuell u.a. davon abhängig, welche Zielsymptome vornehmlich durch die Therapie adressiert werden sollen und welche spezifischen Kontraindikationen es möglicherweise für den einen oder den anderen Zielpunkt gibt.

Es werden sieben Fragestellungen zur THS bei der PK bearbeitet, deren Antworten Hilfestellungen für die wichtigsten klinischen Entscheidungsprozesse in der Anwendung der Therapie der THS bei der PK geben sollen.

Fragestellung 168: Wie effektiv und sicher ist die THS (STN, GPi, VIM) im Vergleich mit der oralen/transdermalen Standardtherapie in der Behandlung der PK mit motorischen Fluktuationen mit und ohne Dyskinesien?

Hintergrund

Hier soll beantwortet werden, wie effektiv und sicher die THS der genannten Zielpunkte jeweils im Vergleich mit der oralen/transdermalen Standardtherapie in der Behandlung der Parkinson-Krankheit mit motorischen Fluktuationen mit und ohne Dyskinesien sind, welche Pat.-Klientel in der präoperativen Auswahl für diese Therapie vorgesehen werden kann und welche Ausschlusskriterien es möglicherweise gibt.

Evidenzgrundlage

Große randomisierte und kontrollierte Studien existieren nur für den Vergleich der STN-THS mit der oralen/transdermalen dopaminergen Substitutionstherapie, es wurden derer sieben identifiziert [1], [2], [3], [4], [5], [6], [7]. Eine dieser Studien [3] untersuchte die Zielpunkte STN und GPi, ohne diese in der Datenauswertung zu trennen, sodass die dort beschriebenen Effekte nicht einem Zielpunkt zugeordnet werden können und diese Studie in die weitere Bewertung der Fragestellung nicht einfließt. Ähnlich verhält es sich mit einer weiteren Studie [4], wobei dort nicht hälftig zwischen GPi und STN aufgeteilt wurde, sondern von 178 Pat. nur 4 eine GPi-THS erhielten.

Für den GPi als Zielpunkt gibt es nur Vergleichsstudien mit dem STN [8], [9], sodass die Bewertung in der Frage 169 dieser Leitlinie zu beantworten ist.

Für den VIM als Zielpunkt konnte keine Studie identifiziert werden, die gegen die medikamentöse Therapie getestet hätte.

Ergebnis

In den vergleichenden Studien von STN-THS gegen die orale/transdermale Substitutionstherapie ergaben sich jeweils relevante Verbesserungen in der Gruppe der STN-THS für die Domänen „Aktivitäten des täglichen Lebens“ und „Lebensqualität“, einhergehend mit einer relevanten Verbesserung der motorischen Parkinson-Symptome inklusive motorischer Fluktuationen und Dyskinesien. Dies drückte sich auch in einer Erhöhung der täglichen Zeit guter Beweglichkeit („On-Zeit“) und einer reduzierten täglichen Zeit schlechter Beweglichkeit (Off-Zeit) aus. Kognitions- und Depressionsskalen blieben ohne Unterschied zwischen den Gruppen.

Als Einschlusskriterien (und damit prinzipielle Indikationsstellung einer THS) werden konservativ-medikamentös nicht ausreichend behandelbare motorische Fluktuationen/medikamentös induzierte Dyskinesien geführt, einhergehend mit einer relevanten Verbesserung der motorischen Parkinson-Symptome durch einen standardisierten Levodopa-Test (mindestens 33% Verbesserung) [1]. Zwei Studien untersuchten aber zusätzlich die Frage, ob Pat. mit motorischen Fluktuationen mit und ohne Dyskinesien von weniger als drei Jahren Dauer, mindestens 50%iger Besserung der motorischen Parkinson-Symptome durch einen standardisierten Levodopa-Test und jünger als 60 Jahre bei mindestens 4-jähriger Krankheitsdauer ebenso von der THS profitieren [2], [6]. Insgesamt waren die Effektgrößen (Lebensqualität, Aktivitäten des täglichen Lebens, motorische Symptome, tägliche On-Zeit ohne Dyskinesien, tägliche Off-Zeit) ähnlich wie in den übrigen Studien von Pat. mit fortgeschrittenerer Erkrankung (im Mittel älter als 60 Jahre mit >10 Jahren Erkrankungsdauer). Schlechtere Ergebnisse in Lebensqualitätsskalen präoperativ waren in dieser kürzer von der PK betroffenen Kohorte ein Prädiktor für ein gutes postoperatives Ergebnis [10]. Die tägliche Dosis der Parkinson-Medikamente konnte reduziert werden. Kognitions- und Apathieskalen blieben unbeeinflusst und es gab einen möglichen positiven Einfluss auf Depressionen.

Bezüglich einer möglichen Prädiktion der postoperativen Verbesserungen nach STN-THS ist gezeigt worden, dass präoperativ niedrigere relative Verbesserungen durch Levodopa mit einem formal schlechteren Ergebnis der THS einhergehen [11]. Erfahrungsgemäß lassen sich Levodopa-responsive Symptome der PK ebenso mit der STN-THS lindern, gleichlautend werden Symptome ohne Besserung durch Levodopa auch durch die STN-THS gemeinhin nicht erreicht. Als Ausnahme ist hier der Parkinson-Tremor zu nennen, der durch die STN-THS meist besser behandelt ist als durch die Levodopa-Medikation (s. hierzu die jeweiligen Kapitel in dieser Leitlinie). Bezüglich des Alters zum Zeitpunkt der Operation waren Pat. jenseits des 70. Lebensjahrs von der Studienteilnahme meist ausgeschlossen, aber ein eindeutiger Effekt des Alters auf das postoperative Ergebnis konnte nicht dargestellt werden. In dieser Frage wäre sicher auch das biologische Alter im konkreten Fall jeweils mit in die Abwägung einer Therapiempfehlung einzubeziehen. Übrige Ausschlusskriterien einer Demenz (Mattis Score >130), floriden Psychose/Halluzinationen, Suizidalität und Depression müssen zusätzlich ebenso bedacht werden wie neurochirurgische Kontraindikationen. Schwerwiegende Zwischenfälle von Suizidgedanken oder vollendeten Suiziden wurden insgesamt häufiger beschrieben als in der Normalbevölkerung erwartbar [12], [13], waren aber nicht unterschiedlich zwischen den Behandlungsgruppen [12], sodass dies möglicherweise kein Effekt der Behandlung an sich ist. Bezüglich der in den Studien sonstig geführten prozeduralen schweren Zwischenfälle (severe adverse

events, SAE) waren in der Gruppe der THS nominal mehr zu verzeichnen (etwa 25%) als in der Medikamentengruppe (etwa 11%), von denen die meisten aber jeweils reversibel waren.

Begründung der Empfehlung

Die Evidenz der Empfehlung ist aufgrund der Anzahl der identifizierten randomisierten und kontrollierten Studien zum Vergleich STN-THS mit der konservativ-medikamentösen Therapie insgesamt hoch. Für die GPi-THS und die VIM-THS existieren keine größeren kontrollierten Studien, die einen Vergleich mit der medikamentösen Therapie beschreiben.

Empfehlung	Modifiziert<br><br>Stand (2023)
▪ Die STN-THS soll unter Beachtung der Kontraindikationen Pat. mit einer PK mit konservativ-medikamentös nicht ausreichend behandelbaren motorischen Fluktuationen mit und ohne Dyskinesien und mindestens 33%iger Verbesserung der motorischen Parkinson-Symptome durch einen standardisierten Levodopa-Test angeboten werden.<br>▪ Die STN-THS sollte ebenso Pat. mit einer PK und mindestens 4-jähriger Krankheitsdauer, mit motorischen Fluktuationen mit und ohne Dyskinesien von weniger als 3 Jahren Dauer, mindestens 50%iger Besserung der motorischen Parkinson-Symptome durch einen standardisierten Levodopa-Test und jünger als 60 Jahre angeboten werden.<br>▪ Die THS ist mit einem operativen Eingriff und damit besonderen Risiken verbunden, die gegen den Gewinn durch die Therapie individuell abgewogen werden müssen.	
Konsensstärke: 96,2%, starker Konsens	

Fragestellung 169: Welches ist die effektivste Form der THS (STN, GPi, VIM) in der Behandlung der PK mit motorischen Fluktuationen mit und ohne Dyskinesien?

Hintergrund

Prinzipiell sind in der Literatur drei Zielpunkte zur Behandlung der PK definiert, wobei der STN der in der klinischen Routine wohl der am häufigsten verwendete ist. Hier soll geklärt werden, ob bzw. welche(r) der Zielpunkte eine klinische Überlegenheit in der Behandlung der PK mit motorischen Fluktuationen mit und ohne Dyskinesien hat.

Evidenzgrundlage

Randomisierte und kontrollierte Studien, die einen Vergleich der Wirksamkeit zwischen den Zielpunkten erlauben, lassen sich nur für die STN-THS gegen die GPi-THS identifizieren [8], [9].

Ergebnis

Für beide Studien [8], [9] wurde 1 : 1 zwischen den Zielpunkten randomisiert und 299 bzw. 125 Pat. eingeschlossen und 2 Jahre bzw. 1 Jahr nachbeobachtet.

Für die niederländische Studie (NSTAPS-Studie; [9]) wurden die primären Endpunkte „Aktivitäten des täglichen Lebens“ (ADL; gewichtet nach medikamentösem Off und On) und ein kombinierter Score

für Kognition, Stimmung und Verhalten definiert. Beide waren nach 12 Monaten nicht unterschiedlich zwischen den Gruppen. Aus den sekundären Endpunkten wird jedoch ersichtlich, dass die STN-THS in dieser Studie möglicherweise wirkstärker als die GPi-THS war. Für die Unterschiede im medikamentösen Off (also nur THS) ergaben sich eine signifikant bessere (und klinisch relevante) Reduktion motorischer Parkinson-Symptome und Verbesserung der ADL durch die STN-THS. Im medikamentösen On ergab sich für die GPi-THS eine prinzipiell bessere Kontrolle von Dyskinesien, für die weiteren sekundären Endpunkte wie tägliche Zeit guter Beweglichkeit (On-Zeit) ohne behindernde Dyskinesien ergaben sich jeweils keine Unterschiede.

In der CSP486-Studie [8] war der primäre Endpunkt als Veränderung der motorischen Funktionen (UPDRS III) durch die jeweilige THS ohne medikamentöse Therapie nach 24 Monaten definiert. Dieser Endpunkt war nicht unterschiedlich zwischen den beiden Behandlungsgruppen. Ebenso waren die sekundären Endpunkte Selbsteinschätzung von Alltagsfunktionen, Lebensqualität, kognitive Funktionen und Nebenwirkungen der THS ohne Unterschiede. In dieser Studie war jedoch eine im Vergleich mit anderen STN-Studien (jeweils etwa 50% Verbesserung; [14]) relativ geringe Verbesserung der motorischen Symptome in der STN-Gruppe von etwa 26% zu verzeichnen. Die Gründe könnten in der Pat.-Klientel mit einem möglicherweise präoperativ relativ niedrigen Grad der Verbesserung der motorischen Parkinson-Symptome durch dopaminerge Medikation liegen [11] oder auch in der Verblindung der Zielpunkte für die Behandelnden in der postoperativen Nachsorge. Für die STN-THS sind erst höhere Stimulationsstärken (und damit höhere Wirkstärken) zu erreichen, wenn die dopaminerge Medikation reduziert wird. So fand sich in der NSTAPS-Studie 43% und in der CSP486-Studie 31% Reduktion der dopaminergen Medikation in der STN-Gruppe. In jedem Fall erscheint von klinischer Relevanz die in den STN-Gruppen jeweils deutlichere Reduktion der dopaminergen Substitution, einhergehend mit einem stärkeren Anstieg der täglichen Dosis dopaminerger Medikamente schon im ersten postoperativen Jahr in der GPi-Gruppe. Komplikationen bei der chirurgischen Prozedur oder der Therapie waren zwischen den Zielpunkten in den Studien nicht unterschiedlich.

Zur Behandlung der PK mit motorischen Fluktuationen und Dyskinesien hat der Thalamus (VIM) als Zielpunkt keine Relevanz, da hier die diesbezüglichen Effektstärken nur aus unkontrollierten Studien ablesbar [15], [16], [17], [18], [19] sind und die Symptome Akinese, Rigor und Dyskinesien nicht gebessert werden.

Begründung der Empfehlung

Die Evidenz begründet sich in zwei randomisierten und kontrollierten Studien, die die Wirksamkeit der Zielpunkte STN und GPi zur Behandlung der PK mittels der THS miteinander verglichen haben [8], [9]. Unterschiede in der Wirksamkeit der Zielpunkte waren nur in einer Studie und auch nur im medikamentösen Off/THS On mit einer Reduktion motorischer Parkinson-Symptome und Verbesserung der ADL zu verzeichnen (NSTAPS-Studie; [9]). Von klinischer Relevanz ist sicherlich die deutlichere Reduktion dopaminerger Substitution bei den mit einer STN-THS behandelten Pat. in beiden Studien.

Empfehlung	Neu<br>Stand (2023)
▪ Die STN-THS sollte der GPi-THS in den differenzialtherapeutischen Abwägungen bei der PK mit motorischen Fluktuationen mit und ohne Dyskinesien vorgezogen werden.<br>▪ Eine VIM-THS soll in der Behandlung der PK mit motorischen Fluktuationen mit und ohne Dyskinesien nicht eingesetzt werden.<br>▪ Die THS ist mit einem operativen Eingriff und damit besonderen Risiken verbunden, die gegen den Gewinn durch die Therapie individuell abgewogen werden müssen.	
Konsensstärke: 100%, starker Konsens	

Fragestellung 170: Wie effektiv und sicher ist die THS (STN, GPi, VIM) im Vergleich mit der oralen/transdermalen Standardtherapie in der Behandlung der PK mit pharmakoresistentem Tremor?

Hintergrund

Als Parkinson-Tremor wird jede pathologische Tremor-Form bei Pat. mit einer PK klassifiziert. Der Ruhetremor ist eines der motorischen Kardinalsymptome der PK und tritt im Verlauf der Erkrankung bei etwa ¾ der Pat. auf. Viele Parkinson-Pat. leiden darüber hinaus unter einem Aktionstremor, entweder in Form eines höherfrequenten Halte- und Bewegungstremors oder in Form eines kurz nach Übergang von Ruhe in Aktion auftretenden Tremors in Ruhetremorfrequenz. Die THS ist ein etabliertes Verfahren zur Behandlung von Pat. mit der PK, die medikamentös nicht behandelbare motorische Fluktuationen mit und ohne Dyskinesien oder einen medikamentös nicht kontrollierbaren Tremor haben (siehe auch LL „Tremor“ [67]). Bei der überwiegenden Zahl der Pat. wird die THS im STN vorgenommen. Als alternative anatomische Zielpunkte kommen in der Parkinson-Therapie der VIM oder der GPi in Frage.

Evidenzgrundlage

Randomisierte, kontrollierte Studien zum Vergleich der THS mit der besten medikamentösen Behandlung (best medical treatment, BMT) liegen nur für die STN-THS vor. Es wurden 6 große RCTs [1], [4], [6], [5], [3], [7] und eine kleinere Pilot-RCT [2] identifiziert. Nur 2 RCTs [4], [2] enthalten Angaben über die spezifische Wirksamkeit in Bezug auf den Parkinson-Tremor. Studien zur Wirksamkeit von GPi-THS im Vergleich zu BMT bei pharmakoresistentem Parkinson-Tremor existieren nicht. Die Wirksamkeit der VIM-THS bei pharmakoresistentem Parkinson-Tremor ist nur in unkontrollierten Studien untersucht worden.

Ergebnis

Die großen RCTs zeigen, dass die STN-THS im Vergleich zu BMT zu einer Verbesserung der Lebensqualität und der Aktivitäten des täglichen Lebens, einem Rückgang der motorischen Beeinträchtigung, Besserung der Dyskinesien und Zunahme der täglichen On-Zeit sowie Reduktion der Off-Zeit führt. Kognition und Depression scheinen nicht wesentlich beeinflusst. Schwerwiegende

Nebenwirkungen waren in der chirurgisch behandelten Pat.-Gruppe (25%) häufiger als in der BMT-Gruppe (11%). Die meisten operationsbedingten Nebenwirkungen waren reversibel. Pat., die sich für eine THS entscheiden, haben mehr Suizidgedanken und Suizide als die Allgemeinbevölkerung [13], aber die Raten in der behandelten Gruppe unterscheiden sich nicht von denen der Vergleichsgruppe der BMT [12], was darauf hindeutet, dass dies keine Wirkung der Behandlung ist. Nur 2 RCTs enthalten Angaben über die spezifische Wirksamkeit in Bezug auf den Parkinson-Tremor.

In der PD-SURG-Studie [4] zeigte sich in einer Subgruppenanalyse von Pat., bei denen die Indikation der STN-THS vor allem aufgrund des Parkinson-Tremors gestellt wurde (jeweils 40% der Studienpopulation), ein signifikanter Effekt mit einer Reduktion der mittleren Punktzahl im PDQ-39 um 4,9±1,6 in der Gruppe der Pat. mit STN-THS und BMT im Vergleich zu einer Reduktion der mittleren Punktzahl um 0,8±1,3 in der Gruppe der Pat. mit BMT [4].

In der Pilot-RCT [2] von 20 Pat. mit der PK mit einer Krankheitsdauer von durchschnittlich 7 Jahren wurde eine signifikante Besserung des Tremors nach 6, 12 und 18 Monaten unter STN-THS im Vergleich zu BMT berichtet.

In einer prospektiven, nicht randomisierten und nicht verblindeten Studie [20] wurde die Effektivität der STN-THS auf den Tremor bei 15 Pat. mit einer PK vom Äquivalenztyp mit motorischen Fluktuationen untersucht. Der Ruhetremor verbesserte sich durchschnittlich um 82% und der Aktionstremor um 78% bei Beurteilung nach 1 bis 12 Monaten.

Bei den meisten der unkontrollierten Langzeitstudien zur Untersuchung der Effekte der STN-THS auf die Parkinson-Symptome ist der Effekt auf den Ruhetremor aufgeführt. Beim Vergleich des postoperativen versus des präoperativen Off-Zustands wird eine mittlere Verbesserung in Studien über 5 Jahre von 78,3 % und in Studien über 8 bis 10 Jahre von 83,7% angegeben.

Studien zur Wirksamkeit von GPi-THS im Vergleich zu BMT auf den Parkinson-Tremor existieren nicht.

Die Wirksamkeit der VIM-THS bei pharmakoresistentem Parkinson-Tremor ist nur in unkontrollierten Studien untersucht worden. Es gibt fünf Studien [15], [16], [17], [18], [19] mit Nachbeobachtungszeiten von bis zu 13 Monaten. In allen fünf Studien wurde die VIM-THS als effektiv in Bezug auf die Kontrolle des Parkinson-Tremors beurteilt bei jeweils signifikanten Verbesserungen des Tremors unter Stimulation. Eine vollständige Kontrolle des Tremors wurde bei 31,6% bis 88,5% der Parkinson-Pat. beschrieben, während bei 0% bis 4,2% kein Effekt auf den Tremor beobachtet wurde. Anhaltende Effekte der Tremor-Kontrolle über 12 Monate wurden beobachtet [17], [19].

In 4 Studien wurde die Wirksamkeit der VIM-THS auf den Parkinson-Tremor ab 1 Jahr bis zu 21 Jahre nachbeobachtet [21], [22], [23], [24].

Alle Studien zeigten eine signifikante Verbesserung des Parkinson-Tremors auf der stimulierten Körperseite unter VIM-THS mit anhaltenden Effekten bis zu 21 Jahre.

Bei unilateraler THS wurde eine Verbesserung des Tremors um 67% nach 1 Jahr [24], um 85% nach 5 Jahren [22], um 58% nach 11–15 Jahren [24] sowie um 63% nach 16-21 Jahren [24] beschrieben. Bei bilateraler THS wurde eine Verbesserung des Tremors um 73% nach 1 Jahr [24], um 71% nach 2

Jahren [21], um 64% nach 6-7 Jahren [21], um 69% nach 11–15 Jahren [24] und um 60% nach 16–21 Jahren [24] berichtet.

Unter bilateraler VIM-THS wurde eine signifikante Verbesserung anderer motorischer Symptome beschrieben. So wurde eine zusätzlich signifikante mäßige Verbesserung der Akinesie bzw. der Rigidität nach 3 Monaten (um je 36%) und nach 1 Jahr (um 34% bzw. 16%) berichtet [19]. Hier muss bei einer offenen Bewertung zwischen THS On und THS Off ein Placebo-Effekt mit in Betracht gezogen werden. Es wurde eine signifikante Verbesserung der motorischen Symptomatik (UPDRS Teil III) um 28% nach 1 Jahr, um 15% nach 6 Jahren und um 44% nach 10 Jahren, jeweils nur unter THS On, beschrieben. [23], [24]. Allerdings wurde eine relevante Verbesserung der funktionellen Behinderung bei den Alltagsaktivitäten nur nach 1 Jahr, nicht aber nach 5–6 Jahren im Vergleich zu vor THS beschrieben [23]. Daher wurde die VIM-THS bei Parkinson-Pat. nur als sinnvoll erachtet, wenn der Tremor zu einer relevanten funktionellen Behinderung beiträgt. Hiermit vereinbar wurde in einer Studie berichtet, dass sich die Levodopa-Äquivalenzdosis pro Tag (LEDD) nach 1 Jahr unter VIM-THS nicht ändert [24].

Unerwünschte Wirkungen der VIM-THS wurden mehrheitlich als mild und stimulationsassoziiert mit Verbesserung nach Anpassung der THS-Parameter beschrieben. Diese traten vorwiegend im ersten postoperativen Jahr während der THS-Programmierung auf. Hierzu gehörten Dysästhesien (bis 45%), Schmerzen (bis 41%), das Auftreten einer Dysarthrie (bis 75%), Gang- und Gleichgewichtsstörungen (bis 93%) und eine Dystonie (bis 0,9%). Operations- bzw. geräteassoziierte Komplikationen traten insgesamt seltener und ebenfalls vorwiegend im ersten postoperativen Jahr auf. Hierzu gehörten intrazerebrale Blutungen (bis 1,9%), extrazerebrale Blutungen (bis 4%), Infektionen (bis 3,8%) und Hardware-Probleme (bis 3,78%). Auch chirurgische Revisionen wurden beschrieben (bis 27%). Eine operationsassoziierte Mortalität trat nicht auf, was aber an der im Vergleich zur STN-Stimulation deutlich geringeren Zahl publizierter Fälle liegen könnte.

Die VIM-THS führt bei Parkinson-Pat. mit Tremor zu einer signifikanten Tremor-Reduktion mit anhaltenden Effekten von bis zu 21 Jahren. Da es nur im ersten postoperativen Jahr zu einer relevanten Verbesserung der funktionellen Behinderung bei den Alltagsaktivitäten kommt und ein Langzeiteffekt auf den Parkinson-Tremor nicht durch prospektive Studien belegt ist, kommen für eine VIM-THS nur Pat. mit der PK mit funktionell behinderndem Tremor in Frage.

Begründung der Empfehlung

Die Evidenz beruht auf 2 randomisierten und kontrollierten Studien für die Wirksamkeit der STN-THS im Vergleich zu BMT und auf unkontrollierten Beobachtungsstudien für die Wirksamkeit der VIM-THS auf den Parkinson-Tremor.

Empfehlung	Modifiziert<br><br>Stand (2023)
▪ Die STN-THS soll unter Beachtung der Kontraindikationen Pat. mit einer PK mit schwerem, konservativ medikamentös nicht ausreichend behandelbarem Tremor angeboten werden und vorzugsweise bilateral erfolgen.<br>▪ Die uni- oder bilaterale VIM-THS und die GPi-THS sind bei medikamentös nicht kontrollierbarem Parkinson-Tremor wirksam und können bei Kontraindikationen für eine STN-THS erwogen werden.<br>▪ Die Therapie ist mit einem operativen Eingriff und damit besonderen Risiken verbunden, die gegen den Gewinn durch die Therapie individuell abgewogen werden müssen.	
Konsensstärke: 92,3%, Konsens	

Fragestellung 171: Welches ist die effektivste Form der THS (STN, GPi, VIM) in der Behandlung der PK mit pharmakoresistentem Tremor?

Hintergrund

Die THS ist ein etabliertes Verfahren zur Behandlung von Parkinson-Pat., die medikamentös nicht behandelbare motorische Fluktuationen mit Dyskinesien und/oder einen medikamentös nicht kontrollierbaren Tremor haben (siehe auch LL „Tremor“). Bei der überwiegenden Zahl der Pat. wird die THS im STN vorgenommen. Als alternative anatomische Zielpunkte kommen der GPi oder der VIM infrage.

Evidenzgrundlage

Ein systematischer Review mit Metaanalyse [25] hat die Wirksamkeit von STN-THS und GPi-THS in der Behandlung des Parkinson-Tremors miteinander verglichen. Dabei wurden 5 RCTs berücksichtigt [26], [9], [27], [28], [29].

Randomisierte, kontrollierte Studien zum Vergleich der Wirksamkeit der STN-THS gegenüber der VIM-THS existieren nicht.

Ergebnis

In einer Metaanalyse [25] wurde bei 489 Pat. aus 5 randomisierten Studien [26], [9], [27], [28], [29] die Wirksamkeit der STN-THS (N=263) und der GPi-THS (N=226) auf den Parkinson-Tremor über eine Beobachtungsdauer von bis zu 60 Monaten analysiert. Bei 2 RCTs [26], [9] wurden über die Hauptautoren/Hauptautorinnen zusätzliche unpublizierte Daten eingeholt und in die Analyse mit einbezogen. Die 5 RCTs wurden einer Random-effects-model-Metaanalyse unterzogen.

Über eine Moderatorenvariablenanalyse wurde überprüft, ob ein Unterschied zwischen den Behandlungseffekten der STN-THS und der GPi-THS vorliegt. Im Gesamtvergleich THS On zu THS Off zeigte sich eine signifikante standardisierte Gesamtdifferenz mit einer mittleren Effektstärke von 0,36 als Hinweis darauf, dass die THS den Parkinson-Tremor mit einer mittleren Effektstärke

reduziert. Die Analyse der Moderatorvariablen STN-THS versus GPI-THS ergab zwei signifikante standardisierte Effektgrößen für STN-THS (0,38) und GPi-THS (0,35), die nicht signifikant unterschiedlich waren, auch wenn in allen 5 Studien die prozentuale Tremor-supprimierende Wirkung bei der STN-THS tendenziell etwas stärker war als bei der GPi-THS.

Die Ergebnisse der Metaanalyse zeigen somit, dass keine statistisch signifikanten Unterschiede zwischen den beiden Zielen in der langfristigen Verbesserung des Parkinson-Tremors bestehen.

Randomisierte, kontrollierte Studien zum Vergleich der Wirksamkeit der STN-THS gegenüber der VIM-THS existieren nicht. Die Wirksamkeit der VIM-THS bei pharmakoresistentem Tremor ist nur in unkontrollierten Studien untersucht worden (siehe hierzu Frage 170).

Begründung der Empfehlung

In Bezug auf einen Parkinson-Tremor sind STN-THS und GPi-THS nach den Ergebnissen eines systematischen Reviews mit einer Metaanalyse von 5 RCTs als nicht unterschiedlich wirksam anzusehen. Die Wahl des Zielgebiets wird daher durch andere Faktoren als durch das Ansprechen auf den Tremor bestimmt. Hierzu zählen die mit der STN-THS einhergehende und klinisch erwünschte Reduktion der dopaminergen Medikation (siehe Frage 169), das individuelle motorische und nicht motorische Symptomprofil sowie die weitgehend fehlende Wirksamkeit der VIM-THS auf andere motorische Parkinson-Symptome außer Tremor.

**Empfehlung**	**Neu**<br>**Stand (2023)**
▪ STN-THS und GPi-THS sind gleichwertig wirksam in der Behandlung des pharmakoresistenten Parkinson-Tremors. Die Wahl des Zielgebiets bei Pat. mit der PK und medikamentös nicht kontrollierbarem Parkinson-Tremor soll daher unter Berücksichtigung des Weiteren individuellen Symptomprofils erfolgen (siehe Frage 169).<br>▪ Die uni- oder bilaterale VIM-THS ist bei medikamentös nicht kontrollierbarem Parkinson-Tremor wirksam und kann bei Kontraindikationen für eine STN-THS oder GPi-THS erwogen werden.	
Konsensstärke: 96,2%, starker Konsens	

Fragestellung 172: Wie effektiv ist die THS (STN, GPi) in der Behandlung der nicht motorischen Parkinson-Symptome und von deren Fluktuationen (Schlaf, Schmerz, autonome Symptome (übermäßiges Schwitzen, Dysurie, gastrointestinale Symptome, orthostatische Hypotonie), neuropsychiatrische Symptome (Apathie, Depression, Angst, Impulskontrollstörung, Punding, Dopamindysregulationssyndrom) im Vergleich mit der oralen/transdermalen Standardtherapie in der Behandlung der PK?

Hintergrund

Neuropsychiatrische Fluktuationen bei der PK sind häufig und behindernd. Sie treten fast systematisch parallel zu den motorischen Fluktuationen auf, können isoliert oder zumindest im

Vordergrund der Beschwerden auftauchen. Neben der Motorik und Kognition fluktuieren auch autonome Symptome sowie sensorische/Schmerzsymptome [30], [31]. In der Routine werden neuropsychiatrische Fluktuationen trotz ihrer Relevanz jedoch kaum erfasst, da es nur wenige geeignete Messinstrumente gibt. Bei fortgeschrittener PK mit motorischen Komplikationen ist die THS eine Therapieoption. Die am häufigsten durchgeführte Therapie ist die STN-THS [32], [33]. Verschiedene Studien konnten in einem THS-On-Off-Design direkte psychotrope Effekte [34] sowie Effekte auf Schmerzempfindung [35], Magenentleerung [36] und Blasenfunktion [37], [38] zeigen. Eine weitere Studie, ebenfalls mit einem THS-On-Off-Design, zeigte keine wesentlichen direkten Effekte der STN-THS auf die Frequenz der meisten nicht motorischen Symptome, wobei lediglich die Frequenz von innerer Unruhe signifikant durch STN-THS reduziert wurde. Die Schwere der meisten nicht motorischen Symptome wurde in dieser Studie durch die STN-THS gelindert, wobei nur die Schwere von exzessivem Schwitzen, Schmerz und Schwindel unverändert blieb [39]. Es konnten keine demographischen oder klinischen Prädiktoren für die Wirksamkeit der STN-THS detektiert werden. Es stellt sich daher die Frage, ob die chronische STN-THS im Vergleich zur medikamentösen Therapie Effekte auf nicht motorische Symptome zeigt, ein bislang relativ wenig untersuchtes Feld.

Evidenzgrundlage

Zur besseren Übersicht wurde die Literaturrecherche in folgende Unterkapitel eingeteilt:

2. **Depression**

   Die erhältlichen randomisierten kontrollierten Studien zum Vergleich von STN-THS gegen medikamentöse Therapie [1], [3], [4], [5], [7] zeigen, dass die STN-THS die Depression wahrscheinlich weder bessert noch verschlechtert [33]. Pat. unter STN-THS haben laut einer Metaanalyse vermehrt suizidale Ideen und es kommt häufiger zu Suiziden im Vergleich zur allgemeinen Parkinson-Population [13]. In randomisierten, kontrollierten Studien unterschieden sich Suizidraten und Suizidideen jedoch nicht von vergleichbaren Populationen, sodass ein Bias in der erstgenannten Studie angenommen wird [12].

3. **Apathie**

   Eine Metaanalyse zeigt eine Verschlechterung der Apathie unter STN-THS im Vergleich zum präoperativen Zustand und zur medikamentösen Kontrollgruppe. Insgesamt gab es jedoch lediglich 3 Studien mit einer Kontrollgruppe [40]. Drapier et al. berichten über eine Verschlechterung der Apathie (Starkstein-Apathie-Skala) unter STN-THS (n=15). Die Pat. wurden nicht randomisiert. Die Kontrollgruppe bestand aus einer Wartegruppe auf der Operationsliste. Die Apathie besserte sich in der Kontrollgruppe. Die Levodopa-Äquivalenzdosis für die Kontrollgruppe wird nicht angegeben, es ist also unklar, ob sie erhöht wurde, was eine Besserung der Apathie erklären könnte [41]. Valldeoriola et al. verglichen die Apathie unter STN-THS mit einer Gruppe mit Levodopa/Carbidopa-Intestinalgel (LCIG) und fanden bei dieser nicht randomisierten Studie keinen Unterschied der Apathie zwischen den Gruppen (Lille-Apathie-Rating-Skala) [42].

   In der einzigen randomisierten Studie zur THS im STN im Vergleich zur besten medikamentösen Therapie bei frühen Fluktuationen (EARLY-STIM-Studie) findet sich in einer großen Kohorte

(n=124 STN-THS, n=127 medikamentöse Therapie) nach 2 Jahren Verlaufsbeobachtung kein signifikanter Unterschied für die Apathie, gemessen anhand der Starkstein-Apathie-Skala [43].

Ein artefizielles Medikamentenmanagement zum besseren Verständnis der Apathie zeigte bei komplettem Absetzen der Dopaminagonisten und maximal möglicher Reduktion von Levodopa eine hohe postoperative Prävalenz von Apathie mit einem verzögerten Auftreten im Schnitt 4 Monate nach der Operation [44]. Bei Wiedereinführung eines Dopaminagonisten besserte sich diese Apathie, die somit als Entzugssyndrom bei zu starker Reduzierung der dopaminergen Therapie zu deuten ist [45].

Die korrekte Behandlung der Apathie mit dopaminerger Therapie ist wichtig, da apathische Pat. unter STN-THS bei gleicher motorischer Besserung im Vergleich zu Pat. unter STN-THS ohne Apathie keinen Gewinn der Lebensqualität verspüren [31].

4. **Neuropsychiatrische Fluktuationen**

   Eine randomisierte und kontrollierte Studie zur STN-THS bei frühen motorischen Komplikationen im Vergleich zur medikamentösen Therapie (EARLY-STIM-Studie) zeigte eine signifikante und relevante Besserung der neuropsychiatrischen Fluktuationen (Ardouin-Skala für Verhalten bei der PK) [43] und bestätigte somit eine frühere große Kohortenstudie [46].

5. **Impulskontrollstörung (ICD)**

   Eine randomisierte, kontrollierte Studie zur STN-THS bei frühen motorischen Komplikationen im Vergleich zur medikamentösen Therapie (EARLY-STIM-Studie) zeigte eine Besserung des hyperdopaminergen Verhaltens (Ardouin-Skala für Verhalten bei der PK) und der Apathie (Starkstein-Apathie-Skala) unter STN-THS im Vergleich zur medikamentösen Therapie. Bei Einschluss bestanden nur wenige schwere Impulskontrollstörungen (ICD), sodass eine Besserung für klinisch relevante ICD/Dopamindysregulationssyndrom (DDS)/Punding nicht gezeigt werden konnte. In Anbetracht der potenziell katastrophalen sozialen Auswirkungen von ICD besteht hier der Bedarf einer kontrollierten Studie, bei der ausschließlich Pat. mit relevanten ICD eingeschlossen werden [43], zumal es zu diesem Thema auch überzeugende größere Kohortenstudien gibt mit positiver Wirkung der STN-THS bei paralleler Medikamentenreduktion, einschließlich Punding und DDS [46], [47]. Außerdem wurde prinzipiell eine Reversibilität von psychostimulanten Effekten von Levodopa nachgewiesen [48].

   Nicht alle Studien berichten über Verbesserung der ICDs unter STN-THS. Eine rezente Studie zeigte keinen Unterschied im Outcome von ICDs über 12 Monate Beobachtung beim Vergleich zweier prospektiver Kohorten, eine unter STN-THS, die andere unter Medikation [49]. Die Schwere der ICDs, gemessen anhand der QUIP-RS, war nicht gleich verteilt, im Schnitt war der Score der QUIP-RS in der STN-THS-Gruppe höher. Die Kontrolle bestand aus einer „convenience sample“ von Pat., die zwar in den gleichen Zentren betreut wurden, jedoch keine chirurgischen Kandidaten/Kandidatinnen waren und für die keine motorische Evaluierung vorliegt. In der operativen Gruppe war die Levodopa-Dosis nur geringfügig reduziert (~30%). In beiden Gruppen traten postoperativ gleich viele De-novo-ICDs auf.

6. **Schlaf**

   Eine kontrollierte Studie (prospektive Follow-up-Studie zur STN-THS gegen eine medikamentös behandelte Kontrollgruppe) zeigte eine Besserung von Schlaf und Tagesmüdigkeit (Non Motor

Symptom Scale, Unterdomäne „sleep/fatigue“) im Vergleich zur Baseline und in einer pseudorandomisierten Subgruppe mit vergleichbarer Schwere und Dauer der Erkrankung sowie dopaminerger Therapie (Levodopa-Äquivalenzdosis) bei Baseline der Erkrankung auch im Vergleich zur medikamentös behandelten Kontrollgruppe. Der Effekt wurde als stark bezeichnet [50].

Arnulf et al. zeigten im Schlaflabor eine Verbesserung der Schlafarchitektur (unter STN-THS) mit Verbesserung der Schlafzeit um 49% bei Pat. mit der PK mit Schlafstörung. Die Effekte wurden als sekundär durch eine Verbesserung der Motorik (Besserung der nächtlichen Akinese, Suppression der schmerzhaften Dystonie) interpretiert [51].

Eine subjektive Besserung von Schlafsymptomen unter bilateraler STN-THS wurde in Studien mit Nachuntersuchungen von bis zu 3 Jahren anhand der Non Motor Symptom Scale [52] sowie der Parkinson's disease sleep scale (PDSS) gezeigt [53], [54].

Schlafstörungen sind multikatoriell (z.B. nächtliche Akinese, Depression, hyperdopaminerge nächtliche Hyperaktivität). Es fehlen prospektive Studien zu Veränderungen des Schlafs unter der THS in Abhängigkeit von den Ursachen.

7. **Autonome Symptome**

Eine kontrollierte Studie (prospektive Follow-up-Studie der STN-THS gegen eine medikamentös behandelte Kontrollgruppe) zeigte eine Besserung der Dysurie (Non Motor Symptom Scale (NMSS), Unterdomäne für Blasenstörungen) im Vergleich zur Baseline und in einer pseudorandomisierten Subgruppe mit vergleichbarer Schwere, Dauer und dopaminerger Therapie (Levodopa-Äquivalenzdosis pro Tag, LEDD) bei Baseline der Erkrankung auch im Vergleich zur medikamentös behandelten Kontrollgruppe. Die Effektgröße wurde als „groß“ bezeichnet [50].

Die SCOPA-Aut-Subscores verbesserten sich signifikant unter der STN-THS für Blasenstörung und Thermoregulation/übermäßiges Schwitzen 6 Monate postoperativ [55].

Eine weitere Studie zeigte eine Beschleunigung der Magenpassage unter der STN-THS, während unter Levodopa kein Effekt auf die Magenpassage dargestellt wurde [36]. Inwieweit sich dieser Befund klinisch auswirkt, konnte bislang nicht gezeigt werden. Insgesamt werden Berichte über Effekte der STN-THS auf gastrointestinale Symptome kontrovers diskutiert [56].

Es gibt keine zuverlässigen Berichte zur Besserung einer orthostatischen Hypotonie unter THS.

8. **Schmerz**

Eine Metaanalyse nach den PRISMA-Guidelines von 26 STN- oder GPi-THS-Studien zeigt im Schnitt eine Besserung der Schmerzscores um 40% im Vergleich zur präoperativen Baseline [57].

Eine frühe Studie zur STN-THS zeigte einen dramatischen Effekt der THS auf die schmerzhafte Off-Dystonie [58]. Systematischere Studien zeigten eine gute Besserung von präoperativen Schmerzen anhand spezifischer Skalen wie des McGill Pain Questionnaire [59]. Dies veranlasste die Autoren/Autorinnen zu überprüfen, ob die STN-THS per se einen Effekt auf neuropathische Schmerzen haben könnte. Sie konnten zeigen, dass das Anschalten der STN-THS die subjektive Schmerzschwelle erhöht und parallel schmerzinduzierte zerebrale Aktivität im sensomotorischen

Kortex reduziert [35]. Auch Levodopa verbessert Schmerzen. Es gibt aber keine vergleichenden Studien zur Wirksamkeit auf Schmerz bei der PK.

Die Mechanismen der Wirkung der STN-THS auf die Domäne „Schmerz“ sind nach jetziger Studienlage heterogen. Zum einen werden Schmerzen wahrscheinlich allein durch eine Reduktion der motorischen Off-Symptome gelindert, zum anderen ergeben sich Hinweise auf einen Einfluss der STN-THS auf die zentrale Schmerzverarbeitung.

Begründung der Empfehlung:

Die STN-THS bessert psychiatrische Symptome wie Apathie oder Depression nicht. Diese Symptome können postoperativ bei einem Dopaminagonisten-Entzugssyndrom verstärkt werden, mit negativen Konsequenzen auf die Lebensqualität, und müssen dann gegebenenfalls durch eine Anpassung der dopaminergen Therapie behandelt werden. Eine aktuell vorliegende schwere Depression stellt eine Kontraindikation zur THS dar. Es finden sich in der Literatur Hinweise darauf, dass folgende nicht motorische Symptome durch STN-THS gebessert werden können: 1) neuropsychiatrische Fluktuationen, 2) Off-bezogene Schmerzen, 3) hyperdopaminerges Verhalten bzw. Impulskontrollstörungen, 4) Schlafstörungen.

Es fehlen jedoch Studien, bei denen nicht motorische Symptome primäres Therapieziel waren. Es gibt heute keine ausreichende Evidenz, die STN-THS mit potenziell relevanten chirurgischen Komplikationen einer optimal eingestellten medikamentösen Therapie aufgrund nicht motorischer Symptome vorzuziehen. Allenfalls können solche Symptome bei bestehender Indikation aufgrund von motorischen Komplikationen als zusätzliche Argumente in die differenzialtherapeutischen Überlegungen mit einfließen.

In Anbetracht der Relevanz nicht motorischer Symptome auf die Lebensqualität wären zukünftige randomisierte, kontrollierte Studien zur THS sinnvoll, bei denen behindernde, nicht motorische Symptome Einschlusskriterium und Hauptzielkriterium darstellen.

In Bezug auf neuropsychiatrische Fluktuationen und hyperdopaminerges Verhalten liegt eine randomisierte, kontrollierte Studie vor, diese Symptome waren jedoch sekundäre Kriterien [43].

Obwohl Impulskontrollstörungen sich unter STN-THS und paralleler Reduktion der dopaminergen Therapie in der Regel bessern, ist bei diesen Pat. eine besonders engmaschige postoperative Betreuung erforderlich. Die Besserung erfordert, dass Intensität der Stimulation und dopaminerge Therapie in Abhängigkeit vom Verhalten angepasst werden. Vor allem in den ersten postoperativen Wochen kann eine zu rasche Erhöhung der Stimulationsintensität hyperdopaminerges Verhalten verstärken und eine Therapieanpassung erfordern, ähnlich wie dies bei Dyskinesien gehandhabt wird. In den ersten postoperativen Monaten kann mit einer sehr allmählichen Desensitisierung ein protrahiert auftretendes (im Schnitt nach 4–6 Monaten, im Einzelfall Verzögerung von mehr als einem Jahr möglich) Dopaminagonisten-Entzugssyndrom mit Apathie, Angst, Depression eine erneute Erhöhung der dopaminergen Therapie in Form einer individuell titrierten Erhöhung von Dopaminagonisten erforderlich machen. Da sich diese hypodopaminergen Symptome negativ auf die Lebensqualität auswirken, sollte eine Überwachung dieser Symptome mit systematischem Screening im ersten postoperativen Jahr häufiger erfolgen als in der allgemeinen Parkinson-Bevölkerung.

Neuropsychiatrische Fluktuationen, Off-bezogene Schmerzen, hyperdopaminerges Verhalten bzw. Impulskontrollstörungen und Schlafstörungen sind in prospektiven Studien unter STN-THS in der Regel gebessert und können bei Vorliegen einer Indikation zur THS aufgrund von motorischen Komplikationen oder therapierefraktärem Tremor allenfalls als zusätzliches Argument für eine Operation herangezogen werden.

Parkinsontypische autonome Störungen wie z.B. ein imperativer Harndrang, der verstärkt im Off auftritt, können sich unter STN-THS bessern, jedoch ist die Effektgröße gering, die Abgrenzung von komorbiden, primär urologischen Ursachen ist schwierig und es fehlen prädiktive Faktoren für eine Besserung. Vor diesem Hintergrund sollten autonome Störungen bei der Indikationsstellung keine Berücksichtigung finden.

**Empfehlung**	**Neu**<br><br>**Stand (2023)**
▪ Nicht motorische Symptome stellen heute keine etablierte Indikation für eine STN-THS oder GPi-THS dar.<br>▪ Das Vorhandensein nicht motorischer Fluktuationen und/oder Impulskontrollstörungen und/oder Schlafstörungen kann die Indikation einer STN-THS bei der PK unterstützen.	
Konsensstärke: 100%, starker Konsens	

Fragestellung 173: Wie nachhaltig ist die klinische Symptomkontrolle durch die THS (STN, GPi, VIM) im Vergleich mit der oralen/transdermalen Standardtherapie in der Behandlung der PK mit motorischen Fluktuationen mit und ohne Dyskinesien?

Hintergrund

Die PK ist eine chronische Erkrankung mit klinischer Progression der Symptome und auch einer möglichen Symptomverschiebung über die Zeit (z.B. Gangstörung, Demenz) im Rahmen der fortschreitenden Neurodegeneration verschiedener funktioneller Systeme. Insofern soll hier die Frage bearbeitet werden, wie die THS (STN, GPi, VIM) gegen die konservative medikamentöse Therapie im Langzeitverlauf bei der PK mit motorischen Fluktuationen mit und ohne Dyskinesien zu bewerten ist.

Evidenzgrundlage

Die erhältlichen randomisierten und kontrollierten Studien zur Wirksamkeit der STN-THS gegen die konservative medikamentöse Therapie [1], [2], [3], [4], [5], [6], [7] bilden einen Beobachtungszeitraum von 3 bis 24 Monaten ab. Es existieren eine Reihe offener Langzeitnachbeobachtungen, die in verschiedenen Metaanalysen der STN-THS zusammengefasst sind [60], [61], [62]. Zusätzlich liegt eine dort noch nicht berücksichtigte Nachbeobachtung von 51 Pat. [63] vor. Allerdings vergleichen diese offenen Nachbeobachtungen die Wirkung der STN-THS gegen den präoperativen Status ohne Medikamente und nicht gegen eine im Beobachtungszeitraum ausschließlich medikamentös behandelte Vergleichsgruppe.

Für die GPi-THS lassen sich zwei offene Langzeitbeobachtungen identifizieren, die den Verlauf von 5 bis 6 Jahren postoperativ dokumentieren [64], [65]. Für die randomisierten und kontrollierten Vergleichsstudien der STN-THS versus der GPi-THS [8], [9] existieren offene Nachbeobachtungen für jeweils 3 Jahre [28], [66].

Für den VIM-THS konnte im Langzeitverlauf eine unkontrollierte offene Nachbeobachtungsstudie von 38 Pat. identifiziert werden, die einen Vergleich von motorischen Funktionen und Aktivitäten des täglichen Lebens (UPDRS II und Schwab-und-England-Skala) präoperativ gegen 6 Jahre postoperativ beschreibt [23].

Ergebnis

In zwei Zusammenfassungen identischer Studien offener Langzeitdaten zur Wirksamkeit der STN-THS [60], [61] wurden 8 Studien zur STN-THS mit einer Nachbeobachtungszeit von 5 Jahren (273 Pat.) und 3 Studien mit einer Nachbeobachtungszeit von 8–10 Jahren (52 Pat.) berichtet. Die Parameter UPDRS II (Aktivitäten des täglichen Lebens) und III (Summenscore der motorischen Funktionen inkl. Tremor), Rigor, Bradykinese, Gang und dopaminerge Substitutionstherapie waren während der STN-THS über die Zeit verschlechtert, aber auch nach 8–10 Jahren in diesen Studien noch relevant verbessert zum präoperativen medikamentösen Off-Zustand. UPDRS IV (Dyskinesien) war nur leicht progredient, der Ruhetremor sogar mindestens stabil kontrolliert. Axiale Symptome waren nach 8–10 Jahren zum präoperativen medikamentösen Off verschlechtert, die Funktion „Sprechen“ im Mittel sogar schon nach 5 Jahren.

In einer weiteren jüngeren Metaanalyse [62] wurden zusätzlich zu den genannten Studien noch 7 weitere identifiziert mit dann insgesamt 551 Pat. mit einer Nachbeobachtungszeit von 5 Jahren. 93 Pat. dieser Kohorten wurden für insgesamt 8–11 Jahre klinisch nachverfolgt. Von den ursprünglich 923 Pat. waren 372 Pat. aus verschiedenen Gründen nicht mehr für die Nachuntersuchungen verfügbar. 99 dieser Pat. waren vorher verstorben (nicht der THS zuzuordnen), die übrigen wurden in anderen Zentren weiterbehandelt oder waren anderweitig eingeschränkt, sodass sie an den Nachuntersuchungen nicht teilnehmen konnten. Aus diesen Studienzusammenfassungen geht hervor, dass die STN-THS motorische Funktionen der PK für mindestens 10 Jahre verbessern kann. Dyskinesien und motorische Fluktuationen zeigten sich verbessert, während sich axiale Symptome und auch Parameter der Lebensqualität über die ersten 5 Jahre teils bis unterhalb der Ausgangswerte verschlechterten. Für den UPDRS II (Aktivitäten des täglichen Lebens) waren Verbesserungen zu präoperativ über die ersten 5 Jahre zu verzeichnen, wenn auch über die Jahre abnehmend. Die Langzeitergebnisse waren variabler mit dann teils auch Verschlechterungen, verglichen mit den Ausgangswerten.

Als mögliche Prädiktoren für einen guten Langzeiteffekt wurden in letzterer Zusammenfassung [62] auf Basis der eingeschlossenen Studien eine korrekte Elektrodenlage im sensomotorischen Anteil des STN, ein niedriges Alter der Pat. zum Zeitpunkt der Operation (<65 Jahre), höhere präoperative Werte des UPDRS im Off, ausgeprägtere motorische Fluktuationen sowie eine ausgeprägte Gangstörung im medikamentösen Off identifiziert. Als Prädiktor für einen schlechteren Langzeiteffekt der STN-THS bei der PK wurden ein Pat.-Alter >65 Jahre (insbesondere bezogen auf axiale motorische Funktionen) identifiziert und eine längere Krankheitsdauer war assoziiert mit schlechteren Werten

der Aktivitäten des täglichen Lebens in den Folgeuntersuchungen. Jüngeres Alter zum Zeitpunkt der Operation, besserer präoperativer kognitiver Status (Mini Mental State Examination) und bessere Werte in den Skalen der Aktivitäten des täglichen Lebens im motorischen Off wurden als Variablen für eine Unabhängigkeit in den Aktivitäten des täglichen Lebens nach 5 Jahren THS im STN beschrieben.

In der retrospektiven Studie von 51 Pat. [63] wurde im Mittel 17 Jahre unter STN-THS nachbeobachtet. Verglichen mit dem präoperativen Zustand, waren die Zeit mit Dyskinesien um 75%, die Zeit im motorischen Off um 58,7%, die Menge dopaminerger Medikamente um 50,6% reduziert und die Lebensqualität um 13,8% und die emotionalen und sozialen Funktionen um 13,6% bzw. 29,9% verbessert.

Für die Wirksamkeit der GPi-THS sind die Fallzahlen im Langzeitverlauf mit 6 Pat. (initiale Kohorte 11 Pat.) [65] bzw. 16 Pat. [64] sehr gering und lassen nur sehr bedingt Rückschlüsse zu. So wird in ersterer Studie nach drei Jahren eine beständige Reduktion im UPDRS III (motorischer Teil) von 43% unter GPi-THS zum präoperativen medikamentösen Off mit einer überdauernden Verbesserung nach 5 Jahren nur noch für das Subitem „Rigor“ angegeben. Dyskinesien waren auch nach 5 Jahren noch relevant kontrolliert. 4 der initial 11 Pat. hatten einen initial fehlenden Benefit der GPi-THS und wurden dann mit einer STN-THS behandelt. In der zweiten Studie (offene, nicht randomisierte prospektive Multicenter-Studie) war nach 5–6 Jahren Nachbeobachtung eine Reduktion im UPDRS III (THS Off vs. THS On) von im Mittel 20% (doppelt verblindet in Bezug auf die THS), bzw. THS Off vs. präoperatives medikamentöses Off (nicht verblindet) von etwa 35% zu verzeichnen. Verglichen mit Pat., die in der gleichen Studie eine STN-THS erhielten, zeigten weniger von ihnen kognitive Einbußen, Dysarthrie sowie Gangstörungen.

Der STN-THS vergleichbare Langzeitdaten (z.B. >10 Jahre) sind für die GPi-THS nicht verfügbar.

Für die 36 Monate Nachbeobachtung der Vergleichsstudien STN-THS versus GPi-THS [28], [66] waren die Ergebnisse ebenso heterogen. So waren in einer der Studien [66] die motorischen Symptome, verglichen mit dem medikamentösen Off in der Gruppe der STN-THS, besser kontrolliert, aber ohne Unterschiede zwischen den Zielpunkten in einer Kombinationaskala von Kognitions-, Stimmungs- und Verhaltenswerten. Die dopaminerge Medikation war stärker reduziert in der Gruppe der STN-THS. Keine Unterschiede gab es bei den Nebenwirkungen, allerdings wurden 8 Pat. aus der Gruppe der GPi-THS im Verlauf mit einer STN-THS behandelt versus 1 Pat. aus der STN-Gruppe im GPi implantiert [66]. In der anderen Studie [28] waren die motorischen Funktionen zwischen den Zielpunkten nach 36 Monaten nicht unterschiedlich. Die nach 6 Monaten gezeigten Verbesserungen in der Lebensqualitätsskala waren über die Zeit der 3 Jahre in beiden Zielpunkten dann nicht mehr nachweisbar, die Kognition (MATTIS dementia rating scale) verschlechterte sich in der STN-Gruppe stärker.

Für die VIM-THS konnte im Langzeitverlauf von 6 Jahren in einer unkontrollierten offenen Nachbeobachtungsstudie von 38 Pat. eine Verbesserung zum präoperativen motorischen Off im UPDRS-III-Summenscore von 15% gezeigt werden, für den Vergleich der Aktivitäten des täglichen Lebens (UPDRS II und Schwab-und-England-Skala) waren die nach 1 Jahr gezeigten signifikanten Effekte nach 6 Jahren dann nicht mehr nachweisbar [23].

Begründung der Empfehlung

Die Evidenz für die STN-THS begründet sich in der Zusammenfassung methodisch heterogener, offener und restrospektiver Beobachtungsstudien, die aber jede für sich zu ähnlichen Ergebnissen kommt. Die Pat.-Zahlen sind insbesondere für die längere Nachbeobachtungszeit von bis zu 11 Jahren eher klein mit einer relevanten Anzahl von Pat. die aus den Studien über die Zeit ausgeschieden sind. Dies kann dazu führen, dass insbesondere Pat. mit gutem Langzeitverlauf eher in die Beobachtungen eingegangen sind. Im Langzeitverlauf bis 11 Jahre kann die STN-THS bei der PK insbesondere in der Verbesserung von Dyskinesien und motorischen Fluktuationen, Rigor, Tremor und Reduktion dopaminerger Substitution weiter wirksam sein. Axiale Symptome und Sprechen sowie Parameter zur Lebensqualität sind im Langzeitverlauf eher verschlechtert, heterogen verhält es sich mit Parametern der Aktivitäten des täglichen Lebens. Für die Wirksamkeit der GPi-THS gegen den präoperativen Zustand sind die Ergebnisse bei sehr kleinen Pat.-Zahlen heterogen und lassen keine Empfehlung zu. Ebenso heterogen waren die offenen Nachbeobachtungen von 36 Monaten der Vergleichsstudien STN-THS versus GPi-THS mit besserer Kontrolle der motorischen Parkinson-Symptome in der STN-Gruppe in der einen Studie [66] und nicht unterschiedlichen motorischen Funktionen in der anderen Studie [28].

**Empfehlung**	**Neu**<br><br>**Stand (2023)**
▪ Die STN-THS ist nach derzeitiger Datenlage im Langzeitverlauf bis 11 Jahre wirksam in der Reduktion von Dyskinesien, motorischen Fluktuationen, Rigor und Tremor sowie in der Reduktion der dopaminergen Substitution. Dabei ergeben sich keine Hinweise auf eine zeitliche Verzögerung des Auftretens von Symptomen, die einer progredienten Neurodegeneration zugeschrieben werden (z.B. Demenz, axiale Symptome, segmentale Akinese). Vergleichbare Langzeitdaten (z.B. >10 Jahre) sind für die GPi-THS nicht verfügbar.<br>▪ Zum Vergleich der Zielpunkte STN und GPi (bis 36 Monate) in der Behandlung der motorischen Symptome der PK lässt die bisherige Datenlage keine Aussage zu.<br>▪ Eine VIM-THS soll in der Behandlung der PK mit motorischen Fluktuationen mit und ohne Dyskinesien nicht eingesetzt werden.	
Konsensstärke: 100%, starker Konsens	

Fragestellung 174: Wie nachhaltig ist die klinische Symptomkontrolle durch die THS (STN, GPi, VIM) im Vergleich mit der oralen/transdermalen Standardtherapie in der Behandlung der PK mit pharmakoresistentem Tremor?

Hintergrund

Die THS ist in den Zielgebieten STN, GPi und VIM wirksam in der Behandlung des pharmakoresistenten Tremors bei der PK. Hier soll die Frage geklärt werden, wie nachhaltig diese klinische Symptomkontrolle gegen die medikamentöse Therapie ist.

Evidenzgrundlage

Es existieren keine randomisiert kontrollierten Langzeitdaten zu dieser Fragestellung. Zur Frage der Nachhaltigkeit der THS in den Zielgebieten STN, GPi und VIM existieren nur unkontrollierte Nachbeobachtungen und auch nicht gegen die medikamentöse Standardtherapie, sondern gegen die präoperative Symptomschwere im medikamentösen Off (STN: [60], [61], [62]; GPi: [65], [64]), im Vergleich GPi-THS versus STN-THS [28], [66] und im Vergleich THS On versus THS Off, bzw. zum präoperativen klinischen Status (VIM: [21], [22], [23], [24]).

Ergebnis

In zwei Zusammenfassungen identischer Studien offener Langzeitdaten zur Wirksamkeit der STN-THS [60], [61] wurden 8 Studien mit einer Nachbeobachtungszeit von 5 Jahren (273 Pat.) und 3 Studien mit einer Nachbeobachtungszeit von 8–10 Jahren (52 Pat.) berichtet. Über den gesamten Beobachtungszeitraum war die Tremor-Kontrolle als stabil anzusehen und um im Mittel etwa 80% zum präoperativen Status reduziert. In einer weiteren jüngeren Metaanalyse [62] wurden zusätzlich zu den genannten Studien noch 5 weitere identifiziert, die den Tremor in der Bewertung separat aufführen mit dann insgesamt 477 Pat. mit einer Nachbeobachtungszeit von 5 Jahren. 93 Pat. dieser Kohorten wurden für insgesamt 8–11 Jahre klinisch nachverfolgt. Es ergab sich im Mittel eine Reduktion der Tremor-Schwere von 73% nach 5 Jahren und 74% nach 8–11 Jahren, jeweils zum präoperativen klinischen Status im medikamentösen Off.

Für die Wirksamkeit der GPi-THS sind die Fallzahlen im Langzeitverlauf mit 6 Pat. (initiale Kohorte 11 Pat.) [65] bzw. 16 Pat. [64] sehr gering und lassen nur sehr bedingt Rückschlüsse zu. So wird in ersterer Studie nach drei Jahren (9 Pat.) wie auch nach fünf Jahren (6 Pat.) keine signifikante Tremor-Reduktion jeweils im Vergleich mit THS Off zum Untersuchungszeitpunkt angegeben. In der zweiten Studie war die Domäne „Tremor“ im medikamentösen Off/THS On nach 5–6 Jahren weiterhin signifikant reduziert (65,5% Verbesserung) vs. präoperativer Status im medikamentösen Off.

Für die Vergleichsstudien STN-THS versus GPi-THS [28], [66] weist nur eine der Arbeiten die motorische Domäne „Tremor“ separat aus [28]. So ergab sich dort nach 6 Monaten zwischen den Zielpunkten STN und GPi im medikamentösen Off/THS On kein Unterschied in der Symptomkontrolle, was über die Beobachtungszeit von 36 Monaten dann stabil blieb (verblindet für STN vs GPi, nicht für THS Off vs. On).

Für die VIM-THS konnten 4 Studien identifiziert werden, mit offenen Nachbeobachtungen von 12 Monaten bis zu 21 Jahren [21], [22], [23], [24]. Teils wurden dort unilaterale und bilaterale THS im VIM getrennt aufgeführt, relevante Effekte einer Tremor-Suppression sind aber durchweg beschrieben: unilateral: nach 1 Jahr 67% Besserung, nach 5 Jahren etwa 85%, nach 11–15 Jahren 58% und nach 16–21 Jahren etwa 63%; bilateral: nach 1 Jahr etwa 73%, nach 6–7 Jahren 64%, nach 11–15 Jahren etwa 69%, nach 16–21 Jahren etwa 60%. Bemerkenswert ist die nur kurz dauernde Verbesserung bei den Alltagsaktivitäten von etwa nur 1 Jahr postoperativ. Stimulationsassoziierte Nebenwirkungen waren der Lage der Elektroden im Zielgebiet geschuldet (bis 45% Dysästhesien, bis 41% Schmerzen, bis 75% Dysarthrie, Gang- und Gleichgewichtsstörungen bis 93%) und vielfach durch eine Nachprogrammierung der THS-Parameter reversibel. Erfahrungsgemäß ist in der für die Tremor-Kontrolle empfänglichen Region des VIM und der posterioren subthalamischen Area (PSA) häufig

eine balancierte Stimulationsprogrammierung notwendig, um eine möglichst vollständige Tremor-Suppression zu erreichen, ohne dem Zielgebiet direkt zuzuschreibende Nebenwirkungen (Ataxie, Dysarthrie, Par- und Dysästhesien).

Begründung der Empfehlung

Die Evidenz stützt sich ausschließlich auf nicht kontrollierte Nachbeobachtungen in sehr unterschiedlichem Studiendesign, klinischer Evaluation der Symptome, Verfahrensweisen der chirurgischen Abläufe (inkl. Zielpunktdefinition) und der postoperativen Programmierung der Stimulation. Auch sind die Studienkohorten mit jeweils eher geringen Pat.-Zahlen ausgestattet worden.

Mit diesen Einschränkungen kann für die THS im STN wie für die THS im VIM eine nachhaltige und relevante Tremor-Kontrolle angenommen werden. Für die THS im GPi ist auch eine nachhaltige Tremor-Kontrolle anzunehmen mit der Einschränkung einer noch geringeren kumulativen Pat.-Zahl.

**Empfehlung**	**Neu**<br><br>**Stand (2023)**
▪ Die STN-THS ist auch im Langzeitverlauf über mindestens 10 Jahre relevant auf einen pharmakoresistenten Parkinson-Tremor wirksam und soll Pat. mit einem pharmakoresistenten Parkinson-Tremor unter Beachtung der Kontraindikationen angeboten werden und vorzugsweise bilateral erfolgen.<br>▪ Die Symptomkontrolle eines Parkinson-Tremors durch die GPi-THS ist nachhaltig. Die Wahl des Zielgebiets bei Pat. mit der PK und medikamentös nicht kontrollierbarem Parkinson-Tremor soll unter Berücksichtigung des Weiteren individuellen Symptomprofils erfolgen (siehe Frage 176).<br>▪ Die uni- oder bilaterale VIM-THS ist bei medikamentös nicht kontrollierbarem Parkinson-Tremor auch im Langzeitverlauf wirksam und kann bei Kontraindikationen für eine STN-THS oder GPi-THS erwogen werden.	
Konsensstärke: 100%, starker Konsens	

Referenzen

1. Deuschl G, Schade-Brittinger C, Krack P, Volkmann J, Schäfer H, Bötzel K, et al. A randomized trial of deep-brain stimulation for Parkinson's disease. N Engl J Med. 2006;355(9):896-908
2. Schüpbach WM, Maltête D, Houeto JL, du Montcel ST, Mallet L, Welter ML, et al. Neurosurgery at an earlier stage of Parkinson disease: a randomized, controlled trial. Neurology. 2007;68(4):267-71
3. Weaver FM, Follett K, Stern M, Hur K, Harris C, Marks WJ, Jr., et al. Bilateral deep brain stimulation vs best medical therapy for patients with advanced Parkinson disease: a randomized controlled trial. Jama. 2009;301(1):63-73

4. Williams A, Gill S, Varma T, Jenkinson C, Quinn N, Mitchell R, et al. Deep brain stimulation plus best medical therapy versus best medical therapy alone for advanced Parkinson's disease (PD SURG trial): a randomised, open-label trial. Lancet Neurol. 2010;9(6):581-91

5. Okun MS, Gallo BV, Mandybur G, Jagid J, Foote KD, Revilla FJ, et al. Subthalamic deep brain stimulation with a constant-current device in Parkinson's disease: an open-label randomised controlled trial. Lancet Neurol. 2012;11(2):140-9

6. Schuepbach WM, Rau J, Knudsen K, Volkmann J, Krack P, Timmermann L, et al. Neurostimulation for Parkinson's disease with early motor complications. N Engl J Med. 2013;368(7):610-22

7. Vitek JL, Jain R, Chen L, Tröster AI, Schrock LE, House PA, et al. Subthalamic nucleus deep brain stimulation with a multiple independent constant current-controlled device in Parkinson's disease (INTREPID): a multicentre, double-blind, randomised, sham-controlled study. Lancet Neurol. 2020;19(6):491-501

8. Follett KA, Weaver FM, Stern M, Hur K, Harris CL, Luo P, et al. Pallidal versus subthalamic deep-brain stimulation for Parkinson's disease. N Engl J Med. 2010;362(22):2077-91

9. Odekerken VJ, van Laar T, Staal MJ, Mosch A, Hoffmann CF, Nijssen PC, et al. Subthalamic nucleus versus globus pallidus bilateral deep brain stimulation for advanced Parkinson's disease (NSTAPS study): a randomised controlled trial. Lancet Neurol. 2013;12(1):37-44

10. Schuepbach WMM, Tonder L, Schnitzler A, Krack P, Rau J, Hartmann A, et al. Quality of life predicts outcome of deep brain stimulation in early Parkinson disease. Neurology. 2019;92(10):e1109-e20

11. Deuschl G, Follett KA, Luo P, Rau J, Weaver FM, Paschen S, et al. Comparing two randomized deep brain stimulation trials for Parkinson's disease. J Neurosurg. 2019;132(5):1376-84

12. Weintraub D, Duda JE, Carlson K, Luo P, Sagher O, Stern M, et al. Suicide ideation and behaviours after STN and GPi DBS surgery for Parkinson's disease: results from a randomised, controlled trial. J Neurol Neurosurg Psychiatry. 2013;84(10):1113-8

13. Xu Y, Yang B, Zhou C, Gu M, Long J, Wang F, et al. Suicide and suicide attempts after subthalamic nucleus stimulation in Parkinson's disease: a systematic review and meta-analysis. Neurol Sci. 2021;42(1):267-74

14. Kleiner-Fisman G, Herzog J, Fisman DN, Tamma F, Lyons KE, Pahwa R, et al. Subthalamic nucleus deep brain stimulation: summary and meta-analysis of outcomes. Mov Disord. 2006;21 Suppl 14:S290-304

15. Benabid AL, Pollak P, Gervason C, Hoffmann D, Gao DM, Hommel M, et al. Long-term suppression of tremor by chronic stimulation of the ventral intermediate thalamic nucleus. Lancet. 1991;337(8738):403-6

16. Hubble JP, Busenbark KL, Wilkinson S, Pahwa R, Paulson GW, Lyons K, Koller WC. Effects of thalamic deep brain stimulation based on tremor type and diagnosis. Mov Disord. 1997;12(3):337-41

17. Koller W, Pahwa R, Busenbark K, Hubble J, Wilkinson S, Lang A, et al. High-frequency unilateral thalamic stimulation in the treatment of essential and parkinsonian tremor. Ann Neurol. 1997;42(3):292-9

18. Ondo W, Jankovic J, Schwartz K, Almaguer M, Simpson RK. Unilateral thalamic deep brain stimulation for refractory essential tremor and Parkinson's disease tremor. Neurology. 1998;51(4):1063-9

19. Limousin P, Speelman JD, Gielen F, Janssens M. Multicentre European study of thalamic stimulation in parkinsonian and essential tremor. J Neurol Neurosurg Psychiatry. 1999;66(3):289-96

20. Krack P, Benazzouz A, Pollak P, Limousin P, Piallat B, Hoffmann D, et al. Treatment of tremor in Parkinson's disease by subthalamic nucleus stimulation. Mov Disord. 1998;13(6):907-14

21. Rehncrona S, Johnels B, Widner H, Törnqvist AL, Hariz M, Sydow O. Long-term efficacy of thalamic deep brain stimulation for tremor: double-blind assessments. Mov Disord. 2003;18(2):163-70

22. Pahwa R, Lyons KE, Wilkinson SB, Simpson RK, Jr., Ondo WG, Tarsy D, et al. Long-term evaluation of deep brain stimulation of the thalamus. J Neurosurg. 2006;104(4):506-12

23. Hariz MI, Krack P, Alesch F, Augustinsson LE, Bosch A, Ekberg R, et al. Multicentre European study of thalamic stimulation for parkinsonian tremor: a 6 year follow-up. J Neurol Neurosurg Psychiatry. 2008;79(6):694-9

24. Cury RG, Fraix V, Castrioto A, Pérez Fernández MA, Krack P, Chabardes S, et al. Thalamic deep brain stimulation for tremor in Parkinson disease, essential tremor, and dystonia. Neurology. 2017;89(13):1416-23

25. Wong JK, Cauraugh JH, Ho KWD, Broderick M, Ramirez-Zamora A, Almeida L, et al. STN vs. GPi deep brain stimulation for tremor suppression in Parkinson disease: A systematic review and meta-analysis. Parkinsonism Relat Disord. 2019;58:56-62

26. Okun MS, Fernandez HH, Wu SS, Kirsch-Darrow L, Bowers D, Bova F, et al. Cognition and mood in Parkinson's disease in subthalamic nucleus versus globus pallidus interna deep brain stimulation: the COMPARE trial. Ann Neurol. 2009;65(5):586-95

27. Anderson VC, Burchiel KJ, Hogarth P, Favre J, Hammerstad JP. Pallidal vs subthalamic nucleus deep brain stimulation in Parkinson disease. Arch Neurol. 2005;62(4):554-60

28. Weaver FM, Follett KA, Stern M, Luo P, Harris CL, Hur K, et al. Randomized trial of deep brain stimulation for Parkinson disease: thirty-six-month outcomes. Neurology. 2012;79(1):55-65

29. Obeso JA, Olanow CW, Rodriguez-Oroz MC, Krack P, Kumar R, Lang AE. Deep-brain stimulation of the subthalamic nucleus or the pars interna of the globus pallidus in Parkinson's disease. N Engl J Med. 2001;345(13):956-63

30. Witjas T, Kaphan E, Azulay JP, Blin O, Ceccaldi M, Pouget J, et al. Nonmotor fluctuations in Parkinson's disease: frequent and disabling. Neurology. 2002;59(3):408-13

31. Martinez-Fernandez R, Pelissier P, Quesada JL, Klinger H, Lhommée E, Schmitt E, et al. Postoperative apathy can neutralise benefits in quality of life after subthalamic stimulation for Parkinson's disease. J Neurol Neurosurg Psychiatry. 2016;87(3):311-8

32. Lachenmayer ML, Mürset M, Antih N, Debove I, Muellner J, Bompart M, et al. Subthalamic and pallidal deep brain stimulation for Parkinson's disease-meta-analysis of outcomes. NPJ Parkinsons Dis. 2021;7(1):77

33. Deuschl G, Antonini A, Costa J, Śmiłowska K, Berg D, Corvol JC, et al. European Academy of Neurology/Movement Disorder Society-European Section Guideline on the Treatment of Parkinson's Disease: I. Invasive Therapies. Mov Disord. 2022;37(7):1360-74

34. Funkiewiez A, Ardouin C, Krack P, Fraix V, Van Blercom N, Xie J, et al. Acute psychotropic effects of bilateral subthalamic nucleus stimulation and levodopa in Parkinson's disease. Mov Disord. 2003;18(5):524-30

35. Dellapina E, Ory-Magne F, Regragui W, Thalamas C, Lazorthes Y, Rascol O, et al. Effect of subthalamic deep brain stimulation on pain in Parkinson's disease. Pain. 2012;153(11):2267-73

36. Arai E, Arai M, Uchiyama T, Higuchi Y, Aoyagi K, Yamanaka Y, et al. Subthalamic deep brain stimulation can improve gastric emptying in Parkinson's disease. Brain. 2012;135(Pt 5):1478-85

37. Seif C, Herzog J, van der Horst C, Schrader B, Volkmann J, Deuschl G, et al. Effect of subthalamic deep brain stimulation on the function of the urinary bladder. Ann Neurol. 2004;55(1):118-20

38. Sartori AM, Kiss B, Mordasini L, Pollo C, Schüpbach M, Burkhard FC, et al. Effects of Deep Brain Stimulation on Lower Urinary Tract Function in Neurological Patients. Eur Urol Focus. 2022;8(6):1775-82

39. Wolz M, Hauschild J, Koy J, Fauser M, Klingelhöfer L, Schackert G, et al. Immediate effects of deep brain stimulation of the subthalamic nucleus on nonmotor symptoms in Parkinson's disease. Parkinsonism Relat Disord. 2012;18(8):994-7

40. Zoon TJC, van Rooijen G, Balm G, Bergfeld IO, Daams JG, Krack P, et al. Apathy Induced by Subthalamic Nucleus Deep Brain Stimulation in Parkinson's Disease: A Meta-Analysis. Mov Disord. 2021;36(2):317-26

41. Drapier D, Drapier S, Sauleau P, Haegelen C, Raoul S, Biseul I, et al. Does subthalamic nucleus stimulation induce apathy in Parkinson's disease? J Neurol. 2006;253(8):1083-91

42. Valldeoriola F, Santacruz P, Ríos J, Compta Y, Rumià J, Muñoz JE, et al. l-Dopa/carbidopa intestinal gel and subthalamic nucleus stimulation: Effects on cognition and behavior. Brain Behav. 2017;7(11):e00848

43. Lhommée E, Wojtecki L, Czernecki V, Witt K, Maier F, Tonder L, et al. Behavioural outcomes of subthalamic stimulation and medical therapy versus medical therapy alone for Parkinson's disease with early motor complications (EARLYSTIM trial): secondary analysis of an open-label randomised trial. Lancet Neurol. 2018;17(3):223-31

44. Thobois S, Ardouin C, Lhommée E, Klinger H, Lagrange C, Xie J, et al. Non-motor dopamine withdrawal syndrome after surgery for Parkinson's disease: predictors and underlying mesolimbic denervation. Brain. 2010;133(Pt 4):1111-27

45. Thobois S, Lhommée E, Klinger H, Ardouin C, Schmitt E, Bichon A, et al. Parkinsonian apathy responds to dopaminergic stimulation of D2/D3 receptors with piribedil. Brain. 2013;136(Pt 5):1568-77

46. Lhommée E, Klinger H, Thobois S, Schmitt E, Ardouin C, Bichon A, et al. Subthalamic stimulation in Parkinson's disease: restoring the balance of motivated behaviours. Brain. 2012;135(Pt 5):1463-77

47. Eusebio A, Witjas T, Cohen J, Fluchère F, Jouve E, Régis J, Azulay JP. Subthalamic nucleus stimulation and compulsive use of dopaminergic medication in Parkinson's disease. J Neurol Neurosurg Psychiatry. 2013;84(8):868-74

48. Castrioto A, Kistner A, Klinger H, Lhommée E, Schmitt E, Fraix V, et al. Psychostimulant effect of levodopa: reversing sensitisation is possible. J Neurol Neurosurg Psychiatry. 2013;84(1):18-22

49. Hernandez-Con P, Lin I, Mamikonyan E, Deeb W, Feldman R, Althouse A, et al. Course of Impulse Control Disorder Symptoms in Parkinson's Disease: Deep Brain Stimulation Versus Medications. Mov Disord Clin Pract. 2023;10(6):903-13

50. Jost ST, Sauerbier A, Visser-Vandewalle V, Ashkan K, Silverdale M, Evans J, et al. A prospective, controlled study of non-motor effects of subthalamic stimulation in Parkinson's disease: results at the 36-month follow-up. J Neurol Neurosurg Psychiatry. 2020;91(7):687-94

51. Arnulf I, Bejjani BP, Garma L, Bonnet AM, Houeto JL, Damier P, et al. Improvement of sleep architecture in PD with subthalamic nucleus stimulation. Neurology. 2000;55(11):1732-4

52. Dafsari HS, Martinez-Martin P, Rizos A, Trost M, Dos Santos Ghilardi MG, Reddy P, et al. EuroInf 2: Subthalamic stimulation, apomorphine, and levodopa infusion in Parkinson's disease. Mov Disord. 2019;34(3):353-65

53. Choi JH, Kim HJ, Lee JY, Yoo D, Im JH, Paek SH, Jeon B. Long-term effects of bilateral subthalamic nucleus stimulation on sleep in patients with Parkinson's disease. PLoS One. 2019;14(8):e0221219

54. Dafsari HS, Ray-Chaudhuri K, Ashkan K, Sachse L, Mahlstedt P, Silverdale M, et al. Beneficial effect of 24-month bilateral subthalamic stimulation on quality of sleep in Parkinson's disease. J Neurol. 2020;267(6):1830-41

55. Zhang F, Wang F, Li CH, Wang JW, Han CL, Fan SY, et al. Subthalamic nucleus-deep brain stimulation improves autonomic dysfunctions in Parkinson's disease. BMC Neurol. 2022;22(1):124

56. Krack P, Volkmann J, Tinkhauser G, Deuschl G. Deep Brain Stimulation in Movement Disorders: From Experimental Surgery to Evidence-Based Therapy. Mov Disord. 2019;34(12):1795-810

57. Flouty O, Yamamoto K, Germann J, Harmsen IE, Jung HH, Cheyuo C, et al. Idiopathic Parkinson's disease and chronic pain in the era of deep brain stimulation: a systematic review and meta-analysis. J Neurosurg. 2022;137(6):1821-30

58. Krack P, Pollak P, Limousin P, Benazzouz A, Deuschl G, Benabid AL. From off-period dystonia to peak-dose chorea. The clinical spectrum of varying subthalamic nucleus activity. Brain. 1999;122 (Pt 6):1133-46

59. Pellaprat J, Ory-Magne F, Canivet C, Simonetta-Moreau M, Lotterie JA, Radji F, et al. Deep brain stimulation of the subthalamic nucleus improves pain in Parkinson's disease. Parkinsonism Relat Disord. 2014;20(6):662-4

60. Deuschl G, Agid Y. Subthalamic neurostimulation for Parkinson's disease with early fluctuations: balancing the risks and benefits. Lancet Neurol. 2013;12(10):1025-34

61. Deuschl G, Paschen S, Witt K. Clinical outcome of deep brain stimulation for Parkinson's disease. Handb Clin Neurol. 2013;116:107-28

62. Limousin P, Foltynie T. Long-term outcomes of deep brain stimulation in Parkinson disease. Nat Rev Neurol. 2019;15(4):234-42

63. Bove F, Mulas D, Cavallieri F, Castrioto A, Chabardès S, Meoni S, et al. Long-term Outcomes (15 Years) After Subthalamic Nucleus Deep Brain Stimulation in Patients With Parkinson Disease. Neurology. 2021

64. Moro E, Lozano AM, Pollak P, Agid Y, Rehncrona S, Volkmann J, et al. Long-term results of a multicenter study on subthalamic and pallidal stimulation in Parkinson's disease. Mov Disord. 2010;25(5):578-86

65. Volkmann J, Allert N, Voges J, Sturm V, Schnitzler A, Freund HJ. Long-term results of bilateral pallidal stimulation in Parkinson's disease. Ann Neurol. 2004;55(6):871-5

66. Odekerken VJ, Boel JA, Schmand BA, de Haan RJ, Figee M, van den Munckhof P, et al. GPi vs STN deep brain stimulation for Parkinson disease: Three-year follow-up. Neurology. 2016;86(8):755-61

67 Deuschl G., Schwingenschuh P. et al., Tremor, S2k-Leitlinie, 2022; in: Deutsche Gesellschaft für Neurologie (Hrsg.), Leitlinien für Diagnostik und Therapie in der Neurologie. Online: www.dgn.org/leitlinien

3.21 Ablative Therapien

Autoren: Thomas Köglsperger, Günter Deuschl

Hintergrund

Unter den ablativen Verfahren werden bei der PK Eingriffe verstanden, die umschriebene Läsionen in einer der als wirksam bekannten Zielstrukturen, nämlich ventromediolateraler Thalamus (Nucleus ventralis intermedius, VIM), Nucleus subthalamicus (STN) oder im internen Pallidum (Globus pallidus internus, GPi) vornehmen [1], [2]. Das älteste Verfahren ist die Thermoläsion mit Radiofrequenzerhitzung über eine durch ein Bohrloch eingeführte spezielle Sonde. Später kam dann die Radiotherapie mit stereotaktisch applizierter Gamma-Strahlung hinzu. Seit wenigen Jahren steht als drittes Verfahren die Magnetresonanz-gestützte fokussierte Ultraschallbehandlung (MR-guided focussed ultrasound, MRgFUS) zur Verfügung. Dabei wird im stereotaktischen Rahmen hochenergetischer Ultraschall zu einer Thermoläsion genutzt. Das erste Verfahren wird nur noch von wenigen Spezialisten in Europa beherrscht, die Strahlenbehandlung wird in den deutschsprachigen Ländern nur noch an einem Ort angeboten, während MRgFUS inzwischen von einer rasch zunehmenden Zahl von Kliniken angeboten wird. Der Hauptgrund für den Rückgang der Radiofrequenztherapie und der Radiotherapie ist die im Vergleich zur THS höhere Nebenwirkungsrate. Bei der Strahlentherapie kommt erschwerend hinzu, dass die Wirkung der Läsion vorher nicht getestet werden kann.

Seit ca. 10 Jahren ist der MRgFUS für die Behandlung des essenziellen Tremors zugelassen, zuletzt auch beidseits mit einem 9-Monate-Abstand zwischen den zwei Interventionen. Seit Kurzem haben FDA und EMA die einseitige Behandlung des Parkinson-Tremors im VIM zugelassen. Neu ist ebenfalls die FDA-Zulassung der MRgFUS-Pallidotomie, aber noch nicht der Läsion des STN (s.u.). Die MRT-gestützte Navigation ermöglicht eine exakte Lokalisation sowie ein Temperaturmonitoring in Echtzeit. Es werden dabei bis zu 1.024 Ultraschallsonden im Zielpunkt gebündelt; durch Umwandlung der Schallenergie in thermische Energie wird dadurch eine umschriebene Koagulation im Millimeterbereich ermöglicht. Bei nur geringer Temperaturerhöhung bis ca. 50 Grad bleibt der Funktionsausfall reversibel, sodass eine Effekttestung vor endgültiger Koagulation möglich ist. Die bei radiochirurgischen Verfahren nicht dosislinearen Nebenwirkungen (wie z.B. passagere Marklagerödeme) [3], [4], [5] sind bei dem MRgFUS nicht zu erwarten. Durch den MRgFUS können möglicherweise eine Verbesserung der Lebensqualität (QoL, quality of life) sowie der Aktivitäten des täglichen Lebens (ADL, activities of daily living) und eine wahrscheinliche Verbesserung der motorischen Symptome erreicht werden [6], [7]. Die meisten therapieassoziierten Nebenwirkungen waren von leichter und vorübergehender Natur. Trotz der fehlenden Inzision handelt es sich um ein läsionelles Verfahren.

Fragestellung 175: Wie effektiv und sicher sind ablative Verfahren (Thermokoagulation, Gamma-/Cyberknife, fokussierter Ultraschall) im Vergleich mit der oralen/transdermalen Standardtherapie in der Behandlung der PK unter Berücksichtigung kurz- und mittelfristiger Therapieeffekte auf Beweglichkeit und Lebensqualität?

Evidenzgrundlage

Im Gegensatz zur THS, für die vergleichende Studien existieren [8], [9], [10], liegen insgesamt nur wenig oder keine randomisierten Studien vor zum Vergleich einer medikamentösen Therapie mit den älteren ablativen Verfahren. Für die Pallidotomie liegen zwei verblindete, randomisierte und kontrollierte Studien mit 36 und 37 Pat. vor [11], [12]. Für die Thalamo- oder Subthalamotomie mittels Radiofrequenzablation gibt es keine randomisierten Studien. Die MRgFUS-gesteuerte Thalamotomie wurde in einer kontrollierten Studie [13] und Fallserien [14], [15] bei der tremordominanten PK untersucht. Für die MRgFUS-gesteuerte einseitige Läsion des STN und die einseitige Pallidotomie bei der einseitig betonten PK gibt es neuerdings je eine randomisierte, doppelblinde kontrollierte Studie [6], [16].

Ergebnis

Die Untersuchungen legen nahe, dass die Radiofrequenz-Pallidotomie die Lebensqualität ebenso verbessern kann wie die ADL (UPDRS II) sowie die motorischen Symptome (UPDRS III). Die Pallidotomie reduziert wahrscheinlich die Komplikationen der Therapie (UPDRS IV). Die Pallidotomie führt häufiger zu (schwerwiegenden) Nebenwirkungen. Ob die Pallidotomie nicht motorische Symptome wie z.B. Depression oder Gangstörung verbessert, bleibt unklar. Langzeitdaten dieser Studien wurden nicht veröffentlicht, Fallserien zu Pallidotomien haben aber positive Langzeitdaten für bis zu 5 Jahre berichtet [17], [18], [19], [20]. Die Nebenwirkungen sind deutlicher als bei der THS und die Behandlung kann immer nur einseitig durchgeführt werden.

Bei den Studien zur Thalamotomie handelt es sich um Fallserien aus dem letzten Jahrhundert und es fehlen rezente detaillierte Berichte über Nebenwirkungen oder Langzeitfolgen. Im Vergleich zur medikamentösen Therapie sind die Komplikationsraten hoch. Bei beidseitiger Anwendung werden Dysarthrien bei bis zu 40 % berichtet [21], weshalb sich der bilaterale Eingriff verbietet. Die höhere Anzahl von unerwünschten Wirkungen mit Radiofrequenz im Vergleich zu Thalamus-THS bei unilateralen und insbesondere bei bilateralen Verfahren wurde in einer kontrollierten Studie gezeigt [22].

Die MRgFUS-Behandlung des Parkinson-Tremors wurde in einer Sham-kontrollierten Studie untersucht und es wurde eine gute Wirkung nachgewiesen. Zwar ist diese Indikation von FDA und EMA bereits zugelassen, eine aktuelle Leitlinie rät aber noch zur Vorsicht, da die Studie zu wenige Pat. eingeschlossen hat [23]. Insbesondere sind Langzeitverläufe noch abzuwarten. Erste Studien gibt es auch zum Einsatz der einseitigen MRgFUS-Behandlung bei der unilateral betonten PK. Eine unilaterale Behandlung des STN wurde nach einer ersten Pilotstudie [7] nun in einer Sham-kontrollierten randomisierten Studie über 3 Monate geprüft [6] und es gibt zu dieser Kohorte bereits eine 3-Jahres-Nachbeobachtung [24]. Das Verfahren hat sowohl die Dyskinesien und Fluktuationen und den Off-Zustand der kontralateralen Seite als auch die Lebensqualität verbessert. Die operationsbedingten Nebenwirkungen waren häufig, leicht bis mittelschwer – aber meist passager. Nach 1 Jahr persistierten Dyskinesien bei 2 Pat. Nach drei Jahren ist die Parkinson-Symptomatik immer noch deutlich gebessert, aber die Lebensqualität hat sich wieder auf den präoperativen Zustand verschlechtert. Für die einseitige MRgFUS-Pallidotomie wurde zunächst ebenfalls eine Pilotstudie mit guten Effekten auf Dyskinesien, Fluktuationen und Beweglichkeit publiziert [25]. Die

nachfolgende Sham-kontrollierten Studie hat ergeben, dass sich die Off-Symptome, die Fluktuationen und Dyskinesien bessern lassen [16], allerdings fiel diese Besserung gering aus und etwa 30% der behandelten Pat. hatten keinen Therapieeffekt. Die Lebensqualität wurde nicht untersucht. Beide Interventionen lassen eine Reduktion der L-Dopa-Dosis nicht zu.

Begründung der Empfehlung

Auf der Grundlage der aufgeführten Untersuchungen wird folgende Empfehlung gegeben.

Empfehlung	Neu<br>Stand (2023)
▪ Die Pallidotomie kann bei fortgeschrittener PK erwogen werden, wenn medikamentös schwer zu kontrollierende Wirkfluktuationen vorliegen und zugleich eine Therapie mittels THS oder einer Pumpentherapie nicht infrage kommt.<br>▪ Die Thalamo- und auch Subthalamotomie mittels Radiofrequenzablation soll bei der PK nicht durchgeführt werden.<br>▪ Die Behandlung mittels radiochirurgischer Verfahren (Gamma-Knife, Cyber-Knife) ist mangels Studien und aufgrund des potenziell hohen Komplikationsrisikos nicht zu empfehlen.<br>▪ Zum Einsatz des MRgFUS bei der Behandlung der PK werden gegenwärtig die notwendigen Studien durchgeführt. Zur Behandlung des Parkinson-Tremors mit unilateralem MRgFUS gibt es in Europa bereits eine Zulassung für alle Kerngebiete (VIM, STN, GPi). Allerdings sollte diese Intervention aktuell im Rahmen von Studien oder Registern durchgeführt werden.<br>▪ Alle ablativen Verfahren, die in dieser Leitlinie empfohlen werden, sollten nur unilateral eingesetzt werden.	
Konsensstärke: 100%, starker Konsens	

Fragestellung 176: Wie effektiv und sicher sind ablative Verfahren (Thermokoagulation, Gamma-/Cyberknife, fokussierter Ultraschall) im Vergleich mit der THS in der Behandlung der PK unter Berücksichtigung kurzfristiger (z.B. UPDRS) und mittelfristiger (z.B. QoL) Therapieeffekte?

Hintergrund

Während die ablativen Verfahren historisch häufig für die Therapie der fortgeschrittenen PK eingesetzt wurden, ist deren Anwendung seit Einführung der THS selten geworden [26]. Der MRgFUS stellt eine vielversprechende neue Therapie dar, die aber zunächst noch zahlreiche Studien zu ihrer Einführung benötigt. Der MRgFUS ist noch zu jung und zu wenig verbreitet.

Evidenzgrundlage

Studien, die die THS direkt mit ablativen Verfahren vergleichen, sind selten. Eine Arbeit von Schuurman et al. zeigte, dass VIM-THS und Thalamotomie für die Unterdrückung von arzneimittelresistentem Tremor gleichermaßen wirksam sind, aber dass die VIM-THS weniger Nebenwirkungen hat und zu einer größeren funktionellen Verbesserung führt [27]. Hinsichtlich der

Pallidotomie und Subthalamotomie existieren keine belastbaren Daten bzgl. der Effektivität und Sicherheit im Vergleich zur THS.

Ergebnis

Mangels belastbarer Studien ist es gegenwärtig schwierig, die Sicherheit und Effektivität des MRgFUS gegenüber der THS abzuwägen. Die Radiofrequenz-Thermoläsion ist der THS klar unterlegen, insbesondere aufgrund der häufigeren und schwerwiegenderen Nebenwirkungen.

Begründung der Empfehlung

Auf der Grundlage der aufgeführten Untersuchungen wird folgende Empfehlung gegeben.

**Empfehlung**	**Neu**<br>**Stand (2023)**
Die Anwendung des MRgFUS ist vielversprechend, kann aber aufgrund der begrenzten Datenlage zu dieser neuen Behandlungsform zur Behandlung der PK außerhalb von klinischen Studien gegenwärtig noch nicht empfohlen werden.	
Konsensstärke: 95,8%, starker Konsens	

Fragestellung 177: Welche Parkinson-Pat. kommen im Vergleich zur THS für die Anwendung ablativer Verfahren in Frage?

Hintergrund

Die THS und ablative Verfahren wirken mutmaßlich durch eine funktionelle (THS) bzw. strukturelle (Thermokoagulation, MRgFUS) Inhibition der Zielgebiete. Es ist daher von einer vergleichbaren neurophysiologischen Wirkung auf die lokale und Netzwerkfunktion auszugehen. Bei der Stimulation kann aber die Wirkung durch Stimulationsanpassung auch Jahre nach dem Eingriff noch modifiziert werden. Ob eine erhöhte Blutungsneigung (z.B. eine Therapie mit oralen Antikoagulantien oder eine Hämophilie) bei ablativen Verfahren weniger relevant ist, ist unklar. Besonders für Pat., für die eine THS nicht infrage kommt, kann der MRgFUS zur Behandlung ausgeprägter Symptome erwogen werden.

Evidenzgrundlage

Es liegen keine Studiendaten vor, die für eine Bevorzugung eines ablativen Verfahrens bei bestimmten Pat. sprechen. Aufgrund der besseren Steuerbarkeit und der genaueren Kenntnis der Wirkungen und Nebenwirkungen [8], [9], [10] ist die THS bei der PK den alten ablativen Verfahren (Radiofrequenzläsion und Radiochirurgie) vorzuziehen. Ein abschließender Vergleich mit dem MRgFUS ist mangels Daten noch nicht möglich.

Ergebnis

Eine evidenzbasierte Differenzialindikation gibt es nicht. Einzelfallentscheidungen für eine ablative Therapie sind hiervon unberührt.

Begründung der Empfehlung

Auf der Grundlage der aufgeführten Untersuchungen wird folgende Empfehlung gegeben.

Empfehlung	Neu<br><br>Stand (2023)
▪ Im Gegensatz zur THS, für die hochqualitative vergleichende Studien existieren, liegen insgesamt nur wenig oder keine randomisierten Studien zu ablativen Verfahren vor. Fehlen eindeutige Kontraindikationen, soll gegenwärtig primär die THS empfohlen werden.<br>▪ Zur Differenzialindikation sollte sowohl der Pat.-Wunsch als auch Kontraindikationen oder Nebendiagnosen herangezogen werden. Z.B. kann eine Therapie mit Immunsuppressiva die Wundheilung beeinträchtigen und somit ein ablatives Verfahren (z.B. MRgFUS) theoretisch begünstigen. Die Entscheidung muss im Einzelfall getroffen werden.	
Konsensstärke: 96%, starker Konsens	

Fragestellung 178: Welche Zielpunkte sind für die ablativen Verfahren auszuwählen bzw. effektiv bei der PK (VIM vs. STN vs. alternative Zielpunkte)?

Hintergrund

Durch interventionelle Therapien sind die gleichen Zielpunkte erreichbar wie mit der THS. Für die Therapie der Symptome der PK stehen prinzipiell der STN, das GPi sowie, im Fall von Tremor-Dominanz, der VIM zur Verfügung. Während für die Zielsymptome Rigor und Bradykinesie entweder GPi oder STN infrage kommen, ist beim Tremor der Thalamus theoretisch denkbar.

Evidenzgrundlage

Eine rezente Metaanalyse legt vergleichbare Tremor-suppressive Effekte aller Zielpunkte nahe [28]. Für die Ablation des STN und der GPi mittels MRgFUS existiert je eine kontrollierte, randomisierte Studie [6], [16].

Ergebnis

Die VIM-Thalamotomie scheint im Hinblick auf den Tremor ähnlich effektiv zu sein wie die Subthalamotomie. Die GPi-Ablation zeigte bei einem Drittel der Behandelten keine Wirkung, verglichen mit BMT.

Begründung der Empfehlung:

Auf der Grundlage der aufgeführten Untersuchungen wird folgende Empfehlung gegeben.

Empfehlung	Neu<br>Stand (2023)
▪ Von der Anwendung der Radiofrequenz-Thalamotomie oder- Subthalamotomie wird abgeraten.<br>▪ Von der Radiochirugie mit Gamma-Strahlen wird grundsätzlich abgeraten.<br>▪ Die Radiofrequenz-Pallidotomie sollte nur erwogen werden, wenn die THS oder Pumpentherapien nicht infrage kommen.<br>▪ Die Indikation zu einer MRgFUS-VIM-Ablation beim Parkinson-Tremor sollte zurückhaltend gestellt werden, da eine gute Tremor-Suppression mutmaßlich mit einer Therapie des STN erreichbar ist und weil initial Tremor-dominante Parkinson-Syndrome im Verlauf in einen Äquivalenztyp übergehen können; in diesem Fall wäre aber eine Therapie der bradyakinetischen Symptomatik bzw. im Verlauf hinzutretender Wirkfluktuationen durch die VIM-Ablation nicht möglich.<br>▪ Die Behandlung mit dem MRgFUS im STN oder im GPi sollte derzeit noch im Rahmen von Studien oder Registern durchgeführt werden.	
Konsensstärke: 95,8%, starker Konsens	

Referenzen

1. Spiegel EA, Wycis HT. Effect of thalamic and pallidal lesions upon involuntary movements in choreoathetosis. Trans Am Neurol Assoc. 1950;51:234-7

2. Spiegel EA, Wycis HT, Marks M, Lee AJ. Stereotaxic Apparatus for Operations on the Human Brain. Science. 1947;106(2754):349-50

3. Higuchi Y, Matsuda S, Serizawa T. Gamma knife radiosurgery in movement disorders: Indications and limitations. Mov Disord. 2017;32(1):28-35

4. Ohye C, Higuchi Y, Shibazaki T, Hashimoto T, Koyama T, Hirai T, et al. Gamma knife thalamotomy for Parkinson disease and essential tremor: a prospective multicenter study. Neurosurgery. 2012;70(3):526-35; discussion 35-6

5. Okun MS, Stover NP, Subramanian T, Gearing M, Wainer BH, Holder CA, et al. Complications of gamma knife surgery for Parkinson disease. Arch Neurol. 2001;58(12):1995-2002

6. Martínez-Fernández R, Máñez-Miró JU, Rodríguez-Rojas R, Del Álamo M, Shah BB, Hernández-Fernández F, et al. Randomized Trial of Focused Ultrasound Subthalamotomy for Parkinson's Disease. N Engl J Med. 2020;383(26):2501-13

7. Martínez-Fernández R, Rodríguez-Rojas R, Del Álamo M, Hernández-Fernández F, Pineda-Pardo JA, Dileone M, et al. Focused ultrasound subthalamotomy in patients with asymmetric Parkinson's disease: a pilot study. Lancet Neurol. 2018;17(1):54-63

8. Deuschl G, Follett KA, Luo P, Rau J, Weaver FM, Paschen S, et al. Comparing two randomized deep brain stimulation trials for Parkinson's disease. J Neurosurg. 2019;132(5):1376-84

9. Weaver F, Follett K, Hur K, Ippolito D, Stern M. Deep brain stimulation in Parkinson disease: a metaanalysis of patient outcomes. J Neurosurg. 2005;103(6):956-67

10. Weaver FM, Follett KA, Stern M, Luo P, Harris CL, Hur K, et al. Randomized trial of deep brain stimulation for Parkinson disease: thirty-six-month outcomes. Neurology. 2012;79(1):55-65

11. de Bie RM, de Haan RJ, Nijssen PC, Rutgers AW, Beute GN, Bosch DA, et al. Unilateral pallidotomy in Parkinson's disease: a randomised, single-blind, multicentre trial. Lancet. 1999;354(9191):1665-9

12. Vitek JL, Bakay RA, Freeman A, Evatt M, Green J, McDonald W, et al. Randomized trial of pallidotomy versus medical therapy for Parkinson's disease. Ann Neurol. 2003;53(5):558-69

13. Bond AE, Shah BB, Huss DS, Dallapiazza RF, Warren A, Harrison MB, et al. Safety and Efficacy of Focused Ultrasound Thalamotomy for Patients With Medication-Refractory, Tremor-Dominant Parkinson Disease: A Randomized Clinical Trial. JAMA Neurol. 2017;74(12):1412-8

14. Schlesinger I, Sinai A, Zaaroor M. Assessing Tremor and Adverse Events in Patients With Tremor-Dominant Parkinson Disease Undergoing Focused Ultrasound Thalamotomy. JAMA Neurol. 2018;75(5):632-3

15. Jung NY, Chang JW. Magnetic Resonance-Guided Focused Ultrasound in Neurosurgery: Taking Lessons from the Past to Inform the Future. J Korean Med Sci. 2018;33(44):e279

16. Krishna V, Fishman PS, Eisenberg HM, Kaplitt M, Baltuch G, Chang JW, et al. Trial of Globus Pallidus Focused Ultrasound Ablation in Parkinson's Disease. N Engl J Med. 2023;388(8):683-93

17. Laitinen LV, Bergenheim AT, Hariz MI. Ventroposterolateral pallidotomy can abolish all parkinsonian symptoms. Stereotact Funct Neurosurg. 1992;58(1-4):14-21

18. Baron MS, Vitek JL, Bakay RA, Green J, McDonald WM, Cole SA, DeLong MR. Treatment of advanced Parkinson's disease by unilateral posterior GPi pallidotomy: 4-year results of a pilot study. Mov Disord. 2000;15(2):230-7

19. Strutt AM, Lai EC, Jankovic J, Atassi F, Soety EM, Levin HS, et al. Five-year follow-up of unilateral posteroventral pallidotomy in Parkinson's disease. Surg Neurol. 2009;71(5):551-8

20. Kleiner-Fisman G, Lozano A, Moro E, Poon YY, Lang AE. Long-term effect of unilateral pallidotomy on levodopa-induced dyskinesia. Mov Disord. 2010;25(10):1496-8

21. Alomar S, King NK, Tam J, Bari AA, Hamani C, Lozano AM. Speech and language adverse effects after thalamotomy and deep brain stimulation in patients with movement disorders: A meta-analysis. Mov Disord. 2017;32(1):53-63

22. Schuurman PR, Bosch DA, Merkus MP, Speelman JD. Long-term follow-up of thalamic stimulation versus thalamotomy for tremor suppression. Mov Disord. 2008;23(8):1146-53

23. Deuschl G, Antonini A, Costa J, Śmiłowska K, Berg D, Corvol JC, et al. European Academy of Neurology/Movement Disorder Society - European Section guideline on the treatment of Parkinson's disease: I. Invasive therapies. Eur J Neurol. 2022;29(9):2580-95

24. Martínez-Fernández R, Natera-Villalba E, Máñez Miró JU, Rodriguez-Rojas R, Marta Del Álamo M, Pineda-Pardo J, et al. Prospective Long-term Follow-up of Focused Ultrasound Unilateral Subthalamotomy for Parkinson Disease. Neurology. 2023;100(13):e1395-e405

25. Eisenberg HM, Krishna V, Elias WJ, Cosgrove GR, Gandhi D, Aldrich CE, Fishman PS. MR-guided focused ultrasound pallidotomy for Parkinson's disease: safety and feasibility. J Neurosurg. 2020;135(3):792-8

26. Lozano AM, Lipsman N, Bergman H, Brown P, Chabardes S, Chang JW, et al. Deep brain stimulation: current challenges and future directions. Nat Rev Neurol. 2019;15(3):148-60

27. Schuurman PR, Bosch DA, Bossuyt PM, Bonsel GJ, van Someren EJ, de Bie RM, et al. A comparison of continuous thalamic stimulation and thalamotomy for suppression of severe tremor. N Engl J Med. 2000;342(7):461-8

28. Lin F, Wu D, Yu J, Weng H, Chen L, Meng F, et al. Comparison of efficacy of deep brain stimulation and focused ultrasound in parkinsonian tremor: a systematic review and network meta-analysis. J Neurol Neurosurg Psychiatry. 2021

3.22 Differenzialindikation invasiver Therapien

Autoren: Thomas Köglsperger, René Reese, Günther Deuschl, Christoph Schrader

In diesem Kapitel werden für die Praxis sehr wichtige Fragen behandelt, die leider nur mit offenen Studien oder sogar allein auf der Grundlage eines Expertenkonsensus beantwortet werden können, da randomisierte, prospektive Vergleichsstudien unter den verschiedenen invasiven Interventionen fehlen. Indikationsstellungen setzen besondere Erfahrungen mit den Therapiemöglichkeiten voraus und werden typischerweise in den Spezialkliniken vorgenommen. In dieser Leitlinie wird daher vor allem die Frage beantwortet, wann an den Einsatz der verschiedenen Methoden gedacht und die Zusammenarbeit mit den Spezialkliniken gesucht werden sollte. Diese Interventionen können die Lebensqualität bei geeigneten Pat. gegenüber der oralen/transkutanen Behandlung z.T. erheblich verbessern, daher sollte ihr Einsatz sie nicht erst als Ultima ratio, sondern frühzeitig beim Eintreten von ersten motorischen Komplikationen (Wirkfluktuationen, Dyskinesien) mit den Pat. erwogen werden.

Fragestellung 179: Welches sind die differenziellen Indikationen und Kontraindikationen/Ausschlusskriterien der invasiven Therapien (Pumpentherapien, Tiefe Hirnstimulation, ablative Verfahren) in der Behandlung der PK?

Hintergrund

Die fortgeschrittene PK ist durch das Kernproblem der motorischen Komplikationen (hyper- und hypokinetische Wirkfluktuationen und Dyskinesien) gekennzeichnet. Damit gehen ebenso wichtige nicht motorische Symptome einher, u.a. ein breites Spektrum psychiatrischer Manifestationen oder autonomer Symptome, aber auch eine reduzierte Lebensqualität, eine zunehmende psychosoziale Belastung, Multimorbidität, Polypharmazie u.v.a. Für die Therapie der motorischen Komplikationen kommen unterschiedliche Behandlungsformen in Betracht. Diese umfassen die Tiefe Hirnstimulation (THS), die Pumpentherapien und sogenannte ablative Therapien, nämlich die Radiofrequenz-Thermokoagulation des Thalamus und des Globus pallidus internus (GPi) sowie die MR-gesteuerte Ablation mit fokussiertem Ultraschall (MRgFUS).

Ablative Behandlungen wurden ursprünglich eingesetzt, weil wirksame Medikamente zur Behandlung von Bradykinesie und Tremor fehlten. Mit der Verfügbarkeit von Levodopa (L-Dopa) in den 1970er-Jahren wurden aber Läsionsoperationen weitgehend aufgegeben, mit Ausnahme weniger unilateraler Pallidotomien bei Dyskinesien/Akinese/Rigor und Thalamotomien bei Tremor. Das Auftreten von Wirkfluktuationen/Dyskinesien unter dopaminerger Therapie in den 80er-Jahren belebte erneut die Suche nach wirksamen Interventionen. Zunächst wurden vor allem in den USA die Pallidotomien wiederentdeckt, und in der Folge erlebte die Anfang der 1990er-Jahre entdeckte THS einen steilen Aufstieg. Aufgrund des günstigeren Nebenwirkungsprofils der THS im Vergleich zu den älteren ablativen Verfahren [1] hat sich diese zum am häufigsten durchgeführten invasiven Eingriff entwickelt. Tatsächlich haben die klassischen ablativen Therapien keine Relevanz mehr bei der Therapie der PK und werden daher hier nicht weiter besprochen. Sie werden zukünftig möglicherweise abgelöst durch die Einführung des MRgFUS. Hierbei handelt es sich um eine inzisionslose, aber dennoch läsionelle Therapie, die vor allem bei essenziellem Tremor getestet

wurde. Studien zur PK stehen gerade erst am Anfang. Diese Technik wird derzeit im Thalamus eingesetzt, es wurde jedoch kürzlich auch über eine Ablation des STN und GPi berichtet [2], [3]. Für eine detaillierte Darstellung und Empfehlungen wird auf das Kapitel „Ablative Therapien“ verwiesen.

Zusätzlich stehen derzeit drei pharmakologische Möglichkeiten zur Verfügung, motorische Komplikationen mittels einer kontinuierlichen Zufuhr von dopaminergen Substanzen über Pumpensysteme zu behandeln, nämlich die kontinuierliche subkutane Apomorphin-Infusion (CSAI) [4], die dauerhafte perkutanen Levodopa/Carbidopa-Intestinal-Gel (LCIG)-Infusion [5] und die Levodopa/Entacapon/Carbidopa-Intestinalgel (LECIG)-Infusion, wobei die Gele (LCIG und LECIG) über eine perkutan endoskopische Gastrostomie mit jejunaler Extension (PEG-J) direkt jejunal infundiert werden, sowie neuerdings die kontinuierliche subkutane Foslevodopa/Foscarbidopa-Infusion (CSFLI) [6], die voraussichtlich demnächst (vermutlich noch in 2023) verfügbar sein wird. Für Letztere gibt es deshalb bislang nur limitierte mittel- oder langfristigen Daten oder klinisch-praktische Erfahrungen [6a].

Alle oben aufgeführten Verfahren, insbesondere die THS und die Pumpenverfahren, sind zugelassen für die Behandlung motorischer Fluktuationen, die sich mit oraler/transkutaner Medikation nicht ausreichend behandeln lassen. Die Konstellationen von nicht motorischen Symptomen können in der fortgeschrittenen Phase der PK mit motorischen Fluktuationen sehr unterschiedlich sein, einige sind sogar ein Ausschlusskriterium für ein jeweiliges invasives Verfahren; mithin sind die Profile der Pat., die von einer jeweiligen Behandlung gut profitieren können, nicht immer identisch. In diesem Kapitel soll deshalb die Evidenz für differenzielle Indikationen und Kontraindikationen/Ausschlusskriterien der verschiedenen invasiven Therapien dargestellt werden.

Evidenzgrundlage

Für die Bewertung der THS existieren mehrere kontrollierte, randomisierte Studien gegen Standard-(Medikamenten-)Behandlung [7], [8], [9]. Für die Bewertung der Wirksamkeit der LCIG-Therapie existieren zwei randomisierte, kontrollierte Studien gegen Standardbehandlung (Best Medical Treatment, BMT), wobei eine Studie (DYSCOVER) nur auf Clinical Trials publiziert wurde [10]. Für die LECIG-Therapie existiert eine Beobachtungsstudie [11]. Für die CSFLI-Therapie existiert eine kontrollierte, randomisierte Studie gegen BMT [6], die eine mit der THS vergleichbare Verbesserung von Wirkfluktuationen/Dyskinesien zeigt. Zum direkten Vergleich der Pumpentherapien untereinander sowie der Pumpentherapien mit der THS existieren lediglich Open-label-Studien [12], [13], [14], [15], [16], [17], [18]. Zum Vergleich von LCIG und THS [13], [14], [19], [20] sind nur Open-label-Studien verfügbar: Es existieren nicht randomisierte prospektive Beobachtungsstudien zum Vergleich von LCIG mit CSAI [21] und zum Vergleich von LCIG, CSAI und THS [12]. Zudem existiert eine randomisierte, open-label, Crossover-Studie zum Vergleich von LCIG und CSAI [22].

Ergebnis

Allgemeine bzw. verfahrensspezifische Aspekte: Im Einzelfall muss entschieden werden, welches Verfahren am besten geeignet, d.h. am besten wirksam und verträglich ist. Dazu können allgemeine bzw. verfahrensspezifische Aspekte relevant sein. Die Infusionstherapien wirken beispielsweise nur während der Dauer der Medikamentengabe, die nächtlich meist ausgesetzt wird (Ausnahme CSFLI),

während die THS und die ablativen Verfahren im gesamten Tagesverlauf wirken. Zudem ist natürlich die Präferenz des Pat. entsprechend zu würdigen.

Ein grundsätzlicher Aspekt bei der Auswahl der Behandlung ist die Frage, ob bei der Symptomenkonstellation der Pat. die medikamentöse Überdosierung mit Dopaminergika eine bedeutsame Rolle spielt (z.B. psychiatrische Nebenwirkungen der dopaminergen Therapie). Die einzige invasive Therapie, die eine Reduktion der Medikation erlaubt, ist die STN-THS, sodass in diesem Fall die THS möglicherweise zu favorisieren wäre. Andererseits ist zu beachten, dass z.B. Halluzinationen häufig mit einer verminderten Frontalhirnfunktion assoziiert sind und diese wiederum als negativer Prädikator für ein gutes Langzeitergebnis nach THS gilt [23].

CSAI und LCIG sind hinsichtlich der motorischen Symptome mutmaßlich ähnlich wirksam wie die THS, mit Ausnahme des Tremors, der auf THS wahrscheinlich besser anspricht [12], [13], [14], [15], [16], [17], [18]. Insgesamt ist die Evidenz einschließlich Langzeitdaten für die THS aktuell am umfangreichsten [24]. Neben der Wirksamkeit auf die motorischen Symptome ist u.a. die Würdigung nicht motorischer Symptome von Bedeutung, da alle Verfahren nicht motorische Symptome in leicht unterschiedlicher Ausprägung beeinflussen können [12], [25] (Tab. 23).

Tab. 23: Der Einfluss verschiedener Interventionen auf wichtige dimensionsübergreifende Outcome-Parameter und auf motorische Symptome

Item	Domäne	CSAI	LCIG	LECIG	CSFLI	THS	MRgFUS (unilateral)
Lebensqualität (PDQ-39; PDQ-8)	QOL	-<br>[26]<br>[27]	+<br>[28]	?	?	++<br>[29]<br>[30]	++<br>[3]
Aktivitäten des täglichen Lebens (ADL; UPDRS II)	ADL	+<br>[31]	+<br>[32]	?	++<br>[6]	++<br>[33]<br>[34]<br>[35]<br>[36]<br>[37]	++<br>[3] (STN)<br>n.s.<br>[38] (GPi)
Motorische Funktion im Off; MED-Off; (UPDRS III); Off-Zeit	MS	++<br>[39]<br>+<br>[40]	++<br>[10]	+<br>[11]	++<br>[6]	++<br>[33]<br>[34]<br>[35]<br>[36]<br>[37]	++<br>[3] (STN)<br>[38] (Gpi)<br>[41] (VIM)
Dyskinesien und Fluktuationen (UPDRS IV)	MS	++<br>[39]	++	+	++	++<br>[33]	++

		+<br><br>[40]	[10]	[11]-	[6]	[34]<br>[35]<br>[36]	[38] (Gpi)
Kardiovaskulär (einschl. Stürze/Orthostase)	NMS[3]	+/-<br><br>[21]<br>[27]	+<br><br>[42]	?	?	-	?
Schlaf/Fatigue	NMS	++<br><br>[43]<br>+<br><br>[12]<br>[27]<br>[21]	+/-<br><br>[12]<br>[44]<br>[45]<br>[46]<br>[47]<br>[48]<br>[21]<br>[49]	?	?	+<br><br>[50]<br>[51]<br>[12]<br>[52]<br>[53]	?
Stimmung/Kognition	NMS	+<br><br>[12]<br>[27]<br>[21]	+/-<br><br>[12]<br>[48]<br>[21]<br>[49]	?	?	++<br><br>[54]	?
Wahrnehmungsprobleme/<br>Halluzinationen[2]	NMS	+<br><br>[12]<br>[27]<br>[55]<br>[21]	+/-<br><br>[48]<br>[21]<br>[49]	?	?	+<br><br>[12]	?
Aufmerksamkeit/<br>Gedächtnis	NMS	+<br><br>[12]<br>[27]<br>[21]	+/-<br><br>[48]<br>[21]<br>[49]	?	?	+<br><br>[7]<br>[8]<br>[9]	?
Gastrointestinale Funktionen	NMS	+<br><br>[27]<br>[21]	+/-<br><br>[12]	?	?	+<br><br>[56]<br>[52]<br>[7]<br>[8]<br>[9]	?

Urogenitale Funktionen	NMS	+ [57] [58] [27] [21]	+/- [48] [21] [49]	?	?	+ [12] [52] [7] [8] [9]	?
Sexuelle Funktionen	NMS	- [27] [21]	+/- [48] [21] [49]	?	?	+ [12] [7] [8] [9]	?
Sonstiges	NMS	+ [12] [27]	+/- [12]	?	?	+ [12] [7] [8] [9]	?

QOL = Lebensqualität, ADL = Aktivitäten des täglichen Lebens, MS = motorische Symptome, NMS = nicht motorische Symptome; CSAI = kontinuierliche subkutane Apomorphin-Infusion; LCIG = Levodopa/Carbidopa Intestinalgel; LECIG = Levodopa/ Entacapon/Carbidopa-Intestinalgel; CSFLI = kontinuierliche subkutane Foscarbidopa/Foslevodopa-Infusion; THS = Tiefe Hirnstimulation; MRgFUS = MR-gesteuerter fokussierter Ultraschall.

- \- in offenen oder kontrollierten Studien keine Wirksamkeit oder Verschlechterung
- ? keine Studien oder keine positiver Expertenkonsens
- \+ nach Expertenmeinung oder offenen Studien gebessert
- ++ nach kontrollierten Studien gebessert

[1]Der MRgFUS im VIM kann aktuell eingeschränkt für ein einseitiges Parkinson-Syndrom mit Tremor-Dominanz empfohlen werden. Die Indikation zu einer VIM-Ablation bei der PK sollte allerdings zurückhaltend gestellt werden, da eine gute Tremor-Suppression auch mit einer DBS oder Läsion des STN erreichbar ist und weil initial Tremor-dominante Parkinson-Syndrome im Verlauf in einen Äquivalenztyp übergehen; in diesem Fall wäre aber eine Therapie der brady-/hypokinetischen Symptomatik bzw. im Verlauf hinzutretender Wirkfluktuationen durch die VIM-Ablation nicht gewährleistet. Ob ein MRgFUS des STN einen Parkinson-assoziierten Tremor im Vergleich zur THS ausreichend und nachhaltig bessert, ist gegenwärtig unklar.

[2]Bei Halluzinationen sollte berücksichtigt werden, ob es sich dabei um eine L-Dopa-induzierte oder um eine unabhängige Symptomatik handelt.

[3]Die nicht motorischen Symptome (NMS) geben die Kategorien der Non-Motor Symptom Scale (NMSS) wieder, die zur Bewertung nicht motorischer Symptome in Studien üblich ist [59].

Motorische Symptome:

Die THS ist nachweislich den anderen Verfahren bei L-Dopa-refraktärem Tremor überlegen, wobei STN- und GPi-THS ähnlich effektiv hinsichtlich der Tremor-Suppression zu sein scheinen [9]. Für alle anderen Pat. existieren keine kontrollierten, randomisierten Studien zum Vergleich von THS mit

Pumpentherapien sowie der Pumpentherapien untereinander [21], [12], [22], [13], [14], [15], [16], [17], [19], [20]). Insgesamt besserten alle untersuchten Verfahren die motorischen Wirkfluktuationen/Dyskinesien gut, wobei LCIG und THS, verglichen mit der CSAI, etwas stärkere Effekte auf die Off-Zeit zu haben scheinen, wenn man die verfügbaren Open-label-Studien wichtet [28]. Teilweise wurden unterschiedlich zusammengesetzte und unterschiedlich große Kohorten verglichen. In der EuroInf2-Studie unterschieden sich die Gruppen signifikant hinsichtlich des Alters bei Therapiebeginn (THS 61,5 ± 9,5, LCIG 65,4 ± 8,8, CSAI 61,6 ± 9,7 Jahre), der Krankheitsdauer bei Therapiebeginn (DBS 10,7 ± 4,8, LCIG 14,6 ± 5,3, CSAI 13,6 ± 5,6 Jahren) sowie dem Schweregrad der nicht motorischen Symptome, gemessen mit der Non-Motor Symptom Scale (THS 55,8 ± 32,5, LCIG 84,6 ± 45,1, CSAI 75,8 ± 53,6), was nahelegt, dass Krankheitsdauer und Ausmaß der nicht motorischen Symptome eine entscheidende Rolle bei der Auswahl der Pat. spielen [12]. Alle Pumpentherapien sind pflegerisch aufwendig und benötigen bei kognitiven Störungen Betreuung.

Nicht motorische Symptome:

Alle Verfahren haben überwiegend günstige Effekte auf die nicht motorische Symptome, wobei prospektive nicht randomisierte Verlaufsuntersuchungen teilweise geringe Unterschiede nahelegen hinsichtlich der Effekte der Verfahren auf nicht motorische Symptome. In der EuroInf-2-Studie ergab sich z.B. im Vergleich von LCIG, CSAI und THS folgendes Ergebnis bezüglich der nicht motorischen Symptome: In der STN-THS-Gruppe wurden signifikante positive Auswirkungen auf Schlaf/Müdigkeit, Stimmung/Kognition, Wahrnehmungsprobleme/Halluzinationen, urogenitale Symptome, sexuelle Funktionen und den Bereich „Sonstiges" gefunden. In der letztgenannten Gruppe gab es positive Effekte für Schmerz sowie Geruchs- und Geschmacksstörungen sowie übermäßiges Schwitzen. In der LCIG-Gruppe fand sich eine signifikante Verbesserung des Schlafs, der Stimmung/Kognition, der gastrointestinalen Symptome und im Bereich „Sonstiges", was durch Geruchs- und Geschmacksstörungen bedingt war. In der CSAI-Gruppe zeigte sich eine signifikante Verbesserung der Stimmung/Kognition, der Wahrnehmungsprobleme/Halluzinationen, der Aufmerksamkeit/des Gedächtnisses und ebenfalls im Bereich „Sonstiges" (Schwitzen) [12]. Bei der THS hängen Art und Ausmaß der nicht motorischen Effekte u.a. von Elektrodenposition und Stimulationsparametern ab [60].

Neben diesen Daten existieren teils hochqualitative Daten zu den verfahrensspezifischen Effekten auf die nicht motorischen Symptome. Die CSAI verbesserte in einer Beobachtungsstudie neben motorischen Symptomen und Lebensqualität auch insbesondere Schlaf, Stimmung, gastrointestinale und urogenitale Symptome und Fatigue, wobei die Effekte für Schlaf und Lebensqualität als ausgeprägt beurteilt wurden [27]. Zu den Effekten von nächtlichem CSAI existiert inzwischen auch eine randomisierte, kontrollierte Studie, die positive Effekte auf den Nachtschlaf anhand der Parkinson's Disease Sleep Scale (PDSS) bei fortgeschrittener PK belegt [43] und diese Einschätzung wird durch weitere Open-label-Studien gestützt [61], [62], [63], [64]. Bereits seit Längerem legen Open-label-Studien einen positiven Effekt der CSAI auf die urogenitale Funktion bei der PK nahe [57], [58], [27]. Zur LCIG existieren ebenfalls Open-label-Studien, die die Wirksamkeit der Therapie für Schlafstörungen nahelegen [44], [45], [46], [47], [48].

Symptomkonstellationen, die gegen eine Intervention sprechen oder sprechen können (absolute/relative Kontraindikationen):

Die Effekte der Verfahren auf motorische und nicht motorische Symptome können als Entscheidungshilfe dienen, nur wenige Kriterien bedingen einen Ausschluss eines Verfahrens. Bestimmte Konstellationen können einen intrazerebralen oder intraabdominellen Eingriff verbieten. Dazu kann eine zu ausgeprägte Hirnatrophie gehören (THS) oder ein schwerer Diabetes mit häufigen Allgemeininfektionen (LCIG, LECIG). Im Einzelfall muss diesbezüglich ein interdisziplinärer Konsens erfolgen, wozu sich interdisziplinäre Indikationskonferenzen als hilfreich erwiesen haben.

Alter:

Früher wurden feste Altersgrenzen für den Einsatz invasiver Therapien formuliert, insbesondere für die THS. Naturgemäß ist das nicht sachgerecht und sollte durch individuelle Betrachtung der Operabilität, Therapienotwendigkeit und Erfolgserwartungen abgelöst werden.

Kognition:

Kognitive Störungen sind bei dieser Pat.-Gruppe häufig und eine neuropsychologische Untersuchung ist zur Indikationsstellung erforderlich. Grenzwerte anerkannter Messinstrumente (z.B. MoCA, MME; Mattis-Demenz-Skala) wurden in der Vergangenheit postuliert, sind aber nicht durch Daten gedeckt. Grundsätzlich sind die Aussichten für die motorische Besserung bei kognitiven Einschränkungen nicht generell schlechter, die Entscheidung ist abhängig von der Symptomschwere und der Intervention [65]. Kurz- und langfristige Daten über die Kognition nach THS sind umstritten; einige Autoren/Autorinnen vermuten eine gewisse negative Auswirkung von THS auf die Kognition [66], [67], [68], insbesondere in den Domänen Exekutivfunktion und Wortflüssigkeit, wohingegen andere keine [69], [70], [71] oder sogar positive Auswirkungen sehen [72]. Eine retrospektive Case-control-Studie hat bei Pat. mit moderater kognitiver Störung vs. Pat. ohne kognitive Störungen vergleichbar gute motorische Effekte gefunden [73]. Kontrollierte randomisierte Studien haben bei der üblichen Studienpopulation kognitiv gesunder Pat. über einen Zeitraum von bis zu 2 Jahren keine Verschlechterung kognitiver Funktionen außer einer verminderten Wortflüssigkeit gefunden. Offene Studien zeigen kein einheitliches Bild der postoperativen Verschlechterung. Im natürlichen Verlauf der Erkrankung ist das Auftreten einer Demenz so häufig, dass angemessene Vergleichsgruppen zwingend erforderlich sind. Einzelfallabwägungen sind daher einer generellen Verweigerung vorzuziehen [65]. Unterschiede in den Zielpunkten scheint es nicht zu geben [74]. Tatsächlich schwanken Prävalenz und Inzidenz von Demenz bei Pat. mit THS in den verschiedenen Zentren weitgehend, da es an Langzeitstudien mit großen Bevölkerungsgruppen und Kontrollgruppen mangelt [75]. Der Effekt der THS auf die Kognition ist in Beobachtungs- und Kohortenstudien nicht eindeutig, und, wenn überhaupt vorhanden, auf wenige einzelne Domänen beschränkt (rev. in [76], [77], [78]), wozu es auch Daten aus kontrollieren, randomisierten Studien gibt [68]. Schließlich bleibt es ungewiss, ob es potenzielle Faktoren gibt, die das Demenzrisiko nach THS erhöhen, und ob sie von THS oder der zugrunde liegenden Pathologie abhängen. Insgesamt sollte aber in der Gesamtkonstellation einer Demenz von einer THS abgesehen werden. Die Diagnosekriterien des ICD/DSM können als Orientierung gelten, nachdem spezifische Cut-off-Werte fehlen. Obwohl die Kognition bei der Indikationsstellung vor THS mehr Beachtung erfahren hat, ist auch für die

Pumpentherapie die kognitive Funktion relevant; besonders die Dauerinfusionstherapien können bei dementen oder verwirrten Pat. undurchführbar sein. Im Zweifelsfall ist die Unterstützung durch das soziale Umfeld (Betreuungspersonen) zu bewerten. In der klinischen Praxis sollten regelhaft alle alternativen Therapien für den/die individuelle/n Pat. abgewogen werden.

Psychische Störungen:

Ähnliches gilt für (schwere oder rezidivierende) psychische Störungen. Schwere Depressionen, Suizidalität oder Psychosen sollten weiterhin als Ausschlusskriterium für die THS gelten [79]. Zu dieser Frage gibt es allerdings keine Daten, die Einschätzung beruht auf einem Expertenkonsens. Bei der Impulskontrollstörung (ICD) gibt es Hinweise aus einer kontrollierten Studie [80] für einen günstigen Effekt, der durch die Reduktion der dopaminergen Medikation vermittelt ist. In dieser Studie wurde gezeigt, dass hyperdopaminerge und hypodopaminerge Fluktuationen signifikant gebessert werden. Die Reduktion von ICD war nicht signifikant, da zu wenige ICD-Pat. eingeschlossen wurden. Insgesamt scheint die THS, wichtet man zudem verfügbare Beobachtungsstudien, einen günstigen Effekt zu haben auf die ICD [81], [54]. Beobachtungsstudien legen auch für die Therapie mit LCIG einen günstigen Einfluss auf ICD nahe [82], [83]. Die Therapie mit CSAI erscheint sicher bei ICD [21], wobei in einer sonst vergleichbaren Konstellation die LCIG mutmaßlich günstiger zu sein scheint [84].

Gang- und Gleichgewichtsstörungen:

Bei Gang- und Gleichgewichtsstörungen ist für die THS entscheidend, ob es sich um einen L-Dopa-responsive Symptomatik handelt. Bestehen L-Dopa-unabhängige Gang- und Gleichgewichtsstörungen, können diese nicht mit THS behandelt werden [85], [86]. Grundsätzlich gilt das auch für die Pumpentherapien.

Begründung für die Empfehlung

Randomisierte, kontrollierte Studien zur Differenzialindikation gibt es nicht. Die Empfehlungen für die Differenzialindikation beruhen deshalb einerseits auf Daten aus den randomisierten, kontrollierten Studien zur Wirksamkeit der jeweiligen Verfahren im Vergleich mit der oralen/transdermalen Therapie, andererseits auf Resultaten von Open-label-Studien, die verschiedene Verfahren gegeneinander vergleichen. In beiden Fällen sind die Pat.-Kohorten aber teilweise nur eingeschränkt vergleichbar (u.a. wegen Unterschieden in Krankheitsdauer, Alter bei Therapiebeginn und Selektionskriterien). In der Vergangenheit wurden bereits Konsensuskriterien erarbeitet [87], [88], [89], [25], die für die Differenzialindikation herangezogen werden können. Die vorliegende Empfehlung beruht auf Quervergleichen der randomisierten, kontrollierten Studien, offenen Vergleichsstudien und auf Expertenkonsens.

Empfehlung	Neu<br>Stand (2023)
▪ Prinzipiell sollten invasive Verfahren insbesondere dann erwogen werden, wenn beeinträchtigende Levodopa-abhängige Wirkfluktuationen auftreten, die sich durch eine Optimierung der oralen/transdermalen Therapie nicht ausreichend verbessern lassen.<br>▪ Die Entscheidung für ein bestimmtes Verfahren soll neben der Wirksamkeit auf die motorische Symptomatik insbesondere auch nicht motorischen Symptome und Pat.-Charakteristika sowie die individuelle Präferenz der Pat. mit einbeziehen (Tab. 23), wobei die Faktoren im Einzelfall zu gewichten sind und in einer interdisziplinären Fallkonferenz und zusammen mit den Pat. abgewogen werden sollen.	
Konsensstärke: 100%, starker Konsens	

Fragestellung 180: Welches sind geeignete Zeitpunkte der differenziellen Indikationsstellung für die invasiven Therapien (Pumpentherapien, Tiefe Hirnstimulation, ablative Verfahren) im klinischen Verlauf der PK?

Hintergrund

In der frühen Phase der PK sind orale/transdermale therapeutische Strategien gewöhnlich effektiv, um eine gleichmäßige Symptomkontrolle zu gewährleisten. Auch die Entwicklung motorischer Fluktuationen lässt sich durch verschiedene Maßnahmen (lang wirksame Dopaminagonisten, COMT- und MAO-B-Inhibition, Fraktionierung von Levodopa) eine Zeit lang kompensieren, aber im weiteren Verlauf der fortgeschrittenen Erkrankung lassen sich mit diesen Maßnahmen motorische und nicht motorische Fluktuationen sowie Dyskinesien zusehends schlechter kontrollieren. In diesem fortgeschrittenen Krankheitsstadium ist es wichtig, solche Pat. zu einem Spezialisten zu überweisen, bevor die Lebensqualität sich verschlechtert und sich Komplikationen infolge der fortgeschrittenen Erkrankung entwickeln [90]. Eine besondere Stellung nimmt medikamentenrefraktärer Tremor ein. Die invasiven Verfahren CSAI, LCIG, LECIG und THS sind zugelassen für die Behandlung konservativ nicht ausreichend zu behandelnder motorischer Fluktuationen. CSFLI wird voraussichtlich im 4. Quartal 2023 verfügbar sein. In diesem Kapitel soll erörtert werden, zu welchem Zeitpunkt der Erkrankung ein invasives Therapieverfahren als Eskalation in Betracht gezogen werden sollte.

Evidenzgrundlage

In der Literaturrecherche für diese Fragestellung fanden sich:

- N = 0 randomisierte, kontrollierte Studien
- N= 1 longitudinale größere Kohortenstudien
- N= 2 Expertenkonsense im Delphiverfahren.

Ergebnis

Kontrollierte Studien existieren zu diesem Thema nicht. Es finden sich lediglich 2 Arbeiten mit im Delphiverfahren ermittelten Expertenempfehlungen und eine Kohortenstudie, die die Konsensus-

Empfehlungen hinsichtlich der Therapiewirklichkeit geprüft hat. Das Programm NAVIGATE PD wurde 2012 etabliert, um existierende Leitlinien zu ergänzen und Empfehlungen zum Management der PK in dem Stadium zu entwickeln, in dem orale/transdermale Therapie nicht mehr ausreichend wirksam ist. Das Programm befragte 103 europäische Expertinnen/Experten in einem Delphiverfahren zum geeigneten Zeitpunkt, zu dem ein invasives Therapieverfahren in Betracht gezogen werden sollte.

Nicht invasive Therapiemaßnahmen können als unzureichend eingeschätzt werden, wenn die Lebensqualität wegen motorischer Fluktuationen mit oder ohne Dyskinesien beeinträchtigt ist und sowohl der behandelnde Arzt/die betreffende Ärztin als auch der Patient/die Patientin dahingehend übereinstimmen, dass eine nicht invasive Behandlung nicht länger effektiv ist. Ein adäquater Versuch nicht invasiver Behandlung sollte Levodopa und – sofern nicht kontraindiziert – Dopaminagonisten, MAO-B- und COMT-Hemmer eingeschlossen haben. Eine Überweisung zu einem Spezialisten für invasive Verfahren sollte in Erwägung gezogen werden, wenn Levodopa mehr als 5 x/Tag (in etwa Einnahmeintervallen von < 3 Std. entsprechend) erforderlich ist, obwohl die Anzahl der Dosen nicht relevant ist, wenn sie von Pat. toleriert wird und eine adäquate Reduktion der Off-Zeit erreicht wird. Pat., die trotz optimierter oraler/transdermaler Behandlung tagsüber mehr als 1<<-2 Stunden Off-Phasen durchleben, sollten als Kandidaten für invasive Therapieverfahren angesehen werden. Neben Einnahmeintervallen sollen (schmerzhafte) Dystonien und Off-Freezing als Indikatoren berücksichtigt werden. Zudem sind Levodopa-abhängige nicht motorische Fluktuationen zu berücksichtigen.

Der Schweregrad und die Qualität von Off-Perioden sind gleichermaßen bedeutsam. Pat. mit schweren Off-Symptomen sollten für ein invasives Therapieverfahren in Betracht gezogen werden, selbst wenn die gesamte (kumulative) Off-Zeit pro Tag akzeptabel erscheint. Sollten die Symptome weiterhin refraktär sein oder sich nicht tolerierbare Nebeneffekte entwickeln, erscheint orale/transdermale Therapie ebenfalls nicht effektiv, und weitere medikamentöse Anpassungen sind sehr unwahrscheinlich effektiv. Motorische Fluktuationen, die von störenden Dyskinesien begleitet werden, die sich nicht mit zusätzlichem Amantadin (100<<-400 mg/Tag) behandeln lassen, werden für gewöhnlich auch als Indikation für eine invasive Behandlung angesehen. Wenn Pat. diese Kriterien erfüllen, sollte eine Zuweisung in Erwägung gezogen werden, auch für Pat., deren Krankheitsdauer weniger als 4 Jahre beträgt [87].

2014/2015 erfolgte eine weitere Konsensbildung unter 17 internationalen Bewegungsstörungsspezialisten in einem Delphiverfahren, dessen Ziel war, klinische Indikatoren für die Identifikation und das Management der fortgeschrittenen PK zu entwickeln.

Folgende Konsensusempfehlungen wurden gebildet: Motorische Fluktuationen, nicht motorische Symptome und funktionelle Beeinträchtigung definieren (einzeln oder in Kombination) eine fortgeschrittene PK und sind die Voraussetzung für die Evaluationen für ein gerätegestütztes Verfahren. Die Schlüsselindikatoren für eine fortgeschrittene PK waren 1) motorische Beeinträchtigung (moderat störende motorische Fluktuationen, ≥1 Stunde störende Dyskinesien pro Tag, ≥ 2 Stunden Off-Symptome pro Tag und ≥ 5 Einnahmezeitpunkte Levodopa/d – „5-2-1-Kriterien“); 2) nicht motorische Symptome (milde Demenz, nicht transitorische störende Halluzinationen; 3) funktionelle Beeinträchtigung (wiederholte Stürze trotz optimaler Behandlung, Beeinträchtigung der Aktivitäten des täglichen Lebens). Pat. mit gutem Ansprechen auf Levodopa,

guter Kognition und einem Alter < 70 können mit allen invasiven Verfahren effektiv behandelt werden. Pat. mit störenden Dyskinesien wurden als gute Kandidaten für LICG oder THS eingeschätzt. Pat. mit therapieresistentem Tremor wurden als gute Kandidaten für THS eingeschätzt [88].

Die 5-2-1-Kriterien spiegeln hinsichtlich der Behandlung mit LCIG die Behandlungswirklichkeit wider: von 139 über 6 Monate mit LCIG behandelten Pat. in der DUOGLOBE-Studie erfüllten retrospektiv 98% mindestens 1 und 68 % 2–3 Kriterien [91]. Analysen der 5-2-1-Kriterien zur Indikationsstellung zur Behandlung mit THS und CSAI existieren noch nicht.

Begründung der Empfehlung

Die Empfehlung beruht auf 2 Konsensuspapieren und einer Kohortenstudie, in der die Behandlungskriterien retrospektiv in der Behandlungswirklichkeit geprüft wurden.

**Empfehlung**	**Neu**<br><br>**Stand (2023)**
▪ Parkinson-Pat. sollten spätestens beim Auftreten erster Fluktuationen über die Möglichkeit invasiver Behandlungen informiert werden.<br>▪ Bei Pat. mit einer Parkinson-Krankheit und mindestens einem der folgenden Kriterien soll die Indikation für ein invasives Verfahren geprüft werden:<br>▪ ≥ 5 Einnahmezeitpunkte Levodopa/Tag (entsprechend Einnahmeintervallen von < 3 h)<br>▪ ≥ 2h Off-Symptome/Tag<br>▪ ≥ 1h störende Dyskinesien/Tag<br>▪ Scores zu den Aktivitäten des täglichen Lebens (ADL) und zur Lebensqualität (QOL, z.B. PDQ-39) sollten in die Entscheidung für oder gegen eine invasive Therapie regelhaft mit einfließen.<br>▪ Zuvor sollte ein adäquater Versuch mit Levodopa in Kombination mit einem Dopaminagonisten, MAO-B- und COMT-Hemmer ineffektiv gewesen sein.<br>▪ Diese Kriterien sind weder notwendig noch hinreichend für die Indikationsstellung, sondern geben einen Hinweis.	
Konsensstärke: 95,2%, starker Konsens	

Fragestellung 181: Welches sind die differenziellen Indikationen und Kontraindikationen für eine Kombination der invasiven Therapien (Pumpentherapien, Tiefe Hirnstimulation, ablative Verfahren) in der Behandlung der PK?

Hintergrund

Voraussetzung für die Wirksamkeit eines invasiven Verfahrens ist das Ansprechen eines jeweiligen Zielsymptoms auf Levodopa. Die Verfahren sind unterschiedlich nachhaltig in der Kontrolle motorischer Fluktuationen, sodass in manchen Fällen mehrere invasive Verfahren aufeinander folgen. In manchen Situationen kann die Kombination von invasiven Verfahren (z.B. im Fall von Wiederkehr von Fluktuationen oder Dyskinesien unter einem bestimmten Verfahren) erforderlich

werden. In diesem Kapitel soll die Evidenz für die Kombination von invasiven Therapien evaluiert werden.

Evidenzgrundlage

Für diese Kapitel: In dieser Literaturrecherche fanden sich:

- N=5 Fallserien.

Ergebnis

Kombination Apomorphin und THS

Eine spanische Studie untersuchte retrospektive Daten von 71 Pat., die mit CSAI und THS behandelt wurden. Die Kohorte wurde in vier Behandlungsgruppen unterteilt: Die Kombination von CSAI und THS reduzierte die Off-Zeit von 3,9 ± 2,6 auf 2,2 ± 1,3 Stunden nach einem Jahr Behandlungsdauer. Es wurde jedoch ein bemerkenswerter kognitiver Abbau im Verlauf dieses Jahres festgestellt [92]

Kombination LCIC und THS

Eine retrospektive Studie aus Slowenien und Israel untersuchte 505 Pat., die mit invasiven Verfahren wie CSAI, LCIG und THS behandelt wurden. Insgesamt wechselten 30 Pat. (6%) entweder ihr Therapieregime (24 Pat., 4,8%) oder erhielten eine Kombinationsbehandlung (6 Pat., 1%). Die Gründe für die Kombination waren persistierende oder wiederkehrende motorische Fluktuationen sowie individuelle Probleme wie Freezing und Dysphagie. Die Effekte der Kombination wurden nur als Verbesserung der globalen Einschätzung angegeben [93]. Eine spanische prospektive Studie mit 40 Pat. (21 mit LCIG, 19 mit THS) zeigte, dass die zusätzliche Behandlung mit LCIG nach durchschnittlich 7,8 Jahren einen signifikanten Effekt auf die motorischen Fluktuationen mit vorwiegend axialen Off-Symptomen hatte [94]. Eine kleine kanadische Fallsammlung mit 7 Pat., die im Durchschnitt 5,1 ± 3,5 Jahre mit DBS behandelt wurden, zeigte einen glättenden Effekt von zusätzlichem LCIG auf motorische Fluktuationen [95]. In einer griechischen Fallsammlung mit 70 Pat. (25 THS, 39 LCIG, 6 Apomorphin) erhielten 8 Pat. (11%) eine Kombinationstherapie und die Kombination hatte bei allen Pat. einen additiven Effekt [96].

Kombination LCIG und Apomorphin

Für diese Kombination fanden sich keine Daten.

Kombination LCIG und ablative Verfahren

Für diese Kombination fanden sich keine Daten.

Kombination Apomorphin und ablative Verfahren

Für diese Kombination fanden sich keine Daten.

Kombination THS und ablative Verfahren

Für diese Kombination fanden sich keine Daten.

Begründung der Empfehlung:

Die Evidenz beruht auf 5 meist retrospektiven Fallserien, die sich ausschließlich mit den Kombinationen von THS mit Pumpentherapien befassen. Prospektive oder kontrollierte Studien existieren nicht.

**Empfehlung**	**Neu**<br>**Stand (2023)**
▪ Bei Wiederkehr motorischer Fluktuationen unter einem invasiven Verfahren steht zunächst die Ursachensuche im Vordergrund. Im Einzelfall kann eine Kombination mit einem weiteren invasiven Verfahren oder eine Umstellung auf eine andere invasive Therapieform in Erwägung gezogen werden.<br>▪ Die Auswahl des Folgeverfahrens muss sich am individuellen Pat.-Profil zum Zeitpunkt der Entscheidung für ein zweites Verfahren orientieren.	
Konsensstärke: 100%, starker Konsens	

Referenzen

1. Schuurman PR, Bosch DA, Bossuyt PM, Bonsel GJ, van Someren EJ, de Bie RM, et al. A comparison of continuous thalamic stimulation and thalamotomy for suppression of severe tremor. N Engl J Med. 2000;342(7):461-8

2. Meng Y, Hynynen K, Lipsman N. Applications of focused ultrasound in the brain: from thermoablation to drug delivery. Nat Rev Neurol. 2021;17(1):7-22

3. Martínez-Fernández R, Máñez-Miró JU, Rodríguez-Rojas R, Del Álamo M, Shah BB, Hernández-Fernández F, et al. Randomized Trial of Focused Ultrasound Subthalamotomy for Parkinson's Disease. N Engl J Med. 2020;383(26):2501-13

4. Stibe C, Lees A, Stern G. Subcutaneous infusion of apomorphine and lisuride in the treatment of parkinsonian on-off fluctuations. Lancet. 1987;1(8537):871

5. Nilsson D, Hansson LE, Johansson K, Nyström C, Paalzow L, Aquilonius SM. Long-term intraduodenal infusion of a water based levodopa-carbidopa dispersion in very advanced Parkinson's disease. Acta Neurol Scand. 1998;97(3):175-83

6. Soileau MJ, Aldred J, Budur K, Fisseha N, Fung VS, Jeong A, et al. Safety and efficacy of continuous subcutaneous foslevodopa-foscarbidopa in patients with advanced Parkinson's disease: a randomised, double-blind, active-controlled, phase 3 trial. Lancet Neurol. 2022;21(12):1099-109

6.a Aldred J, Freire-Alvarez E, Amelin AV, Antonini A, Bergmans B, Bergquist F, Bouchard M, Budur K, Carroll C, Chaudhuri KR, Criswell SR, Danielsen EH, Gandor F, Jia J, Kimber TE, Mochizuki H, Robieson WZ, Spiegel AM, Standaert DG, Talapala S, Facheris MF, Fung VSC. Continuous subcutaneous foslevodopa/foscarbidopa in Parkinson‘s disease: safety and efficacy results from a 12-month, single-arm, open-label, phase 3 study. Neurol Ther 2023; online ahead of print

7. Deuschl G, Follett KA, Luo P, Rau J, Weaver FM, Paschen S, et al. Comparing two randomized deep brain stimulation trials for Parkinson's disease. J Neurosurg. 2019;132(5):1376-84

8. Weaver F, Follett K, Hur K, Ippolito D, Stern M. Deep brain stimulation in Parkinson disease: a metaanalysis of patient outcomes. J Neurosurg. 2005;103(6):956-67

9. Weaver FM, Follett KA, Stern M, Luo P, Harris CL, Hur K, et al. Randomized trial of deep brain stimulation for Parkinson disease: thirty-six-month outcomes. Neurology. 2012;79(1):55-65

10. Olanow CW, Kieburtz K, Odin P, Espay AJ, Standaert DG, Fernandez HH, et al. Continuous intrajejunal infusion of levodopa-carbidopa intestinal gel for patients with advanced Parkinson's disease: a randomised, controlled, double-blind, double-dummy study. Lancet Neurol. 2014;13(2):141-9

11. Senek M, Nielsen EI, Nyholm D. Levodopa-entacapone-carbidopa intestinal gel in Parkinson's disease: A randomized crossover study. Mov Disord. 2017;32(2):283-6

12. Dafsari HS, Martinez-Martin P, Rizos A, Trost M, Dos Santos Ghilardi MG, Reddy P, et al. EuroInf 2: Subthalamic stimulation, apomorphine, and levodopa infusion in Parkinson's disease. Mov Disord. 2019;34(3):353-65

13. Merola A, Zibetti M, Angrisano S, Rizzi L, Lanotte M, Lopiano L. Comparison of subthalamic nucleus deep brain stimulation and Duodopa in the treatment of advanced Parkinson's disease. Mov Disord. 2011;26(4):664-70

14. Elia AE, Dollenz C, Soliveri P, Albanese A. Motor features and response to oral levodopa in patients with Parkinson's disease under continuous dopaminergic infusion or deep brain stimulation. Eur J Neurol. 2012;19(1):76-83

15. Antonini A, Isaias IU, Rodolfi G, Landi A, Natuzzi F, Siri C, Pezzoli G. A 5-year prospective assessment of advanced Parkinson disease patients treated with subcutaneous apomorphine infusion or deep brain stimulation. J Neurol. 2011;258(4):579-85

16. De Gaspari D, Siri C, Landi A, Cilia R, Bonetti A, Natuzzi F, et al. Clinical and neuropsychological follow up at 12 months in patients with complicated Parkinson's disease treated with subcutaneous apomorphine infusion or deep brain stimulation of the subthalamic nucleus. J Neurol Neurosurg Psychiatry. 2006;77(4):450-3

17. Alegret M, Valldeoriola F, Martí M, Pilleri M, Junqué C, Rumià J, Tolosa E. Comparative cognitive effects of bilateral subthalamic stimulation and subcutaneous continuous infusion of apomorphine in Parkinson's disease. Mov Disord. 2004;19(12):1463-9

18. Liu XD, Bao Y, Liu GJ. Comparison Between Levodopa-Carbidopa Intestinal Gel Infusion and Subthalamic Nucleus Deep-Brain Stimulation for Advanced Parkinson's Disease: A Systematic Review and Meta-Analysis. Front Neurol. 2019;10:934

19. Merola A, Espay AJ, Romagnolo A, Bernardini A, Rizzi L, Rosso M, et al. Advanced therapies in Parkinson's disease: Long-term retrospective study. Parkinsonism Relat Disord. 2016;29:104-8

20. Valldeoriola F, Santacruz P, Ríos J, Compta Y, Rumià J, Muñoz JE, et al. l-Dopa/carbidopa intestinal gel and subthalamic nucleus stimulation: Effects on cognition and behavior. Brain Behav. 2017;7(11):e00848

21. Martinez-Martin P, Reddy P, Katzenschlager R, Antonini A, Todorova A, Odin P, et al. EuroInf: a multicenter comparative observational study of apomorphine and levodopa infusion in Parkinson's disease. Mov Disord. 2015;30(4):510-6

22. Rosebraugh M, Stodtmann S, Liu W, Facheris MF. Foslevodopa/foscarbidopa subcutaneous infusion maintains equivalent levodopa exposure to levodopa-carbidopa intestinal gel delivered to the jejunum. Parkinsonism Relat Disord. 2022;97:68-72

23. Cavallieri F, Fraix V, Bove F, Mulas D, Tondelli M, Castrioto A, et al. Predictors of Long-Term Outcome of Subthalamic Stimulation in Parkinson Disease. Ann Neurol. 2021;89(3):587-97

24. Deuschl G, Antonini A, Costa J, Śmiłowska K, Berg D, Corvol JC, et al. European Academy of Neurology/Movement Disorder Society-European Section Guideline on the Treatment of Parkinson's Disease: I. Invasive Therapies. Mov Disord. 2022;37(7):1360-74

25. Leta V, Dafsari HS, Sauerbier A, Metta V, Titova N, Timmermann L, et al. Personalised Advanced Therapies in Parkinson's Disease: The Role of Non-Motor Symptoms Profile. J Pers Med. 2021;11(8)

26. Drapier S, Eusebio A, Degos B, Vérin M, Durif F, Azulay JP, et al. Quality of life in Parkinson's disease improved by apomorphine pump: the OPTIPUMP cohort study. J Neurol. 2016;263(6):1111-9

27. Martinez-Martin P, Reddy P, Antonini A, Henriksen T, Katzenschlager R, Odin P, et al. Chronic subcutaneous infusion therapy with apomorphine in advanced Parkinson's disease compared to conventional therapy: a real life study of non motor effect. J Parkinsons Dis. 2011;1(2):197-203

28. Antonini A, Pahwa R, Odin P, Isaacson SH, Merola A, Wang L, et al. Comparative Effectiveness of Device-Aided Therapies on Quality of Life and Off-Time in Advanced Parkinson's Disease: A Systematic Review and Bayesian Network Meta-analysis. CNS Drugs. 2022;36(12):1269-83

29. Schuepbach WM, Rau J, Knudsen K, Volkmann J, Krack P, Timmermann L, et al. Neurostimulation for Parkinson's disease with early motor complications. N Engl J Med. 2013;368(7):610-22

30. Deuschl G, Schade-Brittinger C, Krack P, Volkmann J, Schäfer H, Bötzel K, et al. A randomized trial of deep-brain stimulation for Parkinson's disease. N Engl J Med. 2006;355(9):896-908

31. Pieroni MA. Investigation of apomorphine during sleep in Parkinson's: Improvement in UPDRS Scores. Neurol Int. 2019;11(4):8207

32. Poewe W, Bergmann L, Kukreja P, Robieson WZ, Antonini A. Levodopa-Carbidopa Intestinal Gel Monotherapy: GLORIA Registry Demographics, Efficacy, and Safety. J Parkinsons Dis. 2019;9(3):531-41

33. Krack P, Batir A, Van Blercom N, Chabardes S, Fraix V, Ardouin C, et al. Five-year follow-up of bilateral stimulation of the subthalamic nucleus in advanced Parkinson's disease. N Engl J Med. 2003;349(20):1925-34

34. Krause M, Fogel W, Mayer P, Kloss M, Tronnier V. Chronic inhibition of the subthalamic nucleus in Parkinson's disease. J Neurol Sci. 2004;219(1-2):119-24

35. Davis JT, Lyons KE, Pahwa R. Freezing of gait after bilateral subthalamic nucleus stimulation for Parkinson's disease. Clin Neurol Neurosurg. 2006;108(5):461-4

36. Li J, Zhang Y, Li Y. Long-term follow-up of bilateral subthalamic nucleus stimulation in Chinese Parkinson's disease patients. Br J Neurosurg. 2015;29(3):329-33

37. Kim R, Yoo D, Jung YJ, Lee WW, Ehm G, Yun JY, et al. Determinants of Functional Independence or Its Loss following Subthalamic Nucleus Stimulation in Parkinson's Disease. Stereotact Funct Neurosurg. 2019;97(2):106-12

38. Krishna V, Fishman PS, Eisenberg HM, Kaplitt M, Baltuch G, Chang JW, et al. Trial of Globus Pallidus Focused Ultrasound Ablation in Parkinson's Disease. N Engl J Med. 2023;388(8):683-93

39. Katzenschlager R, Poewe W, Rascol O, Trenkwalder C, Deuschl G, Chaudhuri KR, et al. Apomorphine subcutaneous infusion in patients with Parkinson's disease with persistent motor fluctuations (TOLEDO): a multicentre, double-blind, randomised, placebo-controlled trial. Lancet Neurol. 2018;17(9):749-59

40. Katzenschlager R, Poewe W, Rascol O, Trenkwalder C, Deuschl G, Chaudhuri KR, et al. Long-term safety and efficacy of apomorphine infusion in Parkinson's disease patients with persistent motor fluctuations: Results of the open-label phase of the TOLEDO study. Parkinsonism Relat Disord. 2021;83:79-85

41. Bond AE, Shah BB, Huss DS, Dallapiazza RF, Warren A, Harrison MB, et al. Safety and Efficacy of Focused Ultrasound Thalamotomy for Patients With Medication-Refractory, Tremor-Dominant Parkinson Disease: A Randomized Clinical Trial. JAMA Neurol. 2017;74(12):1412-8

42. Stanková S, Straka I, Košutzká Z, Valkovič P, Minár M. Levodopa-Carbidopa Intestinal Gel Improves Symptoms of Orthostatic Hypotension in Patients with Parkinson's Disease-Prospective Pilot Interventional Study. J Pers Med. 2022;12(5)

43. De Cock VC, Dodet P, Leu-Semenescu S, Aerts C, Castelnovo G, Abril B, et al. Safety and efficacy of subcutaneous night-time only apomorphine infusion to treat insomnia in patients with Parkinson's

disease (APOMORPHEE): a multicentre, randomised, controlled, double-blind crossover study. Lancet Neurol. 2022;21(5):428-37

44. Standaert DG, Aldred J, Anca-Herschkovitsch M, Bourgeois P, Cubo E, Davis TL, et al. DUOGLOBE: One-Year Outcomes in a Real-World Study of Levodopa Carbidopa Intestinal Gel for Parkinson's Disease. Mov Disord Clin Pract. 2021;8(7):1061-74

45. Diaconu Ş, Irincu L, Ţînţ D, Falup-Pecurariu C. Long-term effects of intrajejunal levodopa infusion on sleep in people with advanced Parkinson's disease. Front Neurol. 2023;14:1105650

46. Ricciardi L, Bove F, Espay KJ, Lena F, Modugno N, Poon YY, et al. 24-Hour infusion of levodopa/carbidopa intestinal gel for nocturnal akinesia in advanced Parkinson's disease. Mov Disord. 2016;31(4):597-8

47. Antonini A, Poewe W, Chaudhuri KR, Jech R, Pickut B, Pirtošek Z, et al. Levodopa-carbidopa intestinal gel in advanced Parkinson's: Final results of the GLORIA registry. Parkinsonism Relat Disord. 2017;45:13-20

48. Honig H, Antonini A, Martinez-Martin P, Forgacs I, Faye GC, Fox T, et al. Intrajejunal levodopa infusion in Parkinson's disease: a pilot multicenter study of effects on nonmotor symptoms and quality of life. Mov Disord. 2009;24(10):1468-74

49. Reddy P, Martinez-Martin P, Rizos A, Martin A, Faye GC, Forgacs I, et al. Intrajejunal levodopa versus conventional therapy in Parkinson disease: motor and nonmotor effects. Clin Neuropharmacol. 2012;35(5):205-7

50. Jost ST, Ray Chaudhuri K, Ashkan K, Loehrer PA, Silverdale M, Rizos A, et al. Subthalamic Stimulation Improves Quality of Sleep in Parkinson Disease: A 36-Month Controlled Study. J Parkinsons Dis. 2021;11(1):323-35

51. Baumann-Vogel H, Imbach LL, Sürücü O, Stieglitz L, Waldvogel D, Baumann CR, Werth E. The Impact of Subthalamic Deep Brain Stimulation on Sleep-Wake Behavior: A Prospective Electrophysiological Study in 50 Parkinson Patients. Sleep. 2017;40(5)

52. Dafsari HS, Reddy P, Herchenbach C, Wawro S, Petry-Schmelzer JN, Visser-Vandewalle V, et al. Beneficial Effects of Bilateral Subthalamic Stimulation on Non-Motor Symptoms in Parkinson's Disease. Brain Stimul. 2016;9(1):78-85

53. Lyons KE, Pahwa R. Effects of bilateral subthalamic nucleus stimulation on sleep, daytime sleepiness, and early morning dystonia in patients with Parkinson disease. J Neurosurg. 2006;104(4):502-5

54. Lhommée E, Klinger H, Thobois S, Schmitt E, Ardouin C, Bichon A, et al. Subthalamic stimulation in Parkinson's disease: restoring the balance of motivated behaviours. Brain. 2012;135(Pt 5):1463-77

55. van Laar T, Postma AG, Drent M. Continuous subcutaneous infusion of apomorphine can be used safely in patients with Parkinson's disease and pre-existing visual hallucinations. Parkinsonism Relat Disord. 2010;16(1):71-2

56. Zibetti M, Torre E, Cinquepalmi A, Rosso M, Ducati A, Bergamasco B, et al. Motor and nonmotor symptom follow-up in parkinsonian patients after deep brain stimulation of the subthalamic nucleus. Eur Neurol. 2007;58(4):218-23

57. Christmas TJ, Kempster PA, Chapple CR, Frankel JP, Lees AJ, Stern GM, Milroy EJ. Role of subcutaneous apomorphine in parkinsonian voiding dysfunction. Lancet. 1988;2(8626-8627):1451-3

58. Todorova A, Ray Chaudhuri K. Subcutaneous apomorphine and non-motor symptoms in Parkinson's disease. Parkinsonism Relat Disord. 2013;19(12):1073-8

59. Storch A, Schneider CB, Klingelhöfer L, Odin P, Fuchs G, Jost WH, et al. Quantitative assessment of non-motor fluctuations in Parkinson's disease using the Non-Motor Symptoms Scale (NMSS). J Neural Transm (Vienna). 2015;122(12):1673-84

60. Petry-Schmelzer JN, Krause M, Dembek TA, Horn A, Evans J, Ashkan K, et al. Non-motor outcomes depend on location of neurostimulation in Parkinson's disease. Brain. 2019;142(11):3592-604

61. Bhidayasiri R, Sringean J, Anan C, Boonpang K, Thanawattano C, Ray Chaudhuri K. Quantitative demonstration of the efficacy of night-time apomorphine infusion to treat nocturnal hypokinesia in Parkinson's disease using wearable sensors. Parkinsonism Relat Disord. 2016;33 Suppl 1:S36-s41

62. Fernández-Pajarín G, Sesar Á, Ares B, Jiménez-Martín I, Gelabert M, Arán-Echabe E, et al. Continuous Subcutaneous Apomorphine Infusion before Subthalamic Deep Brain Stimulation: A Prospective, Comparative Study in 20 Patients. Mov Disord Clin Pract. 2021;8(8):1216-24

63. Reuter I, Ellis CM, Ray Chaudhuri K. Nocturnal subcutaneous apomorphine infusion in Parkinson's disease and restless legs syndrome. Acta Neurol Scand. 1999;100(3):163-7

64. Borgemeester RW, Drent M, van Laar T. Motor and non-motor outcomes of continuous apomorphine infusion in 125 Parkinson's disease patients. Parkinsonism Relat Disord. 2016;23:17-22

65. Abboud H, Floden D, Thompson NR, Genc G, Oravivattanakul S, Alsallom F, et al. Impact of mild cognitive impairment on outcome following deep brain stimulation surgery for Parkinson's disease. Parkinsonism Relat Disord. 2015;21(3):249-53

66. Saint-Cyr JA, Trépanier LL, Kumar R, Lozano AM, Lang AE. Neuropsychological consequences of chronic bilateral stimulation of the subthalamic nucleus in Parkinson's disease. Brain. 2000;123 (Pt 10):2091-108

67. Parsons TD, Rogers SA, Braaten AJ, Woods SP, Tröster AI. Cognitive sequelae of subthalamic nucleus deep brain stimulation in Parkinson's disease: a meta-analysis. Lancet Neurol. 2006;5(7):578-88

68. Witt K, Daniels C, Reiff J, Krack P, Volkmann J, Pinsker MO, et al. Neuropsychological and psychiatric changes after deep brain stimulation for Parkinson's disease: a randomised, multicentre study. Lancet Neurol. 2008;7(7):605-14

69. Brodie HJ. The Function of the Cups of Polyporus conchifer. Science. 1951;114(2972):636

70. Williams A, Gill S, Varma T, Jenkinson C, Quinn N, Mitchell R, et al. Deep brain stimulation plus best medical therapy versus best medical therapy alone for advanced Parkinson's disease (PD SURG trial): a randomised, open-label trial. Lancet Neurol. 2010;9(6):581-91

71. Funkiewiez A, Ardouin C, Caputo E, Krack P, Fraix V, Klinger H, et al. Long term effects of bilateral subthalamic nucleus stimulation on cognitive function, mood, and behaviour in Parkinson's disease. J Neurol Neurosurg Psychiatry. 2004;75(6):834-9

72. Herold BC, McArdle P, Stagnaro-Green A. Translational medicine in the first year: integrative cores. Acad Med. 2002;77(11):1171

73. Block CK, Patel M, Risk BB, Staikova E, Loring D, Esper CD, et al. Patients with Cognitive Impairment in Parkinson's Disease Benefit from Deep Brain Stimulation: A Case-Control Study. Mov Disord Clin Pract. 2023;10(3):382-91

74. Odekerken VJ, van Laar T, Staal MJ, Mosch A, Hoffmann CF, Nijssen PC, et al. Subthalamic nucleus versus globus pallidus bilateral deep brain stimulation for advanced Parkinson's disease (NSTAPS study): a randomised controlled trial. Lancet Neurol. 2013;12(1):37-44

75. Bang Henriksen M, Johnsen EL, Sunde N, Vase A, Gjelstrup MC, Østergaard K. Surviving 10 years with deep brain stimulation for Parkinson's disease--a follow-up of 79 patients. Eur J Neurol. 2016;23(1):53-61

76. Barboza EBEN, Fichman HC. How is cognition in subthalamic nucleus deep brain stimulation Parkinson's disease patients? Dement Neuropsychol. 2019;13(4):367-77

77. Xie H, Zhang Q, Jiang Y, Bai Y, Zhang J. Parkinson's disease with mild cognitive impairment may has a lower risk of cognitive decline after subthalamic nucleus deep brain stimulation: A retrospective cohort study. Front Hum Neurosci. 2022;16:943472

78. Bove F, Fraix V, Cavallieri F, Schmitt E, Lhommée E, Bichon A, et al. Dementia and subthalamic deep brain stimulation in Parkinson disease: A long-term overview. Neurology. 2020;95(4):e384-e92

79. Moro E, Lang AE. Criteria for deep-brain stimulation in Parkinson's disease: review and analysis. Expert Rev Neurother. 2006;6(11):1695-705

80. Lhommée E, Wojtecki L, Czernecki V, Witt K, Maier F, Tonder L, et al. Behavioural outcomes of subthalamic stimulation and medical therapy versus medical therapy alone for Parkinson's disease with early motor complications (EARLYSTIM trial): secondary analysis of an open-label randomised trial. Lancet Neurol. 2018;17(3):223-31

81. Healy S, Shepherd H, Mooney N, Da Costa A, Osman-Farah J, Macerollo A. The effect of deep brain stimulation on impulse control related disorders in Parkinson's disease - A 10-year retrospective study of 137 patients. J Neurol Sci. 2022;440:120339

82. Catalan MJ, Molina-Arjona JA, Mir P, Cubo E, Arbelo JM, Martinez-Martin P. Improvement of impulse control disorders associated with levodopa-carbidopa intestinal gel treatment in advanced Parkinson's disease. J Neurol. 2018;265(6):1279-87

83. Lopiano L, Modugno N, Marano P, Sensi M, Meco G, Solla P, et al. Motor and non-motor outcomes in patients with advanced Parkinson's disease treated with levodopa/carbidopa intestinal gel: final results of the GREENFIELD observational study. J Neurol. 2019;266(9):2164-76

84. Todorova A, Samuel M, Brown RG, Chaudhuri KR. Infusion Therapies and Development of Impulse Control Disorders in Advanced Parkinson Disease: Clinical Experience After 3 Years' Follow-up. Clin Neuropharmacol. 2015;38(4):132-4

85. Fujimiya M, Ataka K, Asakawa A, Chen CY, Kato I, Inui A. Ghrelin, des-acyl ghrelin and obestatin on the gastrointestinal motility. Peptides. 2011;32(11):2348-51

86. Fasano A, Romito LM, Daniele A, Piano C, Zinno M, Bentivoglio AR, Albanese A. Motor and cognitive outcome in patients with Parkinson's disease 8 years after subthalamic implants. Brain. 2010;133(9):2664-76

87. Odin P, Ray Chaudhuri K, Slevin JT, Volkmann J, Dietrichs E, Martinez-Martin P, et al. Collective physician perspectives on non-oral medication approAcetylcholinesterase-Hemmer for the management of clinically relevant unresolved issues in Parkinson's disease: Consensus from an international survey and discussion program. Parkinsonism Relat Disord. 2015;21(10):1133-44

88. Antonini A, Stoessl AJ, Kleinman LS, Skalicky AM, Marshall TS, Sail KR, et al. Developing consensus among movement disorder specialists on clinical indicators for identification and management of advanced Parkinson's disease: a multi-country Delphi-panel approach. Curr Med Res Opin. 2018;34(12):2063-73

89. Timpka J, Henriksen T, Odin P. Non-oral Continuous Drug Delivery Techniques in Parkinson's Disease: For Whom, When, and How? Mov Disord Clin Pract. 2016;3(3):221-9

90. Worth PF. When the going gets tough: how to select patients with Parkinson's disease for advanced therapies. Pract Neurol. 2013;13(3):140-52

91. Aldred J, Anca-Herschkovitsch M, Antonini A, Bajenaru O, Bergmann L, Bourgeois P, et al. Application of the '5-2-1' screening criteria in advanced Parkinson's disease: interim analysis of DUOGLOBE. Neurodegener Dis Manag. 2020;10(5):309-23

92. Sesar Á, Fernández-Pajarín G, Ares B, Relova JL, Arán E, Rivas MT, et al. Continuous subcutaneous apomorphine in advanced Parkinson's disease patients treated with deep brain stimulation. J Neurol. 2019;266(3):659-66

93. Georgiev D, Delalić S, Zupančič Križnar N, Socher A, Gurevich T, Trošt M. Switching and Combining Device-Aided Therapies in Advanced Parkinson's Disease: A Double Centre Retrospective Study. Brain Sci. 2022;12(3)

94. Regidor I, Benita V, Del Álamo de Pedro M, Ley L, Martinez Castrillo JC. Duodenal Levodopa Infusion for Long-Term Deep Brain Stimulation-Refractory Symptoms in Advanced Parkinson Disease. Clin Neuropharmacol. 2017;40(3):103-7

95. Kumar N, Murgai A, Naranian T, Jog M, Fasano A. Levodopa-carbidopa intestinal gel therapy after deep brain stimulation. Mov Disord. 2018;33(2):334-5

96. Boura I, Haliasos N, Giannopoulou I A, Karabetsos D, Spanaki C. Combining Device-Aided Therapies in Parkinson's Disease: A Case Series and a Literature Review. Mov Disord Clin Pract. 2021;8(5):750-7

3.23 Aktivierende Therapien

Autoren: Tobias Wächter, Christian Winkler, Georg Ebersbach, Andres Ceballos-Baumann

Fragestellung 182: Wie effektiv ist die Physiotherapie im Vergleich mit der medizinischen Standardtherapie oder Placebo in der Behandlung der PK? Welche Zeitdauer und Intensität der Physiotherapie ist für Pat. mit PK erforderlich?

Hintergrund

Physiotherapie hat zum Ziel, bei Parkinson-Pat. die Kraft, Koordination, Beweglichkeit, Ausdauer, Balance und Rumpfstabilität sowie Stand- und Gangsicherheit zu steigern, und somit die Motorik und insbesondere die Mobilität zu verbessern. Somit stellen sich die Fragen, ob Physiotherapie einen positiven Effekt bei der Behandlung motorischer Defizite hat und über welche Zeit bzw. mit welcher Intensität die Therapie empfohlen werden kann.

Evidenzgrundlage

Die Literatursuche in Pubmed erbrachte 3511 Studien zu den Begriffen „Parkinson disease AND physiotherapy". Nach Screening der Abstracts und Inhalte wurden weitere Studien aus den Literaturreferenzen extrahiert und letztendlich 23 Studien selektiert, welche in diese Leitlinie einbezogen wurden. Davon waren 16 randomisierte, kontrollierte Studien und 7 Übersichtsartikel/Metaanalysen.

Ergebnis

Unterschiedliche Therapiemethoden

Eine Metaanalyse [1] über 191 Studien an 7.998 Parkinson-Pat. zeigt eine signifikante Verbesserung der motorischen Symptome, des Gangs und der Lebensqualität durch klassische Physiotherapie. Kraft- und Laufbandtraining verbessern den Gang, strategisches Training stärkt Balance und Gang. Tanz, Nordic Walking, Balance-, Gang-, und Kampftraining wirken sich positiv auf motorische Symptome, Balance und Gang aus. Exergaming verbessert die Balance und die Lebensqualität, Wassertherapie nur die Balance, während Dual-Task-Training nach diesem systemischen Review zu keiner signifikanten Verbesserung führte.

Zahlreiche weitere systemische Reviews mit Metaanalysen zeigen eine Verbesserung

- der Beinkraft durch Krafttraining [2]
- der Motorik (UPDRS) durch Wassergymnastik [3], Physiotherapie [4], Ausdauertraining [5] und Exergaming [6]
- der Koordination und Rumpfkontrolle durch Physiotherapie [4] und Wassergymnastik [7]
- des Gleichgewichts durch Tai-Chi [8], Kraft- [2] und Ausdauertraining [9]
- der Gehfähigkeit durch Wassergymnastik [10], Physiotherapie [4], Ausdauertraining [9], Laufbandtraining [11] und Bewegungstraining [12]
- dbei er Zahl von Stürzen durch Bewegungstraining [12]

- von Freezing durch Laufbandtraining [11] und Physiotherapie [13].

Diese systematischen Reviews zeigen auch, dass motorische Symptome der PK nicht verbessert werden durch reines Ausdauertraining [9] oder Nordic Walking [14], klassische Physiotherapie keinen Einfluss hat auf Stürze oder Lebensqualität [4] und reines Krafttraining das Gangbild nicht verbessert [15], [2].

Diese Übersicht zeigt, dass der Einfluss des Ausdauertrainings auf die Motorik und der Effekt von klassischer Physiotherapie auf die Gehfähigkeit auch in Metaanalysen nicht einheitlich nachgewiesen werden kann.

Einzelne randomisierte kontrollierte Studien belegen zudem eine verbesserte Lebensqualität durch Wassertherapie (Ai-Chi) [16] oder Krafttraining [17], eine Schmerzreduktion durch Wassertherapie [18] sowie eine Verbesserung der Rumpfkontrolle und Körpersymmetrie durch sensomotorisches Training [19] und Training der Rumpfstabilität [20]. Physiotherapie kann zudem die Sturzangst bei Parkinson-Pat. reduzieren [21]. Die Aussagekraft dieser einzelnen Studien ist jedoch aufgrund der jeweils eher geringen Fallzahl von unter 50 Probanden/Probandinnen begrenzt.

Komplexität der Therapien

Mehrere randomisierte und kontrollierte Studien geben Hinweise darauf, dass komplexe Ansätze in der Physiotherapie (z.B. aufgabenspezifische oder koordinativ herausfordernde Therapien) deutlich effektiver sind als einfachere Therapien [22], [23], [24], [25], [26] [27]. Dies gilt insbesondere für Gangtraining und Sturzprophylaxe [22], [23], [25]. Diese Erkenntnis könnte ggf. die unterschiedlichen Studienergebnisse zum Ausdauertraining auf die Motorik oder zur klassischen Physiotherapie auf die Gehfähigkeit, die sich auch in den Metaanalysen (s.o.) zeigen, erklären.

Therapiedauer und -intensität

Eine physiotherapeutische Behandlung mit weniger als einer Therapieeinheit/Woche ist nicht effektiv. Das konnte in mehreren randomisierten kontrollierten Studien nachgewiesen werden, die bei einer Fallzahl von mehreren Hundert Parkinson-Pat. keinen signifikanten Effekt der Physiotherapie bei einer Frequenz von einer Einheit/14 d fanden [28], [29], [30]. Eine randomisierte kontrollierte Studie zur Dosisfindung zeigt einen dosisabhängigen Effekt von Physiotherapie auf den motorischen Benefit bei Pat. mit Morbus Parkinson [31].

Ein systematischer Review [10] schließt aus einer Metaanalyse von 14 Studien und 472 Pat., dass eine Therapiedichte von mindestens drei Therapieeinheiten/Woche notwendig ist, um einen klinisch relevanten Benefit zu erzielen.

Die meisten zitierten Studien mit positivem Effekt testen Therapieansätze mit einer Intensität von mindestens 3h Therapie/Woche über einen Zeitraum von 4–12 Wochen. Eine Studie über 24 Monate konnte auch einen positiven Effekt bei 2 Therapieeinheiten/Woche über diesen langen Zeitraum nachweisen [32]. Dies zeigt, dass eine geringere Intensität auf Dauer auch effektiv sein könnte.

Eine Metaanalyse [33] und mehrere kontrollierte Studien [17], [34], [35] zeigen, dass multidisziplinäre Rehabilitationsmaßnahmen mit sehr hoher Intensität (bis zu 17 h/Woche) über

mehrere Wochen einen signifikanten Benefit für die Motorik, Koordination, Rumpfkontrolle, Gangfähigkeit und Lebensqualität der Parkinson-Pat. haben, der bis zu 18 Monate nach der Rehabilitationsmaßnahme anhält [17]. Dies legt nahe, dass komplexe Therapien mit hoher Intensität einen großen und nachhaltigen Benefit erbringen.

Bereits Parkinson-Pat. in einem frühen Stadium und mit milden bis moderaten Symptomen profitieren von intensiver Physiotherapie, insbesondere, wenn die Therapie an die Leistungsfähigkeit der Pat. angepasst sind [10], [31], [33].

Nebenwirkungen

Ein systemischer Review [4] beschreibt Nebenwirkungen als selten. Meist werden Nebenwirkungen in den Studien jedoch nicht erwähnt.

Begründung der Empfehlung

Auf der Basis der oben dargestellten Evidenzgrundlage wird zur Behandlung von Parkinson-Pat. mit Physiotherapie die u.g. Empfehlung gegeben.

**Empfehlung**	**Neu**<br>**Stand (2023)**
Bezüglich der Effektivität, der Behandlungsdauer und der Intensität von Physiotherapie bei Parkinson-Pat. können folgende Empfehlungen gegeben werden:<br>1. Pat. mit der PK und Beeinträchtigung durch motorische Symptome im Alltag sollen Zugang zu einer physiotherapeutischen Behandlung erhalten.<br>2. Die Physiotherapie soll auf die motorischen Defizite der Pat. ausgerichtet sein.<br>3. Die Physiotherapie sollte mit komplexen Therapieansätzen erfolgen und an den Symptomen (ggf. mehreren, z.B. Koordination und Kraft) ausgerichtet sein und an die Leistungsfähigkeit der Pat. angepasst werden.<br>4. Die Physiotherapie sollte mindestens 3 h/Woche erfolgen. Wenn diese Intensität aus z.B. organisatorischen Gründen dauerhaft nicht gehalten werden kann und die Pat. hierzu in der Lage sind, kann ein Teil der Therapie als Eigentraining erfolgen.	
Konsensstärke: 100%, starker Konsens	

Fragestellung 183: Wie effektiv ist eine Ergotherapie im Vergleich mit der medizinischen Standardtherapie oder Placebo in der Behandlung der PK?

Fragestellung 184: Welche Zeitdauer und Intensität der Ergotherapie ist für Pat. mit PK erforderlich?

Einleitung

Ergotherapie wird zur Behandlung von Menschen eingesetzt, die in ihrer Handlungsfähigkeit eingeschränkt oder von Einschränkung bedroht sind. Ziel ist, sie bei der Durchführung für sie bedeutungsvoller Betätigungen in den Bereichen Selbstversorgung, Produktivität und Freizeit in ihrer persönlichen Umwelt zu stärken. Spezifische Aktivitäten, Umweltanpassung und Beratung dienen

dabei dazu, den Menschen Handlungsfähigkeit im Alltag, gesellschaftliche und berufliche Teilhabe und eine Verbesserung ihrer Lebensqualität zu ermöglichen

In der Ergotherapie kommen zum Erhalt der Handlungsfähigkeit und der Teilhabe verschiedene Therapieansätze zum Einsatz:

- Veränderung von Umweltfaktoren (z.B. Wohnraumanpassung, Anpassungen am Arbeitsplatz, Hilfsmittelversorgung, Angehörigenberatung)
- Erarbeitung von Alternativ- bzw. Kompensationsstrategien (z.B. Zeit-, Energie- und Fatigue-Management und Techniken, Sturzprävention, Hilfsmitteltraining, Beratung und Unterstützung bei psychosozialen Anpassungsprozessen (Coping) und der Krankheitsverarbeitung)
- ADL-Training, berufsbezogene Maßnahmen, vorbereitende, direkt am Arbeitsplatz stattfindende Maßnahmen und Training anderer bedeutsamer Aktivitäten (z.B. Mobilitätstraining, Schreibtraining, Ermöglichung der Teilhabe bei der Freizeitgestaltung, z.B. Gartentraining)
- schädigungsspezifische Maßnahmen auf Körperfunktions- und Strukturebene (z.B. Feinmotorik-, Gleichgewichts,- Sensibilitäts-, Mimik- und Krafttraining)

Beantwortung der Frage: Wie effektiv ist eine Ergotherapie im Vergleich mit der medizinischen Standardtherapie oder Placebo in der Behandlung der PK?

Evidenzgrundlage

Es wurde eine große randomisierte Studie identifiziert, in der Ergotherapie mit einer inaktiven Kontrollgruppe (Standardtherapie) verglichen wurde [36]. Drei randomisierte Studien untersuchten die Wirkung von Schreibtraining auf die Fähigkeit, mit der Hand zu schreiben [37], [38], [39]. Ein systematischer Review [40] analysierte insgesamt 10 Studien mit Daten von 1.343 Menschen mit Parkinson. Es wurden nur Studien eingeschlossen, bei denen der Anteil an Ergotherapie über 50% lag. Studien zu multidisziplinären Rehabilitationsprogrammen, bei denen ergotherapeutische Interventionen einen geringeren Anteil an der zu bewertenden Gesamtintervention ausmachten, wurden ausgeschlossen.

Vergleiche gegen aktive Kontrollinterventionen oder Placebo liegen nicht vor. Zwei weitere Reviews befassten sich mit der Wirkung von Ergotherapie auf die instrumentellen Aktivitäten des täglichen Lebens (ADL, [41]) oder dem Einfluss von Ergotherapie auf die Lebensqualität [42]. In allen Reviews ergaben sich bezüglich der Interventionsinhalte Überlappungen mit an anderer Stelle diskutierten Interventionen (Maßnahmen der künstlerischen Therapien, kognitives Training, Maßnahmen der Physiotherapie).

Ergebnis

In einer kontrollierten randomisierten Studie mit 191 Pat. [36] führte ein 10-wöchiges ambulantes Ergotherapieprogramm gegenüber einer inaktiven Kontrollgruppe zur signifikanten Besserung der selbst wahrgenommenen Performanz in bedeutungsvollen ADL, der Zufriedenheit mit der Ausführung von Alltagsaktivitäten und der Teilhabe an verschiedenen ADL (erhoben über das Canadian Occupational Performance Measure). Der Review von Welsby et al. 2019 über 10 Studien

mit einem Anteil an Ergotherapie von mehr als 50% an der Intervention kam zu dem Schluss, dass eine auf individuell bedeutungsvolle Aktivitäten ausgerichtete Ergotherapie die wahrgenommene Ausführung von Aktivitäten (Betätigungsperformanz) steigerte. Therapieprogramme für die oberen Gliedmaßen verbessern die Funktion der oberen Gliedmaßen, obwohl die längerfristigen Auswirkungen unklar sind. Insgesamt kommen die Autoren/Autorinnen zu dem Fazit, dass häusliche Ergotherapie, die auf bedeutungsvolle Aktivitäten abzielt, der vielversprechendste Ansatz zur Verbesserung der Ausführung von Aktivitäten ist und Ergotherapeutinnen/Ergotherapeuten sicherstellen sollten, dass ihre Interventionen die Symptome mit der jeweils größten individuellen Relevanz (z.B. Schlaf, Fatigue, Stürze, Essen, sensorische Einschränkungen, visuelle Störungen und geminderte Stimmung) und ihre funktionellen Implikationen adressieren.

Die Interventionen zur Verbesserung der Handschrift hatten unterschiedliche Behandlungskomponenten (z. B. Handübungen, Schreibtraining mit externen Cues) und variierten in Intensität, Häufigkeit und Dauer. In allen drei Studien wurden signifikante Verbesserungen der Handschrift (UPDRS-Item 2.7., Schriftamplitude) beobachtet.

**Empfehlung**	**Neu**<br>**Stand (2023)**
Ergotherapie sollte Menschen mit einer Parkinson-Erkrankung verordnet werden, die unter Einschränkungen bei den Aktivitäten des täglichen Lebens, der beruflichen Teilhabe oder unter Funktionsstörungen der oberen Extremitäten, einschließlich einer Beeinträchtigung der Handschrift, leiden.	
Konsensstärke: 100%, starker Konsens	

Beantwortung der Frage: Welche Zeitdauer und Intensität der Ergotherapie ist für Pat. mit PK erforderlich?

Evidenzgrundlage

Es wurde eine große randomisierte Studie identifiziert, in der Ergotherapie mit einer inaktiven Kontrollgruppe (Standardtherapie) verglichen wurde [36]. Ein systematischer Review [40] analysierte insgesamt 10 Studien mit Daten von 1.343 Menschen mit Parkinson, die sich Interventionen unterzogen, die zu mehr als 50% aus Ergotherapie bestanden.

Ergebnis

In der kontrollierten randomisierten Studie mit 191 Pat. Umfasste das ambulante Ergotherapieprogramm 16 Therapieeinheiten (mit einer Dauer von je 1 Stunde) innerhalb von 10 Wochen. Dies führte gegenüber einer inaktiven Kontrollgruppe zu einer signifikanten Besserung der selbst wahrgenommenen Performanz in bedeutungsvollen ADL, der Zufriedenheit mit der Ausführung von Alltagsaktivitäten und der Teilhabe an verschiedenen ADL (erhoben über das Canadian Occupational Performance Measure). In einer randomisierten britischen Multicenter-Studie mit 762 Teilnehmenden [28] wurde demgegenüber im aktiven Therapiearm (Physio- und Ergotherapie über 3 Monate) kein Vorteil bezüglich Lebensqualität und ADL im Vergleich zur

inaktiven Kontrollgruppe berichtet. Ein kritischer Aspekt dieser Studie ist die geringe „Dosis“ der untersuchten Therapien (die mittlere Anzahl betrug 4 Ergo- und Physiotherapie-Einheiten innerhalb von 3 Monaten). Welsby et al. [40] konstatierten in ihrer systematischen Übersichtsarbeit, dass intermittierende kurze Therapieintervalle hoher Intensität, die gleichzeitig sowohl die Funktionen der oberen Extremitäten als auch ADL adressieren, für Menschen mit einer PK wahrscheinlich am geeignetsten sind.

**Empfehlung**	**Neu**<br><br>**Stand (2023)**
▪ Die Intensität und Dauer gezielter ergotherapeutischer Interventionen sollten mindestens 16 Stunden über einen Zeitraum von 10 Wochen betragen.<br>▪ Bei persistierenden und potenziell ergotherapeutisch behandelbaren Einschränkungen der Funktionsfähigkeit sollte eine regelmäßige und dauerhafte Ergotherapie erfolgen.	
Konsensstärke: 100%, starker Konsens	

Fragestellung 185: Wie effektiv sind Künstlerische Therapien im Vergleich mit der medizinischen Standardtherapie oder Placebo in der Behandlung der PK?

Hintergrund

Wie andere aktivierende Therapien zielen Künstlerische Therapien (beispielsweise Musik, Tanz, Kunst, Theater) auf die Verbesserung der Lebensqualität, die größtmögliche Selbstständigkeit in den Aktivitäten des täglichen Lebens und eine selbstbestimmte soziale Teilhabe ab. Neben Erhalt beziehungsweise Verbesserung sensomotorischer Funktionen sollen Künstlerische Therapien eine Verbesserung von Stimmung, Selbstwertgefühl und Initiative ermöglichen. Da Tai-Chi in der wissenschaftlichen Literatur sowohl als Physiotherapie als auch mit der Tanztherapie abgehandelt wird, wurde Tai-Chi auch bei den künstlerischen Therapien mitbehandelt.

Evidenzgrundlage

Die Studienlage zu unterschiedlichen künstlerischen Therapien ist variabel. Vergleiche zwischen Studien ähnlicher künstlerischer Therapien sind oft erschwert aufgrund unterschiedlicher Studiendesigns. Kontrollgruppen umfassen häufig ein nicht aktives Pat.-Kollektiv (standard of care). Eine Bewertung des Nutzens von künstlerischen Therapien erfolgte daher überwiegend anhand von Übersichtsartikeln/Metaanalysen.

Ergebnis

In den Metaanalysen werden insbesondere Künstlerische Therapien mit Tanztherapie, Tai-Chi und musikunterstütztem rhythmischem Gangtraining untersucht und zumeist gemeinsam ausgewertet. Bei Tanztherapien werden sowohl Tango als auch Walzer/Foxtrott getanzt. Die Therapiefrequenz betrug zumeist zweimal pro Woche und die Therapiedauer betrug zumeist drei Monate oder mehr.

Es zeigten sich allgemeine Verbesserungen von Mobilität und Gleichgewicht [43] sowie im UPDRS, in der Berg Balance Scale und im Timed Up and Go Test [44], [45], [46], [1]. Die Effekte wurden dabei zumeist als moderat bezeichnet. Teilweise wurden Gangparameter berücksichtigt, bei denen sich eine Erhöhung der Ganggeschwindigkeit zeigte, aber keine signifikante Veränderung von Schrittlänge oder Freezing of Gait [45], [46], [1], [47]. Veränderungen der Lebensqualität anhand des Parkinson Disease Questionnaire-39 ergaben variable Ergebnisse mit Verbesserungen [44] oder fehlendem statistischem Unterschied zu den Vergleichsgruppen [45], [47]. Kognitive Funktionen zeigten sich teilweise verbessert ([46]; im Montreal Cognitive Assessment) und teilweise nicht verbessert [44], [47].

Demnach können Künstlerische Therapien zu einer Verbesserung von motorischen Funktionen führen und hier auch des Gleichgewichts, während sich bei Lebensqualität und kognitiven Funktionen variable Ergebnisse zeigten.

Begründung der Empfehlung

Auf der Basis der oben dargestellten Evidenzgrundlage wird zur Verordnung von künstlerischen Therapien die u.g. Empfehlung gegeben.

**Empfehlung**	**Neu**<br><br>**Stand (2023)**
Bezüglich der Effektivität von künstlerischen Therapien bei Parkinson-Pat. kann folgende Empfehlung gegeben werden:<br>Parkinson-Pat. sollten Zugang zu künstlerischen Therapien erhalten.	
Konsensstärke: 100%, starker Konsens	

Referenzen

1. Radder DLM, Lígia Silva de Lima A, Domingos J, Keus SHJ, van Nimwegen M, Bloem BR, de Vries NM. Physiotherapy in Parkinson's Disease: A Meta-Analysis of Present Treatment Modalities. Neurorehabil Neural Repair. 2020;34(10):871-80
2. Peek AL, Stevens ML. Resistance training for people with Parkinson's disease (PEDro synthesis). Br J Sports Med. 2016;50(18):1158
3. Cugusi L, Manca A, Bergamin M, Di Blasio A, Monticone M, Deriu F, Mercuro G. Aquatic exercise improves motor impairments in people with Parkinson's disease, with similar or greater benefits than land-based exercise: a systematic review. J Physiother. 2019;65(2):65-74
4. Tomlinson CL, Patel S, Meek C, Herd CP, Clarke CE, Stowe R, et al. Physiotherapy versus placebo or no intervention in Parkinson's disease. Cochrane Database Syst Rev. 2013;2013(9):Cd002817
5. Flach A, Jaegers L, Krieger M, Bixler E, Kelly P, Weiss EP, Ahmad SO. Endurance exercise improves function in individuals with Parkinson's disease: A meta-analysis. Neurosci Lett. 2017;659:115-9
6. Garcia-Agundez A, Folkerts AK, Konrad R, Caserman P, Tregel T, Goosses M, et al. Recent advances in rehabilitation for Parkinson's Disease with Exergames: A Systematic Review. J Neuroeng Rehabil. 2019;16(1):17

7. Volpe D, Giantin MG, Manuela P, Filippetto C, Pelosin E, Abbruzzese G, Antonini A. Water-based vs. non-water-based physiotherapy for rehabilitation of postural deformities in Parkinson's disease: a randomized controlled pilot study. Clin Rehabil. 2017;31(8):1107-15

8. Ćwiękała-Lewis KJ, Gallek M, Taylor-Piliae RE. The effects of Tai Chi on physical function and well-being among persons with Parkinson's Disease: A systematic review. J Bodyw Mov Ther. 2017;21(2):414-21

9. Li Y, Song H, Shen L, Wang Y. The efficacy and safety of moderate aerobic exercise for patients with Parkinson's disease: a systematic review and meta-analysis of randomized controlled trials. Ann Palliat Med. 2021;10(3):2638-49

10. Carroll LM, Morris ME, O'Connor WT, Clifford AM. Is Aquatic Therapy Optimally Prescribed for Parkinson's Disease? A Systematic Review and Meta-Analysis. J Parkinsons Dis. 2020;10(1):59-76

11. Rutz DG, Benninger DH. Physical Therapy for Freezing of Gait and Gait Impairments in Parkinson Disease: A Systematic Review. Pm r. 2020;12(11):1140-56

12. Shen X, Wong-Yu IS, Mak MK. Effects of Exercise on Falls, Balance, and Gait Ability in Parkinson's Disease: A Meta-analysis. Neurorehabil Neural Repair. 2016;30(6):512-27

13. Cosentino C, Baccini M, Putzolu M, Ristori D, Avanzino L, Pelosin E. Effectiveness of Physiotherapy on Freezing of Gait in Parkinson's Disease: A Systematic Review and Meta-Analyses. Mov Disord. 2020;35(4):523-36

14. Bombieri F, Schena F, Pellegrini B, Barone P, Tinazzi M, Erro R. Walking on four limbs: A systematic review of Nordic Walking in Parkinson disease. Parkinsonism Relat Disord. 2017;38:8-12

15. Saltychev M, Bärlund E, Paltamaa J, Katajapuu N, Laimi K. Progressive resistance training in Parkinson's disease: a systematic review and meta-analysis. BMJ Open. 2016;6(1):e008756

16. Kurt EE, Büyükturan B, Büyükturan Ö, Erdem HR, Tuncay F. Effects of Ai Chi on balance, quality of life, functional mobility, and motor impairment in patients with Parkinson's disease<sup/>. Disabil Rehabil. 2018;40(7):791-7

17. Santos L, Fernandez-Rio J, Winge K, Barragán-Pérez B, González-Gómez L, Rodríguez-Pérez V, et al. Effects of progressive resistance exercise in akinetic-rigid Parkinson's disease patients: a randomized controlled trial. Eur J Phys Rehabil Med. 2017;53(5):651-63

18. Pérez de la Cruz S. Effectiveness of aquatic therapy for the control of pain and increased functionality in people with Parkinson's disease: a randomized clinical trial. Eur J Phys Rehabil Med. 2017;53(6):825-32

19. Gandolfi M, Tinazzi M, Magrinelli F, Busselli G, Dimitrova E, Polo N, et al. Four-week trunk-specific exercise program decreases forward trunk flexion in Parkinson's disease: A single-blinded, randomized controlled trial. Parkinsonism Relat Disord. 2019;64:268-74

20. Hubble RP, Naughton G, Silburn PA, Cole MH. Trunk Exercises Improve Gait Symmetry in Parkinson Disease: A Blind Phase II Randomized Controlled Trial. Am J Phys Med Rehabil. 2018;97(3):151-9

21. Silva-Batista C, Corcos DM, Kanegusuku H, Piemonte MEP, Gobbi LTB, de Lima-Pardini AC, et al. Balance and fear of falling in subjects with Parkinson's disease is improved after exercises with motor complexity. Gait Posture. 2018;61:90-7

22. Strouwen C, Molenaar E, Münks L, Broeder S, Ginis P, Bloem BR, et al. Determinants of Dual-Task Training Effect Size in Parkinson Disease: Who Will Benefit Most? J Neurol Phys Ther. 2019;43(1):3-11

23. Geroin C, Nonnekes J, de Vries NM, Strouwen C, Smania N, Tinazzi M, et al. Does dual-task training improve spatiotemporal gait parameters in Parkinson's disease? Parkinsonism Relat Disord. 2018;55:86-91

24. Soke F, Guclu-Gunduz A, Kocer B, Fidan I, Keskinoglu P. Task-oriented circuit training combined with aerobic training improves motor performance and balance in people with Parkinson's Disease. Acta Neurol Belg. 2021;121(2):535-43

25. Sparrow D, DeAngelis TR, Hendron K, Thomas CA, Saint-Hilaire M, Ellis T. Highly Challenging Balance Program Reduces Fall Rate in Parkinson Disease. J Neurol Phys Ther. 2016;40(1):24-30

26. Silva-Batista C, Corcos DM, Barroso R, David FJ, Kanegusuku H, Forjaz C, et al. Instability Resistance Training Improves Neuromuscular Outcome in Parkinson's Disease. Med Sci Sports Exerc. 2017;49(4):652-60

27. Silva-Batista C, Corcos DM, Roschel H, Kanegusuku H, Gobbi LT, Piemonte ME, et al. Resistance Training with Instability for Patients with Parkinson's Disease. Med Sci Sports Exerc. 2016;48(9):1678-87

28. Clarke CE, Patel S, Ives N, Rick CE, Dowling F, Woolley R, et al. Physiotherapy and Occupational Therapy vs No Therapy in Mild to Moderate Parkinson Disease: A Randomized Clinical Trial. JAMA Neurol. 2016;73(3):291-9

29. Clarke CE, Patel S, Ives N, Rick CE, Woolley R, Wheatley K, et al. Clinical effectiveness and cost-effectiveness of physiotherapy and occupational therapy versus no therapy in mild to moderate Parkinson's disease: a large pragmatic randomised controlled trial (PD REHAB). Health Technol Assess. 2016;20(63):1-96

30. Ashburn A, Pickering R, McIntosh E, Hulbert S, Rochester L, Roberts HC, et al. Exercise- and strategy-based physiotherapy-delivered intervention for preventing repeat falls in people with Parkinson's: the PDSAFE RCT. Health Technol Assess. 2019;23(36):1-150

31. Schenkman M, Moore CG, Kohrt WM, Hall DA, Delitto A, Comella CL, et al. Effect of High-Intensity Treadmill Exercise on Motor Symptoms in Patients With De Novo Parkinson Disease: A Phase 2 Randomized Clinical Trial. JAMA Neurol. 2018;75(2):219-26

32. Rafferty MR, Prodoehl J, Robichaud JA, David FJ, Poon C, Goelz LC, et al. Effects of 2 Years of Exercise on Gait Impairment in People With Parkinson Disease: The PRET-PD Randomized Trial. J Neurol Phys Ther. 2017;41(1):21-30

33. Cascaes da Silva F, Iop Rda R, Domingos Dos Santos P, Aguiar Bezerra de Melo LM, Barbosa Gutierres Filho PJ, da Silva R. Effects of Physical-Exercise-Based Rehabilitation Programs on the Quality of Life of Patients With Parkinson's Disease: A Systematic Review of Randomized Controlled Trials. J Aging Phys Act. 2016;24(3):484-96

34. Marumoto K, Yokoyama K, Inoue T, Yamamoto H, Kawami Y, Nakatani A, et al. Inpatient Enhanced Multidisciplinary Care Effects on the Quality of Life for Parkinson Disease: A Quasi-Randomized Controlled Trial. J Geriatr Psychiatry Neurol. 2019;32(4):186-94

35. Stożek J, Rudzińska M, Pustułka-Piwnik U, Szczudlik A. The effect of the rehabilitation program on balance, gait, physical performance and trunk rotation in Parkinson's disease. Aging Clin Exp Res. 2016;28(6):1169-77

36. Sturkenboom IH, Graff MJ, Hendriks JC, Veenhuizen Y, Munneke M, Bloem BR, Nijhuis-van der Sanden MW. Efficacy of occupational therapy for patients with Parkinson's disease: a randomised controlled trial. Lancet Neurol. 2014;13(6):557-66

37. Collett J, Franssen M, Winward C, Izadi H, Meaney A, Mahmoud W, et al. A long-term self-managed handwriting intervention for people with Parkinson's disease: results from the control group of a phase II randomized controlled trial. Clin Rehabil. 2017;31(12):1636-45

38. Nackaerts E, Heremans E, Vervoort G, Smits-Engelsman BC, Swinnen SP, Vandenberghe W, et al. Relearning of Writing Skills in Parkinson's Disease After Intensive Amplitude Training. Mov Disord. 2016;31(8):1209-16

39. Ziliotto A, Cersosimo MG, Micheli FE. Handwriting Rehabilitation in Parkinson Disease: A Pilot Study. Ann Rehabil Med. 2015;39(4):586-91

40. Welsby E, Berrigan S, Laver K. Effectiveness of occupational therapy intervention for people with Parkinson's disease: Systematic review. Aust Occup Ther J. 2019;66(6):731-8

41. Foster ER, Carson LG, Archer J, Hunter EG. Occupational Therapy Interventions for Instrumental Activities of Daily Living for Adults With Parkinson's Disease: A Systematic Review. Am J Occup Ther. 2021;75(3):7503190030p1-p24

42. Tofani M, Ranieri A, Fabbrini G, Berardi A, Pelosin E, Valente D, et al. Efficacy of Occupational Therapy Interventions on Quality of Life in Patients with Parkinson's Disease: A Systematic Review and Meta-Analysis. Mov Disord Clin Pract. 2020;7(8):891-901

43. Zhang M, Li F, Wang D, Ba X, Liu Z. Exercise sustains motor function in Parkinson's disease: Evidence from 109 randomized controlled trials on over 4,600 patients. Front Aging Neurosci. 2023;15:1071803

44. Zhang S, Liu D, Ye D, Li H, Chen F. Can music-based movement therapy improve motor dysfunction in patients with Parkinson's disease? Systematic review and meta-analysis. Neurol Sci. 2017;38(9):1629-36

45. Dos Santos Delabary M, Komeroski IG, Monteiro EP, Costa RR, Haas AN. Effects of dance practice on functional mobility, motor symptoms and quality of life in people with Parkinson's disease: a systematic review with meta-analysis. Aging Clin Exp Res. 2018;30(7):727-35

46. Kalyani HHN, Sullivan K, Moyle G, Brauer S, Jeffrey ER, Roeder L, et al. Effects of Dance on Gait, Cognition, and Dual-Tasking in Parkinson's Disease: A Systematic Review and Meta-Analysis. J Parkinsons Dis. 2019;9(2):335-49

47. Lee H, Ko B. Effects of Music-Based Interventions on Motor and Non-Motor Symptoms in Patients with Parkinson's Disease: A Systematic Review and Meta-Analysis. Int J Environ Res Public Health. 2023;20(2)

3.24 Krankheitsmodifizierende Therapien

Autoren: Matthias Höllerhage, Thomas Köglsperger, Paul Lingor, Mathias Bähr

Fragestellung 186: Gibt es eine Evidenz dafür, dass für die symptomatische Therapie der PK zugelassene Medikamente die Progressionsrate der PK reduzieren können?

Hintergrund

Die Therapie der PK ist gegenwärtig ausschließlich symptomatisch [1]. In Zukunft sollte es ein Therapieziel sein, mit der Therapie den Krankheitsverlauf der PK aufzuhalten oder wenigstens zu verzögern. Für die PK zugelassene Medikamente wurden deswegen in der Vergangenheit auch hinsichtlich eines krankheitsmodifizierenden Effekts untersucht.

Evidenzgrundlage

Es gibt eine Reihe von Studien, die einen möglichen krankheitsmodifizierenden Effekt von für die PK zugelassenen Medikamenten untersucht haben. Keine Studie konnte einen krankheitsmodifizierenden Effekt nachweisen. Die Ergebnisse der wichtigsten aktuellen Studien werden im Folgenden vorgestellt.

Ergebnis

Dopaminagonisten: In den 2000er-Jahren wurde ein neuroprotektiver Effekt von Dopaminagonisten diskutiert. Anfang der 2000er-Jahre wurden Pramipexol (CALM-PD) und Ropinirol (REAL-PET) in Bildgebungsstudien mit Levodopa verglichen und eine positive Tendenz bezüglich einer langsameren dopaminergen Degeneration unter Therapie mit den Dopaminagonisten gefunden, die sich aber klinisch nicht widerspiegelte [2]. Neuere Studien, in denen ein möglicher neuroprotektiver Effekt von Dopaminagonisten untersucht wurde, existieren nicht. Zusammenfassend gibt es keinen Beweis dafür, dass Dopaminagonisten neuroprotektiv wirken.

Levodopa: In der ELLDOPA-Studie aus dem Jahr 2004 wurde gefunden, dass Pat., die mit Levodopa behandelt wurden, einen langsameren Krankheitsverlauf hatten [3]. Eine neuere Studie aus dem Jahr 2019 konnte diesen Effekt nicht bestätigen [4]. Zusammenfassend gibt es keinen Beweis dafür, dass Levodopa neuroprotektiv wirkt.

MAO-B-Hemmer: Die Frage nach einem möglichen neuroprotektiven bzw. krankheitsmodifizierenden Effekt von MAO-B-Hemmern ist weiterhin nicht abschließend geklärt. Ein neuroprotektiver bzw. krankheitsmodifizierender Effekt von MAO-B-Hemmern konnte nie eindeutig gezeigt worden. In der TEMPO-Studie waren 371 Parkinson-Pat. zunächst 6 Monate mit 1 mg oder 2 mg Rasagilin (early start) bzw. Placebo behandelt worden. Die Placebogruppe wurde nach 6 Monaten dann ebenfalls mit 2 mg Rasagilin behandelt (delayed start). Dabei hatte sich gezeigt, dass die Gruppe der Pat., die von Anfang an Rasagilin erhalten hatten, nach weiteren 6 Monaten klinisch weniger stark betroffen war als die Gruppe der Pat., die erst nach 6 Monaten Rasagilin erhalten hatten, was als möglicher krankheitsmodifizierender Effekt interpretiert werden könnte [5].

In der ADAGIO-Studie waren 1.176 Parkinson-Pat. randomisiert entweder gleich (early start) oder nach 24 Wochen (delayed start) mit 1 mg oder 2 mg Rasagilin behandelt worden. Im Vergleich der

Gruppen der Pat., die mit 1 mg behandelt wurden (early start vs. delayed start), hatte es nach 72 Wochen einen Anhalt für einen langsameren Krankheitsprozess als bei den Pat., die früher mit Rasagilin behandelt wurden, gezeigt. Interessanterweise wurde in den 2-mg-Gruppen dieser Effekt nicht gefunden [6], womit letztlich die Ergebnisse der TEMPO-Studie nicht reproduziert werden konnten. Die Sekundäranalyse einer Studie, die im Jahr 2017 veröffentlicht wurde, suggeriert, dass eine längere Behandlungsdauer mit MAO-B-Hemmern zu einer weniger starken klinischen Verschlechterung führte [7]. Letztlich gibt es weiterhin keinen eindeutigen Beweis für einen neuroprotektiven Effekt von MAO-B-Hemmern.

COMT-Hemmer: Es gibt keine Studie, in der ein möglicherweise krankheitsmodifizierender Effekt von COMT-Hemmern untersucht wurde.

Begründung der Empfehlung

Auf Basis der o.g. Evidenzgrundlage wird folgende Empfehlung gegeben.

**Empfehlung**	**Neu**<br><br>**Stand (2023)**
Für die symptomatische Therapie einer PK zugelassene Medikamente sollen nicht mit der Indikation der Reduktion der Progressionsrate angeboten werden.	
Konsensstärke: 96,2%, starker Konsens	

Fragestellung 187: Gibt es eine Evidenz dafür, dass für andere Krankheiten zugelassene Medikamente die Progressionsrate der PK reduzieren können?

Hintergrund

„Drug-repurposing“, d.h. die Nutzung von bereits zugelassenen Medikamenten für einen neuen Indikationsbereich, ist neben der Zulassung gänzlich neu entwickelter Substanzen ein wichtiger Pfeiler in der Entwicklung neuer therapeutischer Optionen [8]. Daher wurden in der Vergangenheit zahlreiche Substanzen, die bereits für andere Indikationen beim Menschen zugelassen sind, auf ihre Wirksamkeit in Bezug auf die Verlangsamung der Progressionsrate der PK untersucht [9].

Evidenzgrundlage

Es gibt keine randomisierte placebo-ontrollierte Studie, die zeigen konnte, dass ein nicht für die PK zugelassenes Medikament einen krankheitsmodifizierenden Effekt auf die PK hat. Die Studienlage hinsichtlich dreier prominenter Beispiele ist hier exemplarisch herausgegriffen.

Ergebnis

Ambroxol: Mutationen des Glukozerebrosidase-Gens, *GBA1*, stellen den wichtigsten genetischen Risikofaktor für die PK (PD) dar. In-vitro- und In-vivo-Studien haben gezeigt, dass die als Schleimlöser zugelassene Substanz Ambroxol die Aktivität des Enzyms β-Glukozerebrosidase (GCase) erhöht und damit α-Synuclein-Spiegel reduziert. Aufgrund dieser Beobachtungen wurde Ambroxol als krankheitsmodifizierendes Agens bei Parkinson-Pat. untersucht. Im Rahmen einer monozentrischen

Open-label-, nicht-placebokontrollierten Studie wurden 18 Pat. eingeschlossen, die bis zu 1.26 g Ambroxol pro Tag für 186 Tage erhielten. Die Therapie war sicher und wurde gut vertragen. Eine Aussage bezüglich Wirksamkeit kann mangels Placebogruppe nicht getroffen werden [10]. Prospektive, multizentrische, placebokontrollierte Analysen sind erforderlich, um hier eine belastbare Aussage zu treffen.

Isradipin: Der Dihydropyridin-Kalzium-Kanal-Hemmer Isradipin konnte in Tierversuchen exzitotoxischen Einstrom von extrazellulärem Kalzium reduzieren und dadurch neuroprotektiv wirken. Isradipin ist bereits als Antihypertensivum zugelassen. In einer multizentrischen, doppel-blinden, placebokontrollierten Studie wurden 336 Pat. mit 5 mg Isradipin oder Placebo für 36 Monate behandelt. Das Medikament war sicher und wurde zumeist gut vertragen. Die Analyse des primären Endpunkts, die Veränderung des UPDRS total score zwischen baseline und 36 Monaten (least-squares mean), zeigte keinen signifikanten Unterschied zwischen beiden Behandlungsgruppen (2.99 vs. 3.26, P=0.85) [11].

Deferipron: Der Eisengehalt in der Substantia nigra von Parkinson-Pat. ist erhöht und könnte zur Pathophysiologie der Erkrankung beitragen. Der Eisenchelator Deferipron konnte den Eisengehalt in der Substantia nigra bei Parkinson-Pat. senken [12]. In einer multizentrischen, doppelblinden, placebokontrollierten Studie wurden 372 Parkinson-Pat. mit 15 mg/kgKG Deferipron oder Placebo zweimal täglich für 36 Wochen behandelt. Die Analyse des primären Endpunkts, die Veränderung des UPDRS total score zwischen Baseline und 36 Wochen, zeigte eine signifikante Verschlechterung in der Deferipron-Gruppe im Vergleich zur Placebogruppe (15.6 vs. 6.3 Punkte, P<.001) [13].

Begründung der Empfehlung

Auf Basis der o.g. Evidenzgrundlage wird folgende Empfehlung gegeben.

**Empfehlung**	**Neu**<br><br>**Stand (2023)**
Für andere Erkrankungen zugelassene Medikamente sollen nicht für die Reduktion der Progressionsrate der PK angeboten werden.	
Konsensstärke: 100%, starker Konsens	

Fragestellung 188: Gibt es eine Evidenz dafür, dass Nahrungsergänzungsmittel oder nicht als Medikamente zugelassene Substanzen die Progressionsrate der PK reduzieren können?

Hintergrund

Es gibt eine Reihe von Nahrungsergänzungsmitteln, die möglicherweise einen Effekt auf die Progressionsrate der PK haben könnten. Dabei spielen auf der einen Seite pathophysiologische Erwägungen eine Rolle, sodass solche Substanzen untersucht wurden, die möglicherweise positiv in die Pathophysiologie der PK eingreifen. Zum anderen gibt es epidemiologische Beobachtungen, die für bestimmte Substanzen (v.a. Koffein und Nikotin) einen möglicherweise krankheitsmodifizierenden Effekt suggerieren.

Evidenzgrundlage

In der Vergangenheit wurden eine Reihe von präklinischen und klinischen Studien durchgeführt, in denen verschiedene Nahrungsergänzungsmittel oder nicht als Medikamente zugelassene Substanzen hinsichtlich eines möglichen neuroprotektiven Effekts bei der PK untersucht wurden. Im Folgenden wird die Datenlage für die einzelnen Nahrungsergänzungsmittel oder Substanzen kurz vorgestellt.

Ergebnis

Vitamin E (Alpha-Tocopherol): Vitamin E (Tocopherol), ein fettlösliches Antioxidans, hat allein oder in Kombination keinen klinischen Effekt auf die PK [9]. Daher kann eine Empfehlung für den Einsatz von Vitamin E mit der Indikation, die Progressionsrate der PK zu reduzieren, nicht ausgesprochen werden.

Niacin (Vitamin B3): Niacin hat ist ein Vorläufer von NADH. In präklinischen Toxinmodellen konnte Niacin Neurone in der Substantia nigra schützen [14]. Klinische Daten zur Anwendung von Niacin bei der PK existieren nicht, sodass keine Empfehlung für den klinischen Einsatz ausgesprochen werden kann.

Nikotin: Epidemiologische Daten weisen darauf hin, dass Nikotinkonsum zu einer reduzierten Rate des Auftretens einer PK führt [15]. Zudem haben präklinische Daten suggeriert, dass Nikotin den Krankheitsverlauf verlangsamen könnte [14]. Zuletzt wurde eine klinische Studie (NIC-PD) durchgeführt, bei der De-novo-Parkinson-Pat. mit Nikotinpflaster (N= 79) oder Placebo (N=83) über 18 Monate behandelt wurden. Im Gegensatz zu dem erwarteten Effekt einer geringeren Krankheitsprogression in der mit Nikotin behandelten Gruppe zeigten sich in dieser Gruppe Hinweise auf einen beschleunigten Krankheitsverlauf [16].

Zusammenfassend gibt es keinen Beweis dafür, dass Nikotin die Progressionsrate der PK reduzieren kann. Im Gegenteil gibt es Hinweise für einen möglicherweise symptomatischen negativen Effekt.

Coenzym Q10: Coenzym Q10 ist das Coenzym des Komplex I der Atmungskette. Weil bei Zellen von Pat. mit einer PK niedrige Aktivitäten des Komplex I der Atmungskette gemessen wurden, gibt es eine Rationale dafür, Coenzym Q10 zu substituieren. In klinischen Studien konnte hingegen kein Effekt auf die Progressionsrate der PK durch Coenzym-Q10-Substitution allein oder in Kombination mit Vitamin E beobachtet werden [9], [15]. Zusammenfassend kann keine Empfehlung für den Einsatz von Coenzym Q10 mit der Indikation, die Progressionsrate der PK zu reduzieren, ausgesprochen werden.

Vitamin C: Vitamin C ist ein Antioxidans. In präklinischen Modellen hat Vitamin C einen Effekt auf die Aggregation von Alpha-Synuklein. Die Serumspiegel von Vitamin C unterscheiden sich zwischen Pat. mit einer PK und gesunden Kontrollpersonen nicht. In verschiedenen Studien wurde nicht gefunden, dass die erhöhte Einnahme von Vitamin C das Risiko der Entwicklung einer PK reduziert. In klinischen Studien mit sehr kleiner Pat.-Zahl wurde gefunden, dass die Einnahme großer Mengen von Vitamin C und Vitamin E zu einer Verzögerung der Einnahme von Levodopa um 2,5 Jahre geführt hat. Zusammenfassend gibt es keine Daten, die beweisen, dass Vitamin C die Progressionsrate der PK reduziert [17].

Gluthathion: Glutathion ist ein Antioxidans, das in präklinischen Parkinson-Modellen als Kofaktor protektiv wirkte [14]. Daten aus klinischen Studien bei Parkinson-Pat. existieren nicht, sodass keine Empfehlung für den Einsatz Glutathion mit der Indikation, die Progressionsrate der PK zu reduzieren, ausgesprochen werden kann.

Molkeprotein: In einer Pilotstudie konnte gezeigt werden, dass Molkeprotein einen Effekt auf die Plasma-Glutathion-Spiegel hat. Dieser Effekt hatte aber keinen Einfluss auf den klinischen Befund bei Parkinson-Pat. [18]. Eine Empfehlung für den Einsatz von Molkeprotein mit der Indikation, die Progressionsrate der PK zu reduzieren, kann nicht ausgesprochen werden.

Koffein: Zu Koffein gibt es epidemiologische Daten, die zeigen, dass der Koffeinkonsum bei Parkinson-Pat. niedriger ist als bei gesunden Personen, wobei dies nur auf Männer, nicht aber auf Frauen zutrifft [15], [19]. Es gibt zudem epidemiologische Daten, die darauf hindeuten, dass bei Parkinson-Pat. mit einem höheren Koffeinkonsum später als bei Pat., die wenig oder kein Koffein zu sich nehmen, eine Levodopa-Therapie notwendig wird [20]. Eine klinische Studie, bei der 60 Parkinson-Pat. Koffein eingenommen haben und 61 ein Placebo, hat hingegen keinen positiven klinischen Effekt durch Koffein gezeigt [21]. Eine rezente Metaanalyse konnte bestätigen, dass Koffeinkonsumenten ein geringeres Risiko haben, an einer PK zu erkranken [22].

Zusammenfassend ist ein Beweis dafür, dass Koffein die Progressionsrate der PK reduzieren kann, bisher nicht erbracht worden. Epidemiologische Studien deuten an, dass Koffein protektiv bei der PK wirken könnte. Die Zusammenhänge sollten aber vorsichtig betrachtet werden, da Faktoren wie Primärpersönlichkeit, Suchtverhalten etc. einen Einfluss auf den Zusammenhang zwischen Koffeinkonsum und dem Auftreten einer PK haben, die nicht mit einem krankheitsmodifizierenden Effekt in Zusammenhang stehen.

Curcumin: Curcumin ist ein Polyphenol, das aus der Gelbwurz (Curcuma longa) gewonnen wird. Dem Mittel werden antioxidative und antiinflammatorische Eigenschaften zugeschrieben. Die molekularen Mechanismen, die dahinterstehen, sind aber weitgehend unbekannt. In Zell- und Tiermodellen hat Curcumin neuroprotektive Eigenschaften gezeigt. Studien, die einen neuroprotektiven Effekt bei Parkinson-Pat. zeigen, existieren nicht [23]. Zum gegenwärtigen Zeitpunkt kann daher nicht empfohlen werden, Curcumin mit der Indikation, die Progressionsrate der PK zu reduzieren, einzusetzen.

Omega-3-Fettsäuren: Epidemiologische Studien haben gezeigt, dass ein hoher Konsum ungesättigter Fettsäuren mit einem niedrigeren Risiko, eine PK zu entwickeln, assoziiert ist. Es gibt nur wenige kontrollierte Studien, in denen Omega-3-Fettsäuren bei der PK untersucht wurden, die jeweils keinen Effekt gezeigt haben [24].

Probiotika: Zur Frage nach der Wirkung von Probiotika bei der PK gibt es eine rezente Übersichtsarbeit. Zusammengefasst könnten Probiotika in der Zukunft eine Rolle in der Therapie der PK spielen. Aktuell ist die Studienlage, auch wegen der insgesamt sehr hohen Variabilität des Darmmikrobioms, noch unübersichtlich und weitere Studien sind nötig, um die Frage nach dem Einsatz von Probiotika bei der PK in Zukunft bewerten zu können [25].

Zusammenfassend gibt es keine ausreichenden Beweise dafür, dass Nahrungsergänzungsmittel oder nicht als Medikamente zugelassene Substanzen die Progressionsrate der PK reduzieren können.

Begründung der Empfehlung

Auf Basis der oben dargestellten Evidenzgrundlage wird folgende Empfehlung gegeben.

**Empfehlung**	**Neu**<br><br>**Stand (2023)**
Nahrungsergänzungsmittel oder nicht als Medikamente zugelassene Substanzen sollen nicht mit der Indikation der Reduktion der Progressionsrate einer PK angeboten werden.	
Konsensstärke: 96,6%, starker Konsens	

Fragestellung 189: Gibt es eine Evidenz dafür, dass physiotherapeutische/physikalische Verfahren die Progressionsrate der PK reduzieren können?

Hintergrund

In den letzten zwei Jahrzehnten haben Studien gezeigt, dass physikalische Therapien mit verschiedenen Übungsansätzen in der Rehabilitation bei Parkinson-Pat. effektiv sind [26]. Die erste evidenzbasierte europäische Physiotherapie-Leitlinie für Parkinson empfiehlt physikalische Therapie als Intervention zur Verbesserung von Gleichgewicht, Gang, Muskelkraft, aerober Kapazität und funktioneller Mobilität [27].

Evidenzgrundlage

Für viele physikalische Therapieverfahren existieren randomisierte kontrollierte Studien über einen Beobachtungszeitraum von Wochen bis Monate. Längere Beobachtungszeiträume wurden bis auf wenige Ausnahmen [28] nicht untersucht.

Ergebnis

Körperliche Bewegung und Physiotherapie können bei Parkinson-Pat. motorische Symptome lindern, die Muskelkraft, das Gleichgewicht, die aerobe Kapazität und die körperliche Funktionsfähigkeit verbessern. Eine Cochrane-Metaanalyse ergab positive Auswirkungen von körperlicher Bewegung im Vergleich zu passiven Kontrollgruppen auf die Schwere der motorischen Symptome und die Lebensqualität [29], [30]. Die Unterschiede zwischen den verschiedenen Arten von körperlicher Betätigung waren gering. Einige Studien deuten darauf hin, dass es auch langfristige Effekte gibt, die den Verlauf der PK beeinflussen können [31], [32]. Nordic Walking, hochintensives Laufbandtraining, progressive Widerstandskrafttrainingsstudien [28] und Tangotanz als Form der Tanztherapie zeigten positive Ergebnisse [33].

Begründung der Empfehlung

Auf der Basis der oben dargestellten Evidenzgrundlage wird folgende Empfehlung gegeben.

**Empfehlung**	**Neu**<br>**Stand (2023)**
Physikalische Therapieverfahren verbessern bei der PK wahrscheinlich motorische Symptome, Gang, Gleichgewicht und möglicherweise die Lebensqualität im kurzfristigen Verlauf und verhindern möglicherweise muskuloskelettale Sekundärprobleme. Ob die Progression der PK dadurch signifikant verändert wird, ist unklar.<br>Physikalische Therapieverfahren sollten daher nicht mit der Indikation der Reduktion der Progressionsrate einer PK angeboten werden.	
Konsensstärke: 96,8%, starker Konsens	

Fragestellung 190: Gibt es eine Evidenz dafür, dass interventionelle Verfahren (z.B. Tiefe Hirnstimulation) die Progressionsrate der PK reduzieren können?

Hintergrund

Die Tiefe Hirnstimulation (THS) ist eine bewährte und effektive symptomatische Therapie bei der PK. Verschiedene Studien zeigen, dass die motorischen Symptome mit THS langfristig über einen Zeitraum von bis zu 16 Jahren verbessert werden können. Im MED Off sind die Score-Werte langfristig um 30% bis 50% reduziert und L-Dopa-induzierte motorische Komplikationen verbessern sich erheblich [34], [35], [36]. Daher kann man von einer langfristigen Wirksamkeit der THS ausgehen. Es ist jedoch unklar, ob die THS neben der symptomatischen Verbesserung auch den Verlauf der PK im engeren Sinn verändert (d.h., ob die THS in der Lage ist, den Verlauf der Krankheit über ihre symptomatischen Effekte hinaus positiv zu beeinflussen).

Evidenzgrundlage

Zur Frage, ob die THS die Krankheitsprogression der PK verändert, existieren keine prospektiven kontrollierten randomisierten Studien.

Ergebnis

Zusammenfassend gibt es theoretische und tierexperimentelle Modelle, die darauf hinweisen, dass die THS eine protektive Wirkung haben könnte [37], [38], [39], [40], [41]. Longitudinale Kohortenstudien an STN-THS-Pat. haben gezeigt, dass die jährliche Verschlechterungsrate des UPDRS III im Durchschnitt bei 1,0 Punkten pro Jahr liegt und somit etwas weniger als bei konventionell behandelten Pat. (1,4–2,6 Punkte/Jahr) [35], [42], [43], [44], [45], [46]. Allerdings ist die Aussagekraft dieser Studien begrenzt durch ihre unkontrollierten Studiendesigns und hohe Drop-out-Raten aufgrund des langen Follow-ups. Einige Studien haben gezeigt, dass THS-Pat. aufgrund einer Verlangsamung des Krankheitsverlaufs ein reduziertes Risiko für einen Aufenthalt in einem Pflegeheim, ein verringertes Sturzrisiko und eine geringere Wahrscheinlichkeit für Psychosen haben [47], [48], [49]. Studien zum THS-Entzugssyndrom sprechen gegen klinisch relevante krankheitsmodifizierende Effekte [50]. Einige wenige Studien haben Biomarker zur Krankheitsprogression bei Parkinson-Pat., die mit THS behandelt wurden, untersucht, konnten jedoch keine Hinweise auf potenzielle krankheitsmodifizierende Effekte finden [51]. Insgesamt gibt es keine klinischen Studien, die einen im engeren Sinn pathologie- oder krankheitsmodifizierenden

Effekt nahelegen, wenngleich die THS die mittlere Überlebenszeit etwas zu verlängern scheint [48], [52] [53], [54].

Begründung der Empfehlung

Auf der Basis der oben dargestellten Evidenzgrundlage wird folgende Empfehlung gegeben.

Empfehlung	Neu<br>Stand (2023)
Die STN-THS verzögert möglicherweise einige klinisch wichtige Meilensteine wie Stürze, Psychosen und die Notwendigkeit einer langfristigen Pflege und verlängert möglicherweise das mittlere Überleben, hat wahrscheinlich aber keinen pathologie- oder krankheitsmodifizierender Effekt im engeren Sinn bei der PK. Die THS soll daher nicht mit der Indikation der Reduktion der Progressionsrate einer PK angeboten werden.	
Konsensstärke: Abstimmungsergebnis 93,1%, Konsens	

Fragestellung 191: Gibt es eine Evidenz dafür, dass zellbasierte/gentherapeutische Verfahren die Progressionsrate der PK reduzieren können?

Hintergrund

Bei der PK kommt es zum Zellverlust dopaminerger Neurone in der Substatia nigra. Zellbasierte oder gentherapeutische Verfahren könnten dazu eingesetzt werden, den Zellverlust zu ersetzen oder durch Substitution neurotropher Faktoren den Zellverlust zu verhindern. Antikörper gegen Alpha-Synuklein könnten dazu verwendet werden, die Zell-zu-Zell-Ausbreitung der Alpha-Synuklein-Pathologie zu verhindern.

Evidenzgrundlage

Zur Frage nach zellbasierten/gentherapeutischen Verfahren gibt es wenige Studien. Im Folgenden wird die aktuelle Datenlage vorgestellt.

Ergebnis

In mehreren Phase-1-/Phase-1/2-Studien konnte die mögliche symptomatische Wirksamkeit einer Transplantation von Stammzellen (v.a. embroyonalen Ursprungs) bei einer kleinen Zahl von Pat. mit PK demonstriert werden. Bisher gibt es keine Arbeiten, die einen Einfluss auf die Progressionsrate nachweisen konnten [55]. Neue Studien werden die Wirksamkeit humaner induzierter pluripotenter Stammzellen (hiPSCs) untersuchen.

In Phase-1/2-Studien, in denen über Adeno-assoziierte virenbasierte Gentherapieverfahren verschiedene Gene (für aromatische Amino-Decarboxylase (AADC), Glialer neurotropher Faktor (GDNF)) eingebracht wurden, konnte zwar eine symptomatische Wirksamkeit, aber keine neuroprotektiven Effekte beobachtet werden [56].

In zwei vor Kurzem veröffentlichten Studien, in denen die Wirksamkeit einer Antikörpertherapie mit Bindung an Alpha-Synuklein untersucht wurde, konnte kein die Progressionsrate vermindernder Effekt festgestellt werden [57], [58].

Begründung der Empfehlung

Auf der Basis der oben dargestellten Evidenzgrundlage wird folgende Empfehlung gegeben.

**Empfehlung**	**Neu**<br><br>**Stand (2023)**
Zellbasierte/gentherapeutische Verfahren sollen nicht für die Reduktion der Progressionsrate der PK angeboten werden.	
Konsensstärke: 100%, starker Konsens	

Referenzen

1. Armstrong MJ, Okun MS. Diagnosis and Treatment of Parkinson Disease: A Review. Jama. 2020;323(6):548-60
2. Salamon A, Zádori D, Szpisjak L, Klivényi P, Vécsei L. Neuroprotection in Parkinson's disease: facts and hopes. J Neural Transm (Vienna). 2020;127(5):821-9
3. Fahn S, Oakes D, Shoulson I, Kieburtz K, Rudolph A, Lang A, et al. Levodopa and the progression of Parkinson's disease. N Engl J Med. 2004;351(24):2498-508
4. Verschuur CVM, Suwijn SR, Boel JA, Post B, Bloem BR, van Hilten JJ, et al. Randomized Delayed-Start Trial of Levodopa in Parkinson's Disease. N Engl J Med. 2019;380(4):315-24
5. A controlled, randomized, delayed-start study of rasagiline in early Parkinson disease. Arch Neurol. 2004;61(4):561-6
6. Olanow CW, Rascol O, Hauser R, Feigin PD, Jankovic J, Lang A, et al. A double-blind, delayed-start trial of rasagiline in Parkinson's disease. N Engl J Med. 2009;361(13):1268-78
7. Hauser RA, Li R, Pérez A, Ren X, Weintraub D, Elm J, et al. Longer Duration of MAO-B Hemmer Exposure is Associated with Less Clinical Decline in Parkinson's Disease: An Analysis of NET-PD LS1. J Parkinsons Dis. 2017;7(1):117-27
8. McFarthing K, Rafaloff G, Baptista M, Mursaleen L, Fuest R, Wyse RK, Stott SRW. Parkinson's Disease Drug Therapies in the Clinical Trial Pipeline: 2022 Update. J Parkinsons Dis. 2022;12(4):1073-82
9. Hung AY, Schwarzschild MA. ApproAcetylcholinesterase-Hemmer to Disease Modification for Parkinson's Disease: Clinical Trials and Lessons Learned. Neurotherapeutics. 2020;17(4):1393-405
10. Mullin S, Smith L, Lee K, D'Souza G, Woodgate P, Elflein J, et al. Ambroxol for the Treatment of Patients With Parkinson Disease With and Without Glucocerebrosidase Gene Mutations: A Nonrandomized, Noncontrolled Trial. JAMA Neurol. 2020;77(4):427-34
11. Isradipine Versus Placebo in Early Parkinson Disease: A Randomized Trial. Ann Intern Med. 2020;172(9):591-8
12. Bergsland N, Tavazzi E, Schweser F, Jakimovski D, Hagemeier J, Dwyer MG, Zivadinov R. Targeting Iron Dyshomeostasis for Treatment of Neurodegenerative Disorders. CNS Drugs. 2019;33(11):1073-86
13. Devos D, Labreuche J, Rascol O, Corvol JC, Duhamel A, Guyon Delannoy P, et al. Trial of Deferiprone in Parkinson's Disease. N Engl J Med. 2022;387(22):2045-55

14. Aaseth J, Dusek P, Roos PM. Prevention of progression in Parkinson's disease. Biometals. 2018;31(5):737-47

15. Ascherio A, Schwarzschild MA. The epidemiology of Parkinson's disease: risk factors and prevention. Lancet Neurol. 2016;15(12):1257-72

16. Oertel W, Müller H, Schade-Brittinger C, Kamp C, Balthasar K, Articus K, et al. The NIC-PD-study –A randomized, placebo-controlled, double-blind, multi-centre trial to assess the disease-modifying potential of transdermal nicotine in early Parkinson‘s disease in Germany and N. America [abstract]. Mov Disord. 2018;3328.03.2023

17. Moretti M, Fraga DB, Rodrigues ALS. Preventive and therapeutic potential of ascorbic acid in neurodegenerative diseases. CNS Neurosci Ther. 2017;23(12):921-9

18. Tosukhowong P, Boonla C, Dissayabutra T, Kaewwilai L, Muensri S, Chotipanich C, et al. Biochemical and clinical effects of Whey protein supplementation in Parkinson's disease: A pilot study. J Neurol Sci. 2016;367:162-70

19. Bakshi R, Macklin EA, Hung AY, Hayes MT, Hyman BT, Wills AM, et al. Associations of Lower Caffeine Intake and Plasma Urate Levels with Idiopathic Parkinson's Disease in the Harvard Biomarkers Study. J Parkinsons Dis. 2020;10(2):505-10

20. Moccia M, Erro R, Picillo M, Vitale C, Longo K, Amboni M, et al. Caffeine consumption and the 4-year progression of de novo Parkinson's disease. Parkinsonism Relat Disord. 2016;32:116-9

21. Postuma RB, Anang J, Pelletier A, Joseph L, Moscovich M, Grimes D, et al. Caffeine as symptomatic treatment for Parkinson disease (Café-PD): A randomized trial. Neurology. 2017;89(17):1795-803

22. Hong CT, Chan L, Bai CH. The Effect of Caffeine on the Risk and Progression of Parkinson's Disease: A Meta-Analysis. Nutrients. 2020;12(6)

23. Nebrisi EE. Neuroprotective Activities of Curcumin in Parkinson's Disease: A Review of the Literature. Int J Mol Sci. 2021;22(20)

24. Avallone R, Vitale G, Bertolotti M. Omega-3 Fatty Acids and Neurodegenerative Diseases: New Evidence in Clinical Trials. Int J Mol Sci. 2019;20(17)

25. Mirzaei H, Sedighi S, Kouchaki E, Barati E, Dadgostar E, Aschner M, Tamtaji OR. Probiotics and the Treatment of Parkinson's Disease: An Update. Cell Mol Neurobiol. 2022;42(8):2449-57

26. Mak MKY, Wong-Yu ISK. Exercise for Parkinson's disease. Int Rev Neurobiol. 2019;147:1-44

27. Domingos J, Keus SHJ, Dean J, de Vries NM, Ferreira JJ, Bloem BR. The European Physiotherapy Guideline for Parkinson's Disease: Implications for Neurologists. J Parkinsons Dis. 2018;8(4):499-502

28. Corcos DM, Robichaud JA, David FJ, Leurgans SE, Vaillancourt DE, Poon C, et al. A two-year randomized controlled trial of progressive resistance exercise for Parkinson's disease. Mov Disord. 2013;28(9):1230-40

29. Ernst M, Folkerts AK, Gollan R, Lieker E, Caro-Valenzuela J, Adams A, et al. Physical exercise for people with Parkinson's disease: a systematic review and network meta-analysis. Cochrane Database Syst Rev. 2023;1(1):Cd013856

30. Tomlinson CL, Patel S, Meek C, Herd CP, Clarke CE, Stowe R, et al. Physiotherapy versus placebo or no intervention in Parkinson's disease. Cochrane Database Syst Rev. 2013;2013(9):Cd002817

31. van Eijkeren FJ, Reijmers RS, Kleinveld MJ, Minten A, Bruggen JP, Bloem BR. Nordic walking improves mobility in Parkinson's disease. Mov Disord. 2008;23(15):2239-43

32. Schenkman M, Moore CG, Kohrt WM, Hall DA, Delitto A, Comella CL, et al. Effect of High-Intensity Treadmill Exercise on Motor Symptoms in Patients With De Novo Parkinson Disease: A Phase 2 Randomized Clinical Trial. JAMA Neurol. 2018;75(2):219-26

33. Duncan RP, Earhart GM. Randomized controlled trial of community-based dancing to modify disease progression in Parkinson disease. Neurorehabil Neural Repair. 2012;26(2):132-43

34. Fasano A, Romito LM, Daniele A, Piano C, Zinno M, Bentivoglio AR, Albanese A. Motor and cognitive outcome in patients with Parkinson's disease 8 years after subthalamic implants. Brain. 2010;133(9):2664-76

35. Volonté MA, Clarizio G, Galantucci S, Scamarcia PG, Cardamone R, Barzaghi LR, et al. Long term follow-up in advanced Parkinson's disease treated with DBS of the subthalamic nucleus. J Neurol. 2021;268(8):2821-30

36. Zibetti M, Merola A, Rizzi L, Ricchi V, Angrisano S, Azzaro C, et al. Beyond nine years of continuous subthalamic nucleus deep brain stimulation in Parkinson's disease. Mov Disord. 2011;26(13):2327-34

37. Jakobs M, Fomenko A, Lozano AM, Kiening KL. Cellular, molecular, and clinical mechanisms of action of deep brain stimulation-a systematic review on established indications and outlook on future developments. EMBO Mol Med. 2019;11(4)

38. Knorr S, Musacchio T, Paulat R, Matthies C, Endres H, Wenger N, et al. Experimental deep brain stimulation in rodent models of movement disorders. Exp Neurol. 2022;348:113926

39. Spieles-Engemann AL, Behbehani MM, Collier TJ, Wohlgenant SL, Steece-Collier K, Paumier K, et al. Stimulation of the rat subthalamic nucleus is neuroprotective following significant nigral dopamine neuron loss. Neurobiol Dis. 2010;39(1):105-15

40. Wallace BA, Ashkan K, Heise CE, Foote KD, Torres N, Mitrofanis J, Benabid AL. Survival of midbrain dopaminergic cells after lesion or deep brain stimulation of the subthalamic nucleus in MPTP-treated monkeys. Brain. 2007;130(Pt 8):2129-45

41. Musacchio T, Rebenstorff M, Fluri F, Brotchie JM, Volkmann J, Koprich JB, Ip CW. Subthalamic nucleus deep brain stimulation is neuroprotective in the A53T α-synuclein Parkinson's disease rat model. Ann Neurol. 2017;81(6):825-36

42. Merola A, Romagnolo A, Bernardini A, Rizzi L, Artusi CA, Lanotte M, et al. Earlier versus later subthalamic deep brain stimulation in Parkinson's disease. Parkinsonism Relat Disord. 2015;21(8):972-5

43. Castrioto A, Lozano AM, Poon YY, Lang AE, Fallis M, Moro E. Ten-year outcome of subthalamic stimulation in Parkinson disease: a blinded evaluation. Arch Neurol. 2011;68(12):1550-6

44. Lau B, Meier N, Serra G, Czernecki V, Schuepbach M, Navarro S, et al. Axial symptoms predict mortality in patients with Parkinson disease and subthalamic stimulation. Neurology. 2019;92(22):e2559-e70

45. Cilia R, Cereda E, Akpalu A, Sarfo FS, Cham M, Laryea R, et al. Natural history of motor symptoms in Parkinson's disease and the long-duration response to levodopa. Brain. 2020;143(8):2490-501

46. Mahlknecht P, Foltynie T, Limousin P, Poewe W. How Does Deep Brain Stimulation Change the Course of Parkinson's Disease? Mov Disord. 2022;37(8):1581-92

47. Ngoga D, Mitchell R, Kausar J, Hodson J, Harries A, Pall H. Deep brain stimulation improves survival in severe Parkinson's disease. J Neurol Neurosurg Psychiatry. 2014;85(1):17-22

48. Scelzo E, Beghi E, Rosa M, Angrisano S, Antonini A, Bagella C, et al. Deep brain stimulation in Parkinson's disease: A multicentric, long-term, observational pilot study. J Neurol Sci. 2019;405:116411

49. Mahlknecht P, Peball M, Mair K, Werkmann M, Nocker M, Wolf E, et al. Has Deep Brain Stimulation Changed the Very Long-Term Outcome of Parkinson's Disease? A Controlled Longitudinal Study. Mov Disord Clin Pract. 2020;7(7):782-7

50. Reuter S, Deuschl G, Falk D, Mehdorn M, Witt K. Uncoupling of dopaminergic and subthalamic stimulation: Life-threatening DBS withdrawal syndrome. Mov Disord. 2015;30(10):1407-13

51. Hilker R, Thomas AV, Klein JC, Weisenbach S, Kalbe E, Burghaus L, et al. Dementia in Parkinson disease: functional imaging of cholinergic and dopaminergic pathways. Neurology. 2005;65(11):1716-22

52. Pal GD, Ouyang B, Serrano G, Shill HA, Goetz C, Stebbins G, et al. Comparison of neuropathology in Parkinson's disease subjects with and without deep brain stimulation. Mov Disord. 2017;32(2):274-7

53. Pal G, Ouyang B, Verhagen L, Serrano G, Shill HA, Adler CH, et al. Probing the striatal dopamine system for a putative neuroprotective effect of deep brain stimulation in Parkinson's disease. Mov Disord. 2018;33(4):652-4

54. Weaver FM, Stroupe KT, Smith B, Gonzalez B, Huo Z, Cao L, et al. Survival in patients with Parkinson's disease after deep brain stimulation or medical management. Mov Disord. 2017;32(12):1756-63

55. Cha Y, Park TY, Leblanc P, Kim KS. Current Status and Future Perspectives on Stem Cell-Based Therapies for Parkinson's Disease. J Mov Disord. 2023;16(1):22-41

56. Deverman BE, Ravina BM, Bankiewicz KS, Paul SM, Sah DWY. Gene therapy for neurological disorders: progress and prospects. Nat Rev Drug Discov. 2018;17(9):641-59

57. Lang AE, Siderowf AD, Macklin EA, Poewe W, Brooks DJ, Fernandez HH, et al. Trial of Cinpanemab in Early Parkinson's Disease. N Engl J Med. 2022;387(5):408-20

58. Pagano G, Taylor KI, Anzures-Cabrera J, Marchesi M, Simuni T, Marek K, et al. Trial of Prasinezumab in Early-Stage Parkinson's Disease. N Engl J Med. 2022;387(5):421-32

3.25 Fahrtauglichkeit

Autoren: Günter Höglinger, Carsten Buhmann, Bruno Fimm

Fragestellung 192: Bei welchen Pat. mit der PK liegt eine Minderung der Fahreignung vor?

Hintergrund

Für viele Parkinson-Pat. ist das aktive Autofahren ein wesentlicher Bestandteil ihrer verbliebenen Unabhängigkeit, insbesondere bei krankheitsbedingt eingeschränkter Mobilität. Querschnittsstudien zeigen, dass 60% aller Parkinson-Pat. und 50% der Pat. mit Tiefer Hirnstimulation noch aktiv Auto fahren [1], [2]. Neben motorischen potenziell fahrrelevanten Beeinträchtigungen können Parkinson-Pat. schon in früher Erkrankungsphase Defizite exekutiver Funktionen sowie Beeinträchtigungen von visuellen Basisfunktionen (z.B. Kontrastsensitivität), Aufmerksamkeit und Gedächtnis aufweisen. Diese können sich auf das Fahrverhalten der Pat. negativ auswirken. Zusätzlich können im Verlauf der medikamentösen Behandlung Symptome vermehrter Tagesmüdigkeit, Sekundenschlaf und Störungen der Impulskontrolle als weitere die Fahreignung potenziell mindernde Faktoren auftreten. In der verpflichtenden (Details unter [3]) ärztlichen Beratung der Pat. bezüglich der Fahrkompetenz sollte zwischen einer „Diagnoseaufklärung“ über die Fahrtauglichkeit (syn. Fahreignung) und einer „Therapieaufklärung“ zur Fahrfähigkeit (syn. Fahrtüchtigkeit oder Fahrsicherheit) [4], [5], [6], [7] unterschieden werden. Während die Fahrtauglichkeit die generelle Fahreignung hinsichtlich der geistigen, körperlichen und charakterlichen Eignung beschreibt (z.B. Fahruntauglichkeit bei Demenz), bezieht sich die Fahrfähigkeit auf eine situations- und zeitbezogene Fahrtüchtigkeit, d.h., die aktuelle psychophysische Leistungsfähigkeit des Pat. [4] und ist durch äußere Faktoren sowie durch Beeinträchtigungen des Fahrers/der Fahrerin unter Umständen rasch veränderbar [5] (z.B. Sehstörungen nur im Dunkeln, passagere, medikamentös bedingte Nebenwirkung). Zu beachten ist, dass sowohl Pat. als auch behandelnde Neurologen/Neurologinnen häufig die Fahreignung der Pat. überschätzen [8], [9], [10].

Evidenzgrundlage

Expertenmeinung auf der Basis der zitierten Literatur.

Ergebnis

Obwohl Parkinson-Pat. in frühem bis mittlerem Stadium der Erkrankung im Durchschnitt unsicherer als Gesunde vergleichbaren Alters [1], [8], [3], [11], [12] fahren, sind viele dennoch in der Lage, ein Kraftfahrzeug (Kfz) ausreichend sicher zu führen [13]. Die wesentlichen Faktoren für eine eingeschränkte Fahrtauglichkeit sind bei Parkinson-Pat. ähnlich wie bei Nichterkrankten das Alter, kognitive Defizite [11], [14], [15], [16] und Sehstörungen [12], [17]. Beeinträchtigungen des Fahrverhaltens bei Parkinson-Pat. im Vergleich zu Gesunden ließen sich nur teilweise durch motorische Parameter oder die Medikation vorhersagen und waren stärker mit visuellen Wahrnehmungsleistungen, exekutiven Funktionen und Aufmerksamkeitsfunktionen (Scanning, geteilte Aufmerksamkeit) assoziiert. Kognitive Screeningverfahren erwiesen sich als ungeeignet zur Prüfung der Fahreignung und selbst die Ergebnisse einer differenzierten neuropsychologischen Testung allein ermöglichen häufig keine zufriedenstellende Vorhersagbarkeit des tatsächlichen

Fahrvermögens [18], [19]. Deshalb wird empfohlen, die neuropsychologischen Testergebnisse bevorzugt in Verbindung mit dem Ergebnis einer praktischen Fahrprobe im öffentlichen Straßenverkehr für die Beurteilung der Fahreignung zu werten. Eine Impulskontrollstörung bedarf differenzierter Beurteilung [20]. Pat. mit einer THS sind hinsichtlich ihrer Fahreignung nicht anders zu beraten als andere Parkinson-Pat. auch. Allerdings sollte bei individuellen Pat., die bereits vor der THS-Operation sehr unsicher fuhren, postoperativ die Fahreignung ggf. erneut überprüft werden [21], [22], [23]. Für die Zeit der medikamentösen Umstellung vor der THS-OP und in den ersten 3 Monaten postoperativ sollen Pat. kein Kfz führen [24], [25].

Generell wuerden Zweifel an der Fahrtauglichkeit, die durch Dritte, insbesondere Familienmitglieder, geäußert werden, die den Pat. als Fahrer aus der Vergangenheit einschätzen können, als sehr starkes Indiz für eine verminderte befunden und sollten vom behandelnden Neurologen/der behandelnden Neurologin erfragt werden [3].

Begründung der Empfehlung

Laut Begutachtungsleitlinien der Bundesanstalt für Straßenwesen [26] sind Pat. mit einer extrapyramidalen (oder zerebellären) Erkrankung, „die zu einer herabgesetzten Leistungs- und Belastungsfähigkeit führt, [ist] nicht in der Lage, den gestellten Anforderungen zum Führen von Kraftfahrzeugen der Gruppe 2 gerecht zu werden."

Die Gruppe 2 umfasst z.B. Lastkraftwagen und Busse für die gewerbliche oder geschäftsmäßige Fahrgastbeförderung. Hierbei gelten strengere Regeln als für Gruppe 1, weil die Folgen eines eventuellen Unfalls gravierender sind. Die Ausprägung der klinischen Parkinson-Symptomatik spielt daher hier keine Rolle. Ausnahmen können im Einzelfall im Rahmen eines verkehrsmedizinischen Gutachtens ausgesprochen werden.

Weiterhin führt die Begutachtungsleitlinie der Bundesanstalt für Straßenwesen [26] aus: „Die Fähigkeit, Kraftfahrzeuge der Gruppe 1 sicher zu führen, ist nur bei erfolgreicher Therapie oder in leichteren Fällen der Erkrankungen gegeben. Sie setzt die nervenärztliche/neurologische und, je nach den Umständen, psychologische Zusatzbegutachtung voraus. Nachuntersuchungen in Abständen von einem, zwei und vier Jahren sind je nach den Befunden, die der Einzelfall bietet, zur Auflage zu machen."

Die Gruppe 1 umfasst Mopeds, Kraft- und Leichtkrafträder, Kraftfahrzeuge bis 3,5 t, land- und forstwirtschaftliche Zugmaschinen. Dabei wird hervorgehoben, dass die Beurteilung nicht allein vom Ausprägungsgrad der extrapyramidal-motorischen Symptome abhängig gemacht werden darf ([26], S. 44).

Von einer fehlenden Fahrtauglichkeit für Gruppe 1 ist insbesondere auszugehen bei (nach [9], [10], [25], [27], [28]):

- schwerer motorischer Beeinträchtigung (Akinese, Tremor, Dyskinesien)
- unvorhersagbaren, ausgeprägten motorischen On/Off-Phänomenen
- deutlichen visuell-räumlichen Defiziten (Abstand, Entfernung, Bremspunkt)

- ausgeprägter Visusminderung oder (Farb-)Kontrastsehstörung
- Doppelbildern bei Geradeausblick
- Demenz
- ausgeprägten Aufmerksamkeitsstörungen (Shift/Switch)
- deutlich verminderter Belastbarkeit/Daueraufmerksamkeit
- deutlicher allgemeiner (psychomotorischer) Verlangsamung der Reaktionen
- fahrrelevanten dopaminerg-induzierten Verhaltensstörungen (Aggressivität, Rücksichtslosigkeit, erhöhtes Risikoverhalten)
- Halluzinationen.

**Empfehlung**	**Modifiziert**<br>**Stand (2023)**
▪ Mit der Diagnose einer PK ist für Inhaber/Inhaberinnen der Fahrerlaubnis für Kraftfahrzeuge der Gruppe 2 (Lkw, Bus, Taxi, Personenbeförderung) die Fahreignung in der Regel nicht gegeben.<br>▪ Die Fahrerlaubnis für Kraftfahrzeuge der Gruppe 1 (Pkw, Kraftrad, landwirtschaftliche Zugmaschine) kann bei Parkinson-Pat. nach individueller Beurteilung bei erfolgreicher Therapie oder in leichten Fällen gegeben sein.<br>▪ Bei der Fahreignungsuntersuchung von Parkinson-Pat. sollten außer der Erhebung motorischer Parameter (z.B. UPDRS III im Off) neuropsychologische Verfahren, die u. a. die Prüfung von visuell-räumlichen Aufmerksamkeitsleistungen, geteilter Aufmerksamkeit, exekutiven Funktionen und visuellen Wahrnehmungsleistungen erfassen, sowie ggf. eine Fahrverhaltensprobe durchgeführt werden.<br>▪ Von einer fehlenden Fahreignung ist auszugehen bei schwerer motorischer Beeinträchtigung und/oder unvorhersagbaren ausgeprägten motorischen On/Off-Fluktuationen sowie höhergradigen Störungen der visuellen Funktion, Kognition, Aufmerksamkeit, Psychomotorik, Impulskontrolle oder Halluzinationen.<br>▪ In den ersten 3 Monaten postoperativ nach THS-Operation sollen Pat. kein Kfz führen.	
Konsensstärke: 100%, starker Konsens	

Referenzen

1. Meindorfner C, Körner Y, Möller JC, Stiasny-Kolster K, Oertel WH, Krüger HP. Driving in Parkinson's disease: mobility, accidents, and sudden onset of sleep at the wheel. Mov Disord. 2005;20(7):832-42
2. Buhmann C, Vettorazzi E, Oehlwein C, Rikkers F, Poetter-Nerger M, Gulberti A, et al. Impact of Deep Brain Stimulation on Daily Routine Driving Practice in Patients with Parkinson's Disease. Parkinsons Dis. 2015;2015:608961
3. Cordell R, Lee HC, Granger A, Vieira B, Lee AH. Driving assessment in Parkinson's disease--a novel predictor of performance? Mov Disord. 2008;23(9):1217-22

4. Mattern R, Körner Y, Wagenpfeil T. Fahrtüchtigkeit bei chronischen Erkrankungen - Ärzte in der Pflicht. Dtsch Med Wochenschr. 2006;131(40):2200-1

5. Berghaus G, Brenner-Hartmann J. Fahreignung, Fahrsicherheit und deren Begutachtung. In: Madea B, Mußhoff F, Berghaus G, editors. Verkehrsmedizin: Fahreignung, Fahrsicherheit, Unfallrekonstruktion: Deutscher Ärzte-Verlag; 2012. p. 131-43.

6. Gaidzik P. Ärztliche Beurteilung der Kraftfahreignung. In: Widder B, Gaidzik P, editors. Begutachtungen in der Neurologie: Georg Thieme Verlag; 2011. p. 258-9.

7. Landesärztekammer Baden-Württemberg mit den Bezirksärztekammern. Merkblatt - Die Aufklärungspflichen des Arztes - Stand Januar 2013.

8. Wood JM, Worringham C, Kerr G, Mallon K, Silburn P. Quantitative assessment of driving performance in Parkinson's disease. J Neurol Neurosurg Psychiatry. 2005;76(2):176-80

9. Heikkilä VM, Turkka J, Korpelainen J, Kallanranta T, Summala H. Decreased driving ability in people with Parkinson's disease. J Neurol Neurosurg Psychiatry. 1998;64(3):325-30

10. Klimkeit EI, Bradshaw JL, Charlton J, Stolwyk R, Georgiou-Karistianis N. Driving ability in Parkinson's disease: current status of research. Neurosci Biobehav Rev. 2009;33(3):223-31

11. Uc EY, Rizzo M, Johnson AM, Dastrup E, Anderson SW, Dawson JD. Road safety in drivers with Parkinson disease. Neurology. 2009;73(24):2112-9

12. Crizzle AM, Classen S, Uc EY. Parkinson disease and driving: an evidence-based review. Neurology. 2012;79(20):2067-74

13. Gräcmann N, Albrecht M. Begutachtungs-Leitlinien zur Kraftfahrereignung. Letzte Änderung am 2. November 2009 ed. Bundesanstalt für Straßenwesen (BASt): Carl Schünemann Verlag GmbH; 2010.

14. Singh R, Pentland B, Hunter J, Provan F. Parkinson's disease and driving ability. J Neurol Neurosurg Psychiatry. 2007;78(4):363-6

15. Dubinsky RM, Gray C, Husted D, Busenbark K, Vetere-Overfield B, Wiltfong D, et al. Driving in Parkinson's disease. Neurology. 1991;41(4):517-20

16. Worringham CJ, Wood JM, Kerr GK, Silburn PA. Predictors of driving assessment outcome in Parkinson's disease. Mov Disord. 2006;21(2):230-5

17. Uc EY, Rizzo M, Anderson SW, Dastrup E, Sparks JD, Dawson JD. Driving under low-contrast visibility conditions in Parkinson disease. Neurology. 2009;73(14):1103-10

18. Amick MM, Grace J, Ott BR. Visual and cognitive predictors of driving safety in Parkinson's disease patients. Arch Clin Neuropsychol. 2007;22(8):957-67

19. Classen S, Witter DP, Lanford DN, Okun MS, Rodriguez RL, Romrell J, et al. Usefulness of screening tools for predicting driving performance in people with Parkinson's disease. Am J Occup Ther. 2011;65(5):579-88

20. Fründt O, Fadhel M, Heesen C, Seddiq Zai S, Gerloff C, Vettorazzi E, et al. Do Impulse Control Disorders Impair Car Driving Performance in Patients with Parkinson's Disease? J Parkinsons Dis. 2022;12(7):2261-75

21. Buhmann C, Maintz L, Hierling J, Vettorazzi E, Moll CK, Engel AK, et al. Effect of subthalamic nucleus deep brain stimulation on driving in Parkinson disease. Neurology. 2014;82(1):32-40

22. Fründt O, Mainka T, Vettorazzi E, Baspinar E, Schwarz C, Südmeyer M, et al. Prospective controlled study on the effects of deep brain stimulation on driving in Parkinson's disease. NPJ Parkinsons Dis. 2023;9(1):105

23. Buhmann C, Gerloff C. Autofahren bei Morbus Parkinson - Driving with Parkinson's Disease. Aktuelle Neurologie. 2013;40(06):315-20

24. Buhmann C, Oelsner H, Vesper J. Neurologische Leitlinie "Tiefe Hirnstimulation (THS) und Autofahren bei Morbus Parkinson" der Arbeitsgemeinschaft Tiefe Hirnstimulation e.V. 15-4-2017.

25. Schubert W, Schneider W, Eisenmenger W, Stephan E. Begutachtungsleitlinien zur Kraftfahrereignung - Kommentar. Überarbeitete und erweiterte 2. Auflage ed. Kirschbaum Verlag Bonn, 2005.

26. Begutachtungsleitlinien zur Kraftfahreignung vom 27. Januar 2014 (Verkehrsblatt S. 110) Fassung vom 17.02.2021 (Verkehrsblatt S. 198), in Kraft getreten am 01.06.2022 mit der Fünfzehnten Verordnung zur Änderung der Fahrerlaubnis-Verordnung und an-derer straßenverkehrsrechtlicher Vorschriften (Bundesgesetzblatt Teil I Nr. 11 vom 25. März 2022).

27. Lachenmayer L. Parkinson's disease and the ability to drive. J Neurol. 2000;247 Suppl 4:Iv/28-30

28. Buhmann C, Vesper J, Oelsner H. [Driving ability in Parkinson's disease]. Fortschr Neurol Psychiatr. 2018;86(1):43-8

3.26 Parkinson-Tagesklinik

Autoren: Carsten Buhmann, Per Odin, Jos Becktepe

Fragestellung 193: Für welche Pat. ist die Behandlung in einer Parkinson-Tagesklinik geeignet?

Hintergrund

Das Konzept der tagesklinischen Versorgung von Parkinson-Pat. ist noch relativ neu und es gibt bislang keine einheitliche Definition, welche Pat. in einer Parkinson-Tagesklinik (PTK) behandelt werden sollen.

Evidenzgrundlage

Die Literatursuche in Pubmed erbrachte 11 Studien zu den Begriffen „Parkinson disease OR Parkinson* OR Parkinsonian disorders OR Movement disorders AND day hospital OR day unit OR day patient. Ausgeschlossen wurden die Suchbegriffe „Wolff parkinson white syndrome OR wolff AND parkinson* OR wpw AND syndrome OR wpw“. Inkludiert wurden nur Studien an/mit Menschen („AND humans NOT animals“) im Zeitraum von 2016 bis 2021 in englischer und deutscher Sprache. Nach Screening der Abstracts und Inhalte wurde 1 Publikation als relevant identifiziert. Nach Erweiterung der Recherche um den Suchbegriff „day clinic“ und Extraktion von Publikationen aus den Literaturreferenzen wurden letztendlich 9 Publikationen selektiert, welche in diese Leitlinie einbezogen wurden. Es fanden sich keine randomisierten, kontrollierten Studien in dieser Literaturrecherche, aber eine kontrollierte und zwei longitudinale größere Kohortenstudien.

Ergebnis

Ein multidisziplinärer therapeutischer Ansatz ist für Parkinson-Pat. empfehlenswert [1], [2]. In einer Parkinson-Tagesklinik (PTK) sollten komplexe Parkinson-Pat. behandelt werden, deren klinische Versorgung im ambulanten Bereich personell, zeitlich oder fachlich zu aufwendig oder komplex ist, für die aber aus verschiedenen Gründen eine stationäre Behandlung nicht sinnvoll, möglich oder gewünscht ist [3], [4], [5]. Es handelt sich also um Pat., die aufgrund eines erhöhten Bedarfs an differenzierten und/oder umfangreichen Behandlungsstrategien an der Grenze zwischen stationärer und ambulanter Versorgung stehen.

Für diese Pat. besteht sowohl national als auch international eine Versorgungslücke [6], [7], [8]. Die suffiziente ambulante Versorgung komplexer Parkinson-Pat. ist personell und zeitlich zunehmend schwer umsetzbar, was zu vermehrten unangemessenen Krankenhauseinweisungen führt. Von 2010 bis 2015 hat die Zahl der stationären Parkinson-Fälle in Deutschland stark zugenommen und betrifft vor allem Pat. mit schweren motorischen Komplikationen und birgt die Gefahr einer Überlastung der Behandlungsinfrastruktur [9].

Nachfolgend sind Kriterien gelistet, die bei Vorliegen allein oder in Kombination eine Behandlung in einer PTK als empfehlenswert erscheinen lassen [3], [4], [5].

Geeignete Pat. für die Behandlung in einer PTK

- Pat. mit relevanten motorischen und/oder nicht motorischen Symptomen

- Pat. mit komplexen Medikationsschemata und/oder umfangreichen Therapieregimen
- Pat. mit invasiven Therapiemaßnahmen wie Medikamentenpumpen (Apomorphin, Levodopa/Carbidopa intestinales Gel) oder einer Tiefen Hirnstimulation (THS)
- Pat., bei denen Effekte der Behandlungsanpassung über einen längeren Zeitraum in größeren Abständen als stationär, aber engmaschiger als ambulant möglich evaluiert werden sollten
- Pat. zur Abklärung der Indikation für eine invasive Therapie (Apomorphin- oder Levodopa-Pumpe, THS)
- Pat. zu Intervallkontrollen nach THS-Implantation
- Pat. mit kognitiven Defiziten, die bei vorherigen stationären Aufenthalten oder auswärtigen Übernachtungen im Alltag psychotische oder delirante Symptome gezeigt haben
- Pat., die aus privaten Gründen keinen stationären Aufenthalt wahrnehmen können (beruflich, Pflege von Angehörigen, Versorgung von Tieren etc.)
- Pat. mit ätiologisch unklarer Parkinson-Diagnose

Therapeutische Maßnahmen, die eine Behandlung in der PTK sinnvoll machen

- relevante Änderungen oder Umstellungen komplexer Medikationsschemata
- Dosisanpassungen bei Pumpentherapien
- Neuanlage einer Apomorphin-Pumpe
- Einstellung auf einen Apomorphin-Pen
- Kontakttestung, Neuprogrammierung, Umprogrammierung bei Tiefer Hirnstimulation (THS)
- symptomorientierte aktivierende Therapien (Physiotherapie, Ergotherapie, Logopädie)
- Erstellung eines individuell angepassten mündlichen und schriftlichen Therapiekonzepts für ambulante aktivierende Therapien

Diagnostische Maßnahmen, die eine Behandlung in der PTK sinnvoll machen

- standardisierter Levodopa Test
- Apomorphin-Test
- umfangreiche neuropsychologische Testung
- Schluckdiagnostik (FEES)
- Abklärung der Indikation für aktivierende Therapien (Physiotherapie, Ergotherapie, Logopädie) und/oder Überprüfung der Zielrichtung der bisherigen ambulanten Therapieverfahren.

Als nicht geeignete Pat. für die Behandlung in der PTK wurden identifiziert:

- leicht betroffene Parkinson-Pat., die ambulant suffizient zu behandeln sind

- Parkinson-Pat., die einer stationären Behandlung bedürfen
  - schwer betroffene und/oder multimorbide Pat.
  - immobile Pat.
  - Pat. mit ausgeprägter Demenz
  - Pat. mit unzumutbar weitem Anreiseweg
  - Pat. mit ausgeprägter psychotischer Symptomatik.

Begründung der Empfehlung

Die Empfehlung basiert auf der oben dargestellten Evidenzgrundlage der identifizierten Versorgungslücke für bestimmte Pat. sowie therapeutischen und/oder diagnostischen Maßnahmen. Die Einweisung in eine PTK sollte immer fachärztlich neurologisch erfolgen, um sicherzustellen, dass Pat. aufgenommen werden, für die die individuelle Versorgungssituation nicht ausreichend ist, unter Berücksichtigung der lokalen ambulanten und stationären Besonderheiten (z.B. fachärztliche Kapazitäten, Vorliegen von Parkinson-Schwerpunktpraxen, Zugang zu Hochschulambulanzen, stationäre Versorgungslandschaft). Die Empfehlung ist mehr eine „kann“- als eine „soll“-Empfehlung, da nicht in allen Regionen eine PTK in akzeptabler Entfernung liegt und dann u.g. Pat.-Gruppen ggf. stationär aufgenommen werden müssen.

**Empfehlung: Welche Pat. können/sollten in einer Parkinson-Tagesklinik (PTK) behandelt werden?**	**Neu**<br><br>**Stand (2023)**
**Folgende Pat.-Gruppen können in der PTK behandelt werden:**<br>▪ In einer PTK können Pat. mit unklaren Parkinson-Syndromen zur komplexen Diagnostik und Parkinson-Pat. für komplexe medikamentöse Therapieanpassungen, Voruntersuchungen, Beginn und Nachuntersuchungen von invasiven Verfahren wie Pen- oder Pumpentherapien oder der Tiefen Hirnstimulation aufgenommen werden, die ambulant nicht umgesetzt werden können.<br>**Zusätzlich kann eine Behandlung in einer PTK dann sinnvollerweise angeboten werden, wenn**<br>▪ Effekte der Behandlungsanpassung über einen längeren Zeitraum in größeren Abständen als stationär, aber engmaschiger als ambulant möglich evaluiert werden sollten<br>▪ prinzipiell eine stationäre Behandlung sinnvoll erscheint, diese aber aus individuellen Gründen nicht ratsam (z.B. Gefahr des nächtlichen Delirs bei kognitiv eingeschränkten Pat.) oder möglich (z.B. beruflich, Pflege von Angehörigen) ist.	
Konsensstärke: 100%, starker Konsens	

Fragestellung 194: Worin unterscheidet sich die Behandlung in einer Tagesklinik von der ambulanten oder stationären Behandlung?

Hintergrund

Generell ist die Behandlung von Parkinson-Pat. im ambulanten, tagesklinischen und stationären Setting nicht standardisiert und die zu erbringenden Leistungen und Kompetenzen der Anbieter sind nicht definiert. Entsprechend bestehen in der Versorgungsstruktur erhebliche geografische Unterschiede.

Ambulant werden an Universitätskliniken Parkinson-Pat. zum Teil in spezialisierten poliklinischen Sprechstunden oder Medizinischen Versorgungszentren versorgt, im niedergelassenen Bereich gibt es in bestimmten Regionen Parkinson-Schwerpunktpraxen. Während die Vergütung in den universitären Polikliniken über eine Hochschulpauschale geregelt ist, erfolgt die Vergütung der ärztlichen Leistung im niedergelassenen Bereich nach dem EBM- oder GOÄ-Tarif. Ein großer Teil der Pat. wird von niedergelassenen Neurologen/Neurologinnen oder Nervenärzten/Nervenärztinnen sowie Hausärzten/Hausärztinnen versorgt. Genaue Zahlen über den Anteil der einzelnen Versorger an der Betreuung der Pat. liegen nicht vor.

Tagesklinische Strukturen gewinnen zunehmend an Bedeutung und finden sich bislang überwiegend an Universitätskliniken, aber auch an Kliniken, die eine spezielle Expertise im Bereich Parkinson aufweisen. Die Abrechnung erfolgt über einen speziellen Tagessatz, der mit den Krankenkassen auszuhandeln ist.

Stationär werden Parkinson-Pat. akut-medizinisch in der Regel ortsnah im zuständigen Krankenhaus versorgt, was nach dem DRG-System vergütetwird. Bei besonderen diagnostischen oder therapeutischen Fragen erfolgt die Behandlung i.d.R. in einer stationären Einrichtung mit besonderer Parkinson-Expertise.

Die Deutsche Parkinson Vereinigung (dPV) hat zur Qualitätsverbesserung und Qualitätssicherung mit Anbietern von ambulanten, teilstationären und stationären Versorgern, die als Behandlungsschwerpunkt die Indikation „Morbus Parkinson“ bzw. „Bewegungsstörungen“ angeben, eine Kriterienliste erstellt, die Merkmale zur Strukturqualität und die Grundlage einer Anerkennung und Zertifizierung durch einen externen Zertifizierer als spezialisierte Parkinson-Einrichtungen definiert. Dies gilt für stationäre Akutkliniken und Reha-Einrichtungen, die dann als zertifizierte Parkinson-Fachkliniken ein spezielles diagnostisches und therapeutisches Angebot für Parkinson-Pat. und artverwandte Krankheiten vorhalten. Entsprechende Zertifizierungen und Kriterien gibt es ebenso für Parkinson-Tageskliniken und ambulante Parkinson-Schwerpunktpraxen. Die Zertifikate werden jeweils für 3 Jahre von der dPV erteilt und können auf Antrag der jeweiligen Einrichtung und erneuter Überprüfung für 3 Jahre verlängert werden. Die Zertifizierung kann im Wege der Überprüfung und Zertifizierung nach KTQ oder DIN EN ISO 9001: 2000 geprüft werden.

Zusätzlich besteht für Kliniken die Möglichkeit, den stationären Operationen- und Prozedurenschlüssel (OPS) 8-97d „Multimodale Komplexbehandlung bei Morbus Parkinson und

atypischem Parkinson-Syndrom“ zu erlangen (Strukturprüfung) und eine entsprechende stationäre Komplexpauschale abzurechnen. Es gibt jedoch keine allgemeingültigen Definitionen, welche Pat. innerhalb des komplexen Programms behandelt werden sollten und welche nicht [10]. Für das tagesklinische Setting gibt es bislang keine vergleichbare Möglichkeit einer Vergütung via Komplexpauschale.

Evidenzgrundlage

Die Literatursuche in Pubmed erbrachte 521 Publikationen zu den Begriffen „day hospital OR day unit OR day hospital OR day patient AND ambulant OR outpatient OR Ambulatory Care OR Ambulatory Care OR Ambulatory OR in-patient OR stationary AND distinguish OR different* OR versus”. Inkludiert wurden nur Studien an/mit Menschen („AND humans NOT animals“) im Zeitraum von 2016 bis 2021 in englischer und deutscher Sprache. Nach Screening der Abstracts und Inhalte wurde keine Publikation als relevant identifiziert. Nach Erweiterung der Recherche um den Suchbegriff „day clinic“, Extraktion von Publikationen aus den Literaturreferenzen anderer Recherchen zum Thema Versorgungsstrukturen bei Parkinson und empirischer Suche wurden letztendlich 20 Publikationen selektiert, welche in diese Leitlinie einbezogen wurden. Es fanden sich keine Vergleichsstudien. Als wesentliche Datenbasis wurden die Zertifizierungskriterien für Parkinson-Fachkliniken [https://www.parkinson-vereinigung.de/diverse-inhalte/fachkliniken/zertifizieungskriterien.html], Parkinson-Tageskliniken [https://www.parkinson-vereinigung.de/files/Kliniken%20Parkinson/Checkliste%20Parkinson%20TK_Audithilfe_cb_cf.pdf] und ambulante Parkinson-Schwerpunktpraxen [https://www.parkinson-vereinigung.de/files/Kliniken%20Parkinson/Checkliste%20Parkinson%20SP_Audithilfe.pdf] herangezogen.

Ergebnis

Es findet sich in der Literatur ein Review mit einer Übersicht über verschiedenen Versorgungsmodelle eines gemeindenetzwerkbasierten Modells, eines Tagesklinikmodells, eines akademischen klinikbasierten Modells und eines intensivierten stationären Rehabilitationsmodells [1]. Die Autoren schlussfolgern, dass eine multidisziplinäre Versorgung für die Behandlung der PK unerlässlich ist, jedoch eine breite Umsetzung aus einer Reihe von Gründen begrenzt ist. Als Expertenmeinung wird eine teambasierte Versorgung vorgeschlagen, die dezentralisiert und vertikal und horizontal über Gesundheitssysteme hinweg integriert ist. Der Aufbau einer Evidenzbasis für diese komplexen Interventionen erfordert andere Bewertungsmodelle als randomisierte kontrollierte Studien. Die Behandlung in der PTK sollte die Behandlungsvorteile von stationärer und ambulanter Einrichtung verbinden [9]. In der PTK findet eine Behandlung in einem multiprofessionellen Team statt, in dem die Parkinson-Nurse eine Schlüsselrolle innehat [3], [11].

Unterschiede zur ambulanten Behandlung

- bessere Option der Durchführung einer patientenzentrierten Therapie unter Berücksichtigung des individuellen motorischen und nicht motorischen Subtyps der Parkinson-Erkrankung [12], [13], [14].
- mehr zeitliche, personelle, apparative Kapazitäten

- spezielle Fachexpertise im Vergleich zu Nicht-Parkinson-Schwerpunktpraxen
- multiprofessionelles Team vor Ort
- Möglichkeit, die Pat. über mehrere Stunden am Tag zu beobachten und somit therapeutische Veränderungen unmittelbar zu bewerten, motorische und nicht motorische Fluktuationen und Dyskinesien im Tagesverlauf zu erkennen und die oftmals unsicheren anamnestischen Angaben und Wahrnehmungen [15] zu objektivieren
- Möglichkeit, demn Pat. verschiedene therapeutische Optionen vorzustellen, die nachweislich die motorischen und nicht motorischen Funktionen verbessern [16], [17], [18], [19], und ihn zu motivieren, diese im ambulanten Setting zu nutzen (z.B. Stressbewältigung, autogenes Training, Yoga, Tai-Chi, LSVT® BIG, Musiktherapie)
- Edukative Maßnahmen für Pat. und Angehörige können mehrfach durchgeführt werden, was häufig notwendig ist, um einen erzielten Therapieerfolg zu erreichen und zu halten [20].
- Adressierung der häufig vorhandenen psychosozialen und/oder soziomedizinischen Probleme [21], die im ambulanten Bereich oft und vornehmlich aus Zeitgründen nicht adäquat besprochen und geklärt werden können [6], [22].
- Vergütung über mit den Krankenkassen zu verhandelndem Tagessatz in der PTK statt über Selbstzahler/PKV/GKV oder Hochschulambulanzpauschale ambulant.

Unterschiede zur stationären Behandlung

- Für die PKB sind Strukturmerkmale definiert [https://www.dimdi.de/static/de/klassifikationen/ops/kode-suche/opshtml2019/block-8-97...8-98.htm]. Die Kriterien sind primär auf quantitative Aspekte (Therapiehäufigkeit und Therapiedauer) und weniger auf qualitative Aspekte (allgemeine neurologische und nicht parkinsonspezifische Fachexpertise der Leistungserbringer, apparative Ausstattung) ausgerichtet.
- Therapieanpassung findet unter Alltagsbedingungen statt in der stationären Umgebung mit nicht alltagsadäquaten Belastungen und Tagesrhythmen.
- individuell (Pat.- und/oder Einrichtungsbezogen) gewählte Abstände zwischen den Behandlungstagen
- insgesamt prospektiv längere Beobachtungszeit der Pat. (ggf. über Wochen)
- Therapieeinstellung unter Alltagsbedingungen, da die Pat. in den Tagen zwischen den Aufenthalten in der PTK zu Hause und ihren üblichen täglichen Belastungen und Stressfaktoren ausgesetzt sind (Beruf, Sozialleben, üblicher Tag-Nacht-Rhythmus sowie Zeiten für Mahlzeiten und Medikamenteneinnahmen).
- Keine Begrenzung der Behandlung auf eine mittlere Verweildauer der Parkinson-Diagnose-bezogenen Fallgruppierung (Diagnosis Related Groups; DRG)
- Keine nächtlich veränderte Umgebung, die bei stationären Aufenthalten insbesondere bei Demenz, höherem Erkrankungsalter sowie höherem Krankheitsschweregrad zu deliranten Symptomen mit dem Risiko einer erhöhten stationären Mortalität führen kann [23]

- Vergütung über mit den Krankenkassen zu verhandelndem Tagessatz in der PTK statt über diagnosebezogene Fallgruppierung (Diagnosis Related Groups; DRG)
- Die Behandlung in einer Tagesklinik ist im Gegensatz zur stationären Behandlung begrenzt auf Pat., die in zumutbarer räumlicher bzw. zeitlicher Entfernung zur Tagesklinik ihren Wohnort bzw. ihre Unterkunft haben. Das limitiert die Anzahl an potenziellen geeigneten Pat. erheblich. Es zeigte sich, dass die große Mehrheit von 55.141 evaluierten Pat. in der stationären PKB ihren Wohnbezirk für einen stationären Aufenthalt verlassen musste [10]. Dies deutet darauf hin, dass viele Parkinson-Pat., für die ein multimodales Therapieprogramm sinnvoll erscheint, weite Strecken zurücklegen müssen, um eine spezialisierte Therapie zu erhalten, und deswegen für eine Tagesklinik in einem spezialisierten Parkinsonzentrum eher nicht infrage kommen.

Tab. 24: Unterschiede verschiedener Parkinson-Versorgungskonzepte

	Parkinson-Tagesklinik (PTK)[1]	Parkinson-Komplexbehandlung (PKB)[2]	Parkinson-Schwerpunktpraxis
Indikation	Parkinson-Krankheit und atypische Parkinson-Syndrome		
Setting	tagesklinisch, teilstationär ohne Übernachtung	vollstationär mit Übernachtung	ambulant
Zielgruppe	komplexe Pat. mit der Notwendigkeit zur intensivierten Therapieanpassung unter Alltagsbelastung, die in eine Versorgungslücke zwischen ambulanter und stationärer Behandlung fallen	Pat. mit subakuten, krisenhaften Zuständen oder progredienter klinischer Verschlechterung unter ambulanten Bedingungen	Pat. im frühen und späteren Krankheitsstadium, die mit besonderer Fachexpertise kontinuierlich im Krankheitsverlauf betreut werden sollten oder müssen
Anreise	selbstständig oder mit Hilfe<br>eher wohnortnah	selbstständig oder mit Hilfe<br>eher unabhängig vom Wohnort	selbstständig oder mit Hilfe<br>eher wohnortnah
Aufenthalt	intermittierend, z.B. an 5 Tagen innerhalb von 3 Wochen, ggf. weitere Tage	14–21 Tage je nach Klinik, ggf. Verlängerung um eine Woche	in regelmäßigen Abständen, oft quartalsweise
Vergütung	mit Kassen individuell verhandelter Tagessatz	DRG oder OPS 8-97d für PKB nach erfolgreicher Strukturprüfung durch den MD (https://www.dimdi.de/static/de/klassifikationen/ops/kode-suche/opshtml2020/block-8-97...8-98.htm)	EBM oder GOÄ

	Parkinson-Tagesklinik (PTK)[1]	Parkinson-Komplexbehandlung (PKB)[2]	Parkinson-Schwerpunktpraxis
Konzept	multimodal, interdisziplinär	multimodal, interdisziplinär	kooperativ und vernetzt
Ausrichtung	Therapieoptimierung mit Fokus auf Anpassung komplexer medikamentöser Therapieregime und/oder Einstellungen von Medikamentenpumpen oder der Tiefen Hirnstimulation	Therapieoptimierung mit Fokus auf Training	Therapieoptimierung mit Fokus auf kontinuierlicher Versorgung, einschließlich Pat. mit invasiven Therapien (THS, Pumpen)
Einbezug nicht ärztlichen Personals	ja (mehrere Bereiche aus Logopädie, Ergotherapie, Physiotherapie, Sozialdienst, Ernährungsberatung, Künstlerische Therapien)	ja (mindestens 3 Bereiche aus Sporttherapie, Logopädie, Psychotherapie und Künstlerische Therapien)	Vorhalten einer Parkinson-Assistenz-Kraft (PASS) verpflichtend
Therapiezeit pro Woche	individueller Stundenplan, mindestens 5 Stunden/Tag, davon mindestens 1 ärztlicher Kontakt im Einzelsetting (mind. 30 min)	mindestens 7½ Stunden, davon mindestens 5 Stunden in Einzeltherapie	nicht festgelegt
Teambesprechung	täglich zwischen Arzt und PD-Nurse, wöchentlich im gesamten Team	wöchentlich	nicht festgelegt
Dokumentation	täglich (Behandlungsziele und -ergebnisse)	wöchentlich (Behandlungsziele und -ergebnisse)	nicht festgelegt
Parkinson-Nurse	verpflichtend	wünschenswert	nicht festgelegt
Zertifizierungs-kriterien	primär qualitativ (https://www.parkinson-vereinigung.de/files/Kliniken%20Parkinson/Checkliste%20Parkinson%20TK_Audithilfe_cb_cf.pdf)	primär quantitativ (https://www.parkinson-vereinigung.de/diverse-inhalte/fachkliniken/zertifizierungskriterien.html)	quantitativ und qualitativ (https://www.parkinson-vereinigung.de/files/Kliniken%20Parkinson/Checkliste%20Parkinson%20SP_Audithilfe.pdf)
Evidenz der Wirksamkeit	Evidenzgrad 2<br>1) 16-Monats-Outcome [4]	nicht vorhanden	nicht vorhanden

	Parkinson-Tagesklinik (PTK)[1]	Parkinson-Komplexbehandlung (PKB)[2]	Parkinson-Schwerpunktpraxis
	2) 32-Monats-Outcome [5]		

[1]Hamburger Modell [4], [5]

[2]Informationen in Anlehnung an [24].

Begründung der Empfehlung

Auf der Basis der oben dargestellten Evidenzgrundlage sind oben detailliert Kriterien dargestellt, anhand welcher sich Behandlungen in der Parkinson-Tagesklinik (PTK) nach dem Hamburger Modell von einer stationären Parkinson-Komplexbehandlung (PKB) oder einer ambulanten Behandlung in einer Parkinson-Schwerpunktpraxis unterscheiden.

Auf der Basis der oben dargestellten Evidenzgrundlage und unter Berücksichtigung der lokal unterschiedlichen therapeutischen Landschaft sind nachfolgend objektive und subjektive empfohlene Kriterien genannt, anhand welcher sich Behandlungen in einer Parkinson-Tagesklinik (PTK) von einer stationären Parkinson-Komplexbehandlung (PKB) und einer ambulanten Behandlung in einer neurologischen (auch Parkinson-Schwerpunktpraxis) unterscheiden.

Empfehlung: Worin unterscheidet sich die Behandlung in einer Parkinson-Tagesklinik (PTK) von der ambulanten oder stationären Behandlung?	Neu<br><br>Stand (2023)
**Objektiv**<br>▪ teilstationäre Behandlung, intermittierend häufige, in Bezug auf Zeitintervall, Häufigkeit und Frequenz flexibel determinierbare multidisziplinäre Behandlung<br>▪ Vorhandensein klinischer Kurzzeit-Outcome-Daten<br>▪ In einer PTK kann mit den Krankenkassen eine Abrechnung als Tagessatz entsprechend einem individuellen, vom Behandlungszentrum festgelegten Behandlungskonzept erfolgen.<br>**Subjektiv**<br>▪ therapeutisch komplexe, noch ausreichend mobile Pat. mit der Notwendigkeit zur intensivierten und kontrollierten Therapieanpassung unter Alltagsbelastungen<br>▪ therapeutischer Fokus auf Anpassung komplexer medikamentöser Therapieregime und/oder Einstellungen von Medikamentenpumpen oder der Tiefen Hirnstimulation<br>▪ Im Gegensatz zur stationären Behandlung soll in der PTK eine intensivierte Therapieanpassung unter Alltagsbelastungen erfolgen, im Gegensatz zur ambulanten Behandlung soll ein Monitoring von komplexen oralen Therapieschemata, Pumpentherapie oder nach THS durchgeführt werden.	
Konsensstärke: 100%, starker Konsens	

Fragestellung 195: Welche personellen und apparativen Mindestvoraussetzungen sollte eine Tagesklinik haben?

Empfehlung: Welche personellen und apparativen Mindestvoraussetzungen sollte eine Parkinson-Tagesklinik (PTK) haben?	Neu<br><br>Stand (2023)
Es wird konsentiert, dass die Beantwortung der Frage die Zielsetzung dieser Leitlinie überschreiten würde.	
Konsensstärke: 83%	

Fragestellung 196: Was sind der optimale Behandlungszeitraum und die optimale Behandlungsintensität in einer Tagesklinik?

Hintergrund

Behandlungskonzepte in den bislang noch wenigen Parkinson-Tageskliniken (PTK) in Deutschland variieren von einer täglichen Behandlung über einen kurzen Zeitraum bis zu Behandlungstagen in größeren Abständen über längere Zeiträume sowohl zwischen den einzelnen Einrichtungen als auch patientenbezogen bedarfsgerecht innerhalb der Einrichtungen. Generell bietet das Konzept einer PTK mit festgelegten Tagessätzen die Möglichkeit, Behandlungszeiträume und Behandlungsintensitäten individuell auf die Pat. auszurichten und zu gestalten.

Evidenzgrundlage

Die Literatursuche in Pubmed erbrachte 11 Publikationen zu den Begriffen „day hospital OR day unit OR day patient OR day clinic AND period OR intensity OR frequency OR Time Factors OR duration AND optimal". Inkludiert wurden nur Studien an/mit Menschen („AND humans NOT animals") im Zeitraum von 2016 bis 2021 in englischer und deutscher Sprache. Nach Screening der Abstracts und Inhalte wurde keine Publikation als relevant identifiziert. Nach Erweiterung der Recherche um den Suchbegriff „day clinic", Extraktion von Publikationen aus den Literaturreferenzen anderer Recherchen zum Thema Tagesklinik und empirischer Suche wurden letztendlich 2 Studien selektiert, welche in diese Leitlinie einbezogen wurden.

Ergebnis

Bislang liegen keine Vergleichsstudien zu unterschiedlichen Behandlungszeiträumen und Behandlungsintensitäten in einer Parkinson-Tagesklinik (PTK) vor. Die Autoren schlagen als ein empfehlenswertes Konzept das Hamburger Modell mit einem Behandlungszeitraum über 3 Wochen und einer Behandlungsintensität mit Therapie an 5 Tagen (1. Woche: Montag und Donnerstag, 2. Woche: Mittwoch, 3. Woche: Dienstag und Freitag) vor, da für eine Behandlung nach diesem Konzept objektive und subjektive Therapieerfolge evaluiert wurden [4], [5]. Eine patinetenindividuelle Abweichung von diesem Konzept in der Behandlungsdauer und -intensität erscheint in bestimmten Fällen sowohl Einrichtungs- als auch patientenbezogen ebenfalls sinnvoll.

Begründung der Empfehlung

Auf der Basis der oben dargestellten Evidenzgrundlage erfolgen nachfolgende Empfehlungen zum optimalen Behandlungszeitraum und der optimalen Behandlungsintensität in einer Tagesklinik.

Empfehlung: Was sind der optimale Behandlungszeitraum und die optimale Behandlungsintensität in einer Parkinson-Tagesklinik (PTK)?	Neu<br><br>Stand (2023)
▪ Der Behandlungszeitraum und die Behandlungsintensität in einer PTK sollten mindestens über 3 Wochen verlaufen, mit einer Behandlungsintensität von insgesamt 5 Tagen und einem jeweiligen Abstand von 3–6 Tagen.<br>▪ Behandlungsdauer und -intensität können individuell modifiziert werden, falls erforderlich.	
Konsensstärke: 100%, starker Konsens	

Fragestellung 197: Wie effektiv ist die Therapie in einer Tagesklinik im Vergleich mit der ambulanten Standardbehandlung in der Behandlung der PK?

Hintergrund

Medizinisch und für die Kostenträger ist es sinnvoll zu wissen, ob das recht neue Konzept einer Parkinson-Tagesklinik einer ambulanten Standardbehandlung hinsichtlich des Therapieerfolgs (klinisches Outcome) überlegen ist.

Evidenzgrundlage

Die Literatursuche in Pubmed erbrachte 4 Publikationen zu den Begriffen „Parkinson disease OR Parkinson* OR Parkinsonian disorders OR Movement disorders" AND day hospital OR day unit OR day hospital OR day patient) AND standard treatment OR standard therapy OR standard treatment OR standard therapy OR ambulant OR outpatient OR Ambulatory Care OR Ambulatory". Inkludiert wurden nur Studien an/mit Menschen („AND humans NOT animals") im Zeitraum von 2016 bis 2021 in englischer und deutscher Sprache. Nach Screening der Abstracts und Inhalte wurde keine Publikation als relevant identifiziert. Nach Erweiterung der Recherche um den Suchbegriff „day clinic", Extraktion von Publikationen aus den Literaturreferenzen anderer Recherchen zum Thema Tagesklinik und empirischer Suche wurden letztendlich 4 Publikationen selektiert, welche in diese Leitlinie einbezogen wurden. Es liegt keine Vergleichsstudie vor.

Ergebnis

Es liegen bislang keine publizierten Vergleichsstudien bezüglich der Effektivität eines definierten Tageskliniksettings für Parkinson-Pat. im Vergleich zu einer definierten ambulanten Standardbehandlung vor. Ein Review aus dem Jahr 2008 wies darauf hin, dass generell eine tagesklinische Versorgung älterer Menschen effektiver zu sein scheint als keine Intervention, möglicherweise aber keinen klaren Vorteil gegenüber anderen Formen umfassender altersmedizinischer Dienste zeigt [25].

Eine randomisierte, kontrollierte Crossover-Studie verglich Lebensqualität, Angst/Depression und Motorik (Timed up and go-Test) von Parkinson-Pat., die vier Monate zuvor eine tagesklinische individuelle (nicht systematische) rehabilitative Behandlung über 6 Wochen einmal die Woche erhalten hatten, mit einer Gruppe von Pat. ohne Intervention (ambulantes Umfeld, keine standardisierte Therapie). Generell zeigten sich bei auswertbaren 94 Pat. nach den insgesamt ca. 6 Monaten Behinderung, Lebensqualität und Belastung der Pflegekräfte in beiden Gruppen verschlechtert, aber es ergaben sich Hinweise, dass o.g. tagesklinische Intervention die Mobilität verbessern könnte ($p=0,093$), während sie für die allgemeine und psychische Gesundheit ($p = 0,002$, $p = 0,019$) und tendenziell für die Belastung der Betreuer eher Nachteile mit sich brachte ($p = 0,086$).

Die Effektivität der tagesklinischen Behandlung nach dem zertifizierten multidisziplinären Hamburger Modell wurde in zwei prospektiven, unkontrollierten Studien an 184 bzw. 309 Pat. untersucht. Es zeigte sich eine signifikante objektive Score-basierte Verbesserung der Pat. nach 5 Behandlungstagen innerhalb von 3 Wochen bei Entlassung versus Aufnahme in die PTK für die MDS-UPDRS-III-Skala und alle untersuchten nicht motorischen Scores (BDI-II, MoCA, PDNMS, PDSS-2, KPPS, QUIP, PDQ-39; $p < 0,05$) und somit eine Verbesserung von Motorik, Kognition, Schlaf, Schmerzen, Impulskontrollstörungen und Lebensqualität. Subjektiv bewerteten die Pat. den Aufenthalt mit einer Gesamtschulnote von 1,69 bzw. 1,5 [4], [5]. Eine Kontrollgruppe mit einer ambulanten oder stationären Standardbehandlung bzw. fehlenden oder anderen Intervention gab es nicht. Allerdings waren alle Pat. vor Aufnahme in die PTK fachärztlich neurologisch (und überwiegend von Ärztinnen/Ärzten mit besonderer Parkinson-Expertise) mit einer ambulanten Standardtherapie behandelt worden. Somit ergeben sich Hinweise, dass eine Behandlung in der PTK nach o.g. Modell einer Standardbehandlung mindestens hinsichtlich eines kurzzeitigen Outcomes überlegen ist.

Eine kontrollierte Vergleichsstudie mit einem definierten ambulanten Therapiesetting ist anzustreben.

Begründung der Empfehlung

Auf der Basis der oben dargestellten Evidenzgrundlage erfolgen nachfolgende Empfehlungen, wie effektiv die Therapie in einer Tagesklinik im Vergleich mit der ambulanten Standardbehandlung in der Behandlung der PK ist.

**Empfehlung: Wie effektiv ist die Therapie in einer Parkinson-Tagesklinik im Vergleich mit der ambulanten Standardbehandlung in der Behandlung der PK?**	**Neu**<br><br>**Stand (2023)**
Aufgrund fehlender Evidenz kann diese Frage nicht abschließend beantwortet werden. Es ergeben sich jedoch Hinweise, dass eine Behandlung in der PTK nach o.g. Modell einer ambulanten Standardbehandlung mindestens hinsichtlich eines kurzzeitigen Outcomes überlegen ist.	
Konsensstärke: 96,9%, starker Konsens	

Fragestellung 198: Wie effektiv ist die Therapie in einer Tagesklinik im Vergleich mit der stationären Standardbehandlung in der Behandlung der PK?

Hintergrund

Medizinisch und für die Kostenträger ist es sinnvoll zu wissen, ob das recht neue Konzept einer Parkinson-Tagesklinik einer ambulanten Standardbehandlung hinsichtlich der Therapieerfolgs (klinisches Outcome) überlegen ist.

Evidenzgrundlage

Die Literatursuche in Pubmed erbrachte 3 Publikationen zu den Begriffen „Parkinson disease OR Parkinson* OR Parkinsonian disorders OR Movement disorders AND day hospital OR day unit OR day hospital OR day patient AND in-patient OR inpatient care OR stationary".

Inkludiert wurden nur Studien an/mit Menschen („AND humans NOT animals") im Zeitraum von 2016 bis 2021 in englischer und deutscher Sprache. Nach Screening der Abstracts und Inhalte wurde keine Publikation als relevant identifiziert. Nach Erweiterung der Recherche um den Suchbegriff „day clinic", Extraktion von Publikationen aus den Literaturreferenzen anderer Recherchen zum Thema Tagesklinik und empirischer Suche wurden letztendlich 4 Publikationen selektiert, welche in diese Leitlinie einbezogen wurden. Es liegt keine Vergleichsstudie vor.

Ergebnis

Sowohl für die Parkinson-Komplexbehandlung [26], [27] als auch für das therapeutische Konzept der Tagesklinik nach dem Hamburger Modell [23], [25] konnte gezeigt werden, dass bei Beendigung der Therapie im Vergleich zum Beginn motorische und nicht motorische Symptome verbessert werden konnten. Es liegen jedoch bislang keine Daten vor, die die Effektivität der Behandlung in einem Tageskliniksetting mit der in einem stationären Setting, einschließlich einer spezialisierten stationären Parkinson-Komplexbehandlung (PKB), vergleichen. Hier sind kontrollierte Vergleichsstudien anzustreben.

Begründung der Empfehlung

Auf der Basis der oben dargestellten Evidenzgrundlage erfolgen nachfolgende Empfehlungen, wie effektiv die Therapie in einer Tagesklinik im Vergleich mit der stationären Standardbehandlung in der Behandlung der PK ist.

**Empfehlung: Wie effektiv ist die Therapie in einer Parkinson-Tagesklinik im Vergleich mit der stationären Standardbehandlung in der Behandlung der PK?**	**Neu**<br><br>**Stand (2023)**
Aufgrund fehlender Evidenz kann diese Frage nicht abschließend beantwortet werden.	
Konsensstärke: 100%, starker Konsens	

Referenzen

1. Lidstone SC, Bayley M, Lang AE. The evidence for multidisciplinary care in Parkinson's disease. Expert Rev Neurother. 2020;20(6):539-49
2. Wade DT, Gage H, Owen C, Trend P, Grossmith C, Kaye J. Multidisciplinary rehabilitation for people with Parkinson's disease: a randomised controlled study. J Neurol Neurosurg Psychiatry. 2003;74(2):158-62
3. Prell T, Siebecker F, Lorrain M, Eggers C, Lorenzl S, Klucken J, et al. Recommendations for Standards of Network Care for Patients with Parkinson's Disease in Germany. J Clin Med. 2020;9(5)
4. Fründt O, Mainka T, Schönwald B, Müller B, Dicusar P, Gerloff C, Buhmann C. The Hamburg Parkinson day-clinic: a new treatment concept at the border of in- and outpatient care. J Neural Transm (Vienna). 2018;125(10):1461-72
5. Fründt O, Veliqi E, Schönwald B, Sychla P, Gerloff C, Buhmann C. [The Hamburg Parkinson day-clinic: a new treatment concept at the border of in- and outpatient care]. Fortschr Neurol Psychiatr. 2020;88(6):362-73
6. Buhmann C, Bass H, Hahne M, Jost W, Redecker C, Schwarz M, Woitalla D. [Parkinson's Disease at the Border Between Inpatient and Outpatient Care]. Fortschr Neurol Psychiatr. 2016;84 Suppl 1:S36-40
7. Ellis T, Katz DI, White DK, DePiero TJ, Hohler AD, Saint-Hilaire M. Effectiveness of an inpatient multidisciplinary rehabilitation program for people with Parkinson disease. Phys Ther. 2008;88(7):812-9
8. Oguh O, Videnovic A. Inpatient management of Parkinson disease: current challenges and future directions. Neurohospitalist. 2012;2(1):28-35
9. Tönges L, Bartig D, Muhlack S, Jost W, Gold R, Krogias C. [Characteristics and dynamics of inpatient treatment of patients with Parkinson's disease in Germany : Analysis of 1.5 million patient cases from 2010 to 2015]. Nervenarzt. 2019;90(2):167-74
10. Richter D, Bartig D, Muhlack S, Hartelt E, Scherbaum R, Katsanos AH, et al. Dynamics of Parkinson's Disease Multimodal Complex Treatment in Germany from 2010⁻2016: Patient Characteristics, Access to Treatment, and Formation of Regional Centers. Cells. 2019;8(2)
11. Prell T, Siebecker F, Lorrain M, Tönges L, Warnecke T, Klucken J, et al. Specialized Staff for the Care of People with Parkinson's Disease in Germany: An Overview. J Clin Med. 2020;9(8)
12. Titova N, Chaudhuri KR. Nonmotor Parkinson's and Future Directions. Int Rev Neurobiol. 2017;134:1493-505
13. Titova N, Chaudhuri KR. Personalized Medicine and Nonmotor Symptoms in Parkinson's Disease. Int Rev Neurobiol. 2017;134:1257-81
14. Titova N, Chaudhuri KR. Personalized medicine in Parkinson's disease: Time to be precise. Mov Disord. 2017;32(8):1147-54
15. Utsumi H, Terashi H, Ishimura Y, Takazawa T, Okuma Y, Yoneyama M, Mitoma H. How far do the complaints of patients with Parkinson's disease reflect motor fluctuation? Quantitative analysis using a portable gait rhythmogram. ISRN Neurol. 2012;2012:372030
16. Farley BG, Koshland GF. Training BIG to move faster: the application of the speed-amplitude relation as a rehabilitation strategy for people with Parkinson's disease. Exp Brain Res. 2005;167(3):462-7
17. Song R, Grabowska W, Park M, Osypiuk K, Vergara-Diaz GP, Bonato P, et al. The impact of Tai Chi and Qigong mind-body exercises on motor and non-motor function and quality of life in Parkinson's disease: A systematic review and meta-analysis. Parkinsonism Relat Disord. 2017;41:3-13

18. Zhang S, Liu D, Ye D, Li H, Chen F. Can music-based movement therapy improve motor dysfunction in patients with Parkinson's disease? Systematic review and meta-analysis. Neurol Sci. 2017;38(9):1629-36

19. Buhmann C, Jungnickel D, Lehmann E. Stress Management Training (SMT) Improves Coping of Tremor-Boosting Psychosocial Stressors and Depression in Patients with Parkinson's Disease: A Controlled Prospective Study. Parkinsons Dis. 2018;2018:4240178

20. Straka I, Minár M, Gažová A, Valkovič P, Kyselovič J. Clinical aspects of adherence to pharmacotherapy in Parkinson disease: A PRISMA-compliant systematic review. Medicine (Baltimore). 2018;97(23):e10962

21. Schiavolin S, Raggi A, Quintas R, Cerniauskaite M, Giovannetti AM, Covelli V, et al. Psychosocial difficulties in patients with Parkinson's disease. Int J Rehabil Res. 2017;40(2):112-8

22. de Rooy FBB, Buhmann C, Schönwald B, Martinez-Martin P, Rodriguez-Blazquez C, Putter H, et al. Discussing sexuality with Parkinson's disease patients: a multinational survey among neurologists. J Neural Transm (Vienna). 2019;126(10):1273-80

23. Martins J, Rua A, Vila Chã N. [Hospital Mortality in Parkinson's Disease: Retrospective Analysis in a Portuguese Tertiary Centre]. Acta Med Port. 2016;29(5):315-8

24. Knop M, Sämann PG, Keck ME. Parkinson-Komplextherapie am Max-Planck-Institut für Psychiatrie: Ein multimodales, flexibles stationäres Therapieprogramm für kritische Krankheitsphasen. Psychiatrie und Neurologie 5/2017: 24-29.

25. Forster A, Young J, Lambley R, Langhorne P. Medical day hospital care for the elderly versus alternative forms of care. Cochrane Database Syst Rev. 2008(4):Cd001730

26. Müller T, Öhm G, Eilert K, Möhr K, Rotter S, Haas T, et al. Benefit on motor and non-motor behavior in a specialized unit for Parkinson's disease. J Neural Transm (Vienna). 2017;124(6):715-20

27. Hartelt E, Scherbaum R, Kinkel M, Gold R, Muhlack S, Tönges L. Parkinson's Disease Multimodal Complex Treatment (PD-MCT): Analysis of Therapeutic Effects and Predictors for Improvement. J Clin Med. 2020;9(6)

3.27 Stationäre multidisziplinäre Therapie

Autoren: Lars Tönges, Florian Wegner, Georg Ebersbach

Fragestellung 199: Wie effektiv ist die stationäre multidisziplinäre Therapie (z.B. multimodale Komplexbehandlung) im Vergleich mit der stationären Standardtherapie in der Behandlung der PK?

Hintergrund

Die stationäre multidisziplinäre Therapie der PK wird in Deutschland als multimodale Komplexbehandlung oder Parkinson-Komplextherapie (PKT) in mehr als 200 Krankenhäusern durchgeführt [1]. Hierbei wird die pharmakologische Therapie zusammen mit nicht pharmakologischen Therapien eingesetzt. Diese umfassen Physio- und Ergotherapie sowie Logopädie, Sporttherapie oder Künstlerische Therapien und werden auch als aktivierende Therapien bezeichnet [2]. Die PKT wird in Deutschland mindestens 7, meist jedoch 14 bis 20 Tage lang angewendet [1]. Das Ziel der Therapie ist aus medizinischer Sicht, die Funktionsfähigkeit zu optimieren, die Behinderung zu reduzieren und dadurch die Lebensqualität zu fördern. International bestehen teilweise etwas modifizierte Ansätze dieser Therapie [3], [4], [5], [6].

Evidenzgrundlage

Zur Fragestellung liegen 4 internationale, randomisierte, kontrollierte Studien und 5 longitudinale Kohortenstudien aus Deutschland vor.

Ergebnis

Eine Beobachtungsstudie in Deutschland mit 47 leicht bis moderat betroffenen Pat. mit PK zeigte eine unmittelbare Verbesserung motorischer Symptome, der Lebensqualität und der Depressivität. Die motorischen Symptome wurden nach Abschluss der stationären Behandlung im Durchschnitt um 4,7 Punkte auf der MDS-Unified Parkinson’s Disease Rating Scale III (MDS-UPDRS III) reduziert und blieben in der Nachbeobachtung 6 Wochen nach Entlassung weiterhin verbessert. Der Zugewinn an Lebensqualität stieg in dem Ausmaß an, das sonst innerhalb von zwei Jahren Erkrankungsverlauf verloren wird [7]. In einer weiteren monozentrischen Studie in Deutschland mit primärem Analyseendpunkt motorische Aspekte bei Erfahrungen des täglichen Lebens (MDS-Unified Parkinson’s Disease Rating Scale II – MDS-UPDRS II) wurde an 591 Fällen nachgewiesen, dass in der gesamten Gruppe eine Verbesserung um 2,4 Punkte im MDS-UPDRS II resultierte. Eine gutes, klinisch relevantes Verbesserungspotenzial war abhängig vom Alter und der MDS-UPDRS-II-basierten Erkrankungsschwere zu Behandlungsbeginn. Das Vorliegen psychiatrischer Erkrankungen (F-Diagnosen) oder kognitiver Störungen (F02.3) war mit ungünstigeren Behandlungsergebnissen assoziiert [8]. Weitere Studien aus Deutschland untermauern positive motorische und nicht motorische Effekte dieser Therapie [9], [10], [11].

Auf internationaler Ebene konnten stationäre multidisziplinäre Therapien mit unterschiedlichen Designs in randomisierten kontrollierten Studien die motorischen Symptome, Alltagsaktivitäten und Lebensqualität von Pat. mit PK verbessern. Die Effekte hielten zum Teil bis ein Jahr nach Beendigung der Intervention an [3], [4], [5], [6].

Begründung der Empfehlung

Die Evidenz beruht auf 4 internationalen, randomisierten, kontrollierten Studien und 5 longitudinalen Kohortenstudien aus Deutschland.

Direkte Vergleichsstudien zur Effektivität der stationären multidisziplinären Therapie gegenüber einer stationären Standardtherapie in der Behandlung der PK liegen nicht vor. Hierzu ist anzumerken, dass keine definierten Kriterien für eine „stationäre Standardtherapie“ existieren, sodass ein Vergleich methodisch problematisch wäre. Es ist jedoch davon auszugehen, dass die stationäre multidisziplinäre Therapie aufgrund des definierten Mindestumfangs nicht pharmakologischer aktivierender Therapien der sonst üblichen stationären Versorgung deutlich überlegen ist. Entsprechende wissenschaftliche Untersuchungen sind in Vorbereitung.

**Empfehlung**	**Neu**<br>**Stand (2023)**
Die stationäre multidisziplinäre Therapie (z.B. multimodale Komplexbehandlung) sollte gegenüber der stationären Standardtherapie der PK bevorzugt eingesetzt werden.	
Konsensstärke: 100%, starker Konsens	

Referenzen

1. Richter D, Bartig D, Muhlack S, Hartelt E, Scherbaum R, Katsanos AH, et al. Dynamics of Parkinson's Disease Multimodal Complex Treatment in Germany from 2010⁻2016: Patient Characteristics, Access to Treatment, and Formation of Regional Centers. Cells. 2019;8(2)
2. Witt K, Kalbe E, Erasmi R, Ebersbach G. [Nonpharmacological treatment procedures for Parkinson's disease]. Nervenarzt. 2017;88(4):383-90
3. Monticone M, Ambrosini E, Laurini A, Rocca B, Foti C. In-patient multidisciplinary rehabilitation for Parkinson's disease: A randomized controlled trial. Mov Disord. 2015;30(8):1050-8
4. Ferrazzoli D, Ortelli P, Zivi I, Cian V, Urso E, Ghilardi MF, et al. Efficacy of intensive multidisciplinary rehabilitation in Parkinson's disease: a randomised controlled study. J Neurol Neurosurg Psychiatry. 2018;89(8):828-35
5. Marumoto K, Yokoyama K, Inoue T, Yamamoto H, Kawami Y, Nakatani A, et al. Inpatient Enhanced Multidisciplinary Care Effects on the Quality of Life for Parkinson Disease: A Quasi-Randomized Controlled Trial. J Geriatr Psychiatry Neurol. 2019;32(4):186-94
6. Frazzitta G, Bertotti G, Riboldazzi G, Turla M, Uccellini D, Boveri N, et al. Effectiveness of intensive inpatient rehabilitation treatment on disease progression in parkinsonian patients: a randomized controlled trial with 1-year follow-up. Neurorehabil Neural Repair. 2012;26(2):144-50
7. Scherbaum R, Hartelt E, Kinkel M, Gold R, Muhlack S, Tönges L. Parkinson's Disease Multimodal Complex Treatment improves motor symptoms, depression and quality of life. J Neurol. 2020;267(4):954-65
8. Ziegler K, Messner M, Paulig M, Starrost K, Reuschenbach B, Fietzek UM, Ceballos-Baumann AO. Activities of Daily Living Are Improved by Inpatient Multimodal Complex Treatment for PD-a Real-World Cohort Study. Mov Disord Clin Pract. 2023;10(1):42-54
9. Hartelt E, Scherbaum R, Kinkel M, Gold R, Muhlack S, Tönges L. Parkinson's Disease Multimodal Complex Treatment (PD-MCT): Analysis of Therapeutic Effects and Predictors for Improvement. J Clin Med. 2020;9(6)
10. Müller T, Öhm G, Eilert K, Möhr K, Rotter S, Haas T, et al. Benefit on motor and non-motor behavior in a specialized unit for Parkinson's disease. J Neural Transm (Vienna). 2017;124(6):715-20
11. Heimrich KG, Prell T. Short- and Long-Term Effect of Parkinson's Disease Multimodal Complex Treatment. Brain Sci. 2021;11(11)

3.28 Versorgungsnetzwerke

Autoren: Carsten Eggers, Tobias Warnecke

Fragestellung 200: Wie effektiv ist die integrierte Versorgung in einem Versorgungsnetzwerk im Vergleich mit der ambulanten Standardtherapie in der Behandlung der PK?

Hintergrund

Parkinson-Netzwerke sind regionale Versorgungsansätze zur Verbesserung der Versorgung von Parkinson-Pat. durch eine intensivierte Zusammenarbeit von Berufsgruppen, intensiveren Austausch, verbesserte Kommunikation sowie Wissenstransfer und Fortbildung [1].

Internationale Daten zu einer integrierten Versorgung im Sinne eines koordinierten Versorgungsprozesses zeigen, dass durch eine solche Versorgungskoordination eine Verbesserung der parkinsonbezogenen Lebensqualität erreicht werden kann [2].

In Deutschland gibt es keine flächendeckende Verbreitung von Parkinson-Netzwerken, sodass es sich bisher stets um regionale und überregionale Initiativen mit unterschiedlichen Schwerpunkten handelt.

Angesichts des Informationsbedürfnisses und der zunehmenden Anzahl der Pat. sollte bei Diagnosestellung auf die Bedeutung von Selbsthilfegruppen hingewiesen werden. Selbsthilfegruppen sollten in Versorgungsnetzwerken aktiv mitwirken.

Evidenzgrundlage und Ergebnisse

In der Literaturrecherche fanden sich insgesamt acht randomisierte kontrollierte Studien, die gesundheitsbezogene Lebensqualität (gemessen am PDQ-8/39) als primären Outcome-Parameter hatten. Einschlusskriterium war ein interdisziplinärer Behandlungsansatz mit einem Neurologen plus mind. zwei weiteren Berufsgruppen (Parkinson-Nurse, Therapeutinnen/Therapeuten, Ärztinnen/Ärzte) sowie Parkinson-Diagnose nach UK-Brain Bank-Kriterien, sowohl im stationären als auch im ambulanten Setting. Wichtig war der Einbezug einer/eines „care coordinators“, die Berufsgruppen hierbei waren heterogen gewählt, meistens handelte es sich um Parkinson-Nurses. Zu den Aufgaben zählten vor allem die Überwachung eines erstellten Therapieplans, die Information der Erkrankten zu den notwendigen Therapien oder das Fungieren als Ansprechpartner/Ansprechpartnerin für Erkrankte und Angehörige. Herangezogen wurde eine Metaanalyse aus dem Jahr 2020 als Evidenzgrundlage. Eingeflossen sind hier auch Daten aus dem Kölner Parkinson Netzwerk [3], die zeigen konnten, dass die koordinierte Versorgung durch eine Parkinson-Nurse über einen Zeitraum von sechs Monaten zu einer Verbesserung der Lebensqualität, Reduktion nicht motorischer Symptome und Besserung der motorischen Symptome führt.

Die Daten zeigten insgesamt, dass ein integriertes Behandlungsmodell unter Einbezug einer/eines „care coordinators“ über einen Zeitraum von sechs Monaten zu einer signifikanten Verbesserung der gesundheitsbezogenen Lebensqualität geführt hat. Hier waren die Studien mit einem Behandlungszeitraum von mind. 6 Monaten und im ambulanten Sektor signifikant den Studien

überlegen, die einen kürzeren Behandlungszeitraum vorsahen und/oder im stationären Bereich angelegt waren.

Zudem ist erkennbar, dass Netzwerkstrukturen die Kommunikation und Zusammenarbeit zwischen den professionellen Gesundheitsdienstleistern in Deutschland verbessern können. Anhand einer sozialen Netzwerkanalyse aus dem Parkinson-Netz Münsterland wurde deutlich, dass der regelmäßige Austausch in Plenumssitzungen, strukturierte Kommunikationspfade und Fortbildungen zu einem erhöhten Wissenstransfer sowie erhöhter professioneller Kommunikation und Spezialisierung führen [4].

Begründung der Empfehlung

Integrierte Versorgungsansätze führen bei einer Behandlung im interdisziplinären Team unter Einbezug eines Versorgungskoordinators/einer Versorgungskoordinatorin zu einer Verbesserung der parkinsonbezogenen Lebensqualität sowie zu interprofessionellem Wissenstransfer und Kommunikation.

**Empfehlung**	**Neu**<br>**Stand (2023)**
Es kann empfohlen werden, dass Pat. im Sinne eines integrierten Versorgungsansatzes in Parkinson-Netzwerken behandelt werden.	
Konsensstärke: 100%, starker Konsens	

3.29 Parkinson-Pflege

Autoren: Carsten Eggers, Tobias Warnecke

Fragestellung 201: Wie effektiv ist die Pflege durch eine/n spezialisierte Parkinson-Nurse im Vergleich mit der medizinischen Standardtherapie in der Behandlung der PK?

Hintergrund

Spezialisierte Krankenschwestern und Krankenpfleger für Pat. mit PK, sogenannte Parkinson-Nurses (PN; engl.: Parkinson's Disease Nurse, PDN), gibt es weltweit seit mehr als 40 Jahren, u.a. in England, den USA und den skandinavischen Ländern. Es wurde bisher aber kein einheitlicher internationaler Ausbildungsstandard für PN entwickelt. Die Arbeitsverhältnisse in den nationalen und regionalen Gesundheitssystemen sind sehr unterschiedlich. In England arbeiten PN manchmal mit Neurologinnen/Neurologen oder Geriaterinnen/Geriatern zusammen, oft aber auch mit Hausärzten/Hausärztinnen oder selbstständig, manchmal im Krankenhaus, oft aber in Praxen. In Deutschland arbeiten die meisten PN im Krankenhaus und unter Supervision eines Neurologen/einer Neurologin. Die Arbeitsaufgaben der PN variieren zwischen den verschiedenen Arbeitsplätzen und können z.B. „Case-Management-Aufgaben", Betreuung von Pat. mit aufwendigen Therapieformen wie Pen-/Pumpentherapien und Tiefer Hirnstimulation oder auch klinische Forschungsaufgaben beinhalten. In Skandinavien und den Niederlanden ist eine PN oft Teil eines multiprofessionellen

„Parkinson-Teams", dem auch auf Parkinson spezialisierte Ärztinnen/Ärzte, Physiotherapeuten/Physiotherapeutinnen, Ergotherapeuten/Ergotherapeutinnen und Logopäden/Logopädinnen angehören. In Deutschland ist diese Art von Behandlungsteam im ambulanten Setting noch selten anzutreffen, wird aber zunehmend in Parkinson-Netzwerken angeboten. Diese sehr unterschiedlichen Arbeitsbedingungen müssen berücksichtigt werden, wenn Studienergebnisse zu diesem Thema interpretiert werden sollen. In Deutschland haben die Deutsche Parkinson-Gesellschaft, die Deutsche Parkinson Vereinigung e. V. und das Kompetenznetz Parkinson ein Fortbildungs-Curriculum entwickelt, das den Ausbildungsstandard für PN in Deutschland darstellt. Die erste Fortbildung wurde im Juni 2007 durchgeführt. Jedes Jahr werden aktuell 20 Krankenschwestern und Krankenpfleger zu PN weitergebildet. Diesem Curriculum hat sich 2022 auch die Deutsche Gesellschaft für Neurologie angeschlossen. Das Curriculum wurde 2022 aktualisiert.

Evidenzgrundlage und Ergebnisse

Vier randomisiert kontrollierte [5], [6], [7], [8] Studien und eine Metaanalyse [9] wurden gefunden, die sich mit der Intervention einer PN im Vergleich zu sonstiger nicht ärztlicher Krankenversorgung beschäftigten. Drei Studien wurden in England und eine Studie wurde in den USA durchgeführt. In der Metaanalyse wurden auch Studien berücksichtigt, in denen der/die PN Mitglied eines multiprofessionellen Behandlungsteams war. Aus solchen Studien kann der alleinige Effekt der PN-Intervention im Vergleich mit einer medizinischen Standardtherapie nicht abgeleitet werden [9]. Zusammenfassend gibt es nur wenige relevante Studien. Die Studienmethoden limitieren die Aussagekraft der Ergebnisse: Die Pat.-Zahlen waren niedrig, das Einzugsgebiet der Pat. und das Design der Intervention heterogen. Daten aus Deutschland liegen nicht vor.

Begründung der Empfehlung

Insgesamt gibt es noch verhältnismäßig wenig Evidenz zum Effekt einer PN-Intervention bei Pat. mit PK. Die bisherigen Ergebnisse spiegeln nur begrenzt die Vorteile bezüglich der klinischen Symptomatologie wider, wenn eine Betreuung durch eine/einen PN zusätzlich zum behandelnden Hausarzt/zu der behandelnden Hausärztin oder Neurologen/Neurologin angeboten wird. Die Resultate zeigen aber, dass ausreichende Kompetenz und Kapazität zur Überwachung, Anpassung der Medikation, Information und Unterstützung wesentlich sind und von unterschiedlichen Berufsgruppen angeboten werden können. Die jüngste, methodisch hochwertige randomisierte kontrollierte Studie zeigt einen signifikanten positiven Effekt auf die Adhärenz der Pat. zu 18 die Lebensqualität betreffenden Parkinson-Qualitätsindikatoren [5]. In der Metaanalyse konnte aufgrund der großen Heterogenität im Studiendesign und der untersuchten Interventionen kein Effekt der personalisierten Versorgungsmodelle auf die Lebensqualität nachgewiesen werden, PN konnten aber zu allen Aspekten einer personalisierten Parkinson-Versorgung beitragen [9]. Die Evidenz bezüglich Kosteneffektivität durch eine PN ist bisher nicht konklusiv. Gleichzeitig muss betont werden, dass nach Expertenmeinung die klinische Erfahrung und die subjektive Pat.-Beurteilung bezüglich Betreuung durch eine PN oft sehr positiv sind.

Empfehlung	Modifiziert<br>Stand (2023)
▪ Es kann empfohlen werden, dass Personen mit PK regelmäßigen Zugang zu einer Betreuung durch eine/einen PN haben.<br>▪ Die/der PN kann in Absprache mit den behandelnden Ärzten/Ärztinnen in die klinische Überwachung und Anpassung der Medikation sowie die Kontrolle einer Tiefen Hirnstimulation eingebunden sein.<br>▪ Die/der PN kann in regelmäßigem Kontakt zu betreuenden Personen, auch mit Hausbesuch, wenn angemessen, stehen.<br>▪ Die/der PN kann als zuverlässige Informationsquelle über klinische und soziale Angelegenheiten, die Parkinson-Pat. und ihre Pflegeperson/Familien betreffen, zur Verfügung stehen.	
Konsensstärke: 100%, starker Konsens	

1. Stangl S, Haas K, Eggers C, Reese JP, Tönges L, Volkmann J. [Care of patients with Parkinson's disease in Germany]. Nervenarzt. 2020;91(6):493-502
2. Rajan R, Brennan L, Bloem BR, Dahodwala N, Gardner J, Goldman JG, et al. Integrated Care in Parkinson's Disease: A Systematic Review and Meta-Analysis. Mov Disord. 2020;35(9):1509-31
3. Eggers C, Dano R, Schill J, Fink GR, Hellmich M, Timmermann L. Patient-centered integrated healthcare improves quality of life in Parkinson's disease patients: a randomized controlled trial. J Neurol. 2018;265(4):764-73
4. Kerkemeyer L, Claus I, Kutscher M, von Stülpnagel V, Zur Nieden P, Huchtemann T, Warnecke T. Strengthening Communication and Collaboration in the Fragmented German Healthcare System: A Mixed-Method Evaluation of an Interdisciplinary Network for Parkinson's Disease. J Parkinsons Dis. 2022;12(4):1307-17
5. Connor KI, Cheng EM, Barry F, Siebens HC, Lee ML, Ganz DA, et al. Randomized trial of care management to improve Parkinson disease care quality. Neurology. 2019;92(16):e1831-e42
6. Hurwitz B, Jarman B, Cook A, Bajekal M. Scientific evaluation of community-based Parkinson's disease nurse specialists on patient outcomes and health care costs. J Eval Clin Pract. 2005;11(2):97-110
7. Jarman B, Hurwitz B, Cook A, Bajekal M, Lee A. Effects of community based nurses specialising in Parkinson's disease on health outcome and costs: randomised controlled trial. Bmj. 2002;324(7345):1072-5
8. Reynolds H, Wilson-Barnett J, Richardson G. Evaluation of the role of the Parkinson's disease nurse specialist. Int J Nurs Stud. 2000;37(4):337-49
9. van Munster M, Stümpel J, Thieken F, Ratajczak F, Rascol O, Fabbri M, et al. The Role of Parkinson Nurses for Personalizing Care in Parkinson's Disease: A Systematic Review and Meta-Analysis. J Parkinsons Dis. 2022;12(6):1807-31

3.30 Palliativversorgung

Autoren: Carsten Eggers, Martin Klietz, Stefan Lorenzl

Fragestellung 202: Welche speziellen Symptome treten im finalen Krankheitsstadium im Vergleich zum vorherigen Krankheitsverlauf der PK auf?

Hintergrund

Bisher gibt es keine einheitlich verwendete Definition der palliativen Phase der PK. In einer aktuellen Definition wird ein Hoehn-&-Yahr-Stadium von III oder höher gemeinsam mit einem Autonomieverlust der Pat. als palliatives Interventionsstadium gewertet [1], [2], [3]. Zum anderen wird das Spätstadium der PK mit einer Krankheitsdauer von mindestens 7 Jahren und einem Hoehn-&-Yahr-Stadium von mindestens IV oder profunden Einschränkungen in den Aktivitäten des täglichen Lebens definiert [4].

Evidenzgrundlage

Die Literaturrecherche ergab, dass keine randomisierten kontrollierten Studien zu diesem Thema vorhanden waren. Es wurden größtenteils Daten aus Pat.-Kohorten und Übersichtsartikeln zur Bearbeitung der Frage herangezogen.

In diesem Erkrankungsstadium dominieren nicht direkt dopaminerg beeinflussbare Symptome. In einer prospektiven internationalen multizentrischen Studie mit 692 Pat. im weit fortgeschrittenen Erkrankungsstadium konnte gezeigt werden, dass die Pat. unter ausgeprägten und zum Teil auch sehr komplexen Wirkfluktuationen der dopaminergen Therapie wie behindernde Dyskinesien und Off-Phasen leiden [4]. Es bestehen komplexe axiale Symptome wie zum Beispiel ein Pisa-Syndrom und die Pat. erleben eine posturale Instabilität mit vermehrten Stürzen.

Zusätzlich sind nicht motorische Symptome ein wesentlicher Einflussfaktor für die reduzierte Lebensqualität der Pat. [4], [1].

Im Bereich der nicht motorischen Symptome spielen neuropsychiatrische Symptome eine besondere Rolle. In einer internationalen prospektiven multizentrischen Studie an weit fortgeschrittenen Parkinson-Pat. konnte gezeigt werden, dass die Prävalenz von depressiven Symptomen, Apathie und Ängsten sehr hoch war [5]. In einer fragebogenbasierten Studie an 89 fortgeschrittenen Parkinson-Pat. konnte gezeigt werden, dass neuropsychiatrische Symptome einen großen Einfluss auf die Lebensqualität von Parkinson-Pat. haben [6]. Zusätzlich konnte in einer prospektiven Kohorte aus Australien gezeigt werden, dass nach 15–18 Jahren Krankheitsdauer ein Großteil der Parkinson-Pat. (84%) eine kognitive Störung zeigt und 48% sogar die Kriterien einer Demenz erfüllen [7].

Ebersbach et al. berichten in einer Literaturübersicht darüber, dass in diesem Stadium der Erkrankung die Pat. sehr vulnerabel für delirante Symptome sind, sodass bei Verdacht eine sorgfältige Evaluation erfolgen und ggf. eine spezifische Therapie eingeleitet werden sollte [8].

Dysphagie ist im finalen Stadium der PK ein sehr häufiges Symptom und kann zu Aspirationspneumonien und Dose-failure-Phänomen führen [9].

Alle oben genannten Symptome treffen in der finalen Erkrankungsphase auf Pat. mit geriatrischen Merkmalen wie Polypharmakotherapie und Multimorbidität zu, was einer aktuellen Literaturübersicht zu entnehmen ist [10]. In einer retrospektiven Analyse der Medikation von 123 stationären fortgeschrittenen Parkinson-Pat. zeigten Greten et al. die hohe Vulnerabilität für Komplikationen und Medikamentennebenwirkungen bzw. Interaktionen in dieser Pat.-Gruppe [11].

Ergebnis

Zusammenfassend gibt es in diesem Themenbereich keine randomisiert kontrollierten prospektiven Studien, die Evidenz wurde größtenteils aus Pat.-Kohorten und Fallserien generiert. Hierbei ist aber insbesondere die CLASP-Studie [5] mit einem prospektiven internationalen multizentrischen Ansatz als wertvolle Informationsquelle mit sehr hoher Datenqualität zu nennen.

Das finale Stadium der PK ist durch eine fortgeschrittene motorische PK mit deutlichen Einschränkungen in der Autonomie bzw. der Selbstständigkeit in den Aktivitäten des täglichen Lebens definiert. Die Pat. zeigen komplexe motorische Fluktuationen und leiden neben anderen nicht motorischen Symptomen an schwerwiegenden neuropsychiatrischen Symptomen.

Begründung der Empfehlung

Es können anhand der aktuell vorliegenden Evidenz keine klaren Empfehlungen für die Charakterisierung der palliativen Erkrankungsphase gegeben werden, da dieser Erkrankungsabschnitt international unterschiedlich definiert wird.

<table>
<tr><th>Empfehlung</th><th>Neu

Stand (2023)</th></tr>
<tr><td colspan="2">Es kann aus den vorliegenden Daten extrapoliert werden,
▪ dass die palliative Phase der PK durch Autonomieverlust und eine sehr hohe Symptomlast charakterisiert ist
▪ dass insbesondere neuropsychiatrische Symptome in dieser Erkrankungsphase eine wichtige Rolle spielen und zur schlechten Lebensqualität beitragen
▪ dass Schluckstörungen, Delirien und mit Polypharmazie assoziierte Aspekte eine wichtige Rolle im Management der Pat. spielen.</td></tr>
<tr><td colspan="2">Konsensstärke: 93,1%, Konsens</td></tr>
</table>

Fragestellung 203: Welche spezielle medikamentöse und nicht medikamentöse Symptomkontrolle ist im finalen Krankheitsstadium im Vergleich zur Standardbehandlung der PK möglich?

Hintergrund

Parkinson-Pat. im Spätstadium der Krankheit sind in hohem Maße auf Hilfe bei Aktivitäten des täglichen Lebens angewiesen und haben einen erheblichen medizinischen Bedarf. Es bestehen eine erhebliche Belastung der pflegenden Angehörigen und des professionellen Pflegepersonals sowie größere ökonomische Belastungen für die Familien und das Gesundheitswesen. Das klinische Bild ist im Spätstadium vermehrt durch nicht motorische Symptome gekennzeichnet. In dieser Phase der PK

ist die orale Aufnahme von Medikamenten häufig erschwert oder nur verzögert möglich. Das schränkt die Gabe von Medikamenten zur Symptomkontrolle ein. Zum anderen sind durch die kognitiven Veränderungen nicht medikamentöse Therapieverfahren nur bedingt möglich. Die zunehmende Einschränkung der Bewegungsfähigkeit führt häufig zu sekundären Komplikationen wie Frakturen oder Pneumonien.

Evidenzgrundlage

Es existieren lediglich Übersichtsartikel zur medikamentösen und nicht medikamentösen Therapie und keine kontrollierten Studien. In einem Scoping Review wurden 3.821 Artikel gesichtet und letztendlich 20 Artikel für die Datenextraktion ausgewählt. Es konnten lediglich Fallberichte und Übersichtsartikel ausgewertet werden. Zusammengefasst wurden zur dopaminergen Therapie ohne klinische Studienevidenz am häufigsten Rotigotin-Pflaster und Apormorphin-Injektionen empfohlen [12]. Die Anwendung von Rotigotin wurde in einer weiteren retrospektiven Studie untersucht, die eine gute Symptomkontrolle in allerdings niedrigen Dosierungen angibt [13].

Eine weitere Übersichtsarbeit schreibt, dass in den meisten Fällen die dopaminerge Therapie nur suboptimal oder gar nicht die Symptome kontrollieren könne, und dass Interventionen wie Physiotherapie, kognitive Stimulation, Logopädie, Ergotherapie und eine spezialisierte Parkinson-Pflegerin/ein spezialisierter Parkinson-Pfleger eine Schlüsselrolle spielen würden. Eine wissenschaftliche Datengrundlage wird nicht angegeben. Es wird auf das H2020-Projekt PD_PAL verwiesen, welches detailliertere Einsichten ermöglichen werde [14]. Gerade diejenigen Symptome, die nicht auf eine dopaminerge Therapie ansprechen, werden in der Spätphase als problematisch angesehen [15].

In einer Studie wird anhand von ethnografischen Familieninterviews festgestellt, dass das Leben in einem Pflegeheim, das den Bedürfnissen der Pat. gerecht wird, die beste Option für Parkinson-Pat. im Endstadium ihrer Krankheit sowie für deren Angehörige ist [16]. Auch eine telemedizinische Unterstützung kann in der fortgeschrittenen Phase hilfreich sein, um den Kontakt zu den Pat. und den Familien aufrechtzuerhalten [17].

Ergebnis

Zusammenfassend gibt es keine randomisiert kontrollierten prospektiven Studien. Auch hier wurde die Evidenz aus Pat.-Kohorten und Fallserien generiert. Erneut müssen die CLASP- und die PD_PAL-Studie mit einem prospektiven internationalen multizentrischen Ansatz genannt werden, deren Ergebnisse aber größtenteils noch ausstehen. Es wird daher über medikamentöse und nicht medikamentöse Therapieverfahren spekuliert.

Begründung der Empfehlung

Anhand der vorliegenden Datenlage ist eine evidenzbasierte Empfehlung zur medikamentösen und nicht medikamentösen Therapie in der Spätphase nicht gerechtfertigt.

Empfehlung	Neu<br>Stand (2023)
Folgende therapeutischen Möglichkeiten können angeboten werden:<br>▪ Anwendung von Levodopa bei erhaltener Schluckfähigkeit oder über eine PEG-Sonde; eine Alternative insbesondere bei Schluckstörung oder Ileus-Symptomatik ist die Gabe von rektal zu applizierendem, schnell löslichem Levodopa.<br>▪ Anwendung des Rotigotin-Pflasters in niedriger Dosis, v.a. bei Schluckstörungen<br>▪ Anwendung von Apomorphin s.c. (Anwendung mit zunächst niedrig dosierter Pumpentherapie, keine Einzelinjektionen, da palliativmedizinisch möglichst wenig Manipulationen am Pat. vorgenommen werden sollen)<br>▪ Fortführen stimulierender physiotherapeutischer und neuropsychologischer Therapiemaßnahmen.	
Konsensstärke: 90,9%, Konsens	

Fragestellung 204: Welche palliative ambulante oder stationäre Versorgung benötigen Pat. und Angehörige mit der PK im finalen Krankheitsstadium?

Hintergrund

Die palliative Phase der PK ist bislang wenig untersucht. In den letzten Jahren ist es jedoch zu einem Paradigmenwechsel bezüglich der Definition des Einsatzgebiets gekommen. So zielt Palliative Care nicht nur auf Linderung des Leidens im Endstadium der Erkrankung ab, sondern berücksichtigt auch frühere Krankheitsphasen. Der Ansatz der Palliativpflege beschränkt sich dabei nicht nur auf die Schmerzbekämpfung, sondern befasst sich auch mit allen körperlichen, psychologischen und spirituellen Problemen, die die Lebensqualität im Verlauf der Krankheit verschlechtern. Der hierbei geprägte Begriff der „early integration“ umfasst die frühe Einbeziehung von Palliativstrukturen und kann hilfreich sein in der Krankheitsbewältigung und der Aufrechterhaltung von Lebensqualität. Der genaue Zeitpunkt der Aufnahme von palliativer Betreuung ist unklar.

Die physische und emotionale Belastung für die Pat. und deren Familien nimmt in dieser Phase noch deutlich zu, ebenso die direkten und indirekten Krankheitskosten. Die meisten Angehörigen sehen sich in der Pflicht, die schwer kranken Pat. zu Hause zu versorgen, oft in Unkenntnis der zur Verfügung stehenden sozialen Unterstützungsmöglichkeiten.

Als Grundsätze für Palliative Care (sowohl ambulant als auch stationär) können gelten:

- Pat. sollten Zugang zu einem multidisziplinären Team haben, das u.a. aus Physiotherapeut/Physiotherapeutin, Ergotherapeut/Ergotherapeutin, Logopäde/Logopädin, Psychologe/Psychologin, Sozialarbeiter/Sozialarbeiterin, Krankenpflegekraft und Arzt/Ärztin besteht. Der Einsatz eines multidisziplinären Teams kann zu einer Verbesserung der Lebensqualität führen.
- Die Kommunikation mit Pat. und Angehörigen sollte offen und strukturiert sein sowie konkrete Therapieziele und -optionen beinhalten.

- Es ist sehr empfohlen, advanced care planning früh im Erkrankungsprozess zu berücksichtigen, insbesondere vor der Entwicklung von kognitiven Defiziten.
- Vorsorgevollmachten können in der Versorgungsplanung hilfreich sein, sind jedoch bisher oft zu unspezifisch bezüglich der Parkinson-spezifischen Vorsorgeinhalte. Es sollte eine strukturierte Beratung und Aufklärung über Möglichkeiten und Inhalte durch den betreuenden Arzt/die betreuende Ärztin erfolgen.

Evidenzgrundlage

Die Literaturrecherche ergab, dass keine randomisierten kontrollierten Studien vorhanden waren. Es wurden 15 Falldarstellungen und Review-Artikel einbezogen.

In einer systematischen Befragung wurden 6.442 Mitglieder der International Parkinson and Movement Disorder Society kontaktiert, hiervon antworteten 652 auf einen Fragebogen zur Einstellung und zu Zugangswegen zu palliativer Versorgung von Pat. mit Bewegungsstörungen. Es zeigte sich, dass eine knappe Mehrheit der Pat. Zugang zu institutionellen (palliativen) Pflegeeinrichtungen hatte (54%). 89% der Befragten gaben an, dass eine interdisziplinäre palliative Zusammenarbeit stattfindet. Der Zugang zu palliativen Versorgungsstrukturen fand meistens anlassbezogen statt (in 75% der Fälle) [18].

Zur Frage der Vorsorgevollmacht konnte erhoben werden, dass zwar die Mehrheit der Pat. eine Vorsorgevollmacht besitzt (69%), die Inhalte jedoch bisher oft zu unspezifisch bezüglich der Parkinson-spezifischen Vorsorgeinhalte sind. Es sollte eine strukturierte Beratung und Aufklärung über Möglichkeiten und Inhalte durch den betreuenden Arzt/die betreuende Ärztin erfolgen [19].

Anhand von semistrukturierten Interviews mit Parkinson-Erkrankten, die seit einem Jahr in einem Pflegeheim aufgenommen waren, konnte herausgearbeitet werden, dass bisher kein klarer Kriterienkatalog für die institutionelle/palliative Pflege in der späten Krankheitsphase der PK existiert. Zugleich konnten die Daten zeigen, dass die Angehörigenbelastung sowie die depressive Symptomatik bei den Angehörigen nach der Pflegeheimaufnahme sinken. Hauptgründe für eine stationäre Pflegeheimaufnahme waren die Angst vor dem Umgangs mit sehr schweren Parkinson-Symptomen sowie die übermäßig hohe Belastung der pflegenden Angehörigen [20].

Es gibt eine hohe Rate von suizidalen Ideationen bei Parkinson-Erkrankten. Ein Chart Review bei einer Schweizer Organisation für selbstbestimmtes Sterben ergab, dass ein Anteil von 7,2% der Gesamtanfragen von Pat. mit PK und atypischem Parkinson-Syndrom stammte. Dieser hohe Anteil zeigt eine Überrepräsentation von Parkinson-Erkrankten im Vergleich zur Gesamtpopulation von Anfragen nach einem assistierten Suizid und demonstriert, dass insbesondere auch in der Frühphase eine intensive psychologische Betreuung von Erkrankten notwendig ist [21].

Aufgrund der Schwere der Symptomatik in der späten Krankheitsphase wurden Parkinson-Erkrankte bisher häufig nicht in systematische Studien eingeschlossen. In einem Expertenreview wurde ermittelt, dass das individuelle Versorgungskonzept eines/einer Pat. sich an den Wünschen, Bedürfnissen und lokalen Voraussetzungen orientieren sollte. Dies kann die Versorgung in einer Pflegeeinrichtung, einem Hospiz, die ambulante/stationäre Palliativversorgung oder auch die

hausärztliche Versorgung beinhalten. Als Hinweise zur Ermittlung von Risikopat. in der späten Krankheitsphase dienen die folgenden Kriterien: Hoehn-&-Yahr-Stadium IV–V, schwere Bradykinese, axiale Symptome, mittelgradige Dysphagie, nächtliche Schlafstörungen und/oder Tagesschläfrigkeit, Demenz, psychotische Symptomatik, depressive Störung.

Risikofaktoren für einen erhöhten palliativmedizinischen Versorgungsbedarf sind Schluckprobleme, wiederholte Infekte, sichtbarer körperlicher Abbau, Aspirationspneumonie, deutlicher kognitiver Abbau oder Gewichtsverlust [22].

Ein plötzlicher Ausfall der Tiefen Hirnstimulation kann zum Tiefe-Hirnstimulation-Entzugssyndrom führen. In einer retrospektiven Analyse von 446 Pat. konnte gezeigt werden, dass ein Generatoraustausch in der späten Krankheitsphase (n=11) nicht mit einem erhöhten Komplikationsrisiko behaftet ist [23].

In einem Review stellten die Autoren dar, dass Pat. möglicherweise einen Symptomprogress nicht ihren Ärzten/Ärztinnen mitteilen, da sie Sorge vor einer Änderung der Medikation haben. Zudem führt eine mangelhafte Kommunikation zwischen Ärztinnen/Ärzten, Pflegekräften und Therapeuten/Therapeutinnen zu Frustration und Unsicherheit bei Pat. und Angehörigen [24].

Ergebnis

Zusammenfassend gibt es nur wenige relevante Studien. Die Studienmethoden limitieren die Aussagekraft der Ergebnisse: Die Pat.-Zahlen waren niedrig und das Design der Interventionen heterogen. Im Wesentlichen bestehen nur Expertenmeinungen zur Frage der ambulanten oder stationären Versorgung in der palliativen Krankheitsphase.

Die Resultate zeigen vor allem, dass ein multiprofessioneller Teameinsatz für die Pat., aber vor allem auch die pflegenden Angehörigen eine Entlastung bringen kann. Auch die Versorgung in einer stationären Pflegeeinrichtung kann zur Entlastung förderlich sein.

Es hat eine hohe Bedeutung, dass in der Spätphase der PK eine detaillierte Symptomanalyse durchgeführt wird. Ein multiprofessionelles Team sollte die Bedürfnisse der Pat. und der Angehörigen zusammenführen und durch eine bestmögliche Symptomkontrolle das natürliche Sterben begleiten. Eine auf die PK abgestimmte und detaillierte Vorsorgevollmacht hilft, die individuellen Wünsche der Pat. für die letzte Lebensphase zu konkretisieren. Ein Schrittmacheraggregat zur Tiefen Hirnstimulation kann auch in der späten Krankheitsphase ausgetauscht werden.

Begründung der Empfehlung

Es können anhand der vorliegenden Evidenz bisher keine konkreten Empfehlungen für die ambulante oder stationäre Versorgung von Parkinson-Pat. in der letzten Lebensphase gegeben werden.

Empfehlung	Neu<br><br>Stand (2023)
Es kann erwogen werden,<br><br>▪ dass eine Vorsorgevollmacht mit konkreten Parkinson-bezogenen Inhalten erstellt wird<br>▪ eine stationäre Pflegeheimaufnahme zur Entlastung von Pat. und Angehörigen dienen kann<br>▪ ein Aggregat für die Tiefe Hirnstimulation auch in der späten Krankheitsphase ausgetauscht werden kann, insbesondere beim Vorliegen eines Tiefe-Hirnstimulation-Entzugssyndroms<br>▪ ein multiprofessionelles Team auf die individuellen Bedürfnisse des Pat. ausgerichtet zusammenarbeiten sollte.	
Konsensstärke: 96,6%, starker Konsens	

Referenzen

1. Klietz M, Tulke A, Müschen LH, Paracka L, Schrader C, Dressler DW, Wegner F. Impaired Quality of Life and Need for Palliative Care in a German Cohort of Advanced Parkinson's Disease Patients. Front Neurol. 2018;9:120
2. Miyasaki JM. Palliative care in Parkinson's disease. Curr Neurol Neurosci Rep. 2013;13(8):367
3. Antonini A, Stoessl AJ, Kleinman LS, Skalicky AM, Marshall TS, Sail KR, et al. Developing consensus among movement disorder specialists on clinical indicators for identification and management of advanced Parkinson's disease: a multi-country Delphi-panel approach. Curr Med Res Opin. 2018;34(12):2063-73
4. Schrag A, Hommel A, Lorenzl S, Meissner WG, Odin P, Coelho M, et al. The late stage of Parkinson's -results of a large multinational study on motor and non-motor complications. Parkinsonism Relat Disord. 2020;75:91-6
5. Hommel A, Meinders MJ, Lorenzl S, Dodel R, Coelho M, Ferreira JJ, et al. The Prevalence and Determinants of Neuropsychiatric Symptoms in Late-Stage Parkinsonism. Mov Disord Clin Pract. 2020;7(5):531-42
6. Eichel HV, Heine J, Wegner F, Rogozinski S, Stiel S, Groh A, et al. Neuropsychiatric Symptoms in Parkinson's Disease Patients Are Associated with Reduced Health-Related Quality of Life and Increased Caregiver Burden. Brain Sci. 2022;12(1)
7. Hely MA, Morris JG, Reid WG, Trafficante R. Sydney Multicenter Study of Parkinson's disease: non-L-dopa-responsive problems dominate at 15 years. Mov Disord. 2005;20(2):190-9
8. Ebersbach G, Ip CW, Klebe S, Koschel J, Lorenzl S, Schrader C, et al. Management of delirium in Parkinson's disease. J Neural Transm (Vienna). 2019;126(7):905-12
9. Suttrup I, Warnecke T. Dysphagia in Parkinson's Disease. Dysphagia. 2016;31(1):24-32
10. Greten S, Müller-Funogea JI, Wegner F, Höglinger GU, Simon N, Junius-Walker U, et al. Drug safety profiles in geriatric patients with Parkinson's disease using the FORTA (Fit fOR The Aged) classification: results from a mono-centric retrospective analysis. J Neural Transm (Vienna). 2021;128(1):49-60
11. Klietz M, Greten S, Wegner F, Höglinger GU. Safety and Tolerability of Pharmacotherapies for Parkinson's Disease in Geriatric Patients. Drugs Aging. 2019;36(6):511-30
12. Kim W, Watt CL, Enright P, Sikora L, Zwicker J. Management of Motor Symptoms for Patients with Advanced Parkinson's Disease without Safe Oral Access: A Scoping Review. J Palliat Med. 2023;26(1):131-41

13. Hindmarsh J, Hindmarsh S, Lee M. Idiopathic Parkinson's Disease at the End of Life: A Retrospective Evaluation of Symptom Prevalence, Pharmacological Symptom Management and Transdermal Rotigotine Dosing. Clin Drug Investig. 2021;41(8):675-83

14. Fabbri M, Coelho M, Garon M, Biundo R, Mestre TA, Antonini A, On Behalf Of i C-PC. Personalized Care in Late-Stage Parkinson's Disease: Challenges and Opportunities. J Pers Med. 2022;12(5)

15. Rukavina K, Batzu L, Boogers A, Abundes-Corona A, Bruno V, Chaudhuri KR. Non-motor complications in late stage Parkinson's disease: recognition, management and unmet needs. Expert Rev Neurother. 2021;21(3):335-52

16. Lex KM, Larkin P, Osterbrink J, Lorenzl S. A Pilgrim's Journey-When Parkinson's Disease Comes to an End in Nursing Homes. Front Neurol. 2018;9:1068

17. Katz M. Telehealth increases access to palliative care for people with Parkinson's disease and related disorders. Ann Palliat Med. 2020;9(Suppl 1):S75-s9

18. Miyasaki JM, Lim SY, Chaudhuri KR, Antonini A, Piemonte M, Richfield E, et al. Access and Attitudes Toward Palliative Care Among Movement Disorders Clinicians. Mov Disord. 2022;37(1):182-9

19. Klietz M, Öcalan Ö, Schneider N, Dressler D, Stiel S, Wegner F. Advance Directives of German People with Parkinson's Disease Are Unspecific in regard to Typical Complications. Parkinsons Dis. 2019;2019:2107821

20. Jensen I, Lescher E, Stiel S, Wegner F, Höglinger G, Klietz M. Analysis of Transition of Patients with Parkinson's Disease into Institutional Care: A Retrospective Pilot Study. Brain Sci. 2021;11(11)

21. Nuebling GS, Butzhammer E, Lorenzl S. Assisted Suicide in Parkinsonian Disorders. Front Neurol. 2021;12:656599

22. Fabbri M, Kauppila LA, Ferreira JJ, Rascol O. Challenges and Perspectives in the Management of Late-Stage Parkinson's Disease. J Parkinsons Dis. 2020;10(s1):S75-s83

23. Carlson JD, Palmer M, Hoefer A, Cox E, McLeod P, Mark J, Aldred J. Deep Brain Stimulation Generator Replacement in End-Stage Parkinson Disease. World Neurosurg. 2019;128:e683-e7

24. Bouça-Machado R, Titova N, Chaudhuri KR, Bloem BR, Ferreira JJ. Palliative Care for Patients and Families With Parkinson's Disease. Int Rev Neurobiol. 2017;132:475-50

Abkürzungsverzeichnis

AADC	aromatische Amino-Decarboxylase
ACE	Addenbrooke's Cognitive Examination
ACE-I	Version I der Addenbrooke's Cognitive Examination
ACE-III	Version III der Addenbrooke's Cognitive Examination
ACE-R	revidierte Version der Addenbrooke's Cognitive Examination
AChE	Acetylcholinesterase-Hemmer
ADAS-cog	Alzheimer Disease Assessment Scale – Abschnitt zur Kognition
ADL	Aktivitäten des täglichen Lebens (engl. Activities of Daily Living)
AES	Apathy Evaluation Scale
BADL	Basis-Aktivitäten des täglichen Lebens (engl. Basic Activities of Daily Living)
BDI	Beck-Depressions-Inventar
BMT	Best Medical Therapy
BNT	Boston Naming Test
B-SIT	Brief 12-item Smell Identification Test
CAM	Confusion Assessment Method
CAMCOG-R	Cambridge Cognitive Assessment – Revised
CBD	kortikobasale Degeneration
CBS	kortikobasales Syndrom
CDS	Continuous Dopaminergic Stimulation
CERAD-Plus	Consortium to Establish a Registry for Alzheimer's Disease
CESD-R	Center for Epidemiologic Studies Depression Rating Scale – Revised
CGI	Clinical Global Impression
CSAI	kontinuierliche subkutane Apomorphin-Infusion
CSDD	Cornell Scale for Depression in Dementia
CSFLI	kontinuierliche subkutane Foslevodopa/Foscarbidopa-Infusion
CTE	chronisch-traumatische Enzephalopathie
d	Effektstärke Cohen's d
DA	Dopaminagonist
DAWS	Dopaminagonisten-Entzugssyndrom (engl. dopamine agonist withdrawal syndrome)
DBS	Deep brain stimulation
DDCI	Dopadecarboxylase-Inhibitor
DDS	Dopaminerges Dysregulations-Syndrom
DDS-PC	Parkinson's Disease Dopamine Dysregulation Syndrome – Patient and Caregiver Inventory
DEGUM	Deutsche Gesellschaft für Ultraschall in der Medizin
D-FIS	Fatigue Impact Scale for Daily Use

DGKN	Deutsche Gesellschaft für Klinische Neurophysiologie und Funktionelle Bildgebung
DKI	Diffusions-Kurtosis-Bildgebung (engl. diffusion kurtosis imaging)
DLK/DLB	Demenz mit Lewy-Körperchen
DOS-S	Delirium Observation Scale
DPG	Deutsche Gesellschaft für Parkinson und Bewegungsstörungen e. V.
dPV	Deutsche Parkinson Vereinigung
DRG	Diagnosis Related Groups
DSM	Diagnostic and Statistical Manual of Mental Disorders
DTI	Diffusion tensor imaging
EAU	European Association of Urology
EBM	Evidence Based Medicine
EG	Experimentalgruppe
EMA	European Medicines Agency
EMST	Expiratory Muscle Strength Training
ESC	European Society of Cardiology
ESS	Epworth Sleepiness Scale
FAB	Frontal Assessment Battery
FACIT-F	Functional Assessment of Chronic Illness Therapy – Fatigue
FDA	Food and Drug Administration
FEES	flexible endoskopische Evaluation des Schluckakts
FoG	Freezing of Gait
FSS	Fatigue Severity Scale
GAI	Geriatric Anxiety Inventory
GDS-15	Geriatrische Depressionsskala
GHB	Gammahydroxybuttersäure
GPi	Globus pallidus internus
HADS	Hospital Anxiety and Depression Scale
HAM-A	Hamilton Anxiety Rating Scale
HAM-D	Hamilton Depression Rating Scale
HRM	High-Resolution-Manometrie
IADL	Instrumentelle Aktivitäten des täglichen Lebens (engl. Instrumental Activities of Daily Living)
IASP	International Association for the Study of Pain
ICD	Internationale statistische Klassifikation der Krankheiten und verwandter Gesundheitsprobleme (engl. International Statistical Classification of Diseases and Related Health Problems)
ICSD	International Classification of Sleep Disorders
IDS	Inventory for Depressive Symptoms
IKS	Impulskontrollstörungen
PK	Parkinson-Krankheit
iRBD	isolierte REM-Schlaf-Verhaltensstörung

KG	Kontrollgruppe
KHK	koronare Herzkrankheit
KI	Konfidenzintervall
KPPS	King's Parkinson's Disease Pain Scale
KSS	Karolinska Sleepiness Scale
KVT	kognitive Verhaltenstherapie
LCIG	Levodopa-Carbidopa Intestinales Gel
LECIG	Levodopa/Entacapon/Carbidopa intestinales Gel
LEDD	Levodopa-Äquivalenzdosis
LID	Levodopa-induzierte Dyskinesie
LKK	Lewy-Körperchen-Krankheit
LL	Leitlinie
LR	Likelihood Ratio
LSVT	Lee Silverman Voice Treatment
MADRS	Montgomery–Asberg Depression Rating Scale
MCI	Mild Cognitive Impairment
MCP	Middle cerebellar peduncle
MDES	malignes dopaminerges Entzugssyndrom
MDS	Movement Disorder Society
MDS-NMS	MDS Non-Motor Rating Scale
MDT-PD	Münchener Dysphagie-Test-Parkinson's Disease
MFI	Multidimensional Fatigue Inventory
Mini-ACE	Kurzversion der Addenbrooke's Cognitive Examination
MMST	Mini-Mental-Status-Test
MoCA	Montreal Cognitive Assessment
MRgFUS	Magnetresonanz-gestützte fokussierte Ultraschallbehandlung
MRPI	MR-Parkinsonismus-Index
MSA	Multisystematrophie
MSLT	Multipler Schlaflatenztest
MWT	Multipler Wachbleibetest
NAA-ADL	Nürnberger-Alters-Inventar-Aktivitäten-Skala
NAB-ADL	Nürnberger-Alters-Inventar-Beobachtungsskala
NBIA	Neurodegeneration mit Eisenspeicherung im Gehirn (engl. Neurodegeneration with Brain Iron Accumulation)
NfL	Neurofilament-Leichtketten (engl. Neurofilament Light Chains)
NL	Nucleus lentiformis
NMSS	Non-Motor Symptom Scale
NMSQ	Non-Motor Symptom Questionnaire
NOSGER	Nurses' Observation Scale for Geriatric Patients
NPI	Neuropsychiatric Inventory
NRI	Noradrenalin-Wiederaufnahmehemmung
Nu-DESC	Nurse Delirium Screening Scale
ODT	oral lösliche Tablette (engl. orally disintegrating tablets)

OH	orthostatische Hypotonie
OR	Odds Ratio
OSA	obstruktive Schlafapnoe
PA-F	Progredienzangst-Fragebogen
PANDA	Parkinson Neuropsychometric Dementia Assessment
PANSS	Positive and Negative Syndrome Scale
PAS	Parkinson's Anxiety Scale
PASE	Physical Activity Scale for the Elderly
PD	Parkinson's Disease
PD-CRFT	Parkinson's Disease Cognitive Functional Rating Scale
PD-CRS	Parkinson's Disease-Cognitive Rating Scale
PD-MCI	PK mit leichter kognitiver Störung (engl. Parkinson's Disease with mild cognitive impairment)
PD-PCS	Parkinson's Disease Pain Classification System
PDQ	Parkinson's Disease Questionnaire
PDSS-2	Parkinson's Disease Sleep Scale
PHQ-9	Patient Health Questionnaire
PICO	Population Intervention Comparison Outcome
PICS	Parkinson's Impulse-Control Scale for the Severity Rating of Impulse Control Behaviors in Parkinson's Disease
PK	PK
PKB	Parkinson-Komplexbehandlung
PKT	Parkinson-Komplextherapie
PKD	PK mit Demenz
PLMD	Periodic Limb Movement Disorder
PLVT	Pitch Limiting Voice Treatment
PN	Parkinson Nurse
PPMI	Parkinson's Progression Marker Initiative
PPV	positiver prädiktiver Wert
PSG	Polysomnographie
PSP	progressive supranukleäre Blickparese
PSQI	Pittsburgh Sleep Quality Index
PTK	Parkinson-Tagesklinik
PVT RT	Psychomotor Vigilance Test Reaction Time
QoL	Lebensqualität (engl. Quality of Life)
QUIP	Questionnaire for Impulsive-Compulsive Disorders in Parkinson's Disease
RBD	REM-Schlaf-Verhaltensstörung
RCT	randomisierte kontrollierte Studie (engl. Randomized controlled Trial)
RLS	Restless-Legs-Syndrom
ROCFT	Rey-Osterrieth Complex Figure Test
rTMS	repetitive transkranielle Magnetstimulation
SAA	Seed Amplification Assays
SAPS	Schedule for Assessment of Positive Symptoms

SBAS	Schlafbezogene Atmungsstörungen
SCOPA-COG	Scales for Outcomes in Parkinson's Disease – Cognition
SCOPA-S	Scales for Outcomes in Parkinson's Disease – Sleep Scale
SCP	Superior cerebellar peduncle
SDQ	Swallowing Disturbance Questionnaire
SHAPS	Snaith-Hamilton Pleasure Scale
SN	Substantia nigra
SNP	Single nucleotide polymorphism
STN	Nucleus subthalamicus
SWEDD	Subjects without evidence of dopaminergic deficit
TAP	Testbatterie zur Aufmerksamkeitsprüfung
TCS	transkranielle Sonographie
THS	Tiefe Hirnstimulation
THS-ES	Tiefe-Hirnstimulations-Entzugssyndrom
TMT	Trail Making Test
TSS	Tagesschläfrigkeit
VAST	Videoassistierte logopädische Schlucktherapie
UPDRS	Unified Parkinson's Disease Rating Scale
UPSIT	University of Pennsylvania Smell identification Test
VFSS	Videofluoroskopie des Schluckens
VIM	Ncl. ventro-intermedius thalami/posteriore subthalamische Area
VP	Vascular Parkinsonism
VOSP	Testbatterie für visuelle Objekt- und Raumwahrnehmung
WASO	Wake after sleep onset
WHO-5	WHO-5 Well-being Index

Impressum

© 2023 Deutsche Gesellschaft für Neurologie,
Reinhardtstr. 27 C, 10117 Berlin

Kommission Leitlinien der DGN

Vorsitzende
Prof. Dr. med. Helmuth Steinmetz
PD Dr. med. Oliver Kastrup (stellv.)

Mitglieder
Dr. med. Klaus Gehring (Vertreter der Niedergelassenen)
Prof. Dr. med. Peter U. Heuschmann
Prof. Dr. med. Dagny Holle-Lee
Prof. Dr. med. Günter Höglinger
Prof. Dr. med. Matthias Klein
Prof. Dr. med. Susanne Knake
Prof. Dr. med. Thomas Lempert
Prof. Dr. med. Matthias Maschke (Vertreter der Chefärztinnen/Chefärzte)
Prof. Dr. med. Thomas Platz
Prof. Dr. med. Heinz Reichmann
Prof. Dr. med. Peter Sandor (Vertreter der SNG)
Prof. Dr. med. Christiane Schneider-Gold
Prof. Dr. med. Claudia Sommer
Prof. Dr. med. Bernhard J. Steinhoff
Prof. Dr. med. Götz Thomalla
Prof. Dr. med. Lars Timmermann (DGN-Präsident)
Prof. Dr. med. Claus W. Wallesch
Prof. Dr. med. Jörg R. Weber (Vertreter der ÖGN)
Prof. Dr. med. Christian Weimar
Prof. Dr. med. Michael Weller
Prof. Dr. med. Wolfgang Wick

Editorial Office der DGN

Redaktion: Katja Ziegler, Sonja van Eys,
DGN Dienstleistungsgesellschaft mbH,
Reinhardtstr. 27 C, 10117 Berlin

Kontakt: leitlinien@dgn.org

**Versionsnummer:**	**8.1**
**Erstveröffentlichung:**	**01/1997**
**Überarbeitung von:**	**10/2023**
**Nächste Überprüfung geplant:**	**10/2028**

Die AWMF erfasst und publiziert die Leitlinien der Fachgesellschaften mit größtmöglicher Sorgfalt - dennoch kann die AWMF für die Richtigkeit des Inhalts keine Verantwortung übernehmen. **Insbesondere bei Dosierungsangaben sind stets die Angaben der Hersteller zu beachten!**

Autorisiert für elektronische Publikation: AWMF online
